# A yeast phenomic model for the gene interaction network modulating CFTR-ΔF508 protein biogenesis

**DOI:** 10.1186/gm404

**Published:** 2012-12-27

**Authors:** Raymond J Louie, Jingyu Guo, John W Rodgers, Rick White, Najaf A Shah, Silvere Pagant, Peter Kim, Michael Livstone, Kara Dolinski, Brett A McKinney, Jeong Hong, Eric J Sorscher, Jennifer Bryan, Elizabeth A Miller, John L Hartman

**Affiliations:** 1 Department of Genetics, University of Alabama at Birmingham, 730 Hugh Kaul Human Genetics Building, 720 20th Street South, Birmingham, AL 35294 USA; 2 Gregory James Cystic Fibrosis Center, University of Alabama at Birmingham, 790 McCallum Basic Health Sciences Building, 1918 University Boulevard, Birmingham, AL 35294 USA; 3 Department of Biology, Columbia University, 1212 Amsterdam Ave. MC2456, New York, NY 10027 USA; 4 Department of Statistics and Michael Smith Laboratories, University of British Columbia, 3182 Earth Sciences Building, 2207 Main Mall, Vancouver, BC V6T 1Z4 Canada; 5 Lewis-Sigler Institute for Integrative Genomics, Princeton University, Washington Road, Princeton, NJ 08544 USA; 6 Department of Computer Science, University of Tulsa, 2145 J. Newton Rayzor Hall, E 5th Pl, Tulsa, OK, 74104 USA

**Keywords:** Gene interaction, Genetic buffering, Genotype-phenotype complexity, Phenomics, Quantitative high throughput cell array phenotyping (Q-HTCP), Cystic fibrosis transmembrane conductance regulator (CFTR), ER membrane complex (EMC), ATP binding cassette (ABC) transporter, Membrane protein biogenesis, Yeast model of human disease, Comparative functional genomics

## Abstract

**Background:**

The overall influence of gene interaction in human disease is unknown. In cystic fibrosis (CF) a single allele of the cystic fibrosis transmembrane conductance regulator (CFTR-ΔF508) accounts for most of the disease. In cell models, CFTR-ΔF508 exhibits defective protein biogenesis and degradation rather than proper trafficking to the plasma membrane where CFTR normally functions. Numerous genes function in the biogenesis of CFTR and influence the fate of CFTR-ΔF508. However it is not known whether genetic variation in such genes contributes to disease severity in patients. Nor is there an easy way to study how numerous gene interactions involving CFTR-ΔF would manifest phenotypically.

**Methods:**

To gain insight into the function and evolutionary conservation of a gene interaction network that regulates biogenesis of a misfolded ABC transporter, we employed yeast genetics to develop a 'phenomic' model, in which the CFTR-ΔF508-equivalent residue of a yeast homolog is mutated (Yor1-ΔF670), and where the genome is scanned quantitatively for interaction. We first confirmed that Yor1-ΔF undergoes protein misfolding and has reduced half-life, analogous to CFTR-ΔF. Gene interaction was then assessed quantitatively by growth curves for approximately 5,000 double mutants, based on alteration in the dose response to growth inhibition by oligomycin, a toxin extruded from the cell at the plasma membrane by Yor1.

**Results:**

From a comparative genomic perspective, yeast gene interactions influencing Yor1-ΔF biogenesis were representative of human homologs previously found to modulate processing of CFTR-ΔF in mammalian cells. Additional evolutionarily conserved pathways were implicated by the study, and a ΔF-specific pro-biogenesis function of the recently discovered ER membrane complex (EMC) was evident from the yeast screen. This novel function was validated biochemically by siRNA of an EMC ortholog in a human cell line expressing CFTR-ΔF508. The precision and accuracy of quantitative high throughput cell array phenotyping (Q-HTCP), which captures tens of thousands of growth curves simultaneously, provided powerful resolution to measure gene interaction on a phenomic scale, based on discrete cell proliferation parameters.

**Conclusion:**

We propose phenomic analysis of Yor1-ΔF as a model for investigating gene interaction networks that can modulate cystic fibrosis disease severity. Although the clinical relevance of the Yor1-ΔF gene interaction network for cystic fibrosis remains to be defined, the model appears to be informative with respect to human cell models of CFTR-ΔF. Moreover, the general strategy of yeast phenomics can be employed in a systematic manner to model gene interaction for other diseases relating to pathologies that result from protein misfolding or potentially any disease involving evolutionarily conserved genetic pathways.

## Background

Since release of the human genome sequence, genome-wide association studies (GWAS) and other advances in genomic technology have challenged simplistic notions of the genetic basis of human disease. Even Mendelian disease phenotypes are now thought to be driven by complex genetic relationships [1]. For example, modifier genes can influence the severity of cystic fibrosis [2]. However, the influence on disease contributed by multi-locus, combination-specific pairs of allelic variants remains largely unmapped and uncharacterized biologically. Moreover, most disease traits are non-Mendelian (that is, 'complex' traits), where expression of the phenotype involves multiple different gene activities, none of which is individually required or accounts for a large fraction of heritability [3,4]. Thus Mendelian and complex traits can be seen as different ends of the same continuum in which multiple genetic and environmental effects impact disease risk and/or severity in a combination-dependent manner. It is presumed that in some genetic or environmental contexts particular variant alleles are phenotypically expressed, and in other contexts they are buffered. However, whether principles for disease variation can be deduced through systematic analysis of gene-gene interaction remains unknown [5]. In this study we developed a yeast model of gene interaction for a clinically relevant disease mutation, CFTR-ΔF508, to investigate whether it can potentially serve as a useful tool to better understand the genetic complexity underlying the human disease, cystic fibrosis [6]. *Saccharomyces cerevisiae *is a workhorse for fundamental biology, but the extent to which experimental models of gene-gene interaction employing an endogenous yeast cellular context could provide disease-relevant insight via gene homology is unknown [5]. To investigate this question, we applied the Q-HTCP method to systematically query the yeast genome for modifiers of a specific phenotype resulting from Yor1-ΔF670, and provide evidence validating this yeast phenomic (genome-wide analysis of phenotypic modification due to gene interaction) model for CFTR-ΔF508, the most prevalent human allele causing cystic fibrosis [7].

To model the evolutionarily conserved network of gene interaction involving CFTR-ΔF508, we introduced the homologous yeast ABC transporter, Yor1-ΔF670 [8,9], into the library of non-essential yeast gene deletion strains [10-12], and used Q-HTCP [13,14] to measure the influence of gene-gene interactions on cell proliferation in the presence of oligomycin, a toxin extruded from cells by Yor1. From a drug discovery perspective, protein regulators of CFTR-ΔF biogenesis represent novel targets, and cell culture experiments indicate such targets are numerous [15,16]. Many of these regulators are evolutionarily conserved, thus a quantitative systems level model of a gene interaction network model derived from yeast could complement human and animal studies [17]. From a systems biology perspective, the quantitative description of a gene network that modulates biogenesis of a misfolded ABC transporter could provide useful insight for understanding the phenotypic complexity of cystic fibrosis in association with human genetic data, and might similarly aid study of other diseases related to protein misfolding. If successful for cystic fibrosis, the same general strategy of yeast phenomic modeling should be applicable to derive understanding about disease complexity involving any conserved cellular pathway.

### Methods

#### Yeast strains

**Figure 1:**
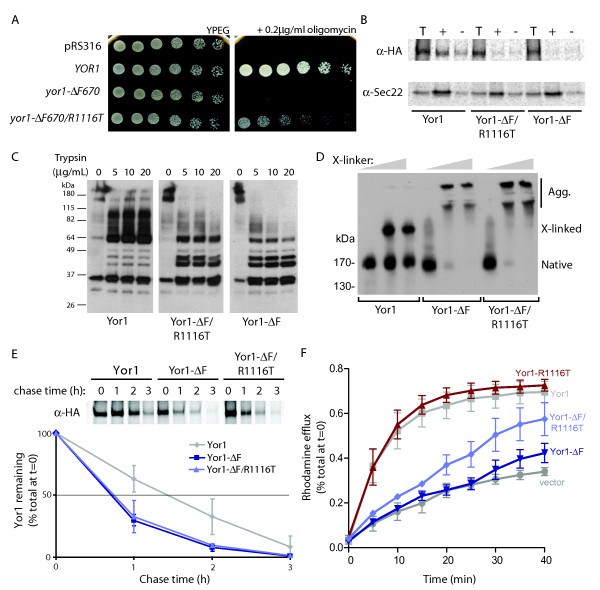
**Characterization of Yor1-ΔF/R1116T**. **(A) **The initial characterization of *YOR1 *alleles was performed using plasmid-based mutagenesis. A *yor1 *null strain, *yor1-Δ0*, was transformed with a plasmid control (pRS316), or plasmids expressing *YOR1*, *yor1-ΔF670*, or *yor1-ΔF670/R1116T *as indicated, and the strains were serially diluted and spotted onto YPEG media with and without 0.2 µg/mL oligomycin. The *yor1-ΔF670 *mutation was associated with a trafficking and pump defect that rendered it phenotypically equivalent to *yor1-Δ0*. However, an additional intragenic mutation, Yor1-ΔF670-R1116T, exhibited an intermediate phenotype. **(B) **Capture of Yor1 into ER-derived transport vesicles was measured using an *in vitro *vesicle budding assay that quantifies uptake of newly synthesized cargo proteins from radiolabeled permeabilized cells after addition of purified COPII proteins in the presence (+) and absence (-) of GTP. Total membranes (T) were separated from the liberated vesicles by differential centrifugation. Packaging of Yor1 into the vesicle fraction was monitored by immunoprecipitation; Sec22 is a control cargo protein that demonstrates efficient vesicle production even in the absence of packaging of the mutant forms of Yor1. Neither Yor1-ΔF nor Yor1-ΔF/R1116T were captured into COPII vesicles whereas wild-type Yor1 was packaged normally. **(C) **Trypsin sensitivity of Yor1 was assessed by limited proteolysis of microsomal membranes expressing wild type and mutant forms of Yor1. Increasing concentrations of trypsin were added as indicated prior to processing of membranes for immunoblot analysis. Wild-type Yor1 is cleaved to several stable bands whereas both Yor1-ΔF and Yor1-ΔF/R1116T were significantly more susceptible to proteolytic attack. **(D) **Cross-linking between transmembrane domains of Yor1 was measured following introduction of paired cysteine substitutions into wild-type and mutant Yor1 as indicated. Addition of increasing concentrations of cross-linker resulted in the accumulation of wild-type Yor1 in a cross-linked species with distinct gel mobility. Cross-linking of Yor1-ΔF and Yor1-ΔF/R1116T resulted in the disappearance of the non-cross-linked species and the appearance of high molecular weight aggregates, suggesting abnormal assembly of transmembrane domains in these mutants. **(E) **Yor1 stability was monitored by pulse-chase analysis. Cells expressing wild-type or mutant forms of Yor1 were radiolabeled for 10 min, and then chased for 180 min with non-radioactive amino acids. The amount of Yor1 present at each time point was determined by immunoprecipitation and autoradiography (top panel). The percentage of Yor1 remaining was calculated relative to the starting material at t = 0 (bottom panel). **(F) **Yor1 function was probed using a rhodamine-pumping assay. Yor1-Δ0/Pdr5-Δ0 cells carrying the indicated Yor1 alleles on a plasmid were loaded with the fluorescent dye, rhodamine, and the amount of fluorescence released over time into the culture supernatant was measured. The Yor1-ΔF670-R1116T mutation was associated with rhodamine extrusion intermediate between that of the wild-type Yor1 allele and either the Yor1-Δ0 or Yor1-ΔF670 mutant forms (which were functionally equivalent in this assay, as in the oligomycin resistance growth phenotype assay).

Deletion mutants were from the MATa collection, created by the Saccharomyces Genome Deletion Project [18], and obtained from Open Biosystems. The query strain background for double mutant construction was 15578-1.2b [10]. The R1116T mutation (Figure 1) was introduced into pSM2056 (*yor1-ΔF670-HA-GFP::URA3 *integrating vector) [19] by Quik Change mutagenesis (Stratagene) to create plasmid pRL026. This vector was used as a template to amplify a PCR fragment corresponding to *yor1-ΔF670/R1116T-HA-GFP-*3'UTR which was combined with another PCR fragment encoding the *NATMX *cassette flanked by further *YOR1 *3'UTR sequence by splice overlap PCR. The full product *(yor1-ΔF670/R1116T-HA-GFP-3'UTR-NATMX-3'UTR*) was transformed into yeast and selected for on media containing nourseothricin ('ClonNat', Werner BioAgents); the presence of the genomic *ΔF670/R1116T *mutation was confirmed by sequence analysis, creating strain RL4. The endogenous *YOR1 *promoter was replaced with a Tet-OFF regulatable element by insertion of pJH023, as previously described [20], at the *YOR1 *locus to create strain RL8. RL8 was mated to the MATa deletion strain collection and double mutants selected by the synthetic genetic array (SGA) method [11].

#### Yeast media

For SGA [11], media was prepared with the following modifications. Mating was carried out in YPD liquid followed by diploid selection in YPD containing G418 and ClonNat, and a second round of diploid selection substituting Pre-Spo media 5 for YPD as described [21]. Cultures were sporulated at room temperature for 1 week, before two rounds of transfer to haploid double mutant selection media [11]. For Q-HTCP, YPEG media (10 g/L yeast extract, 20 g/L peptone, 3% ETOH, 3% glycerol, and 1.5% agar) was used with 2 ng/mL doxycycline and concentrations of oligomycin ranged from 0.05 to 0.25 ug/mL for *yor1-ΔF *strains, and 0.05 to 0.35 ug/mL for *YOR1 *strains. Doxycycline was used at 2 ng/mL to optimize the expression level of Yor1-ΔF for phenotypic screening to detect enhancers and suppressors at the indicated concentrations of oligomycin.

#### Cell proliferation measurements and quantification of gene interaction

**Figure 2:**
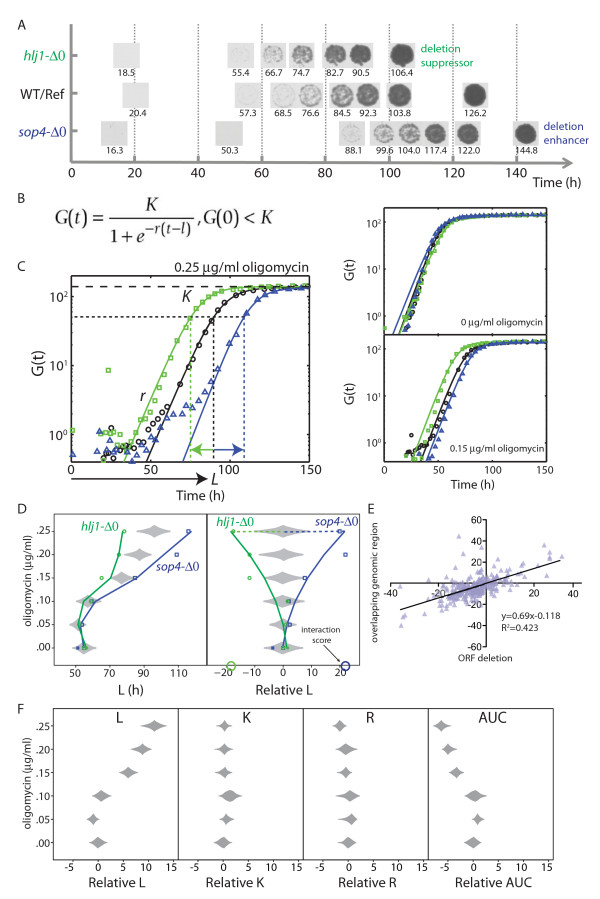
**A genome-wide screen for Yor1-ΔF gene interaction**. (**A**) Time-lapse imaging was used to measure the growth phenotypes of haploid double mutants. Shown are example spot cultures (time indicated below each image) for strains with deletion suppressor and deletion enhancer effects on oligomycin sensitivity, along with a *yor1-ΔF *single mutant control, grown on media containing 0.2 μg/mL oligomycin. (**B**) To quantify phenotypes, spot culture image series were analyzed for pixel density and fit to a logistic growth equation [14]. See Materials and Methods and Additional File 1 - Discussion B for further details. (**C**) Multiple concentrations of oligomycin were used to assess the interaction strength for each gene deletion, using the growth parameter, L, corresponding to the time at which a culture reaches its half maximal density, K (r denotes the maximum specific rate). Gene deletion suppressor effects (interactions reducing L) are highlighted in green, whereas gene deletion enhancer effects (interactions increasing L) are indicated by blue. The three panels contain growth curves for the deletion strains shown in panel A at different oligomycin concentrations (0, 0.15, and 0.25 μg/mL). G(t) is the logistic fit for the data obtained for each culture time series; raw values for culture growth are indicated by black circles (WT/Ref), green squares (*hlj1-Δ0 *strain), and blue triangles (*sop4-Δ0 *strain). (**D**) Gene interaction is shown for *hlj1-Δ0 *(green squares) and *sop4-Δ0 *(blue triangles). Divergence of L for the double mutants is displayed as a function of oligomycin concentration, compared to the phenotypic distribution of replicates of the *yor1-ΔF670/R1116T *single mutant (gray diamonds represent the distribution of central 95% of L values for 768 single mutant replicates). The data for the double mutants were shifted by their difference with the single mutant (median response) at the zero oligomycin concentration (filled symbols), correcting for growth differences not attributable to oligomycin response. To quantify interactions, the data for each deletion mutant were first fit to a quadratic equation, and then the difference between the deletion mutant and the reference median was taken at an indicated concentration of oligomycin. To highlight the interactions, the raw data (left panel) were transformed to remove the oligomycin dose effect (right panel). (**E**) A scatter plot of interaction scores for pairs of gene deletion strains with overlapping open reading frames (obtained at oligomycin = 0.25 µg/mL). The open reading frames with a greater degree functional annotation in SGD were designated as the 'ORFs', and those with less functional annotation designated 'overlapping genomic regions' [78]. (**F**) The affect of oligomycin dose on the growth curve parameters (left to right), L (time to half carrying capacity, K (carrying capacity), and r (maximum specific rate), and the area under the curve ('A'), for 384 replicates of the *yor1-ΔF *single mutant strain. Each diamond represents the central 95% of the standardized data for that oligomycin dose. The data for each parameter at each dose was standardized (arbitrary units) by subtracting the mean and dividing by the standard deviation of the group not treated with oligomycin. The oligomycin = 0 group is centered at 0. Using standard units for the data allows the dose trend between the panels to be directly compared. The oligomycin dose effect is greatest for L, followed by AUC, and with minor effects on K and r.

Cells were inoculated from glycerol stocks in a 384 well format and grown for 36 to 48 hours in YPD with G418 (200 ug/mL) and ClonNat (100 ug/mL), and without doxycycline. Overnight-grown cell arrays were spotted to agar plates using a 384-pin tool (FP6 pins from V-P Scientific) after first transferring to a 'dilution plate' to reduce the number of cells transferred, as described previously [13]. Quantitative high throughput cell array phenotyping was used to obtain growth parameters by time lapse imaging of cell arrays and fitting to a logistic growth equation (Figure 2B), as described previously [13,14]. The parameter L, which is equivalent to the time at which half the final carrying capacity is reached, was used to quantify interactions (Figure 2C). The growth curve parameters obtained from the fitted curves are provided in Additional File 1. Interactions were quantified on the basis of a change in the response to oligomycin attributable to a gene deletion (Figure 2D), where interaction strength is a function of oligomycin response as determined by departure of the L value for a given double mutant strain *vs*. the Yor1-ΔF single mutant across all oligomycin concentrations. To compute the interaction strength, the following algorithm was used to determine the difference between each double mutant and the *yor1-ΔF670 *single mutant:

Y_i _= Observed growth parameter for the knockout at dose i (D_i_)

K_i _= the effect of the knockout and its interaction with *yor1-ΔF *at a dose of oligomycin

K_0 _= the effect of knockout when no oligomycin is present (D_0_)

L_i _= the interaction effect of a knockout with yor1-ΔF at each dose of oligomycin

1. Compute the average value of the 768 reference cultures at (D_i_): RD_i_,

To simplify visualization of the interaction graphically,

2. Remove the dose effect to oligomycin on the reference: K_i _= Y_i _- RD_i_

3. Remove the effect of knockout (K_0_) when no oligomycin is present (D_0_): L_i _= K_i _- K_0_

Therefore L_0 _= 0 by definition.

4. Fit a quadratic curve: L_i _= A + B*D_i _+ C*D_i_^2^

5. Compute the interaction value at the max dose: L_i-max_= INT = A + B*D_max _+ C*D_max_^2^

Positive interaction values, termed 'deletion enhancers', denote increasing L and thus indicate exacerbation of the growth delay induced by oligomycin. For deletion strains failing to grow at the higher concentrations of oligomycin, interactions were ranked in tiers, with the strains failing to grow at a greater number of concentrations grouped as stronger deletion enhancers (Additional File 1). Conversely, strains that grew faster (shorter time to reach L) had negative interaction values and we refer to loss of the gene having a 'deletion suppressor' effect on the oligomycin sensitivity phenotype. Interaction plots for each gene deletion strain in both the context of wild-type *YOR1 *and *yor1-ΔF670/R1116T *expression are given in Additional Files 2 and 3. The graphs are ranked by the interaction strength of the *yor1-ΔF670/R1116T *allele. To help further partition the list of genes influencing the *yor1-ΔF/R1116T *phenotype, gene-drug interaction data were incorporated with the primary screen data for clustering (described below). For gene-drug interactions, the number of concentrations of each drug tested was too few to fit a quadratic, thus each perturbation was considered separately and interactions were quantified as the difference between the deletion and the wild-type reference strains and plotted after adjusting for the dose effect of oligomycin and the effect of the deletion on growth in the control media. The interaction data submitted to BioGRID [22] for inclusion in the BioGRID database and SGD [23] are indicated in Additional File 5 in column L of the worksheet 'REMc_data and clustering'.

#### Recursive expectation-maximization clustering (REMc)

Interaction values selected for clustering represented the union of genes from the *yor1-ΔF670/R1116T *screen with interaction values >10 or <-16 and the screen with wild-type *YOR1 *in the same background with interaction values >10 or <-12. These thresholds were chosen to represent the tails of the distributions of interaction strength. Among deletion strains not growing at one or more concentrations of oligomycin, higher interaction values were assigned for cultures that failed to grow at lower concentrations (see Additional File 5). Gene-drug interaction data were incorporated to create profiles for genes selected from the primary screen, as previously described [13]. REMc was used to identify groups of genes having similar interaction profiles [24]. To obtain a dendrogram and finer grain view of each REMc cluster, hierarchical clustering using Euclidian distance and complete linkage was performed using Matlab. For all heat maps, the order of the perturbations is the same and labels indicate the interaction values from: (A) the *yor1-ΔF670/R1116T*/gene deletion double mutants; (B) the screen of single-mutant (wild-type *YOR1 *background) gene deletion strains; (C) the growth defect of the deletion strain in Cold Spring Harbor SC media [25]; gene-drug interactions on the following media (D) SC media lacking threonine (using media in (C) as the reference); (E) SC media lacking threonine and with 80 ug/mL beta-chloro-alanine (using media in (D) as the reference); SC media supplemented with (F) 0.7 nM rapamycin; (G) 1.4 nM rapamycin; (H) 1 nM FK-506; (I) 0.7 nM rapamycin and 1 nM FK-506; (J) 50 mM hydroxyurea; (K) 125 mM hydroxyurea; (L) 75 ng/mL cycloheximide; (M) 125 ng/mL cycloheximide; (N) 150 nM miconazole; or (O) 225 nM miconazole (see Additional File 5).

#### Gene homology mapping

The Princeton Protein Orthology Database [26] was used to identify yeast-human homologs for relating the results of our yeast screen to the larger literature of CFTR-ΔF508 protein biogenesis factors [27]. In cases where homology was not one-to-one, the best functional matches were discussed [28]. For example, human isoforms of HSP90 (HSP90A and HSP90B) have opposite effects on CFTR-ΔF508 biogenesis when knocked down by siRNA [16], thus deletion of yeast *HSP82*, an HSP90 family member in yeast that acts as a deletion suppressor, mimics only the effect of siRNA knockdown of HSP90A. As another example, yeast *HLJ1 *and three different homologous human proteins (CSP, DNAJB12, and DNJB2) exert comparable effects on Yor1-ΔF and CFTR-ΔF biogenesis, respectively (see Additional File 1 - Discussion C).

#### Biochemical analysis of Yor1-ΔF670 and Yor1-ΔF670/R1116T

*In-vitro *uptake of Yor1 into COPII vesicles was performed from radiolabeled semi-intact cells, and limited proteolysis, chemical cross-linking, and *in-vivo *pulse-chase experiments were all performed as described [29].

#### Rhodamine efflux assay

*yor1-Δ0/pdr5-Δ0 *double mutant strains bearing plasmids expressing *YOR1 *variant alleles (as indicated in Figure 1) were grown to mid-log phase (OD_600 _of approximately 0.5) in SD-ura medium (0.67% yeast nitrogen base, 20% glucose, -ura dropout mix). Cells equivalent to fifty OD_600 _units were harvested, washed with 50 mM HEPES pH 7.0, and loaded with rhodamine B (Sigma-Aldrich) by incubating cells in 5 mL of 50 mM HEPES, pH 7.0, 5 mM 2-deoxyglucose, and 100 μg/mL rhodamine B for 2 h at 30ºC. Cells were washed and resuspended in 5 mL of 50 mM HEPES, pH 7.0, supplemented with 10 mM D-glucose (Sigma-Aldrich). Every 2 min, 500 μL aliquots of cell suspension were removed, cells collected by centrifugation, and the rhodamine-containing supernatant was removed and quantified by measuring absorbance at OD_555_.

#### siRNA experiments

For TTC35 mRNA knockdown experiments, HeLa cells (CCL2, ATCC) were transfected with pcDNA-CFTR-*Δ*F508 plasmids using TransIT-HeLaMONSTER® transfection reagent (Mirus Bio, Madison, WI, USA) per instruction manual. Cells were split into a 12-well plate and the next day transfected with TTC35 specific siRNA (sc-77588, Santa Cruz Biotechnology, Santa Cruz, CA, USA) at 10 or 25 nM, using RNAiMAX (Invitrogen). As a negative control siRNA, Stealth RNAi™ siRNA negative control lo GC (Invitrogen, 12935-200) was used at 25 nM final concentration. The next day, cells were moved to 27°C and incubated for an additional 72 h before harvest. For western blot analysis, cells were lysed in RIPA containing Halt protease inhibitor cocktail (Thermo-Pierce), and then analyzed on 4% to 20% gradient SDS-PAGE (Invitrogen). After blotting onto a PVDF membrane, the blot was cut laterally into three pieces at 75kD and 35kD markers. The top piece (>75kD) was developed for CFTR protein (150 to 180 kD) using 3G11 rat monoclonal antibody [30] , the middle piece (between 75kD and 35kD) was probed for α-tubulin (approximately 55kD) as an internal control (DM1A antibody, GeneTex), and the bottom piece (<35kD) was probed with TTC35 antibody (sc-166011, Santa Cruz Biotechnology). Blots were developed using SuperSignal West Pico Chemiluminescent substrate (Thermo-Pierce), and exposed to Kodak BioMax MR film. Densitometry was performed using ChemiDoc XRS and Image Lab software (BioRad).

## Results

### Yor1-ΔF and CFTR-ΔF are membrane proteins with shared biogenesis defects

Yor1 is a close homolog of CFTR in the ATP-binding cassette family of membrane transporters that includes pleiotropic drug transporters [31], and it is the primary determinant of oligomycin resistance due its plasma membrane-localized function in extruding oligomycin from the cell [32]. Analogous to CFTR-ΔF508, mutation of the highly conserved phenylalanine residue in the first nucleotide binding domain, Yor1-ΔF670, results in ER-retention and degradation by proteolysis, yielding an oligomycin-sensitive phenotype [13]. However, unlike CFTR-ΔF508, Yor1-ΔF670 appears not to retain residual membrane transport function [8]. Therefore, we performed an intragenic suppressor screen and identified a second site mutation (R1116T) that restored partial pump function (Figure 1 and Additional File 1 - Discussion A). The oligomycin growth phenotype associated with Yor1-ΔF670-R1116T was intermediate between that of Yor1-ΔF670 (which was indistinguishable from the *yor1-Δ0 *deletion mutant) and wild-type Yor1 (Figure 1A). The intracellular fate of the partially functional R1116T mutant was identical to that of the original Yor1-ΔF mutant: the protein was less efficiently packaged into transport vesicles reconstituted *in vitro *(Figure 1B), Yor1-ΔF670-R1116T was misfolded, as detected by limited proteolysis (Figure 1C) and intramolecular cross-linking (Figure 1D), and turnover was indistinguishable from Yor1-ΔF670 by pulse-chase analysis (Figure 1E). We assessed the effect of the R1116T mutation on pump function using a rhodamine exclusion assay, which revealed partial rescue of Yor1-ΔF670-R1116T relative to Yor1-ΔF670 (Figure 1F). Although we do not know the precise mechanism by which the R1116T mutation impacts the activity of Yor1-ΔF, the aggregate of our evidence suggests that it is a dominant gain-of-function mutation that confers additional drug-pumping activity (see Additional File 1 - Discussion A and Additional file 1, Figure S1 for further description and characterization of the R1116T mutation). The molecular characteristics and intermediate oligomycin resistance conferred by Yor1-ΔF670-R1116T (referred to here forward as 'Yor1-ΔF') resemble the defects of CFTR-ΔF508, and thus provided a model to screen the yeast genome for canonical protein regulators of 'ΔF-associated' biogenesis by introducing *yor1-ΔF *into the yeast gene deletion strain collection [10,11].

### Measurement of gene interaction strength from growth curves

For quantitative phenotypic analysis of the genomic collection of deletion strains, we used growth curve analysis at multiple concentrations of oligomycin, and examined the entire library alternatively in the context of expression of Yor1-ΔF or Yor1 wild-type protein. The phenomic method of time series analysis of cell array images (Figure 2A) provides growth curves on a genomic scale for measuring strength of gene interaction [13]. The kinetic analysis is based on density of each spot culture over time [13,33], in contrast to qualitative methods or quantitative strategies that employ single time points of culture area [34,35]. Q-HTCP, by virtue of imaging cultures arrayed on agar rather than measuring optical density of liquid cultures in multi-well plates, provides orders of magnitude greater throughput, with spot density time series for each strain (Figure 2A) that fit to a logistic growth equation (Figure 2B) [14]. We used a parameter from the curve fitting to quantify each gene interaction by comparing growth inhibition between the Yor1-ΔF single mutant and each respective double mutant across multiple oligomycin concentrations (Figure 2C).

In this study, we focused on a specific parameter of logistic growth, termed L, which represents the time it takes a culture to reach half its final density, K [14] (Additional File 1 - Discussion B). Thus, the L parameter is inversely proportional to fitness, such that double mutant strains exhibiting a shorter L relative to the *yor1-ΔF *single mutant (that is, deletion suppressors of the oligomycin sensitivity phenotype) indicate genes that (when present) function to prohibit biogenesis of misfolded Yor1-ΔF. Conversely, gene interactions resulting in a longer L (that is, deletion enhancers) correspond to candidates that normally promote Yor1-ΔF biogenesis (Figure 2C). The null hypothesis for gene interaction [36] was defined by a neutrality function consisting of the median L value from replicate cultures of the Yor1-ΔF single mutant across increasing oligomycin concentration, to account for the drug effect. In addition, to account for the gene deletion effect on growth (independent of oligomycin treatment) the L value of each double mutant culture was adjusted (for every oligomycin dose) by the constant difference between it and the Yor1-ΔF reference mutant median at the zero-oligomycin concentration (Figure 2D, left panel). Next, a quadratic equation was fit to the L-value differences for each double mutant over all oligomycin concentrations. The difference between this quadratic fit and the reference median at the highest concentration of oligomycin having measurable growth was defined as the interaction score (enhancing interactions were further ranked according to the number of oligomycin concentrations where growth was completely inhibited). To more clearly visualize only the interactions, the data were transformed to remove the dose effect of oligomycin on the *yor1-ΔF *single mutant cultures (Figure 2D, right panel).

Our screen, by virtue of incorporating multiple concentrations of oligomycin and examining the trend of response, contains an intrinsic form of replication. The consistent trends of phenotypic response observed serves as evidence of technical reproducibility in the phenotypic analysis. We also repeated the entire screen at all concentrations, which again indicated high reproducibility (Additional file 1, Figure S2).

Reproducibility of the gene interaction measurements was further evidenced by positive correlation between values obtained for deletion strains that shared chromosomal strand overlap in their open reading frames (Figure 2E). To assess this type of correlation, each overlapping ORF pair member was assigned to one of two groups according to it being the 'better' or 'less well' annotated gene/orf. Less well-annotated orfs would, for example, include computationally determined chromosomal regions that were systematically knocked out by the Yeast Gene Deletion Consortium, but do not necessarily encode expressed genes [37]. Stronger interactions tended to correlate with the extent of gene annotation, perhaps due to residual functional activity in the non-overlapping regions of the better annotated genes that were not deleted by removal of overlapping ORFs (Figure 2E). The phenotypic parameter, L, which we used in this study to quantify interactions was more sensitive to detect the growth inhibitory effect of oligomycin (Figure 2F). This is the first study we are aware of demonstrating the utility of genome-scale growth curve acquisition and use of the L parameter for quantitative assessment of gene interaction in phenomic analysis.

### Detection of molecular mechanisms associated with weak gene interaction

**Figure 3:**
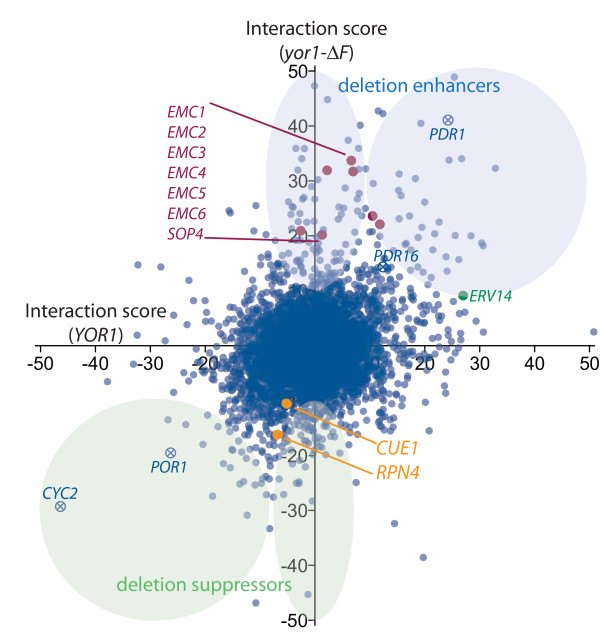
**Gene-gene interaction with Yor1 and Yor1-ΔF serves as a resource for identification of novel regulators of membrane protein biogenesis**. Interaction scores for individual yeast mutants in the context of either wild type Yor1 (x-axis) or Yor1-ΔF (y-axis) were plotted to illustrate classes of protein biogenesis regulators suggested by the screen. Deletion enhancers (positive value indicates prolonged L and slower growth) and suppressors of oligomycin sensitivity represent factors predicted to promote or prohibit protein biogenesis, respectively. Interactions along the y-axis may indicate proteins that act more specifically on misfolded proteins, while those in the upper right and lower left quadrants are considered to have more general effects on Yor1 protein biogenesis, or to affect the phenotype independently of Yor1/Yor1-ΔF. Examples of genes with functions suspected to directly influence oligomycin resistance, without necessarily acting through Yor1 biogenesis are denoted by 'x'. EMC members are colored in burgundy. *CUE1*, *RPN4*, and *ERV14 *are other genes that were further validated by molecular studies (see Figures 4, 6, and 7).

We found *yor1-ΔF *gene interaction to occur abundantly, across the genome and with wide-ranging strengths of effect. To help clarify the many interactions, we performed a similar analysis of oligomycin growth inhibition in the gene deletion strain collection endogenously expressing wild-type *YOR1 *(Figure 3). The comparison of Yor1 and Yor1-ΔF candidate regulators was focused on four general classes: those that impact (positively or negatively) only Yor1-ΔF, and those that impact (positively or negatively) both wild-type and the misfolded form of Yor1. Each class of mutant holds potential for uncovering novel mechanistic insight into biogenesis of topologically complex membrane proteins. Yor1-ΔF-specific interaction suggests pathways that recognize the misfolded protein, whereas interaction with both the misfolded and wild-type forms of the protein could represent either proteins that generally influence ABC transporter biogenesis or genes that affect oligomycin resistance independent of Yor1 function, such as pleiotropic drug resistance (PDR) genes or mitochondrial components (Figure 3).

**Figure 4:**
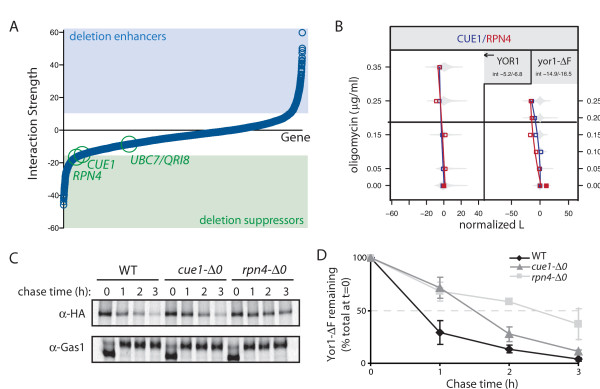
**Molecular validation of weak gene interactions**. **(A) **Ranking of interaction strength shows the distribution of phenotypic influence of deletion mutants. Some physiologically relevant hits (for example, *UBC7/QRI8*) fell below our clustering thresholds of >10 or <-16 (blue and green shading, respectively); the related components, *CUE1 *and *RPN4*, were on the cusp of our threshold but were still functionally relevant. (**B**) The *cue1-Δ0 *and *rpn4-Δ0 *strains showed deletion suppressor phenotypes specific for *yor1-ΔF *(at right), since the single mutant (that is, in the context of wild-type Yor1) did not affect oligomycin resistance. (**C**) By pulse-chase analysis, Yor1-ΔF turnover was reduced in the *cue1-Δ0 *and *rpn4-Δ0 *backgrounds relative to the wild-type background. Maturation of a control protein, Gas1, was unaffected. (**D**) Quantification of three replicates of the experiment shown in Figure 2B; error bars represent standard deviation.

Given the high sensitivity of the cell array method for measuring gene interaction, we sought perspective as to whether weak interactions reflected effects on Yor1 biogenesis that could be detected with molecular assays. Cue1 is an ER membrane protein that serves to recruit the ubiquitin-conjugating enzyme, Ubc7, to the ER, where it is required for ubiquitination of misfolded proteins prior to their disposal by proteasome-mediated ER-associated degradation. Rpn4 is a transcription factor that activates expression of proteasome genes; the depletion of proteasome subunits in an *rpn4-Δ0 *null strain would be expected to impair ER-associated degradation of misfolded proteins, potentially increasing their biogenesis. Yor1-ΔF670 turnover has been previously reported as diminished by mutation of *UBC7/QRI8 *[8,9]. Therefore, we examined Yor1-ΔF670 stability in the functionally related *cue1-Δ0 *and *rpn4-Δ0 *mutants, which showed weak interaction (Figure 4A, B). The half-life of Yor1-ΔF670 was indeed prolonged in both the *cue1-Δ0 *and *rpn4-Δ0 *strains relative to wild type (Figure 4C, D). Thus, the screen was sensitive to genes affecting proteasome-mediated turnover of Yor1-ΔF670, validating the yeast model with respect to this aspect of CFTR-ΔF biology [38,39] and confirming the molecular basis of phenotypic effects revealed by the screen.

### Genes interacting with Yor1-ΔF map to homologous regulators of CFTR-ΔF508

An open question is the extent to which gene interaction is evolutionarily conserved, and thus the extent to which simple genetic systems like yeast can reveal principles about gene interaction relevant to human disease [5]. A study comparing worms and yeast concluded gene interaction lacks conservation [40], whereas studies comparing evolutionarily divergent yeast have found that substantial conservation exists [41,42]. However, previous studies were not designed to model a specific disease-related mutation. Our data represented an opportunity to probe conservation of gene interaction within a clearly defined molecular and cellular context, namely biogenesis of homologous ABC proteins carrying mutation of a conserved disease-causing residue [5,13,14].

**Figure 5:**
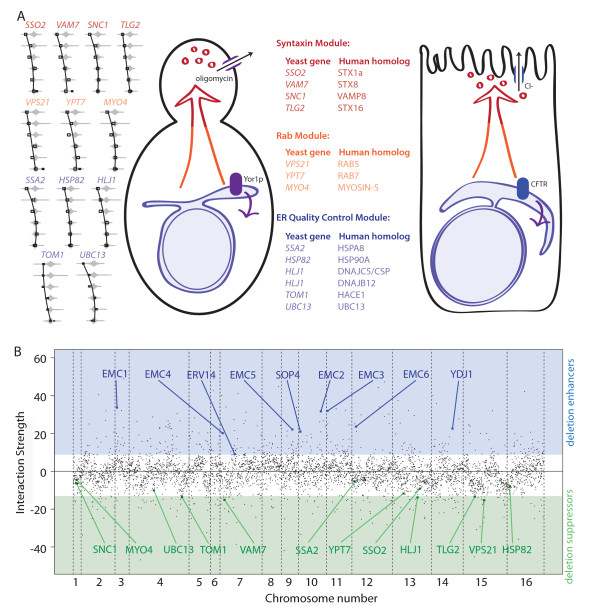
**Modulation of CFTR-ΔF and Yor1-ΔF biogenesis occurs via homologous gene interaction**. (**A**) At left, gene interactions are illustrated by plotting the parameter L *vs*. oligomycin concentration (increasing upward: 0, 0.05, 0.1, 0.15, 0.2, and 0.25 µg/mL) for the double mutant and the full distribution of 768 Yor1-ΔF single mutant cultures (gray diamonds). Leftward departure indicates faster growth and improved biogenesis of Yor1-ΔF. At right, human genes found in the literature to modulate CFTR-ΔF biogenesis are paired with homologous yeast genes. Gene pairs are grouped into protein classes associated with discrete cellular functions (see text and Additional File 1 - Discussion C). (**B**) The strength of gene interaction is depicted with respect to chromosomal position and highlighted gene interactions indicate those discussed in the manuscript (interactions are calculated at an oligomycin concentration of 0.2 µg/mL). Vertical lines demarcate chromosomes.

To assess relevance of our dataset to CFTR-ΔF biogenesis, we surveyed the literature for evidence of evolutionarily conserved cellular responses to the 'ΔF-like' folding defect. The (P-POD) [26] was used to identify homologous genes [27], yielding many examples of functional concordance between biogenesis factors for Yor1-ΔF and those known for CFTR-ΔF (Figure 5A). Most CFTR-ΔF protein regulators have been characterized using RNAi methods aimed at identifying targets for increasing CFTR-ΔF processing by small molecule inhibitors [15,16]. Accordingly, the majority of homologous yeast gene deletions found to modulate Yor1-ΔF biogenesis also functioned to enhance biogenesis. Broadly defined functional categories highlighted the shared fates of Yor1-ΔF and CFTR-ΔF, falling into at least three classes including (Figure 5A and Additional File 1 - Discussion C): Syntaxins, which mediate vesicle fusion within the secretory pathway and may also regulate CFTR channel activity more directly [43-46]; Rab proteins, which regulate vesicular trafficking of CFTR-ΔF and other plasma membrane proteins [47,48]; and ER quality control machineries, a class of regulators of that encompasses chaperones and other machineries that can influence folding and ER-associated degradation (ERAD) to govern the fate of misfolded proteins in the ER [16,49-52]. Each of these regulator classes exhibited homologous genes that encode regulators of CFTR-ΔF biogenesis (see Additional File 1 - Discussion C for further explanation of homologies). Moreover, because the homologous regulators were not the strongest in effect from the overall screen, additional conserved regulators were likely identified (Figure 5B). Together, these results indicate evolutionary conservation of gene interaction and suggest novel interactors from the Yor1-ΔF screen may represent as yet uncharacterized modifiers CFTR-ΔF biogenesis.

### Identification of functional gene modules by clustering analysis

We used REMc to search for functional gene modules [24]. Gene profiles selected for clustering had Yor1-ΔF interaction scores >10 or <-16, or in the context of wild-type Yor1 had gene-drug interaction >10 or <-12. We created interaction profiles for each gene by including additional gene-drug interaction data, and then assessed modularity (similar influence on the phenotype across different perturbations) by clustering [13,53,48]. The REMc algorithm objectively specified the number of clusters and provided an indication of cluster quality [24]. We used the GOid_z method to quantify overall enrichment of gene ontology (GO) functional information within clusters [24]. GOTermFinder, was used to identify specific terms associated with each cluster as well as the representative genes [54]. All clusters were enriched for functional information and many were associated with specific GO terms (Additional File 1 - Table S1 and Additional File 5). We also note that some functionally related genes appeared in different clusters, even though they exerted similar effects on Yor1-ΔF biogenesis (for example, the EMC genes described further below). This suggests that though they cooperate to determine the fate of Yor1-ΔF, they can function differentially in other cellular contexts. Other explanations for the appearance in different clusters of genes known to be functionally related include over-estimation of the number of clusters, measurement error, and the gene-specific functional relevance of particular gene interaction profiles selected for clustering.

### Validation of Erv14 as a cargo-specific sorting factor for Yor1

**Figure 6:**
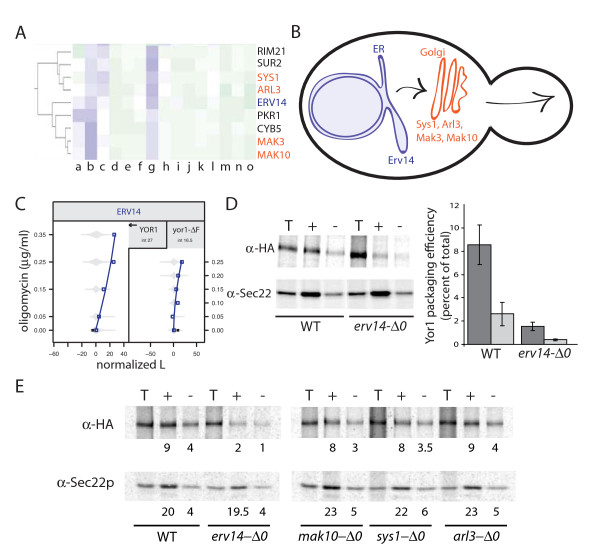
***ERV14 *promotes capture of Yor1 into COPII vesicles**. (**A**) The genetic interaction profile of the *erv14-Δ0 *strain clustered with those for the *sys1-Δ0*, *arl3-Δ0*, *mak3-Δ0*, and *mak10-Δ0 *strains. Columns represent diverse gene-drug interactions as described in methods. (**B**) Based on known data [55], and supported by our phenotypic and molecular findings, Erv14, Sys1, Arl3, Mak3, and Mak10 appear to function in a pathway with Erv14 acting in the ER and Sys1, Arl3, Mak3, and Mak10 functioning in the Golgi. (**C**) Gene-oligomycin interactions for *erv14-Δ0 *strains, in the context of either wild-type Yor1 or Yor1-ΔF, suggested Erv14 promotes the biogenesis of both Yor1 and Yor1-ΔF. (**D**) Packaging of wild-type Yor1 into COPII vesicles was quantified using an *in-vitro *budding assay that measures capture of newly synthesized cargo proteins from radio-labeled permeabilized cells after addition of purified COPII proteins in the presence ('+') or absence ('-') of GTP [9], followed by immunoprecipitation of the cargo protein of interest. 'T' indicates the total membrane pool of labeled Yor1-HA. Erv14-containing membranes showed approximately four-fold more efficient capture into vesicles of HA-tagged Yor1 than *erv14-Δ0 *membranes. The defect showed specificity, since *ERV14 *deletion did not affect packaging of another cargo protein, Sec22. Quantification of three independent experiments is shown at right; error bars represent standard deviation. (**E**) Similar vesicle budding assays from *mak10-Δ0*, *sys1-Δ0*, and *arl3-Δ0 *membranes showed no defects in Yor1 capture into COPII vesicles in these mutants, suggesting they function downstream of Erv14.

Cluster 2-0.1-1 contained genes previously shown to function cooperatively in protein transport through the secretory pathway (Figure 6A). Namely, Sys1, Mak3, and Mak10 cooperate in the recruitment of the ARF-like GTPase, Arl3, to the Golgi to regulate vesicular transport [55]. Given the clustering of these gene interaction profiles with Erv14, which can function as a cargo adaptor for enrichment of newly synthesized proteins into ER-derived transport vesicles, we suspected they may function in a common pathway, with Erv14 acting in an upstream compartment distinct from the others in the cluster (Figure 6B). Moreover, we observed oligomycin sensitivity to be more strongly dependent on *ERV14 *in the context of wild-type Yor1 than Yor1-ΔF (Figure 6C). This difference raised the possibility that Erv14 promotes capture of wild-type Yor1 into ER-derived transport vesicles [56] more efficiently than it does for the misfolded Yor1-ΔF substrate. According to this hypothesis, reduced recognition of the misfolded Yor1-ΔF by Erv14 would lessen the phenotypic impact of the *erv14-Δ0 *null allele on oligomycin sensitivity in an allele-specific manner (Figure 6C). We tested Erv14 function by *in vitro *reconstitution of COPII vesicle formation [57], comparing capture of Yor1 in the presence or absence of Erv14. Indeed, *ERV14 *deletion specifically reduced capture of Yor1, leaving a control cargo, Sec22, unaffected (Figure 6D). Yor1-ΔF capture was weak regardless of *ERV14 *status. Thus the gene-drug interaction between *erv14 *and oligomycin can be explained by a physical interaction between Erv14 and Yor1 that promotes ER export. Accordingly, our vesicle budding assay revealed no defects associated with capture into ER-derived vesicles in *sys1-Δ0, arl3-Δ0, mak3-Δ0*, and *mak10-Δ0 *strains (Figure 6E). Thus, taken together, the phenotypic and molecular data suggest this cluster of genes functions as a linear pathway to regulate progress of Yor1 out of the ER and through the Golgi for delivery to the plasma membrane.

### An ER membrane complex discovered in yeast promotes biogenesis of CFTR-ΔF

**Figure 7:**
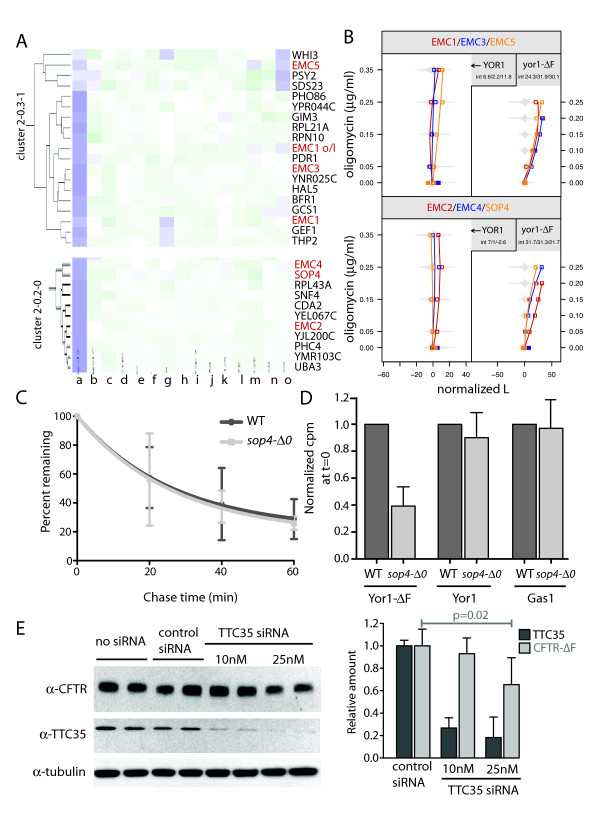
**A conserved ER membrane complex discovered in yeast promotes CFTR-ΔF biogenesis**. (**A**) Six of seven members of the recently described ER membrane complex (EMC) fell into two clusters (*EMC *genes are labeled in red; '*EMC1 *o/l' indicates *YCL046w*, which overlaps *EMC1/YCL045c*). Columns represent different gene-drug interactions as described in methods. (**B**) The interaction plots for *EMC *genes (corresponding to genes in panel A) suggested the complex has a pro-biogenesis effect specific for Yor1-ΔF. The similarity in profiles is consistent with the hypothesis that each *EMC *gene is required for the function of the complex in promoting Yor1-ΔF biogenesis. (**C**) By pulse chase analysis, deletion of *SOP4 *did not affect the half-life of Yor1-ΔF670. Quantification of three independent experiments is shown; error bars represent standard deviation. (**D**) Yor1-ΔF670 synthesis was reduced in the *sop4-Δ0 *strain based on the total amount of ^35^S-Met/Cys incorporated during the initial 10-min pulse. Three independent experiments were quantified; error bars represent standard deviation. Wild-type Yor1 and the GPI-anchored protein, Gas1, were unaffected by *SOP4 *deletion. (**E**) TTC35, the human homolog of *EMC2*, is required for normal biogenesis of CFTR-ΔF. HeLa cells, transiently expressing CFTR-ΔF at 27°C, were co-transfected with control siRNA, 10 nM TTC35 siRNA, or 25 nM TTC35 siRNA as indicated. Protein levels of TTC35, CFTR-ΔF, and α-tubulin were monitored from whole cell lysates by western blot, and (at right) relative abundance from three independent experiments was quantified by densitometry; error bars represent standard deviation, significance was assessed by a paired two-tailed *t*-test.

In light of homologous genes exerting analogous influences on Yor1-ΔF in yeast and CFTR-ΔF processing in human cells, respectively, we anticipated other yeast gene interactions identified by our screen would similarly represent homologs that function as conserved, uncharacterized CFTR-ΔF modulators. Our attention was drawn to a cluster (2-0.3-1) that contained *EMC1*, *EMC3*, and *EMC5*, three components of a recently described ER membrane complex [58]. Three additional members of this complex, *EMC2*, *EMC4*, and *SOP4 *grouped together in cluster 2-0.2-0 (Figure 7A). All seven of the EMC members were deletion enhancers with interaction specificity for the Yor1-ΔF mutant protein (Figure 7B), and all had comparable strengths of effect, suggesting removal of any one of the genes disrupts a function common to all [13,35].

The molecular function(s) of the EMC are only beginning to be characterized. Deletion of *EMC3 *(but not other EMC members) activated an unfolded protein response element (UPRE)-GFP reporter in a genome-wide screen, which led to identification of the complex. However, the EMC effect on Yor1-ΔF biogenesis appeared to be independent of any association with induction of the UPR, because deletion of *HAC1 *or *IRE1 *(which blocks the UPR) exerted no effect on oligomycin resistance, and there was very weak association between the strength of UPR activation and the influence of Yor1-ΔF biogenesis given the same gene deletion [58,59] (Additional file 1, Figure S3). Alternatively, EMC components might directly promote folding of Yor1-ΔF, such that loss of EMC function results in ER retention of Yor1-ΔF specifically, followed by ERAD-mediated degradation, with reduced delivery and/or stability at the plasma membrane. However, pulse-chase analysis revealed that the Yor1-ΔF half-life was not altered in EMC mutants (Figure 7C and data not shown). Instead, we observed that less Yor1-ΔF was synthesized in the initial 10-min pulse-labeling period when *SOP4*, a member of the EMC, was deleted (Figure 7D). Reduced labeling without increased degradation suggested a role for the complex in early stages of Yor1-ΔF biogenesis, such as during synthesis and translocation through the Sec61 translocation pore. Interestingly, this pro-biogenesis effect seemed specific to the misfolded protein, since the oligomycin phenotype associated with wild-type Yor1 was unaffected by deletion of *EMC *genes (Figure 7B). Furthermore, wild-type Yor1 and an unrelated plasma membrane protein, Gas1, were synthesized normally in the *sop4-Δ0 *mutant (Figure 7D).

Potential relevance of the EMC components to CFTR-ΔF processing was suggested by CFTR protein-protein interaction data indicating the homolog of *EMC2*, TTC35, physically associates with CFTR-ΔF but not wild-type CFTR (see supplemental data of reference [60]). However, at the time of that study, the EMC complex had not been characterized and only one subunit of the complex was identified by the interactome study. In contrast, we determined that all of the subunits give the same quantitative strength of interaction and cluster together in their phenotypic gene interaction profiles across several chemical perturbations. Thus our screen data provided a potential link between two high impact studies involving the CFTR interactome and the identification of the novel EMC complex [58,60]. To test for functional homology, CFTR-ΔF was monitored by immunoblot in the context of a TTC35 knockdown by siRNA. HeLa cells were transiently transfected with a plasmid expressing CFTR-ΔF, co-transfected with TTC35 siRNA or control siRNA, and shifted to 27°C. The shift from 37°C to 27°C was to allow adequate rescue of CFTR-ΔF protein so that we could see the detrimental impact of losing function of a presumed pro-biogenesis factor. Additionally, keeping the cells at 37°C during the knockdown of TTC35 provided elimination of CFTR-ΔF protein pools prior to TTC35 knockdown and shift to conditions where CFTR-ΔF biogenesis can occur. Under the experimental conditions performed, knockdown of TTC35 reduced CFTR-ΔF expression by 30% to 50% (Figure 7E and Additional File 1 - Additional file 1, Figure S4). Thus CFTR-ΔF processing is dependent upon expression of TTC35, validating the prediction from the yeast data for EMC involvement in biogenesis of ΔF-misfolded ABC transporters.

## Discussion

Although it is well known that genes, proteins, and pathways are conserved across evolution, conservation of interactions between genetic pathways having the potential to differentially regulate expression of phenotypes is only just beginning to be characterized in model systems [61,62]. Therefore, the clinical relevance of such networks remains to be elucidated [5,63,64]. In this regard, our data suggest the intriguing possibility that quantitative phenotypic analysis of Yor1-ΔF gene interaction reports on a complex trait in yeast of relevance to biogenesis of CFTR-ΔF508. Thus, evolutionary conservation is sufficient to usefully model human genetic disease in yeast - at least in the case of CF. This opens a door for efforts to dissect gene interaction underlying phenotypic complexity through integration of yeast phenomic data with human genetic data. A few clinically relevant genetic modifiers of cystic fibrosis disease were recently identified, however these variants are not suspected to function in CFTR protein biogenesis pathways [2]. The genetic interaction model we have developed could be useful to mine CFTR-ΔF508 GWAS data for variant alleles that that modulate disease through effects on protein biogenesis. The Yor1-ΔF model suggests the potential existence of a large number of such modifiers. Thus, the yeast phenomic model may inform human genetic studies, where systematic, comprehensive, and quantitative analysis of gene interaction is of interest. Furthermore, given the large number of interactions, it will likely be important in the future to analyze higher order epistasis networks (for example, comprehensive three-way gene-gene-gene interaction experiments), which is unforeseen employing human genetic data alone.

The outbred genetic structure of human populations, due to its combinatorial complexity, severely limits the power to analyze phenotypes with respect to gene interaction [65]. Thus, tractable yeast phenomic models could provide a powerful and complementary tool for dissecting disease complexity if the principle of evolutionary conservation of gene interaction applies [5]. Our work provides evidence in support of this concept, as we demonstrate that gene interactions discovered from the yeast Yor1-ΔF model resemble by homology gene interactions similarly characterized for CFTR-ΔF biogenesis in human cell models. The findings support the notion that even when the phenotypic manifestations of homologous gene interaction appear unrelated (for example, oligomycin resistance in yeast *vs*. maintenance of peri-ciliary fluid depth in lungs), the principle network modulating the associated phenotypes can nevertheless be similar [5,66].

We examined whether homologous modifiers of CFTR-ΔF were among the stronger Yor1-ΔF interactions (Figure 5B). Conserved interactions were not necessarily the strongest overall, raising points for consideration in future studies: (1) although strong hits from genetic screens receive the most attention, weak and intermediate strength interactions are also important for understanding the evolution of phenotypic variation; (2) the throughput and precision of Q-HTCP, which provides over 50,000 growth curves per experiment, is an enabling technology to map disease-relevant gene interaction networks, particularly regarding high quantitative accuracy to detect weak and intermediate strength interaction with high confidence; (3) high confidence measures of gene interaction across the entire genome will advance the opportunity to assess conservation of between homologs at a systems level to deduce functional modules that are most rapidly evolving within gene networks [42,67]; and (4) the elucidation of conserved aspects of a 'ΔF biogenesis network' provides a starting point to predict novel human homologs of Yor1-ΔF regulators, and ultimately define higher-order interactions from a gene network perspective [65,68]. Thus, the Yor1-ΔF phenomic model can serve in several ways as a tool to discover and prioritize targets for therapeutic development as well as potential modifiers of CF disease severity.

We chose the CFTR-ΔF508 allele causing cystic fibrosis as proof of principle for modeling a human disease-relevant gene interaction network in yeast, because CFTR-ΔF508 is arguably the best-characterized human genetic disease mutation. However, we anticipate that other CFTR mutations in addition to CFTR-ΔF508 as well as other diseases entirely can be analogously modeled in yeast to generate useful insight and new hypotheses as to how networks of interacting genes might modulate disease expression. For diseases not having a single locus that accounts for a high fraction of the phenotypic variation, the power of experimentally tractable yeast epistasis models may be even more beneficial [65]. Furthermore, yeast gene interactions also have been useful for uncovering genetic modifiers of foreign proteins; in one example, yeast gene interactions modulating alpha-synuclein toxicity uncovered homologs that functioned similarly in animal models of Parkinson's disease, even though alpha-synuclein is not encoded by yeast genomes [69]. In a second example, an informatics approach discovered 'phenologs', defined as overlapping sets of homologous genes associated with diverse phenotypic outcomes across various species, thus discovering novel genetic relationships between diverse phenotypes. Multiple predictions were validated experimentally, including homologs of genes functioning in yeast cellular resistance to HMG-CoA reductase inhibition influence angiogenesis in *Xenopus *embryos [66]. In a third example, a genome-wide screen revealed unexpectedly that threonine metabolism is required to buffer a deficiency of dNTP biosynthesis, through augmenting provision of metabolic intermediates to overcome inhibition of a key enzyme, ribonucleotide reductase [20]. Although threonine biosynthesis does not occur in multicellular eukaryotes, it was nevertheless shown that threonine catabolism is required in a developmentally-regulated way for DNA synthesis in mouse embryonic stem cells [70], and also for maintenance of stem cell chromatin state through S-adenosyl-methionine metabolism and histone methylation [71]. Our study, together with these and other models indicate the power and utility of yeast genetic screens for generating useful new hypotheses about the role of gene interaction in phenotypic diversity, including human disease [5,72].

A novel aspect of the phenomic approach described here is the acquisition and analysis of time series data from proliferating cell arrays. These data fit well to a logistic growth equation so that growth curve parameters of individual cultures can be employed to precisely and accurately quantify gene interaction (Figure 2). Coupling this method with a gradation in perturbation states (for example, multiple oligomycin concentrations) brings a new level of resolution to the powerful *S. cerevisiae *methods for analyzing gene interaction. Previous large-scale gene interaction studies have used endpoint measurements of phenotypes (for example, colony outgrowth at one time point) and binary perturbation states, which have less sensitivity for detecting gene interaction due to lower precision and accuracy of quantifying growth phenotypes [36]. The enhancement in quantitative resolution provided by Q-HTCP was significant, because many conserved interactions were intermediate in strength, and thus were more likely to have been missed by less quantitative methods (Figures 3 and 4) [13]. The validity of weak to intermediate strength interaction was further clarified biochemically in several cases (Figures 4 to 7).

The finding that gene interactions with Yor1-ΔF recapitulate homologous gene products interacting with human CFTR-ΔF in mammalian cell-based studies provides evidence that gene interaction networks can be conserved over great evolutionary distances (Figure 5). Thus, despite differential selective pressure that these distantly related ABC transporters have been subjected to, the cellular context in terms of interacting proteins that govern the biogenesis of Yor1 and CFTR is conserved and renders yeast a useful and powerful model for cystic fibrosis. Although it remains to be tested, we speculate that GWAS-based efforts to identify genetic modifiers of human disease could be aided by comprehensive and quantitative epistasis data from yeast models [2]. An integrative/comparative approach could help prioritize findings diluted by multiple comparisons from human genetic analysis. The yeast phenomic model provides a biological framework for identifying, within quantitative trait loci, candidate genes with putative functions worthy of further study [73].

As another speculative example, it is plausible that deficiency of a cargo adapter protein, such as from Erv14 deletion, could give rise to a CF-like phenotype without mutations in CFTR itself (Figure 6). That Yor1 required an ER export adaptor was in fact somewhat surprising, because we had previously correlated ER export of Yor1 with interaction between a well-characterized basic binding pocket on the surface of the vesicle cargo adaptor, Sec24, and a di-acidic export motif on Yor1 [9]. Thus a potential explanation for the present study findings is that Erv14 facilitates the Yor1/Sec24 interaction. CFTR also employs a di-acidic motif, albeit in a distinct domain from that of Yor1, and Erv14 is well conserved in metazoans [74], and therefore a similar mechanism of ERV14 facilitating interaction during capture into transport vesicles is plausible for selection of CFTR into ER-derived vesicles, and remains to be tested.

A potentially clinically relevant outcome of our study was the discovery of a novel function for the recently described ER membrane complex. The EMC was discovered in a screen to find ER folding factors in yeast [58]. We now show that deletion of any one of the members of the evolutionarily conserved protein complex yields a quantitatively similar deletion enhancer phenotype with respect to Yor1-ΔF biogenesis (Figure 7). Interestingly, this interaction effect appears specific for the misfolded protein only, as deletion of members of the EMC did not affect oligomycin sensitivity in the context of wild-type Yor1 expression. Further studies are needed to clarify these findings, however we postulate a role for the EMC in the early secretory pathway, and suspect it acts in a pro-biogenesis manner as part of the co-translational mechanism - perhaps for proteins prone to misfolding. We did not see a role for the EMC proteins in protein turnover, since the half life or Yor1-ΔF was identical either in the presence or absence of their expression. Instead, we observed in the *sop4-Δ0 *mutant a reduced rate of production of Yor1-ΔF (Figure 7).

Consistent with the above hypothesis, it was previously noted that deletion of the EMC proteins yields a genetic interaction profile similar to over-expression of the *sec61-2 *mutation; thus, deletion of the EMC mimics genetic perturbation of the Sec61 translocon. Furthermore, deletion of *UBC7 *or *CUE1 *(genes functioning in ERAD) was aggravating in combination with deletion of either the *EMC *genes or *sec61-2 *overexpression [58,75]. Our interpretation of these data is that EMC and Sec61 act in a functionally distinct pathway from ERAD, pathways that can buffer loss of one another [5,76]. Other evidence suggesting a role for the EMC in the early secretory pathway comes from a high content microscopy screen, which discovered loss of the EMC causes increased ER retention of the Mrh1-GFP fusion protein [77]. Importantly, we note that the role of the EMC and other secretory protein biogenesis network factors appears cargo-specific, since other factors that were found in the Mrh1-GFP screen exerted qualitatively different effects in our Yor1-ΔF screen [77]. From a detailed comparison of our screen with the list of genes described by Bircham *et al*. to be required for forward transport of Mrh1-GFP, we noted that the *EMC *genes and *SOP4 *were ΔF-specific deletion enhancers; *GYP1*, *RAV2*, *VAC14*, and *MON2 *were ΔF-specific deletion suppressors; *PKR1 *was a non-specific deletion enhancer; and most other genes (*GOS1*, *PEP4*, *SPF1*, *VPS51*, *VPS53*, *VPS60*, *VTA1*, *YPT6*, and *OPI3*) showed no effect. Thus, while several genes were found in both studies, only loss of function alleles of the EMC complex appeared to have a consistent effect on prohibiting biogenesis of membrane proteins. Furthermore, for Yor1, prohibited biogenesis was specific to the misfolded Yor1-ΔF.

To test whether the EMC functions in a conserved manner as a pro-biogenesis factor for CFTR-ΔF, we knocked down *TTC35/EMC2 *in transfected HeLa cells expressing CFTR-ΔF under temperature rescue conditions. Since we did not observe an effect of disrupting the EMC on Yor1-ΔF turnover, but rather a defect in Yor1-ΔF production, we tested for a pro-biogenesis function of EMC2 on temperature-rescued CFTR-ΔF. We found that loss of EMC2 reduced the steady state level of CFTR-ΔF, consistent with our Yor1-ΔF findings. These results provide a strong rationale to utilize both yeast and human cells to clarify the pro-biogenesis mechanism for the EMC on ΔF-misfolded proteins (Figure 7).

In summary, the datasets provided here will serve as a resource for further identification and prioritization among candidate genes and pathways contributing to cellular processing of misfolded proteins. Novel cellular pathways in addition to the ones discussed, were suggested by this study to be of importance for biogenesis of misfolded ABC transporter proteins and include mRNA processing (for example, SKI complex) and ribosome-associated functions, both of which were strong Yor1-ΔF-specific deletion suppressors. The similarity in their impact on Yor1-ΔF biogenesis could occur by influencing rates of translation and/or altering protein-folding dynamics, although other mechanistic explanations are plausible. Future studies will be required to clarify the role of these and other genes in Yor1-ΔF biogenesis and their potential relevance to CFTR-ΔF. Additionally, given the abundance of pair-wise interactions revealed in our study, three-way interaction analysis will become increasingly important to understand functional hierarchies in higher-order epistasis networks that modulate Yor1-ΔF and, by extension, CFTR-ΔF protein biogenesis.

## Conclusion

The Yor1-ΔF670 gene interaction network was found to be representative of CFTR-ΔF protein regulators identified from human cell models. In addition, multiple new functional categories of proteins were found to modulate the activity of Yor1-ΔF, suggesting potential importance of their homologs for CFTR-ΔF biogenesis. Validation of Yor1-ΔF interactors using biochemical assays provided confidence in the functional significance of the screening results, and led to the discovery that an evolutionarily conserved ER membrane complex similarly impacts biogenesis of Yor1-ΔF and CFTR-ΔF. The overall result suggests quantitative phenotyping of double mutant yeast expressing Yor1-ΔF is useful for modeling an evolutionarily conserved gene interaction network functioning to modulate CFTR-ΔF biogenesis. The clinical relevance of the Yor1-ΔF gene interaction network to cystic fibrosis remains to be established in patients. Yet in principle, Q-HTCP affords a general platform to leverage the power of yeast genetics for exploring the influence of gene interaction using other yeast phenomic models of disease. The approach could be extended, for example, to other cystic fibrosis-relevant mutations in Yor1, other molecular models of protein misfolding related disease, and homologous mutations in proteins covering a wide range of molecular functions where the cellular basis of disease involves evolutionarily conserved processes.

## List of abbreviations

ABC: ATP-binding cassette; CF: Cystic fibrosis; CFTR: Cystic fibrosis transmembrane conductance regulator; ER: endoplasmic reticulum; ERAD: ER-associated degradation; EMC: ER membrane complex; GO: Gene ontology; GWAS: Genome-wide association studies;P-POD: Princeton Protein Orthology Database; Q-HTCP: Quantitative high throughput cell array phenotyping; REMc: Recursive expectation-maximization clustering; SGA: Synthetic genetic array; UPR: Unfolded protein response; UPRE: Unfolded protein response element

## Competing interests

JLH has a financial interest in Spectrum PhenomX, LLC, which holds a license to commercialize Q-HTCP technology. All other authors declare no competing interests.

## Authors' contributions

EAM and JLH conceptualized the project and wrote the manuscript. JG and RJL performed the screen. JWR and JLH carried out the Q-HTCP analysis. RW, JLH, and JB devised the interaction model. JG, JLH, and BAM performed the REMc analysis. ML, JLH, and KD performed the homology mapping. RJL, SP, PK, and EAM carried out the molecular validation experiments in yeast, and JH and EJS performed the RNAi experiments in HeLa cells. All authors read and approved the final manuscript.

## Supplementary information

There are five additional files. Additional File 1 contains one table and four figures, as well as three supplemental discussion sections. All of the interaction data are available in Additional Files 2, 3, and 4. REMc clustering results are provided in Additional File 5, and high confidence Yor1-ΔF interactions submitted to BioGRID are indicated in column L of the 'REMc_data and clustering' worksheet. The criteria for selecting genes as high confidence are described in the 'readme' page of Additional File 5. Only high-confidence, manually reviewed interactions (instead of all interactions beyond a certain quantitative threshold) were submitted to BioGRID (http://thebiogrid.org), for inclusion in the BioGRID database and SGD (http://yeastgenome.org). Interactions that were considered lower confidence were excluded based on criteria such as a large effect of the gene deletion on growth in the absence of oligomycin or if gene-drug interaction occurred in the presence of wild-type Yor1 expression, or if the dose response of interaction across all oligomycin concentrations was not well fit to the quadratic equation.

## Supplementary Material

Additional File 1 **This file contains three supplemental discussion sections, one table and four figures**. Click here for file

Additional File 2 **This file contains tables of screen-related data**. Two screens were performed in the genomic collection of non-essential gene knockouts. One screen was with the unmodified collection (wild-type *YOR1 *allele), and the other was with the *yor1-ΔF *allele that was introduced by the SGA method. Results from each screen are given in two sheets. The first sheets (named '_screen') have columns indicating: (A) the oligomycin concentration; (B) name of the ORF; (C) name of the gene deletion; (D) area under the growth curve; (E) the carrying capacity ('K') from the logistic growth equation fit; (F) the rate ('r') from the logistic growth equation fit; (G) 'L' from the logistic growth equation fit; (H) R-squared value indicating residual after logistic growth fitting; and (I-N) upper and lower bounds (95% confidence intervals) on the logistic growth curve parameters. The second sheets (named '_interactions') contain the following fields: (A, B) ORF and gene name for deletion strain; (C) 'ORF effect'- indicating the difference in L between the double mutant and the median of 768 replicate *yor1-ΔF *single mutant cultures grown in the absence of oligomycin; (D) the interactions quantified by quadratic fitting of the difference in L between double mutants and the median of 768 replicate *yor1-ΔF *single mutant cultures, across increasing concentrations of oligomycin, and taking the difference at the highest oligomycin concentration observed for the double mutant; and (E) the number of high oligomycin concentrations where no growth was observed for the deletion mutant (that is, deletion enhancer effects). Click here for file

Additional File 3 **This file contains graphs of oligomycin response for deletion strains in background of wild-type *YOR1 *or *yor1-ΔF*, searchable by gene and ORF names**. The graphs are ordered by descending interaction score for *yor1-ΔF *double mutants, grouped also by the number of high oligomycin concentrations at which no growth was observed (that is, from strongest deletion enhancer to strongest deletion suppressor). 'miss' indicates the number of high oligomycin concentrations at which no growth occurred; 'ORF' indicates the difference in L between the deletion mutant and its wild-type reference strain; 'int' is the interaction quantity at the highest oligomycin concentration observed; 'DR' indicates the difference between the ranks of the deletion mutant in each of the two screens to indicate differential interactions in the context of wild-type Yor1 *vs*. Yor1-ΔF. The filled symbols indicate the raw 'L' value at the zero-oligomycin concentration for the deletion mutant, by which all L values for the deletion mutants were adjusted to quantify the interaction. The gray diamonds represent the 95% central distribution of 768 replicates of the reference strain, from which the dose effect at each oligomycin concentration has been removed. Click here for file

Additional File 4 **Like Supplemental Data File 2, this file contains graphs of oligomycin response for deletion strains in background of wild-type *YOR1 *or *yor1-****Δ**F***. Herein, less-interactive and non-interactive genes have been included for completeness. Click here for file

Additional File 5 **This file contains input data used for REMc, REMc results, and results of GO Term Finder analysis of REMc clusters**. The sheet 'REMc_data and clustering' contains the following columns of data: (A) an arbitrary cluster # indicating discrete clusters for each gene; (B) the ORF and (C) gene name for the deletion strain; (D) the root and (E) round 1, (F) round 2, and (G) round 3 cluster names; (H-K and M-V) the interaction values for each gene-perturbation combination, for which the column labels are described in order in methods and materials; column L contains gene interactors submitted to BioGRID. The sheet 'cluster summary' contains the following columns of data: (A) REMc cluster ID; (B) the number of genes in the cluster; (C) the log-likelihood of the cluster; (D) the GOid_z (Gene Ontology information divergence score), a measure of functional enrichment (across all GO terms) among genes in each cluster; (E) the number of enriched GO Terms for each first round cluster; (G-K) REMc data for Round 2 clusters; (M-Q) REMc data for Round 3 clusters. The sheet 'GTF' contains the following columns of data: (A) REMc cluster ID; (B) 'selective' GO Terms, meaning terms empirically chosen to highlight relatively specific cellular processes to which a small number of gene functions is ascribed. These are highlighted as hypothesis-generating terms worthy of further validation due to the presence of multiple genes from the same biological module exerting similar patterns of gene-gene and gene-drug interaction. Manual review indicated in the 'REMc_data and clustering' sheet (column M) refers to interactions that were filtered out before submission to BioGRID based on inspection of the interaction graphs in Supplemental Data File 2 with attention to the following criteria: (1) the oligomycin dose response was uncharacteristic (for example, reaching a plateau); (2) the dose response was not well fit to the quadratic; (3) the Yor1-ΔF double mutant strain was very slow growing in the absence of oligomycin; (4) the deletion mutant in the context of Yor1 wild type suggested that oligomycin drug interaction was independent of Yor1-ΔF expression; (5) weak effects. Click here for file
