# Supplementary material for: A yeast phenomic model for the gene interaction network modulating CFTR-ΔF508 protein biogenesis
Source: Genome Med. 2012 Dec 27;4(12):103. doi: 10.1186/gm404 (PMC3906889; doi:10.1186/gm404)
Supplement: Additional File 1 — This file contains three supplemental discussion sections, one table and four figures. [file gm404-S1.DOCX]

**Additional File 1: Outline**

**1. Supplemental Results/Discussion Topics in Additional File 1**

**a. Characterization of the folding and ER export of Yor1, Yor1-ΔF and Yor1-ΔF/R1116T**

**b. Use of the ‘L’ parameter for quantifying interactions**

**c. Conservation of gene interactions influencing CFTR-ΔF and Yor1-ΔF biogenesis**

**2. Supplemental Table and Figures in Additional File 1**

1. **Table S1.** Summary of REMc results presented in Additional File 5.
2. **Figure S1.** The functional effect of the R1116T on wild type Yor1 pump function.

**c.** **Figure S2.** Reproducibility of genome-wide screen.

**d. Figure S3:** Correlation between gene deletions that activate the UPR or reduce Yor1-∆F biogenesis.

**e. Figure S4:** A second representative experiment demonstrating the requirement of TTC35 expression for CFTR-∆F biogenesis.

**Supplemental Results/Discussion A: Characterization of the folding and ER export of Yor1, Yor1-ΔF and Yor1-ΔF/R1116T**

CFTR-∆F508 (CFTR-DF) maintains residual chloride channel activity when rescued to the cell surface, despite the misfolding and trafficking defect. However, this did not appear to be true for Yor1-∆F670 (Yor1-DF), since the mutant was as sensitive to oligomycin as the *yor1-∆0* strain (**Figure 1A**). A second site mutation, Yor1-∆F670-R1116T restored partial oligomycin resistance, so we investigated the molecular nature of this mutation. We used several biochemical assays to directly monitor the folding, assembly, forward transport and kinetic stability of Yor1, Yor1-ΔF and Yor1-ΔF/R1116T (**Figure 1**). An *in vitro* vesicle budding assay that recapitulates COPII vesicle formation from the ER membrane was used to directly monitor release of proteins from the ER [1]. Cells expressing HA-tagged forms of wild type or mutant Yor1 were radiolabeled, permeabilized and incubated with purified COPII coat proteins, which represent the minimal machinery required to generate ER-derived transport vesicles. These COPII transport vesicles were separated from the donor membranes by differential centrifugation and capture of cargo proteins into vesicles analyzed by immunoprecipitation, SDS-PAGE and autoradiography. Wild-type Yor1 was efficiently captured into COPII vesicles in the presence of GTP, whereas Yor1-ΔF was not detected in the vesicle fraction (**Figure 1B**). Yor1-ΔF/R1116T also failed to enter into COPII vesicles, suggesting the R1116T mutation does not rescue forward trafficking. Consistent with inefficient capture into ER-derived COPII vesicles, Yor1-∆F/R1116T was predominantly localized to the ER, similar to Yor1-ΔF, as detected by fluorescence microscopy of the GFP fusions (data not shown).

We further confirmed that the R1116T mutation does not rescue the aberrant fold of Yor1-∆F by subjecting membranes containing either wild type or mutant Yor1 to limited proteolysis, using increasing concentrations of trypsin to cleave Yor1 into smaller fragments that were analyzed by immunoblotting (**Figure 1C**). In the presence of increasing concentrations of trypsin, wild-type Yor1 was cleaved to two relatively stable ~90 kDa and 60 kDa bands whereas Yor1-ΔF was more susceptible to proteolytic attack, yielding a number of smaller fragments (**Figure 1C**). The increased susceptibility of Yor1-ΔF to trypsinolysis mirrors that of CFTR-ΔF and is consistent with the hypothesis that the mutant protein is improperly assembled, exposing additional trypsin cleavage sites that are obscured in the correctly folded protein. The trypsin digest profile of Yor1-ΔF/R1116T was most similar to that of Yor1-ΔF, suggesting that the R1116T substitution did not dramatically rescue the global conformation of the protein.

A third biochemical approach uses chemical cross-linking to probe the assembly of the transmembrane domains (TMDs) of Yor1 [2]. Similar studies on CFTR and P-glycoprotein introduced cysteine residues into specific TMDs to detect inter-domain cross-links that indicate an intimate association between TMDs [3]. Misfolding mutations, including the ΔF lesion, result in an inability to form these cross-links, suggesting that these TMDs fail to assemble correctly. We introduced cysteine residues at specific positions in the 6^th^ and 12^th^ TMDs of Yor1, equivalent to the sites used for CFTR. Membranes expressing wild-type *YOR1* that contained cysteine substitutions at F481 (TM6) and L1162 (TM12) were exposed to a methanethiosulfonate cross-linker with a 13-Å spacer arm, and the mobility of the protein was monitored by non-reducing SDS-PAGE and immunoblotting. On exposure to cross-linker, a species with reduced mobility was detected, similar to that observed for CFTR. This cross-linked species likely represents an intramolecular modification, because when the individual F481C and L1162C substitutions were introduced into Yor1 separately on two different plasmids and co-transformed into cells, no cross-linked proteins were detected [2]; instead, the unmodified protein disappeared and the majority of the protein presented as a very high-molecular weight aggregate that largely failed to enter the resolving gel [2]. Yor1-ΔF/R1116T closely resembled Yor1-ΔF in these experiments, with no detectable cross-linked species but an abundance of high molecular weight aggregates (**Figure 1D**).

Finally, we probed the kinetic stability of Yor1-ΔF/R1116T *in vivo*: a hallmark of Yor1-ΔF, and misfolded proteins in general, is that they display remarkable instability, being rapidly turned over by the cytoplasmic ubiquitin/proteasome system [2, 4]. We asked whether the turnover of Yor1-ΔF/R1116T more closely resembled that of wild-type Yor1 or Yor1-ΔF. Cells expressing HA-tagged forms of Yor1 were pulse-labeled, then chased with non-radioactive amino acids for several hours. Cells were removed at various time points and the amount of Yor1 remaining was calculated by immunoprecipitation and Phosphor-Image analysis. As expected, Yor1-ΔF was much less stable than wild-type Yor1, and Yor1-ΔF/R1116T displayed the same degradation kinetics as Yor1-ΔF (**Figure 1E**).

Together, the localization, folding and stability assays described above suggest that the “rescued” form of Yor1-ΔF, Yor1-ΔF/R1116T, is a misfolded protein, subject to the same ER quality control processes that regulate the membrane delivery of Yor1-ΔF. Given that the R1116T mutation lies remote from the primary ΔF670 lesion, we considered the possibility that R1116T has a direct effect on pump activity, partially restoring the protein’s function to clear oligomycin from the cytosol. According to this hypothesis, a small amount of Yor1-ΔF is capable of leaving the ER and is delivered to the plasma membrane. If this protein pool acts as an efficient drug pump, then sufficient toxin can be cleared to yield an increase in fitness. We used a rhodamine-pumping assay, which monitors ATP-dependent release of a fluorescent dye from intact cells, to test this hypothesis. Although rhodamine is a substrate for Yor1, another ATP-Binding Cassette (ABC) transporter, Pdr5, is the predominant pump that clears rhodamine from cells. Therefore, a strain with chromosomal deletions at both the *YOR1* and *PDR5* loci was used to measure the rhodamine pumping effect of wild type and mutant forms of Yor1 introduced on plasmids. Similar to the oligomycin phenotype, Yor1-ΔF/R1116T conferred intermediate rhodamine resistance in a *yor1-Δ0 pdr5-Δ0* strain (data not shown). To assay rhodamine efflux, cells were starved of ATP by incubation with 2-deoxyglucose, during which time rhodamine was loaded into cells by passive diffusion. Cells were washed and glucose added to initiate ATP-dependent pumping activity. Aliquots of the culture were removed over time and the amount of rhodamine in the culture supernatant was measured using a spectrophotometer. Cells expressing *yor1-ΔF* exhibited a very low level of rhodamine release, equivalent to that of the *yor1-Δ0 pdr5-Δ0* parental strain. However, Yor1-ΔF/R1116T conferred a small amount of rhodamine pumping activity, consistent with the intermediate growth phenotypes associated with this additional mutation (**Figure 1F**). The exact mechanism remains to be determined, though it is perhaps worth noting that, by multiple sequence alignment, the R1116 residue is conserved among ABC transporters, including CFTR (R1097) and another related yeast ABC transporter, Ycf1p (R1174). We note there is a slight upward inflection of rhodamine transport at 15 minutes in the assay: this was a small, though somewhat recurrent observation, for which we do not know the mechanism.

Although the R1116T mutation does not affect the oligomycin-sensitivity phenotype, nor does it alter the capacity of Yor1 for pumping rhodamine at room temperature relative to wild type Yor1, we also tested the rhodamine pumping assay at 10 degrees, aiming to slow pump action to potentially observe an otherwise undetectable difference in enzyme activity. Indeed, at the reduced temperature we see an effect of the R1116T mutation on the wild type protein, suggesting that it improves pump efficiency (**Figure S1**). Taken together with data in **Figure** **1** (showing that the misfolding and trafficking defects are similar for both Yor1-∆F670 and Yor10∆F670-R1116T), the temperature effect on the Yor1-R1116T function supports a model where the mutation increase pump efficiency, which is separable from the misfolding defect induced by ∆F670. Thus, R1116T enables detection of modifiers of the oligomycin phenotype associated with the ∆F670 mutation by virtue of partially restoring oligomycin pumping activity of the misfolded Yor1-∆F670 protein, which otherwise could not be detected by a phenotypic assay; however, in the absence of the ∆F670 mutation, R1116T has no phenotypic effect.

Thus, the R1116T mutation improves the functionality of Yor1-ΔF, improving cellular resistance to oligomycin over that of Yor1-ΔF, which is equivalent to that of a *yor1-Δ0* null strain. The intermediate phenotype conferred by this mutation provided an opportunity to identify genes that modulate the biogenesis of Yor1-∆F in both a positive and negative manner.

The identification of candidate regulators serves as a starting point to confirm aspects of the yeast model relevant to CFTR-∆F trafficking, through gene homology analysis and literature mining. On the other hand, biochemical assays can be used to assess the fate of Yor1-ΔF. Thus, the combined genetic and biochemical analyses, afford an opportunity to validate existing and to map new pathways of protein folding and ER quality control that regulate the biogenesis of eukaryotic ABC transporters.

**Supplementary Results/Discussion B: Use of the ‘L’ parameter for quantifying interactions:**

The carrying capacity (K), the rate (r) and lag (L) are parameters of the logistic growth equation to which we fit our time series data of cell proliferation [5]. We found in this study that L, which represents the time it takes for a culture to grow to half its carrying capacity, reflected most accurately and precisely growth inhibition due to oligomycin (**Figure 2**). The area under the growth curve (AUGC), which we have used previously, was next most informative, but is more cumbersome to work with, since it is a time x density area affected by all three parameters, while the L value is a discrete measure of time. Accordingly, interaction values calculated with the L value correlated strongly (R^2^ =-0.91) with interaction values calculated by AUGC, whereas correlations for interaction values calculated with other parameters (rate and carrying capacity) were weaker (R^2^ <0.5 or >-0.5).

Inherent to the logistic model is an assumption that the growth rate is maximal at time = zero, however for practical purpose the biological lag time can be accounted for in the model by not constraining N_0_, the population size at t=0. Although we do not know the precise number of initial colony forming units (N_0_) for each culture, they should be approximately equal since cultures are all grown to a saturating density in liquid media before being diluted and spotted. To correct for growth differences between the single and double mutants not attributable to oligomycin response, L values for each gene deletion strain are normalized by its difference with the reference strain in the control media (this distance is indicated by the solid symbols in **Additional File 3**). Thus, by maintaining the real N_0_ relatively constant for all cultures by pre-growing all strains to saturation phase, but not constraining N_0_ in the model, and since the method is sensitive enough to rate measures directly (i.e. during the logarithmic growth phase), alterations in L reflect not only changes in rate, but also physiological adaptation time. In this study, the growth delay induced by oligomycin did not seem to be much reflected in the actual growth rate, thus it appeared to be attributable to physiologic lag, or adaptation time.

**Supplemental Results/Discussion C: Evolutionary conservation of genes regulating CFTR-ΔF508 and Yor1-ΔF670 biogenesis**

Through homology search and literature mining, we identified several biologically relevant classes of Yor1-∆F deletion interactors that correspond to known CFTR-∆F protein regulators, as described below:

*Syntaxins*

STX1A was among the first protein regulators of CFTR to be identified [6], and involvement of additional syntaxins has made this an important biological theme for understanding CFTR function and processing [7]. STX1A has been shown to regulate wild type CFTR channel activity directly in addition to its role in regulating vesicle fusion and protein delivery [8, 9]. The yeast homolog of STX1A, *SSO2*, appeared to negatively influence Yor1-ΔF function (**Figure 5**), however the effect was not seen in the context of wild type Yor1 expression (**Additional File 3**), as described for CFTR [8]. Deletion suppressor phenotypes were also observed in the *vam7-∆0* or *snc1-∆0* strains, specifically in the context of *yor1-∆F*, consistent with the effects seen for *sso2-∆0*. The human homologs of *VAM7* and *SNC1,* STX8 and VAMP8, both function, like STX1A, in negative regulation of CFTR [10, 11]. Interestingly, recent findings from the CF Foundation Folding Consortium (EJS, unpublished data) indicates that RNAi-mediated knockdown of STX8 and STX1A robustly rescues cell surface expression of CFTR-∆F at physiologic temperature in CFBE cells (a bronchial epithelial cell line derived from a cystic fibrosis patient). Thus, interactions of these homologous syntaxins appear to be conserved with respect to CFTR-∆F and *yor1-∆F*. By contrast, yeast *TLG2*, was a deletion suppressor of Yor1-∆F-associated oligomycin sensitivity, however knockdown of the human homolog STX16 promoted CFTR biogenesis, representing an apparent point of functional divergence [12]

*Rab protein-regulated endocytic vesicular trafficking*

Rab family proteins regulate vesicular trafficking, and among these RAB5, RAB7, RAB9 and RAB11 regulate CFTR-∆F endocytosis and plasma membrane recycling [13]. Yeast *VPS21* and *YPT7*, the homologs of human RAB5 and RAB7, influenced the phenotype associated with misfolded Yor1-ΔF in a manner analogous to effects upon CFTR-∆F (**Figure 5**). Human RAB9 does not have a yeast homolog. Deletion of yeast *YPT31* or *YPT32*, two yeast paralogs homologous to Rab11 (which rescues CFTR-ΔF when over-expressed), did not influence the Yor1-ΔF phenotype, perhaps due to redundancy between Ypt31 and Ypt32 [14]. However, some conservation of a RAB11-linked pathway was suggested by the finding that *MYO4* exerted a phenotypic influence consistent with the function of its human homolog MYOSIN-5, which functions in a RAB11-specific way to negatively regulate CFTR-ΔF biogenesis [15].

*ER Quality Control, Chaperones, and ERAD*

One third of eukaryotic proteins traverse the ER during biogenesis en route to their cellular locations [16]. CFTR has served as a model for understanding molecular mechanisms by which cells coordinate protein quality assurance while regulating transport, and the efficiency with which wild type CFTR is processed or turned over varies 2-3 fold between different human cell types [17, 18] Moreover in yeast, wild type CFTR is turned over by ER-associated degradation (**ERAD**), rather than being trafficked to the plasma membrane [19]. Despite the variable handling of wild-type CFTR, yeast has been a useful model for understanding chaperone requirements for CFTR ERAD [20], just as different mammalian cell types have revealed complementary information about chaperones that function in ER quality control processes. In this regard, our *yor1-∆F* gene interaction screen provides a comprehensive and quantitative assessment of epistasis useful for specifying functionally homologous interactive genes within expansive gene families, such as those regulating protein trafficking. Below, we note examples of yeast-human conservation among ‘∆F-biogenesis factors’ that appear to function in ER quality control and/or influence trafficking at the cell periphery [21].

Members of the Hsp70 family participate in protein quality control and ERAD; disruption of their function can slow degradation and increase the amount of aberrant CFTR-ΔF at the plasma membrane [19, 22]. Accordingly, the cytosolic Hsp70, *SSA2*, was identified as a deletion suppressor in our screen. However, evolutionary expansion of the Hsp70 family has rendered computational assignment of gene homology ambiguous due to the presence of numerous paralogs. However, Hsp70 proteins are known to display substrate specificity [20]; and indeed knockout of *SSA1* (the Hsp70 regulating ERAD of CFTR expressed in yeast [19]) was not a deletion suppressor in our screen, nor did other non-essential Hsp70s impact the phenotype associated with Yor1-ΔF. *SSA2* was the strongest Yor1-∆F interactor among the yeast Hsp70 proteins, and thus could be considered functionally homologous to knockdown of HSPA8 (Hsc70), which had the strongest effect on CFTR-ΔF in a recent study [21]. HSP90 proteins comprise another pertinent chaperone family, and we found that knockout of yeast *HSP82*, but not *HSC82*, promoted Yor1-ΔF biogenesis. Interestingly, this seeming divergence in the function of paralogous genes was also seen in the case of CFTR-ΔF biogenesis, where knockdown of Hsp90A but not Hsp90b augmented functional expression of CFTR-∆F in human cells [21].

The degradative fate of proteins subjected to ER quality control is signaled by ubiquitination, followed by retrotranslocation, proteasomal targeting, and degradation [20]. HSP40 co-chaperones (J-domain proteins) are exchange factors that cooperate with the HSP70 and HSP90 ATPases to facilitate entry into the ubiquitination pathway [23]. DNAJC5/CSP, DNAJB12, and DNJB2 are human HSP40 paralogs for which RNAi-mediated knockdown of either protein increases CFTR-ΔF biogenesis [24-26]. Accordingly, our data for *HLJ1*, a yeast homolog for both proteins, suggests it exerts a similar influence on Yor1-∆F biogenesis (**Figure 5)**. Degradation of CFTR-∆F is promoted by human RMA1, a ubiquitin E3 ligase that cooperates with Hsc70 and DNAJB12 [24]. RMA1 has no homolog in *S. cerevisiae*, however deletion of the yeast E3-ligase, *TOM1* mimicked knockdown of its human homolog, HACE1, an E3 ubiquitin ligase, which promotes biogenesis of CFTR-∆F508 [21]. *YDJ1*, a yeast Hsp40 residing in a different homology cluster from *HLJ1/Dnajb12/Dnajc5* was a deletion enhancer of oligomycin sensitivity (**Additional File 3**), suggesting it counteracts ERAD of Yor1-ΔF, analogous (but not homologous) to HRD1 which negatively regulates RMA1 in CFTR-ΔF degradation. As a final example, knockout of yeast *UBC13* (**Figure 5**) and knockdown of mammalian UBC13 [21] indicated conservation of the functional influence of these homologous E2 ubiquitin-conjugating enzymes.

Most of the conserved gene interactions we found, as described above, were deletion suppressors, which is the class of interactions most reported in the CFTR literature. However, we also discovered the recently described EMC as a deletion enhancer of Yor1-∆F and then validated a conserved role for it in CFTR-∆F biogenesis. The *lhs1-∆0* strain also appeared as a deletion enhancer, suggesting the presence of additional pro-biogenesis factors acting on Yor1-∆F at the ER membrane. Lhs1p is an ER resident HSP70 family member, which functions with Kar2p for ER protein translocation [27]. The *lhs1-∆0* null mutation, like the *emc* null strains, was associated with UPR induction [28] and enhanced oligomycin sensitivity specifically in the context of the Yor1-∆F (see **Additional File 3)**; in contrast, deletion of *SIL1*, a second HSP70 ATPase that cooperates with KAR2/BiP in protein translocation induced the UPR, but exerted a deletion suppressor phenotype [27, 28]. Taken together, the results indicate that multiple proteins associated with ER protein translocation differentially affect Yor1-∆F, and that these effects cannot be simply explained by UPR activation. Moreover, this example makes an important point that even hits that do not have recognizable homologs can point to relevant cellular processes based on their known functions in yeast. *SIL1* and *LHS1* both serve as nucleotide exchange factors for *KAR2*, but have been show to operate with mechanistically distinct functions [27]. Thus, even though *SIL1* does not have a human homolog, its know relationships to *KAR2* and *LHS1* together with the fact that it exerts an opposite effect than *LHS1* on the Yor1-∆F phenotype lends additional credence to *LHS1* and its human homolog, HYOU1, as functionally homologous quality control factors.

*Different trajectories of interaction strength across increasing oligomycin concentration*

We note that genes have different trajectories of interaction strength. For example, the *vam7-∆0, vps21-∆0*, and *hlj1-∆0* mutants show gradual increase in their interaction strength with increasing oligomycin concentration, while *snc1-∆0, myo4-∆0*, and *ssa2-∆0* interact more strongly at the highest oligomycin concentration (**Figure 5**). Since the different oligomycin concentrations serve as a sort of replicate test for every gene deletion strain, we tend to prioritize hits with steady trajectories like *vam7-∆0, vps21-∆0,* and *hlj1-∆0*, over hits with late trajectory like *snc1-∆0, myo4-∆0*, and *ssa2-∆0*. However, the trajectory itself could be indicative of biologically distinct type of gene interaction, an area for future investigation.

*Non-modularity among conserved interactors*

The yeast genes described above, having functional homologs relevant to CFTR-∆F trafficking, were distributed across several clusters, indicating they are not, as a group, highly modular with respect to their broader phenotypic influence, i.e., though a few cluster together, together they exert diverse influence on phenotypic responses to different cellular perturbations.

**Table S1. Summary of REMc results [29].**


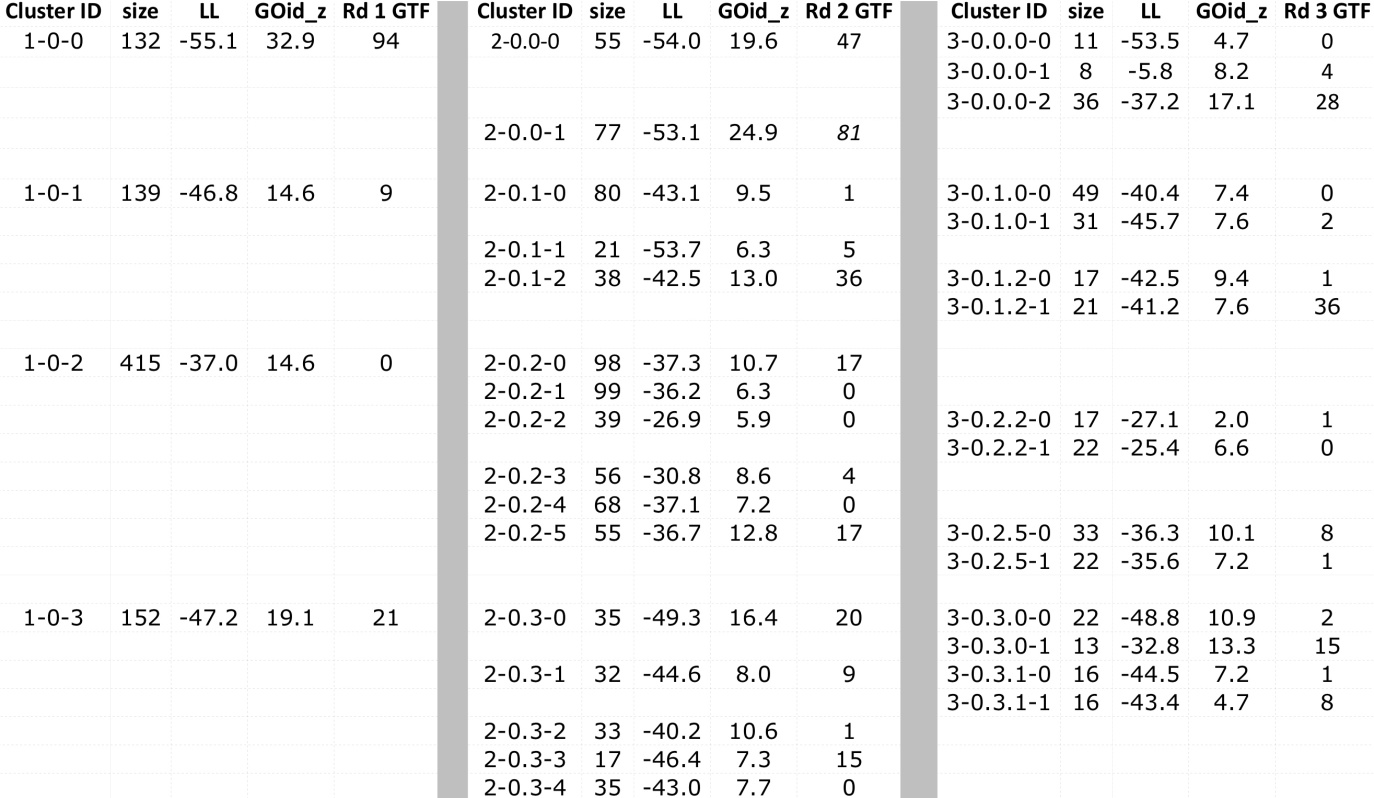


**Supplemental Figures**


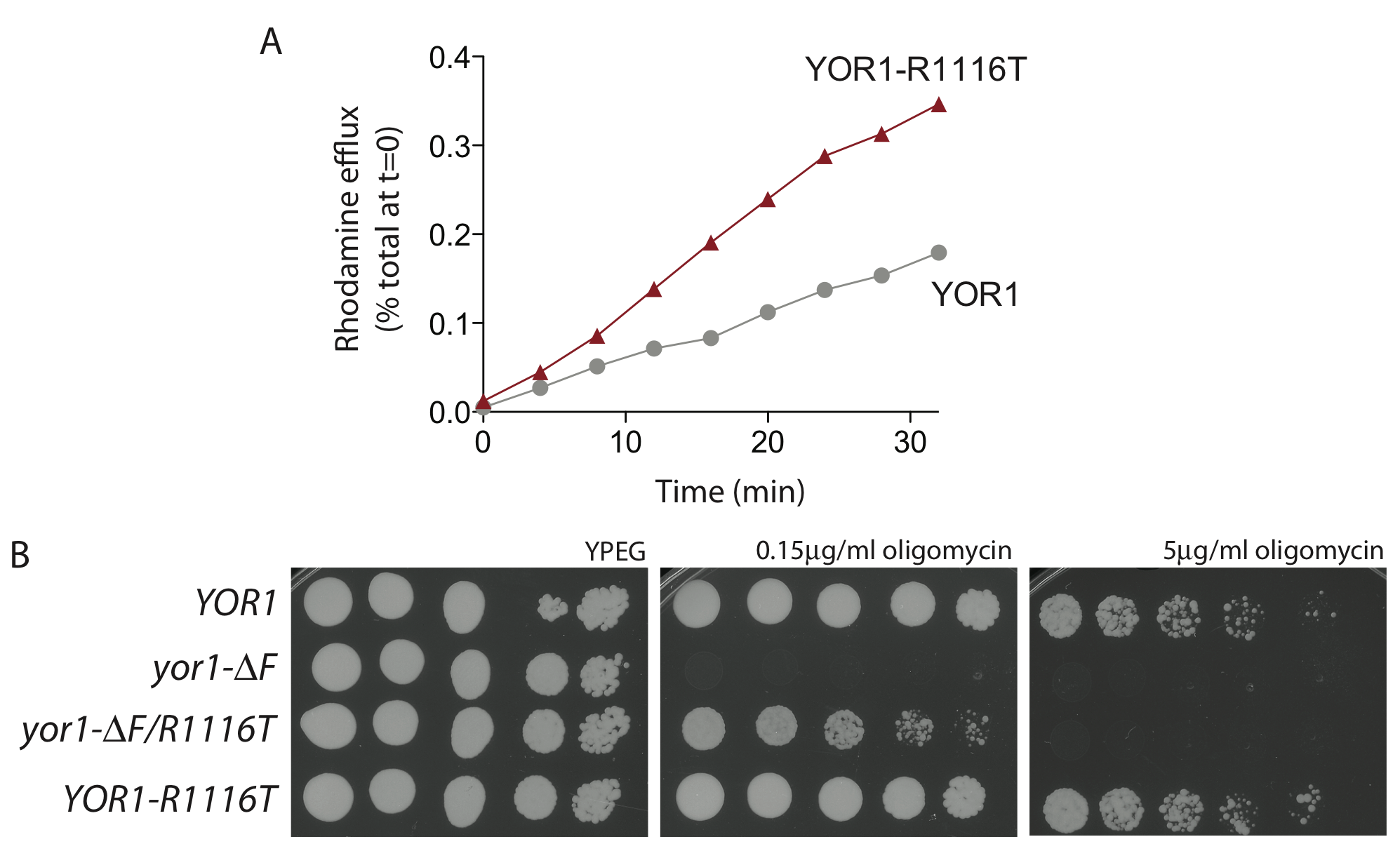


**Supplemental Figure S1**: *Effect of R1116T mutation on function of wild type Yor1 rhodamine efflux activity.* Yor1-R1116T function (see also **Figure 1F**), was assayed for effect on rhodamine efflux at a reduced temperature of 10^o^ C (Panel A), and effect on the growth phenotype (Panel B). A effect on increasing pump function can be seen *in vitro* at reduced temperature, but this is not seen in the phenotype when cells are grown at physiologic temperature, where the wild type protein functions as well with or without the augmentation-of-function mutation, R1116T.


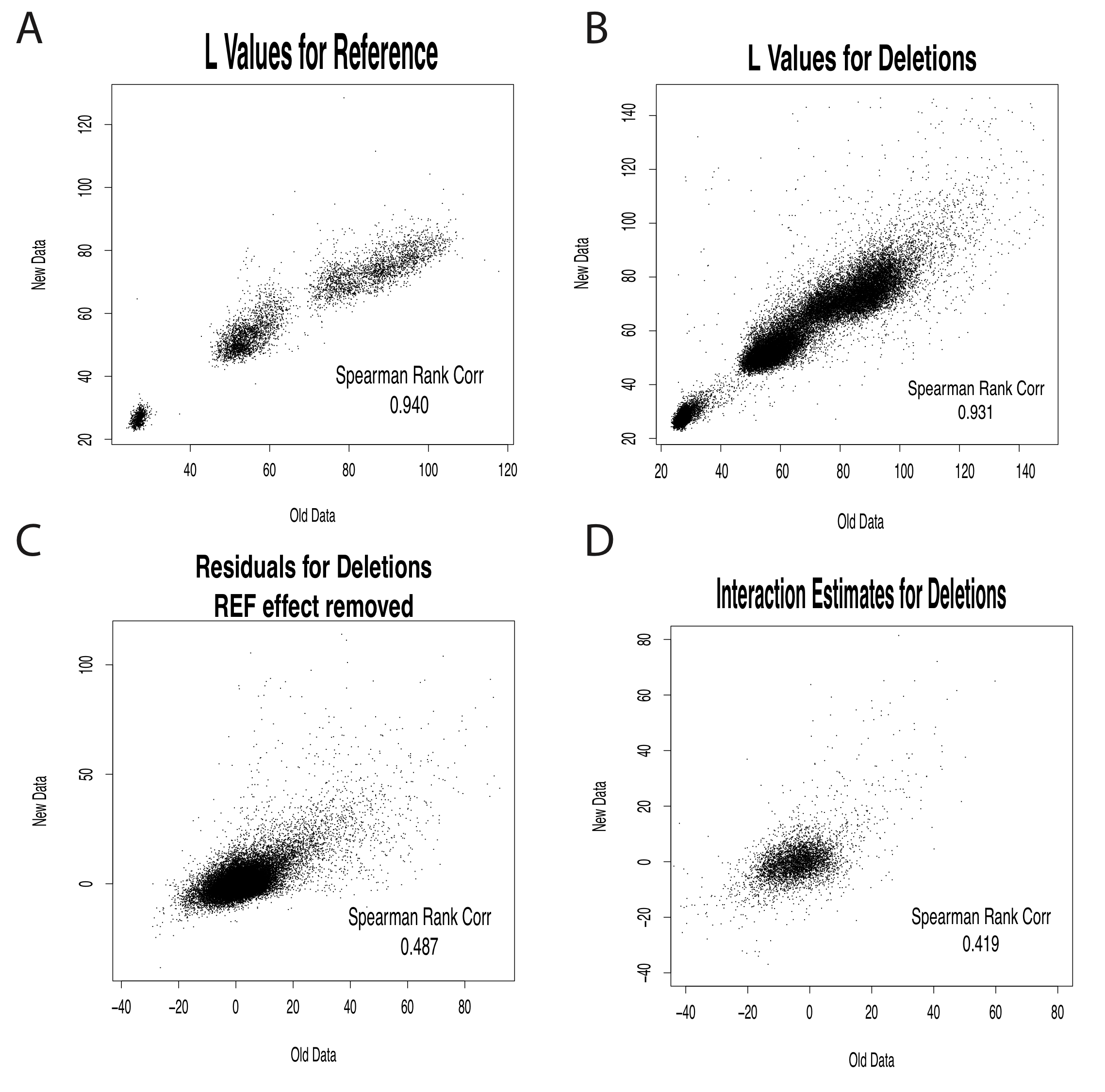


**Supplemental Figure S2:** *Reproducibility of screen data.* To assess reproducibility, we repeated the entire screen and plotted the data in different ways: A) the ‘L’ values for the reference strain cultures (Yor1-∆F single mutants) at all concentrations of oligomycin; B) the ‘L’ values for deletion strains (double mutants with Yor1-∆F in background) at all concentrations of oligomycin; C) the residual values of L for the each deletion strain culture when the oligomycin effect on the REF strain was removed; D) the interaction values for each deletion mutant calculated by fitting the oligomycin response curve (based on ‘L) and comparing to that of the REF strain. Positive Spearman rank correlations between across these two independent experiments indicate reproducibility of the method.


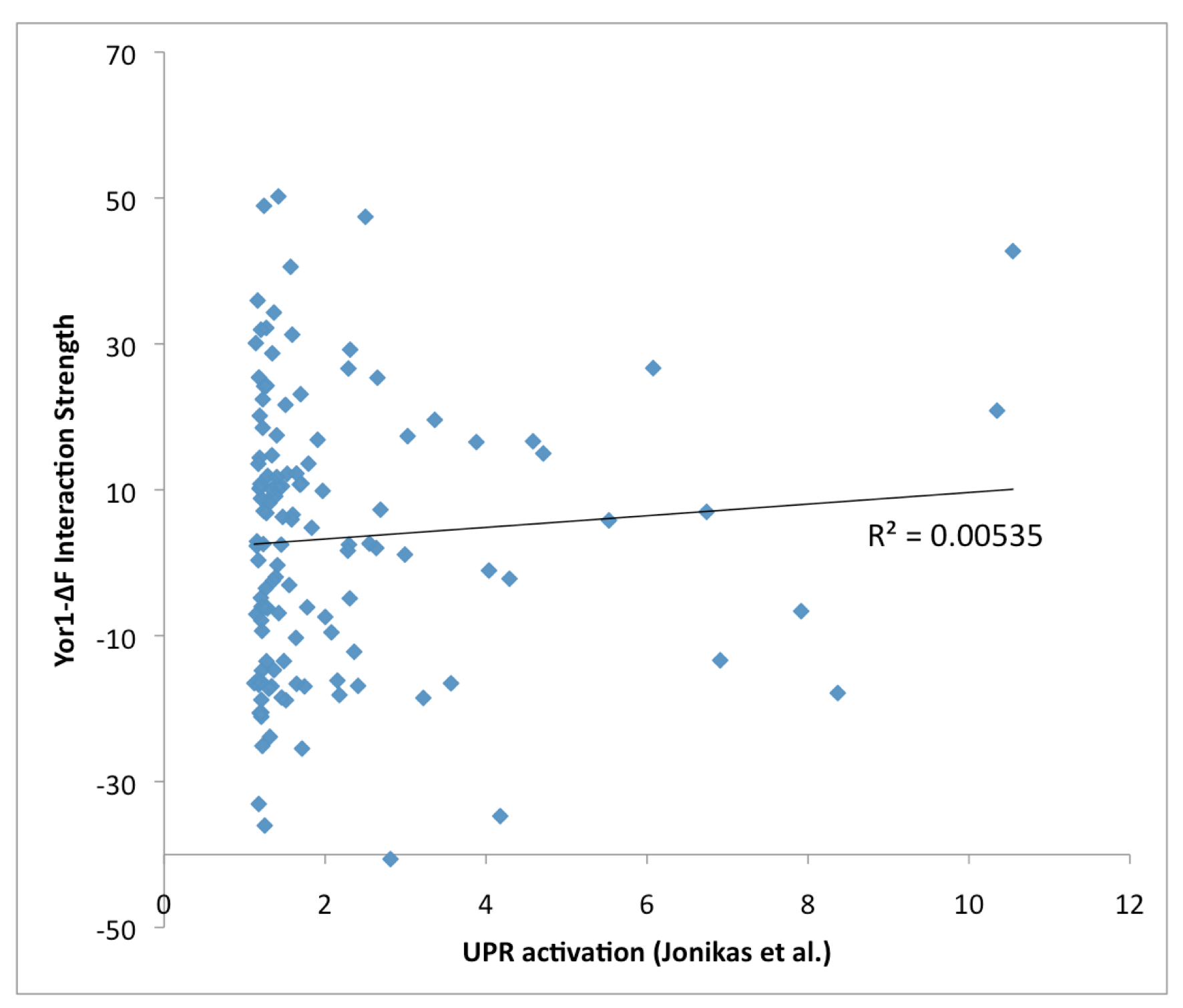


**Supplemental Figure S3:** *Correlation between gene deletions that both activate the UPR and reduce Yor1-∆F biogenesis*: To test the hypothesis that genes which, when deleted, both activate the UPR negatively impact Yor1-∆F biogenesis, we plotted the two against one another. The low degree of correlation suggests that gene deletion strains with reduced Yor1-∆F biogenesis cannot be mechanistically explained by general activation of the UPR leading to non-specific degradation of Yor1-∆F.


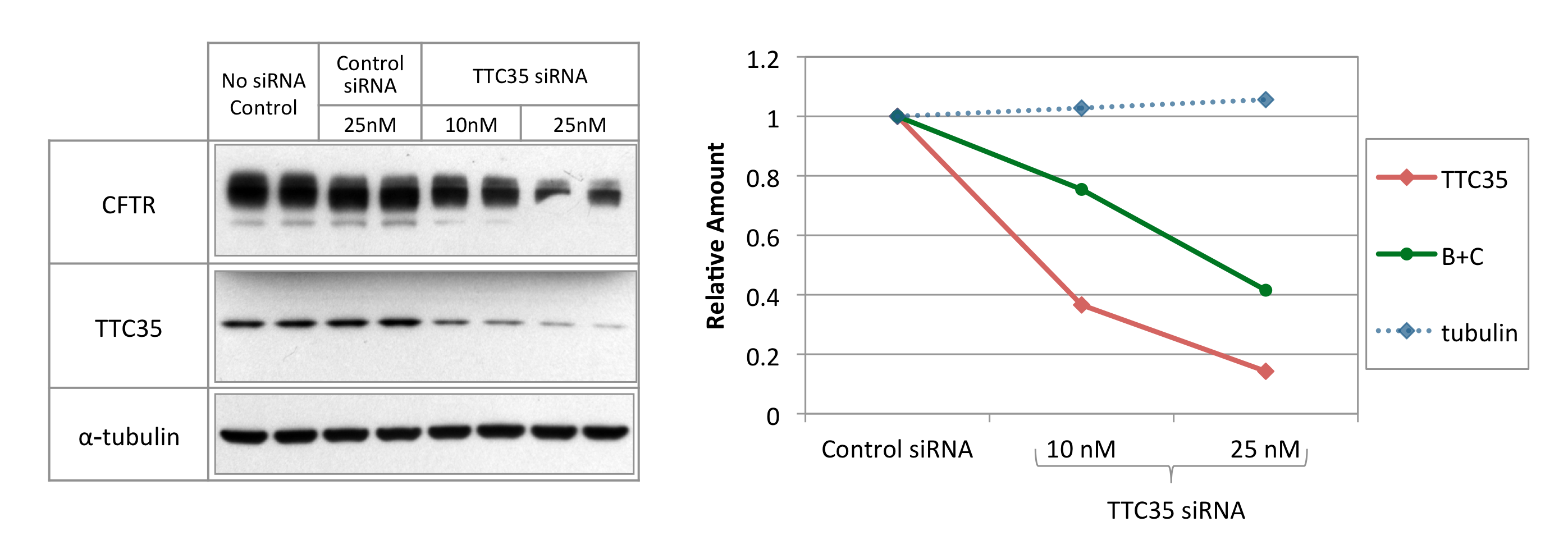


**Supplemental Figure S4:** *Knockdown of TTC35/EMC2 in HeLa cells reduces CFTR-∆F508 biogenesis.* A second representative experiment, analogous to the one shown in **Figure 7E**, illustrates the requirement of TTC35 expression for CFTR-∆F biogenesis. Lane 7 was not included in the quantification of the blot due to a bubble that prevented complete transfer, precluding its analysis.

**Supplemental References**

1. Barlowe C, Orci L, Yeung T, Hosobuchi M, Hamamoto S, Salama N, Rexach MF, Ravazzola M, Amherdt M, Schekman R: **COPII: a membrane coat formed by Sec proteins that drive vesicle budding from the endoplasmic reticulum**. *Cell* 1994, **77**:895-907.

2. Pagant S, Kung L, Dorrington M, Lee MC, Miller EA: **Inhibiting endoplasmic reticulum (ER)-associated degradation of misfolded Yor1p does not permit ER export despite the presence of a diacidic sorting signal**. *Mol Biol Cell* 2007, **18**:3398-3413.

3. Chen CZ, Calero M, DeRegis CJ, Heidtman M, Barlowe C, Collins RN: **Genetic analysis of yeast Yip1p function reveals a requirement for Golgi-localized rab proteins and rab-Guanine nucleotide dissociation inhibitor**. *Genetics* 2004, **168**:1827-1841.

4. Katzmann DJ, Epping EA, Moye-Rowley WS: **Mutational disruption of plasma membrane trafficking of Saccharomyces cerevisiae Yor1p, a homologue of mammalian multidrug resistance protein**. *Mol Cell Biol* 1999, **19**:2998-3009.

5. Shah NA, Laws RJ, Wardman B, Zhao LP, Hartman IV JL: **Accurate, precise modeling of cell proliferation kinetics from time-lapse imaging and automated image analysis of agar yeast culture arrays**. *BMC Syst Biol* 2007, **1**:3.

6. Naren AP, Nelson DJ, Xie W, Jovov B, Pevsner J, Bennett MK, Benos DJ, Quick MW, Kirk KL: **Regulation of CFTR chloride channels by syntaxin and Munc18 isoforms**. *Nature* 1997, **390**:302-305.

7. Tang BL, Gee HY, Lee MG: **The cystic fibrosis transmembrane conductance regulator's expanding SNARE interactome**. *Traffic* 2011, **12**:364-371.

8. Naren AP, Quick MW, Collawn JF, Nelson DJ, Kirk KL: **Syntaxin 1A inhibits CFTR chloride channels by means of domain-specific protein-protein interactions**. *Proc Natl Acad Sci U S A* 1998, **95**:10972-10977.

9. Peters KW, Qi J, Watkins SC, Frizzell RA: **Syntaxin 1A inhibits regulated CFTR trafficking in xenopus oocytes**. *Am J Physiol* 1999, **277**:C174-180.

10. Bilan F, Nacfer M, Fresquet F, Norez C, Melin P, Martin-Berge A, Costa de Beauregard MA, Becq F, Kitzis A, Thoreau V: **Endosomal SNARE proteins regulate CFTR activity and trafficking in epithelial cells**. *Exp Cell Res* 2008, **314**:2199-2211.

11. Bilan F, Thoreau V, Nacfer M, Derand R, Norez C, Cantereau A, Garcia M, Becq F, Kitzis A: **Syntaxin 8 impairs trafficking of cystic fibrosis transmembrane conductance regulator (CFTR) and inhibits its channel activity**. *J Cell Sci* 2004, **117**:1923-1935.

12. Gee HY, Tang BL, Kim KH, Lee MG: **Syntaxin 16 binds to cystic fibrosis transmembrane conductance regulator and regulates its membrane trafficking in epithelial cells**. *J Biol Chem* 2010, **285**:35519-35527.

13. Gentzsch M, Chang XB, Cui L, Wu Y, Ozols VV, Choudhury A, Pagano RE, Riordan JR: **Endocytic trafficking routes of wild type and DeltaF508 cystic fibrosis transmembrane conductance regulator**. *Mol Biol Cell* 2004, **15**:2684-2696.

14. Benli M, Doring F, Robinson DG, Yang X, Gallwitz D: **Two GTPase isoforms, Ypt31p and Ypt32p, are essential for Golgi function in yeast**. *EMBO J* 1996, **15**:6460-6475.

15. Swiatecka-Urban A, Talebian L, Kanno E, Moreau-Marquis S, Coutermarsh B, Hansen K, Karlson KH, Barnaby R, Cheney RE, Langford GM *et al*: **Myosin Vb is required for trafficking of the cystic fibrosis transmembrane conductance regulator in Rab11a-specific apical recycling endosomes in polarized human airway epithelial cells**. *J Biol Chem* 2007, **282**:23725-23736.

16. Huh WK, Falvo JV, Gerke LC, Carroll AS, Howson RW, Weissman JS, O'Shea EK: **Global analysis of protein localization in budding yeast**. *Nature* 2003, **425**:686-691.

17. Varga K, Jurkuvenaite A, Wakefield J, Hong JS, Guimbellot JS, Venglarik CJ, Niraj A, Mazur M, Sorscher EJ, Collawn JF *et al*: **Efficient intracellular processing of the endogenous cystic fibrosis transmembrane conductance regulator in epithelial cell lines**. *J Biol Chem* 2004, **279**:22578-22584.

18. Ward CL, Omura S, Kopito RR: **Degradation of CFTR by the ubiquitin-proteasome pathway**. *Cell* 1995, **83**:121-127.

19. Zhang Y, Nijbroek G, Sullivan ML, McCracken AA, Watkins SC, Michaelis S, Brodsky JL: **Hsp70 Molecular Chaperone Facilitates Endoplasmic Reticulum-associated Protein Degradation of Cystic Fibrosis Transmembrane Conductance Regulator in Yeast**. *Mol Biol Cell* 2001, **12**:1303-1314.

20. Goeckeler JL, Brodsky JL: **Molecular chaperones and substrate ubiquitination control the efficiency of endoplasmic reticulum-associated degradation**. *Diabetes Obes Metab* 2010, **12 Suppl 2**:32-38.

21. Okiyoneda T, Barriere H, Bagdany M, Rabeh WM, Du K, Hohfeld J, Young JC, Lukacs GL: **Peripheral protein quality control removes unfolded CFTR from the plasma membrane**. *Science* 2010, **329**:805-810.

22. Yang Y, Janich S, Cohn JA, Wilson JM: **The common variant of cystic fibrosis transmembrane conductance regulator is recognized by hsp70 and degraded in a pre-Golgi nonlysosomal compartment**. *Proc Natl Acad Sci U S A* 1993, **90**:9480-9484.

23. Kampinga HH, Craig EA: **The HSP70 chaperone machinery: J proteins as drivers of functional specificity**. *Nat Rev Mol Cell Biol* 2010, **11**:579-592.

24. Grove DE, Fan CY, Ren HY, Cyr DM: **The endoplasmic reticulum-associated Hsp40 DNAJB12 and Hsc70 cooperate to facilitate RMA1 E3-dependent degradation of nascent CFTRDeltaF508**. *Mol Biol Cell* 2011, **22**:301-314.

25. Schmidt BZ, Watts RJ, Aridor M, Frizzell RA: **Cysteine string protein promotes proteasomal degradation of the cystic fibrosis transmembrane conductance regulator (CFTR) by increasing its interaction with the C terminus of Hsp70-interacting protein and promoting CFTR ubiquitylation**. *J Biol Chem* 2009, **284**:4168-4178.

26. Yamamoto YH, Kimura T, Momohara S, Takeuchi M, Tani T, Kimata Y, Kadokura H, Kohno K: **A novel ER J-protein DNAJB12 accelerates ER-associated degradation of membrane proteins including CFTR**. *Cell Struct Funct* 2010, **35**:107-116.

27. Hale SJ, Lovell SC, de Keyzer J, Stirling CJ: **Interactions between Kar2p and its nucleotide exchange factors Sil1p and Lhs1p are mechanistically distinct**. *J Biol Chem* 2010, **285**:21600-21606.

28. Jonikas MC, Collins SR, Denic V, Oh E, Quan EM, Schmid V, Weibezahn J, Schwappach B, Walter P, Weissman JS *et al*: **Comprehensive characterization of genes required for protein folding in the endoplasmic reticulum**. *Science* 2009, **323**:1693-1697.

29. Guo J, Tian D, McKinney BA, Hartman JL: **Recursive expectation-maximization clustering: a method for identifying buffering mechanisms composed of phenomic modules**. *Chaos* 2010, **20**:026103.
