# Supplementary material for: A yeast phenomic model for the gene interaction network modulating CFTR-ΔF508 protein biogenesis
Source: Genome Med. 2012 Dec 27;4(12):103. doi: 10.1186/gm404 (PMC3906889; doi:10.1186/gm404)
Supplement: Additional File 4 — Like Supplemental Data File 2, this file contains graphs of oligomycin response for deletion strains in background of wild-type YOR1 or yor1-ΔF. Herein, less-interactive and non-interactive genes have been included for completeness. [file gm404-S4.PDF]

c. Additional File 4: Like Additinoal File 3, this file contains graphs of oligomycin response for deletion strains in background of wild type *YOR1* or *yor1*–Δ*F*. Herein, less-interactive and non-interactive genes have been included for completeness.

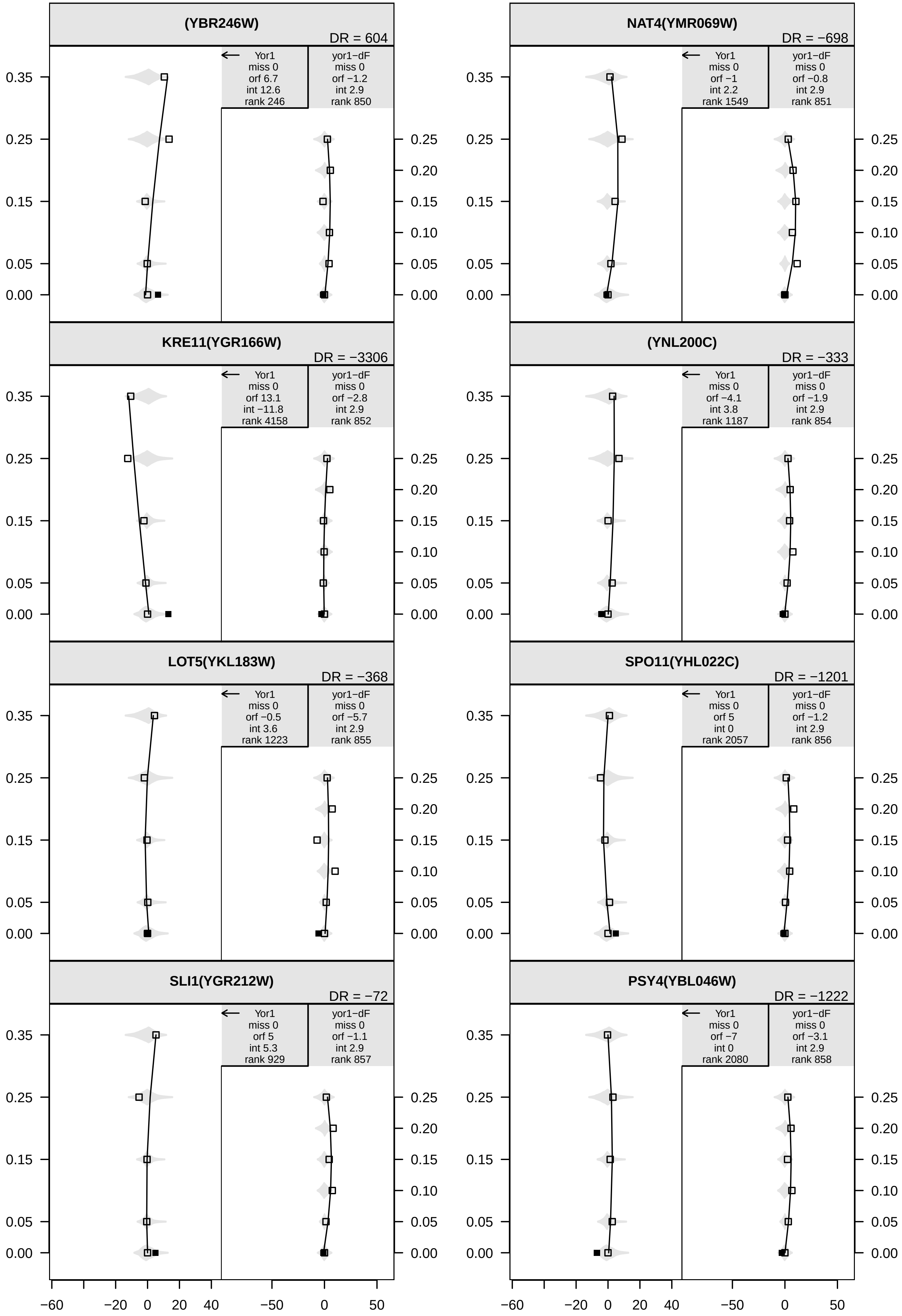
(YBR246W)
DR = 604
Yor1 miss 0 orf 6.7 int 12.6 rank 246
yor1−dF miss 0 orf −1.2 int 2.9 rank 850
NAT4(YMR069W)
DR = −698
Yor1 miss 0 orf −1 int 2.2 rank 1549
yor1−dF miss 0 orf −0.8 int 2.9 rank 851
KRE11(YGR166W)
DR = −3306
Yor1 miss 0 orf 13.1 int −11.8 rank 4158
yor1−dF miss 0 orf −2.8 int 2.9 rank 852
(YNL200C)
DR = −333
Yor1 miss 0 orf −4.1 int 3.8 rank 1187
yor1−dF miss 0 orf −1.9 int 2.9 rank 854
LOT5(YKL183W)
DR = −368
Yor1 miss 0 orf −0.5 int 3.6 rank 1223
yor1−dF miss 0 orf −5.7 int 2.9 rank 855
SPO11(YHL022C)
DR = −1201
Yor1 miss 0 orf 5 int 0 rank 2057
yor1−dF miss 0 orf −1.2 int 2.9 rank 856
SLI1(YGR212W)
DR = −72
Yor1 miss 0 orf 5 int 5.3 rank 929
yor1−dF miss 0 orf −1.1 int 2.9 rank 857
PSY4(YBL046W)
DR = −1222
Yor1 miss 0 orf −7 int 0 rank 2080
yor1−dF miss 0 orf −3.1 int 2.9 rank 858
0.35 0.25 0.15 0.05 0.00
0.25 0.20 0.15 0.10 0.05 0.00
−60 −20 0 20 40 −50 0 50

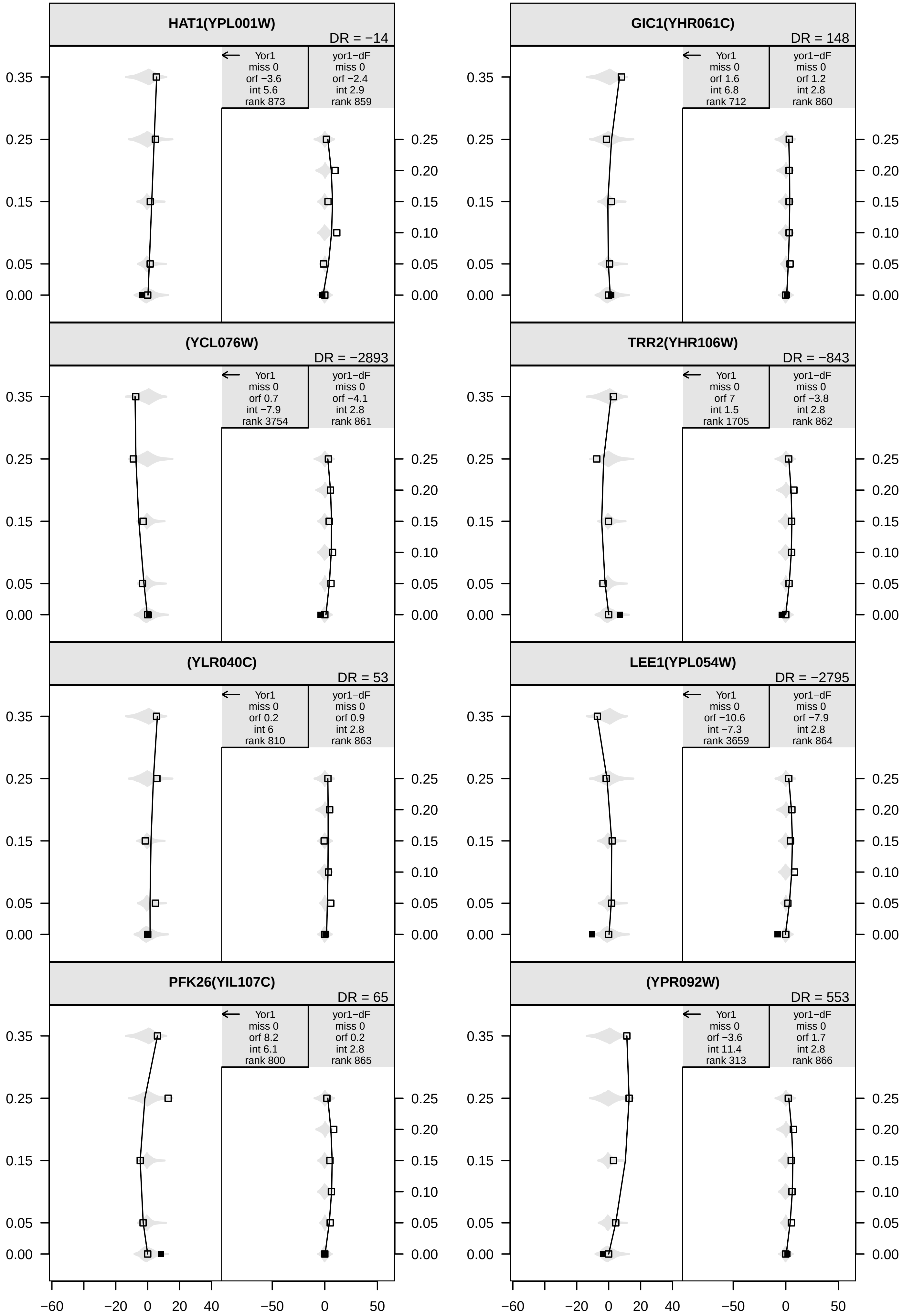
HAT1(YPL001W)
DR = −14
Yor1 miss 0 orf −3.6 int 5.6 rank 873
yor1−dF miss 0 orf −2.4 int 2.9 rank 859
GIC1(YHR061C)
DR = 148
Yor1 miss 0 orf 1.6 int 6.8 rank 712
yor1−dF miss 0 orf 1.2 int 2.8 rank 860
(YCL076W)
DR = −2893
Yor1 miss 0 orf 0.7 int −7.9 rank 3754
yor1−dF miss 0 orf −4.1 int 2.8 rank 861
TRR2(YHR106W)
DR = −843
Yor1 miss 0 orf 7 int 1.5 rank 1705
yor1−dF miss 0 orf −3.8 int 2.8 rank 862
(YLR040C)
DR = 53
Yor1 miss 0 orf 0.2 int 6 rank 810
yor1−dF miss 0 orf 0.9 int 2.8 rank 863
LEE1(YPL054W)
DR = −2795
Yor1 miss 0 orf −10.6 int −7.3 rank 3659
yor1−dF miss 0 orf −7.9 int 2.8 rank 864
PFK26(YIL107C)
DR = 65
Yor1 miss 0 orf 8.2 int 6.1 rank 800
yor1−dF miss 0 orf 0.2 int 2.8 rank 865
(YPR092W)
DR = 553
Yor1 miss 0 orf −3.6 int 11.4 rank 313
yor1−dF miss 0 orf 1.7 int 2.8 rank 866
0.35
0.25
0.20
0.15
0.10
0.05
0.00
−60
−20
0
20
40
−50
0
50

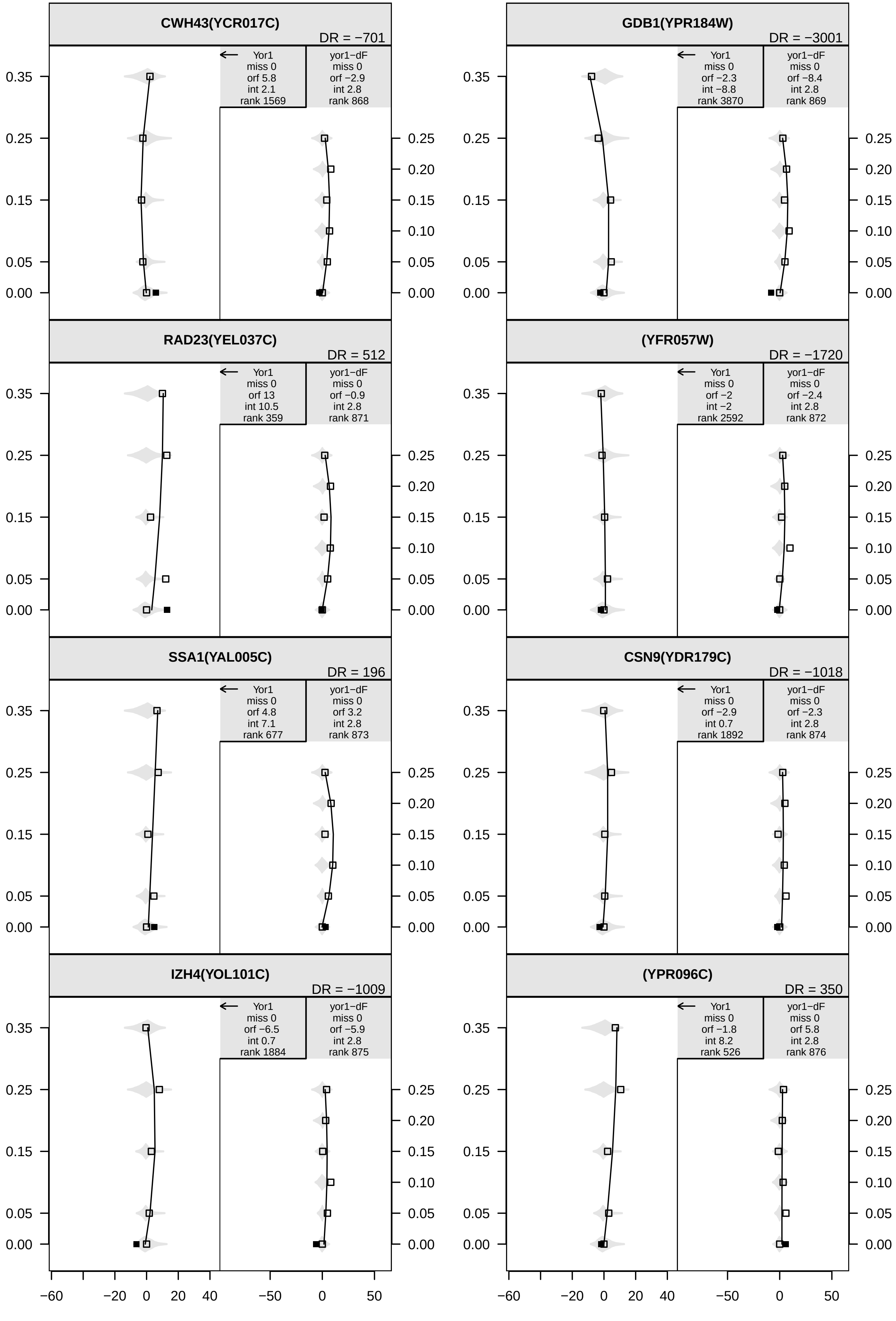

CWH43(YCR017C)
DR = −701
Yor1 miss 0 orf 5.8 int 2.1 rank 1569
yor1−dF miss 0 orf −2.9 int 2.8 rank 868
GDB1(YPR184W)
DR = −3001
Yor1 miss 0 orf −2.3 int −8.8 rank 3870
yor1−dF miss 0 orf −8.4 int 2.8 rank 869
RAD23(YEL037C)
DR = 512
Yor1 miss 0 orf 13 int 10.5 rank 359
yor1−dF miss 0 orf −0.9 int 2.8 rank 871
(YFR057W)
DR = −1720
Yor1 miss 0 orf −2 int −2 rank 2592
yor1−dF miss 0 orf −2.4 int 2.8 rank 872
SSA1(YAL005C)
DR = 196
Yor1 miss 0 orf 4.8 int 7.1 rank 677
yor1−dF miss 0 orf 3.2 int 2.8 rank 873
CSN9(YDR179C)
DR = −1018
Yor1 miss 0 orf −2.9 int 0.7 rank 1892
yor1−dF miss 0 orf −2.3 int 2.8 rank 874
IZH4(YOL101C)
DR = −1009
Yor1 miss 0 orf −6.5 int 0.7 rank 1884
yor1−dF miss 0 orf −5.9 int 2.8 rank 875
(YPR096C)
DR = 350
Yor1 miss 0 orf −1.8 int 8.2 rank 526
yor1−dF miss 0 orf 5.8 int 2.8 rank 876
0.35 0.25 0.15 0.05 0.00
0.25 0.20 0.15 0.10 0.05 0.00
−60 −20 0 20 40
−50 0 50

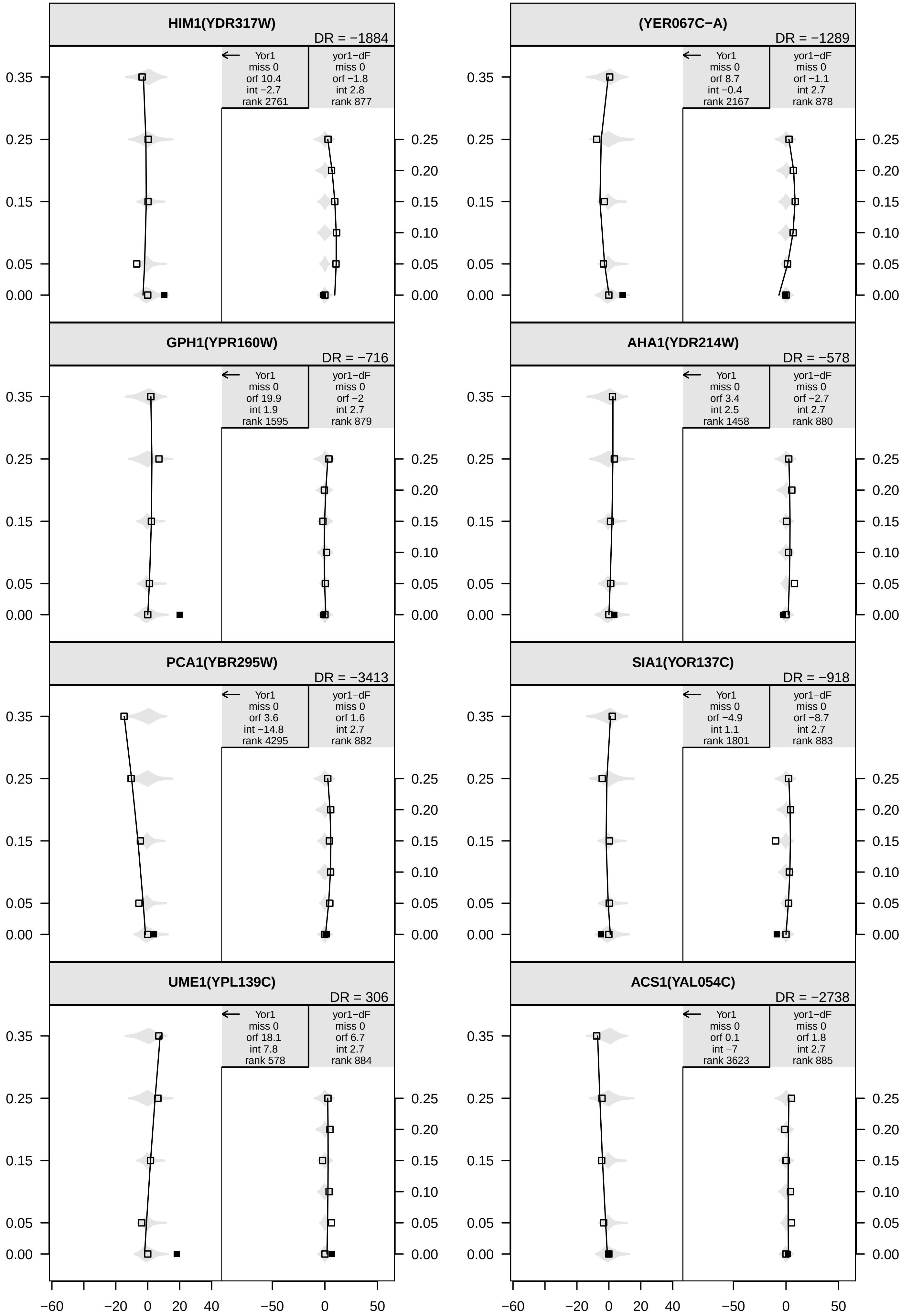
HIM1(YDR317W)
DR = −1884
Yor1 miss 0 orf 10.4 int −2.7 rank 2761
yor1−dF miss 0 orf −1.8 int 2.8 rank 877
(YER067C−A)
DR = −1289
Yor1 miss 0 orf 8.7 int −0.4 rank 2167
yor1−dF miss 0 orf −1.1 int 2.7 rank 878
GPH1(YPR160W)
DR = −716
Yor1 miss 0 orf 19.9 int 1.9 rank 1595
yor1−dF miss 0 orf −2 int 2.7 rank 879
AHA1(YDR214W)
DR = −578
Yor1 miss 0 orf 3.4 int 2.5 rank 1458
yor1−dF miss 0 orf −2.7 int 2.7 rank 880
PCA1(YBR295W)
DR = −3413
Yor1 miss 0 orf 3.6 int −14.8 rank 4295
yor1−dF miss 0 orf 1.6 int 2.7 rank 882
SIA1(YOR137C)
DR = −918
Yor1 miss 0 orf −4.9 int 1.1 rank 1801
yor1−dF miss 0 orf −8.7 int 2.7 rank 883
UME1(YPL139C)
DR = 306
Yor1 miss 0 orf 18.1 int 7.8 rank 578
yor1−dF miss 0 orf 6.7 int 2.7 rank 884
ACS1(YAL054C)
DR = −2738
Yor1 miss 0 orf 0.1 int −7 rank 3623
yor1−dF miss 0 orf 1.8 int 2.7 rank 885
0.00 0.05 0.10 0.15 0.20 0.25 0.35
−60 −20 0 20 40 −50 0 50

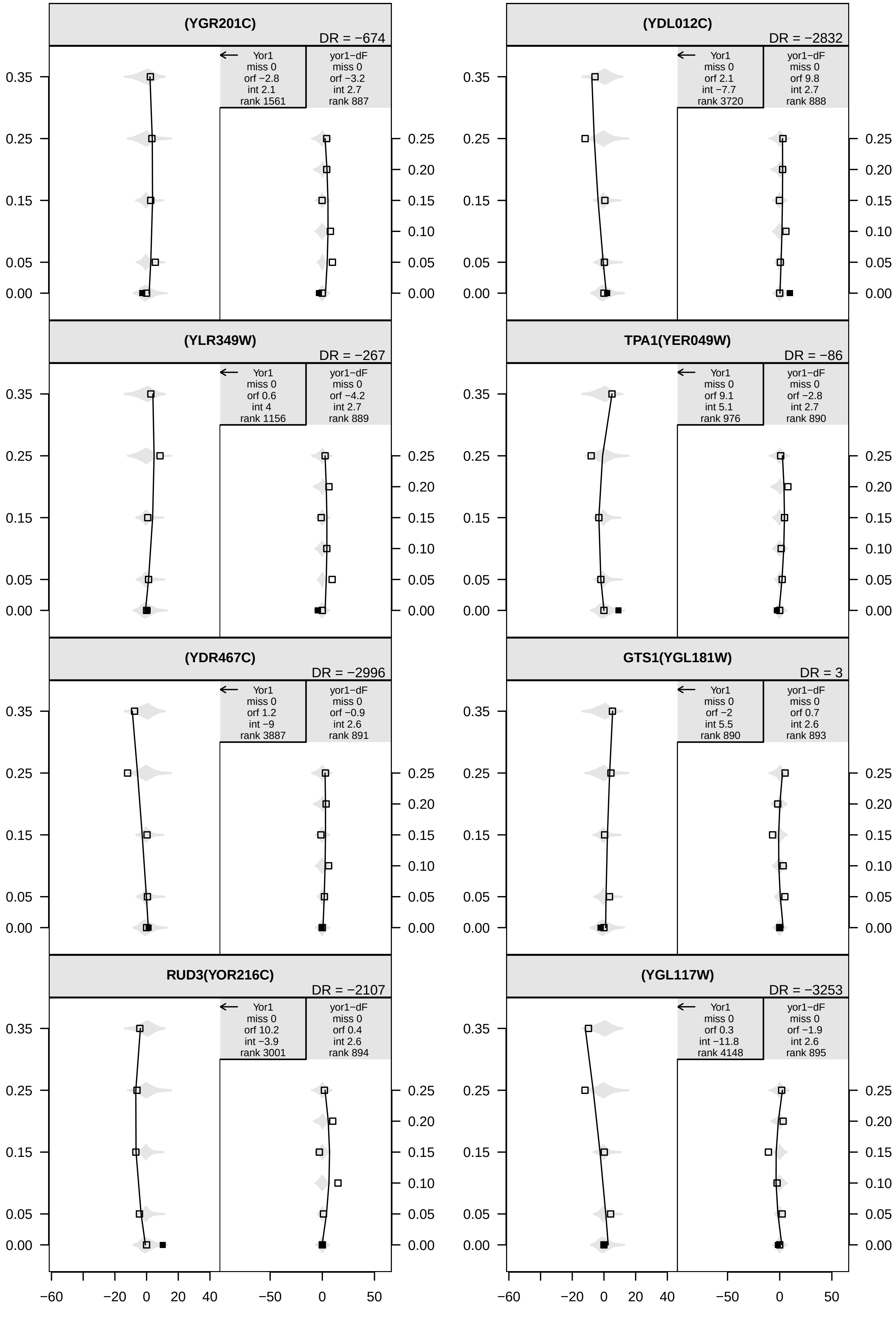
(YGR201C)
DR = −674
Yor1
miss 0
orf −2.8
int 2.1
rank 1561
yor1−dF
miss 0
orf −3.2
int 2.7
rank 887
(YDL012C)
DR = −2832
Yor1
miss 0
orf 2.1
int −7.7
rank 3720
yor1−dF
miss 0
orf 9.8
int 2.7
rank 888
(YLR349W)
DR = −267
Yor1
miss 0
orf 0.6
int 4
rank 1156
yor1−dF
miss 0
orf −4.2
int 2.7
rank 889
TPA1(YER049W)
DR = −86
Yor1
miss 0
orf 9.1
int 5.1
rank 976
yor1−dF
miss 0
orf −2.8
int 2.7
rank 890
(YDR467C)
DR = −2996
Yor1
miss 0
orf 1.2
int −9
rank 3887
yor1−dF
miss 0
orf −0.9
int 2.6
rank 891
GTS1(YGL181W)
DR = 3
Yor1
miss 0
orf −2
int 5.5
rank 890
yor1−dF
miss 0
orf 0.7
int 2.6
rank 893
RUD3(YOR216C)
DR = −2107
Yor1
miss 0
orf 10.2
int −3.9
rank 3001
yor1−dF
miss 0
orf 0.4
int 2.6
rank 894
(YGL117W)
DR = −3253
Yor1
miss 0
orf 0.3
int −11.8
rank 4148
yor1−dF
miss 0
orf −1.9
int 2.6
rank 895
0.35
0.25
0.20
0.15
0.10
0.05
0.00
−60
−20
0
20
40
−50
0
50

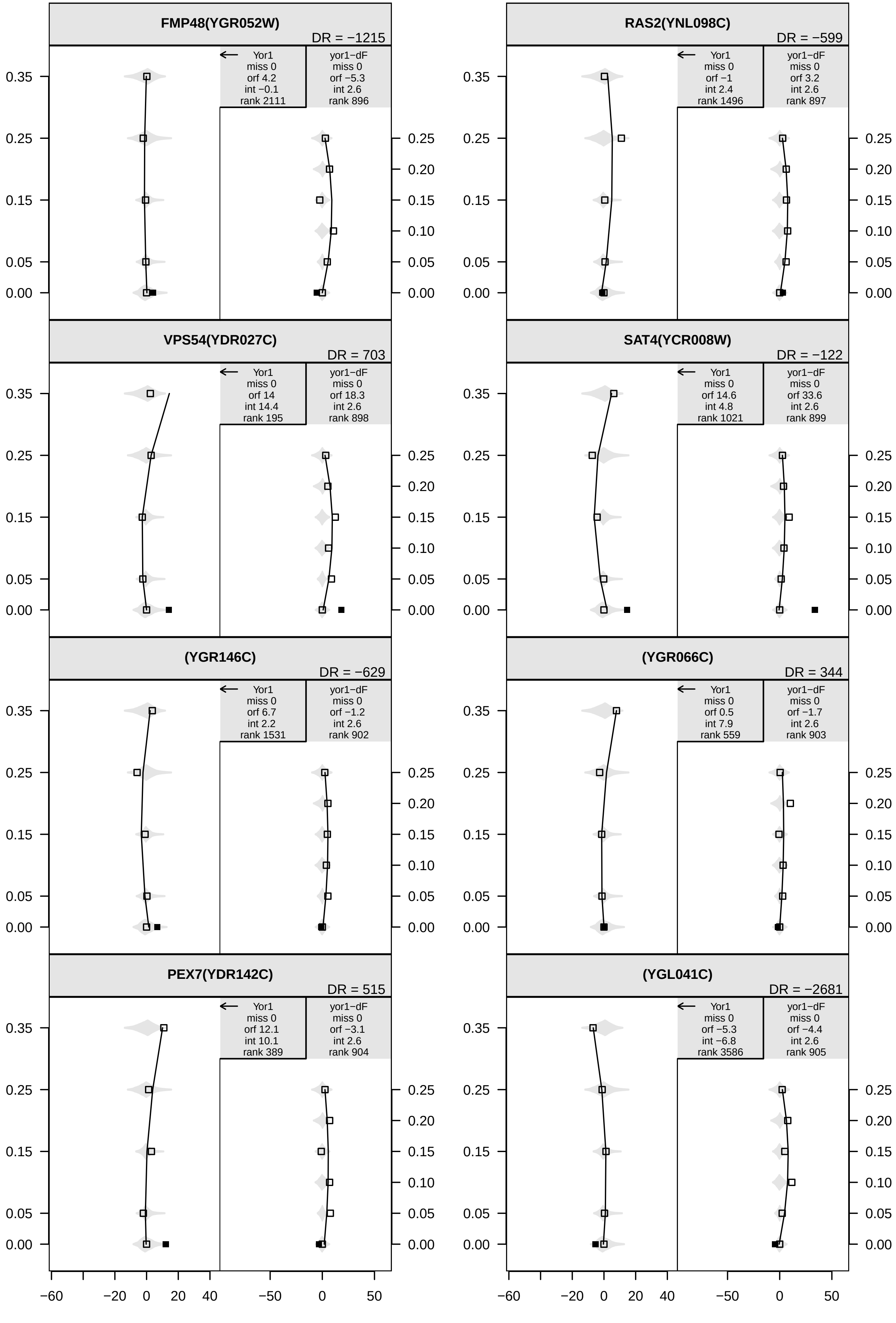
FMP48(YGR052W)
DR = −1215
Yor1 miss 0 orf 4.2 int −0.1 rank 2111
yor1−dF miss 0 orf −5.3 int 2.6 rank 896
RAS2(YNL098C)
DR = −599
Yor1 miss 0 orf −1 int 2.4 rank 1496
yor1−dF miss 0 orf 3.2 int 2.6 rank 897
VPS54(YDR027C)
DR = 703
Yor1 miss 0 orf 14 int 14.4 rank 195
yor1−dF miss 0 orf 18.3 int 2.6 rank 898
SAT4(YCR008W)
DR = −122
Yor1 miss 0 orf 14.6 int 4.8 rank 1021
yor1−dF miss 0 orf 33.6 int 2.6 rank 899
(YGR146C)
DR = −629
Yor1 miss 0 orf 6.7 int 2.2 rank 1531
yor1−dF miss 0 orf −1.2 int 2.6 rank 902
(YGR066C)
DR = 344
Yor1 miss 0 orf 0.5 int 7.9 rank 559
yor1−dF miss 0 orf −1.7 int 2.6 rank 903
PEX7(YDR142C)
DR = 515
Yor1 miss 0 orf 12.1 int 10.1 rank 389
yor1−dF miss 0 orf −3.1 int 2.6 rank 904
(YGL041C)
DR = −2681
Yor1 miss 0 orf −5.3 int −6.8 rank 3586
yor1−dF miss 0 orf −4.4 int 2.6 rank 905
0.35 0.25 0.20 0.15 0.10 0.05 0.00
−60 −20 0 20 40 −50 0 50

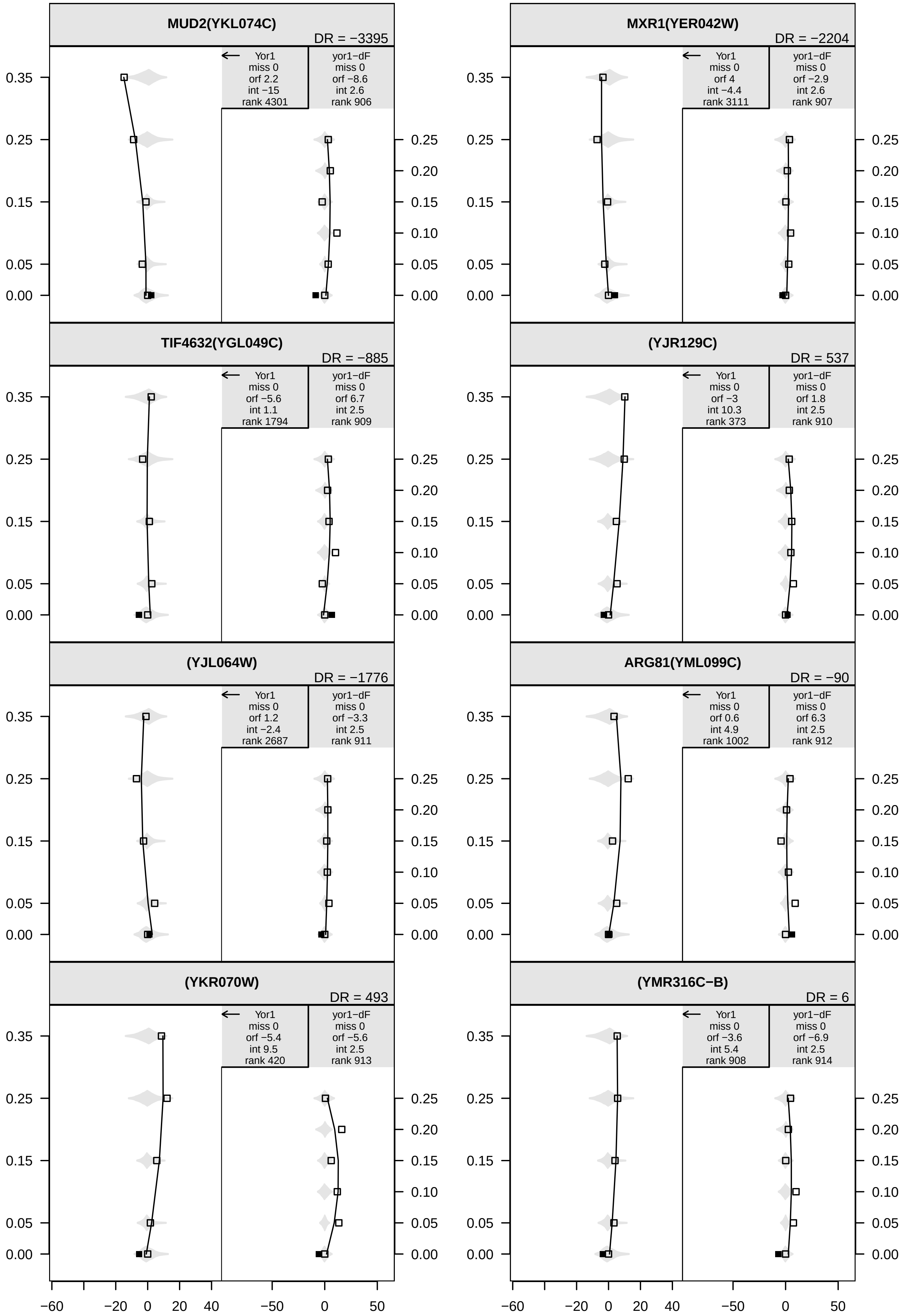
MUD2(YKL074C)
DR = −3395
Yor1 miss 0 orf 2.2 int −15 rank 4301
yor1−dF miss 0 orf −8.6 int 2.6 rank 906
MXR1(YER042W)
DR = −2204
Yor1 miss 0 orf 4 int −4.4 rank 3111
yor1−dF miss 0 orf −2.9 int 2.6 rank 907
TIF4632(YGL049C)
DR = −885
Yor1 miss 0 orf −5.6 int 1.1 rank 1794
yor1−dF miss 0 orf 6.7 int 2.5 rank 909
(YJR129C)
DR = 537
Yor1 miss 0 orf −3 int 10.3 rank 373
yor1−dF miss 0 orf 1.8 int 2.5 rank 910
(YJL064W)
DR = −1776
Yor1 miss 0 orf 1.2 int −2.4 rank 2687
yor1−dF miss 0 orf −3.3 int 2.5 rank 911
ARG81(YML099C)
DR = −90
Yor1 miss 0 orf 0.6 int 4.9 rank 1002
yor1−dF miss 0 orf 6.3 int 2.5 rank 912
(YKR070W)
DR = 493
Yor1 miss 0 orf −5.4 int 9.5 rank 420
yor1−dF miss 0 orf −5.6 int 2.5 rank 913
(YMR316C−B)
DR = 6
Yor1 miss 0 orf −3.6 int 5.4 rank 908
yor1−dF miss 0 orf −6.9 int 2.5 rank 914

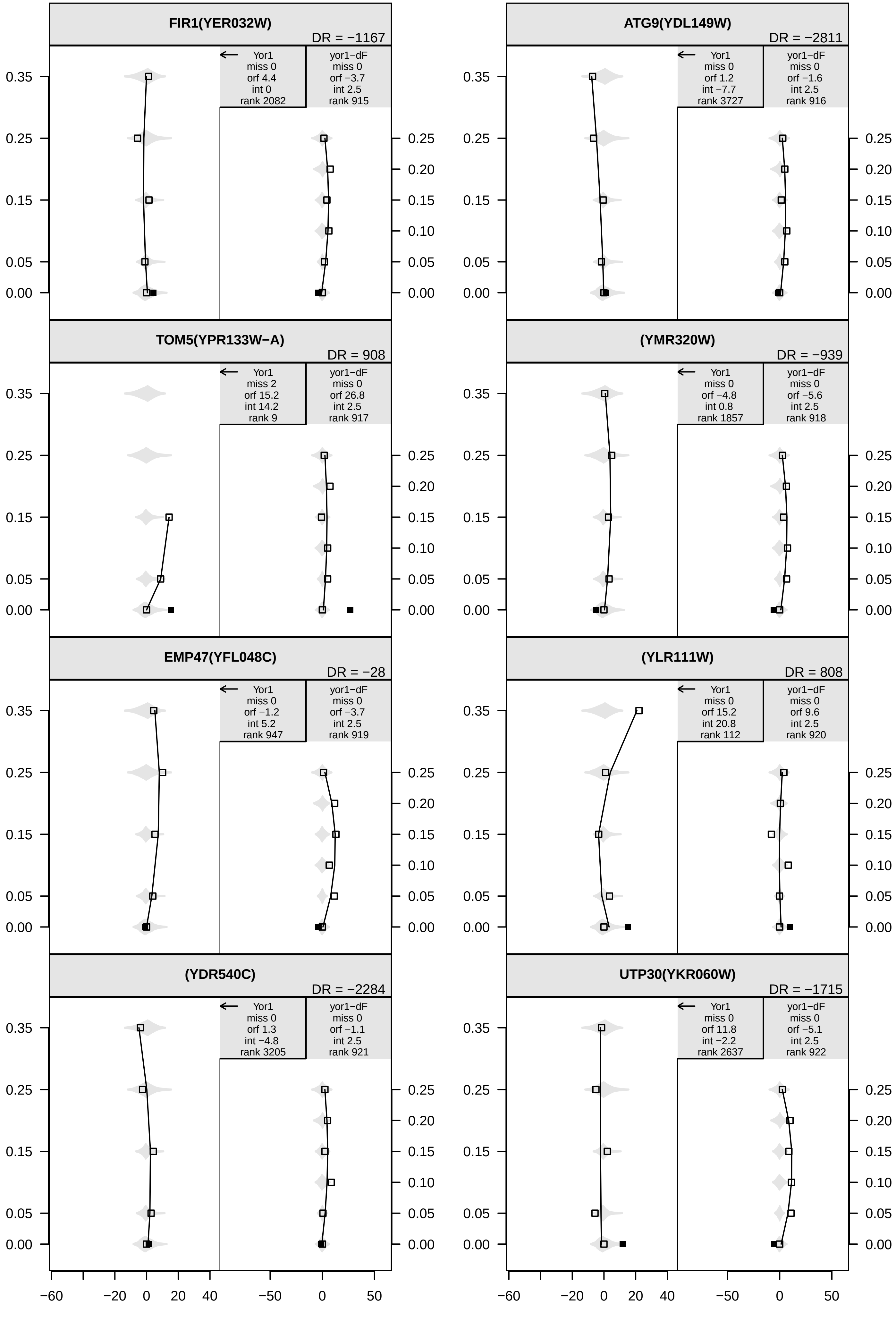
FIR1(YER032W)
DR = −1167
Yor1 miss 0 orf 4.4 int 0 rank 2082
yor1−dF miss 0 orf −3.7 int 2.5 rank 915
ATG9(YDL149W)
DR = −2811
Yor1 miss 0 orf 1.2 int −7.7 rank 3727
yor1−dF miss 0 orf −1.6 int 2.5 rank 916
TOM5(YPR133W−A)
DR = 908
Yor1 miss 2 orf 15.2 int 14.2 rank 9
yor1−dF miss 0 orf 26.8 int 2.5 rank 917
(YMR320W)
DR = −939
Yor1 miss 0 orf −4.8 int 0.8 rank 1857
yor1−dF miss 0 orf −5.6 int 2.5 rank 918
EMP47(YFL048C)
DR = −28
Yor1 miss 0 orf −1.2 int 5.2 rank 947
yor1−dF miss 0 orf −3.7 int 2.5 rank 919
(YLR111W)
DR = 808
Yor1 miss 0 orf 15.2 int 20.8 rank 112
yor1−dF miss 0 orf 9.6 int 2.5 rank 920
(YDR540C)
DR = −2284
Yor1 miss 0 orf 1.3 int −4.8 rank 3205
yor1−dF miss 0 orf −1.1 int 2.5 rank 921
UTP30(YKR060W)
DR = −1715
Yor1 miss 0 orf 11.8 int −2.2 rank 2637
yor1−dF miss 0 orf −5.1 int 2.5 rank 922
0.35
0.25
0.20
0.15
0.10
0.05
0.00
−60
−20
0
20
40
−50
0
50

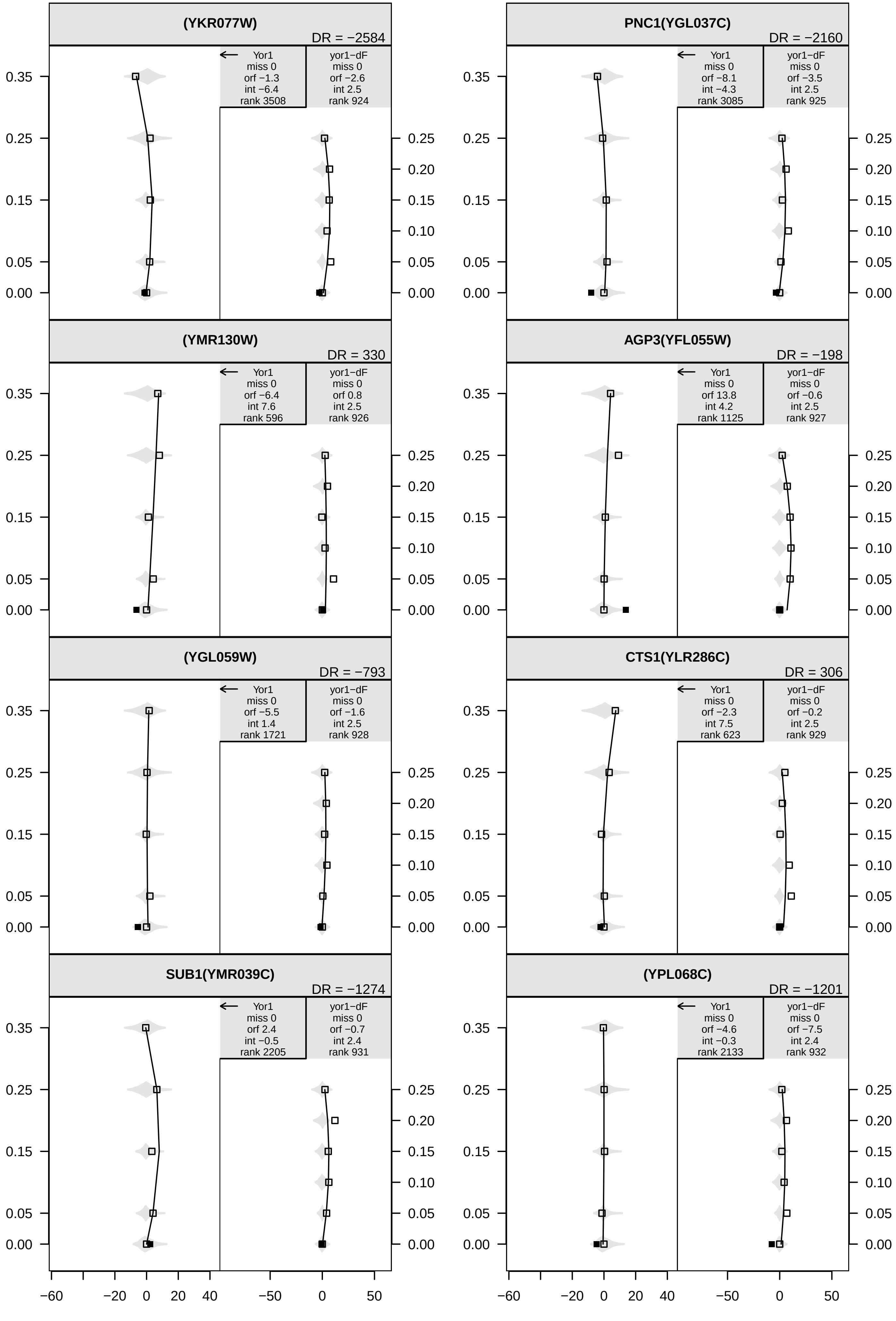

(YKR077W)
DR = −2584
Yor1 miss 0 orf −1.3 int −6.4 rank 3508
yor1−dF miss 0 orf −2.6 int 2.5 rank 924
PNC1(YGL037C)
DR = −2160
Yor1 miss 0 orf −8.1 int −4.3 rank 3085
yor1−dF miss 0 orf −3.5 int 2.5 rank 925
(YMR130W)
DR = 330
Yor1 miss 0 orf −6.4 int 7.6 rank 596
yor1−dF miss 0 orf 0.8 int 2.5 rank 926
AGP3(YFL055W)
DR = −198
Yor1 miss 0 orf 13.8 int 4.2 rank 1125
yor1−dF miss 0 orf −0.6 int 2.5 rank 927
(YGL059W)
DR = −793
Yor1 miss 0 orf −5.5 int 1.4 rank 1721
yor1−dF miss 0 orf −1.6 int 2.5 rank 928
CTS1(YLR286C)
DR = 306
Yor1 miss 0 orf −2.3 int 7.5 rank 623
yor1−dF miss 0 orf −0.2 int 2.5 rank 929
SUB1(YMR039C)
DR = −1274
Yor1 miss 0 orf 2.4 int −0.5 rank 2205
yor1−dF miss 0 orf −0.7 int 2.4 rank 931
(YPL068C)
DR = −1201
Yor1 miss 0 orf −4.6 int −0.3 rank 2133
yor1−dF miss 0 orf −7.5 int 2.4 rank 932
0.35
0.25
0.20
0.15
0.10
0.05
0.00
−60
−20
0
20
40
−50
0
50

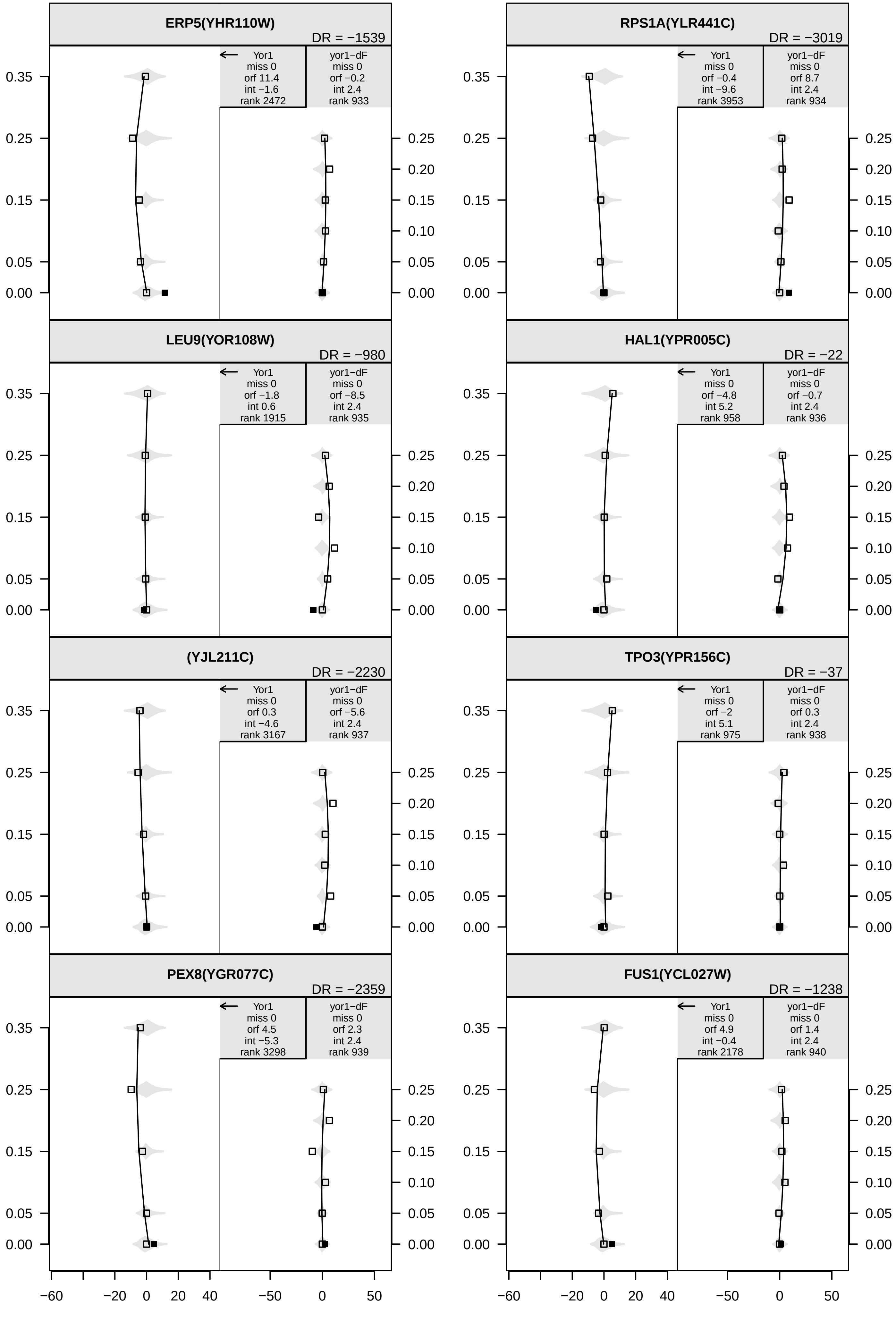
ERP5(YHR110W)
DR = −1539
Yor1 miss 0 orf 11.4 int −1.6 rank 2472
yor1−dF miss 0 orf −0.2 int 2.4 rank 933
RPS1A(YLR441C)
DR = −3019
Yor1 miss 0 orf −0.4 int −9.6 rank 3953
yor1−dF miss 0 orf 8.7 int 2.4 rank 934
LEU9(YOR108W)
DR = −980
Yor1 miss 0 orf −1.8 int 0.6 rank 1915
yor1−dF miss 0 orf −8.5 int 2.4 rank 935
HAL1(YPR005C)
DR = −22
Yor1 miss 0 orf −4.8 int 5.2 rank 958
yor1−dF miss 0 orf −0.7 int 2.4 rank 936
(YJL211C)
DR = −2230
Yor1 miss 0 orf 0.3 int −4.6 rank 3167
yor1−dF miss 0 orf −5.6 int 2.4 rank 937
TPO3(YPR156C)
DR = −37
Yor1 miss 0 orf −2 int 5.1 rank 975
yor1−dF miss 0 orf 0.3 int 2.4 rank 938
PEX8(YGR077C)
DR = −2359
Yor1 miss 0 orf 4.5 int −5.3 rank 3298
yor1−dF miss 0 orf 2.3 int 2.4 rank 939
FUS1(YCL027W)
DR = −1238
Yor1 miss 0 orf 4.9 int −0.4 rank 2178
yor1−dF miss 0 orf 1.4 int 2.4 rank 940
0.35 0.25 0.15 0.05 0.00
0.25 0.20 0.15 0.10 0.05 0.00
−60 −20 0 20 40
−50 0 50

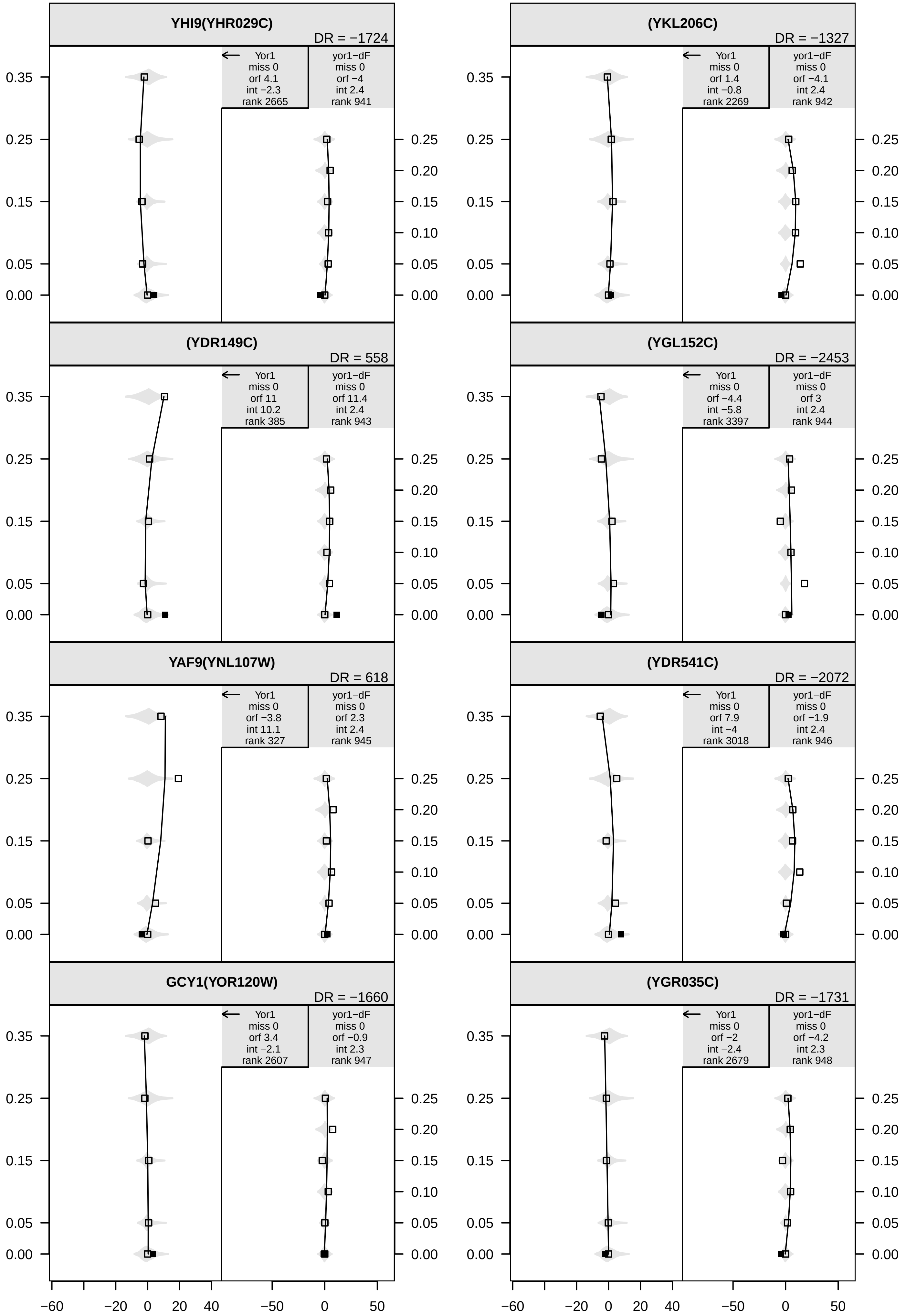

YHI9(YHR029C)
DR = −1724
Yor1 miss 0 orf 4.1 int −2.3 rank 2665
yor1−dF miss 0 orf −4 int 2.4 rank 941
(YKL206C)
DR = −1327
Yor1 miss 0 orf 1.4 int −0.8 rank 2269
yor1−dF miss 0 orf −4.1 int 2.4 rank 942
(YDR149C)
DR = 558
Yor1 miss 0 orf 11 int 10.2 rank 385
yor1−dF miss 0 orf 11.4 int 2.4 rank 943
(YGL152C)
DR = −2453
Yor1 miss 0 orf −4.4 int −5.8 rank 3397
yor1−dF miss 0 orf 3 int 2.4 rank 944
YAF9(YNL107W)
DR = 618
Yor1 miss 0 orf −3.8 int 11.1 rank 327
yor1−dF miss 0 orf 2.3 int 2.4 rank 945
(YDR541C)
DR = −2072
Yor1 miss 0 orf 7.9 int −4 rank 3018
yor1−dF miss 0 orf −1.9 int 2.4 rank 946
GCY1(YOR120W)
DR = −1660
Yor1 miss 0 orf 3.4 int −2.1 rank 2607
yor1−dF miss 0 orf −0.9 int 2.3 rank 947
(YGR035C)
DR = −1731
Yor1 miss 0 orf −2 int −2.4 rank 2679
yor1−dF miss 0 orf −4.2 int 2.3 rank 948
0.00 0.05 0.10 0.15 0.20 0.25 0.35
−60 −20 0 20 40 −50 0 50

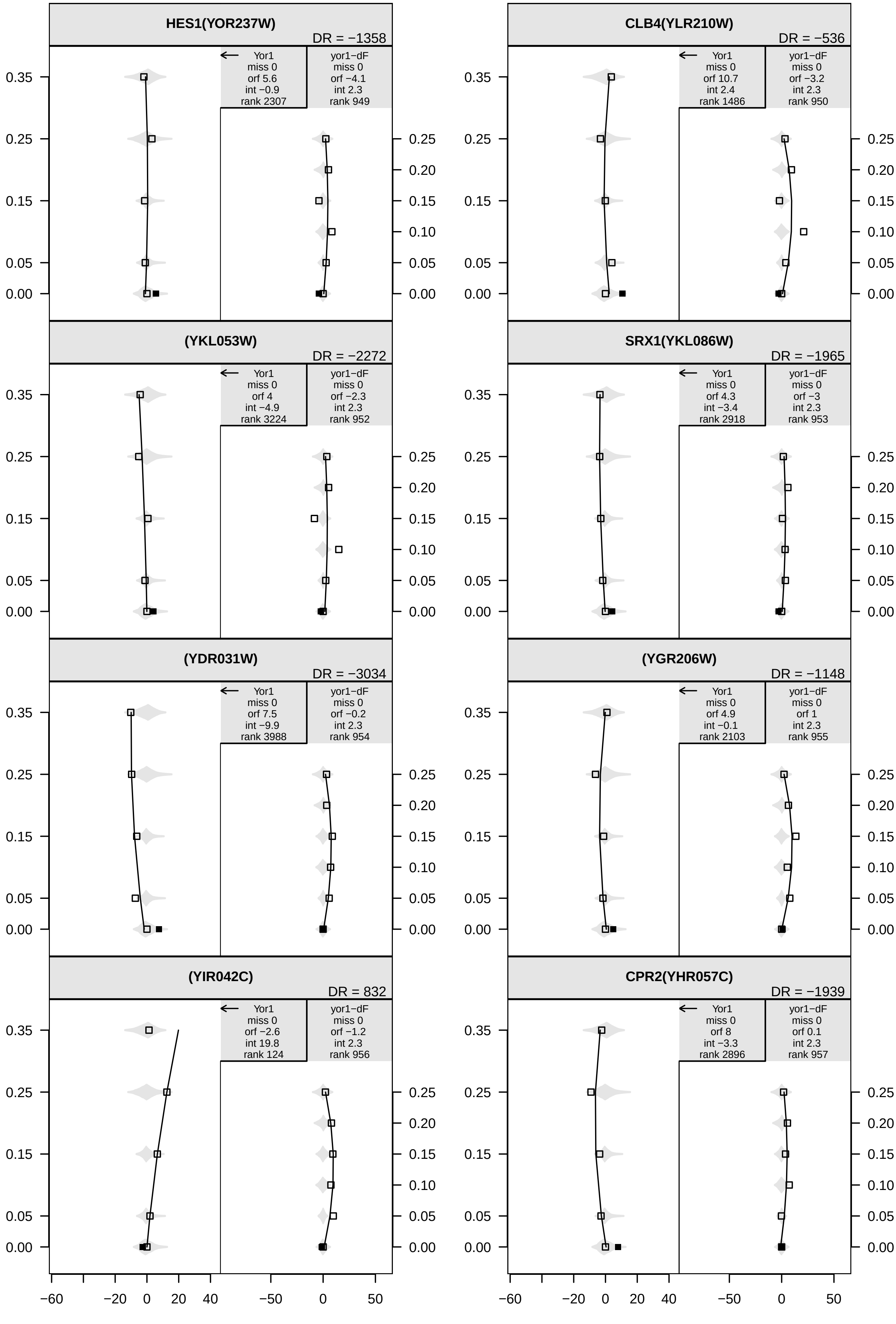

HES1(YOR237W)
DR = −1358
Yor1 miss 0 orf 5.6 int −0.9 rank 2307
yor1−dF miss 0 orf −4.1 int 2.3 rank 949
CLB4(YLR210W)
DR = −536
Yor1 miss 0 orf 10.7 int 2.4 rank 1486
yor1−dF miss 0 orf −3.2 int 2.3 rank 950
(YKL053W)
DR = −2272
Yor1 miss 0 orf 4 int −4.9 rank 3224
yor1−dF miss 0 orf −2.3 int 2.3 rank 952
SRX1(YKL086W)
DR = −1965
Yor1 miss 0 orf 4.3 int −3.4 rank 2918
yor1−dF miss 0 orf −3 int 2.3 rank 953
(YDR031W)
DR = −3034
Yor1 miss 0 orf 7.5 int −9.9 rank 3988
yor1−dF miss 0 orf −0.2 int 2.3 rank 954
(YGR206W)
DR = −1148
Yor1 miss 0 orf 4.9 int −0.1 rank 2103
yor1−dF miss 0 orf 1 int 2.3 rank 955
(YIR042C)
DR = 832
Yor1 miss 0 orf −2.6 int 19.8 rank 124
yor1−dF miss 0 orf −1.2 int 2.3 rank 956
CPR2(YHR057C)
DR = −1939
Yor1 miss 0 orf 8 int −3.3 rank 2896
yor1−dF miss 0 orf 0.1 int 2.3 rank 957
0.35 0.25 0.15 0.05 0.00
0.25 0.20 0.15 0.10 0.05 0.00
−60 −20 0 20 40
−50 0 50

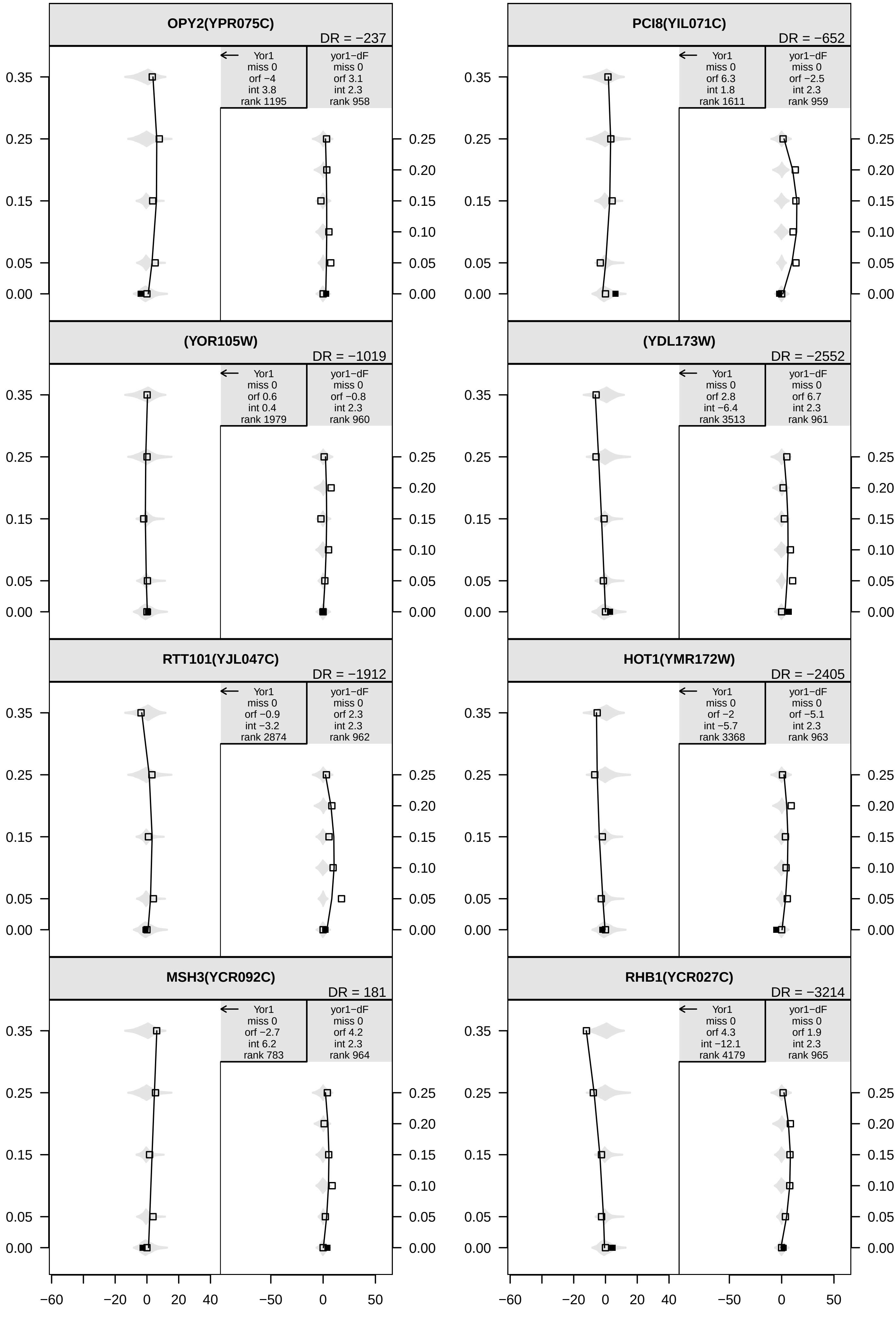

OPY2(YPR075C)
DR = −237
Yor1 miss 0 orf −4 int 3.8 rank 1195
yor1−dF miss 0 orf 3.1 int 2.3 rank 958
PCI8(YIL071C)
DR = −652
Yor1 miss 0 orf 6.3 int 1.8 rank 1611
yor1−dF miss 0 orf −2.5 int 2.3 rank 959
(YOR105W)
DR = −1019
Yor1 miss 0 orf 0.6 int 0.4 rank 1979
yor1−dF miss 0 orf −0.8 int 2.3 rank 960
(YDL173W)
DR = −2552
Yor1 miss 0 orf 2.8 int −6.4 rank 3513
yor1−dF miss 0 orf 6.7 int 2.3 rank 961
RTT101(YJL047C)
DR = −1912
Yor1 miss 0 orf −0.9 int −3.2 rank 2874
yor1−dF miss 0 orf 2.3 int 2.3 rank 962
HOT1(YMR172W)
DR = −2405
Yor1 miss 0 orf −2 int −5.7 rank 3368
yor1−dF miss 0 orf −5.1 int 2.3 rank 963
MSH3(YCR092C)
DR = 181
Yor1 miss 0 orf −2.7 int 6.2 rank 783
yor1−dF miss 0 orf 4.2 int 2.3 rank 964
RHB1(YCR027C)
DR = −3214
Yor1 miss 0 orf 4.3 int −12.1 rank 4179
yor1−dF miss 0 orf 1.9 int 2.3 rank 965

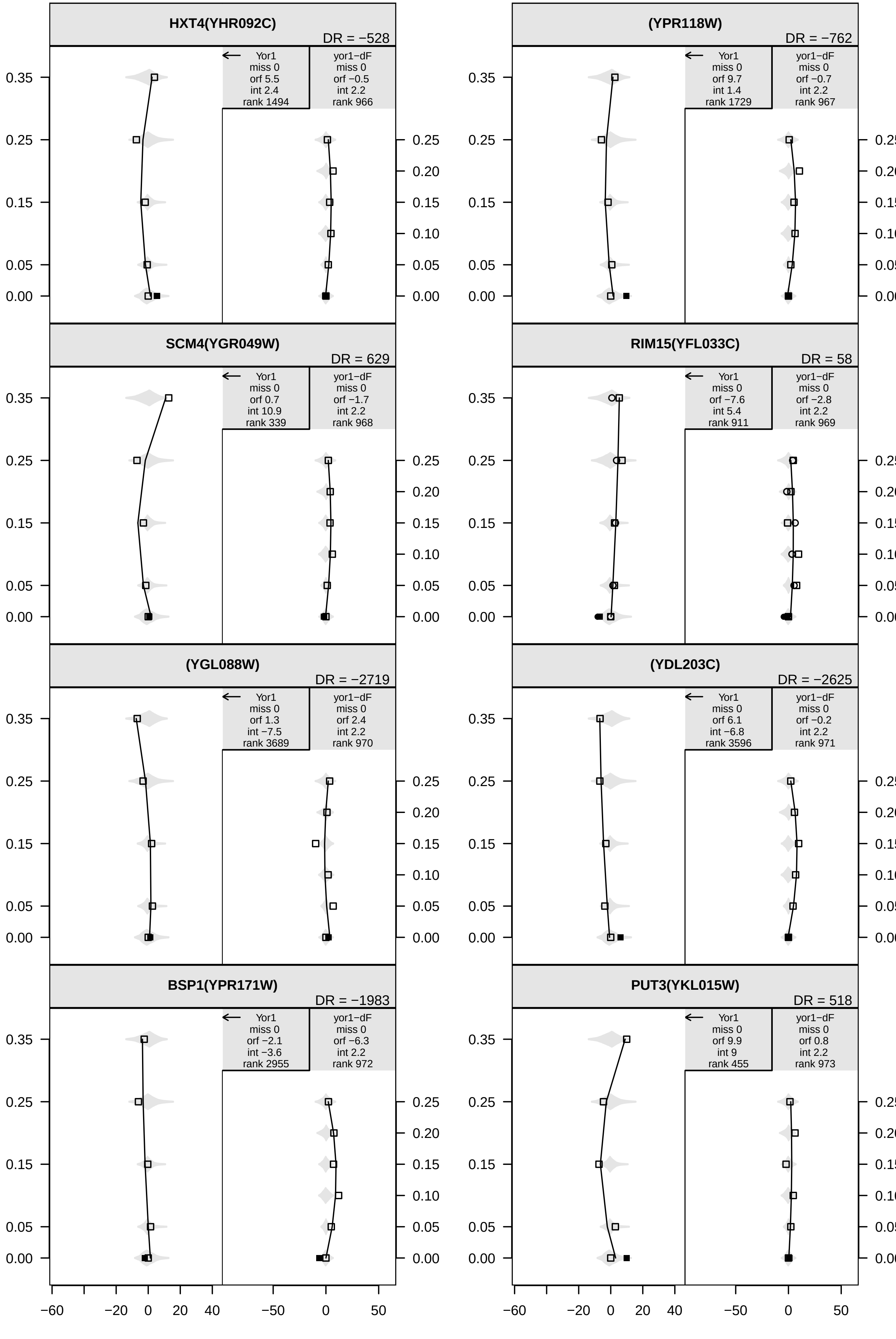
HXT4(YHR092C)
DR = −528
Yor1 miss 0 orf 5.5 int 2.4 rank 1494
yor1−dF miss 0 orf −0.5 int 2.2 rank 966
(YPR118W)
DR = −762
Yor1 miss 0 orf 9.7 int 1.4 rank 1729
yor1−dF miss 0 orf −0.7 int 2.2 rank 967
SCM4(YGR049W)
DR = 629
Yor1 miss 0 orf 0.7 int 10.9 rank 339
yor1−dF miss 0 orf −1.7 int 2.2 rank 968
RIM15(YFL033C)
DR = 58
Yor1 miss 0 orf −7.6 int 5.4 rank 911
yor1−dF miss 0 orf −2.8 int 2.2 rank 969
(YGL088W)
DR = −2719
Yor1 miss 0 orf 1.3 int −7.5 rank 3689
yor1−dF miss 0 orf 2.4 int 2.2 rank 970
(YDL203C)
DR = −2625
Yor1 miss 0 orf 6.1 int −6.8 rank 3596
yor1−dF miss 0 orf −0.2 int 2.2 rank 971
BSP1(YPR171W)
DR = −1983
Yor1 miss 0 orf −2.1 int −3.6 rank 2955
yor1−dF miss 0 orf −6.3 int 2.2 rank 972
PUT3(YKL015W)
DR = 518
Yor1 miss 0 orf 9.9 int 9 rank 455
yor1−dF miss 0 orf 0.8 int 2.2 rank 973
0.00 0.05 0.10 0.15 0.20 0.25 0.35
−60 −20 0 20 40 −50 0 50

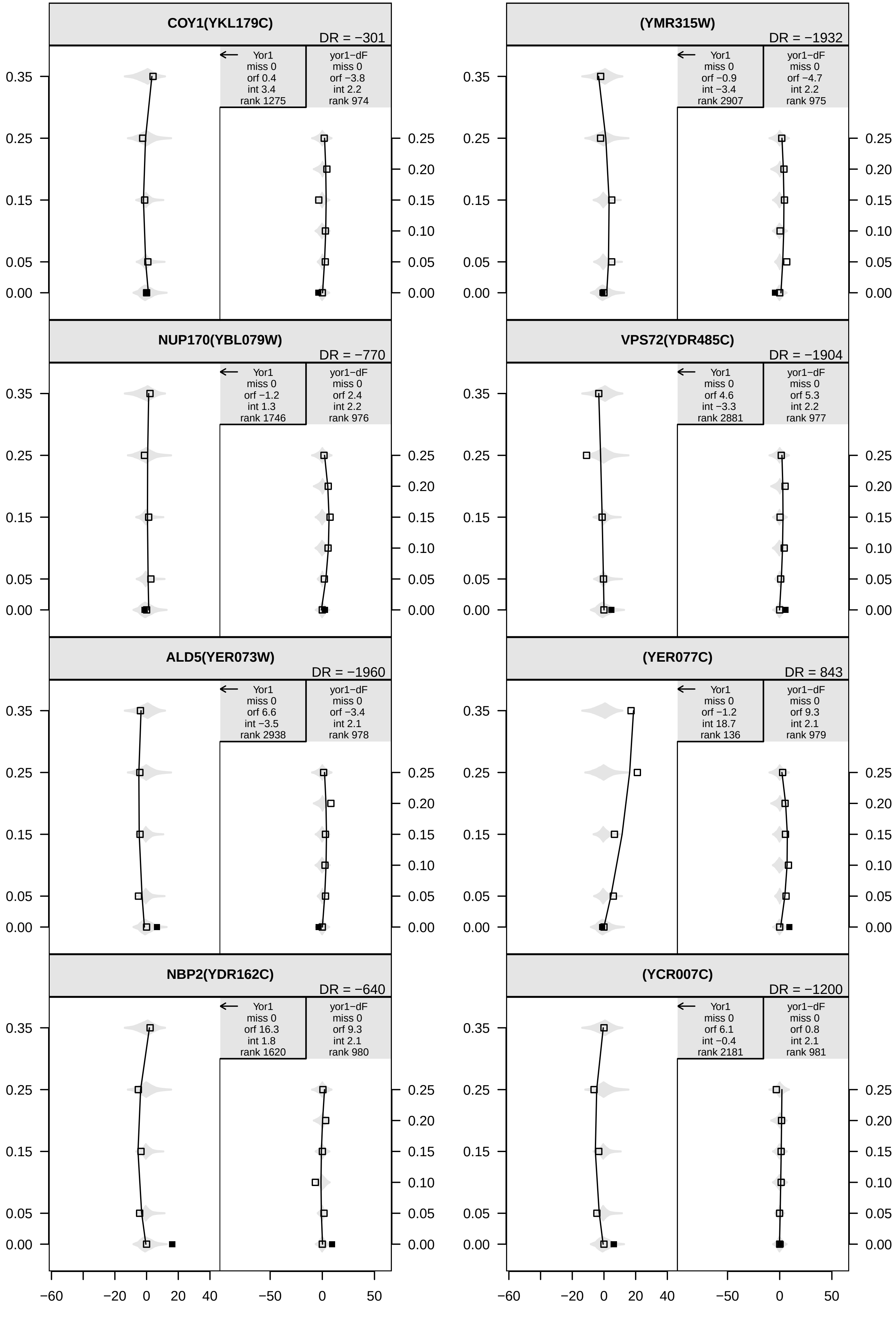
COY1(YKL179C)
DR = −301
Yor1
miss 0
orf 0.4
int 3.4
rank 1275
yor1−dF
miss 0
orf −3.8
int 2.2
rank 974
(YMR315W)
DR = −1932
Yor1
miss 0
orf −0.9
int −3.4
rank 2907
yor1−dF
miss 0
orf −4.7
int 2.2
rank 975
NUP170(YBL079W)
DR = −770
Yor1
miss 0
orf −1.2
int 1.3
rank 1746
yor1−dF
miss 0
orf 2.4
int 2.2
rank 976
VPS72(YDR485C)
DR = −1904
Yor1
miss 0
orf 4.6
int −3.3
rank 2881
yor1−dF
miss 0
orf 5.3
int 2.2
rank 977
ALD5(YER073W)
DR = −1960
Yor1
miss 0
orf 6.6
int −3.5
rank 2938
yor1−dF
miss 0
orf −3.4
int 2.1
rank 978
(YER077C)
DR = 843
Yor1
miss 0
orf −1.2
int 18.7
rank 136
yor1−dF
miss 0
orf 9.3
int 2.1
rank 979
NBP2(YDR162C)
DR = −640
Yor1
miss 0
orf 16.3
int 1.8
rank 1620
yor1−dF
miss 0
orf 9.3
int 2.1
rank 980
(YCR007C)
DR = −1200
Yor1
miss 0
orf 6.1
int −0.4
rank 2181
yor1−dF
miss 0
orf 0.8
int 2.1
rank 981
0.35
0.25
0.20
0.15
0.10
0.05
0.00
−60
−20
0
20
40
−50
0
50

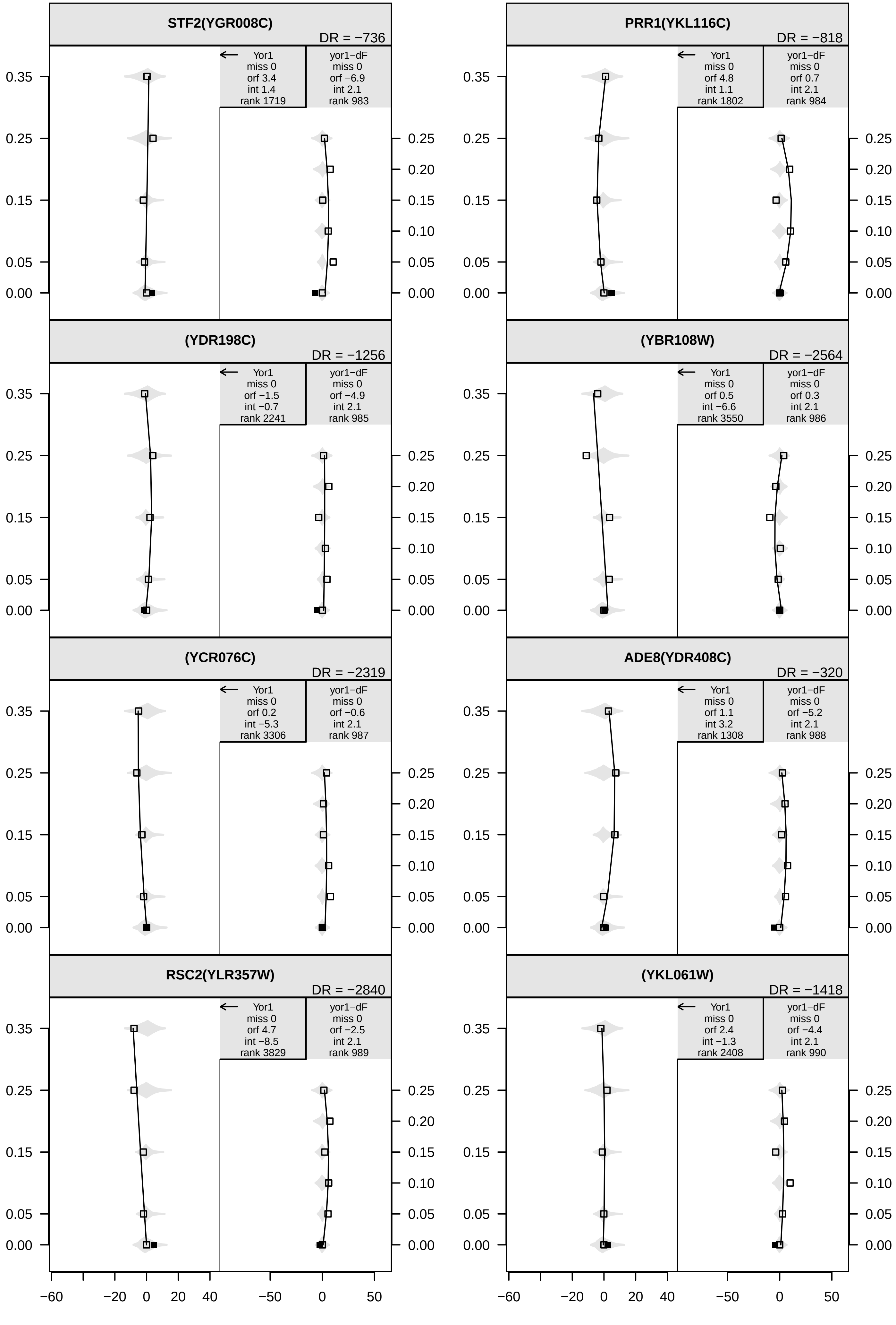
STF2(YGR008C)
DR = −736
Yor1
miss 0
orf 3.4
int 1.4
rank 1719
yor1−dF
miss 0
orf −6.9
int 2.1
rank 983
PRR1(YKL116C)
DR = −818
Yor1
miss 0
orf 4.8
int 1.1
rank 1802
yor1−dF
miss 0
orf 0.7
int 2.1
rank 984
(YDR198C)
DR = −1256
Yor1
miss 0
orf −1.5
int −0.7
rank 2241
yor1−dF
miss 0
orf −4.9
int 2.1
rank 985
(YBR108W)
DR = −2564
Yor1
miss 0
orf 0.5
int −6.6
rank 3550
yor1−dF
miss 0
orf 0.3
int 2.1
rank 986
(YCR076C)
DR = −2319
Yor1
miss 0
orf 0.2
int −5.3
rank 3306
yor1−dF
miss 0
orf −0.6
int 2.1
rank 987
ADE8(YDR408C)
DR = −320
Yor1
miss 0
orf 1.1
int 3.2
rank 1308
yor1−dF
miss 0
orf −5.2
int 2.1
rank 988
RSC2(YLR357W)
DR = −2840
Yor1
miss 0
orf 4.7
int −8.5
rank 3829
yor1−dF
miss 0
orf −2.5
int 2.1
rank 989
(YKL061W)
DR = −1418
Yor1
miss 0
orf 2.4
int −1.3
rank 2408
yor1−dF
miss 0
orf −4.4
int 2.1
rank 990
0.35
0.25
0.20
0.15
0.10
0.05
0.00
−60
−20
0
20
40
−50
0
50

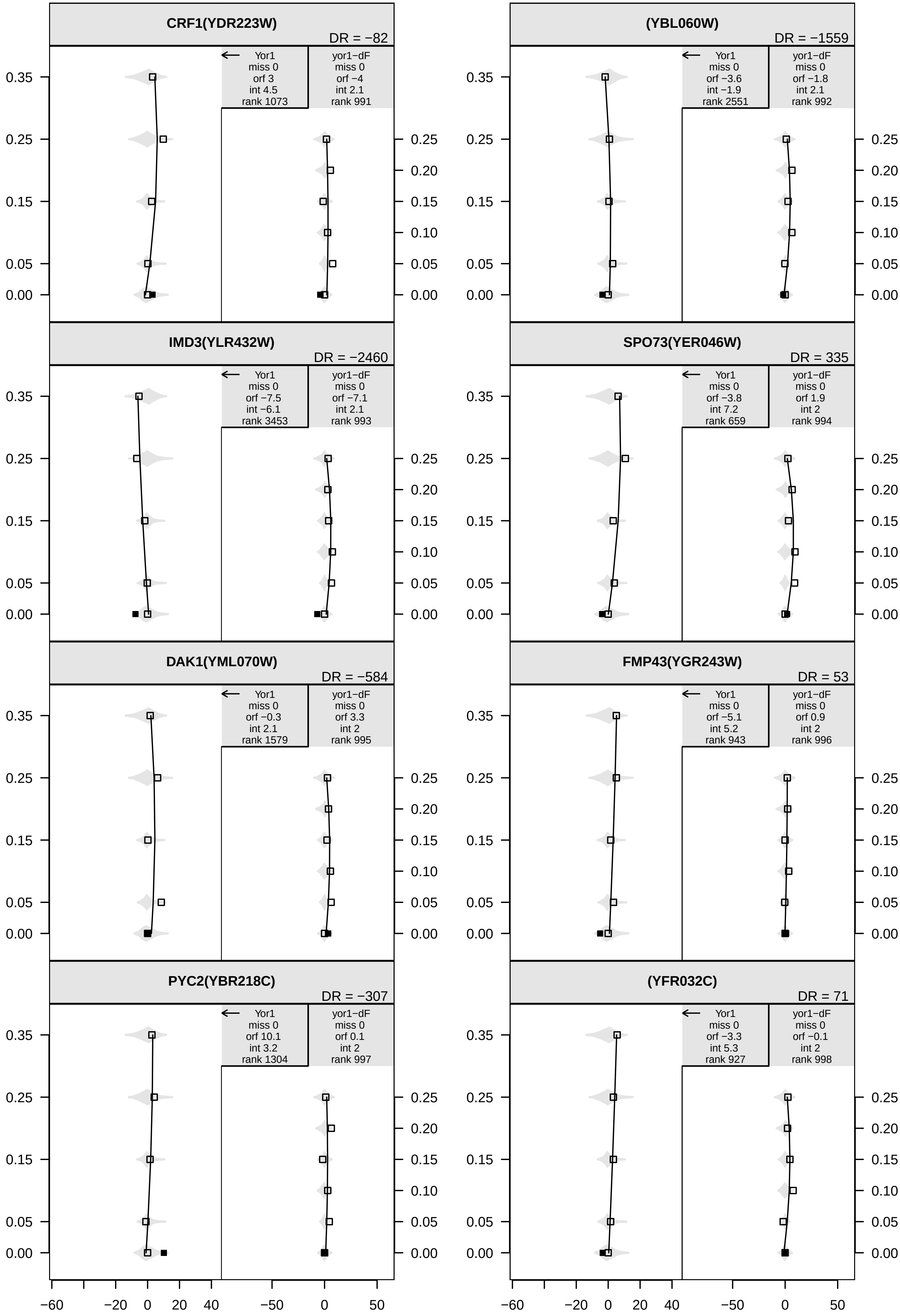
CRF1(YDR223W)
DR = −82
Yor1
miss 0
orf 3
int 4.5
rank 1073
yor1−dF
miss 0
orf −4
int 2.1
rank 991
(YBL060W)
DR = −1559
Yor1
miss 0
orf −3.6
int −1.9
rank 2551
yor1−dF
miss 0
orf −1.8
int 2.1
rank 992
IMD3(YLR432W)
DR = −2460
Yor1
miss 0
orf −7.5
int −6.1
rank 3453
yor1−dF
miss 0
orf −7.1
int 2.1
rank 993
SPO73(YER046W)
DR = 335
Yor1
miss 0
orf −3.8
int 7.2
rank 659
yor1−dF
miss 0
orf 1.9
int 2
rank 994
DAK1(YML070W)
DR = −584
Yor1
miss 0
orf −0.3
int 2.1
rank 1579
yor1−dF
miss 0
orf 3.3
int 2
rank 995
FMP43(YGR243W)
DR = 53
Yor1
miss 0
orf −5.1
int 5.2
rank 943
yor1−dF
miss 0
orf 0.9
int 2
rank 996
PYC2(YBR218C)
DR = −307
Yor1
miss 0
orf 10.1
int 3.2
rank 1304
yor1−dF
miss 0
orf 0.1
int 2
rank 997
(YFR032C)
DR = 71
Yor1
miss 0
orf −3.3
int 5.3
rank 927
yor1−dF
miss 0
orf −0.1
int 2
rank 998
0.35
0.25
0.20
0.15
0.10
0.05
0.00
−60
−20
0
20
40
−50
0
50

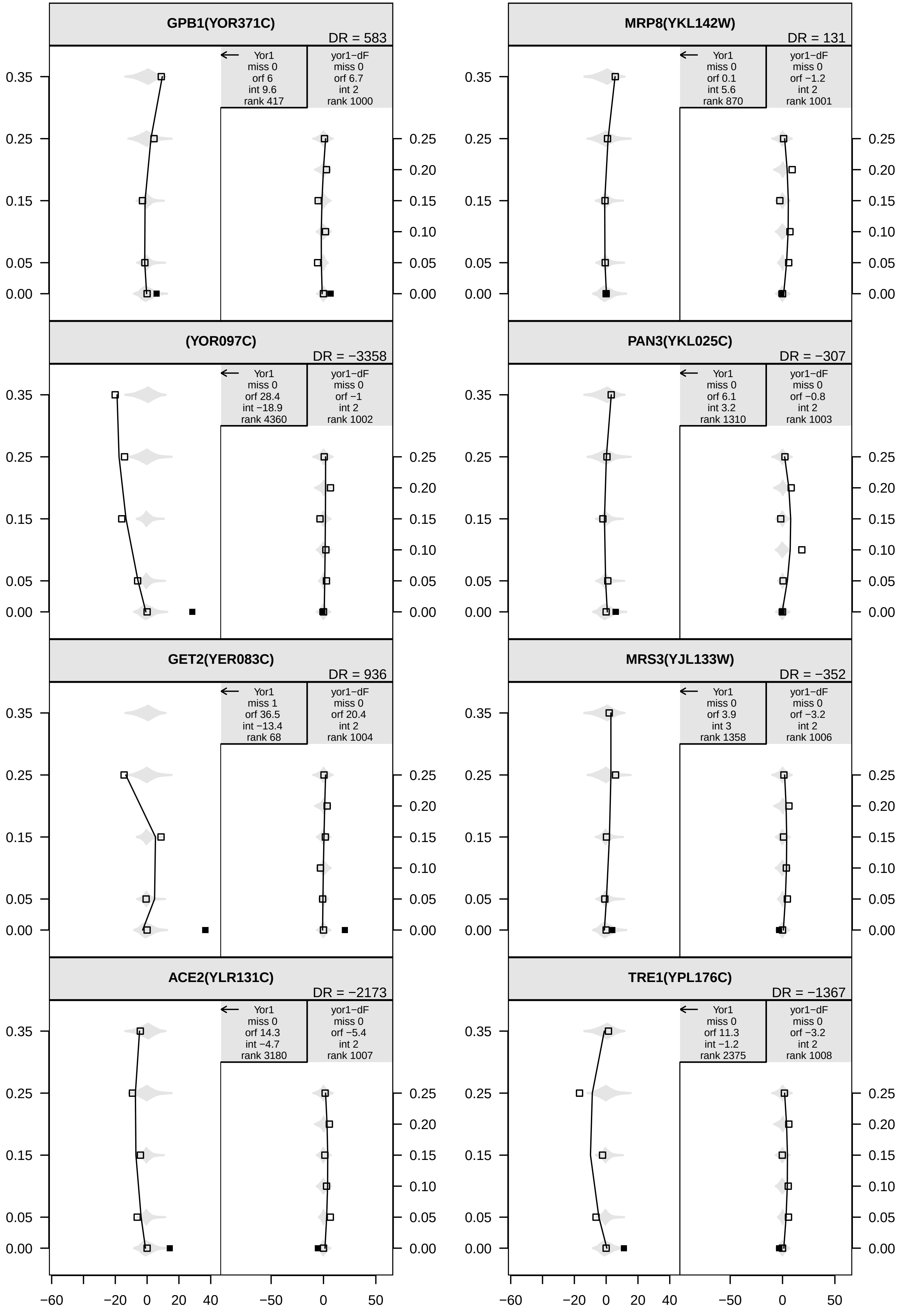
GPB1(YOR371C)
DR = 583
Yor1 miss 0 orf 6 int 9.6 rank 417
yor1−dF miss 0 orf 6.7 int 2 rank 1000
MRP8(YKL142W)
DR = 131
Yor1 miss 0 orf 0.1 int 5.6 rank 870
yor1−dF miss 0 orf −1.2 int 2 rank 1001
(YOR097C)
DR = −3358
Yor1 miss 0 orf 28.4 int −18.9 rank 4360
yor1−dF miss 0 orf −1 int 2 rank 1002
PAN3(YKL025C)
DR = −307
Yor1 miss 0 orf 6.1 int 3.2 rank 1310
yor1−dF miss 0 orf −0.8 int 2 rank 1003
GET2(YER083C)
DR = 936
Yor1 miss 1 orf 36.5 int −13.4 rank 68
yor1−dF miss 0 orf 20.4 int 2 rank 1004
MRS3(YJL133W)
DR = −352
Yor1 miss 0 orf 3.9 int 3 rank 1358
yor1−dF miss 0 orf −3.2 int 2 rank 1006
ACE2(YLR131C)
DR = −2173
Yor1 miss 0 orf 14.3 int −4.7 rank 3180
yor1−dF miss 0 orf −5.4 int 2 rank 1007
TRE1(YPL176C)
DR = −1367
Yor1 miss 0 orf 11.3 int −1.2 rank 2375
yor1−dF miss 0 orf −3.2 int 2 rank 1008
0.00 0.05 0.10 0.15 0.20 0.25 0.35
−60 −20 0 20 40 −50 0 50

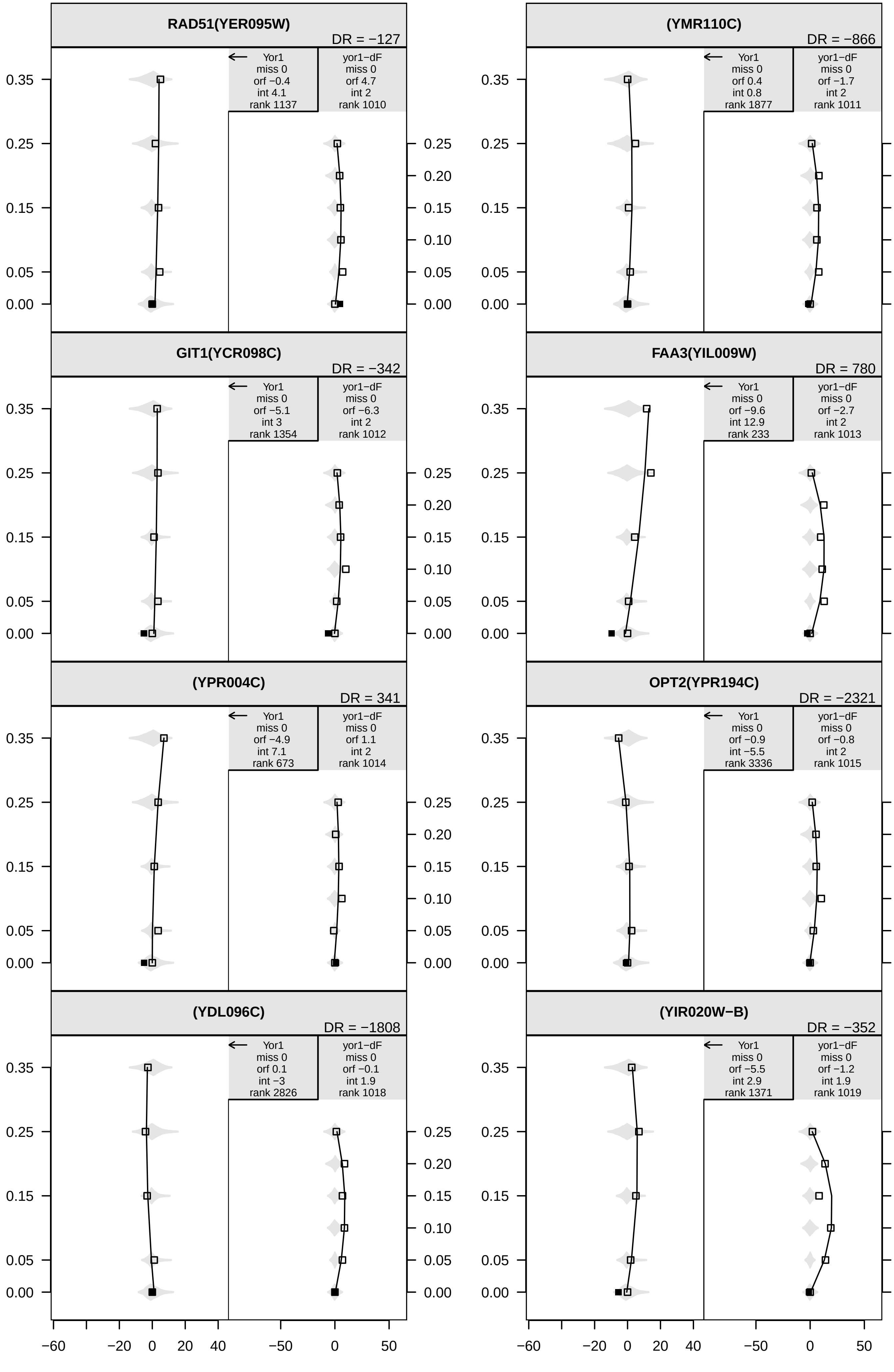

RAD51(YER095W)
DR = −127
Yor1 miss 0 orf −0.4 int 4.1 rank 1137
yor1−dF miss 0 orf 4.7 int 2 rank 1010
(YMR110C)
DR = −866
Yor1 miss 0 orf 0.4 int 0.8 rank 1877
yor1−dF miss 0 orf −1.7 int 2 rank 1011
GIT1(YCR098C)
DR = −342
Yor1 miss 0 orf −5.1 int 3 rank 1354
yor1−dF miss 0 orf −6.3 int 2 rank 1012
FAA3(YIL009W)
DR = 780
Yor1 miss 0 orf −9.6 int 12.9 rank 233
yor1−dF miss 0 orf −2.7 int 2 rank 1013
(YPR004C)
DR = 341
Yor1 miss 0 orf −4.9 int 7.1 rank 673
yor1−dF miss 0 orf 1.1 int 2 rank 1014
OPT2(YPR194C)
DR = −2321
Yor1 miss 0 orf −0.9 int −5.5 rank 3336
yor1−dF miss 0 orf −0.8 int 2 rank 1015
(YDL096C)
DR = −1808
Yor1 miss 0 orf 0.1 int −3 rank 2826
yor1−dF miss 0 orf −0.1 int 1.9 rank 1018
(YIR020W−B)
DR = −352
Yor1 miss 0 orf −5.5 int 2.9 rank 1371
yor1−dF miss 0 orf −1.2 int 1.9 rank 1019
0.00 0.05 0.10 0.15 0.20 0.25 0.35
−60 −20 0 20 40 −50 0 50

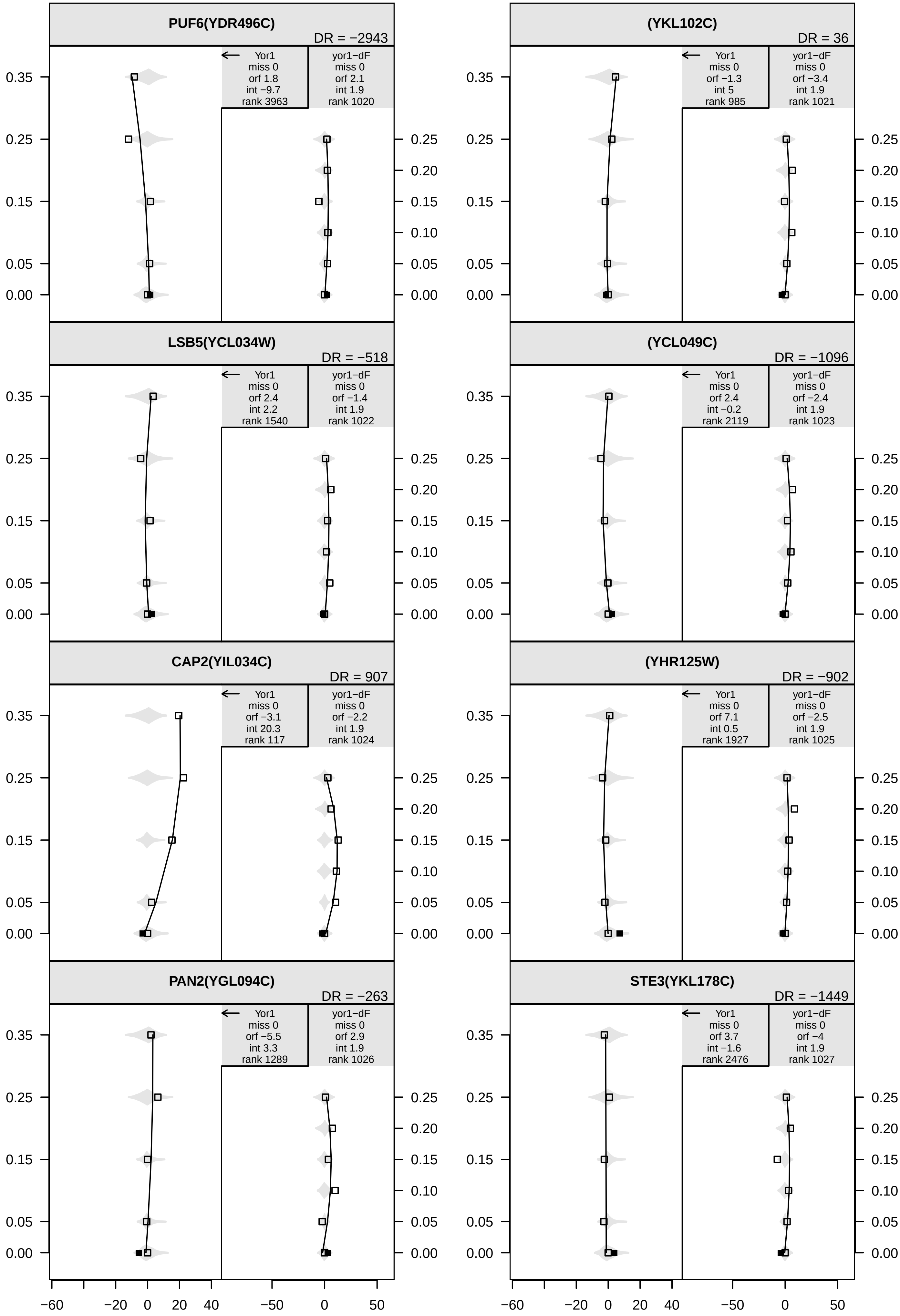
PUF6(YDR496C)
DR = −2943
Yor1 miss 0 orf 1.8 int −9.7 rank 3963
yor1−dF miss 0 orf 2.1 int 1.9 rank 1020
(YKL102C)
DR = 36
Yor1 miss 0 orf −1.3 int 5 rank 985
yor1−dF miss 0 orf −3.4 int 1.9 rank 1021
LSB5(YCL034W)
DR = −518
Yor1 miss 0 orf 2.4 int 2.2 rank 1540
yor1−dF miss 0 orf −1.4 int 1.9 rank 1022
(YCL049C)
DR = −1096
Yor1 miss 0 orf 2.4 int −0.2 rank 2119
yor1−dF miss 0 orf −2.4 int 1.9 rank 1023
CAP2(YIL034C)
DR = 907
Yor1 miss 0 orf −3.1 int 20.3 rank 117
yor1−dF miss 0 orf −2.2 int 1.9 rank 1024
(YHR125W)
DR = −902
Yor1 miss 0 orf 7.1 int 0.5 rank 1927
yor1−dF miss 0 orf −2.5 int 1.9 rank 1025
PAN2(YGL094C)
DR = −263
Yor1 miss 0 orf −5.5 int 3.3 rank 1289
yor1−dF miss 0 orf 2.9 int 1.9 rank 1026
STE3(YKL178C)
DR = −1449
Yor1 miss 0 orf 3.7 int −1.6 rank 2476
yor1−dF miss 0 orf −4 int 1.9 rank 1027
0.35 0.25 0.15 0.05 0.00
0.25 0.20 0.15 0.10 0.05 0.00
−60 −20 0 20 40 −50 0 50

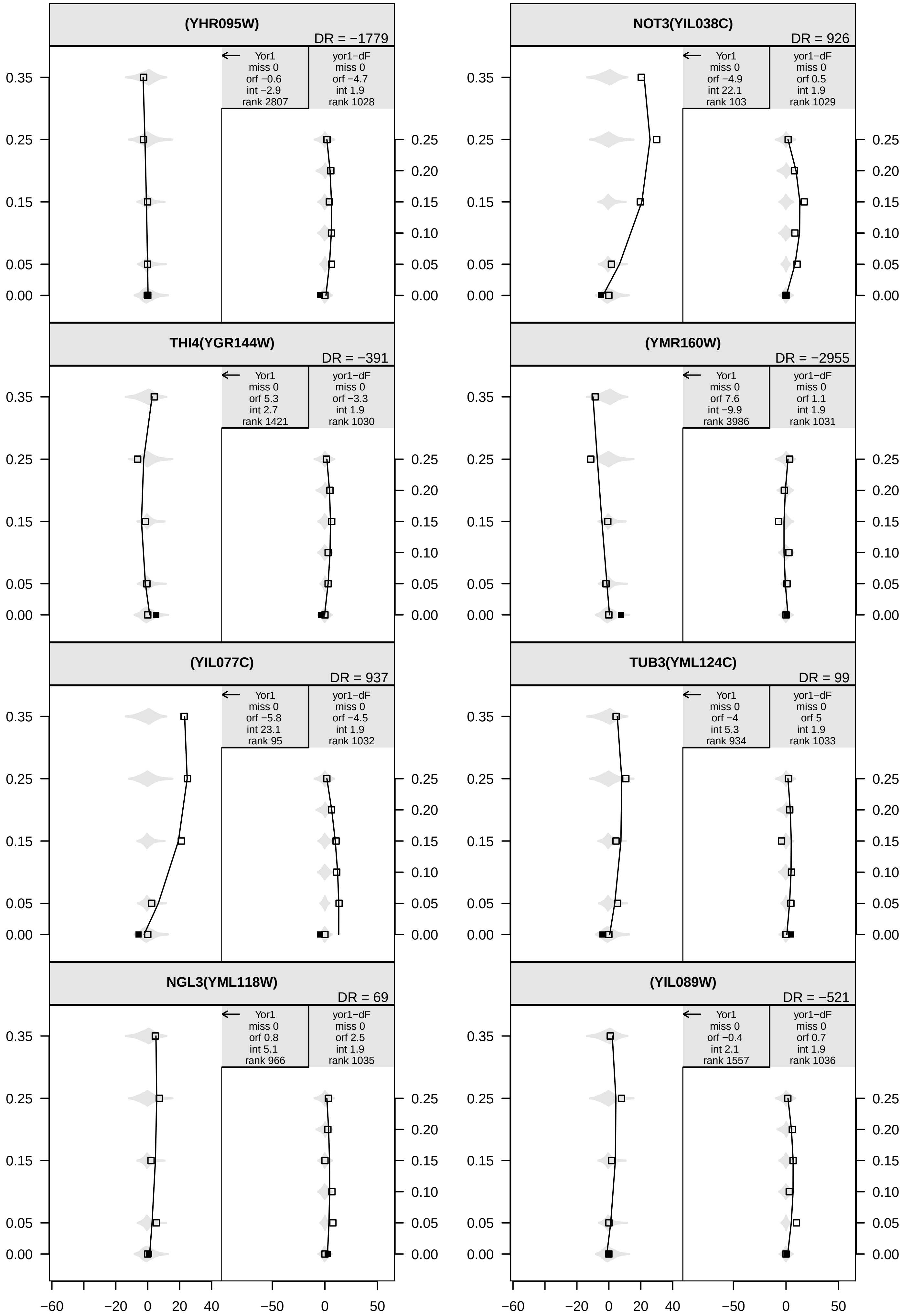

(YHR095W)
DR = −1779
Yor1
miss 0
orf −0.6
int −2.9
rank 2807
yor1−dF
miss 0
orf −4.7
int 1.9
rank 1028
NOT3(YIL038C)
DR = 926
Yor1
miss 0
orf −4.9
int 22.1
rank 103
yor1−dF
miss 0
orf 0.5
int 1.9
rank 1029
THI4(YGR144W)
DR = −391
Yor1
miss 0
orf 5.3
int 2.7
rank 1421
yor1−dF
miss 0
orf −3.3
int 1.9
rank 1030
(YMR160W)
DR = −2955
Yor1
miss 0
orf 7.6
int −9.9
rank 3986
yor1−dF
miss 0
orf 1.1
int 1.9
rank 1031
(YIL077C)
DR = 937
Yor1
miss 0
orf −5.8
int 23.1
rank 95
yor1−dF
miss 0
orf −4.5
int 1.9
rank 1032
TUB3(YML124C)
DR = 99
Yor1
miss 0
orf −4
int 5.3
rank 934
yor1−dF
miss 0
orf 5
int 1.9
rank 1033
NGL3(YML118W)
DR = 69
Yor1
miss 0
orf 0.8
int 5.1
rank 966
yor1−dF
miss 0
orf 2.5
int 1.9
rank 1035
(YIL089W)
DR = −521
Yor1
miss 0
orf −0.4
int 2.1
rank 1557
yor1−dF
miss 0
orf 0.7
int 1.9
rank 1036
0.35
0.25
0.20
0.15
0.10
0.05
0.00
−60
−20
0
20
40
−50
0
50

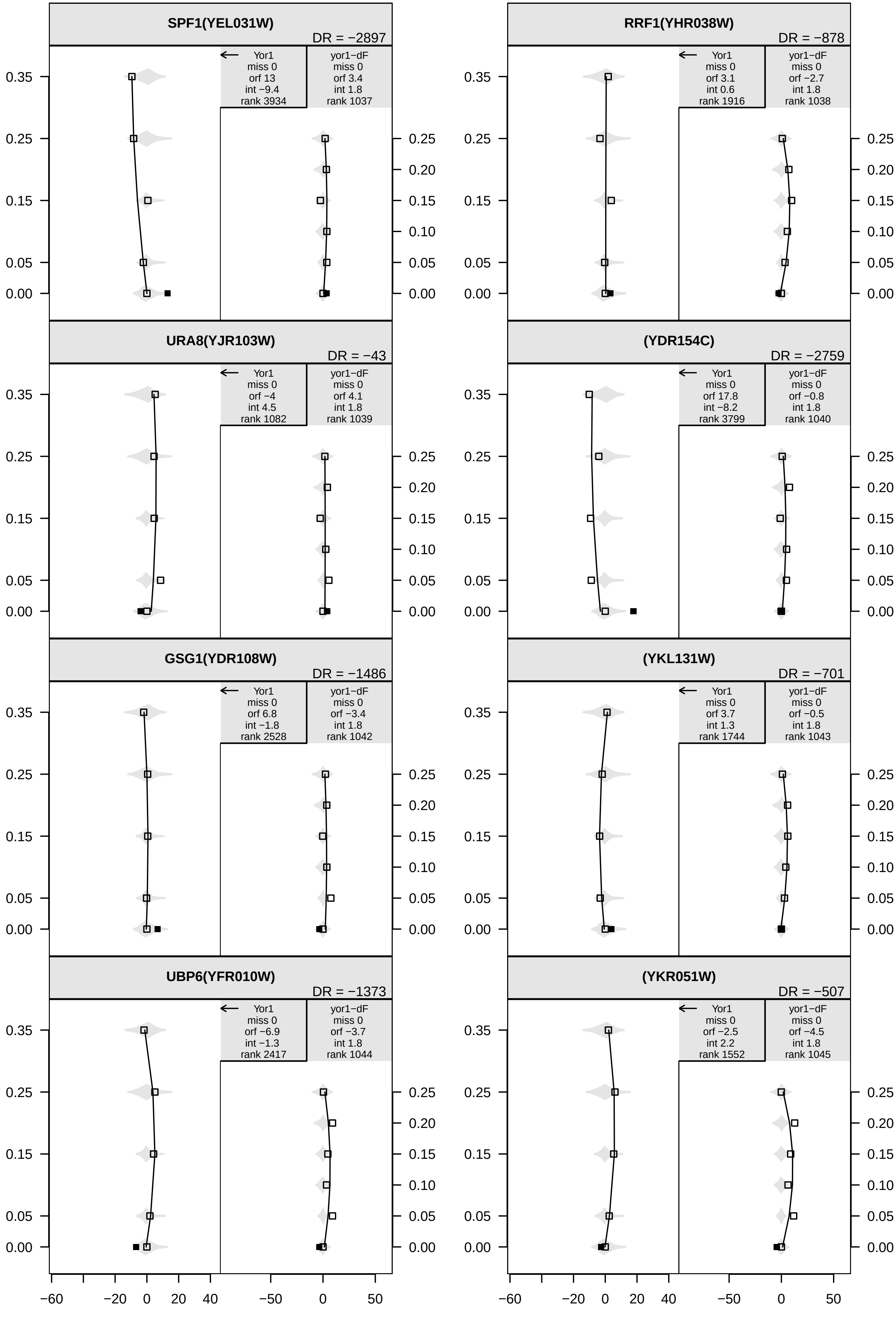
SPF1(YEL031W)
DR = −2897
Yor1 miss 0 orf 13 int −9.4 rank 3934
yor1−dF miss 0 orf 3.4 int 1.8 rank 1037
RRF1(YHR038W)
DR = −878
Yor1 miss 0 orf 3.1 int 0.6 rank 1916
yor1−dF miss 0 orf −2.7 int 1.8 rank 1038
URA8(YJR103W)
DR = −43
Yor1 miss 0 orf −4 int 4.5 rank 1082
yor1−dF miss 0 orf 4.1 int 1.8 rank 1039
(YDR154C)
DR = −2759
Yor1 miss 0 orf 17.8 int −8.2 rank 3799
yor1−dF miss 0 orf −0.8 int 1.8 rank 1040
GSG1(YDR108W)
DR = −1486
Yor1 miss 0 orf 6.8 int −1.8 rank 2528
yor1−dF miss 0 orf −3.4 int 1.8 rank 1042
(YKL131W)
DR = −701
Yor1 miss 0 orf 3.7 int 1.3 rank 1744
yor1−dF miss 0 orf −0.5 int 1.8 rank 1043
UBP6(YFR010W)
DR = −1373
Yor1 miss 0 orf −6.9 int −1.3 rank 2417
yor1−dF miss 0 orf −3.7 int 1.8 rank 1044
(YKR051W)
DR = −507
Yor1 miss 0 orf −2.5 int 2.2 rank 1552
yor1−dF miss 0 orf −4.5 int 1.8 rank 1045
0.35 0.25 0.15 0.05 0.00
0.25 0.20 0.15 0.10 0.05 0.00
−60 −20 0 20 40 −50 0 50

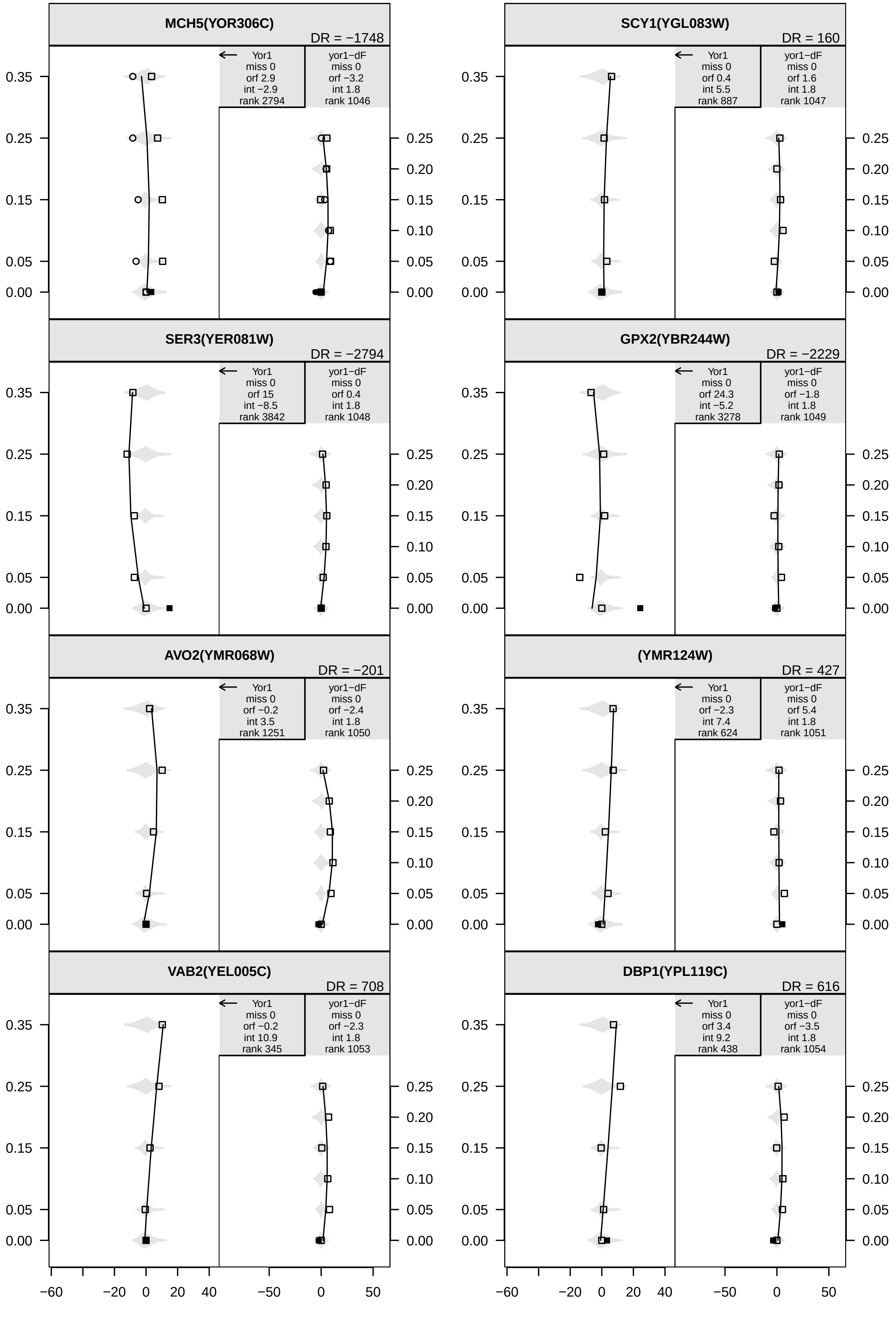
MCH5(YOR306C)
DR = −1748
Yor1 miss 0 orf 2.9 int −2.9 rank 2794
yor1−dF miss 0 orf −3.2 int 1.8 rank 1046
SCY1(YGL083W)
DR = 160
Yor1 miss 0 orf 0.4 int 5.5 rank 887
yor1−dF miss 0 orf 1.6 int 1.8 rank 1047
SER3(YER081W)
DR = −2794
Yor1 miss 0 orf 15 int −8.5 rank 3842
yor1−dF miss 0 orf 0.4 int 1.8 rank 1048
GPX2(YBR244W)
DR = −2229
Yor1 miss 0 orf 24.3 int −5.2 rank 3278
yor1−dF miss 0 orf −1.8 int 1.8 rank 1049
AVO2(YMR068W)
DR = −201
Yor1 miss 0 orf −0.2 int 3.5 rank 1251
yor1−dF miss 0 orf −2.4 int 1.8 rank 1050
(YMR124W)
DR = 427
Yor1 miss 0 orf −2.3 int 7.4 rank 624
yor1−dF miss 0 orf 5.4 int 1.8 rank 1051
VAB2(YEL005C)
DR = 708
Yor1 miss 0 orf −0.2 int 10.9 rank 345
yor1−dF miss 0 orf −2.3 int 1.8 rank 1053
DBP1(YPL119C)
DR = 616
Yor1 miss 0 orf 3.4 int 9.2 rank 438
yor1−dF miss 0 orf −3.5 int 1.8 rank 1054
0.35 0.25 0.15 0.05 0.00
0.25 0.20 0.15 0.10 0.05 0.00
−60 −20 0 20 40 −50 0 50

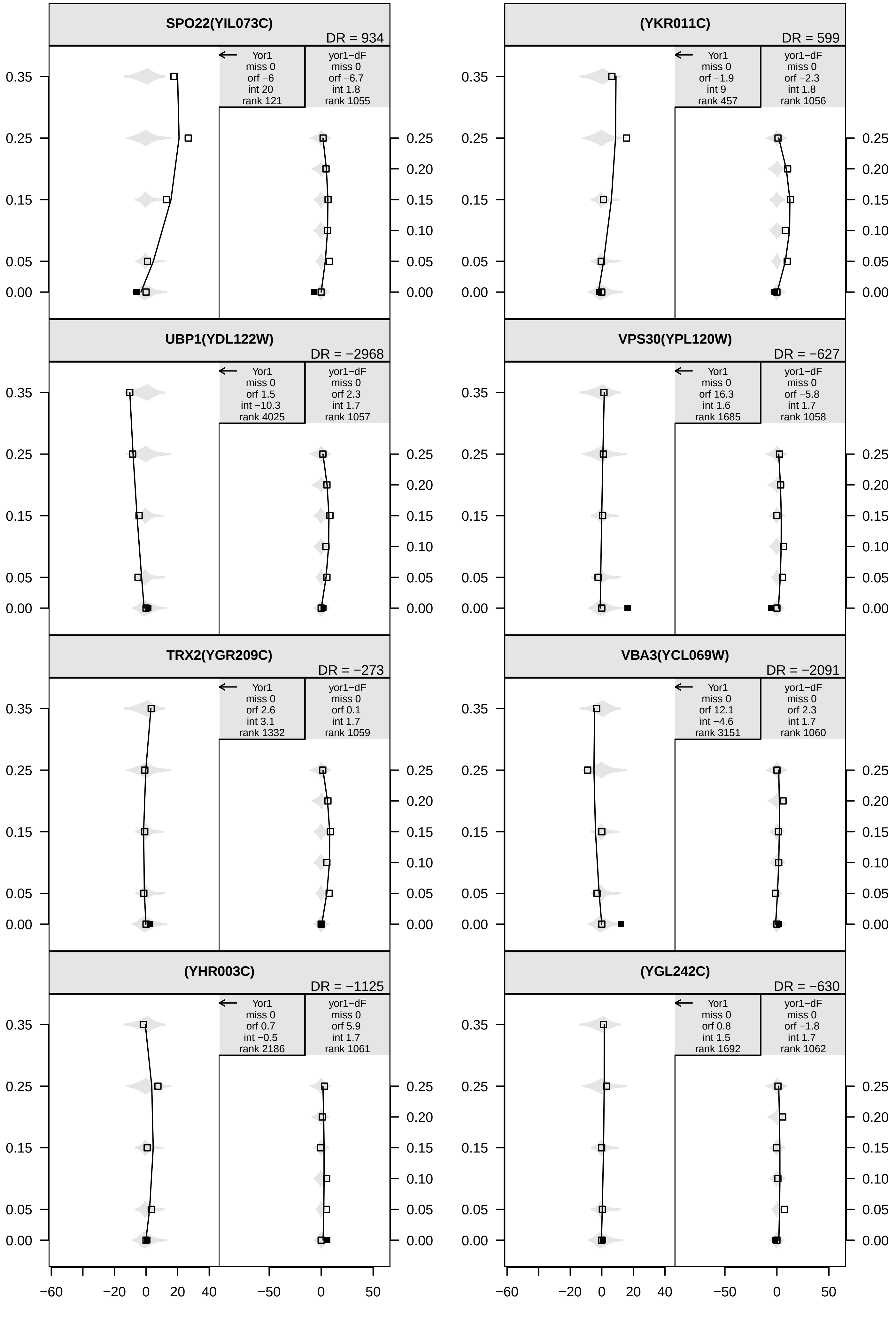
SPO22(YIL073C)
DR = 934
Yor1
miss 0
orf −6
int 20
rank 121
yor1−dF
miss 0
orf −6.7
int 1.8
rank 1055
(YKR011C)
DR = 599
Yor1
miss 0
orf −1.9
int 9
rank 457
yor1−dF
miss 0
orf −2.3
int 1.8
rank 1056
UBP1(YDL122W)
DR = −2968
Yor1
miss 0
orf 1.5
int −10.3
rank 4025
yor1−dF
miss 0
orf 2.3
int 1.7
rank 1057
VPS30(YPL120W)
DR = −627
Yor1
miss 0
orf 16.3
int 1.6
rank 1685
yor1−dF
miss 0
orf −5.8
int 1.7
rank 1058
TRX2(YGR209C)
DR = −273
Yor1
miss 0
orf 2.6
int 3.1
rank 1332
yor1−dF
miss 0
orf 0.1
int 1.7
rank 1059
VBA3(YCL069W)
DR = −2091
Yor1
miss 0
orf 12.1
int −4.6
rank 3151
yor1−dF
miss 0
orf 2.3
int 1.7
rank 1060
(YHR003C)
DR = −1125
Yor1
miss 0
orf 0.7
int −0.5
rank 2186
yor1−dF
miss 0
orf 5.9
int 1.7
rank 1061
(YGL242C)
DR = −630
Yor1
miss 0
orf 0.8
int 1.5
rank 1692
yor1−dF
miss 0
orf −1.8
int 1.7
rank 1062
0.00
0.05
0.10
0.15
0.20
0.25
0.35
−60
−20
0
20
40
−50
0
50

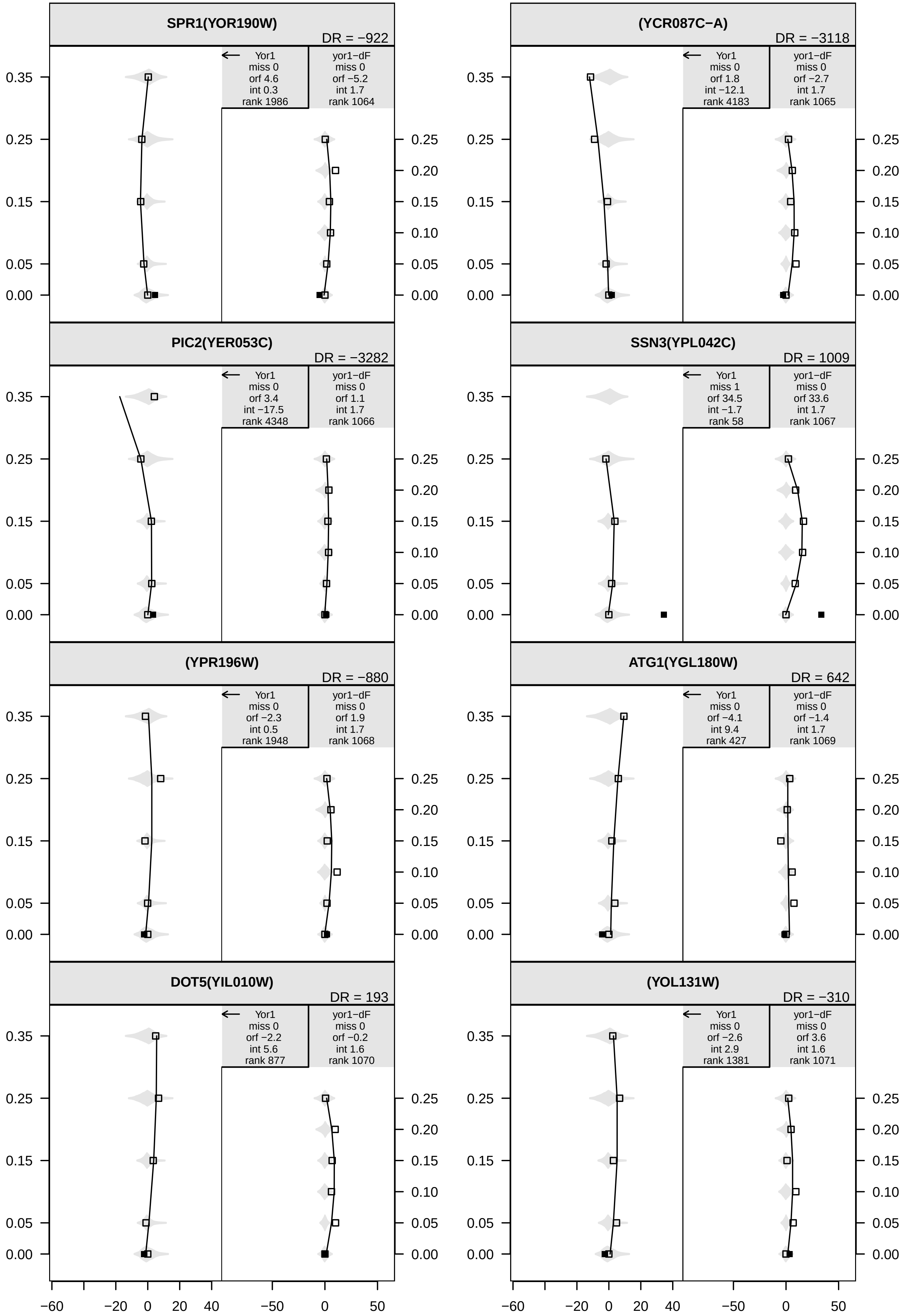
SPR1(YOR190W)
DR = −922
Yor1 miss 0 orf 4.6 int 0.3 rank 1986
yor1−dF miss 0 orf −5.2 int 1.7 rank 1064
(YCR087C−A)
DR = −3118
Yor1 miss 0 orf 1.8 int −12.1 rank 4183
yor1−dF miss 0 orf −2.7 int 1.7 rank 1065
PIC2(YER053C)
DR = −3282
Yor1 miss 0 orf 3.4 int −17.5 rank 4348
yor1−dF miss 0 orf 1.1 int 1.7 rank 1066
SSN3(YPL042C)
DR = 1009
Yor1 miss 1 orf 34.5 int −1.7 rank 58
yor1−dF miss 0 orf 33.6 int 1.7 rank 1067
(YPR196W)
DR = −880
Yor1 miss 0 orf −2.3 int 0.5 rank 1948
yor1−dF miss 0 orf 1.9 int 1.7 rank 1068
ATG1(YGL180W)
DR = 642
Yor1 miss 0 orf −4.1 int 9.4 rank 427
yor1−dF miss 0 orf −1.4 int 1.7 rank 1069
DOT5(YIL010W)
DR = 193
Yor1 miss 0 orf −2.2 int 5.6 rank 877
yor1−dF miss 0 orf −0.2 int 1.6 rank 1070
(YOL131W)
DR = −310
Yor1 miss 0 orf −2.6 int 2.9 rank 1381
yor1−dF miss 0 orf 3.6 int 1.6 rank 1071
0.35 0.25 0.15 0.05 0.00
0.25 0.20 0.15 0.10 0.05 0.00
−60 −20 0 20 40 −50 0 50

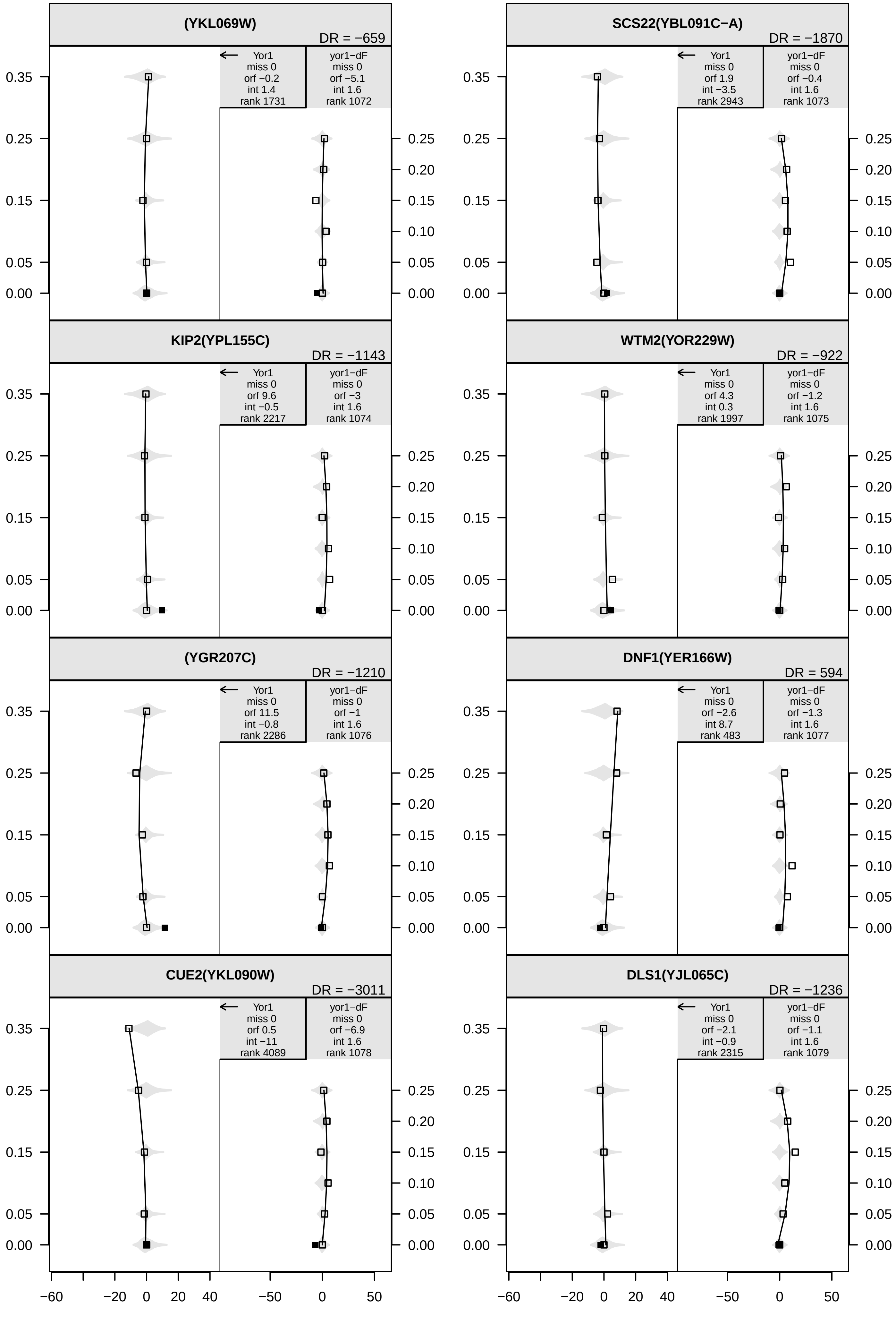

(YKL069W)
DR = −659
Yor1
miss 0
orf −0.2
int 1.4
rank 1731
yor1−dF
miss 0
orf −5.1
int 1.6
rank 1072
SCS22(YBL091C−A)
DR = −1870
Yor1
miss 0
orf 1.9
int −3.5
rank 2943
yor1−dF
miss 0
orf −0.4
int 1.6
rank 1073
KIP2(YPL155C)
DR = −1143
Yor1
miss 0
orf 9.6
int −0.5
rank 2217
yor1−dF
miss 0
orf −3
int 1.6
rank 1074
WTM2(YOR229W)
DR = −922
Yor1
miss 0
orf 4.3
int 0.3
rank 1997
yor1−dF
miss 0
orf −1.2
int 1.6
rank 1075
(YGR207C)
DR = −1210
Yor1
miss 0
orf 11.5
int −0.8
rank 2286
yor1−dF
miss 0
orf −1
int 1.6
rank 1076
DNF1(YER166W)
DR = 594
Yor1
miss 0
orf −2.6
int 8.7
rank 483
yor1−dF
miss 0
orf −1.3
int 1.6
rank 1077
CUE2(YKL090W)
DR = −3011
Yor1
miss 0
orf 0.5
int −11
rank 4089
yor1−dF
miss 0
orf −6.9
int 1.6
rank 1078
DLS1(YJL065C)
DR = −1236
Yor1
miss 0
orf −2.1
int −0.9
rank 2315
yor1−dF
miss 0
orf −1.1
int 1.6
rank 1079
0.35
0.25
0.20
0.15
0.10
0.05
0.00
−60
−20
0
20
40
−50
0
50

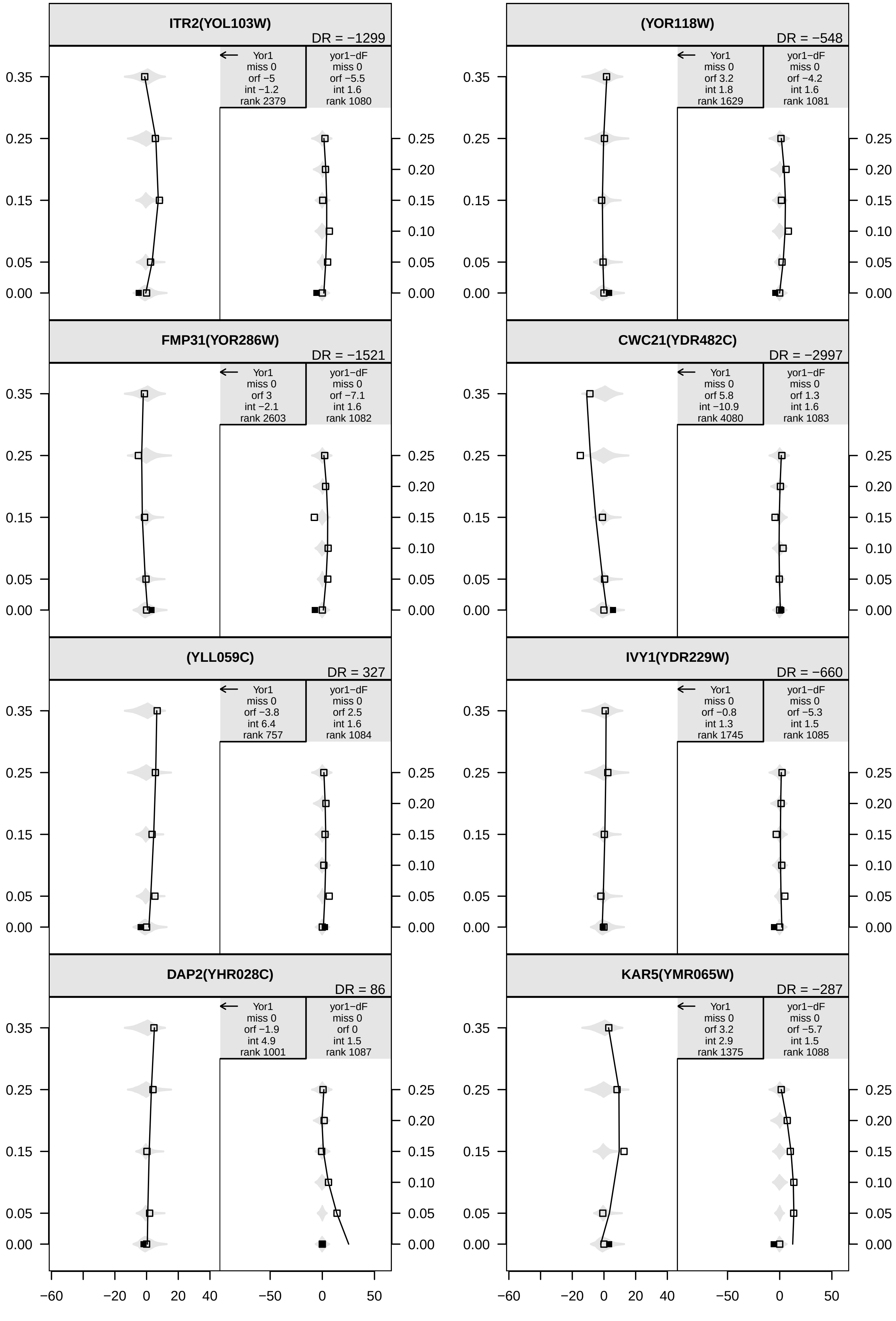

ITR2(YOL103W)
DR = −1299
Yor1 miss 0 orf −5 int −1.2 rank 2379
yor1−dF miss 0 orf −5.5 int 1.6 rank 1080
(YOR118W)
DR = −548
Yor1 miss 0 orf 3.2 int 1.8 rank 1629
yor1−dF miss 0 orf −4.2 int 1.6 rank 1081
FMP31(YOR286W)
DR = −1521
Yor1 miss 0 orf 3 int −2.1 rank 2603
yor1−dF miss 0 orf −7.1 int 1.6 rank 1082
CWC21(YDR482C)
DR = −2997
Yor1 miss 0 orf 5.8 int −10.9 rank 4080
yor1−dF miss 0 orf 1.3 int 1.6 rank 1083
(YLL059C)
DR = 327
Yor1 miss 0 orf −3.8 int 6.4 rank 757
yor1−dF miss 0 orf 2.5 int 1.6 rank 1084
IVY1(YDR229W)
DR = −660
Yor1 miss 0 orf −0.8 int 1.3 rank 1745
yor1−dF miss 0 orf −5.3 int 1.5 rank 1085
DAP2(YHR028C)
DR = 86
Yor1 miss 0 orf −1.9 int 4.9 rank 1001
yor1−dF miss 0 orf 0 int 1.5 rank 1087
KAR5(YMR065W)
DR = −287
Yor1 miss 0 orf 3.2 int 2.9 rank 1375
yor1−dF miss 0 orf −5.7 int 1.5 rank 1088
0.00 0.05 0.10 0.15 0.20 0.25 0.35
−60 −20 0 20 40 −50 0 50

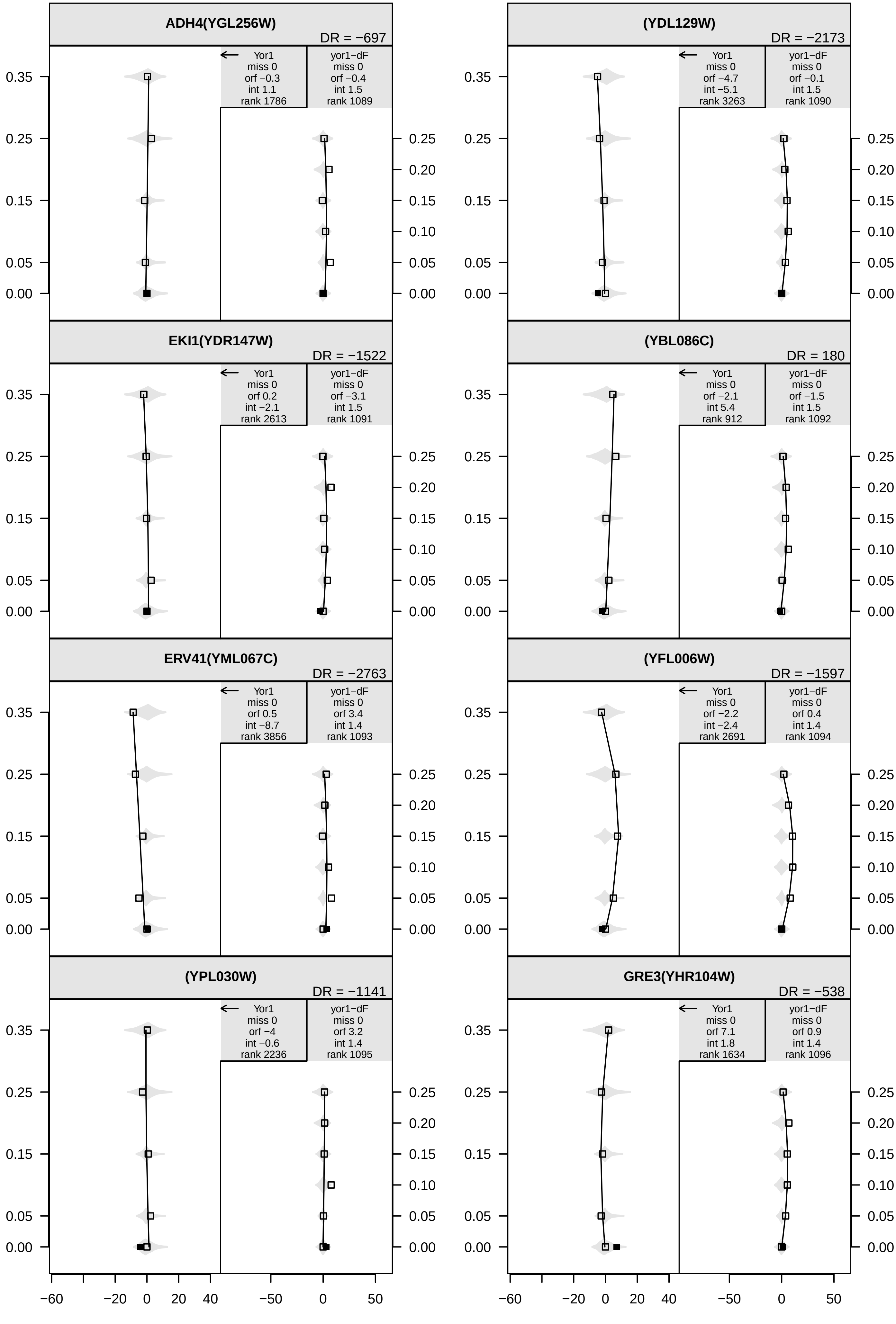
ADH4(YGL256W)
DR = −697
Yor1 miss 0 orf −0.3 int 1.1 rank 1786
yor1−dF miss 0 orf −0.4 int 1.5 rank 1089
(YDL129W)
DR = −2173
Yor1 miss 0 orf −4.7 int −5.1 rank 3263
yor1−dF miss 0 orf −0.1 int 1.5 rank 1090
EKI1(YDR147W)
DR = −1522
Yor1 miss 0 orf 0.2 int −2.1 rank 2613
yor1−dF miss 0 orf −3.1 int 1.5 rank 1091
(YBL086C)
DR = 180
Yor1 miss 0 orf −2.1 int 5.4 rank 912
yor1−dF miss 0 orf −1.5 int 1.5 rank 1092
ERV41(YML067C)
DR = −2763
Yor1 miss 0 orf 0.5 int −8.7 rank 3856
yor1−dF miss 0 orf 3.4 int 1.4 rank 1093
(YFL006W)
DR = −1597
Yor1 miss 0 orf −2.2 int −2.4 rank 2691
yor1−dF miss 0 orf 0.4 int 1.4 rank 1094
(YPL030W)
DR = −1141
Yor1 miss 0 orf −4 int −0.6 rank 2236
yor1−dF miss 0 orf 3.2 int 1.4 rank 1095
GRE3(YHR104W)
DR = −538
Yor1 miss 0 orf 7.1 int 1.8 rank 1634
yor1−dF miss 0 orf 0.9 int 1.4 rank 1096
0.00
0.05
0.10
0.15
0.20
0.25
0.35
−60
−20
0
20
40
−50
0
50

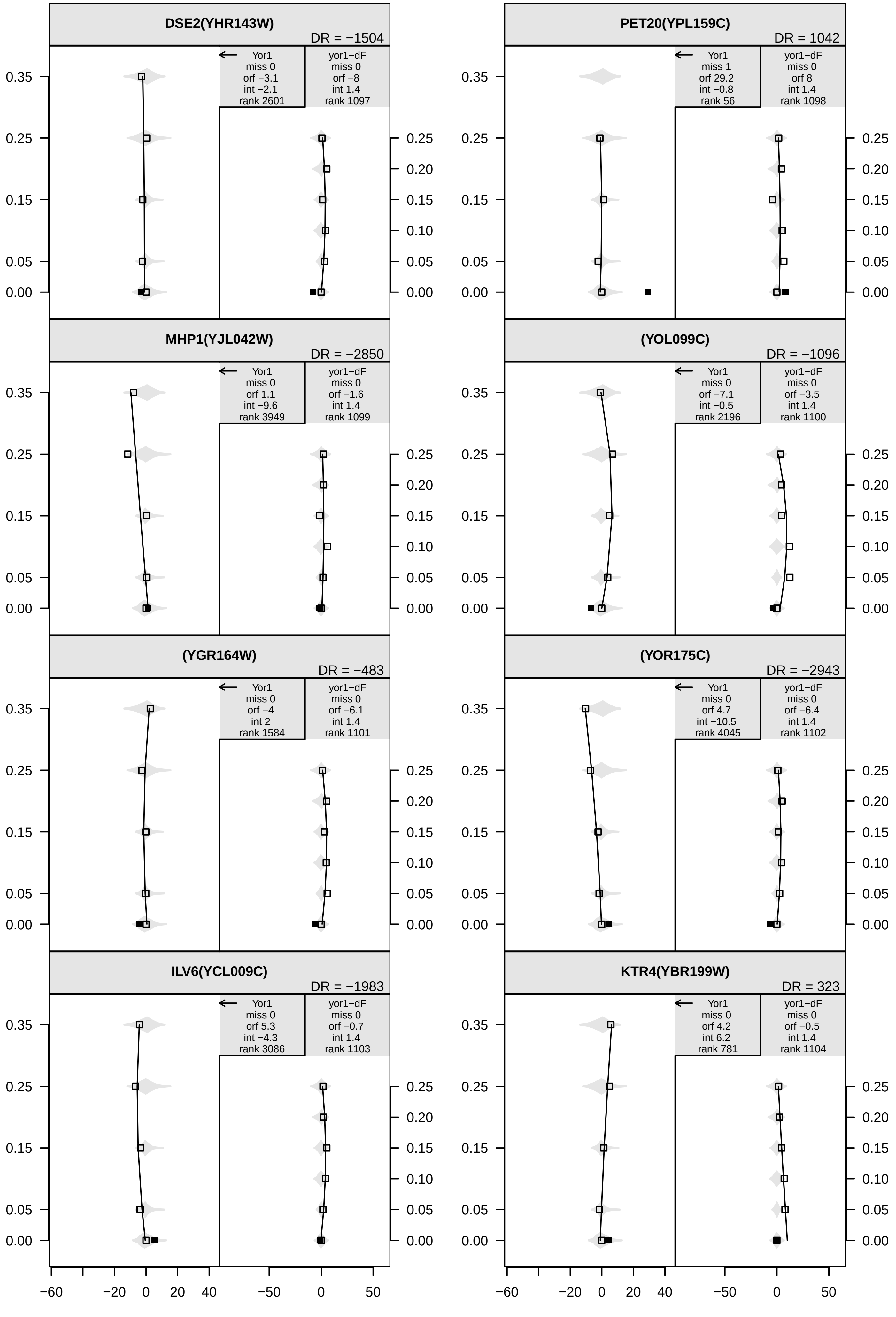

DSE2(YHR143W)
DR = −1504
Yor1 miss 0 orf −3.1 int −2.1 rank 2601
yor1−dF miss 0 orf −8 int 1.4 rank 1097
PET20(YPL159C)
DR = 1042
Yor1 miss 1 orf 29.2 int −0.8 rank 56
yor1−dF miss 0 orf 8 int 1.4 rank 1098
MHP1(YJL042W)
DR = −2850
Yor1 miss 0 orf 1.1 int −9.6 rank 3949
yor1−dF miss 0 orf −1.6 int 1.4 rank 1099
(YOL099C)
DR = −1096
Yor1 miss 0 orf −7.1 int −0.5 rank 2196
yor1−dF miss 0 orf −3.5 int 1.4 rank 1100
(YGR164W)
DR = −483
Yor1 miss 0 orf −4 int 2 rank 1584
yor1−dF miss 0 orf −6.1 int 1.4 rank 1101
(YOR175C)
DR = −2943
Yor1 miss 0 orf 4.7 int −10.5 rank 4045
yor1−dF miss 0 orf −6.4 int 1.4 rank 1102
ILV6(YCL009C)
DR = −1983
Yor1 miss 0 orf 5.3 int −4.3 rank 3086
yor1−dF miss 0 orf −0.7 int 1.4 rank 1103
KTR4(YBR199W)
DR = 323
Yor1 miss 0 orf 4.2 int 6.2 rank 781
yor1−dF miss 0 orf −0.5 int 1.4 rank 1104
0.00 0.05 0.10 0.15 0.20 0.25 0.35
−60 −20 0 20 40 −50 0 50

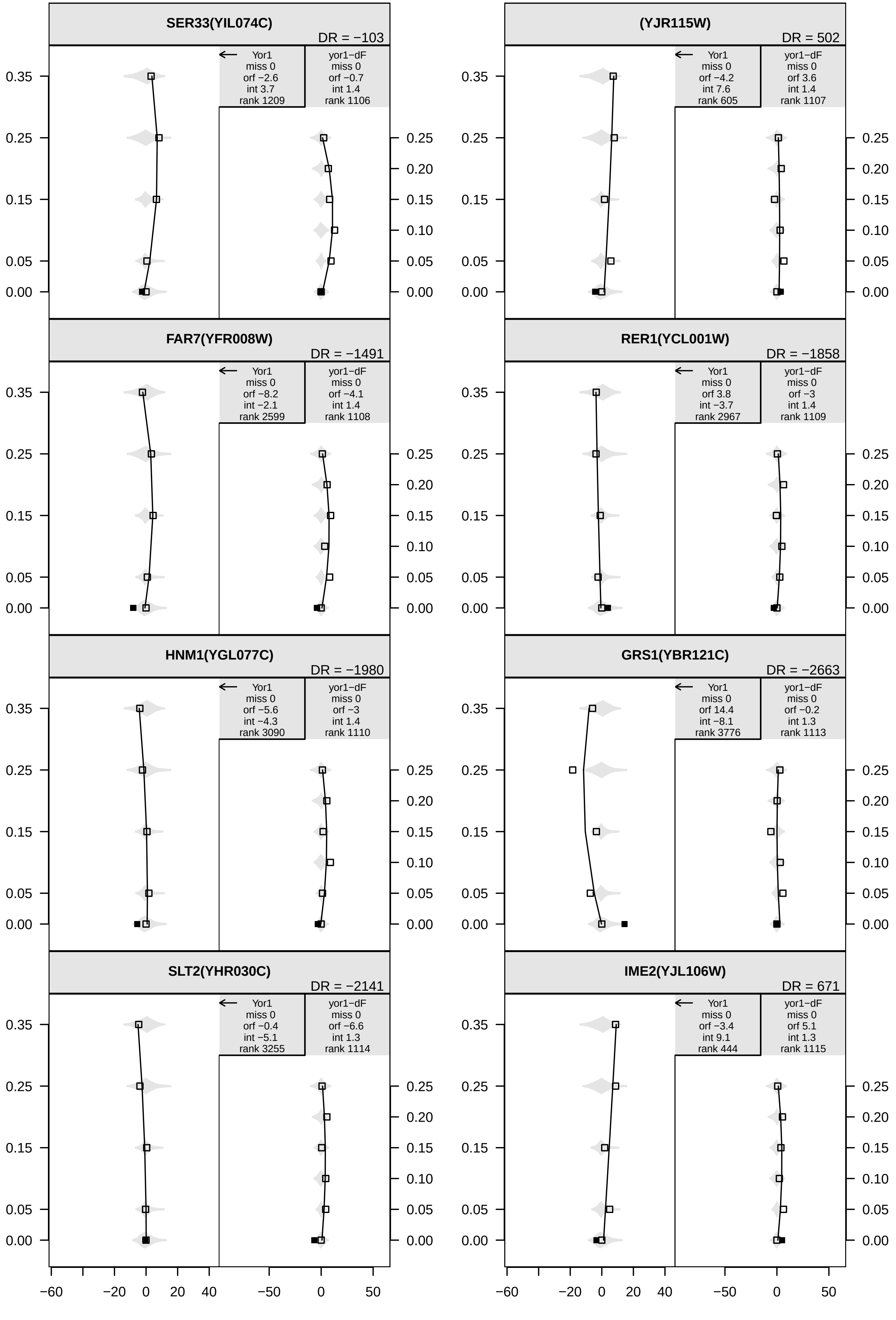

SER33(YIL074C)
DR = −103
Yor1 miss 0 orf −2.6 int 3.7 rank 1209
yor1−dF miss 0 orf −0.7 int 1.4 rank 1106
(YJR115W)
DR = 502
Yor1 miss 0 orf −4.2 int 7.6 rank 605
yor1−dF miss 0 orf 3.6 int 1.4 rank 1107
FAR7(YFR008W)
DR = −1491
Yor1 miss 0 orf −8.2 int −2.1 rank 2599
yor1−dF miss 0 orf −4.1 int 1.4 rank 1108
RER1(YCL001W)
DR = −1858
Yor1 miss 0 orf 3.8 int −3.7 rank 2967
yor1−dF miss 0 orf −3 int 1.4 rank 1109
HNM1(YGL077C)
DR = −1980
Yor1 miss 0 orf −5.6 int −4.3 rank 3090
yor1−dF miss 0 orf −3 int 1.4 rank 1110
GRS1(YBR121C)
DR = −2663
Yor1 miss 0 orf 14.4 int −8.1 rank 3776
yor1−dF miss 0 orf −0.2 int 1.3 rank 1113
SLT2(YHR030C)
DR = −2141
Yor1 miss 0 orf −0.4 int −5.1 rank 3255
yor1−dF miss 0 orf −6.6 int 1.3 rank 1114
IME2(YJL106W)
DR = 671
Yor1 miss 0 orf −3.4 int 9.1 rank 444
yor1−dF miss 0 orf 5.1 int 1.3 rank 1115

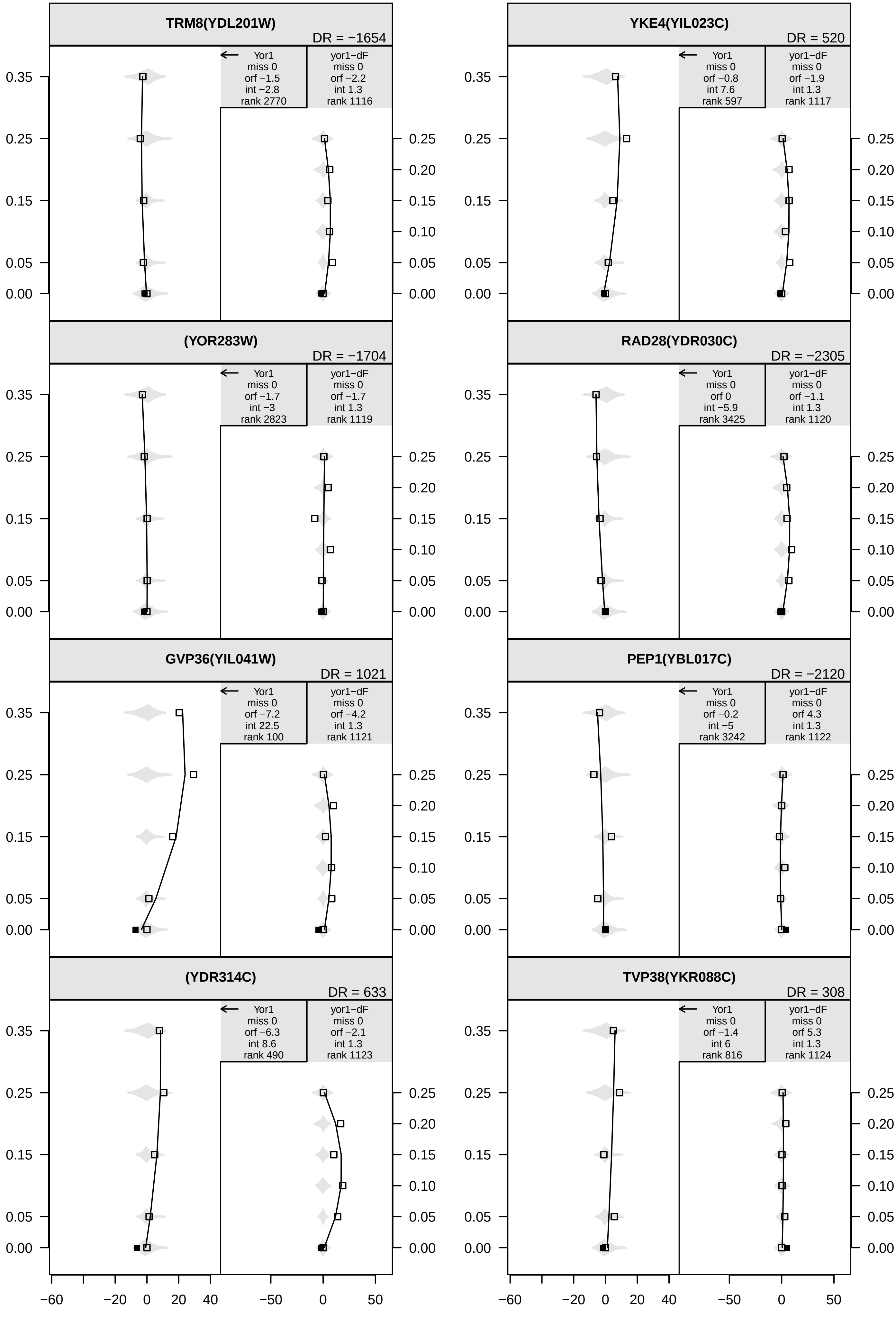
TRM8(YDL201W)
DR = −1654
Yor1 miss 0 orf −1.5 int −2.8 rank 2770
yor1−dF miss 0 orf −2.2 int 1.3 rank 1116
YKE4(YIL023C)
DR = 520
Yor1 miss 0 orf −0.8 int 7.6 rank 597
yor1−dF miss 0 orf −1.9 int 1.3 rank 1117
(YOR283W)
DR = −1704
Yor1 miss 0 orf −1.7 int −3 rank 2823
yor1−dF miss 0 orf −1.7 int 1.3 rank 1119
RAD28(YDR030C)
DR = −2305
Yor1 miss 0 orf 0 int −5.9 rank 3425
yor1−dF miss 0 orf −1.1 int 1.3 rank 1120
GVP36(YIL041W)
DR = 1021
Yor1 miss 0 orf −7.2 int 22.5 rank 100
yor1−dF miss 0 orf −4.2 int 1.3 rank 1121
PEP1(YBL017C)
DR = −2120
Yor1 miss 0 orf −0.2 int −5 rank 3242
yor1−dF miss 0 orf 4.3 int 1.3 rank 1122
(YDR314C)
DR = 633
Yor1 miss 0 orf −6.3 int 8.6 rank 490
yor1−dF miss 0 orf −2.1 int 1.3 rank 1123
TVP38(YKR088C)
DR = 308
Yor1 miss 0 orf −1.4 int 6 rank 816
yor1−dF miss 0 orf 5.3 int 1.3 rank 1124
0.00 0.05 0.15 0.25 0.35
0.00 0.05 0.10 0.15 0.20 0.25
−60 −20 0 20 40 −50 0 50

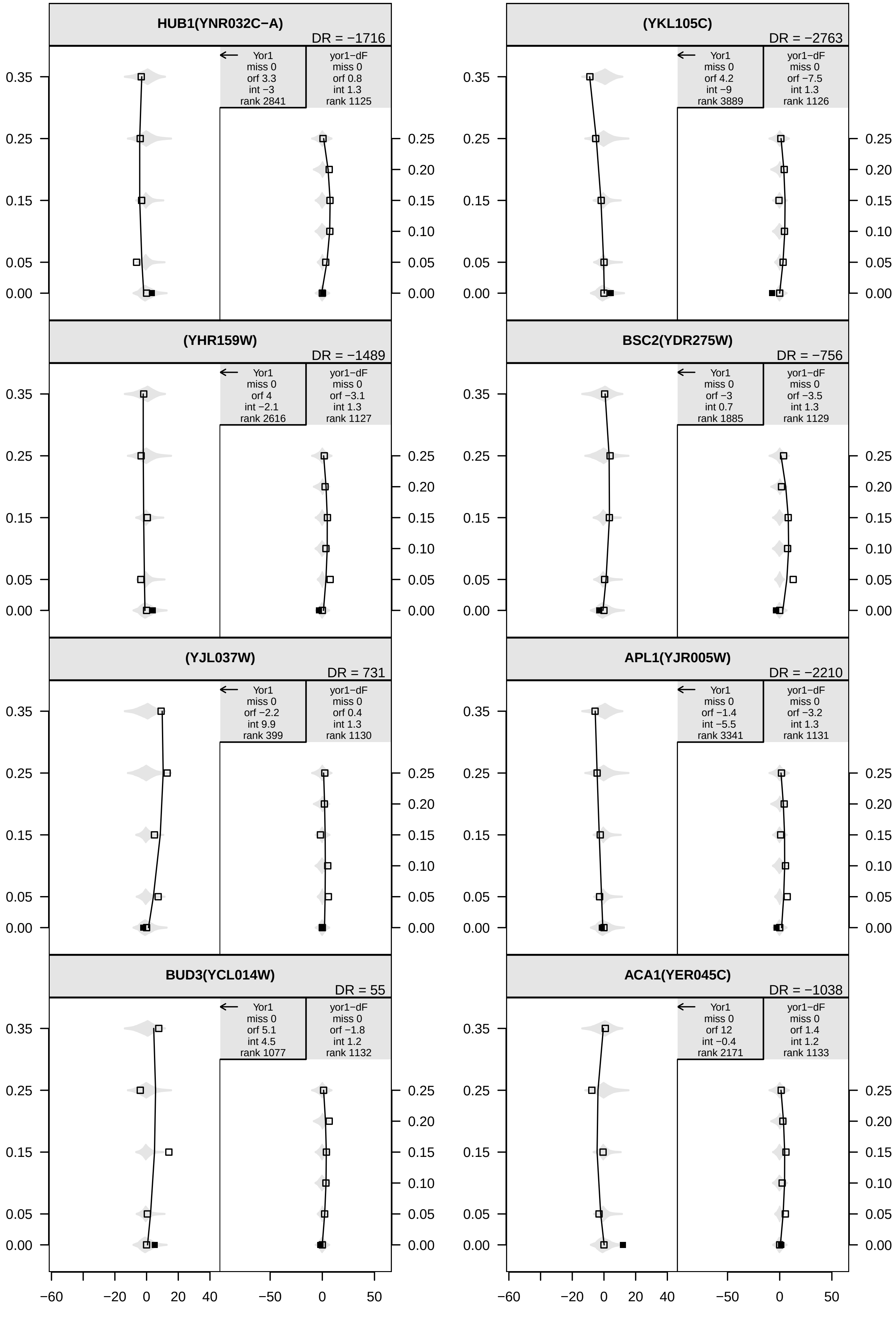

HUB1(YNR032C−A)
DR = −1716
Yor1 miss 0 orf 3.3 int −3 rank 2841
yor1−dF miss 0 orf 0.8 int 1.3 rank 1125
(YKL105C)
DR = −2763
Yor1 miss 0 orf 4.2 int −9 rank 3889
yor1−dF miss 0 orf −7.5 int 1.3 rank 1126
(YHR159W)
DR = −1489
Yor1 miss 0 orf 4 int −2.1 rank 2616
yor1−dF miss 0 orf −3.1 int 1.3 rank 1127
BSC2(YDR275W)
DR = −756
Yor1 miss 0 orf −3 int 0.7 rank 1885
yor1−dF miss 0 orf −3.5 int 1.3 rank 1129
(YJL037W)
DR = 731
Yor1 miss 0 orf −2.2 int 9.9 rank 399
yor1−dF miss 0 orf 0.4 int 1.3 rank 1130
APL1(YJR005W)
DR = −2210
Yor1 miss 0 orf −1.4 int −5.5 rank 3341
yor1−dF miss 0 orf −3.2 int 1.3 rank 1131
BUD3(YCL014W)
DR = 55
Yor1 miss 0 orf 5.1 int 4.5 rank 1077
yor1−dF miss 0 orf −1.8 int 1.2 rank 1132
ACA1(YER045C)
DR = −1038
Yor1 miss 0 orf 12 int −0.4 rank 2171
yor1−dF miss 0 orf 1.4 int 1.2 rank 1133

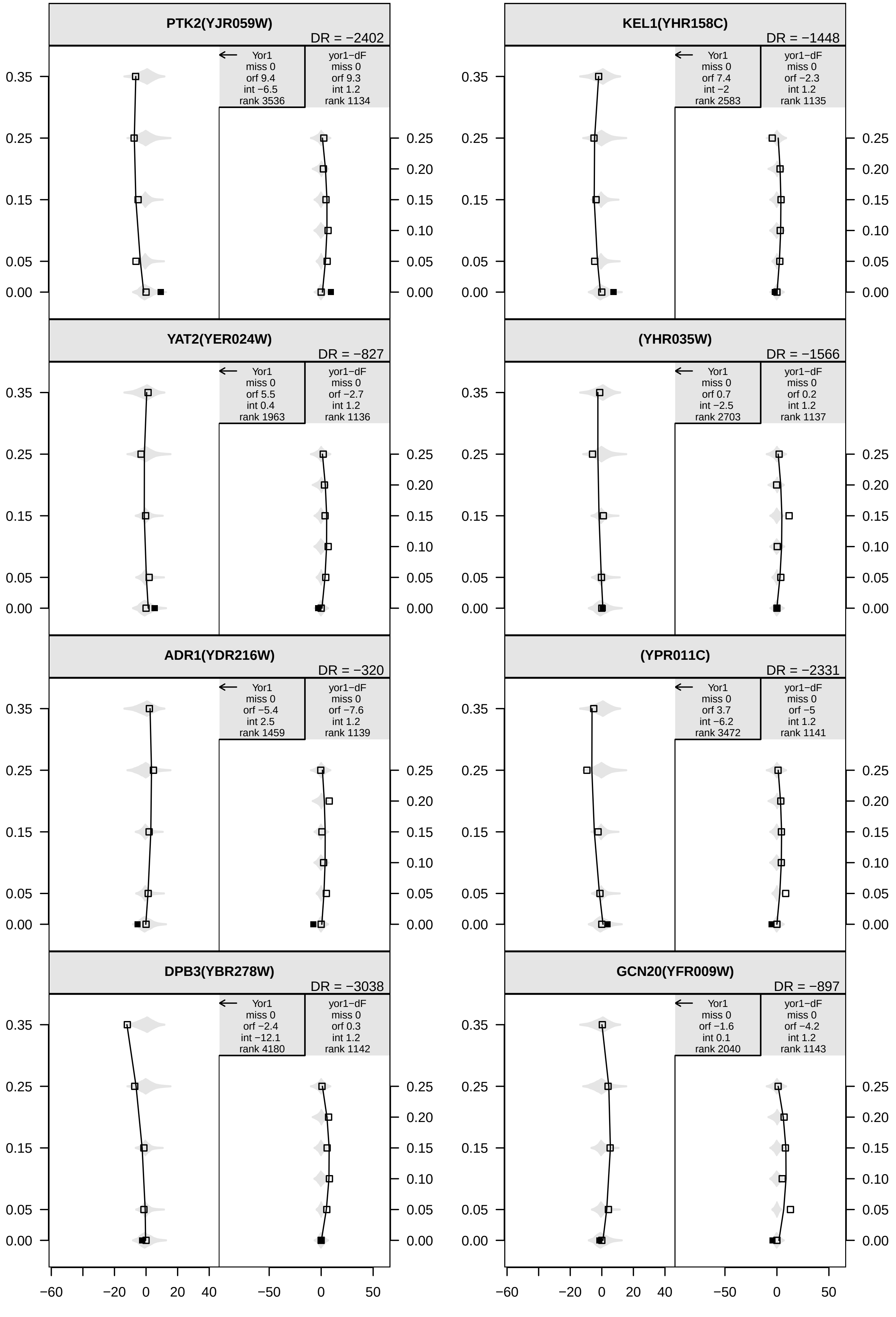

PTK2(YJR059W)
DR = −2402
Yor1 miss 0 orf 9.4 int −6.5 rank 3536
yor1−dF miss 0 orf 9.3 int 1.2 rank 1134
KEL1(YHR158C)
DR = −1448
Yor1 miss 0 orf 7.4 int −2 rank 2583
yor1−dF miss 0 orf −2.3 int 1.2 rank 1135
YAT2(YER024W)
DR = −827
Yor1 miss 0 orf 5.5 int 0.4 rank 1963
yor1−dF miss 0 orf −2.7 int 1.2 rank 1136
(YHR035W)
DR = −1566
Yor1 miss 0 orf 0.7 int −2.5 rank 2703
yor1−dF miss 0 orf 0.2 int 1.2 rank 1137
ADR1(YDR216W)
DR = −320
Yor1 miss 0 orf −5.4 int 2.5 rank 1459
yor1−dF miss 0 orf −7.6 int 1.2 rank 1139
(YPR011C)
DR = −2331
Yor1 miss 0 orf 3.7 int −6.2 rank 3472
yor1−dF miss 0 orf −5 int 1.2 rank 1141
DPB3(YBR278W)
DR = −3038
Yor1 miss 0 orf −2.4 int −12.1 rank 4180
yor1−dF miss 0 orf 0.3 int 1.2 rank 1142
GCN20(YFR009W)
DR = −897
Yor1 miss 0 orf −1.6 int 0.1 rank 2040
yor1−dF miss 0 orf −4.2 int 1.2 rank 1143
0.35
0.25
0.20
0.15
0.10
0.05
0.00
−60
−20
0
20
40
−50
0
50

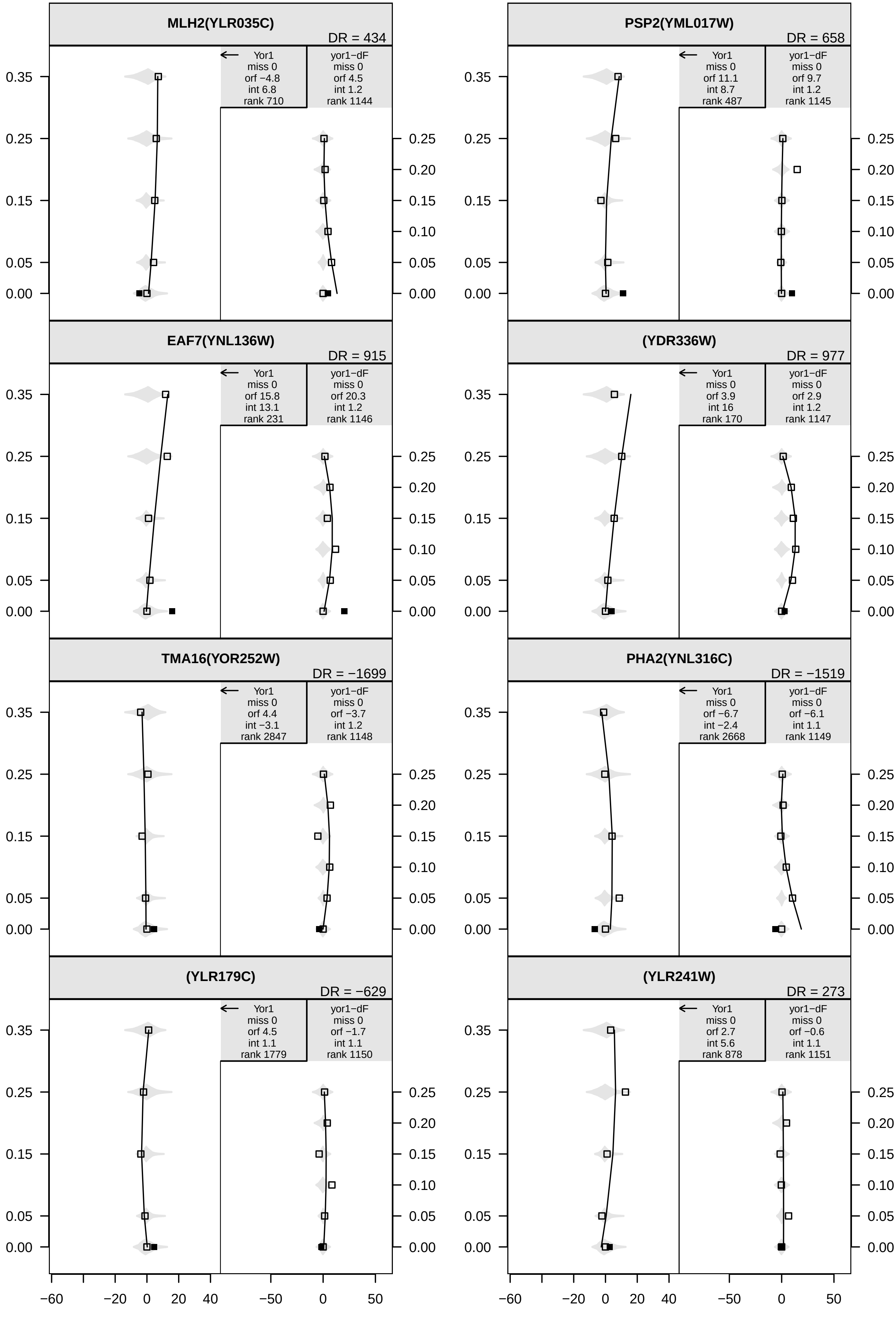

MLH2(YLR035C)
DR = 434
Yor1 miss 0 orf −4.8 int 6.8 rank 710
yor1−dF miss 0 orf 4.5 int 1.2 rank 1144
PSP2(YML017W)
DR = 658
Yor1 miss 0 orf 11.1 int 8.7 rank 487
yor1−dF miss 0 orf 9.7 int 1.2 rank 1145
EAF7(YNL136W)
DR = 915
Yor1 miss 0 orf 15.8 int 13.1 rank 231
yor1−dF miss 0 orf 20.3 int 1.2 rank 1146
(YDR336W)
DR = 977
Yor1 miss 0 orf 3.9 int 16 rank 170
yor1−dF miss 0 orf 2.9 int 1.2 rank 1147
TMA16(YOR252W)
DR = −1699
Yor1 miss 0 orf 4.4 int −3.1 rank 2847
yor1−dF miss 0 orf −3.7 int 1.2 rank 1148
PHA2(YNL316C)
DR = −1519
Yor1 miss 0 orf −6.7 int −2.4 rank 2668
yor1−dF miss 0 orf −6.1 int 1.1 rank 1149
(YLR179C)
DR = −629
Yor1 miss 0 orf 4.5 int 1.1 rank 1779
yor1−dF miss 0 orf −1.7 int 1.1 rank 1150
(YLR241W)
DR = 273
Yor1 miss 0 orf 2.7 int 5.6 rank 878
yor1−dF miss 0 orf −0.6 int 1.1 rank 1151
0.00 0.05 0.10 0.15 0.20 0.25 0.35
−60 −20 0 20 40 −50 0 50

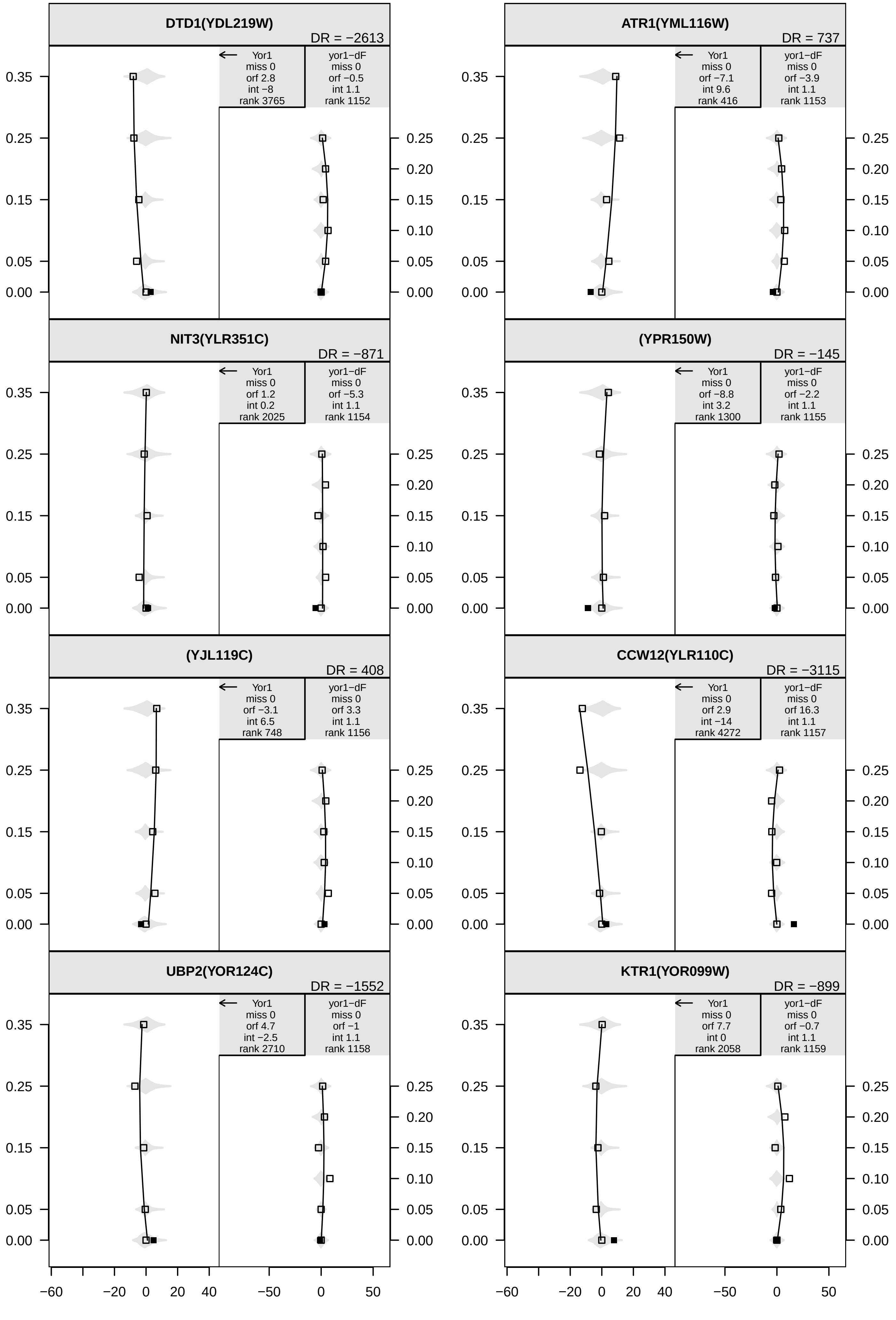

DTD1(YDL219W)
DR = −2613
Yor1
miss 0
orf 2.8
int −8
rank 3765
yor1−dF
miss 0
orf −0.5
int 1.1
rank 1152
ATR1(YML116W)
DR = 737
Yor1
miss 0
orf −7.1
int 9.6
rank 416
yor1−dF
miss 0
orf −3.9
int 1.1
rank 1153
NIT3(YLR351C)
DR = −871
Yor1
miss 0
orf 1.2
int 0.2
rank 2025
yor1−dF
miss 0
orf −5.3
int 1.1
rank 1154
(YPR150W)
DR = −145
Yor1
miss 0
orf −8.8
int 3.2
rank 1300
yor1−dF
miss 0
orf −2.2
int 1.1
rank 1155
(YJL119C)
DR = 408
Yor1
miss 0
orf −3.1
int 6.5
rank 748
yor1−dF
miss 0
orf 3.3
int 1.1
rank 1156
CCW12(YLR110C)
DR = −3115
Yor1
miss 0
orf 2.9
int −14
rank 4272
yor1−dF
miss 0
orf 16.3
int 1.1
rank 1157
UBP2(YOR124C)
DR = −1552
Yor1
miss 0
orf 4.7
int −2.5
rank 2710
yor1−dF
miss 0
orf −1
int 1.1
rank 1158
KTR1(YOR099W)
DR = −899
Yor1
miss 0
orf 7.7
int 0
rank 2058
yor1−dF
miss 0
orf −0.7
int 1.1
rank 1159
0.35
0.25
0.20
0.15
0.10
0.05
0.00
−60
−20
0
20
40
−50
0
50

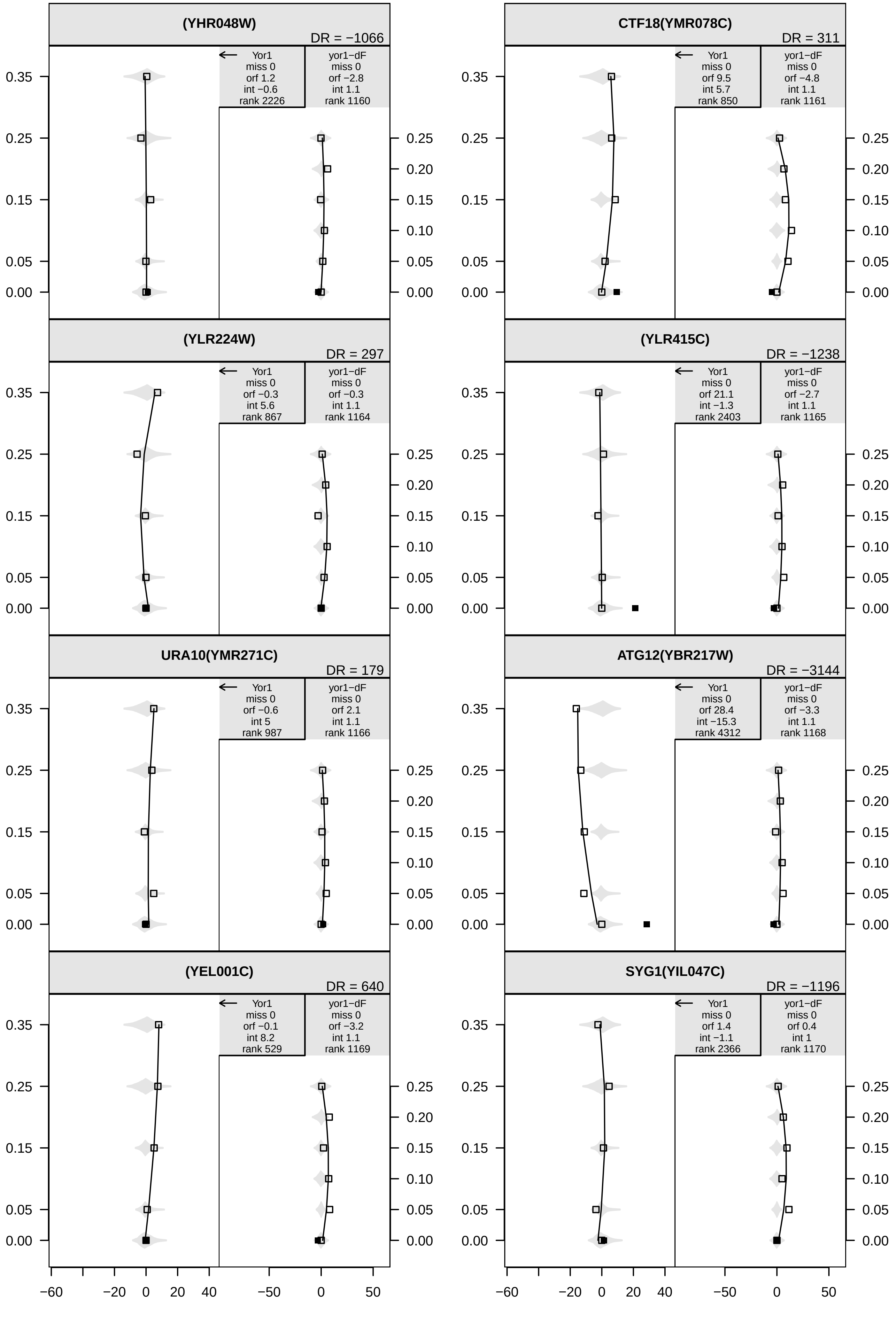

(YHR048W)
DR = −1066
Yor1
miss 0
orf 1.2
int −0.6
rank 2226
yor1−dF
miss 0
orf −2.8
int 1.1
rank 1160
CTF18(YMR078C)
DR = 311
Yor1
miss 0
orf 9.5
int 5.7
rank 850
yor1−dF
miss 0
orf −4.8
int 1.1
rank 1161
(YLR224W)
DR = 297
Yor1
miss 0
orf −0.3
int 5.6
rank 867
yor1−dF
miss 0
orf −0.3
int 1.1
rank 1164
(YLR415C)
DR = −1238
Yor1
miss 0
orf 21.1
int −1.3
rank 2403
yor1−dF
miss 0
orf −2.7
int 1.1
rank 1165
URA10(YMR271C)
DR = 179
Yor1
miss 0
orf −0.6
int 5
rank 987
yor1−dF
miss 0
orf 2.1
int 1.1
rank 1166
ATG12(YBR217W)
DR = −3144
Yor1
miss 0
orf 28.4
int −15.3
rank 4312
yor1−dF
miss 0
orf −3.3
int 1.1
rank 1168
(YEL001C)
DR = 640
Yor1
miss 0
orf −0.1
int 8.2
rank 529
yor1−dF
miss 0
orf −3.2
int 1.1
rank 1169
SYG1(YIL047C)
DR = −1196
Yor1
miss 0
orf 1.4
int −1.1
rank 2366
yor1−dF
miss 0
orf 0.4
int 1
rank 1170
0.35
0.25
0.20
0.15
0.10
0.05
0.00
−60
−20
0
20
40
−50
0
50

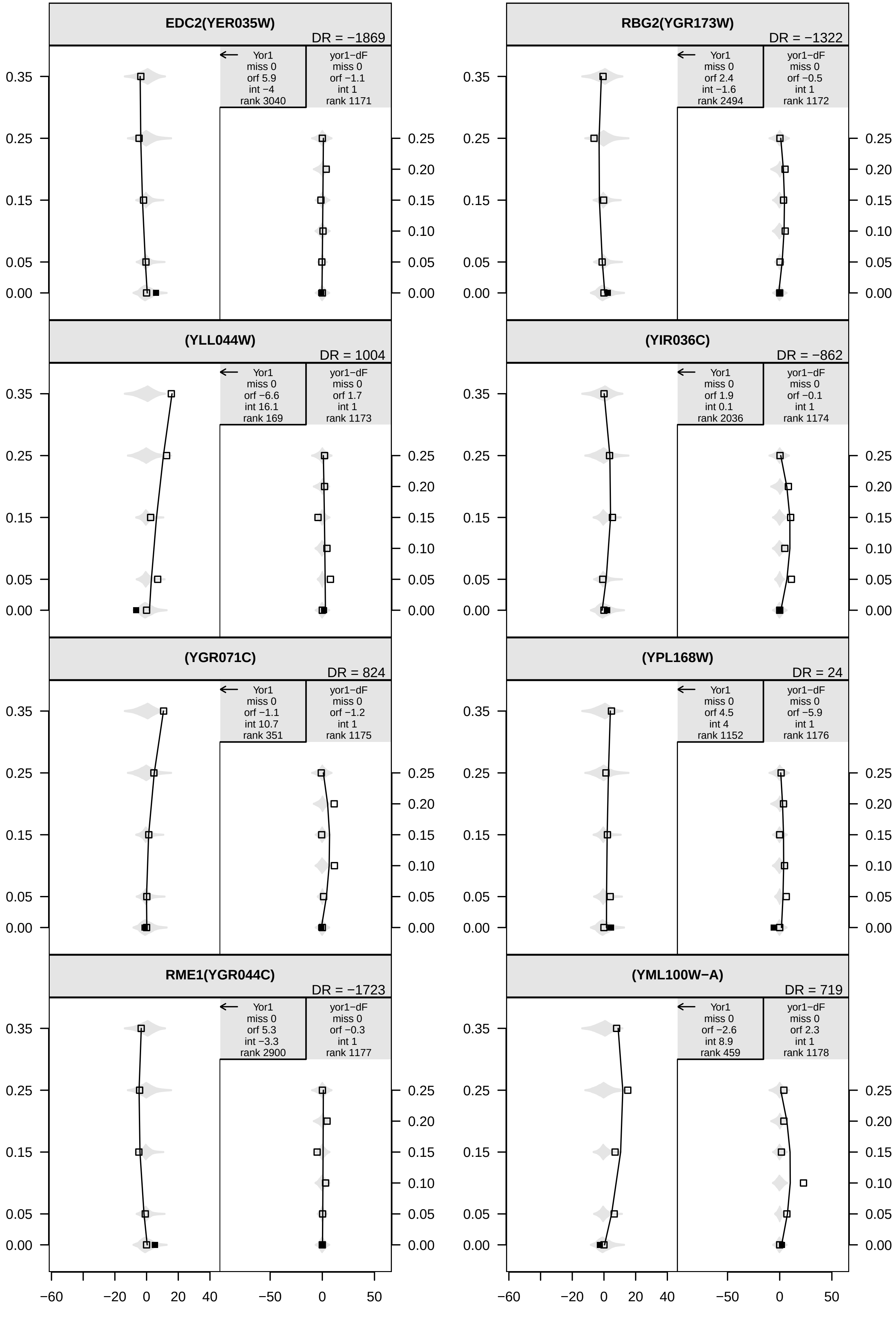
EDC2(YER035W)
DR = −1869
Yor1
miss 0
orf 5.9
int −4
rank 3040
yor1−dF
miss 0
orf −1.1
int 1
rank 1171
RBG2(YGR173W)
DR = −1322
Yor1
miss 0
orf 2.4
int −1.6
rank 2494
yor1−dF
miss 0
orf −0.5
int 1
rank 1172
(YLL044W)
DR = 1004
Yor1
miss 0
orf −6.6
int 16.1
rank 169
yor1−dF
miss 0
orf 1.7
int 1
rank 1173
(YIR036C)
DR = −862
Yor1
miss 0
orf 1.9
int 0.1
rank 2036
yor1−dF
miss 0
orf −0.1
int 1
rank 1174
(YGR071C)
DR = 824
Yor1
miss 0
orf −1.1
int 10.7
rank 351
yor1−dF
miss 0
orf −1.2
int 1
rank 1175
(YPL168W)
DR = 24
Yor1
miss 0
orf 4.5
int 4
rank 1152
yor1−dF
miss 0
orf −5.9
int 1
rank 1176
RME1(YGR044C)
DR = −1723
Yor1
miss 0
orf 5.3
int −3.3
rank 2900
yor1−dF
miss 0
orf −0.3
int 1
rank 1177
(YML100W−A)
DR = 719
Yor1
miss 0
orf −2.6
int 8.9
rank 459
yor1−dF
miss 0
orf 2.3
int 1
rank 1178
0.00
0.05
0.10
0.15
0.20
0.25
0.35
−60
−20
0
20
40
−50
0
50

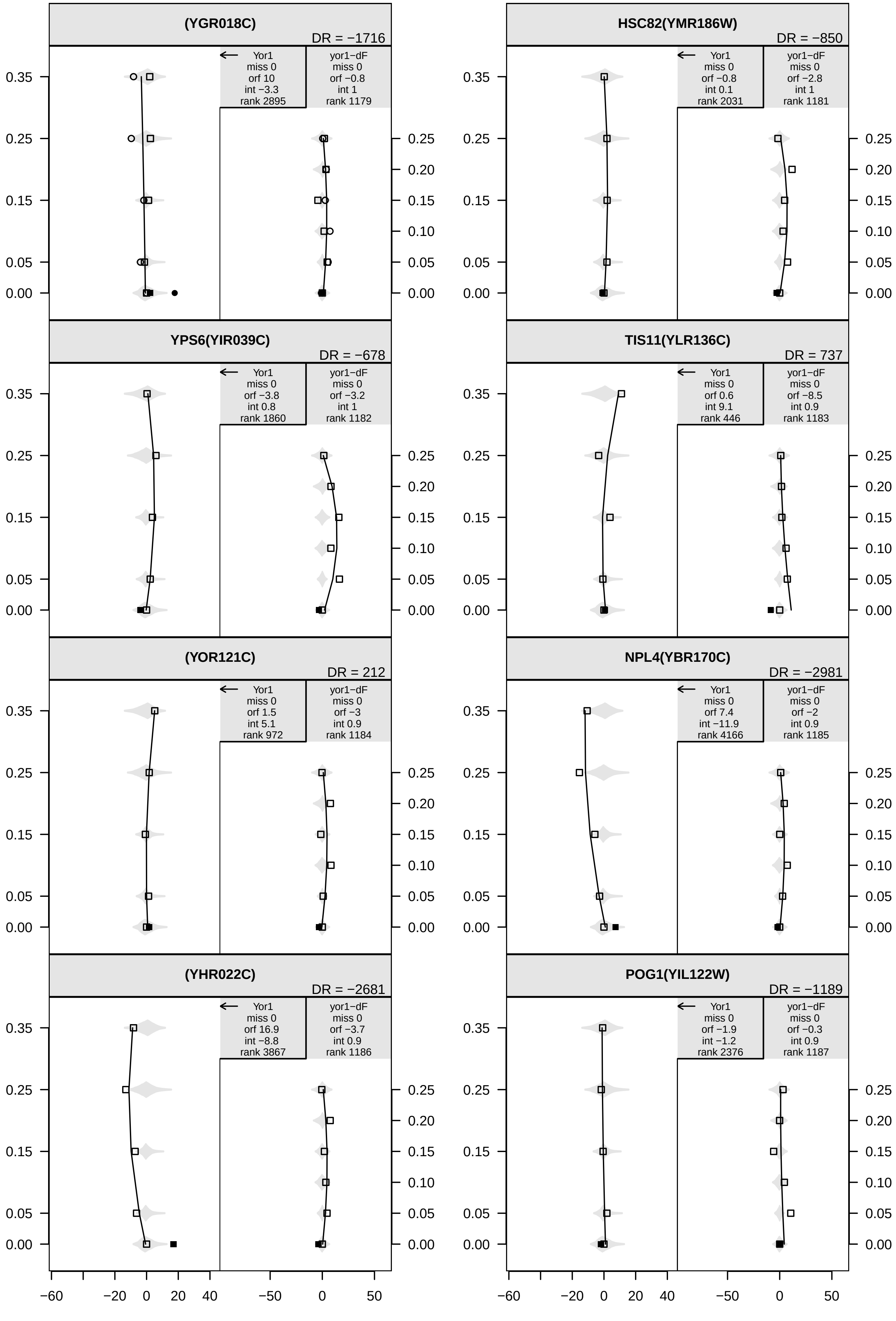
(YGR018C)
DR = −1716
Yor1 miss 0 orf 10 int −3.3 rank 2895
yor1−dF miss 0 orf −0.8 int 1 rank 1179
HSC82(YMR186W)
DR = −850
Yor1 miss 0 orf −0.8 int 0.1 rank 2031
yor1−dF miss 0 orf −2.8 int 1 rank 1181
YPS6(YIR039C)
DR = −678
Yor1 miss 0 orf −3.8 int 0.8 rank 1860
yor1−dF miss 0 orf −3.2 int 1 rank 1182
TIS11(YLR136C)
DR = 737
Yor1 miss 0 orf 0.6 int 9.1 rank 446
yor1−dF miss 0 orf −8.5 int 0.9 rank 1183
(YOR121C)
DR = 212
Yor1 miss 0 orf 1.5 int 5.1 rank 972
yor1−dF miss 0 orf −3 int 0.9 rank 1184
NPL4(YBR170C)
DR = −2981
Yor1 miss 0 orf 7.4 int −11.9 rank 4166
yor1−dF miss 0 orf −2 int 0.9 rank 1185
(YHR022C)
DR = −2681
Yor1 miss 0 orf 16.9 int −8.8 rank 3867
yor1−dF miss 0 orf −3.7 int 0.9 rank 1186
POG1(YIL122W)
DR = −1189
Yor1 miss 0 orf −1.9 int −1.2 rank 2376
yor1−dF miss 0 orf −0.3 int 0.9 rank 1187

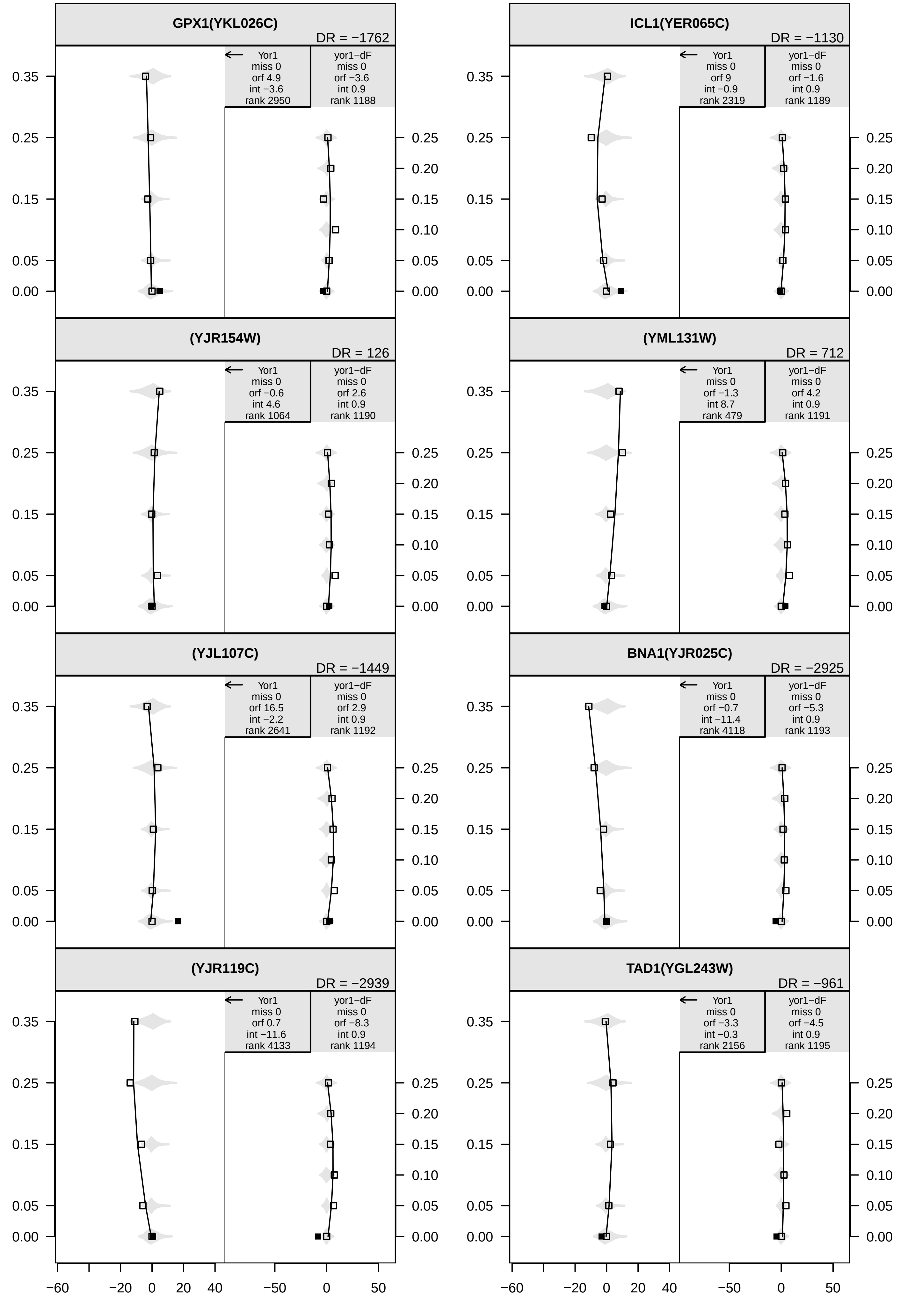
GPX1(YKL026C)
DR = −1762
Yor1 miss 0 orf 4.9 int −3.6 rank 2950
yor1−dF miss 0 orf −3.6 int 0.9 rank 1188
ICL1(YER065C)
DR = −1130
Yor1 miss 0 orf 9 int −0.9 rank 2319
yor1−dF miss 0 orf −1.6 int 0.9 rank 1189
(YJR154W)
DR = 126
Yor1 miss 0 orf −0.6 int 4.6 rank 1064
yor1−dF miss 0 orf 2.6 int 0.9 rank 1190
(YML131W)
DR = 712
Yor1 miss 0 orf −1.3 int 8.7 rank 479
yor1−dF miss 0 orf 4.2 int 0.9 rank 1191
(YJL107C)
DR = −1449
Yor1 miss 0 orf 16.5 int −2.2 rank 2641
yor1−dF miss 0 orf 2.9 int 0.9 rank 1192
BNA1(YJR025C)
DR = −2925
Yor1 miss 0 orf −0.7 int −11.4 rank 4118
yor1−dF miss 0 orf −5.3 int 0.9 rank 1193
(YJR119C)
DR = −2939
Yor1 miss 0 orf 0.7 int −11.6 rank 4133
yor1−dF miss 0 orf −8.3 int 0.9 rank 1194
TAD1(YGL243W)
DR = −961
Yor1 miss 0 orf −3.3 int −0.3 rank 2156
yor1−dF miss 0 orf −4.5 int 0.9 rank 1195
0.00 0.05 0.10 0.15 0.20 0.25 0.35
−60 −20 0 20 40 −50 0 50

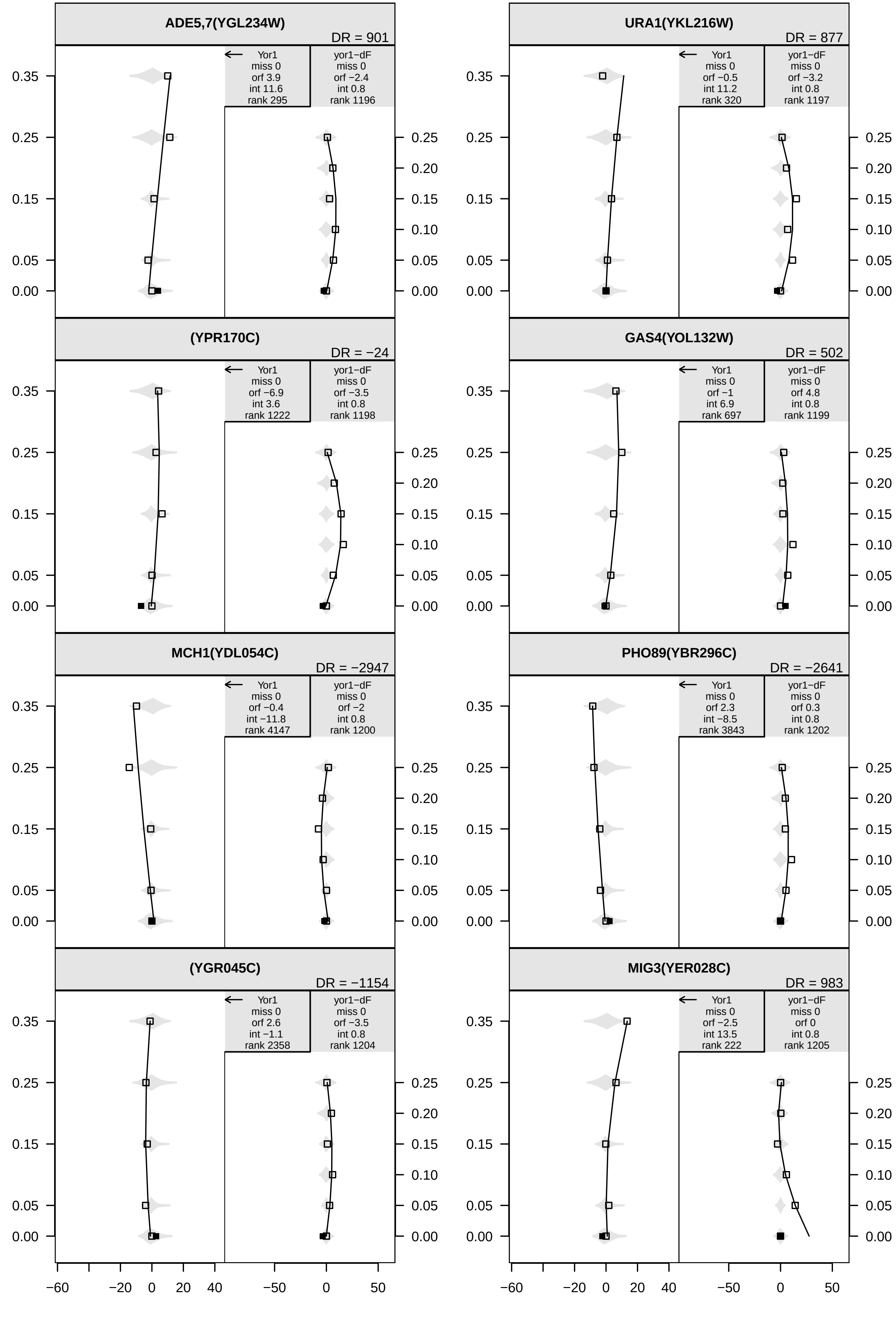

ADE5,7(YGL234W)
DR = 901
Yor1 miss 0 orf 3.9 int 11.6 rank 295
yor1−dF miss 0 orf −2.4 int 0.8 rank 1196
URA1(YKL216W)
DR = 877
Yor1 miss 0 orf −0.5 int 11.2 rank 320
yor1−dF miss 0 orf −3.2 int 0.8 rank 1197
(YPR170C)
DR = −24
Yor1 miss 0 orf −6.9 int 3.6 rank 1222
yor1−dF miss 0 orf −3.5 int 0.8 rank 1198
GAS4(YOL132W)
DR = 502
Yor1 miss 0 orf −1 int 6.9 rank 697
yor1−dF miss 0 orf 4.8 int 0.8 rank 1199
MCH1(YDL054C)
DR = −2947
Yor1 miss 0 orf −0.4 int −11.8 rank 4147
yor1−dF miss 0 orf −2 int 0.8 rank 1200
PHO89(YBR296C)
DR = −2641
Yor1 miss 0 orf 2.3 int −8.5 rank 3843
yor1−dF miss 0 orf 0.3 int 0.8 rank 1202
(YGR045C)
DR = −1154
Yor1 miss 0 orf 2.6 int −1.1 rank 2358
yor1−dF miss 0 orf −3.5 int 0.8 rank 1204
MIG3(YER028C)
DR = 983
Yor1 miss 0 orf −2.5 int 13.5 rank 222
yor1−dF miss 0 orf 0 int 0.8 rank 1205
0.35 0.25 0.20 0.15 0.10 0.05 0.00
−60 −20 0 20 40 −50 0 50

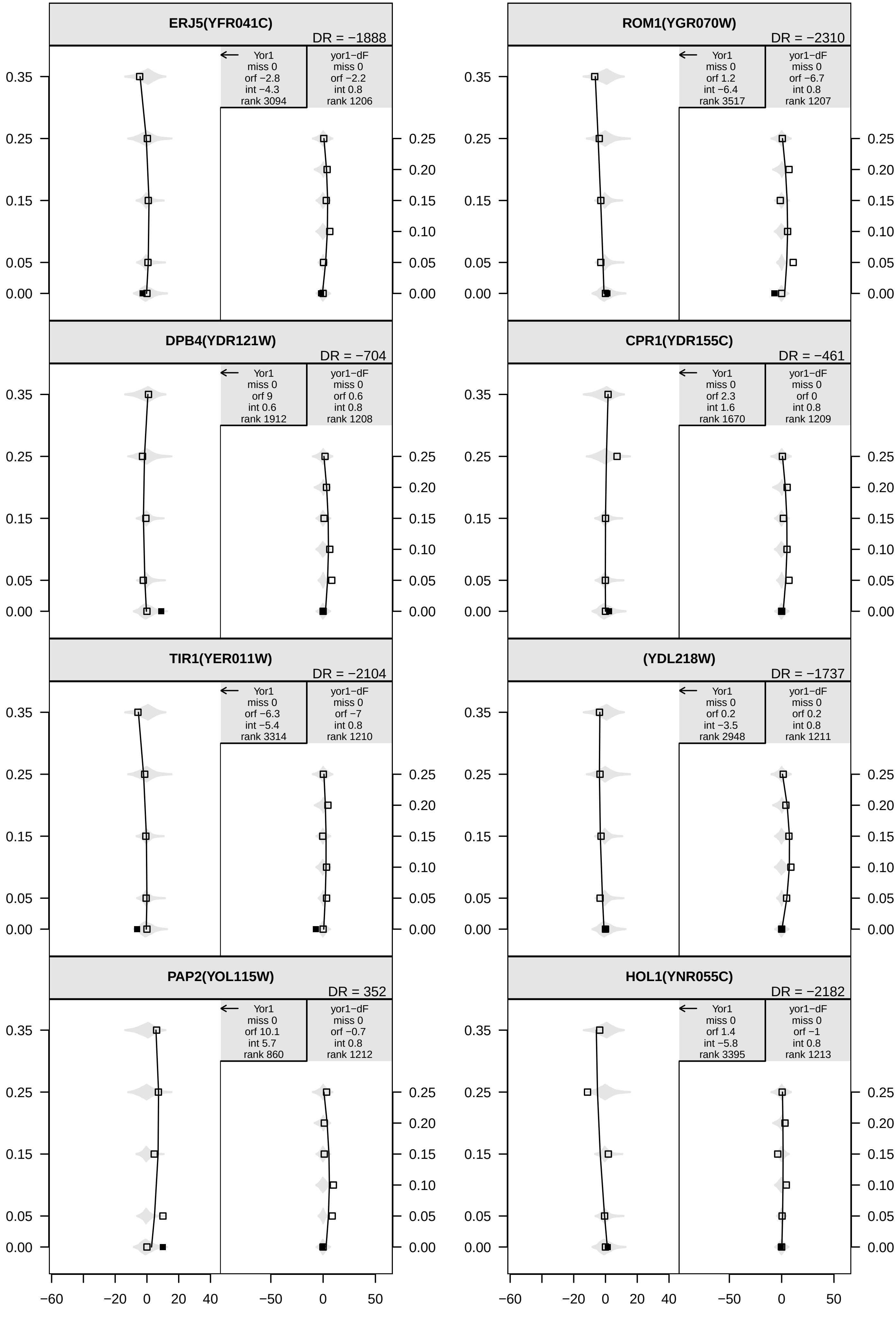
ERJ5(YFR041C)
DR = −1888
Yor1 miss 0 orf −2.8 int −4.3 rank 3094
yor1−dF miss 0 orf −2.2 int 0.8 rank 1206
ROM1(YGR070W)
DR = −2310
Yor1 miss 0 orf 1.2 int −6.4 rank 3517
yor1−dF miss 0 orf −6.7 int 0.8 rank 1207
DPB4(YDR121W)
DR = −704
Yor1 miss 0 orf 9 int 0.6 rank 1912
yor1−dF miss 0 orf 0.6 int 0.8 rank 1208
CPR1(YDR155C)
DR = −461
Yor1 miss 0 orf 2.3 int 1.6 rank 1670
yor1−dF miss 0 orf 0 int 0.8 rank 1209
TIR1(YER011W)
DR = −2104
Yor1 miss 0 orf −6.3 int −5.4 rank 3314
yor1−dF miss 0 orf −7 int 0.8 rank 1210
(YDL218W)
DR = −1737
Yor1 miss 0 orf 0.2 int −3.5 rank 2948
yor1−dF miss 0 orf 0.2 int 0.8 rank 1211
PAP2(YOL115W)
DR = 352
Yor1 miss 0 orf 10.1 int 5.7 rank 860
yor1−dF miss 0 orf −0.7 int 0.8 rank 1212
HOL1(YNR055C)
DR = −2182
Yor1 miss 0 orf 1.4 int −5.8 rank 3395
yor1−dF miss 0 orf −1 int 0.8 rank 1213
0.35 0.25 0.15 0.05 0.00
0.25 0.20 0.15 0.10 0.05 0.00
−60 −20 0 20 40 −50 0 50

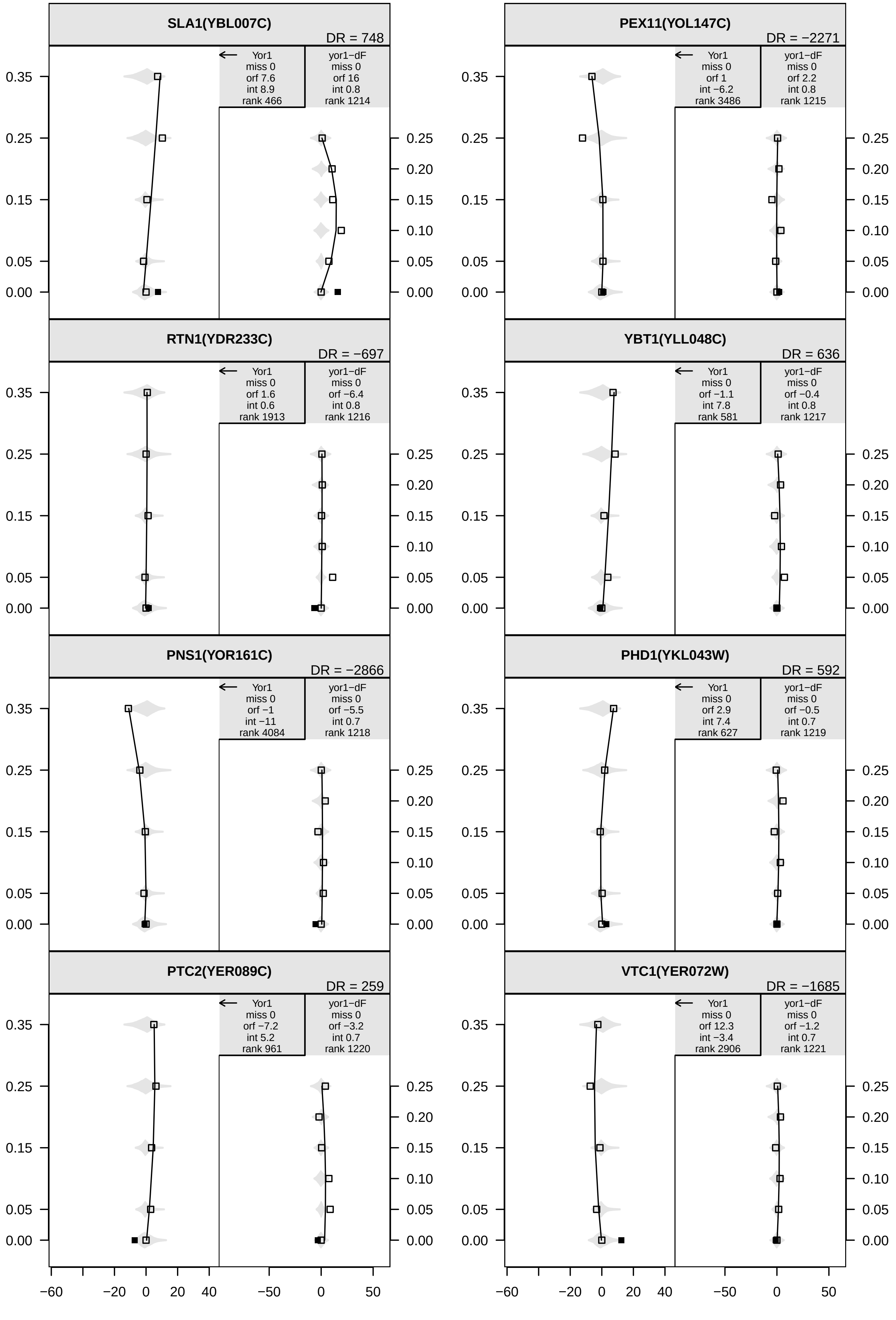
SLA1(YBL007C)
DR = 748
Yor1 miss 0 orf 7.6 int 8.9 rank 466
yor1−dF miss 0 orf 16 int 0.8 rank 1214
PEX11(YOL147C)
DR = −2271
Yor1 miss 0 orf 1 int −6.2 rank 3486
yor1−dF miss 0 orf 2.2 int 0.8 rank 1215
RTN1(YDR233C)
DR = −697
Yor1 miss 0 orf 1.6 int 0.6 rank 1913
yor1−dF miss 0 orf −6.4 int 0.8 rank 1216
YBT1(YLL048C)
DR = 636
Yor1 miss 0 orf −1.1 int 7.8 rank 581
yor1−dF miss 0 orf −0.4 int 0.8 rank 1217
PNS1(YOR161C)
DR = −2866
Yor1 miss 0 orf −1 int −11 rank 4084
yor1−dF miss 0 orf −5.5 int 0.7 rank 1218
PHD1(YKL043W)
DR = 592
Yor1 miss 0 orf 2.9 int 7.4 rank 627
yor1−dF miss 0 orf −0.5 int 0.7 rank 1219
PTC2(YER089C)
DR = 259
Yor1 miss 0 orf −7.2 int 5.2 rank 961
yor1−dF miss 0 orf −3.2 int 0.7 rank 1220
VTC1(YER072W)
DR = −1685
Yor1 miss 0 orf 12.3 int −3.4 rank 2906
yor1−dF miss 0 orf −1.2 int 0.7 rank 1221
0.35 0.25 0.15 0.05 0.00
0.25 0.20 0.15 0.10 0.05 0.00
−60 −20 0 20 40 −50 0 50

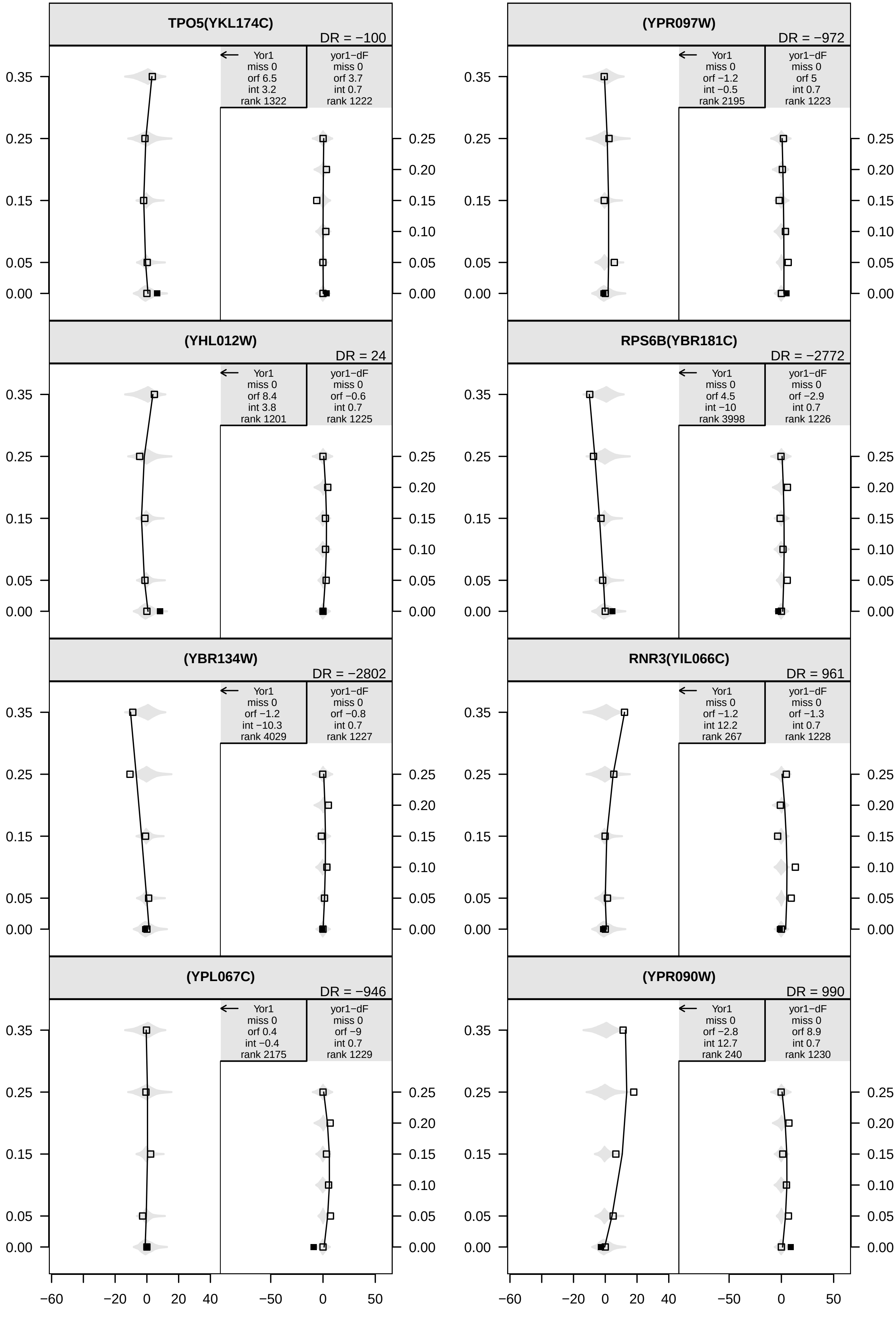
TPO5(YKL174C)
DR = −100
Yor1
miss 0
orf 6.5
int 3.2
rank 1322
yor1−dF
miss 0
orf 3.7
int 0.7
rank 1222
(YPR097W)
DR = −972
Yor1
miss 0
orf −1.2
int −0.5
rank 2195
yor1−dF
miss 0
orf 5
int 0.7
rank 1223
(YHL012W)
DR = 24
Yor1
miss 0
orf 8.4
int 3.8
rank 1201
yor1−dF
miss 0
orf −0.6
int 0.7
rank 1225
RPS6B(YBR181C)
DR = −2772
Yor1
miss 0
orf 4.5
int −10
rank 3998
yor1−dF
miss 0
orf −2.9
int 0.7
rank 1226
(YBR134W)
DR = −2802
Yor1
miss 0
orf −1.2
int −10.3
rank 4029
yor1−dF
miss 0
orf −0.8
int 0.7
rank 1227
RNR3(YIL066C)
DR = 961
Yor1
miss 0
orf −1.2
int 12.2
rank 267
yor1−dF
miss 0
orf −1.3
int 0.7
rank 1228
(YPL067C)
DR = −946
Yor1
miss 0
orf 0.4
int −0.4
rank 2175
yor1−dF
miss 0
orf −9
int 0.7
rank 1229
(YPR090W)
DR = 990
Yor1
miss 0
orf −2.8
int 12.7
rank 240
yor1−dF
miss 0
orf 8.9
int 0.7
rank 1230
0.35
0.25
0.20
0.15
0.10
0.05
0.00
−60
−20
0
20
40
−50
0
50

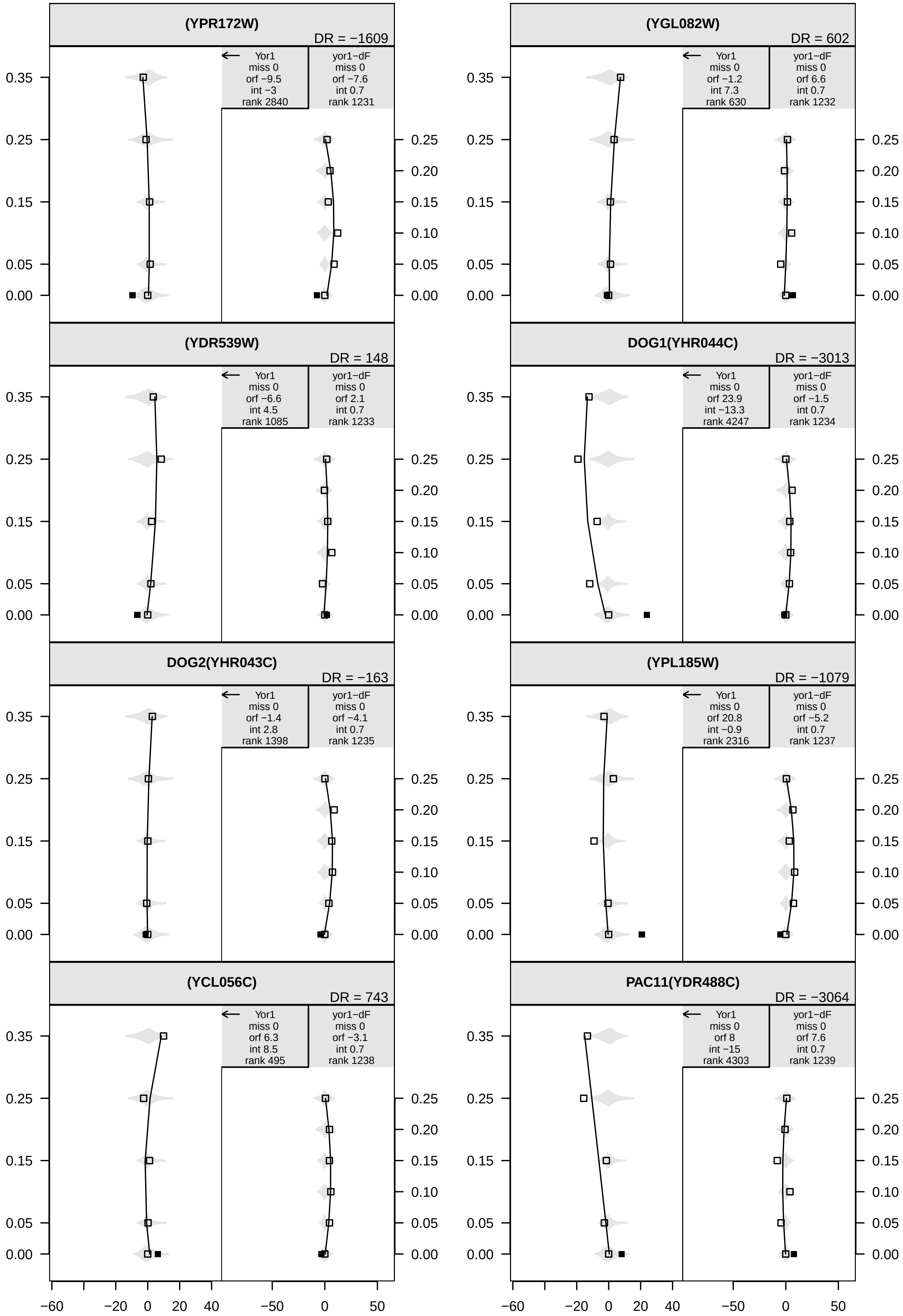
(YPR172W)
DR = −1609
Yor1 miss 0 orf −9.5 int −3 rank 2840
yor1−dF miss 0 orf −7.6 int 0.7 rank 1231
(YGL082W)
DR = 602
Yor1 miss 0 orf −1.2 int 7.3 rank 630
yor1−dF miss 0 orf 6.6 int 0.7 rank 1232
(YDR539W)
DR = 148
Yor1 miss 0 orf −6.6 int 4.5 rank 1085
yor1−dF miss 0 orf 2.1 int 0.7 rank 1233
DOG1(YHR044C)
DR = −3013
Yor1 miss 0 orf 23.9 int −13.3 rank 4247
yor1−dF miss 0 orf −1.5 int 0.7 rank 1234
DOG2(YHR043C)
DR = −163
Yor1 miss 0 orf −1.4 int 2.8 rank 1398
yor1−dF miss 0 orf −4.1 int 0.7 rank 1235
(YPL185W)
DR = −1079
Yor1 miss 0 orf 20.8 int −0.9 rank 2316
yor1−dF miss 0 orf −5.2 int 0.7 rank 1237
(YCL056C)
DR = 743
Yor1 miss 0 orf 6.3 int 8.5 rank 495
yor1−dF miss 0 orf −3.1 int 0.7 rank 1238
PAC11(YDR488C)
DR = −3064
Yor1 miss 0 orf 8 int −15 rank 4303
yor1−dF miss 0 orf 7.6 int 0.7 rank 1239

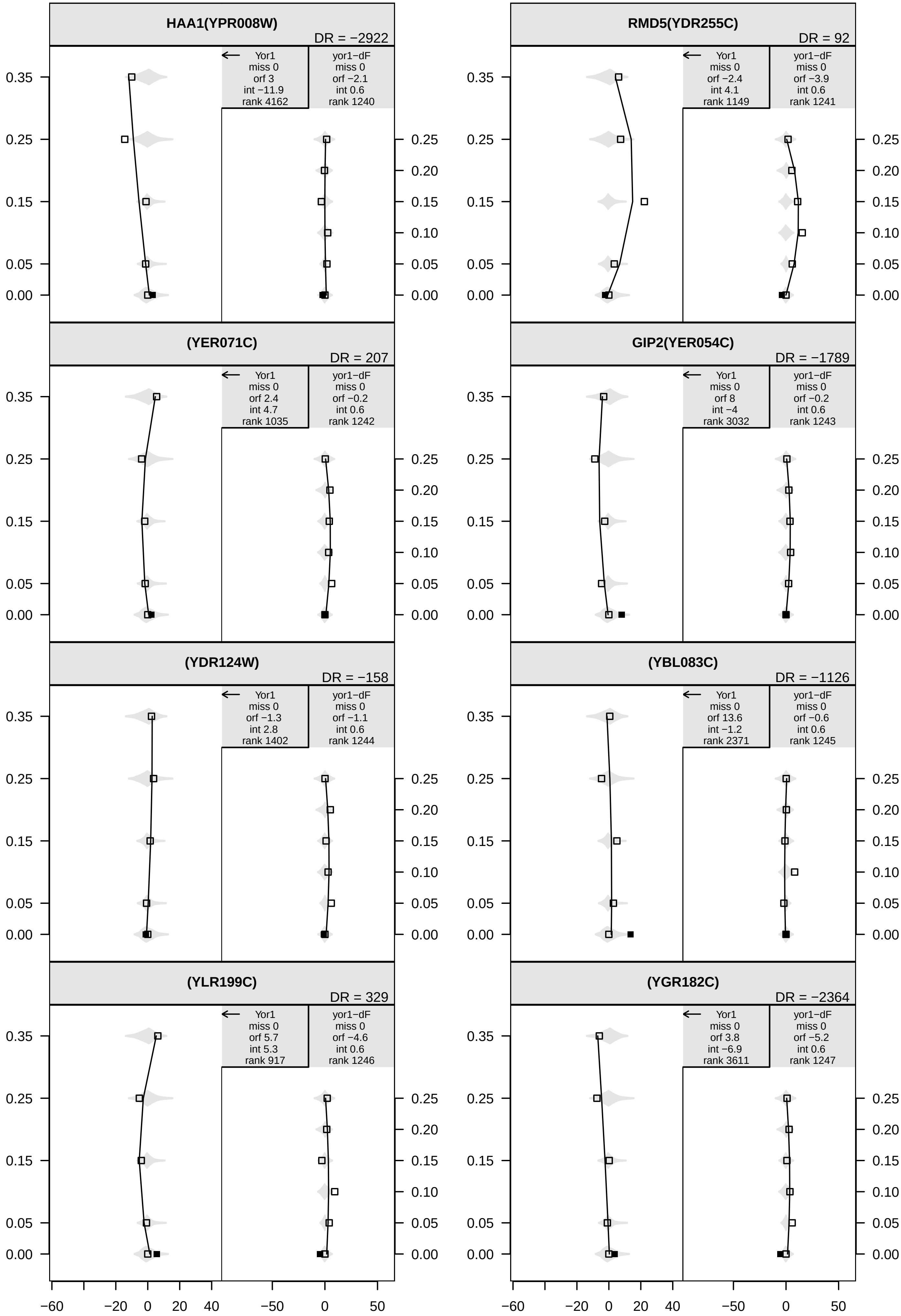
HAA1(YPR008W)
DR = −2922
Yor1 miss 0 orf 3 int −11.9 rank 4162
yor1−dF miss 0 orf −2.1 int 0.6 rank 1240
RMD5(YDR255C)
DR = 92
Yor1 miss 0 orf −2.4 int 4.1 rank 1149
yor1−dF miss 0 orf −3.9 int 0.6 rank 1241
(YER071C)
DR = 207
Yor1 miss 0 orf 2.4 int 4.7 rank 1035
yor1−dF miss 0 orf −0.2 int 0.6 rank 1242
GIP2(YER054C)
DR = −1789
Yor1 miss 0 orf 8 int −4 rank 3032
yor1−dF miss 0 orf −0.2 int 0.6 rank 1243
(YDR124W)
DR = −158
Yor1 miss 0 orf −1.3 int 2.8 rank 1402
yor1−dF miss 0 orf −1.1 int 0.6 rank 1244
(YBL083C)
DR = −1126
Yor1 miss 0 orf 13.6 int −1.2 rank 2371
yor1−dF miss 0 orf −0.6 int 0.6 rank 1245
(YLR199C)
DR = 329
Yor1 miss 0 orf 5.7 int 5.3 rank 917
yor1−dF miss 0 orf −4.6 int 0.6 rank 1246
(YGR182C)
DR = −2364
Yor1 miss 0 orf 3.8 int −6.9 rank 3611
yor1−dF miss 0 orf −5.2 int 0.6 rank 1247
0.35 0.25 0.15 0.05 0.00
0.25 0.20 0.15 0.10 0.05 0.00
−60 −20 0 20 40 −50 0 50

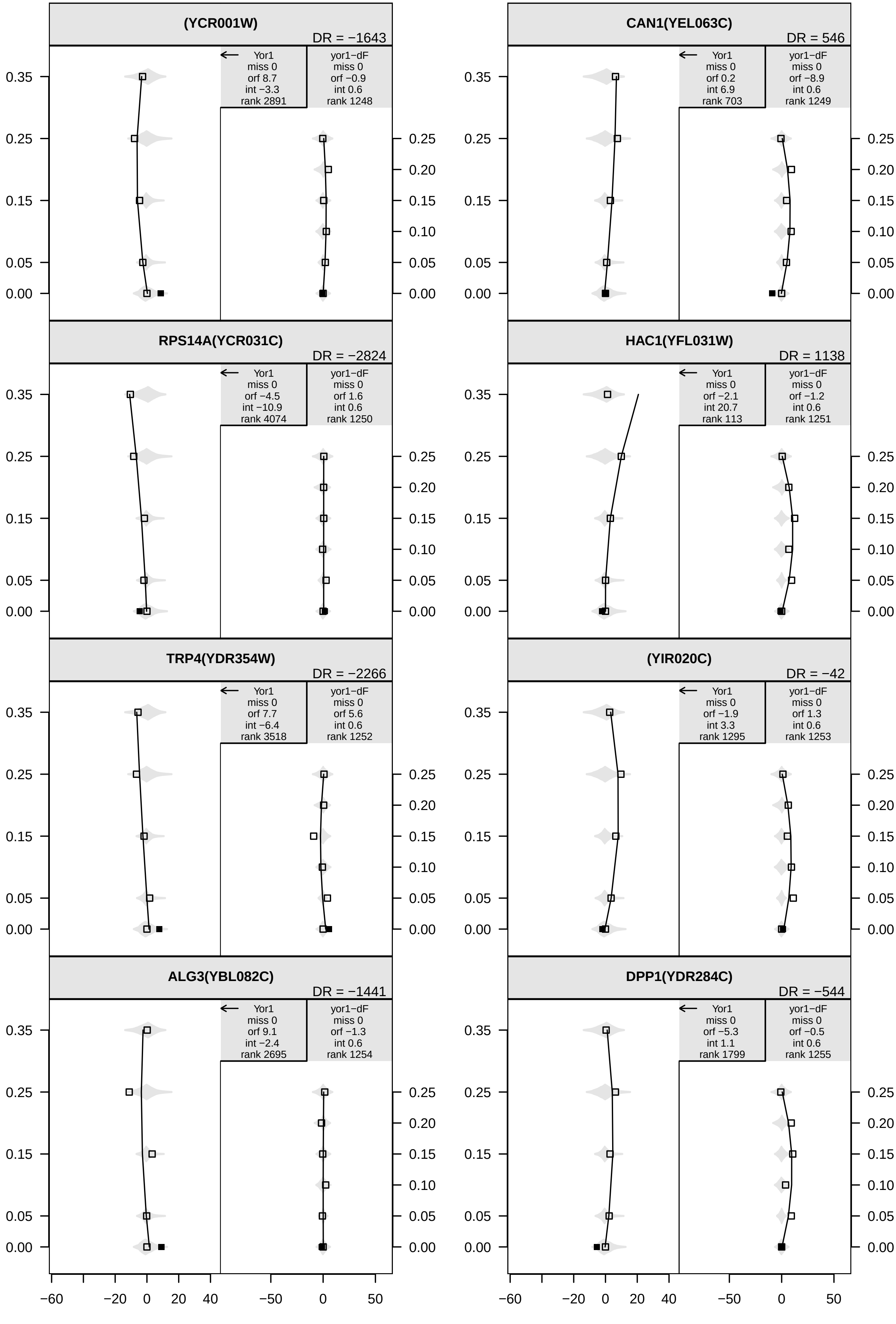

(YCR001W)
DR = −1643
Yor1 miss 0 orf 8.7 int −3.3 rank 2891
yor1−dF miss 0 orf −0.9 int 0.6 rank 1248
CAN1(YEL063C)
DR = 546
Yor1 miss 0 orf 0.2 int 6.9 rank 703
yor1−dF miss 0 orf −8.9 int 0.6 rank 1249
RPS14A(YCR031C)
DR = −2824
Yor1 miss 0 orf −4.5 int −10.9 rank 4074
yor1−dF miss 0 orf 1.6 int 0.6 rank 1250
HAC1(YFL031W)
DR = 1138
Yor1 miss 0 orf −2.1 int 20.7 rank 113
yor1−dF miss 0 orf −1.2 int 0.6 rank 1251
TRP4(YDR354W)
DR = −2266
Yor1 miss 0 orf 7.7 int −6.4 rank 3518
yor1−dF miss 0 orf 5.6 int 0.6 rank 1252
(YIR020C)
DR = −42
Yor1 miss 0 orf −1.9 int 3.3 rank 1295
yor1−dF miss 0 orf 1.3 int 0.6 rank 1253
ALG3(YBL082C)
DR = −1441
Yor1 miss 0 orf 9.1 int −2.4 rank 2695
yor1−dF miss 0 orf −1.3 int 0.6 rank 1254
DPP1(YDR284C)
DR = −544
Yor1 miss 0 orf −5.3 int 1.1 rank 1799
yor1−dF miss 0 orf −0.5 int 0.6 rank 1255
0.35 0.25 0.15 0.05 0.00
0.25 0.20 0.15 0.10 0.05 0.00
−60 −20 0 20 40 −50 0 50

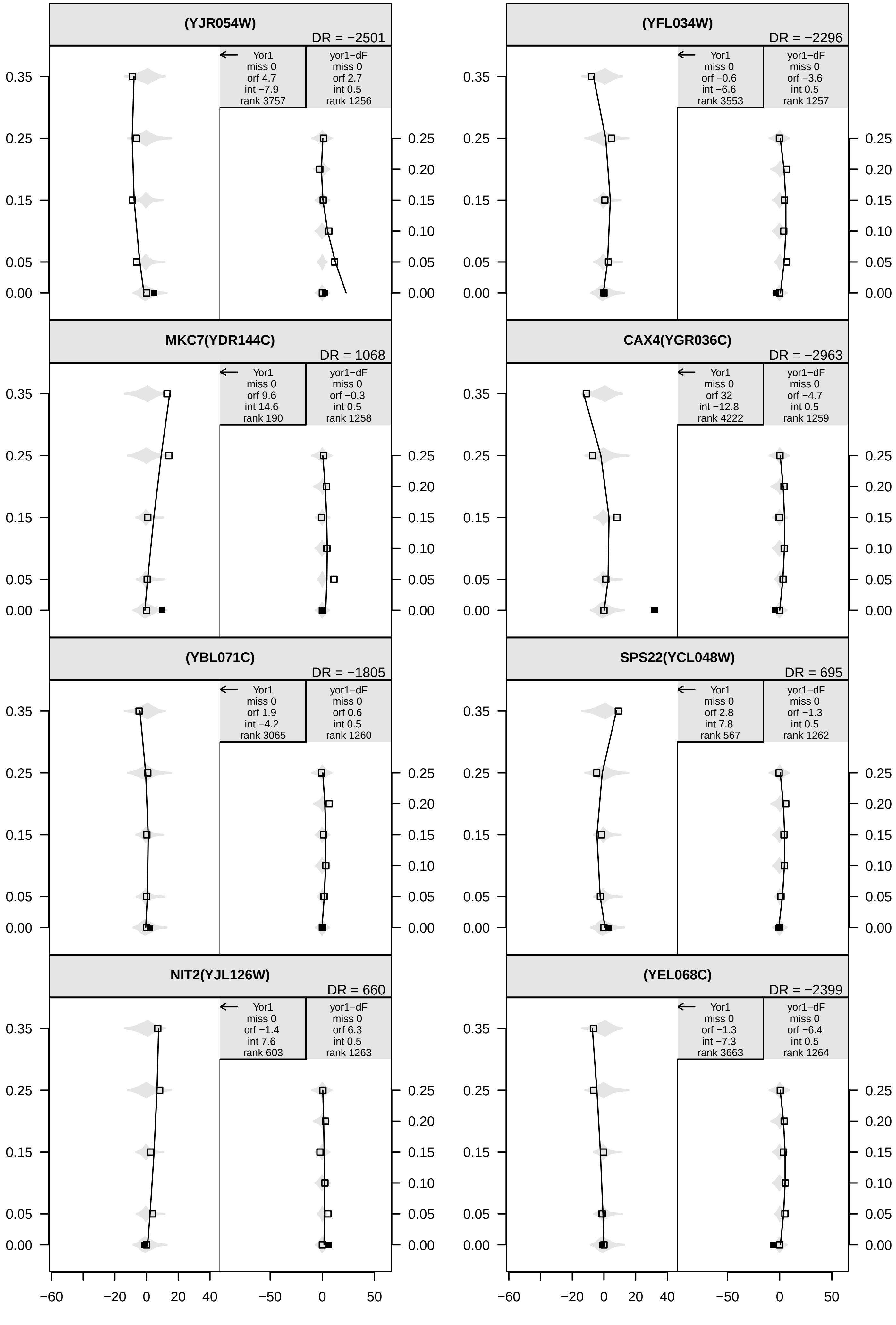

(YJR054W)
DR = −2501
Yor1 miss 0 orf 4.7 int −7.9 rank 3757
yor1−dF miss 0 orf 2.7 int 0.5 rank 1256
(YFL034W)
DR = −2296
Yor1 miss 0 orf −0.6 int −6.6 rank 3553
yor1−dF miss 0 orf −3.6 int 0.5 rank 1257
MKC7(YDR144C)
DR = 1068
Yor1 miss 0 orf 9.6 int 14.6 rank 190
yor1−dF miss 0 orf −0.3 int 0.5 rank 1258
CAX4(YGR036C)
DR = −2963
Yor1 miss 0 orf 32 int −12.8 rank 4222
yor1−dF miss 0 orf −4.7 int 0.5 rank 1259
(YBL071C)
DR = −1805
Yor1 miss 0 orf 1.9 int −4.2 rank 3065
yor1−dF miss 0 orf 0.6 int 0.5 rank 1260
SPS22(YCL048W)
DR = 695
Yor1 miss 0 orf 2.8 int 7.8 rank 567
yor1−dF miss 0 orf −1.3 int 0.5 rank 1262
NIT2(YJL126W)
DR = 660
Yor1 miss 0 orf −1.4 int 7.6 rank 603
yor1−dF miss 0 orf 6.3 int 0.5 rank 1263
(YEL068C)
DR = −2399
Yor1 miss 0 orf −1.3 int −7.3 rank 3663
yor1−dF miss 0 orf −6.4 int 0.5 rank 1264
0.35 0.25 0.15 0.05 0.00
0.25 0.20 0.15 0.10 0.05 0.00
−60 −20 0 20 40 −50 0 50

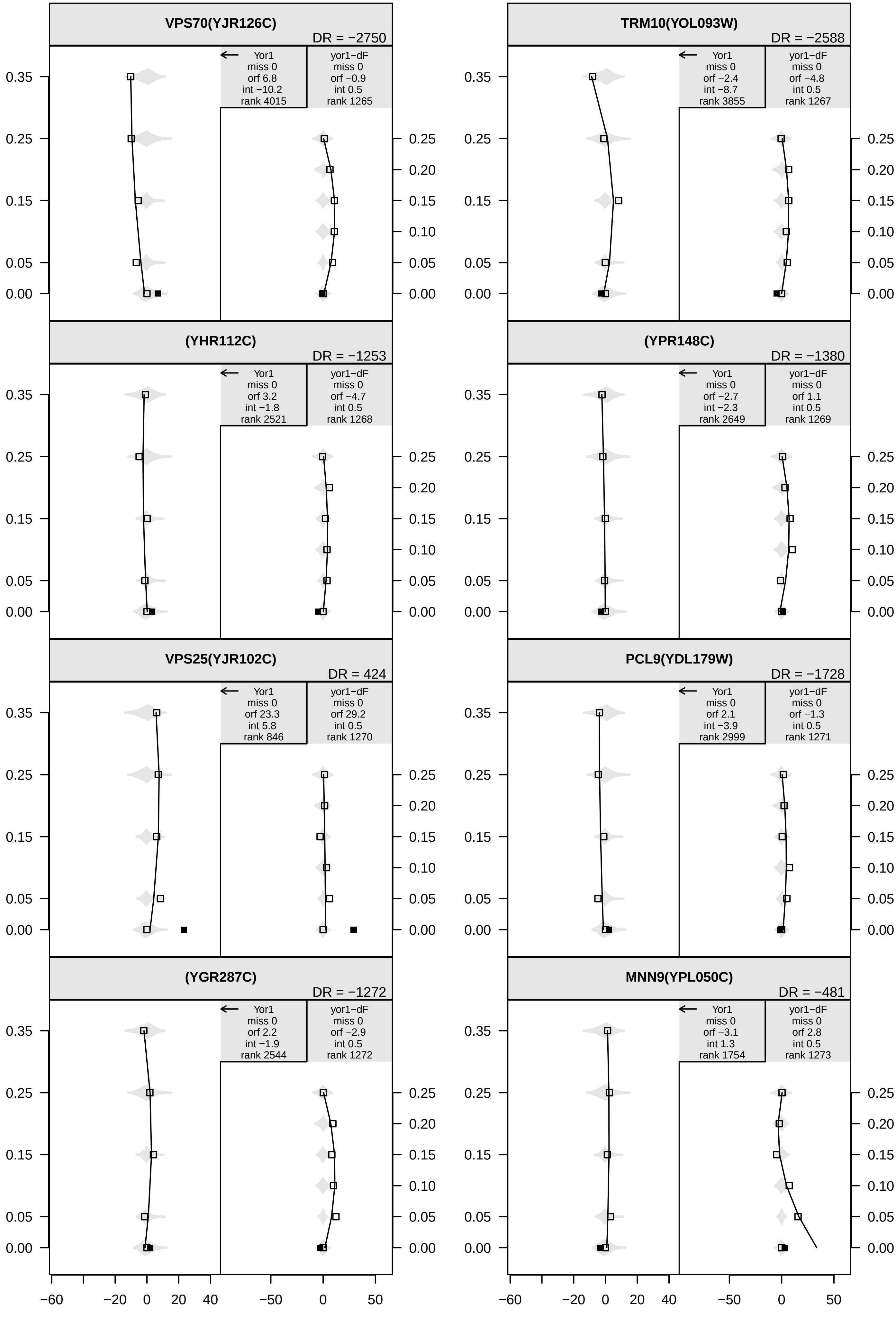
VPS70(YJR126C)
DR = −2750
Yor1 miss 0 orf 6.8 int −10.2 rank 4015
yor1−dF miss 0 orf −0.9 int 0.5 rank 1265
TRM10(YOL093W)
DR = −2588
Yor1 miss 0 orf −2.4 int −8.7 rank 3855
yor1−dF miss 0 orf −4.8 int 0.5 rank 1267
(YHR112C)
DR = −1253
Yor1 miss 0 orf 3.2 int −1.8 rank 2521
yor1−dF miss 0 orf −4.7 int 0.5 rank 1268
(YPR148C)
DR = −1380
Yor1 miss 0 orf −2.7 int −2.3 rank 2649
yor1−dF miss 0 orf 1.1 int 0.5 rank 1269
VPS25(YJR102C)
DR = 424
Yor1 miss 0 orf 23.3 int 5.8 rank 846
yor1−dF miss 0 orf 29.2 int 0.5 rank 1270
PCL9(YDL179W)
DR = −1728
Yor1 miss 0 orf 2.1 int −3.9 rank 2999
yor1−dF miss 0 orf −1.3 int 0.5 rank 1271
(YGR287C)
DR = −1272
Yor1 miss 0 orf 2.2 int −1.9 rank 2544
yor1−dF miss 0 orf −2.9 int 0.5 rank 1272
MNN9(YPL050C)
DR = −481
Yor1 miss 0 orf −3.1 int 1.3 rank 1754
yor1−dF miss 0 orf 2.8 int 0.5 rank 1273

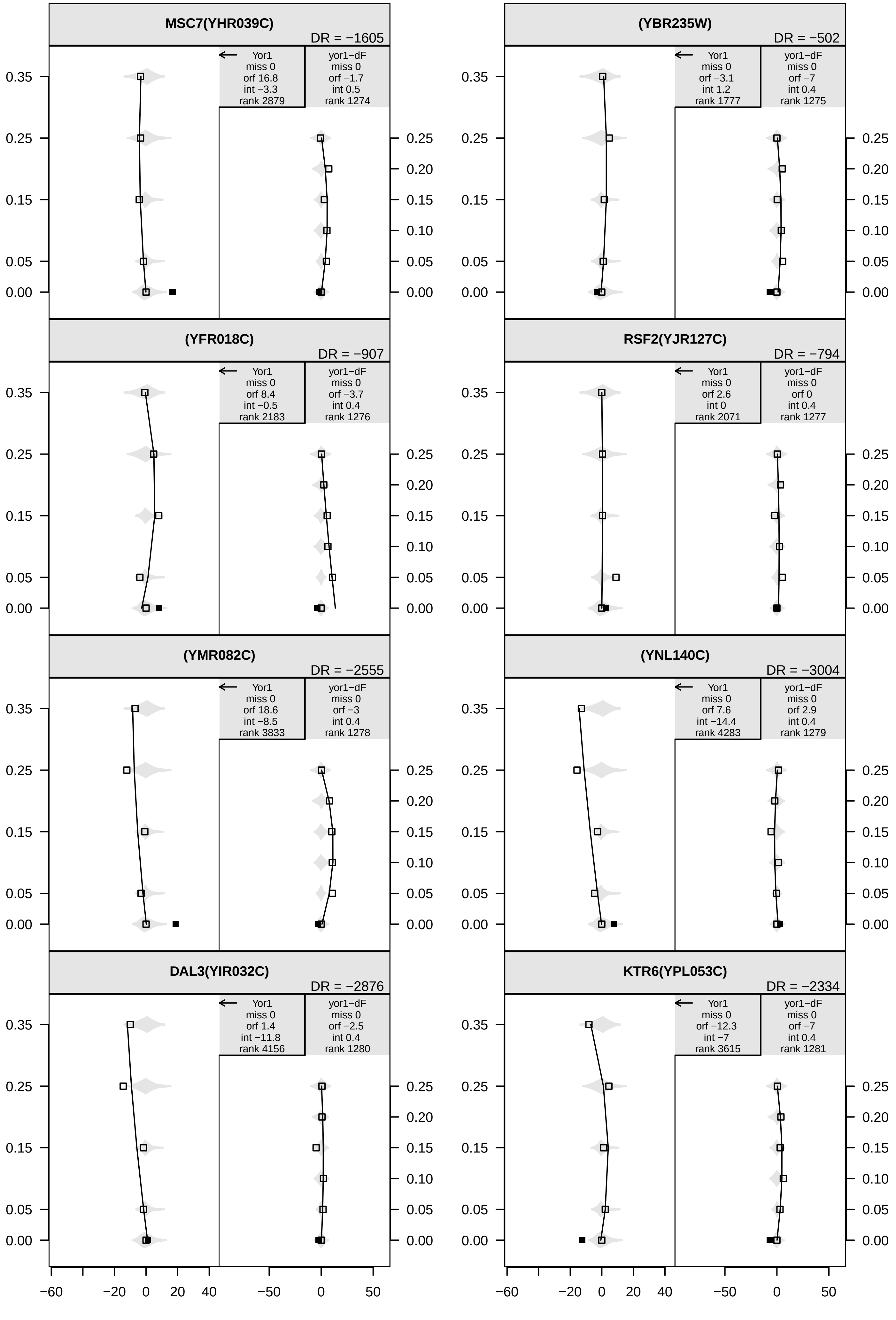

MSC7(YHR039C)
DR = −1605
Yor1 miss 0 orf 16.8 int −3.3 rank 2879
yor1−dF miss 0 orf −1.7 int 0.5 rank 1274
(YBR235W)
DR = −502
Yor1 miss 0 orf −3.1 int 1.2 rank 1777
yor1−dF miss 0 orf −7 int 0.4 rank 1275
(YFR018C)
DR = −907
Yor1 miss 0 orf 8.4 int −0.5 rank 2183
yor1−dF miss 0 orf −3.7 int 0.4 rank 1276
RSF2(YJR127C)
DR = −794
Yor1 miss 0 orf 2.6 int 0 rank 2071
yor1−dF miss 0 orf 0 int 0.4 rank 1277
(YMR082C)
DR = −2555
Yor1 miss 0 orf 18.6 int −8.5 rank 3833
yor1−dF miss 0 orf −3 int 0.4 rank 1278
(YNL140C)
DR = −3004
Yor1 miss 0 orf 7.6 int −14.4 rank 4283
yor1−dF miss 0 orf 2.9 int 0.4 rank 1279
DAL3(YIR032C)
DR = −2876
Yor1 miss 0 orf 1.4 int −11.8 rank 4156
yor1−dF miss 0 orf −2.5 int 0.4 rank 1280
KTR6(YPL053C)
DR = −2334
Yor1 miss 0 orf −12.3 int −7 rank 3615
yor1−dF miss 0 orf −7 int 0.4 rank 1281
0.35 0.25 0.15 0.05 0.00
0.25 0.20 0.15 0.10 0.05 0.00
−60 −20 0 20 40
−50 0 50

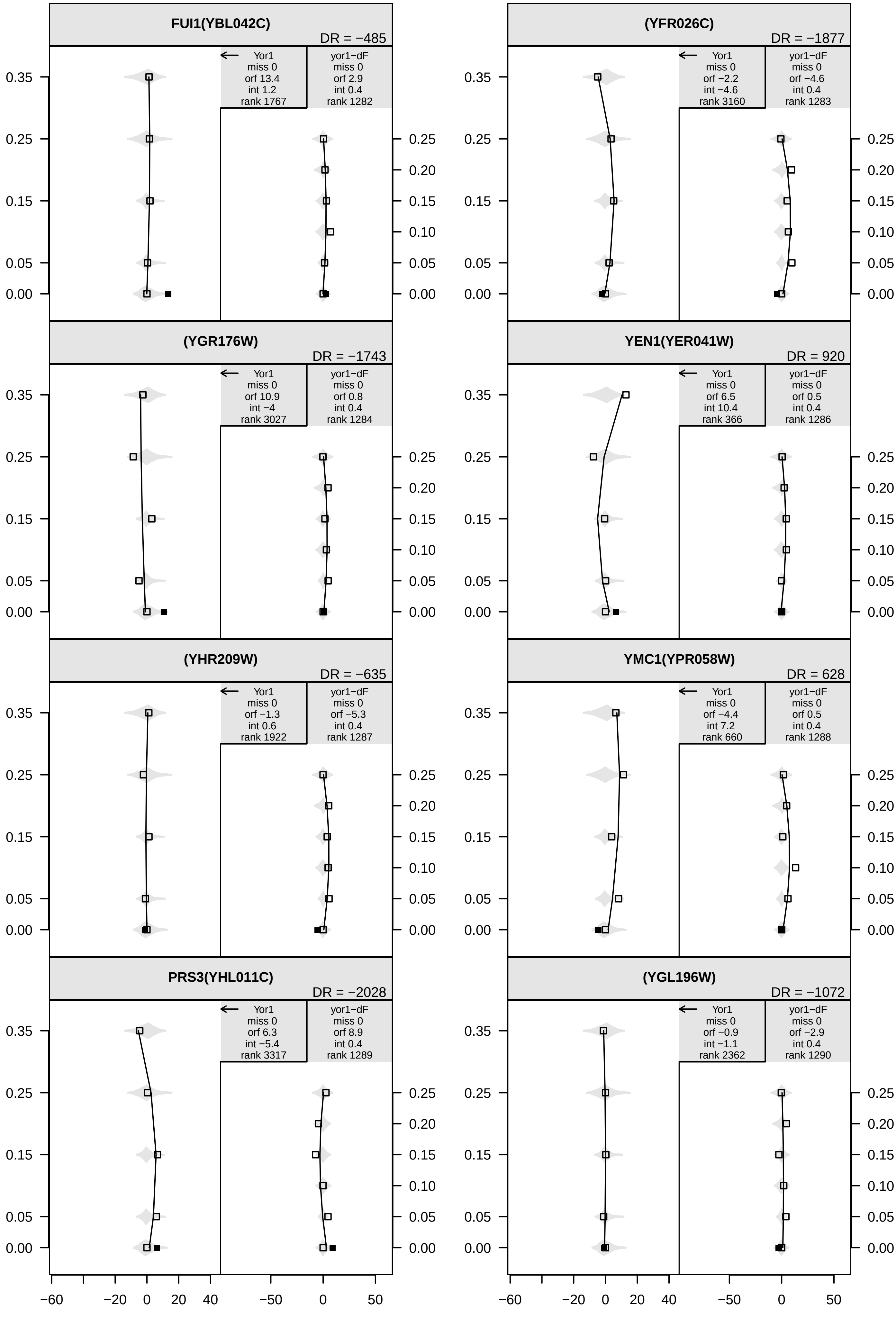
FUI1(YBL042C)
DR = −485
Yor1
miss 0
orf 13.4
int 1.2
rank 1767
yor1−dF
miss 0
orf 2.9
int 0.4
rank 1282
(YFR026C)
DR = −1877
Yor1
miss 0
orf −2.2
int −4.6
rank 3160
yor1−dF
miss 0
orf −4.6
int 0.4
rank 1283
(YGR176W)
DR = −1743
Yor1
miss 0
orf 10.9
int −4
rank 3027
yor1−dF
miss 0
orf 0.8
int 0.4
rank 1284
YEN1(YER041W)
DR = 920
Yor1
miss 0
orf 6.5
int 10.4
rank 366
yor1−dF
miss 0
orf 0.5
int 0.4
rank 1286
(YHR209W)
DR = −635
Yor1
miss 0
orf −1.3
int 0.6
rank 1922
yor1−dF
miss 0
orf −5.3
int 0.4
rank 1287
YMC1(YPR058W)
DR = 628
Yor1
miss 0
orf −4.4
int 7.2
rank 660
yor1−dF
miss 0
orf 0.5
int 0.4
rank 1288
PRS3(YHL011C)
DR = −2028
Yor1
miss 0
orf 6.3
int −5.4
rank 3317
yor1−dF
miss 0
orf 8.9
int 0.4
rank 1289
(YGL196W)
DR = −1072
Yor1
miss 0
orf −0.9
int −1.1
rank 2362
yor1−dF
miss 0
orf −2.9
int 0.4
rank 1290
0.35
0.25
0.20
0.15
0.10
0.05
0.00
−60
−20
0
20
40
−50
0
50

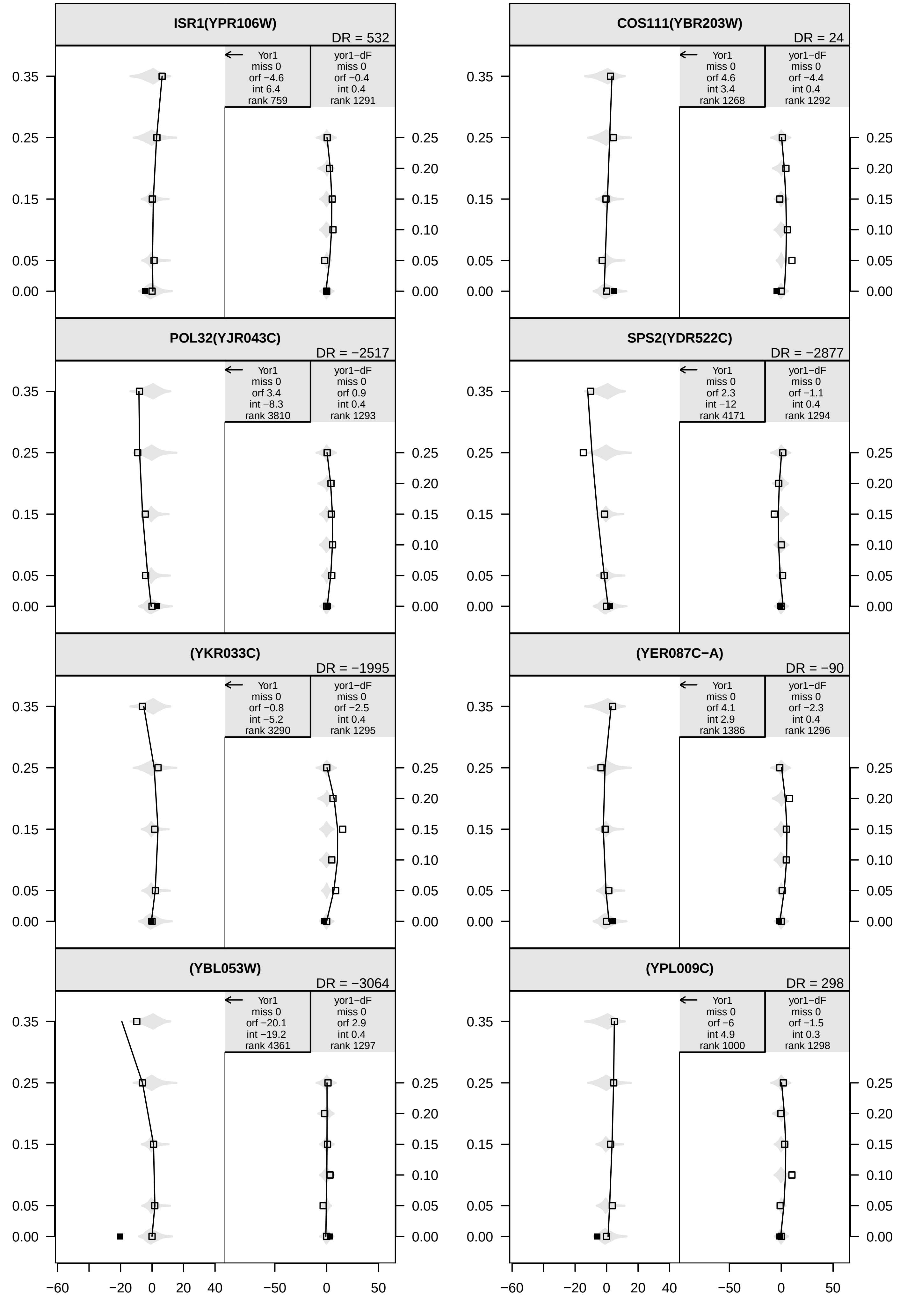

ISR1(YPR106W)
DR = 532
Yor1 miss 0 orf −4.6 int 6.4 rank 759
yor1−dF miss 0 orf −0.4 int 0.4 rank 1291
COS111(YBR203W)
DR = 24
Yor1 miss 0 orf 4.6 int 3.4 rank 1268
yor1−dF miss 0 orf −4.4 int 0.4 rank 1292
POL32(YJR043C)
DR = −2517
Yor1 miss 0 orf 3.4 int −8.3 rank 3810
yor1−dF miss 0 orf 0.9 int 0.4 rank 1293
SPS2(YDR522C)
DR = −2877
Yor1 miss 0 orf 2.3 int −12 rank 4171
yor1−dF miss 0 orf −1.1 int 0.4 rank 1294
(YKR033C)
DR = −1995
Yor1 miss 0 orf −0.8 int −5.2 rank 3290
yor1−dF miss 0 orf −2.5 int 0.4 rank 1295
(YER087C−A)
DR = −90
Yor1 miss 0 orf 4.1 int 2.9 rank 1386
yor1−dF miss 0 orf −2.3 int 0.4 rank 1296
(YBL053W)
DR = −3064
Yor1 miss 0 orf −20.1 int −19.2 rank 4361
yor1−dF miss 0 orf 2.9 int 0.4 rank 1297
(YPL009C)
DR = 298
Yor1 miss 0 orf −6 int 4.9 rank 1000
yor1−dF miss 0 orf −1.5 int 0.3 rank 1298
0.00 0.05 0.10 0.15 0.20 0.25 0.35
−60 −20 0 20 40 −50 0 50

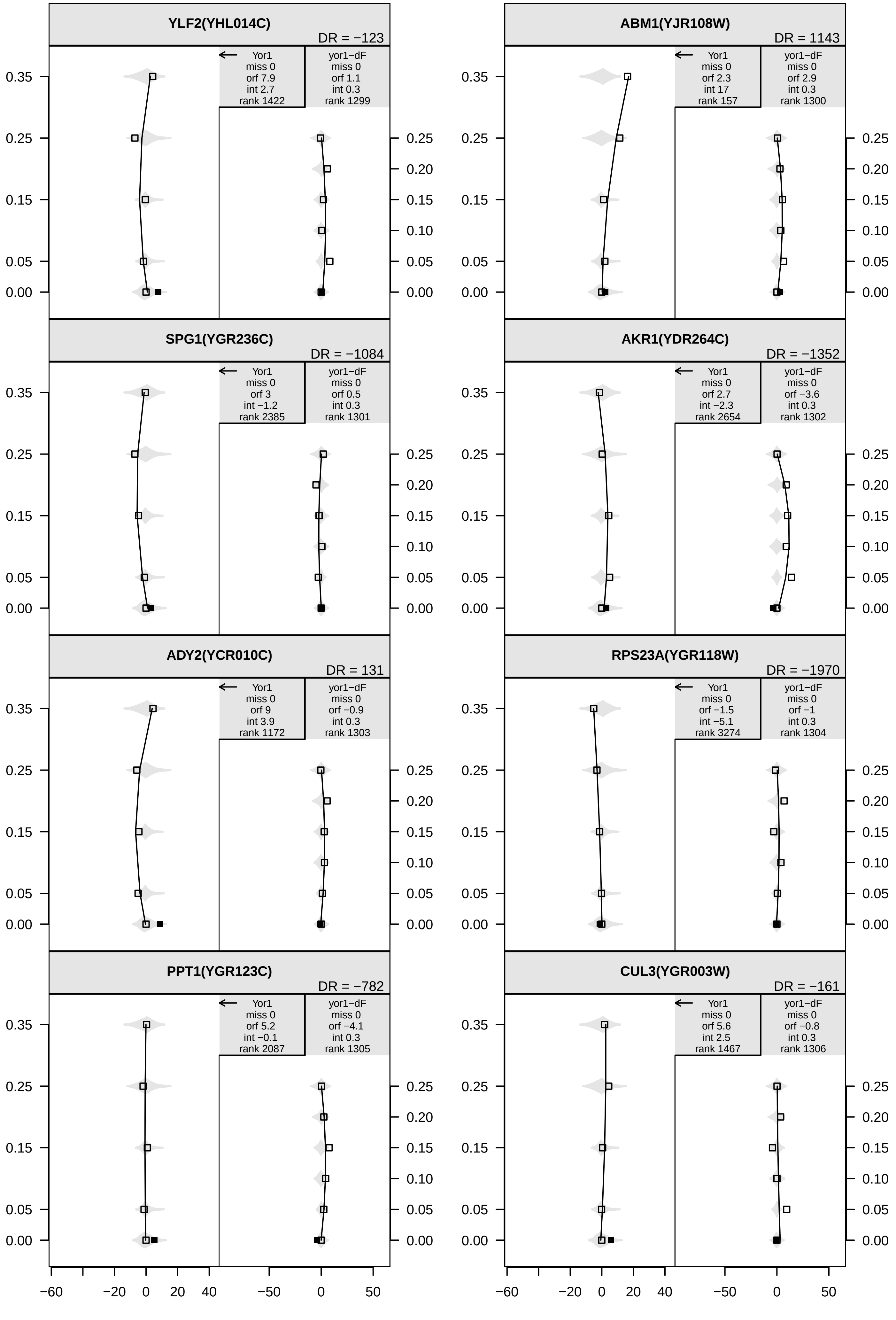
YLF2(YHL014C)
DR = −123
Yor1 miss 0 orf 7.9 int 2.7 rank 1422
yor1−dF miss 0 orf 1.1 int 0.3 rank 1299
ABM1(YJR108W)
DR = 1143
Yor1 miss 0 orf 2.3 int 17 rank 157
yor1−dF miss 0 orf 2.9 int 0.3 rank 1300
SPG1(YGR236C)
DR = −1084
Yor1 miss 0 orf 3 int −1.2 rank 2385
yor1−dF miss 0 orf 0.5 int 0.3 rank 1301
AKR1(YDR264C)
DR = −1352
Yor1 miss 0 orf 2.7 int −2.3 rank 2654
yor1−dF miss 0 orf −3.6 int 0.3 rank 1302
ADY2(YCR010C)
DR = 131
Yor1 miss 0 orf 9 int 3.9 rank 1172
yor1−dF miss 0 orf −0.9 int 0.3 rank 1303
RPS23A(YGR118W)
DR = −1970
Yor1 miss 0 orf −1.5 int −5.1 rank 3274
yor1−dF miss 0 orf −1 int 0.3 rank 1304
PPT1(YGR123C)
DR = −782
Yor1 miss 0 orf 5.2 int −0.1 rank 2087
yor1−dF miss 0 orf −4.1 int 0.3 rank 1305
CUL3(YGR003W)
DR = −161
Yor1 miss 0 orf 5.6 int 2.5 rank 1467
yor1−dF miss 0 orf −0.8 int 0.3 rank 1306
0.35 0.25 0.15 0.05 0.00
0.25 0.20 0.15 0.10 0.05 0.00
−60 −20 0 20 40
−50 0 50

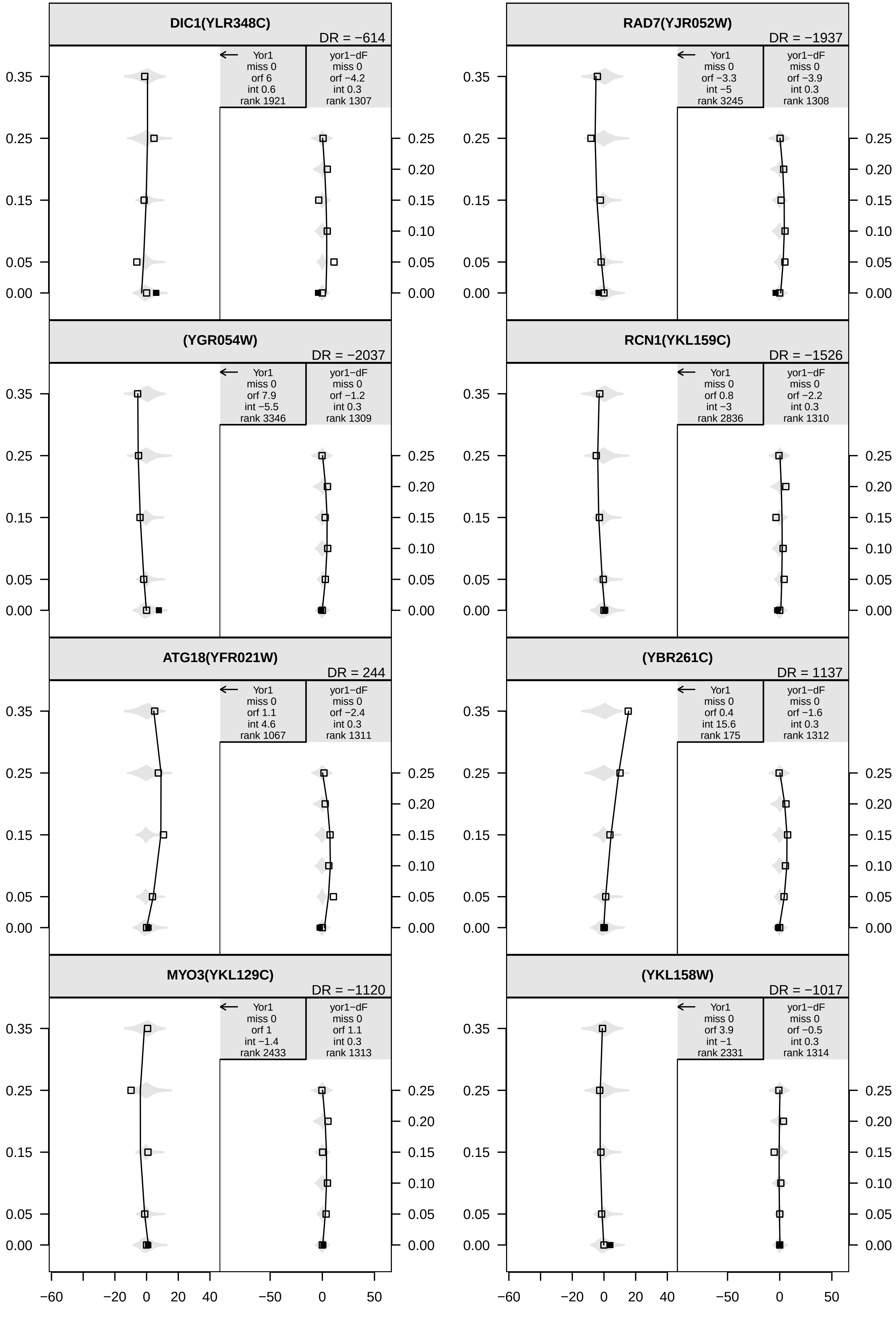
DIC1(YLR348C)
DR = −614
Yor1
miss 0
orf 6
int 0.6
rank 1921
yor1−dF
miss 0
orf −4.2
int 0.3
rank 1307
RAD7(YJR052W)
DR = −1937
Yor1
miss 0
orf −3.3
int −5
rank 3245
yor1−dF
miss 0
orf −3.9
int 0.3
rank 1308
(YGR054W)
DR = −2037
Yor1
miss 0
orf 7.9
int −5.5
rank 3346
yor1−dF
miss 0
orf −1.2
int 0.3
rank 1309
RCN1(YKL159C)
DR = −1526
Yor1
miss 0
orf 0.8
int −3
rank 2836
yor1−dF
miss 0
orf −2.2
int 0.3
rank 1310
ATG18(YFR021W)
DR = 244
Yor1
miss 0
orf 1.1
int 4.6
rank 1067
yor1−dF
miss 0
orf −2.4
int 0.3
rank 1311
(YBR261C)
DR = 1137
Yor1
miss 0
orf 0.4
int 15.6
rank 175
yor1−dF
miss 0
orf −1.6
int 0.3
rank 1312
MYO3(YKL129C)
DR = −1120
Yor1
miss 0
orf 1
int −1.4
rank 2433
yor1−dF
miss 0
orf 1.1
int 0.3
rank 1313
(YKL158W)
DR = −1017
Yor1
miss 0
orf 3.9
int −1
rank 2331
yor1−dF
miss 0
orf −0.5
int 0.3
rank 1314
0.35
0.25
0.20
0.15
0.10
0.05
0.00
−60
−20
0
20
40
−50
0
50

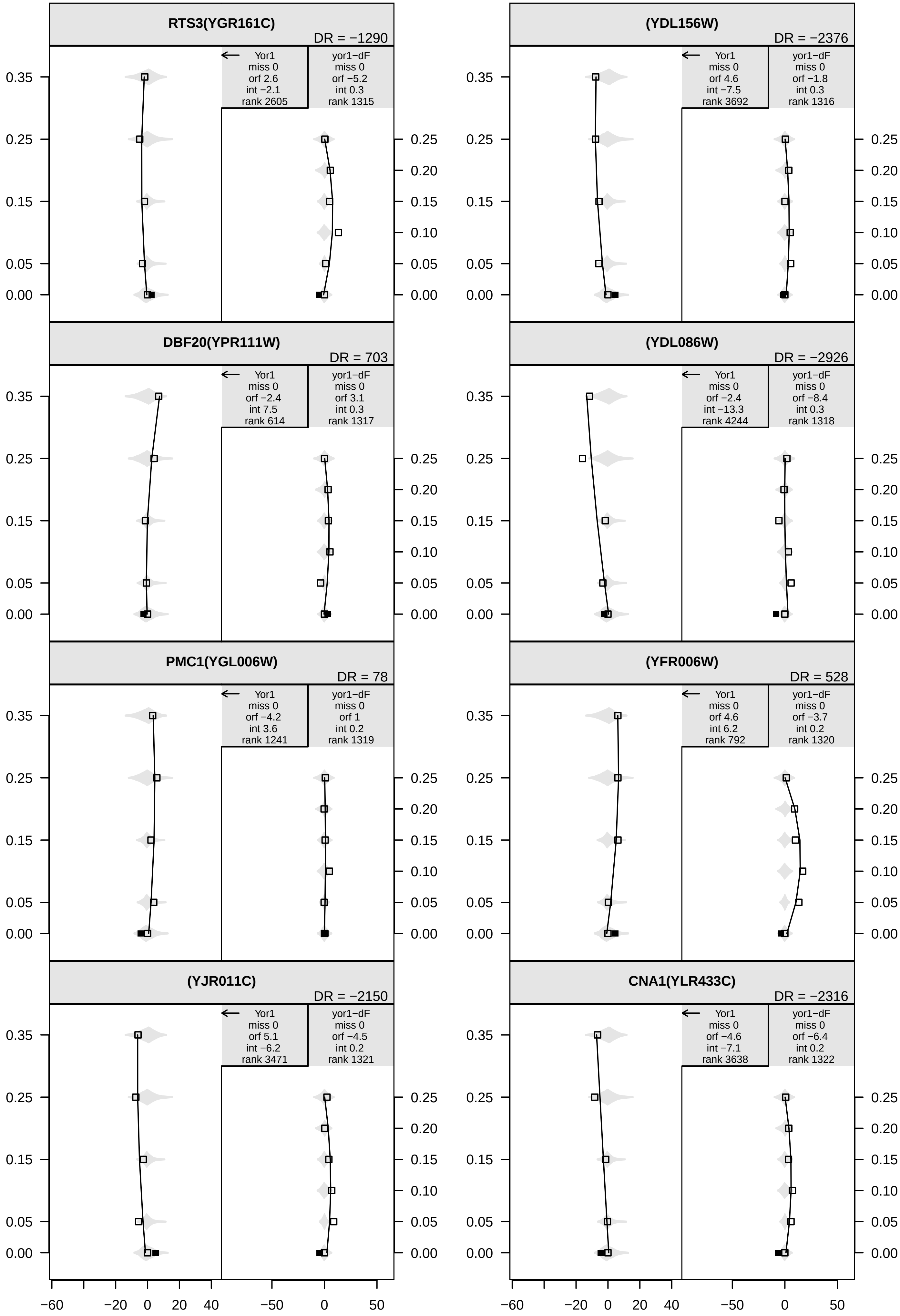
RTS3(YGR161C)
DR = −1290
Yor1 miss 0 orf 2.6 int −2.1 rank 2605
yor1−dF miss 0 orf −5.2 int 0.3 rank 1315
(YDL156W)
DR = −2376
Yor1 miss 0 orf 4.6 int −7.5 rank 3692
yor1−dF miss 0 orf −1.8 int 0.3 rank 1316
DBF20(YPR111W)
DR = 703
Yor1 miss 0 orf −2.4 int 7.5 rank 614
yor1−dF miss 0 orf 3.1 int 0.3 rank 1317
(YDL086W)
DR = −2926
Yor1 miss 0 orf −2.4 int −13.3 rank 4244
yor1−dF miss 0 orf −8.4 int 0.3 rank 1318
PMC1(YGL006W)
DR = 78
Yor1 miss 0 orf −4.2 int 3.6 rank 1241
yor1−dF miss 0 orf 1 int 0.2 rank 1319
(YFR006W)
DR = 528
Yor1 miss 0 orf 4.6 int 6.2 rank 792
yor1−dF miss 0 orf −3.7 int 0.2 rank 1320
(YJR011C)
DR = −2150
Yor1 miss 0 orf 5.1 int −6.2 rank 3471
yor1−dF miss 0 orf −4.5 int 0.2 rank 1321
CNA1(YLR433C)
DR = −2316
Yor1 miss 0 orf −4.6 int −7.1 rank 3638
yor1−dF miss 0 orf −6.4 int 0.2 rank 1322
0.35
0.25
0.20
0.15
0.10
0.05
0.00
−60
−20
0
20
40
−50
0
50

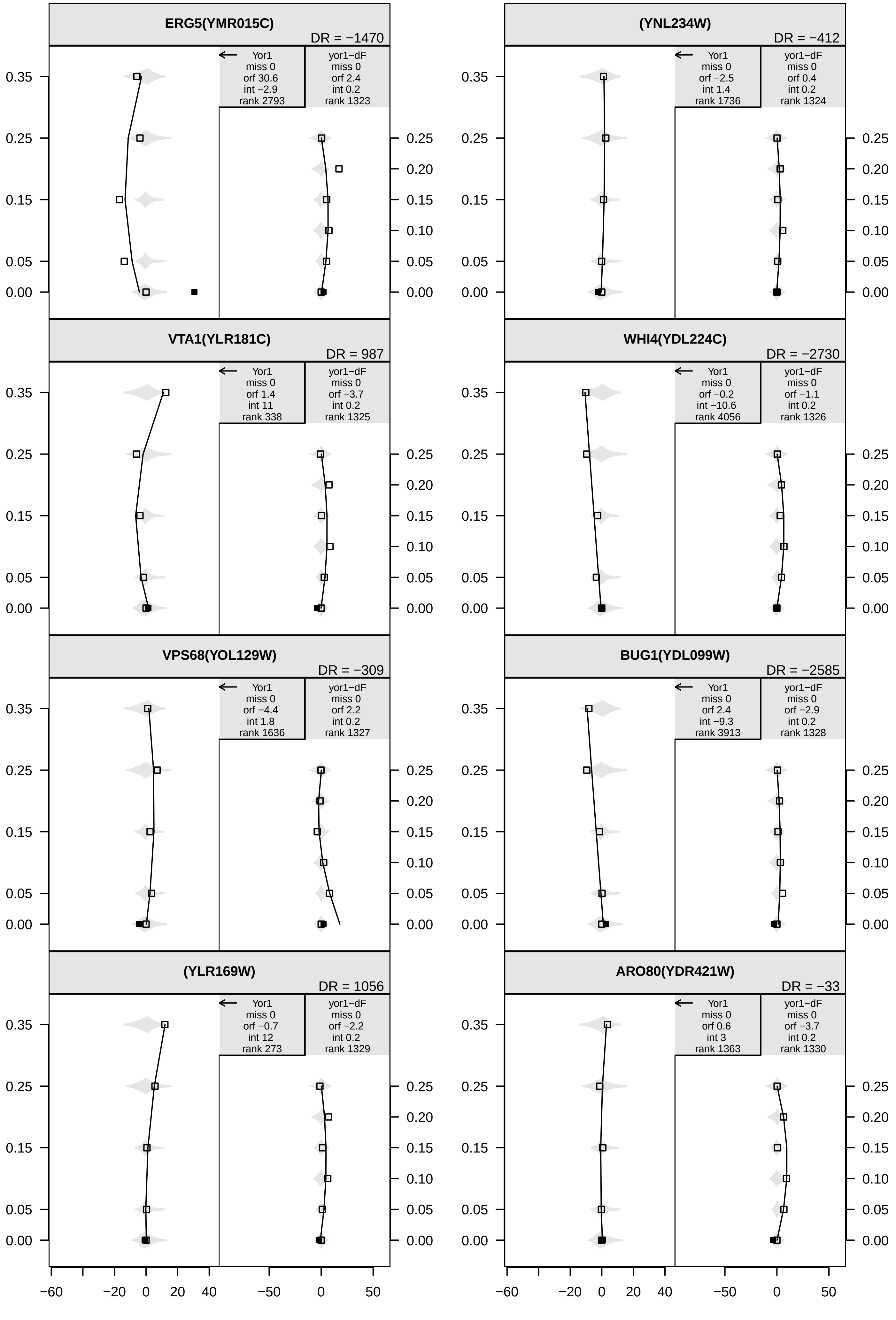
ERG5(YMR015C)
DR = −1470
Yor1 miss 0 orf 30.6 int −2.9 rank 2793
yor1−dF miss 0 orf 2.4 int 0.2 rank 1323
(YNL234W)
DR = −412
Yor1 miss 0 orf −2.5 int 1.4 rank 1736
yor1−dF miss 0 orf 0.4 int 0.2 rank 1324
VTA1(YLR181C)
DR = 987
Yor1 miss 0 orf 1.4 int 11 rank 338
yor1−dF miss 0 orf −3.7 int 0.2 rank 1325
WHI4(YDL224C)
DR = −2730
Yor1 miss 0 orf −0.2 int −10.6 rank 4056
yor1−dF miss 0 orf −1.1 int 0.2 rank 1326
VPS68(YOL129W)
DR = −309
Yor1 miss 0 orf −4.4 int 1.8 rank 1636
yor1−dF miss 0 orf 2.2 int 0.2 rank 1327
BUG1(YDL099W)
DR = −2585
Yor1 miss 0 orf 2.4 int −9.3 rank 3913
yor1−dF miss 0 orf −2.9 int 0.2 rank 1328
(YLR169W)
DR = 1056
Yor1 miss 0 orf −0.7 int 12 rank 273
yor1−dF miss 0 orf −2.2 int 0.2 rank 1329
ARO80(YDR421W)
DR = −33
Yor1 miss 0 orf 0.6 int 3 rank 1363
yor1−dF miss 0 orf −3.7 int 0.2 rank 1330
0.00 0.05 0.10 0.15 0.20 0.25 0.35
−60 −20 0 20 40 −50 0 50

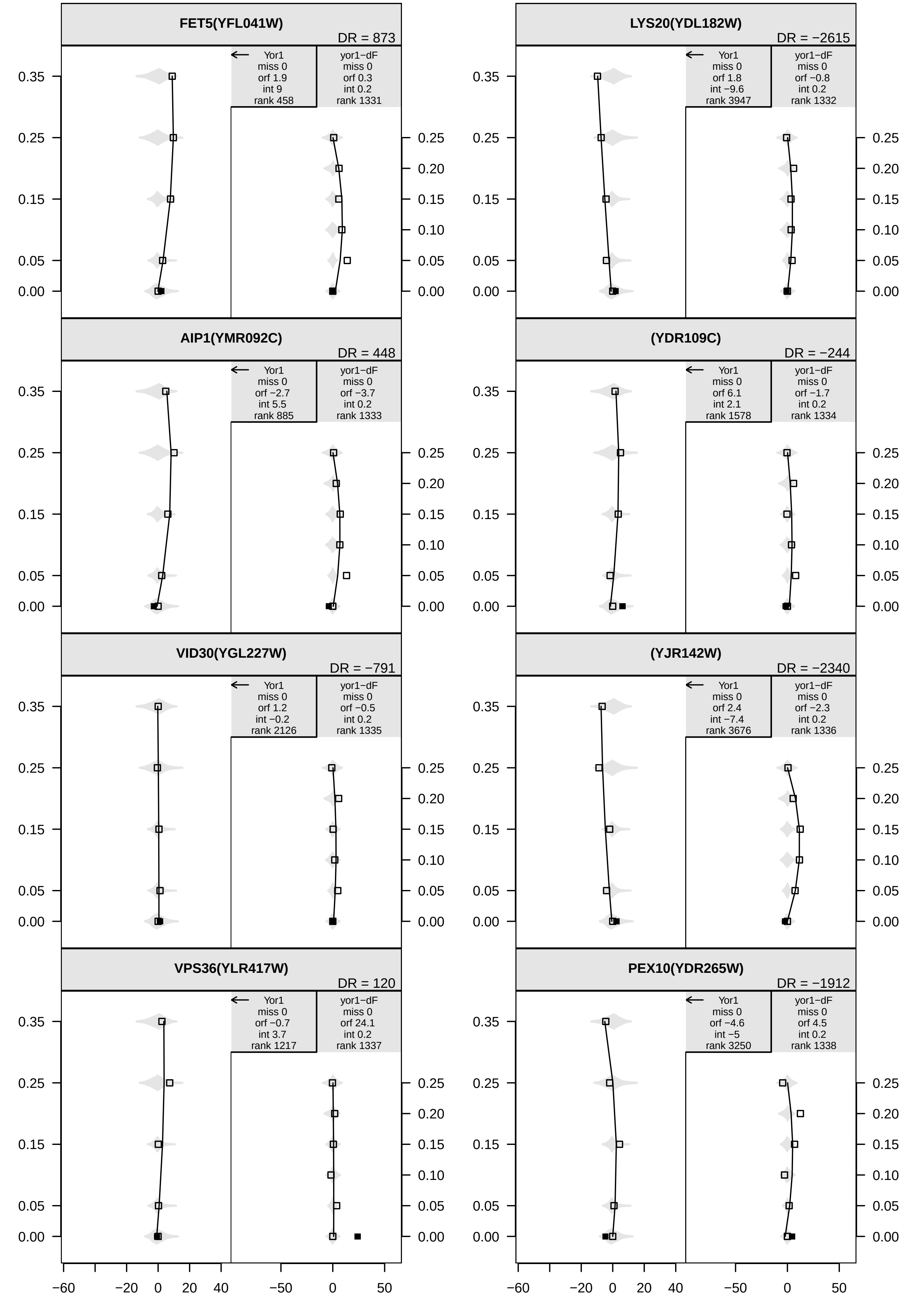
FET5(YFL041W)
DR = 873
Yor1
miss 0
orf 1.9
int 9
rank 458
yor1−dF
miss 0
orf 0.3
int 0.2
rank 1331
LYS20(YDL182W)
DR = −2615
Yor1
miss 0
orf 1.8
int −9.6
rank 3947
yor1−dF
miss 0
orf −0.8
int 0.2
rank 1332
AIP1(YMR092C)
DR = 448
Yor1
miss 0
orf −2.7
int 5.5
rank 885
yor1−dF
miss 0
orf −3.7
int 0.2
rank 1333
(YDR109C)
DR = −244
Yor1
miss 0
orf 6.1
int 2.1
rank 1578
yor1−dF
miss 0
orf −1.7
int 0.2
rank 1334
VID30(YGL227W)
DR = −791
Yor1
miss 0
orf 1.2
int −0.2
rank 2126
yor1−dF
miss 0
orf −0.5
int 0.2
rank 1335
(YJR142W)
DR = −2340
Yor1
miss 0
orf 2.4
int −7.4
rank 3676
yor1−dF
miss 0
orf −2.3
int 0.2
rank 1336
VPS36(YLR417W)
DR = 120
Yor1
miss 0
orf −0.7
int 3.7
rank 1217
yor1−dF
miss 0
orf 24.1
int 0.2
rank 1337
PEX10(YDR265W)
DR = −1912
Yor1
miss 0
orf −4.6
int −5
rank 3250
yor1−dF
miss 0
orf 4.5
int 0.2
rank 1338
0.35
0.25
0.20
0.15
0.10
0.05
0.00
−60
−20
0
20
40
−50
0
50

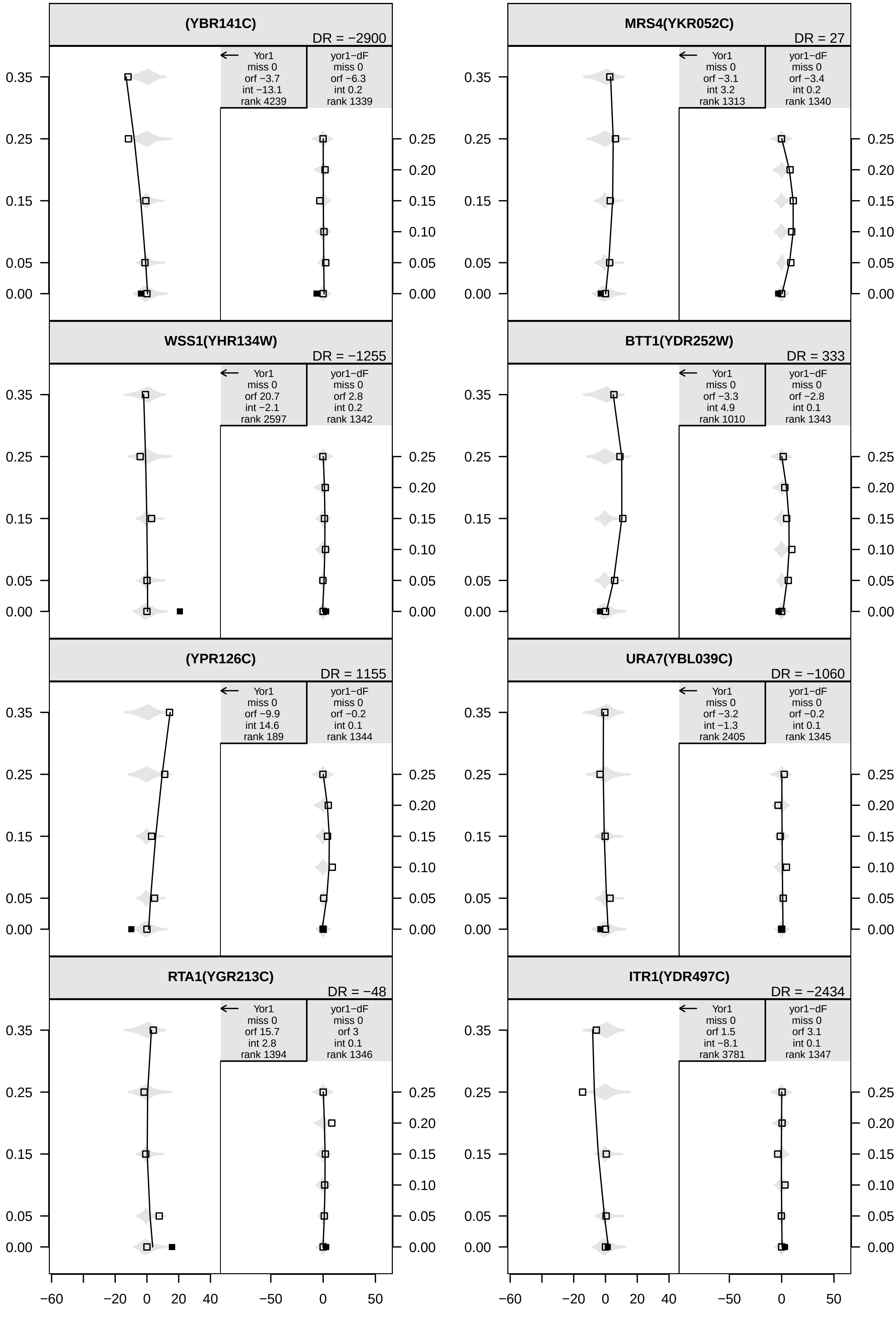

(YBR141C)
DR = −2900
Yor1 miss 0 orf −3.7 int −13.1 rank 4239
yor1−dF miss 0 orf −6.3 int 0.2 rank 1339
MRS4(YKR052C)
DR = 27
Yor1 miss 0 orf −3.1 int 3.2 rank 1313
yor1−dF miss 0 orf −3.4 int 0.2 rank 1340
WSS1(YHR134W)
DR = −1255
Yor1 miss 0 orf 20.7 int −2.1 rank 2597
yor1−dF miss 0 orf 2.8 int 0.2 rank 1342
BTT1(YDR252W)
DR = 333
Yor1 miss 0 orf −3.3 int 4.9 rank 1010
yor1−dF miss 0 orf −2.8 int 0.1 rank 1343
(YPR126C)
DR = 1155
Yor1 miss 0 orf −9.9 int 14.6 rank 189
yor1−dF miss 0 orf −0.2 int 0.1 rank 1344
URA7(YBL039C)
DR = −1060
Yor1 miss 0 orf −3.2 int −1.3 rank 2405
yor1−dF miss 0 orf −0.2 int 0.1 rank 1345
RTA1(YGR213C)
DR = −48
Yor1 miss 0 orf 15.7 int 2.8 rank 1394
yor1−dF miss 0 orf 3 int 0.1 rank 1346
ITR1(YDR497C)
DR = −2434
Yor1 miss 0 orf 1.5 int −8.1 rank 3781
yor1−dF miss 0 orf 3.1 int 0.1 rank 1347

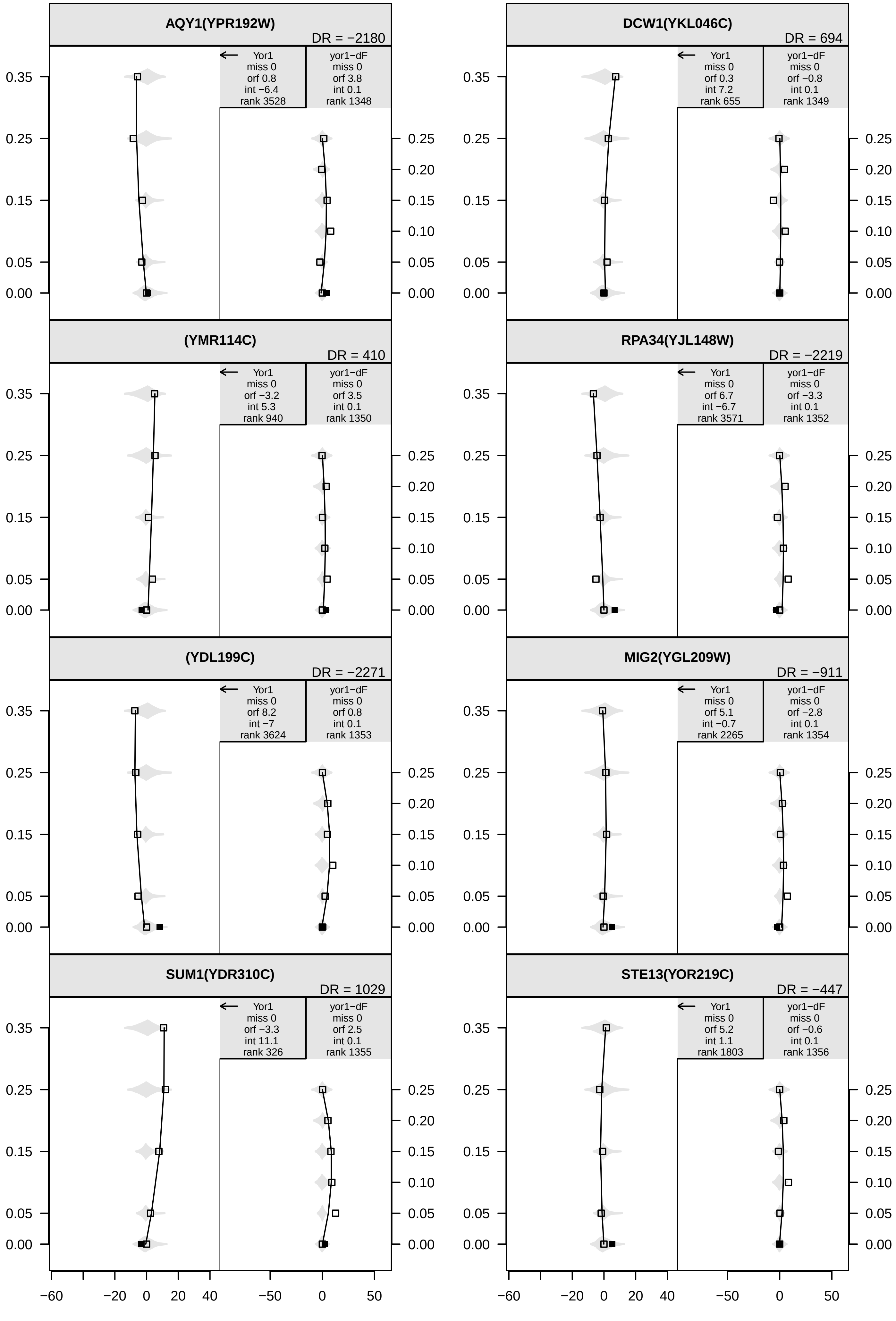
AQY1(YPR192W)
DR = −2180
Yor1 miss 0 orf 0.8 int −6.4 rank 3528
yor1−dF miss 0 orf 3.8 int 0.1 rank 1348
DCW1(YKL046C)
DR = 694
Yor1 miss 0 orf 0.3 int 7.2 rank 655
yor1−dF miss 0 orf −0.8 int 0.1 rank 1349
(YMR114C)
DR = 410
Yor1 miss 0 orf −3.2 int 5.3 rank 940
yor1−dF miss 0 orf 3.5 int 0.1 rank 1350
RPA34(YJL148W)
DR = −2219
Yor1 miss 0 orf 6.7 int −6.7 rank 3571
yor1−dF miss 0 orf −3.3 int 0.1 rank 1352
(YDL199C)
DR = −2271
Yor1 miss 0 orf 8.2 int −7 rank 3624
yor1−dF miss 0 orf 0.8 int 0.1 rank 1353
MIG2(YGL209W)
DR = −911
Yor1 miss 0 orf 5.1 int −0.7 rank 2265
yor1−dF miss 0 orf −2.8 int 0.1 rank 1354
SUM1(YDR310C)
DR = 1029
Yor1 miss 0 orf −3.3 int 11.1 rank 326
yor1−dF miss 0 orf 2.5 int 0.1 rank 1355
STE13(YOR219C)
DR = −447
Yor1 miss 0 orf 5.2 int 1.1 rank 1803
yor1−dF miss 0 orf −0.6 int 0.1 rank 1356
0.35
0.25
0.20
0.15
0.10
0.05
0.00
−60
−20
0
20
40
−50
0
50

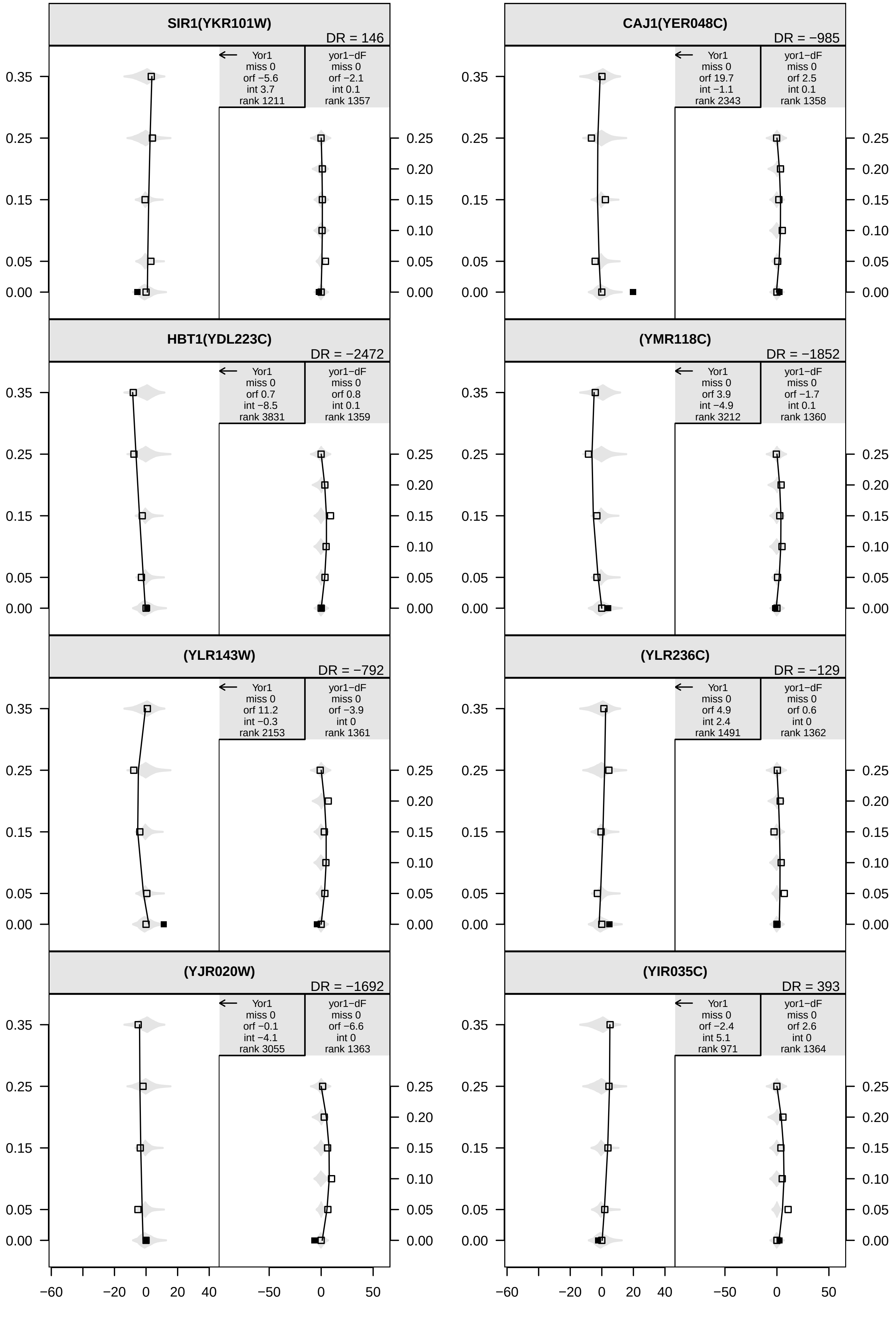
SIR1(YKR101W)
DR = 146
Yor1 miss 0 orf −5.6 int 3.7 rank 1211
yor1−dF miss 0 orf −2.1 int 0.1 rank 1357
CAJ1(YER048C)
DR = −985
Yor1 miss 0 orf 19.7 int −1.1 rank 2343
yor1−dF miss 0 orf 2.5 int 0.1 rank 1358
HBT1(YDL223C)
DR = −2472
Yor1 miss 0 orf 0.7 int −8.5 rank 3831
yor1−dF miss 0 orf 0.8 int 0.1 rank 1359
(YMR118C)
DR = −1852
Yor1 miss 0 orf 3.9 int −4.9 rank 3212
yor1−dF miss 0 orf −1.7 int 0.1 rank 1360
(YLR143W)
DR = −792
Yor1 miss 0 orf 11.2 int −0.3 rank 2153
yor1−dF miss 0 orf −3.9 int 0 rank 1361
(YLR236C)
DR = −129
Yor1 miss 0 orf 4.9 int 2.4 rank 1491
yor1−dF miss 0 orf 0.6 int 0 rank 1362
(YJR020W)
DR = −1692
Yor1 miss 0 orf −0.1 int −4.1 rank 3055
yor1−dF miss 0 orf −6.6 int 0 rank 1363
(YIR035C)
DR = 393
Yor1 miss 0 orf −2.4 int 5.1 rank 971
yor1−dF miss 0 orf 2.6 int 0 rank 1364
0.35 0.25 0.15 0.05 0.00
0.25 0.20 0.15 0.10 0.05 0.00
−60 −20 0 20 40 −50 0 50

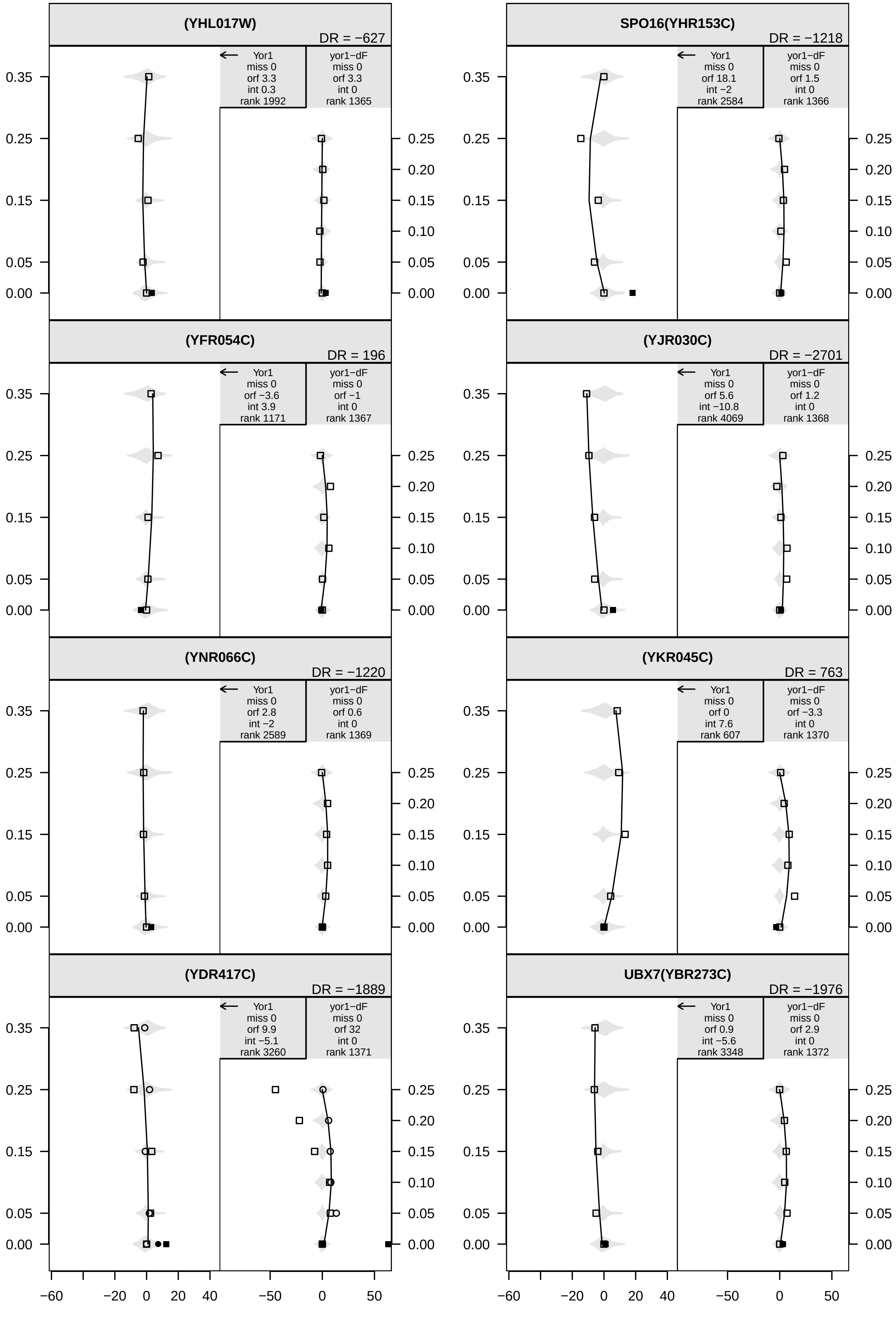

(YHL017W)
DR = −627
Yor1
miss 0
orf 3.3
int 0.3
rank 1992
yor1−dF
miss 0
orf 3.3
int 0
rank 1365
SPO16(YHR153C)
DR = −1218
Yor1
miss 0
orf 18.1
int −2
rank 2584
yor1−dF
miss 0
orf 1.5
int 0
rank 1366
(YFR054C)
DR = 196
Yor1
miss 0
orf −3.6
int 3.9
rank 1171
yor1−dF
miss 0
orf −1
int 0
rank 1367
(YJR030C)
DR = −2701
Yor1
miss 0
orf 5.6
int −10.8
rank 4069
yor1−dF
miss 0
orf 1.2
int 0
rank 1368
(YNR066C)
DR = −1220
Yor1
miss 0
orf 2.8
int −2
rank 2589
yor1−dF
miss 0
orf 0.6
int 0
rank 1369
(YKR045C)
DR = 763
Yor1
miss 0
orf 0
int 7.6
rank 607
yor1−dF
miss 0
orf −3.3
int 0
rank 1370
(YDR417C)
DR = −1889
Yor1
miss 0
orf 9.9
int −5.1
rank 3260
yor1−dF
miss 0
orf 32
int 0
rank 1371
UBX7(YBR273C)
DR = −1976
Yor1
miss 0
orf 0.9
int −5.6
rank 3348
yor1−dF
miss 0
orf 2.9
int 0
rank 1372
0.35
0.25
0.20
0.15
0.10
0.05
0.00
−60
−20
0
20
40
−50
0
50

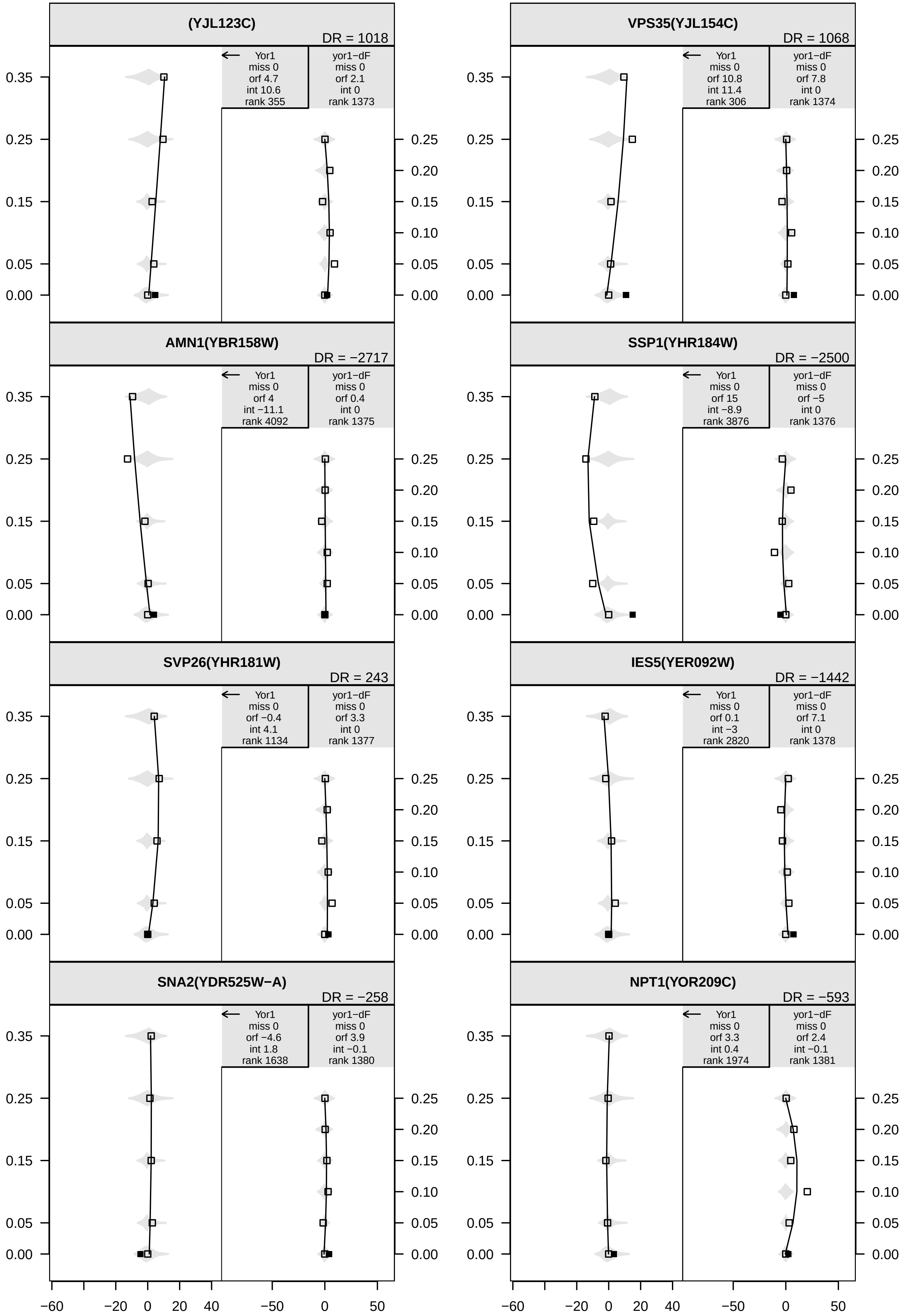
(YJL123C)
DR = 1018
Yor1 miss 0 orf 4.7 int 10.6 rank 355
yor1−dF miss 0 orf 2.1 int 0 rank 1373
VPS35(YJL154C)
DR = 1068
Yor1 miss 0 orf 10.8 int 11.4 rank 306
yor1−dF miss 0 orf 7.8 int 0 rank 1374
AMN1(YBR158W)
DR = −2717
Yor1 miss 0 orf 4 int −11.1 rank 4092
yor1−dF miss 0 orf 0.4 int 0 rank 1375
SSP1(YHR184W)
DR = −2500
Yor1 miss 0 orf 15 int −8.9 rank 3876
yor1−dF miss 0 orf −5 int 0 rank 1376
SVP26(YHR181W)
DR = 243
Yor1 miss 0 orf −0.4 int 4.1 rank 1134
yor1−dF miss 0 orf 3.3 int 0 rank 1377
IES5(YER092W)
DR = −1442
Yor1 miss 0 orf 0.1 int −3 rank 2820
yor1−dF miss 0 orf 7.1 int 0 rank 1378
SNA2(YDR525W−A)
DR = −258
Yor1 miss 0 orf −4.6 int 1.8 rank 1638
yor1−dF miss 0 orf 3.9 int −0.1 rank 1380
NPT1(YOR209C)
DR = −593
Yor1 miss 0 orf 3.3 int 0.4 rank 1974
yor1−dF miss 0 orf 2.4 int −0.1 rank 1381
0.35 0.25 0.15 0.05 0.00
0.25 0.20 0.15 0.10 0.05 0.00
−60 −20 0 20 40
−50 0 50

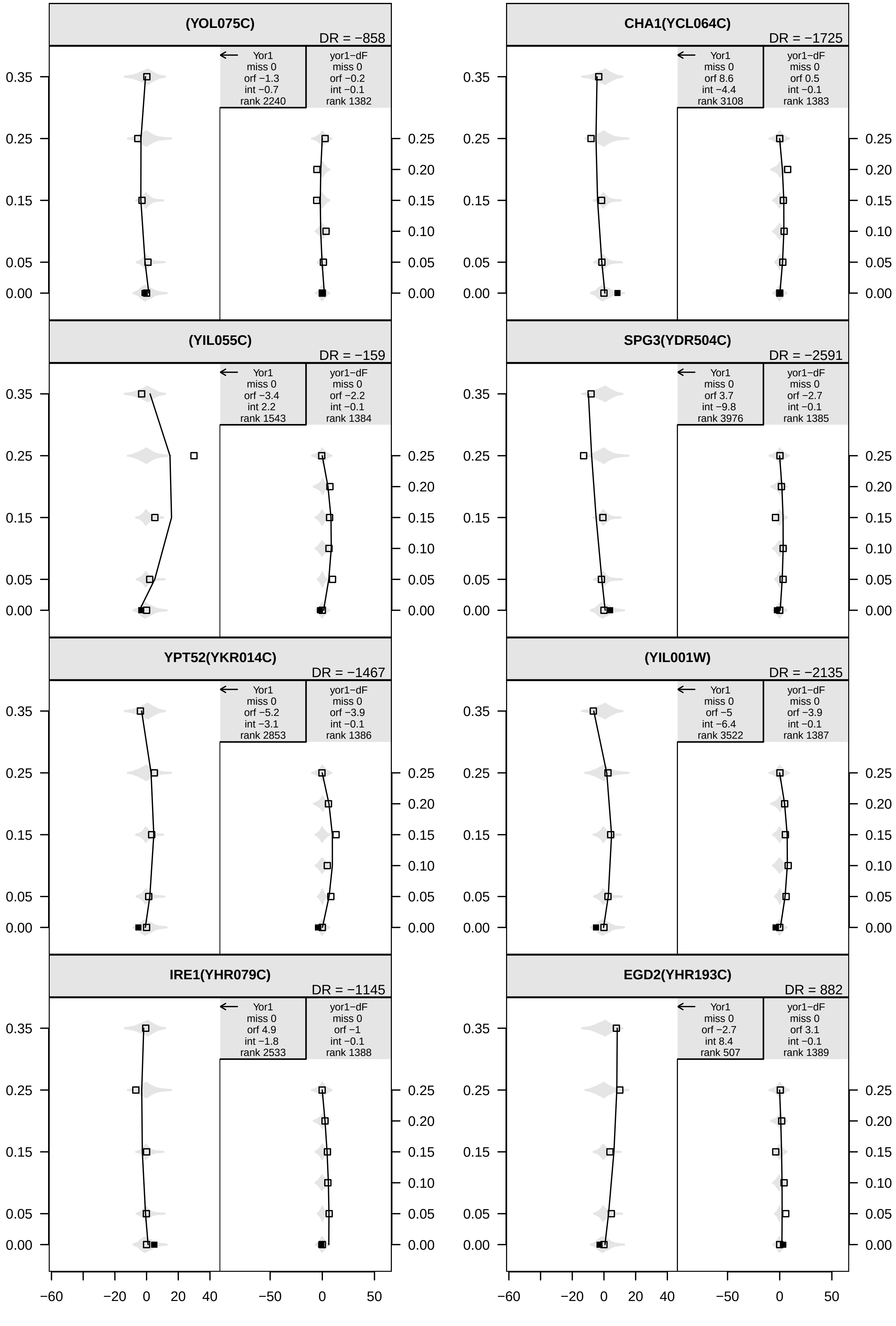
(YOL075C)
DR = −858
Yor1
miss 0
orf −1.3
int −0.7
rank 2240
yor1−dF
miss 0
orf −0.2
int −0.1
rank 1382
CHA1(YCL064C)
DR = −1725
Yor1
miss 0
orf 8.6
int −4.4
rank 3108
yor1−dF
miss 0
orf 0.5
int −0.1
rank 1383
(YIL055C)
DR = −159
Yor1
miss 0
orf −3.4
int 2.2
rank 1543
yor1−dF
miss 0
orf −2.2
int −0.1
rank 1384
SPG3(YDR504C)
DR = −2591
Yor1
miss 0
orf 3.7
int −9.8
rank 3976
yor1−dF
miss 0
orf −2.7
int −0.1
rank 1385
YPT52(YKR014C)
DR = −1467
Yor1
miss 0
orf −5.2
int −3.1
rank 2853
yor1−dF
miss 0
orf −3.9
int −0.1
rank 1386
(YIL001W)
DR = −2135
Yor1
miss 0
orf −5
int −6.4
rank 3522
yor1−dF
miss 0
orf −3.9
int −0.1
rank 1387
IRE1(YHR079C)
DR = −1145
Yor1
miss 0
orf 4.9
int −1.8
rank 2533
yor1−dF
miss 0
orf −1
int −0.1
rank 1388
EGD2(YHR193C)
DR = 882
Yor1
miss 0
orf −2.7
int 8.4
rank 507
yor1−dF
miss 0
orf 3.1
int −0.1
rank 1389
0.35
0.25
0.20
0.15
0.10
0.05
0.00
−60
−20
0
20
40
−50
0
50

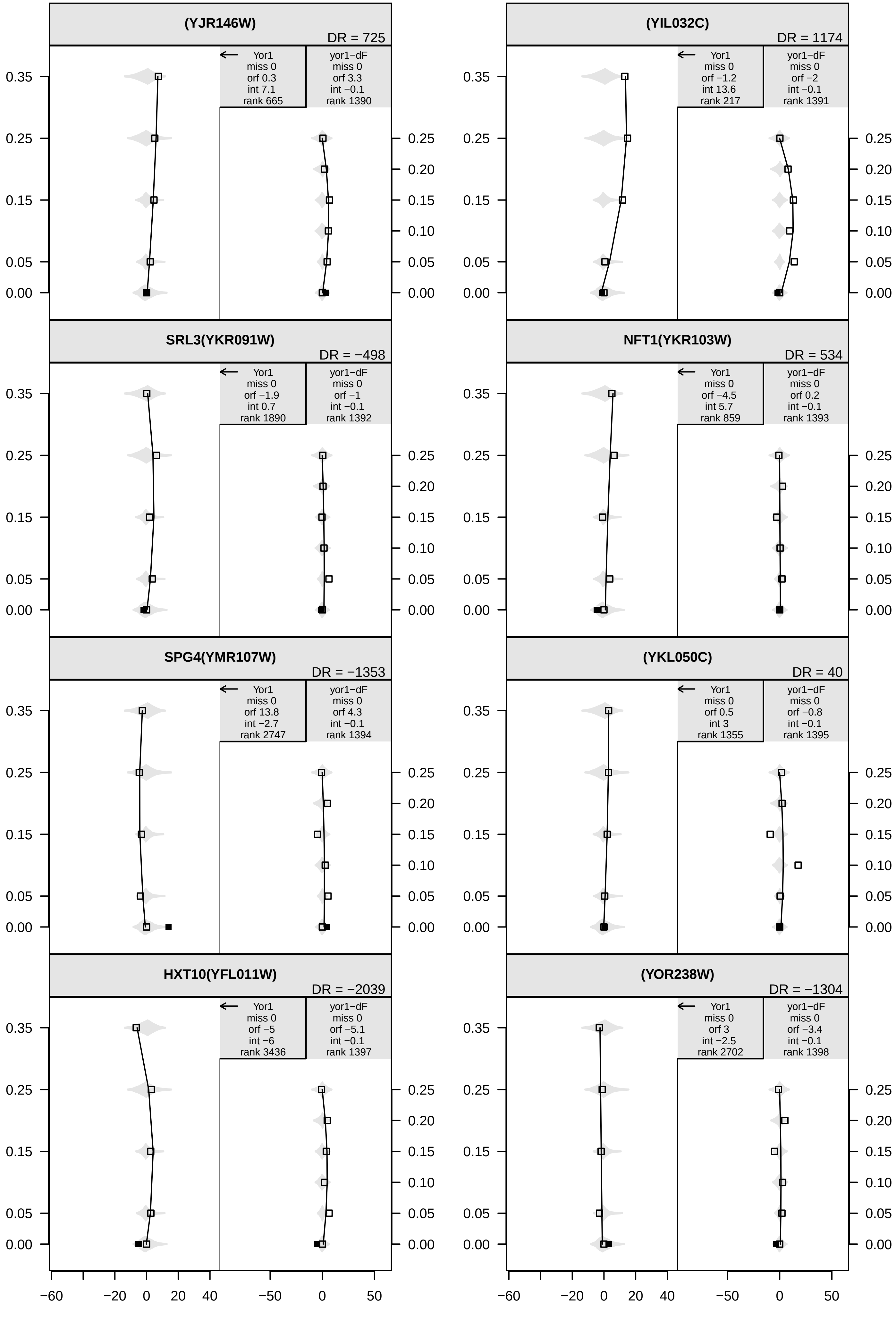
(YJR146W)
DR = 725
Yor1 miss 0 orf 0.3 int 7.1 rank 665
yor1−dF miss 0 orf 3.3 int −0.1 rank 1390
(YIL032C)
DR = 1174
Yor1 miss 0 orf −1.2 int 13.6 rank 217
yor1−dF miss 0 orf −2 int −0.1 rank 1391
SRL3(YKR091W)
DR = −498
Yor1 miss 0 orf −1.9 int 0.7 rank 1890
yor1−dF miss 0 orf −1 int −0.1 rank 1392
NFT1(YKR103W)
DR = 534
Yor1 miss 0 orf −4.5 int 5.7 rank 859
yor1−dF miss 0 orf 0.2 int −0.1 rank 1393
SPG4(YMR107W)
DR = −1353
Yor1 miss 0 orf 13.8 int −2.7 rank 2747
yor1−dF miss 0 orf 4.3 int −0.1 rank 1394
(YKL050C)
DR = 40
Yor1 miss 0 orf 0.5 int 3 rank 1355
yor1−dF miss 0 orf −0.8 int −0.1 rank 1395
HXT10(YFL011W)
DR = −2039
Yor1 miss 0 orf −5 int −6 rank 3436
yor1−dF miss 0 orf −5.1 int −0.1 rank 1397
(YOR238W)
DR = −1304
Yor1 miss 0 orf 3 int −2.5 rank 2702
yor1−dF miss 0 orf −3.4 int −0.1 rank 1398
0.35 0.25 0.20 0.15 0.10 0.05 0.00
−60 −20 0 20 40 −50 0 50

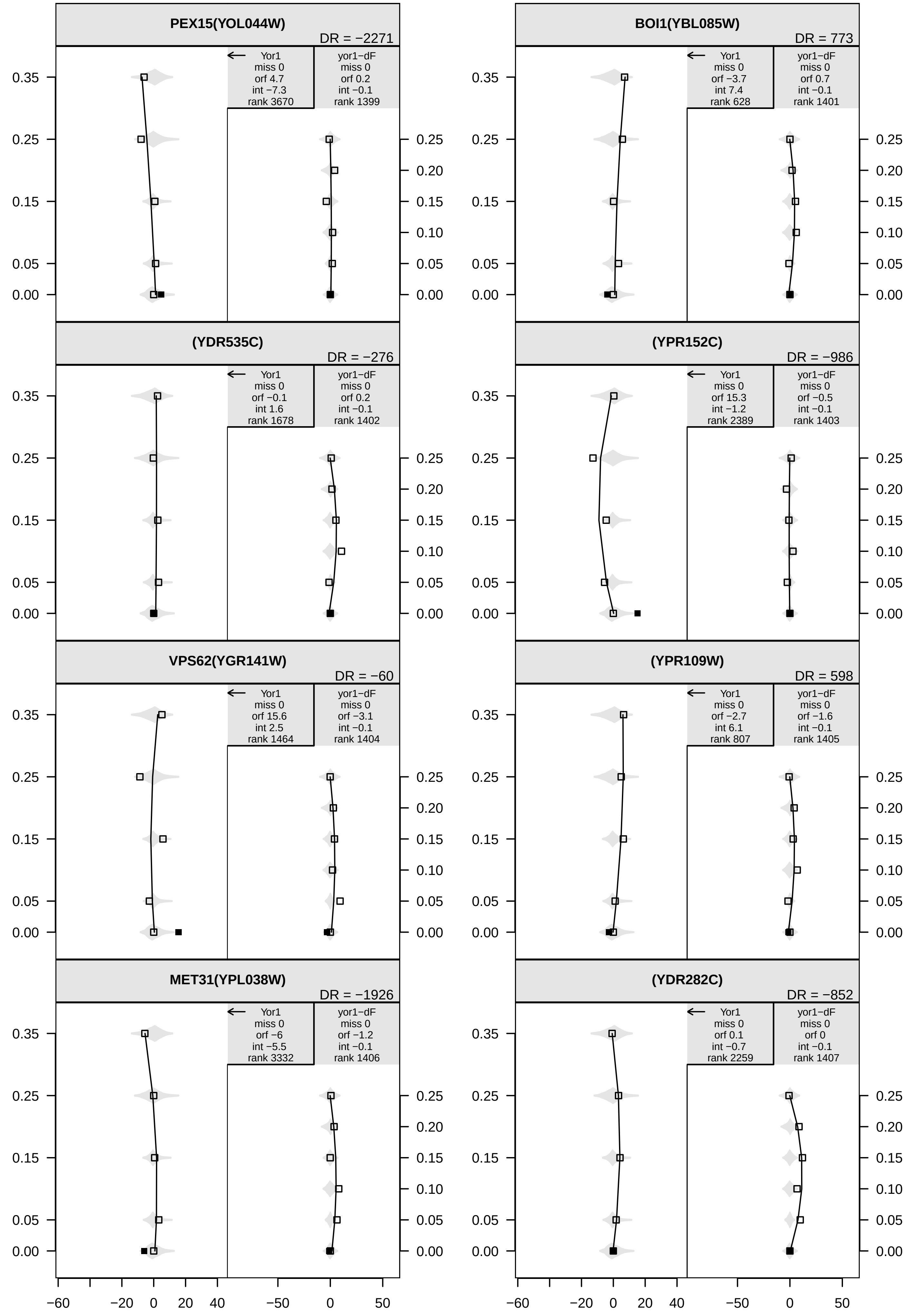
PEX15(YOL044W)
DR = −2271
Yor1
miss 0
orf 4.7
int −7.3
rank 3670
yor1−dF
miss 0
orf 0.2
int −0.1
rank 1399
BOI1(YBL085W)
DR = 773
Yor1
miss 0
orf −3.7
int 7.4
rank 628
yor1−dF
miss 0
orf 0.7
int −0.1
rank 1401
(YDR535C)
DR = −276
Yor1
miss 0
orf −0.1
int 1.6
rank 1678
yor1−dF
miss 0
orf 0.2
int −0.1
rank 1402
(YPR152C)
DR = −986
Yor1
miss 0
orf 15.3
int −1.2
rank 2389
yor1−dF
miss 0
orf −0.5
int −0.1
rank 1403
VPS62(YGR141W)
DR = −60
Yor1
miss 0
orf 15.6
int 2.5
rank 1464
yor1−dF
miss 0
orf −3.1
int −0.1
rank 1404
(YPR109W)
DR = 598
Yor1
miss 0
orf −2.7
int 6.1
rank 807
yor1−dF
miss 0
orf −1.6
int −0.1
rank 1405
MET31(YPL038W)
DR = −1926
Yor1
miss 0
orf −6
int −5.5
rank 3332
yor1−dF
miss 0
orf −1.2
int −0.1
rank 1406
(YDR282C)
DR = −852
Yor1
miss 0
orf 0.1
int −0.7
rank 2259
yor1−dF
miss 0
orf 0
int −0.1
rank 1407
0.35
0.25
0.20
0.15
0.10
0.05
0.00
−60
−20
0
20
40
−50
0
50

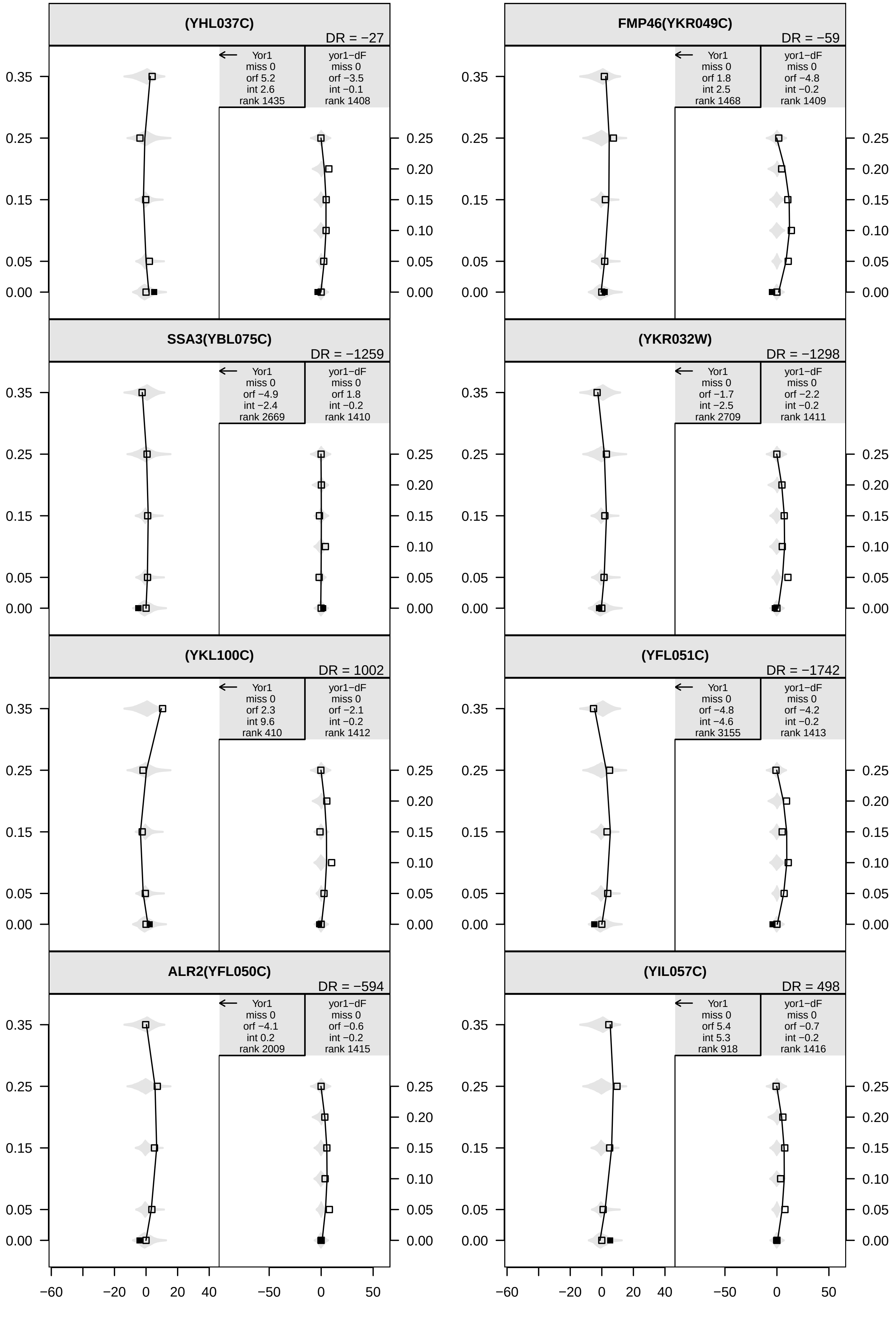
(YHL037C)
DR = −27
Yor1 miss 0 orf 5.2 int 2.6 rank 1435
yor1−dF miss 0 orf −3.5 int −0.1 rank 1408
FMP46(YKR049C)
DR = −59
Yor1 miss 0 orf 1.8 int 2.5 rank 1468
yor1−dF miss 0 orf −4.8 int −0.2 rank 1409
SSA3(YBL075C)
DR = −1259
Yor1 miss 0 orf −4.9 int −2.4 rank 2669
yor1−dF miss 0 orf 1.8 int −0.2 rank 1410
(YKR032W)
DR = −1298
Yor1 miss 0 orf −1.7 int −2.5 rank 2709
yor1−dF miss 0 orf −2.2 int −0.2 rank 1411
(YKL100C)
DR = 1002
Yor1 miss 0 orf 2.3 int 9.6 rank 410
yor1−dF miss 0 orf −2.1 int −0.2 rank 1412
(YFL051C)
DR = −1742
Yor1 miss 0 orf −4.8 int −4.6 rank 3155
yor1−dF miss 0 orf −4.2 int −0.2 rank 1413
ALR2(YFL050C)
DR = −594
Yor1 miss 0 orf −4.1 int 0.2 rank 2009
yor1−dF miss 0 orf −0.6 int −0.2 rank 1415
(YIL057C)
DR = 498
Yor1 miss 0 orf 5.4 int 5.3 rank 918
yor1−dF miss 0 orf −0.7 int −0.2 rank 1416
0.00 0.05 0.15 0.25 0.35
0.00 0.05 0.10 0.15 0.20 0.25
−60 −20 0 20 40
−50 0 50

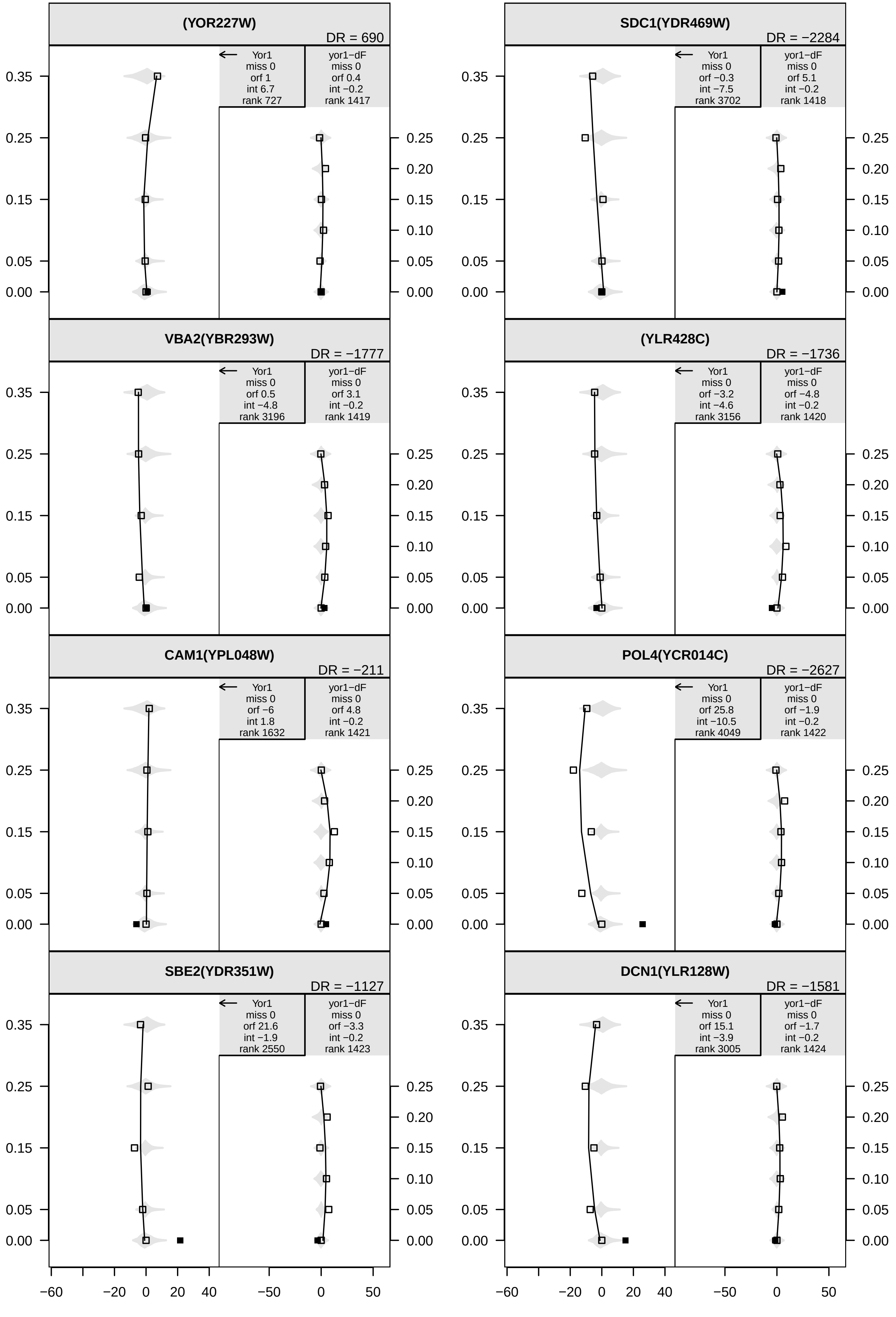

(YOR227W)
DR = 690
Yor1 miss 0 orf 1 int 6.7 rank 727
yor1−dF miss 0 orf 0.4 int −0.2 rank 1417
SDC1(YDR469W)
DR = −2284
Yor1 miss 0 orf −0.3 int −7.5 rank 3702
yor1−dF miss 0 orf 5.1 int −0.2 rank 1418
VBA2(YBR293W)
DR = −1777
Yor1 miss 0 orf 0.5 int −4.8 rank 3196
yor1−dF miss 0 orf 3.1 int −0.2 rank 1419
(YLR428C)
DR = −1736
Yor1 miss 0 orf −3.2 int −4.6 rank 3156
yor1−dF miss 0 orf −4.8 int −0.2 rank 1420
CAM1(YPL048W)
DR = −211
Yor1 miss 0 orf −6 int 1.8 rank 1632
yor1−dF miss 0 orf 4.8 int −0.2 rank 1421
POL4(YCR014C)
DR = −2627
Yor1 miss 0 orf 25.8 int −10.5 rank 4049
yor1−dF miss 0 orf −1.9 int −0.2 rank 1422
SBE2(YDR351W)
DR = −1127
Yor1 miss 0 orf 21.6 int −1.9 rank 2550
yor1−dF miss 0 orf −3.3 int −0.2 rank 1423
DCN1(YLR128W)
DR = −1581
Yor1 miss 0 orf 15.1 int −3.9 rank 3005
yor1−dF miss 0 orf −1.7 int −0.2 rank 1424

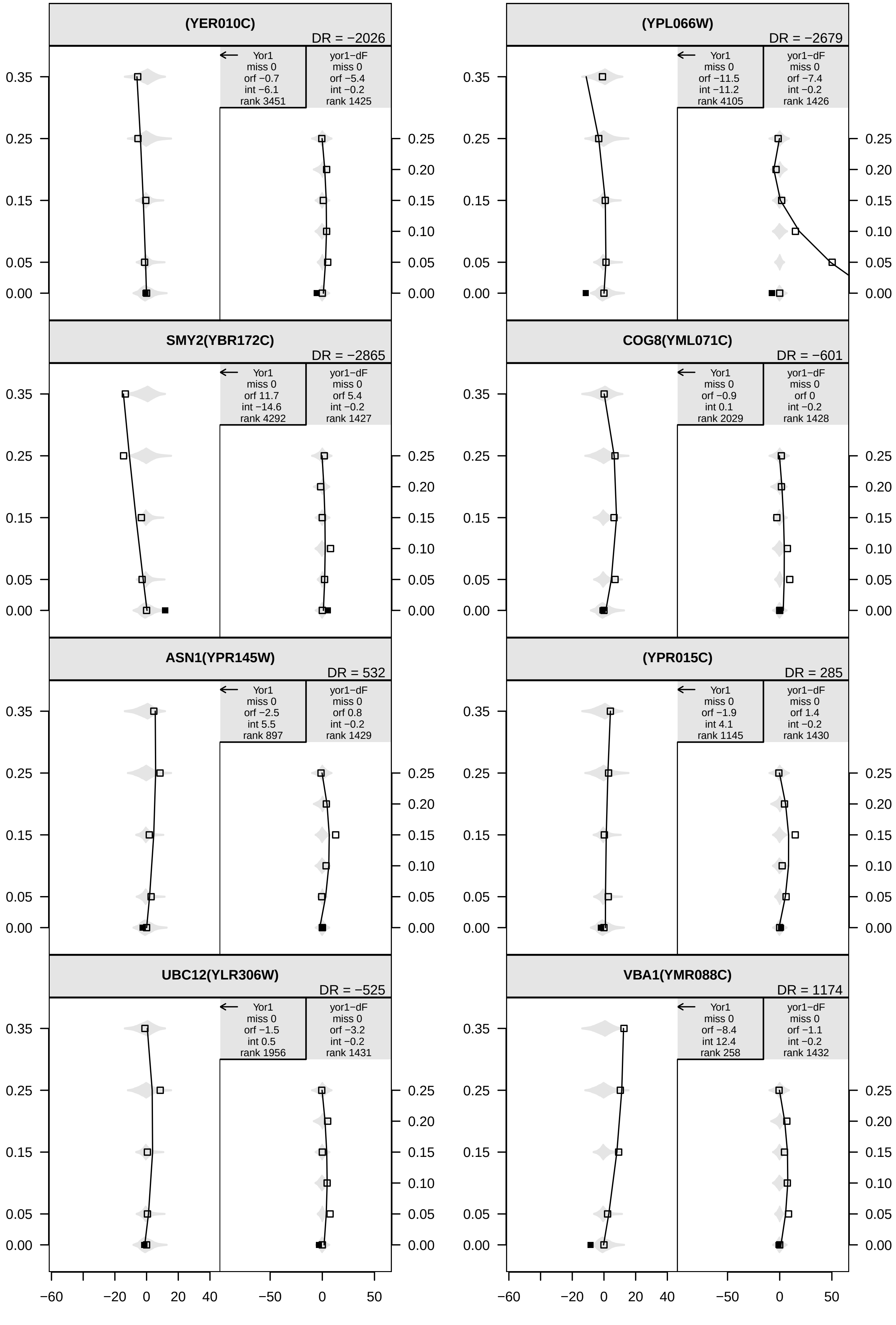

(YER010C)
DR = −2026
Yor1
miss 0
orf −0.7
int −6.1
rank 3451
yor1−dF
miss 0
orf −5.4
int −0.2
rank 1425
(YPL066W)
DR = −2679
Yor1
miss 0
orf −11.5
int −11.2
rank 4105
yor1−dF
miss 0
orf −7.4
int −0.2
rank 1426
SMY2(YBR172C)
DR = −2865
Yor1
miss 0
orf 11.7
int −14.6
rank 4292
yor1−dF
miss 0
orf 5.4
int −0.2
rank 1427
COG8(YML071C)
DR = −601
Yor1
miss 0
orf −0.9
int 0.1
rank 2029
yor1−dF
miss 0
orf 0
int −0.2
rank 1428
ASN1(YPR145W)
DR = 532
Yor1
miss 0
orf −2.5
int 5.5
rank 897
yor1−dF
miss 0
orf 0.8
int −0.2
rank 1429
(YPR015C)
DR = 285
Yor1
miss 0
orf −1.9
int 4.1
rank 1145
yor1−dF
miss 0
orf 1.4
int −0.2
rank 1430
UBC12(YLR306W)
DR = −525
Yor1
miss 0
orf −1.5
int 0.5
rank 1956
yor1−dF
miss 0
orf −3.2
int −0.2
rank 1431
VBA1(YMR088C)
DR = 1174
Yor1
miss 0
orf −8.4
int 12.4
rank 258
yor1−dF
miss 0
orf −1.1
int −0.2
rank 1432

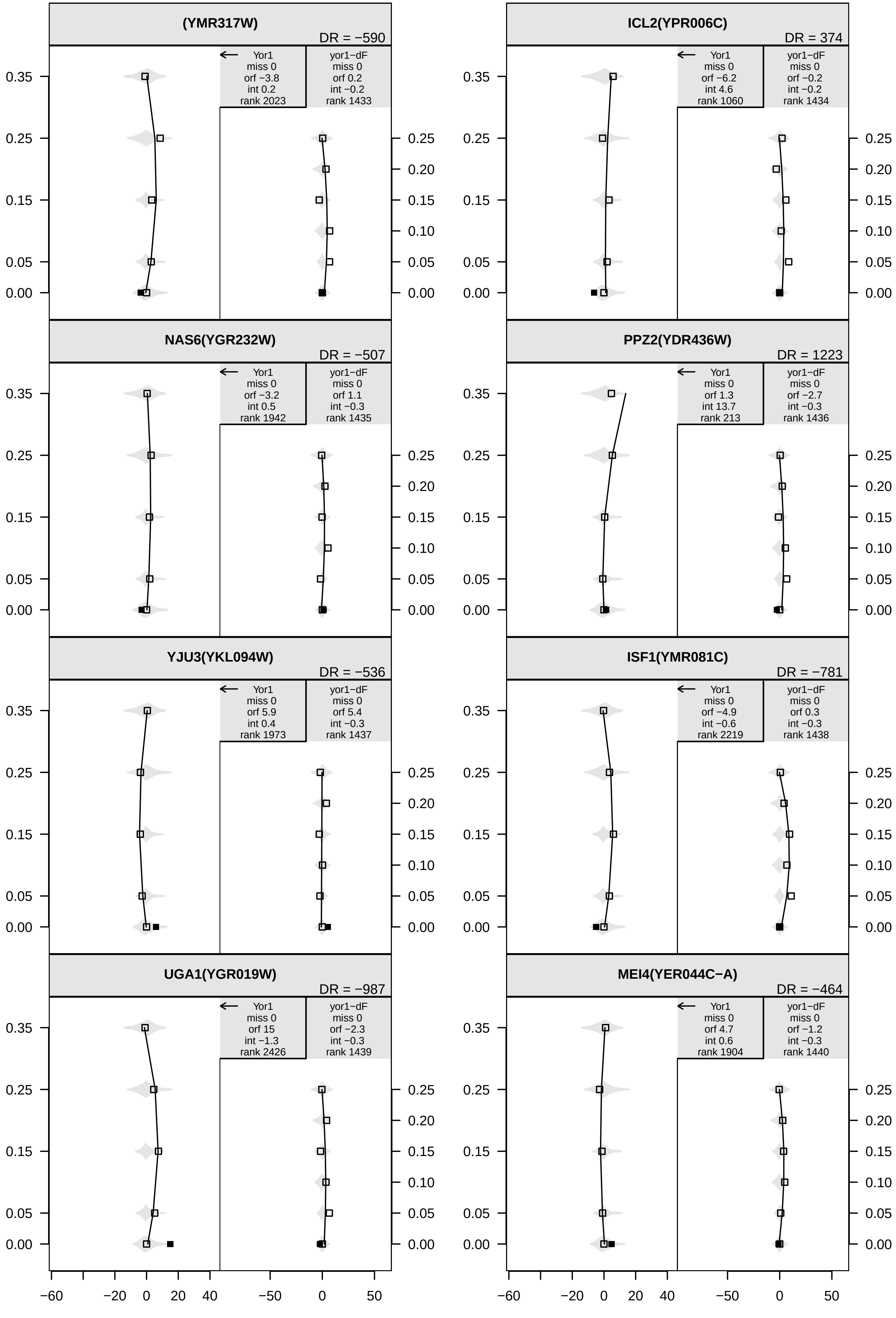

(YMR317W)
DR = −590
Yor1 miss 0 orf −3.8 int 0.2 rank 2023
yor1−dF miss 0 orf 0.2 int −0.2 rank 1433
ICL2(YPR006C)
DR = 374
Yor1 miss 0 orf −6.2 int 4.6 rank 1060
yor1−dF miss 0 orf −0.2 int −0.2 rank 1434
NAS6(YGR232W)
DR = −507
Yor1 miss 0 orf −3.2 int 0.5 rank 1942
yor1−dF miss 0 orf 1.1 int −0.3 rank 1435
PPZ2(YDR436W)
DR = 1223
Yor1 miss 0 orf 1.3 int 13.7 rank 213
yor1−dF miss 0 orf −2.7 int −0.3 rank 1436
YJU3(YKL094W)
DR = −536
Yor1 miss 0 orf 5.9 int 0.4 rank 1973
yor1−dF miss 0 orf 5.4 int −0.3 rank 1437
ISF1(YMR081C)
DR = −781
Yor1 miss 0 orf −4.9 int −0.6 rank 2219
yor1−dF miss 0 orf 0.3 int −0.3 rank 1438
UGA1(YGR019W)
DR = −987
Yor1 miss 0 orf 15 int −1.3 rank 2426
yor1−dF miss 0 orf −2.3 int −0.3 rank 1439
MEI4(YER044C−A)
DR = −464
Yor1 miss 0 orf 4.7 int 0.6 rank 1904
yor1−dF miss 0 orf −1.2 int −0.3 rank 1440
0.35
0.25
0.20
0.15
0.10
0.05
0.00
−60
−20
0
20
40
−50
0
50

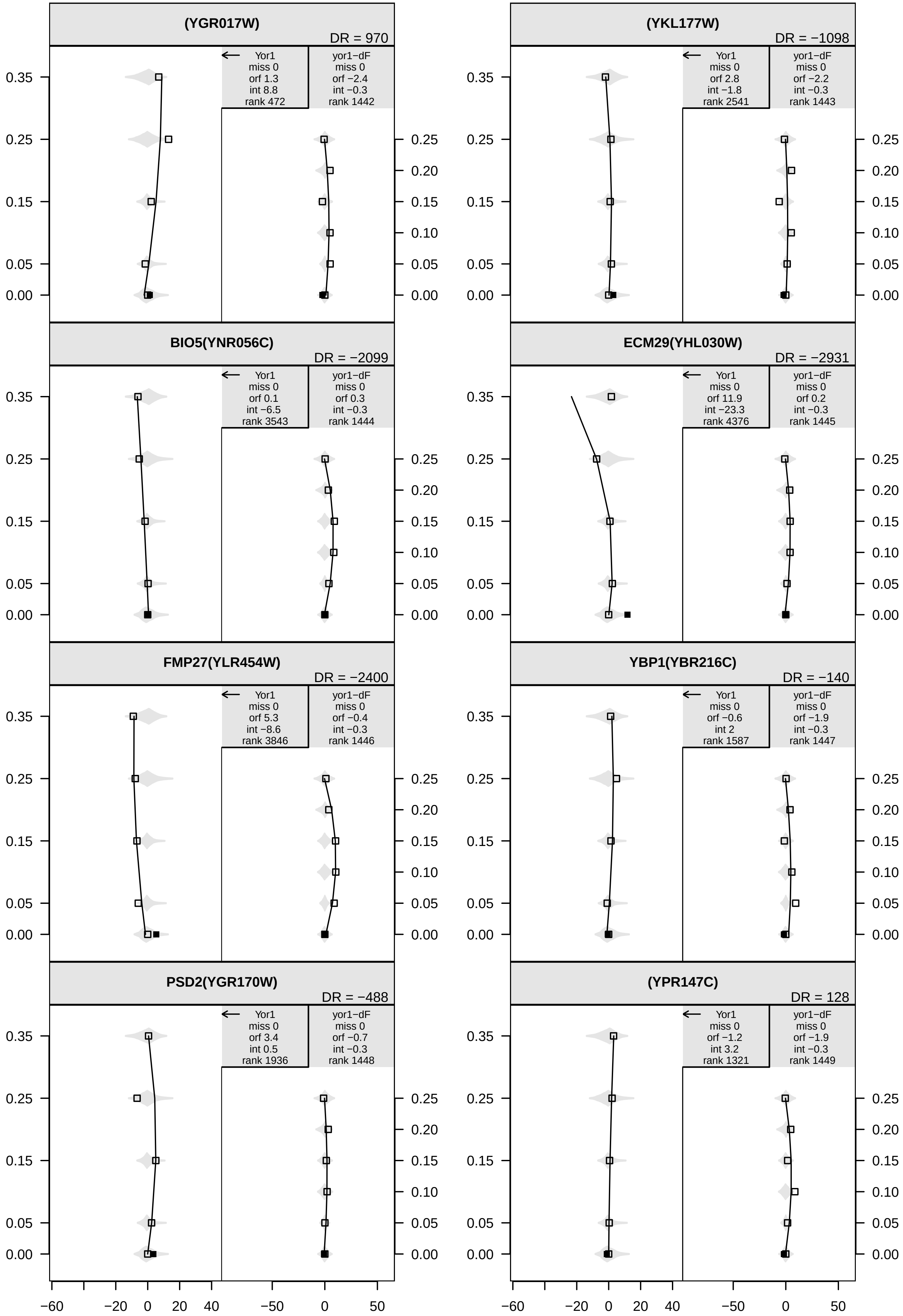

(YGR017W)
DR = 970
Yor1 miss 0 orf 1.3 int 8.8 rank 472
yor1−dF miss 0 orf −2.4 int −0.3 rank 1442
(YKL177W)
DR = −1098
Yor1 miss 0 orf 2.8 int −1.8 rank 2541
yor1−dF miss 0 orf −2.2 int −0.3 rank 1443
BIO5(YNR056C)
DR = −2099
Yor1 miss 0 orf 0.1 int −6.5 rank 3543
yor1−dF miss 0 orf 0.3 int −0.3 rank 1444
ECM29(YHL030W)
DR = −2931
Yor1 miss 0 orf 11.9 int −23.3 rank 4376
yor1−dF miss 0 orf 0.2 int −0.3 rank 1445
FMP27(YLR454W)
DR = −2400
Yor1 miss 0 orf 5.3 int −8.6 rank 3846
yor1−dF miss 0 orf −0.4 int −0.3 rank 1446
YBP1(YBR216C)
DR = −140
Yor1 miss 0 orf −0.6 int 2 rank 1587
yor1−dF miss 0 orf −1.9 int −0.3 rank 1447
PSD2(YGR170W)
DR = −488
Yor1 miss 0 orf 3.4 int 0.5 rank 1936
yor1−dF miss 0 orf −0.7 int −0.3 rank 1448
(YPR147C)
DR = 128
Yor1 miss 0 orf −1.2 int 3.2 rank 1321
yor1−dF miss 0 orf −1.9 int −0.3 rank 1449
0.35
0.25
0.20
0.15
0.10
0.05
0.00
−60
−20
0
20
40
−50
0
50

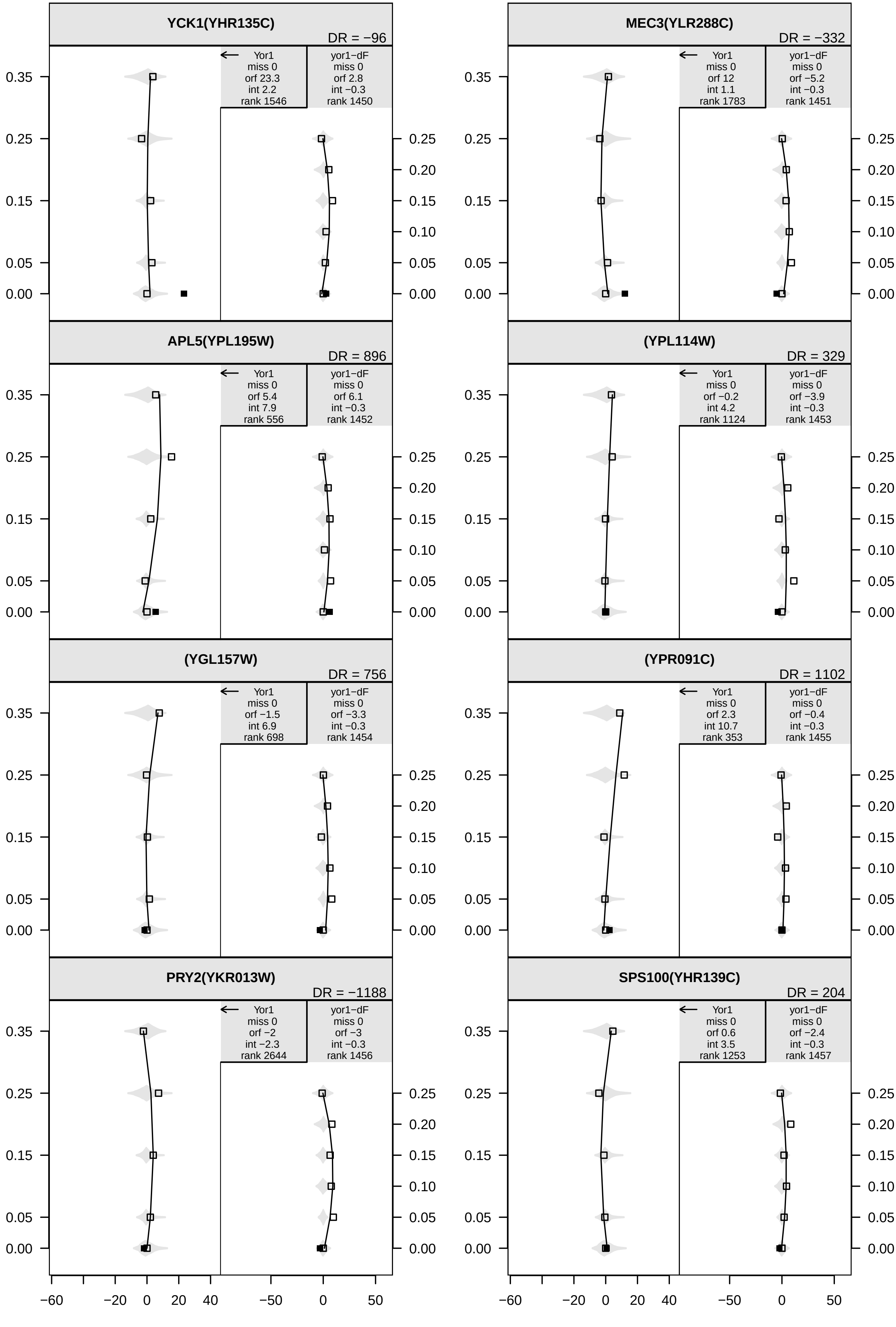

YCK1(YHR135C)
DR = −96
Yor1 miss 0 orf 23.3 int 2.2 rank 1546
yor1−dF miss 0 orf 2.8 int −0.3 rank 1450
MEC3(YLR288C)
DR = −332
Yor1 miss 0 orf 12 int 1.1 rank 1783
yor1−dF miss 0 orf −5.2 int −0.3 rank 1451
APL5(YPL195W)
DR = 896
Yor1 miss 0 orf 5.4 int 7.9 rank 556
yor1−dF miss 0 orf 6.1 int −0.3 rank 1452
(YPL114W)
DR = 329
Yor1 miss 0 orf −0.2 int 4.2 rank 1124
yor1−dF miss 0 orf −3.9 int −0.3 rank 1453
(YGL157W)
DR = 756
Yor1 miss 0 orf −1.5 int 6.9 rank 698
yor1−dF miss 0 orf −3.3 int −0.3 rank 1454
(YPR091C)
DR = 1102
Yor1 miss 0 orf 2.3 int 10.7 rank 353
yor1−dF miss 0 orf −0.4 int −0.3 rank 1455
PRY2(YKR013W)
DR = −1188
Yor1 miss 0 orf −2 int −2.3 rank 2644
yor1−dF miss 0 orf −3 int −0.3 rank 1456
SPS100(YHR139C)
DR = 204
Yor1 miss 0 orf 0.6 int 3.5 rank 1253
yor1−dF miss 0 orf −2.4 int −0.3 rank 1457
0.35 0.25 0.15 0.05 0.00
0.25 0.20 0.15 0.10 0.05 0.00
−60 −20 0 20 40 −50 0 50

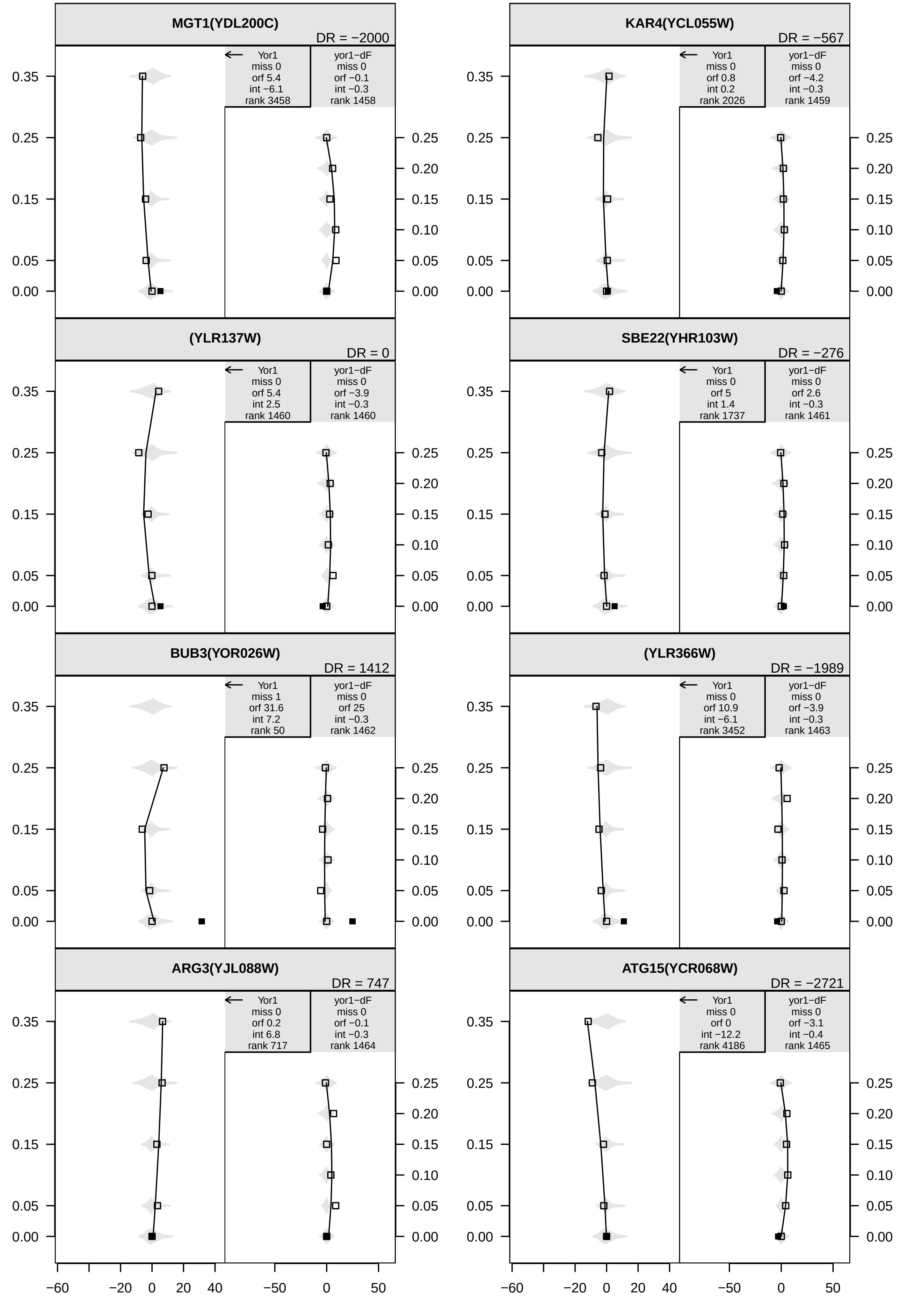

MGT1(YDL200C)
DR = −2000
Yor1 miss 0 orf 5.4 int −6.1 rank 3458
yor1−dF miss 0 orf −0.1 int −0.3 rank 1458
KAR4(YCL055W)
DR = −567
Yor1 miss 0 orf 0.8 int 0.2 rank 2026
yor1−dF miss 0 orf −4.2 int −0.3 rank 1459
(YLR137W)
DR = 0
Yor1 miss 0 orf 5.4 int 2.5 rank 1460
yor1−dF miss 0 orf −3.9 int −0.3 rank 1460
SBE22(YHR103W)
DR = −276
Yor1 miss 0 orf 5 int 1.4 rank 1737
yor1−dF miss 0 orf 2.6 int −0.3 rank 1461
BUB3(YOR026W)
DR = 1412
Yor1 miss 1 orf 31.6 int 7.2 rank 50
yor1−dF miss 0 orf 25 int −0.3 rank 1462
(YLR366W)
DR = −1989
Yor1 miss 0 orf 10.9 int −6.1 rank 3452
yor1−dF miss 0 orf −3.9 int −0.3 rank 1463
ARG3(YJL088W)
DR = 747
Yor1 miss 0 orf 0.2 int 6.8 rank 717
yor1−dF miss 0 orf −0.1 int −0.3 rank 1464
ATG15(YCR068W)
DR = −2721
Yor1 miss 0 orf 0 int −12.2 rank 4186
yor1−dF miss 0 orf −3.1 int −0.4 rank 1465
0.00 0.05 0.10 0.15 0.20 0.25 0.35
−60 −20 0 20 40 −50 0 50

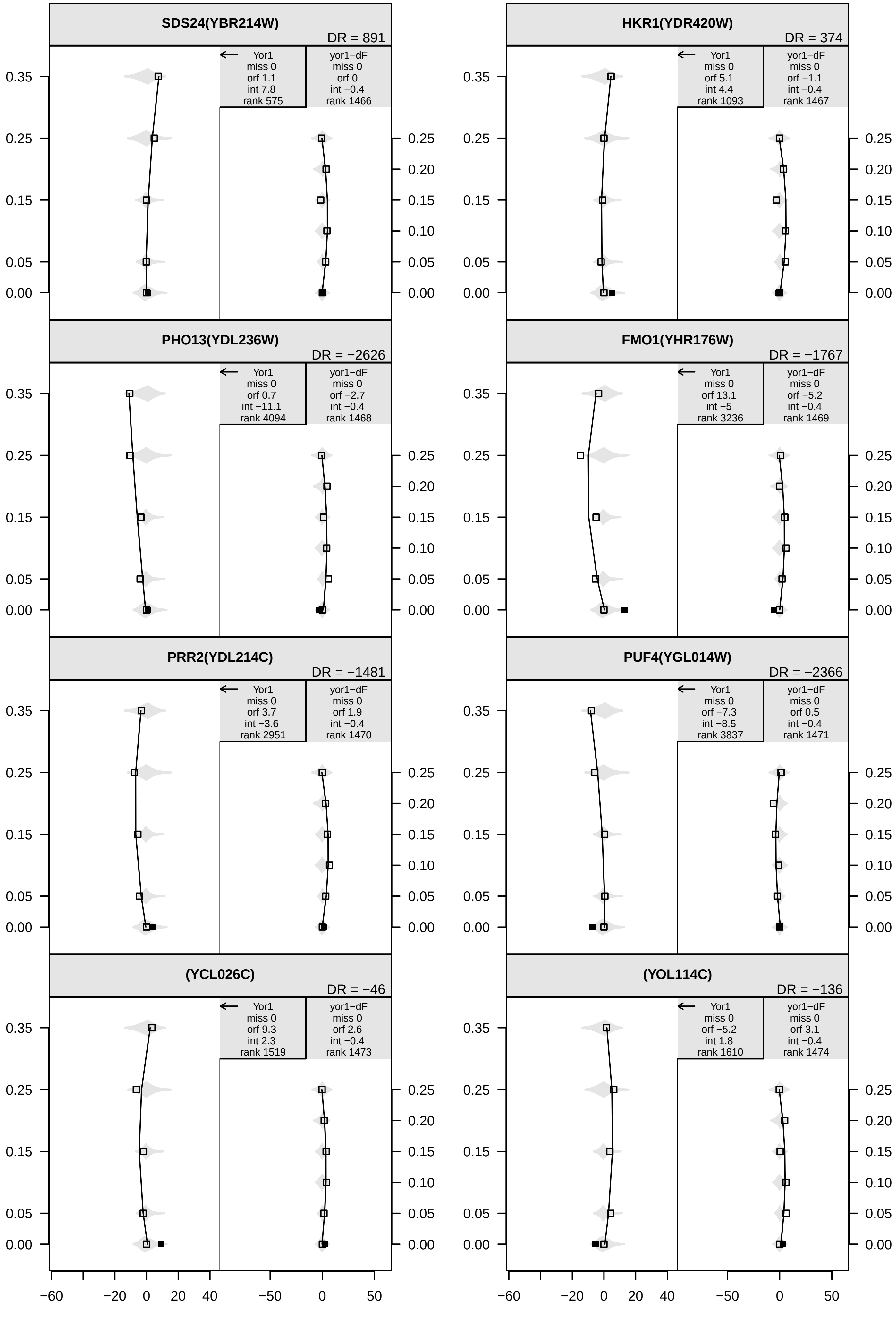

SDS24(YBR214W)
DR = 891
Yor1 miss 0 orf 1.1 int 7.8 rank 575
yor1−dF miss 0 orf 0 int −0.4 rank 1466
HKR1(YDR420W)
DR = 374
Yor1 miss 0 orf 5.1 int 4.4 rank 1093
yor1−dF miss 0 orf −1.1 int −0.4 rank 1467
PHO13(YDL236W)
DR = −2626
Yor1 miss 0 orf 0.7 int −11.1 rank 4094
yor1−dF miss 0 orf −2.7 int −0.4 rank 1468
FMO1(YHR176W)
DR = −1767
Yor1 miss 0 orf 13.1 int −5 rank 3236
yor1−dF miss 0 orf −5.2 int −0.4 rank 1469
PRR2(YDL214C)
DR = −1481
Yor1 miss 0 orf 3.7 int −3.6 rank 2951
yor1−dF miss 0 orf 1.9 int −0.4 rank 1470
PUF4(YGL014W)
DR = −2366
Yor1 miss 0 orf −7.3 int −8.5 rank 3837
yor1−dF miss 0 orf 0.5 int −0.4 rank 1471
(YCL026C)
DR = −46
Yor1 miss 0 orf 9.3 int 2.3 rank 1519
yor1−dF miss 0 orf 2.6 int −0.4 rank 1473
(YOL114C)
DR = −136
Yor1 miss 0 orf −5.2 int 1.8 rank 1610
yor1−dF miss 0 orf 3.1 int −0.4 rank 1474
0.00 0.05 0.10 0.15 0.20 0.25 0.35
−60 −20 0 20 40 −50 0 50

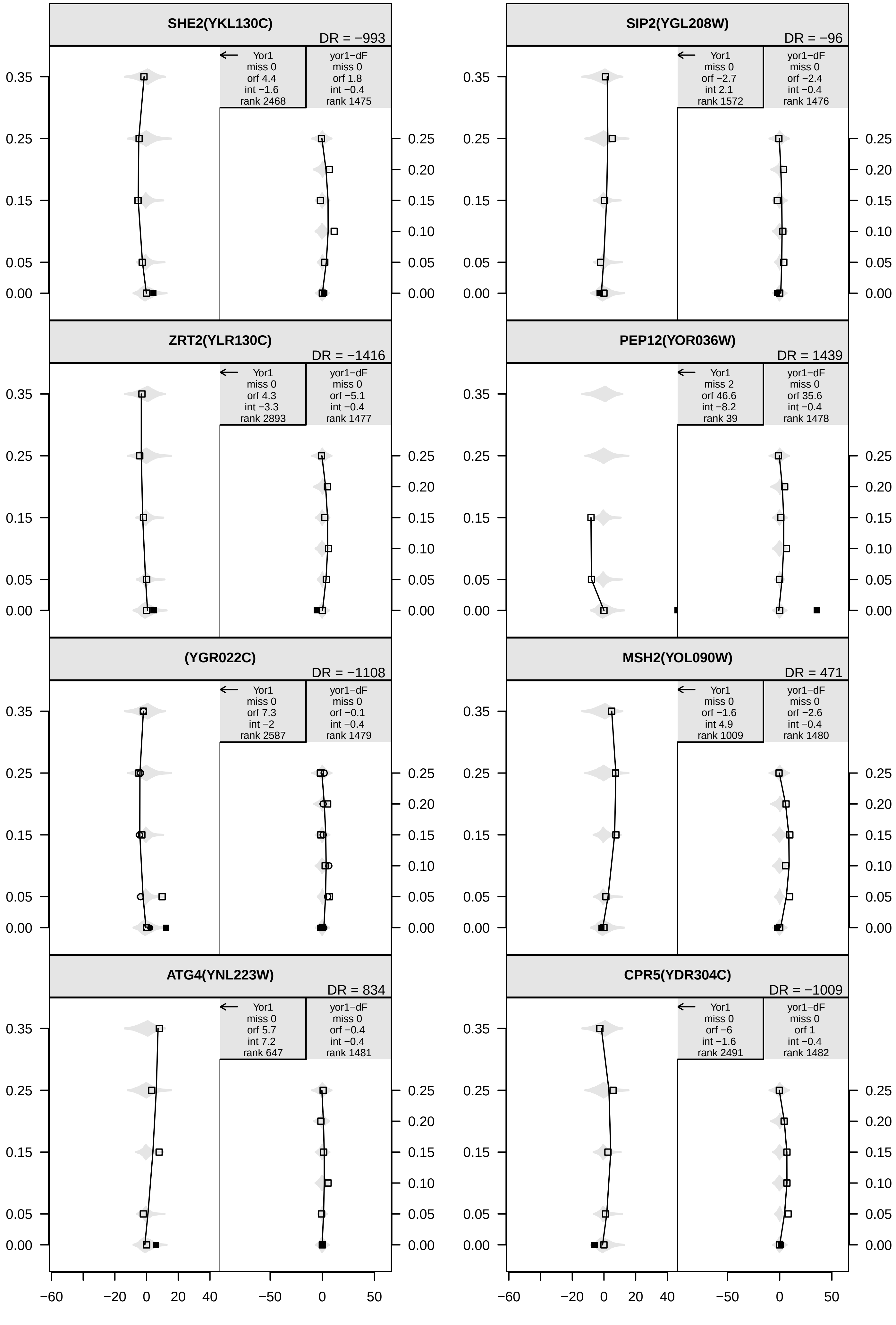

SHE2(YKL130C)
DR = −993
Yor1 miss 0 orf 4.4 int −1.6 rank 2468
yor1−dF miss 0 orf 1.8 int −0.4 rank 1475
SIP2(YGL208W)
DR = −96
Yor1 miss 0 orf −2.7 int 2.1 rank 1572
yor1−dF miss 0 orf −2.4 int −0.4 rank 1476
ZRT2(YLR130C)
DR = −1416
Yor1 miss 0 orf 4.3 int −3.3 rank 2893
yor1−dF miss 0 orf −5.1 int −0.4 rank 1477
PEP12(YOR036W)
DR = 1439
Yor1 miss 2 orf 46.6 int −8.2 rank 39
yor1−dF miss 0 orf 35.6 int −0.4 rank 1478
(YGR022C)
DR = −1108
Yor1 miss 0 orf 7.3 int −2 rank 2587
yor1−dF miss 0 orf −0.1 int −0.4 rank 1479
MSH2(YOL090W)
DR = 471
Yor1 miss 0 orf −1.6 int 4.9 rank 1009
yor1−dF miss 0 orf −2.6 int −0.4 rank 1480
ATG4(YNL223W)
DR = 834
Yor1 miss 0 orf 5.7 int 7.2 rank 647
yor1−dF miss 0 orf −0.4 int −0.4 rank 1481
CPR5(YDR304C)
DR = −1009
Yor1 miss 0 orf −6 int −1.6 rank 2491
yor1−dF miss 0 orf 1 int −0.4 rank 1482
0.00 0.05 0.10 0.15 0.20 0.25 0.35
−60 −20 0 20 40 −50 0 50

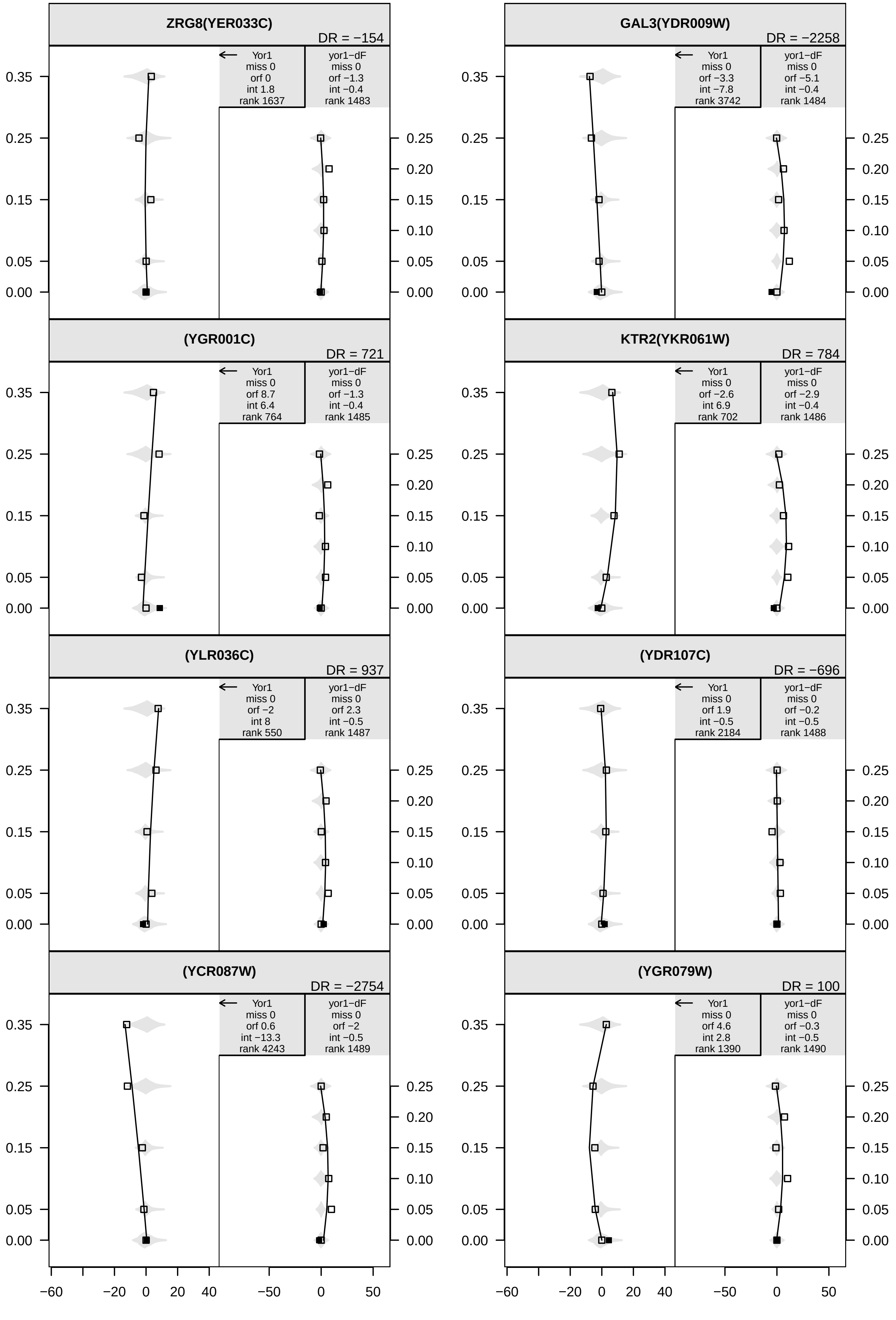

ZRG8(YER033C)
DR = −154
Yor1 miss 0 orf 0 int 1.8 rank 1637
yor1−dF miss 0 orf −1.3 int −0.4 rank 1483
GAL3(YDR009W)
DR = −2258
Yor1 miss 0 orf −3.3 int −7.8 rank 3742
yor1−dF miss 0 orf −5.1 int −0.4 rank 1484
(YGR001C)
DR = 721
Yor1 miss 0 orf 8.7 int 6.4 rank 764
yor1−dF miss 0 orf −1.3 int −0.4 rank 1485
KTR2(YKR061W)
DR = 784
Yor1 miss 0 orf −2.6 int 6.9 rank 702
yor1−dF miss 0 orf −2.9 int −0.4 rank 1486
(YLR036C)
DR = 937
Yor1 miss 0 orf −2 int 8 rank 550
yor1−dF miss 0 orf 2.3 int −0.5 rank 1487
(YDR107C)
DR = −696
Yor1 miss 0 orf 1.9 int −0.5 rank 2184
yor1−dF miss 0 orf −0.2 int −0.5 rank 1488
(YCR087W)
DR = −2754
Yor1 miss 0 orf 0.6 int −13.3 rank 4243
yor1−dF miss 0 orf −2 int −0.5 rank 1489
(YGR079W)
DR = 100
Yor1 miss 0 orf 4.6 int 2.8 rank 1390
yor1−dF miss 0 orf −0.3 int −0.5 rank 1490
0.35 0.25 0.15 0.05 0.00
0.25 0.20 0.15 0.10 0.05 0.00
−60 −20 0 20 40 −50 0 50

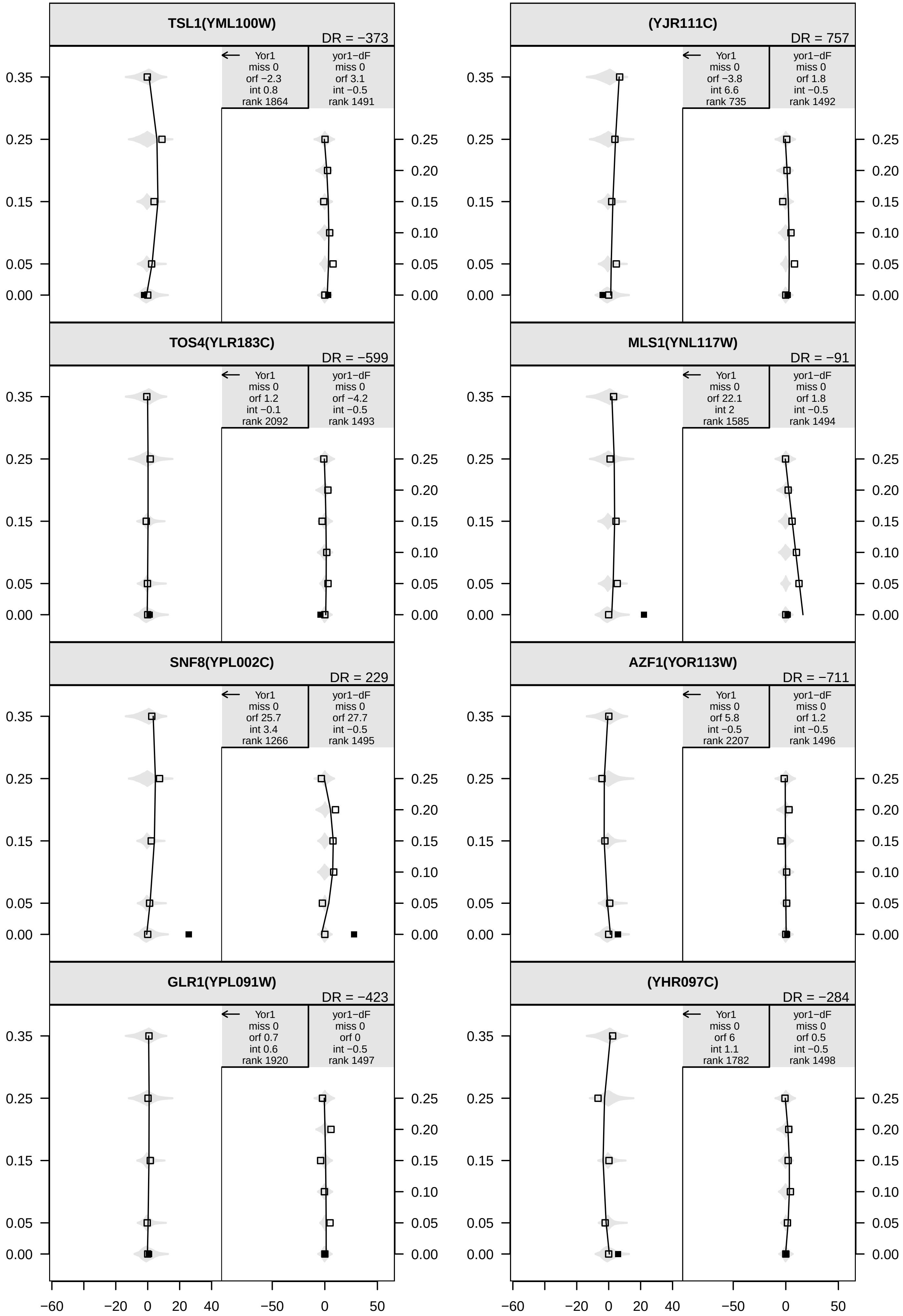

TSL1(YML100W)
DR = −373
Yor1 miss 0 orf −2.3 int 0.8 rank 1864
yor1−dF miss 0 orf 3.1 int −0.5 rank 1491
(YJR111C)
DR = 757
Yor1 miss 0 orf −3.8 int 6.6 rank 735
yor1−dF miss 0 orf 1.8 int −0.5 rank 1492
TOS4(YLR183C)
DR = −599
Yor1 miss 0 orf 1.2 int −0.1 rank 2092
yor1−dF miss 0 orf −4.2 int −0.5 rank 1493
MLS1(YNL117W)
DR = −91
Yor1 miss 0 orf 22.1 int 2 rank 1585
yor1−dF miss 0 orf 1.8 int −0.5 rank 1494
SNF8(YPL002C)
DR = 229
Yor1 miss 0 orf 25.7 int 3.4 rank 1266
yor1−dF miss 0 orf 27.7 int −0.5 rank 1495
AZF1(YOR113W)
DR = −711
Yor1 miss 0 orf 5.8 int −0.5 rank 2207
yor1−dF miss 0 orf 1.2 int −0.5 rank 1496
GLR1(YPL091W)
DR = −423
Yor1 miss 0 orf 0.7 int 0.6 rank 1920
yor1−dF miss 0 orf 0 int −0.5 rank 1497
(YHR097C)
DR = −284
Yor1 miss 0 orf 6 int 1.1 rank 1782
yor1−dF miss 0 orf 0.5 int −0.5 rank 1498
0.00 0.05 0.10 0.15 0.20 0.25 0.35
−60 −20 0 20 40 −50 0 50

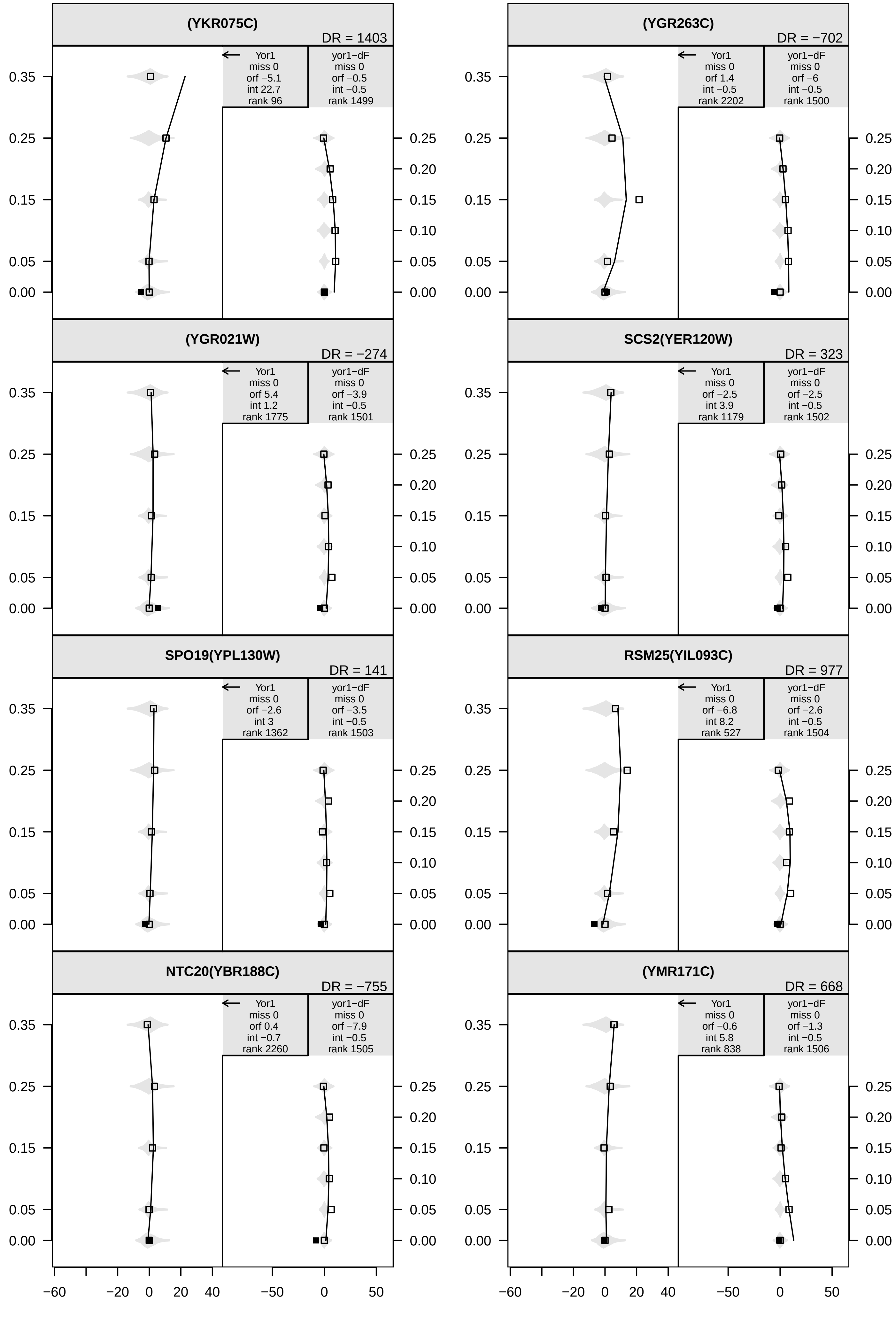
(YKR075C)
DR = 1403
Yor1
miss 0
orf −5.1
int 22.7
rank 96
yor1−dF
miss 0
orf −0.5
int −0.5
rank 1499
(YGR263C)
DR = −702
Yor1
miss 0
orf 1.4
int −0.5
rank 2202
yor1−dF
miss 0
orf −6
int −0.5
rank 1500
(YGR021W)
DR = −274
Yor1
miss 0
orf 5.4
int 1.2
rank 1775
yor1−dF
miss 0
orf −3.9
int −0.5
rank 1501
SCS2(YER120W)
DR = 323
Yor1
miss 0
orf −2.5
int 3.9
rank 1179
yor1−dF
miss 0
orf −2.5
int −0.5
rank 1502
SPO19(YPL130W)
DR = 141
Yor1
miss 0
orf −2.6
int 3
rank 1362
yor1−dF
miss 0
orf −3.5
int −0.5
rank 1503
RSM25(YIL093C)
DR = 977
Yor1
miss 0
orf −6.8
int 8.2
rank 527
yor1−dF
miss 0
orf −2.6
int −0.5
rank 1504
NTC20(YBR188C)
DR = −755
Yor1
miss 0
orf 0.4
int −0.7
rank 2260
yor1−dF
miss 0
orf −7.9
int −0.5
rank 1505
(YMR171C)
DR = 668
Yor1
miss 0
orf −0.6
int 5.8
rank 838
yor1−dF
miss 0
orf −1.3
int −0.5
rank 1506
0.35
0.25
0.20
0.15
0.10
0.05
0.00
−60
−20
0
20
40
−50
0
50

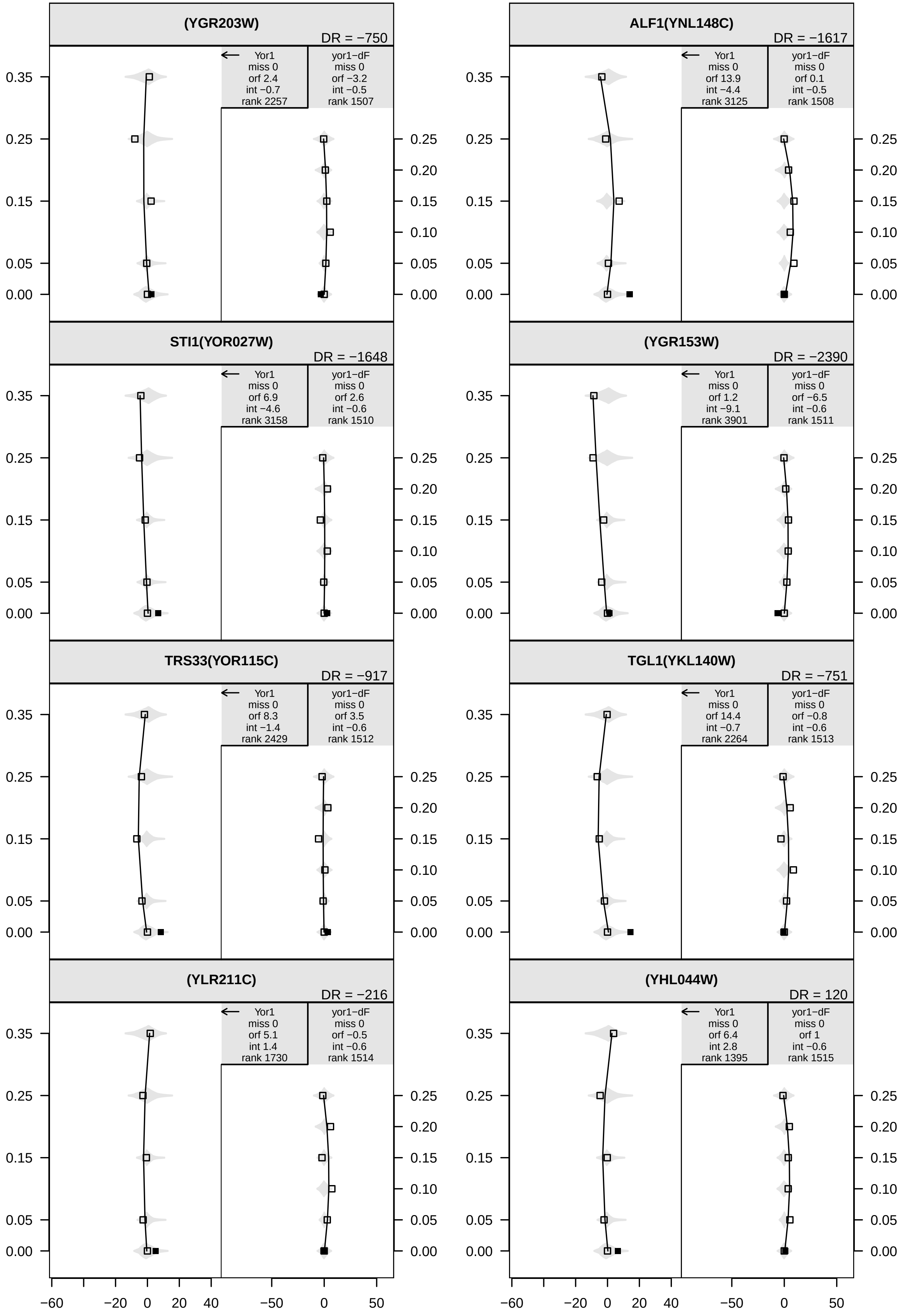

(YGR203W)
DR = −750
Yor1
miss 0
orf 2.4
int −0.7
rank 2257
yor1−dF
miss 0
orf −3.2
int −0.5
rank 1507
ALF1(YNL148C)
DR = −1617
Yor1
miss 0
orf 13.9
int −4.4
rank 3125
yor1−dF
miss 0
orf 0.1
int −0.5
rank 1508
STI1(YOR027W)
DR = −1648
Yor1
miss 0
orf 6.9
int −4.6
rank 3158
yor1−dF
miss 0
orf 2.6
int −0.6
rank 1510
(YGR153W)
DR = −2390
Yor1
miss 0
orf 1.2
int −9.1
rank 3901
yor1−dF
miss 0
orf −6.5
int −0.6
rank 1511
TRS33(YOR115C)
DR = −917
Yor1
miss 0
orf 8.3
int −1.4
rank 2429
yor1−dF
miss 0
orf 3.5
int −0.6
rank 1512
TGL1(YKL140W)
DR = −751
Yor1
miss 0
orf 14.4
int −0.7
rank 2264
yor1−dF
miss 0
orf −0.8
int −0.6
rank 1513
(YLR211C)
DR = −216
Yor1
miss 0
orf 5.1
int 1.4
rank 1730
yor1−dF
miss 0
orf −0.5
int −0.6
rank 1514
(YHL044W)
DR = 120
Yor1
miss 0
orf 6.4
int 2.8
rank 1395
yor1−dF
miss 0
orf 1
int −0.6
rank 1515
0.35
0.25
0.15
0.05
0.00
0.20
0.10
−60
−20
0
20
40
−50
50

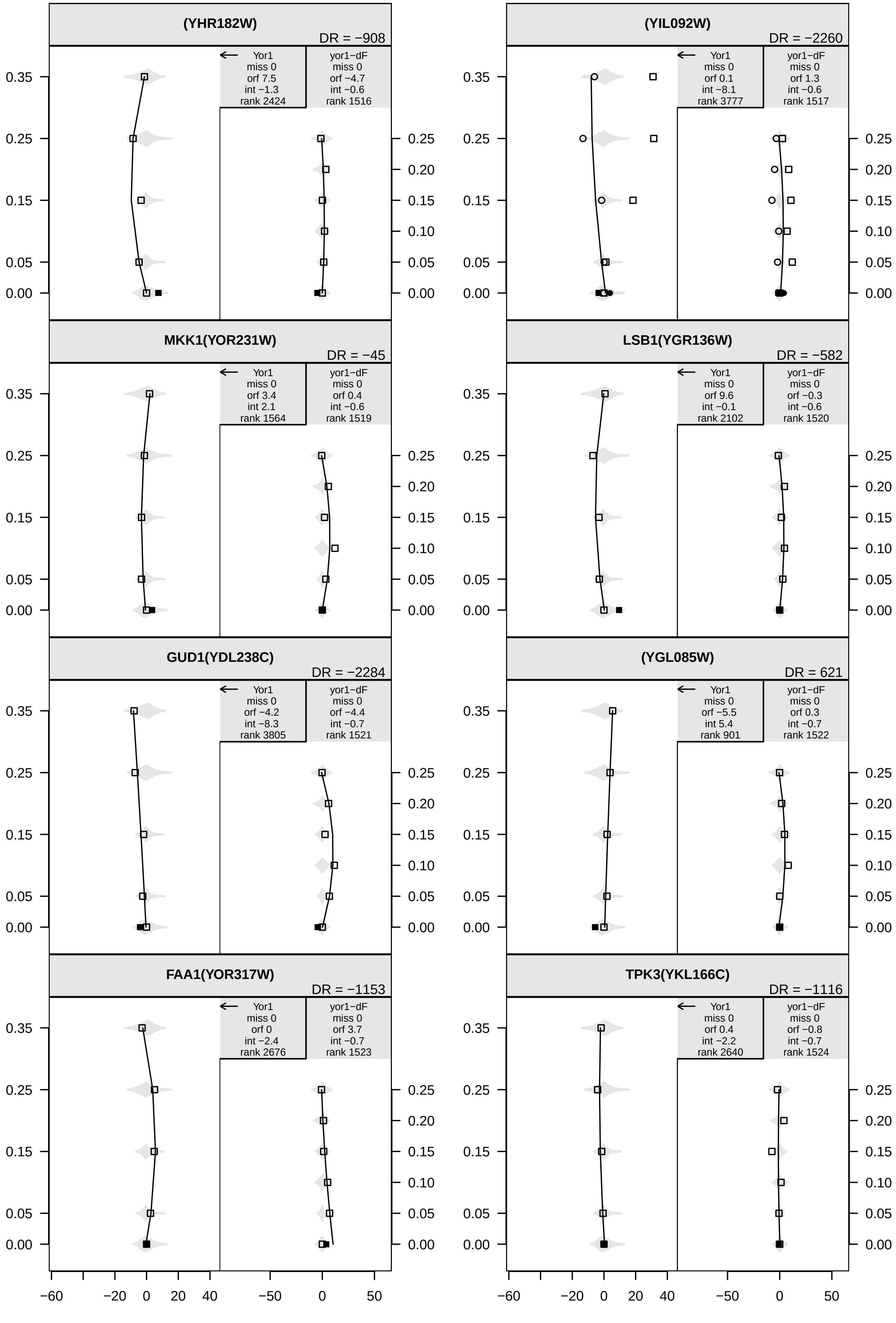
(YHR182W)
DR = −908
Yor1
miss 0
orf 7.5
int −1.3
rank 2424
yor1−dF
miss 0
orf −4.7
int −0.6
rank 1516
(YIL092W)
DR = −2260
Yor1
miss 0
orf 0.1
int −8.1
rank 3777
yor1−dF
miss 0
orf 1.3
int −0.6
rank 1517
MKK1(YOR231W)
DR = −45
Yor1
miss 0
orf 3.4
int 2.1
rank 1564
yor1−dF
miss 0
orf 0.4
int −0.6
rank 1519
LSB1(YGR136W)
DR = −582
Yor1
miss 0
orf 9.6
int −0.1
rank 2102
yor1−dF
miss 0
orf −0.3
int −0.6
rank 1520
GUD1(YDL238C)
DR = −2284
Yor1
miss 0
orf −4.2
int −8.3
rank 3805
yor1−dF
miss 0
orf −4.4
int −0.7
rank 1521
(YGL085W)
DR = 621
Yor1
miss 0
orf −5.5
int 5.4
rank 901
yor1−dF
miss 0
orf 0.3
int −0.7
rank 1522
FAA1(YOR317W)
DR = −1153
Yor1
miss 0
orf 0
int −2.4
rank 2676
yor1−dF
miss 0
orf 3.7
int −0.7
rank 1523
TPK3(YKL166C)
DR = −1116
Yor1
miss 0
orf 0.4
int −2.2
rank 2640
yor1−dF
miss 0
orf −0.8
int −0.7
rank 1524
0.35
0.25
0.20
0.15
0.10
0.05
0.00
−60
−20
0
20
40
−50
0
50

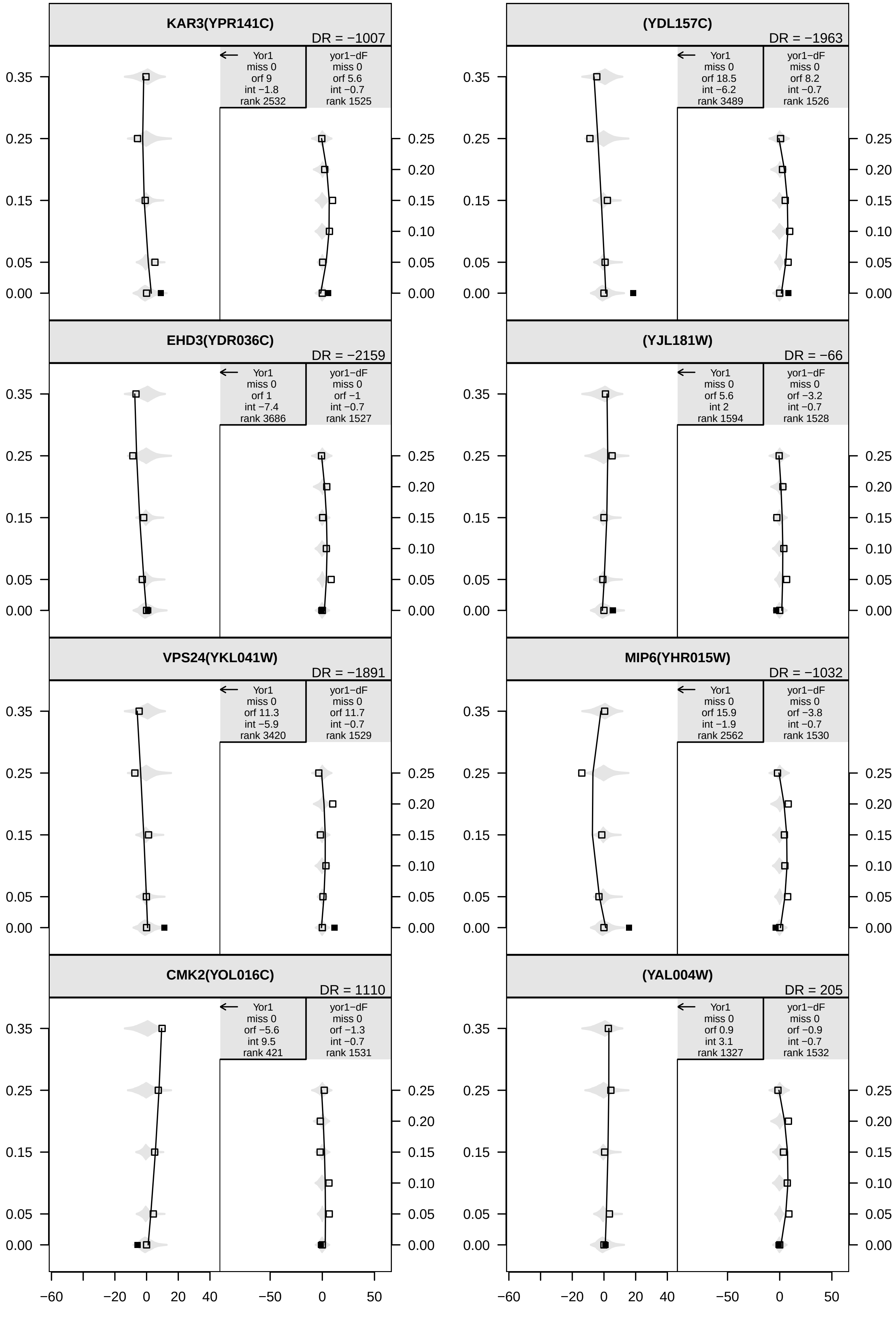
KAR3(YPR141C)
DR = −1007
Yor1 miss 0 orf 9 int −1.8 rank 2532
yor1−dF miss 0 orf 5.6 int −0.7 rank 1525
(YDL157C)
DR = −1963
Yor1 miss 0 orf 18.5 int −6.2 rank 3489
yor1−dF miss 0 orf 8.2 int −0.7 rank 1526
EHD3(YDR036C)
DR = −2159
Yor1 miss 0 orf 1 int −7.4 rank 3686
yor1−dF miss 0 orf −1 int −0.7 rank 1527
(YJL181W)
DR = −66
Yor1 miss 0 orf 5.6 int 2 rank 1594
yor1−dF miss 0 orf −3.2 int −0.7 rank 1528
VPS24(YKL041W)
DR = −1891
Yor1 miss 0 orf 11.3 int −5.9 rank 3420
yor1−dF miss 0 orf 11.7 int −0.7 rank 1529
MIP6(YHR015W)
DR = −1032
Yor1 miss 0 orf 15.9 int −1.9 rank 2562
yor1−dF miss 0 orf −3.8 int −0.7 rank 1530
CMK2(YOL016C)
DR = 1110
Yor1 miss 0 orf −5.6 int 9.5 rank 421
yor1−dF miss 0 orf −1.3 int −0.7 rank 1531
(YAL004W)
DR = 205
Yor1 miss 0 orf 0.9 int 3.1 rank 1327
yor1−dF miss 0 orf −0.9 int −0.7 rank 1532
0.00 0.05 0.15 0.25 0.35
0.00 0.05 0.10 0.15 0.20 0.25
−60 −20 0 20 40 −50 0 50

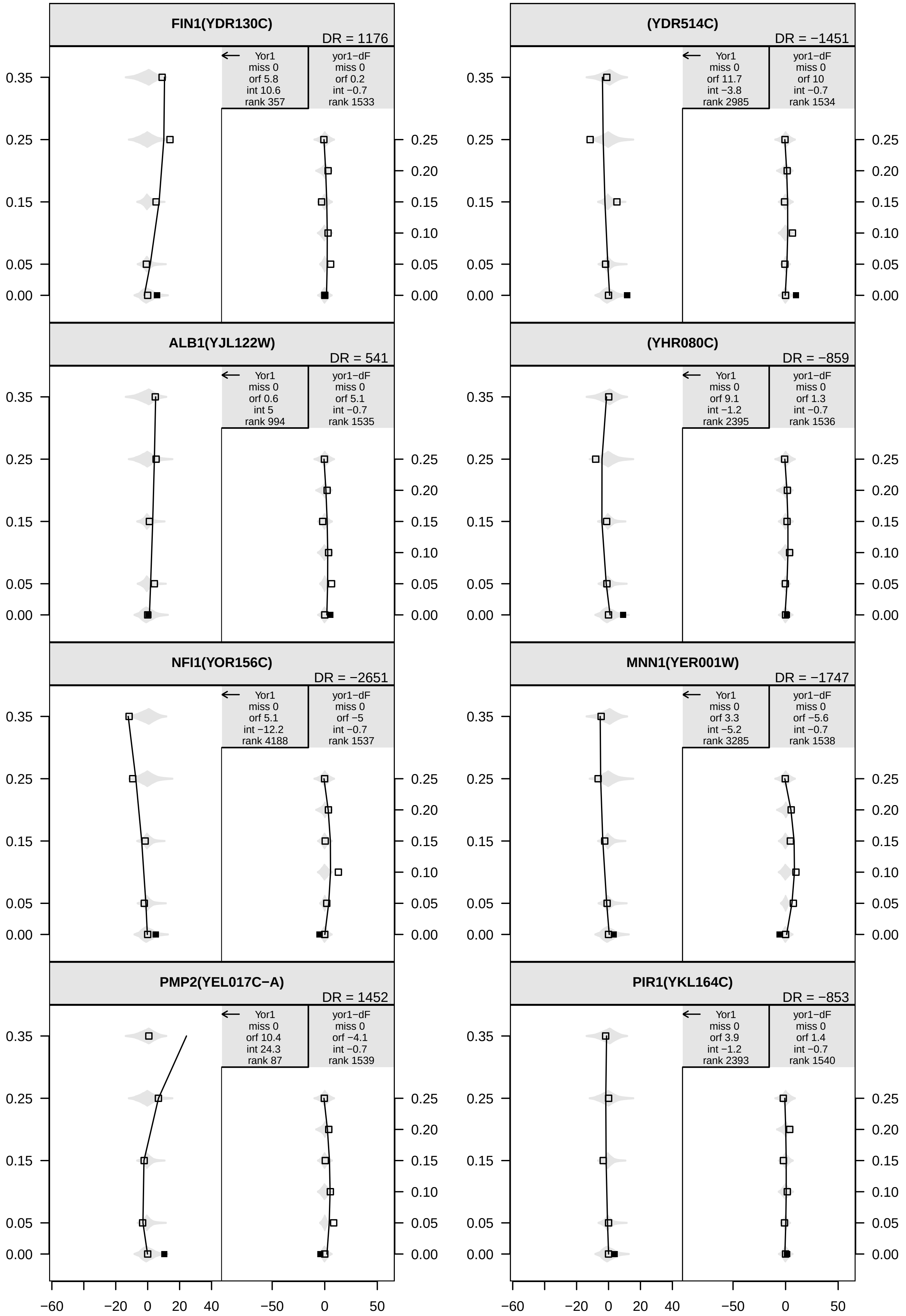
FIN1(YDR130C)
DR = 1176
Yor1 miss 0 orf 5.8 int 10.6 rank 357
yor1−dF miss 0 orf 0.2 int −0.7 rank 1533
(YDR514C)
DR = −1451
Yor1 miss 0 orf 11.7 int −3.8 rank 2985
yor1−dF miss 0 orf 10 int −0.7 rank 1534
ALB1(YJL122W)
DR = 541
Yor1 miss 0 orf 0.6 int 5 rank 994
yor1−dF miss 0 orf 5.1 int −0.7 rank 1535
(YHR080C)
DR = −859
Yor1 miss 0 orf 9.1 int −1.2 rank 2395
yor1−dF miss 0 orf 1.3 int −0.7 rank 1536
NFI1(YOR156C)
DR = −2651
Yor1 miss 0 orf 5.1 int −12.2 rank 4188
yor1−dF miss 0 orf −5 int −0.7 rank 1537
MNN1(YER001W)
DR = −1747
Yor1 miss 0 orf 3.3 int −5.2 rank 3285
yor1−dF miss 0 orf −5.6 int −0.7 rank 1538
PMP2(YEL017C−A)
DR = 1452
Yor1 miss 0 orf 10.4 int 24.3 rank 87
yor1−dF miss 0 orf −4.1 int −0.7 rank 1539
PIR1(YKL164C)
DR = −853
Yor1 miss 0 orf 3.9 int −1.2 rank 2393
yor1−dF miss 0 orf 1.4 int −0.7 rank 1540
0.00 0.05 0.10 0.15 0.20 0.25 0.35
−60 −20 0 20 40 −50 0 50

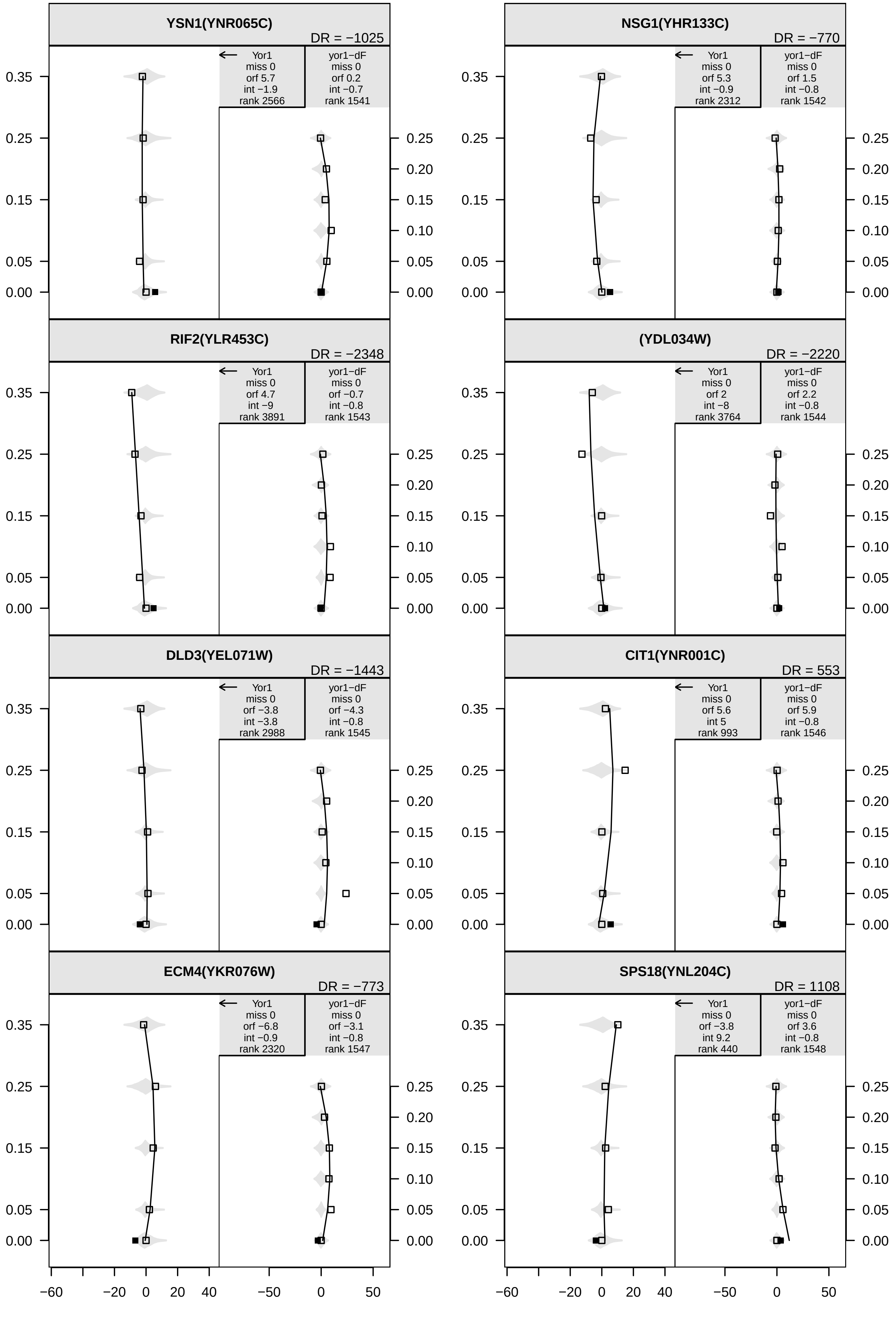

YSN1(YNR065C)
DR = −1025
Yor1 miss 0 orf 5.7 int −1.9 rank 2566
yor1−dF miss 0 orf 0.2 int −0.7 rank 1541
NSG1(YHR133C)
DR = −770
Yor1 miss 0 orf 5.3 int −0.9 rank 2312
yor1−dF miss 0 orf 1.5 int −0.8 rank 1542
RIF2(YLR453C)
DR = −2348
Yor1 miss 0 orf 4.7 int −9 rank 3891
yor1−dF miss 0 orf −0.7 int −0.8 rank 1543
(YDL034W)
DR = −2220
Yor1 miss 0 orf 2 int −8 rank 3764
yor1−dF miss 0 orf 2.2 int −0.8 rank 1544
DLD3(YEL071W)
DR = −1443
Yor1 miss 0 orf −3.8 int −3.8 rank 2988
yor1−dF miss 0 orf −4.3 int −0.8 rank 1545
CIT1(YNR001C)
DR = 553
Yor1 miss 0 orf 5.6 int 5 rank 993
yor1−dF miss 0 orf 5.9 int −0.8 rank 1546
ECM4(YKR076W)
DR = −773
Yor1 miss 0 orf −6.8 int −0.9 rank 2320
yor1−dF miss 0 orf −3.1 int −0.8 rank 1547
SPS18(YNL204C)
DR = 1108
Yor1 miss 0 orf −3.8 int 9.2 rank 440
yor1−dF miss 0 orf 3.6 int −0.8 rank 1548
0.35 0.25 0.15 0.05 0.00
0.25 0.20 0.15 0.10 0.05 0.00
−60 −20 0 20 40
−50 0 50

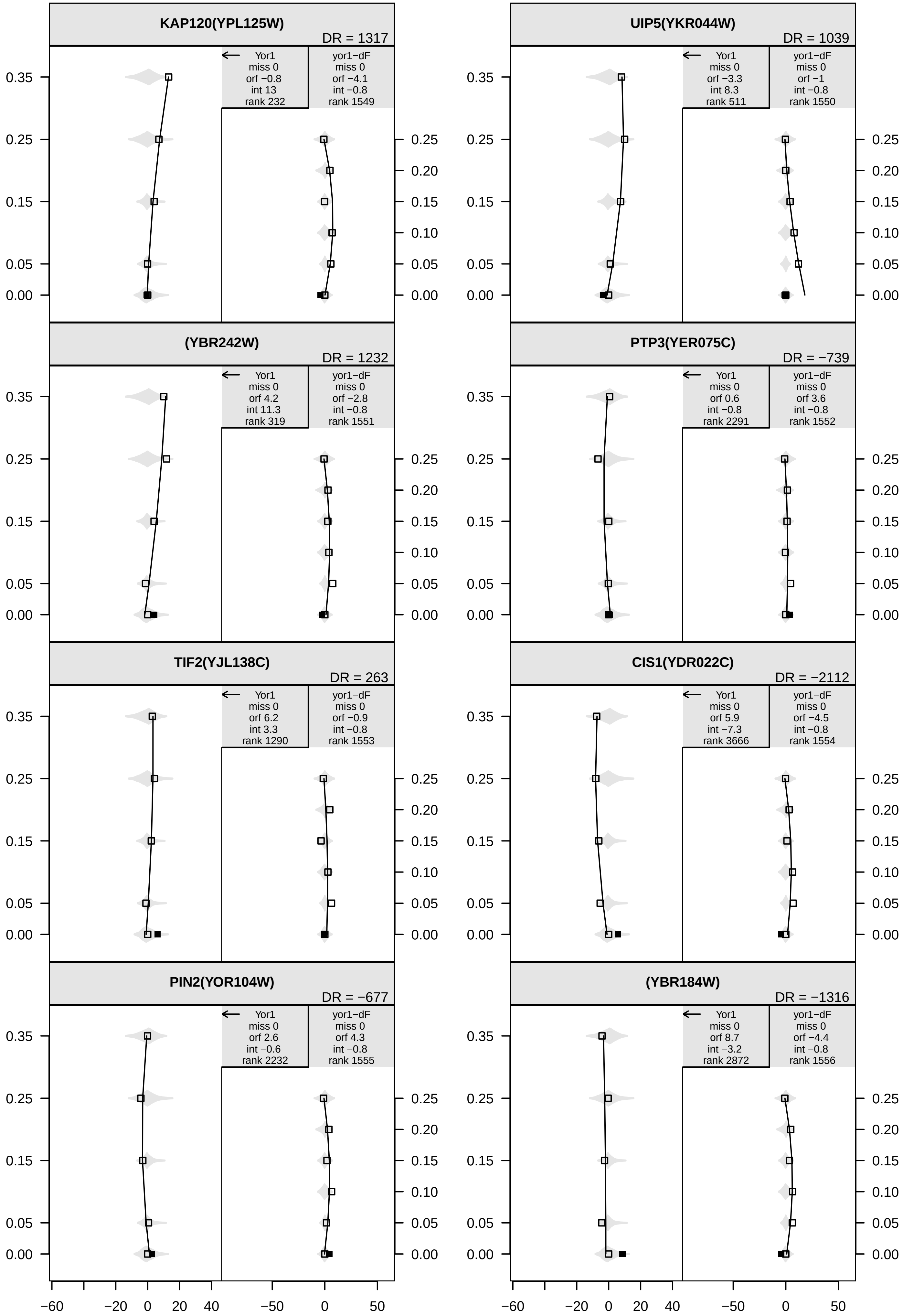
KAP120(YPL125W)
DR = 1317
Yor1
miss 0
orf −0.8
int 13
rank 232
yor1−dF
miss 0
orf −4.1
int −0.8
rank 1549
UIP5(YKR044W)
DR = 1039
Yor1
miss 0
orf −3.3
int 8.3
rank 511
yor1−dF
miss 0
orf −1
int −0.8
rank 1550
(YBR242W)
DR = 1232
Yor1
miss 0
orf 4.2
int 11.3
rank 319
yor1−dF
miss 0
orf −2.8
int −0.8
rank 1551
PTP3(YER075C)
DR = −739
Yor1
miss 0
orf 0.6
int −0.8
rank 2291
yor1−dF
miss 0
orf 3.6
int −0.8
rank 1552
TIF2(YJL138C)
DR = 263
Yor1
miss 0
orf 6.2
int 3.3
rank 1290
yor1−dF
miss 0
orf −0.9
int −0.8
rank 1553
CIS1(YDR022C)
DR = −2112
Yor1
miss 0
orf 5.9
int −7.3
rank 3666
yor1−dF
miss 0
orf −4.5
int −0.8
rank 1554
PIN2(YOR104W)
DR = −677
Yor1
miss 0
orf 2.6
int −0.6
rank 2232
yor1−dF
miss 0
orf 4.3
int −0.8
rank 1555
(YBR184W)
DR = −1316
Yor1
miss 0
orf 8.7
int −3.2
rank 2872
yor1−dF
miss 0
orf −4.4
int −0.8
rank 1556
0.35
0.25
0.20
0.15
0.10
0.05
0.00
−60
−20
0
20
40
−50
0
50

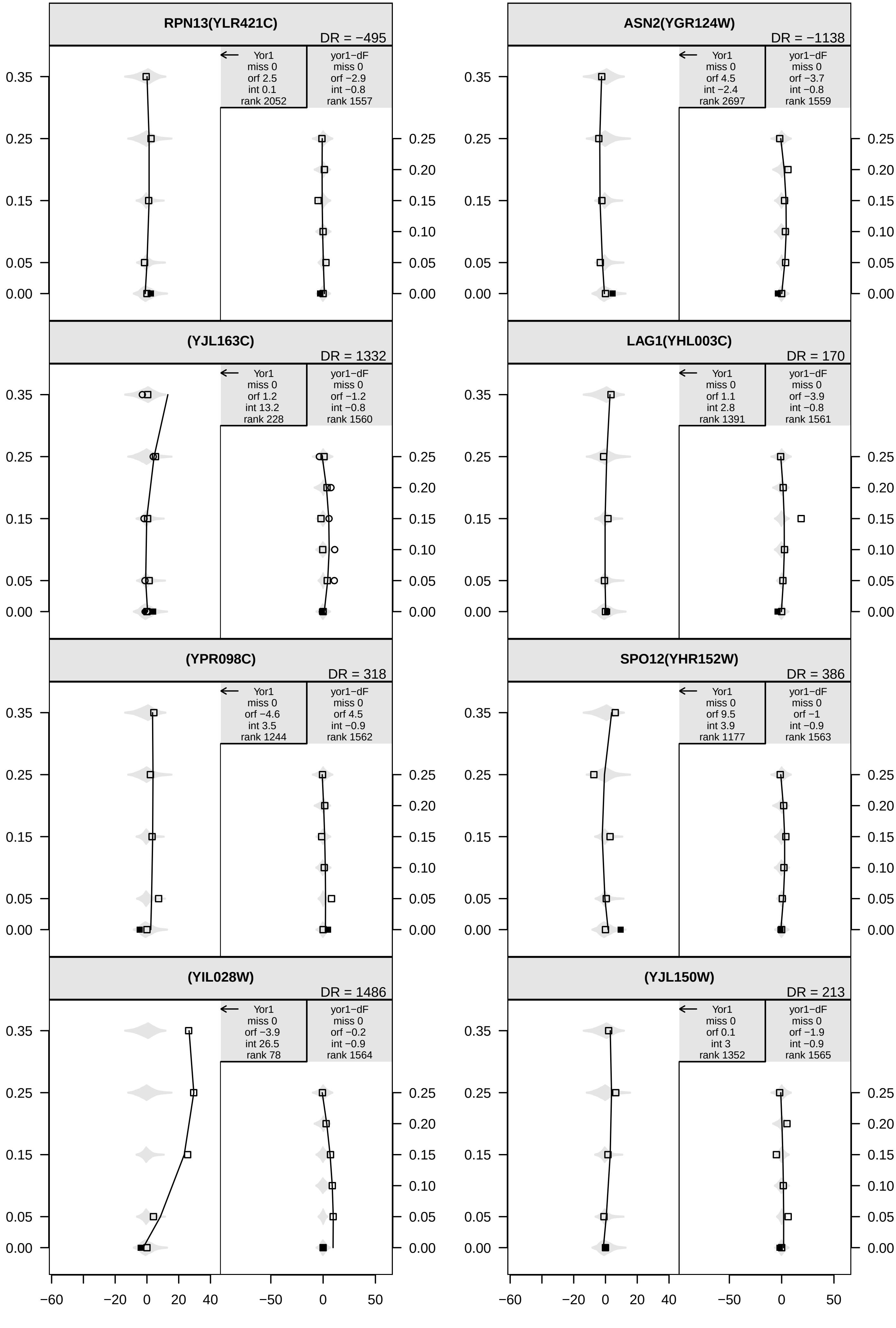

RPN13(YLR421C)
DR = −495
Yor1 miss 0 orf 2.5 int 0.1 rank 2052
yor1−dF miss 0 orf −2.9 int −0.8 rank 1557
ASN2(YGR124W)
DR = −1138
Yor1 miss 0 orf 4.5 int −2.4 rank 2697
yor1−dF miss 0 orf −3.7 int −0.8 rank 1559
(YJL163C)
DR = 1332
Yor1 miss 0 orf 1.2 int 13.2 rank 228
yor1−dF miss 0 orf −1.2 int −0.8 rank 1560
LAG1(YHL003C)
DR = 170
Yor1 miss 0 orf 1.1 int 2.8 rank 1391
yor1−dF miss 0 orf −3.9 int −0.8 rank 1561
(YPR098C)
DR = 318
Yor1 miss 0 orf −4.6 int 3.5 rank 1244
yor1−dF miss 0 orf 4.5 int −0.9 rank 1562
SPO12(YHR152W)
DR = 386
Yor1 miss 0 orf 9.5 int 3.9 rank 1177
yor1−dF miss 0 orf −1 int −0.9 rank 1563
(YIL028W)
DR = 1486
Yor1 miss 0 orf −3.9 int 26.5 rank 78
yor1−dF miss 0 orf −0.2 int −0.9 rank 1564
(YJL150W)
DR = 213
Yor1 miss 0 orf 0.1 int 3 rank 1352
yor1−dF miss 0 orf −1.9 int −0.9 rank 1565
0.35 0.25 0.15 0.05 0.00
0.25 0.20 0.15 0.10 0.05 0.00
−60 −20 0 20 40 −50 0 50

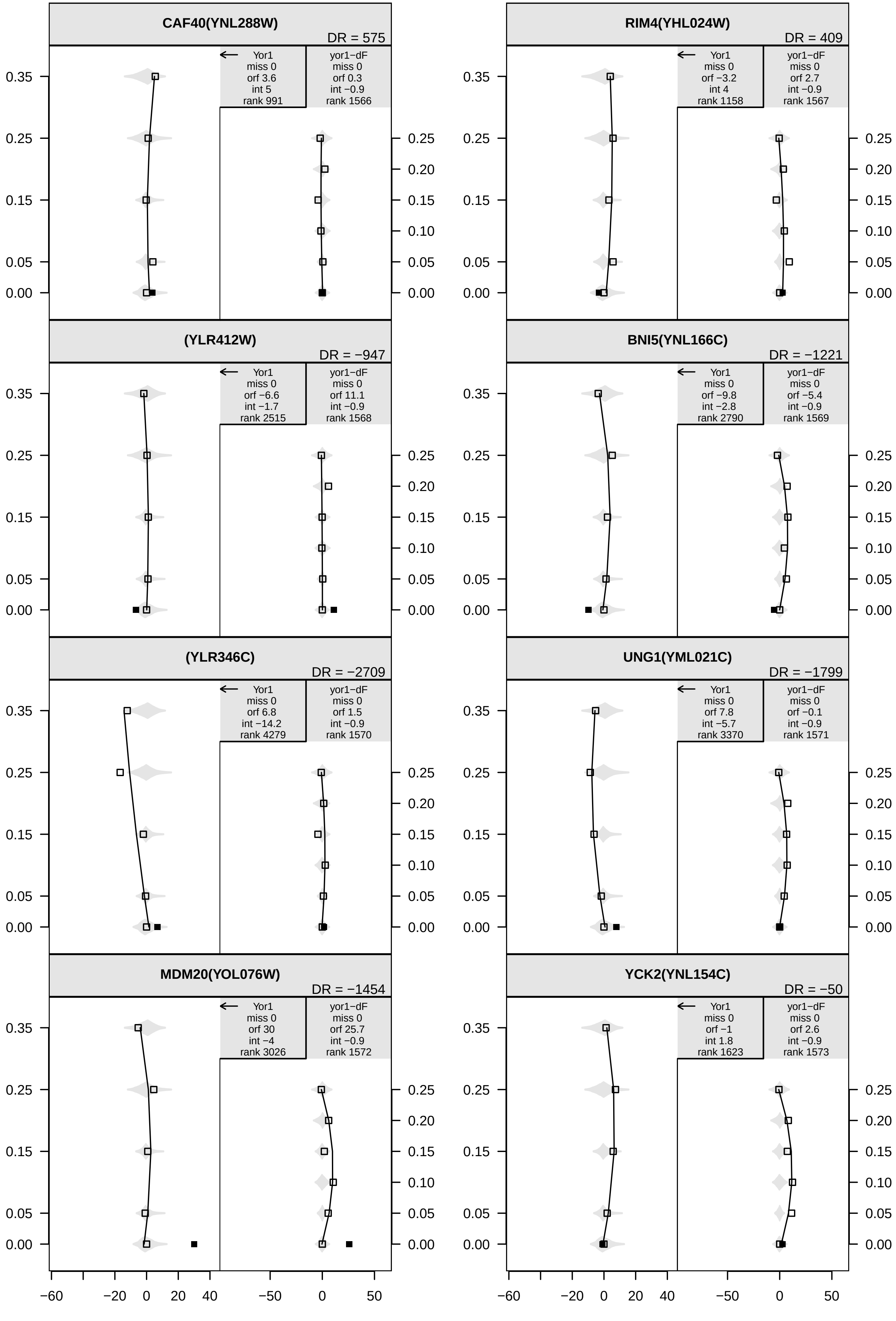
CAF40(YNL288W)
DR = 575
Yor1 miss 0 orf 3.6 int 5 rank 991
yor1−dF miss 0 orf 0.3 int −0.9 rank 1566
RIM4(YHL024W)
DR = 409
Yor1 miss 0 orf −3.2 int 4 rank 1158
yor1−dF miss 0 orf 2.7 int −0.9 rank 1567
(YLR412W)
DR = −947
Yor1 miss 0 orf −6.6 int −1.7 rank 2515
yor1−dF miss 0 orf 11.1 int −0.9 rank 1568
BNI5(YNL166C)
DR = −1221
Yor1 miss 0 orf −9.8 int −2.8 rank 2790
yor1−dF miss 0 orf −5.4 int −0.9 rank 1569
(YLR346C)
DR = −2709
Yor1 miss 0 orf 6.8 int −14.2 rank 4279
yor1−dF miss 0 orf 1.5 int −0.9 rank 1570
UNG1(YML021C)
DR = −1799
Yor1 miss 0 orf 7.8 int −5.7 rank 3370
yor1−dF miss 0 orf −0.1 int −0.9 rank 1571
MDM20(YOL076W)
DR = −1454
Yor1 miss 0 orf 30 int −4 rank 3026
yor1−dF miss 0 orf 25.7 int −0.9 rank 1572
YCK2(YNL154C)
DR = −50
Yor1 miss 0 orf −1 int 1.8 rank 1623
yor1−dF miss 0 orf 2.6 int −0.9 rank 1573
0.00 0.05 0.10 0.15 0.20 0.25 0.35
−60 −20 0 20 40 −50 0 50

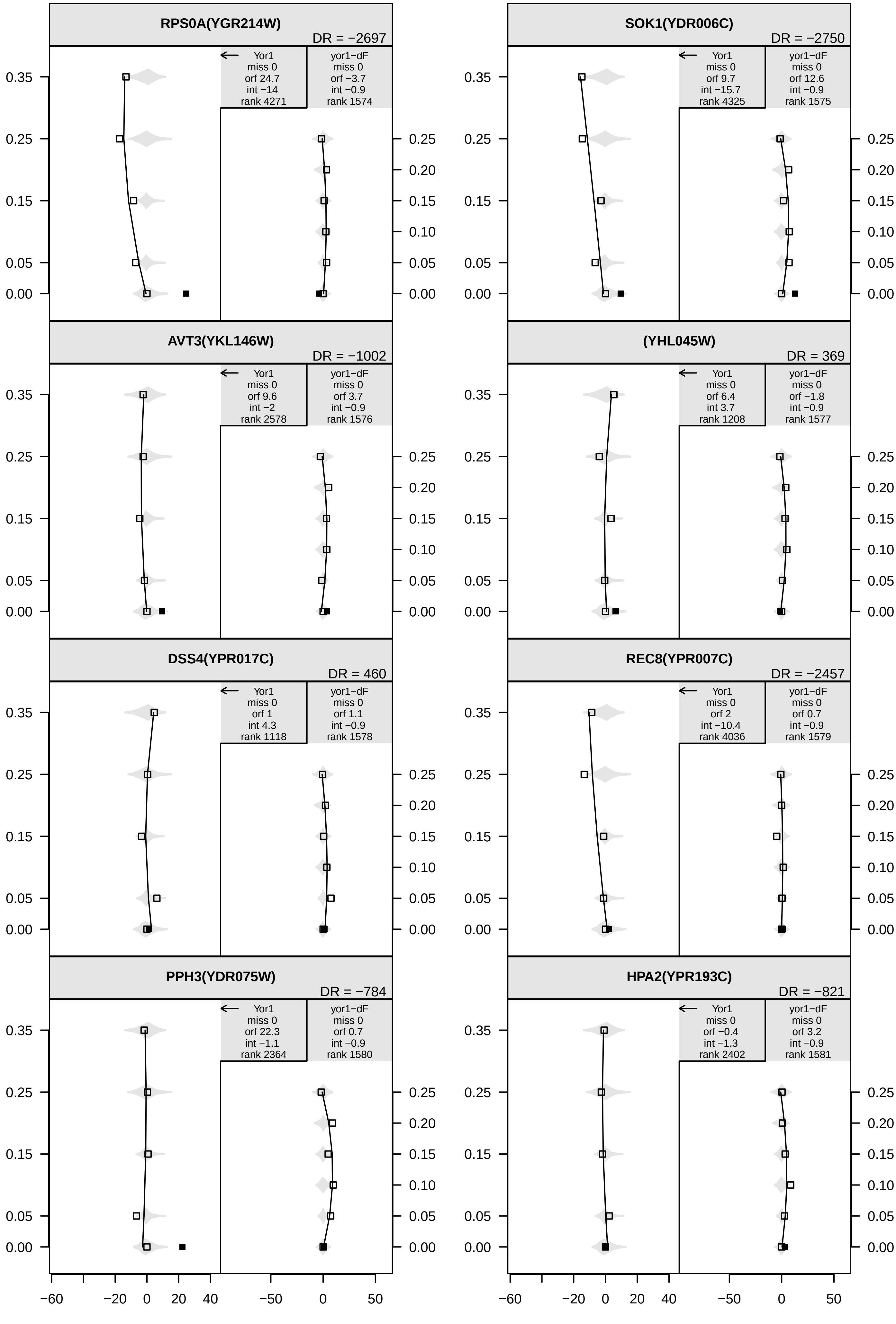

RPS0A(YGR214W)
DR = −2697
Yor1 miss 0 orf 24.7 int −14 rank 4271
yor1−dF miss 0 orf −3.7 int −0.9 rank 1574
SOK1(YDR006C)
DR = −2750
Yor1 miss 0 orf 9.7 int −15.7 rank 4325
yor1−dF miss 0 orf 12.6 int −0.9 rank 1575
AVT3(YKL146W)
DR = −1002
Yor1 miss 0 orf 9.6 int −2 rank 2578
yor1−dF miss 0 orf 3.7 int −0.9 rank 1576
(YHL045W)
DR = 369
Yor1 miss 0 orf 6.4 int 3.7 rank 1208
yor1−dF miss 0 orf −1.8 int −0.9 rank 1577
DSS4(YPR017C)
DR = 460
Yor1 miss 0 orf 1 int 4.3 rank 1118
yor1−dF miss 0 orf 1.1 int −0.9 rank 1578
REC8(YPR007C)
DR = −2457
Yor1 miss 0 orf 2 int −10.4 rank 4036
yor1−dF miss 0 orf 0.7 int −0.9 rank 1579
PPH3(YDR075W)
DR = −784
Yor1 miss 0 orf 22.3 int −1.1 rank 2364
yor1−dF miss 0 orf 0.7 int −0.9 rank 1580
HPA2(YPR193C)
DR = −821
Yor1 miss 0 orf −0.4 int −1.3 rank 2402
yor1−dF miss 0 orf 3.2 int −0.9 rank 1581
0.00 0.05 0.10 0.15 0.20 0.25 0.35
−60 −20 0 20 40 −50 0 50

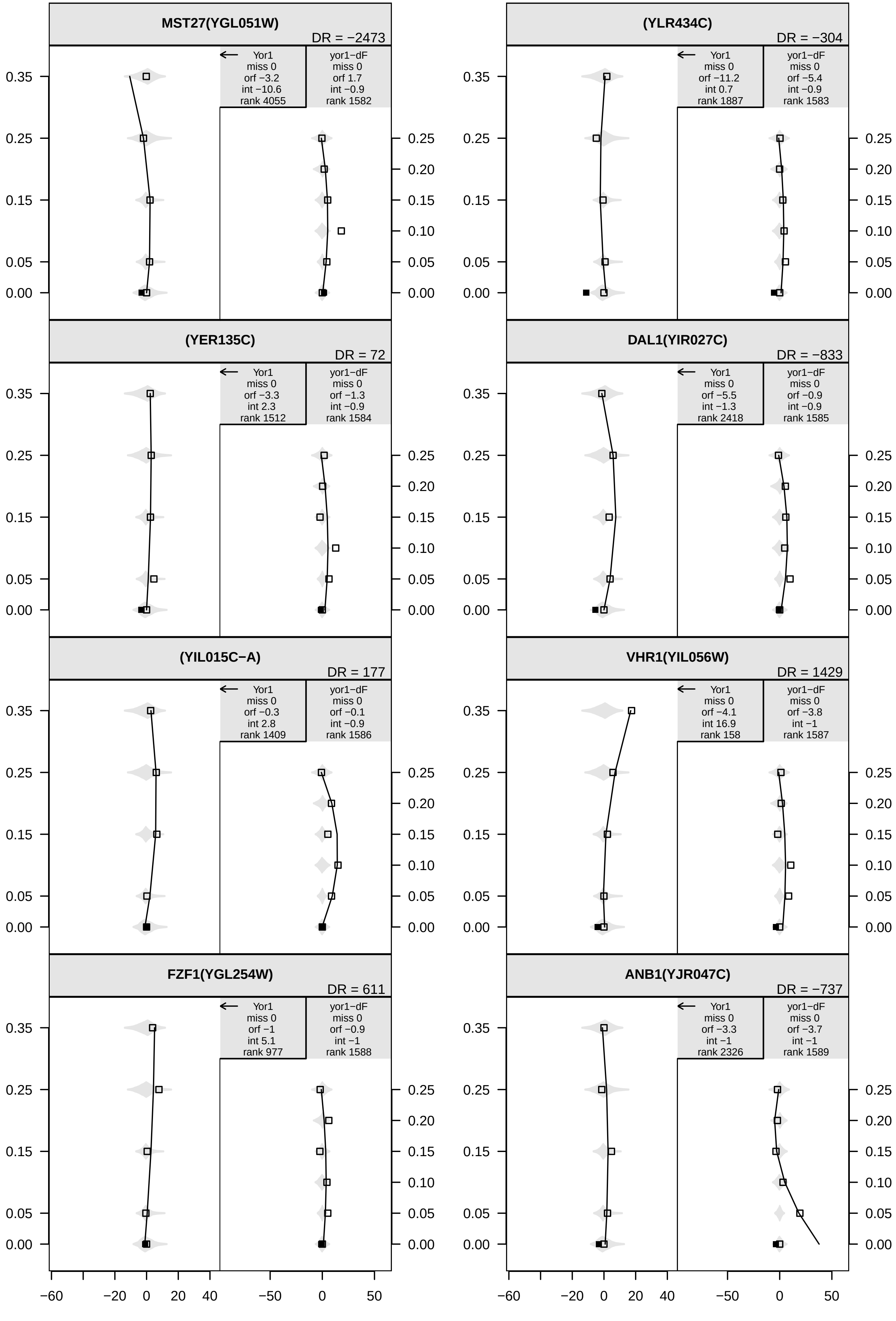
MST27(YGL051W)
DR = −2473
Yor1 miss 0 orf −3.2 int −10.6 rank 4055
yor1−dF miss 0 orf 1.7 int −0.9 rank 1582
(YLR434C)
DR = −304
Yor1 miss 0 orf −11.2 int 0.7 rank 1887
yor1−dF miss 0 orf −5.4 int −0.9 rank 1583
(YER135C)
DR = 72
Yor1 miss 0 orf −3.3 int 2.3 rank 1512
yor1−dF miss 0 orf −1.3 int −0.9 rank 1584
DAL1(YIR027C)
DR = −833
Yor1 miss 0 orf −5.5 int −1.3 rank 2418
yor1−dF miss 0 orf −0.9 int −0.9 rank 1585
(YIL015C−A)
DR = 177
Yor1 miss 0 orf −0.3 int 2.8 rank 1409
yor1−dF miss 0 orf −0.1 int −0.9 rank 1586
VHR1(YIL056W)
DR = 1429
Yor1 miss 0 orf −4.1 int 16.9 rank 158
yor1−dF miss 0 orf −3.8 int −1 rank 1587
FZF1(YGL254W)
DR = 611
Yor1 miss 0 orf −1 int 5.1 rank 977
yor1−dF miss 0 orf −0.9 int −1 rank 1588
ANB1(YJR047C)
DR = −737
Yor1 miss 0 orf −3.3 int −1 rank 2326
yor1−dF miss 0 orf −3.7 int −1 rank 1589
0.35
0.25
0.20
0.15
0.10
0.05
0.00
−60
−20
0
20
40
−50
0
50

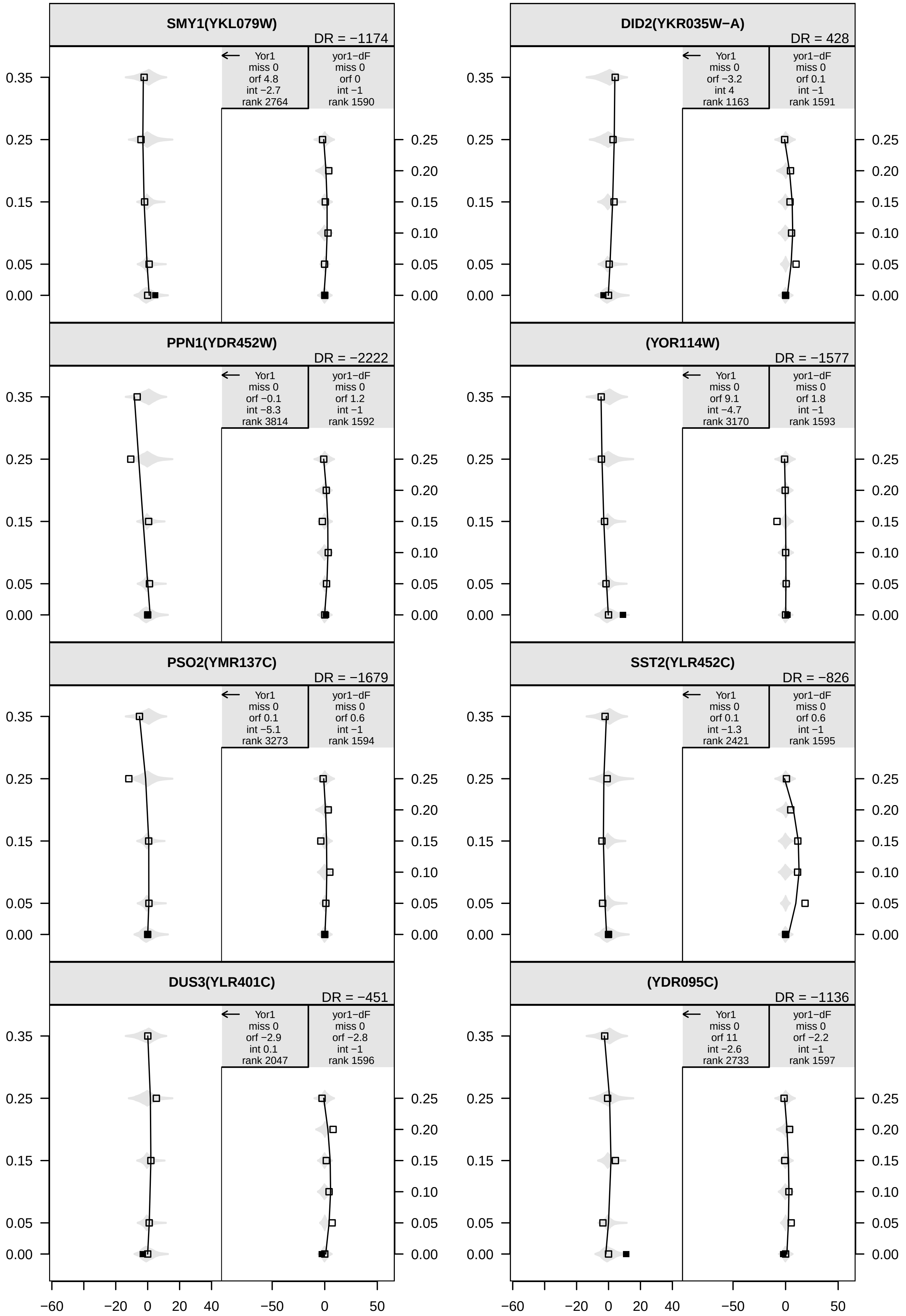
SMY1(YKL079W)
DR = −1174
Yor1 miss 0 orf 4.8 int −2.7 rank 2764
yor1−dF miss 0 orf 0 int −1 rank 1590
DID2(YKR035W−A)
DR = 428
Yor1 miss 0 orf −3.2 int 4 rank 1163
yor1−dF miss 0 orf 0.1 int −1 rank 1591
PPN1(YDR452W)
DR = −2222
Yor1 miss 0 orf −0.1 int −8.3 rank 3814
yor1−dF miss 0 orf 1.2 int −1 rank 1592
(YOR114W)
DR = −1577
Yor1 miss 0 orf 9.1 int −4.7 rank 3170
yor1−dF miss 0 orf 1.8 int −1 rank 1593
PSO2(YMR137C)
DR = −1679
Yor1 miss 0 orf 0.1 int −5.1 rank 3273
yor1−dF miss 0 orf 0.6 int −1 rank 1594
SST2(YLR452C)
DR = −826
Yor1 miss 0 orf 0.1 int −1.3 rank 2421
yor1−dF miss 0 orf 0.6 int −1 rank 1595
DUS3(YLR401C)
DR = −451
Yor1 miss 0 orf −2.9 int 0.1 rank 2047
yor1−dF miss 0 orf −2.8 int −1 rank 1596
(YDR095C)
DR = −1136
Yor1 miss 0 orf 11 int −2.6 rank 2733
yor1−dF miss 0 orf −2.2 int −1 rank 1597
0.35 0.25 0.15 0.05 0.00
0.25 0.20 0.15 0.10 0.05 0.00
−60 −20 0 20 40 −50 0 50

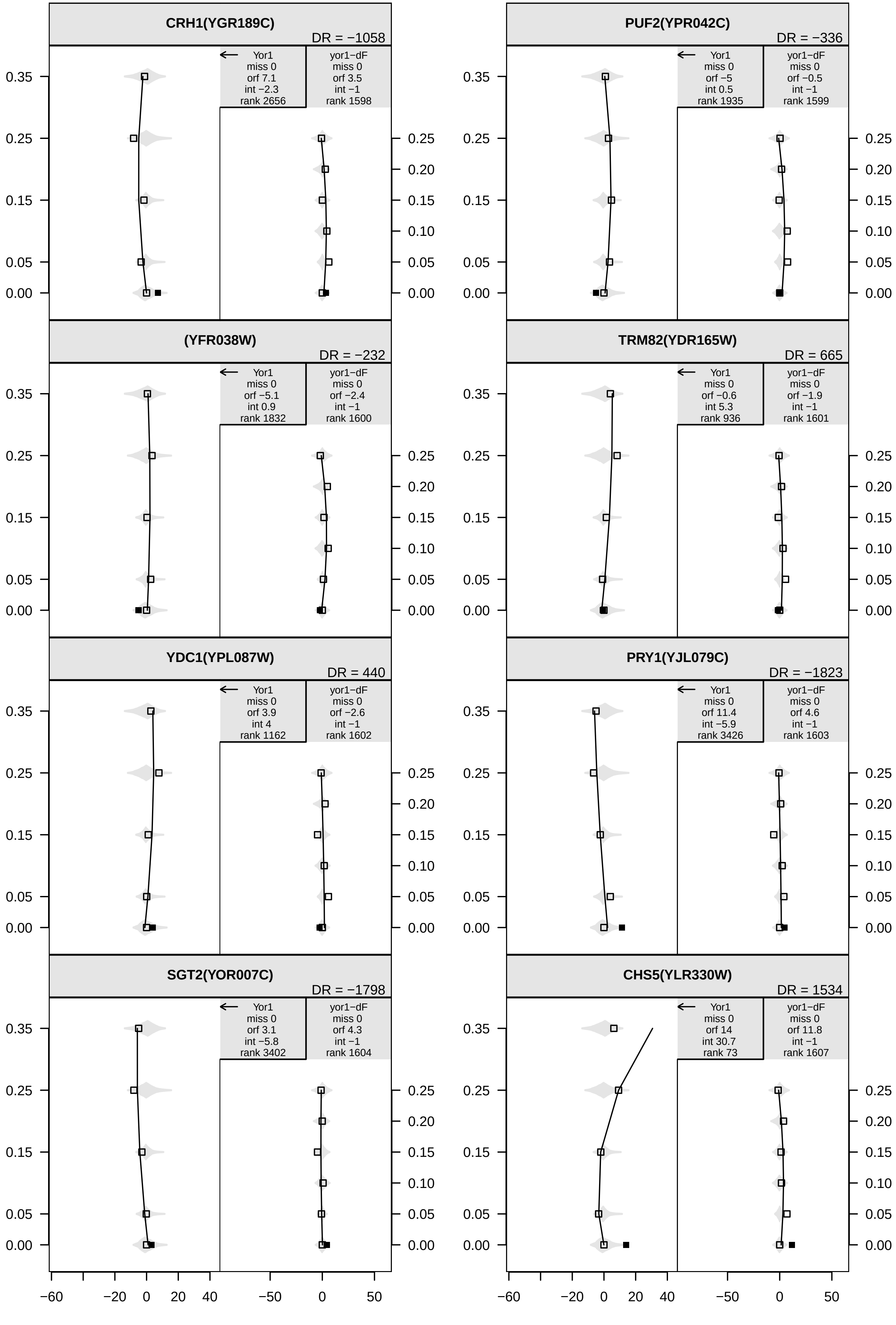

CRH1(YGR189C)
DR = −1058
Yor1 miss 0 orf 7.1 int −2.3 rank 2656
yor1−dF miss 0 orf 3.5 int −1 rank 1598
PUF2(YPR042C)
DR = −336
Yor1 miss 0 orf −5 int 0.5 rank 1935
yor1−dF miss 0 orf −0.5 int −1 rank 1599
(YFR038W)
DR = −232
Yor1 miss 0 orf −5.1 int 0.9 rank 1832
yor1−dF miss 0 orf −2.4 int −1 rank 1600
TRM82(YDR165W)
DR = 665
Yor1 miss 0 orf −0.6 int 5.3 rank 936
yor1−dF miss 0 orf −1.9 int −1 rank 1601
YDC1(YPL087W)
DR = 440
Yor1 miss 0 orf 3.9 int 4 rank 1162
yor1−dF miss 0 orf −2.6 int −1 rank 1602
PRY1(YJL079C)
DR = −1823
Yor1 miss 0 orf 11.4 int −5.9 rank 3426
yor1−dF miss 0 orf 4.6 int −1 rank 1603
SGT2(YOR007C)
DR = −1798
Yor1 miss 0 orf 3.1 int −5.8 rank 3402
yor1−dF miss 0 orf 4.3 int −1 rank 1604
CHS5(YLR330W)
DR = 1534
Yor1 miss 0 orf 14 int 30.7 rank 73
yor1−dF miss 0 orf 11.8 int −1 rank 1607
0.35 0.25 0.20 0.15 0.10 0.05 0.00
−60 −20 0 20 40 −50 0 50

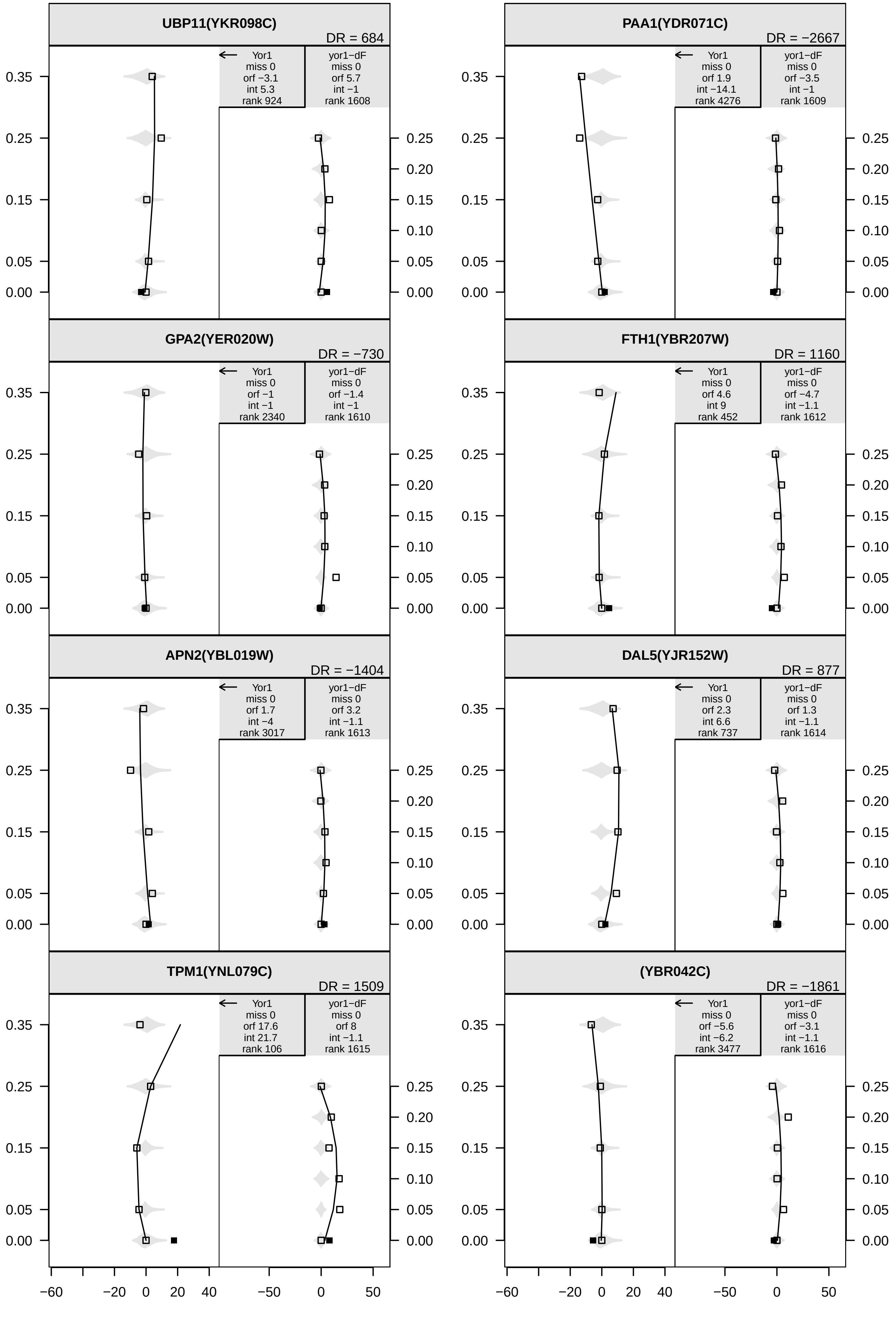
UBP11(YKR098C)
DR = 684
Yor1 miss 0 orf −3.1 int 5.3 rank 924
yor1−dF miss 0 orf 5.7 int −1 rank 1608
PAA1(YDR071C)
DR = −2667
Yor1 miss 0 orf 1.9 int −14.1 rank 4276
yor1−dF miss 0 orf −3.5 int −1 rank 1609
GPA2(YER020W)
DR = −730
Yor1 miss 0 orf −1 int −1 rank 2340
yor1−dF miss 0 orf −1.4 int −1 rank 1610
FTH1(YBR207W)
DR = 1160
Yor1 miss 0 orf 4.6 int 9 rank 452
yor1−dF miss 0 orf −4.7 int −1.1 rank 1612
APN2(YBL019W)
DR = −1404
Yor1 miss 0 orf 1.7 int −4 rank 3017
yor1−dF miss 0 orf 3.2 int −1.1 rank 1613
DAL5(YJR152W)
DR = 877
Yor1 miss 0 orf 2.3 int 6.6 rank 737
yor1−dF miss 0 orf 1.3 int −1.1 rank 1614
TPM1(YNL079C)
DR = 1509
Yor1 miss 0 orf 17.6 int 21.7 rank 106
yor1−dF miss 0 orf 8 int −1.1 rank 1615
(YBR042C)
DR = −1861
Yor1 miss 0 orf −5.6 int −6.2 rank 3477
yor1−dF miss 0 orf −3.1 int −1.1 rank 1616
0.35 0.25 0.15 0.05 0.00
0.25 0.20 0.15 0.10 0.05 0.00
−60 −20 0 20 40
−50 0 50

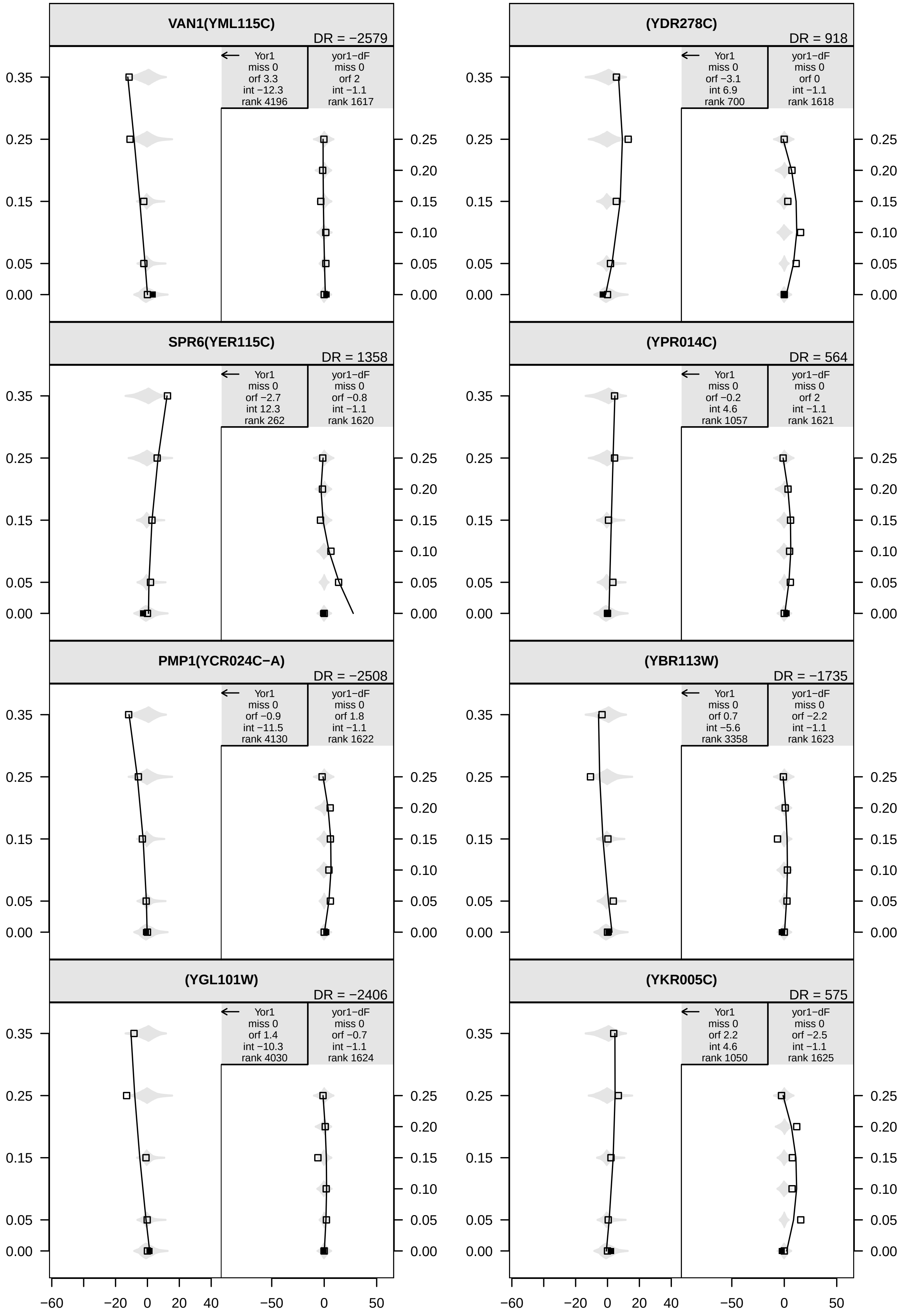

VAN1(YML115C)
DR = −2579
Yor1 miss 0 orf 3.3 int −12.3 rank 4196
yor1−dF miss 0 orf 2 int −1.1 rank 1617
(YDR278C)
DR = 918
Yor1 miss 0 orf −3.1 int 6.9 rank 700
yor1−dF miss 0 orf 0 int −1.1 rank 1618
SPR6(YER115C)
DR = 1358
Yor1 miss 0 orf −2.7 int 12.3 rank 262
yor1−dF miss 0 orf −0.8 int −1.1 rank 1620
(YPR014C)
DR = 564
Yor1 miss 0 orf −0.2 int 4.6 rank 1057
yor1−dF miss 0 orf 2 int −1.1 rank 1621
PMP1(YCR024C−A)
DR = −2508
Yor1 miss 0 orf −0.9 int −11.5 rank 4130
yor1−dF miss 0 orf 1.8 int −1.1 rank 1622
(YBR113W)
DR = −1735
Yor1 miss 0 orf 0.7 int −5.6 rank 3358
yor1−dF miss 0 orf −2.2 int −1.1 rank 1623
(YGL101W)
DR = −2406
Yor1 miss 0 orf 1.4 int −10.3 rank 4030
yor1−dF miss 0 orf −0.7 int −1.1 rank 1624
(YKR005C)
DR = 575
Yor1 miss 0 orf 2.2 int 4.6 rank 1050
yor1−dF miss 0 orf −2.5 int −1.1 rank 1625
0.35
0.25
0.20
0.15
0.10
0.05
0.00
−60
−20
0
20
40
−50
0
50

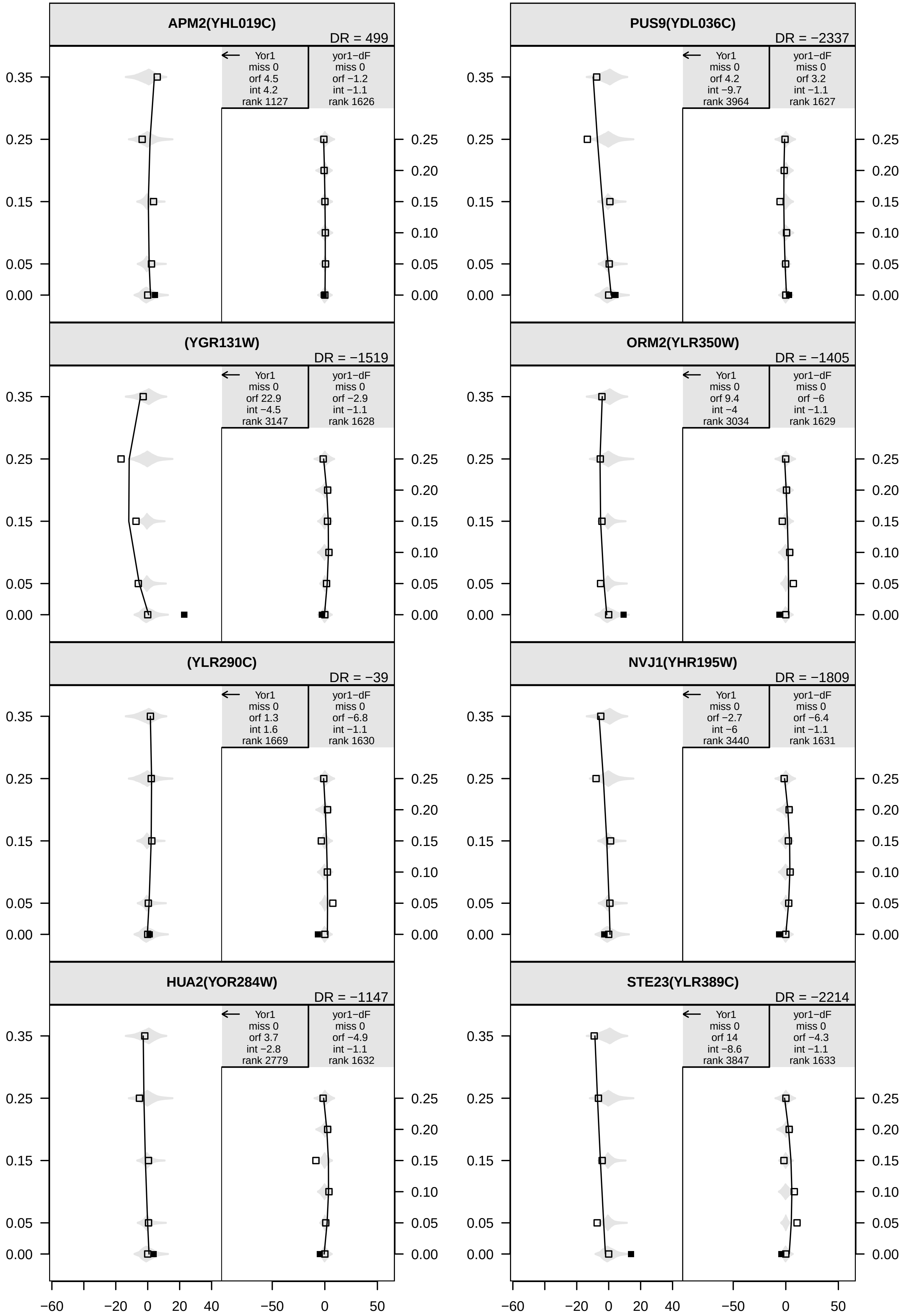
APM2(YHL019C)
DR = 499
Yor1
miss 0
orf 4.5
int 4.2
rank 1127
yor1−dF
miss 0
orf −1.2
int −1.1
rank 1626
PUS9(YDL036C)
DR = −2337
Yor1
miss 0
orf 4.2
int −9.7
rank 3964
yor1−dF
miss 0
orf 3.2
int −1.1
rank 1627
(YGR131W)
DR = −1519
Yor1
miss 0
orf 22.9
int −4.5
rank 3147
yor1−dF
miss 0
orf −2.9
int −1.1
rank 1628
ORM2(YLR350W)
DR = −1405
Yor1
miss 0
orf 9.4
int −4
rank 3034
yor1−dF
miss 0
orf −6
int −1.1
rank 1629
(YLR290C)
DR = −39
Yor1
miss 0
orf 1.3
int 1.6
rank 1669
yor1−dF
miss 0
orf −6.8
int −1.1
rank 1630
NVJ1(YHR195W)
DR = −1809
Yor1
miss 0
orf −2.7
int −6
rank 3440
yor1−dF
miss 0
orf −6.4
int −1.1
rank 1631
HUA2(YOR284W)
DR = −1147
Yor1
miss 0
orf 3.7
int −2.8
rank 2779
yor1−dF
miss 0
orf −4.9
int −1.1
rank 1632
STE23(YLR389C)
DR = −2214
Yor1
miss 0
orf 14
int −8.6
rank 3847
yor1−dF
miss 0
orf −4.3
int −1.1
rank 1633
0.35
0.25
0.20
0.15
0.10
0.05
0.00
−60
−20
0
20
40
−50
0
50

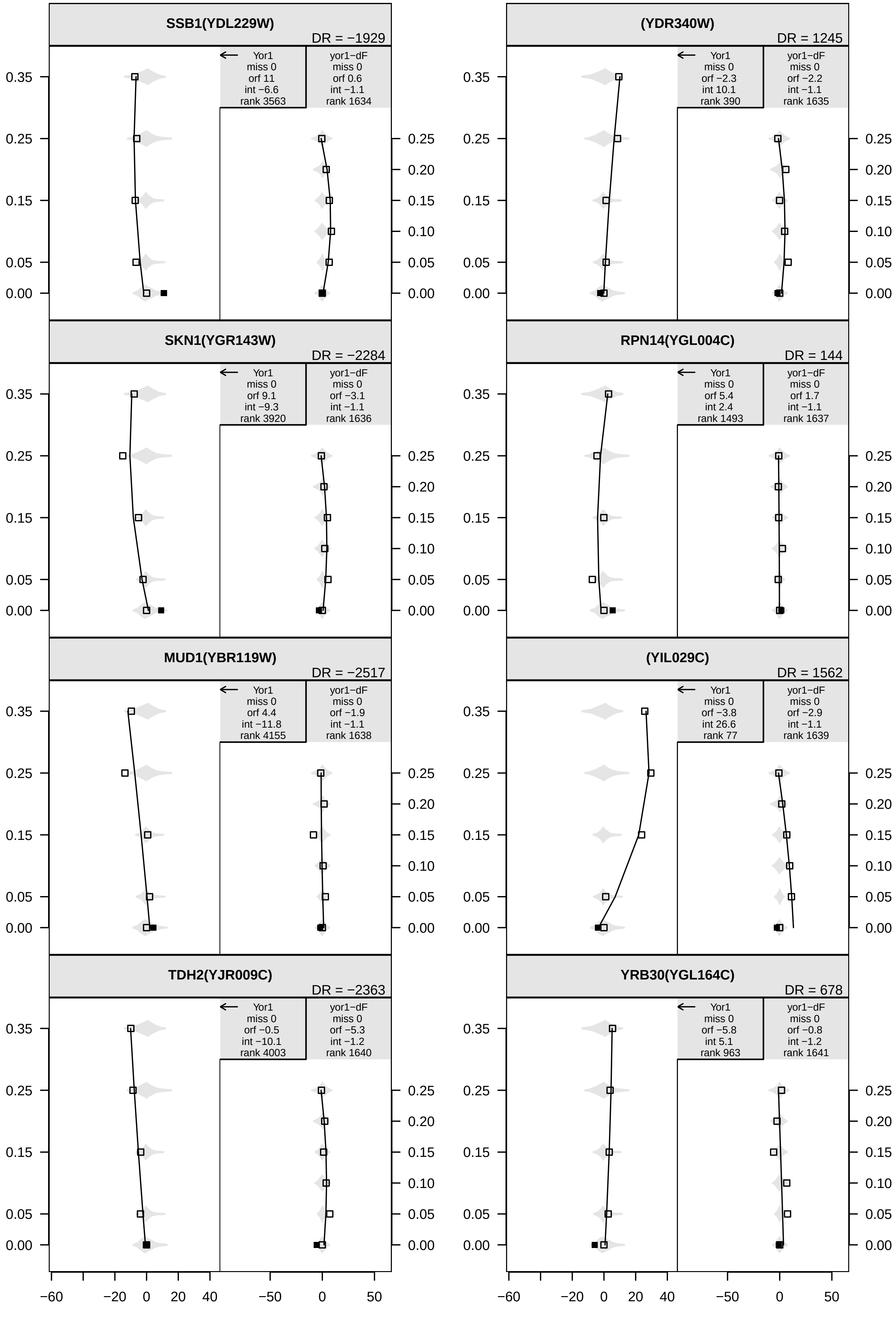

SSB1(YDL229W)
DR = −1929
Yor1 miss 0 orf 11 int −6.6 rank 3563
yor1−dF miss 0 orf 0.6 int −1.1 rank 1634
(YDR340W)
DR = 1245
Yor1 miss 0 orf −2.3 int 10.1 rank 390
yor1−dF miss 0 orf −2.2 int −1.1 rank 1635
SKN1(YGR143W)
DR = −2284
Yor1 miss 0 orf 9.1 int −9.3 rank 3920
yor1−dF miss 0 orf −3.1 int −1.1 rank 1636
RPN14(YGL004C)
DR = 144
Yor1 miss 0 orf 5.4 int 2.4 rank 1493
yor1−dF miss 0 orf 1.7 int −1.1 rank 1637
MUD1(YBR119W)
DR = −2517
Yor1 miss 0 orf 4.4 int −11.8 rank 4155
yor1−dF miss 0 orf −1.9 int −1.1 rank 1638
(YIL029C)
DR = 1562
Yor1 miss 0 orf −3.8 int 26.6 rank 77
yor1−dF miss 0 orf −2.9 int −1.1 rank 1639
TDH2(YJR009C)
DR = −2363
Yor1 miss 0 orf −0.5 int −10.1 rank 4003
yor1−dF miss 0 orf −5.3 int −1.2 rank 1640
YRB30(YGL164C)
DR = 678
Yor1 miss 0 orf −5.8 int 5.1 rank 963
yor1−dF miss 0 orf −0.8 int −1.2 rank 1641
0.00 0.05 0.15 0.25 0.35
0.00 0.05 0.10 0.15 0.20 0.25
−60 −20 0 20 40
−50 0 50

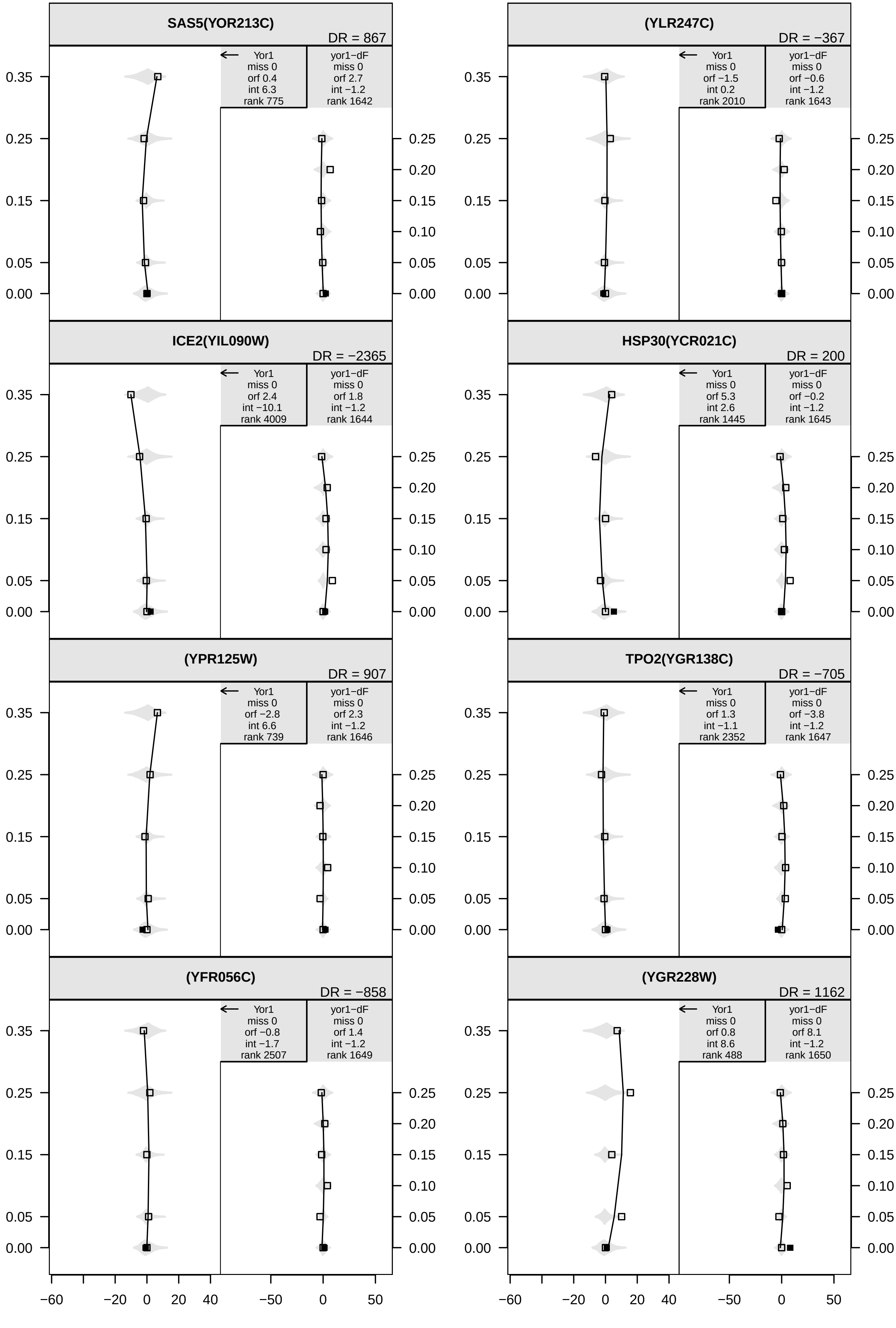

SAS5(YOR213C)
DR = 867
Yor1 miss 0 orf 0.4 int 6.3 rank 775
yor1−dF miss 0 orf 2.7 int −1.2 rank 1642
(YLR247C)
DR = −367
Yor1 miss 0 orf −1.5 int 0.2 rank 2010
yor1−dF miss 0 orf −0.6 int −1.2 rank 1643
ICE2(YIL090W)
DR = −2365
Yor1 miss 0 orf 2.4 int −10.1 rank 4009
yor1−dF miss 0 orf 1.8 int −1.2 rank 1644
HSP30(YCR021C)
DR = 200
Yor1 miss 0 orf 5.3 int 2.6 rank 1445
yor1−dF miss 0 orf −0.2 int −1.2 rank 1645
(YPR125W)
DR = 907
Yor1 miss 0 orf −2.8 int 6.6 rank 739
yor1−dF miss 0 orf 2.3 int −1.2 rank 1646
TPO2(YGR138C)
DR = −705
Yor1 miss 0 orf 1.3 int −1.1 rank 2352
yor1−dF miss 0 orf −3.8 int −1.2 rank 1647
(YFR056C)
DR = −858
Yor1 miss 0 orf −0.8 int −1.7 rank 2507
yor1−dF miss 0 orf 1.4 int −1.2 rank 1649
(YGR228W)
DR = 1162
Yor1 miss 0 orf 0.8 int 8.6 rank 488
yor1−dF miss 0 orf 8.1 int −1.2 rank 1650
0.35 0.25 0.15 0.05 0.00
0.25 0.20 0.15 0.10 0.05 0.00
−60 −20 0 20 40
−50 0 50

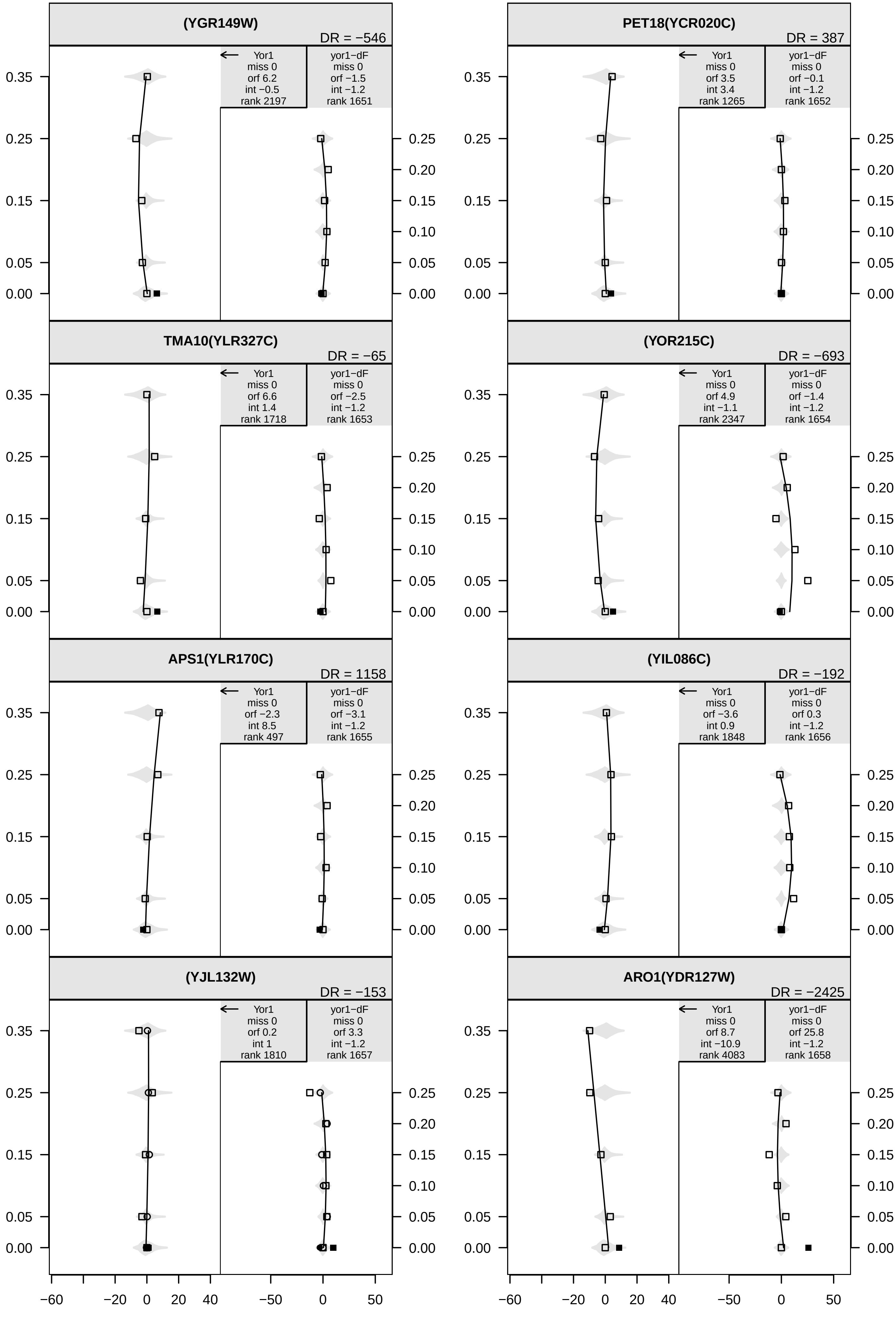

(YGR149W)
DR = −546
Yor1 miss 0 orf 6.2 int −0.5 rank 2197
yor1−dF miss 0 orf −1.5 int −1.2 rank 1651
PET18(YCR020C)
DR = 387
Yor1 miss 0 orf 3.5 int 3.4 rank 1265
yor1−dF miss 0 orf −0.1 int −1.2 rank 1652
TMA10(YLR327C)
DR = −65
Yor1 miss 0 orf 6.6 int 1.4 rank 1718
yor1−dF miss 0 orf −2.5 int −1.2 rank 1653
(YOR215C)
DR = −693
Yor1 miss 0 orf 4.9 int −1.1 rank 2347
yor1−dF miss 0 orf −1.4 int −1.2 rank 1654
APS1(YLR170C)
DR = 1158
Yor1 miss 0 orf −2.3 int 8.5 rank 497
yor1−dF miss 0 orf −3.1 int −1.2 rank 1655
(YIL086C)
DR = −192
Yor1 miss 0 orf −3.6 int 0.9 rank 1848
yor1−dF miss 0 orf 0.3 int −1.2 rank 1656
(YJL132W)
DR = −153
Yor1 miss 0 orf 0.2 int 1 rank 1810
yor1−dF miss 0 orf 3.3 int −1.2 rank 1657
ARO1(YDR127W)
DR = −2425
Yor1 miss 0 orf 8.7 int −10.9 rank 4083
yor1−dF miss 0 orf 25.8 int −1.2 rank 1658
0.00 0.05 0.10 0.15 0.20 0.25 0.35
−60 −20 0 20 40 −50 0 50

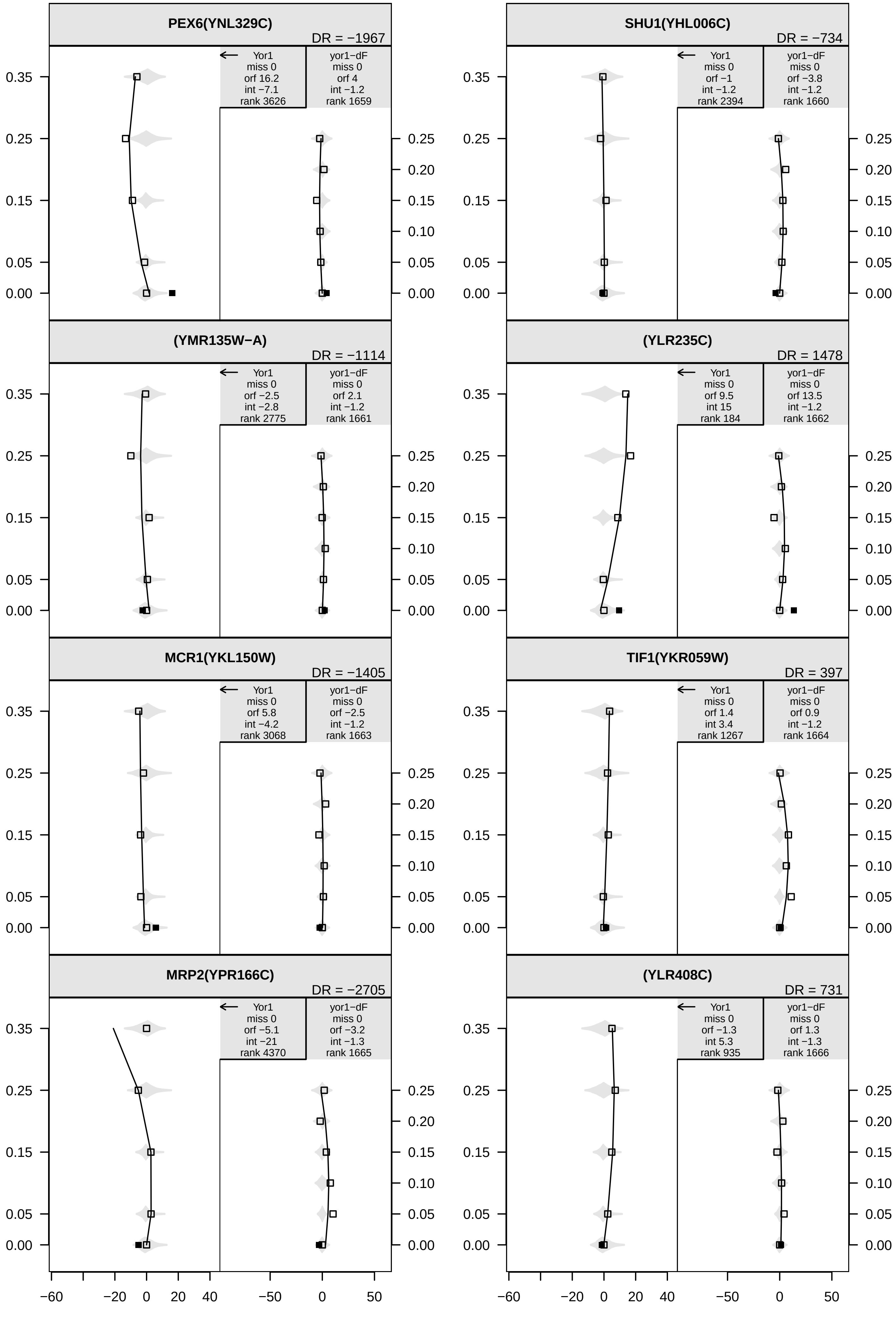
PEX6(YNL329C)
DR = −1967
Yor1
miss 0
orf 16.2
int −7.1
rank 3626
yor1−dF
miss 0
orf 4
int −1.2
rank 1659
SHU1(YHL006C)
DR = −734
Yor1
miss 0
orf −1
int −1.2
rank 2394
yor1−dF
miss 0
orf −3.8
int −1.2
rank 1660
(YMR135W−A)
DR = −1114
Yor1
miss 0
orf −2.5
int −2.8
rank 2775
yor1−dF
miss 0
orf 2.1
int −1.2
rank 1661
(YLR235C)
DR = 1478
Yor1
miss 0
orf 9.5
int 15
rank 184
yor1−dF
miss 0
orf 13.5
int −1.2
rank 1662
MCR1(YKL150W)
DR = −1405
Yor1
miss 0
orf 5.8
int −4.2
rank 3068
yor1−dF
miss 0
orf −2.5
int −1.2
rank 1663
TIF1(YKR059W)
DR = 397
Yor1
miss 0
orf 1.4
int 3.4
rank 1267
yor1−dF
miss 0
orf 0.9
int −1.2
rank 1664
MRP2(YPR166C)
DR = −2705
Yor1
miss 0
orf −5.1
int −21
rank 4370
yor1−dF
miss 0
orf −3.2
int −1.3
rank 1665
(YLR408C)
DR = 731
Yor1
miss 0
orf −1.3
int 5.3
rank 935
yor1−dF
miss 0
orf 1.3
int −1.3
rank 1666
0.35
0.25
0.20
0.15
0.10
0.05
0.00
−60
−20
0
20
40
−50
0
50

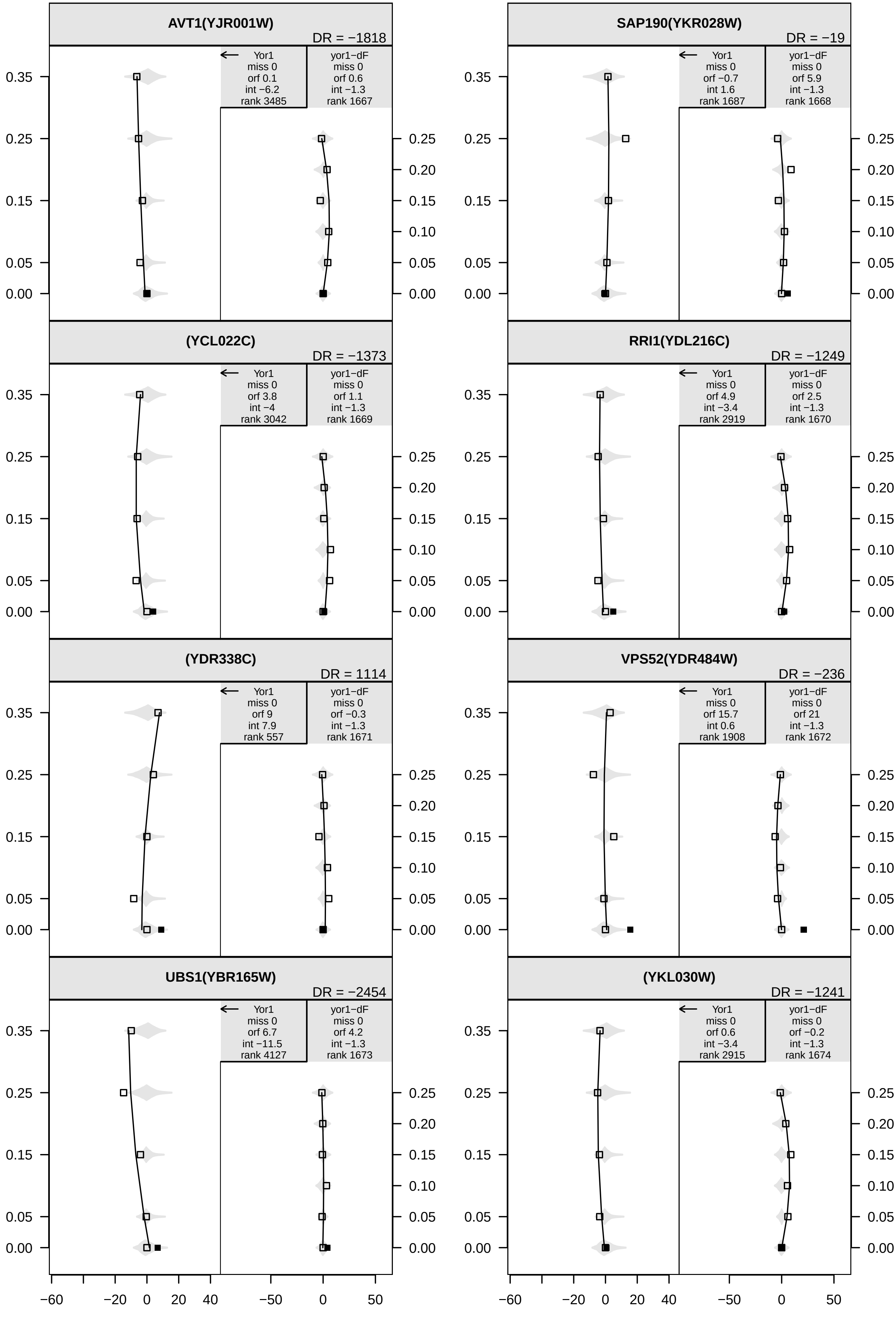
AVT1(YJR001W)
DR = −1818
Yor1
miss 0
orf 0.1
int −6.2
rank 3485
yor1−dF
miss 0
orf 0.6
int −1.3
rank 1667
SAP190(YKR028W)
DR = −19
Yor1
miss 0
orf −0.7
int 1.6
rank 1687
yor1−dF
miss 0
orf 5.9
int −1.3
rank 1668
(YCL022C)
DR = −1373
Yor1
miss 0
orf 3.8
int −4
rank 3042
yor1−dF
miss 0
orf 1.1
int −1.3
rank 1669
RRI1(YDL216C)
DR = −1249
Yor1
miss 0
orf 4.9
int −3.4
rank 2919
yor1−dF
miss 0
orf 2.5
int −1.3
rank 1670
(YDR338C)
DR = 1114
Yor1
miss 0
orf 9
int 7.9
rank 557
yor1−dF
miss 0
orf −0.3
int −1.3
rank 1671
VPS52(YDR484W)
DR = −236
Yor1
miss 0
orf 15.7
int 0.6
rank 1908
yor1−dF
miss 0
orf 21
int −1.3
rank 1672
UBS1(YBR165W)
DR = −2454
Yor1
miss 0
orf 6.7
int −11.5
rank 4127
yor1−dF
miss 0
orf 4.2
int −1.3
rank 1673
(YKL030W)
DR = −1241
Yor1
miss 0
orf 0.6
int −3.4
rank 2915
yor1−dF
miss 0
orf −0.2
int −1.3
rank 1674
0.35
0.25
0.20
0.15
0.10
0.05
0.00
−60
−20
0
20
40
−50
0
50

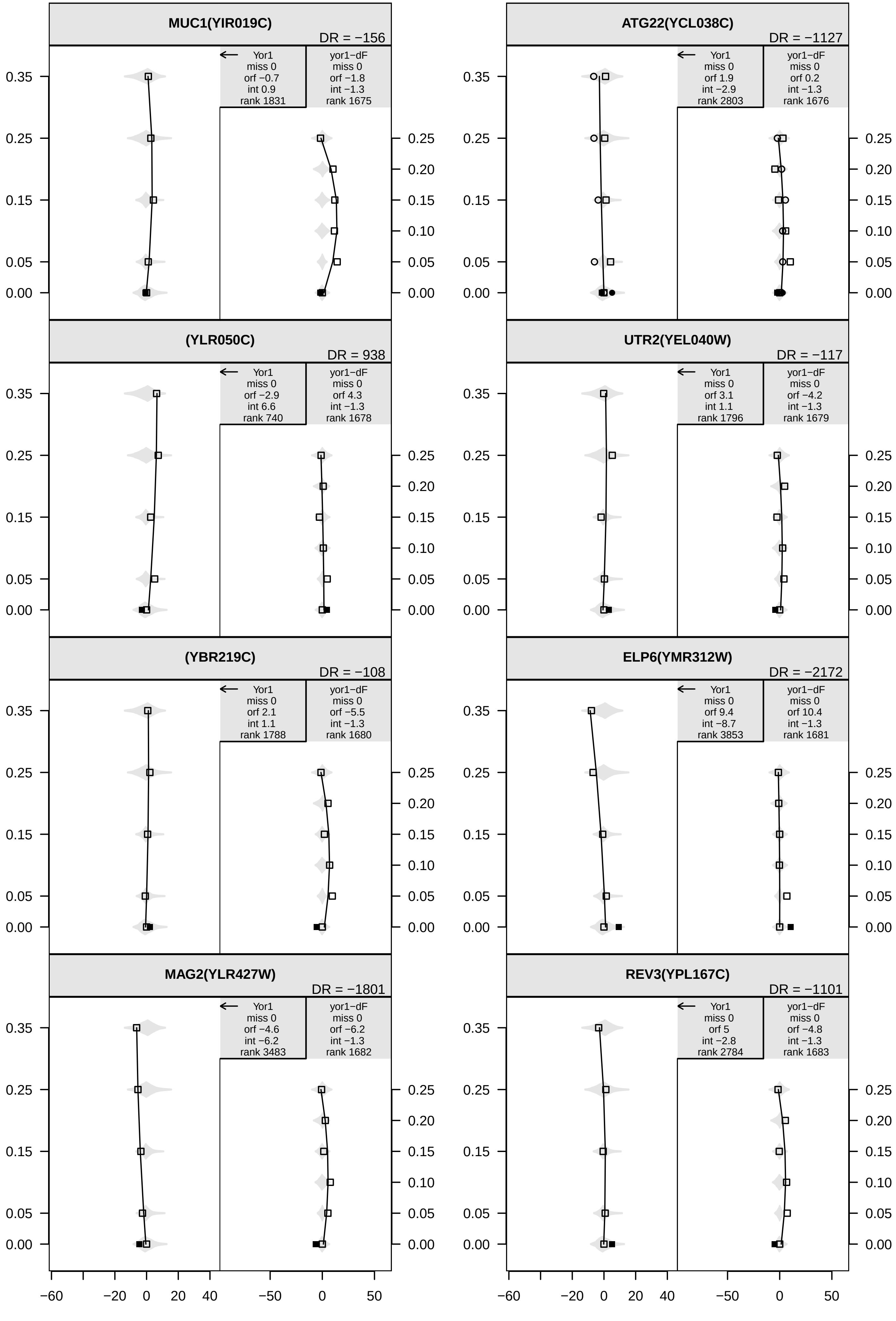

MUC1(YIR019C)
DR = −156
Yor1 miss 0 orf −0.7 int 0.9 rank 1831
yor1−dF miss 0 orf −1.8 int −1.3 rank 1675
ATG22(YCL038C)
DR = −1127
Yor1 miss 0 orf 1.9 int −2.9 rank 2803
yor1−dF miss 0 orf 0.2 int −1.3 rank 1676
(YLR050C)
DR = 938
Yor1 miss 0 orf −2.9 int 6.6 rank 740
yor1−dF miss 0 orf 4.3 int −1.3 rank 1678
UTR2(YEL040W)
DR = −117
Yor1 miss 0 orf 3.1 int 1.1 rank 1796
yor1−dF miss 0 orf −4.2 int −1.3 rank 1679
(YBR219C)
DR = −108
Yor1 miss 0 orf 2.1 int 1.1 rank 1788
yor1−dF miss 0 orf −5.5 int −1.3 rank 1680
ELP6(YMR312W)
DR = −2172
Yor1 miss 0 orf 9.4 int −8.7 rank 3853
yor1−dF miss 0 orf 10.4 int −1.3 rank 1681
MAG2(YLR427W)
DR = −1801
Yor1 miss 0 orf −4.6 int −6.2 rank 3483
yor1−dF miss 0 orf −6.2 int −1.3 rank 1682
REV3(YPL167C)
DR = −1101
Yor1 miss 0 orf 5 int −2.8 rank 2784
yor1−dF miss 0 orf −4.8 int −1.3 rank 1683
0.35 0.25 0.15 0.05 0.00
0.25 0.20 0.15 0.10 0.05 0.00
−60 −20 0 20 40
−50 0 50

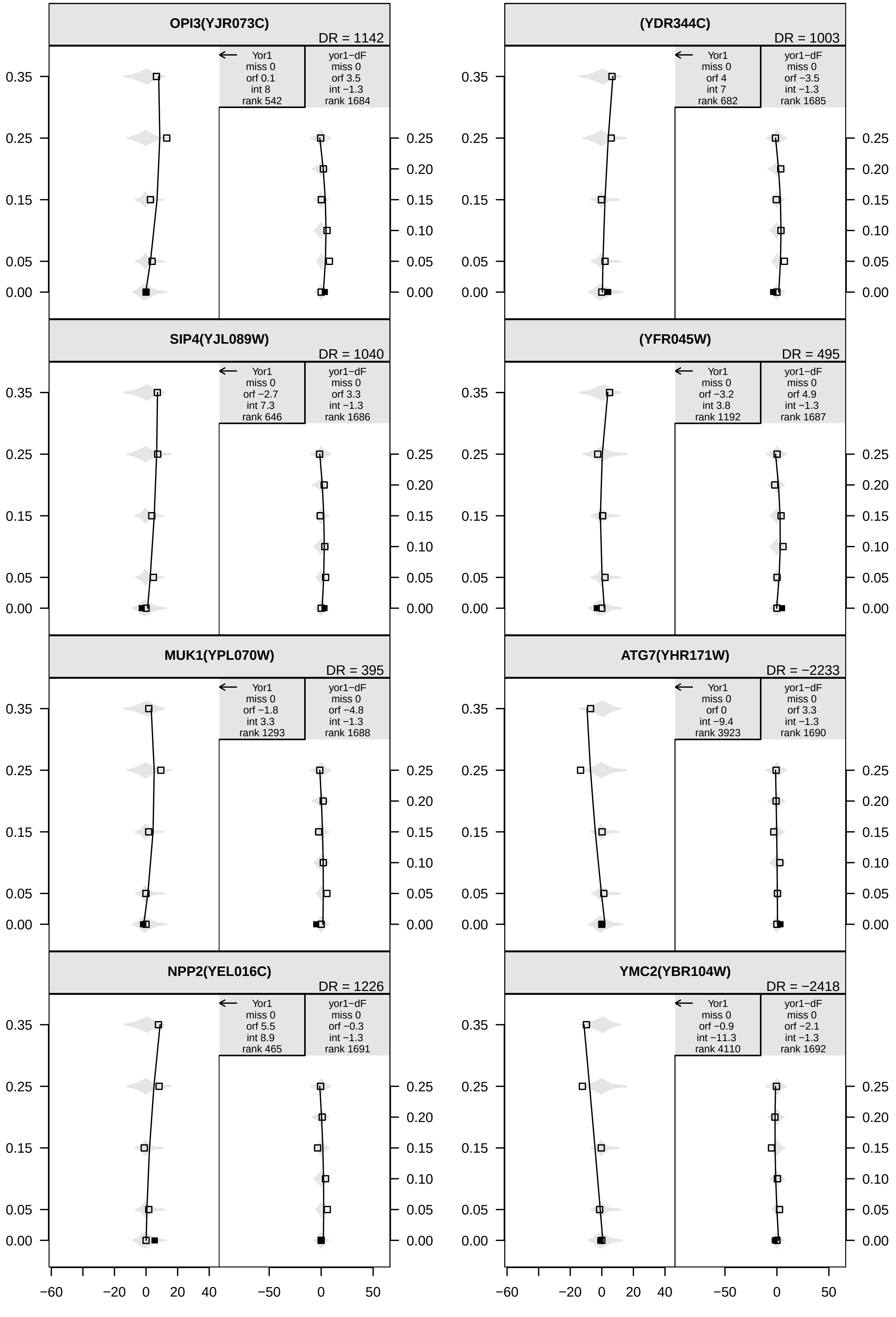
OPI3(YJR073C)
DR = 1142
Yor1 miss 0 orf 0.1 int 8 rank 542
yor1−dF miss 0 orf 3.5 int −1.3 rank 1684
(YDR344C)
DR = 1003
Yor1 miss 0 orf 4 int 7 rank 682
yor1−dF miss 0 orf −3.5 int −1.3 rank 1685
SIP4(YJL089W)
DR = 1040
Yor1 miss 0 orf −2.7 int 7.3 rank 646
yor1−dF miss 0 orf 3.3 int −1.3 rank 1686
(YFR045W)
DR = 495
Yor1 miss 0 orf −3.2 int 3.8 rank 1192
yor1−dF miss 0 orf 4.9 int −1.3 rank 1687
MUK1(YPL070W)
DR = 395
Yor1 miss 0 orf −1.8 int 3.3 rank 1293
yor1−dF miss 0 orf −4.8 int −1.3 rank 1688
ATG7(YHR171W)
DR = −2233
Yor1 miss 0 orf 0 int −9.4 rank 3923
yor1−dF miss 0 orf 3.3 int −1.3 rank 1690
NPP2(YEL016C)
DR = 1226
Yor1 miss 0 orf 5.5 int 8.9 rank 465
yor1−dF miss 0 orf −0.3 int −1.3 rank 1691
YMC2(YBR104W)
DR = −2418
Yor1 miss 0 orf −0.9 int −11.3 rank 4110
yor1−dF miss 0 orf −2.1 int −1.3 rank 1692
0.35 0.25 0.15 0.05 0.00
0.25 0.20 0.15 0.10 0.05 0.00
−60 −20 0 20 40 −50 0 50

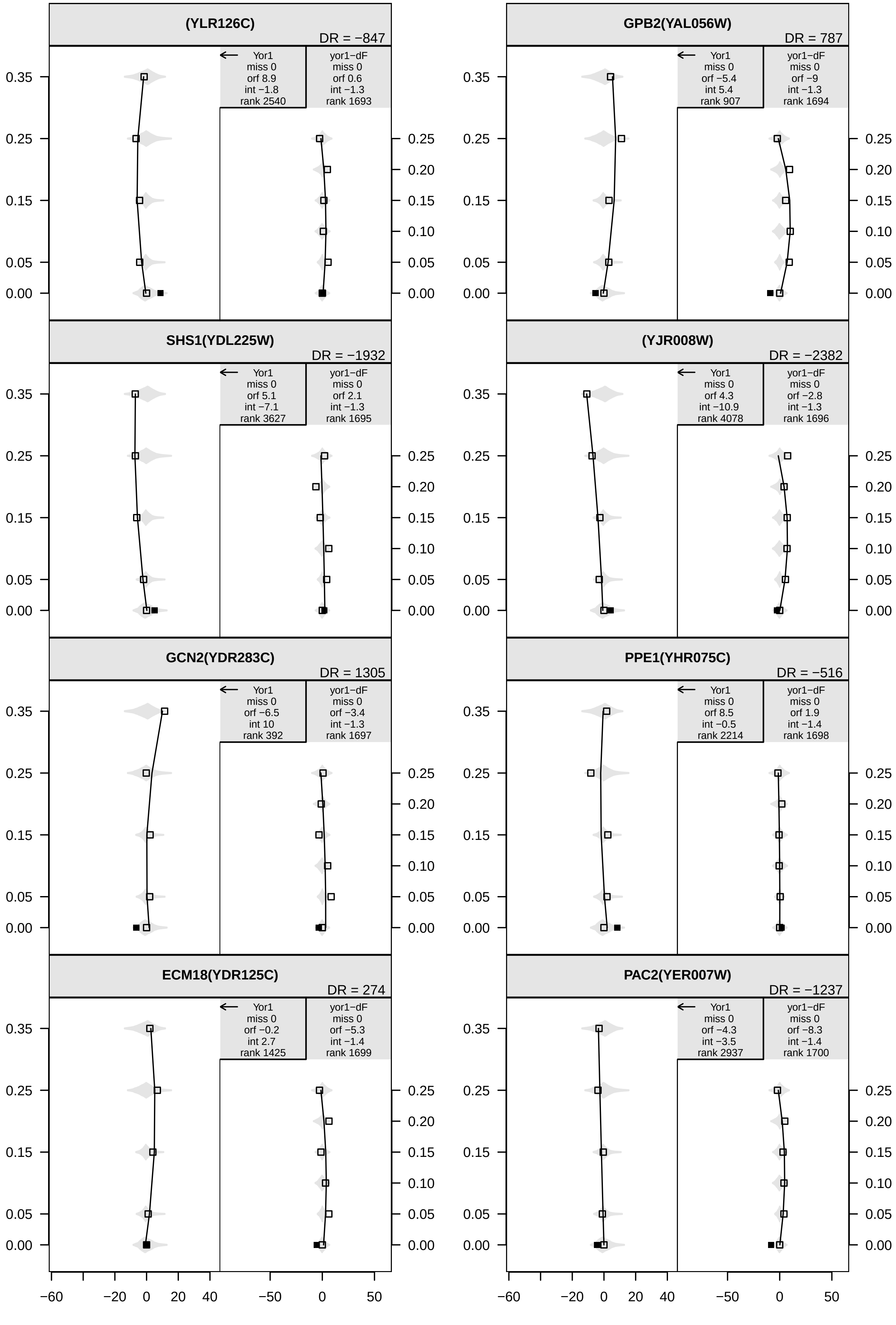

(YLR126C)
DR = −847
Yor1
miss 0
orf 8.9
int −1.8
rank 2540
yor1−dF
miss 0
orf 0.6
int −1.3
rank 1693
GPB2(YAL056W)
DR = 787
Yor1
miss 0
orf −5.4
int 5.4
rank 907
yor1−dF
miss 0
orf −9
int −1.3
rank 1694
SHS1(YDL225W)
DR = −1932
Yor1
miss 0
orf 5.1
int −7.1
rank 3627
yor1−dF
miss 0
orf 2.1
int −1.3
rank 1695
(YJR008W)
DR = −2382
Yor1
miss 0
orf 4.3
int −10.9
rank 4078
yor1−dF
miss 0
orf −2.8
int −1.3
rank 1696
GCN2(YDR283C)
DR = 1305
Yor1
miss 0
orf −6.5
int 10
rank 392
yor1−dF
miss 0
orf −3.4
int −1.3
rank 1697
PPE1(YHR075C)
DR = −516
Yor1
miss 0
orf 8.5
int −0.5
rank 2214
yor1−dF
miss 0
orf 1.9
int −1.4
rank 1698
ECM18(YDR125C)
DR = 274
Yor1
miss 0
orf −0.2
int 2.7
rank 1425
yor1−dF
miss 0
orf −5.3
int −1.4
rank 1699
PAC2(YER007W)
DR = −1237
Yor1
miss 0
orf −4.3
int −3.5
rank 2937
yor1−dF
miss 0
orf −8.3
int −1.4
rank 1700
0.35
0.25
0.15
0.05
0.00
0.20
0.10
−60
−20
0
20
40
−50
50

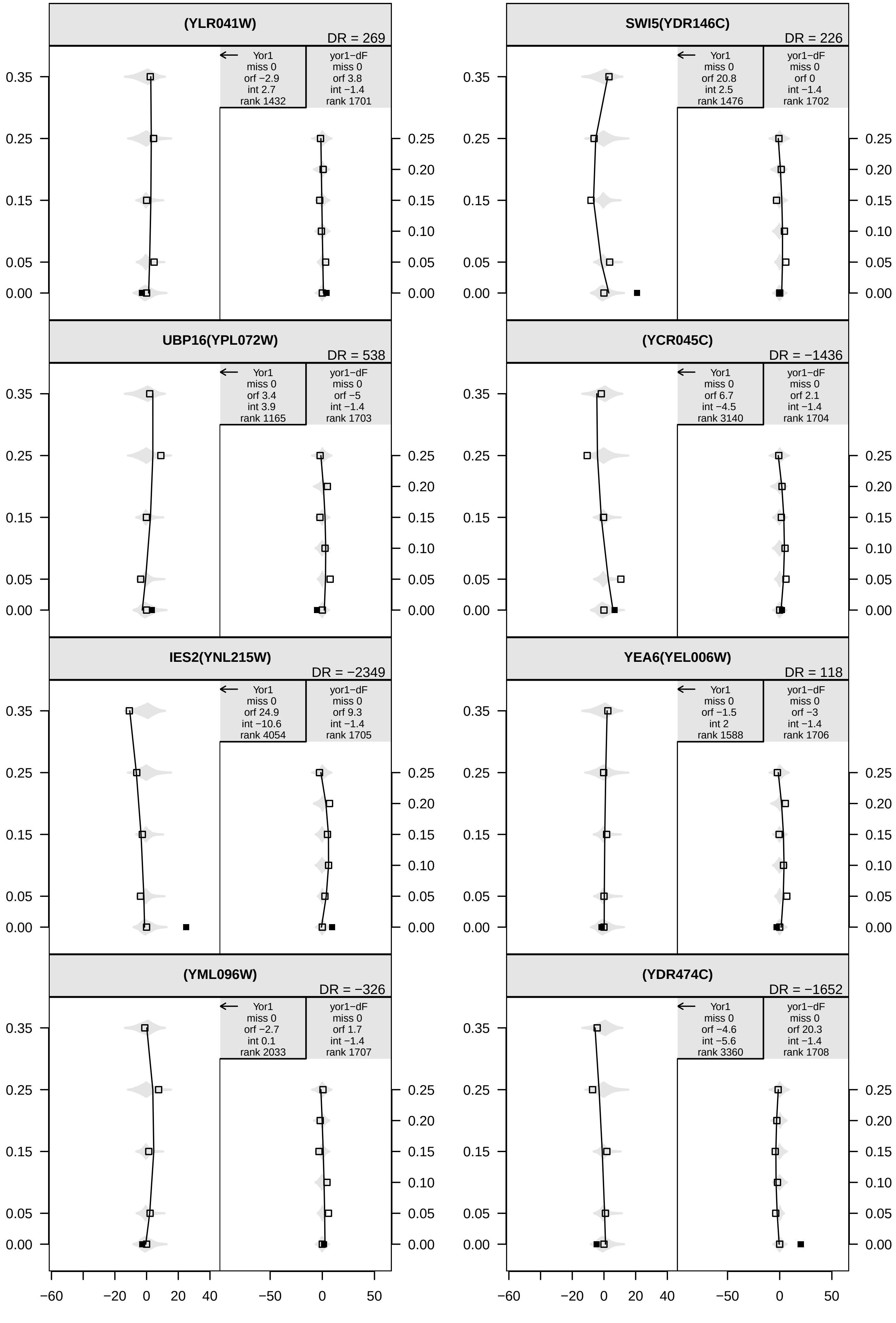

(YLR041W)
DR = 269
Yor1
miss 0
orf −2.9
int 2.7
rank 1432
yor1−dF
miss 0
orf 3.8
int −1.4
rank 1701
SWI5(YDR146C)
DR = 226
Yor1
miss 0
orf 20.8
int 2.5
rank 1476
yor1−dF
miss 0
orf 0
int −1.4
rank 1702
UBP16(YPL072W)
DR = 538
Yor1
miss 0
orf 3.4
int 3.9
rank 1165
yor1−dF
miss 0
orf −5
int −1.4
rank 1703
(YCR045C)
DR = −1436
Yor1
miss 0
orf 6.7
int −4.5
rank 3140
yor1−dF
miss 0
orf 2.1
int −1.4
rank 1704
IES2(YNL215W)
DR = −2349
Yor1
miss 0
orf 24.9
int −10.6
rank 4054
yor1−dF
miss 0
orf 9.3
int −1.4
rank 1705
YEA6(YEL006W)
DR = 118
Yor1
miss 0
orf −1.5
int 2
rank 1588
yor1−dF
miss 0
orf −3
int −1.4
rank 1706
(YML096W)
DR = −326
Yor1
miss 0
orf −2.7
int 0.1
rank 2033
yor1−dF
miss 0
orf 1.7
int −1.4
rank 1707
(YDR474C)
DR = −1652
Yor1
miss 0
orf −4.6
int −5.6
rank 3360
yor1−dF
miss 0
orf 20.3
int −1.4
rank 1708
0.35 0.25 0.20 0.15 0.10 0.05 0.00
−60 −20 0 20 40 −50 0 50

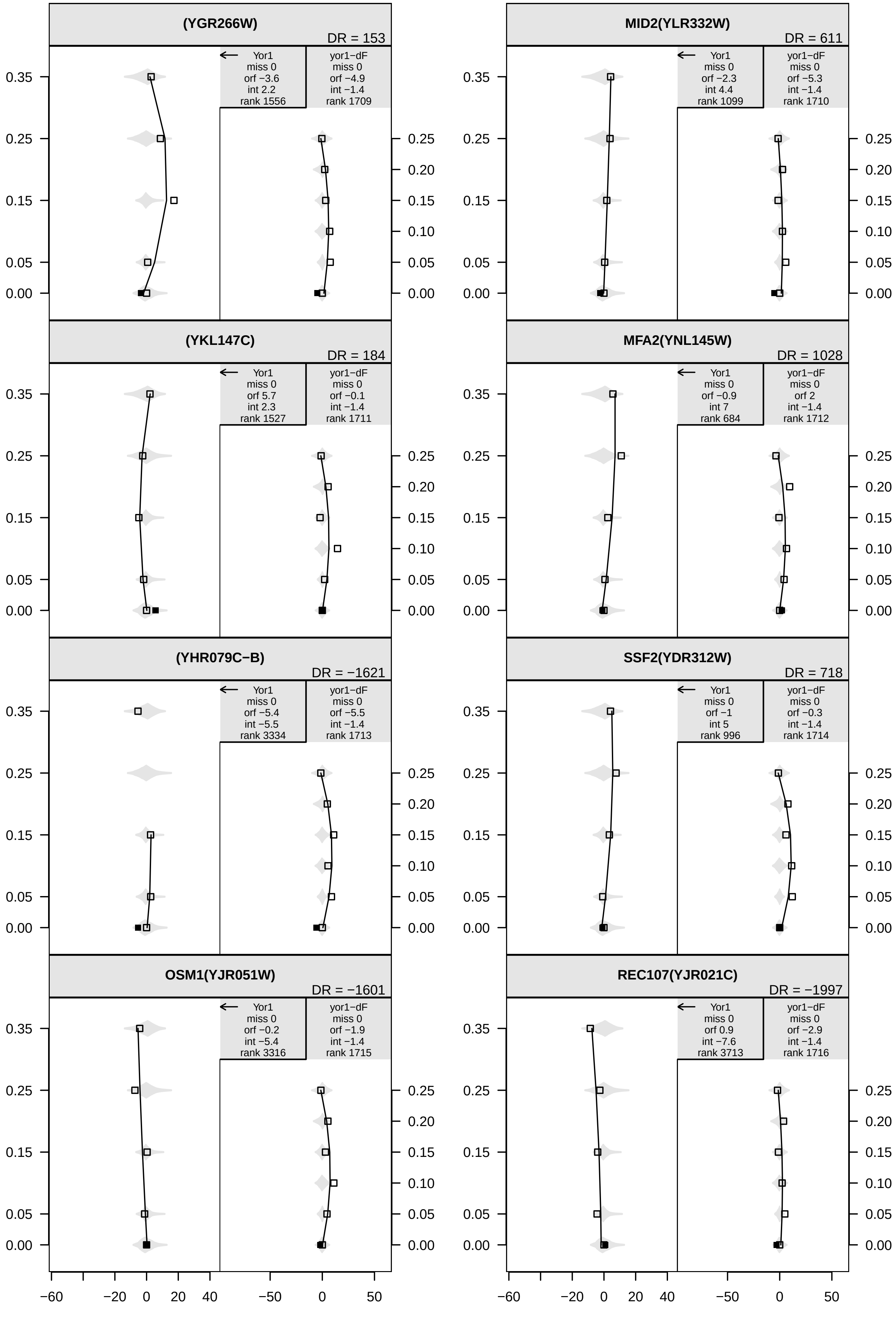
(YGR266W)
DR = 153
Yor1 miss 0 orf −3.6 int 2.2 rank 1556
yor1−dF miss 0 orf −4.9 int −1.4 rank 1709
MID2(YLR332W)
DR = 611
Yor1 miss 0 orf −2.3 int 4.4 rank 1099
yor1−dF miss 0 orf −5.3 int −1.4 rank 1710
(YKL147C)
DR = 184
Yor1 miss 0 orf 5.7 int 2.3 rank 1527
yor1−dF miss 0 orf −0.1 int −1.4 rank 1711
MFA2(YNL145W)
DR = 1028
Yor1 miss 0 orf −0.9 int 7 rank 684
yor1−dF miss 0 orf 2 int −1.4 rank 1712
(YHR079C−B)
DR = −1621
Yor1 miss 0 orf −5.4 int −5.5 rank 3334
yor1−dF miss 0 orf −5.5 int −1.4 rank 1713
SSF2(YDR312W)
DR = 718
Yor1 miss 0 orf −1 int 5 rank 996
yor1−dF miss 0 orf −0.3 int −1.4 rank 1714
OSM1(YJR051W)
DR = −1601
Yor1 miss 0 orf −0.2 int −5.4 rank 3316
yor1−dF miss 0 orf −1.9 int −1.4 rank 1715
REC107(YJR021C)
DR = −1997
Yor1 miss 0 orf 0.9 int −7.6 rank 3713
yor1−dF miss 0 orf −2.9 int −1.4 rank 1716
0.35 0.25 0.15 0.05 0.00
0.25 0.20 0.15 0.10 0.05 0.00
−60 −20 0 20 40
−50 0 50

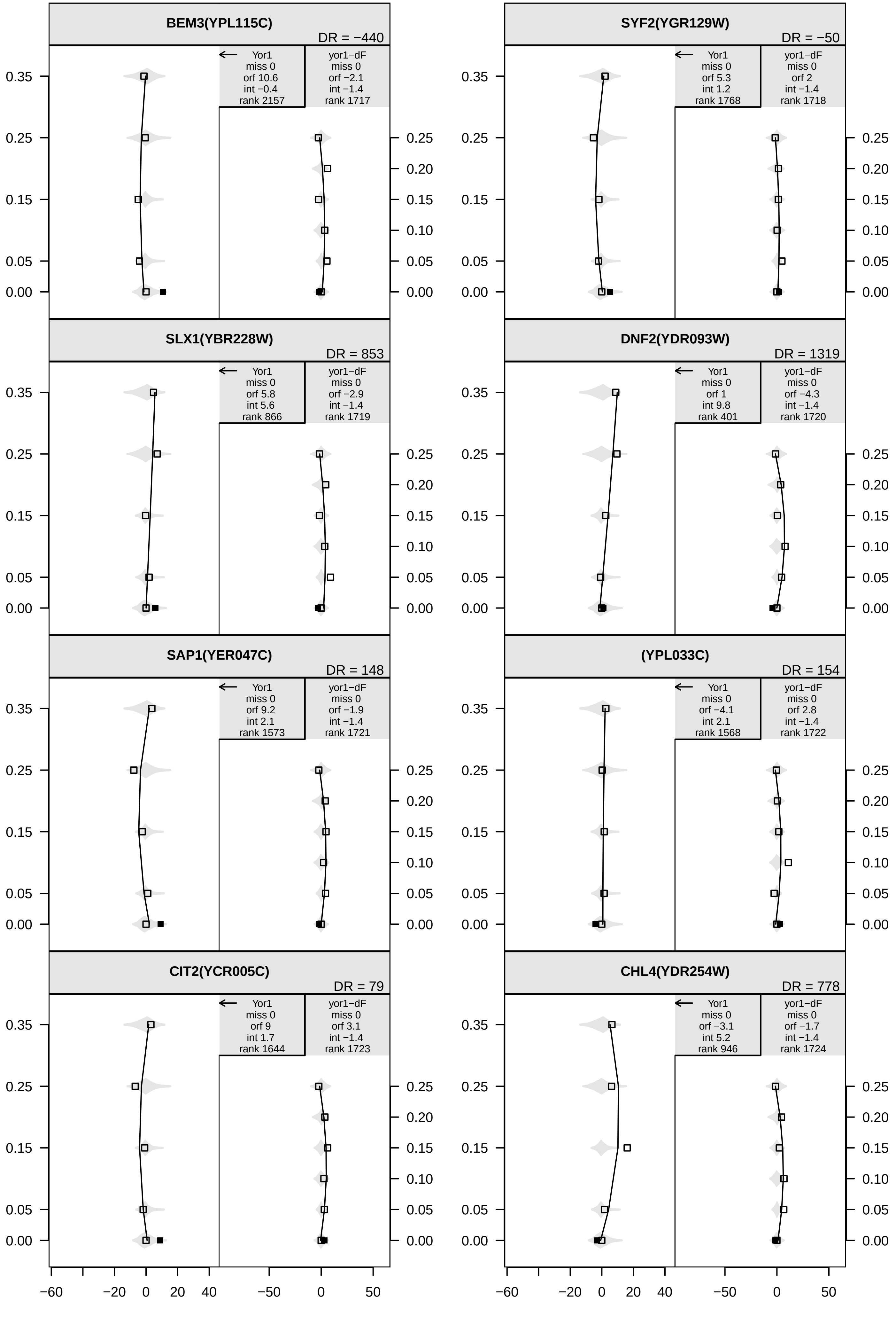

BEM3(YPL115C)
DR = −440
Yor1 miss 0 orf 10.6 int −0.4 rank 2157
yor1−dF miss 0 orf −2.1 int −1.4 rank 1717
SYF2(YGR129W)
DR = −50
Yor1 miss 0 orf 5.3 int 1.2 rank 1768
yor1−dF miss 0 orf 2 int −1.4 rank 1718
SLX1(YBR228W)
DR = 853
Yor1 miss 0 orf 5.8 int 5.6 rank 866
yor1−dF miss 0 orf −2.9 int −1.4 rank 1719
DNF2(YDR093W)
DR = 1319
Yor1 miss 0 orf 1 int 9.8 rank 401
yor1−dF miss 0 orf −4.3 int −1.4 rank 1720
SAP1(YER047C)
DR = 148
Yor1 miss 0 orf 9.2 int 2.1 rank 1573
yor1−dF miss 0 orf −1.9 int −1.4 rank 1721
(YPL033C)
DR = 154
Yor1 miss 0 orf −4.1 int 2.1 rank 1568
yor1−dF miss 0 orf 2.8 int −1.4 rank 1722
CIT2(YCR005C)
DR = 79
Yor1 miss 0 orf 9 int 1.7 rank 1644
yor1−dF miss 0 orf 3.1 int −1.4 rank 1723
CHL4(YDR254W)
DR = 778
Yor1 miss 0 orf −3.1 int 5.2 rank 946
yor1−dF miss 0 orf −1.7 int −1.4 rank 1724
0.00 0.05 0.10 0.15 0.20 0.25 0.35
−60 −20 0 20 40 −50 0 50

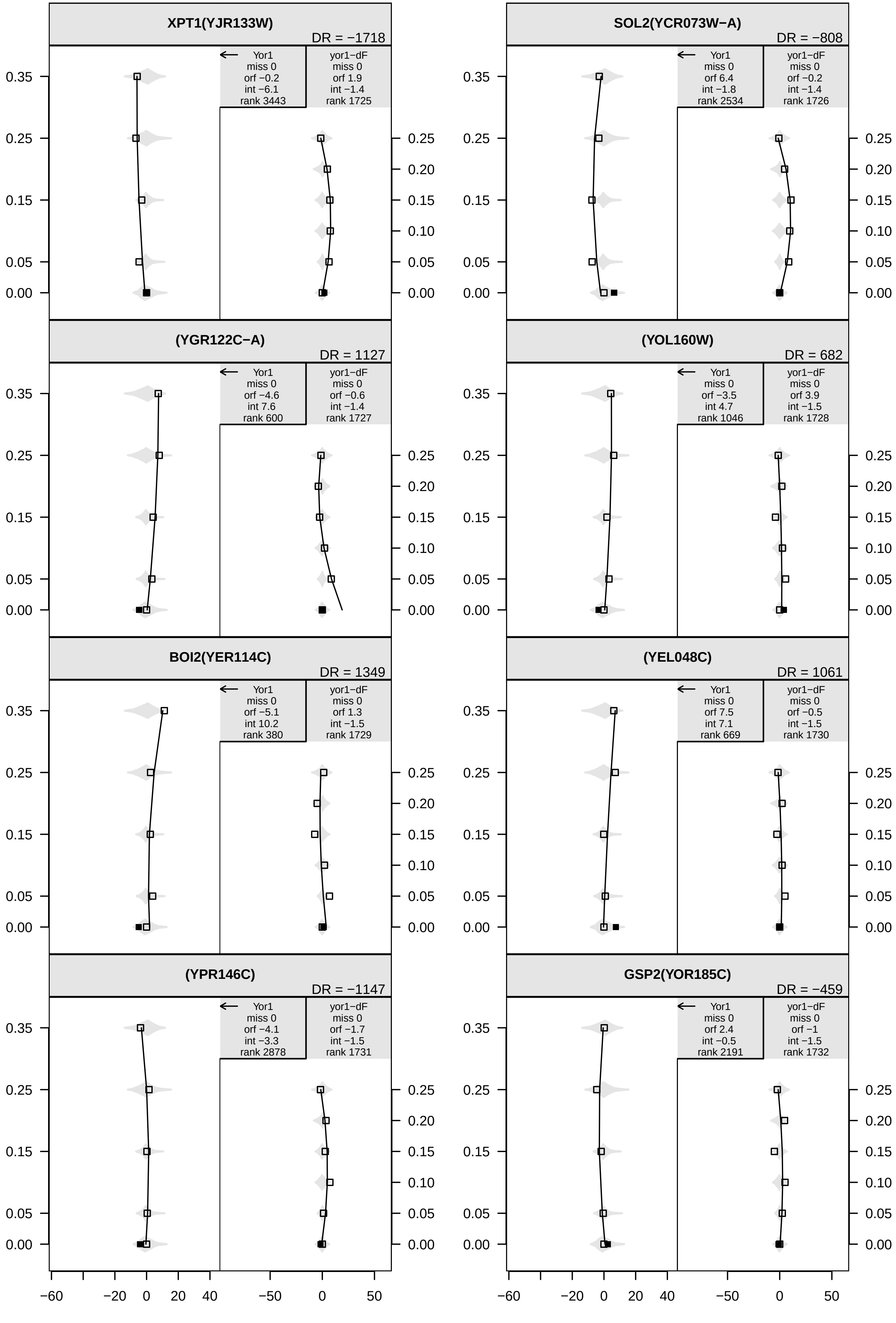
XPT1(YJR133W)
DR = −1718
Yor1
miss 0
orf −0.2
int −6.1
rank 3443
yor1−dF
miss 0
orf 1.9
int −1.4
rank 1725
SOL2(YCR073W−A)
DR = −808
Yor1
miss 0
orf 6.4
int −1.8
rank 2534
yor1−dF
miss 0
orf −0.2
int −1.4
rank 1726
(YGR122C−A)
DR = 1127
Yor1
miss 0
orf −4.6
int 7.6
rank 600
yor1−dF
miss 0
orf −0.6
int −1.4
rank 1727
(YOL160W)
DR = 682
Yor1
miss 0
orf −3.5
int 4.7
rank 1046
yor1−dF
miss 0
orf 3.9
int −1.5
rank 1728
BOI2(YER114C)
DR = 1349
Yor1
miss 0
orf −5.1
int 10.2
rank 380
yor1−dF
miss 0
orf 1.3
int −1.5
rank 1729
(YEL048C)
DR = 1061
Yor1
miss 0
orf 7.5
int 7.1
rank 669
yor1−dF
miss 0
orf −0.5
int −1.5
rank 1730
(YPR146C)
DR = −1147
Yor1
miss 0
orf −4.1
int −3.3
rank 2878
yor1−dF
miss 0
orf −1.7
int −1.5
rank 1731
GSP2(YOR185C)
DR = −459
Yor1
miss 0
orf 2.4
int −0.5
rank 2191
yor1−dF
miss 0
orf −1
int −1.5
rank 1732
0.35
0.25
0.20
0.15
0.10
0.05
0.00
−60
−20
0
20
40
−50
0
50

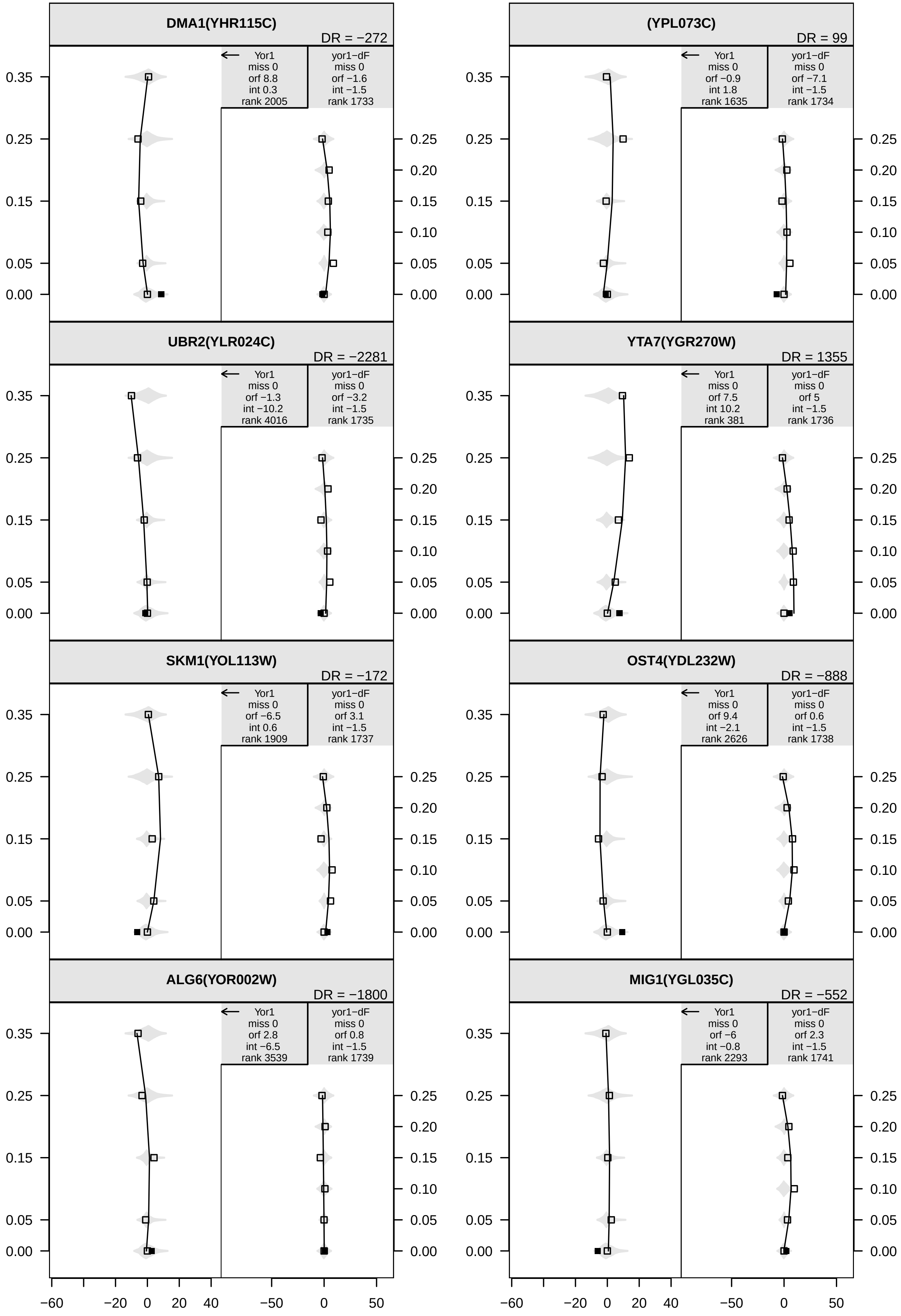

DMA1(YHR115C)
DR = −272
Yor1 miss 0 orf 8.8 int 0.3 rank 2005
yor1−dF miss 0 orf −1.6 int −1.5 rank 1733
(YPL073C)
DR = 99
Yor1 miss 0 orf −0.9 int 1.8 rank 1635
yor1−dF miss 0 orf −7.1 int −1.5 rank 1734
UBR2(YLR024C)
DR = −2281
Yor1 miss 0 orf −1.3 int −10.2 rank 4016
yor1−dF miss 0 orf −3.2 int −1.5 rank 1735
YTA7(YGR270W)
DR = 1355
Yor1 miss 0 orf 7.5 int 10.2 rank 381
yor1−dF miss 0 orf 5 int −1.5 rank 1736
SKM1(YOL113W)
DR = −172
Yor1 miss 0 orf −6.5 int 0.6 rank 1909
yor1−dF miss 0 orf 3.1 int −1.5 rank 1737
OST4(YDL232W)
DR = −888
Yor1 miss 0 orf 9.4 int −2.1 rank 2626
yor1−dF miss 0 orf 0.6 int −1.5 rank 1738
ALG6(YOR002W)
DR = −1800
Yor1 miss 0 orf 2.8 int −6.5 rank 3539
yor1−dF miss 0 orf 0.8 int −1.5 rank 1739
MIG1(YGL035C)
DR = −552
Yor1 miss 0 orf −6 int −0.8 rank 2293
yor1−dF miss 0 orf 2.3 int −1.5 rank 1741
0.35 0.25 0.20 0.15 0.10 0.05 0.00
−60 −20 0 20 40 −50 0 50

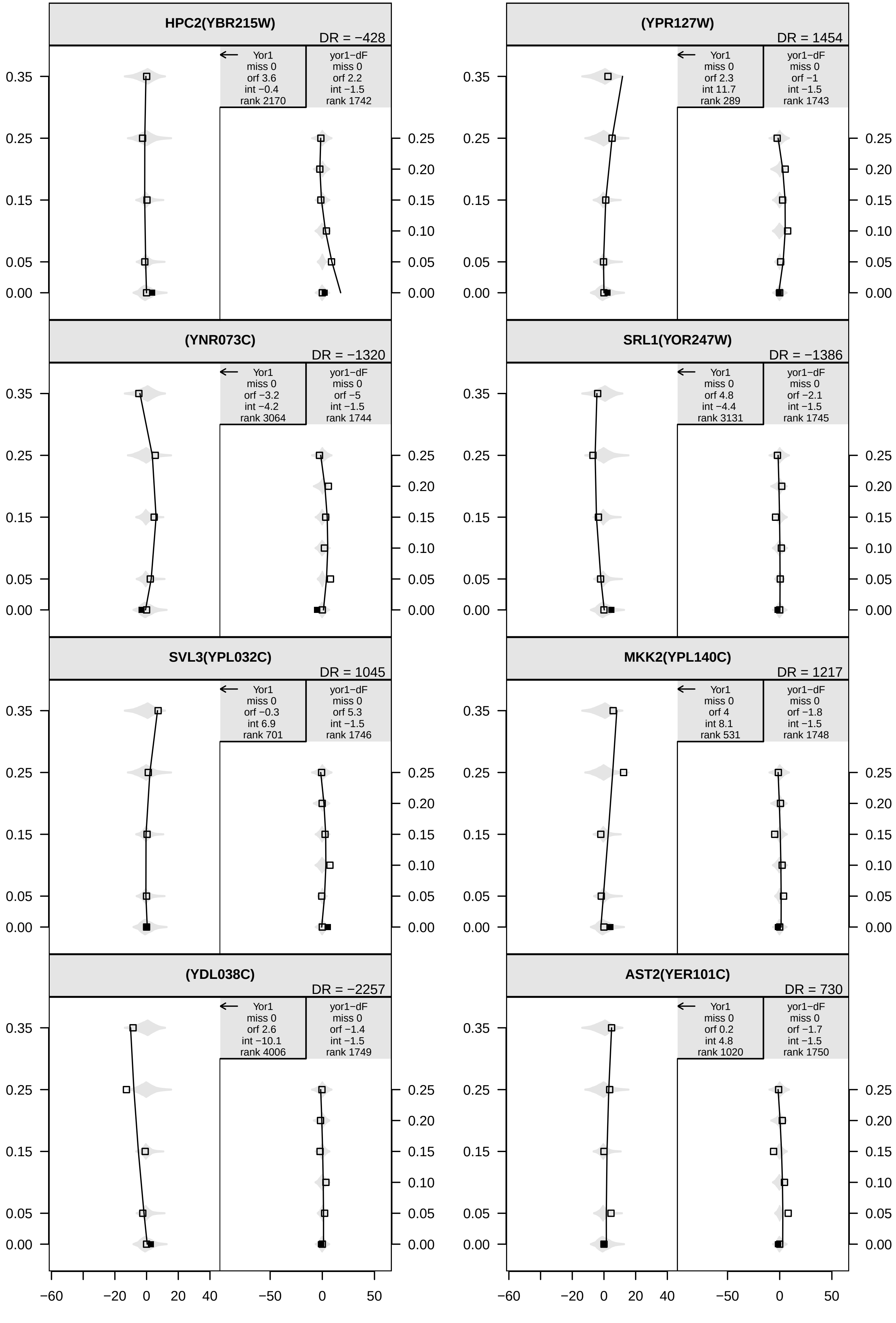

HPC2(YBR215W)
DR = −428
Yor1 miss 0 orf 3.6 int −0.4 rank 2170
yor1−dF miss 0 orf 2.2 int −1.5 rank 1742
(YPR127W)
DR = 1454
Yor1 miss 0 orf 2.3 int 11.7 rank 289
yor1−dF miss 0 orf −1 int −1.5 rank 1743
(YNR073C)
DR = −1320
Yor1 miss 0 orf −3.2 int −4.2 rank 3064
yor1−dF miss 0 orf −5 int −1.5 rank 1744
SRL1(YOR247W)
DR = −1386
Yor1 miss 0 orf 4.8 int −4.4 rank 3131
yor1−dF miss 0 orf −2.1 int −1.5 rank 1745
SVL3(YPL032C)
DR = 1045
Yor1 miss 0 orf −0.3 int 6.9 rank 701
yor1−dF miss 0 orf 5.3 int −1.5 rank 1746
MKK2(YPL140C)
DR = 1217
Yor1 miss 0 orf 4 int 8.1 rank 531
yor1−dF miss 0 orf −1.8 int −1.5 rank 1748
(YDL038C)
DR = −2257
Yor1 miss 0 orf 2.6 int −10.1 rank 4006
yor1−dF miss 0 orf −1.4 int −1.5 rank 1749
AST2(YER101C)
DR = 730
Yor1 miss 0 orf 0.2 int 4.8 rank 1020
yor1−dF miss 0 orf −1.7 int −1.5 rank 1750
0.35 0.25 0.15 0.05 0.00
0.25 0.20 0.15 0.10 0.05 0.00
−60 −20 0 20 40 −50 0 50

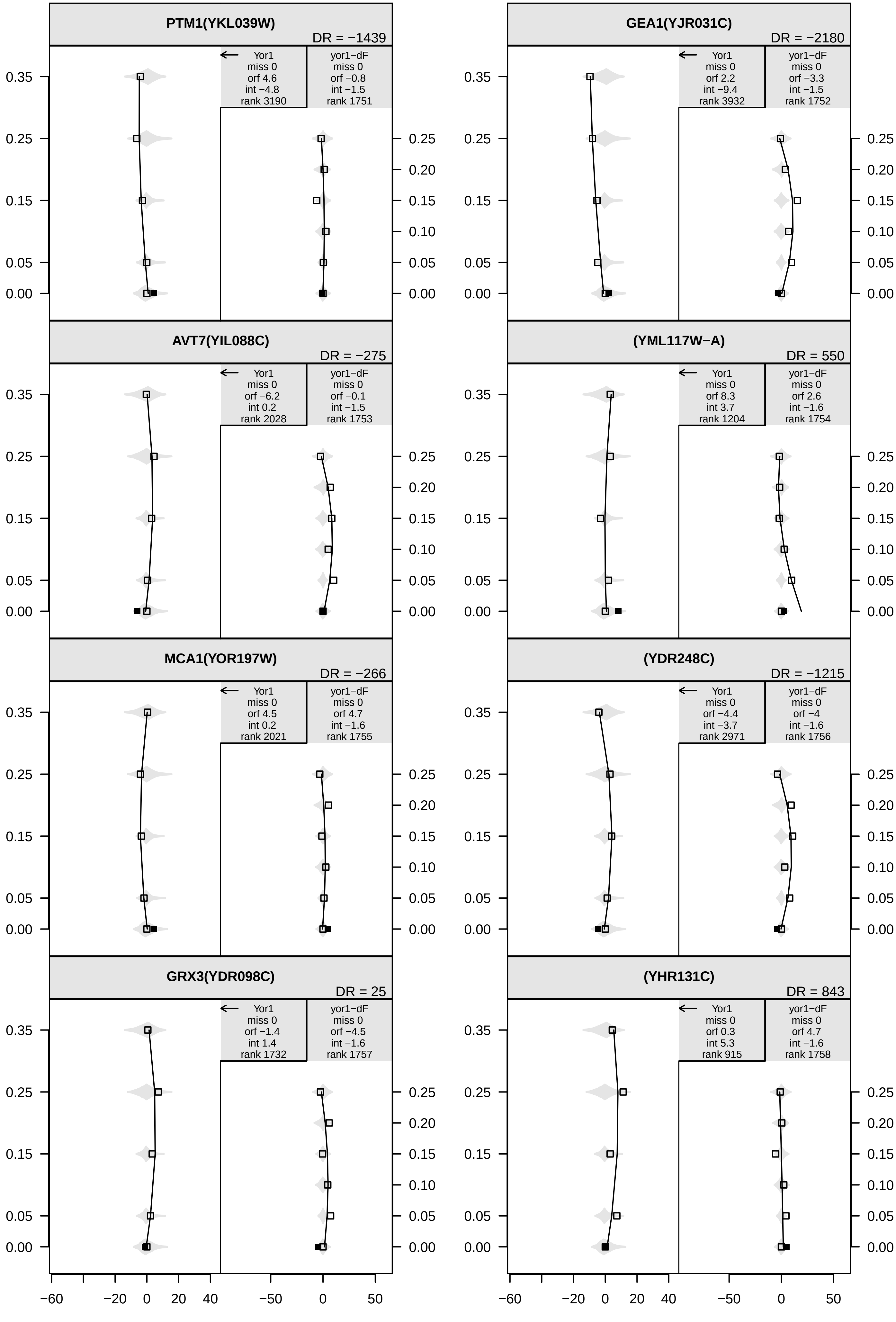
PTM1(YKL039W)
DR = −1439
Yor1
miss 0
orf 4.6
int −4.8
rank 3190
yor1−dF
miss 0
orf −0.8
int −1.5
rank 1751
GEA1(YJR031C)
DR = −2180
Yor1
miss 0
orf 2.2
int −9.4
rank 3932
yor1−dF
miss 0
orf −3.3
int −1.5
rank 1752
AVT7(YIL088C)
DR = −275
Yor1
miss 0
orf −6.2
int 0.2
rank 2028
yor1−dF
miss 0
orf −0.1
int −1.5
rank 1753
(YML117W−A)
DR = 550
Yor1
miss 0
orf 8.3
int 3.7
rank 1204
yor1−dF
miss 0
orf 2.6
int −1.6
rank 1754
MCA1(YOR197W)
DR = −266
Yor1
miss 0
orf 4.5
int 0.2
rank 2021
yor1−dF
miss 0
orf 4.7
int −1.6
rank 1755
(YDR248C)
DR = −1215
Yor1
miss 0
orf −4.4
int −3.7
rank 2971
yor1−dF
miss 0
orf −4
int −1.6
rank 1756
GRX3(YDR098C)
DR = 25
Yor1
miss 0
orf −1.4
int 1.4
rank 1732
yor1−dF
miss 0
orf −4.5
int −1.6
rank 1757
(YHR131C)
DR = 843
Yor1
miss 0
orf 0.3
int 5.3
rank 915
yor1−dF
miss 0
orf 4.7
int −1.6
rank 1758
0.35
0.25
0.20
0.15
0.10
0.05
0.00
−60
−20
0
20
40
−50
0
50

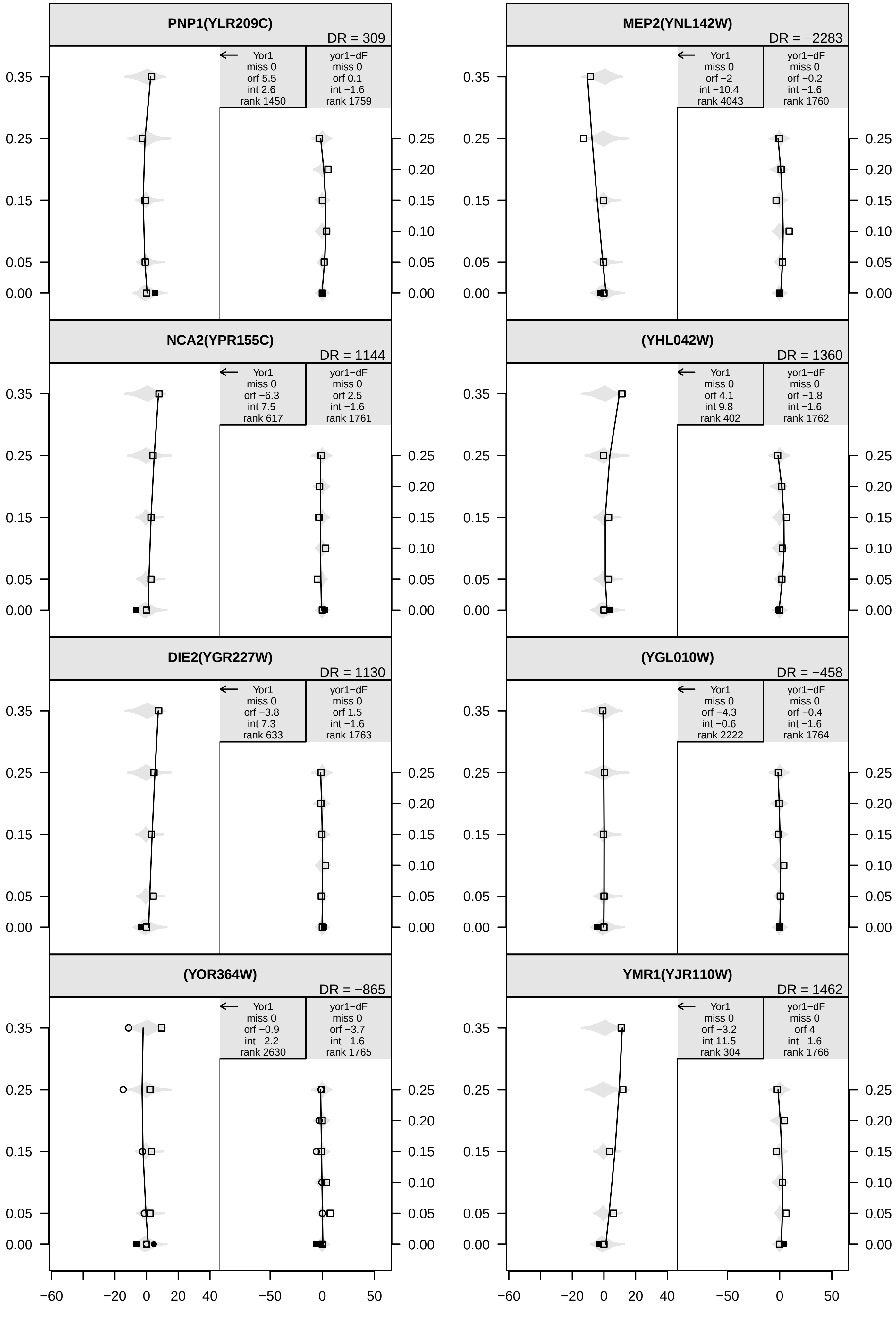
PNP1(YLR209C)
DR = 309
Yor1
miss 0
orf 5.5
int 2.6
rank 1450
yor1−dF
miss 0
orf 0.1
int −1.6
rank 1759
MEP2(YNL142W)
DR = −2283
Yor1
miss 0
orf −2
int −10.4
rank 4043
yor1−dF
miss 0
orf −0.2
int −1.6
rank 1760
NCA2(YPR155C)
DR = 1144
Yor1
miss 0
orf −6.3
int 7.5
rank 617
yor1−dF
miss 0
orf 2.5
int −1.6
rank 1761
(YHL042W)
DR = 1360
Yor1
miss 0
orf 4.1
int 9.8
rank 402
yor1−dF
miss 0
orf −1.8
int −1.6
rank 1762
DIE2(YGR227W)
DR = 1130
Yor1
miss 0
orf −3.8
int 7.3
rank 633
yor1−dF
miss 0
orf 1.5
int −1.6
rank 1763
(YGL010W)
DR = −458
Yor1
miss 0
orf −4.3
int −0.6
rank 2222
yor1−dF
miss 0
orf −0.4
int −1.6
rank 1764
(YOR364W)
DR = −865
Yor1
miss 0
orf −0.9
int −2.2
rank 2630
yor1−dF
miss 0
orf −3.7
int −1.6
rank 1765
YMR1(YJR110W)
DR = 1462
Yor1
miss 0
orf −3.2
int 11.5
rank 304
yor1−dF
miss 0
orf 4
int −1.6
rank 1766
0.35
0.25
0.15
0.05
0.00
0.20
0.10
−60
−20
0
20
40
−50
50

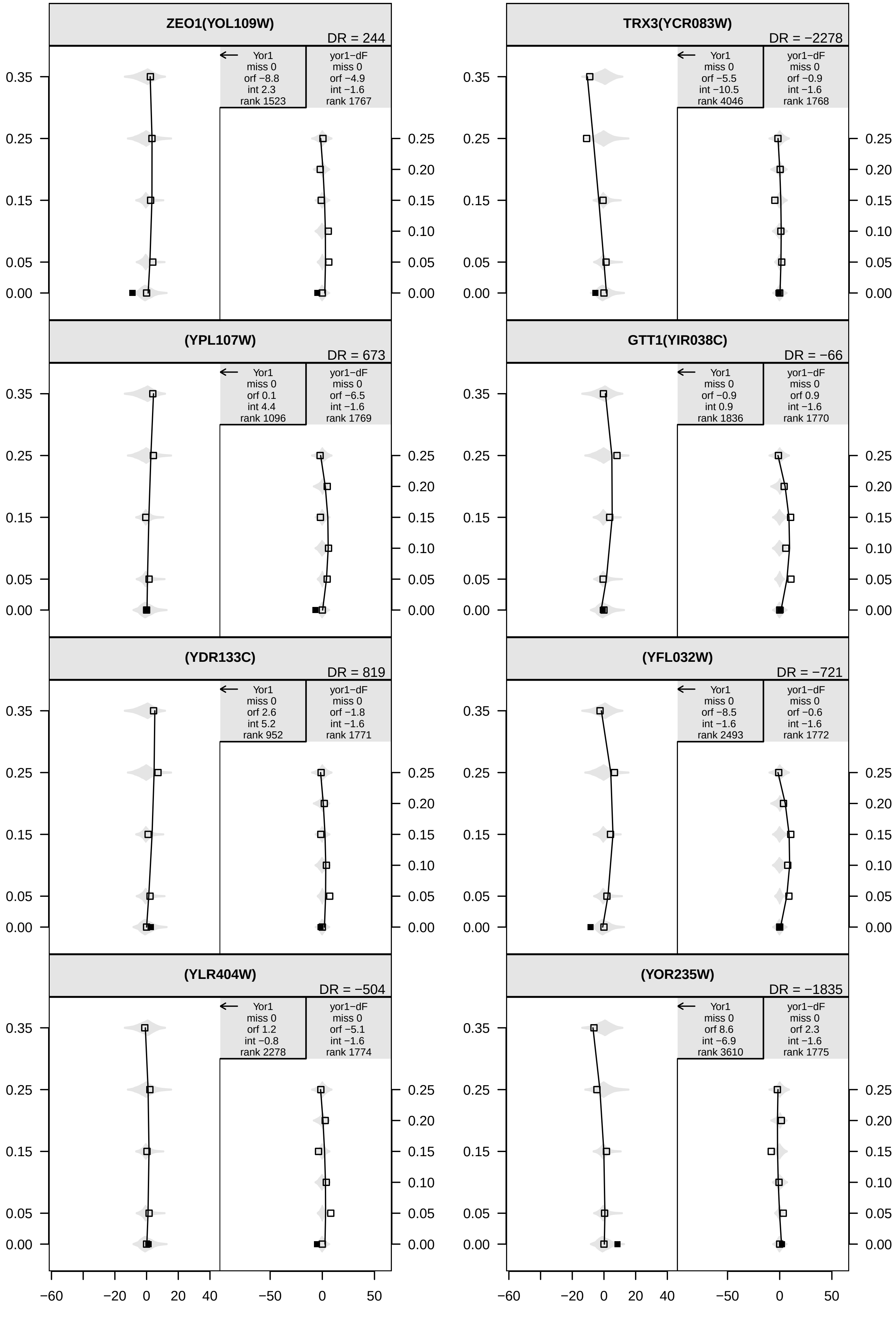
ZEO1(YOL109W)
DR = 244
Yor1 miss 0 orf −8.8 int 2.3 rank 1523
yor1−dF miss 0 orf −4.9 int −1.6 rank 1767
TRX3(YCR083W)
DR = −2278
Yor1 miss 0 orf −5.5 int −10.5 rank 4046
yor1−dF miss 0 orf −0.9 int −1.6 rank 1768
(YPL107W)
DR = 673
Yor1 miss 0 orf 0.1 int 4.4 rank 1096
yor1−dF miss 0 orf −6.5 int −1.6 rank 1769
GTT1(YIR038C)
DR = −66
Yor1 miss 0 orf −0.9 int 0.9 rank 1836
yor1−dF miss 0 orf 0.9 int −1.6 rank 1770
(YDR133C)
DR = 819
Yor1 miss 0 orf 2.6 int 5.2 rank 952
yor1−dF miss 0 orf −1.8 int −1.6 rank 1771
(YFL032W)
DR = −721
Yor1 miss 0 orf −8.5 int −1.6 rank 2493
yor1−dF miss 0 orf −0.6 int −1.6 rank 1772
(YLR404W)
DR = −504
Yor1 miss 0 orf 1.2 int −0.8 rank 2278
yor1−dF miss 0 orf −5.1 int −1.6 rank 1774
(YOR235W)
DR = −1835
Yor1 miss 0 orf 8.6 int −6.9 rank 3610
yor1−dF miss 0 orf 2.3 int −1.6 rank 1775
0.35 0.25 0.15 0.05 0.00
0.25 0.20 0.15 0.10 0.05 0.00
−60 −20 0 20 40 −50 0 50

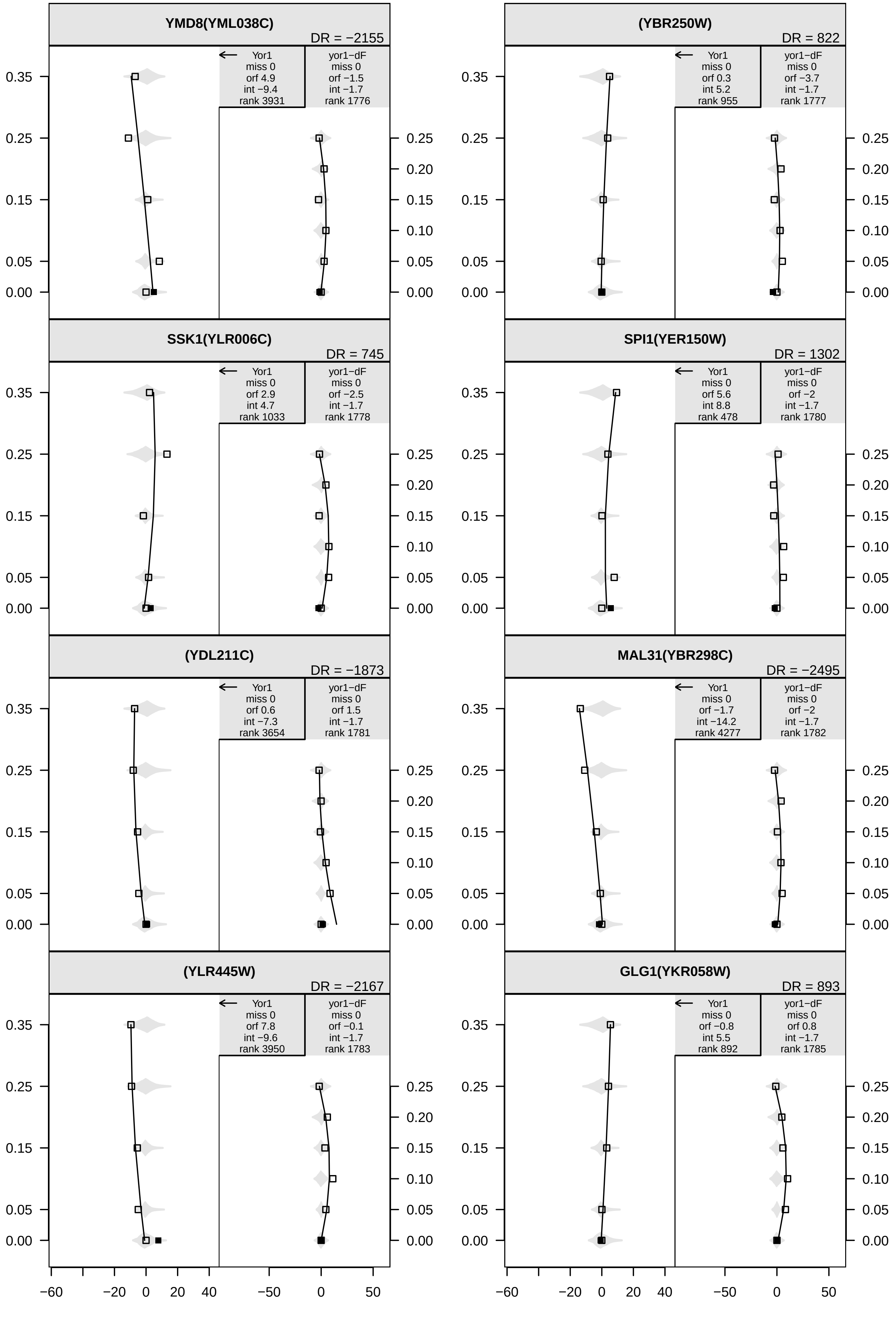

YMD8(YML038C)
DR = −2155
Yor1 miss 0 orf 4.9 int −9.4 rank 3931
yor1−dF miss 0 orf −1.5 int −1.7 rank 1776
(YBR250W)
DR = 822
Yor1 miss 0 orf 0.3 int 5.2 rank 955
yor1−dF miss 0 orf −3.7 int −1.7 rank 1777
SSK1(YLR006C)
DR = 745
Yor1 miss 0 orf 2.9 int 4.7 rank 1033
yor1−dF miss 0 orf −2.5 int −1.7 rank 1778
SPI1(YER150W)
DR = 1302
Yor1 miss 0 orf 5.6 int 8.8 rank 478
yor1−dF miss 0 orf −2 int −1.7 rank 1780
(YDL211C)
DR = −1873
Yor1 miss 0 orf 0.6 int −7.3 rank 3654
yor1−dF miss 0 orf 1.5 int −1.7 rank 1781
MAL31(YBR298C)
DR = −2495
Yor1 miss 0 orf −1.7 int −14.2 rank 4277
yor1−dF miss 0 orf −2 int −1.7 rank 1782
(YLR445W)
DR = −2167
Yor1 miss 0 orf 7.8 int −9.6 rank 3950
yor1−dF miss 0 orf −0.1 int −1.7 rank 1783
GLG1(YKR058W)
DR = 893
Yor1 miss 0 orf −0.8 int 5.5 rank 892
yor1−dF miss 0 orf 0.8 int −1.7 rank 1785
0.35 0.25 0.20 0.15 0.10 0.05 0.00
−60 −20 0 20 40 −50 0 50

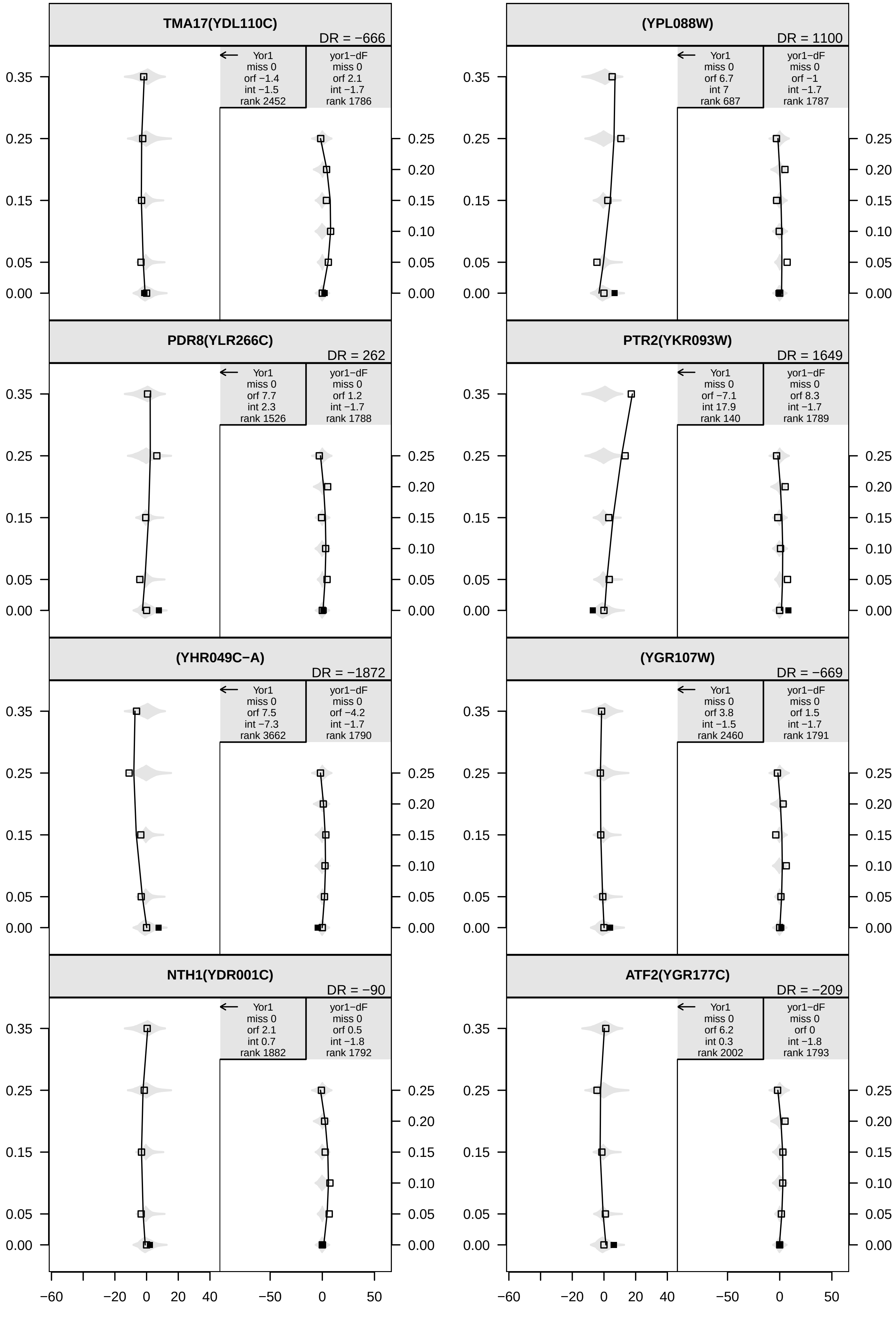

TMA17(YDL110C)
DR = −666
Yor1
miss 0
orf −1.4
int −1.5
rank 2452
yor1−dF
miss 0
orf 2.1
int −1.7
rank 1786
(YPL088W)
DR = 1100
Yor1
miss 0
orf 6.7
int 7
rank 687
yor1−dF
miss 0
orf −1
int −1.7
rank 1787
PDR8(YLR266C)
DR = 262
Yor1
miss 0
orf 7.7
int 2.3
rank 1526
yor1−dF
miss 0
orf 1.2
int −1.7
rank 1788
PTR2(YKR093W)
DR = 1649
Yor1
miss 0
orf −7.1
int 17.9
rank 140
yor1−dF
miss 0
orf 8.3
int −1.7
rank 1789
(YHR049C−A)
DR = −1872
Yor1
miss 0
orf 7.5
int −7.3
rank 3662
yor1−dF
miss 0
orf −4.2
int −1.7
rank 1790
(YGR107W)
DR = −669
Yor1
miss 0
orf 3.8
int −1.5
rank 2460
yor1−dF
miss 0
orf 1.5
int −1.7
rank 1791
NTH1(YDR001C)
DR = −90
Yor1
miss 0
orf 2.1
int 0.7
rank 1882
yor1−dF
miss 0
orf 0.5
int −1.8
rank 1792
ATF2(YGR177C)
DR = −209
Yor1
miss 0
orf 6.2
int 0.3
rank 2002
yor1−dF
miss 0
orf 0
int −1.8
rank 1793
0.35
0.25
0.20
0.15
0.10
0.05
0.00
−60
−20
0
20
40
−50
0
50

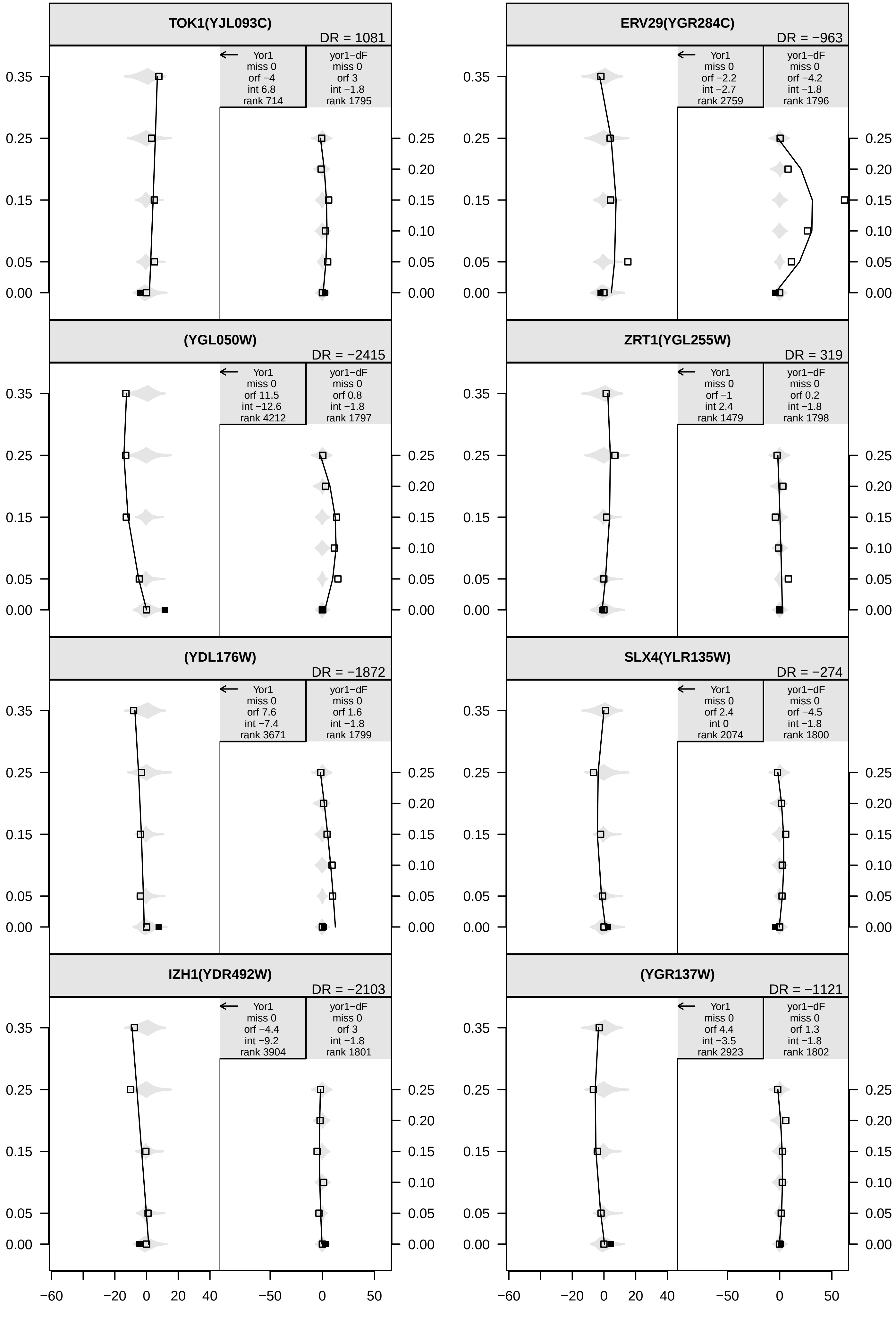

TOK1(YJL093C)
DR = 1081
Yor1 miss 0 orf −4 int 6.8 rank 714
yor1−dF miss 0 orf 3 int −1.8 rank 1795
ERV29(YGR284C)
DR = −963
Yor1 miss 0 orf −2.2 int −2.7 rank 2759
yor1−dF miss 0 orf −4.2 int −1.8 rank 1796
(YGL050W)
DR = −2415
Yor1 miss 0 orf 11.5 int −12.6 rank 4212
yor1−dF miss 0 orf 0.8 int −1.8 rank 1797
ZRT1(YGL255W)
DR = 319
Yor1 miss 0 orf −1 int 2.4 rank 1479
yor1−dF miss 0 orf 0.2 int −1.8 rank 1798
(YDL176W)
DR = −1872
Yor1 miss 0 orf 7.6 int −7.4 rank 3671
yor1−dF miss 0 orf 1.6 int −1.8 rank 1799
SLX4(YLR135W)
DR = −274
Yor1 miss 0 orf 2.4 int 0 rank 2074
yor1−dF miss 0 orf −4.5 int −1.8 rank 1800
IZH1(YDR492W)
DR = −2103
Yor1 miss 0 orf −4.4 int −9.2 rank 3904
yor1−dF miss 0 orf 3 int −1.8 rank 1801
(YGR137W)
DR = −1121
Yor1 miss 0 orf 4.4 int −3.5 rank 2923
yor1−dF miss 0 orf 1.3 int −1.8 rank 1802
0.00 0.05 0.10 0.15 0.20 0.25 0.35
−60 −20 0 20 40 −50 0 50

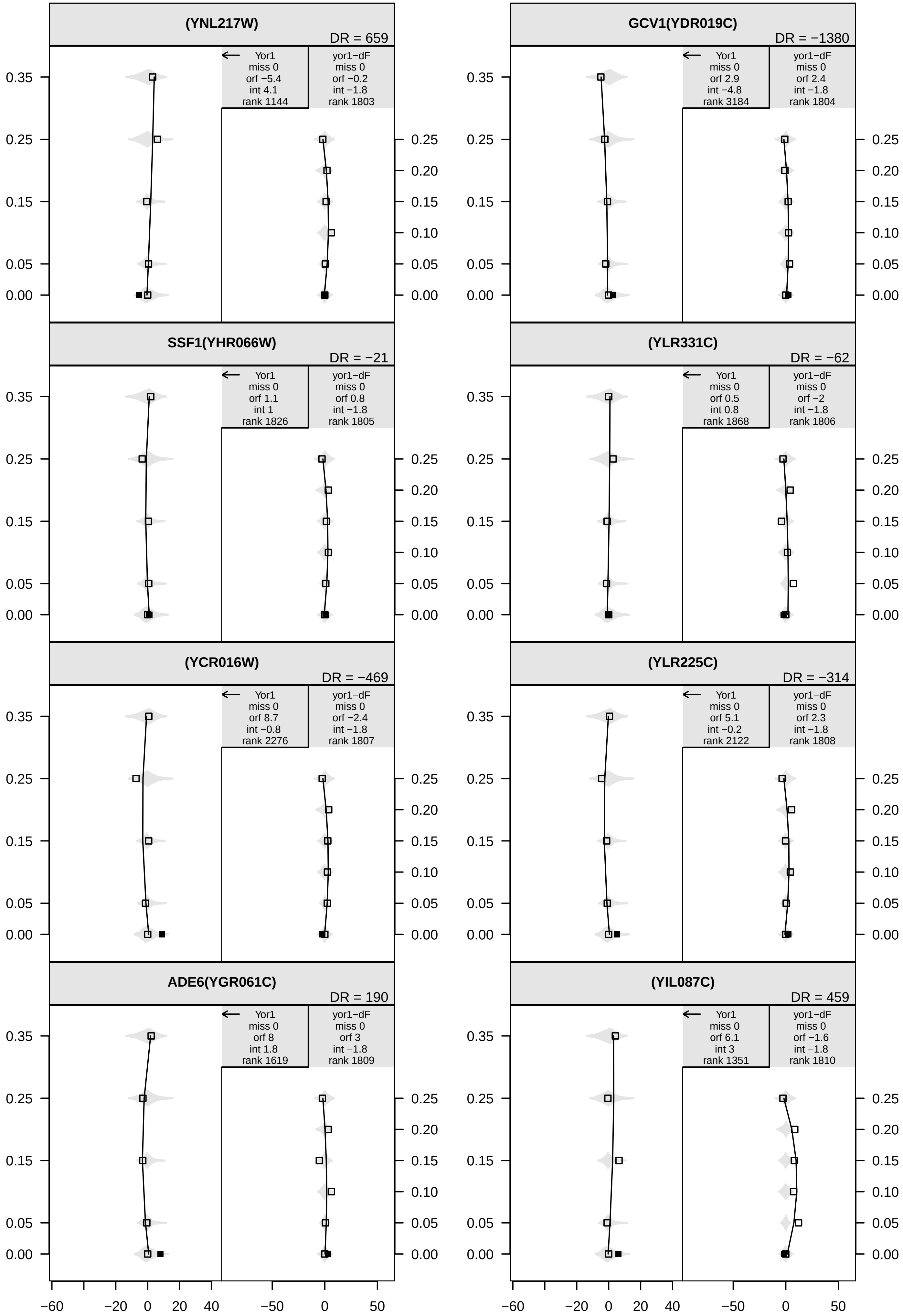

(YNL217W)
DR = 659
Yor1
miss 0
orf −5.4
int 4.1
rank 1144
yor1−dF
miss 0
orf −0.2
int −1.8
rank 1803
GCV1(YDR019C)
DR = −1380
Yor1
miss 0
orf 2.9
int −4.8
rank 3184
yor1−dF
miss 0
orf 2.4
int −1.8
rank 1804
SSF1(YHR066W)
DR = −21
Yor1
miss 0
orf 1.1
int 1
rank 1826
yor1−dF
miss 0
orf 0.8
int −1.8
rank 1805
(YLR331C)
DR = −62
Yor1
miss 0
orf 0.5
int 0.8
rank 1868
yor1−dF
miss 0
orf −2
int −1.8
rank 1806
(YCR016W)
DR = −469
Yor1
miss 0
orf 8.7
int −0.8
rank 2276
yor1−dF
miss 0
orf −2.4
int −1.8
rank 1807
(YLR225C)
DR = −314
Yor1
miss 0
orf 5.1
int −0.2
rank 2122
yor1−dF
miss 0
orf 2.3
int −1.8
rank 1808
ADE6(YGR061C)
DR = 190
Yor1
miss 0
orf 8
int 1.8
rank 1619
yor1−dF
miss 0
orf 3
int −1.8
rank 1809
(YIL087C)
DR = 459
Yor1
miss 0
orf 6.1
int 3
rank 1351
yor1−dF
miss 0
orf −1.6
int −1.8
rank 1810
0.35 0.25 0.15 0.05 0.00
0.25 0.20 0.15 0.10 0.05 0.00
−60 −20 0 20 40 −50 0 50

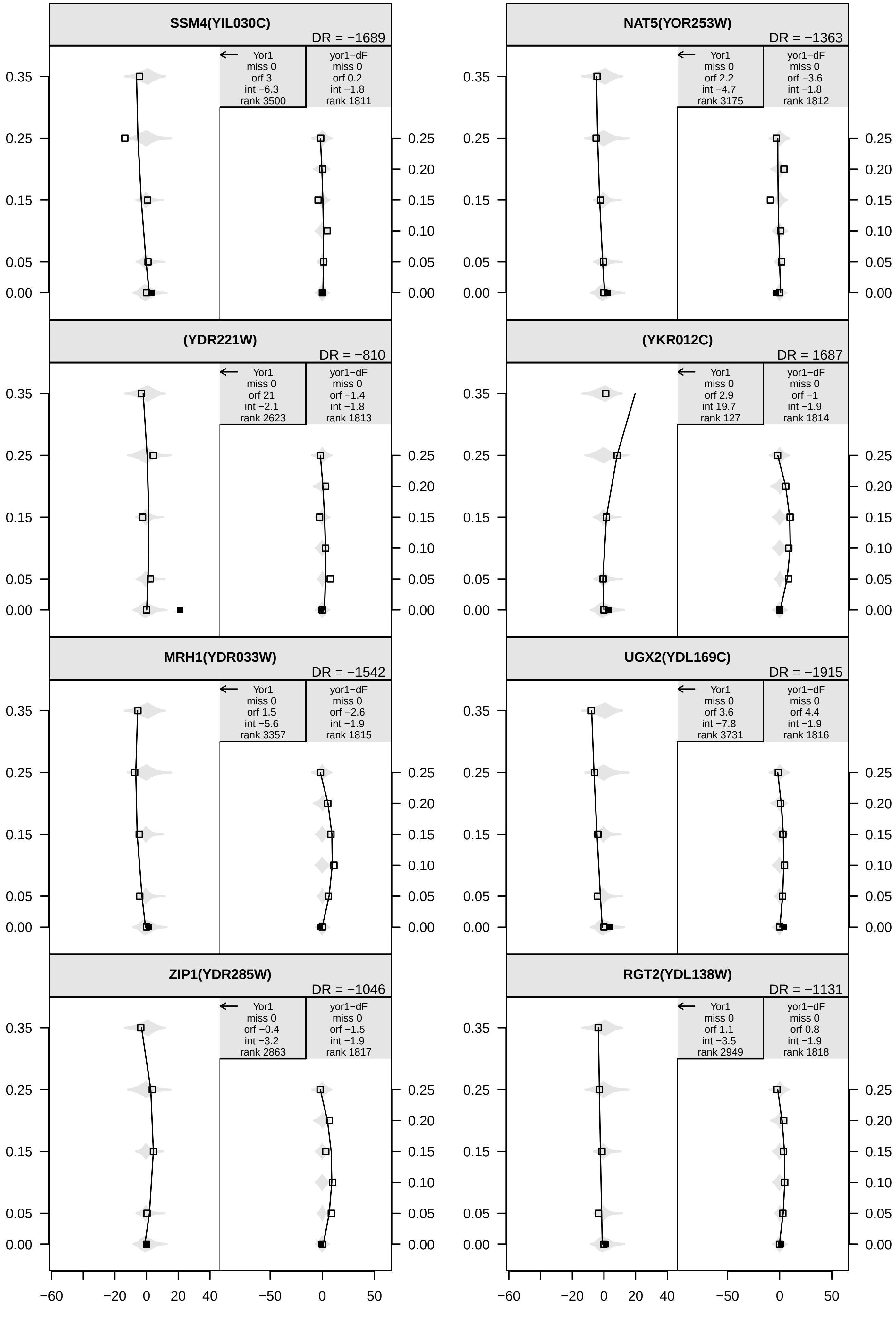
SSM4(YIL030C)
DR = −1689
Yor1 miss 0 orf 3 int −6.3 rank 3500
yor1−dF miss 0 orf 0.2 int −1.8 rank 1811
NAT5(YOR253W)
DR = −1363
Yor1 miss 0 orf 2.2 int −4.7 rank 3175
yor1−dF miss 0 orf −3.6 int −1.8 rank 1812
(YDR221W)
DR = −810
Yor1 miss 0 orf 21 int −2.1 rank 2623
yor1−dF miss 0 orf −1.4 int −1.8 rank 1813
(YKR012C)
DR = 1687
Yor1 miss 0 orf 2.9 int 19.7 rank 127
yor1−dF miss 0 orf −1 int −1.9 rank 1814
MRH1(YDR033W)
DR = −1542
Yor1 miss 0 orf 1.5 int −5.6 rank 3357
yor1−dF miss 0 orf −2.6 int −1.9 rank 1815
UGX2(YDL169C)
DR = −1915
Yor1 miss 0 orf 3.6 int −7.8 rank 3731
yor1−dF miss 0 orf 4.4 int −1.9 rank 1816
ZIP1(YDR285W)
DR = −1046
Yor1 miss 0 orf −0.4 int −3.2 rank 2863
yor1−dF miss 0 orf −1.5 int −1.9 rank 1817
RGT2(YDL138W)
DR = −1131
Yor1 miss 0 orf 1.1 int −3.5 rank 2949
yor1−dF miss 0 orf 0.8 int −1.9 rank 1818
0.35
0.25
0.20
0.15
0.10
0.05
0.00
−60
−20
0
20
40
−50
50

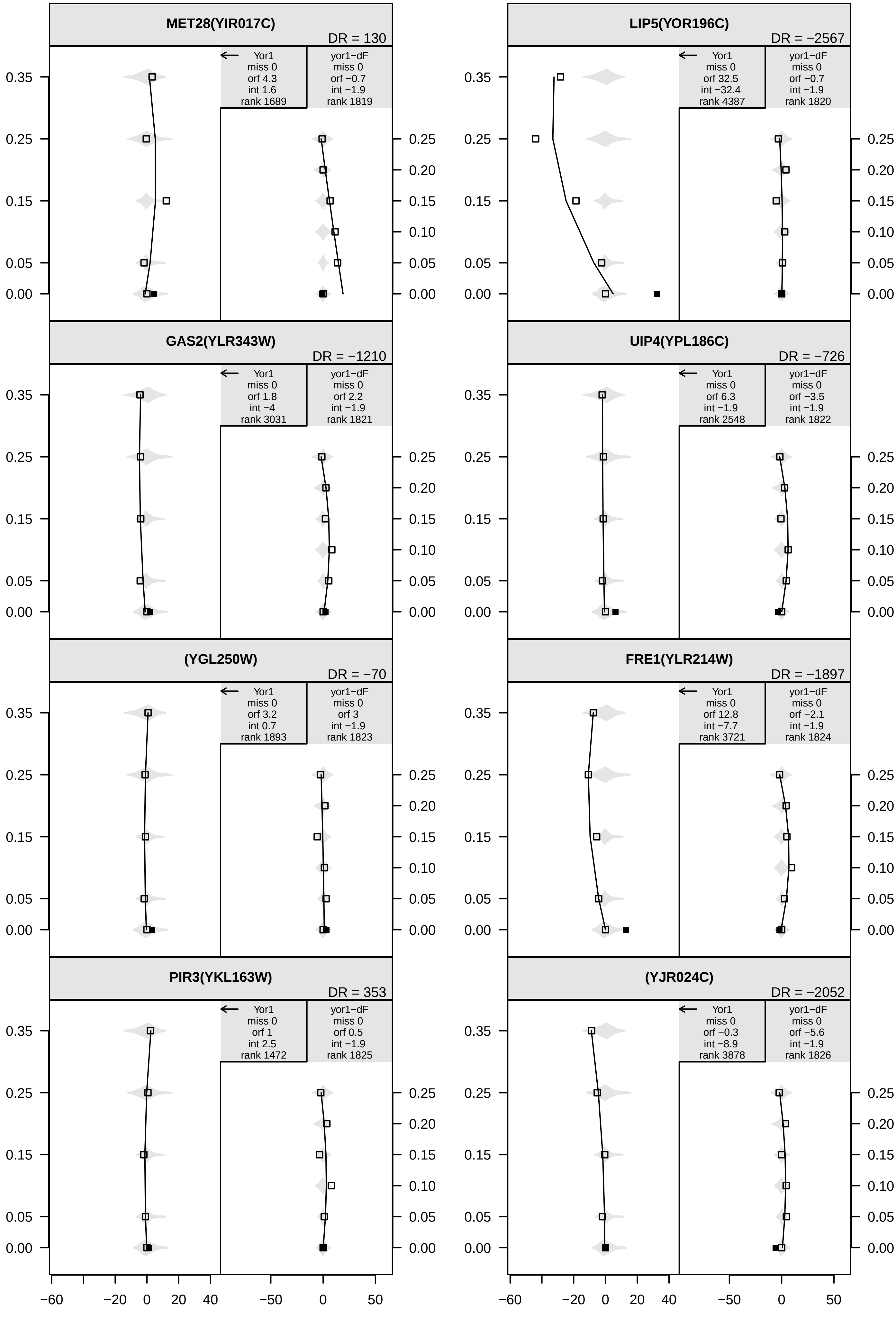

MET28(YIR017C)
DR = 130
Yor1 miss 0 orf 4.3 int 1.6 rank 1689
yor1−dF miss 0 orf −0.7 int −1.9 rank 1819
LIP5(YOR196C)
DR = −2567
Yor1 miss 0 orf 32.5 int −32.4 rank 4387
yor1−dF miss 0 orf −0.7 int −1.9 rank 1820
GAS2(YLR343W)
DR = −1210
Yor1 miss 0 orf 1.8 int −4 rank 3031
yor1−dF miss 0 orf 2.2 int −1.9 rank 1821
UIP4(YPL186C)
DR = −726
Yor1 miss 0 orf 6.3 int −1.9 rank 2548
yor1−dF miss 0 orf −3.5 int −1.9 rank 1822
(YGL250W)
DR = −70
Yor1 miss 0 orf 3.2 int 0.7 rank 1893
yor1−dF miss 0 orf 3 int −1.9 rank 1823
FRE1(YLR214W)
DR = −1897
Yor1 miss 0 orf 12.8 int −7.7 rank 3721
yor1−dF miss 0 orf −2.1 int −1.9 rank 1824
PIR3(YKL163W)
DR = 353
Yor1 miss 0 orf 1 int 2.5 rank 1472
yor1−dF miss 0 orf 0.5 int −1.9 rank 1825
(YJR024C)
DR = −2052
Yor1 miss 0 orf −0.3 int −8.9 rank 3878
yor1−dF miss 0 orf −5.6 int −1.9 rank 1826
0.00 0.05 0.10 0.15 0.20 0.25 0.35
−60 −20 0 20 40 −50 0 50

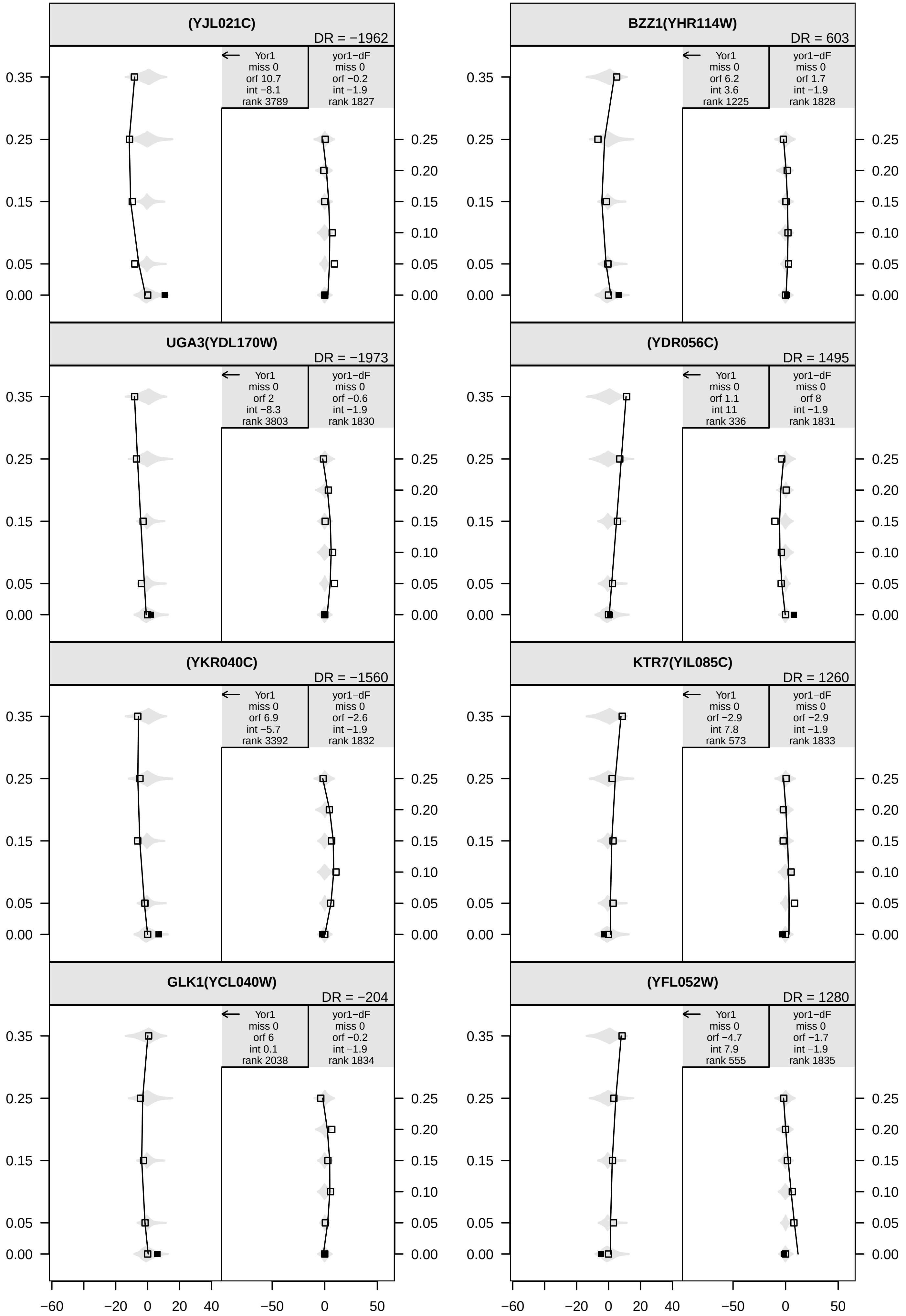

(YJL021C)
DR = −1962
Yor1
miss 0
orf 10.7
int −8.1
rank 3789
yor1−dF
miss 0
orf −0.2
int −1.9
rank 1827
BZZ1(YHR114W)
DR = 603
Yor1
miss 0
orf 6.2
int 3.6
rank 1225
yor1−dF
miss 0
orf 1.7
int −1.9
rank 1828
UGA3(YDL170W)
DR = −1973
Yor1
miss 0
orf 2
int −8.3
rank 3803
yor1−dF
miss 0
orf −0.6
int −1.9
rank 1830
(YDR056C)
DR = 1495
Yor1
miss 0
orf 1.1
int 11
rank 336
yor1−dF
miss 0
orf 8
int −1.9
rank 1831
(YKR040C)
DR = −1560
Yor1
miss 0
orf 6.9
int −5.7
rank 3392
yor1−dF
miss 0
orf −2.6
int −1.9
rank 1832
KTR7(YIL085C)
DR = 1260
Yor1
miss 0
orf −2.9
int 7.8
rank 573
yor1−dF
miss 0
orf −2.9
int −1.9
rank 1833
GLK1(YCL040W)
DR = −204
Yor1
miss 0
orf 6
int 0.1
rank 2038
yor1−dF
miss 0
orf −0.2
int −1.9
rank 1834
(YFL052W)
DR = 1280
Yor1
miss 0
orf −4.7
int 7.9
rank 555
yor1−dF
miss 0
orf −1.7
int −1.9
rank 1835
0.35
0.25
0.20
0.15
0.10
0.05
0.00
−60
−20
0
20
40
−50
0
50

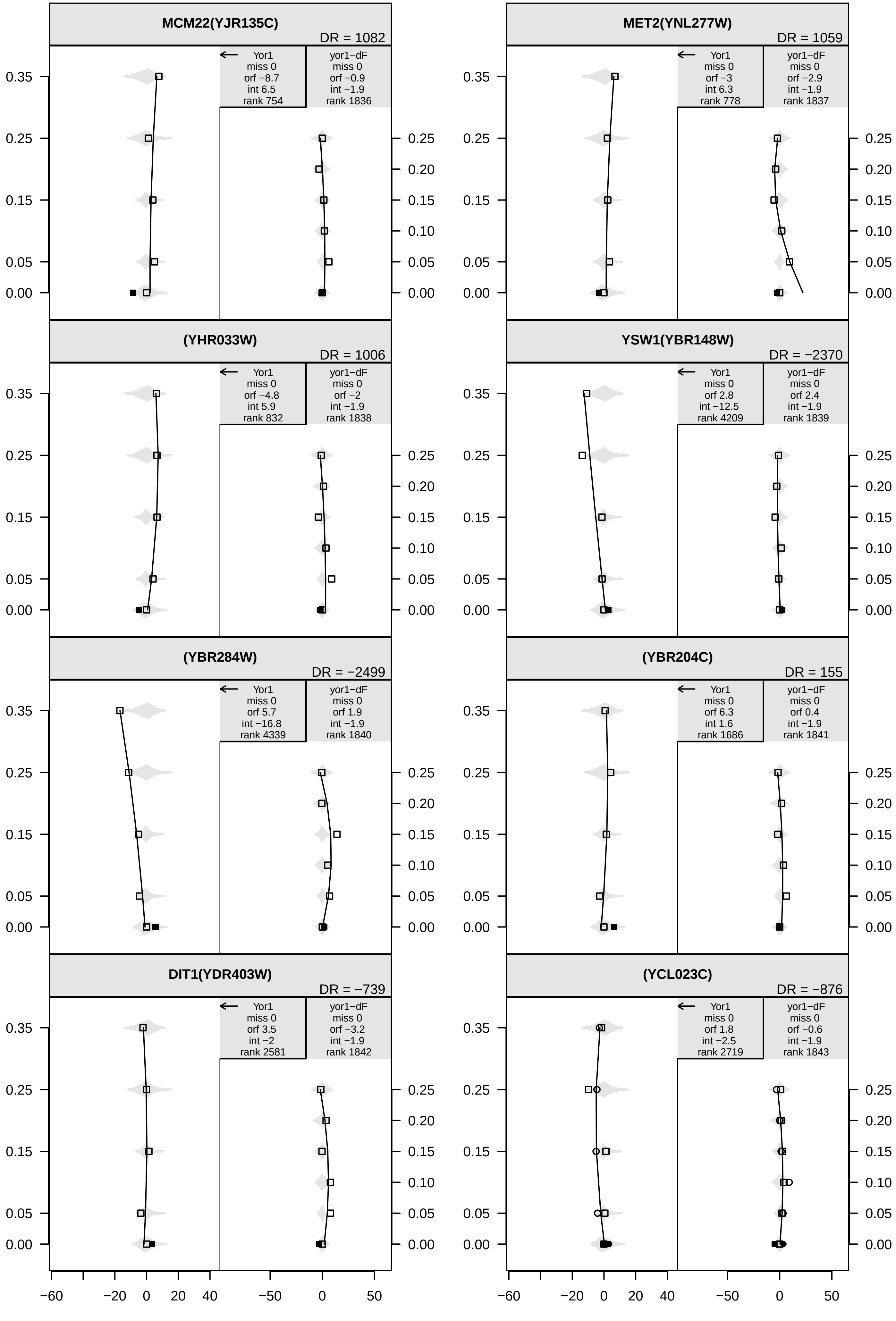

MCM22(YJR135C)
DR = 1082
Yor1 miss 0 orf −8.7 int 6.5 rank 754
yor1−dF miss 0 orf −0.9 int −1.9 rank 1836
MET2(YNL277W)
DR = 1059
Yor1 miss 0 orf −3 int 6.3 rank 778
yor1−dF miss 0 orf −2.9 int −1.9 rank 1837
(YHR033W)
DR = 1006
Yor1 miss 0 orf −4.8 int 5.9 rank 832
yor1−dF miss 0 orf −2 int −1.9 rank 1838
YSW1(YBR148W)
DR = −2370
Yor1 miss 0 orf 2.8 int −12.5 rank 4209
yor1−dF miss 0 orf 2.4 int −1.9 rank 1839
(YBR284W)
DR = −2499
Yor1 miss 0 orf 5.7 int −16.8 rank 4339
yor1−dF miss 0 orf 1.9 int −1.9 rank 1840
(YBR204C)
DR = 155
Yor1 miss 0 orf 6.3 int 1.6 rank 1686
yor1−dF miss 0 orf 0.4 int −1.9 rank 1841
DIT1(YDR403W)
DR = −739
Yor1 miss 0 orf 3.5 int −2 rank 2581
yor1−dF miss 0 orf −3.2 int −1.9 rank 1842
(YCL023C)
DR = −876
Yor1 miss 0 orf 1.8 int −2.5 rank 2719
yor1−dF miss 0 orf −0.6 int −1.9 rank 1843
0.00 0.05 0.10 0.15 0.20 0.25 0.35
−60 −20 0 20 40 −50 0 50

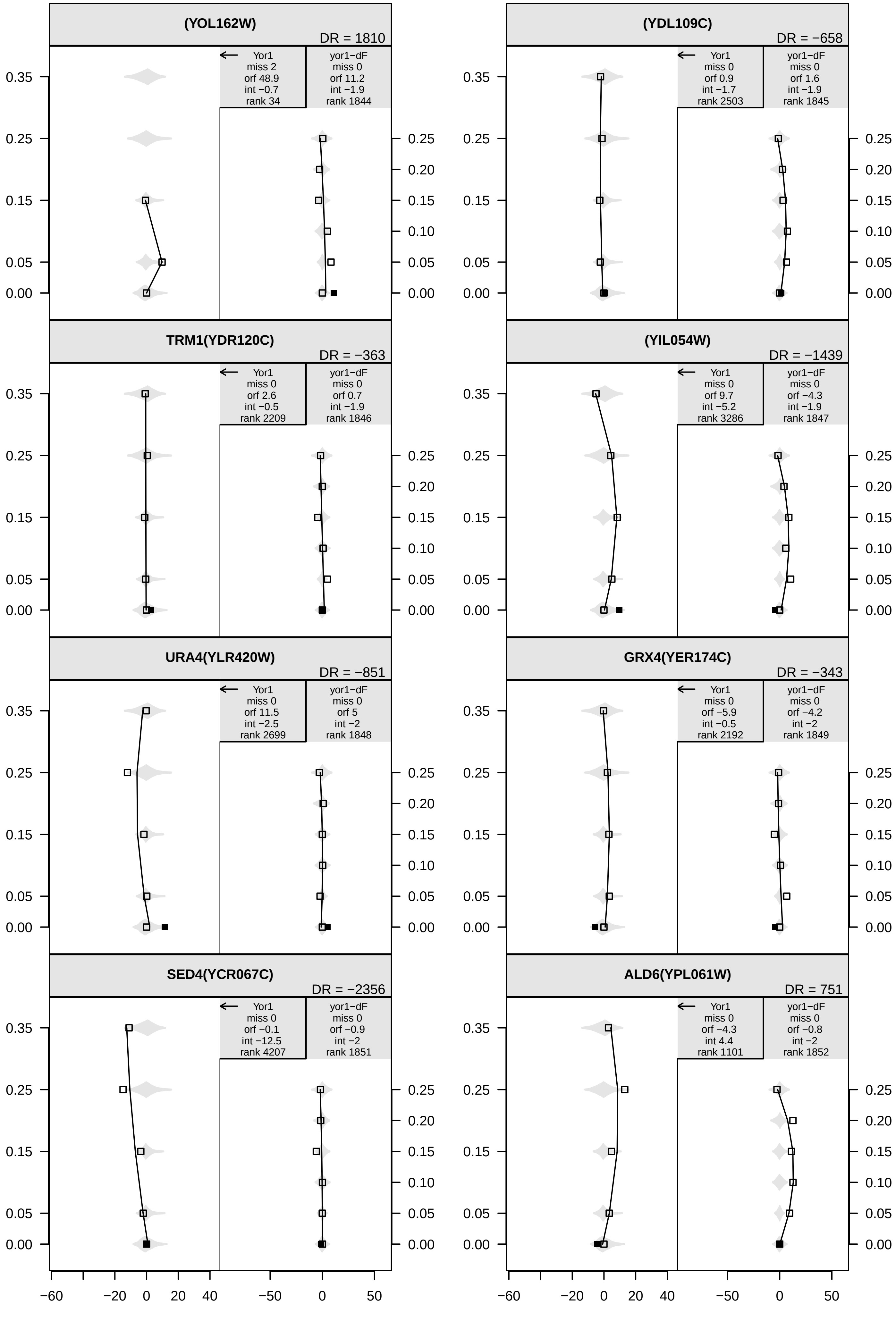

(YOL162W)
DR = 1810
Yor1
miss 2
orf 48.9
int −0.7
rank 34
yor1−dF
miss 0
orf 11.2
int −1.9
rank 1844
(YDL109C)
DR = −658
Yor1
miss 0
orf 0.9
int −1.7
rank 2503
yor1−dF
miss 0
orf 1.6
int −1.9
rank 1845
TRM1(YDR120C)
DR = −363
Yor1
miss 0
orf 2.6
int −0.5
rank 2209
yor1−dF
miss 0
orf 0.7
int −1.9
rank 1846
(YIL054W)
DR = −1439
Yor1
miss 0
orf 9.7
int −5.2
rank 3286
yor1−dF
miss 0
orf −4.3
int −1.9
rank 1847
URA4(YLR420W)
DR = −851
Yor1
miss 0
orf 11.5
int −2.5
rank 2699
yor1−dF
miss 0
orf 5
int −2
rank 1848
GRX4(YER174C)
DR = −343
Yor1
miss 0
orf −5.9
int −0.5
rank 2192
yor1−dF
miss 0
orf −4.2
int −2
rank 1849
SED4(YCR067C)
DR = −2356
Yor1
miss 0
orf −0.1
int −12.5
rank 4207
yor1−dF
miss 0
orf −0.9
int −2
rank 1851
ALD6(YPL061W)
DR = 751
Yor1
miss 0
orf −4.3
int 4.4
rank 1101
yor1−dF
miss 0
orf −0.8
int −2
rank 1852
0.35
0.25
0.20
0.15
0.10
0.05
0.00
−60
−20
0
20
40
−50
0
50

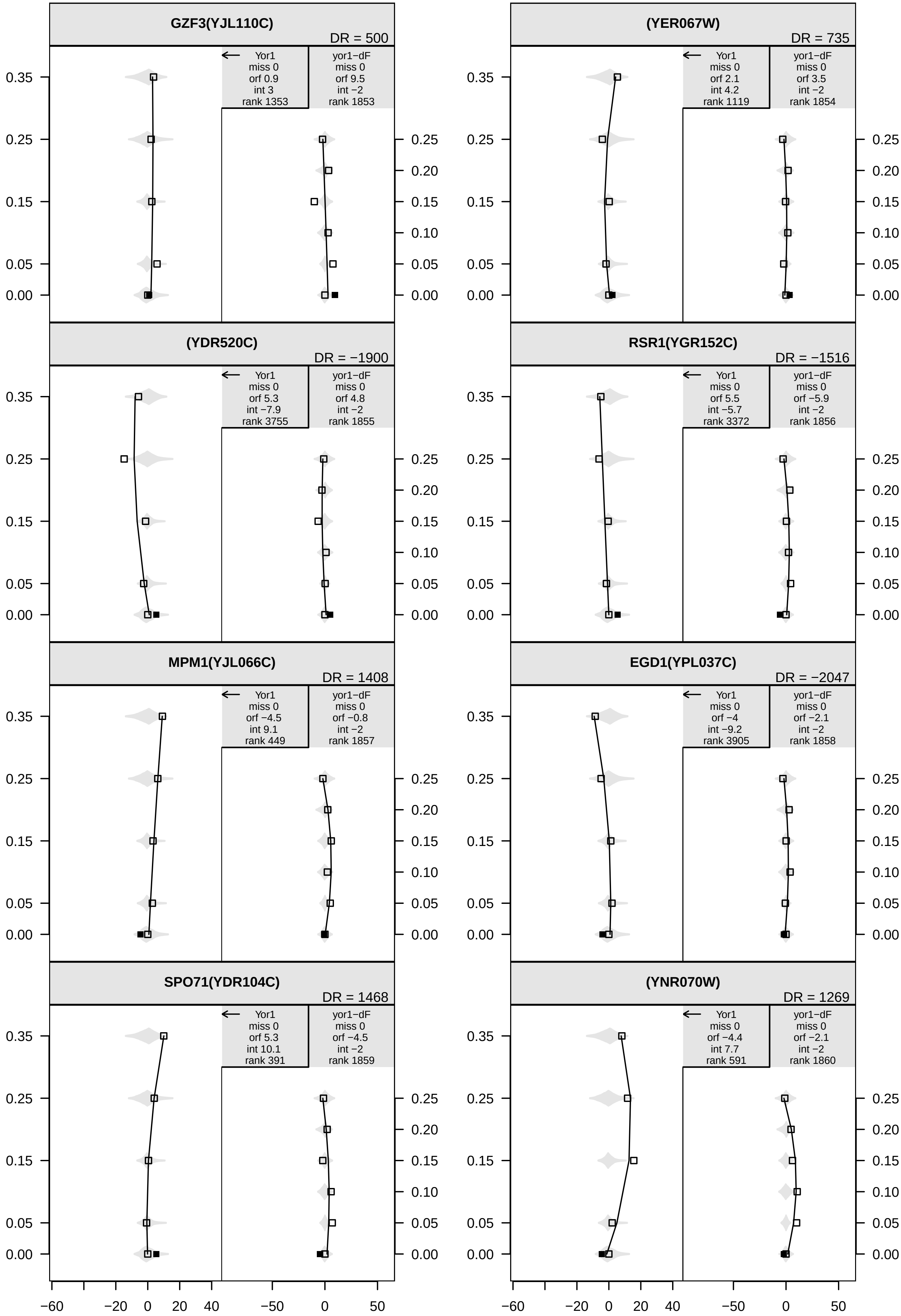

GZF3(YJL110C)
DR = 500
Yor1 miss 0 orf 0.9 int 3 rank 1353
yor1−dF miss 0 orf 9.5 int −2 rank 1853
(YER067W)
DR = 735
Yor1 miss 0 orf 2.1 int 4.2 rank 1119
yor1−dF miss 0 orf 3.5 int −2 rank 1854
(YDR520C)
DR = −1900
Yor1 miss 0 orf 5.3 int −7.9 rank 3755
yor1−dF miss 0 orf 4.8 int −2 rank 1855
RSR1(YGR152C)
DR = −1516
Yor1 miss 0 orf 5.5 int −5.7 rank 3372
yor1−dF miss 0 orf −5.9 int −2 rank 1856
MPM1(YJL066C)
DR = 1408
Yor1 miss 0 orf −4.5 int 9.1 rank 449
yor1−dF miss 0 orf −0.8 int −2 rank 1857
EGD1(YPL037C)
DR = −2047
Yor1 miss 0 orf −4 int −9.2 rank 3905
yor1−dF miss 0 orf −2.1 int −2 rank 1858
SPO71(YDR104C)
DR = 1468
Yor1 miss 0 orf 5.3 int 10.1 rank 391
yor1−dF miss 0 orf −4.5 int −2 rank 1859
(YNR070W)
DR = 1269
Yor1 miss 0 orf −4.4 int 7.7 rank 591
yor1−dF miss 0 orf −2.1 int −2 rank 1860
0.35 0.25 0.15 0.05 0.00
0.25 0.20 0.15 0.10 0.05 0.00
−60 −20 0 20 40 −50 0 50

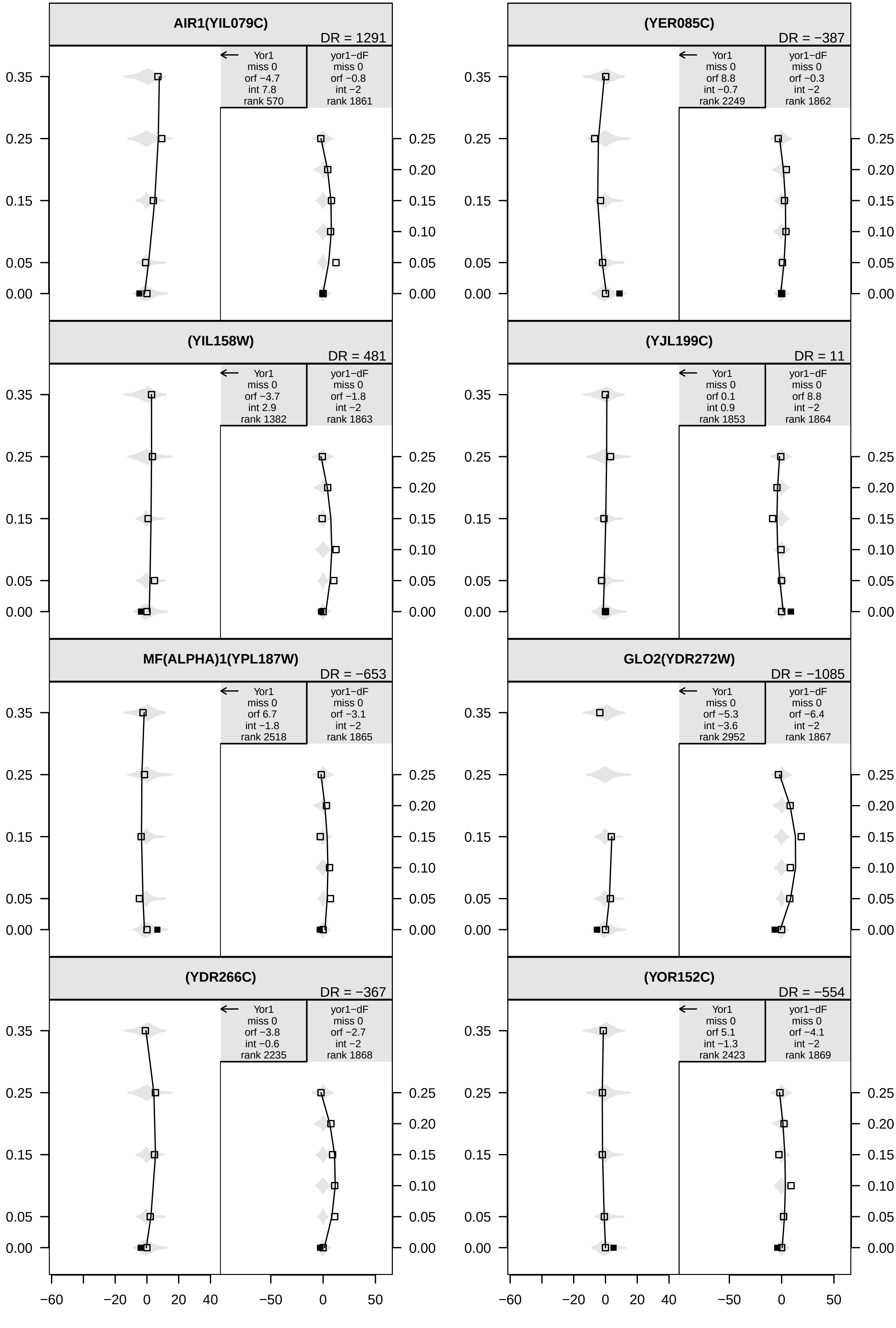
AIR1(YIL079C)
DR = 1291
Yor1 miss 0 orf −4.7 int 7.8 rank 570
yor1−dF miss 0 orf −0.8 int −2 rank 1861
(YER085C)
DR = −387
Yor1 miss 0 orf 8.8 int −0.7 rank 2249
yor1−dF miss 0 orf −0.3 int −2 rank 1862
(YIL158W)
DR = 481
Yor1 miss 0 orf −3.7 int 2.9 rank 1382
yor1−dF miss 0 orf −1.8 int −2 rank 1863
(YJL199C)
DR = 11
Yor1 miss 0 orf 0.1 int 0.9 rank 1853
yor1−dF miss 0 orf 8.8 int −2 rank 1864
MF(ALPHA)1(YPL187W)
DR = −653
Yor1 miss 0 orf 6.7 int −1.8 rank 2518
yor1−dF miss 0 orf −3.1 int −2 rank 1865
GLO2(YDR272W)
DR = −1085
Yor1 miss 0 orf −5.3 int −3.6 rank 2952
yor1−dF miss 0 orf −6.4 int −2 rank 1867
(YDR266C)
DR = −367
Yor1 miss 0 orf −3.8 int −0.6 rank 2235
yor1−dF miss 0 orf −2.7 int −2 rank 1868
(YOR152C)
DR = −554
Yor1 miss 0 orf 5.1 int −1.3 rank 2423
yor1−dF miss 0 orf −4.1 int −2 rank 1869
0.35 0.25 0.20 0.15 0.10 0.05 0.00
−60 −20 0 20 40 −50 0 50

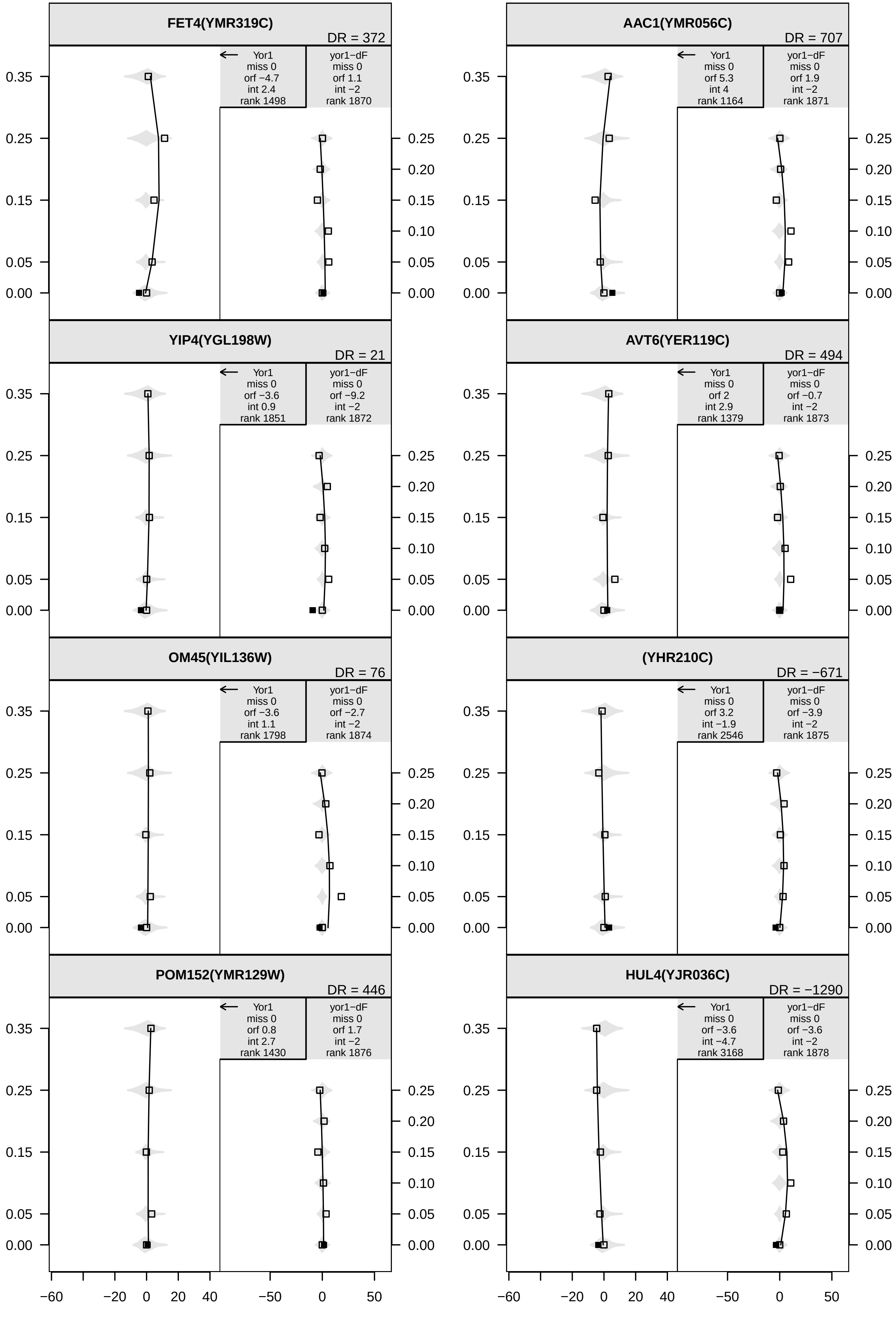
FET4(YMR319C)
DR = 372
Yor1
miss 0
orf −4.7
int 2.4
rank 1498
yor1−dF
miss 0
orf 1.1
int −2
rank 1870
AAC1(YMR056C)
DR = 707
Yor1
miss 0
orf 5.3
int 4
rank 1164
yor1−dF
miss 0
orf 1.9
int −2
rank 1871
YIP4(YGL198W)
DR = 21
Yor1
miss 0
orf −3.6
int 0.9
rank 1851
yor1−dF
miss 0
orf −9.2
int −2
rank 1872
AVT6(YER119C)
DR = 494
Yor1
miss 0
orf 2
int 2.9
rank 1379
yor1−dF
miss 0
orf −0.7
int −2
rank 1873
OM45(YIL136W)
DR = 76
Yor1
miss 0
orf −3.6
int 1.1
rank 1798
yor1−dF
miss 0
orf −2.7
int −2
rank 1874
(YHR210C)
DR = −671
Yor1
miss 0
orf 3.2
int −1.9
rank 2546
yor1−dF
miss 0
orf −3.9
int −2
rank 1875
POM152(YMR129W)
DR = 446
Yor1
miss 0
orf 0.8
int 2.7
rank 1430
yor1−dF
miss 0
orf 1.7
int −2
rank 1876
HUL4(YJR036C)
DR = −1290
Yor1
miss 0
orf −3.6
int −4.7
rank 3168
yor1−dF
miss 0
orf −3.6
int −2
rank 1878
0.35
0.25
0.20
0.15
0.10
0.05
0.00
−60
−20
0
20
40
−50
0
50

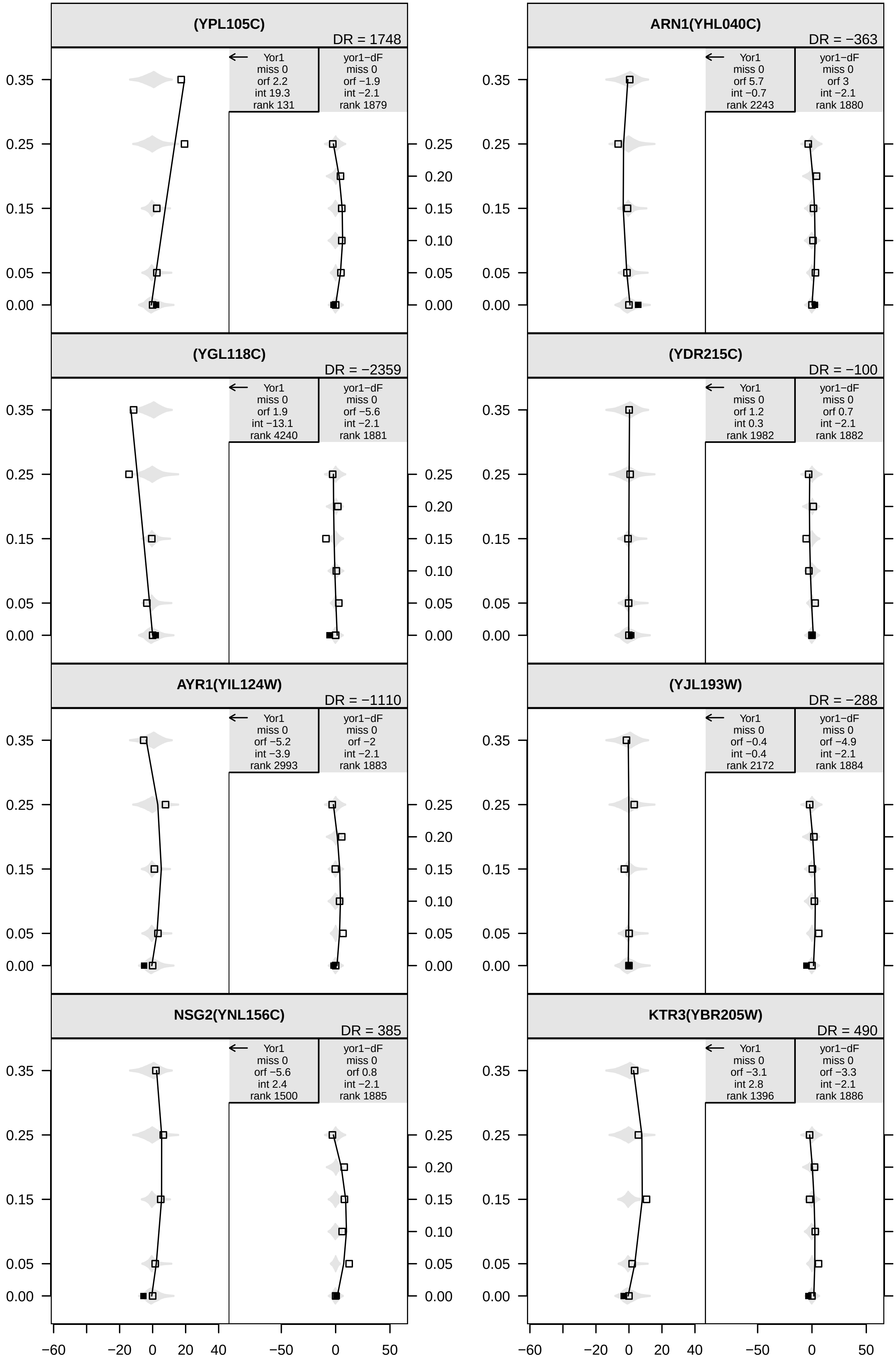

(YPL105C)
DR = 1748
Yor1 miss 0 orf 2.2 int 19.3 rank 131
yor1−dF miss 0 orf −1.9 int −2.1 rank 1879
ARN1(YHL040C)
DR = −363
Yor1 miss 0 orf 5.7 int −0.7 rank 2243
yor1−dF miss 0 orf 3 int −2.1 rank 1880
(YGL118C)
DR = −2359
Yor1 miss 0 orf 1.9 int −13.1 rank 4240
yor1−dF miss 0 orf −5.6 int −2.1 rank 1881
(YDR215C)
DR = −100
Yor1 miss 0 orf 1.2 int 0.3 rank 1982
yor1−dF miss 0 orf 0.7 int −2.1 rank 1882
AYR1(YIL124W)
DR = −1110
Yor1 miss 0 orf −5.2 int −3.9 rank 2993
yor1−dF miss 0 orf −2 int −2.1 rank 1883
(YJL193W)
DR = −288
Yor1 miss 0 orf −0.4 int −0.4 rank 2172
yor1−dF miss 0 orf −4.9 int −2.1 rank 1884
NSG2(YNL156C)
DR = 385
Yor1 miss 0 orf −5.6 int 2.4 rank 1500
yor1−dF miss 0 orf 0.8 int −2.1 rank 1885
KTR3(YBR205W)
DR = 490
Yor1 miss 0 orf −3.1 int 2.8 rank 1396
yor1−dF miss 0 orf −3.3 int −2.1 rank 1886
0.35 0.25 0.20 0.15 0.10 0.05 0.00
−60 −20 0 20 40 −50 0 50

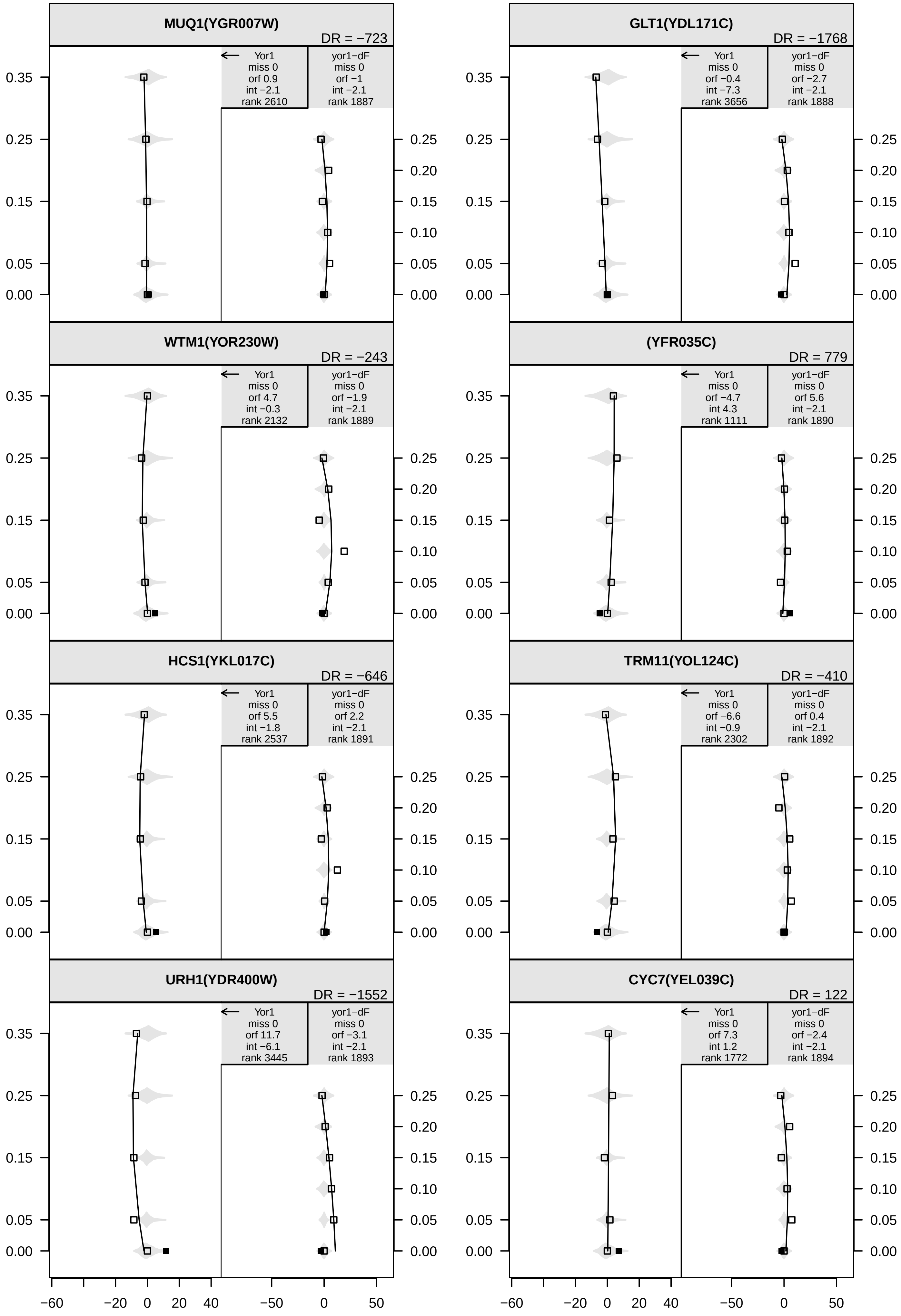
MUQ1(YGR007W)
DR = −723
Yor1
miss 0
orf 0.9
int −2.1
rank 2610
yor1−dF
miss 0
orf −1
int −2.1
rank 1887
GLT1(YDL171C)
DR = −1768
Yor1
miss 0
orf −0.4
int −7.3
rank 3656
yor1−dF
miss 0
orf −2.7
int −2.1
rank 1888
WTM1(YOR230W)
DR = −243
Yor1
miss 0
orf 4.7
int −0.3
rank 2132
yor1−dF
miss 0
orf −1.9
int −2.1
rank 1889
(YFR035C)
DR = 779
Yor1
miss 0
orf −4.7
int 4.3
rank 1111
yor1−dF
miss 0
orf 5.6
int −2.1
rank 1890
HCS1(YKL017C)
DR = −646
Yor1
miss 0
orf 5.5
int −1.8
rank 2537
yor1−dF
miss 0
orf 2.2
int −2.1
rank 1891
TRM11(YOL124C)
DR = −410
Yor1
miss 0
orf −6.6
int −0.9
rank 2302
yor1−dF
miss 0
orf 0.4
int −2.1
rank 1892
URH1(YDR400W)
DR = −1552
Yor1
miss 0
orf 11.7
int −6.1
rank 3445
yor1−dF
miss 0
orf −3.1
int −2.1
rank 1893
CYC7(YEL039C)
DR = 122
Yor1
miss 0
orf 7.3
int 1.2
rank 1772
yor1−dF
miss 0
orf −2.4
int −2.1
rank 1894
0.35
0.25
0.20
0.15
0.10
0.05
0.00
−60
−20
0
20
40
−50
0
50

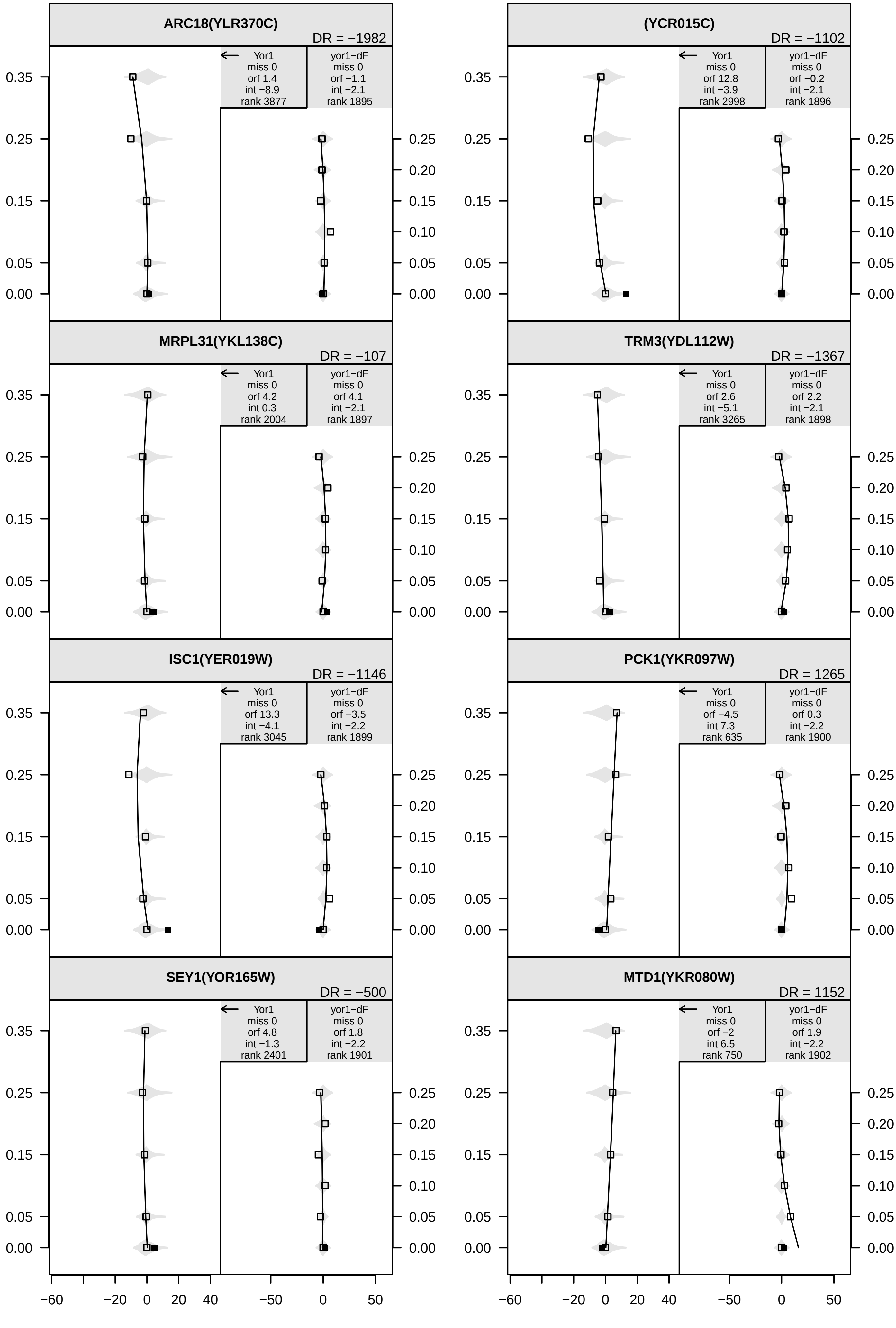
ARC18(YLR370C)
DR = −1982
Yor1
miss 0
orf 1.4
int −8.9
rank 3877
yor1−dF
miss 0
orf −1.1
int −2.1
rank 1895
(YCR015C)
DR = −1102
Yor1
miss 0
orf 12.8
int −3.9
rank 2998
yor1−dF
miss 0
orf −0.2
int −2.1
rank 1896
MRPL31(YKL138C)
DR = −107
Yor1
miss 0
orf 4.2
int 0.3
rank 2004
yor1−dF
miss 0
orf 4.1
int −2.1
rank 1897
TRM3(YDL112W)
DR = −1367
Yor1
miss 0
orf 2.6
int −5.1
rank 3265
yor1−dF
miss 0
orf 2.2
int −2.1
rank 1898
ISC1(YER019W)
DR = −1146
Yor1
miss 0
orf 13.3
int −4.1
rank 3045
yor1−dF
miss 0
orf −3.5
int −2.2
rank 1899
PCK1(YKR097W)
DR = 1265
Yor1
miss 0
orf −4.5
int 7.3
rank 635
yor1−dF
miss 0
orf 0.3
int −2.2
rank 1900
SEY1(YOR165W)
DR = −500
Yor1
miss 0
orf 4.8
int −1.3
rank 2401
yor1−dF
miss 0
orf 1.8
int −2.2
rank 1901
MTD1(YKR080W)
DR = 1152
Yor1
miss 0
orf −2
int 6.5
rank 750
yor1−dF
miss 0
orf 1.9
int −2.2
rank 1902
0.35
0.25
0.20
0.15
0.10
0.05
0.00
−60
−20
0
20
40
−50
0
50

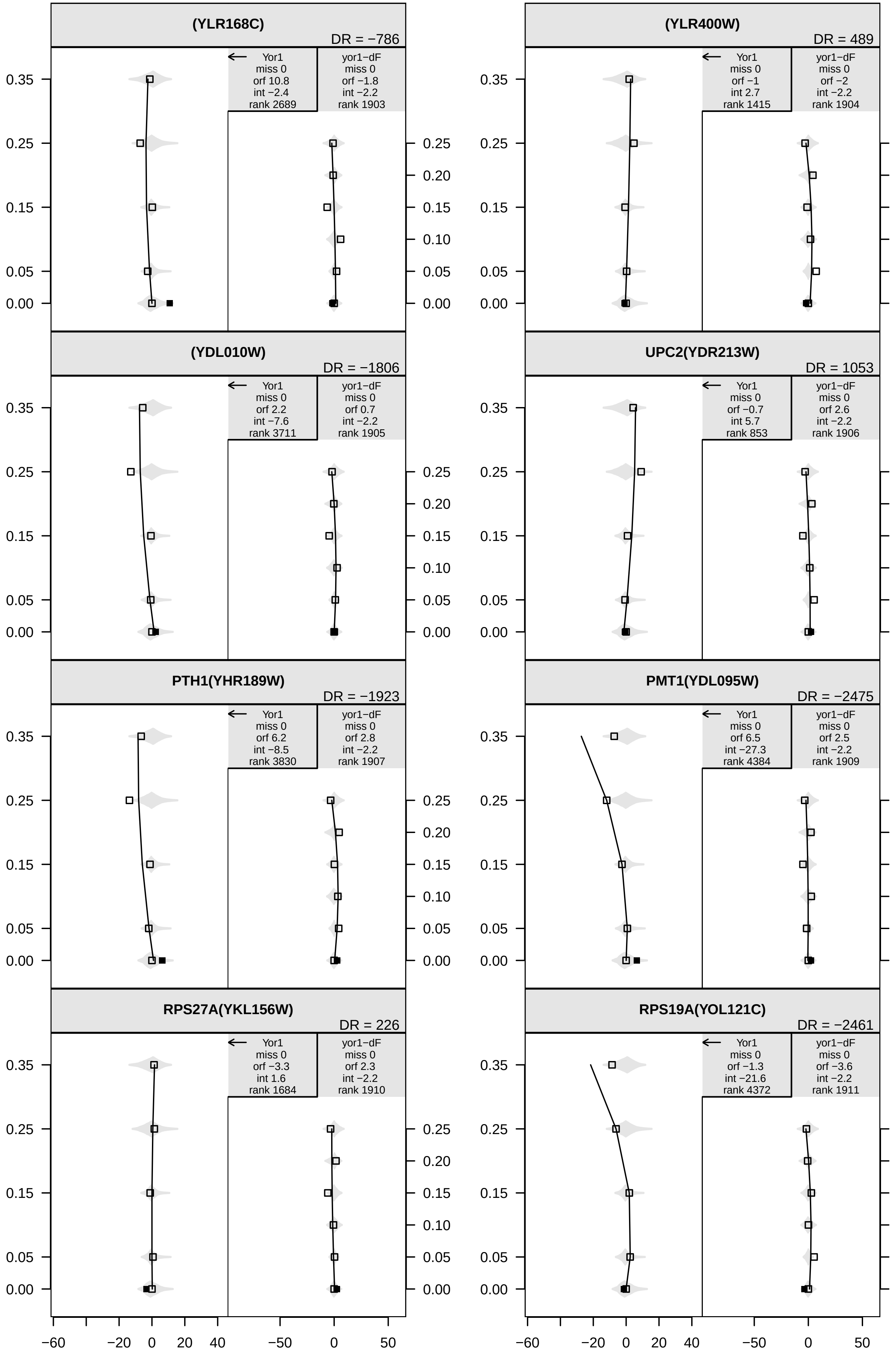
(YLR168C)
DR = −786
Yor1 miss 0 orf 10.8 int −2.4 rank 2689
yor1−dF miss 0 orf −1.8 int −2.2 rank 1903
(YLR400W)
DR = 489
Yor1 miss 0 orf −1 int 2.7 rank 1415
yor1−dF miss 0 orf −2 int −2.2 rank 1904
(YDL010W)
DR = −1806
Yor1 miss 0 orf 2.2 int −7.6 rank 3711
yor1−dF miss 0 orf 0.7 int −2.2 rank 1905
UPC2(YDR213W)
DR = 1053
Yor1 miss 0 orf −0.7 int 5.7 rank 853
yor1−dF miss 0 orf 2.6 int −2.2 rank 1906
PTH1(YHR189W)
DR = −1923
Yor1 miss 0 orf 6.2 int −8.5 rank 3830
yor1−dF miss 0 orf 2.8 int −2.2 rank 1907
PMT1(YDL095W)
DR = −2475
Yor1 miss 0 orf 6.5 int −27.3 rank 4384
yor1−dF miss 0 orf 2.5 int −2.2 rank 1909
RPS27A(YKL156W)
DR = 226
Yor1 miss 0 orf −3.3 int 1.6 rank 1684
yor1−dF miss 0 orf 2.3 int −2.2 rank 1910
RPS19A(YOL121C)
DR = −2461
Yor1 miss 0 orf −1.3 int −21.6 rank 4372
yor1−dF miss 0 orf −3.6 int −2.2 rank 1911
0.35 0.25 0.20 0.15 0.10 0.05 0.00
−60 −20 0 20 40 −50 0 50

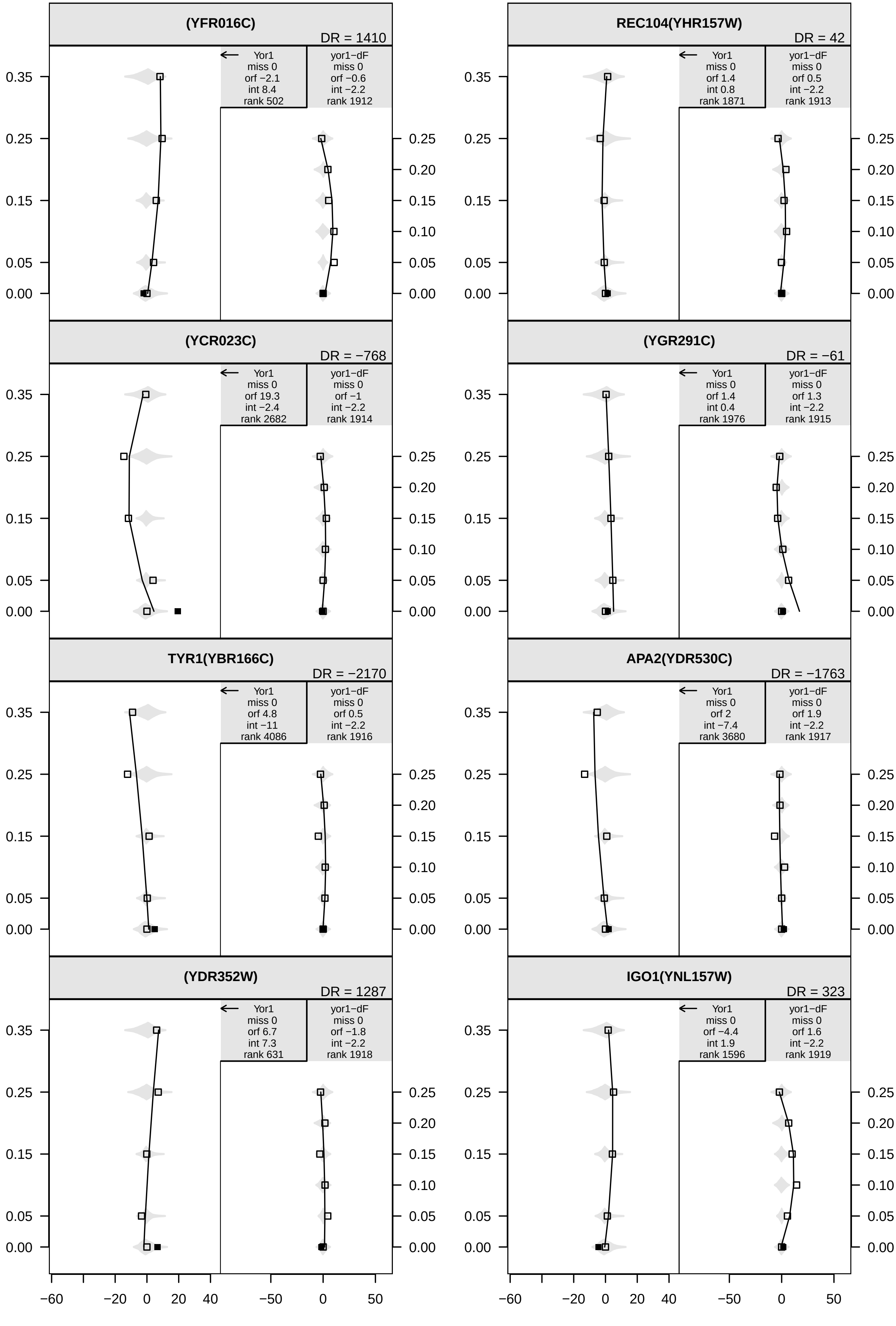

(YFR016C)
DR = 1410
Yor1 miss 0 orf −2.1 int 8.4 rank 502
yor1−dF miss 0 orf −0.6 int −2.2 rank 1912
REC104(YHR157W)
DR = 42
Yor1 miss 0 orf 1.4 int 0.8 rank 1871
yor1−dF miss 0 orf 0.5 int −2.2 rank 1913
(YCR023C)
DR = −768
Yor1 miss 0 orf 19.3 int −2.4 rank 2682
yor1−dF miss 0 orf −1 int −2.2 rank 1914
(YGR291C)
DR = −61
Yor1 miss 0 orf 1.4 int 0.4 rank 1976
yor1−dF miss 0 orf 1.3 int −2.2 rank 1915
TYR1(YBR166C)
DR = −2170
Yor1 miss 0 orf 4.8 int −11 rank 4086
yor1−dF miss 0 orf 0.5 int −2.2 rank 1916
APA2(YDR530C)
DR = −1763
Yor1 miss 0 orf 2 int −7.4 rank 3680
yor1−dF miss 0 orf 1.9 int −2.2 rank 1917
(YDR352W)
DR = 1287
Yor1 miss 0 orf 6.7 int 7.3 rank 631
yor1−dF miss 0 orf −1.8 int −2.2 rank 1918
IGO1(YNL157W)
DR = 323
Yor1 miss 0 orf −4.4 int 1.9 rank 1596
yor1−dF miss 0 orf 1.6 int −2.2 rank 1919
0.00 0.05 0.10 0.15 0.20 0.25 0.35
−60 −20 0 20 40 −50 0 50

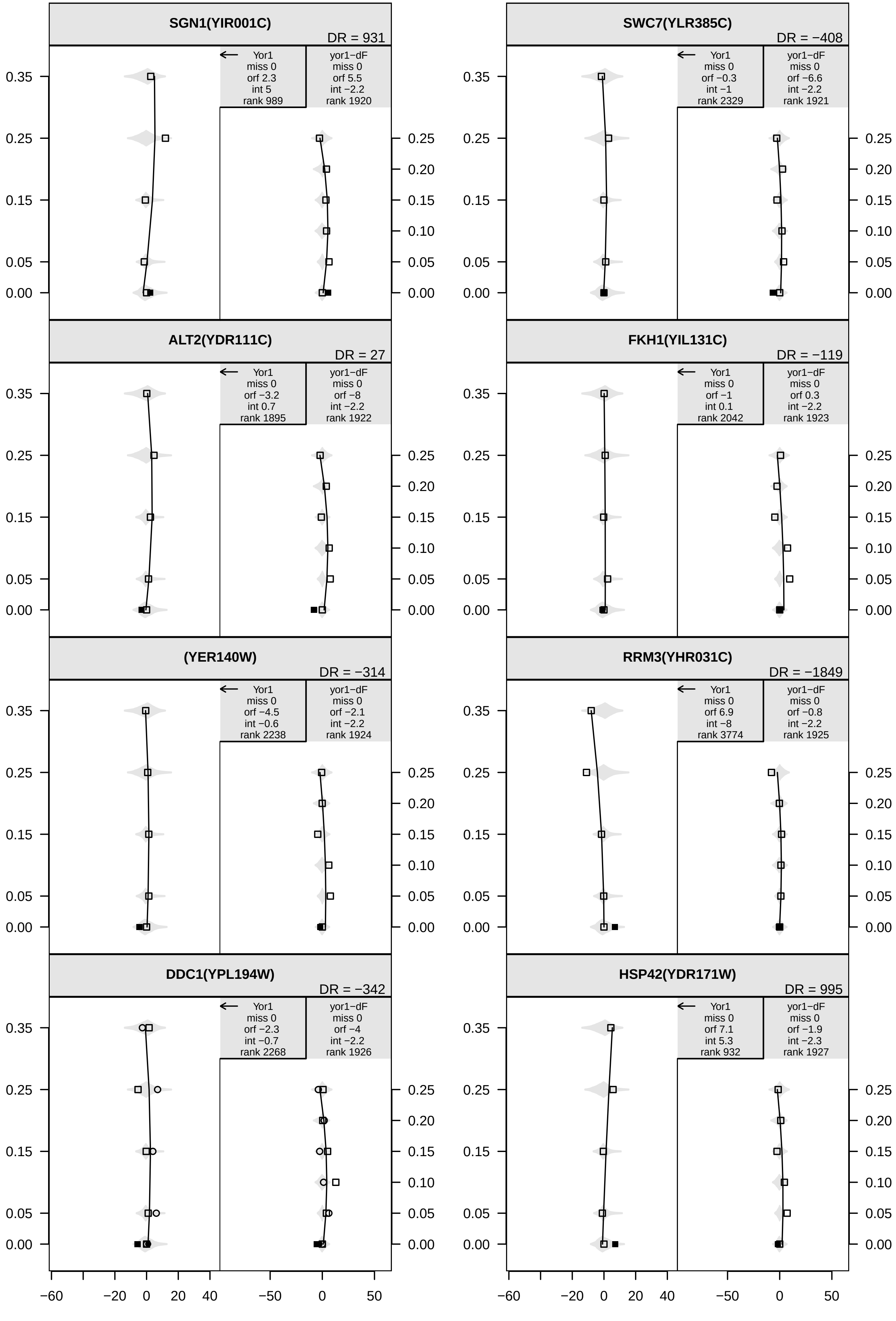

SGN1(YIR001C)
DR = 931
Yor1 miss 0 orf 2.3 int 5 rank 989
yor1−dF miss 0 orf 5.5 int −2.2 rank 1920
SWC7(YLR385C)
DR = −408
Yor1 miss 0 orf −0.3 int −1 rank 2329
yor1−dF miss 0 orf −6.6 int −2.2 rank 1921
ALT2(YDR111C)
DR = 27
Yor1 miss 0 orf −3.2 int 0.7 rank 1895
yor1−dF miss 0 orf −8 int −2.2 rank 1922
FKH1(YIL131C)
DR = −119
Yor1 miss 0 orf −1 int 0.1 rank 2042
yor1−dF miss 0 orf 0.3 int −2.2 rank 1923
(YER140W)
DR = −314
Yor1 miss 0 orf −4.5 int −0.6 rank 2238
yor1−dF miss 0 orf −2.1 int −2.2 rank 1924
RRM3(YHR031C)
DR = −1849
Yor1 miss 0 orf 6.9 int −8 rank 3774
yor1−dF miss 0 orf −0.8 int −2.2 rank 1925
DDC1(YPL194W)
DR = −342
Yor1 miss 0 orf −2.3 int −0.7 rank 2268
yor1−dF miss 0 orf −4 int −2.2 rank 1926
HSP42(YDR171W)
DR = 995
Yor1 miss 0 orf 7.1 int 5.3 rank 932
yor1−dF miss 0 orf −1.9 int −2.3 rank 1927
0.35 0.25 0.20 0.15 0.10 0.05 0.00
−60 −20 0 20 40 −50 0 50

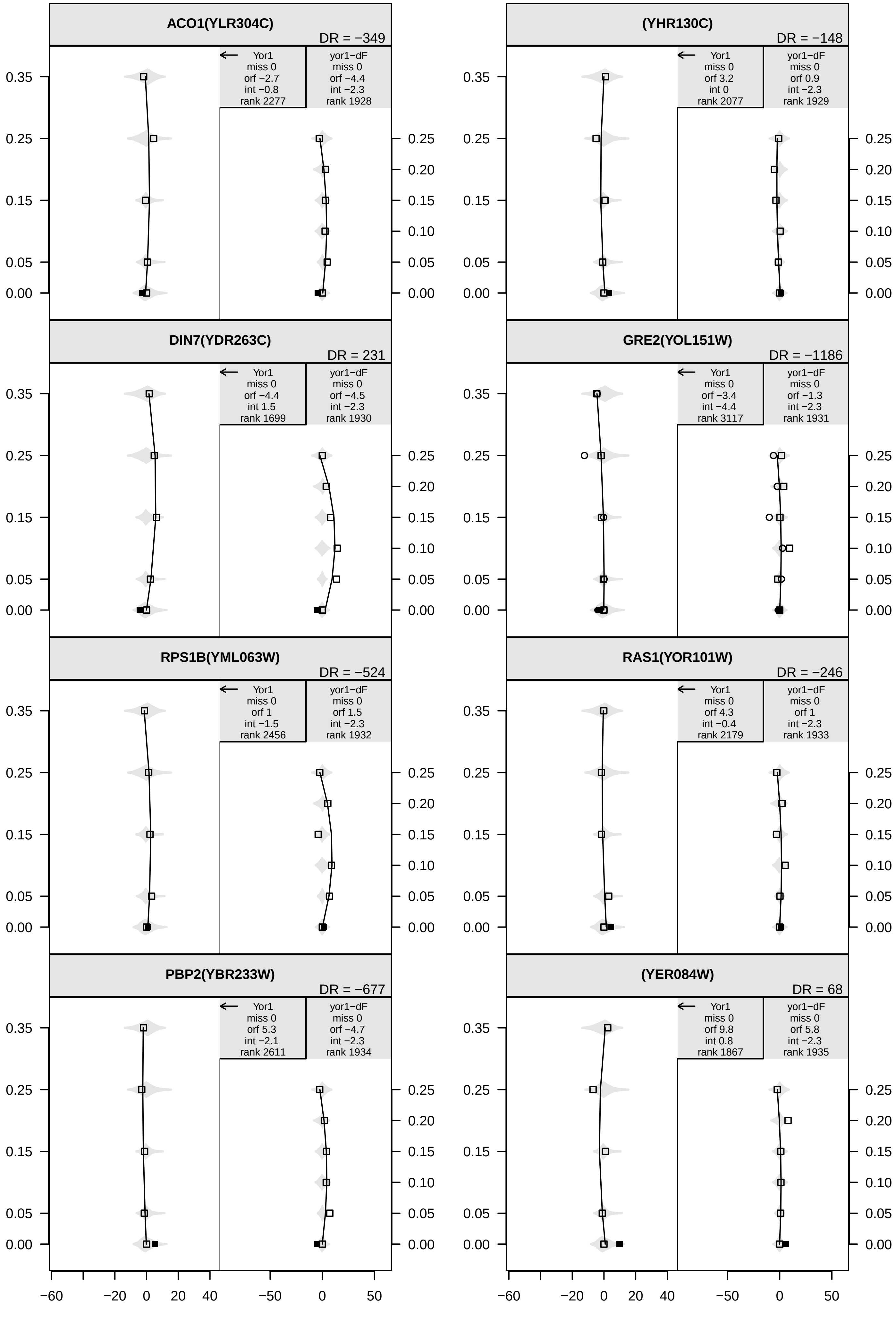
ACO1(YLR304C)
DR = −349
Yor1 miss 0 orf −2.7 int −0.8 rank 2277
yor1−dF miss 0 orf −4.4 int −2.3 rank 1928
(YHR130C)
DR = −148
Yor1 miss 0 orf 3.2 int 0 rank 2077
yor1−dF miss 0 orf 0.9 int −2.3 rank 1929
DIN7(YDR263C)
DR = 231
Yor1 miss 0 orf −4.4 int 1.5 rank 1699
yor1−dF miss 0 orf −4.5 int −2.3 rank 1930
GRE2(YOL151W)
DR = −1186
Yor1 miss 0 orf −3.4 int −4.4 rank 3117
yor1−dF miss 0 orf −1.3 int −2.3 rank 1931
RPS1B(YML063W)
DR = −524
Yor1 miss 0 orf 1 int −1.5 rank 2456
yor1−dF miss 0 orf 1.5 int −2.3 rank 1932
RAS1(YOR101W)
DR = −246
Yor1 miss 0 orf 4.3 int −0.4 rank 2179
yor1−dF miss 0 orf 1 int −2.3 rank 1933
PBP2(YBR233W)
DR = −677
Yor1 miss 0 orf 5.3 int −2.1 rank 2611
yor1−dF miss 0 orf −4.7 int −2.3 rank 1934
(YER084W)
DR = 68
Yor1 miss 0 orf 9.8 int 0.8 rank 1867
yor1−dF miss 0 orf 5.8 int −2.3 rank 1935
0.00 0.05 0.10 0.15 0.20 0.25 0.35
−60 −20 0 20 40 −50 0 50

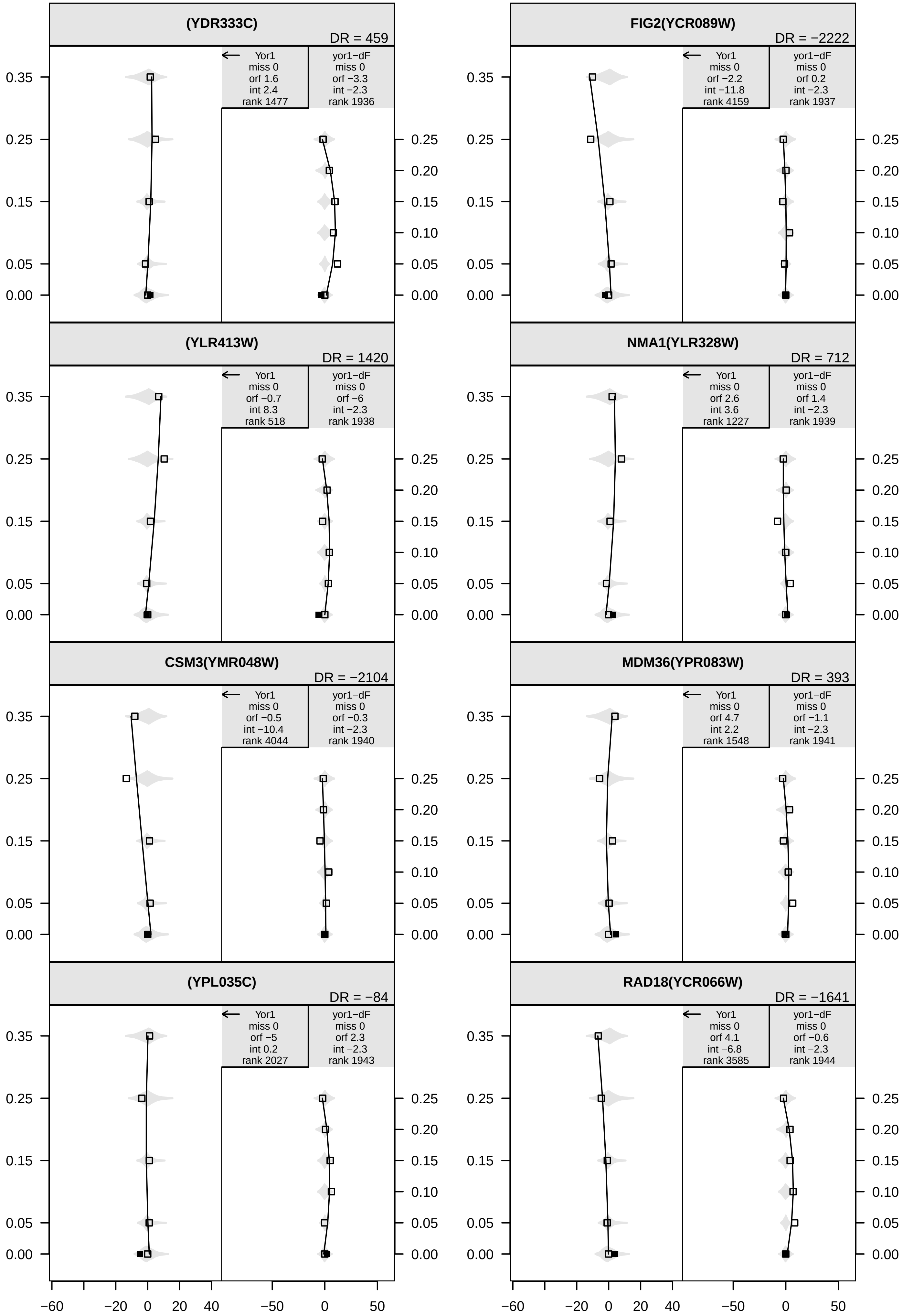
(YDR333C)
DR = 459
Yor1 miss 0 orf 1.6 int 2.4 rank 1477
yor1−dF miss 0 orf −3.3 int −2.3 rank 1936
FIG2(YCR089W)
DR = −2222
Yor1 miss 0 orf −2.2 int −11.8 rank 4159
yor1−dF miss 0 orf 0.2 int −2.3 rank 1937
(YLR413W)
DR = 1420
Yor1 miss 0 orf −0.7 int 8.3 rank 518
yor1−dF miss 0 orf −6 int −2.3 rank 1938
NMA1(YLR328W)
DR = 712
Yor1 miss 0 orf 2.6 int 3.6 rank 1227
yor1−dF miss 0 orf 1.4 int −2.3 rank 1939
CSM3(YMR048W)
DR = −2104
Yor1 miss 0 orf −0.5 int −10.4 rank 4044
yor1−dF miss 0 orf −0.3 int −2.3 rank 1940
MDM36(YPR083W)
DR = 393
Yor1 miss 0 orf 4.7 int 2.2 rank 1548
yor1−dF miss 0 orf −1.1 int −2.3 rank 1941
(YPL035C)
DR = −84
Yor1 miss 0 orf −5 int 0.2 rank 2027
yor1−dF miss 0 orf 2.3 int −2.3 rank 1943
RAD18(YCR066W)
DR = −1641
Yor1 miss 0 orf 4.1 int −6.8 rank 3585
yor1−dF miss 0 orf −0.6 int −2.3 rank 1944

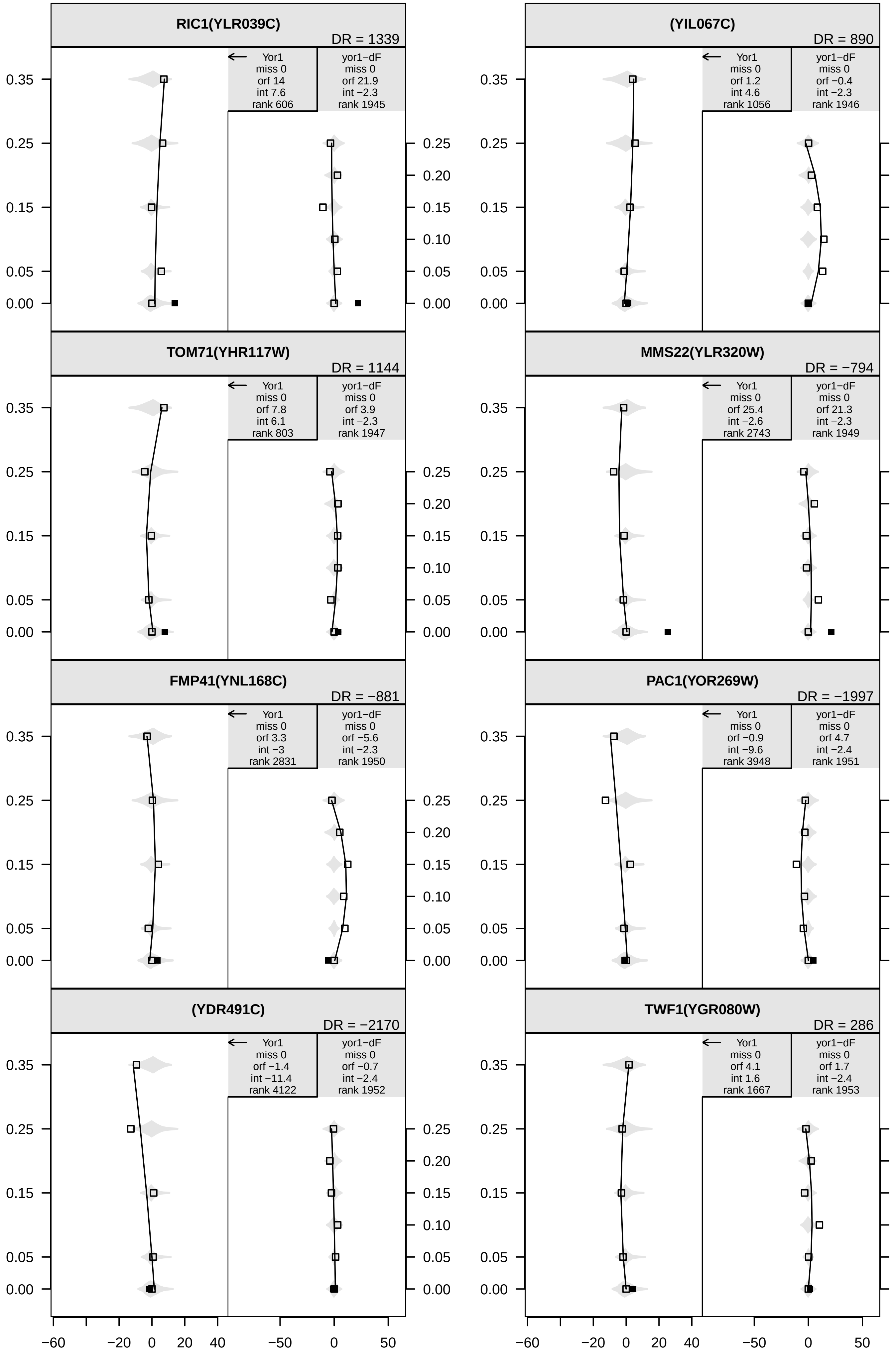

RIC1(YLR039C)
DR = 1339
Yor1 miss 0 orf 14 int 7.6 rank 606
yor1−dF miss 0 orf 21.9 int −2.3 rank 1945
(YIL067C)
DR = 890
Yor1 miss 0 orf 1.2 int 4.6 rank 1056
yor1−dF miss 0 orf −0.4 int −2.3 rank 1946
TOM71(YHR117W)
DR = 1144
Yor1 miss 0 orf 7.8 int 6.1 rank 803
yor1−dF miss 0 orf 3.9 int −2.3 rank 1947
MMS22(YLR320W)
DR = −794
Yor1 miss 0 orf 25.4 int −2.6 rank 2743
yor1−dF miss 0 orf 21.3 int −2.3 rank 1949
FMP41(YNL168C)
DR = −881
Yor1 miss 0 orf 3.3 int −3 rank 2831
yor1−dF miss 0 orf −5.6 int −2.3 rank 1950
PAC1(YOR269W)
DR = −1997
Yor1 miss 0 orf −0.9 int −9.6 rank 3948
yor1−dF miss 0 orf 4.7 int −2.4 rank 1951
(YDR491C)
DR = −2170
Yor1 miss 0 orf −1.4 int −11.4 rank 4122
yor1−dF miss 0 orf −0.7 int −2.4 rank 1952
TWF1(YGR080W)
DR = 286
Yor1 miss 0 orf 4.1 int 1.6 rank 1667
yor1−dF miss 0 orf 1.7 int −2.4 rank 1953
0.35 0.25 0.20 0.15 0.10 0.05 0.00
−60 −20 0 20 40 −50 0 50

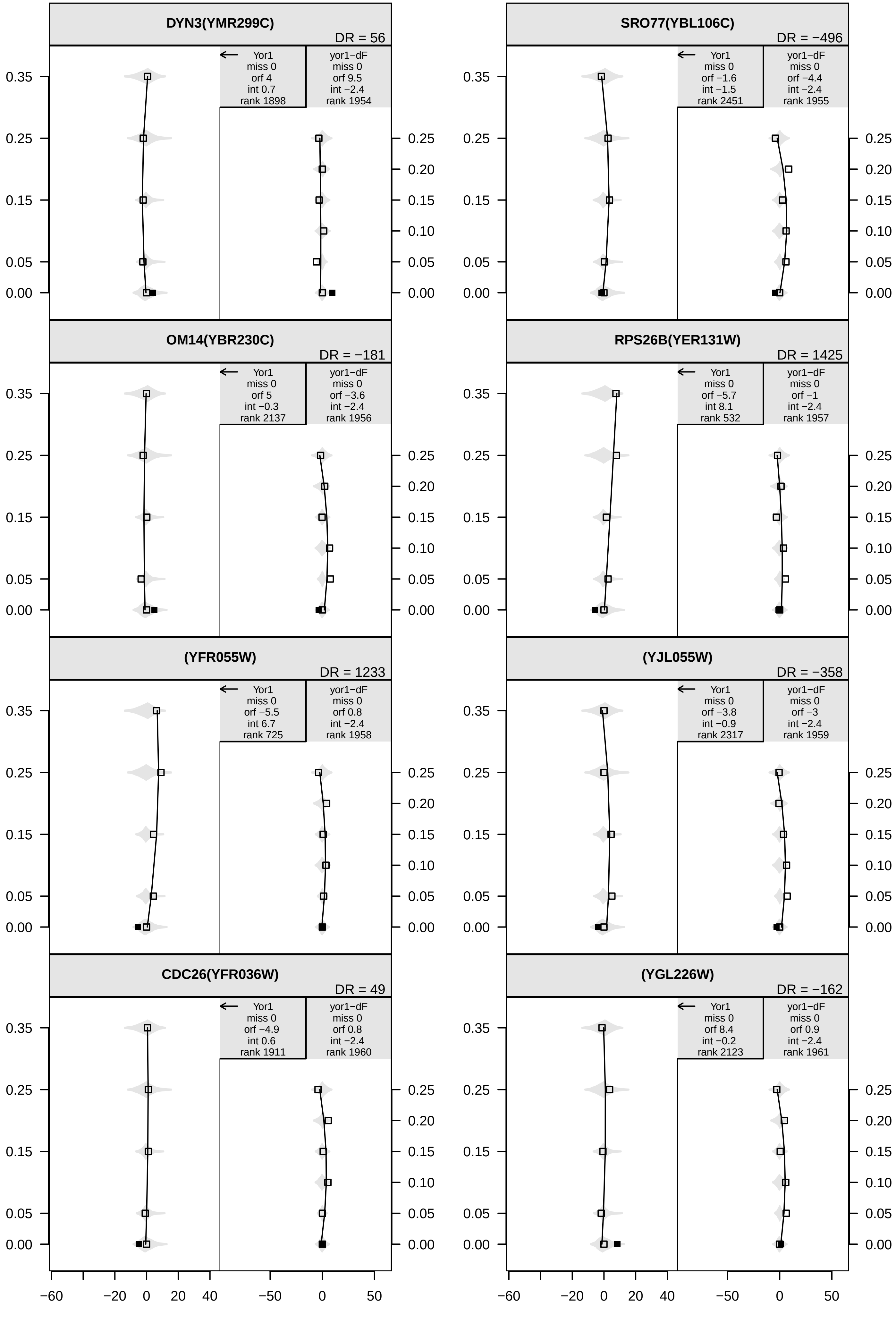
DYN3(YMR299C)
DR = 56
Yor1 miss 0 orf 4 int 0.7 rank 1898
yor1−dF miss 0 orf 9.5 int −2.4 rank 1954
SRO77(YBL106C)
DR = −496
Yor1 miss 0 orf −1.6 int −1.5 rank 2451
yor1−dF miss 0 orf −4.4 int −2.4 rank 1955
OM14(YBR230C)
DR = −181
Yor1 miss 0 orf 5 int −0.3 rank 2137
yor1−dF miss 0 orf −3.6 int −2.4 rank 1956
RPS26B(YER131W)
DR = 1425
Yor1 miss 0 orf −5.7 int 8.1 rank 532
yor1−dF miss 0 orf −1 int −2.4 rank 1957
(YFR055W)
DR = 1233
Yor1 miss 0 orf −5.5 int 6.7 rank 725
yor1−dF miss 0 orf 0.8 int −2.4 rank 1958
(YJL055W)
DR = −358
Yor1 miss 0 orf −3.8 int −0.9 rank 2317
yor1−dF miss 0 orf −3 int −2.4 rank 1959
CDC26(YFR036W)
DR = 49
Yor1 miss 0 orf −4.9 int 0.6 rank 1911
yor1−dF miss 0 orf 0.8 int −2.4 rank 1960
(YGL226W)
DR = −162
Yor1 miss 0 orf 8.4 int −0.2 rank 2123
yor1−dF miss 0 orf 0.9 int −2.4 rank 1961
0.35
0.25
0.20
0.15
0.10
0.05
0.00
−60
−20
0
20
40
−50
0
50

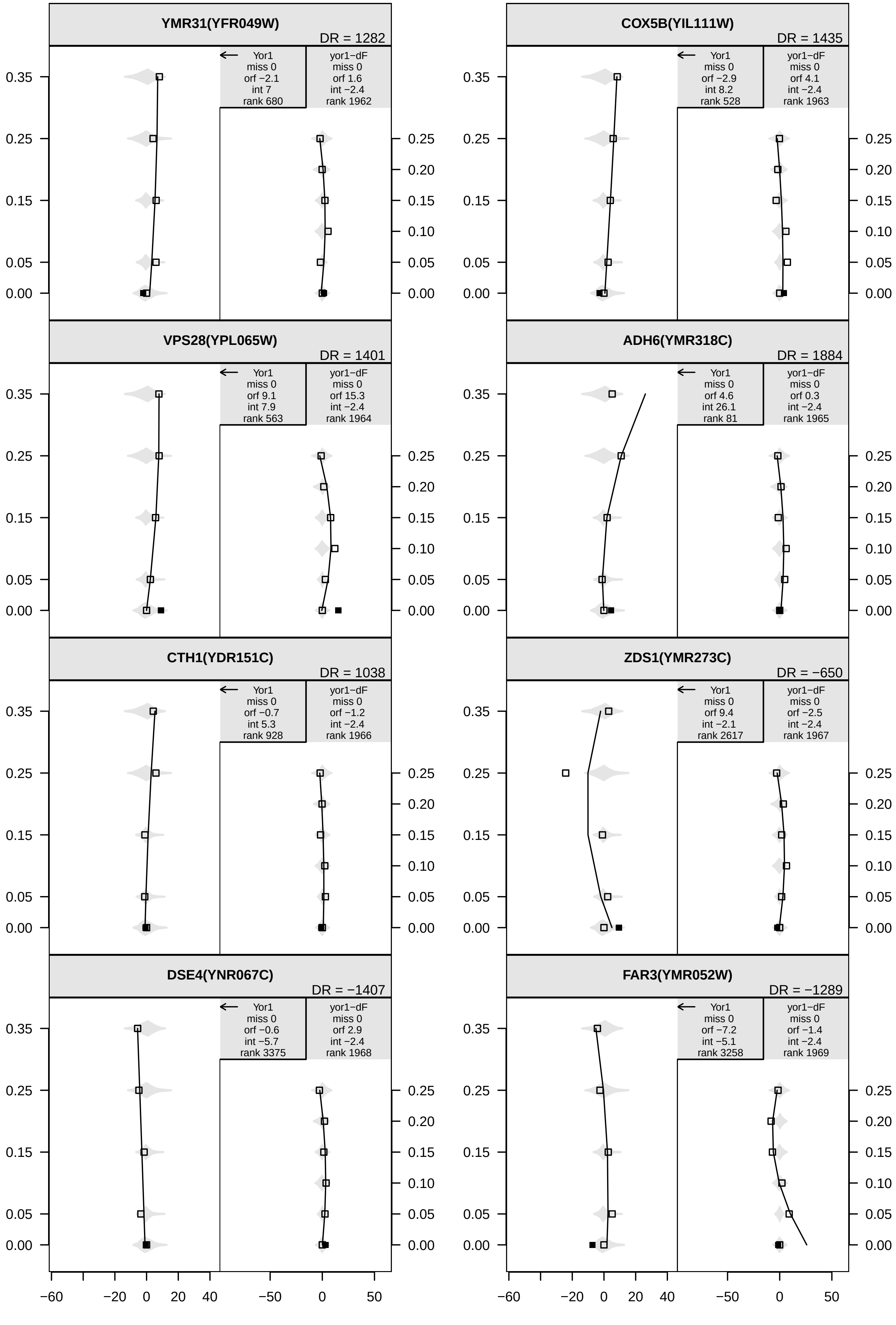
YMR31(YFR049W)
DR = 1282
Yor1
miss 0
orf −2.1
int 7
rank 680
yor1−dF
miss 0
orf 1.6
int −2.4
rank 1962
COX5B(YIL111W)
DR = 1435
Yor1
miss 0
orf −2.9
int 8.2
rank 528
yor1−dF
miss 0
orf 4.1
int −2.4
rank 1963
VPS28(YPL065W)
DR = 1401
Yor1
miss 0
orf 9.1
int 7.9
rank 563
yor1−dF
miss 0
orf 15.3
int −2.4
rank 1964
ADH6(YMR318C)
DR = 1884
Yor1
miss 0
orf 4.6
int 26.1
rank 81
yor1−dF
miss 0
orf 0.3
int −2.4
rank 1965
CTH1(YDR151C)
DR = 1038
Yor1
miss 0
orf −0.7
int 5.3
rank 928
yor1−dF
miss 0
orf −1.2
int −2.4
rank 1966
ZDS1(YMR273C)
DR = −650
Yor1
miss 0
orf 9.4
int −2.1
rank 2617
yor1−dF
miss 0
orf −2.5
int −2.4
rank 1967
DSE4(YNR067C)
DR = −1407
Yor1
miss 0
orf −0.6
int −5.7
rank 3375
yor1−dF
miss 0
orf 2.9
int −2.4
rank 1968
FAR3(YMR052W)
DR = −1289
Yor1
miss 0
orf −7.2
int −5.1
rank 3258
yor1−dF
miss 0
orf −1.4
int −2.4
rank 1969
0.35
0.25
0.20
0.15
0.10
0.05
0.00
−60
−20
0
20
40
−50
0
50

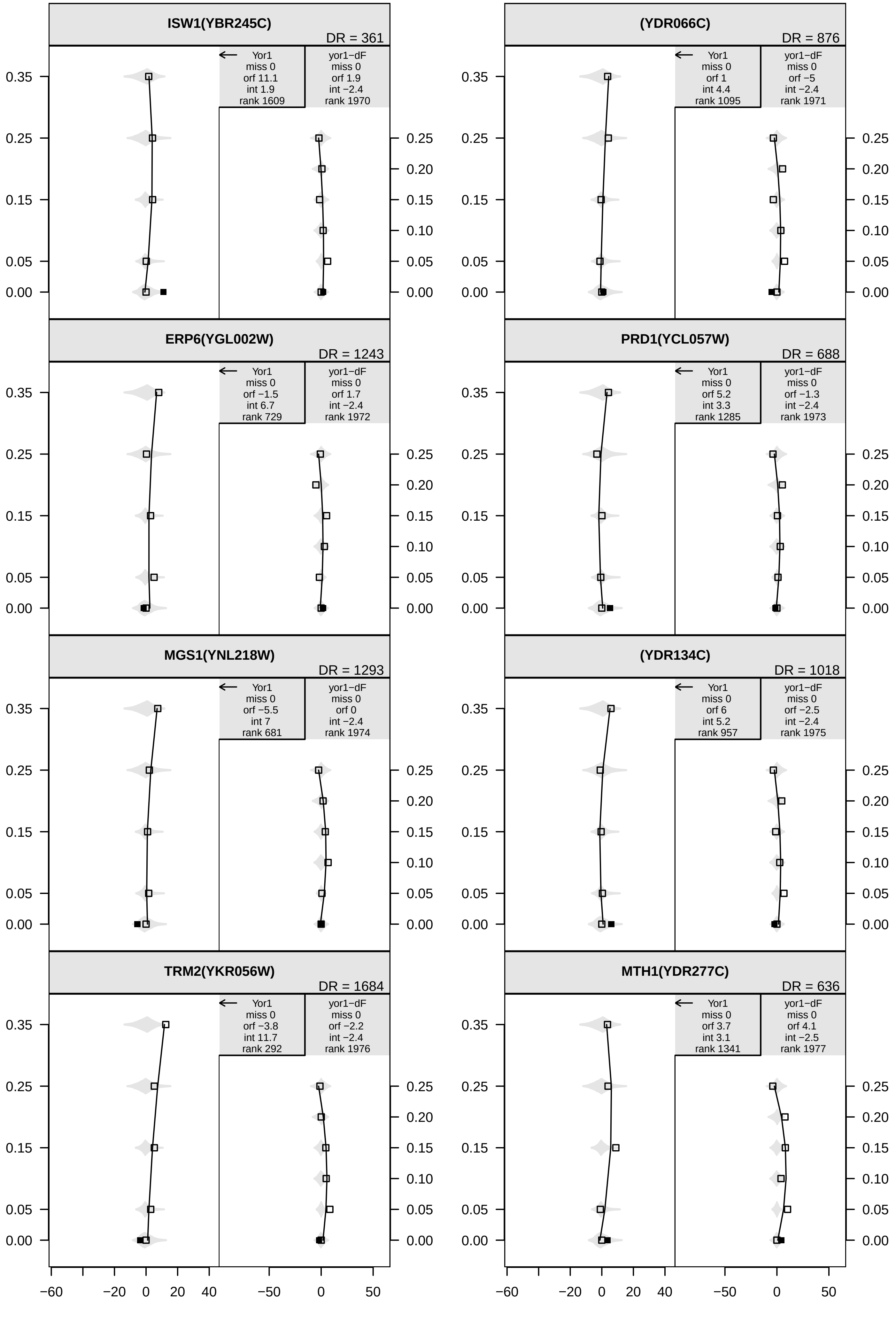
ISW1(YBR245C)
DR = 361
Yor1 miss 0 orf 11.1 int 1.9 rank 1609
yor1−dF miss 0 orf 1.9 int −2.4 rank 1970
(YDR066C)
DR = 876
Yor1 miss 0 orf 1 int 4.4 rank 1095
yor1−dF miss 0 orf −5 int −2.4 rank 1971
ERP6(YGL002W)
DR = 1243
Yor1 miss 0 orf −1.5 int 6.7 rank 729
yor1−dF miss 0 orf 1.7 int −2.4 rank 1972
PRD1(YCL057W)
DR = 688
Yor1 miss 0 orf 5.2 int 3.3 rank 1285
yor1−dF miss 0 orf −1.3 int −2.4 rank 1973
MGS1(YNL218W)
DR = 1293
Yor1 miss 0 orf −5.5 int 7 rank 681
yor1−dF miss 0 orf 0 int −2.4 rank 1974
(YDR134C)
DR = 1018
Yor1 miss 0 orf 6 int 5.2 rank 957
yor1−dF miss 0 orf −2.5 int −2.4 rank 1975
TRM2(YKR056W)
DR = 1684
Yor1 miss 0 orf −3.8 int 11.7 rank 292
yor1−dF miss 0 orf −2.2 int −2.4 rank 1976
MTH1(YDR277C)
DR = 636
Yor1 miss 0 orf 3.7 int 3.1 rank 1341
yor1−dF miss 0 orf 4.1 int −2.5 rank 1977
0.35 0.25 0.15 0.05 0.00
0.25 0.20 0.15 0.10 0.05 0.00
−60 −20 0 20 40
−50 0 50

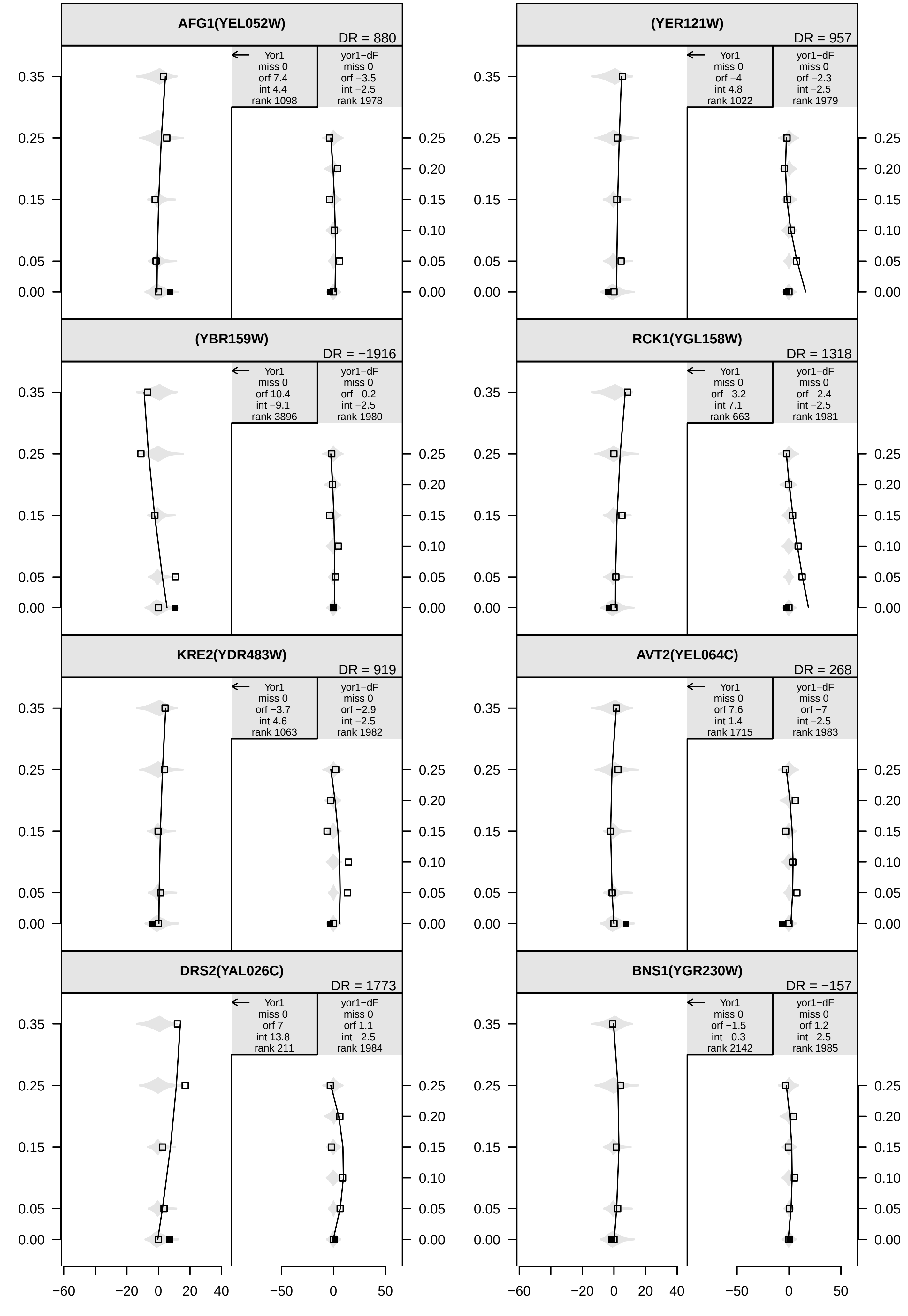

AFG1(YEL052W)
DR = 880
Yor1 miss 0 orf 7.4 int 4.4 rank 1098
yor1−dF miss 0 orf −3.5 int −2.5 rank 1978
(YER121W)
DR = 957
Yor1 miss 0 orf −4 int 4.8 rank 1022
yor1−dF miss 0 orf −2.3 int −2.5 rank 1979
(YBR159W)
DR = −1916
Yor1 miss 0 orf 10.4 int −9.1 rank 3896
yor1−dF miss 0 orf −0.2 int −2.5 rank 1980
RCK1(YGL158W)
DR = 1318
Yor1 miss 0 orf −3.2 int 7.1 rank 663
yor1−dF miss 0 orf −2.4 int −2.5 rank 1981
KRE2(YDR483W)
DR = 919
Yor1 miss 0 orf −3.7 int 4.6 rank 1063
yor1−dF miss 0 orf −2.9 int −2.5 rank 1982
AVT2(YEL064C)
DR = 268
Yor1 miss 0 orf 7.6 int 1.4 rank 1715
yor1−dF miss 0 orf −7 int −2.5 rank 1983
DRS2(YAL026C)
DR = 1773
Yor1 miss 0 orf 7 int 13.8 rank 211
yor1−dF miss 0 orf 1.1 int −2.5 rank 1984
BNS1(YGR230W)
DR = −157
Yor1 miss 0 orf −1.5 int −0.3 rank 2142
yor1−dF miss 0 orf 1.2 int −2.5 rank 1985
0.00 0.05 0.15 0.25 0.35
0.00 0.05 0.10 0.15 0.20 0.25
−60 −20 0 20 40
−50 0 50

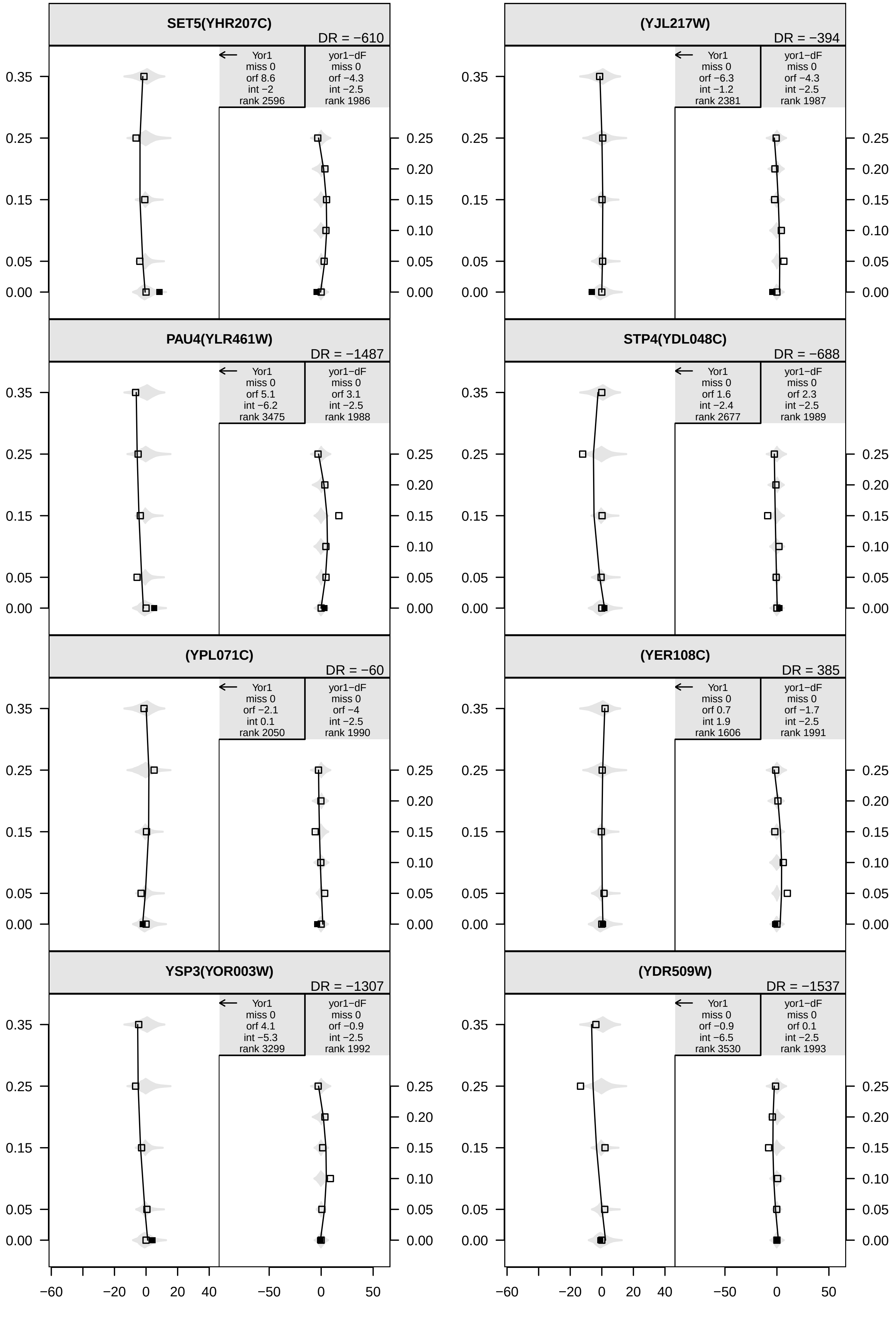
SET5(YHR207C)
DR = −610
Yor1 miss 0 orf 8.6 int −2 rank 2596
yor1−dF miss 0 orf −4.3 int −2.5 rank 1986
(YJL217W)
DR = −394
Yor1 miss 0 orf −6.3 int −1.2 rank 2381
yor1−dF miss 0 orf −4.3 int −2.5 rank 1987
PAU4(YLR461W)
DR = −1487
Yor1 miss 0 orf 5.1 int −6.2 rank 3475
yor1−dF miss 0 orf 3.1 int −2.5 rank 1988
STP4(YDL048C)
DR = −688
Yor1 miss 0 orf 1.6 int −2.4 rank 2677
yor1−dF miss 0 orf 2.3 int −2.5 rank 1989
(YPL071C)
DR = −60
Yor1 miss 0 orf −2.1 int 0.1 rank 2050
yor1−dF miss 0 orf −4 int −2.5 rank 1990
(YER108C)
DR = 385
Yor1 miss 0 orf 0.7 int 1.9 rank 1606
yor1−dF miss 0 orf −1.7 int −2.5 rank 1991
YSP3(YOR003W)
DR = −1307
Yor1 miss 0 orf 4.1 int −5.3 rank 3299
yor1−dF miss 0 orf −0.9 int −2.5 rank 1992
(YDR509W)
DR = −1537
Yor1 miss 0 orf −0.9 int −6.5 rank 3530
yor1−dF miss 0 orf 0.1 int −2.5 rank 1993
0.35 0.25 0.15 0.05 0.00
0.25 0.20 0.15 0.10 0.05 0.00
−60 −20 0 20 40 −50 0 50

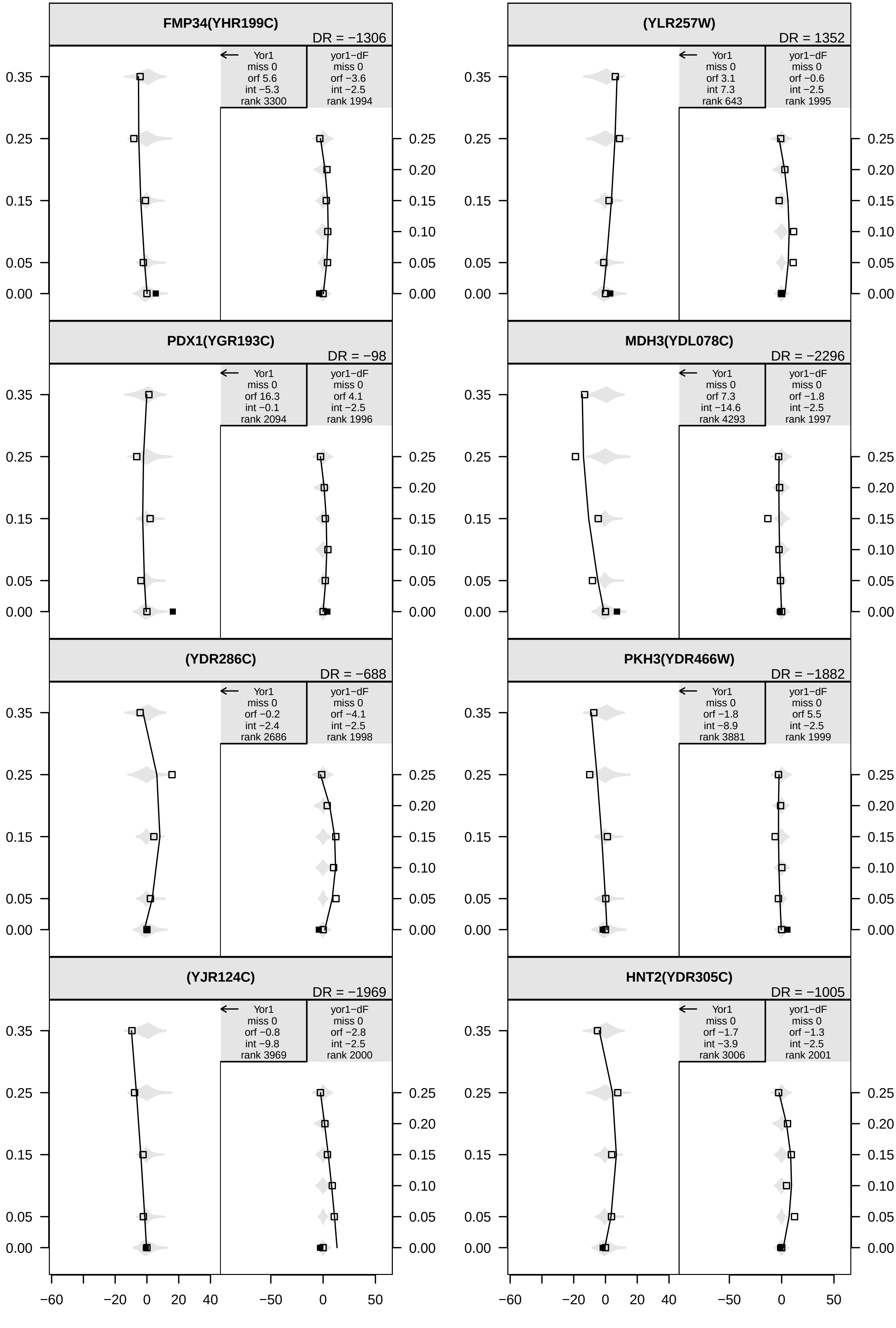
FMP34(YHR199C)
DR = −1306
Yor1 miss 0 orf 5.6 int −5.3 rank 3300
yor1−dF miss 0 orf −3.6 int −2.5 rank 1994
(YLR257W)
DR = 1352
Yor1 miss 0 orf 3.1 int 7.3 rank 643
yor1−dF miss 0 orf −0.6 int −2.5 rank 1995
PDX1(YGR193C)
DR = −98
Yor1 miss 0 orf 16.3 int −0.1 rank 2094
yor1−dF miss 0 orf 4.1 int −2.5 rank 1996
MDH3(YDL078C)
DR = −2296
Yor1 miss 0 orf 7.3 int −14.6 rank 4293
yor1−dF miss 0 orf −1.8 int −2.5 rank 1997
(YDR286C)
DR = −688
Yor1 miss 0 orf −0.2 int −2.4 rank 2686
yor1−dF miss 0 orf −4.1 int −2.5 rank 1998
PKH3(YDR466W)
DR = −1882
Yor1 miss 0 orf −1.8 int −8.9 rank 3881
yor1−dF miss 0 orf 5.5 int −2.5 rank 1999
(YJR124C)
DR = −1969
Yor1 miss 0 orf −0.8 int −9.8 rank 3969
yor1−dF miss 0 orf −2.8 int −2.5 rank 2000
HNT2(YDR305C)
DR = −1005
Yor1 miss 0 orf −1.7 int −3.9 rank 3006
yor1−dF miss 0 orf −1.3 int −2.5 rank 2001
0.35 0.25 0.15 0.05 0.00
0.25 0.20 0.15 0.10 0.05 0.00
−60 −20 0 20 40 −50 0 50

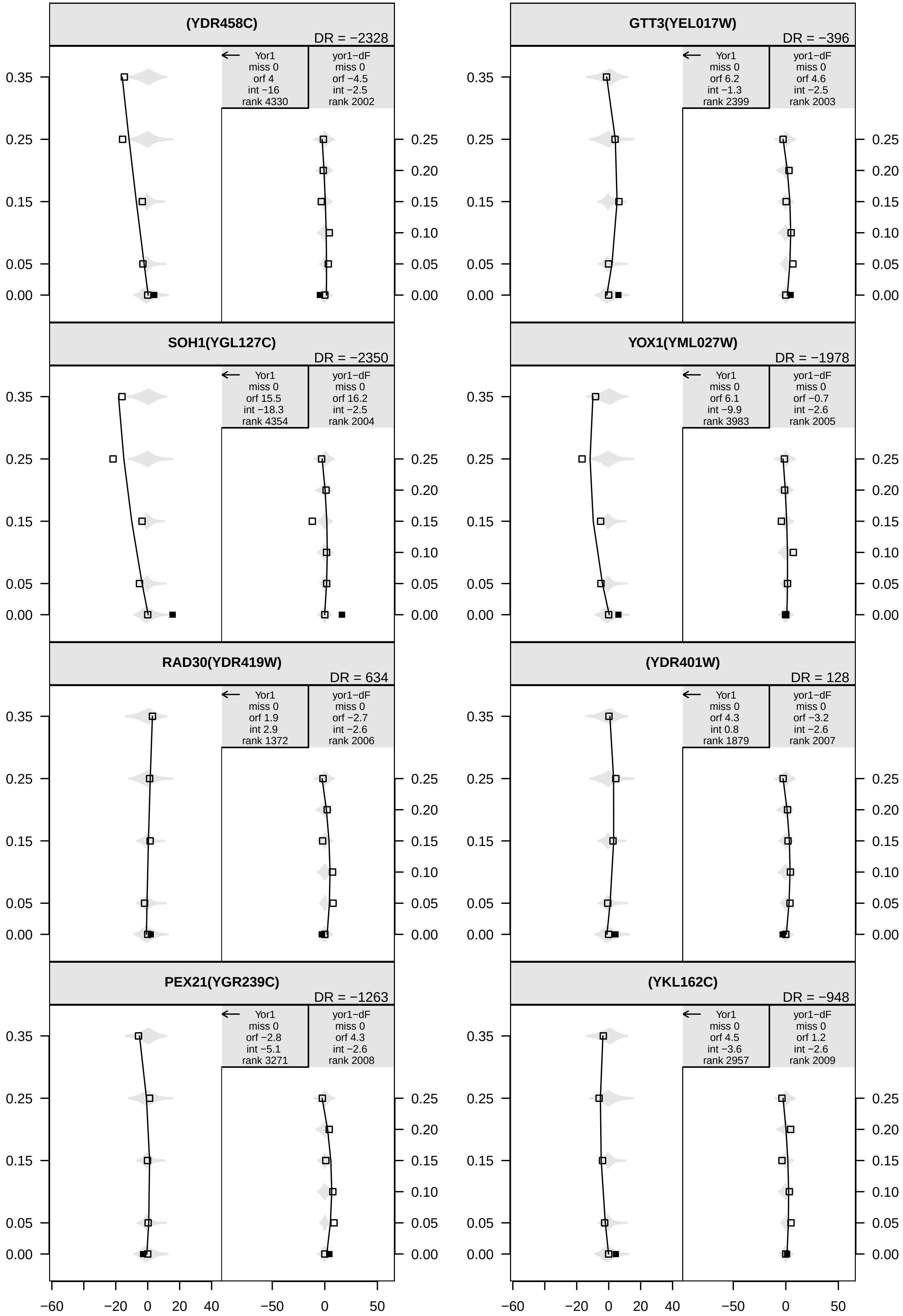
(YDR458C)
DR = −2328
Yor1
miss 0
orf 4
int −16
rank 4330
yor1−dF
miss 0
orf −4.5
int −2.5
rank 2002
GTT3(YEL017W)
DR = −396
Yor1
miss 0
orf 6.2
int −1.3
rank 2399
yor1−dF
miss 0
orf 4.6
int −2.5
rank 2003
SOH1(YGL127C)
DR = −2350
Yor1
miss 0
orf 15.5
int −18.3
rank 4354
yor1−dF
miss 0
orf 16.2
int −2.5
rank 2004
YOX1(YML027W)
DR = −1978
Yor1
miss 0
orf 6.1
int −9.9
rank 3983
yor1−dF
miss 0
orf −0.7
int −2.6
rank 2005
RAD30(YDR419W)
DR = 634
Yor1
miss 0
orf 1.9
int 2.9
rank 1372
yor1−dF
miss 0
orf −2.7
int −2.6
rank 2006
(YDR401W)
DR = 128
Yor1
miss 0
orf 4.3
int 0.8
rank 1879
yor1−dF
miss 0
orf −3.2
int −2.6
rank 2007
PEX21(YGR239C)
DR = −1263
Yor1
miss 0
orf −2.8
int −5.1
rank 3271
yor1−dF
miss 0
orf 4.3
int −2.6
rank 2008
(YKL162C)
DR = −948
Yor1
miss 0
orf 4.5
int −3.6
rank 2957
yor1−dF
miss 0
orf 1.2
int −2.6
rank 2009
0.35 0.25 0.15 0.05 0.00
0.25 0.20 0.15 0.10 0.05 0.00
−60 −20 0 20 40 −50 0 50

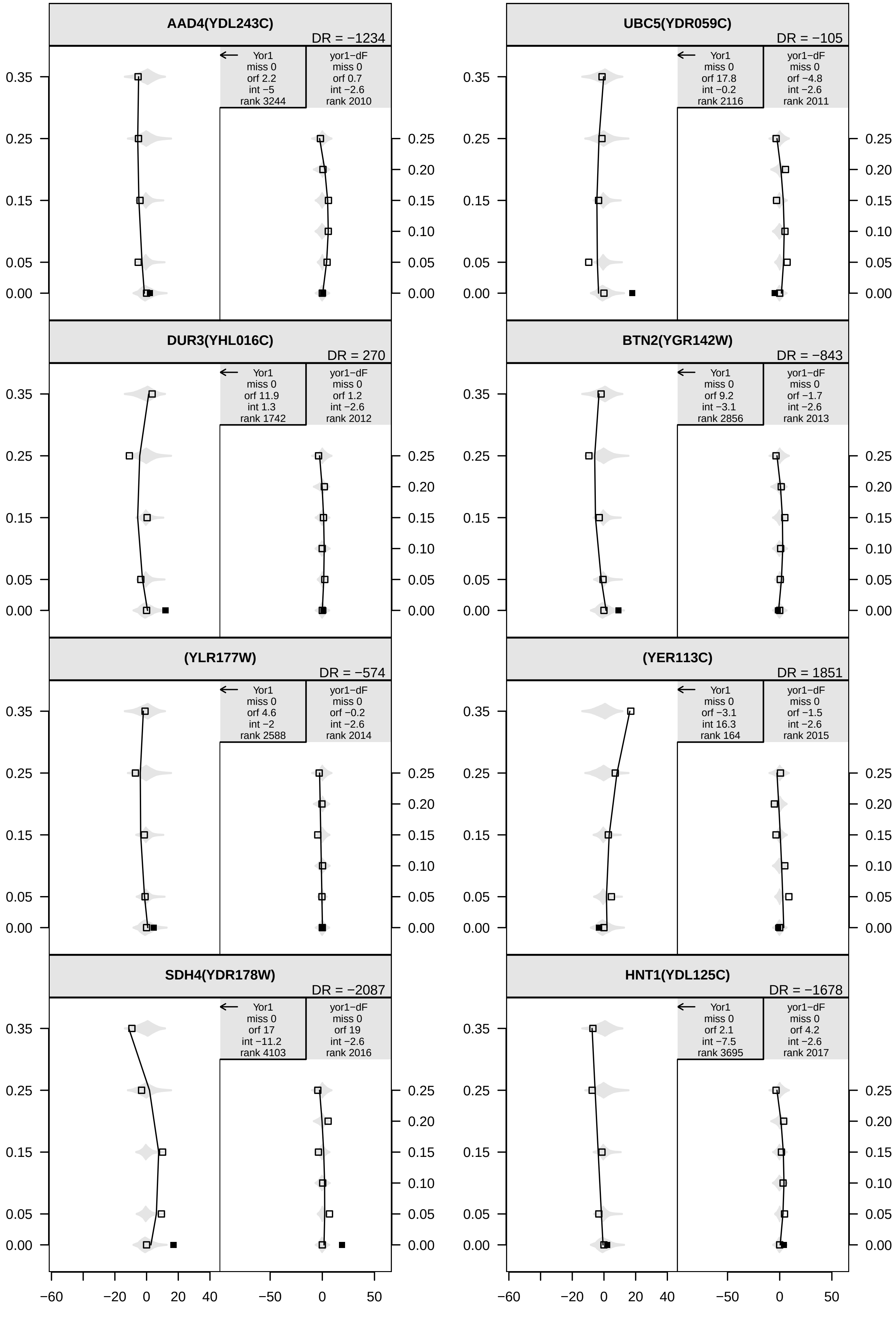
AAD4(YDL243C)
DR = −1234
Yor1 miss 0 orf 2.2 int −5 rank 3244
yor1−dF miss 0 orf 0.7 int −2.6 rank 2010
UBC5(YDR059C)
DR = −105
Yor1 miss 0 orf 17.8 int −0.2 rank 2116
yor1−dF miss 0 orf −4.8 int −2.6 rank 2011
DUR3(YHL016C)
DR = 270
Yor1 miss 0 orf 11.9 int 1.3 rank 1742
yor1−dF miss 0 orf 1.2 int −2.6 rank 2012
BTN2(YGR142W)
DR = −843
Yor1 miss 0 orf 9.2 int −3.1 rank 2856
yor1−dF miss 0 orf −1.7 int −2.6 rank 2013
(YLR177W)
DR = −574
Yor1 miss 0 orf 4.6 int −2 rank 2588
yor1−dF miss 0 orf −0.2 int −2.6 rank 2014
(YER113C)
DR = 1851
Yor1 miss 0 orf −3.1 int 16.3 rank 164
yor1−dF miss 0 orf −1.5 int −2.6 rank 2015
SDH4(YDR178W)
DR = −2087
Yor1 miss 0 orf 17 int −11.2 rank 4103
yor1−dF miss 0 orf 19 int −2.6 rank 2016
HNT1(YDL125C)
DR = −1678
Yor1 miss 0 orf 2.1 int −7.5 rank 3695
yor1−dF miss 0 orf 4.2 int −2.6 rank 2017
0.00 0.05 0.10 0.15 0.20 0.25 0.35
−60 −20 0 20 40 −50 0 50

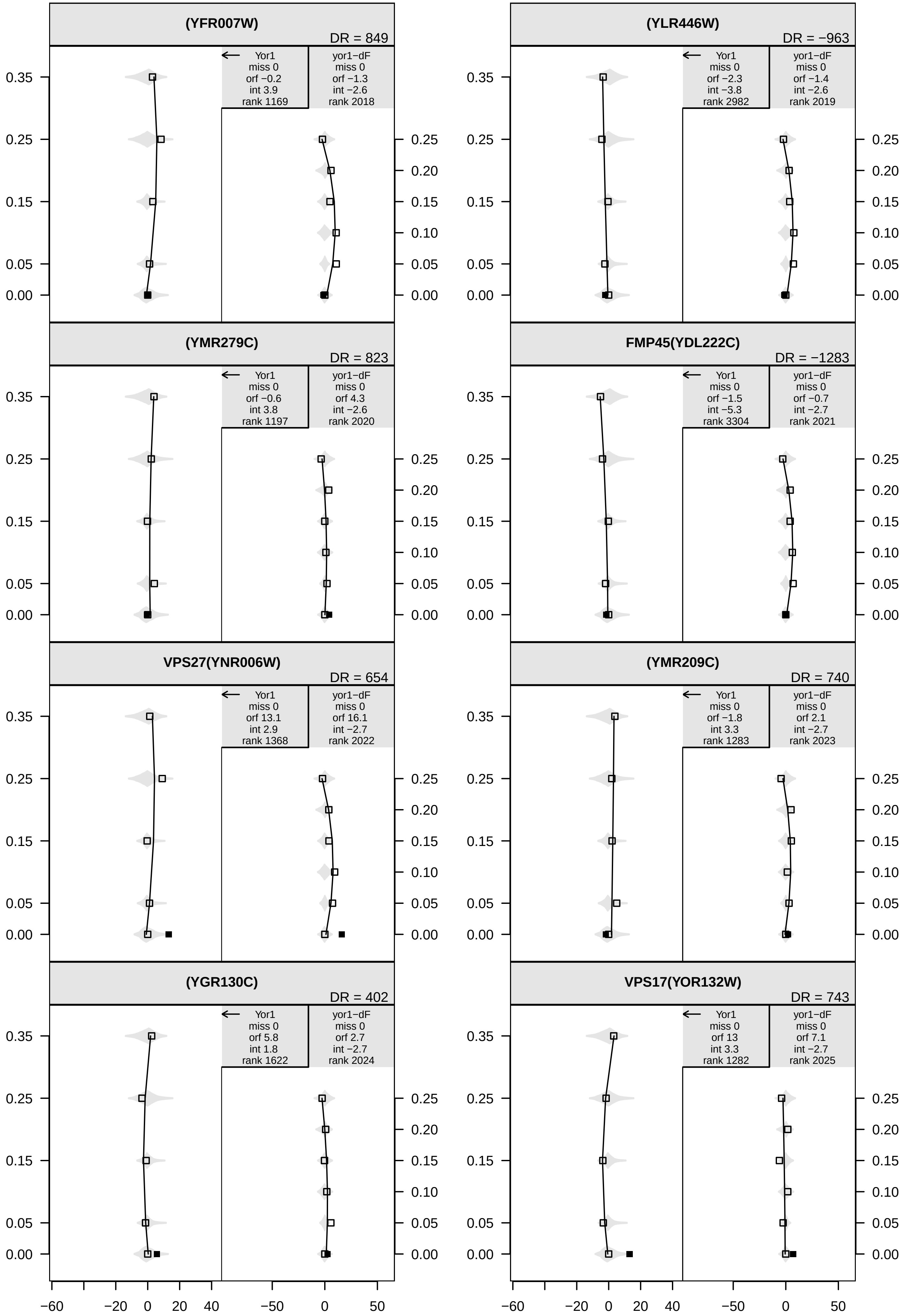
(YFR007W)
DR = 849
Yor1
miss 0
orf −0.2
int 3.9
rank 1169
yor1−dF
miss 0
orf −1.3
int −2.6
rank 2018
(YLR446W)
DR = −963
Yor1
miss 0
orf −2.3
int −3.8
rank 2982
yor1−dF
miss 0
orf −1.4
int −2.6
rank 2019
(YMR279C)
DR = 823
Yor1
miss 0
orf −0.6
int 3.8
rank 1197
yor1−dF
miss 0
orf 4.3
int −2.6
rank 2020
FMP45(YDL222C)
DR = −1283
Yor1
miss 0
orf −1.5
int −5.3
rank 3304
yor1−dF
miss 0
orf −0.7
int −2.7
rank 2021
VPS27(YNR006W)
DR = 654
Yor1
miss 0
orf 13.1
int 2.9
rank 1368
yor1−dF
miss 0
orf 16.1
int −2.7
rank 2022
(YMR209C)
DR = 740
Yor1
miss 0
orf −1.8
int 3.3
rank 1283
yor1−dF
miss 0
orf 2.1
int −2.7
rank 2023
(YGR130C)
DR = 402
Yor1
miss 0
orf 5.8
int 1.8
rank 1622
yor1−dF
miss 0
orf 2.7
int −2.7
rank 2024
VPS17(YOR132W)
DR = 743
Yor1
miss 0
orf 13
int 3.3
rank 1282
yor1−dF
miss 0
orf 7.1
int −2.7
rank 2025
0.35
0.25
0.20
0.15
0.10
0.05
0.00
−60
−20
0
20
40
−50
0
50

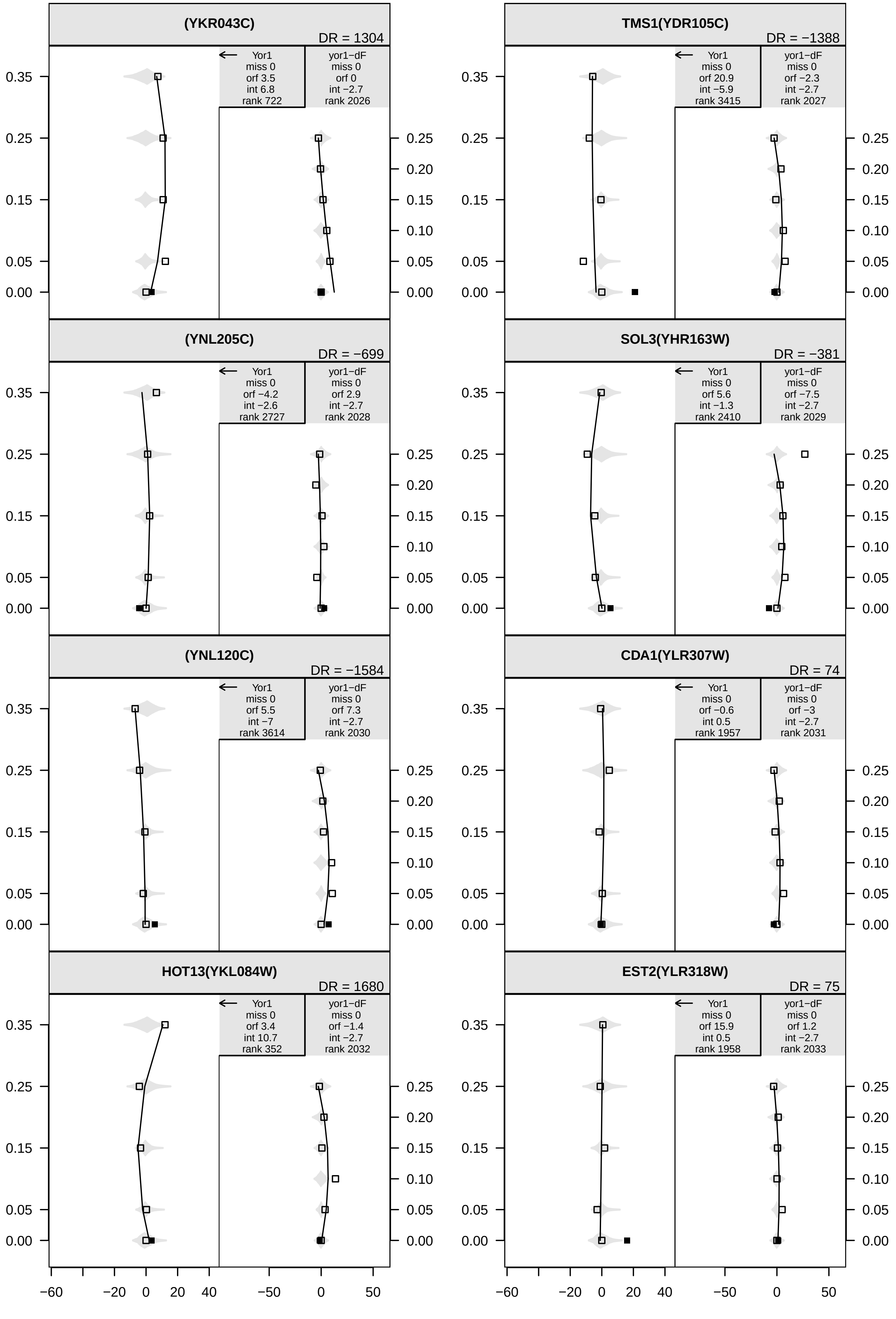

(YKR043C)
DR = 1304
Yor1 miss 0 orf 3.5 int 6.8 rank 722
yor1−dF miss 0 orf 0 int −2.7 rank 2026
TMS1(YDR105C)
DR = −1388
Yor1 miss 0 orf 20.9 int −5.9 rank 3415
yor1−dF miss 0 orf −2.3 int −2.7 rank 2027
(YNL205C)
DR = −699
Yor1 miss 0 orf −4.2 int −2.6 rank 2727
yor1−dF miss 0 orf 2.9 int −2.7 rank 2028
SOL3(YHR163W)
DR = −381
Yor1 miss 0 orf 5.6 int −1.3 rank 2410
yor1−dF miss 0 orf −7.5 int −2.7 rank 2029
(YNL120C)
DR = −1584
Yor1 miss 0 orf 5.5 int −7 rank 3614
yor1−dF miss 0 orf 7.3 int −2.7 rank 2030
CDA1(YLR307W)
DR = 74
Yor1 miss 0 orf −0.6 int 0.5 rank 1957
yor1−dF miss 0 orf −3 int −2.7 rank 2031
HOT13(YKL084W)
DR = 1680
Yor1 miss 0 orf 3.4 int 10.7 rank 352
yor1−dF miss 0 orf −1.4 int −2.7 rank 2032
EST2(YLR318W)
DR = 75
Yor1 miss 0 orf 15.9 int 0.5 rank 1958
yor1−dF miss 0 orf 1.2 int −2.7 rank 2033

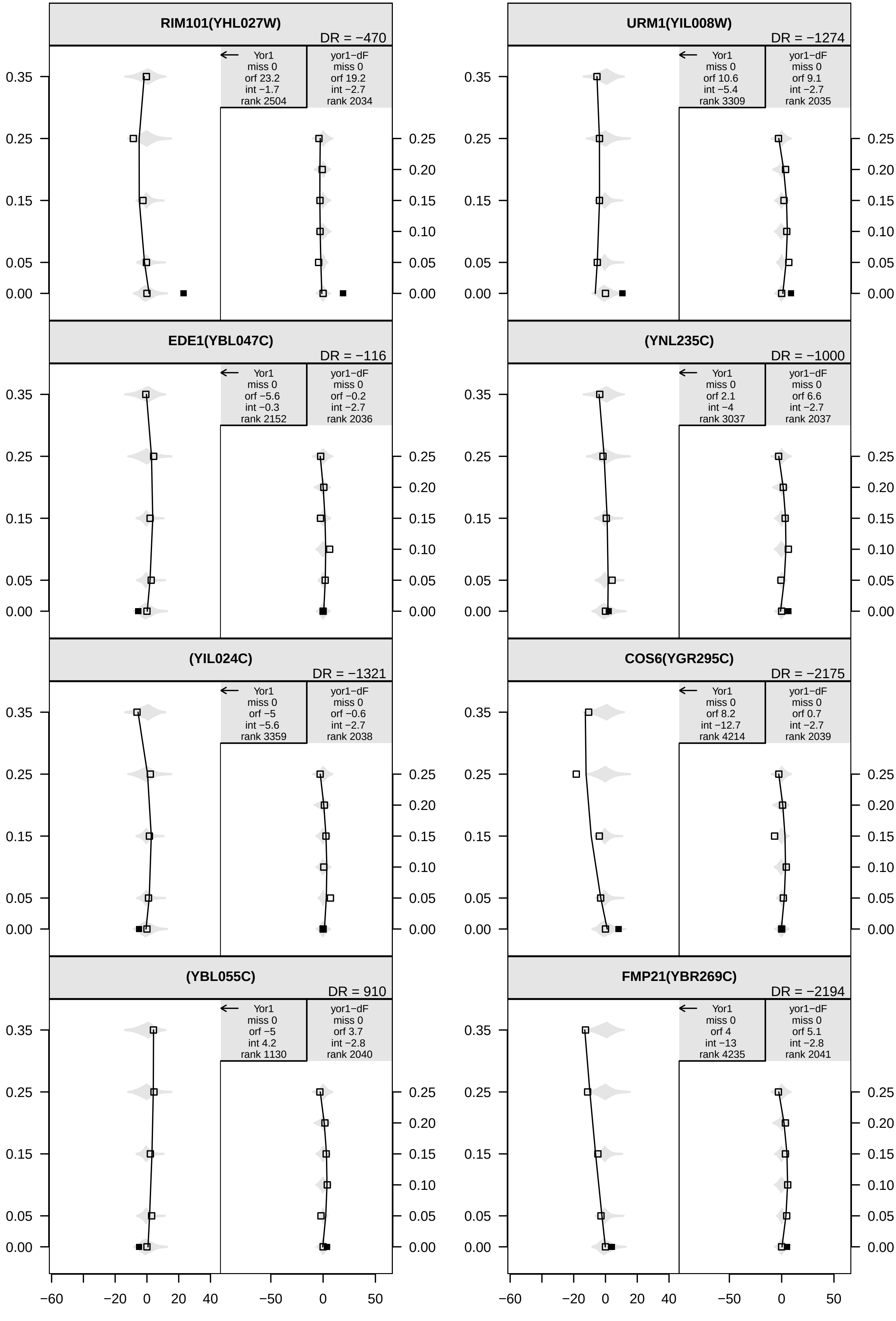
RIM101(YHL027W)
DR = −470
Yor1 miss 0 orf 23.2 int −1.7 rank 2504
yor1−dF miss 0 orf 19.2 int −2.7 rank 2034
URM1(YIL008W)
DR = −1274
Yor1 miss 0 orf 10.6 int −5.4 rank 3309
yor1−dF miss 0 orf 9.1 int −2.7 rank 2035
EDE1(YBL047C)
DR = −116
Yor1 miss 0 orf −5.6 int −0.3 rank 2152
yor1−dF miss 0 orf −0.2 int −2.7 rank 2036
(YNL235C)
DR = −1000
Yor1 miss 0 orf 2.1 int −4 rank 3037
yor1−dF miss 0 orf 6.6 int −2.7 rank 2037
(YIL024C)
DR = −1321
Yor1 miss 0 orf −5 int −5.6 rank 3359
yor1−dF miss 0 orf −0.6 int −2.7 rank 2038
COS6(YGR295C)
DR = −2175
Yor1 miss 0 orf 8.2 int −12.7 rank 4214
yor1−dF miss 0 orf 0.7 int −2.7 rank 2039
(YBL055C)
DR = 910
Yor1 miss 0 orf −5 int 4.2 rank 1130
yor1−dF miss 0 orf 3.7 int −2.8 rank 2040
FMP21(YBR269C)
DR = −2194
Yor1 miss 0 orf 4 int −13 rank 4235
yor1−dF miss 0 orf 5.1 int −2.8 rank 2041
0.35 0.25 0.15 0.05 0.00
0.25 0.20 0.15 0.10 0.05 0.00
−60 −20 0 20 40
−50 0 50

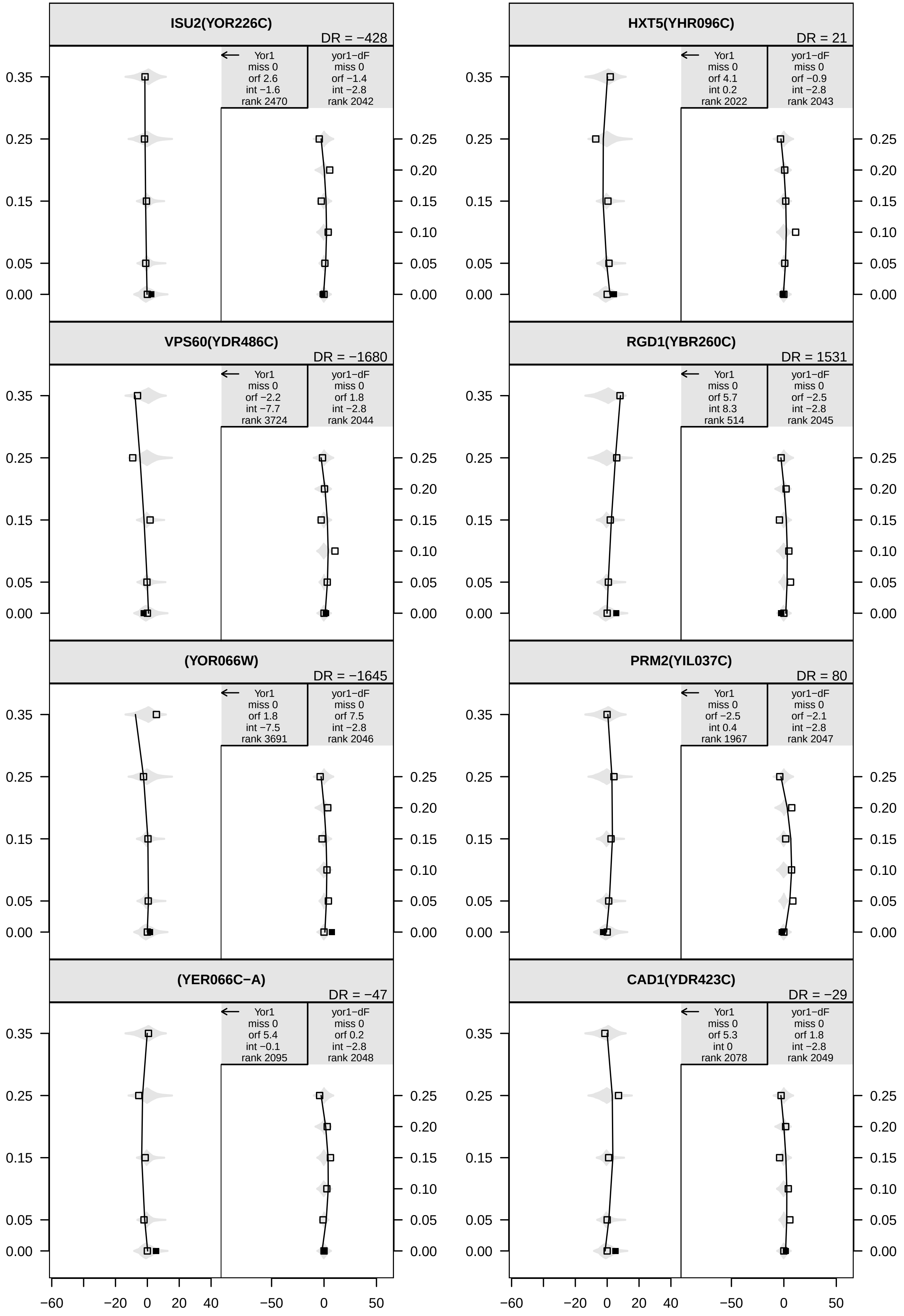
ISU2(YOR226C)
DR = −428
Yor1
miss 0
orf 2.6
int −1.6
rank 2470
yor1−dF
miss 0
orf −1.4
int −2.8
rank 2042
HXT5(YHR096C)
DR = 21
Yor1
miss 0
orf 4.1
int 0.2
rank 2022
yor1−dF
miss 0
orf −0.9
int −2.8
rank 2043
VPS60(YDR486C)
DR = −1680
Yor1
miss 0
orf −2.2
int −7.7
rank 3724
yor1−dF
miss 0
orf 1.8
int −2.8
rank 2044
RGD1(YBR260C)
DR = 1531
Yor1
miss 0
orf 5.7
int 8.3
rank 514
yor1−dF
miss 0
orf −2.5
int −2.8
rank 2045
(YOR066W)
DR = −1645
Yor1
miss 0
orf 1.8
int −7.5
rank 3691
yor1−dF
miss 0
orf 7.5
int −2.8
rank 2046
PRM2(YIL037C)
DR = 80
Yor1
miss 0
orf −2.5
int 0.4
rank 1967
yor1−dF
miss 0
orf −2.1
int −2.8
rank 2047
(YER066C−A)
DR = −47
Yor1
miss 0
orf 5.4
int −0.1
rank 2095
yor1−dF
miss 0
orf 0.2
int −2.8
rank 2048
CAD1(YDR423C)
DR = −29
Yor1
miss 0
orf 5.3
int 0
rank 2078
yor1−dF
miss 0
orf 1.8
int −2.8
rank 2049
0.35
0.25
0.20
0.15
0.10
0.05
0.00
−60
−20
0
20
40
−50
0
50

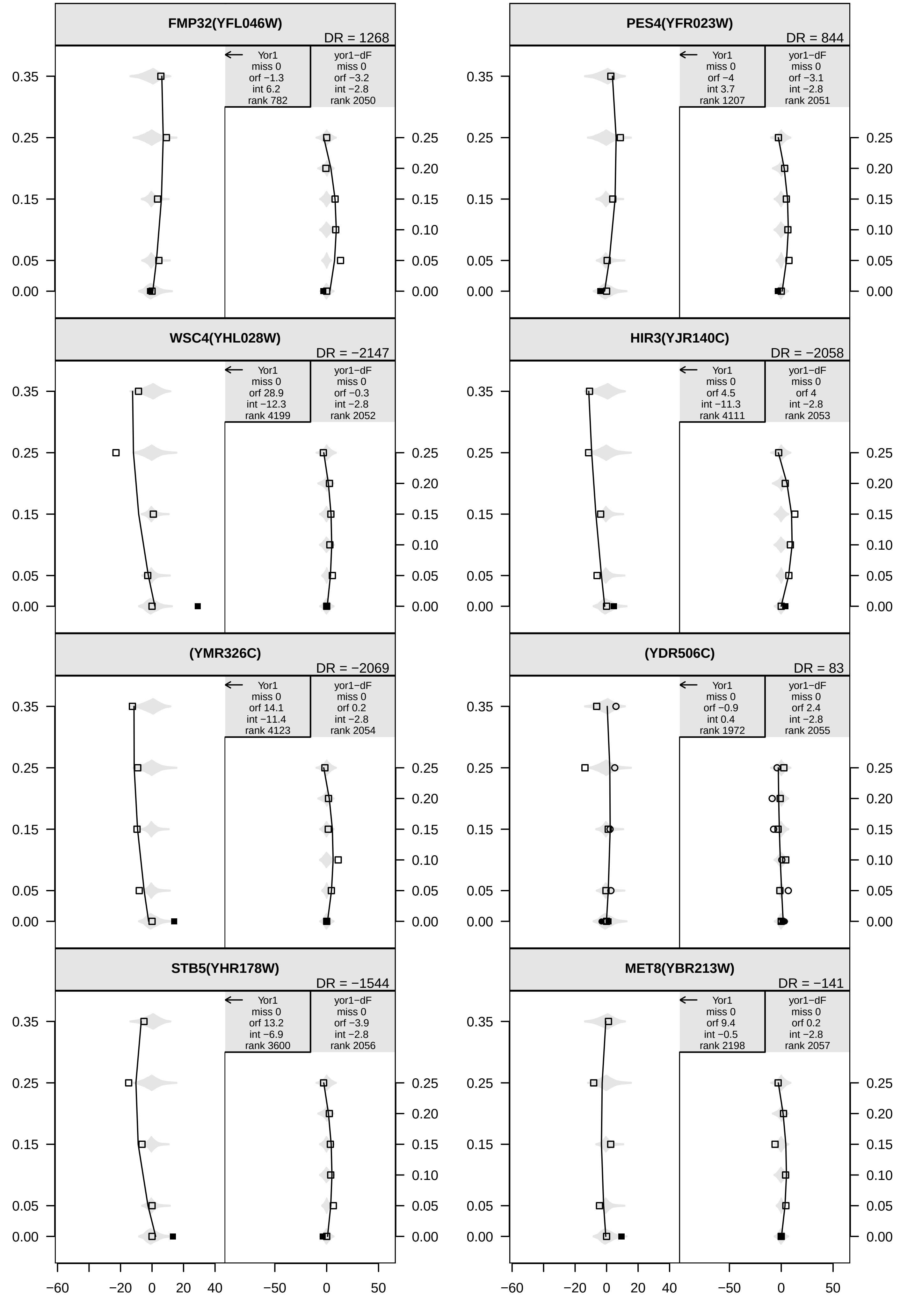
FMP32(YFL046W)
DR = 1268
Yor1 miss 0 orf −1.3 int 6.2 rank 782
yor1−dF miss 0 orf −3.2 int −2.8 rank 2050
PES4(YFR023W)
DR = 844
Yor1 miss 0 orf −4 int 3.7 rank 1207
yor1−dF miss 0 orf −3.1 int −2.8 rank 2051
WSC4(YHL028W)
DR = −2147
Yor1 miss 0 orf 28.9 int −12.3 rank 4199
yor1−dF miss 0 orf −0.3 int −2.8 rank 2052
HIR3(YJR140C)
DR = −2058
Yor1 miss 0 orf 4.5 int −11.3 rank 4111
yor1−dF miss 0 orf 4 int −2.8 rank 2053
(YMR326C)
DR = −2069
Yor1 miss 0 orf 14.1 int −11.4 rank 4123
yor1−dF miss 0 orf 0.2 int −2.8 rank 2054
(YDR506C)
DR = 83
Yor1 miss 0 orf −0.9 int 0.4 rank 1972
yor1−dF miss 0 orf 2.4 int −2.8 rank 2055
STB5(YHR178W)
DR = −1544
Yor1 miss 0 orf 13.2 int −6.9 rank 3600
yor1−dF miss 0 orf −3.9 int −2.8 rank 2056
MET8(YBR213W)
DR = −141
Yor1 miss 0 orf 9.4 int −0.5 rank 2198
yor1−dF miss 0 orf 0.2 int −2.8 rank 2057
0.35 0.25 0.20 0.15 0.10 0.05 0.00
−60 −20 0 20 40 −50 0 50

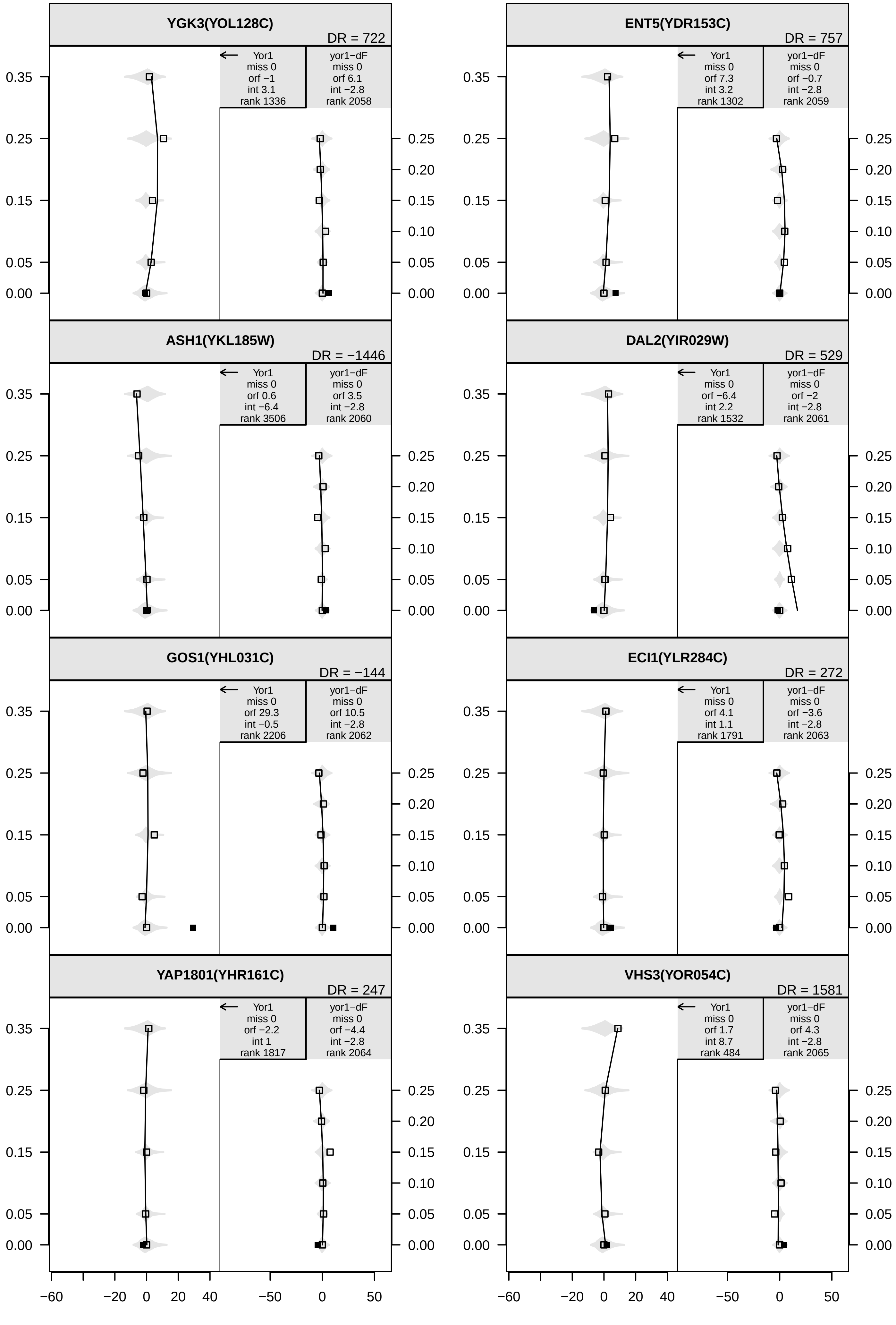
YGK3(YOL128C)
DR = 722
Yor1 miss 0 orf −1 int 3.1 rank 1336
yor1−dF miss 0 orf 6.1 int −2.8 rank 2058
ENT5(YDR153C)
DR = 757
Yor1 miss 0 orf 7.3 int 3.2 rank 1302
yor1−dF miss 0 orf −0.7 int −2.8 rank 2059
ASH1(YKL185W)
DR = −1446
Yor1 miss 0 orf 0.6 int −6.4 rank 3506
yor1−dF miss 0 orf 3.5 int −2.8 rank 2060
DAL2(YIR029W)
DR = 529
Yor1 miss 0 orf −6.4 int 2.2 rank 1532
yor1−dF miss 0 orf −2 int −2.8 rank 2061
GOS1(YHL031C)
DR = −144
Yor1 miss 0 orf 29.3 int −0.5 rank 2206
yor1−dF miss 0 orf 10.5 int −2.8 rank 2062
ECI1(YLR284C)
DR = 272
Yor1 miss 0 orf 4.1 int 1.1 rank 1791
yor1−dF miss 0 orf −3.6 int −2.8 rank 2063
YAP1801(YHR161C)
DR = 247
Yor1 miss 0 orf −2.2 int 1 rank 1817
yor1−dF miss 0 orf −4.4 int −2.8 rank 2064
VHS3(YOR054C)
DR = 1581
Yor1 miss 0 orf 1.7 int 8.7 rank 484
yor1−dF miss 0 orf 4.3 int −2.8 rank 2065
0.35
0.25
0.20
0.15
0.10
0.05
0.00
−60
−20
0
20
40
−50
0
50

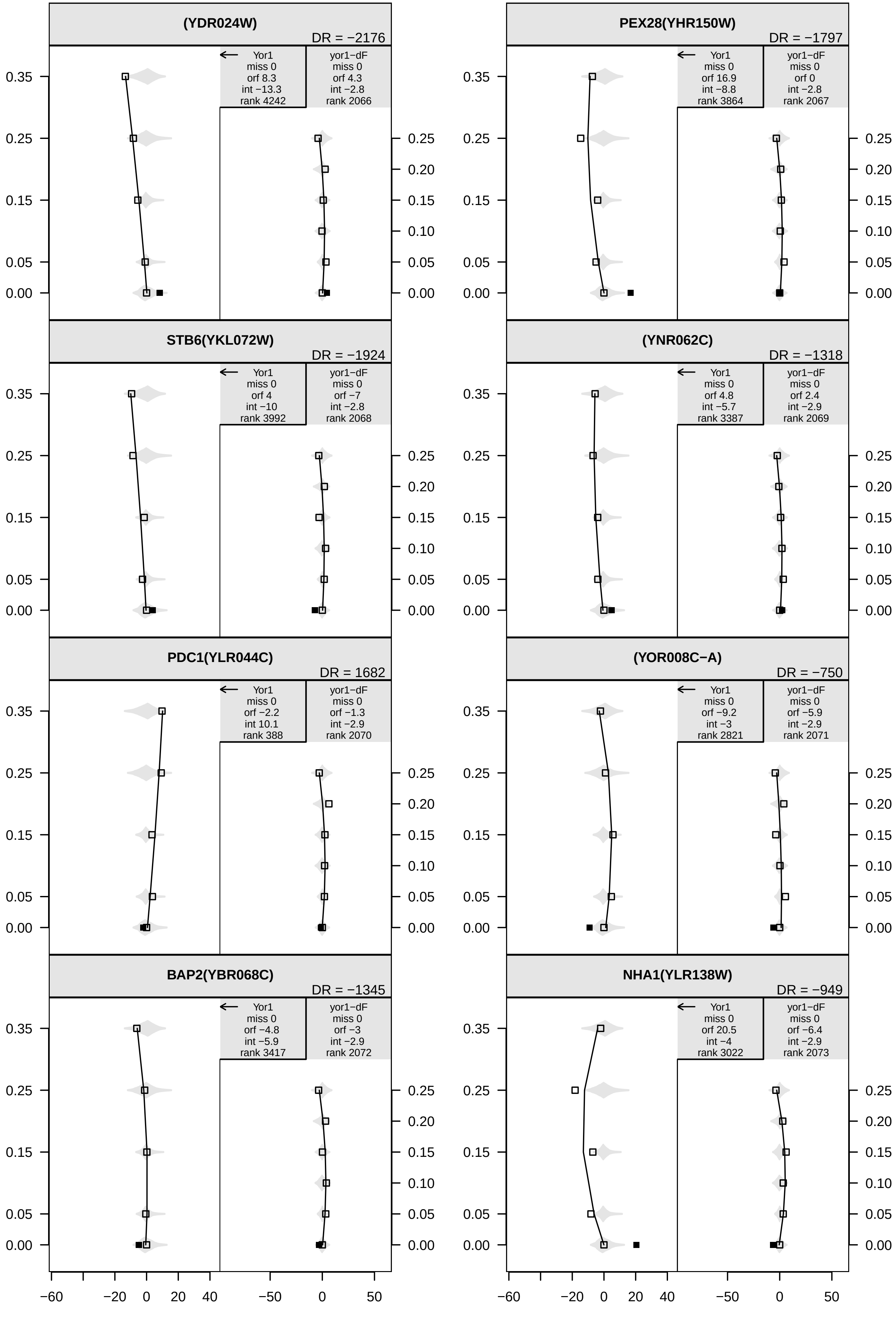
(YDR024W)
DR = −2176
Yor1
miss 0
orf 8.3
int −13.3
rank 4242
yor1−dF
miss 0
orf 4.3
int −2.8
rank 2066
PEX28(YHR150W)
DR = −1797
Yor1
miss 0
orf 16.9
int −8.8
rank 3864
yor1−dF
miss 0
orf 0
int −2.8
rank 2067
STB6(YKL072W)
DR = −1924
Yor1
miss 0
orf 4
int −10
rank 3992
yor1−dF
miss 0
orf −7
int −2.8
rank 2068
(YNR062C)
DR = −1318
Yor1
miss 0
orf 4.8
int −5.7
rank 3387
yor1−dF
miss 0
orf 2.4
int −2.9
rank 2069
PDC1(YLR044C)
DR = 1682
Yor1
miss 0
orf −2.2
int 10.1
rank 388
yor1−dF
miss 0
orf −1.3
int −2.9
rank 2070
(YOR008C−A)
DR = −750
Yor1
miss 0
orf −9.2
int −3
rank 2821
yor1−dF
miss 0
orf −5.9
int −2.9
rank 2071
BAP2(YBR068C)
DR = −1345
Yor1
miss 0
orf −4.8
int −5.9
rank 3417
yor1−dF
miss 0
orf −3
int −2.9
rank 2072
NHA1(YLR138W)
DR = −949
Yor1
miss 0
orf 20.5
int −4
rank 3022
yor1−dF
miss 0
orf −6.4
int −2.9
rank 2073
0.35
0.25
0.20
0.15
0.10
0.05
0.00
−60
−20
0
20
40
−50
0
50

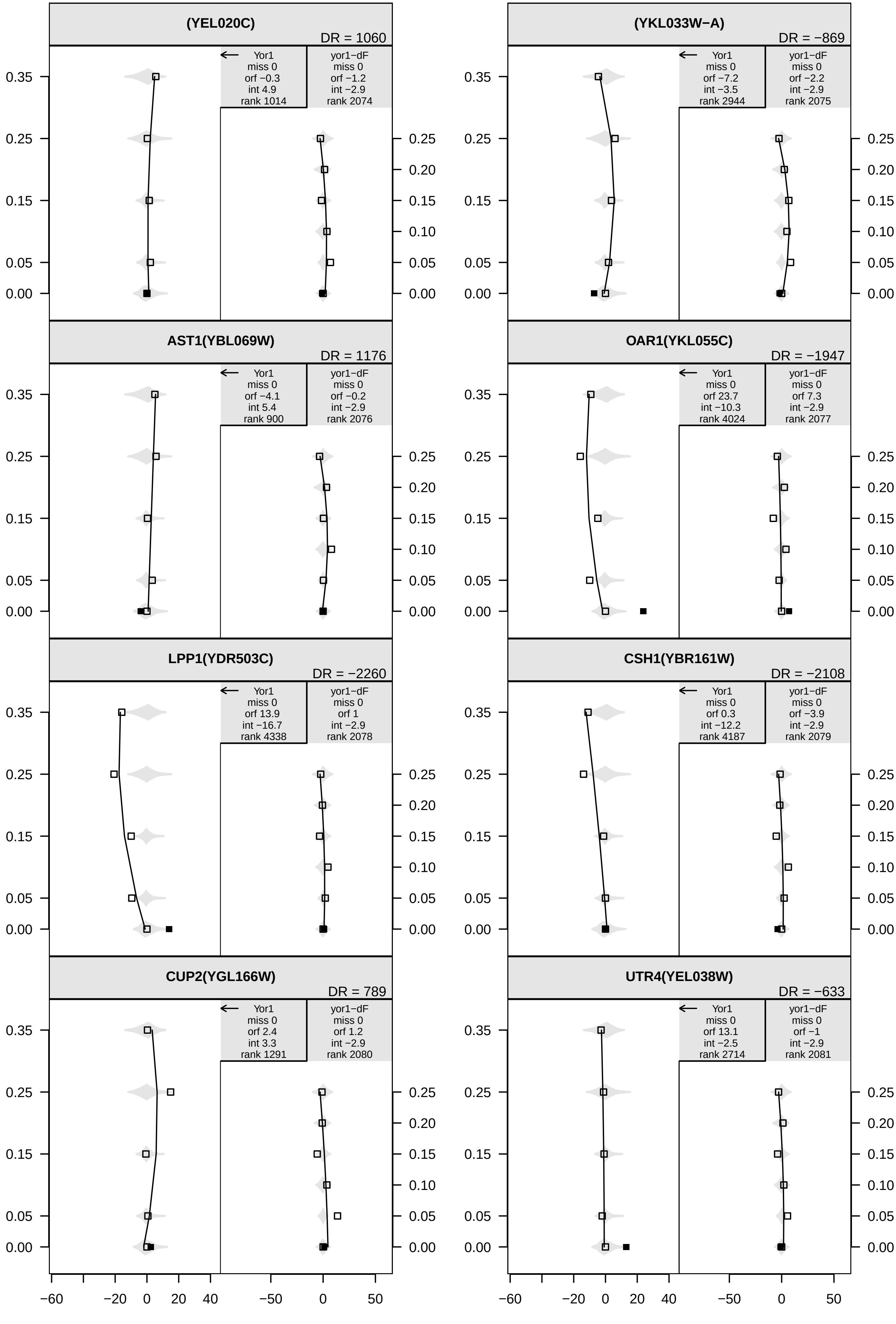
(YEL020C)
DR = 1060
Yor1 miss 0 orf −0.3 int 4.9 rank 1014
yor1−dF miss 0 orf −1.2 int −2.9 rank 2074
(YKL033W−A)
DR = −869
Yor1 miss 0 orf −7.2 int −3.5 rank 2944
yor1−dF miss 0 orf −2.2 int −2.9 rank 2075
AST1(YBL069W)
DR = 1176
Yor1 miss 0 orf −4.1 int 5.4 rank 900
yor1−dF miss 0 orf −0.2 int −2.9 rank 2076
OAR1(YKL055C)
DR = −1947
Yor1 miss 0 orf 23.7 int −10.3 rank 4024
yor1−dF miss 0 orf 7.3 int −2.9 rank 2077
LPP1(YDR503C)
DR = −2260
Yor1 miss 0 orf 13.9 int −16.7 rank 4338
yor1−dF miss 0 orf 1 int −2.9 rank 2078
CSH1(YBR161W)
DR = −2108
Yor1 miss 0 orf 0.3 int −12.2 rank 4187
yor1−dF miss 0 orf −3.9 int −2.9 rank 2079
CUP2(YGL166W)
DR = 789
Yor1 miss 0 orf 2.4 int 3.3 rank 1291
yor1−dF miss 0 orf 1.2 int −2.9 rank 2080
UTR4(YEL038W)
DR = −633
Yor1 miss 0 orf 13.1 int −2.5 rank 2714
yor1−dF miss 0 orf −1 int −2.9 rank 2081
0.00 0.05 0.15 0.25 0.35
0.00 0.05 0.10 0.15 0.20 0.25
−60 −20 0 20 40 −50 0 50

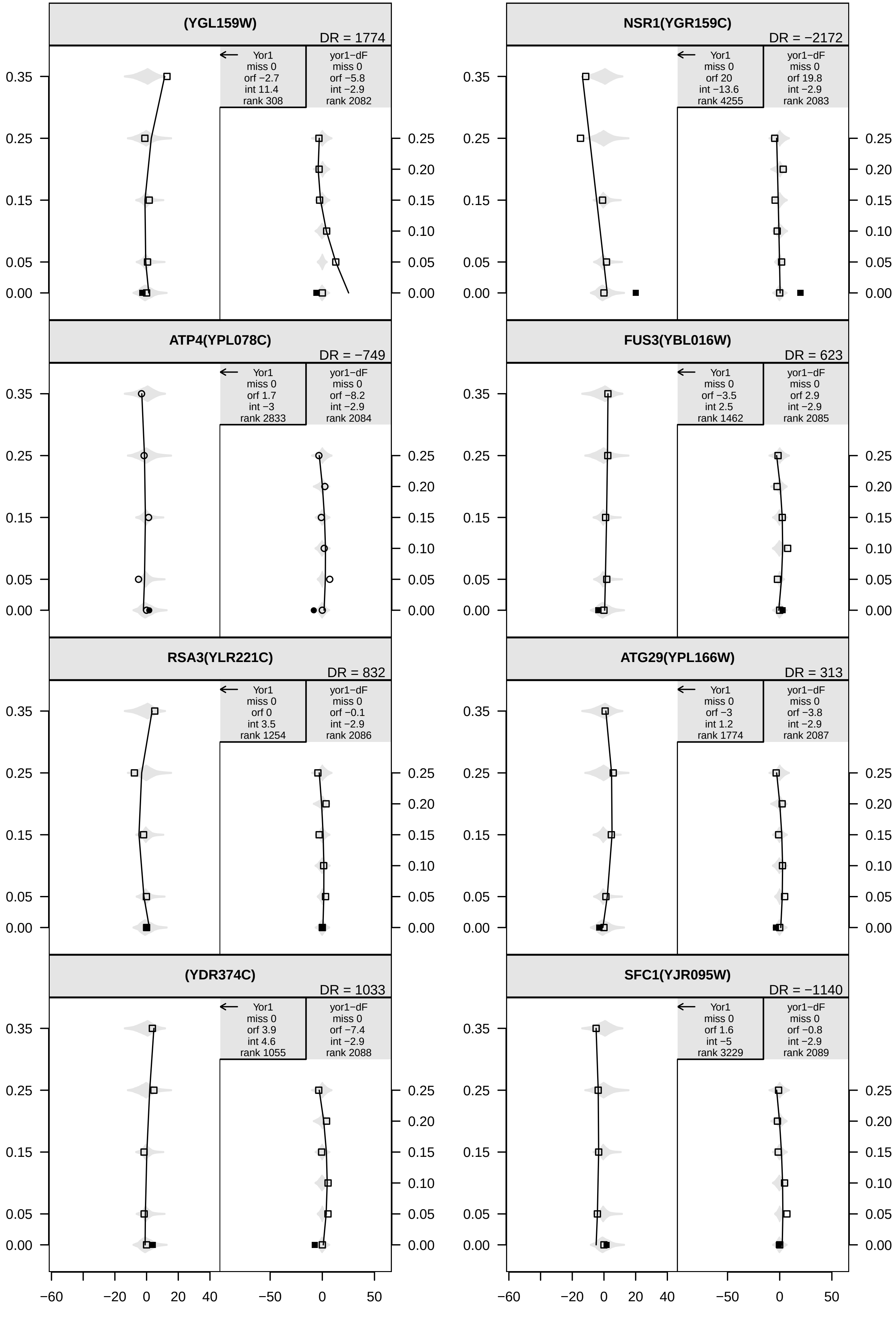
(YGL159W)
DR = 1774
Yor1 miss 0 orf −2.7 int 11.4 rank 308
yor1−dF miss 0 orf −5.8 int −2.9 rank 2082
NSR1(YGR159C)
DR = −2172
Yor1 miss 0 orf 20 int −13.6 rank 4255
yor1−dF miss 0 orf 19.8 int −2.9 rank 2083
ATP4(YPL078C)
DR = −749
Yor1 miss 0 orf 1.7 int −3 rank 2833
yor1−dF miss 0 orf −8.2 int −2.9 rank 2084
FUS3(YBL016W)
DR = 623
Yor1 miss 0 orf −3.5 int 2.5 rank 1462
yor1−dF miss 0 orf 2.9 int −2.9 rank 2085
RSA3(YLR221C)
DR = 832
Yor1 miss 0 orf 0 int 3.5 rank 1254
yor1−dF miss 0 orf −0.1 int −2.9 rank 2086
ATG29(YPL166W)
DR = 313
Yor1 miss 0 orf −3 int 1.2 rank 1774
yor1−dF miss 0 orf −3.8 int −2.9 rank 2087
(YDR374C)
DR = 1033
Yor1 miss 0 orf 3.9 int 4.6 rank 1055
yor1−dF miss 0 orf −7.4 int −2.9 rank 2088
SFC1(YJR095W)
DR = −1140
Yor1 miss 0 orf 1.6 int −5 rank 3229
yor1−dF miss 0 orf −0.8 int −2.9 rank 2089
