# Supplementary material for: A yeast phenomic model for the gene interaction network modulating CFTR-ΔF508 protein biogenesis
Source: Genome Med. 2012 Dec 27;4(12):103. doi: 10.1186/gm404 (PMC3906889; doi:10.1186/gm404)
Supplement: Additional File 3 — This file contains graphs of oligomycin response for deletion strains in background of wild-type YOR1 or yor1-ΔF, searchable by gene and ORF names. The graphs are ordered by descending interaction score for yor1-ΔF double mutants, grouped also by the number of high oligomycin concentrations at which no growth was observed (that is, from strongest deletion enhancer to strongest deletion suppressor). 'miss' indicates the number of high oligomycin concentrations at which no growth occurred; 'ORF' indicates the difference in L between the deletion mutant and its wild-type reference strain; 'int' is the interaction quantity at the highest oligomycin concentration observed; 'DR' indicates the difference between the ranks of the deletion mutant in each of the two screens to indicate differential interactions in the context of wild-type Yor1 vs. Yor1-ΔF. The filled symbols indicate the raw 'L' value at the zero-oligomycin concentration for the deletion mutant, by which all L values for the deletion mutants were adjusted to quantify the interaction. The gray diamonds represent the 95% central distribution of 768 replicates of the reference strain, from which the dose effect at each oligomycin concentration has been removed. [file gm404-S3.PDF]

b. Aditional File 3: This file contains graphs of oligomycin response for deletion strains in background of wild type *YOR1* or *yor1-ΔF*, searchable by gene and ORF names. The graphs are ordered by descending interaction score for *yor1-ΔF* double mutants, grouped also by the number of high oligomycin concentrations at which no growth was observed (i.e., from strongest deletion enhancer to strongest deletion suppressor). 'miss' indicates the number of high oligomycin concentrations at which no growth occurred; 'ORF' indicates the difference in L between the deletion mutant and its wild type reference strain; 'int' is the interaction quantity at the highest oligomycin concentration observed; 'DR' indicates the difference between the ranks of the deletion mutant in each of the two screens to indicate differential interactions in the context of wild type Yor1 vs. Yor1-ΔF. The filled symbols indicate the raw 'L' value at the zero-oligomycin concentration for the deletion mutant, by which all L values for the deletion mutants were adjusted to quantify the interaction. The gray diamonds represent the 95% central distribution of 768 replicates of the reference strain, from which the dose effect at each oligomycin concentration has been removed.

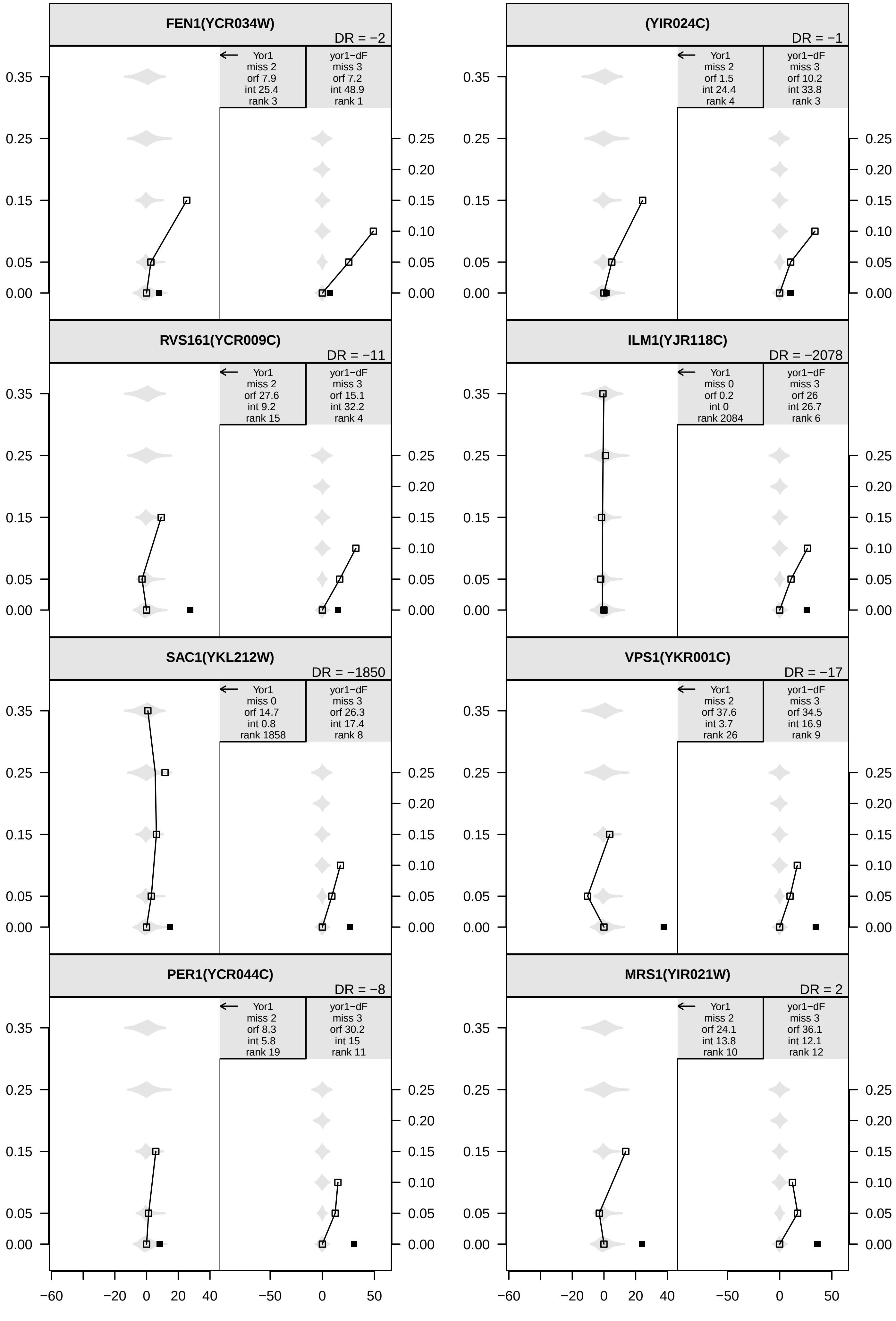
FEN1(YCR034W)
DR = −2
Yor1
miss 2
orf 7.9
int 25.4
rank 3
yor1−dF
miss 3
orf 7.2
int 48.9
rank 1
(YIR024C)
DR = −1
Yor1
miss 2
orf 1.5
int 24.4
rank 4
yor1−dF
miss 3
orf 10.2
int 33.8
rank 3
RVS161(YCR009C)
DR = −11
Yor1
miss 2
orf 27.6
int 9.2
rank 15
yor1−dF
miss 3
orf 15.1
int 32.2
rank 4
ILM1(YJR118C)
DR = −2078
Yor1
miss 0
orf 0.2
int 0
rank 2084
yor1−dF
miss 3
orf 26
int 26.7
rank 6
SAC1(YKL212W)
DR = −1850
Yor1
miss 0
orf 14.7
int 0.8
rank 1858
yor1−dF
miss 3
orf 26.3
int 17.4
rank 8
VPS1(YKR001C)
DR = −17
Yor1
miss 2
orf 37.6
int 3.7
rank 26
yor1−dF
miss 3
orf 34.5
int 16.9
rank 9
PER1(YCR044C)
DR = −8
Yor1
miss 2
orf 8.3
int 5.8
rank 19
yor1−dF
miss 3
orf 30.2
int 15
rank 11
MRS1(YIR021W)
DR = 2
Yor1
miss 2
orf 24.1
int 13.8
rank 10
yor1−dF
miss 3
orf 36.1
int 12.1
rank 12
0.35
0.25
0.20
0.15
0.10
0.05
0.00
−60
−20
0
20
40
−50
0
50

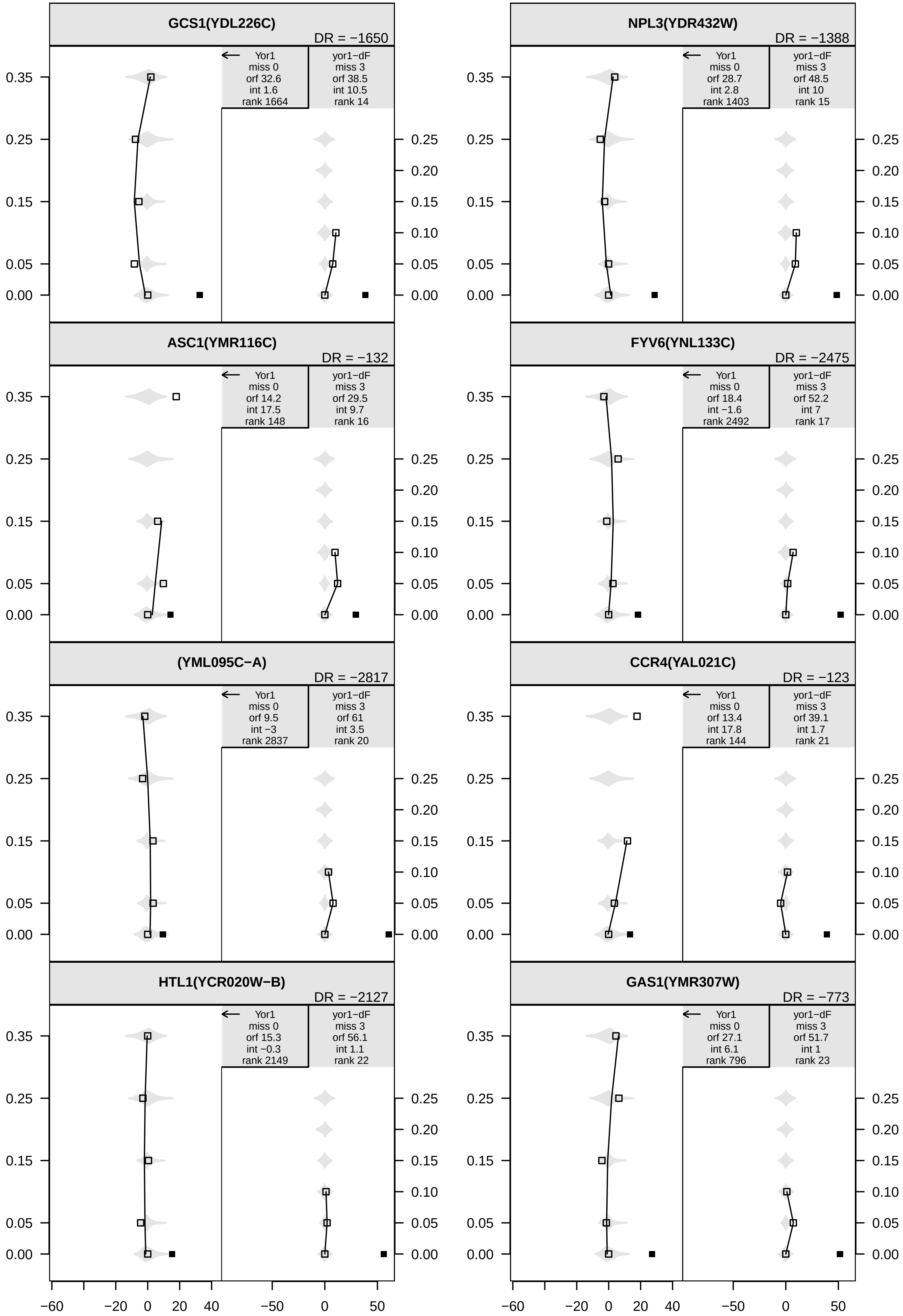

GCS1(YDL226C)
DR = −1650
Yor1 miss 0 orf 32.6 int 1.6 rank 1664
yor1−dF miss 3 orf 38.5 int 10.5 rank 14
NPL3(YDR432W)
DR = −1388
Yor1 miss 0 orf 28.7 int 2.8 rank 1403
yor1−dF miss 3 orf 48.5 int 10 rank 15
ASC1(YMR116C)
DR = −132
Yor1 miss 0 orf 14.2 int 17.5 rank 148
yor1−dF miss 3 orf 29.5 int 9.7 rank 16
FYV6(YNL133C)
DR = −2475
Yor1 miss 0 orf 18.4 int −1.6 rank 2492
yor1−dF miss 3 orf 52.2 int 7 rank 17
(YML095C−A)
DR = −2817
Yor1 miss 0 orf 9.5 int −3 rank 2837
yor1−dF miss 3 orf 61 int 3.5 rank 20
CCR4(YAL021C)
DR = −123
Yor1 miss 0 orf 13.4 int 17.8 rank 144
yor1−dF miss 3 orf 39.1 int 1.7 rank 21
HTL1(YCR020W−B)
DR = −2127
Yor1 miss 0 orf 15.3 int −0.3 rank 2149
yor1−dF miss 3 orf 56.1 int 1.1 rank 22
GAS1(YMR307W)
DR = −773
Yor1 miss 0 orf 27.1 int 6.1 rank 796
yor1−dF miss 3 orf 51.7 int 1 rank 23

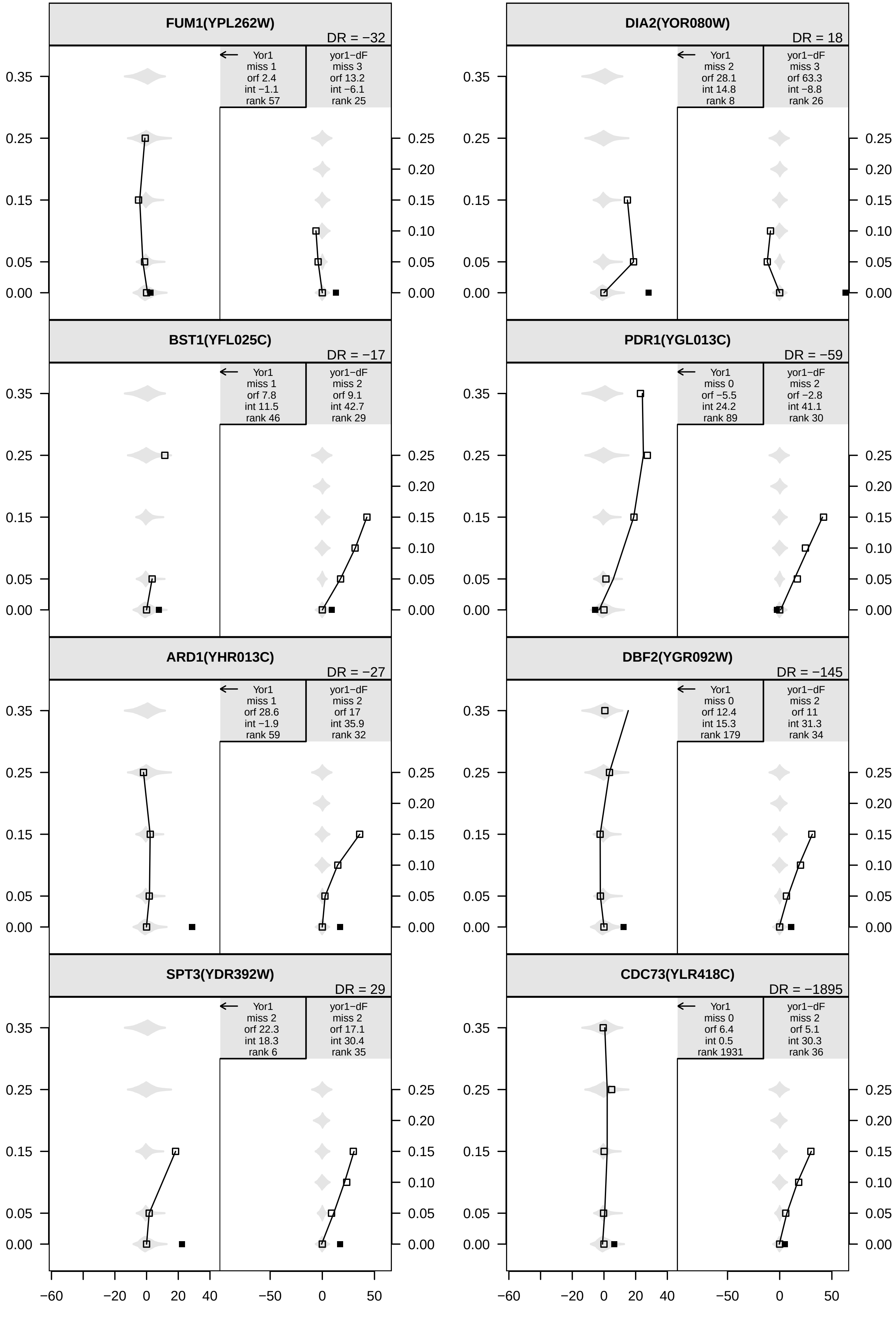
FUM1(YPL262W)
DR = −32
Yor1 miss 1 orf 2.4 int −1.1 rank 57
yor1−dF miss 3 orf 13.2 int −6.1 rank 25
DIA2(YOR080W)
DR = 18
Yor1 miss 2 orf 28.1 int 14.8 rank 8
yor1−dF miss 3 orf 63.3 int −8.8 rank 26
BST1(YFL025C)
DR = −17
Yor1 miss 1 orf 7.8 int 11.5 rank 46
yor1−dF miss 2 orf 9.1 int 42.7 rank 29
PDR1(YGL013C)
DR = −59
Yor1 miss 0 orf −5.5 int 24.2 rank 89
yor1−dF miss 2 orf −2.8 int 41.1 rank 30
ARD1(YHR013C)
DR = −27
Yor1 miss 1 orf 28.6 int −1.9 rank 59
yor1−dF miss 2 orf 17 int 35.9 rank 32
DBF2(YGR092W)
DR = −145
Yor1 miss 0 orf 12.4 int 15.3 rank 179
yor1−dF miss 2 orf 11 int 31.3 rank 34
SPT3(YDR392W)
DR = 29
Yor1 miss 2 orf 22.3 int 18.3 rank 6
yor1−dF miss 2 orf 17.1 int 30.4 rank 35
CDC73(YLR418C)
DR = −1895
Yor1 miss 0 orf 6.4 int 0.5 rank 1931
yor1−dF miss 2 orf 5.1 int 30.3 rank 36
0.00 0.05 0.10 0.15 0.20 0.25 0.35
−60 −20 0 20 40 −50 0 50

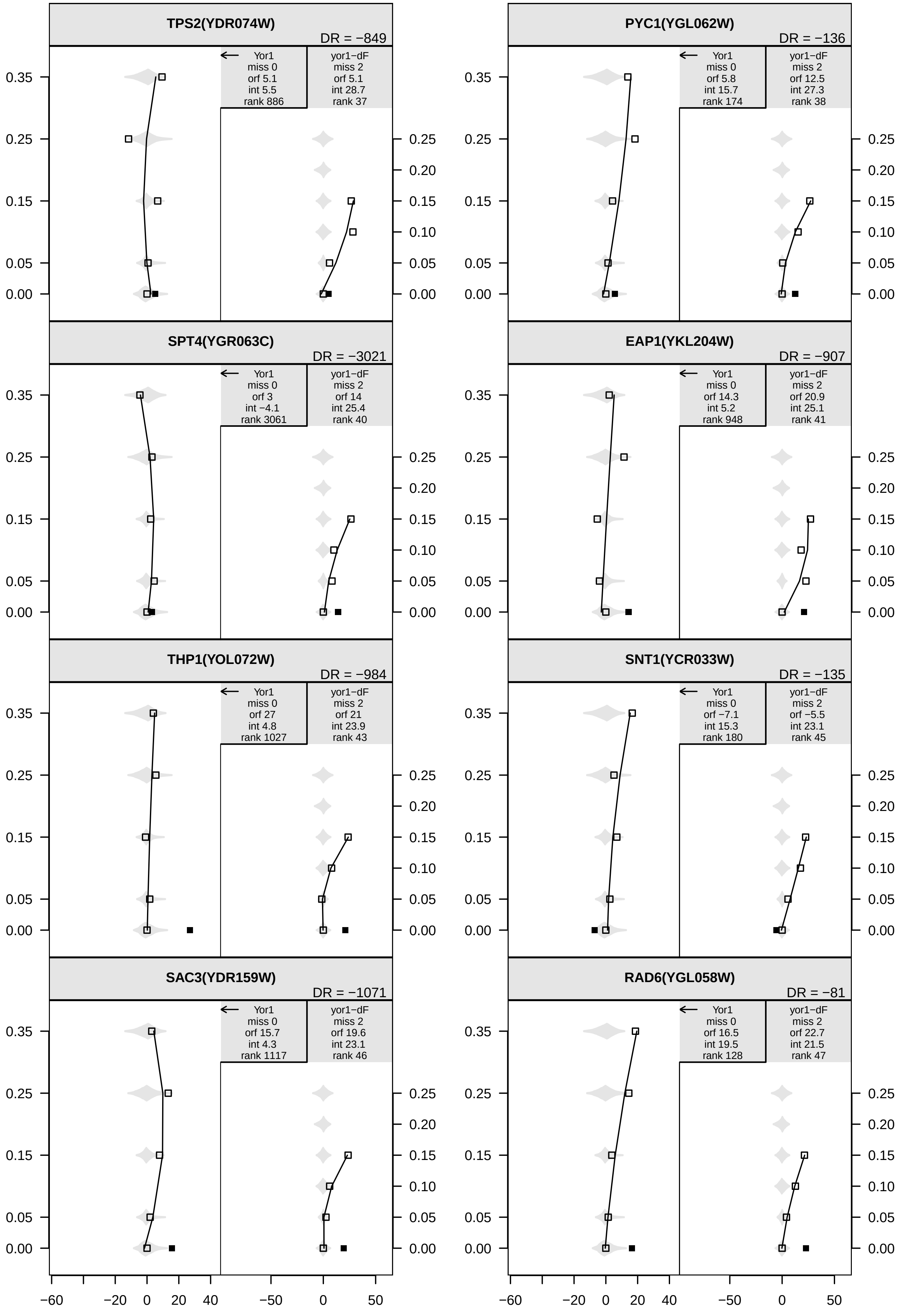

TPS2(YDR074W)
DR = −849
Yor1 miss 0 orf 5.1 int 5.5 rank 886
yor1−dF miss 2 orf 5.1 int 28.7 rank 37
PYC1(YGL062W)
DR = −136
Yor1 miss 0 orf 5.8 int 15.7 rank 174
yor1−dF miss 2 orf 12.5 int 27.3 rank 38
SPT4(YGR063C)
DR = −3021
Yor1 miss 0 orf 3 int −4.1 rank 3061
yor1−dF miss 2 orf 14 int 25.4 rank 40
EAP1(YKL204W)
DR = −907
Yor1 miss 0 orf 14.3 int 5.2 rank 948
yor1−dF miss 2 orf 20.9 int 25.1 rank 41
THP1(YOL072W)
DR = −984
Yor1 miss 0 orf 27 int 4.8 rank 1027
yor1−dF miss 2 orf 21 int 23.9 rank 43
SNT1(YCR033W)
DR = −135
Yor1 miss 0 orf −7.1 int 15.3 rank 180
yor1−dF miss 2 orf −5.5 int 23.1 rank 45
SAC3(YDR159W)
DR = −1071
Yor1 miss 0 orf 15.7 int 4.3 rank 1117
yor1−dF miss 2 orf 19.6 int 23.1 rank 46
RAD6(YGL058W)
DR = −81
Yor1 miss 0 orf 16.5 int 19.5 rank 128
yor1−dF miss 2 orf 22.7 int 21.5 rank 47
0.35 0.25 0.15 0.05 0.00
0.25 0.20 0.15 0.10 0.05 0.00
−60 −20 0 20 40 −50 0 50

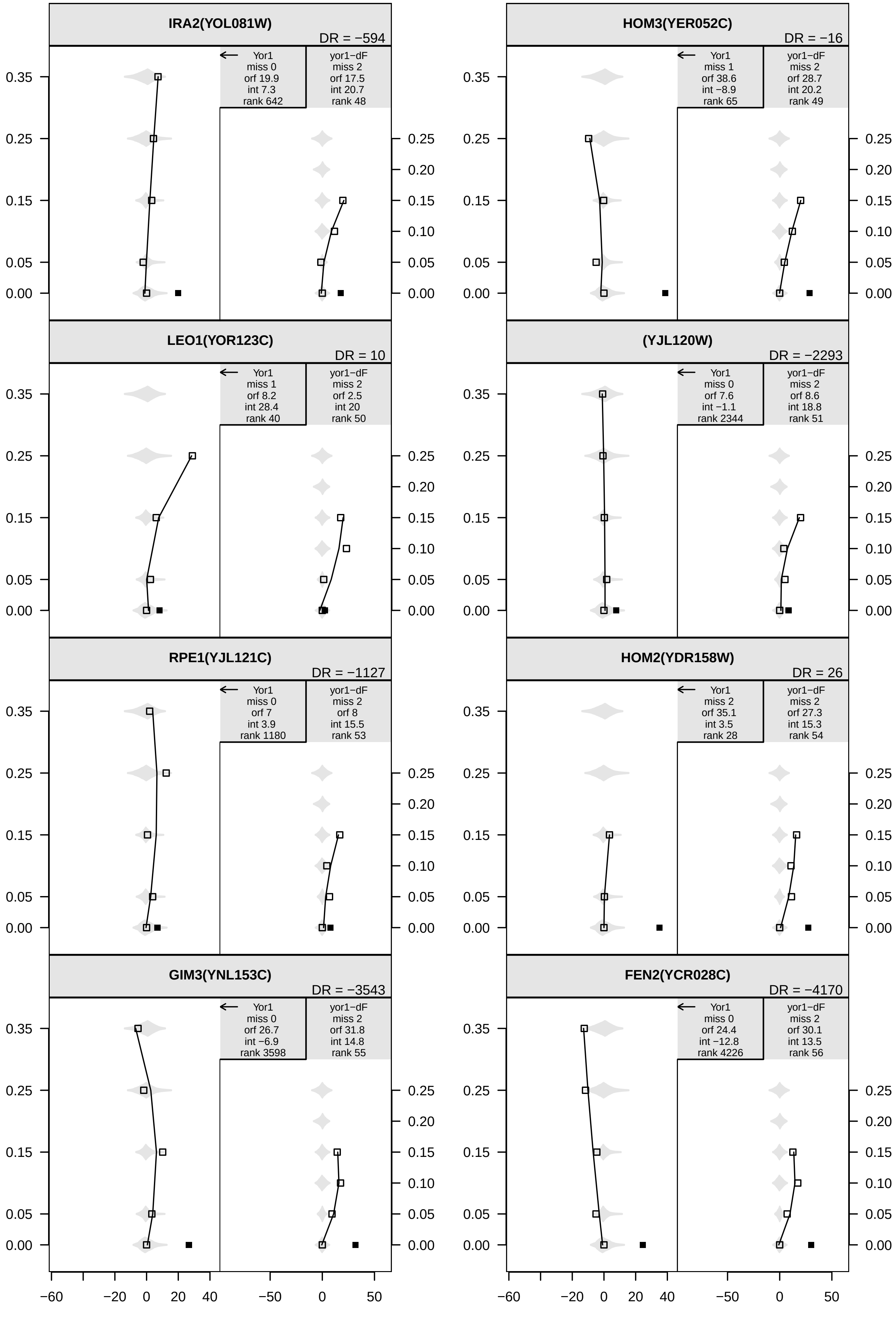

IRA2(YOL081W)
DR = −594
Yor1
miss 0
orf 19.9
int 7.3
rank 642
yor1−dF
miss 2
orf 17.5
int 20.7
rank 48
HOM3(YER052C)
DR = −16
Yor1
miss 1
orf 38.6
int −8.9
rank 65
yor1−dF
miss 2
orf 28.7
int 20.2
rank 49
LEO1(YOR123C)
DR = 10
Yor1
miss 1
orf 8.2
int 28.4
rank 40
yor1−dF
miss 2
orf 2.5
int 20
rank 50
(YJL120W)
DR = −2293
Yor1
miss 0
orf 7.6
int −1.1
rank 2344
yor1−dF
miss 2
orf 8.6
int 18.8
rank 51
RPE1(YJL121C)
DR = −1127
Yor1
miss 0
orf 7
int 3.9
rank 1180
yor1−dF
miss 2
orf 8
int 15.5
rank 53
HOM2(YDR158W)
DR = 26
Yor1
miss 2
orf 35.1
int 3.5
rank 28
yor1−dF
miss 2
orf 27.3
int 15.3
rank 54
GIM3(YNL153C)
DR = −3543
Yor1
miss 0
orf 26.7
int −6.9
rank 3598
yor1−dF
miss 2
orf 31.8
int 14.8
rank 55
FEN2(YCR028C)
DR = −4170
Yor1
miss 0
orf 24.4
int −12.8
rank 4226
yor1−dF
miss 2
orf 30.1
int 13.5
rank 56
0.35
0.25
0.20
0.15
0.10
0.05
0.00
−60
−20
0
20
40
−50
0
50

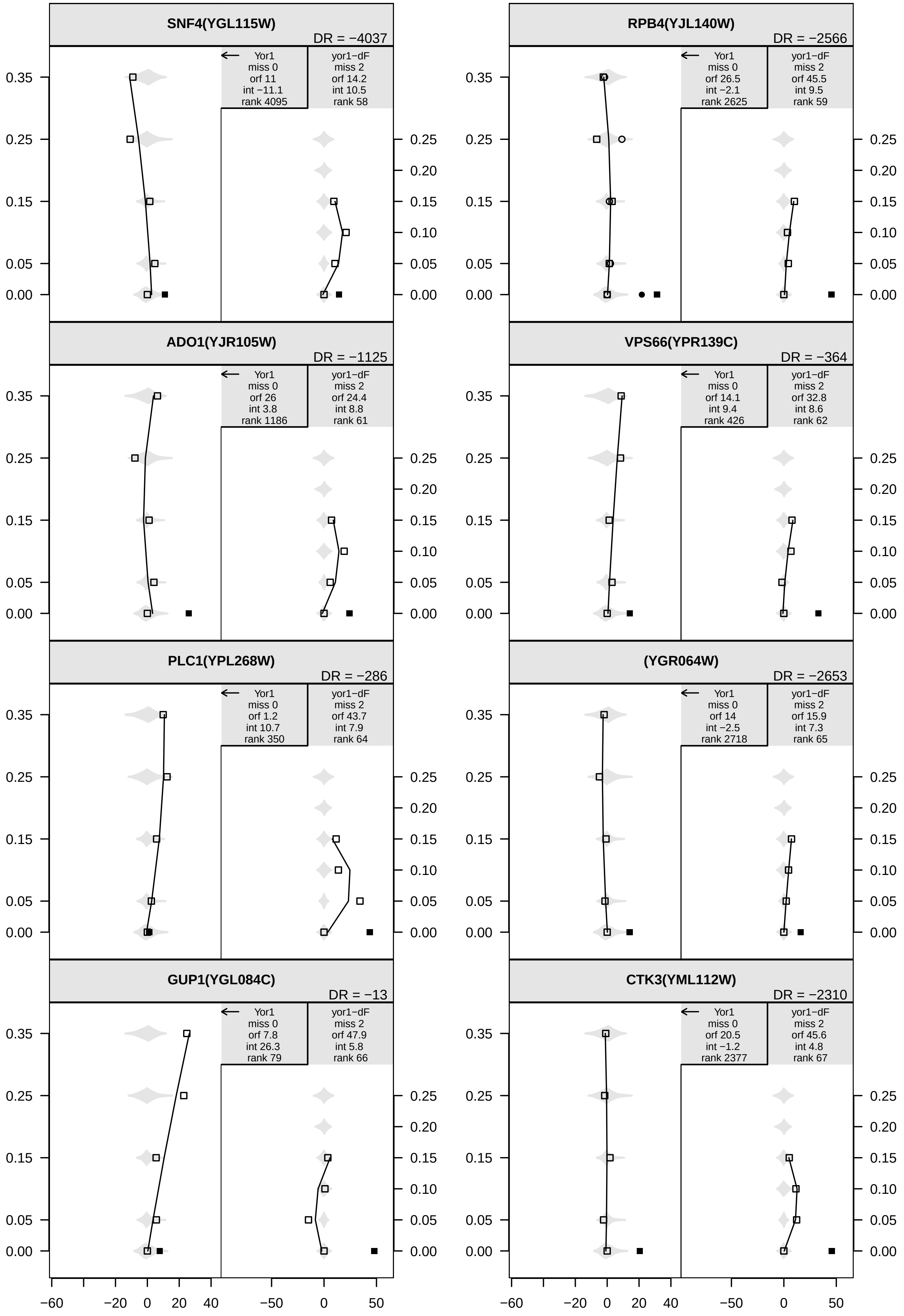
SNF4(YGL115W)
DR = −4037
Yor1
miss 0
orf 11
int −11.1
rank 4095
yor1−dF
miss 2
orf 14.2
int 10.5
rank 58
RPB4(YJL140W)
DR = −2566
Yor1
miss 0
orf 26.5
int −2.1
rank 2625
yor1−dF
miss 2
orf 45.5
int 9.5
rank 59
ADO1(YJR105W)
DR = −1125
Yor1
miss 0
orf 26
int 3.8
rank 1186
yor1−dF
miss 2
orf 24.4
int 8.8
rank 61
VPS66(YPR139C)
DR = −364
Yor1
miss 0
orf 14.1
int 9.4
rank 426
yor1−dF
miss 2
orf 32.8
int 8.6
rank 62
PLC1(YPL268W)
DR = −286
Yor1
miss 0
orf 1.2
int 10.7
rank 350
yor1−dF
miss 2
orf 43.7
int 7.9
rank 64
(YGR064W)
DR = −2653
Yor1
miss 0
orf 14
int −2.5
rank 2718
yor1−dF
miss 2
orf 15.9
int 7.3
rank 65
GUP1(YGL084C)
DR = −13
Yor1
miss 0
orf 7.8
int 26.3
rank 79
yor1−dF
miss 2
orf 47.9
int 5.8
rank 66
CTK3(YML112W)
DR = −2310
Yor1
miss 0
orf 20.5
int −1.2
rank 2377
yor1−dF
miss 2
orf 45.6
int 4.8
rank 67
0.35
0.25
0.20
0.15
0.10
0.05
0.00
−60
−20
0
20
40
−50
0
50

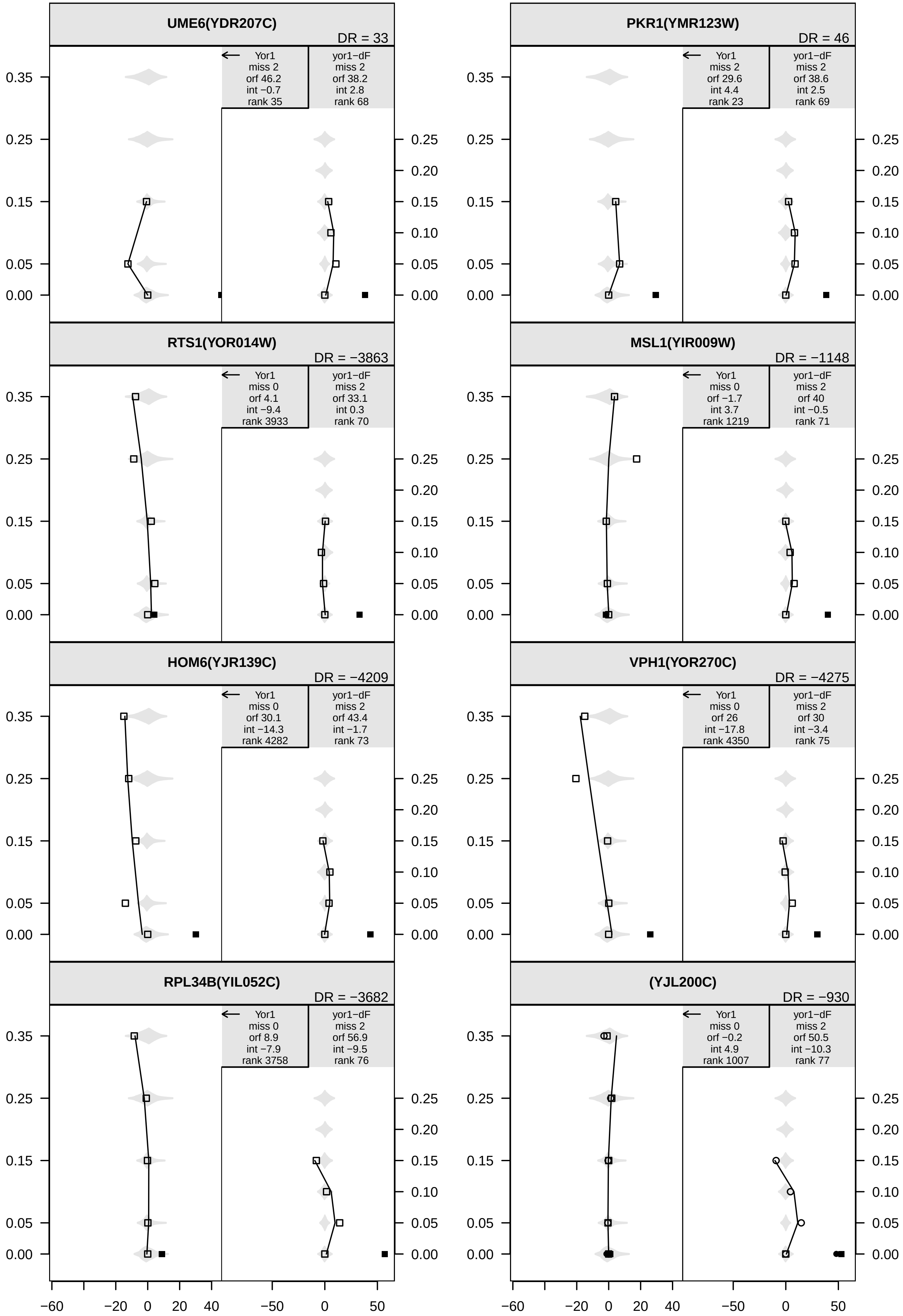

UME6(YDR207C)
DR = 33
Yor1 miss 2 orf 46.2 int −0.7 rank 35
yor1−dF miss 2 orf 38.2 int 2.8 rank 68
PKR1(YMR123W)
DR = 46
Yor1 miss 2 orf 29.6 int 4.4 rank 23
yor1−dF miss 2 orf 38.6 int 2.5 rank 69
RTS1(YOR014W)
DR = −3863
Yor1 miss 0 orf 4.1 int −9.4 rank 3933
yor1−dF miss 2 orf 33.1 int 0.3 rank 70
MSL1(YIR009W)
DR = −1148
Yor1 miss 0 orf −1.7 int 3.7 rank 1219
yor1−dF miss 2 orf 40 int −0.5 rank 71
HOM6(YJR139C)
DR = −4209
Yor1 miss 0 orf 30.1 int −14.3 rank 4282
yor1−dF miss 2 orf 43.4 int −1.7 rank 73
VPH1(YOR270C)
DR = −4275
Yor1 miss 0 orf 26 int −17.8 rank 4350
yor1−dF miss 2 orf 30 int −3.4 rank 75
RPL34B(YIL052C)
DR = −3682
Yor1 miss 0 orf 8.9 int −7.9 rank 3758
yor1−dF miss 2 orf 56.9 int −9.5 rank 76
(YJL200C)
DR = −930
Yor1 miss 0 orf −0.2 int 4.9 rank 1007
yor1−dF miss 2 orf 50.5 int −10.3 rank 77

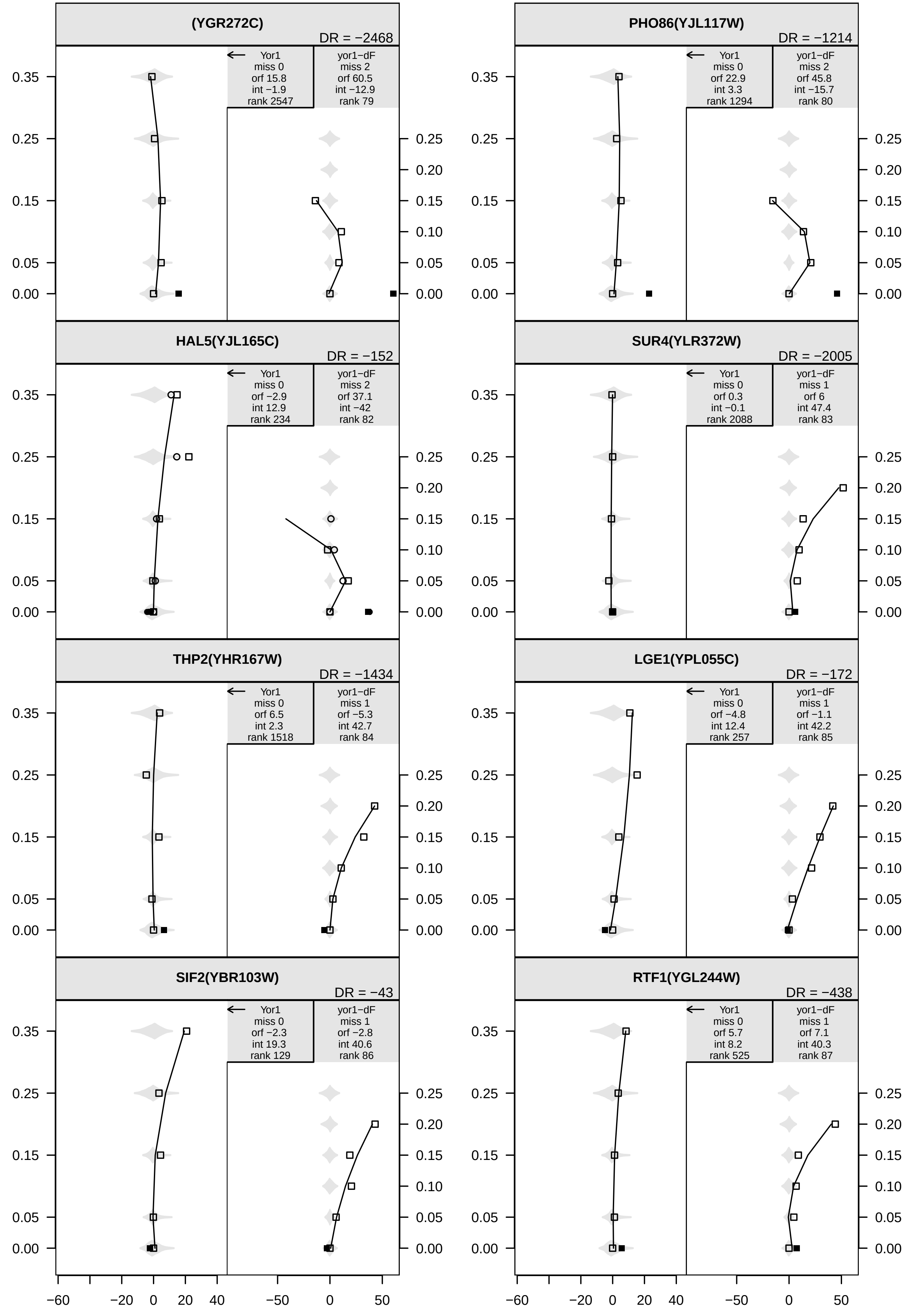
(YGR272C)
DR = −2468
Yor1 miss 0 orf 15.8 int −1.9 rank 2547
yor1−dF miss 2 orf 60.5 int −12.9 rank 79
PHO86(YJL117W)
DR = −1214
Yor1 miss 0 orf 22.9 int 3.3 rank 1294
yor1−dF miss 2 orf 45.8 int −15.7 rank 80
HAL5(YJL165C)
DR = −152
Yor1 miss 0 orf −2.9 int 12.9 rank 234
yor1−dF miss 2 orf 37.1 int −42 rank 82
SUR4(YLR372W)
DR = −2005
Yor1 miss 0 orf 0.3 int −0.1 rank 2088
yor1−dF miss 1 orf 6 int 47.4 rank 83
THP2(YHR167W)
DR = −1434
Yor1 miss 0 orf 6.5 int 2.3 rank 1518
yor1−dF miss 1 orf −5.3 int 42.7 rank 84
LGE1(YPL055C)
DR = −172
Yor1 miss 0 orf −4.8 int 12.4 rank 257
yor1−dF miss 1 orf −1.1 int 42.2 rank 85
SIF2(YBR103W)
DR = −43
Yor1 miss 0 orf −2.3 int 19.3 rank 129
yor1−dF miss 1 orf −2.8 int 40.6 rank 86
RTF1(YGL244W)
DR = −438
Yor1 miss 0 orf 5.7 int 8.2 rank 525
yor1−dF miss 1 orf 7.1 int 40.3 rank 87
0.00 0.05 0.10 0.15 0.20 0.25 0.35
−60 −20 0 20 40
−50 0 50

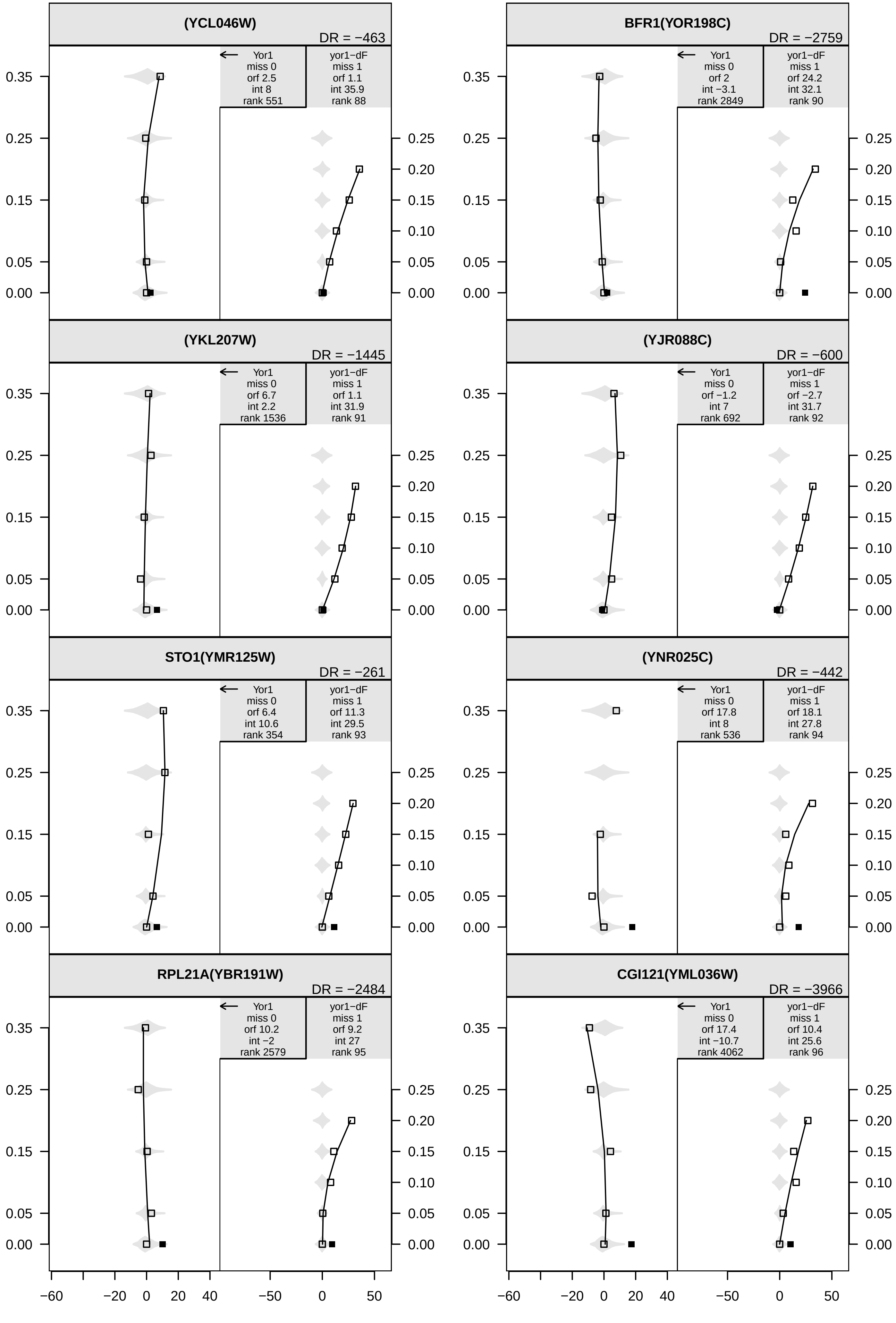
(YCL046W)
DR = −463
Yor1
miss 0
orf 2.5
int 8
rank 551
yor1−dF
miss 1
orf 1.1
int 35.9
rank 88
BFR1(YOR198C)
DR = −2759
Yor1
miss 0
orf 2
int −3.1
rank 2849
yor1−dF
miss 1
orf 24.2
int 32.1
rank 90
(YKL207W)
DR = −1445
Yor1
miss 0
orf 6.7
int 2.2
rank 1536
yor1−dF
miss 1
orf 1.1
int 31.9
rank 91
(YJR088C)
DR = −600
Yor1
miss 0
orf −1.2
int 7
rank 692
yor1−dF
miss 1
orf −2.7
int 31.7
rank 92
STO1(YMR125W)
DR = −261
Yor1
miss 0
orf 6.4
int 10.6
rank 354
yor1−dF
miss 1
orf 11.3
int 29.5
rank 93
(YNR025C)
DR = −442
Yor1
miss 0
orf 17.8
int 8
rank 536
yor1−dF
miss 1
orf 18.1
int 27.8
rank 94
RPL21A(YBR191W)
DR = −2484
Yor1
miss 0
orf 10.2
int −2
rank 2579
yor1−dF
miss 1
orf 9.2
int 27
rank 95
CGI121(YML036W)
DR = −3966
Yor1
miss 0
orf 17.4
int −10.7
rank 4062
yor1−dF
miss 1
orf 10.4
int 25.6
rank 96
0.35
0.25
0.20
0.15
0.10
0.05
0.00
−60
−20
0
20
40
−50
0
50

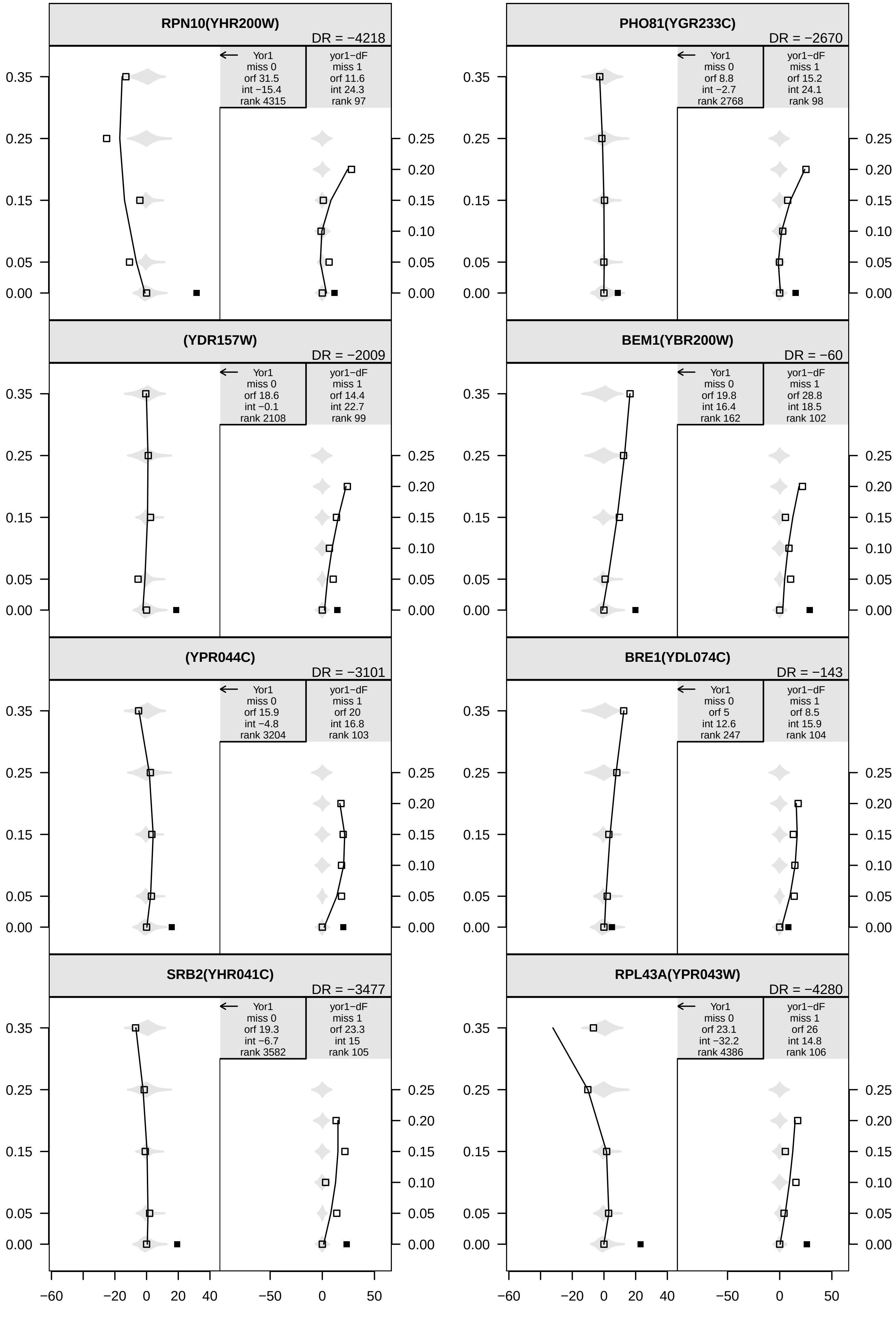
RPN10(YHR200W)
DR = −4218
Yor1 miss 0 orf 31.5 int −15.4 rank 4315
yor1−dF miss 1 orf 11.6 int 24.3 rank 97
PHO81(YGR233C)
DR = −2670
Yor1 miss 0 orf 8.8 int −2.7 rank 2768
yor1−dF miss 1 orf 15.2 int 24.1 rank 98
(YDR157W)
DR = −2009
Yor1 miss 0 orf 18.6 int −0.1 rank 2108
yor1−dF miss 1 orf 14.4 int 22.7 rank 99
BEM1(YBR200W)
DR = −60
Yor1 miss 0 orf 19.8 int 16.4 rank 162
yor1−dF miss 1 orf 28.8 int 18.5 rank 102
(YPR044C)
DR = −3101
Yor1 miss 0 orf 15.9 int −4.8 rank 3204
yor1−dF miss 1 orf 20 int 16.8 rank 103
BRE1(YDL074C)
DR = −143
Yor1 miss 0 orf 5 int 12.6 rank 247
yor1−dF miss 1 orf 8.5 int 15.9 rank 104
SRB2(YHR041C)
DR = −3477
Yor1 miss 0 orf 19.3 int −6.7 rank 3582
yor1−dF miss 1 orf 23.3 int 15 rank 105
RPL43A(YPR043W)
DR = −4280
Yor1 miss 0 orf 23.1 int −32.2 rank 4386
yor1−dF miss 1 orf 26 int 14.8 rank 106
0.35 0.25 0.15 0.05 0.00
0.25 0.20 0.15 0.10 0.05 0.00
−60 −20 0 20 40
−50 0 50

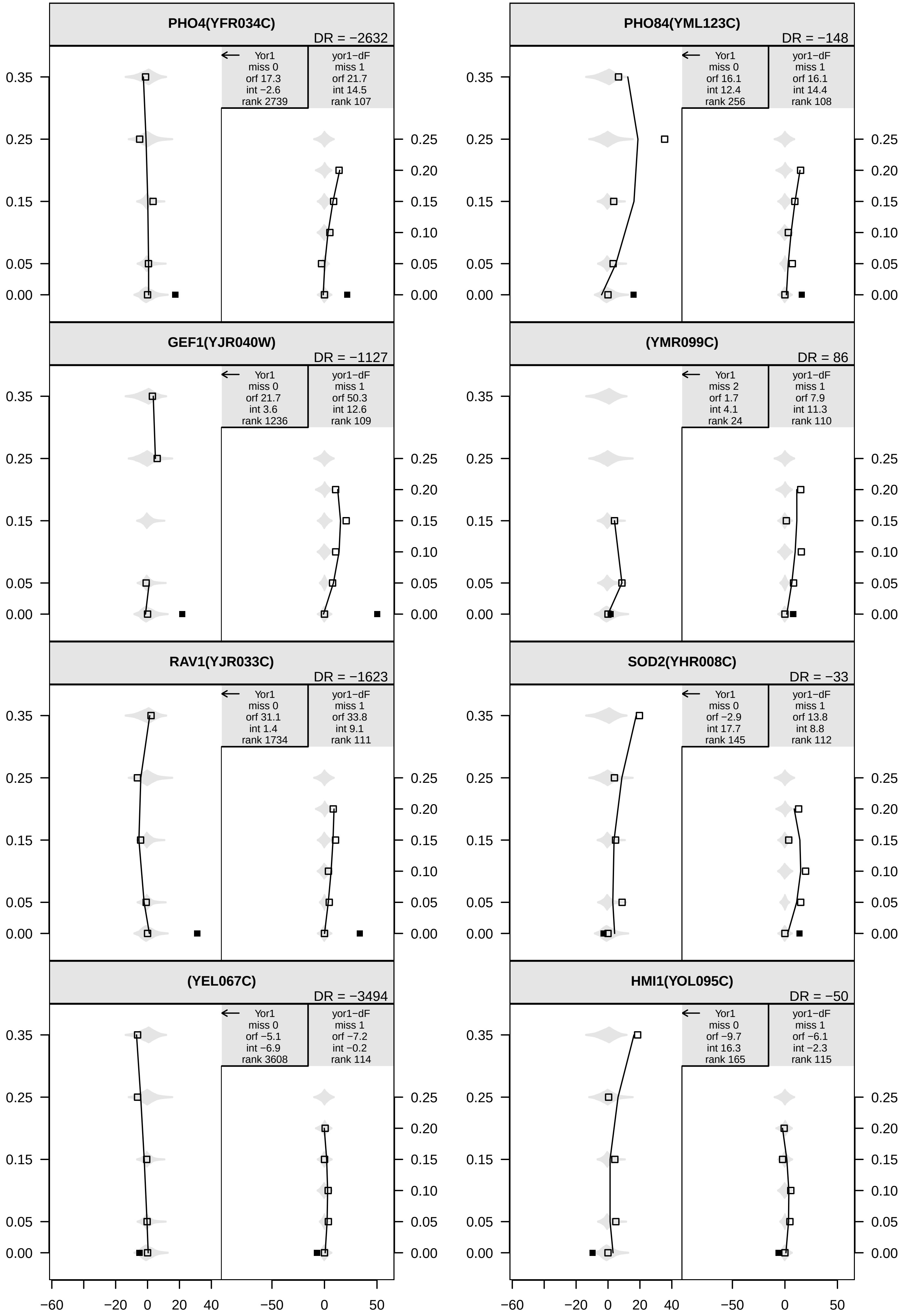

PHO4(YFR034C)
DR = −2632
Yor1 miss 0 orf 17.3 int −2.6 rank 2739
yor1−dF miss 1 orf 21.7 int 14.5 rank 107
PHO84(YML123C)
DR = −148
Yor1 miss 0 orf 16.1 int 12.4 rank 256
yor1−dF miss 1 orf 16.1 int 14.4 rank 108
GEF1(YJR040W)
DR = −1127
Yor1 miss 0 orf 21.7 int 3.6 rank 1236
yor1−dF miss 1 orf 50.3 int 12.6 rank 109
(YMR099C)
DR = 86
Yor1 miss 2 orf 1.7 int 4.1 rank 24
yor1−dF miss 1 orf 7.9 int 11.3 rank 110
RAV1(YJR033C)
DR = −1623
Yor1 miss 0 orf 31.1 int 1.4 rank 1734
yor1−dF miss 1 orf 33.8 int 9.1 rank 111
SOD2(YHR008C)
DR = −33
Yor1 miss 0 orf −2.9 int 17.7 rank 145
yor1−dF miss 1 orf 13.8 int 8.8 rank 112
(YEL067C)
DR = −3494
Yor1 miss 0 orf −5.1 int −6.9 rank 3608
yor1−dF miss 1 orf −7.2 int −0.2 rank 114
HMI1(YOL095C)
DR = −50
Yor1 miss 0 orf −9.7 int 16.3 rank 165
yor1−dF miss 1 orf −6.1 int −2.3 rank 115
0.00 0.05 0.10 0.15 0.20 0.25 0.35
−60 −20 0 20 40 −50 0 50

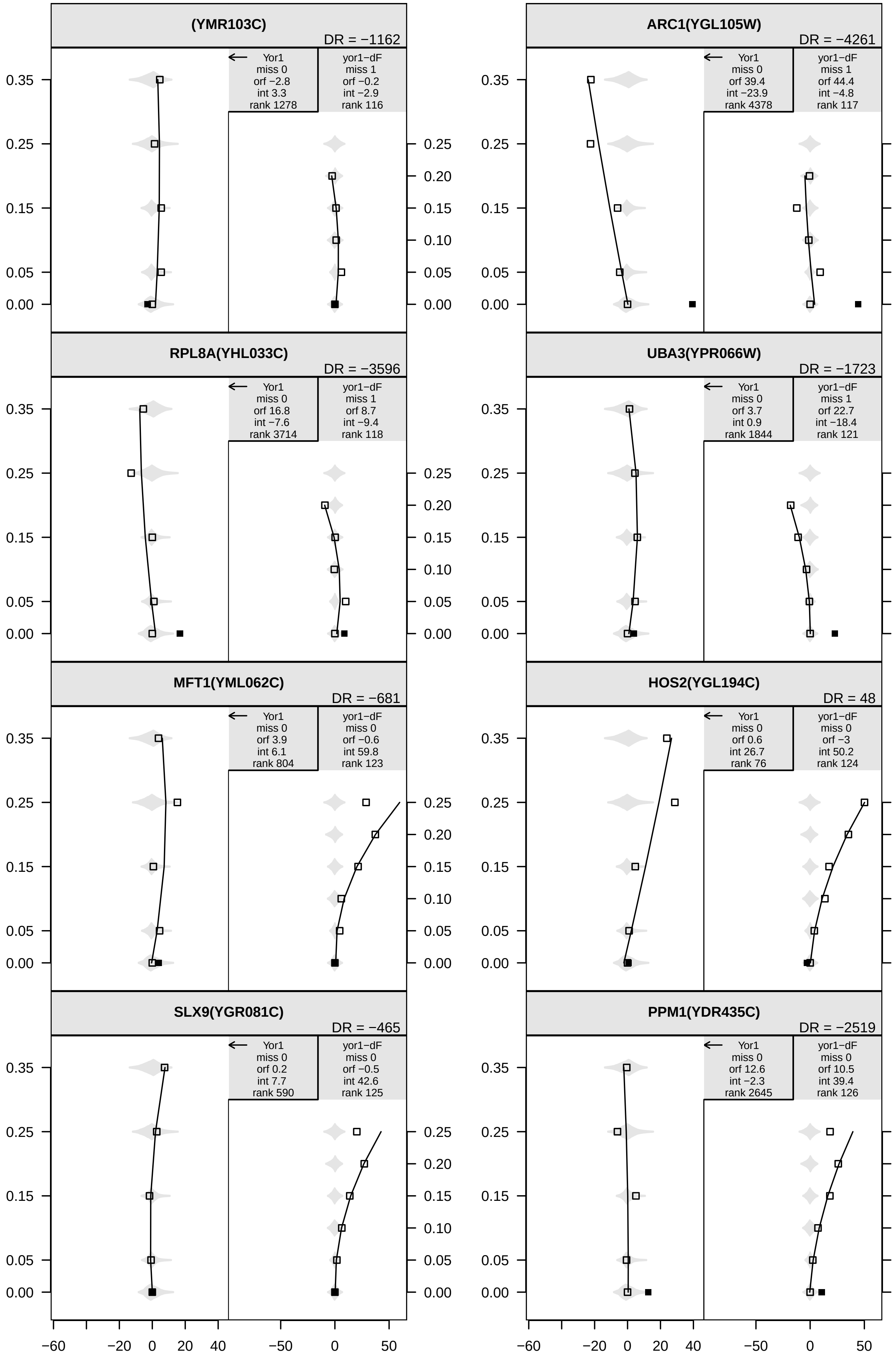

(YMR103C)
DR = −1162
Yor1
miss 0
orf −2.8
int 3.3
rank 1278
yor1−dF
miss 1
orf −0.2
int −2.9
rank 116
ARC1(YGL105W)
DR = −4261
Yor1
miss 0
orf 39.4
int −23.9
rank 4378
yor1−dF
miss 1
orf 44.4
int −4.8
rank 117
RPL8A(YHL033C)
DR = −3596
Yor1
miss 0
orf 16.8
int −7.6
rank 3714
yor1−dF
miss 1
orf 8.7
int −9.4
rank 118
UBA3(YPR066W)
DR = −1723
Yor1
miss 0
orf 3.7
int 0.9
rank 1844
yor1−dF
miss 1
orf 22.7
int −18.4
rank 121
MFT1(YML062C)
DR = −681
Yor1
miss 0
orf 3.9
int 6.1
rank 804
yor1−dF
miss 0
orf −0.6
int 59.8
rank 123
HOS2(YGL194C)
DR = 48
Yor1
miss 0
orf 0.6
int 26.7
rank 76
yor1−dF
miss 0
orf −3
int 50.2
rank 124
SLX9(YGR081C)
DR = −465
Yor1
miss 0
orf 0.2
int 7.7
rank 590
yor1−dF
miss 0
orf −0.5
int 42.6
rank 125
PPM1(YDR435C)
DR = −2519
Yor1
miss 0
orf 12.6
int −2.3
rank 2645
yor1−dF
miss 0
orf 10.5
int 39.4
rank 126
0.35
0.25
0.20
0.15
0.10
0.05
0.00
−60
−20
0
20
40
−50
0
50

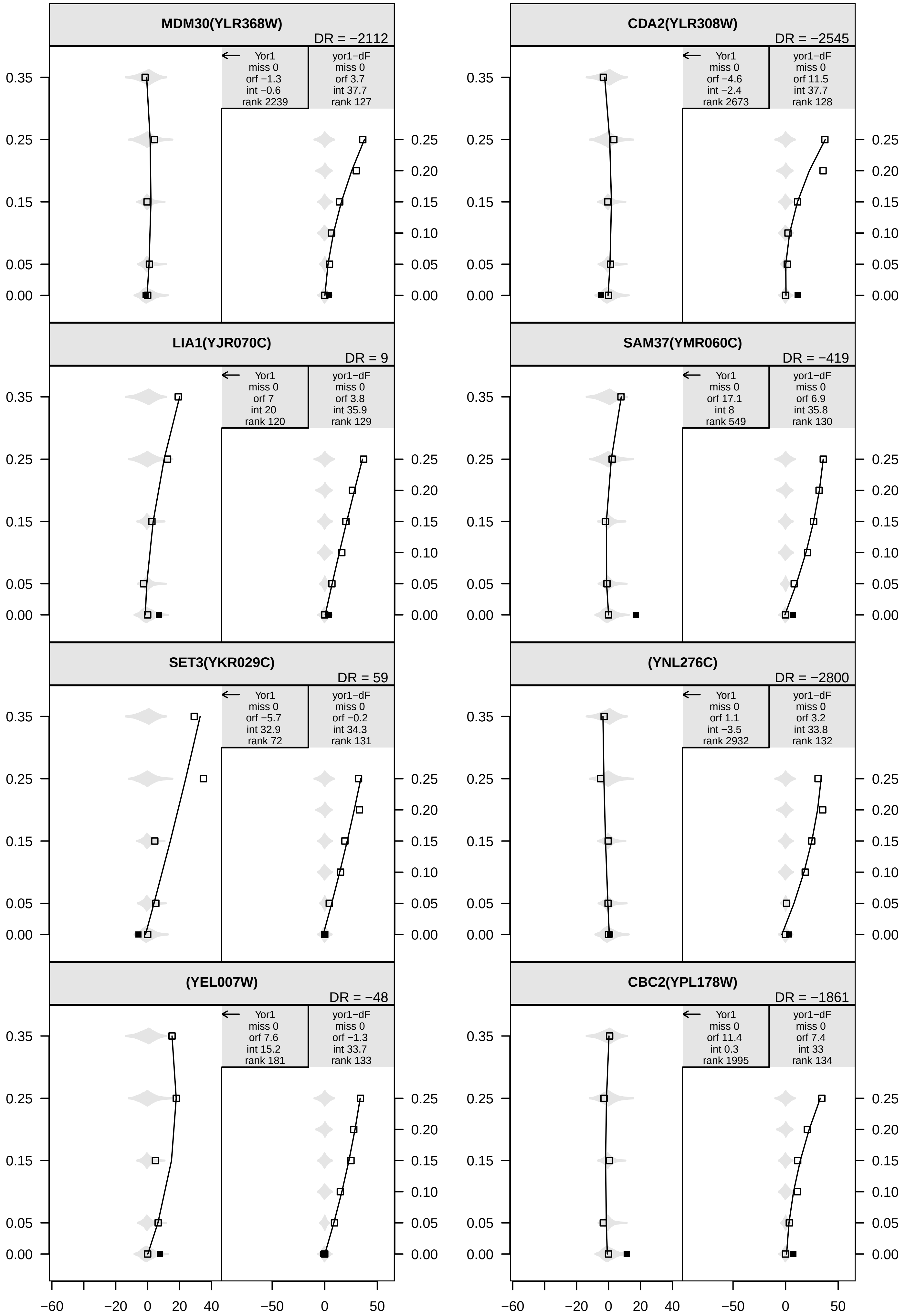
MDM30(YLR368W)
DR = −2112
Yor1 miss 0 orf −1.3 int −0.6 rank 2239
yor1−dF miss 0 orf 3.7 int 37.7 rank 127
CDA2(YLR308W)
DR = −2545
Yor1 miss 0 orf −4.6 int −2.4 rank 2673
yor1−dF miss 0 orf 11.5 int 37.7 rank 128
LIA1(YJR070C)
DR = 9
Yor1 miss 0 orf 7 int 20 rank 120
yor1−dF miss 0 orf 3.8 int 35.9 rank 129
SAM37(YMR060C)
DR = −419
Yor1 miss 0 orf 17.1 int 8 rank 549
yor1−dF miss 0 orf 6.9 int 35.8 rank 130
SET3(YKR029C)
DR = 59
Yor1 miss 0 orf −5.7 int 32.9 rank 72
yor1−dF miss 0 orf −0.2 int 34.3 rank 131
(YNL276C)
DR = −2800
Yor1 miss 0 orf 1.1 int −3.5 rank 2932
yor1−dF miss 0 orf 3.2 int 33.8 rank 132
(YEL007W)
DR = −48
Yor1 miss 0 orf 7.6 int 15.2 rank 181
yor1−dF miss 0 orf −1.3 int 33.7 rank 133
CBC2(YPL178W)
DR = −1861
Yor1 miss 0 orf 11.4 int 0.3 rank 1995
yor1−dF miss 0 orf 7.4 int 33 rank 134

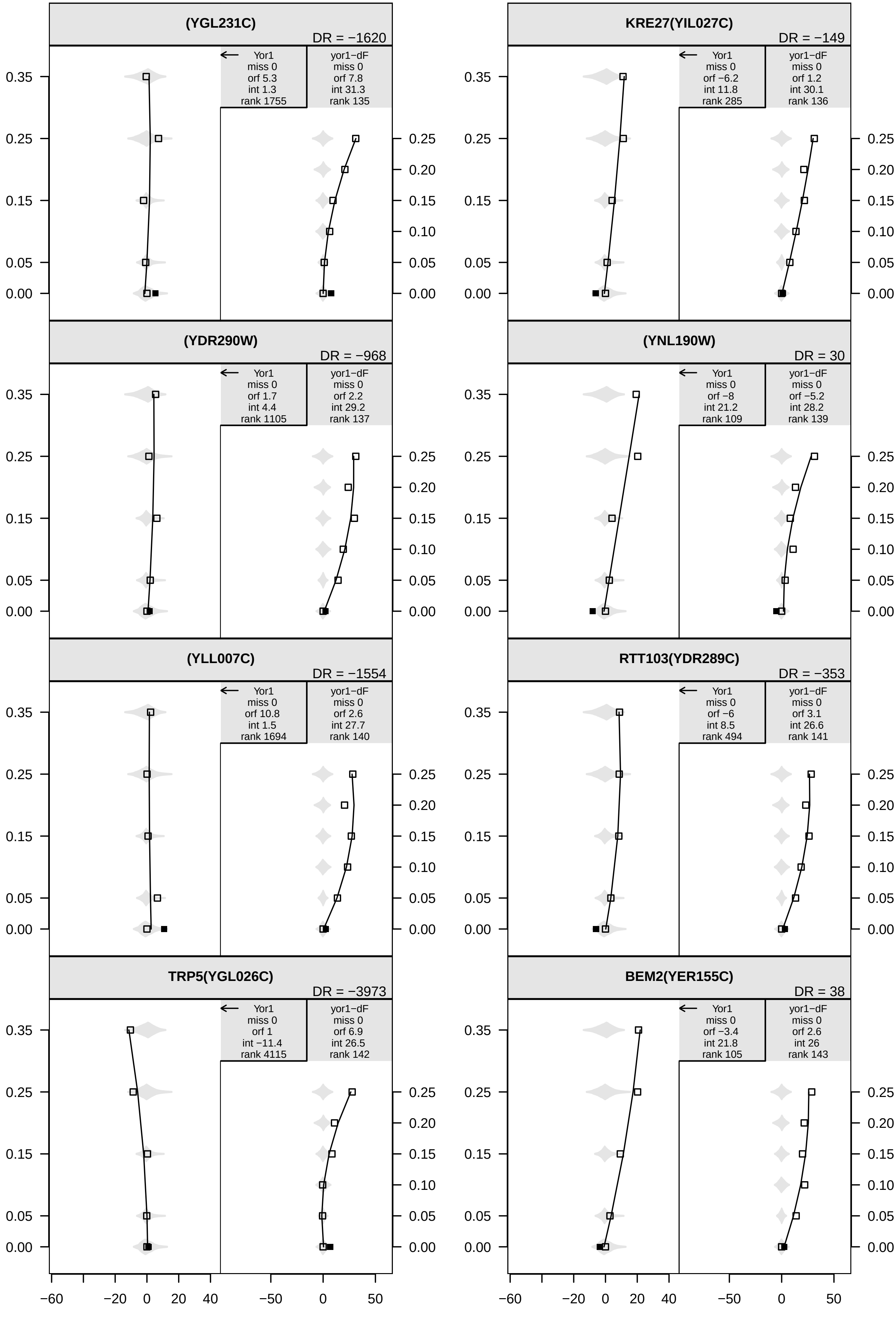

(YGL231C)
DR = −1620
Yor1 miss 0 orf 5.3 int 1.3 rank 1755
yor1−dF miss 0 orf 7.8 int 31.3 rank 135
KRE27(YIL027C)
DR = −149
Yor1 miss 0 orf −6.2 int 11.8 rank 285
yor1−dF miss 0 orf 1.2 int 30.1 rank 136
(YDR290W)
DR = −968
Yor1 miss 0 orf 1.7 int 4.4 rank 1105
yor1−dF miss 0 orf 2.2 int 29.2 rank 137
(YNL190W)
DR = 30
Yor1 miss 0 orf −8 int 21.2 rank 109
yor1−dF miss 0 orf −5.2 int 28.2 rank 139
(YLL007C)
DR = −1554
Yor1 miss 0 orf 10.8 int 1.5 rank 1694
yor1−dF miss 0 orf 2.6 int 27.7 rank 140
RTT103(YDR289C)
DR = −353
Yor1 miss 0 orf −6 int 8.5 rank 494
yor1−dF miss 0 orf 3.1 int 26.6 rank 141
TRP5(YGL026C)
DR = −3973
Yor1 miss 0 orf 1 int −11.4 rank 4115
yor1−dF miss 0 orf 6.9 int 26.5 rank 142
BEM2(YER155C)
DR = 38
Yor1 miss 0 orf −3.4 int 21.8 rank 105
yor1−dF miss 0 orf 2.6 int 26 rank 143
0.35 0.25 0.15 0.05 0.00
0.25 0.20 0.15 0.10 0.05 0.00
−60 −20 0 20 40 −50 0 50

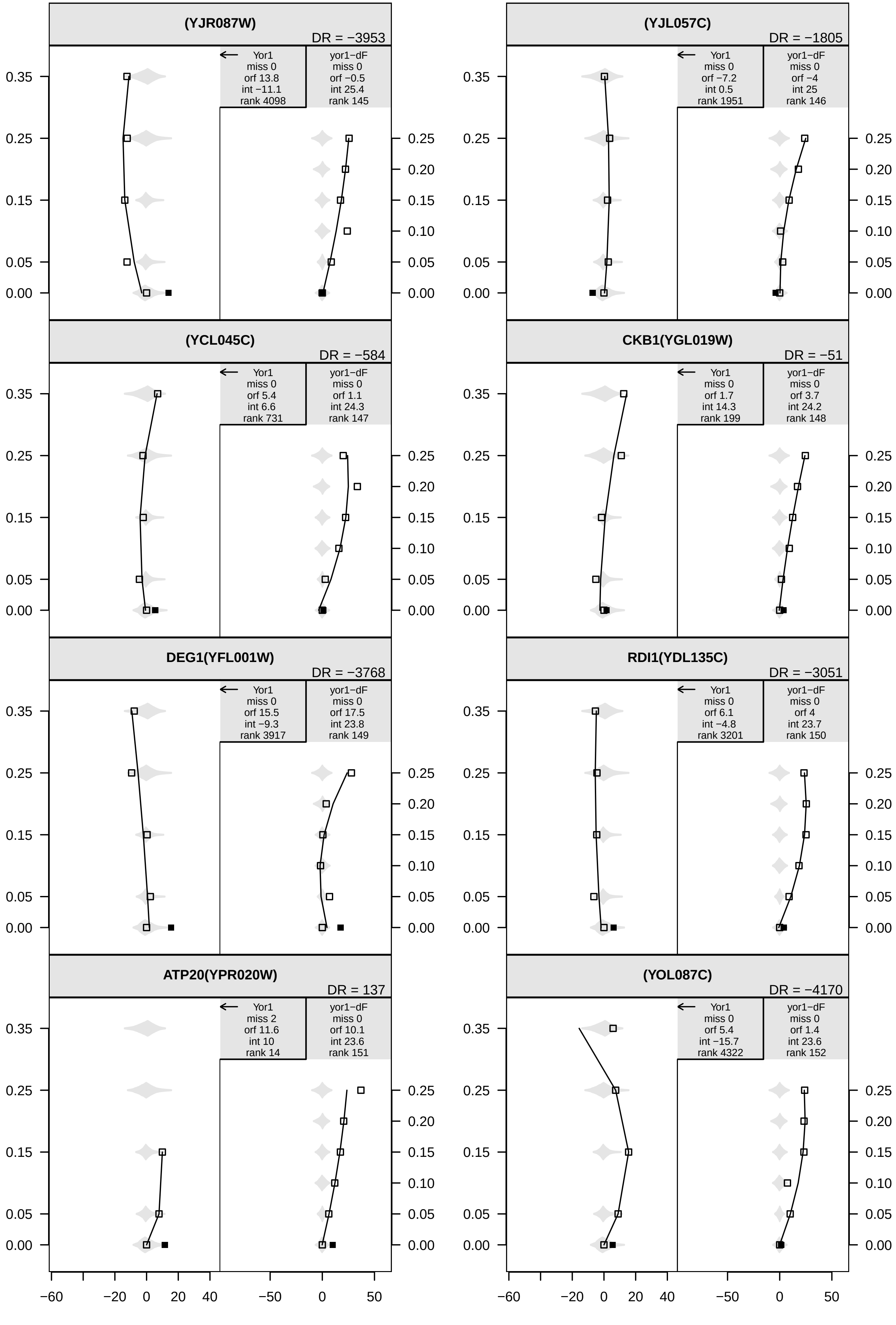

(YJR087W)
DR = −3953
Yor1 miss 0 orf 13.8 int −11.1 rank 4098
yor1−dF miss 0 orf −0.5 int 25.4 rank 145
(YJL057C)
DR = −1805
Yor1 miss 0 orf −7.2 int 0.5 rank 1951
yor1−dF miss 0 orf −4 int 25 rank 146
(YCL045C)
DR = −584
Yor1 miss 0 orf 5.4 int 6.6 rank 731
yor1−dF miss 0 orf 1.1 int 24.3 rank 147
CKB1(YGL019W)
DR = −51
Yor1 miss 0 orf 1.7 int 14.3 rank 199
yor1−dF miss 0 orf 3.7 int 24.2 rank 148
DEG1(YFL001W)
DR = −3768
Yor1 miss 0 orf 15.5 int −9.3 rank 3917
yor1−dF miss 0 orf 17.5 int 23.8 rank 149
RDI1(YDL135C)
DR = −3051
Yor1 miss 0 orf 6.1 int −4.8 rank 3201
yor1−dF miss 0 orf 4 int 23.7 rank 150
ATP20(YPR020W)
DR = 137
Yor1 miss 2 orf 11.6 int 10 rank 14
yor1−dF miss 0 orf 10.1 int 23.6 rank 151
(YOL087C)
DR = −4170
Yor1 miss 0 orf 5.4 int −15.7 rank 4322
yor1−dF miss 0 orf 1.4 int 23.6 rank 152
0.35 0.25 0.15 0.05 0.00
0.25 0.20 0.15 0.10 0.05 0.00
−60 −20 0 20 40 −50 0 50

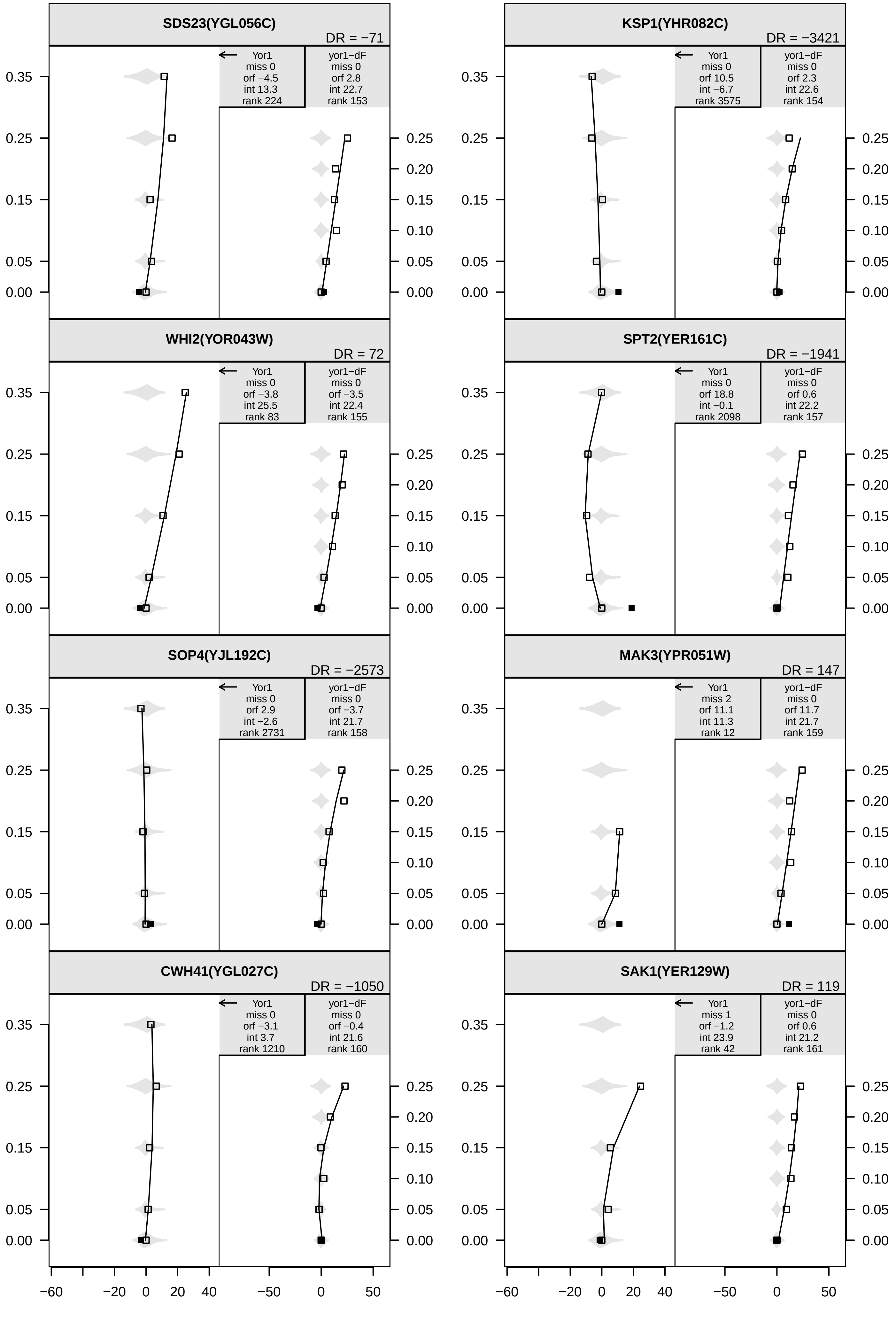

SDS23(YGL056C)
DR = −71
Yor1 miss 0 orf −4.5 int 13.3 rank 224
yor1−dF miss 0 orf 2.8 int 22.7 rank 153
KSP1(YHR082C)
DR = −3421
Yor1 miss 0 orf 10.5 int −6.7 rank 3575
yor1−dF miss 0 orf 2.3 int 22.6 rank 154
WHI2(YOR043W)
DR = 72
Yor1 miss 0 orf −3.8 int 25.5 rank 83
yor1−dF miss 0 orf −3.5 int 22.4 rank 155
SPT2(YER161C)
DR = −1941
Yor1 miss 0 orf 18.8 int −0.1 rank 2098
yor1−dF miss 0 orf 0.6 int 22.2 rank 157
SOP4(YJL192C)
DR = −2573
Yor1 miss 0 orf 2.9 int −2.6 rank 2731
yor1−dF miss 0 orf −3.7 int 21.7 rank 158
MAK3(YPR051W)
DR = 147
Yor1 miss 2 orf 11.1 int 11.3 rank 12
yor1−dF miss 0 orf 11.7 int 21.7 rank 159
CWH41(YGL027C)
DR = −1050
Yor1 miss 0 orf −3.1 int 3.7 rank 1210
yor1−dF miss 0 orf −0.4 int 21.6 rank 160
SAK1(YER129W)
DR = 119
Yor1 miss 1 orf −1.2 int 23.9 rank 42
yor1−dF miss 0 orf 0.6 int 21.2 rank 161
0.35
0.25
0.20
0.15
0.10
0.05
0.00
−60
−20
0
20
40
−50
0
50

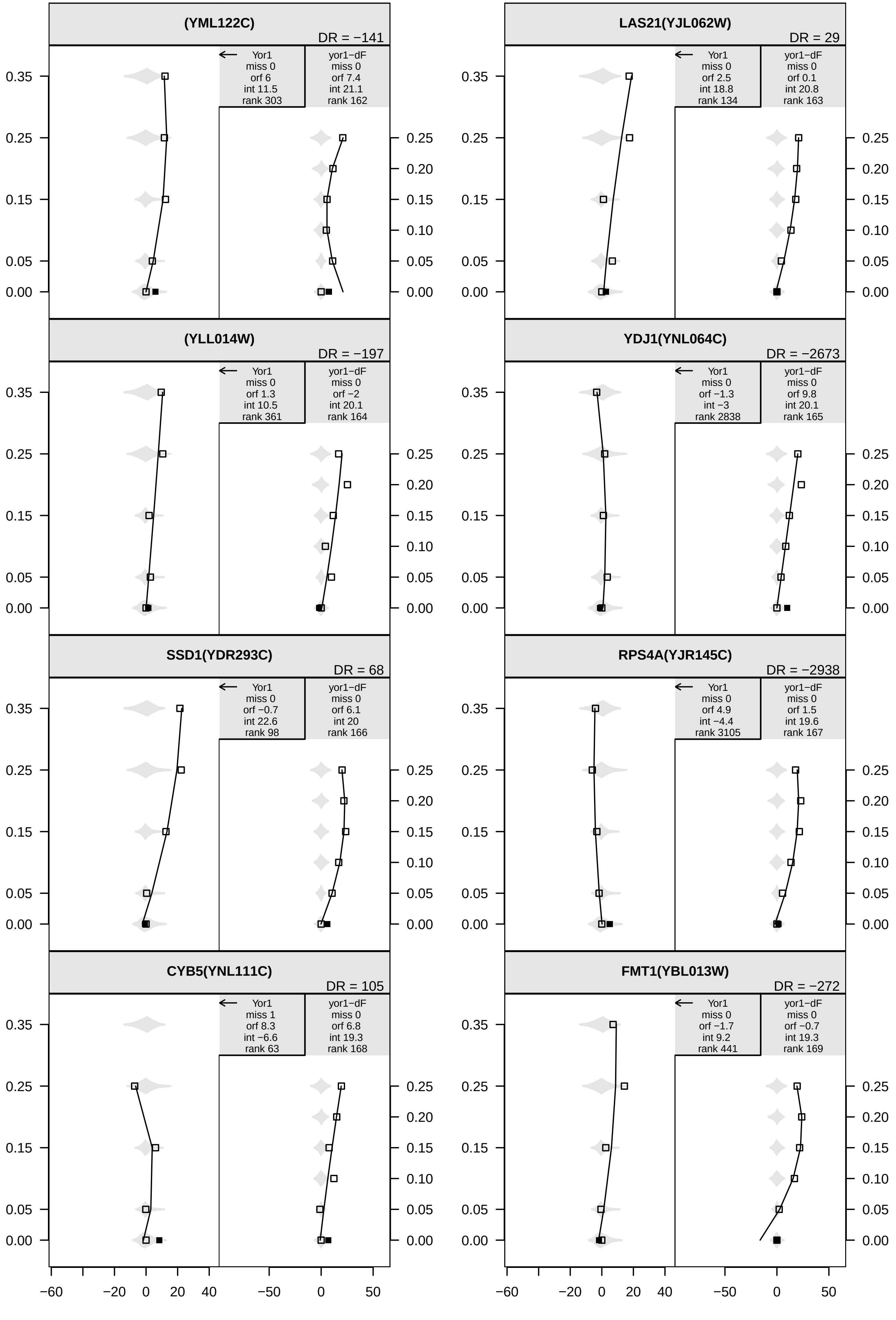
(YML122C)
DR = −141
Yor1
miss 0
orf 6
int 11.5
rank 303
yor1−dF
miss 0
orf 7.4
int 21.1
rank 162
LAS21(YJL062W)
DR = 29
Yor1
miss 0
orf 2.5
int 18.8
rank 134
yor1−dF
miss 0
orf 0.1
int 20.8
rank 163
(YLL014W)
DR = −197
Yor1
miss 0
orf 1.3
int 10.5
rank 361
yor1−dF
miss 0
orf −2
int 20.1
rank 164
YDJ1(YNL064C)
DR = −2673
Yor1
miss 0
orf −1.3
int −3
rank 2838
yor1−dF
miss 0
orf 9.8
int 20.1
rank 165
SSD1(YDR293C)
DR = 68
Yor1
miss 0
orf −0.7
int 22.6
rank 98
yor1−dF
miss 0
orf 6.1
int 20
rank 166
RPS4A(YJR145C)
DR = −2938
Yor1
miss 0
orf 4.9
int −4.4
rank 3105
yor1−dF
miss 0
orf 1.5
int 19.6
rank 167
CYB5(YNL111C)
DR = 105
Yor1
miss 1
orf 8.3
int −6.6
rank 63
yor1−dF
miss 0
orf 6.8
int 19.3
rank 168
FMT1(YBL013W)
DR = −272
Yor1
miss 0
orf −1.7
int 9.2
rank 441
yor1−dF
miss 0
orf −0.7
int 19.3
rank 169
0.35
0.25
0.20
0.15
0.10
0.05
0.00
−60
−20
0
20
40
−50
0
50

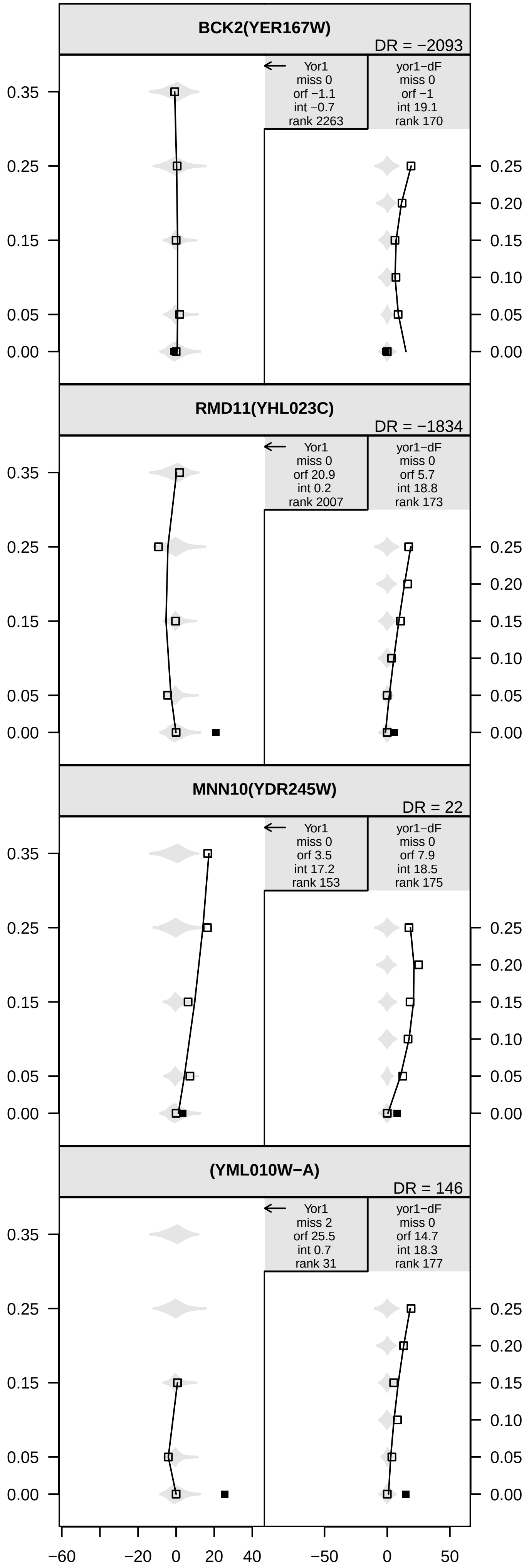
BCK2(YER167W)
DR = −2093
Yor1
miss 0
orf −1.1
int −0.7
rank 2263
yor1−dF
miss 0
orf −1
int 19.1
rank 170
(YCL047C)
DR = −3085
Yor1
miss 0
orf 25.7
int −5.1
rank 3256
yor1−dF
miss 0
orf 5.1
int 18.9
rank 171
RMD11(YHL023C)
DR = −1834
Yor1
miss 0
orf 20.9
int 0.2
rank 2007
yor1−dF
miss 0
orf 5.7
int 18.8
rank 173
FMC1(YIL098C)
DR = 173
Yor1
miss 2
orf 1.4
int 30.4
rank 1
yor1−dF
miss 0
orf 3.8
int 18.6
rank 174
MNN10(YDR245W)
DR = 22
Yor1
miss 0
orf 3.5
int 17.2
rank 153
yor1−dF
miss 0
orf 7.9
int 18.5
rank 175
NPR2(YEL062W)
DR = −377
Yor1
miss 0
orf 9.6
int 8
rank 553
yor1−dF
miss 0
orf −6.9
int 18.3
rank 176
(YML010W−A)
DR = 146
Yor1
miss 2
orf 25.5
int 0.7
rank 31
yor1−dF
miss 0
orf 14.7
int 18.3
rank 177
BSD2(YBR290W)
DR = −187
Yor1
miss 0
orf 4.9
int 10.5
rank 365
yor1−dF
miss 0
orf 5.2
int 18.2
rank 178
0.35
0.25
0.20
0.15
0.10
0.05
0.00
−60
−20
0
20
40
−50
0
50

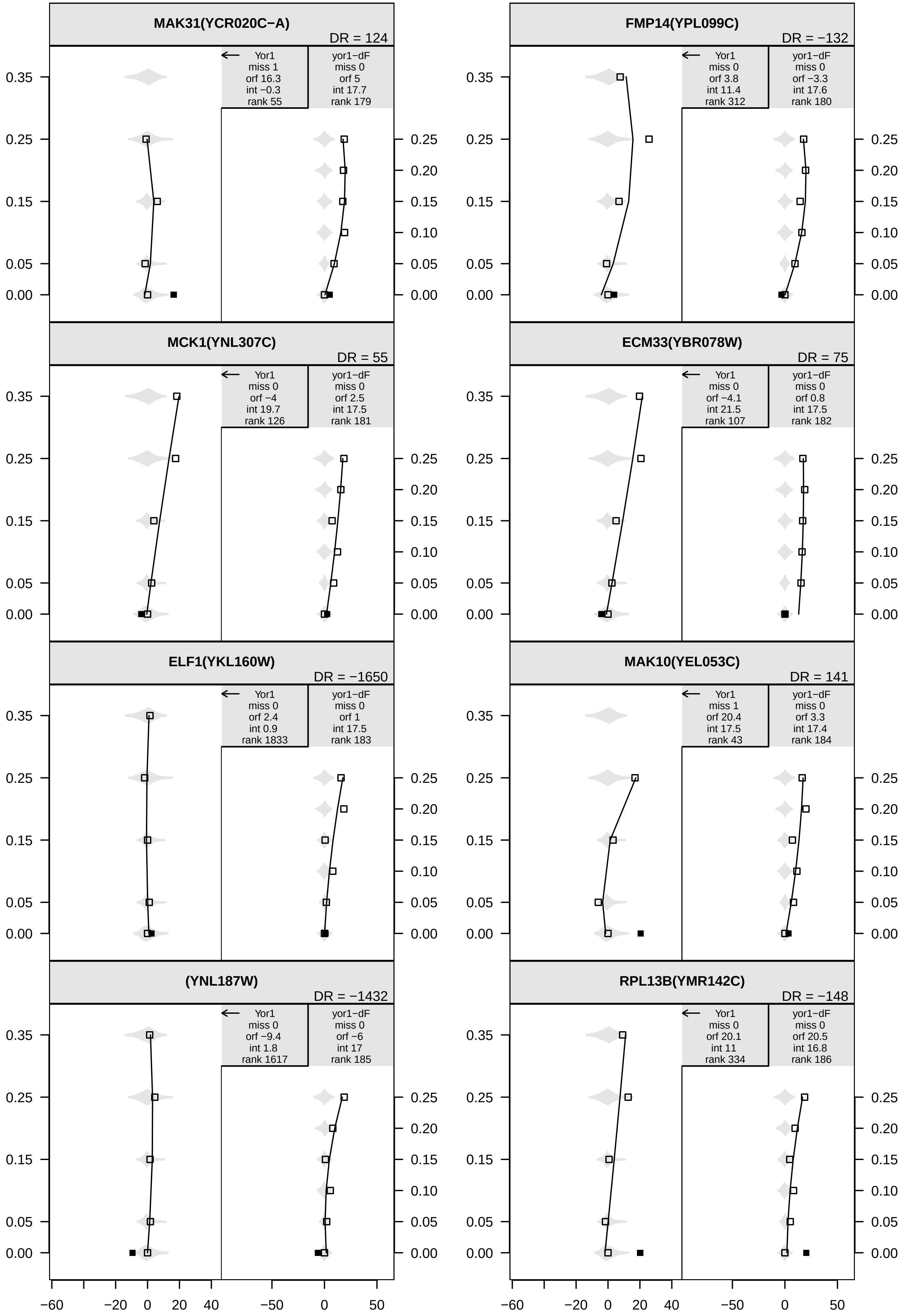
MAK31(YCR020C−A)
DR = 124
Yor1 miss 1 orf 16.3 int −0.3 rank 55
yor1−dF miss 0 orf 5 int 17.7 rank 179
FMP14(YPL099C)
DR = −132
Yor1 miss 0 orf 3.8 int 11.4 rank 312
yor1−dF miss 0 orf −3.3 int 17.6 rank 180
MCK1(YNL307C)
DR = 55
Yor1 miss 0 orf −4 int 19.7 rank 126
yor1−dF miss 0 orf 2.5 int 17.5 rank 181
ECM33(YBR078W)
DR = 75
Yor1 miss 0 orf −4.1 int 21.5 rank 107
yor1−dF miss 0 orf 0.8 int 17.5 rank 182
ELF1(YKL160W)
DR = −1650
Yor1 miss 0 orf 2.4 int 0.9 rank 1833
yor1−dF miss 0 orf 1 int 17.5 rank 183
MAK10(YEL053C)
DR = 141
Yor1 miss 1 orf 20.4 int 17.5 rank 43
yor1−dF miss 0 orf 3.3 int 17.4 rank 184
(YNL187W)
DR = −1432
Yor1 miss 0 orf −9.4 int 1.8 rank 1617
yor1−dF miss 0 orf −6 int 17 rank 185
RPL13B(YMR142C)
DR = −148
Yor1 miss 0 orf 20.1 int 11 rank 334
yor1−dF miss 0 orf 20.5 int 16.8 rank 186
0.35
0.25
0.20
0.15
0.10
0.05
0.00
−60
−20
0
20
40
−50
50

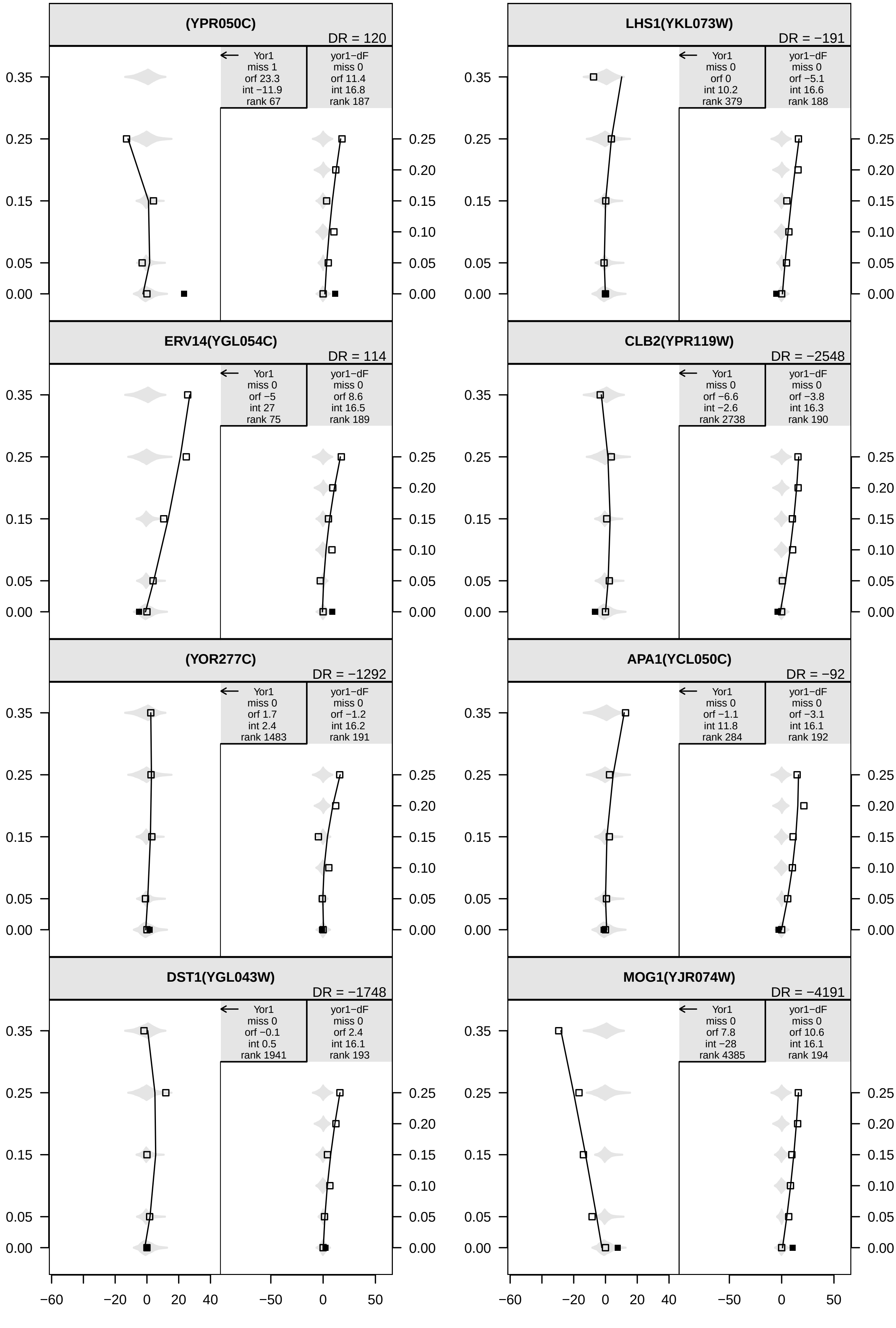

(YPR050C)
DR = 120
Yor1 miss 1 orf 23.3 int −11.9 rank 67
yor1−dF miss 0 orf 11.4 int 16.8 rank 187
LHS1(YKL073W)
DR = −191
Yor1 miss 0 orf 0 int 10.2 rank 379
yor1−dF miss 0 orf −5.1 int 16.6 rank 188
ERV14(YGL054C)
DR = 114
Yor1 miss 0 orf −5 int 27 rank 75
yor1−dF miss 0 orf 8.6 int 16.5 rank 189
CLB2(YPR119W)
DR = −2548
Yor1 miss 0 orf −6.6 int −2.6 rank 2738
yor1−dF miss 0 orf −3.8 int 16.3 rank 190
(YOR277C)
DR = −1292
Yor1 miss 0 orf 1.7 int 2.4 rank 1483
yor1−dF miss 0 orf −1.2 int 16.2 rank 191
APA1(YCL050C)
DR = −92
Yor1 miss 0 orf −1.1 int 11.8 rank 284
yor1−dF miss 0 orf −3.1 int 16.1 rank 192
DST1(YGL043W)
DR = −1748
Yor1 miss 0 orf −0.1 int 0.5 rank 1941
yor1−dF miss 0 orf 2.4 int 16.1 rank 193
MOG1(YJR074W)
DR = −4191
Yor1 miss 0 orf 7.8 int −28 rank 4385
yor1−dF miss 0 orf 10.6 int 16.1 rank 194

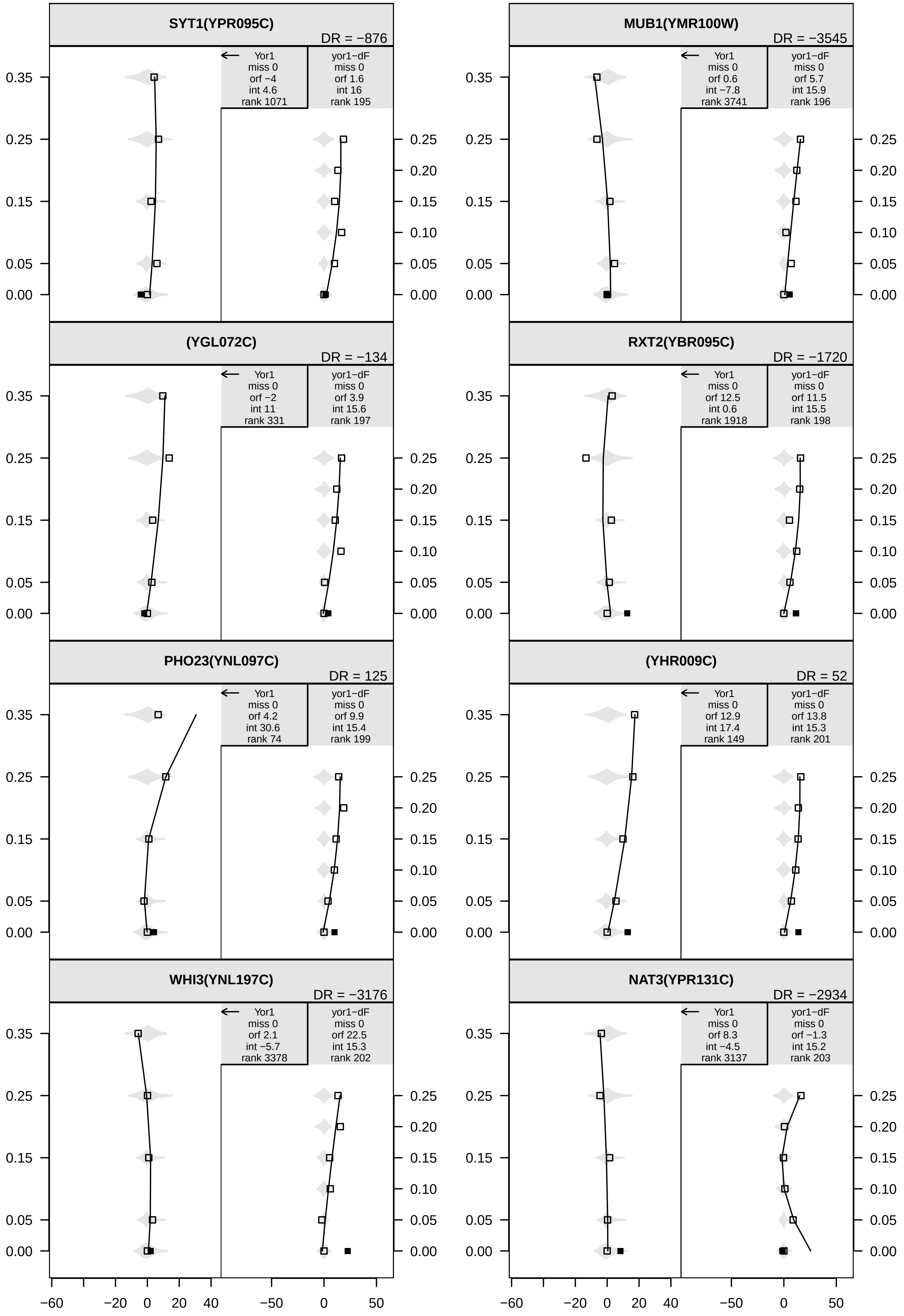

SYT1(YPR095C)
DR = −876
Yor1 miss 0 orf −4 int 4.6 rank 1071
yor1−dF miss 0 orf 1.6 int 16 rank 195
MUB1(YMR100W)
DR = −3545
Yor1 miss 0 orf 0.6 int −7.8 rank 3741
yor1−dF miss 0 orf 5.7 int 15.9 rank 196
(YGL072C)
DR = −134
Yor1 miss 0 orf −2 int 11 rank 331
yor1−dF miss 0 orf 3.9 int 15.6 rank 197
RXT2(YBR095C)
DR = −1720
Yor1 miss 0 orf 12.5 int 0.6 rank 1918
yor1−dF miss 0 orf 11.5 int 15.5 rank 198
PHO23(YNL097C)
DR = 125
Yor1 miss 0 orf 4.2 int 30.6 rank 74
yor1−dF miss 0 orf 9.9 int 15.4 rank 199
(YHR009C)
DR = 52
Yor1 miss 0 orf 12.9 int 17.4 rank 149
yor1−dF miss 0 orf 13.8 int 15.3 rank 201
WHI3(YNL197C)
DR = −3176
Yor1 miss 0 orf 2.1 int −5.7 rank 3378
yor1−dF miss 0 orf 22.5 int 15.3 rank 202
NAT3(YPR131C)
DR = −2934
Yor1 miss 0 orf 8.3 int −4.5 rank 3137
yor1−dF miss 0 orf −1.3 int 15.2 rank 203

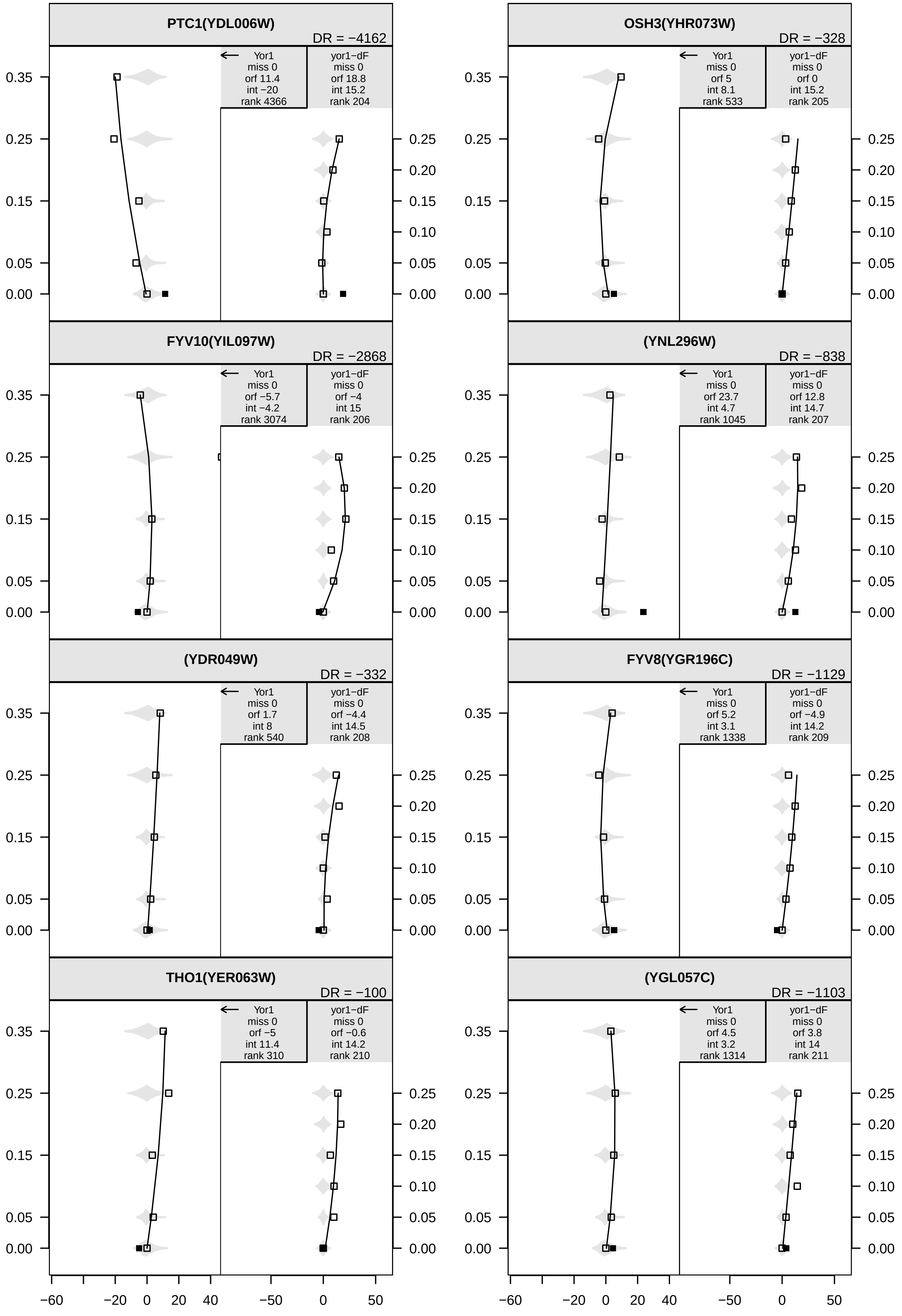

PTC1(YDL006W)
DR = −4162
Yor1 miss 0 orf 11.4 int −20 rank 4366
yor1−dF miss 0 orf 18.8 int 15.2 rank 204
OSH3(YHR073W)
DR = −328
Yor1 miss 0 orf 5 int 8.1 rank 533
yor1−dF miss 0 orf 0 int 15.2 rank 205
FYV10(YIL097W)
DR = −2868
Yor1 miss 0 orf −5.7 int −4.2 rank 3074
yor1−dF miss 0 orf −4 int 15 rank 206
(YNL296W)
DR = −838
Yor1 miss 0 orf 23.7 int 4.7 rank 1045
yor1−dF miss 0 orf 12.8 int 14.7 rank 207
(YDR049W)
DR = −332
Yor1 miss 0 orf 1.7 int 8 rank 540
yor1−dF miss 0 orf −4.4 int 14.5 rank 208
FYV8(YGR196C)
DR = −1129
Yor1 miss 0 orf 5.2 int 3.1 rank 1338
yor1−dF miss 0 orf −4.9 int 14.2 rank 209
THO1(YER063W)
DR = −100
Yor1 miss 0 orf −5 int 11.4 rank 310
yor1−dF miss 0 orf −0.6 int 14.2 rank 210
(YGL057C)
DR = −1103
Yor1 miss 0 orf 4.5 int 3.2 rank 1314
yor1−dF miss 0 orf 3.8 int 14 rank 211
0.35 0.25 0.20 0.15 0.10 0.05 0.00
−60 −20 0 20 40 −50 0 50

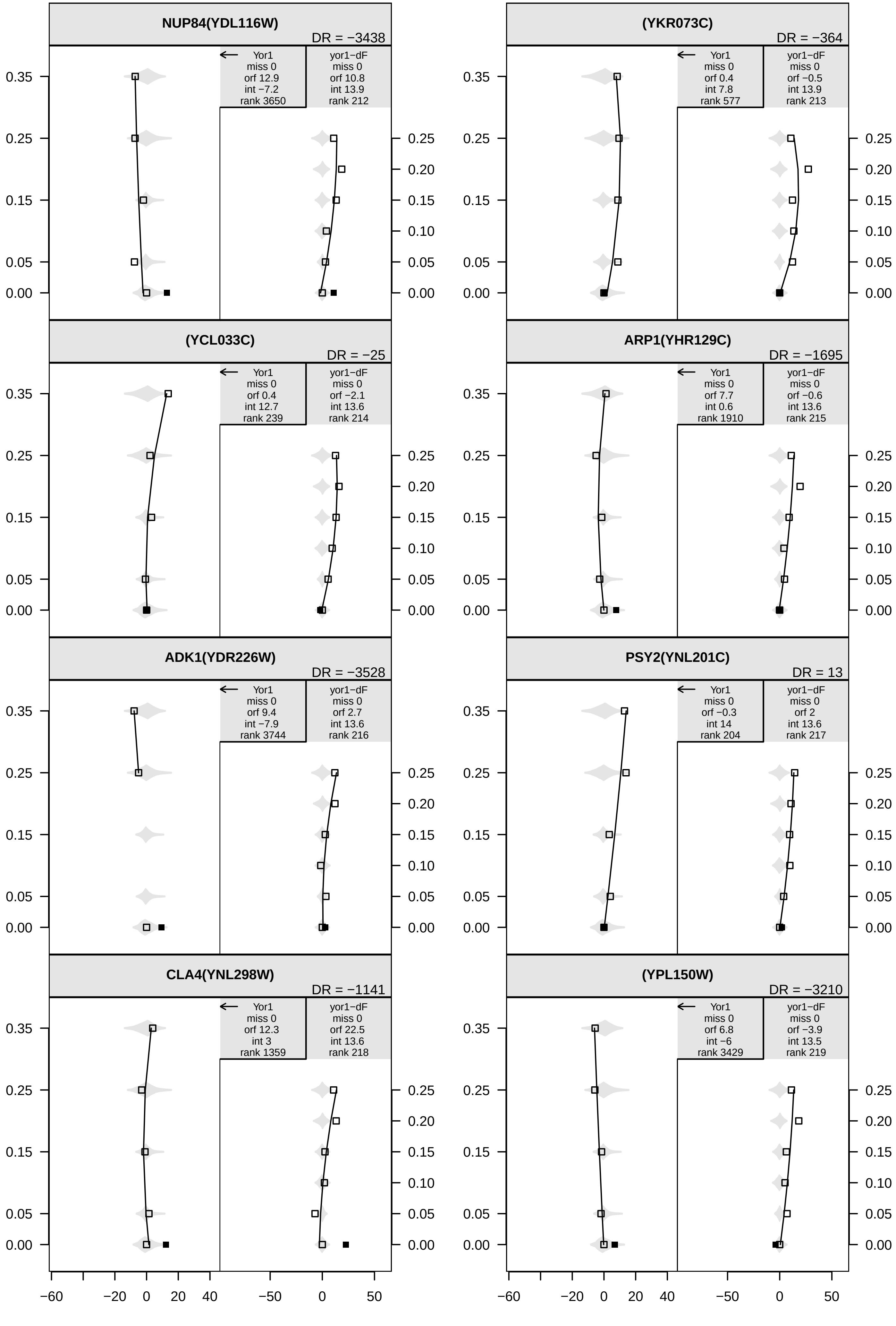

NUP84(YDL116W)
DR = −3438
Yor1 miss 0 orf 12.9 int −7.2 rank 3650
yor1−dF miss 0 orf 10.8 int 13.9 rank 212
(YKR073C)
DR = −364
Yor1 miss 0 orf 0.4 int 7.8 rank 577
yor1−dF miss 0 orf −0.5 int 13.9 rank 213
(YCL033C)
DR = −25
Yor1 miss 0 orf 0.4 int 12.7 rank 239
yor1−dF miss 0 orf −2.1 int 13.6 rank 214
ARP1(YHR129C)
DR = −1695
Yor1 miss 0 orf 7.7 int 0.6 rank 1910
yor1−dF miss 0 orf −0.6 int 13.6 rank 215
ADK1(YDR226W)
DR = −3528
Yor1 miss 0 orf 9.4 int −7.9 rank 3744
yor1−dF miss 0 orf 2.7 int 13.6 rank 216
PSY2(YNL201C)
DR = 13
Yor1 miss 0 orf −0.3 int 14 rank 204
yor1−dF miss 0 orf 2 int 13.6 rank 217
CLA4(YNL298W)
DR = −1141
Yor1 miss 0 orf 12.3 int 3 rank 1359
yor1−dF miss 0 orf 22.5 int 13.6 rank 218
(YPL150W)
DR = −3210
Yor1 miss 0 orf 6.8 int −6 rank 3429
yor1−dF miss 0 orf −3.9 int 13.5 rank 219
0.35 0.25 0.15 0.05 0.00
0.25 0.20 0.15 0.10 0.05 0.00
−60 −20 0 20 40
−50 0 50

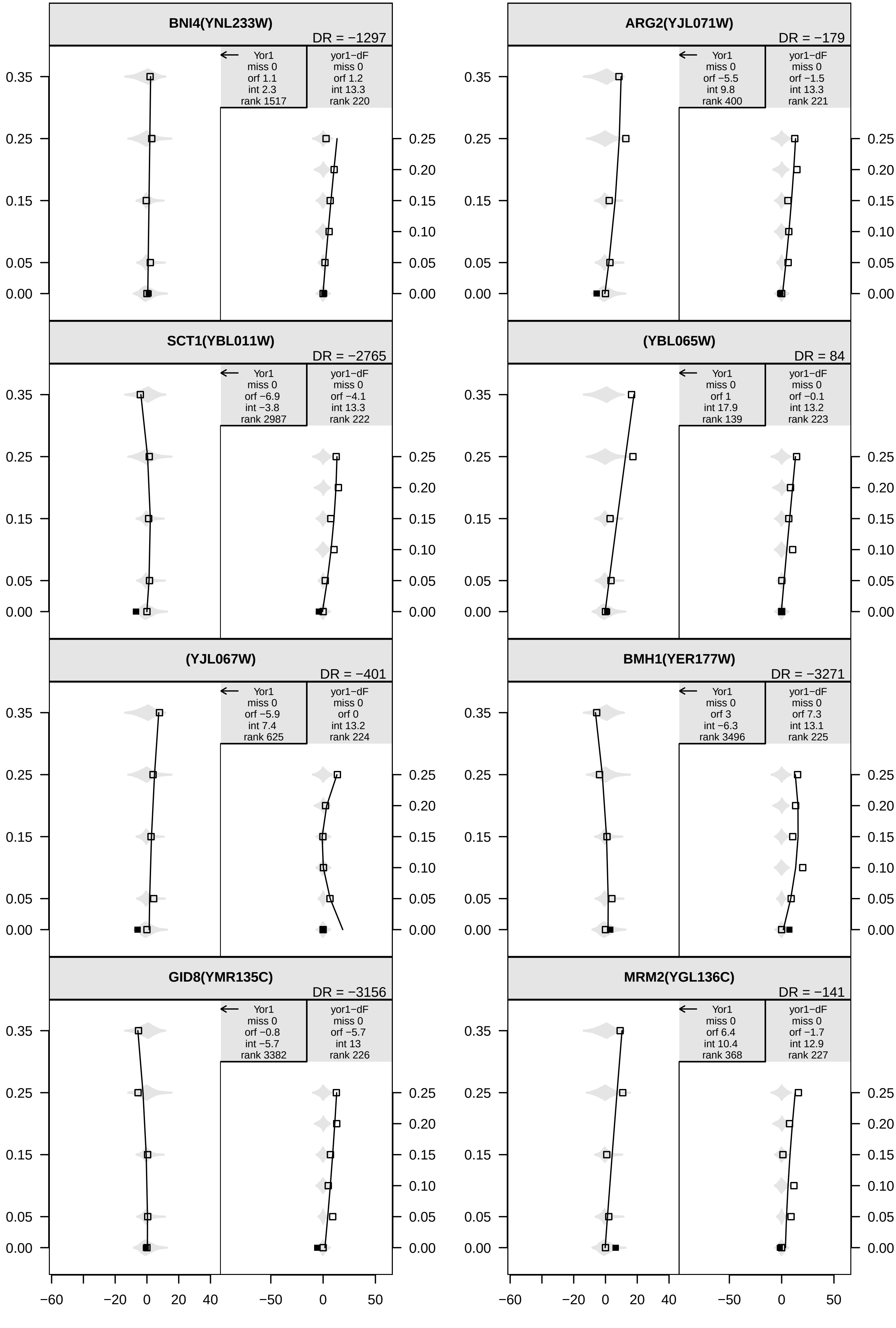
BNI4(YNL233W)
DR = −1297
Yor1 miss 0 orf 1.1 int 2.3 rank 1517
yor1−dF miss 0 orf 1.2 int 13.3 rank 220
ARG2(YJL071W)
DR = −179
Yor1 miss 0 orf −5.5 int 9.8 rank 400
yor1−dF miss 0 orf −1.5 int 13.3 rank 221
SCT1(YBL011W)
DR = −2765
Yor1 miss 0 orf −6.9 int −3.8 rank 2987
yor1−dF miss 0 orf −4.1 int 13.3 rank 222
(YBL065W)
DR = 84
Yor1 miss 0 orf 1 int 17.9 rank 139
yor1−dF miss 0 orf −0.1 int 13.2 rank 223
(YJL067W)
DR = −401
Yor1 miss 0 orf −5.9 int 7.4 rank 625
yor1−dF miss 0 orf 0 int 13.2 rank 224
BMH1(YER177W)
DR = −3271
Yor1 miss 0 orf 3 int −6.3 rank 3496
yor1−dF miss 0 orf 7.3 int 13.1 rank 225
GID8(YMR135C)
DR = −3156
Yor1 miss 0 orf −0.8 int −5.7 rank 3382
yor1−dF miss 0 orf −5.7 int 13 rank 226
MRM2(YGL136C)
DR = −141
Yor1 miss 0 orf 6.4 int 10.4 rank 368
yor1−dF miss 0 orf −1.7 int 12.9 rank 227

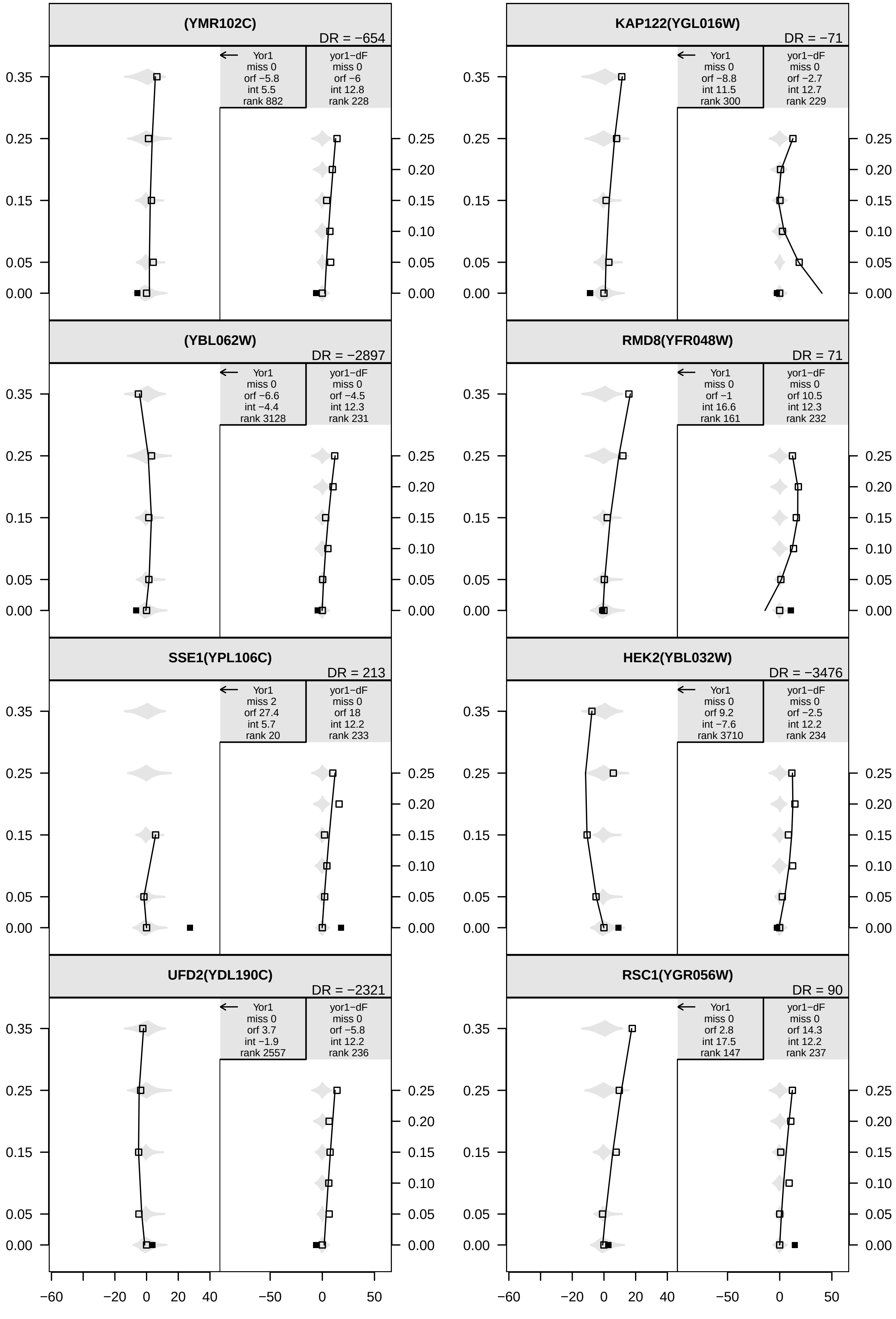
(YMR102C)
DR = −654
Yor1 miss 0 orf −5.8 int 5.5 rank 882
yor1−dF miss 0 orf −6 int 12.8 rank 228
KAP122(YGL016W)
DR = −71
Yor1 miss 0 orf −8.8 int 11.5 rank 300
yor1−dF miss 0 orf −2.7 int 12.7 rank 229
(YBL062W)
DR = −2897
Yor1 miss 0 orf −6.6 int −4.4 rank 3128
yor1−dF miss 0 orf −4.5 int 12.3 rank 231
RMD8(YFR048W)
DR = 71
Yor1 miss 0 orf −1 int 16.6 rank 161
yor1−dF miss 0 orf 10.5 int 12.3 rank 232
SSE1(YPL106C)
DR = 213
Yor1 miss 2 orf 27.4 int 5.7 rank 20
yor1−dF miss 0 orf 18 int 12.2 rank 233
HEK2(YBL032W)
DR = −3476
Yor1 miss 0 orf 9.2 int −7.6 rank 3710
yor1−dF miss 0 orf −2.5 int 12.2 rank 234
UFD2(YDL190C)
DR = −2321
Yor1 miss 0 orf 3.7 int −1.9 rank 2557
yor1−dF miss 0 orf −5.8 int 12.2 rank 236
RSC1(YGR056W)
DR = 90
Yor1 miss 0 orf 2.8 int 17.5 rank 147
yor1−dF miss 0 orf 14.3 int 12.2 rank 237
0.35 0.25 0.15 0.05 0.00
0.25 0.20 0.15 0.10 0.05 0.00
−60 −20 0 20 40
−50 0 50

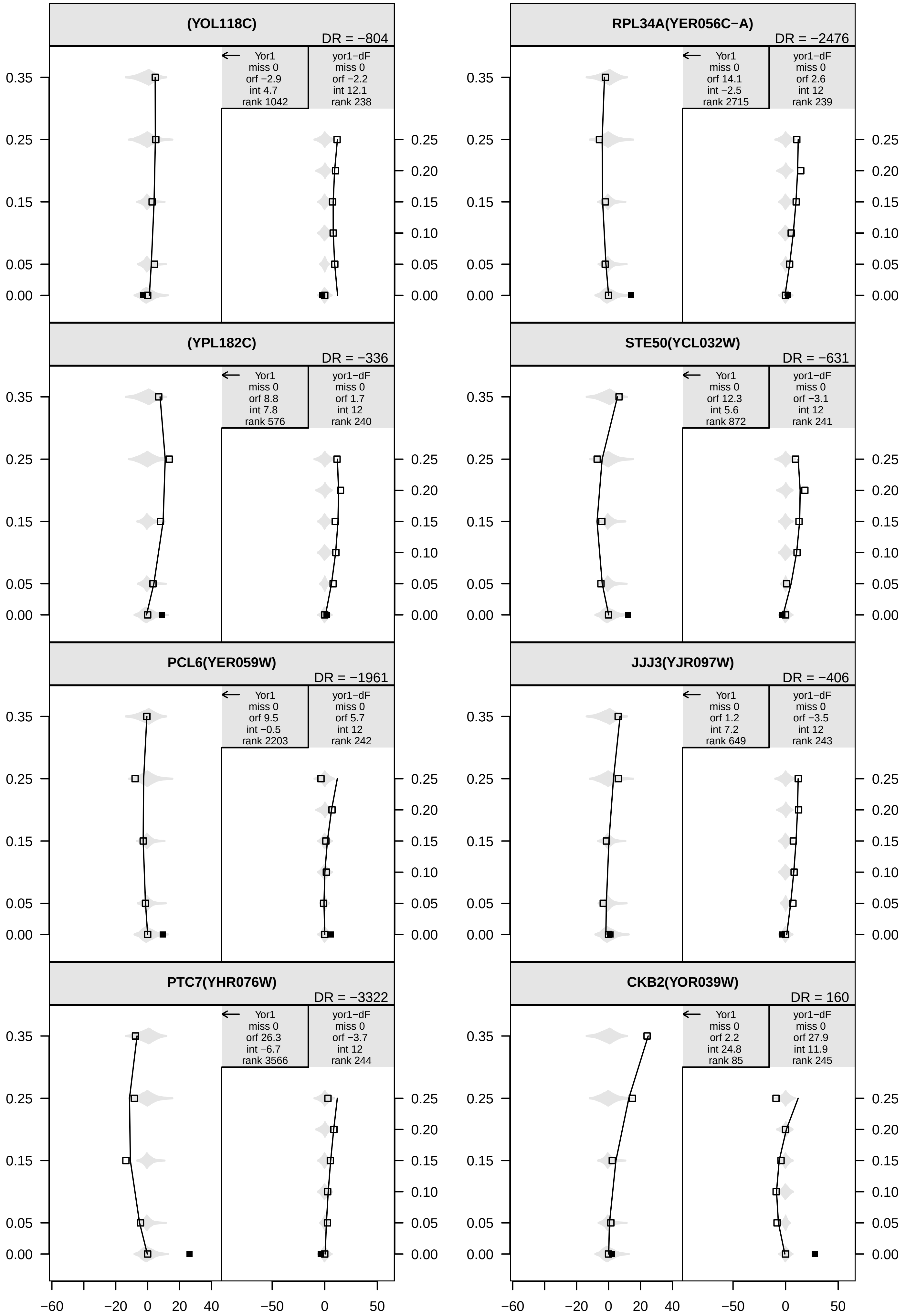
(YOL118C)
DR = −804
Yor1
miss 0
orf −2.9
int 4.7
rank 1042
yor1−dF
miss 0
orf −2.2
int 12.1
rank 238
RPL34A(YER056C−A)
DR = −2476
Yor1
miss 0
orf 14.1
int −2.5
rank 2715
yor1−dF
miss 0
orf 2.6
int 12
rank 239
(YPL182C)
DR = −336
Yor1
miss 0
orf 8.8
int 7.8
rank 576
yor1−dF
miss 0
orf 1.7
int 12
rank 240
STE50(YCL032W)
DR = −631
Yor1
miss 0
orf 12.3
int 5.6
rank 872
yor1−dF
miss 0
orf −3.1
int 12
rank 241
PCL6(YER059W)
DR = −1961
Yor1
miss 0
orf 9.5
int −0.5
rank 2203
yor1−dF
miss 0
orf 5.7
int 12
rank 242
JJJ3(YJR097W)
DR = −406
Yor1
miss 0
orf 1.2
int 7.2
rank 649
yor1−dF
miss 0
orf −3.5
int 12
rank 243
PTC7(YHR076W)
DR = −3322
Yor1
miss 0
orf 26.3
int −6.7
rank 3566
yor1−dF
miss 0
orf −3.7
int 12
rank 244
CKB2(YOR039W)
DR = 160
Yor1
miss 0
orf 2.2
int 24.8
rank 85
yor1−dF
miss 0
orf 27.9
int 11.9
rank 245
0.35
0.25
0.20
0.15
0.10
0.05
0.00
−60
−20
0
20
40
−50
0
50

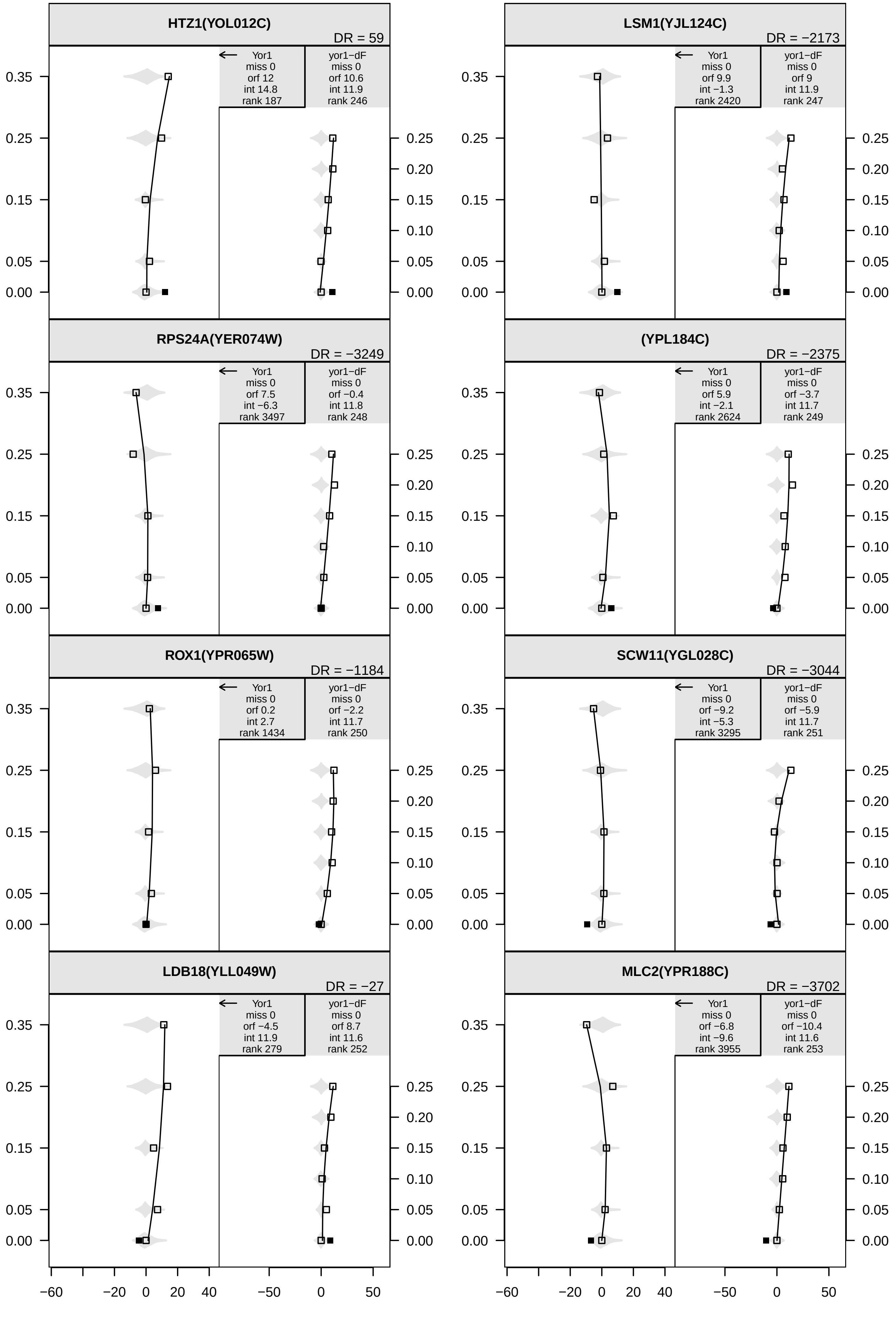
HTZ1(YOL012C)
DR = 59
Yor1 miss 0 orf 12 int 14.8 rank 187
yor1−dF miss 0 orf 10.6 int 11.9 rank 246
LSM1(YJL124C)
DR = −2173
Yor1 miss 0 orf 9.9 int −1.3 rank 2420
yor1−dF miss 0 orf 9 int 11.9 rank 247
RPS24A(YER074W)
DR = −3249
Yor1 miss 0 orf 7.5 int −6.3 rank 3497
yor1−dF miss 0 orf −0.4 int 11.8 rank 248
(YPL184C)
DR = −2375
Yor1 miss 0 orf 5.9 int −2.1 rank 2624
yor1−dF miss 0 orf −3.7 int 11.7 rank 249
ROX1(YPR065W)
DR = −1184
Yor1 miss 0 orf 0.2 int 2.7 rank 1434
yor1−dF miss 0 orf −2.2 int 11.7 rank 250
SCW11(YGL028C)
DR = −3044
Yor1 miss 0 orf −9.2 int −5.3 rank 3295
yor1−dF miss 0 orf −5.9 int 11.7 rank 251
LDB18(YLL049W)
DR = −27
Yor1 miss 0 orf −4.5 int 11.9 rank 279
yor1−dF miss 0 orf 8.7 int 11.6 rank 252
MLC2(YPR188C)
DR = −3702
Yor1 miss 0 orf −6.8 int −9.6 rank 3955
yor1−dF miss 0 orf −10.4 int 11.6 rank 253
0.35 0.25 0.15 0.05 0.00
0.25 0.20 0.15 0.10 0.05 0.00
−60 −20 0 20 40
−50 0 50

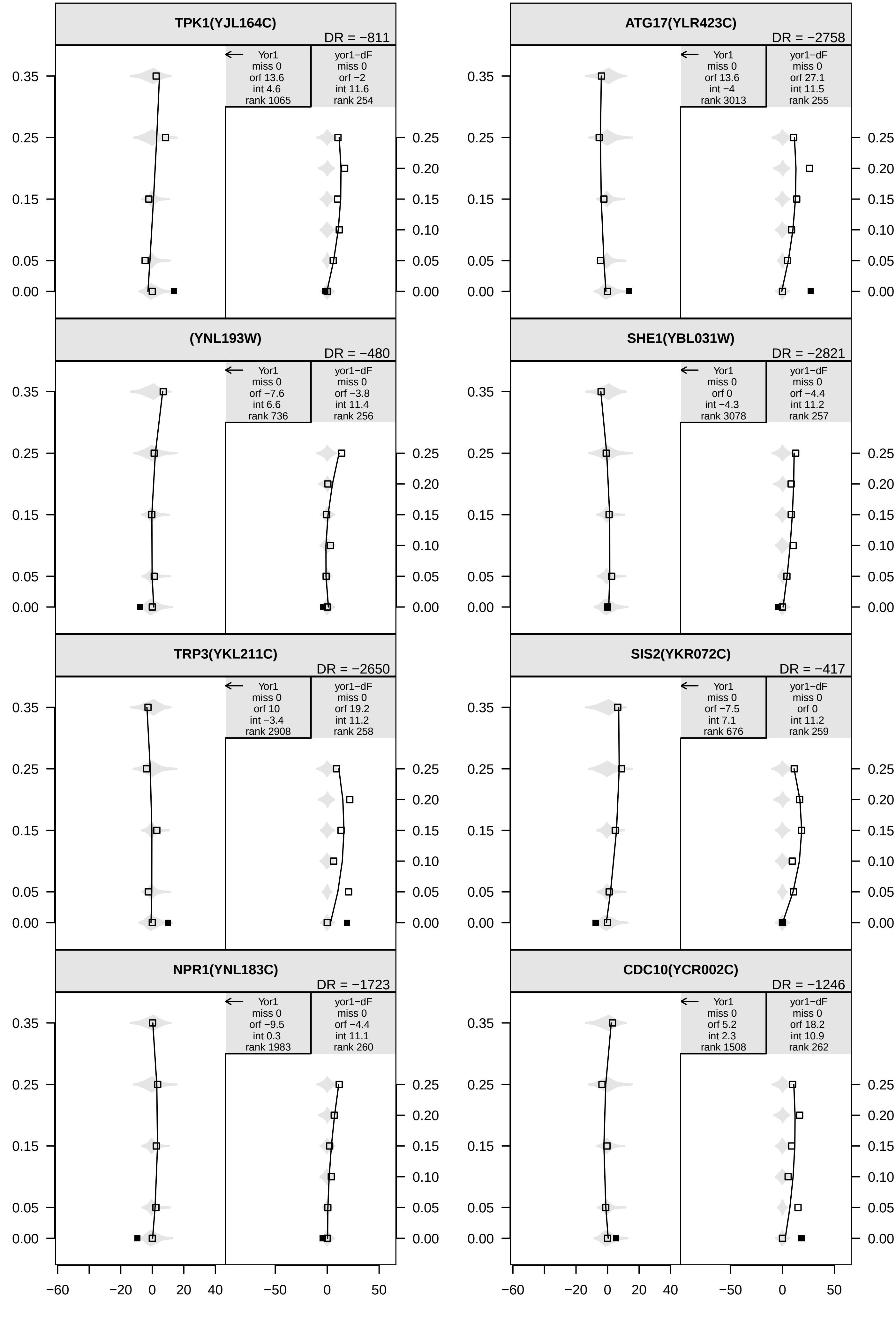
TPK1(YJL164C)
DR = −811
Yor1 miss 0 orf 13.6 int 4.6 rank 1065
yor1−dF miss 0 orf −2 int 11.6 rank 254
ATG17(YLR423C)
DR = −2758
Yor1 miss 0 orf 13.6 int −4 rank 3013
yor1−dF miss 0 orf 27.1 int 11.5 rank 255
(YNL193W)
DR = −480
Yor1 miss 0 orf −7.6 int 6.6 rank 736
yor1−dF miss 0 orf −3.8 int 11.4 rank 256
SHE1(YBL031W)
DR = −2821
Yor1 miss 0 orf 0 int −4.3 rank 3078
yor1−dF miss 0 orf −4.4 int 11.2 rank 257
TRP3(YKL211C)
DR = −2650
Yor1 miss 0 orf 10 int −3.4 rank 2908
yor1−dF miss 0 orf 19.2 int 11.2 rank 258
SIS2(YKR072C)
DR = −417
Yor1 miss 0 orf −7.5 int 7.1 rank 676
yor1−dF miss 0 orf 0 int 11.2 rank 259
NPR1(YNL183C)
DR = −1723
Yor1 miss 0 orf −9.5 int 0.3 rank 1983
yor1−dF miss 0 orf −4.4 int 11.1 rank 260
CDC10(YCR002C)
DR = −1246
Yor1 miss 0 orf 5.2 int 2.3 rank 1508
yor1−dF miss 0 orf 18.2 int 10.9 rank 262
0.00 0.05 0.10 0.15 0.20 0.25 0.35
−60 −20 0 20 40
−50 0 50

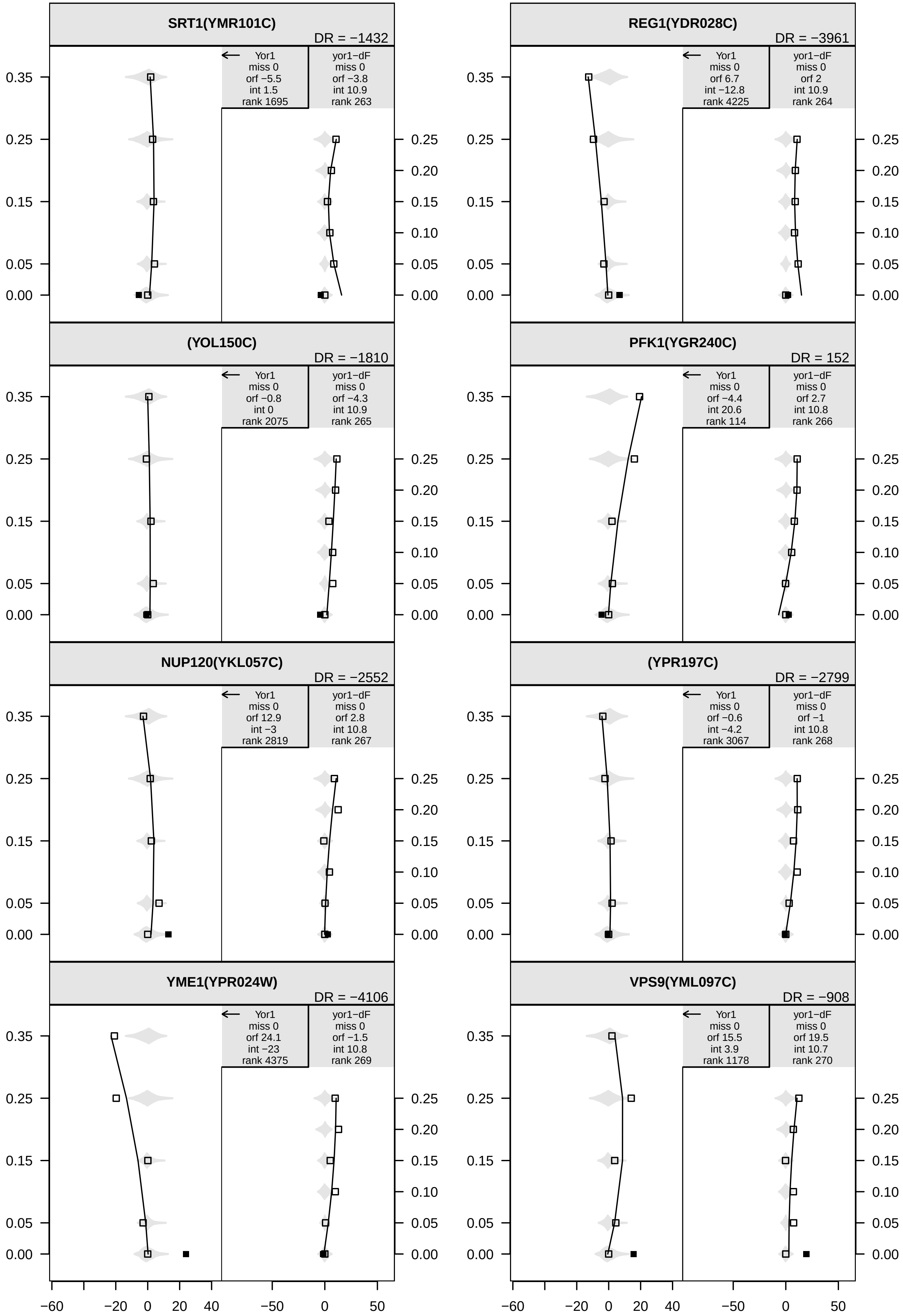
SRT1(YMR101C)
DR = −1432
Yor1
miss 0
orf −5.5
int 1.5
rank 1695
yor1−dF
miss 0
orf −3.8
int 10.9
rank 263
REG1(YDR028C)
DR = −3961
Yor1
miss 0
orf 6.7
int −12.8
rank 4225
yor1−dF
miss 0
orf 2
int 10.9
rank 264
(YOL150C)
DR = −1810
Yor1
miss 0
orf −0.8
int 0
rank 2075
yor1−dF
miss 0
orf −4.3
int 10.9
rank 265
PFK1(YGR240C)
DR = 152
Yor1
miss 0
orf −4.4
int 20.6
rank 114
yor1−dF
miss 0
orf 2.7
int 10.8
rank 266
NUP120(YKL057C)
DR = −2552
Yor1
miss 0
orf 12.9
int −3
rank 2819
yor1−dF
miss 0
orf 2.8
int 10.8
rank 267
(YPR197C)
DR = −2799
Yor1
miss 0
orf −0.6
int −4.2
rank 3067
yor1−dF
miss 0
orf −1
int 10.8
rank 268
YME1(YPR024W)
DR = −4106
Yor1
miss 0
orf 24.1
int −23
rank 4375
yor1−dF
miss 0
orf −1.5
int 10.8
rank 269
VPS9(YML097C)
DR = −908
Yor1
miss 0
orf 15.5
int 3.9
rank 1178
yor1−dF
miss 0
orf 19.5
int 10.7
rank 270
0.35
0.25
0.20
0.15
0.10
0.05
0.00
−60
−20
0
20
40
−50
0
50

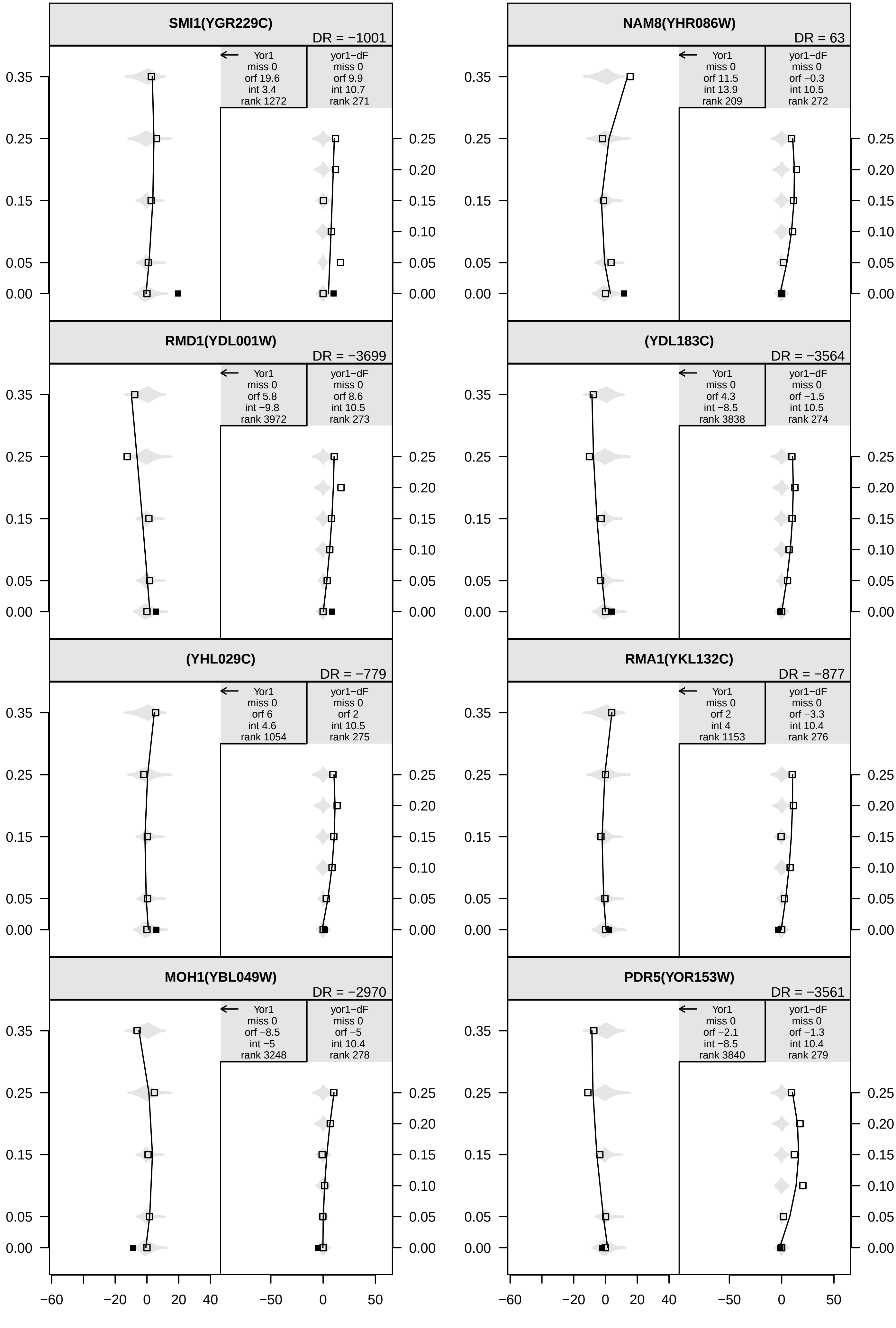

SMI1(YGR229C)
DR = −1001
Yor1 miss 0 orf 19.6 int 3.4 rank 1272
yor1−dF miss 0 orf 9.9 int 10.7 rank 271
NAM8(YHR086W)
DR = 63
Yor1 miss 0 orf 11.5 int 13.9 rank 209
yor1−dF miss 0 orf −0.3 int 10.5 rank 272
RMD1(YDL001W)
DR = −3699
Yor1 miss 0 orf 5.8 int −9.8 rank 3972
yor1−dF miss 0 orf 8.6 int 10.5 rank 273
(YDL183C)
DR = −3564
Yor1 miss 0 orf 4.3 int −8.5 rank 3838
yor1−dF miss 0 orf −1.5 int 10.5 rank 274
(YHL029C)
DR = −779
Yor1 miss 0 orf 6 int 4.6 rank 1054
yor1−dF miss 0 orf 2 int 10.5 rank 275
RMA1(YKL132C)
DR = −877
Yor1 miss 0 orf 2 int 4 rank 1153
yor1−dF miss 0 orf −3.3 int 10.4 rank 276
MOH1(YBL049W)
DR = −2970
Yor1 miss 0 orf −8.5 int −5 rank 3248
yor1−dF miss 0 orf −5 int 10.4 rank 278
PDR5(YOR153W)
DR = −3561
Yor1 miss 0 orf −2.1 int −8.5 rank 3840
yor1−dF miss 0 orf −1.3 int 10.4 rank 279
0.35 0.25 0.15 0.05 0.00
0.25 0.20 0.15 0.10 0.05 0.00
−60 −20 0 20 40 −50 0 50

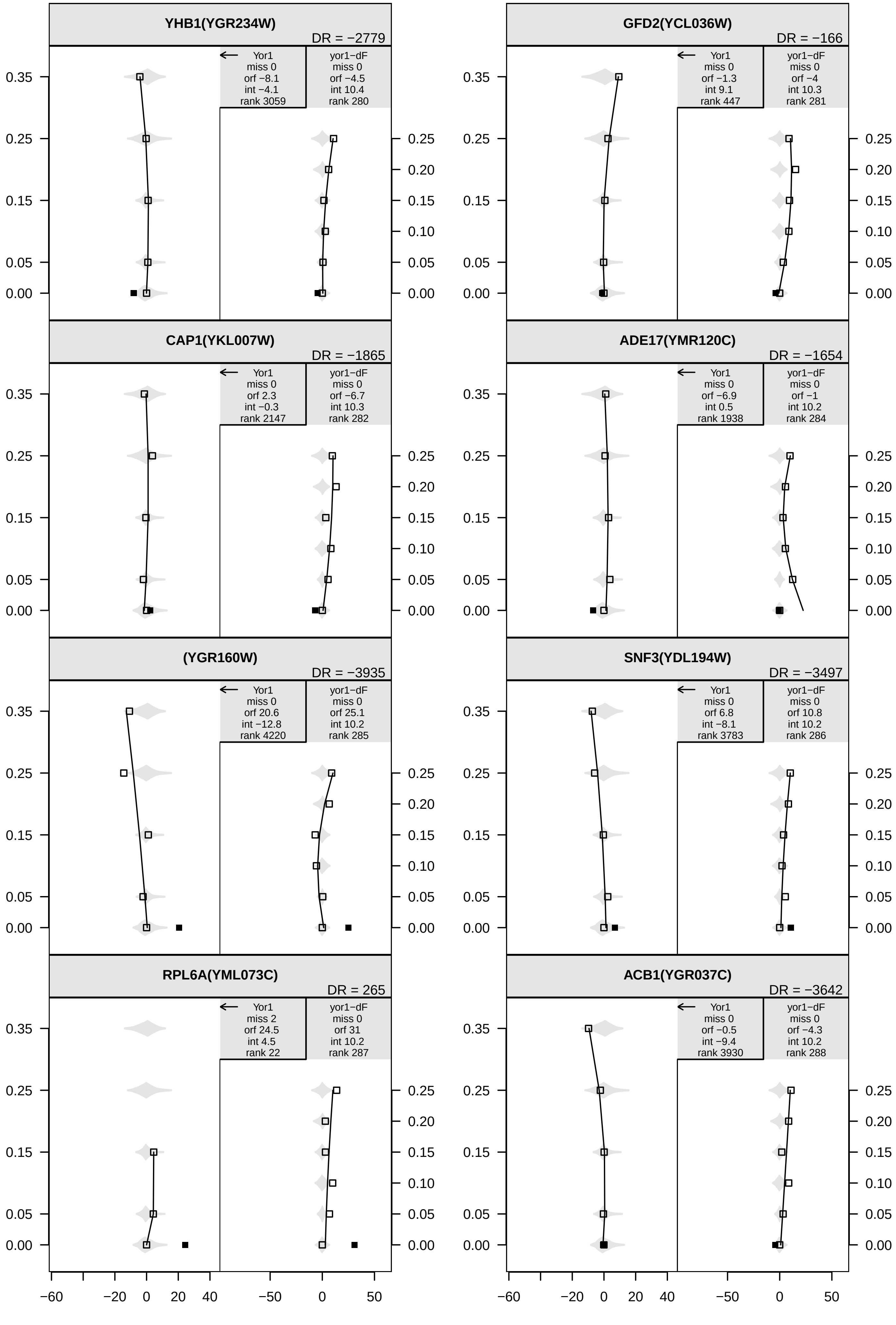

YHB1(YGR234W)
DR = −2779
Yor1 miss 0 orf −8.1 int −4.1 rank 3059
yor1−dF miss 0 orf −4.5 int 10.4 rank 280
GFD2(YCL036W)
DR = −166
Yor1 miss 0 orf −1.3 int 9.1 rank 447
yor1−dF miss 0 orf −4 int 10.3 rank 281
CAP1(YKL007W)
DR = −1865
Yor1 miss 0 orf 2.3 int −0.3 rank 2147
yor1−dF miss 0 orf −6.7 int 10.3 rank 282
ADE17(YMR120C)
DR = −1654
Yor1 miss 0 orf −6.9 int 0.5 rank 1938
yor1−dF miss 0 orf −1 int 10.2 rank 284
(YGR160W)
DR = −3935
Yor1 miss 0 orf 20.6 int −12.8 rank 4220
yor1−dF miss 0 orf 25.1 int 10.2 rank 285
SNF3(YDL194W)
DR = −3497
Yor1 miss 0 orf 6.8 int −8.1 rank 3783
yor1−dF miss 0 orf 10.8 int 10.2 rank 286
RPL6A(YML073C)
DR = 265
Yor1 miss 2 orf 24.5 int 4.5 rank 22
yor1−dF miss 0 orf 31 int 10.2 rank 287
ACB1(YGR037C)
DR = −3642
Yor1 miss 0 orf −0.5 int −9.4 rank 3930
yor1−dF miss 0 orf −4.3 int 10.2 rank 288
0.00 0.05 0.10 0.15 0.20 0.25 0.35
−60 −20 0 20 40 −50 0 50

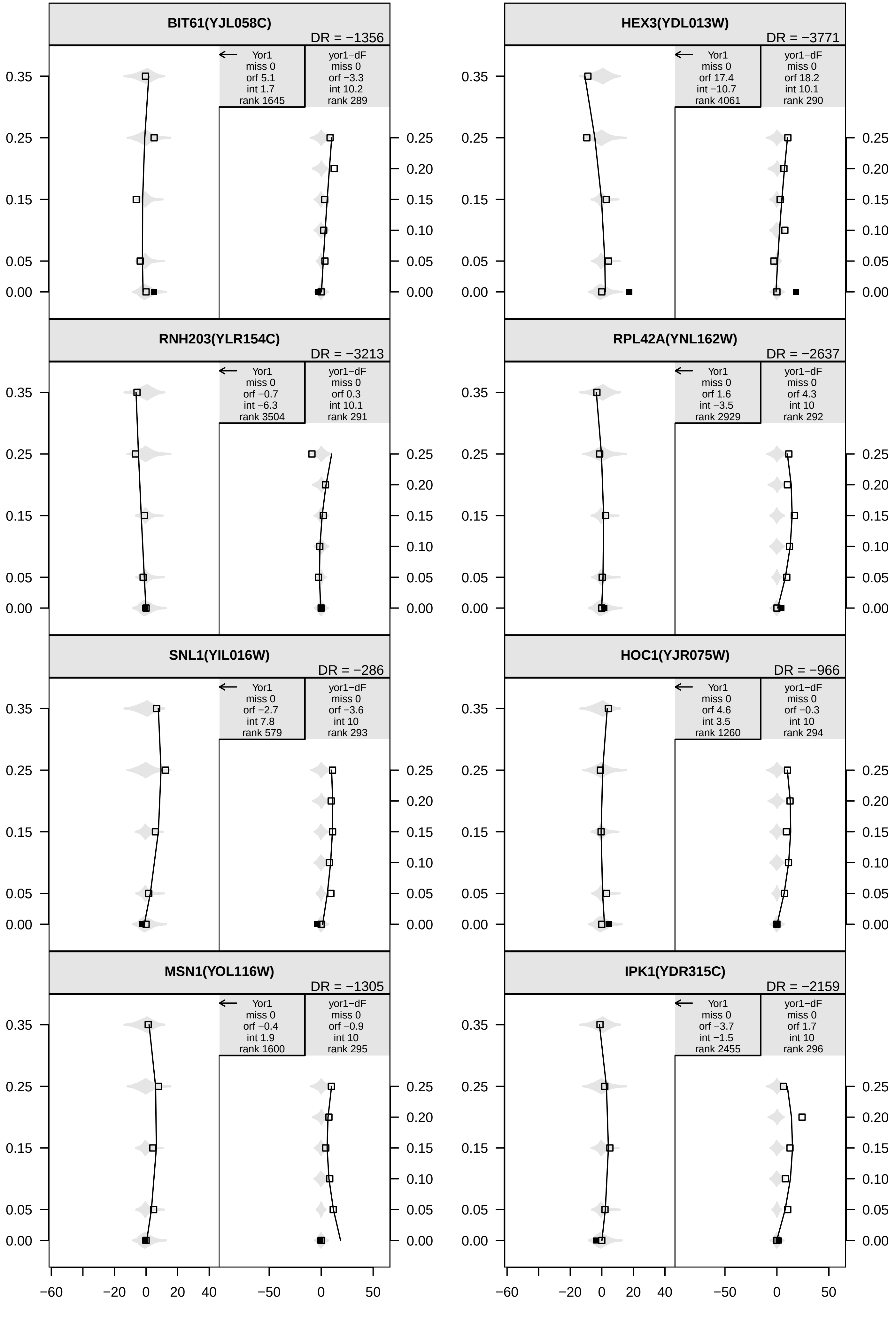
BIT61(YJL058C)
DR = −1356
Yor1 miss 0 orf 5.1 int 1.7 rank 1645
yor1−dF miss 0 orf −3.3 int 10.2 rank 289
HEX3(YDL013W)
DR = −3771
Yor1 miss 0 orf 17.4 int −10.7 rank 4061
yor1−dF miss 0 orf 18.2 int 10.1 rank 290
RNH203(YLR154C)
DR = −3213
Yor1 miss 0 orf −0.7 int −6.3 rank 3504
yor1−dF miss 0 orf 0.3 int 10.1 rank 291
RPL42A(YNL162W)
DR = −2637
Yor1 miss 0 orf 1.6 int −3.5 rank 2929
yor1−dF miss 0 orf 4.3 int 10 rank 292
SNL1(YIL016W)
DR = −286
Yor1 miss 0 orf −2.7 int 7.8 rank 579
yor1−dF miss 0 orf −3.6 int 10 rank 293
HOC1(YJR075W)
DR = −966
Yor1 miss 0 orf 4.6 int 3.5 rank 1260
yor1−dF miss 0 orf −0.3 int 10 rank 294
MSN1(YOL116W)
DR = −1305
Yor1 miss 0 orf −0.4 int 1.9 rank 1600
yor1−dF miss 0 orf −0.9 int 10 rank 295
IPK1(YDR315C)
DR = −2159
Yor1 miss 0 orf −3.7 int −1.5 rank 2455
yor1−dF miss 0 orf 1.7 int 10 rank 296
0.35 0.25 0.15 0.05 0.00
0.25 0.20 0.15 0.10 0.05 0.00
−60 −20 0 20 40 −50 0 50

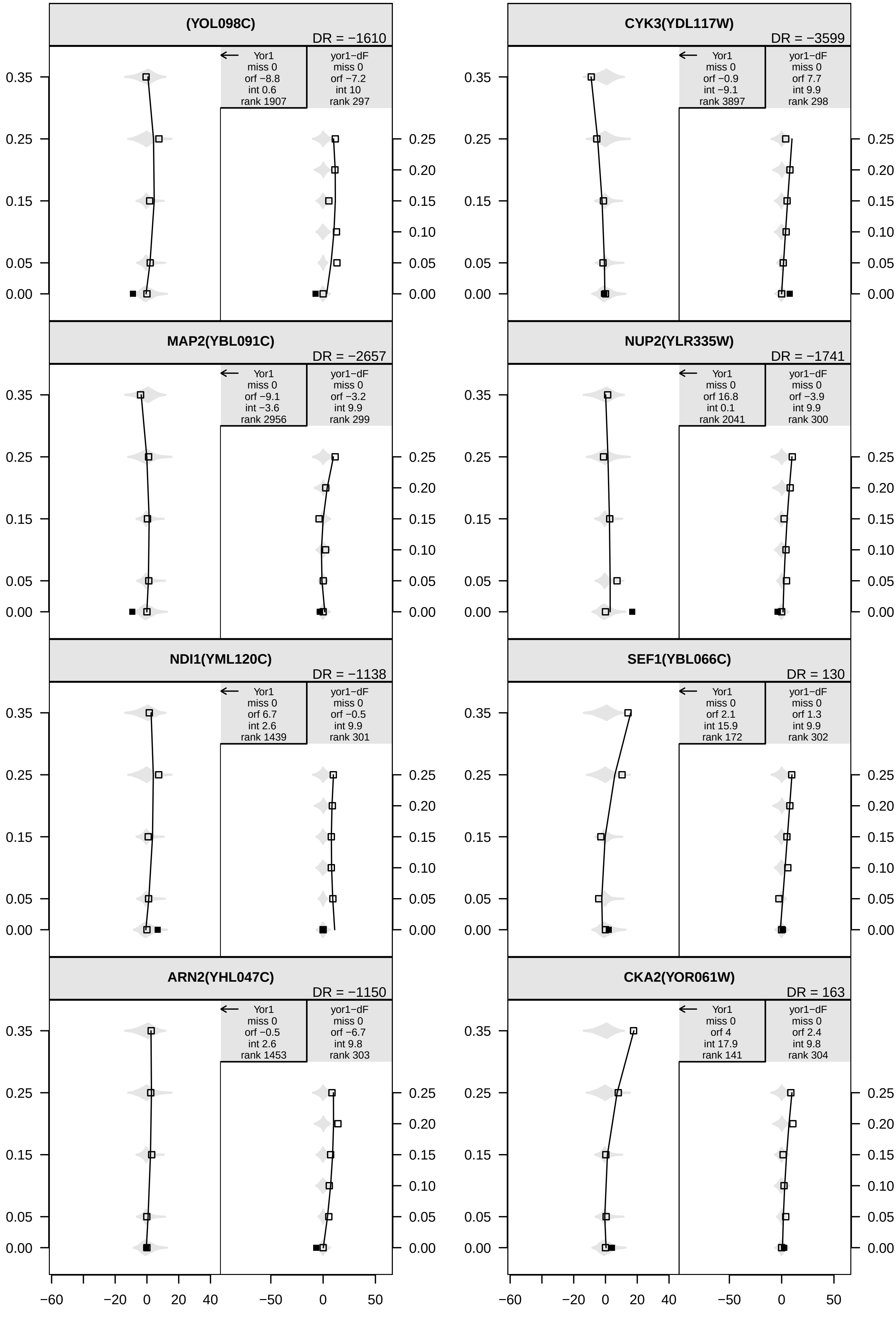

(YOL098C)
DR = −1610
Yor1 miss 0 orf −8.8 int 0.6 rank 1907
yor1−dF miss 0 orf −7.2 int 10 rank 297
CYK3(YDL117W)
DR = −3599
Yor1 miss 0 orf −0.9 int −9.1 rank 3897
yor1−dF miss 0 orf 7.7 int 9.9 rank 298
MAP2(YBL091C)
DR = −2657
Yor1 miss 0 orf −9.1 int −3.6 rank 2956
yor1−dF miss 0 orf −3.2 int 9.9 rank 299
NUP2(YLR335W)
DR = −1741
Yor1 miss 0 orf 16.8 int 0.1 rank 2041
yor1−dF miss 0 orf −3.9 int 9.9 rank 300
NDI1(YML120C)
DR = −1138
Yor1 miss 0 orf 6.7 int 2.6 rank 1439
yor1−dF miss 0 orf −0.5 int 9.9 rank 301
SEF1(YBL066C)
DR = 130
Yor1 miss 0 orf 2.1 int 15.9 rank 172
yor1−dF miss 0 orf 1.3 int 9.9 rank 302
ARN2(YHL047C)
DR = −1150
Yor1 miss 0 orf −0.5 int 2.6 rank 1453
yor1−dF miss 0 orf −6.7 int 9.8 rank 303
CKA2(YOR061W)
DR = 163
Yor1 miss 0 orf 4 int 17.9 rank 141
yor1−dF miss 0 orf 2.4 int 9.8 rank 304

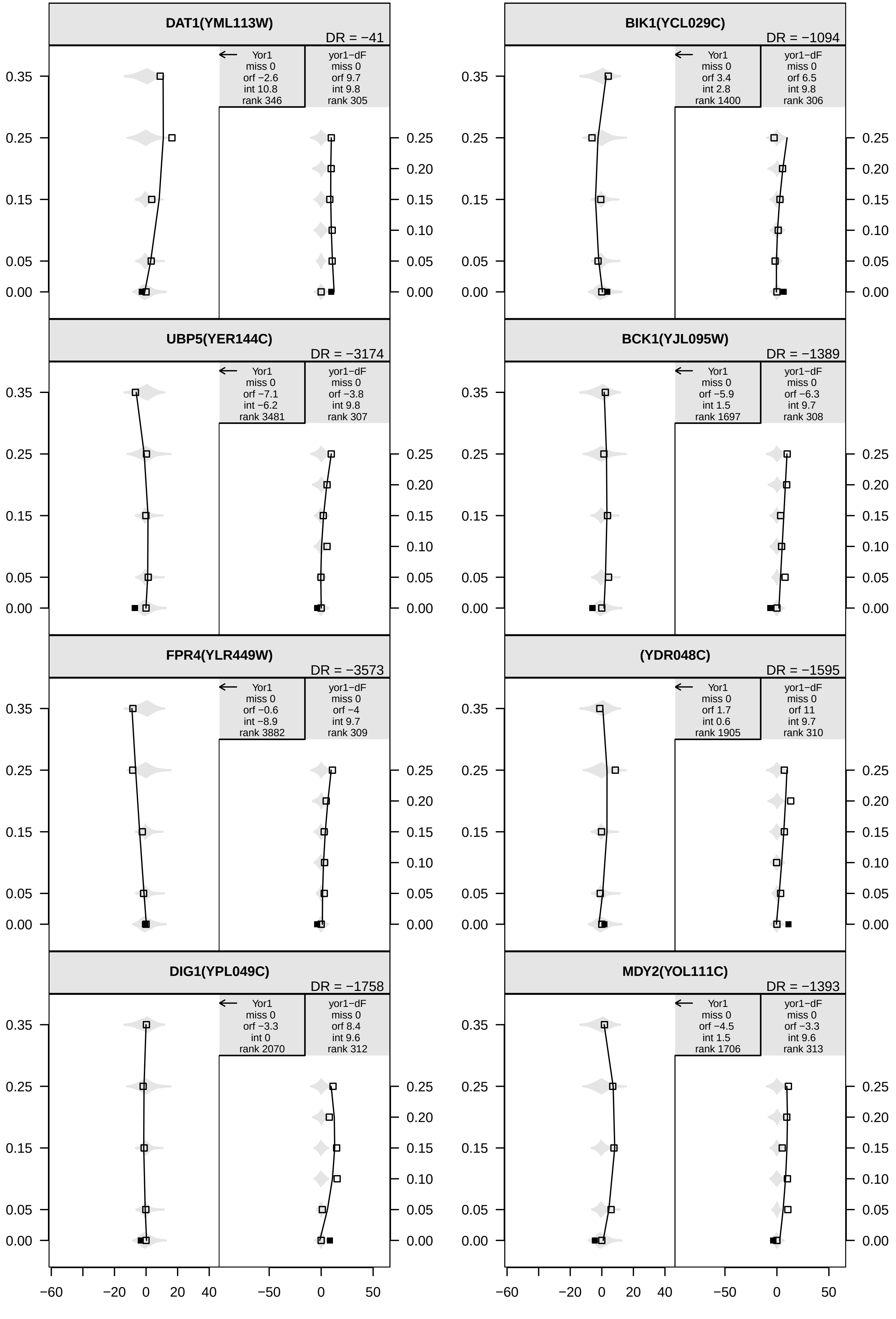

DAT1(YML113W)
DR = −41
Yor1
miss 0
orf −2.6
int 10.8
rank 346
yor1−dF
miss 0
orf 9.7
int 9.8
rank 305
BIK1(YCL029C)
DR = −1094
Yor1
miss 0
orf 3.4
int 2.8
rank 1400
yor1−dF
miss 0
orf 6.5
int 9.8
rank 306
UBP5(YER144C)
DR = −3174
Yor1
miss 0
orf −7.1
int −6.2
rank 3481
yor1−dF
miss 0
orf −3.8
int 9.8
rank 307
BCK1(YJL095W)
DR = −1389
Yor1
miss 0
orf −5.9
int 1.5
rank 1697
yor1−dF
miss 0
orf −6.3
int 9.7
rank 308
FPR4(YLR449W)
DR = −3573
Yor1
miss 0
orf −0.6
int −8.9
rank 3882
yor1−dF
miss 0
orf −4
int 9.7
rank 309
(YDR048C)
DR = −1595
Yor1
miss 0
orf 1.7
int 0.6
rank 1905
yor1−dF
miss 0
orf 11
int 9.7
rank 310
DIG1(YPL049C)
DR = −1758
Yor1
miss 0
orf −3.3
int 0
rank 2070
yor1−dF
miss 0
orf 8.4
int 9.6
rank 312
MDY2(YOL111C)
DR = −1393
Yor1
miss 0
orf −4.5
int 1.5
rank 1706
yor1−dF
miss 0
orf −3.3
int 9.6
rank 313
0.35
0.25
0.20
0.15
0.10
0.05
0.00
−60
−20
0
20
40
−50
0
50

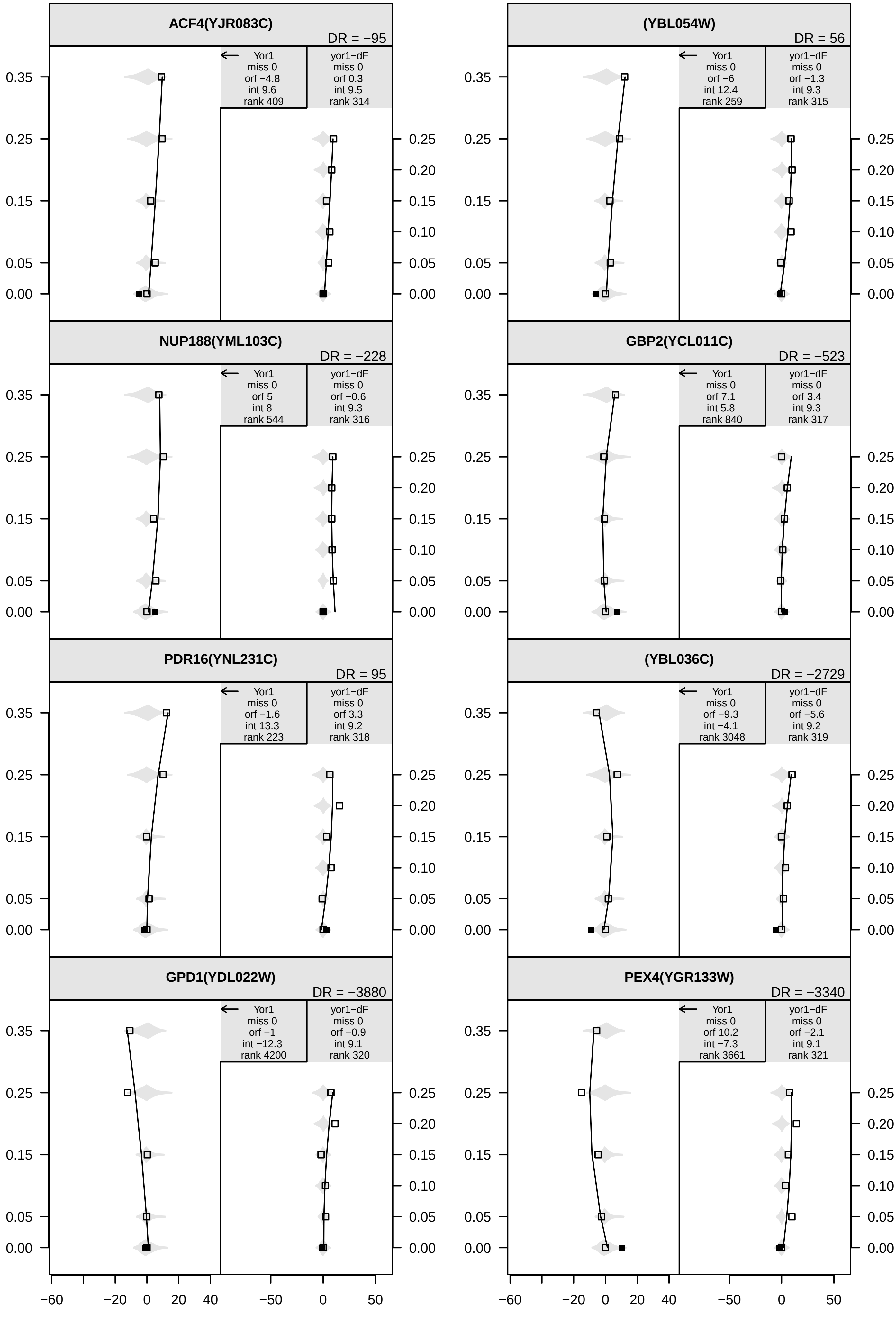
ACF4(YJR083C)
DR = −95
Yor1 miss 0 orf −4.8 int 9.6 rank 409
yor1−dF miss 0 orf 0.3 int 9.5 rank 314
(YBL054W)
DR = 56
Yor1 miss 0 orf −6 int 12.4 rank 259
yor1−dF miss 0 orf −1.3 int 9.3 rank 315
NUP188(YML103C)
DR = −228
Yor1 miss 0 orf 5 int 8 rank 544
yor1−dF miss 0 orf −0.6 int 9.3 rank 316
GBP2(YCL011C)
DR = −523
Yor1 miss 0 orf 7.1 int 5.8 rank 840
yor1−dF miss 0 orf 3.4 int 9.3 rank 317
PDR16(YNL231C)
DR = 95
Yor1 miss 0 orf −1.6 int 13.3 rank 223
yor1−dF miss 0 orf 3.3 int 9.2 rank 318
(YBL036C)
DR = −2729
Yor1 miss 0 orf −9.3 int −4.1 rank 3048
yor1−dF miss 0 orf −5.6 int 9.2 rank 319
GPD1(YDL022W)
DR = −3880
Yor1 miss 0 orf −1 int −12.3 rank 4200
yor1−dF miss 0 orf −0.9 int 9.1 rank 320
PEX4(YGR133W)
DR = −3340
Yor1 miss 0 orf 10.2 int −7.3 rank 3661
yor1−dF miss 0 orf −2.1 int 9.1 rank 321
0.00 0.05 0.10 0.15 0.20 0.25 0.35
−60 −20 0 20 40 −50 0 50

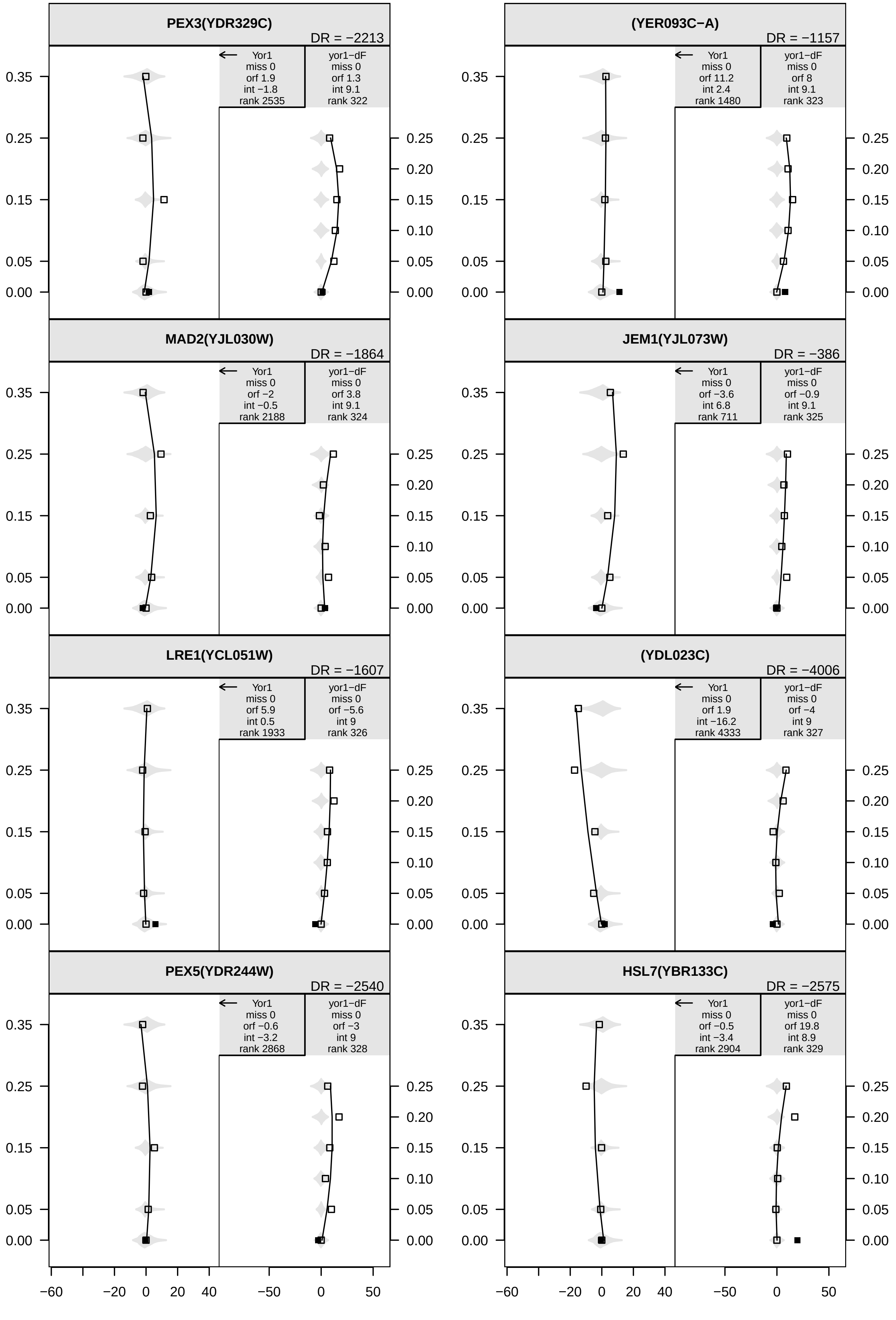
PEX3(YDR329C)
DR = −2213
Yor1 miss 0 orf 1.9 int −1.8 rank 2535
yor1−dF miss 0 orf 1.3 int 9.1 rank 322
(YER093C−A)
DR = −1157
Yor1 miss 0 orf 11.2 int 2.4 rank 1480
yor1−dF miss 0 orf 8 int 9.1 rank 323
MAD2(YJL030W)
DR = −1864
Yor1 miss 0 orf −2 int −0.5 rank 2188
yor1−dF miss 0 orf 3.8 int 9.1 rank 324
JEM1(YJL073W)
DR = −386
Yor1 miss 0 orf −3.6 int 6.8 rank 711
yor1−dF miss 0 orf −0.9 int 9.1 rank 325
LRE1(YCL051W)
DR = −1607
Yor1 miss 0 orf 5.9 int 0.5 rank 1933
yor1−dF miss 0 orf −5.6 int 9 rank 326
(YDL023C)
DR = −4006
Yor1 miss 0 orf 1.9 int −16.2 rank 4333
yor1−dF miss 0 orf −4 int 9 rank 327
PEX5(YDR244W)
DR = −2540
Yor1 miss 0 orf −0.6 int −3.2 rank 2868
yor1−dF miss 0 orf −3 int 9 rank 328
HSL7(YBR133C)
DR = −2575
Yor1 miss 0 orf −0.5 int −3.4 rank 2904
yor1−dF miss 0 orf 19.8 int 8.9 rank 329

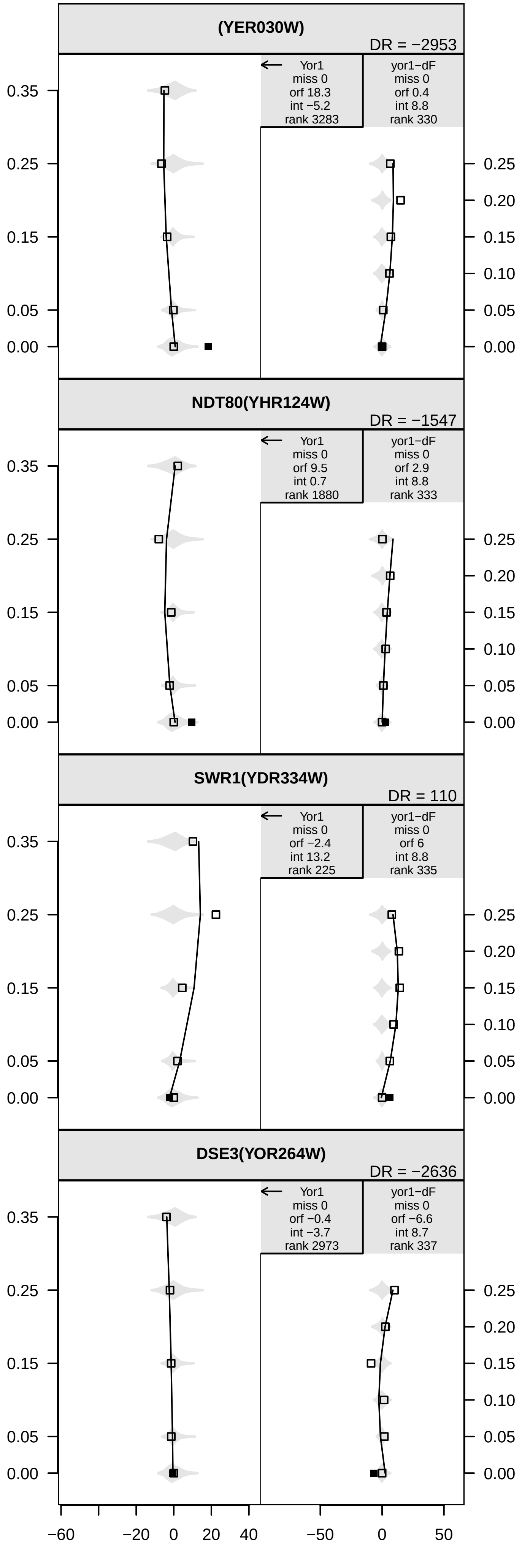

(YER030W)
DR = −2953
Yor1 miss 0 orf 18.3 int −5.2 rank 3283
yor1−dF miss 0 orf 0.4 int 8.8 rank 330
(YKL037W)
DR = −1509
Yor1 miss 0 orf 5.8 int 0.9 rank 1841
yor1−dF miss 0 orf 3.6 int 8.8 rank 332
NDT80(YHR124W)
DR = −1547
Yor1 miss 0 orf 9.5 int 0.7 rank 1880
yor1−dF miss 0 orf 2.9 int 8.8 rank 333
RPL2B(YIL018W)
DR = 146
Yor1 miss 0 orf 1 int 14.8 rank 188
yor1−dF miss 0 orf 3.7 int 8.8 rank 334
SWR1(YDR334W)
DR = 110
Yor1 miss 0 orf −2.4 int 13.2 rank 225
yor1−dF miss 0 orf 6 int 8.8 rank 335
RDS1(YCR106W)
DR = −2853
Yor1 miss 0 orf −8.4 int −4.8 rank 3189
yor1−dF miss 0 orf −5.6 int 8.8 rank 336
DSE3(YOR264W)
DR = −2636
Yor1 miss 0 orf −0.4 int −3.7 rank 2973
yor1−dF miss 0 orf −6.6 int 8.7 rank 337
PUS7(YOR243C)
DR = −863
Yor1 miss 0 orf 6.3 int 3.8 rank 1202
yor1−dF miss 0 orf 2 int 8.7 rank 339

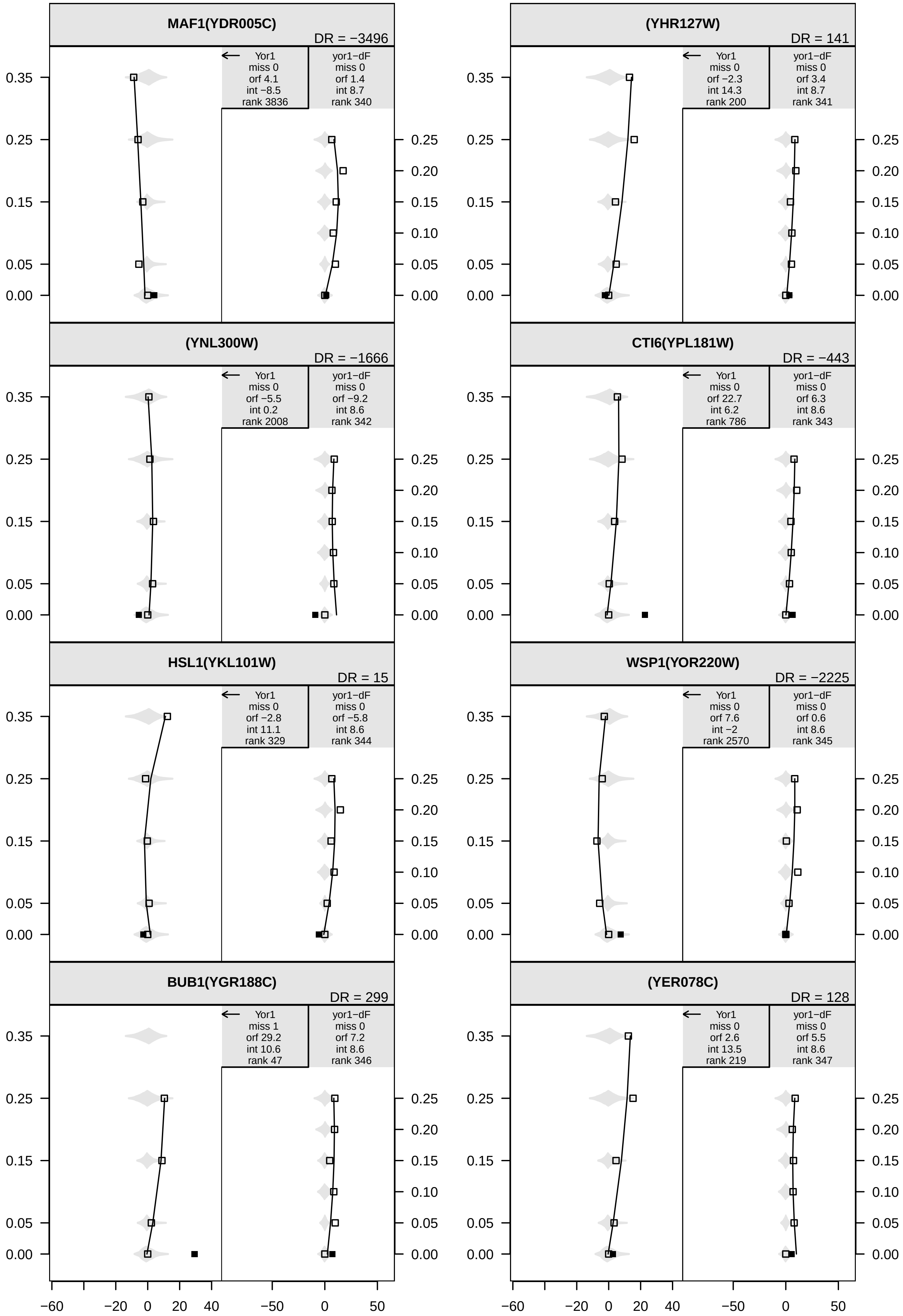
MAF1(YDR005C)
DR = −3496
Yor1 miss 0 orf 4.1 int −8.5 rank 3836
yor1−dF miss 0 orf 1.4 int 8.7 rank 340
(YHR127W)
DR = 141
Yor1 miss 0 orf −2.3 int 14.3 rank 200
yor1−dF miss 0 orf 3.4 int 8.7 rank 341
(YNL300W)
DR = −1666
Yor1 miss 0 orf −5.5 int 0.2 rank 2008
yor1−dF miss 0 orf −9.2 int 8.6 rank 342
CTI6(YPL181W)
DR = −443
Yor1 miss 0 orf 22.7 int 6.2 rank 786
yor1−dF miss 0 orf 6.3 int 8.6 rank 343
HSL1(YKL101W)
DR = 15
Yor1 miss 0 orf −2.8 int 11.1 rank 329
yor1−dF miss 0 orf −5.8 int 8.6 rank 344
WSP1(YOR220W)
DR = −2225
Yor1 miss 0 orf 7.6 int −2 rank 2570
yor1−dF miss 0 orf 0.6 int 8.6 rank 345
BUB1(YGR188C)
DR = 299
Yor1 miss 1 orf 29.2 int 10.6 rank 47
yor1−dF miss 0 orf 7.2 int 8.6 rank 346
(YER078C)
DR = 128
Yor1 miss 0 orf 2.6 int 13.5 rank 219
yor1−dF miss 0 orf 5.5 int 8.6 rank 347
0.35
0.25
0.20
0.15
0.10
0.05
0.00
−60
−20
0
20
40
−50
0
50

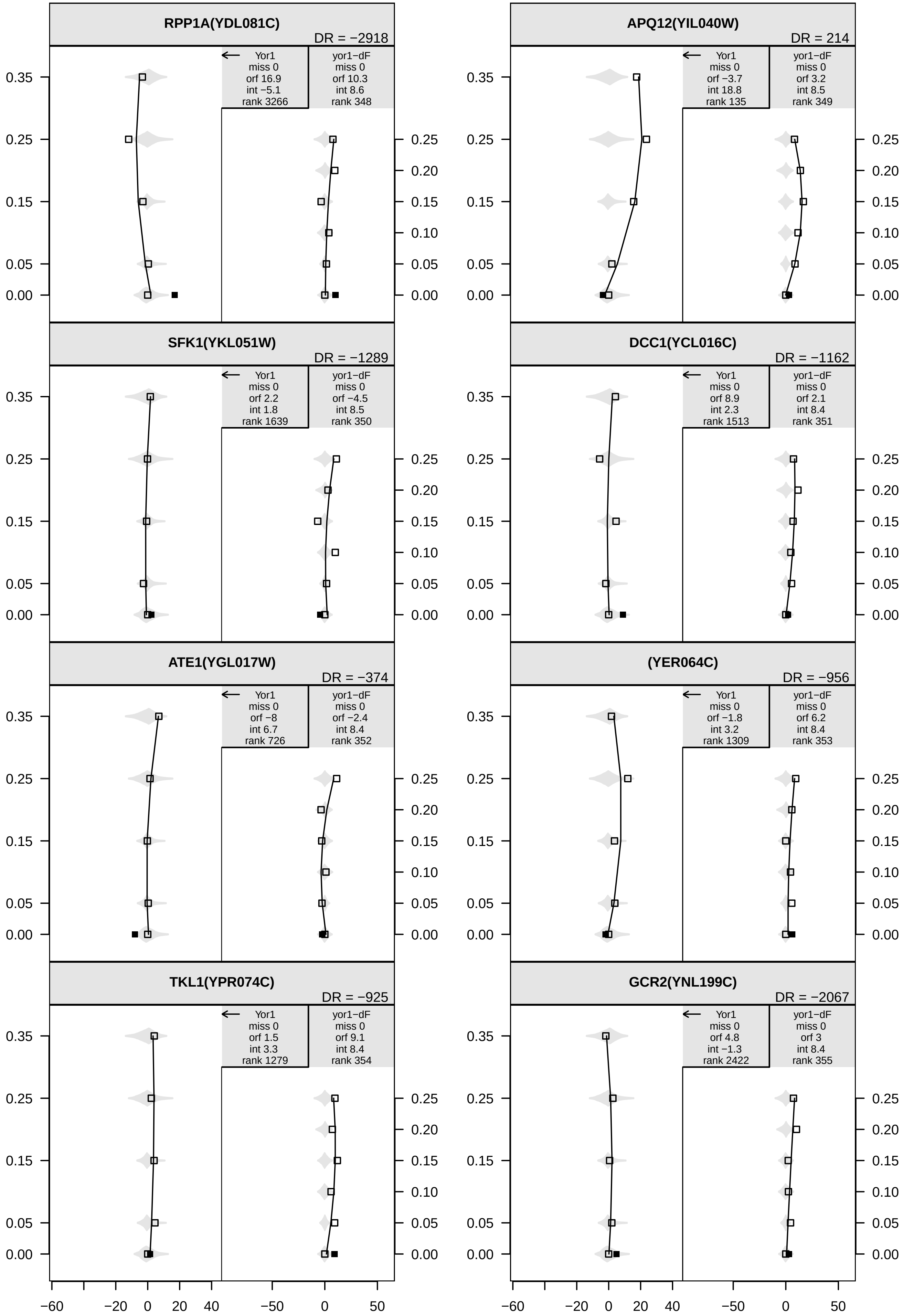

RPP1A(YDL081C)
DR = −2918
Yor1
miss 0
orf 16.9
int −5.1
rank 3266
yor1−dF
miss 0
orf 10.3
int 8.6
rank 348
APQ12(YIL040W)
DR = 214
Yor1
miss 0
orf −3.7
int 18.8
rank 135
yor1−dF
miss 0
orf 3.2
int 8.5
rank 349
SFK1(YKL051W)
DR = −1289
Yor1
miss 0
orf 2.2
int 1.8
rank 1639
yor1−dF
miss 0
orf −4.5
int 8.5
rank 350
DCC1(YCL016C)
DR = −1162
Yor1
miss 0
orf 8.9
int 2.3
rank 1513
yor1−dF
miss 0
orf 2.1
int 8.4
rank 351
ATE1(YGL017W)
DR = −374
Yor1
miss 0
orf −8
int 6.7
rank 726
yor1−dF
miss 0
orf −2.4
int 8.4
rank 352
(YER064C)
DR = −956
Yor1
miss 0
orf −1.8
int 3.2
rank 1309
yor1−dF
miss 0
orf 6.2
int 8.4
rank 353
TKL1(YPR074C)
DR = −925
Yor1
miss 0
orf 1.5
int 3.3
rank 1279
yor1−dF
miss 0
orf 9.1
int 8.4
rank 354
GCR2(YNL199C)
DR = −2067
Yor1
miss 0
orf 4.8
int −1.3
rank 2422
yor1−dF
miss 0
orf 3
int 8.4
rank 355
0.35
0.25
0.20
0.15
0.10
0.05
0.00
−60
−20
0
20
40
−50
0
50

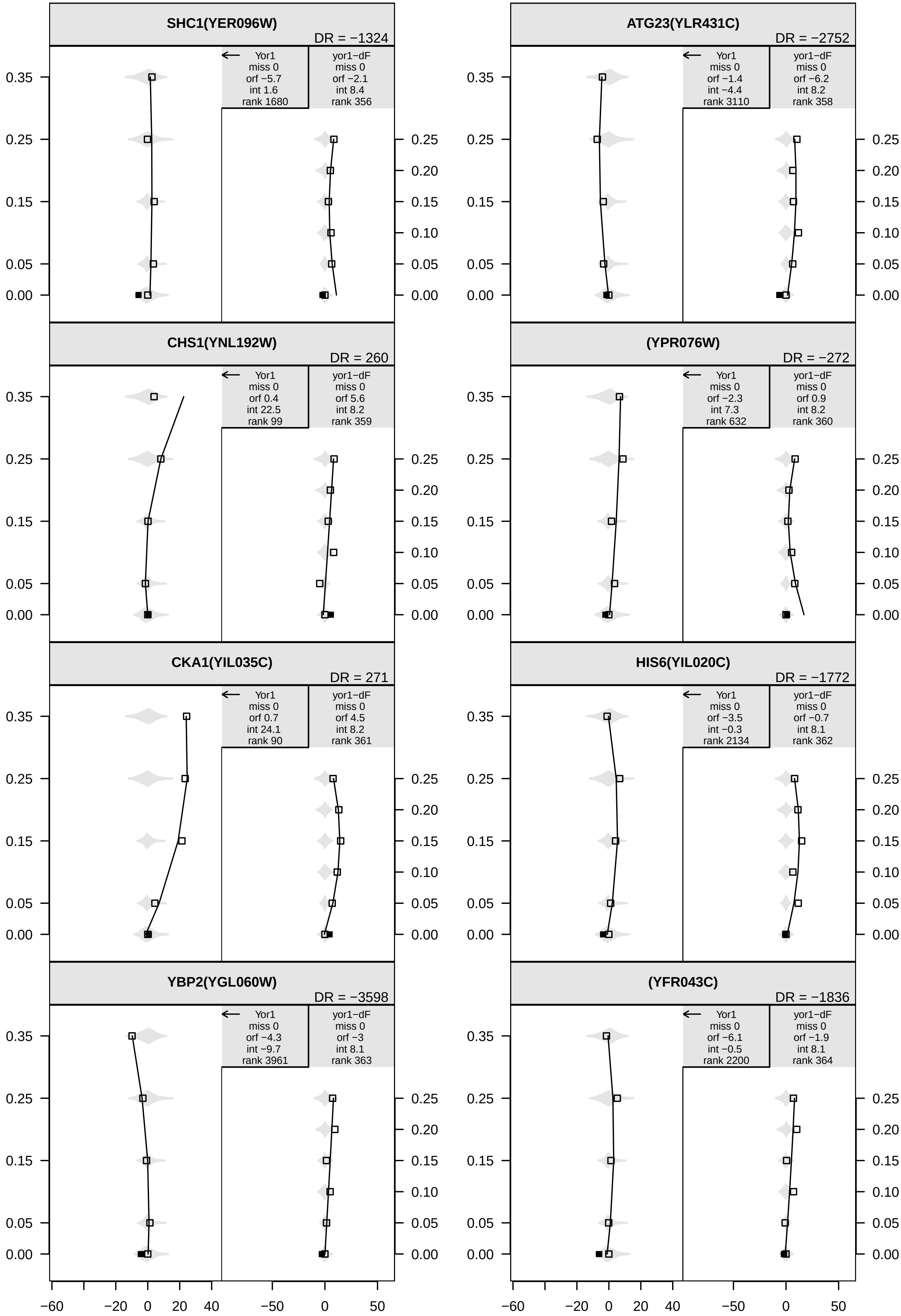

SHC1(YER096W)
DR = −1324
Yor1 miss 0 orf −5.7 int 1.6 rank 1680
yor1−dF miss 0 orf −2.1 int 8.4 rank 356
ATG23(YLR431C)
DR = −2752
Yor1 miss 0 orf −1.4 int −4.4 rank 3110
yor1−dF miss 0 orf −6.2 int 8.2 rank 358
CHS1(YNL192W)
DR = 260
Yor1 miss 0 orf 0.4 int 22.5 rank 99
yor1−dF miss 0 orf 5.6 int 8.2 rank 359
(YPR076W)
DR = −272
Yor1 miss 0 orf −2.3 int 7.3 rank 632
yor1−dF miss 0 orf 0.9 int 8.2 rank 360
CKA1(YIL035C)
DR = 271
Yor1 miss 0 orf 0.7 int 24.1 rank 90
yor1−dF miss 0 orf 4.5 int 8.2 rank 361
HIS6(YIL020C)
DR = −1772
Yor1 miss 0 orf −3.5 int −0.3 rank 2134
yor1−dF miss 0 orf −0.7 int 8.1 rank 362
YBP2(YGL060W)
DR = −3598
Yor1 miss 0 orf −4.3 int −9.7 rank 3961
yor1−dF miss 0 orf −3 int 8.1 rank 363
(YFR043C)
DR = −1836
Yor1 miss 0 orf −6.1 int −0.5 rank 2200
yor1−dF miss 0 orf −1.9 int 8.1 rank 364
0.00 0.05 0.10 0.15 0.20 0.25 0.35
−60 −20 0 20 40
−50 0 50

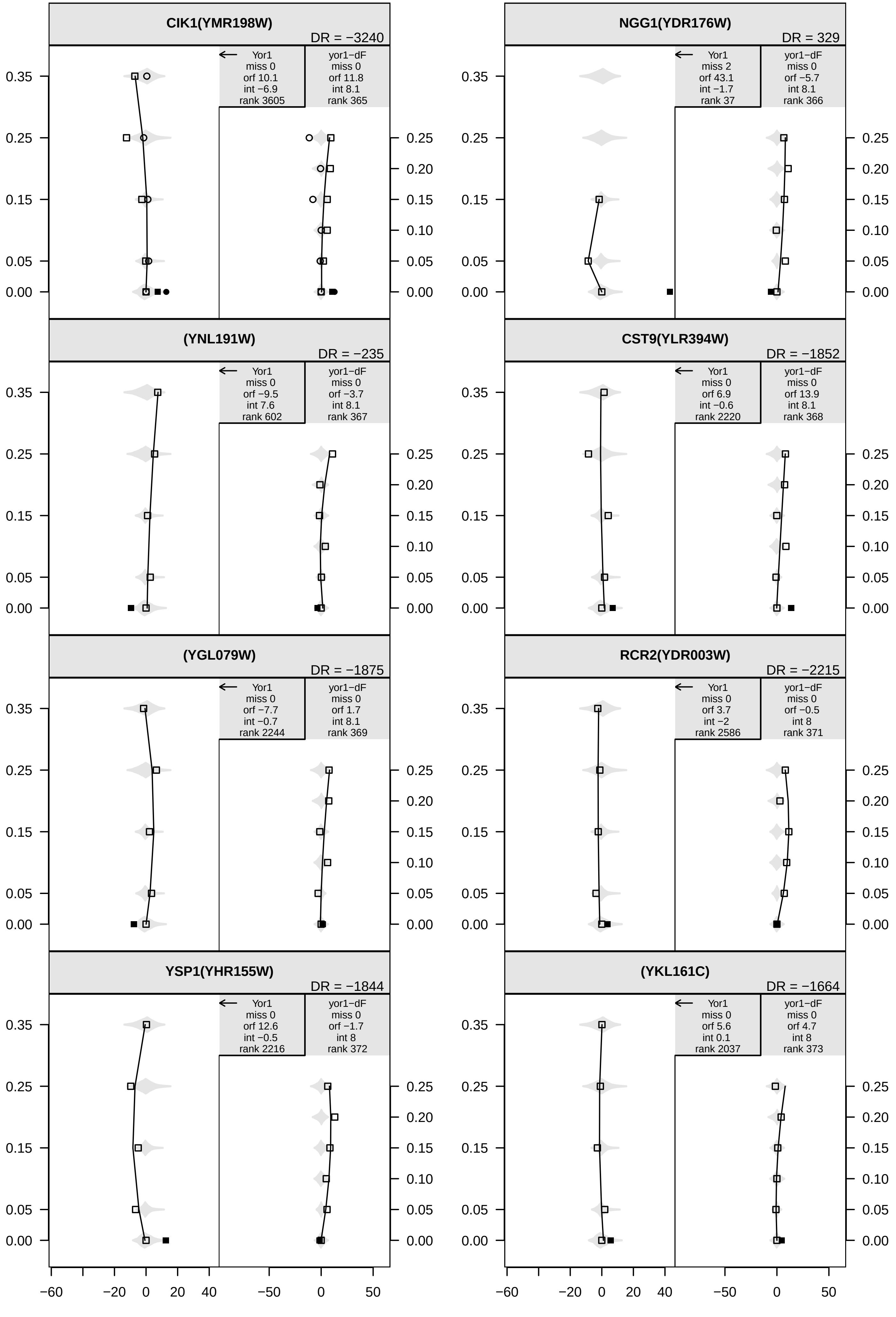

CIK1(YMR198W)
DR = −3240
Yor1 miss 0 orf 10.1 int −6.9 rank 3605
yor1−dF miss 0 orf 11.8 int 8.1 rank 365
NGG1(YDR176W)
DR = 329
Yor1 miss 2 orf 43.1 int −1.7 rank 37
yor1−dF miss 0 orf −5.7 int 8.1 rank 366
(YNL191W)
DR = −235
Yor1 miss 0 orf −9.5 int 7.6 rank 602
yor1−dF miss 0 orf −3.7 int 8.1 rank 367
CST9(YLR394W)
DR = −1852
Yor1 miss 0 orf 6.9 int −0.6 rank 2220
yor1−dF miss 0 orf 13.9 int 8.1 rank 368
(YGL079W)
DR = −1875
Yor1 miss 0 orf −7.7 int −0.7 rank 2244
yor1−dF miss 0 orf 1.7 int 8.1 rank 369
RCR2(YDR003W)
DR = −2215
Yor1 miss 0 orf 3.7 int −2 rank 2586
yor1−dF miss 0 orf −0.5 int 8 rank 371
YSP1(YHR155W)
DR = −1844
Yor1 miss 0 orf 12.6 int −0.5 rank 2216
yor1−dF miss 0 orf −1.7 int 8 rank 372
(YKL161C)
DR = −1664
Yor1 miss 0 orf 5.6 int 0.1 rank 2037
yor1−dF miss 0 orf 4.7 int 8 rank 373
0.35
0.25
0.20
0.15
0.10
0.05
0.00
−60
−20
0
20
40
−50
0
50

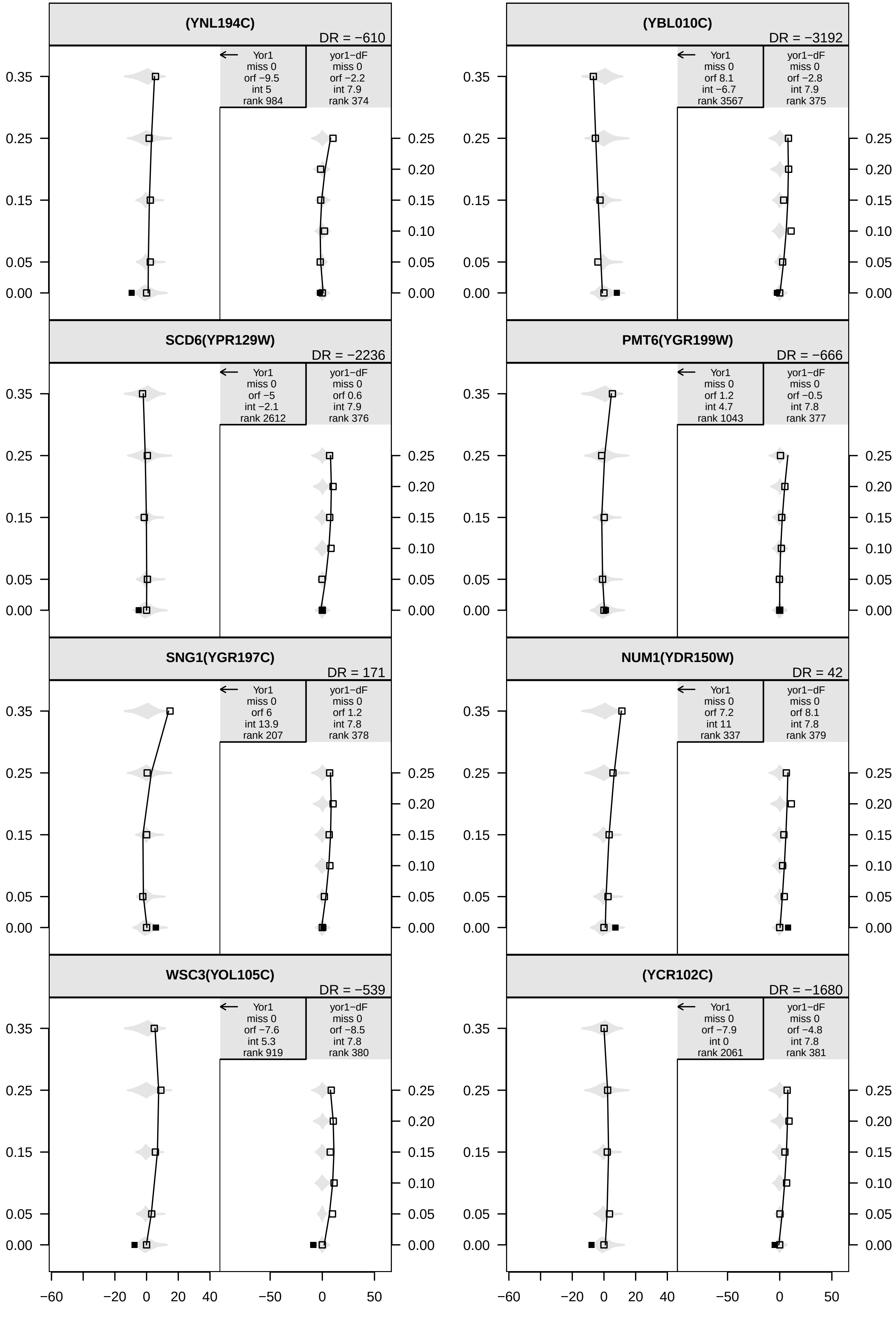

(YNL194C)
DR = −610
Yor1
miss 0
orf −9.5
int 5
rank 984
yor1−dF
miss 0
orf −2.2
int 7.9
rank 374
(YBL010C)
DR = −3192
Yor1
miss 0
orf 8.1
int −6.7
rank 3567
yor1−dF
miss 0
orf −2.8
int 7.9
rank 375
SCD6(YPR129W)
DR = −2236
Yor1
miss 0
orf −5
int −2.1
rank 2612
yor1−dF
miss 0
orf 0.6
int 7.9
rank 376
PMT6(YGR199W)
DR = −666
Yor1
miss 0
orf 1.2
int 4.7
rank 1043
yor1−dF
miss 0
orf −0.5
int 7.8
rank 377
SNG1(YGR197C)
DR = 171
Yor1
miss 0
orf 6
int 13.9
rank 207
yor1−dF
miss 0
orf 1.2
int 7.8
rank 378
NUM1(YDR150W)
DR = 42
Yor1
miss 0
orf 7.2
int 11
rank 337
yor1−dF
miss 0
orf 8.1
int 7.8
rank 379
WSC3(YOL105C)
DR = −539
Yor1
miss 0
orf −7.6
int 5.3
rank 919
yor1−dF
miss 0
orf −8.5
int 7.8
rank 380
(YCR102C)
DR = −1680
Yor1
miss 0
orf −7.9
int 0
rank 2061
yor1−dF
miss 0
orf −4.8
int 7.8
rank 381
0.35
0.25
0.20
0.15
0.10
0.05
0.00
−60
−20
0
20
40
−50
0
50

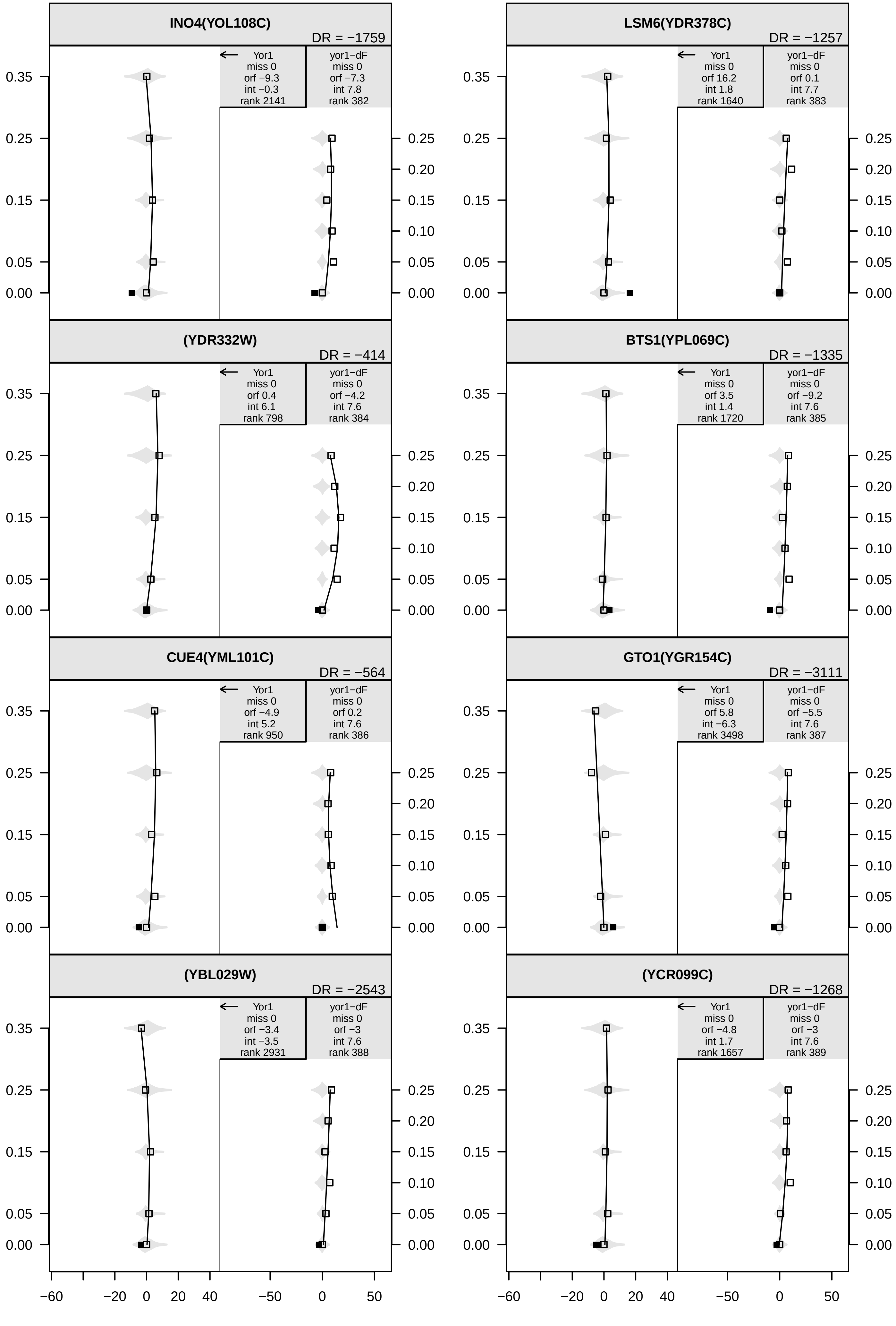

INO4(YOL108C)
DR = −1759
Yor1 miss 0 orf −9.3 int −0.3 rank 2141
yor1−dF miss 0 orf −7.3 int 7.8 rank 382
LSM6(YDR378C)
DR = −1257
Yor1 miss 0 orf 16.2 int 1.8 rank 1640
yor1−dF miss 0 orf 0.1 int 7.7 rank 383
(YDR332W)
DR = −414
Yor1 miss 0 orf 0.4 int 6.1 rank 798
yor1−dF miss 0 orf −4.2 int 7.6 rank 384
BTS1(YPL069C)
DR = −1335
Yor1 miss 0 orf 3.5 int 1.4 rank 1720
yor1−dF miss 0 orf −9.2 int 7.6 rank 385
CUE4(YML101C)
DR = −564
Yor1 miss 0 orf −4.9 int 5.2 rank 950
yor1−dF miss 0 orf 0.2 int 7.6 rank 386
GTO1(YGR154C)
DR = −3111
Yor1 miss 0 orf 5.8 int −6.3 rank 3498
yor1−dF miss 0 orf −5.5 int 7.6 rank 387
(YBL029W)
DR = −2543
Yor1 miss 0 orf −3.4 int −3.5 rank 2931
yor1−dF miss 0 orf −3 int 7.6 rank 388
(YCR099C)
DR = −1268
Yor1 miss 0 orf −4.8 int 1.7 rank 1657
yor1−dF miss 0 orf −3 int 7.6 rank 389
0.35 0.25 0.15 0.05 0.00
0.25 0.20 0.15 0.10 0.05 0.00
−60 −20 0 20 40
−50 0 50

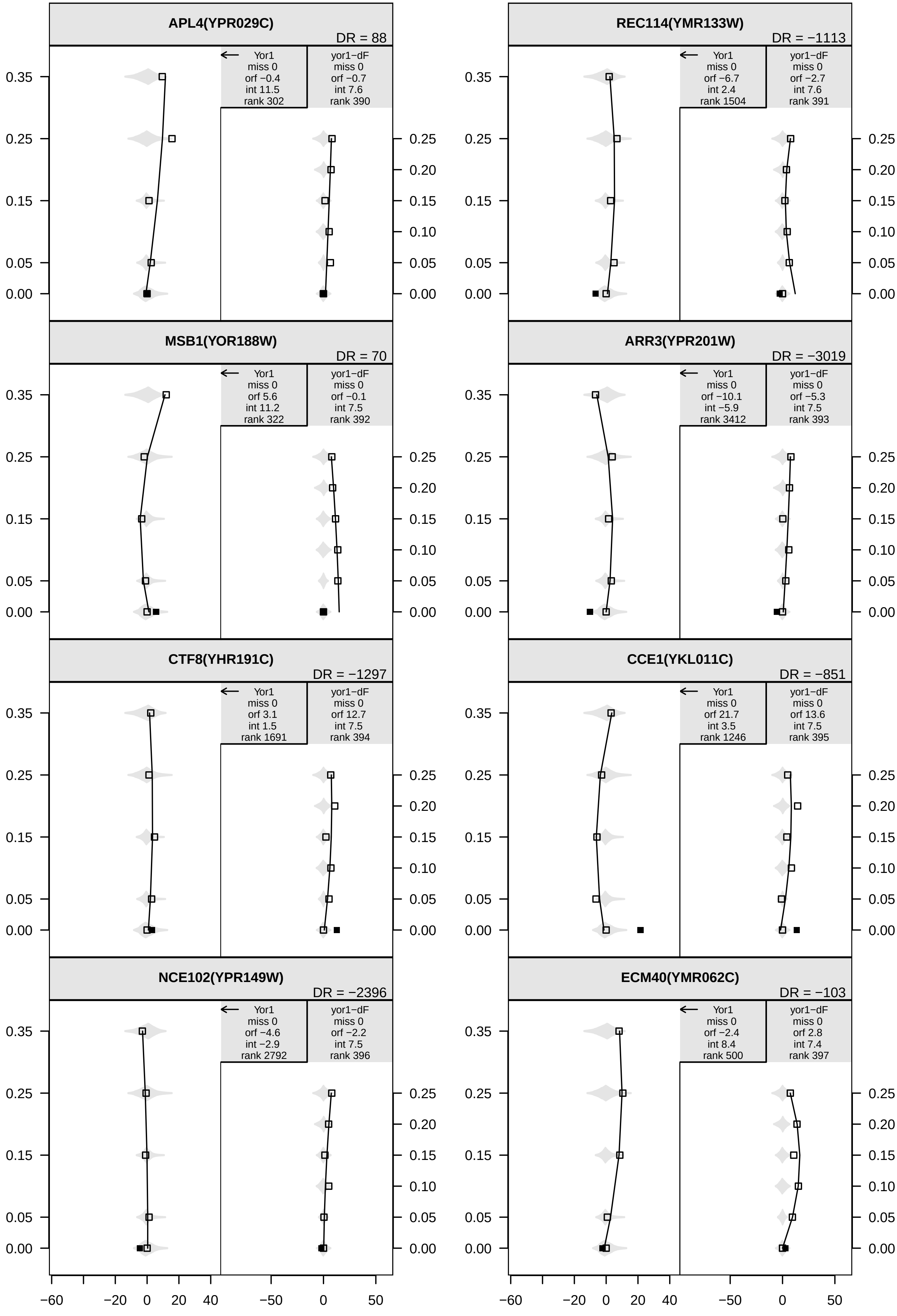
APL4(YPR029C)
DR = 88
Yor1 miss 0 orf −0.4 int 11.5 rank 302
yor1−dF miss 0 orf −0.7 int 7.6 rank 390
REC114(YMR133W)
DR = −1113
Yor1 miss 0 orf −6.7 int 2.4 rank 1504
yor1−dF miss 0 orf −2.7 int 7.6 rank 391
MSB1(YOR188W)
DR = 70
Yor1 miss 0 orf 5.6 int 11.2 rank 322
yor1−dF miss 0 orf −0.1 int 7.5 rank 392
ARR3(YPR201W)
DR = −3019
Yor1 miss 0 orf −10.1 int −5.9 rank 3412
yor1−dF miss 0 orf −5.3 int 7.5 rank 393
CTF8(YHR191C)
DR = −1297
Yor1 miss 0 orf 3.1 int 1.5 rank 1691
yor1−dF miss 0 orf 12.7 int 7.5 rank 394
CCE1(YKL011C)
DR = −851
Yor1 miss 0 orf 21.7 int 3.5 rank 1246
yor1−dF miss 0 orf 13.6 int 7.5 rank 395
NCE102(YPR149W)
DR = −2396
Yor1 miss 0 orf −4.6 int −2.9 rank 2792
yor1−dF miss 0 orf −2.2 int 7.5 rank 396
ECM40(YMR062C)
DR = −103
Yor1 miss 0 orf −2.4 int 8.4 rank 500
yor1−dF miss 0 orf 2.8 int 7.4 rank 397
0.35 0.25 0.20 0.15 0.10 0.05 0.00
−60 −20 0 20 40 −50 0 50

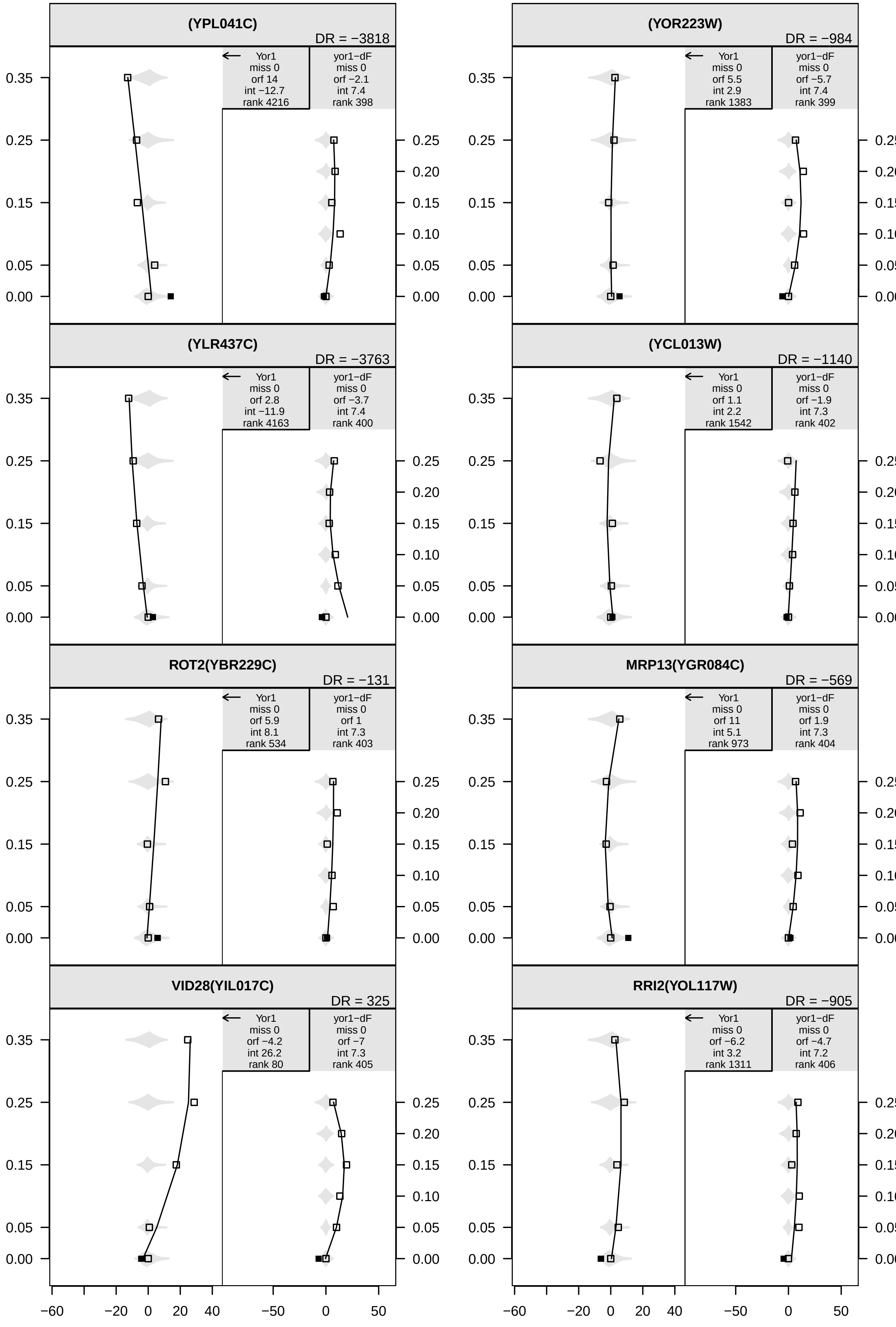
(YPL041C)
DR = −3818
Yor1 miss 0 orf 14 int −12.7 rank 4216
yor1−dF miss 0 orf −2.1 int 7.4 rank 398
(YOR223W)
DR = −984
Yor1 miss 0 orf 5.5 int 2.9 rank 1383
yor1−dF miss 0 orf −5.7 int 7.4 rank 399
(YLR437C)
DR = −3763
Yor1 miss 0 orf 2.8 int −11.9 rank 4163
yor1−dF miss 0 orf −3.7 int 7.4 rank 400
(YCL013W)
DR = −1140
Yor1 miss 0 orf 1.1 int 2.2 rank 1542
yor1−dF miss 0 orf −1.9 int 7.3 rank 402
ROT2(YBR229C)
DR = −131
Yor1 miss 0 orf 5.9 int 8.1 rank 534
yor1−dF miss 0 orf 1 int 7.3 rank 403
MRP13(YGR084C)
DR = −569
Yor1 miss 0 orf 11 int 5.1 rank 973
yor1−dF miss 0 orf 1.9 int 7.3 rank 404
VID28(YIL017C)
DR = 325
Yor1 miss 0 orf −4.2 int 26.2 rank 80
yor1−dF miss 0 orf −7 int 7.3 rank 405
RRI2(YOL117W)
DR = −905
Yor1 miss 0 orf −6.2 int 3.2 rank 1311
yor1−dF miss 0 orf −4.7 int 7.2 rank 406
0.35 0.25 0.20 0.15 0.10 0.05 0.00
−60 −20 0 20 40 −50 0 50

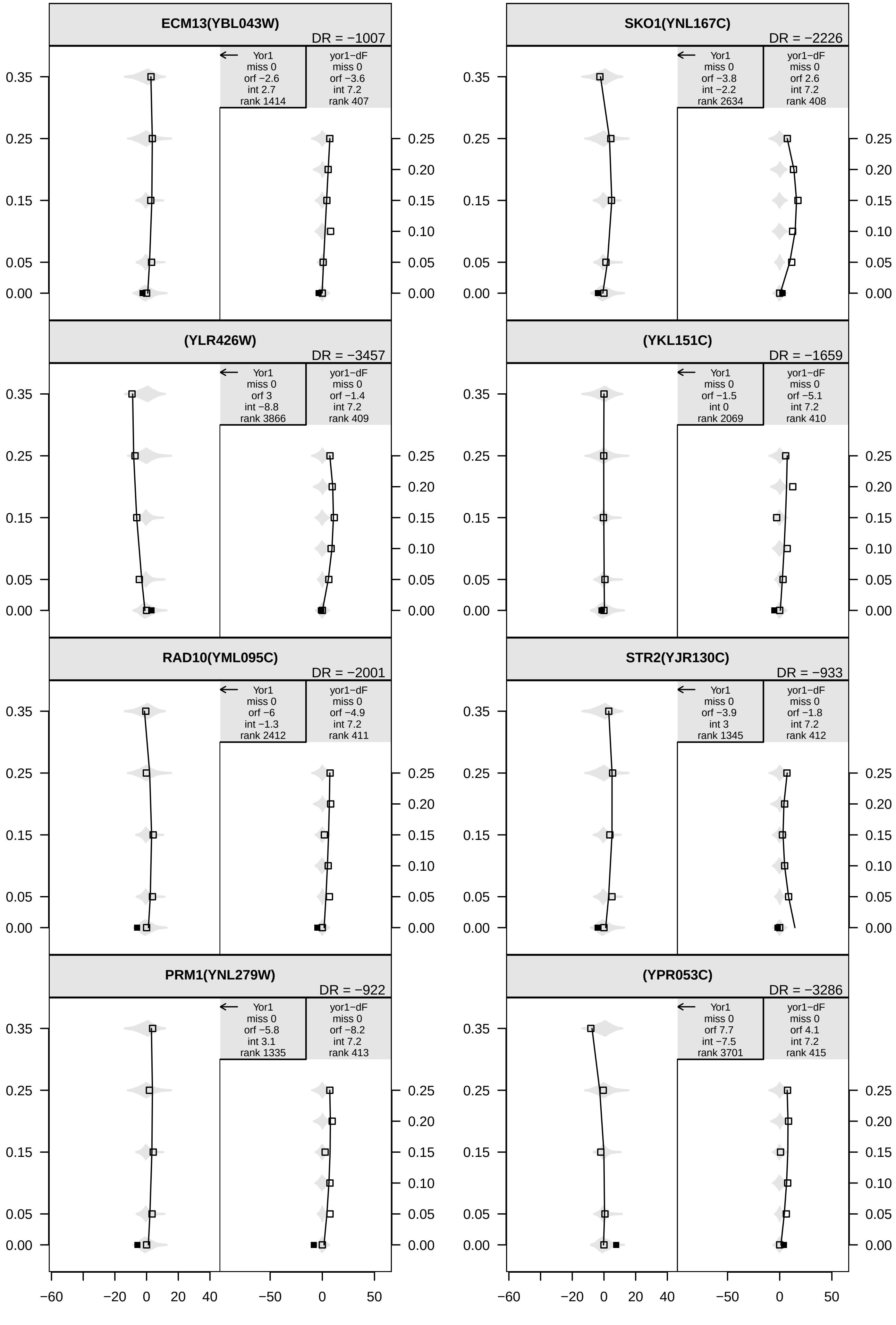
ECM13(YBL043W)
DR = −1007
Yor1 miss 0 orf −2.6 int 2.7 rank 1414
yor1−dF miss 0 orf −3.6 int 7.2 rank 407
SKO1(YNL167C)
DR = −2226
Yor1 miss 0 orf −3.8 int −2.2 rank 2634
yor1−dF miss 0 orf 2.6 int 7.2 rank 408
(YLR426W)
DR = −3457
Yor1 miss 0 orf 3 int −8.8 rank 3866
yor1−dF miss 0 orf −1.4 int 7.2 rank 409
(YKL151C)
DR = −1659
Yor1 miss 0 orf −1.5 int 0 rank 2069
yor1−dF miss 0 orf −5.1 int 7.2 rank 410
RAD10(YML095C)
DR = −2001
Yor1 miss 0 orf −6 int −1.3 rank 2412
yor1−dF miss 0 orf −4.9 int 7.2 rank 411
STR2(YJR130C)
DR = −933
Yor1 miss 0 orf −3.9 int 3 rank 1345
yor1−dF miss 0 orf −1.8 int 7.2 rank 412
PRM1(YNL279W)
DR = −922
Yor1 miss 0 orf −5.8 int 3.1 rank 1335
yor1−dF miss 0 orf −8.2 int 7.2 rank 413
(YPR053C)
DR = −3286
Yor1 miss 0 orf 7.7 int −7.5 rank 3701
yor1−dF miss 0 orf 4.1 int 7.2 rank 415
0.35
0.25
0.20
0.15
0.10
0.05
0.00
−60
−20
0
20
40
−50
0
50

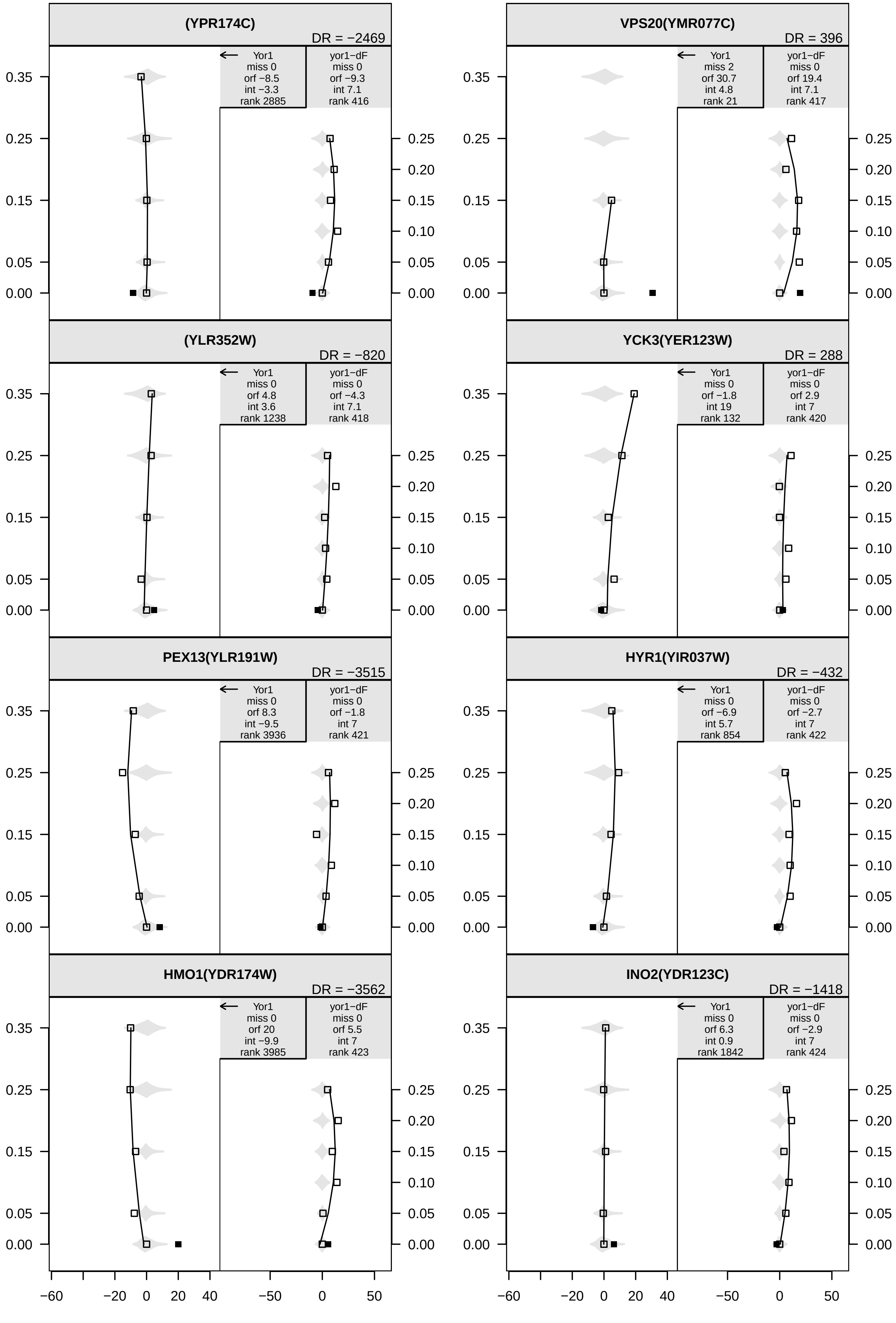
(YPR174C)
DR = −2469
Yor1 miss 0 orf −8.5 int −3.3 rank 2885
yor1−dF miss 0 orf −9.3 int 7.1 rank 416
VPS20(YMR077C)
DR = 396
Yor1 miss 2 orf 30.7 int 4.8 rank 21
yor1−dF miss 0 orf 19.4 int 7.1 rank 417
(YLR352W)
DR = −820
Yor1 miss 0 orf 4.8 int 3.6 rank 1238
yor1−dF miss 0 orf −4.3 int 7.1 rank 418
YCK3(YER123W)
DR = 288
Yor1 miss 0 orf −1.8 int 19 rank 132
yor1−dF miss 0 orf 2.9 int 7 rank 420
PEX13(YLR191W)
DR = −3515
Yor1 miss 0 orf 8.3 int −9.5 rank 3936
yor1−dF miss 0 orf −1.8 int 7 rank 421
HYR1(YIR037W)
DR = −432
Yor1 miss 0 orf −6.9 int 5.7 rank 854
yor1−dF miss 0 orf −2.7 int 7 rank 422
HMO1(YDR174W)
DR = −3562
Yor1 miss 0 orf 20 int −9.9 rank 3985
yor1−dF miss 0 orf 5.5 int 7 rank 423
INO2(YDR123C)
DR = −1418
Yor1 miss 0 orf 6.3 int 0.9 rank 1842
yor1−dF miss 0 orf −2.9 int 7 rank 424
0.00 0.05 0.10 0.15 0.20 0.25 0.35
−60 −20 0 20 40 −50 0 50

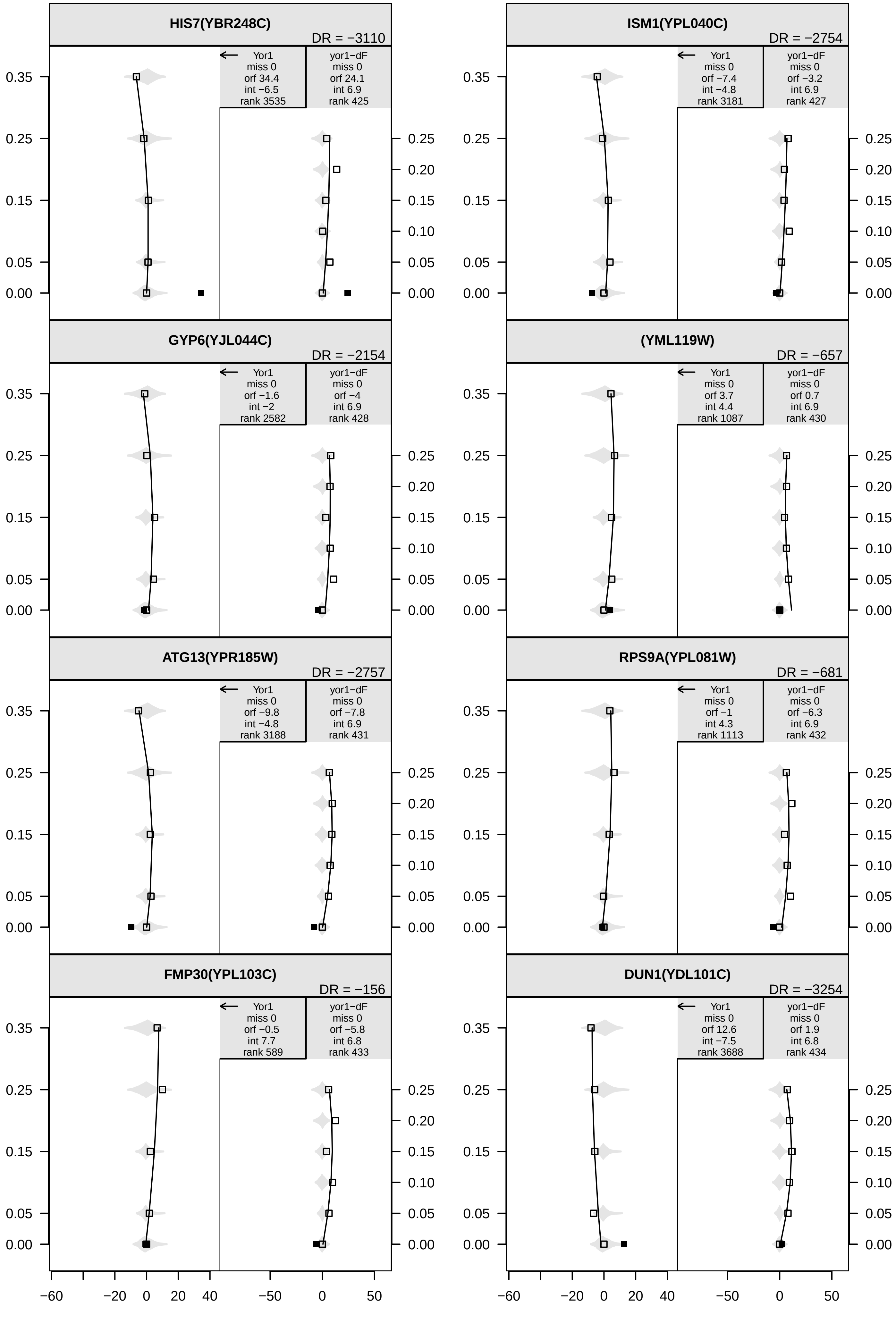
HIS7(YBR248C)
DR = −3110
Yor1
miss 0
orf 34.4
int −6.5
rank 3535
yor1−dF
miss 0
orf 24.1
int 6.9
rank 425
ISM1(YPL040C)
DR = −2754
Yor1
miss 0
orf −7.4
int −4.8
rank 3181
yor1−dF
miss 0
orf −3.2
int 6.9
rank 427
GYP6(YJL044C)
DR = −2154
Yor1
miss 0
orf −1.6
int −2
rank 2582
yor1−dF
miss 0
orf −4
int 6.9
rank 428
(YML119W)
DR = −657
Yor1
miss 0
orf 3.7
int 4.4
rank 1087
yor1−dF
miss 0
orf 0.7
int 6.9
rank 430
ATG13(YPR185W)
DR = −2757
Yor1
miss 0
orf −9.8
int −4.8
rank 3188
yor1−dF
miss 0
orf −7.8
int 6.9
rank 431
RPS9A(YPL081W)
DR = −681
Yor1
miss 0
orf −1
int 4.3
rank 1113
yor1−dF
miss 0
orf −6.3
int 6.9
rank 432
FMP30(YPL103C)
DR = −156
Yor1
miss 0
orf −0.5
int 7.7
rank 589
yor1−dF
miss 0
orf −5.8
int 6.8
rank 433
DUN1(YDL101C)
DR = −3254
Yor1
miss 0
orf 12.6
int −7.5
rank 3688
yor1−dF
miss 0
orf 1.9
int 6.8
rank 434
0.35
0.25
0.20
0.15
0.10
0.05
0.00
−60
−20
0
20
40
−50
0
50

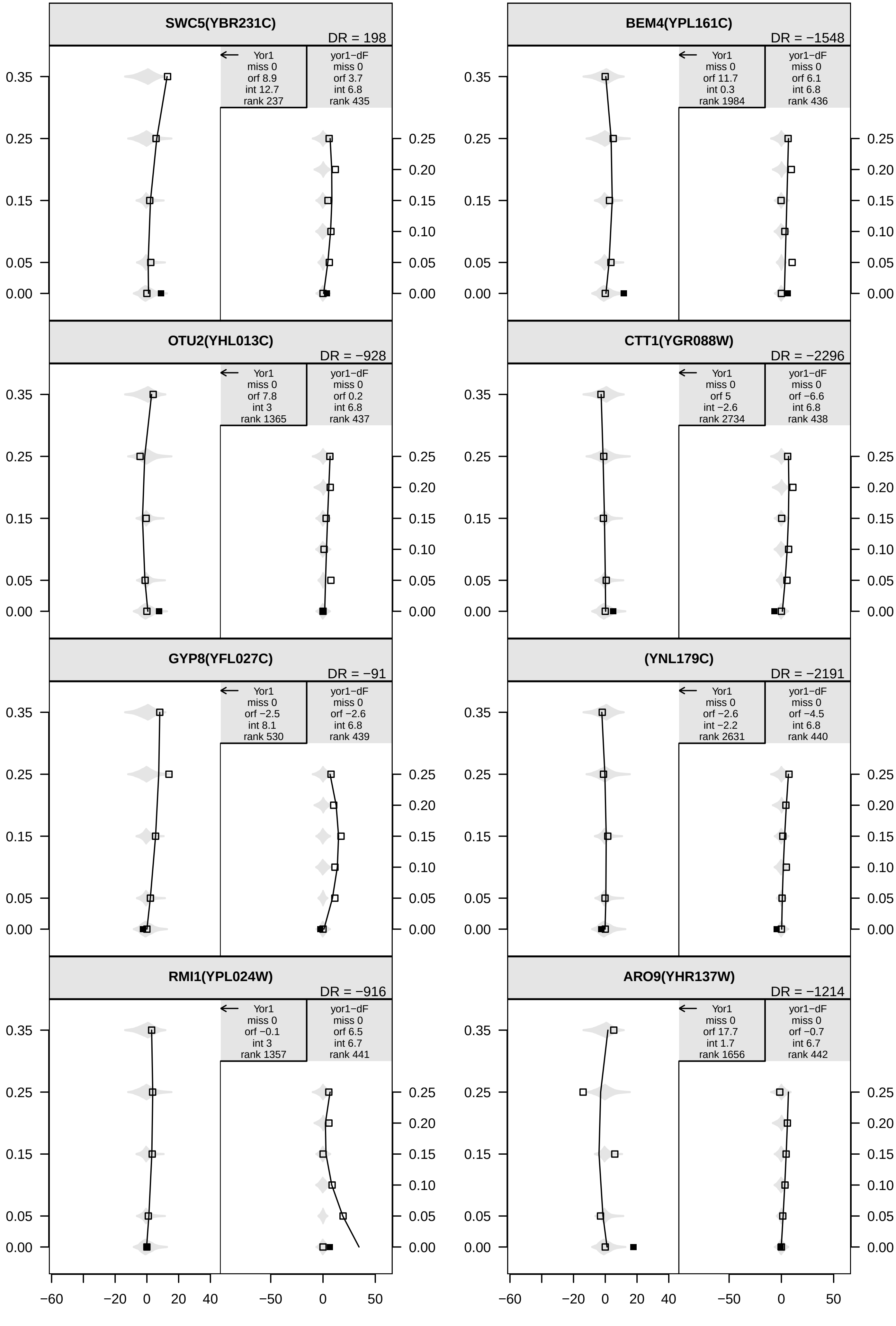
SWC5(YBR231C)
DR = 198
Yor1 miss 0 orf 8.9 int 12.7 rank 237
yor1−dF miss 0 orf 3.7 int 6.8 rank 435
BEM4(YPL161C)
DR = −1548
Yor1 miss 0 orf 11.7 int 0.3 rank 1984
yor1−dF miss 0 orf 6.1 int 6.8 rank 436
OTU2(YHL013C)
DR = −928
Yor1 miss 0 orf 7.8 int 3 rank 1365
yor1−dF miss 0 orf 0.2 int 6.8 rank 437
CTT1(YGR088W)
DR = −2296
Yor1 miss 0 orf 5 int −2.6 rank 2734
yor1−dF miss 0 orf −6.6 int 6.8 rank 438
GYP8(YFL027C)
DR = −91
Yor1 miss 0 orf −2.5 int 8.1 rank 530
yor1−dF miss 0 orf −2.6 int 6.8 rank 439
(YNL179C)
DR = −2191
Yor1 miss 0 orf −2.6 int −2.2 rank 2631
yor1−dF miss 0 orf −4.5 int 6.8 rank 440
RMI1(YPL024W)
DR = −916
Yor1 miss 0 orf −0.1 int 3 rank 1357
yor1−dF miss 0 orf 6.5 int 6.7 rank 441
ARO9(YHR137W)
DR = −1214
Yor1 miss 0 orf 17.7 int 1.7 rank 1656
yor1−dF miss 0 orf −0.7 int 6.7 rank 442

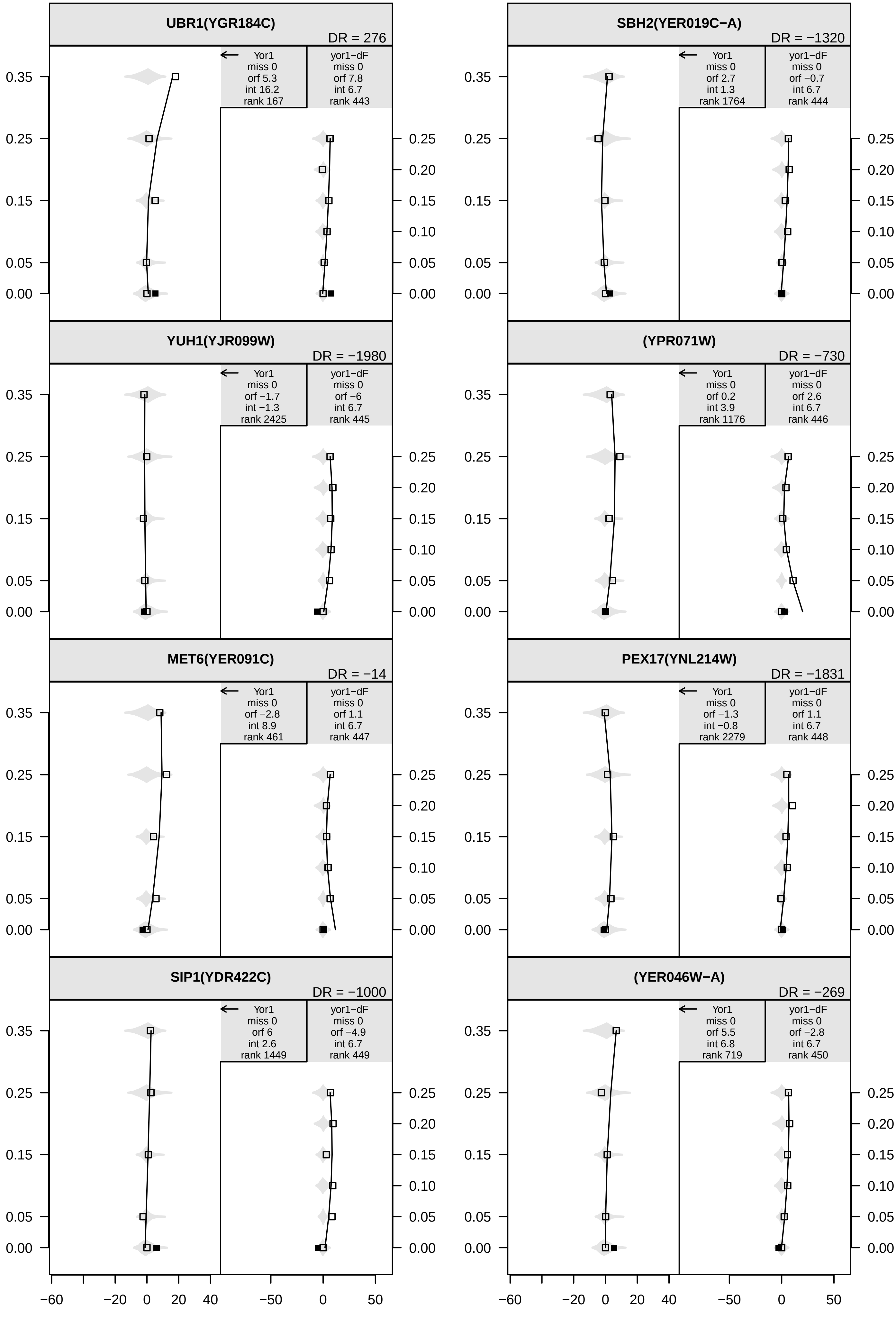

UBR1(YGR184C)
DR = 276
Yor1
miss 0
orf 5.3
int 16.2
rank 167
yor1−dF
miss 0
orf 7.8
int 6.7
rank 443
SBH2(YER019C−A)
DR = −1320
Yor1
miss 0
orf 2.7
int 1.3
rank 1764
yor1−dF
miss 0
orf −0.7
int 6.7
rank 444
YUH1(YJR099W)
DR = −1980
Yor1
miss 0
orf −1.7
int −1.3
rank 2425
yor1−dF
miss 0
orf −6
int 6.7
rank 445
(YPR071W)
DR = −730
Yor1
miss 0
orf 0.2
int 3.9
rank 1176
yor1−dF
miss 0
orf 2.6
int 6.7
rank 446
MET6(YER091C)
DR = −14
Yor1
miss 0
orf −2.8
int 8.9
rank 461
yor1−dF
miss 0
orf 1.1
int 6.7
rank 447
PEX17(YNL214W)
DR = −1831
Yor1
miss 0
orf −1.3
int −0.8
rank 2279
yor1−dF
miss 0
orf 1.1
int 6.7
rank 448
SIP1(YDR422C)
DR = −1000
Yor1
miss 0
orf 6
int 2.6
rank 1449
yor1−dF
miss 0
orf −4.9
int 6.7
rank 449
(YER046W−A)
DR = −269
Yor1
miss 0
orf 5.5
int 6.8
rank 719
yor1−dF
miss 0
orf −2.8
int 6.7
rank 450
0.35 0.25 0.20 0.15 0.10 0.05 0.00
−60 −20 0 20 40 −50 0 50

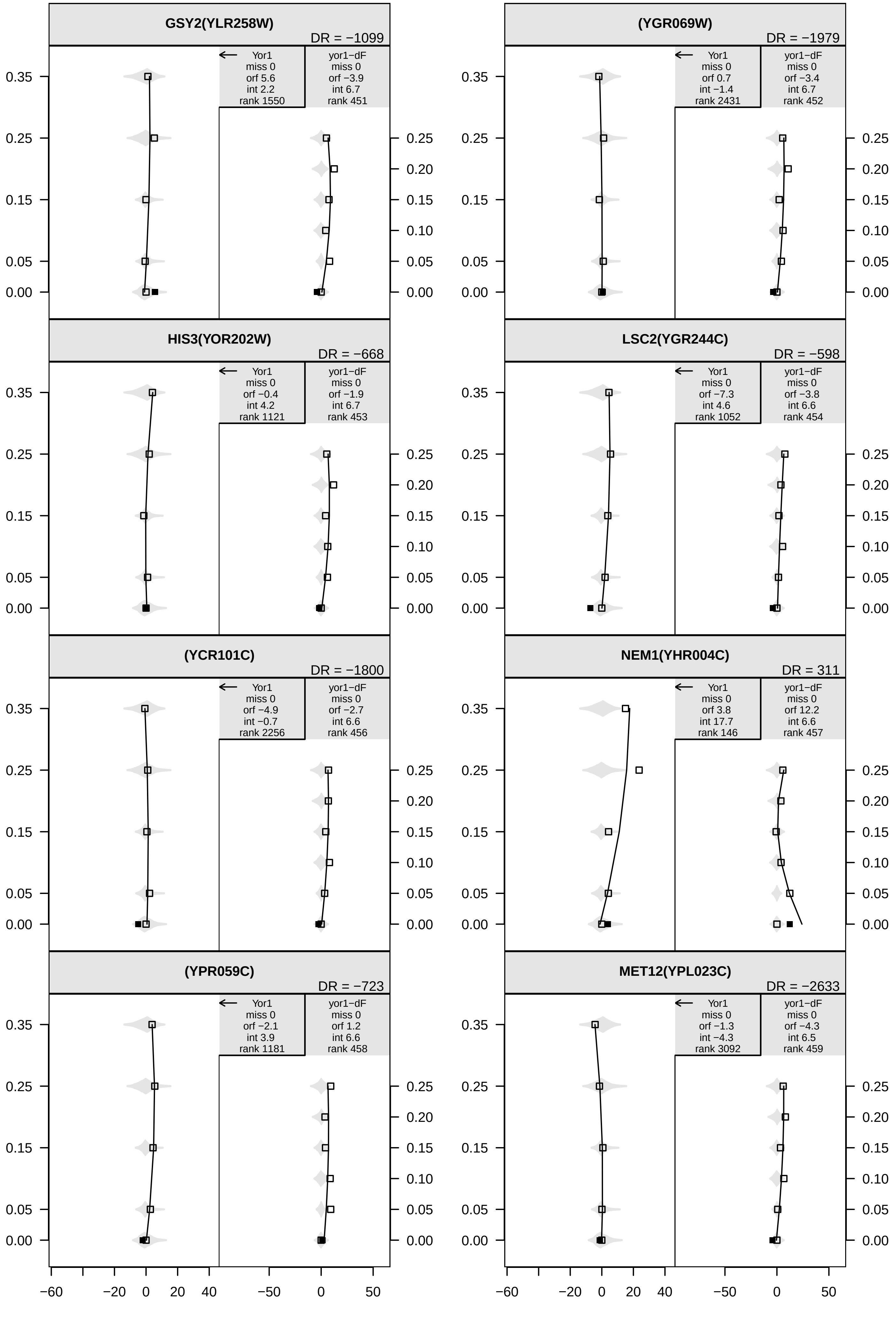

GSY2(YLR258W)
DR = −1099
Yor1 miss 0 orf 5.6 int 2.2 rank 1550
yor1−dF miss 0 orf −3.9 int 6.7 rank 451
(YGR069W)
DR = −1979
Yor1 miss 0 orf 0.7 int −1.4 rank 2431
yor1−dF miss 0 orf −3.4 int 6.7 rank 452
HIS3(YOR202W)
DR = −668
Yor1 miss 0 orf −0.4 int 4.2 rank 1121
yor1−dF miss 0 orf −1.9 int 6.7 rank 453
LSC2(YGR244C)
DR = −598
Yor1 miss 0 orf −7.3 int 4.6 rank 1052
yor1−dF miss 0 orf −3.8 int 6.6 rank 454
(YCR101C)
DR = −1800
Yor1 miss 0 orf −4.9 int −0.7 rank 2256
yor1−dF miss 0 orf −2.7 int 6.6 rank 456
NEM1(YHR004C)
DR = 311
Yor1 miss 0 orf 3.8 int 17.7 rank 146
yor1−dF miss 0 orf 12.2 int 6.6 rank 457
(YPR059C)
DR = −723
Yor1 miss 0 orf −2.1 int 3.9 rank 1181
yor1−dF miss 0 orf 1.2 int 6.6 rank 458
MET12(YPL023C)
DR = −2633
Yor1 miss 0 orf −1.3 int −4.3 rank 3092
yor1−dF miss 0 orf −4.3 int 6.5 rank 459

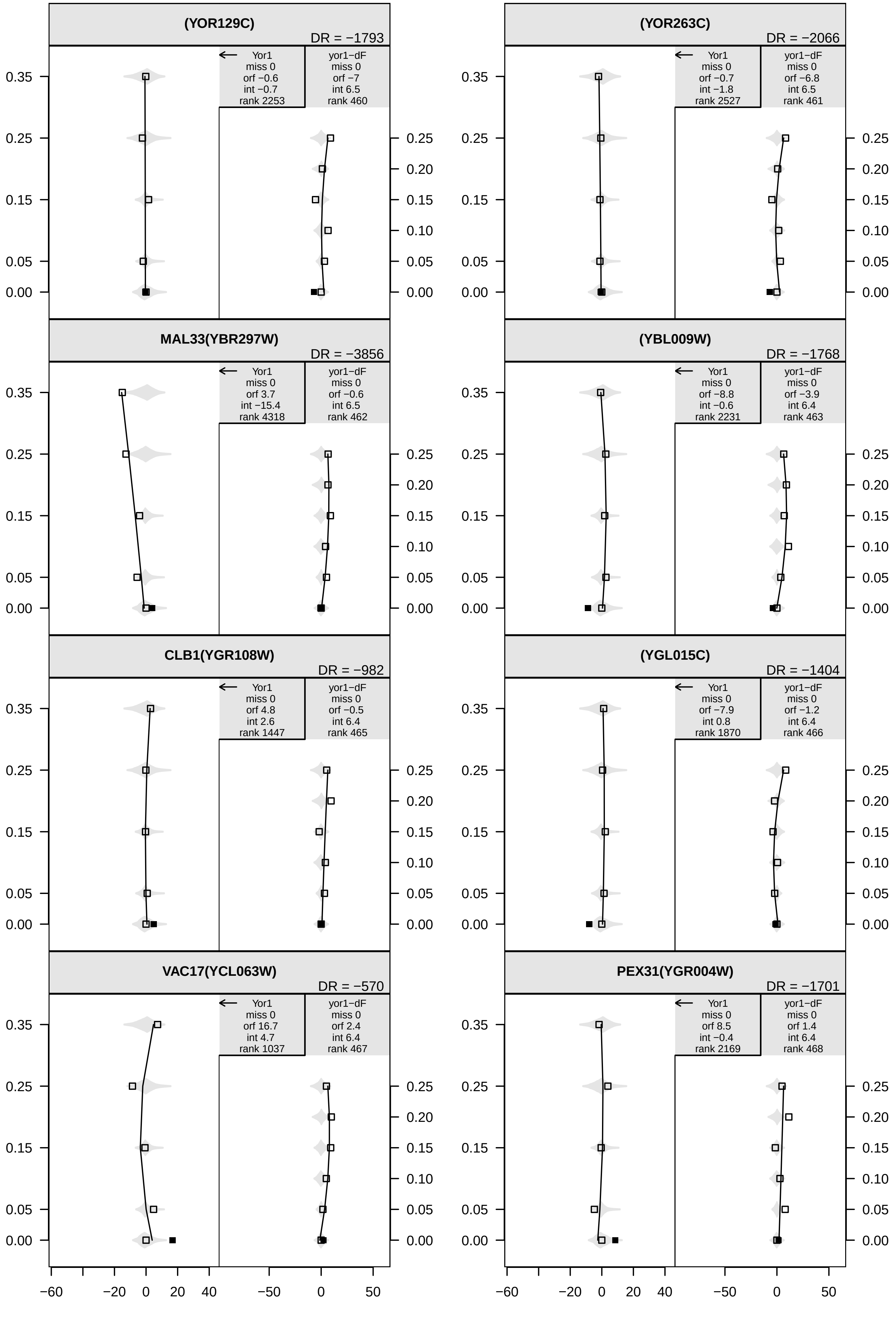

(YOR129C)
DR = −1793
Yor1 miss 0 orf −0.6 int −0.7 rank 2253
yor1−dF miss 0 orf −7 int 6.5 rank 460
(YOR263C)
DR = −2066
Yor1 miss 0 orf −0.7 int −1.8 rank 2527
yor1−dF miss 0 orf −6.8 int 6.5 rank 461
MAL33(YBR297W)
DR = −3856
Yor1 miss 0 orf 3.7 int −15.4 rank 4318
yor1−dF miss 0 orf −0.6 int 6.5 rank 462
(YBL009W)
DR = −1768
Yor1 miss 0 orf −8.8 int −0.6 rank 2231
yor1−dF miss 0 orf −3.9 int 6.4 rank 463
CLB1(YGR108W)
DR = −982
Yor1 miss 0 orf 4.8 int 2.6 rank 1447
yor1−dF miss 0 orf −0.5 int 6.4 rank 465
(YGL015C)
DR = −1404
Yor1 miss 0 orf −7.9 int 0.8 rank 1870
yor1−dF miss 0 orf −1.2 int 6.4 rank 466
VAC17(YCL063W)
DR = −570
Yor1 miss 0 orf 16.7 int 4.7 rank 1037
yor1−dF miss 0 orf 2.4 int 6.4 rank 467
PEX31(YGR004W)
DR = −1701
Yor1 miss 0 orf 8.5 int −0.4 rank 2169
yor1−dF miss 0 orf 1.4 int 6.4 rank 468

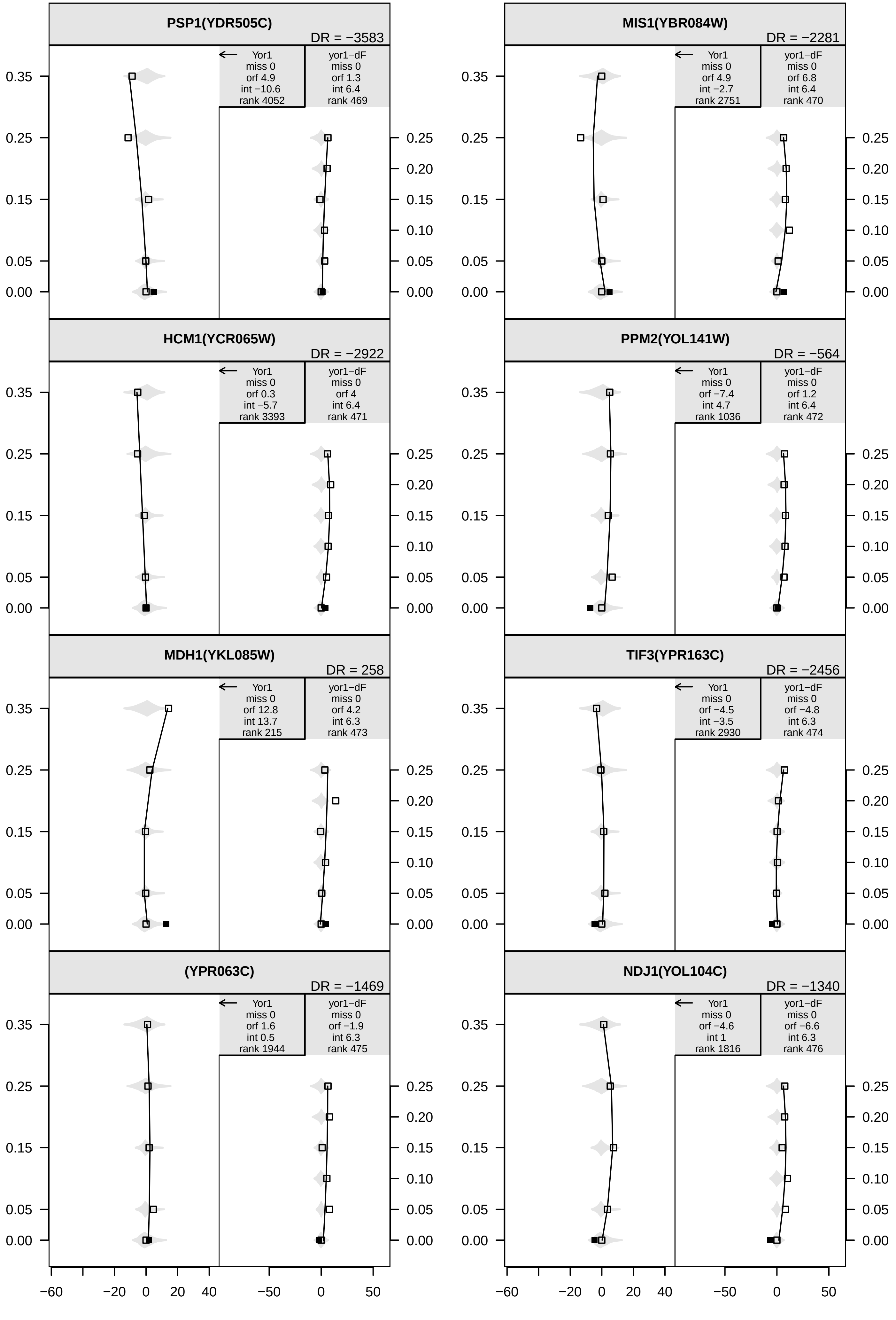
PSP1(YDR505C)
DR = −3583
Yor1
miss 0
orf 4.9
int −10.6
rank 4052
yor1−dF
miss 0
orf 1.3
int 6.4
rank 469
MIS1(YBR084W)
DR = −2281
Yor1
miss 0
orf 4.9
int −2.7
rank 2751
yor1−dF
miss 0
orf 6.8
int 6.4
rank 470
HCM1(YCR065W)
DR = −2922
Yor1
miss 0
orf 0.3
int −5.7
rank 3393
yor1−dF
miss 0
orf 4
int 6.4
rank 471
PPM2(YOL141W)
DR = −564
Yor1
miss 0
orf −7.4
int 4.7
rank 1036
yor1−dF
miss 0
orf 1.2
int 6.4
rank 472
MDH1(YKL085W)
DR = 258
Yor1
miss 0
orf 12.8
int 13.7
rank 215
yor1−dF
miss 0
orf 4.2
int 6.3
rank 473
TIF3(YPR163C)
DR = −2456
Yor1
miss 0
orf −4.5
int −3.5
rank 2930
yor1−dF
miss 0
orf −4.8
int 6.3
rank 474
(YPR063C)
DR = −1469
Yor1
miss 0
orf 1.6
int 0.5
rank 1944
yor1−dF
miss 0
orf −1.9
int 6.3
rank 475
NDJ1(YOL104C)
DR = −1340
Yor1
miss 0
orf −4.6
int 1
rank 1816
yor1−dF
miss 0
orf −6.6
int 6.3
rank 476
0.35 0.25 0.15 0.05 0.00
0.25 0.20 0.15 0.10 0.05 0.00
−60 −20 0 20 40 −50 0 50

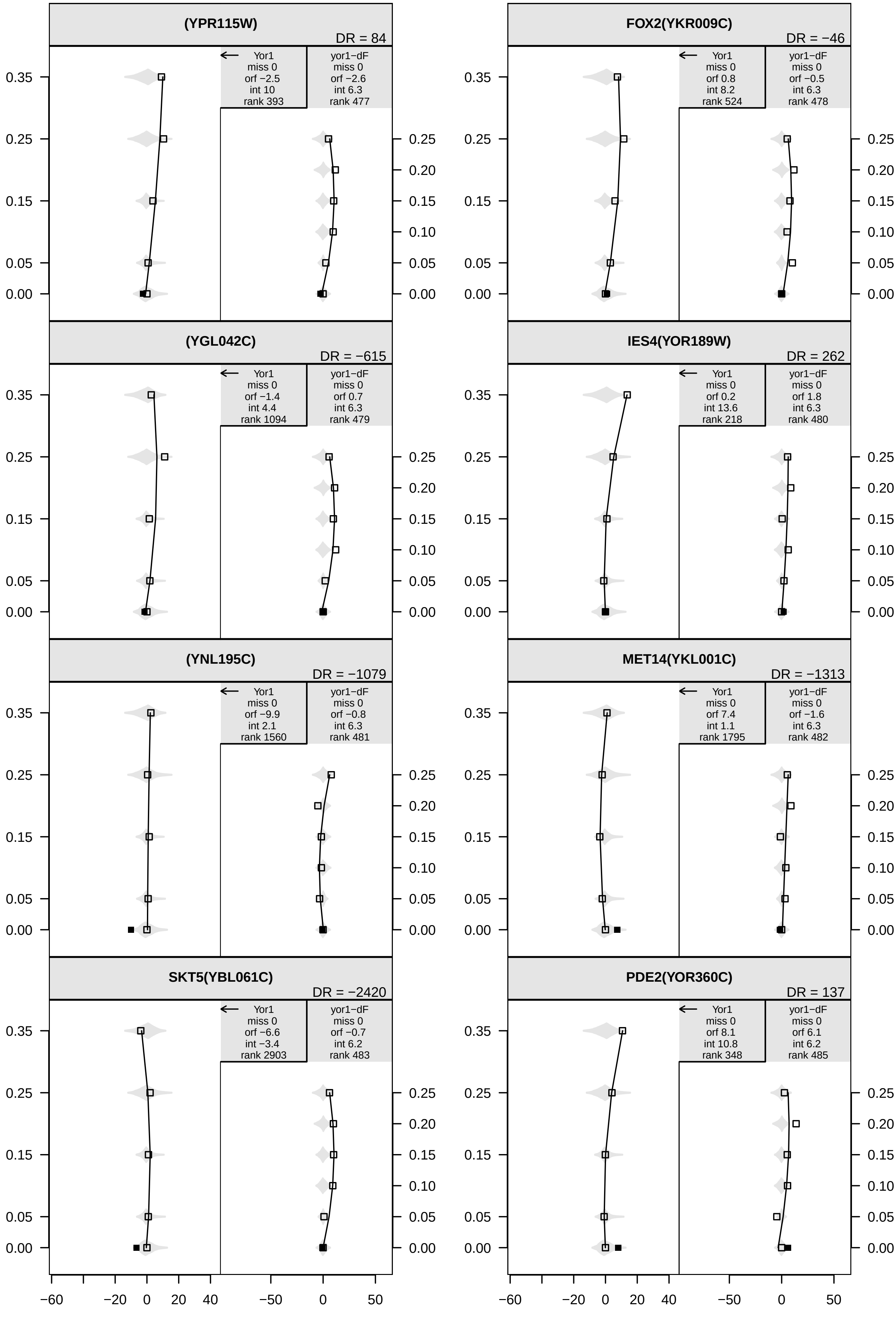

(YPR115W)
DR = 84
Yor1 miss 0 orf −2.5 int 10 rank 393
yor1−dF miss 0 orf −2.6 int 6.3 rank 477
FOX2(YKR009C)
DR = −46
Yor1 miss 0 orf 0.8 int 8.2 rank 524
yor1−dF miss 0 orf −0.5 int 6.3 rank 478
(YGL042C)
DR = −615
Yor1 miss 0 orf −1.4 int 4.4 rank 1094
yor1−dF miss 0 orf 0.7 int 6.3 rank 479
IES4(YOR189W)
DR = 262
Yor1 miss 0 orf 0.2 int 13.6 rank 218
yor1−dF miss 0 orf 1.8 int 6.3 rank 480
(YNL195C)
DR = −1079
Yor1 miss 0 orf −9.9 int 2.1 rank 1560
yor1−dF miss 0 orf −0.8 int 6.3 rank 481
MET14(YKL001C)
DR = −1313
Yor1 miss 0 orf 7.4 int 1.1 rank 1795
yor1−dF miss 0 orf −1.6 int 6.3 rank 482
SKT5(YBL061C)
DR = −2420
Yor1 miss 0 orf −6.6 int −3.4 rank 2903
yor1−dF miss 0 orf −0.7 int 6.2 rank 483
PDE2(YOR360C)
DR = 137
Yor1 miss 0 orf 8.1 int 10.8 rank 348
yor1−dF miss 0 orf 6.1 int 6.2 rank 485

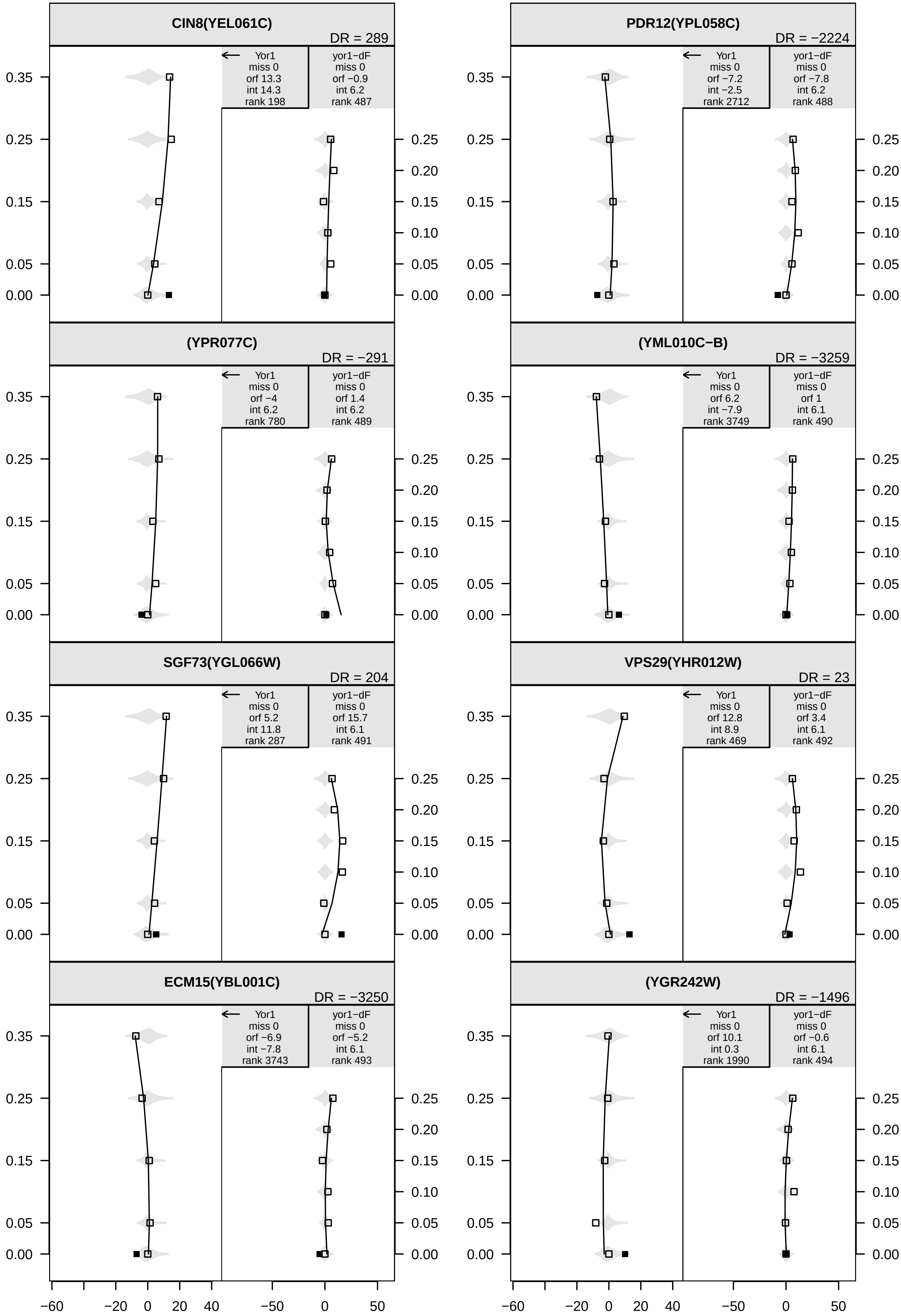

CIN8(YEL061C)
DR = 289
Yor1
miss 0
orf 13.3
int 14.3
rank 198
yor1−dF
miss 0
orf −0.9
int 6.2
rank 487
PDR12(YPL058C)
DR = −2224
Yor1
miss 0
orf −7.2
int −2.5
rank 2712
yor1−dF
miss 0
orf −7.8
int 6.2
rank 488
(YPR077C)
DR = −291
Yor1
miss 0
orf −4
int 6.2
rank 780
yor1−dF
miss 0
orf 1.4
int 6.2
rank 489
(YML010C−B)
DR = −3259
Yor1
miss 0
orf 6.2
int −7.9
rank 3749
yor1−dF
miss 0
orf 1
int 6.1
rank 490
SGF73(YGL066W)
DR = 204
Yor1
miss 0
orf 5.2
int 11.8
rank 287
yor1−dF
miss 0
orf 15.7
int 6.1
rank 491
VPS29(YHR012W)
DR = 23
Yor1
miss 0
orf 12.8
int 8.9
rank 469
yor1−dF
miss 0
orf 3.4
int 6.1
rank 492
ECM15(YBL001C)
DR = −3250
Yor1
miss 0
orf −6.9
int −7.8
rank 3743
yor1−dF
miss 0
orf −5.2
int 6.1
rank 493
(YGR242W)
DR = −1496
Yor1
miss 0
orf 10.1
int 0.3
rank 1990
yor1−dF
miss 0
orf −0.6
int 6.1
rank 494
0.35 0.25 0.20 0.15 0.10 0.05 0.00
−60 −20 0 20 40 −50 0 50

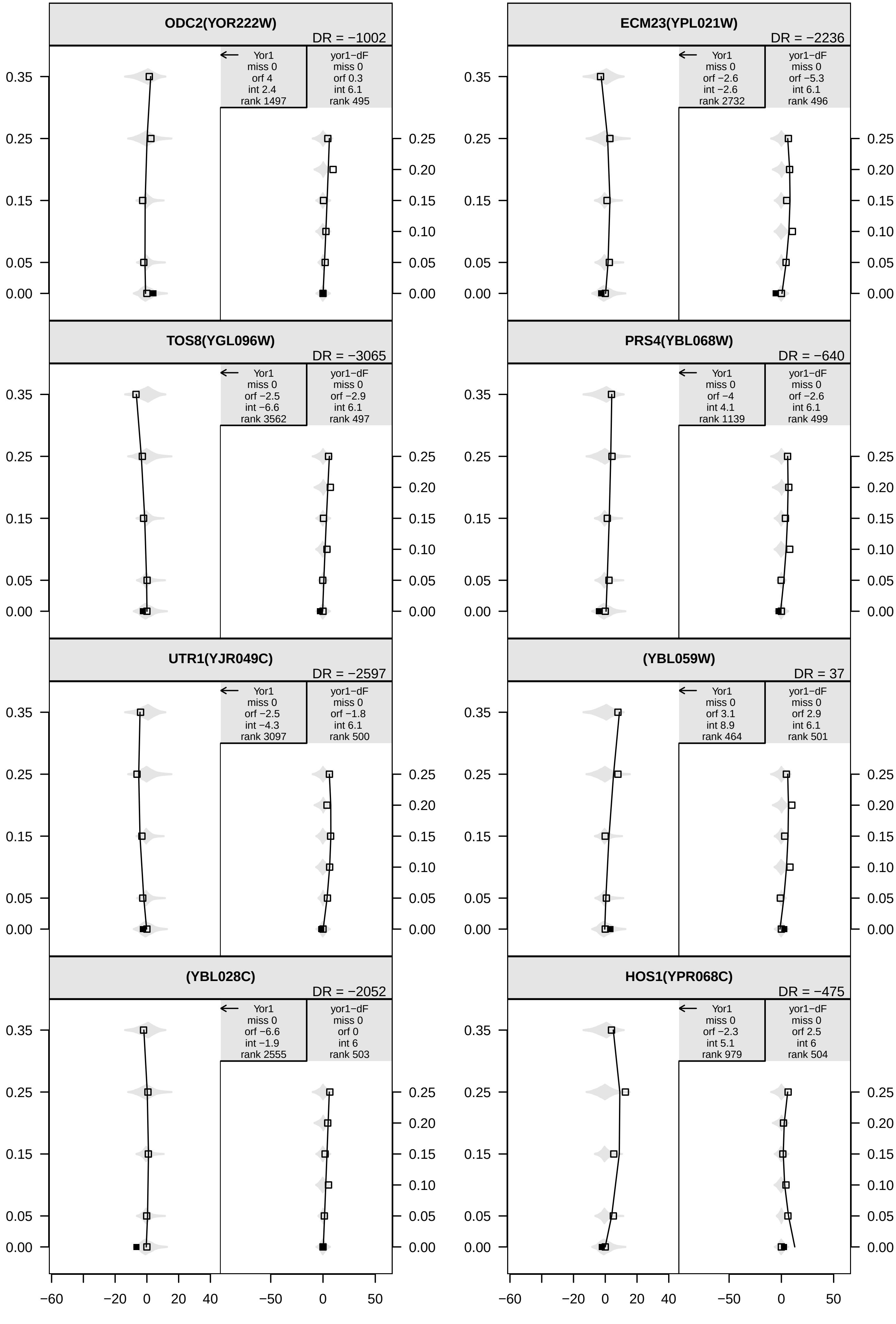
ODC2(YOR222W)
DR = −1002
Yor1 miss 0 orf 4 int 2.4 rank 1497
yor1−dF miss 0 orf 0.3 int 6.1 rank 495
ECM23(YPL021W)
DR = −2236
Yor1 miss 0 orf −2.6 int −2.6 rank 2732
yor1−dF miss 0 orf −5.3 int 6.1 rank 496
TOS8(YGL096W)
DR = −3065
Yor1 miss 0 orf −2.5 int −6.6 rank 3562
yor1−dF miss 0 orf −2.9 int 6.1 rank 497
PRS4(YBL068W)
DR = −640
Yor1 miss 0 orf −4 int 4.1 rank 1139
yor1−dF miss 0 orf −2.6 int 6.1 rank 499
UTR1(YJR049C)
DR = −2597
Yor1 miss 0 orf −2.5 int −4.3 rank 3097
yor1−dF miss 0 orf −1.8 int 6.1 rank 500
(YBL059W)
DR = 37
Yor1 miss 0 orf 3.1 int 8.9 rank 464
yor1−dF miss 0 orf 2.9 int 6.1 rank 501
(YBL028C)
DR = −2052
Yor1 miss 0 orf −6.6 int −1.9 rank 2555
yor1−dF miss 0 orf 0 int 6 rank 503
HOS1(YPR068C)
DR = −475
Yor1 miss 0 orf −2.3 int 5.1 rank 979
yor1−dF miss 0 orf 2.5 int 6 rank 504
0.35 0.25 0.20 0.15 0.10 0.05 0.00
−60 −20 0 20 40 −50 0 50

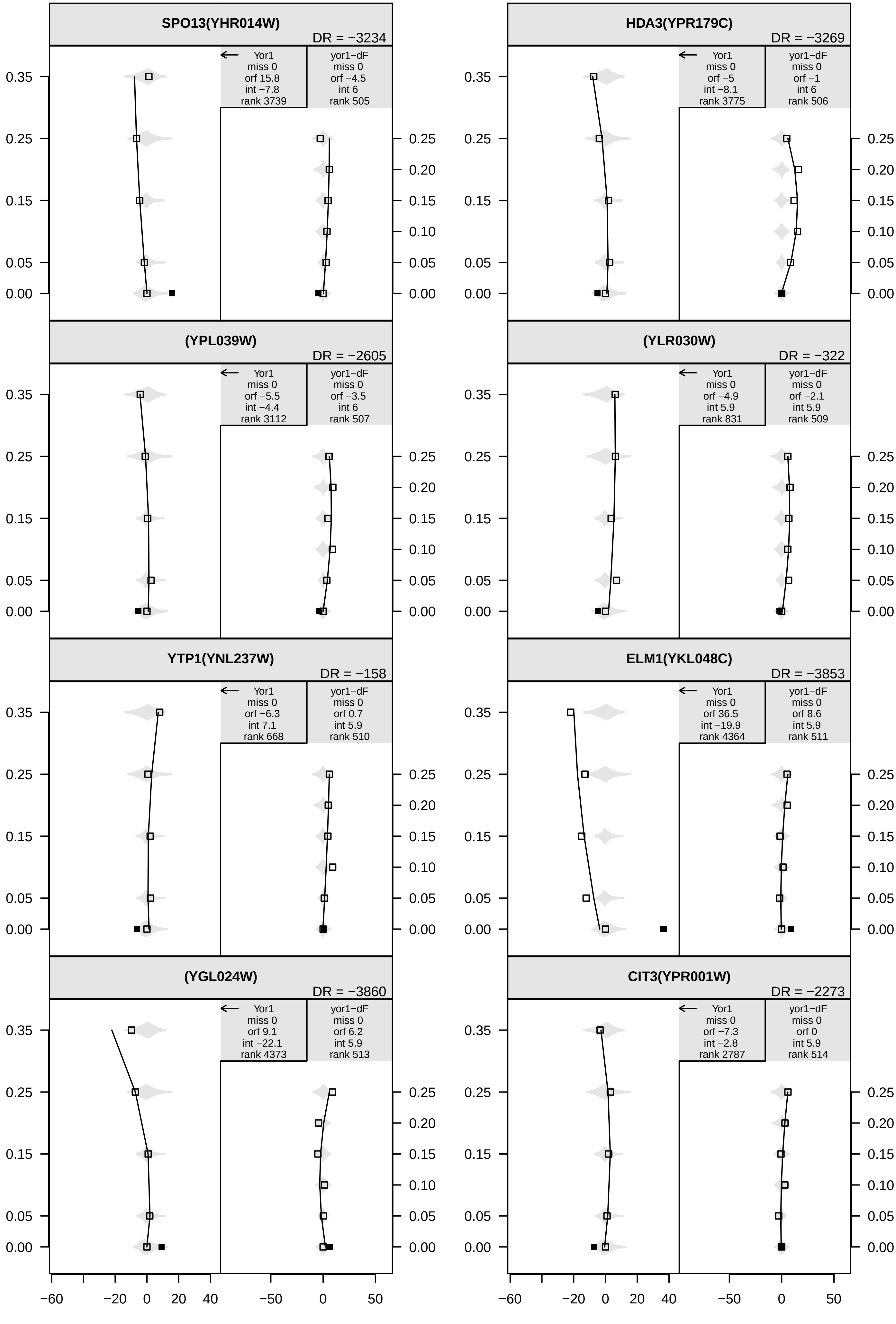
SPO13(YHR014W)
DR = −3234
Yor1 miss 0 orf 15.8 int −7.8 rank 3739
yor1−dF miss 0 orf −4.5 int 6 rank 505
HDA3(YPR179C)
DR = −3269
Yor1 miss 0 orf −5 int −8.1 rank 3775
yor1−dF miss 0 orf −1 int 6 rank 506
(YPL039W)
DR = −2605
Yor1 miss 0 orf −5.5 int −4.4 rank 3112
yor1−dF miss 0 orf −3.5 int 6 rank 507
(YLR030W)
DR = −322
Yor1 miss 0 orf −4.9 int 5.9 rank 831
yor1−dF miss 0 orf −2.1 int 5.9 rank 509
YTP1(YNL237W)
DR = −158
Yor1 miss 0 orf −6.3 int 7.1 rank 668
yor1−dF miss 0 orf 0.7 int 5.9 rank 510
ELM1(YKL048C)
DR = −3853
Yor1 miss 0 orf 36.5 int −19.9 rank 4364
yor1−dF miss 0 orf 8.6 int 5.9 rank 511
(YGL024W)
DR = −3860
Yor1 miss 0 orf 9.1 int −22.1 rank 4373
yor1−dF miss 0 orf 6.2 int 5.9 rank 513
CIT3(YPR001W)
DR = −2273
Yor1 miss 0 orf −7.3 int −2.8 rank 2787
yor1−dF miss 0 orf 0 int 5.9 rank 514

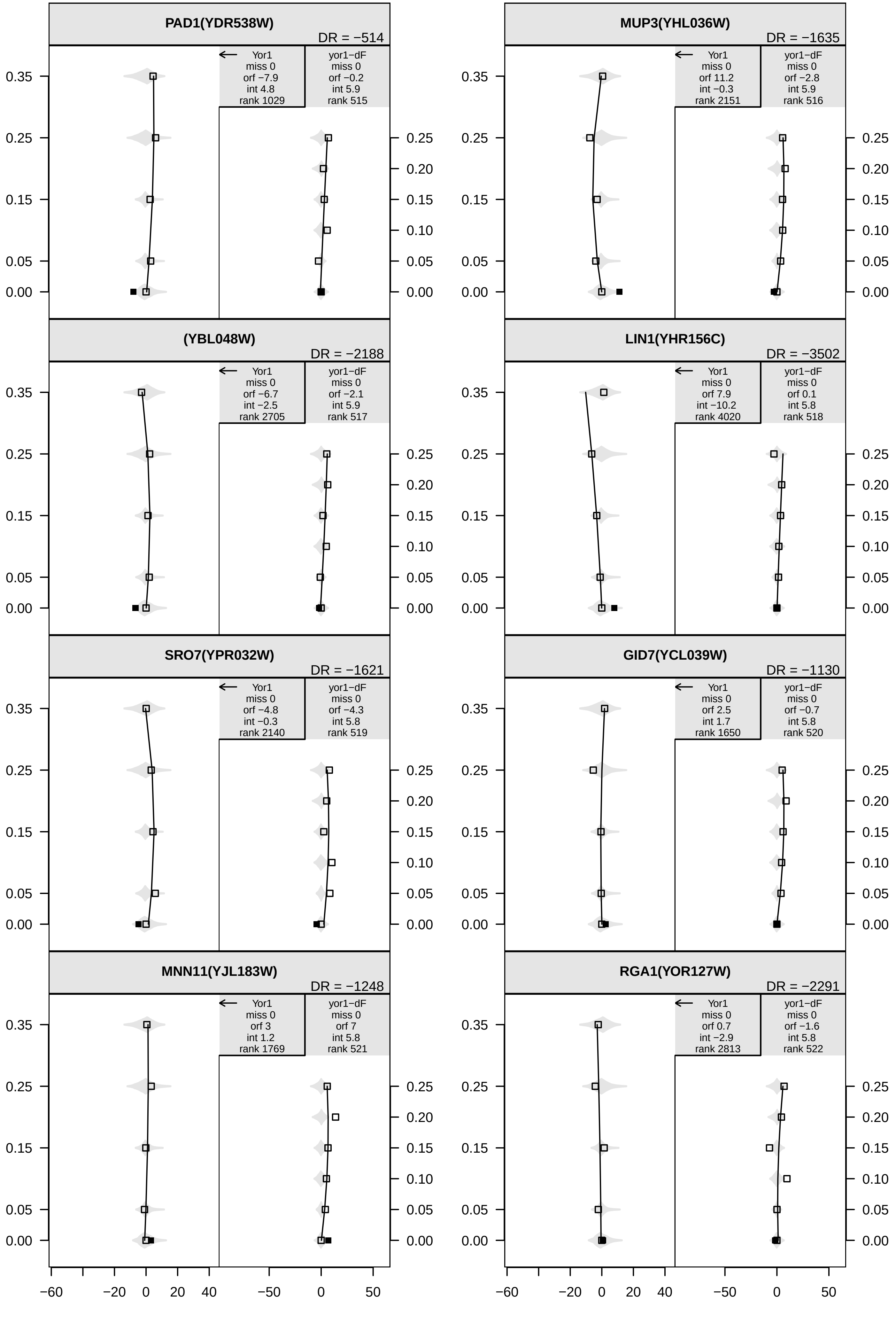

PAD1(YDR538W)
DR = −514
Yor1
miss 0
orf −7.9
int 4.8
rank 1029
yor1−dF
miss 0
orf −0.2
int 5.9
rank 515
MUP3(YHL036W)
DR = −1635
Yor1
miss 0
orf 11.2
int −0.3
rank 2151
yor1−dF
miss 0
orf −2.8
int 5.9
rank 516
(YBL048W)
DR = −2188
Yor1
miss 0
orf −6.7
int −2.5
rank 2705
yor1−dF
miss 0
orf −2.1
int 5.9
rank 517
LIN1(YHR156C)
DR = −3502
Yor1
miss 0
orf 7.9
int −10.2
rank 4020
yor1−dF
miss 0
orf 0.1
int 5.8
rank 518
SRO7(YPR032W)
DR = −1621
Yor1
miss 0
orf −4.8
int −0.3
rank 2140
yor1−dF
miss 0
orf −4.3
int 5.8
rank 519
GID7(YCL039W)
DR = −1130
Yor1
miss 0
orf 2.5
int 1.7
rank 1650
yor1−dF
miss 0
orf −0.7
int 5.8
rank 520
MNN11(YJL183W)
DR = −1248
Yor1
miss 0
orf 3
int 1.2
rank 1769
yor1−dF
miss 0
orf 7
int 5.8
rank 521
RGA1(YOR127W)
DR = −2291
Yor1
miss 0
orf 0.7
int −2.9
rank 2813
yor1−dF
miss 0
orf −1.6
int 5.8
rank 522
0.35
0.25
0.20
0.15
0.10
0.05
0.00
−60
−20
0
20
40
−50
0
50

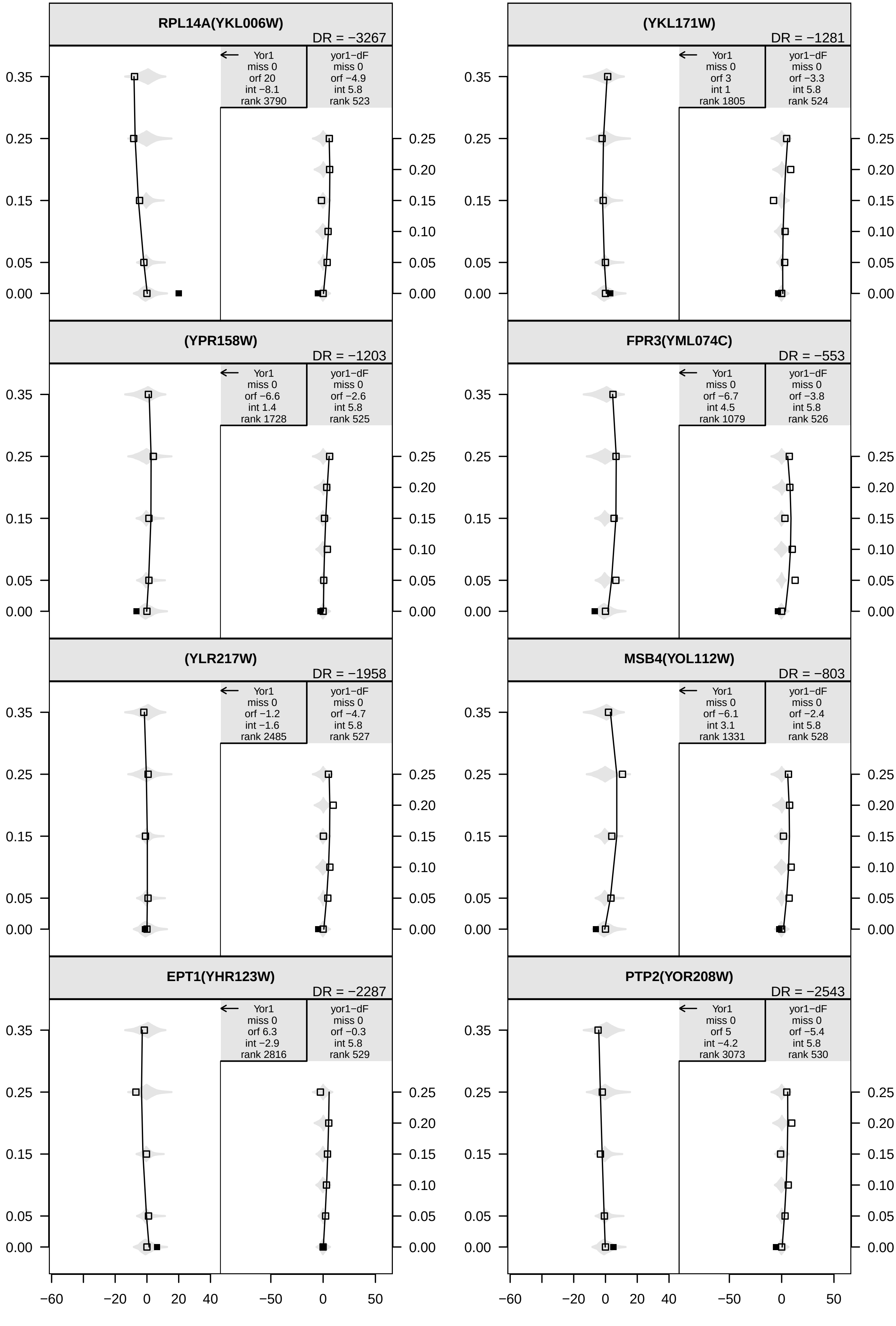
RPL14A(YKL006W)
DR = −3267
Yor1
miss 0
orf 20
int −8.1
rank 3790
yor1−dF
miss 0
orf −4.9
int 5.8
rank 523
(YKL171W)
DR = −1281
Yor1
miss 0
orf 3
int 1
rank 1805
yor1−dF
miss 0
orf −3.3
int 5.8
rank 524
(YPR158W)
DR = −1203
Yor1
miss 0
orf −6.6
int 1.4
rank 1728
yor1−dF
miss 0
orf −2.6
int 5.8
rank 525
FPR3(YML074C)
DR = −553
Yor1
miss 0
orf −6.7
int 4.5
rank 1079
yor1−dF
miss 0
orf −3.8
int 5.8
rank 526
(YLR217W)
DR = −1958
Yor1
miss 0
orf −1.2
int −1.6
rank 2485
yor1−dF
miss 0
orf −4.7
int 5.8
rank 527
MSB4(YOL112W)
DR = −803
Yor1
miss 0
orf −6.1
int 3.1
rank 1331
yor1−dF
miss 0
orf −2.4
int 5.8
rank 528
EPT1(YHR123W)
DR = −2287
Yor1
miss 0
orf 6.3
int −2.9
rank 2816
yor1−dF
miss 0
orf −0.3
int 5.8
rank 529
PTP2(YOR208W)
DR = −2543
Yor1
miss 0
orf 5
int −4.2
rank 3073
yor1−dF
miss 0
orf −5.4
int 5.8
rank 530
0.35
0.25
0.20
0.15
0.10
0.05
0.00
−60
−20
0
20
40
−50
0
50

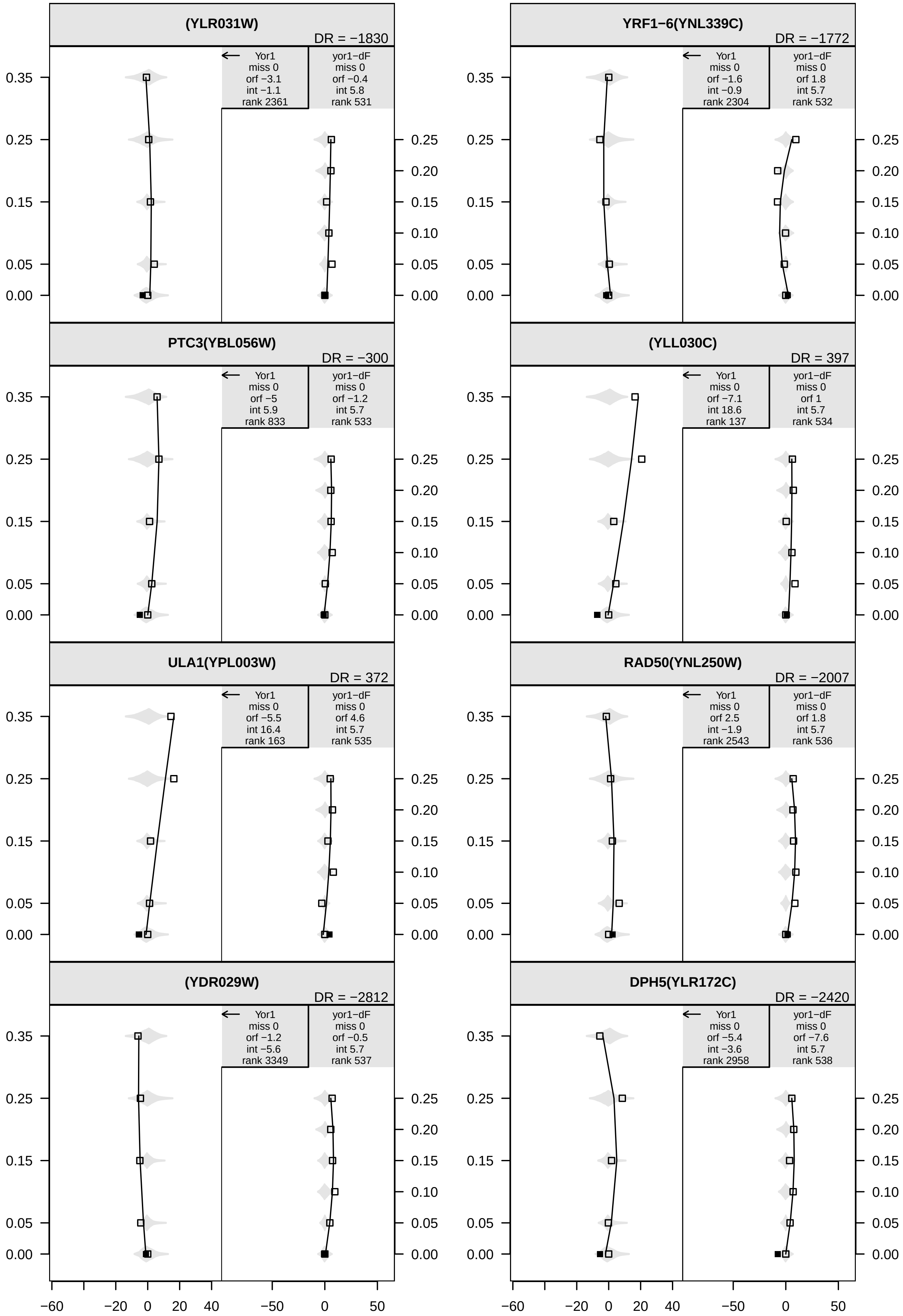
(YLR031W)
DR = −1830
Yor1 miss 0 orf −3.1 int −1.1 rank 2361
yor1−dF miss 0 orf −0.4 int 5.8 rank 531
YRF1−6(YNL339C)
DR = −1772
Yor1 miss 0 orf −1.6 int −0.9 rank 2304
yor1−dF miss 0 orf 1.8 int 5.7 rank 532
PTC3(YBL056W)
DR = −300
Yor1 miss 0 orf −5 int 5.9 rank 833
yor1−dF miss 0 orf −1.2 int 5.7 rank 533
(YLL030C)
DR = 397
Yor1 miss 0 orf −7.1 int 18.6 rank 137
yor1−dF miss 0 orf 1 int 5.7 rank 534
ULA1(YPL003W)
DR = 372
Yor1 miss 0 orf −5.5 int 16.4 rank 163
yor1−dF miss 0 orf 4.6 int 5.7 rank 535
RAD50(YNL250W)
DR = −2007
Yor1 miss 0 orf 2.5 int −1.9 rank 2543
yor1−dF miss 0 orf 1.8 int 5.7 rank 536
(YDR029W)
DR = −2812
Yor1 miss 0 orf −1.2 int −5.6 rank 3349
yor1−dF miss 0 orf −0.5 int 5.7 rank 537
DPH5(YLR172C)
DR = −2420
Yor1 miss 0 orf −5.4 int −3.6 rank 2958
yor1−dF miss 0 orf −7.6 int 5.7 rank 538

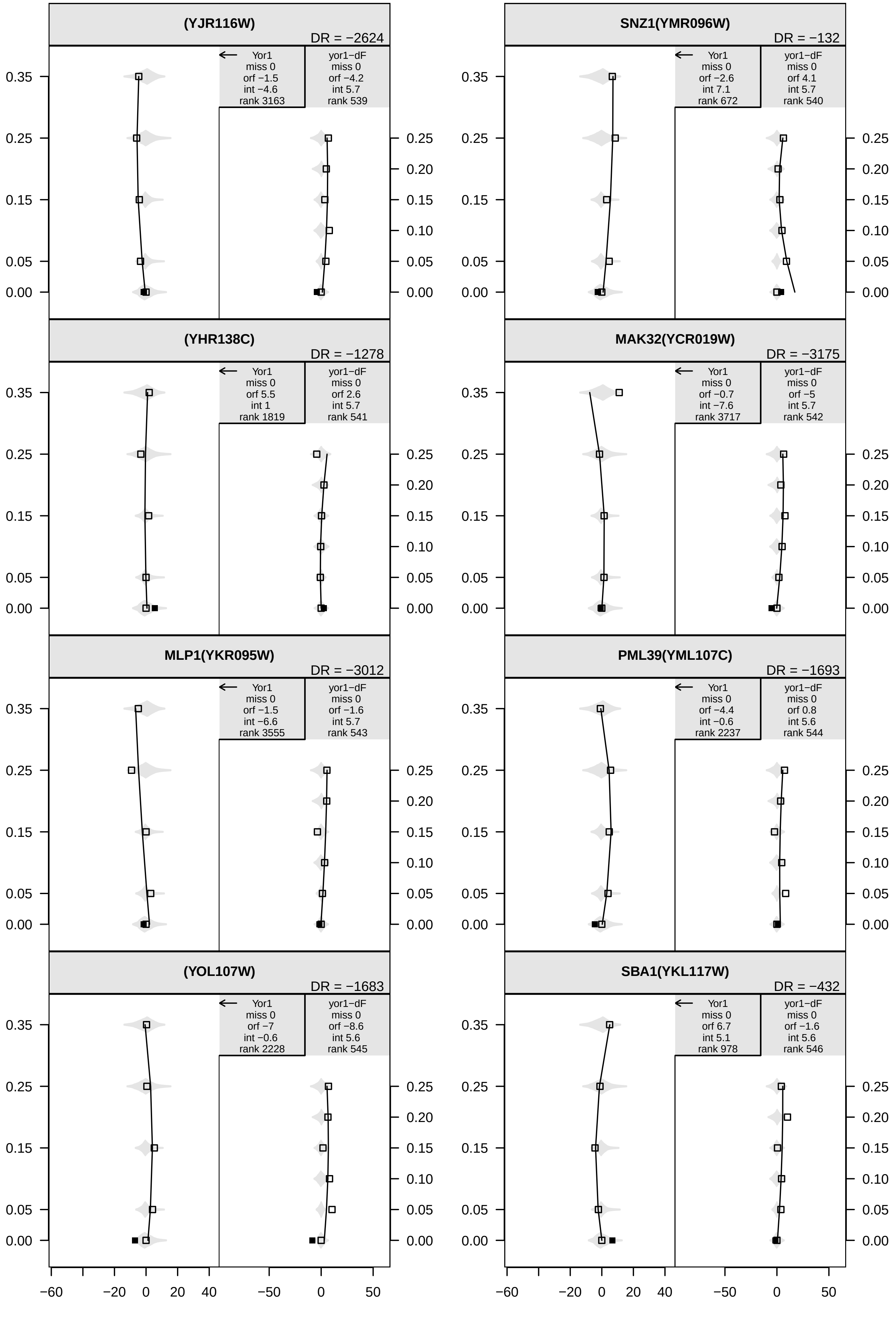

(YJR116W)
DR = −2624
Yor1
miss 0
orf −1.5
int −4.6
rank 3163
yor1−dF
miss 0
orf −4.2
int 5.7
rank 539
SNZ1(YMR096W)
DR = −132
Yor1
miss 0
orf −2.6
int 7.1
rank 672
yor1−dF
miss 0
orf 4.1
int 5.7
rank 540
(YHR138C)
DR = −1278
Yor1
miss 0
orf 5.5
int 1
rank 1819
yor1−dF
miss 0
orf 2.6
int 5.7
rank 541
MAK32(YCR019W)
DR = −3175
Yor1
miss 0
orf −0.7
int −7.6
rank 3717
yor1−dF
miss 0
orf −5
int 5.7
rank 542
MLP1(YKR095W)
DR = −3012
Yor1
miss 0
orf −1.5
int −6.6
rank 3555
yor1−dF
miss 0
orf −1.6
int 5.7
rank 543
PML39(YML107C)
DR = −1693
Yor1
miss 0
orf −4.4
int −0.6
rank 2237
yor1−dF
miss 0
orf 0.8
int 5.6
rank 544
(YOL107W)
DR = −1683
Yor1
miss 0
orf −7
int −0.6
rank 2228
yor1−dF
miss 0
orf −8.6
int 5.6
rank 545
SBA1(YKL117W)
DR = −432
Yor1
miss 0
orf 6.7
int 5.1
rank 978
yor1−dF
miss 0
orf −1.6
int 5.6
rank 546
0.35
0.25
0.20
0.15
0.10
0.05
0.00
−60
−20
0
20
40
−50
0
50

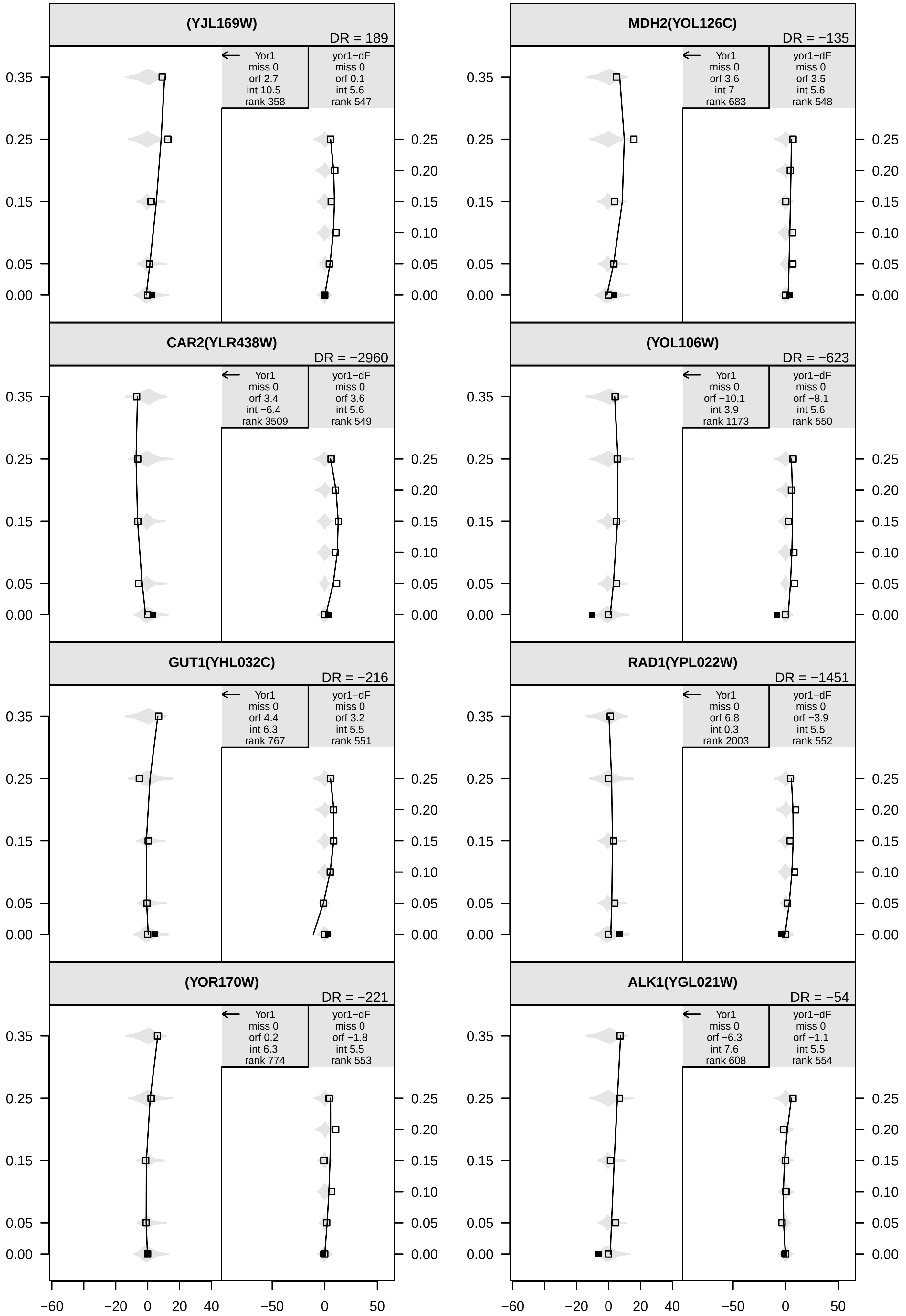
(YJL169W)
DR = 189
Yor1 miss 0 orf 2.7 int 10.5 rank 358
yor1−dF miss 0 orf 0.1 int 5.6 rank 547
MDH2(YOL126C)
DR = −135
Yor1 miss 0 orf 3.6 int 7 rank 683
yor1−dF miss 0 orf 3.5 int 5.6 rank 548
CAR2(YLR438W)
DR = −2960
Yor1 miss 0 orf 3.4 int −6.4 rank 3509
yor1−dF miss 0 orf 3.6 int 5.6 rank 549
(YOL106W)
DR = −623
Yor1 miss 0 orf −10.1 int 3.9 rank 1173
yor1−dF miss 0 orf −8.1 int 5.6 rank 550
GUT1(YHL032C)
DR = −216
Yor1 miss 0 orf 4.4 int 6.3 rank 767
yor1−dF miss 0 orf 3.2 int 5.5 rank 551
RAD1(YPL022W)
DR = −1451
Yor1 miss 0 orf 6.8 int 0.3 rank 2003
yor1−dF miss 0 orf −3.9 int 5.5 rank 552
(YOR170W)
DR = −221
Yor1 miss 0 orf 0.2 int 6.3 rank 774
yor1−dF miss 0 orf −1.8 int 5.5 rank 553
ALK1(YGL021W)
DR = −54
Yor1 miss 0 orf −6.3 int 7.6 rank 608
yor1−dF miss 0 orf −1.1 int 5.5 rank 554
0.00 0.05 0.15 0.25 0.35
0.00 0.05 0.10 0.15 0.20 0.25
−60 −20 0 20 40 −50 0 50

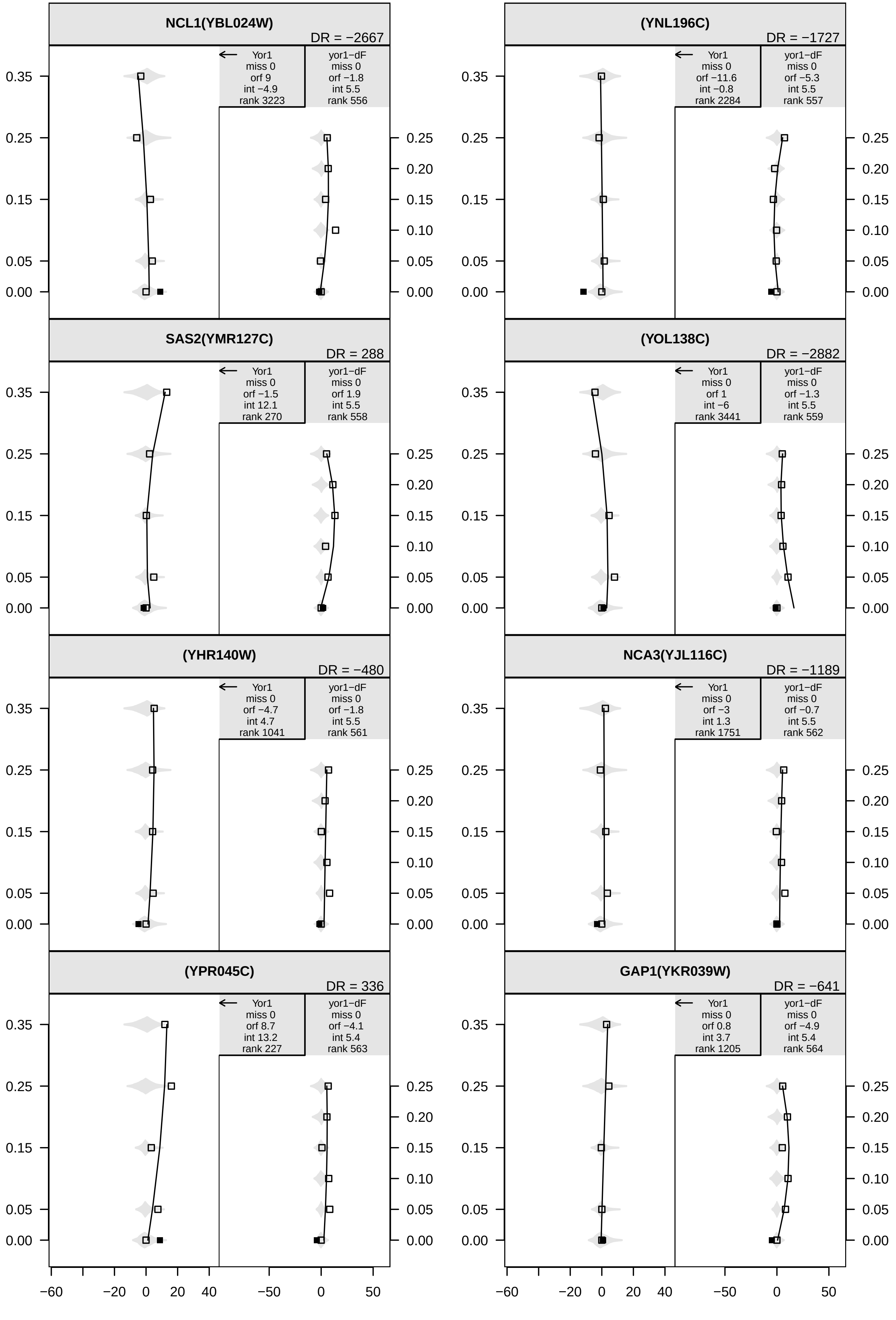
NCL1(YBL024W)
DR = −2667
Yor1 miss 0 orf 9 int −4.9 rank 3223
yor1−dF miss 0 orf −1.8 int 5.5 rank 556
(YNL196C)
DR = −1727
Yor1 miss 0 orf −11.6 int −0.8 rank 2284
yor1−dF miss 0 orf −5.3 int 5.5 rank 557
SAS2(YMR127C)
DR = 288
Yor1 miss 0 orf −1.5 int 12.1 rank 270
yor1−dF miss 0 orf 1.9 int 5.5 rank 558
(YOL138C)
DR = −2882
Yor1 miss 0 orf 1 int −6 rank 3441
yor1−dF miss 0 orf −1.3 int 5.5 rank 559
(YHR140W)
DR = −480
Yor1 miss 0 orf −4.7 int 4.7 rank 1041
yor1−dF miss 0 orf −1.8 int 5.5 rank 561
NCA3(YJL116C)
DR = −1189
Yor1 miss 0 orf −3 int 1.3 rank 1751
yor1−dF miss 0 orf −0.7 int 5.5 rank 562
(YPR045C)
DR = 336
Yor1 miss 0 orf 8.7 int 13.2 rank 227
yor1−dF miss 0 orf −4.1 int 5.4 rank 563
GAP1(YKR039W)
DR = −641
Yor1 miss 0 orf 0.8 int 3.7 rank 1205
yor1−dF miss 0 orf −4.9 int 5.4 rank 564
0.35 0.25 0.15 0.05 0.00
0.25 0.20 0.15 0.10 0.05 0.00
−60 −20 0 20 40
−50 0 50

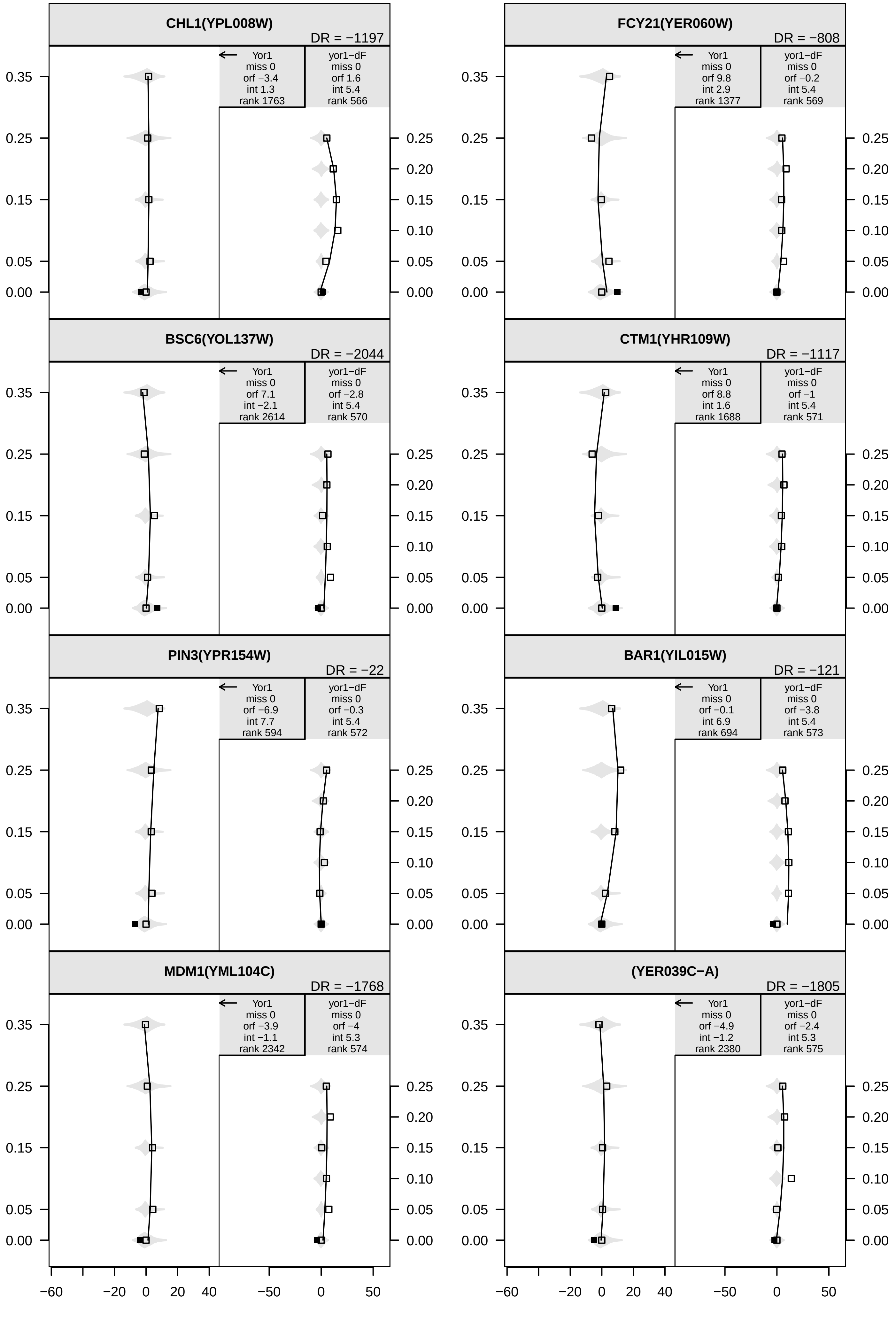
CHL1(YPL008W)
DR = −1197
Yor1 miss 0 orf −3.4 int 1.3 rank 1763
yor1−dF miss 0 orf 1.6 int 5.4 rank 566
FCY21(YER060W)
DR = −808
Yor1 miss 0 orf 9.8 int 2.9 rank 1377
yor1−dF miss 0 orf −0.2 int 5.4 rank 569
BSC6(YOL137W)
DR = −2044
Yor1 miss 0 orf 7.1 int −2.1 rank 2614
yor1−dF miss 0 orf −2.8 int 5.4 rank 570
CTM1(YHR109W)
DR = −1117
Yor1 miss 0 orf 8.8 int 1.6 rank 1688
yor1−dF miss 0 orf −1 int 5.4 rank 571
PIN3(YPR154W)
DR = −22
Yor1 miss 0 orf −6.9 int 7.7 rank 594
yor1−dF miss 0 orf −0.3 int 5.4 rank 572
BAR1(YIL015W)
DR = −121
Yor1 miss 0 orf −0.1 int 6.9 rank 694
yor1−dF miss 0 orf −3.8 int 5.4 rank 573
MDM1(YML104C)
DR = −1768
Yor1 miss 0 orf −3.9 int −1.1 rank 2342
yor1−dF miss 0 orf −4 int 5.3 rank 574
(YER039C−A)
DR = −1805
Yor1 miss 0 orf −4.9 int −1.2 rank 2380
yor1−dF miss 0 orf −2.4 int 5.3 rank 575
0.35 0.25 0.15 0.05 0.00
0.25 0.20 0.15 0.10 0.05 0.00
−60 −20 0 20 40 −50 0 50

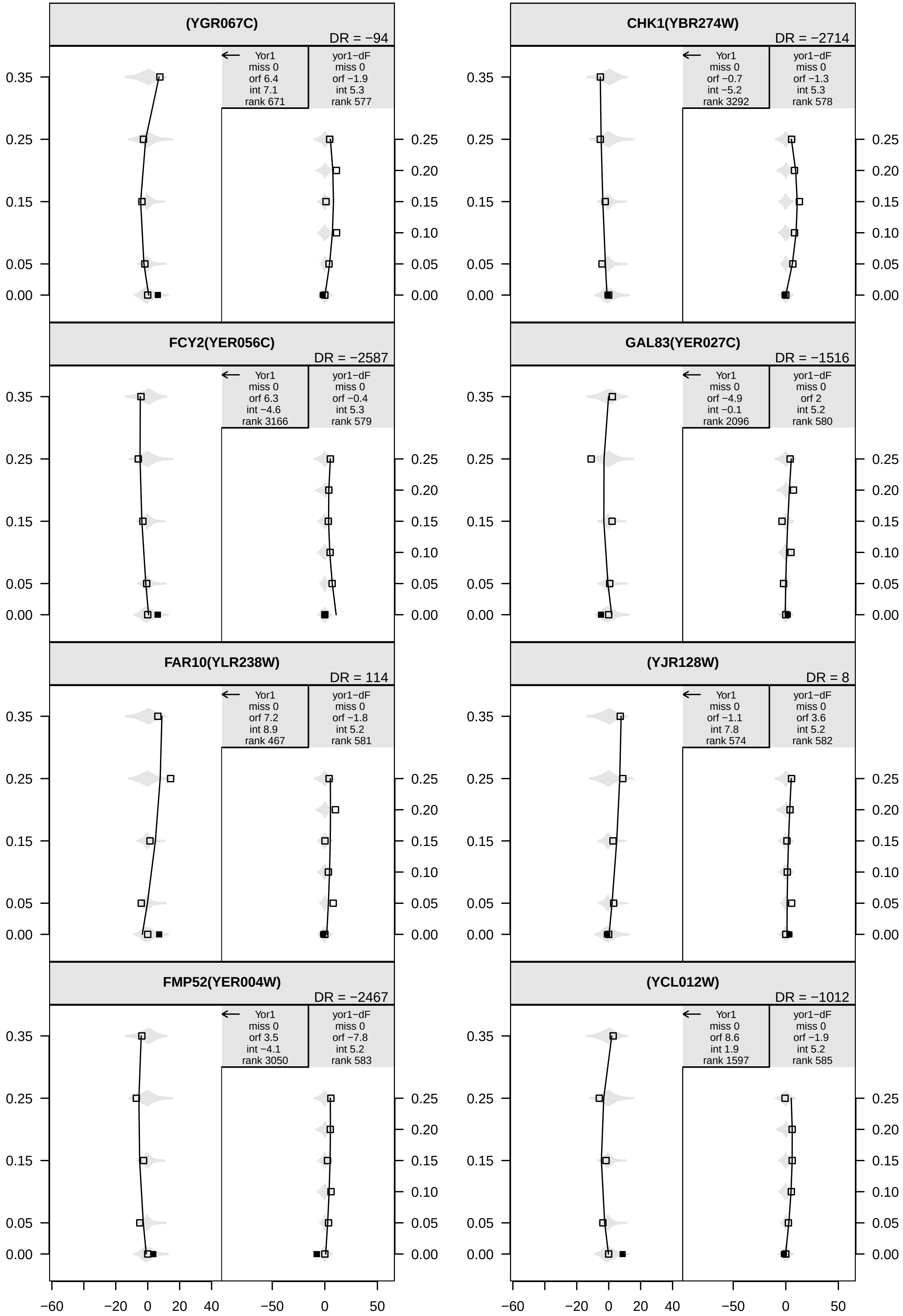
(YGR067C)
DR = −94
Yor1 miss 0 orf 6.4 int 7.1 rank 671
yor1−dF miss 0 orf −1.9 int 5.3 rank 577
CHK1(YBR274W)
DR = −2714
Yor1 miss 0 orf −0.7 int −5.2 rank 3292
yor1−dF miss 0 orf −1.3 int 5.3 rank 578
FCY2(YER056C)
DR = −2587
Yor1 miss 0 orf 6.3 int −4.6 rank 3166
yor1−dF miss 0 orf −0.4 int 5.3 rank 579
GAL83(YER027C)
DR = −1516
Yor1 miss 0 orf −4.9 int −0.1 rank 2096
yor1−dF miss 0 orf 2 int 5.2 rank 580
FAR10(YLR238W)
DR = 114
Yor1 miss 0 orf 7.2 int 8.9 rank 467
yor1−dF miss 0 orf −1.8 int 5.2 rank 581
(YJR128W)
DR = 8
Yor1 miss 0 orf −1.1 int 7.8 rank 574
yor1−dF miss 0 orf 3.6 int 5.2 rank 582
FMP52(YER004W)
DR = −2467
Yor1 miss 0 orf 3.5 int −4.1 rank 3050
yor1−dF miss 0 orf −7.8 int 5.2 rank 583
(YCL012W)
DR = −1012
Yor1 miss 0 orf 8.6 int 1.9 rank 1597
yor1−dF miss 0 orf −1.9 int 5.2 rank 585
0.35 0.25 0.15 0.05 0.00
0.25 0.20 0.15 0.10 0.05 0.00
−60 −20 0 20 40 −50 0 50

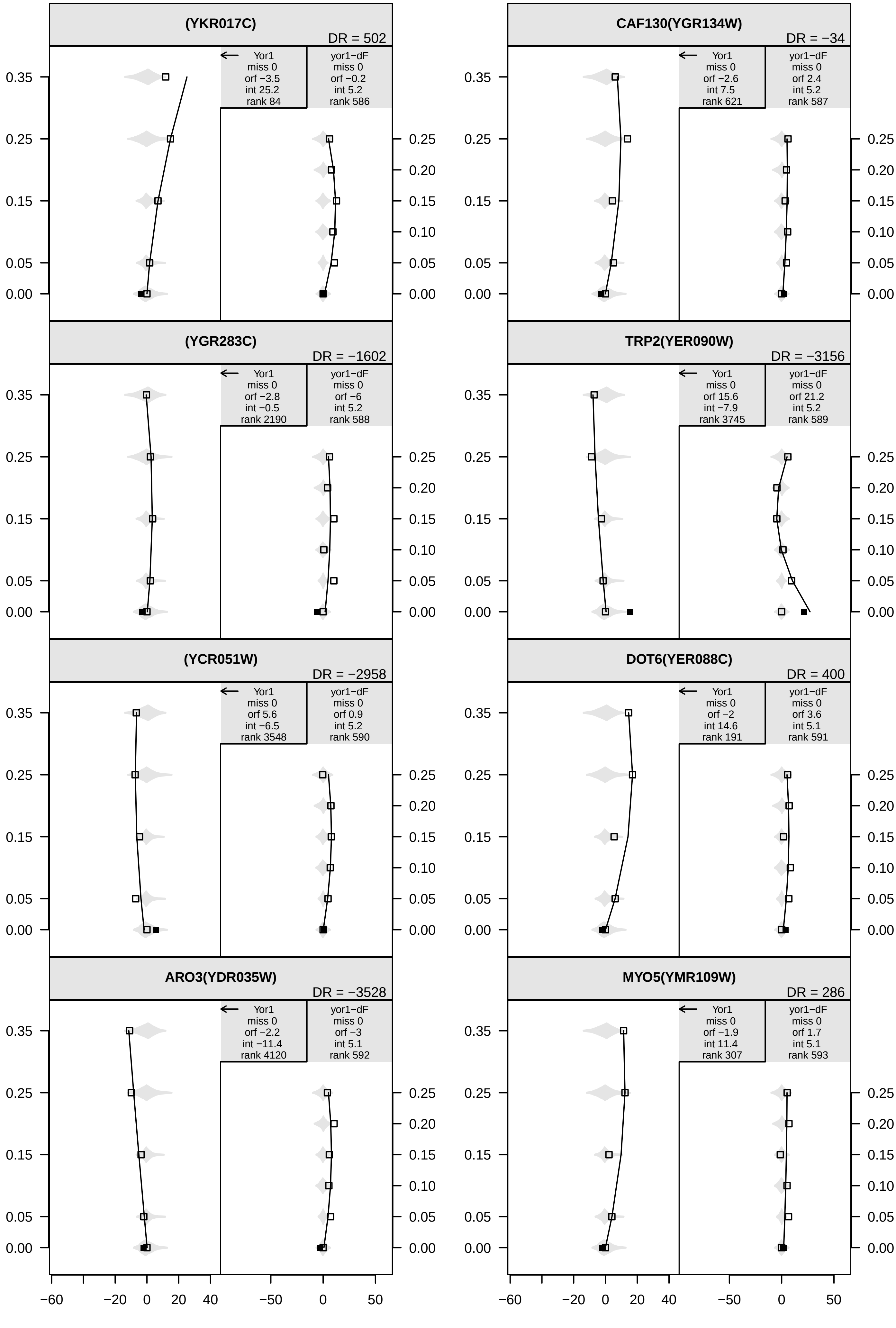

(YKR017C)
DR = 502
Yor1 miss 0 orf −3.5 int 25.2 rank 84
yor1−dF miss 0 orf −0.2 int 5.2 rank 586
CAF130(YGR134W)
DR = −34
Yor1 miss 0 orf −2.6 int 7.5 rank 621
yor1−dF miss 0 orf 2.4 int 5.2 rank 587
(YGR283C)
DR = −1602
Yor1 miss 0 orf −2.8 int −0.5 rank 2190
yor1−dF miss 0 orf −6 int 5.2 rank 588
TRP2(YER090W)
DR = −3156
Yor1 miss 0 orf 15.6 int −7.9 rank 3745
yor1−dF miss 0 orf 21.2 int 5.2 rank 589
(YCR051W)
DR = −2958
Yor1 miss 0 orf 5.6 int −6.5 rank 3548
yor1−dF miss 0 orf 0.9 int 5.2 rank 590
DOT6(YER088C)
DR = 400
Yor1 miss 0 orf −2 int 14.6 rank 191
yor1−dF miss 0 orf 3.6 int 5.1 rank 591
ARO3(YDR035W)
DR = −3528
Yor1 miss 0 orf −2.2 int −11.4 rank 4120
yor1−dF miss 0 orf −3 int 5.1 rank 592
MYO5(YMR109W)
DR = 286
Yor1 miss 0 orf −1.9 int 11.4 rank 307
yor1−dF miss 0 orf 1.7 int 5.1 rank 593

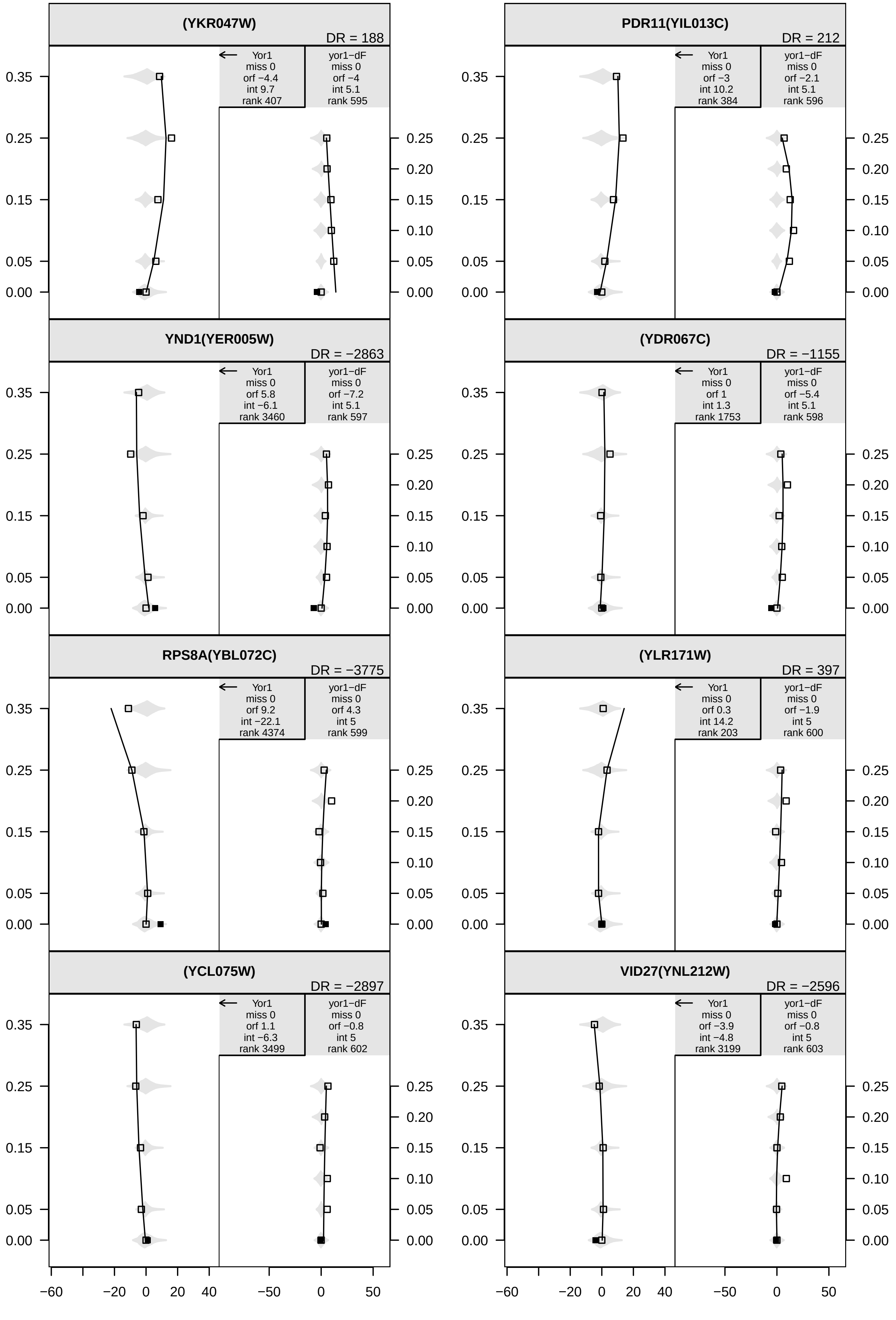

(YKR047W)
DR = 188
Yor1 miss 0 orf −4.4 int 9.7 rank 407
yor1−dF miss 0 orf −4 int 5.1 rank 595
PDR11(YIL013C)
DR = 212
Yor1 miss 0 orf −3 int 10.2 rank 384
yor1−dF miss 0 orf −2.1 int 5.1 rank 596
YND1(YER005W)
DR = −2863
Yor1 miss 0 orf 5.8 int −6.1 rank 3460
yor1−dF miss 0 orf −7.2 int 5.1 rank 597
(YDR067C)
DR = −1155
Yor1 miss 0 orf 1 int 1.3 rank 1753
yor1−dF miss 0 orf −5.4 int 5.1 rank 598
RPS8A(YBL072C)
DR = −3775
Yor1 miss 0 orf 9.2 int −22.1 rank 4374
yor1−dF miss 0 orf 4.3 int 5 rank 599
(YLR171W)
DR = 397
Yor1 miss 0 orf 0.3 int 14.2 rank 203
yor1−dF miss 0 orf −1.9 int 5 rank 600
(YCL075W)
DR = −2897
Yor1 miss 0 orf 1.1 int −6.3 rank 3499
yor1−dF miss 0 orf −0.8 int 5 rank 602
VID27(YNL212W)
DR = −2596
Yor1 miss 0 orf −3.9 int −4.8 rank 3199
yor1−dF miss 0 orf −0.8 int 5 rank 603
0.00 0.05 0.10 0.15 0.20 0.25 0.35
−60 −20 0 20 40 −50 0 50

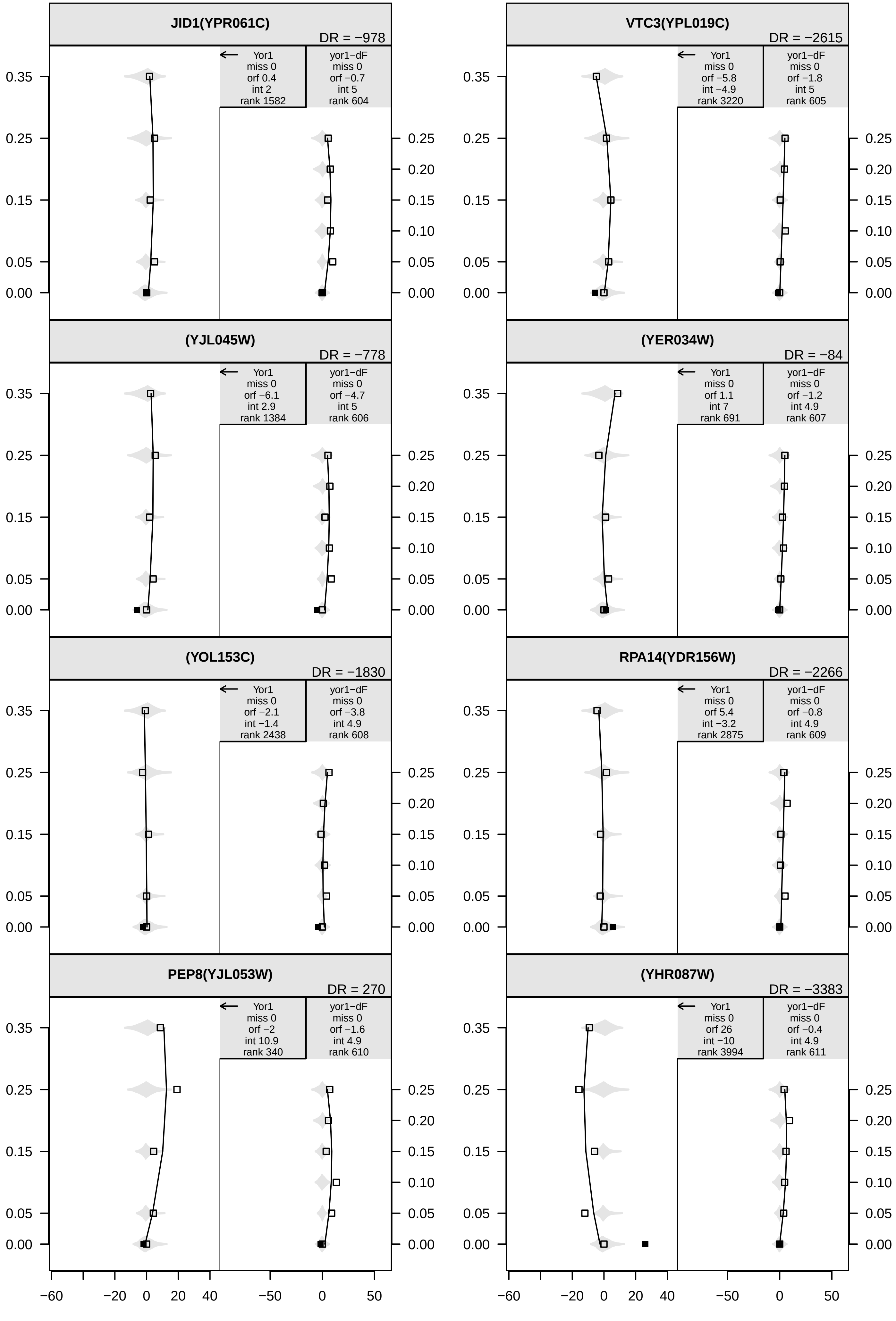
JID1(YPR061C)
DR = −978
Yor1 miss 0 orf 0.4 int 2 rank 1582
yor1−dF miss 0 orf −0.7 int 5 rank 604
VTC3(YPL019C)
DR = −2615
Yor1 miss 0 orf −5.8 int −4.9 rank 3220
yor1−dF miss 0 orf −1.8 int 5 rank 605
(YJL045W)
DR = −778
Yor1 miss 0 orf −6.1 int 2.9 rank 1384
yor1−dF miss 0 orf −4.7 int 5 rank 606
(YER034W)
DR = −84
Yor1 miss 0 orf 1.1 int 7 rank 691
yor1−dF miss 0 orf −1.2 int 4.9 rank 607
(YOL153C)
DR = −1830
Yor1 miss 0 orf −2.1 int −1.4 rank 2438
yor1−dF miss 0 orf −3.8 int 4.9 rank 608
RPA14(YDR156W)
DR = −2266
Yor1 miss 0 orf 5.4 int −3.2 rank 2875
yor1−dF miss 0 orf −0.8 int 4.9 rank 609
PEP8(YJL053W)
DR = 270
Yor1 miss 0 orf −2 int 10.9 rank 340
yor1−dF miss 0 orf −1.6 int 4.9 rank 610
(YHR087W)
DR = −3383
Yor1 miss 0 orf 26 int −10 rank 3994
yor1−dF miss 0 orf −0.4 int 4.9 rank 611

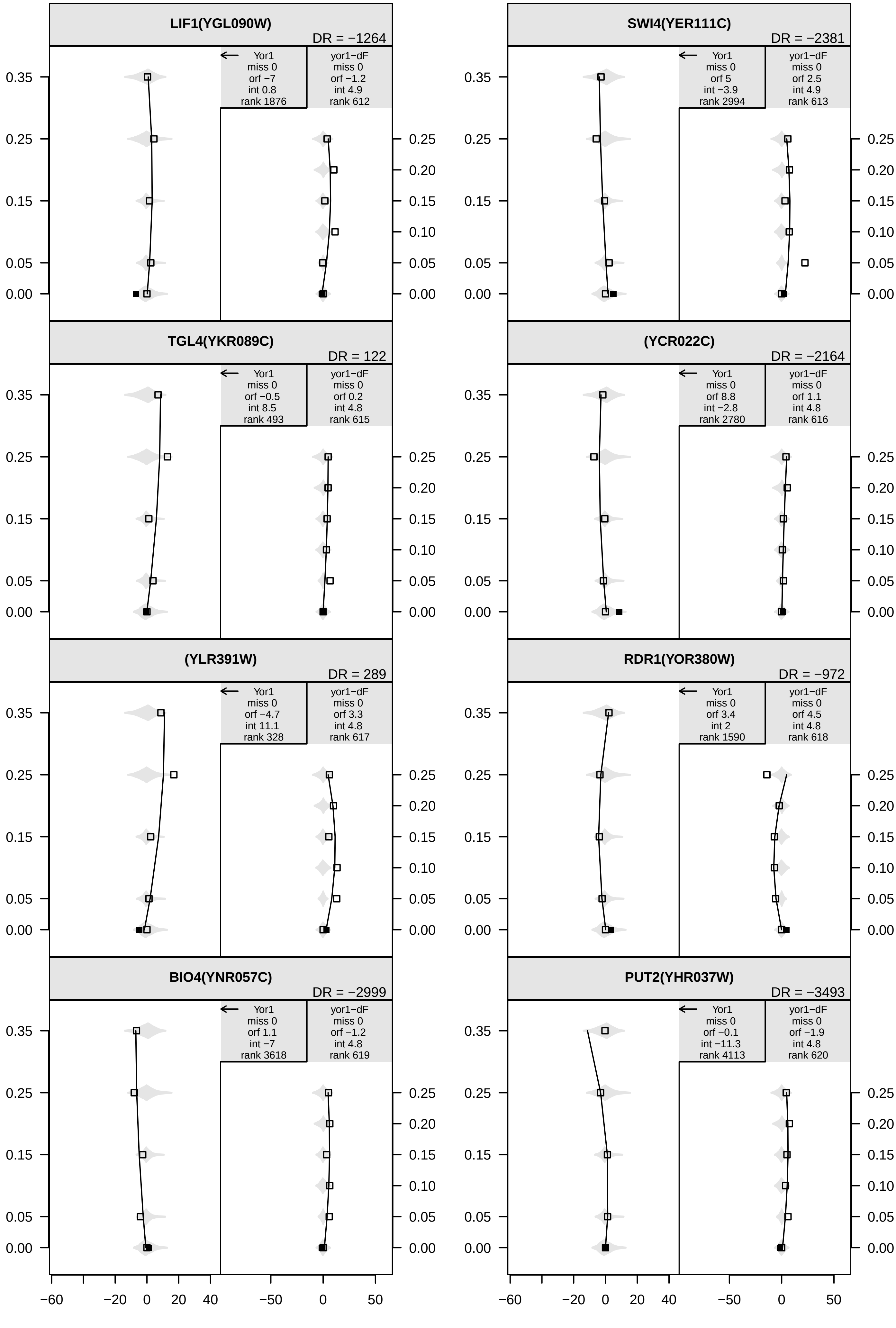

LIF1(YGL090W)
DR = −1264
Yor1 miss 0 orf −7 int 0.8 rank 1876
yor1−dF miss 0 orf −1.2 int 4.9 rank 612
SWI4(YER111C)
DR = −2381
Yor1 miss 0 orf 5 int −3.9 rank 2994
yor1−dF miss 0 orf 2.5 int 4.9 rank 613
TGL4(YKR089C)
DR = 122
Yor1 miss 0 orf −0.5 int 8.5 rank 493
yor1−dF miss 0 orf 0.2 int 4.8 rank 615
(YCR022C)
DR = −2164
Yor1 miss 0 orf 8.8 int −2.8 rank 2780
yor1−dF miss 0 orf 1.1 int 4.8 rank 616
(YLR391W)
DR = 289
Yor1 miss 0 orf −4.7 int 11.1 rank 328
yor1−dF miss 0 orf 3.3 int 4.8 rank 617
RDR1(YOR380W)
DR = −972
Yor1 miss 0 orf 3.4 int 2 rank 1590
yor1−dF miss 0 orf 4.5 int 4.8 rank 618
BIO4(YNR057C)
DR = −2999
Yor1 miss 0 orf 1.1 int −7 rank 3618
yor1−dF miss 0 orf −1.2 int 4.8 rank 619
PUT2(YHR037W)
DR = −3493
Yor1 miss 0 orf −0.1 int −11.3 rank 4113
yor1−dF miss 0 orf −1.9 int 4.8 rank 620
0.35 0.25 0.15 0.05 0.00
0.25 0.20 0.15 0.10 0.05 0.00
−60 −20 0 20 40 −50 0 50

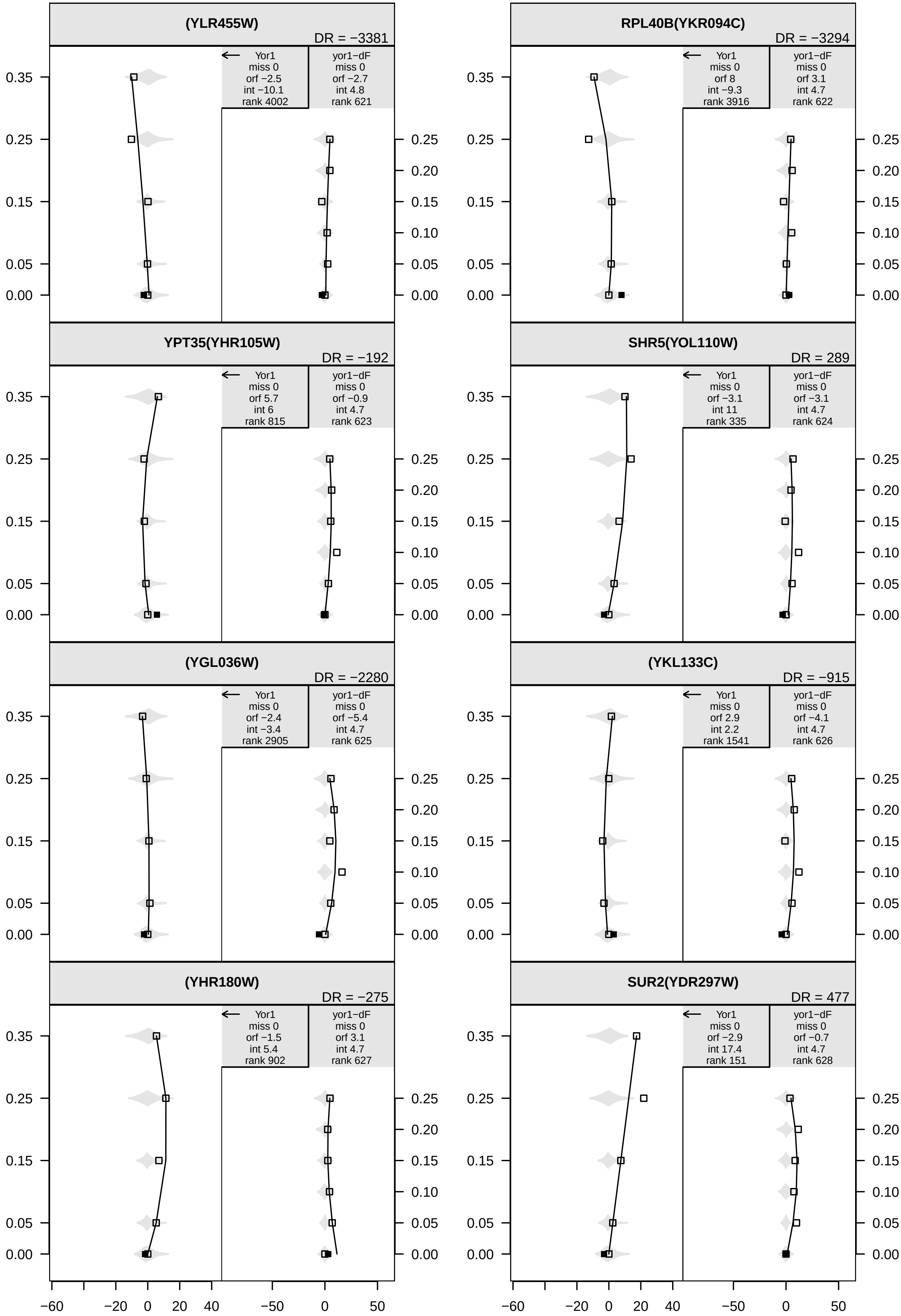
(YLR455W)
DR = −3381
Yor1
miss 0
orf −2.5
int −10.1
rank 4002
yor1−dF
miss 0
orf −2.7
int 4.8
rank 621
RPL40B(YKR094C)
DR = −3294
Yor1
miss 0
orf 8
int −9.3
rank 3916
yor1−dF
miss 0
orf 3.1
int 4.7
rank 622
YPT35(YHR105W)
DR = −192
Yor1
miss 0
orf 5.7
int 6
rank 815
yor1−dF
miss 0
orf −0.9
int 4.7
rank 623
SHR5(YOL110W)
DR = 289
Yor1
miss 0
orf −3.1
int 11
rank 335
yor1−dF
miss 0
orf −3.1
int 4.7
rank 624
(YGL036W)
DR = −2280
Yor1
miss 0
orf −2.4
int −3.4
rank 2905
yor1−dF
miss 0
orf −5.4
int 4.7
rank 625
(YKL133C)
DR = −915
Yor1
miss 0
orf 2.9
int 2.2
rank 1541
yor1−dF
miss 0
orf −4.1
int 4.7
rank 626
(YHR180W)
DR = −275
Yor1
miss 0
orf −1.5
int 5.4
rank 902
yor1−dF
miss 0
orf 3.1
int 4.7
rank 627
SUR2(YDR297W)
DR = 477
Yor1
miss 0
orf −2.9
int 17.4
rank 151
yor1−dF
miss 0
orf −0.7
int 4.7
rank 628
0.35
0.25
0.20
0.15
0.10
0.05
0.00
−60
−20
0
20
40
−50
0
50

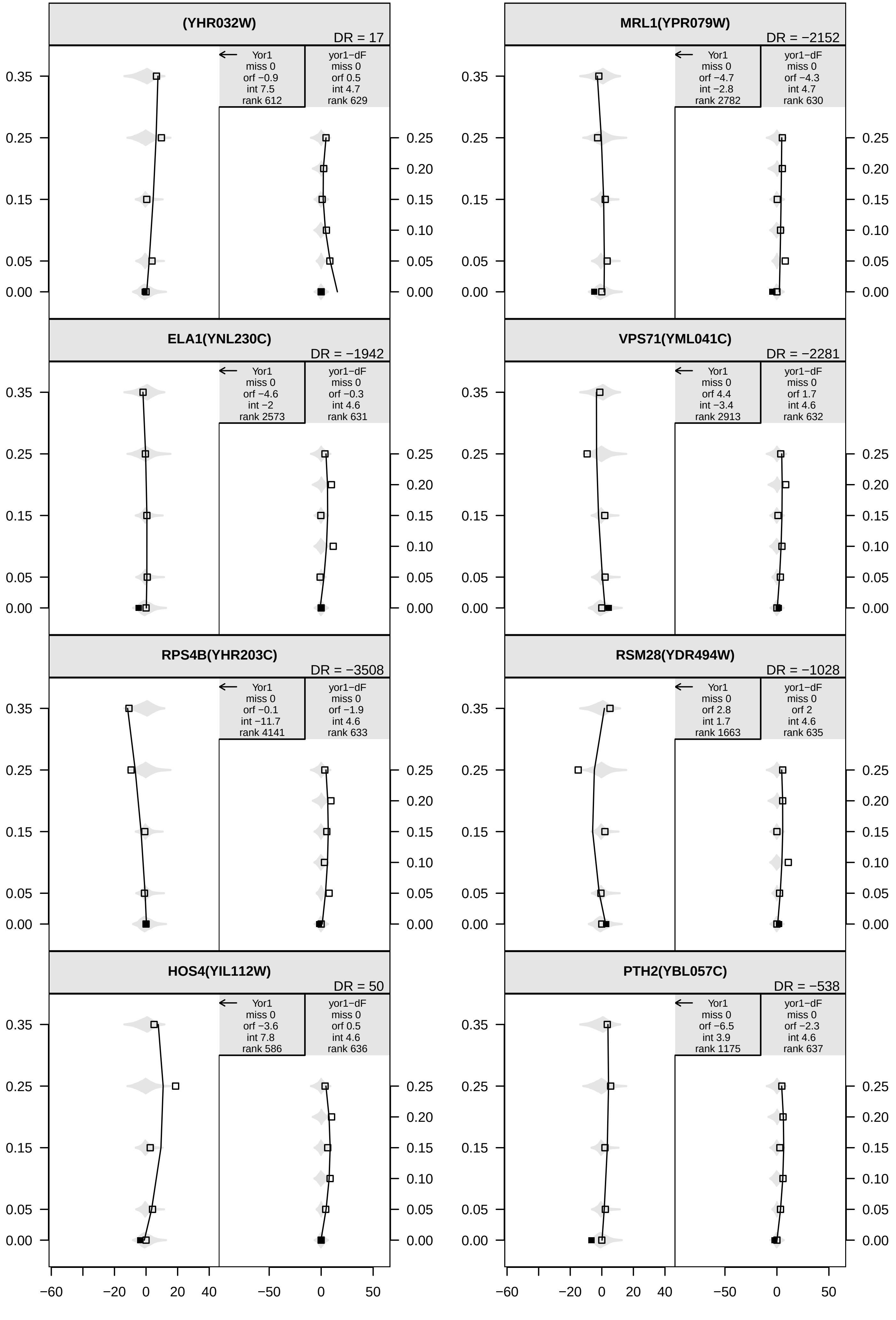

(YHR032W)
DR = 17
Yor1 miss 0 orf −0.9 int 7.5 rank 612
yor1−dF miss 0 orf 0.5 int 4.7 rank 629
MRL1(YPR079W)
DR = −2152
Yor1 miss 0 orf −4.7 int −2.8 rank 2782
yor1−dF miss 0 orf −4.3 int 4.7 rank 630
ELA1(YNL230C)
DR = −1942
Yor1 miss 0 orf −4.6 int −2 rank 2573
yor1−dF miss 0 orf −0.3 int 4.6 rank 631
VPS71(YML041C)
DR = −2281
Yor1 miss 0 orf 4.4 int −3.4 rank 2913
yor1−dF miss 0 orf 1.7 int 4.6 rank 632
RPS4B(YHR203C)
DR = −3508
Yor1 miss 0 orf −0.1 int −11.7 rank 4141
yor1−dF miss 0 orf −1.9 int 4.6 rank 633
RSM28(YDR494W)
DR = −1028
Yor1 miss 0 orf 2.8 int 1.7 rank 1663
yor1−dF miss 0 orf 2 int 4.6 rank 635
HOS4(YIL112W)
DR = 50
Yor1 miss 0 orf −3.6 int 7.8 rank 586
yor1−dF miss 0 orf 0.5 int 4.6 rank 636
PTH2(YBL057C)
DR = −538
Yor1 miss 0 orf −6.5 int 3.9 rank 1175
yor1−dF miss 0 orf −2.3 int 4.6 rank 637

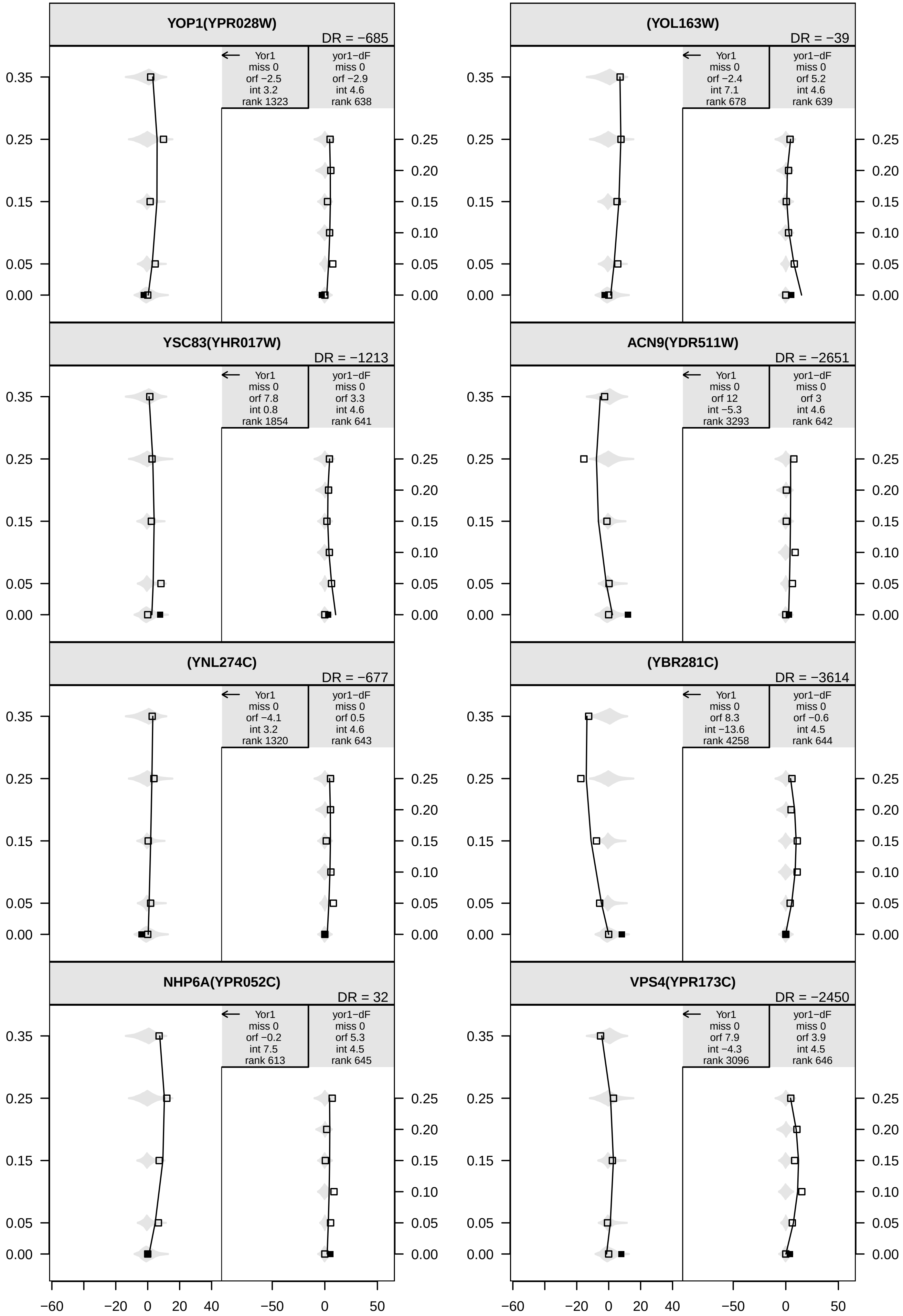

YOP1(YPR028W)
DR = −685
Yor1 miss 0 orf −2.5 int 3.2 rank 1323
yor1−dF miss 0 orf −2.9 int 4.6 rank 638
(YOL163W)
DR = −39
Yor1 miss 0 orf −2.4 int 7.1 rank 678
yor1−dF miss 0 orf 5.2 int 4.6 rank 639
YSC83(YHR017W)
DR = −1213
Yor1 miss 0 orf 7.8 int 0.8 rank 1854
yor1−dF miss 0 orf 3.3 int 4.6 rank 641
ACN9(YDR511W)
DR = −2651
Yor1 miss 0 orf 12 int −5.3 rank 3293
yor1−dF miss 0 orf 3 int 4.6 rank 642
(YNL274C)
DR = −677
Yor1 miss 0 orf −4.1 int 3.2 rank 1320
yor1−dF miss 0 orf 0.5 int 4.6 rank 643
(YBR281C)
DR = −3614
Yor1 miss 0 orf 8.3 int −13.6 rank 4258
yor1−dF miss 0 orf −0.6 int 4.5 rank 644
NHP6A(YPR052C)
DR = 32
Yor1 miss 0 orf −0.2 int 7.5 rank 613
yor1−dF miss 0 orf 5.3 int 4.5 rank 645
VPS4(YPR173C)
DR = −2450
Yor1 miss 0 orf 7.9 int −4.3 rank 3096
yor1−dF miss 0 orf 3.9 int 4.5 rank 646
0.35 0.25 0.20 0.15 0.10 0.05 0.00
−60 −20 0 20 40 −50 0 50

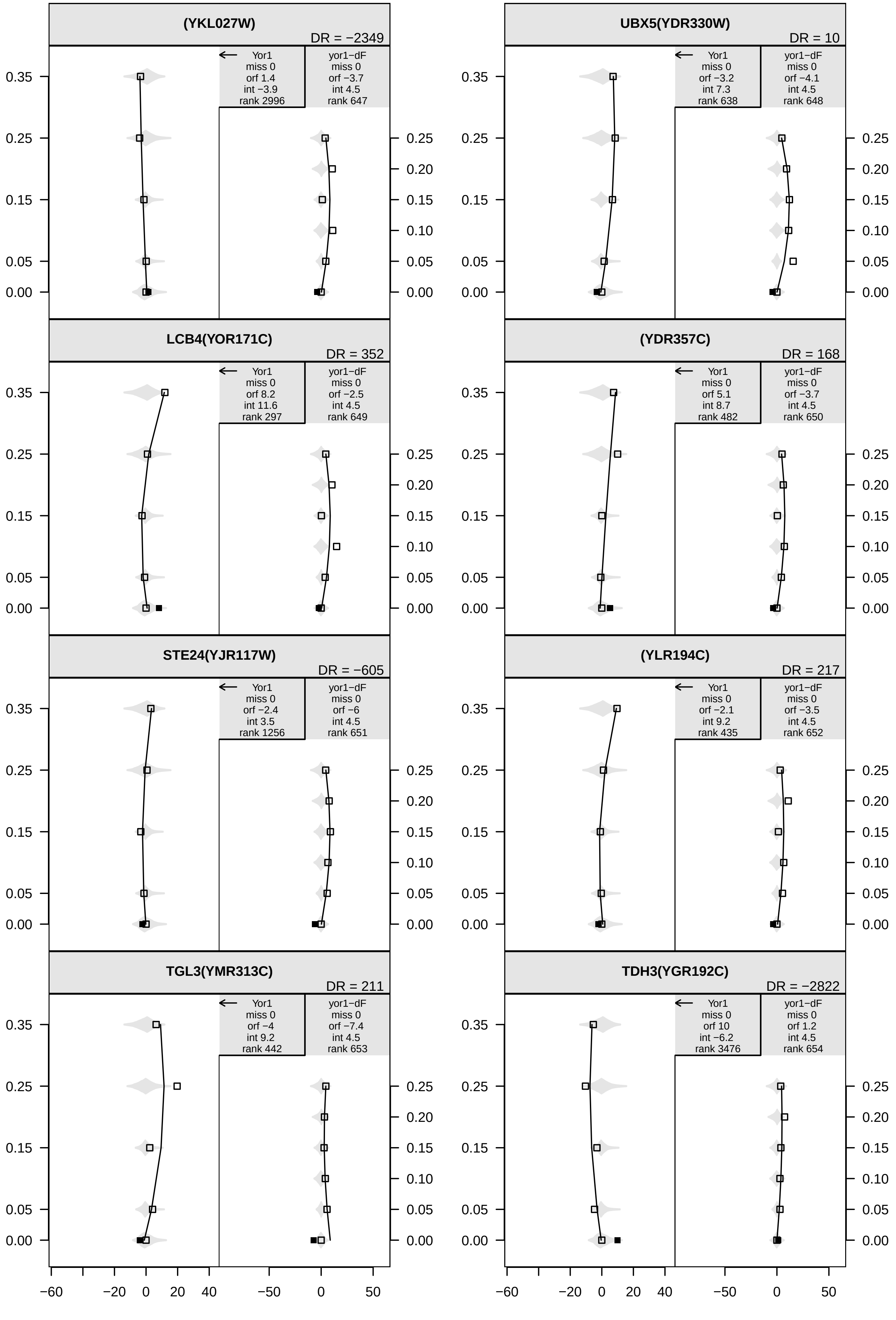
(YKL027W)
DR = −2349
Yor1 miss 0 orf 1.4 int −3.9 rank 2996
yor1−dF miss 0 orf −3.7 int 4.5 rank 647
UBX5(YDR330W)
DR = 10
Yor1 miss 0 orf −3.2 int 7.3 rank 638
yor1−dF miss 0 orf −4.1 int 4.5 rank 648
LCB4(YOR171C)
DR = 352
Yor1 miss 0 orf 8.2 int 11.6 rank 297
yor1−dF miss 0 orf −2.5 int 4.5 rank 649
(YDR357C)
DR = 168
Yor1 miss 0 orf 5.1 int 8.7 rank 482
yor1−dF miss 0 orf −3.7 int 4.5 rank 650
STE24(YJR117W)
DR = −605
Yor1 miss 0 orf −2.4 int 3.5 rank 1256
yor1−dF miss 0 orf −6 int 4.5 rank 651
(YLR194C)
DR = 217
Yor1 miss 0 orf −2.1 int 9.2 rank 435
yor1−dF miss 0 orf −3.5 int 4.5 rank 652
TGL3(YMR313C)
DR = 211
Yor1 miss 0 orf −4 int 9.2 rank 442
yor1−dF miss 0 orf −7.4 int 4.5 rank 653
TDH3(YGR192C)
DR = −2822
Yor1 miss 0 orf 10 int −6.2 rank 3476
yor1−dF miss 0 orf 1.2 int 4.5 rank 654
0.00 0.05 0.10 0.15 0.20 0.25 0.35
−60 −20 0 20 40 −50 0 50

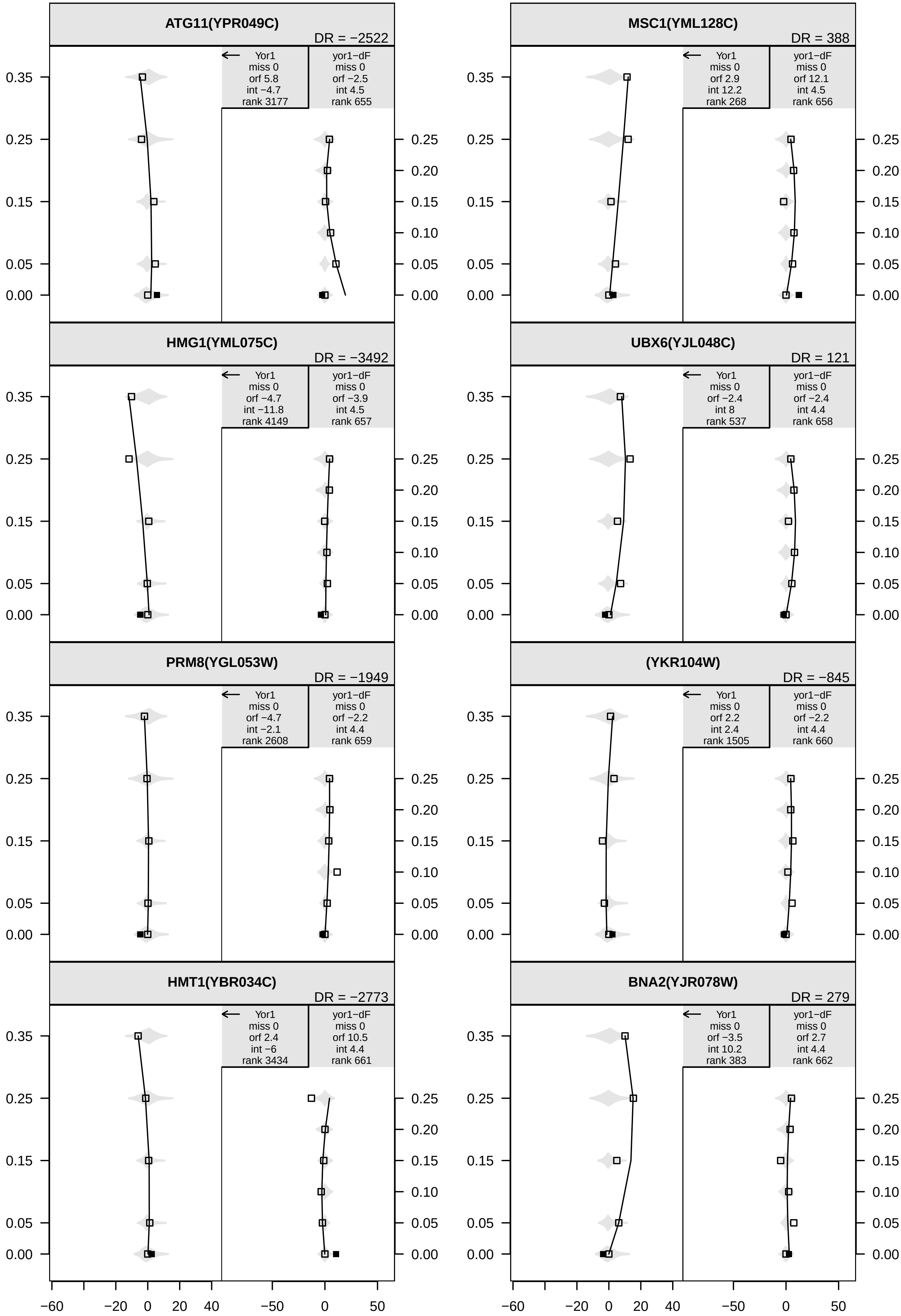
ATG11(YPR049C)
DR = −2522
Yor1 miss 0 orf 5.8 int −4.7 rank 3177
yor1−dF miss 0 orf −2.5 int 4.5 rank 655
MSC1(YML128C)
DR = 388
Yor1 miss 0 orf 2.9 int 12.2 rank 268
yor1−dF miss 0 orf 12.1 int 4.5 rank 656
HMG1(YML075C)
DR = −3492
Yor1 miss 0 orf −4.7 int −11.8 rank 4149
yor1−dF miss 0 orf −3.9 int 4.5 rank 657
UBX6(YJL048C)
DR = 121
Yor1 miss 0 orf −2.4 int 8 rank 537
yor1−dF miss 0 orf −2.4 int 4.4 rank 658
PRM8(YGL053W)
DR = −1949
Yor1 miss 0 orf −4.7 int −2.1 rank 2608
yor1−dF miss 0 orf −2.2 int 4.4 rank 659
(YKR104W)
DR = −845
Yor1 miss 0 orf 2.2 int 2.4 rank 1505
yor1−dF miss 0 orf −2.2 int 4.4 rank 660
HMT1(YBR034C)
DR = −2773
Yor1 miss 0 orf 2.4 int −6 rank 3434
yor1−dF miss 0 orf 10.5 int 4.4 rank 661
BNA2(YJR078W)
DR = 279
Yor1 miss 0 orf −3.5 int 10.2 rank 383
yor1−dF miss 0 orf 2.7 int 4.4 rank 662
0.35 0.25 0.20 0.15 0.10 0.05 0.00
−60 −20 0 20 40 −50 0 50

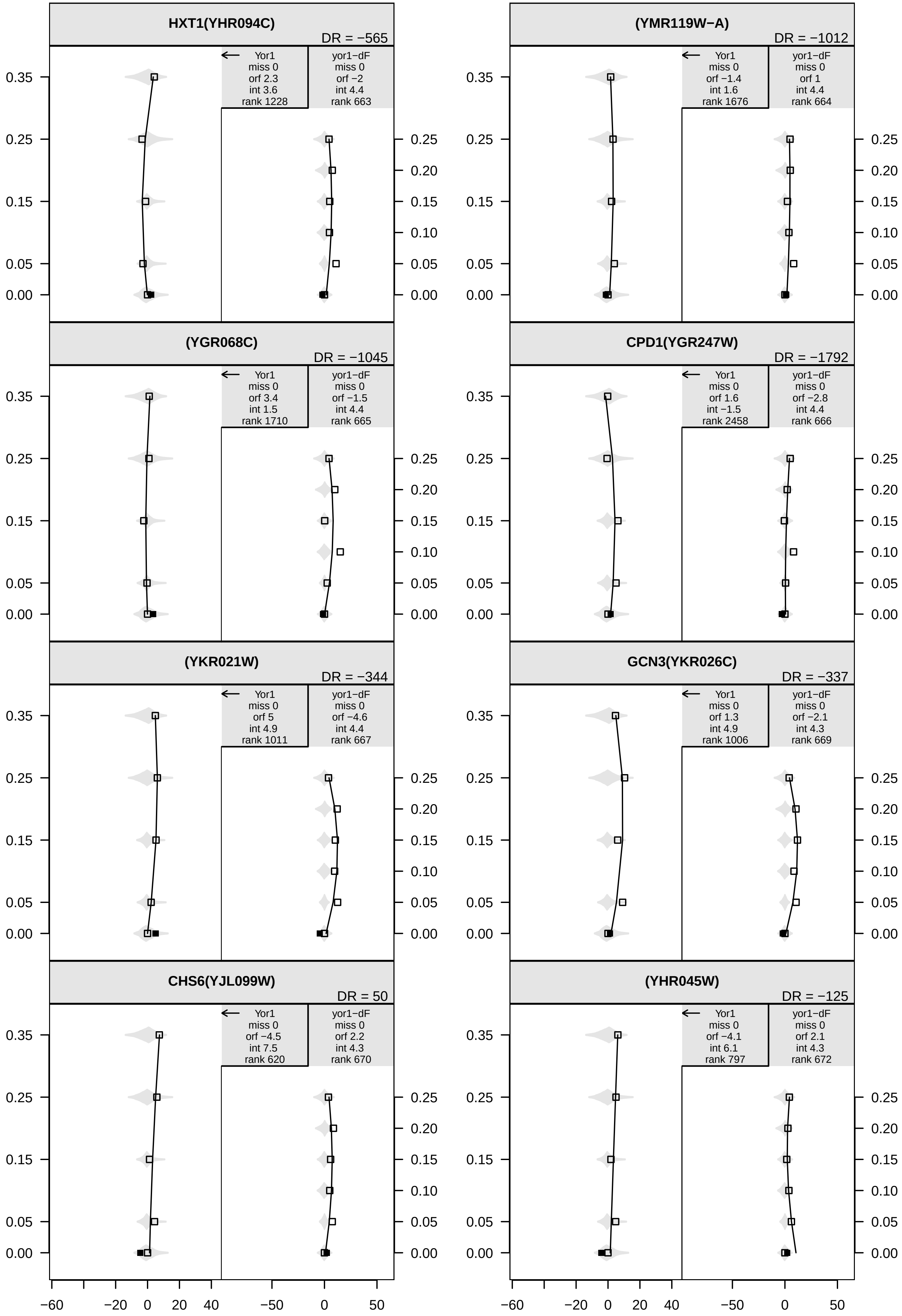
HXT1(YHR094C)
DR = −565
Yor1 miss 0 orf 2.3 int 3.6 rank 1228
yor1−dF miss 0 orf −2 int 4.4 rank 663
(YMR119W−A)
DR = −1012
Yor1 miss 0 orf −1.4 int 1.6 rank 1676
yor1−dF miss 0 orf 1 int 4.4 rank 664
(YGR068C)
DR = −1045
Yor1 miss 0 orf 3.4 int 1.5 rank 1710
yor1−dF miss 0 orf −1.5 int 4.4 rank 665
CPD1(YGR247W)
DR = −1792
Yor1 miss 0 orf 1.6 int −1.5 rank 2458
yor1−dF miss 0 orf −2.8 int 4.4 rank 666
(YKR021W)
DR = −344
Yor1 miss 0 orf 5 int 4.9 rank 1011
yor1−dF miss 0 orf −4.6 int 4.4 rank 667
GCN3(YKR026C)
DR = −337
Yor1 miss 0 orf 1.3 int 4.9 rank 1006
yor1−dF miss 0 orf −2.1 int 4.3 rank 669
CHS6(YJL099W)
DR = 50
Yor1 miss 0 orf −4.5 int 7.5 rank 620
yor1−dF miss 0 orf 2.2 int 4.3 rank 670
(YHR045W)
DR = −125
Yor1 miss 0 orf −4.1 int 6.1 rank 797
yor1−dF miss 0 orf 2.1 int 4.3 rank 672
0.35 0.25 0.20 0.15 0.10 0.05 0.00
−60 −20 0 20 40 −50 0 50

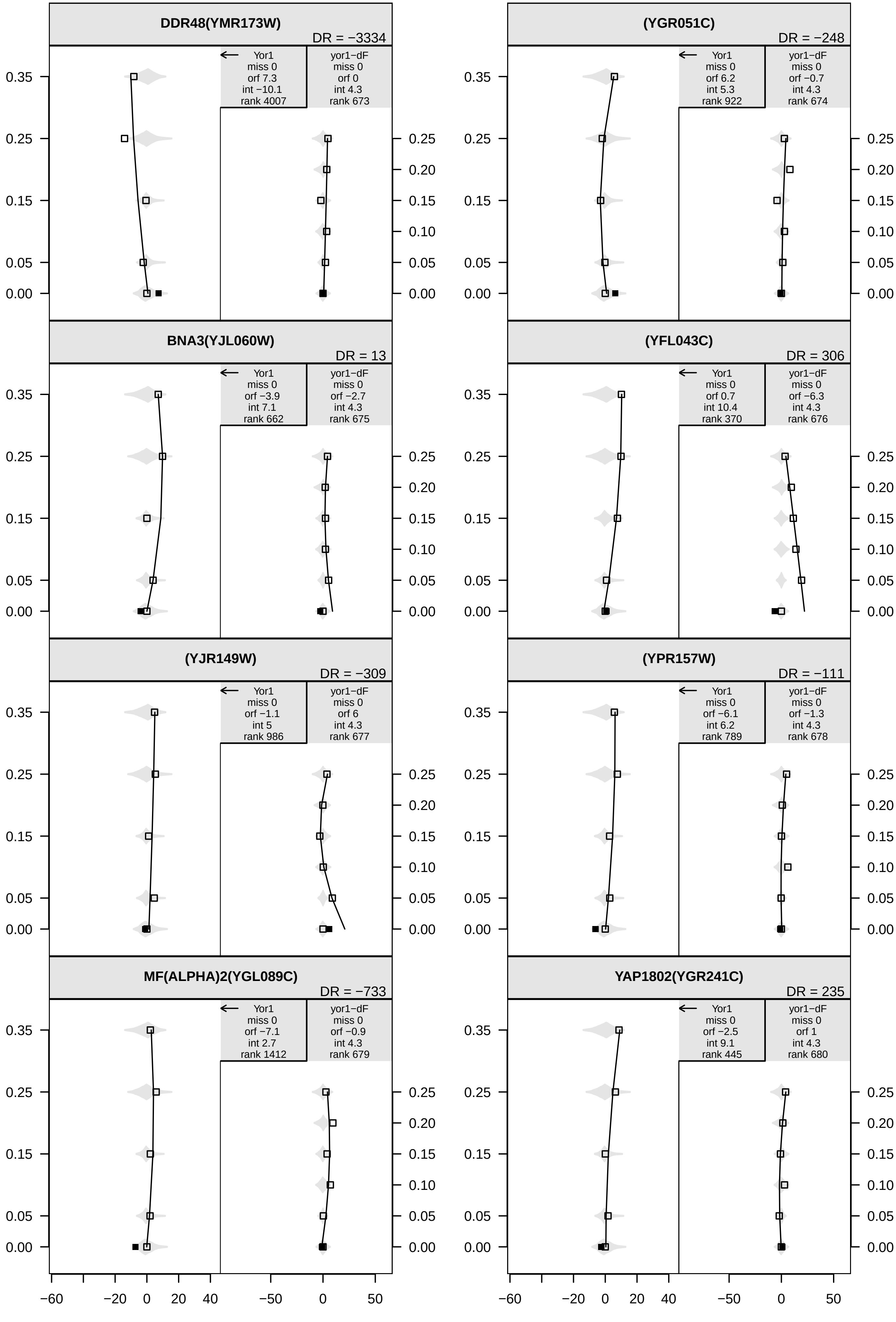

DDR48(YMR173W)
DR = −3334
Yor1 miss 0 orf 7.3 int −10.1 rank 4007
yor1−dF miss 0 orf 0 int 4.3 rank 673
(YGR051C)
DR = −248
Yor1 miss 0 orf 6.2 int 5.3 rank 922
yor1−dF miss 0 orf −0.7 int 4.3 rank 674
BNA3(YJL060W)
DR = 13
Yor1 miss 0 orf −3.9 int 7.1 rank 662
yor1−dF miss 0 orf −2.7 int 4.3 rank 675
(YFL043C)
DR = 306
Yor1 miss 0 orf 0.7 int 10.4 rank 370
yor1−dF miss 0 orf −6.3 int 4.3 rank 676
(YJR149W)
DR = −309
Yor1 miss 0 orf −1.1 int 5 rank 986
yor1−dF miss 0 orf 6 int 4.3 rank 677
(YPR157W)
DR = −111
Yor1 miss 0 orf −6.1 int 6.2 rank 789
yor1−dF miss 0 orf −1.3 int 4.3 rank 678
MF(ALPHA)2(YGL089C)
DR = −733
Yor1 miss 0 orf −7.1 int 2.7 rank 1412
yor1−dF miss 0 orf −0.9 int 4.3 rank 679
YAP1802(YGR241C)
DR = 235
Yor1 miss 0 orf −2.5 int 9.1 rank 445
yor1−dF miss 0 orf 1 int 4.3 rank 680
0.35 0.25 0.15 0.05 0.00
0.25 0.20 0.15 0.10 0.05 0.00
−60 −20 0 20 40 −50 0 50

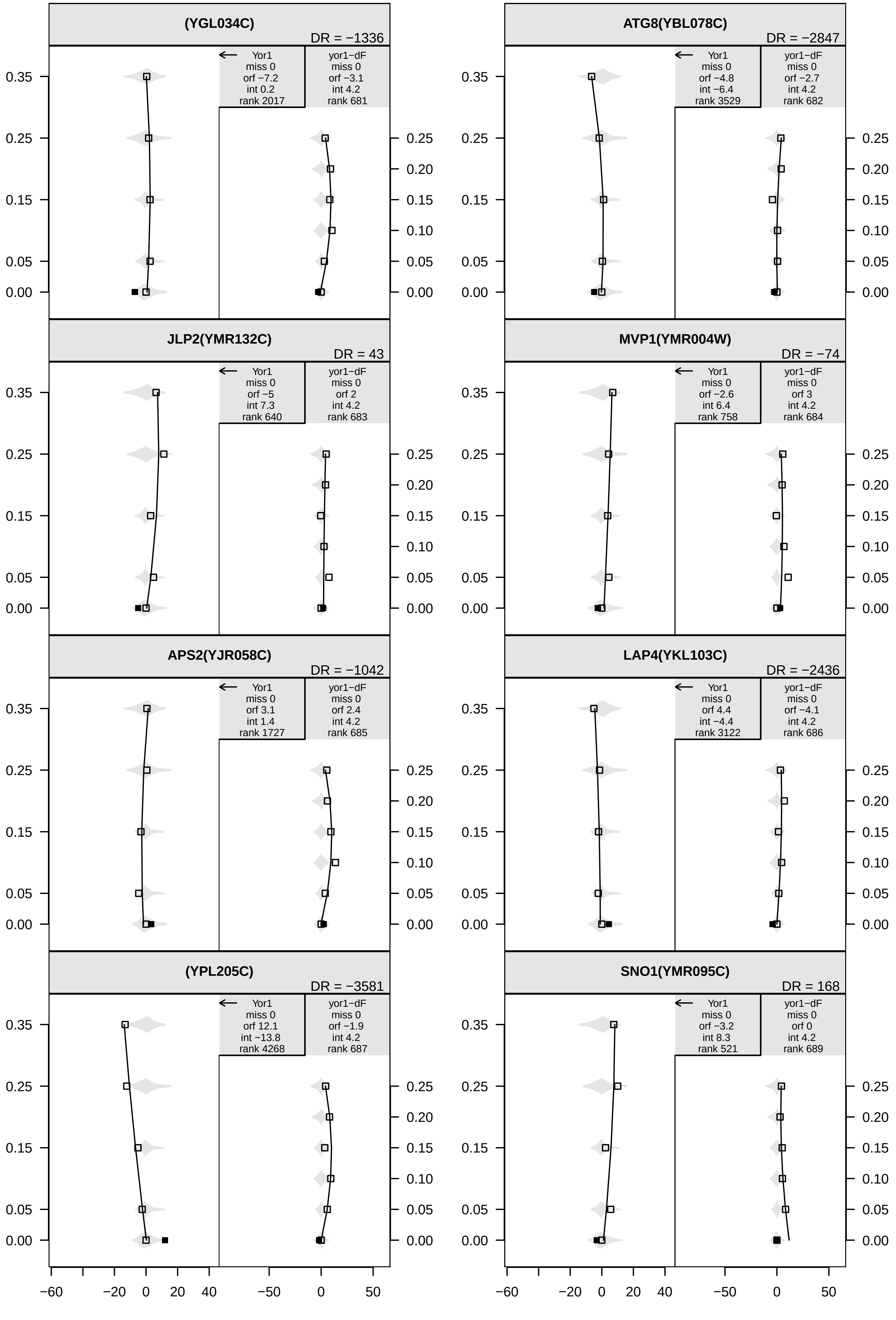
(YGL034C)
DR = −1336
Yor1
miss 0
orf −7.2
int 0.2
rank 2017
yor1−dF
miss 0
orf −3.1
int 4.2
rank 681
ATG8(YBL078C)
DR = −2847
Yor1
miss 0
orf −4.8
int −6.4
rank 3529
yor1−dF
miss 0
orf −2.7
int 4.2
rank 682
JLP2(YMR132C)
DR = 43
Yor1
miss 0
orf −5
int 7.3
rank 640
yor1−dF
miss 0
orf 2
int 4.2
rank 683
MVP1(YMR004W)
DR = −74
Yor1
miss 0
orf −2.6
int 6.4
rank 758
yor1−dF
miss 0
orf 3
int 4.2
rank 684
APS2(YJR058C)
DR = −1042
Yor1
miss 0
orf 3.1
int 1.4
rank 1727
yor1−dF
miss 0
orf 2.4
int 4.2
rank 685
LAP4(YKL103C)
DR = −2436
Yor1
miss 0
orf 4.4
int −4.4
rank 3122
yor1−dF
miss 0
orf −4.1
int 4.2
rank 686
(YPL205C)
DR = −3581
Yor1
miss 0
orf 12.1
int −13.8
rank 4268
yor1−dF
miss 0
orf −1.9
int 4.2
rank 687
SNO1(YMR095C)
DR = 168
Yor1
miss 0
orf −3.2
int 8.3
rank 521
yor1−dF
miss 0
orf 0
int 4.2
rank 689
0.35
0.25
0.20
0.15
0.10
0.05
0.00
−60
−20
0
20
40
−50
0
50

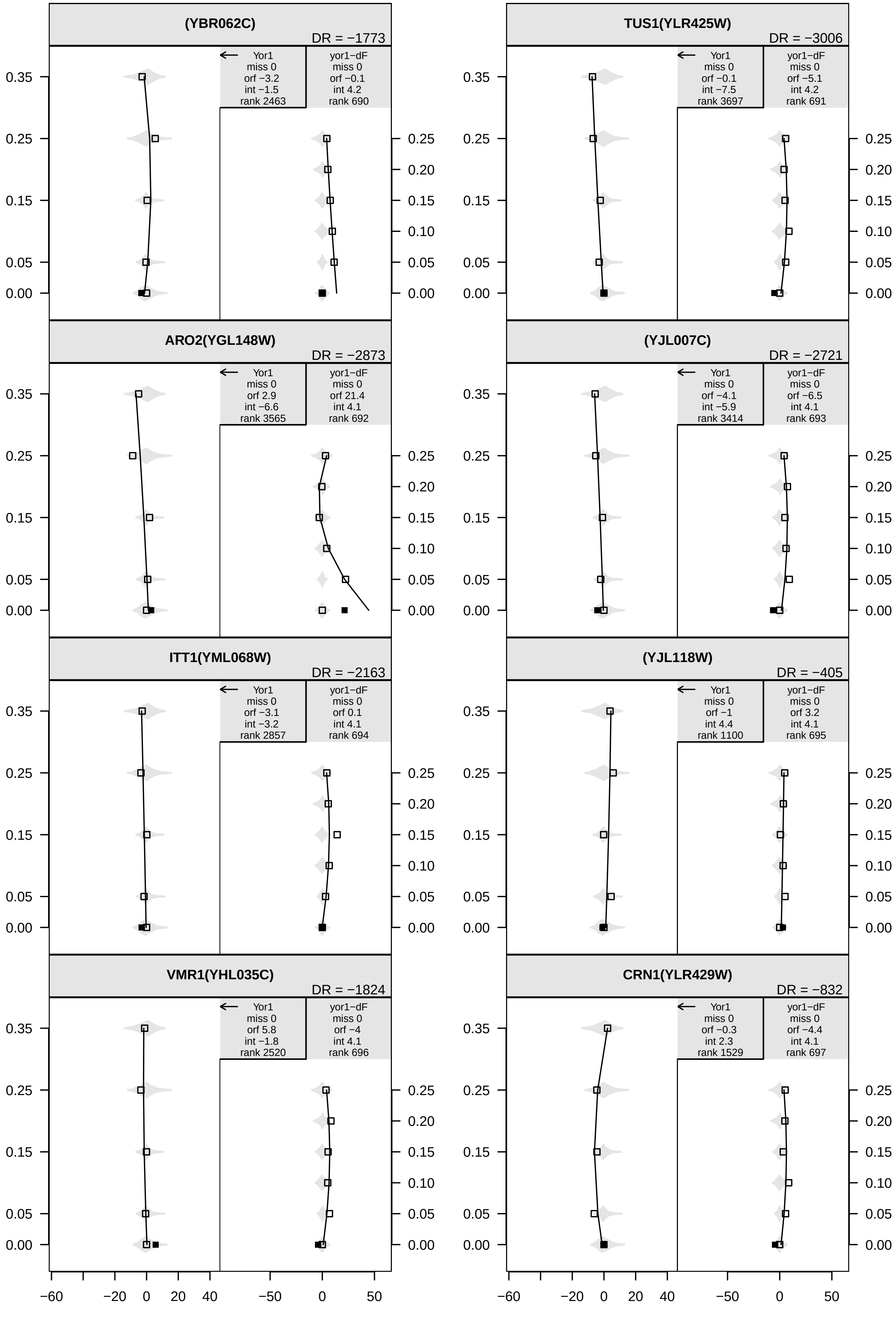
(YBR062C)
DR = −1773
Yor1
miss 0
orf −3.2
int −1.5
rank 2463
yor1−dF
miss 0
orf −0.1
int 4.2
rank 690
TUS1(YLR425W)
DR = −3006
Yor1
miss 0
orf −0.1
int −7.5
rank 3697
yor1−dF
miss 0
orf −5.1
int 4.2
rank 691
ARO2(YGL148W)
DR = −2873
Yor1
miss 0
orf 2.9
int −6.6
rank 3565
yor1−dF
miss 0
orf 21.4
int 4.1
rank 692
(YJL007C)
DR = −2721
Yor1
miss 0
orf −4.1
int −5.9
rank 3414
yor1−dF
miss 0
orf −6.5
int 4.1
rank 693
ITT1(YML068W)
DR = −2163
Yor1
miss 0
orf −3.1
int −3.2
rank 2857
yor1−dF
miss 0
orf 0.1
int 4.1
rank 694
(YJL118W)
DR = −405
Yor1
miss 0
orf −1
int 4.4
rank 1100
yor1−dF
miss 0
orf 3.2
int 4.1
rank 695
VMR1(YHL035C)
DR = −1824
Yor1
miss 0
orf 5.8
int −1.8
rank 2520
yor1−dF
miss 0
orf −4
int 4.1
rank 696
CRN1(YLR429W)
DR = −832
Yor1
miss 0
orf −0.3
int 2.3
rank 1529
yor1−dF
miss 0
orf −4.4
int 4.1
rank 697
0.35
0.25
0.20
0.15
0.10
0.05
0.00
−60
−20
0
20
40
−50
0
50

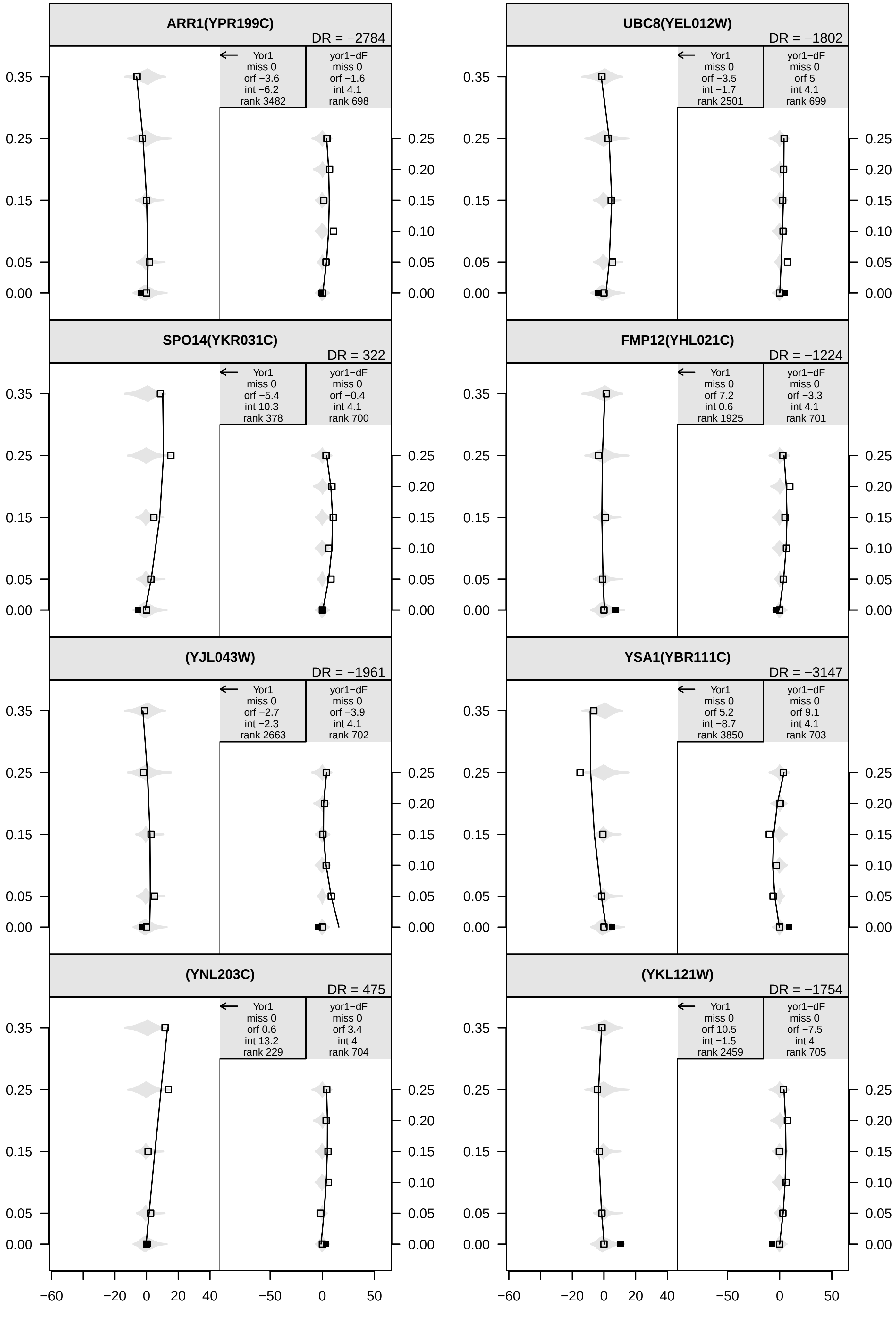

ARR1(YPR199C)
DR = −2784
Yor1 miss 0 orf −3.6 int −6.2 rank 3482
yor1−dF miss 0 orf −1.6 int 4.1 rank 698
UBC8(YEL012W)
DR = −1802
Yor1 miss 0 orf −3.5 int −1.7 rank 2501
yor1−dF miss 0 orf 5 int 4.1 rank 699
SPO14(YKR031C)
DR = 322
Yor1 miss 0 orf −5.4 int 10.3 rank 378
yor1−dF miss 0 orf −0.4 int 4.1 rank 700
FMP12(YHL021C)
DR = −1224
Yor1 miss 0 orf 7.2 int 0.6 rank 1925
yor1−dF miss 0 orf −3.3 int 4.1 rank 701
(YJL043W)
DR = −1961
Yor1 miss 0 orf −2.7 int −2.3 rank 2663
yor1−dF miss 0 orf −3.9 int 4.1 rank 702
YSA1(YBR111C)
DR = −3147
Yor1 miss 0 orf 5.2 int −8.7 rank 3850
yor1−dF miss 0 orf 9.1 int 4.1 rank 703
(YNL203C)
DR = 475
Yor1 miss 0 orf 0.6 int 13.2 rank 229
yor1−dF miss 0 orf 3.4 int 4 rank 704
(YKL121W)
DR = −1754
Yor1 miss 0 orf 10.5 int −1.5 rank 2459
yor1−dF miss 0 orf −7.5 int 4 rank 705
0.35
0.25
0.20
0.15
0.10
0.05
0.00
−60
−20
0
20
40
−50
0
50

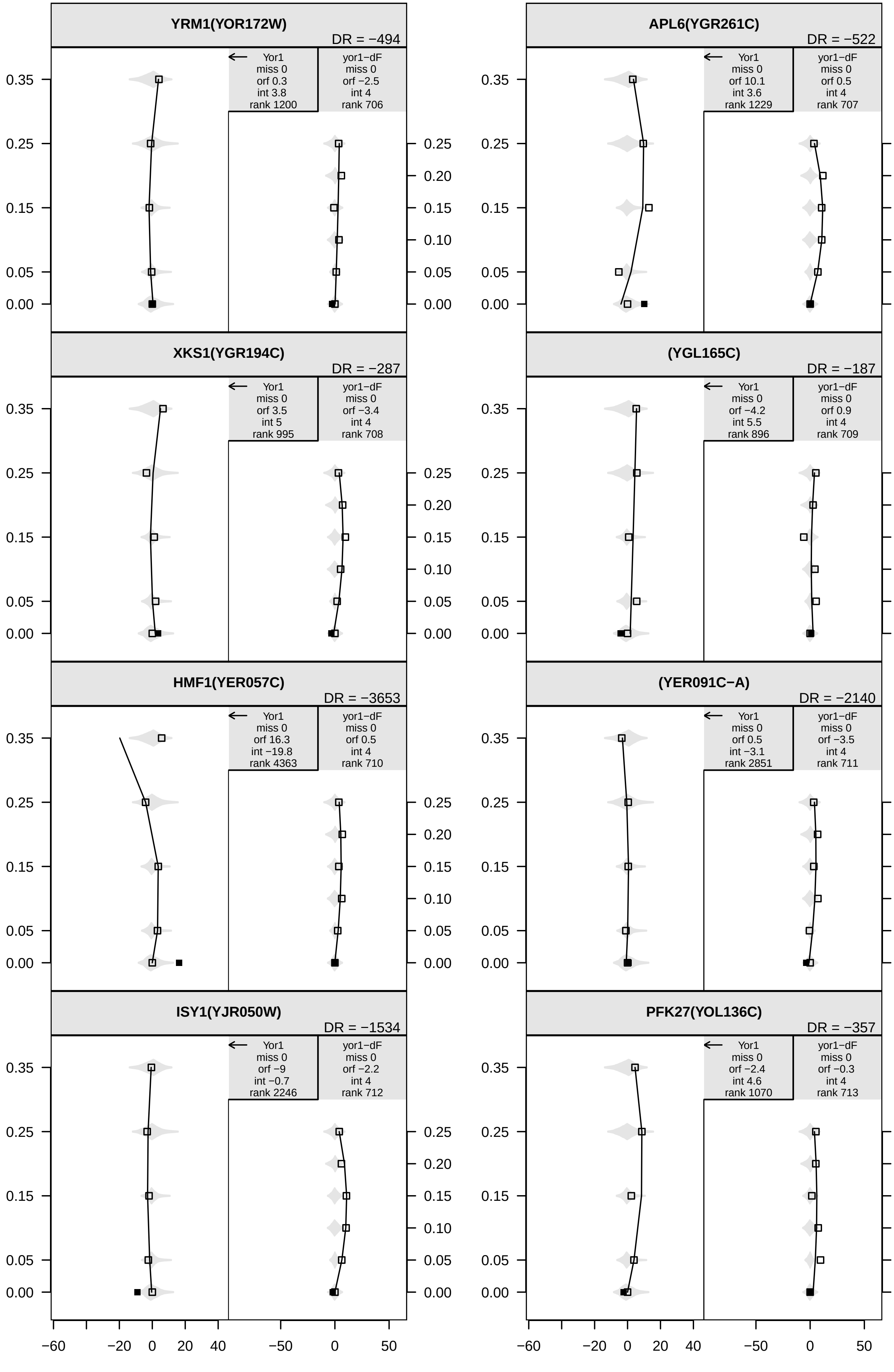

YRM1(YOR172W)
DR = −494
Yor1 miss 0 orf 0.3 int 3.8 rank 1200
yor1−dF miss 0 orf −2.5 int 4 rank 706
APL6(YGR261C)
DR = −522
Yor1 miss 0 orf 10.1 int 3.6 rank 1229
yor1−dF miss 0 orf 0.5 int 4 rank 707
XKS1(YGR194C)
DR = −287
Yor1 miss 0 orf 3.5 int 5 rank 995
yor1−dF miss 0 orf −3.4 int 4 rank 708
(YGL165C)
DR = −187
Yor1 miss 0 orf −4.2 int 5.5 rank 896
yor1−dF miss 0 orf 0.9 int 4 rank 709
HMF1(YER057C)
DR = −3653
Yor1 miss 0 orf 16.3 int −19.8 rank 4363
yor1−dF miss 0 orf 0.5 int 4 rank 710
(YER091C−A)
DR = −2140
Yor1 miss 0 orf 0.5 int −3.1 rank 2851
yor1−dF miss 0 orf −3.5 int 4 rank 711
ISY1(YJR050W)
DR = −1534
Yor1 miss 0 orf −9 int −0.7 rank 2246
yor1−dF miss 0 orf −2.2 int 4 rank 712
PFK27(YOL136C)
DR = −357
Yor1 miss 0 orf −2.4 int 4.6 rank 1070
yor1−dF miss 0 orf −0.3 int 4 rank 713
0.35 0.25 0.15 0.05 0.00
0.25 0.20 0.15 0.10 0.05 0.00
−60 −20 0 20 40
−50 0 50

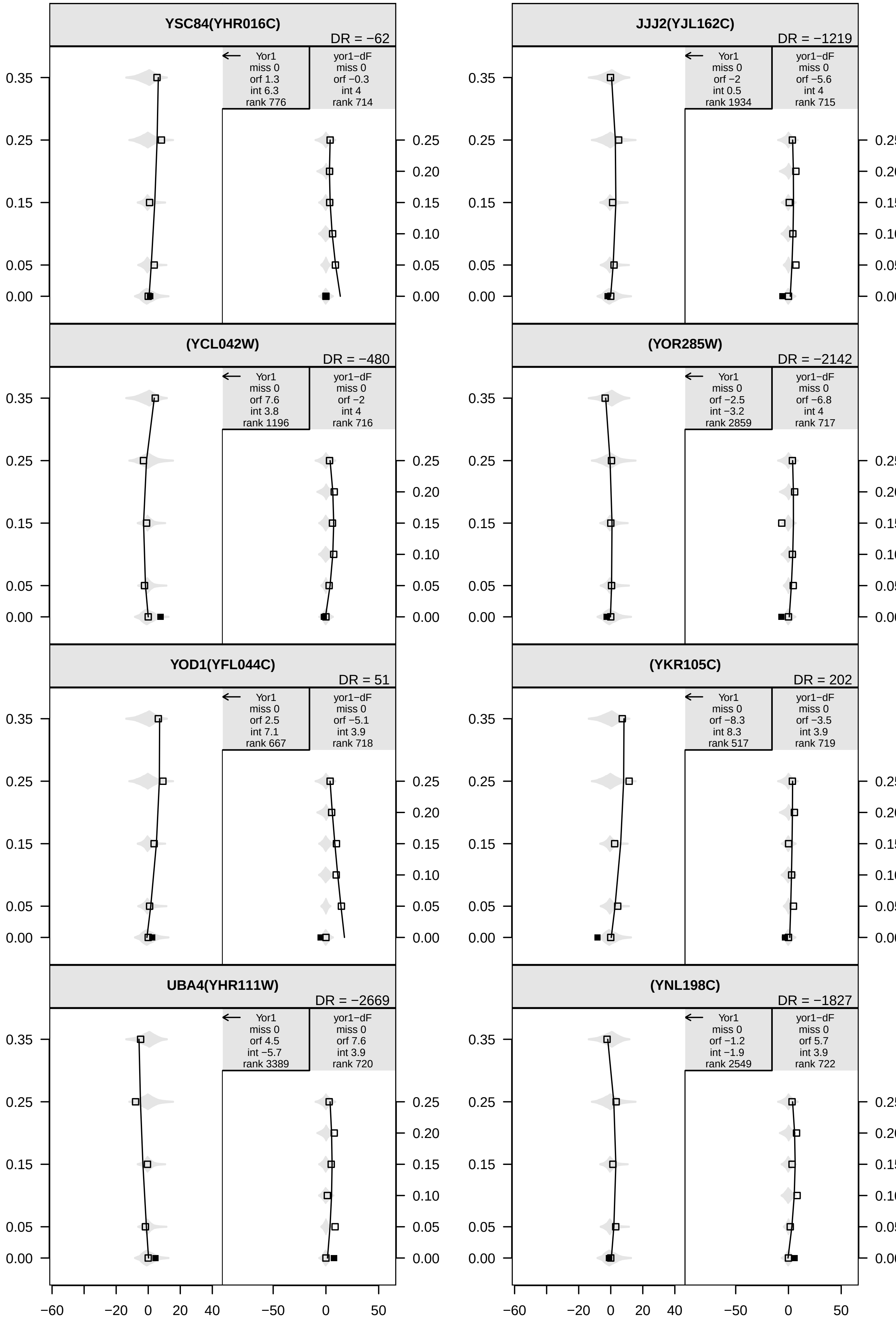

YSC84(YHR016C)
DR = −62
Yor1 miss 0 orf 1.3 int 6.3 rank 776
yor1−dF miss 0 orf −0.3 int 4 rank 714
JJJ2(YJL162C)
DR = −1219
Yor1 miss 0 orf −2 int 0.5 rank 1934
yor1−dF miss 0 orf −5.6 int 4 rank 715
(YCL042W)
DR = −480
Yor1 miss 0 orf 7.6 int 3.8 rank 1196
yor1−dF miss 0 orf −2 int 4 rank 716
(YOR285W)
DR = −2142
Yor1 miss 0 orf −2.5 int −3.2 rank 2859
yor1−dF miss 0 orf −6.8 int 4 rank 717
YOD1(YFL044C)
DR = 51
Yor1 miss 0 orf 2.5 int 7.1 rank 667
yor1−dF miss 0 orf −5.1 int 3.9 rank 718
(YKR105C)
DR = 202
Yor1 miss 0 orf −8.3 int 8.3 rank 517
yor1−dF miss 0 orf −3.5 int 3.9 rank 719
UBA4(YHR111W)
DR = −2669
Yor1 miss 0 orf 4.5 int −5.7 rank 3389
yor1−dF miss 0 orf 7.6 int 3.9 rank 720
(YNL198C)
DR = −1827
Yor1 miss 0 orf −1.2 int −1.9 rank 2549
yor1−dF miss 0 orf 5.7 int 3.9 rank 722

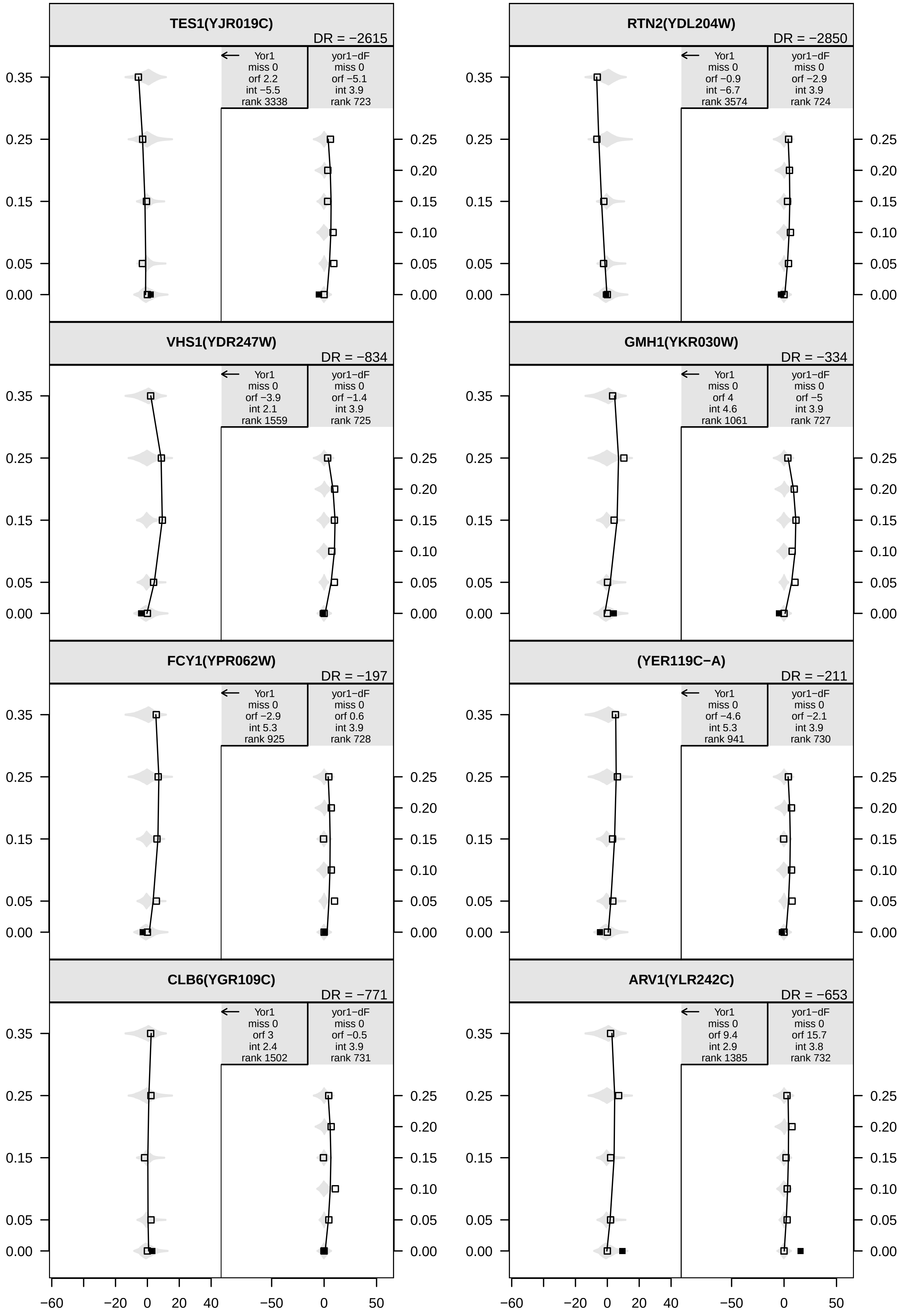
TES1(YJR019C)
DR = −2615
Yor1
miss 0
orf 2.2
int −5.5
rank 3338
yor1−dF
miss 0
orf −5.1
int 3.9
rank 723
RTN2(YDL204W)
DR = −2850
Yor1
miss 0
orf −0.9
int −6.7
rank 3574
yor1−dF
miss 0
orf −2.9
int 3.9
rank 724
VHS1(YDR247W)
DR = −834
Yor1
miss 0
orf −3.9
int 2.1
rank 1559
yor1−dF
miss 0
orf −1.4
int 3.9
rank 725
GMH1(YKR030W)
DR = −334
Yor1
miss 0
orf 4
int 4.6
rank 1061
yor1−dF
miss 0
orf −5
int 3.9
rank 727
FCY1(YPR062W)
DR = −197
Yor1
miss 0
orf −2.9
int 5.3
rank 925
yor1−dF
miss 0
orf 0.6
int 3.9
rank 728
(YER119C−A)
DR = −211
Yor1
miss 0
orf −4.6
int 5.3
rank 941
yor1−dF
miss 0
orf −2.1
int 3.9
rank 730
CLB6(YGR109C)
DR = −771
Yor1
miss 0
orf 3
int 2.4
rank 1502
yor1−dF
miss 0
orf −0.5
int 3.9
rank 731
ARV1(YLR242C)
DR = −653
Yor1
miss 0
orf 9.4
int 2.9
rank 1385
yor1−dF
miss 0
orf 15.7
int 3.8
rank 732
0.35
0.25
0.20
0.15
0.10
0.05
0.00
−60
−20
0
20
40
−50
0
50

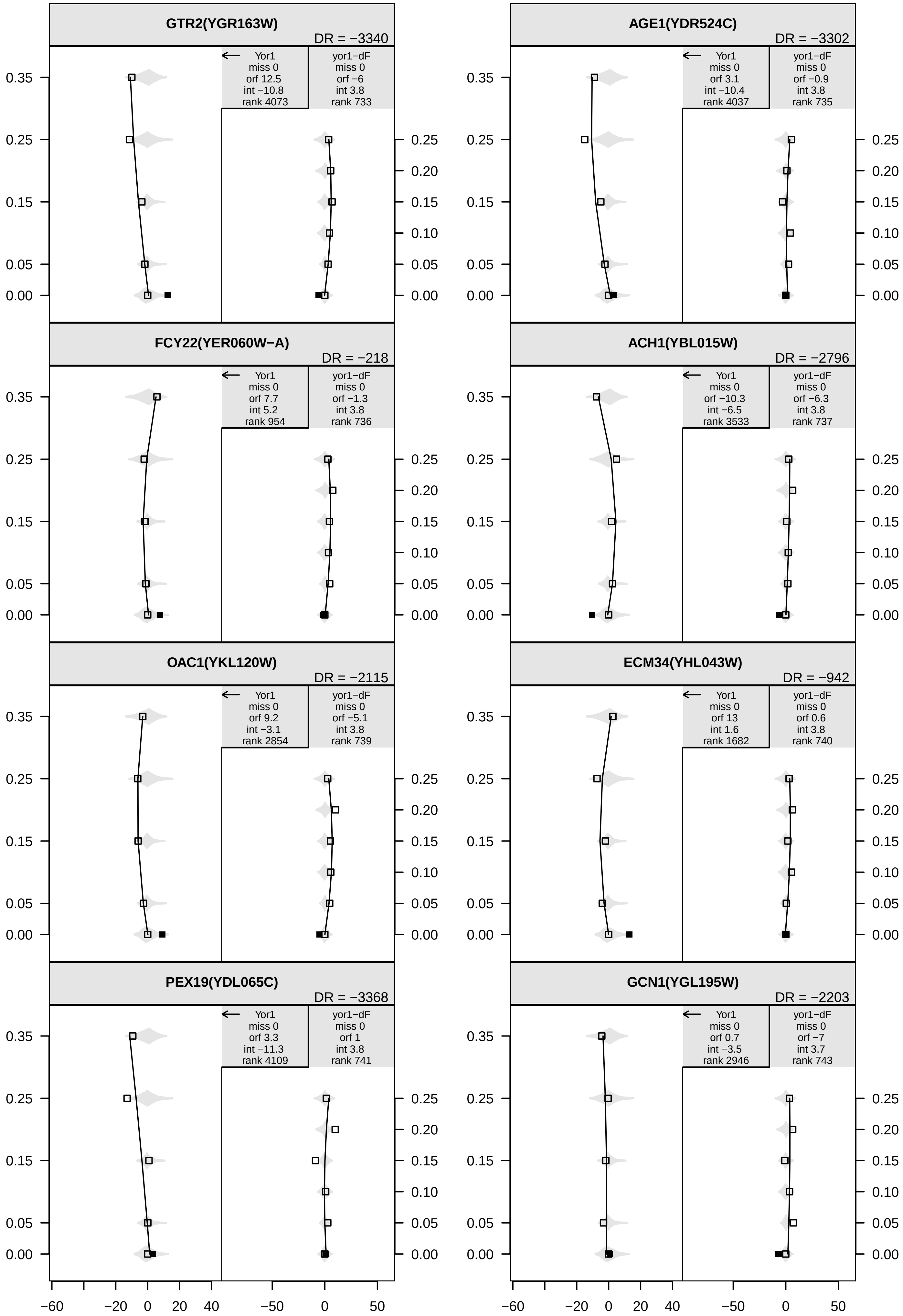

GTR2(YGR163W)
DR = −3340
Yor1 miss 0 orf 12.5 int −10.8 rank 4073
yor1−dF miss 0 orf −6 int 3.8 rank 733
AGE1(YDR524C)
DR = −3302
Yor1 miss 0 orf 3.1 int −10.4 rank 4037
yor1−dF miss 0 orf −0.9 int 3.8 rank 735
FCY22(YER060W−A)
DR = −218
Yor1 miss 0 orf 7.7 int 5.2 rank 954
yor1−dF miss 0 orf −1.3 int 3.8 rank 736
ACH1(YBL015W)
DR = −2796
Yor1 miss 0 orf −10.3 int −6.5 rank 3533
yor1−dF miss 0 orf −6.3 int 3.8 rank 737
OAC1(YKL120W)
DR = −2115
Yor1 miss 0 orf 9.2 int −3.1 rank 2854
yor1−dF miss 0 orf −5.1 int 3.8 rank 739
ECM34(YHL043W)
DR = −942
Yor1 miss 0 orf 13 int 1.6 rank 1682
yor1−dF miss 0 orf 0.6 int 3.8 rank 740
PEX19(YDL065C)
DR = −3368
Yor1 miss 0 orf 3.3 int −11.3 rank 4109
yor1−dF miss 0 orf 1 int 3.8 rank 741
GCN1(YGL195W)
DR = −2203
Yor1 miss 0 orf 0.7 int −3.5 rank 2946
yor1−dF miss 0 orf −7 int 3.7 rank 743
0.35 0.25 0.15 0.05 0.00
0.25 0.20 0.15 0.10 0.05 0.00
−60 −20 0 20 40 −50 0 50

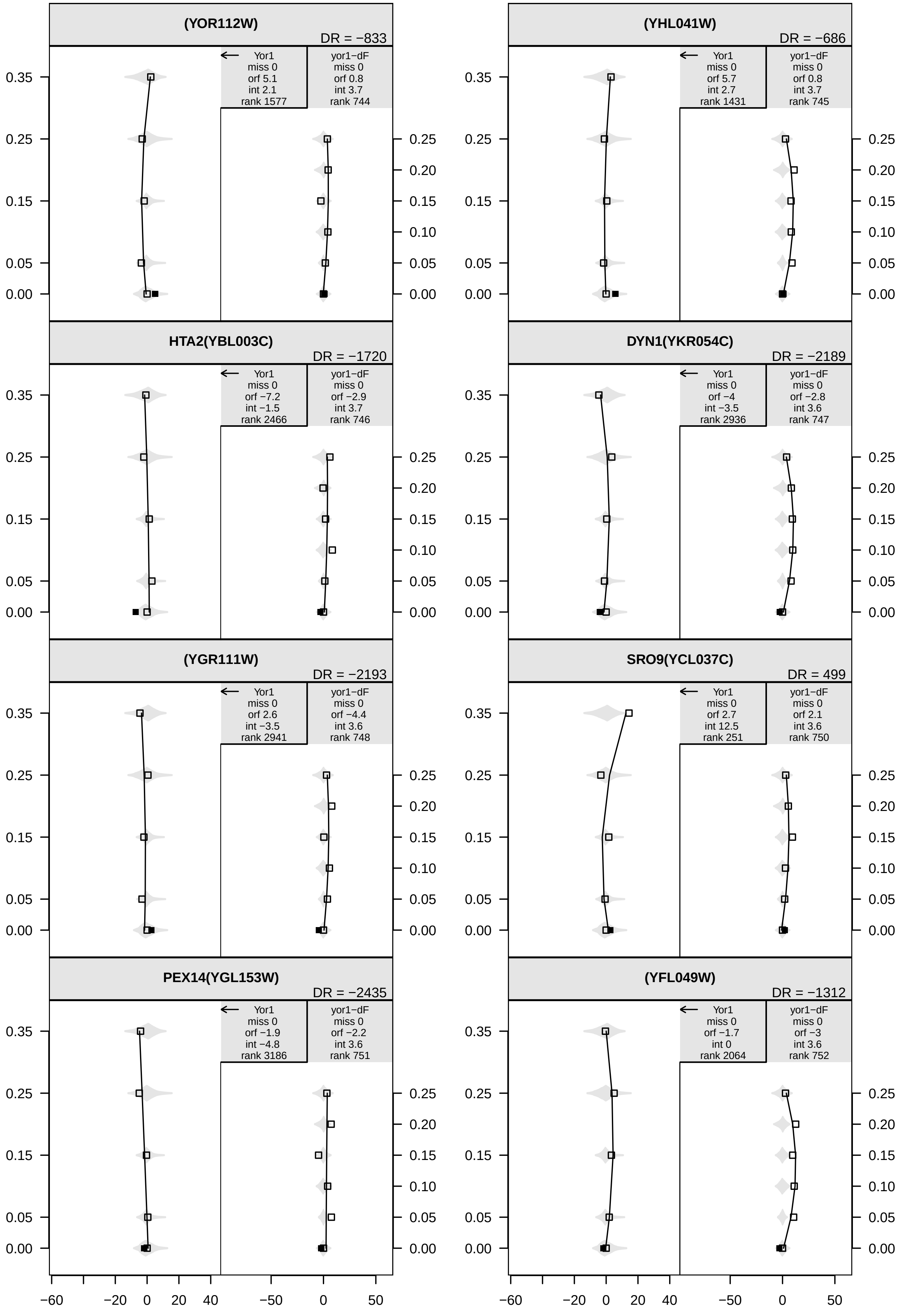
(YOR112W)
DR = −833
Yor1
miss 0
orf 5.1
int 2.1
rank 1577
yor1−dF
miss 0
orf 0.8
int 3.7
rank 744
(YHL041W)
DR = −686
Yor1
miss 0
orf 5.7
int 2.7
rank 1431
yor1−dF
miss 0
orf 0.8
int 3.7
rank 745
HTA2(YBL003C)
DR = −1720
Yor1
miss 0
orf −7.2
int −1.5
rank 2466
yor1−dF
miss 0
orf −2.9
int 3.7
rank 746
DYN1(YKR054C)
DR = −2189
Yor1
miss 0
orf −4
int −3.5
rank 2936
yor1−dF
miss 0
orf −2.8
int 3.6
rank 747
(YGR111W)
DR = −2193
Yor1
miss 0
orf 2.6
int −3.5
rank 2941
yor1−dF
miss 0
orf −4.4
int 3.6
rank 748
SRO9(YCL037C)
DR = 499
Yor1
miss 0
orf 2.7
int 12.5
rank 251
yor1−dF
miss 0
orf 2.1
int 3.6
rank 750
PEX14(YGL153W)
DR = −2435
Yor1
miss 0
orf −1.9
int −4.8
rank 3186
yor1−dF
miss 0
orf −2.2
int 3.6
rank 751
(YFL049W)
DR = −1312
Yor1
miss 0
orf −1.7
int 0
rank 2064
yor1−dF
miss 0
orf −3
int 3.6
rank 752
0.35
0.25
0.20
0.15
0.10
0.05
0.00
−60
−20
0
20
40
−50
0
50

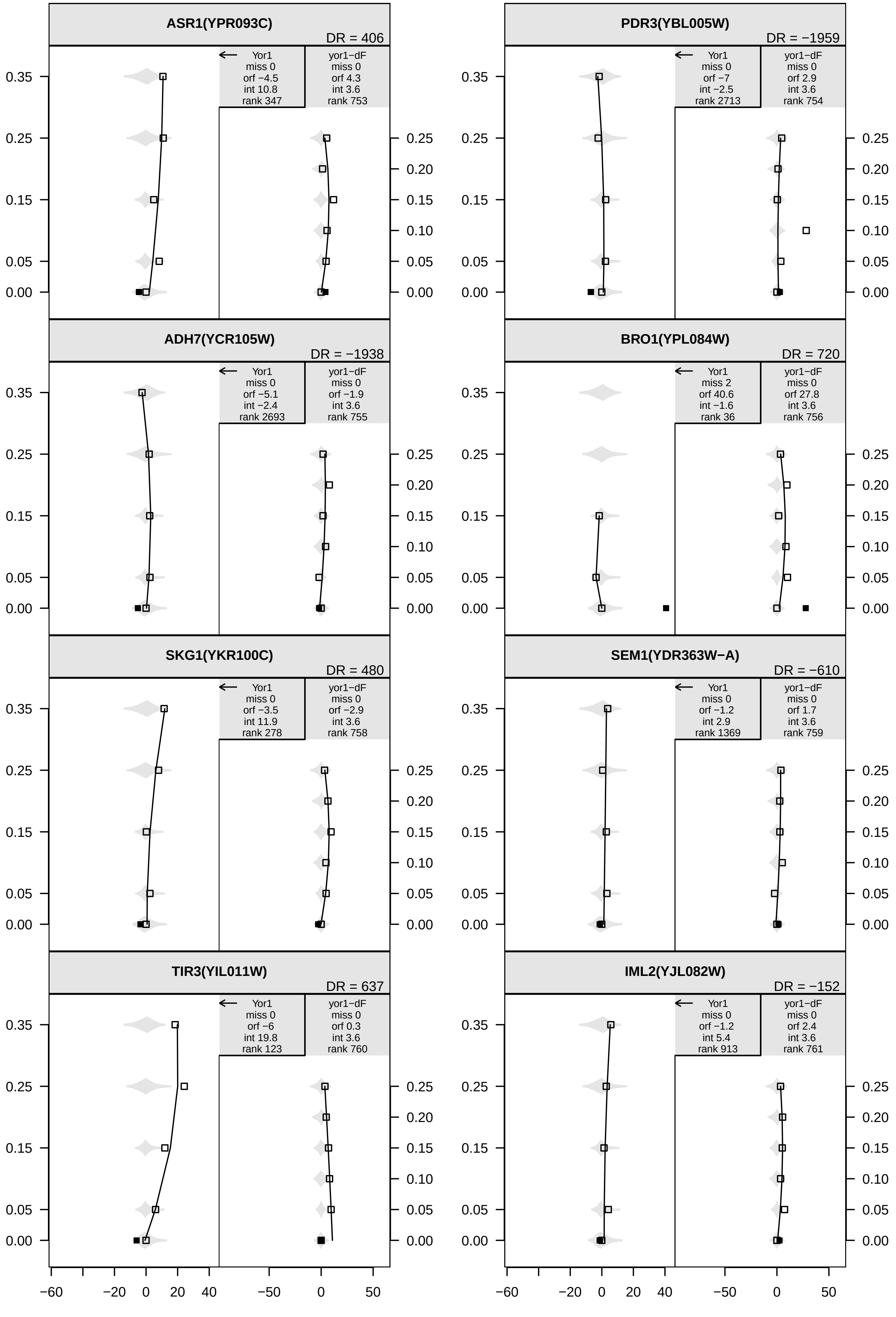
ASR1(YPR093C)
DR = 406
Yor1 miss 0 orf −4.5 int 10.8 rank 347
yor1−dF miss 0 orf 4.3 int 3.6 rank 753
PDR3(YBL005W)
DR = −1959
Yor1 miss 0 orf −7 int −2.5 rank 2713
yor1−dF miss 0 orf 2.9 int 3.6 rank 754
ADH7(YCR105W)
DR = −1938
Yor1 miss 0 orf −5.1 int −2.4 rank 2693
yor1−dF miss 0 orf −1.9 int 3.6 rank 755
BRO1(YPL084W)
DR = 720
Yor1 miss 2 orf 40.6 int −1.6 rank 36
yor1−dF miss 0 orf 27.8 int 3.6 rank 756
SKG1(YKR100C)
DR = 480
Yor1 miss 0 orf −3.5 int 11.9 rank 278
yor1−dF miss 0 orf −2.9 int 3.6 rank 758
SEM1(YDR363W−A)
DR = −610
Yor1 miss 0 orf −1.2 int 2.9 rank 1369
yor1−dF miss 0 orf 1.7 int 3.6 rank 759
TIR3(YIL011W)
DR = 637
Yor1 miss 0 orf −6 int 19.8 rank 123
yor1−dF miss 0 orf 0.3 int 3.6 rank 760
IML2(YJL082W)
DR = −152
Yor1 miss 0 orf −1.2 int 5.4 rank 913
yor1−dF miss 0 orf 2.4 int 3.6 rank 761
0.35 0.25 0.15 0.05 0.00
0.25 0.20 0.15 0.10 0.05 0.00
−60 −20 0 20 40 −50 0 50

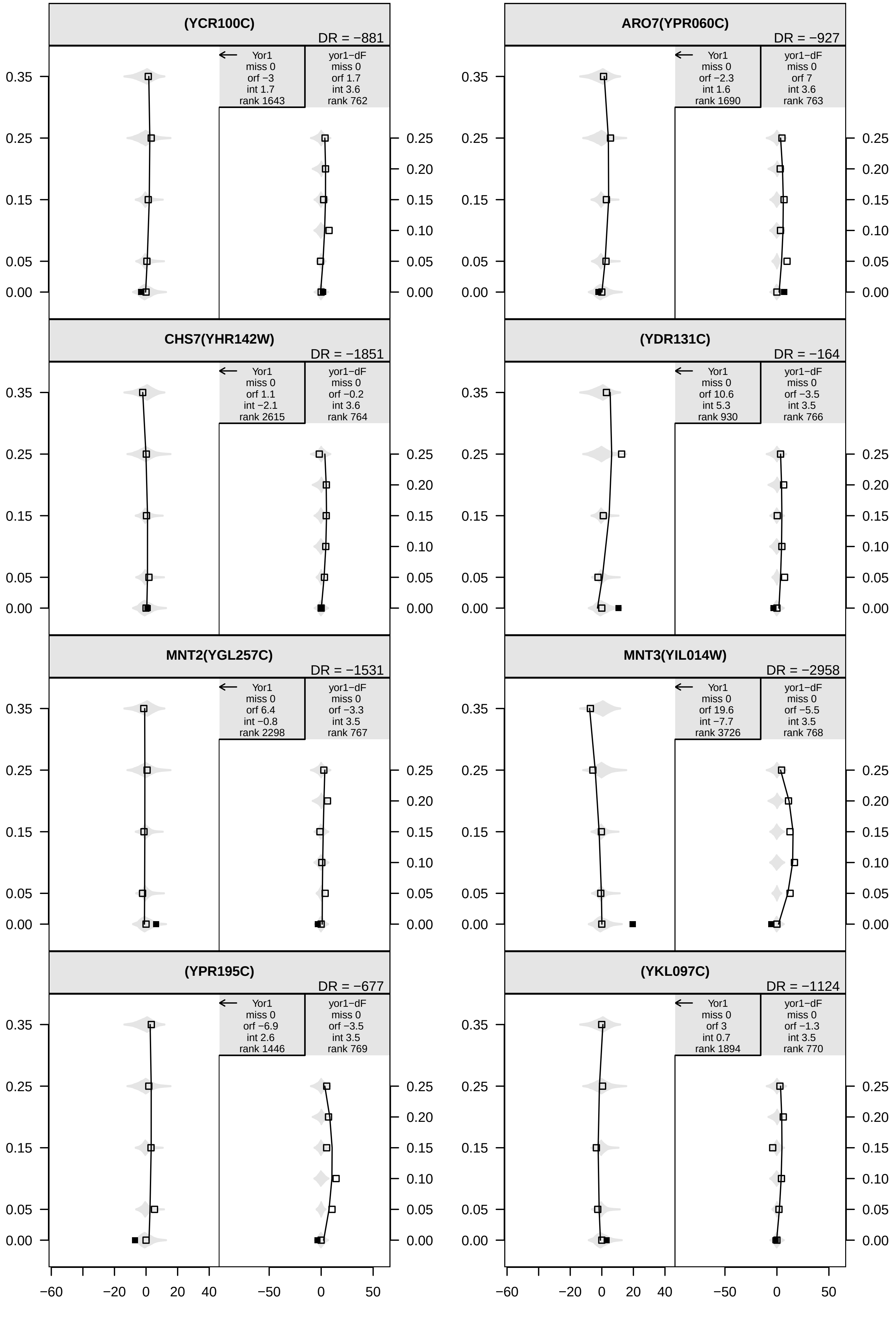

(YCR100C)
DR = −881
Yor1
miss 0
orf −3
int 1.7
rank 1643
yor1−dF
miss 0
orf 1.7
int 3.6
rank 762
ARO7(YPR060C)
DR = −927
Yor1
miss 0
orf −2.3
int 1.6
rank 1690
yor1−dF
miss 0
orf 7
int 3.6
rank 763
CHS7(YHR142W)
DR = −1851
Yor1
miss 0
orf 1.1
int −2.1
rank 2615
yor1−dF
miss 0
orf −0.2
int 3.6
rank 764
(YDR131C)
DR = −164
Yor1
miss 0
orf 10.6
int 5.3
rank 930
yor1−dF
miss 0
orf −3.5
int 3.5
rank 766
MNT2(YGL257C)
DR = −1531
Yor1
miss 0
orf 6.4
int −0.8
rank 2298
yor1−dF
miss 0
orf −3.3
int 3.5
rank 767
MNT3(YIL014W)
DR = −2958
Yor1
miss 0
orf 19.6
int −7.7
rank 3726
yor1−dF
miss 0
orf −5.5
int 3.5
rank 768
(YPR195C)
DR = −677
Yor1
miss 0
orf −6.9
int 2.6
rank 1446
yor1−dF
miss 0
orf −3.5
int 3.5
rank 769
(YKL097C)
DR = −1124
Yor1
miss 0
orf 3
int 0.7
rank 1894
yor1−dF
miss 0
orf −1.3
int 3.5
rank 770
0.35
0.25
0.20
0.15
0.10
0.05
0.00
−60
−20
0
20
40
−50
0
50

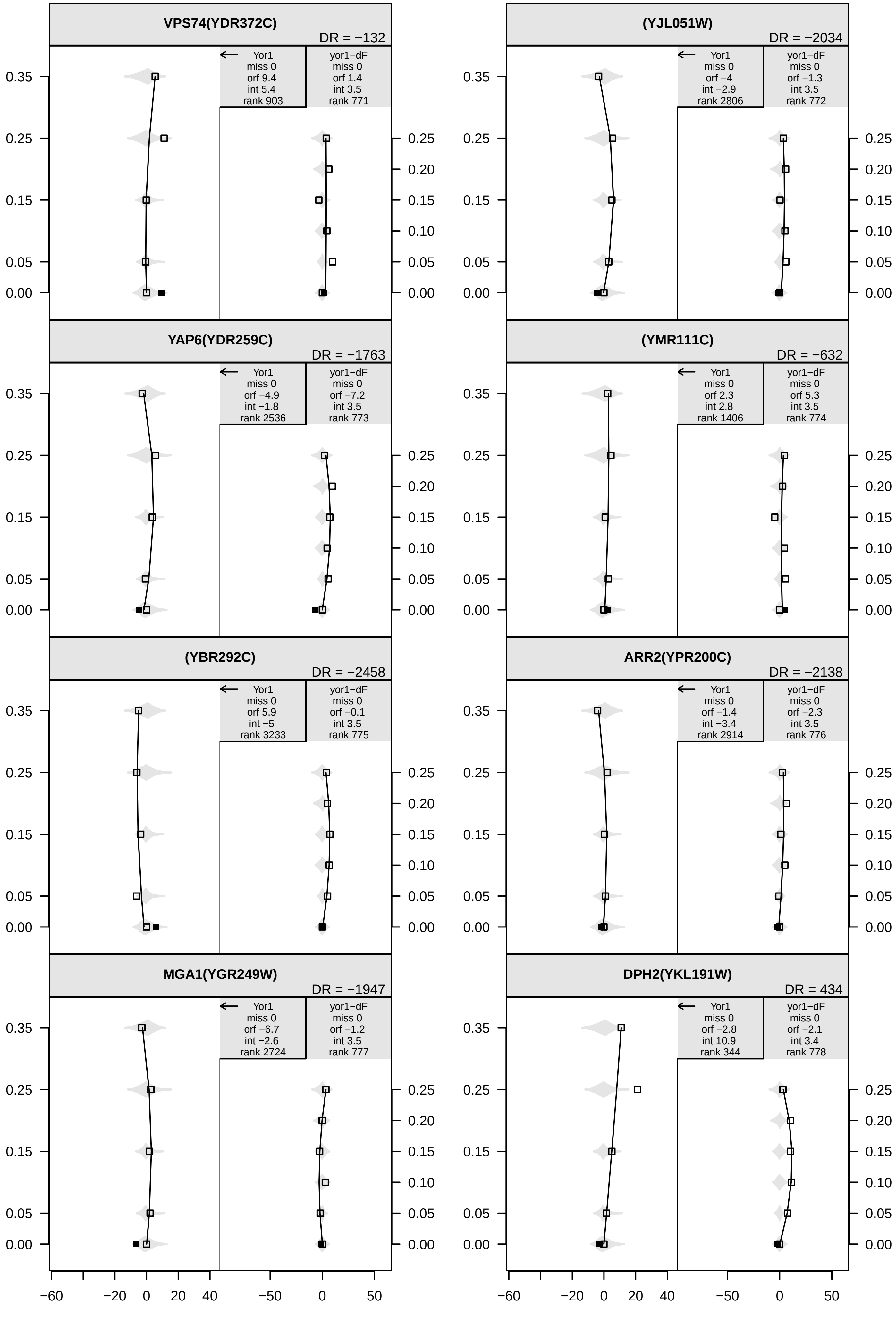
VPS74(YDR372C)
DR = −132
Yor1 miss 0 orf 9.4 int 5.4 rank 903
yor1−dF miss 0 orf 1.4 int 3.5 rank 771
(YJL051W)
DR = −2034
Yor1 miss 0 orf −4 int −2.9 rank 2806
yor1−dF miss 0 orf −1.3 int 3.5 rank 772
YAP6(YDR259C)
DR = −1763
Yor1 miss 0 orf −4.9 int −1.8 rank 2536
yor1−dF miss 0 orf −7.2 int 3.5 rank 773
(YMR111C)
DR = −632
Yor1 miss 0 orf 2.3 int 2.8 rank 1406
yor1−dF miss 0 orf 5.3 int 3.5 rank 774
(YBR292C)
DR = −2458
Yor1 miss 0 orf 5.9 int −5 rank 3233
yor1−dF miss 0 orf −0.1 int 3.5 rank 775
ARR2(YPR200C)
DR = −2138
Yor1 miss 0 orf −1.4 int −3.4 rank 2914
yor1−dF miss 0 orf −2.3 int 3.5 rank 776
MGA1(YGR249W)
DR = −1947
Yor1 miss 0 orf −6.7 int −2.6 rank 2724
yor1−dF miss 0 orf −1.2 int 3.5 rank 777
DPH2(YKL191W)
DR = 434
Yor1 miss 0 orf −2.8 int 10.9 rank 344
yor1−dF miss 0 orf −2.1 int 3.4 rank 778
0.00 0.05 0.10 0.15 0.20 0.25 0.35
−60 −20 0 20 40 −50 0 50

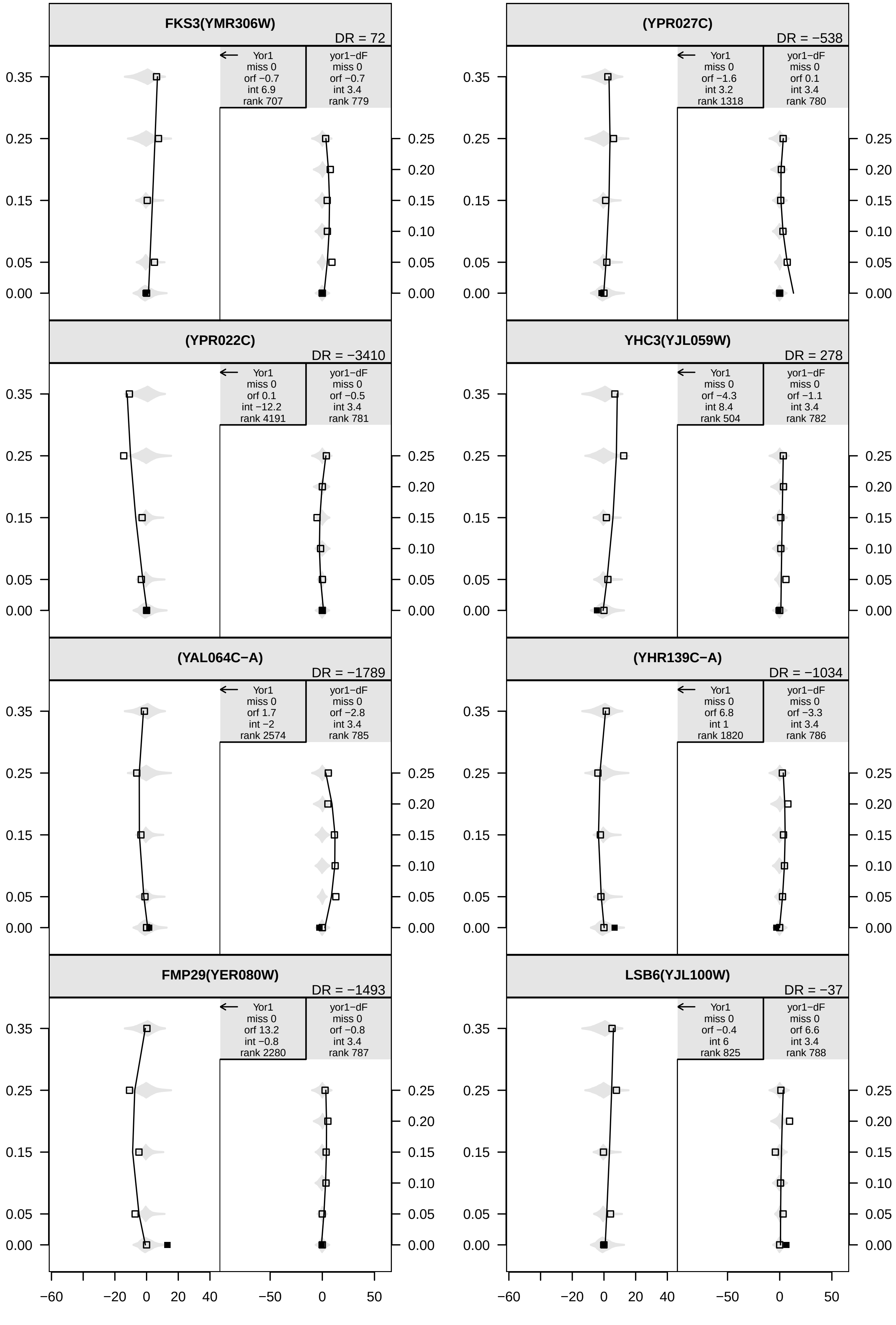

FKS3(YMR306W)
DR = 72
Yor1
miss 0
orf −0.7
int 6.9
rank 707
yor1−dF
miss 0
orf −0.7
int 3.4
rank 779
(YPR027C)
DR = −538
Yor1
miss 0
orf −1.6
int 3.2
rank 1318
yor1−dF
miss 0
orf 0.1
int 3.4
rank 780
(YPR022C)
DR = −3410
Yor1
miss 0
orf 0.1
int −12.2
rank 4191
yor1−dF
miss 0
orf −0.5
int 3.4
rank 781
YHC3(YJL059W)
DR = 278
Yor1
miss 0
orf −4.3
int 8.4
rank 504
yor1−dF
miss 0
orf −1.1
int 3.4
rank 782
(YAL064C−A)
DR = −1789
Yor1
miss 0
orf 1.7
int −2
rank 2574
yor1−dF
miss 0
orf −2.8
int 3.4
rank 785
(YHR139C−A)
DR = −1034
Yor1
miss 0
orf 6.8
int 1
rank 1820
yor1−dF
miss 0
orf −3.3
int 3.4
rank 786
FMP29(YER080W)
DR = −1493
Yor1
miss 0
orf 13.2
int −0.8
rank 2280
yor1−dF
miss 0
orf −0.8
int 3.4
rank 787
LSB6(YJL100W)
DR = −37
Yor1
miss 0
orf −0.4
int 6
rank 825
yor1−dF
miss 0
orf 6.6
int 3.4
rank 788
0.35
0.25
0.20
0.15
0.10
0.05
0.00
−60
−20
0
20
40
−50
0
50

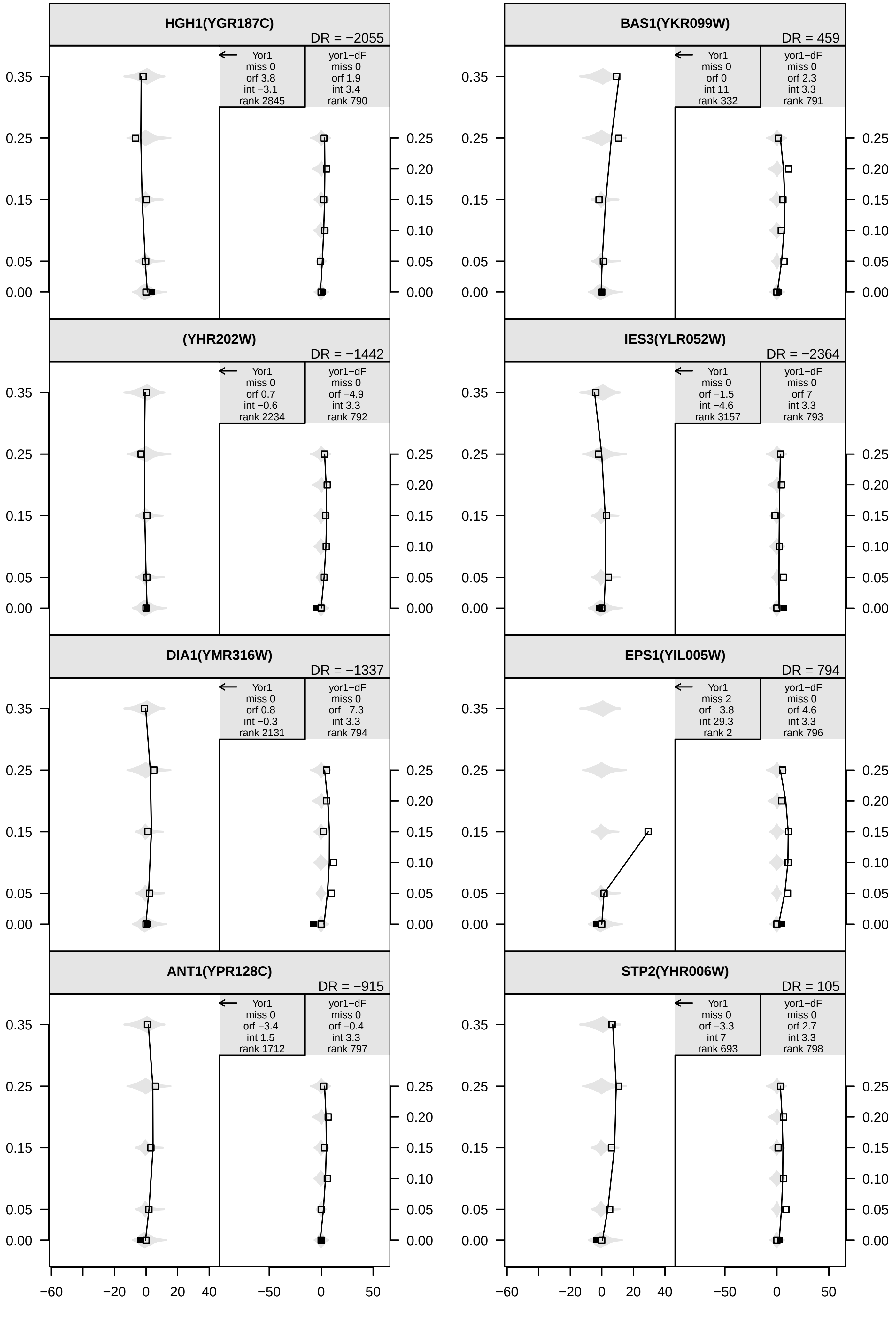
HGH1(YGR187C)
DR = −2055
Yor1 miss 0 orf 3.8 int −3.1 rank 2845
yor1−dF miss 0 orf 1.9 int 3.4 rank 790
BAS1(YKR099W)
DR = 459
Yor1 miss 0 orf 0 int 11 rank 332
yor1−dF miss 0 orf 2.3 int 3.3 rank 791
(YHR202W)
DR = −1442
Yor1 miss 0 orf 0.7 int −0.6 rank 2234
yor1−dF miss 0 orf −4.9 int 3.3 rank 792
IES3(YLR052W)
DR = −2364
Yor1 miss 0 orf −1.5 int −4.6 rank 3157
yor1−dF miss 0 orf 7 int 3.3 rank 793
DIA1(YMR316W)
DR = −1337
Yor1 miss 0 orf 0.8 int −0.3 rank 2131
yor1−dF miss 0 orf −7.3 int 3.3 rank 794
EPS1(YIL005W)
DR = 794
Yor1 miss 2 orf −3.8 int 29.3 rank 2
yor1−dF miss 0 orf 4.6 int 3.3 rank 796
ANT1(YPR128C)
DR = −915
Yor1 miss 0 orf −3.4 int 1.5 rank 1712
yor1−dF miss 0 orf −0.4 int 3.3 rank 797
STP2(YHR006W)
DR = 105
Yor1 miss 0 orf −3.3 int 7 rank 693
yor1−dF miss 0 orf 2.7 int 3.3 rank 798
0.35 0.25 0.15 0.05 0.00
0.25 0.20 0.15 0.10 0.05 0.00
−60 −20 0 20 40 −50 0 50

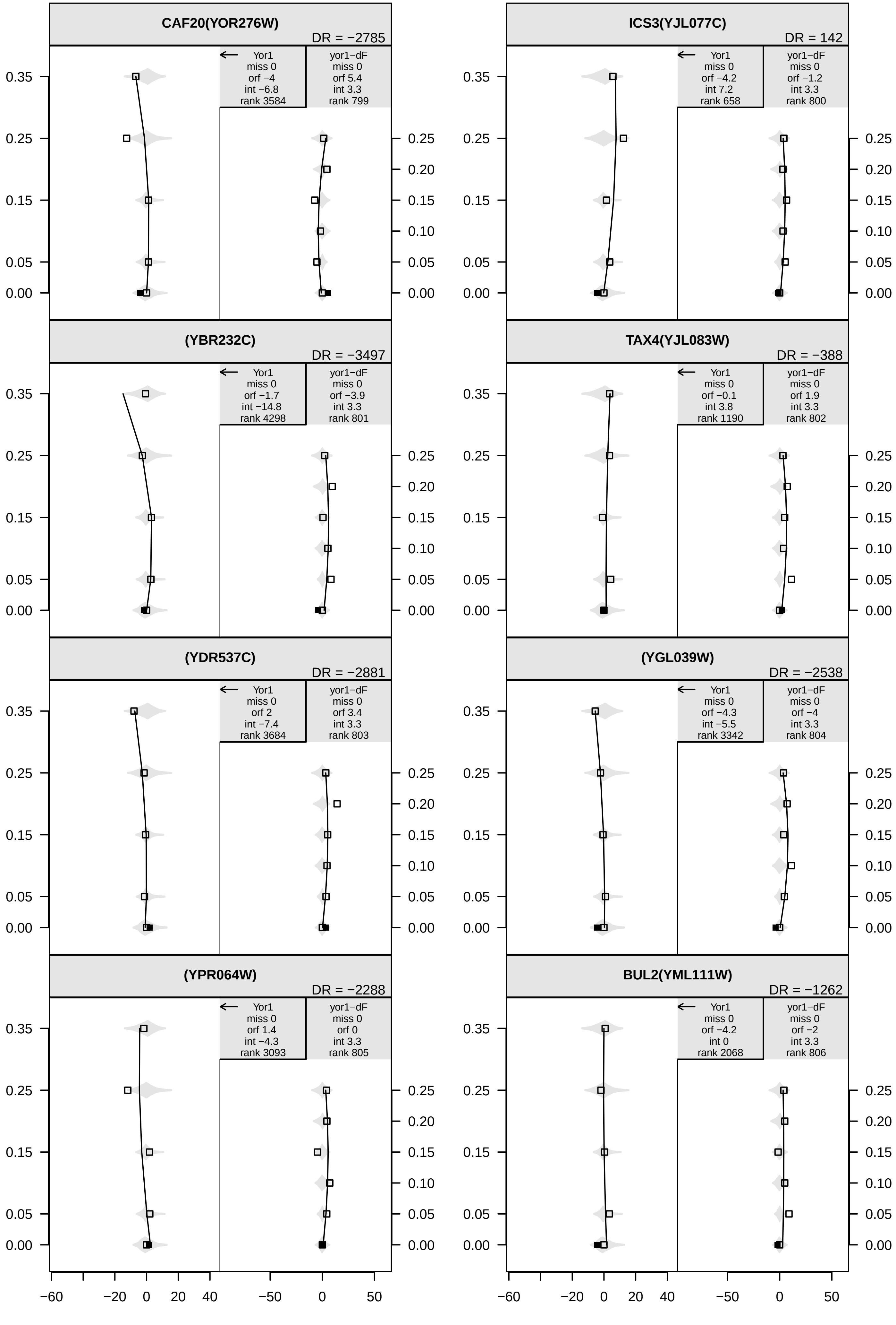

CAF20(YOR276W)
DR = −2785
Yor1
miss 0
orf −4
int −6.8
rank 3584
yor1−dF
miss 0
orf 5.4
int 3.3
rank 799
ICS3(YJL077C)
DR = 142
Yor1
miss 0
orf −4.2
int 7.2
rank 658
yor1−dF
miss 0
orf −1.2
int 3.3
rank 800
(YBR232C)
DR = −3497
Yor1
miss 0
orf −1.7
int −14.8
rank 4298
yor1−dF
miss 0
orf −3.9
int 3.3
rank 801
TAX4(YJL083W)
DR = −388
Yor1
miss 0
orf −0.1
int 3.8
rank 1190
yor1−dF
miss 0
orf 1.9
int 3.3
rank 802
(YDR537C)
DR = −2881
Yor1
miss 0
orf 2
int −7.4
rank 3684
yor1−dF
miss 0
orf 3.4
int 3.3
rank 803
(YGL039W)
DR = −2538
Yor1
miss 0
orf −4.3
int −5.5
rank 3342
yor1−dF
miss 0
orf −4
int 3.3
rank 804
(YPR064W)
DR = −2288
Yor1
miss 0
orf 1.4
int −4.3
rank 3093
yor1−dF
miss 0
orf 0
int 3.3
rank 805
BUL2(YML111W)
DR = −1262
Yor1
miss 0
orf −4.2
int 0
rank 2068
yor1−dF
miss 0
orf −2
int 3.3
rank 806
0.35
0.25
0.20
0.15
0.10
0.05
0.00
−60
−20
0
20
40
−50
0
50

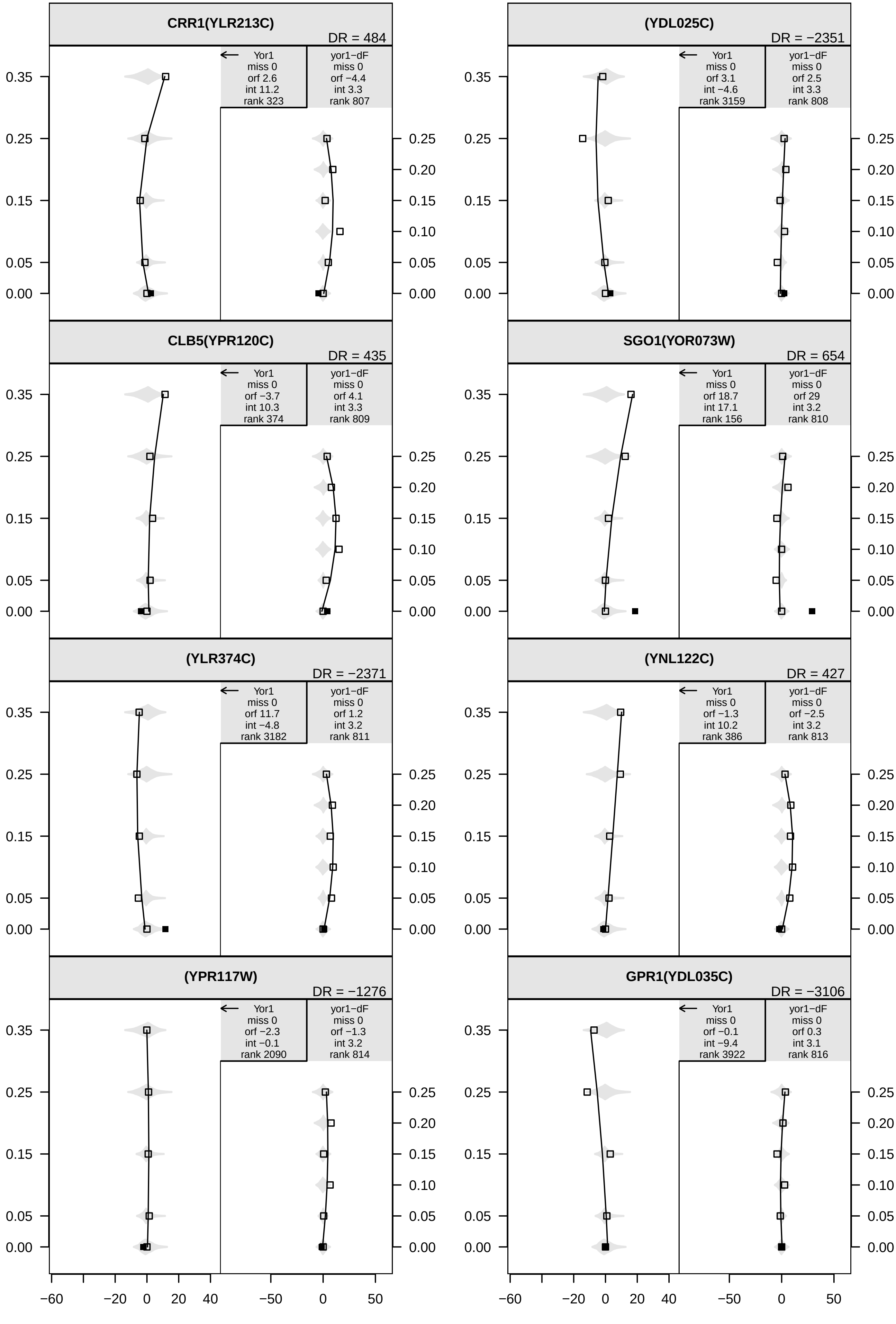

CRR1(YLR213C)
DR = 484
Yor1
miss 0
orf 2.6
int 11.2
rank 323
yor1−dF
miss 0
orf −4.4
int 3.3
rank 807
(YDL025C)
DR = −2351
Yor1
miss 0
orf 3.1
int −4.6
rank 3159
yor1−dF
miss 0
orf 2.5
int 3.3
rank 808
CLB5(YPR120C)
DR = 435
Yor1
miss 0
orf −3.7
int 10.3
rank 374
yor1−dF
miss 0
orf 4.1
int 3.3
rank 809
SGO1(YOR073W)
DR = 654
Yor1
miss 0
orf 18.7
int 17.1
rank 156
yor1−dF
miss 0
orf 29
int 3.2
rank 810
(YLR374C)
DR = −2371
Yor1
miss 0
orf 11.7
int −4.8
rank 3182
yor1−dF
miss 0
orf 1.2
int 3.2
rank 811
(YNL122C)
DR = 427
Yor1
miss 0
orf −1.3
int 10.2
rank 386
yor1−dF
miss 0
orf −2.5
int 3.2
rank 813
(YPR117W)
DR = −1276
Yor1
miss 0
orf −2.3
int −0.1
rank 2090
yor1−dF
miss 0
orf −1.3
int 3.2
rank 814
GPR1(YDL035C)
DR = −3106
Yor1
miss 0
orf −0.1
int −9.4
rank 3922
yor1−dF
miss 0
orf 0.3
int 3.1
rank 816
0.35
0.25
0.20
0.15
0.10
0.05
0.00
−60
−20
0
20
40
−50
0
50

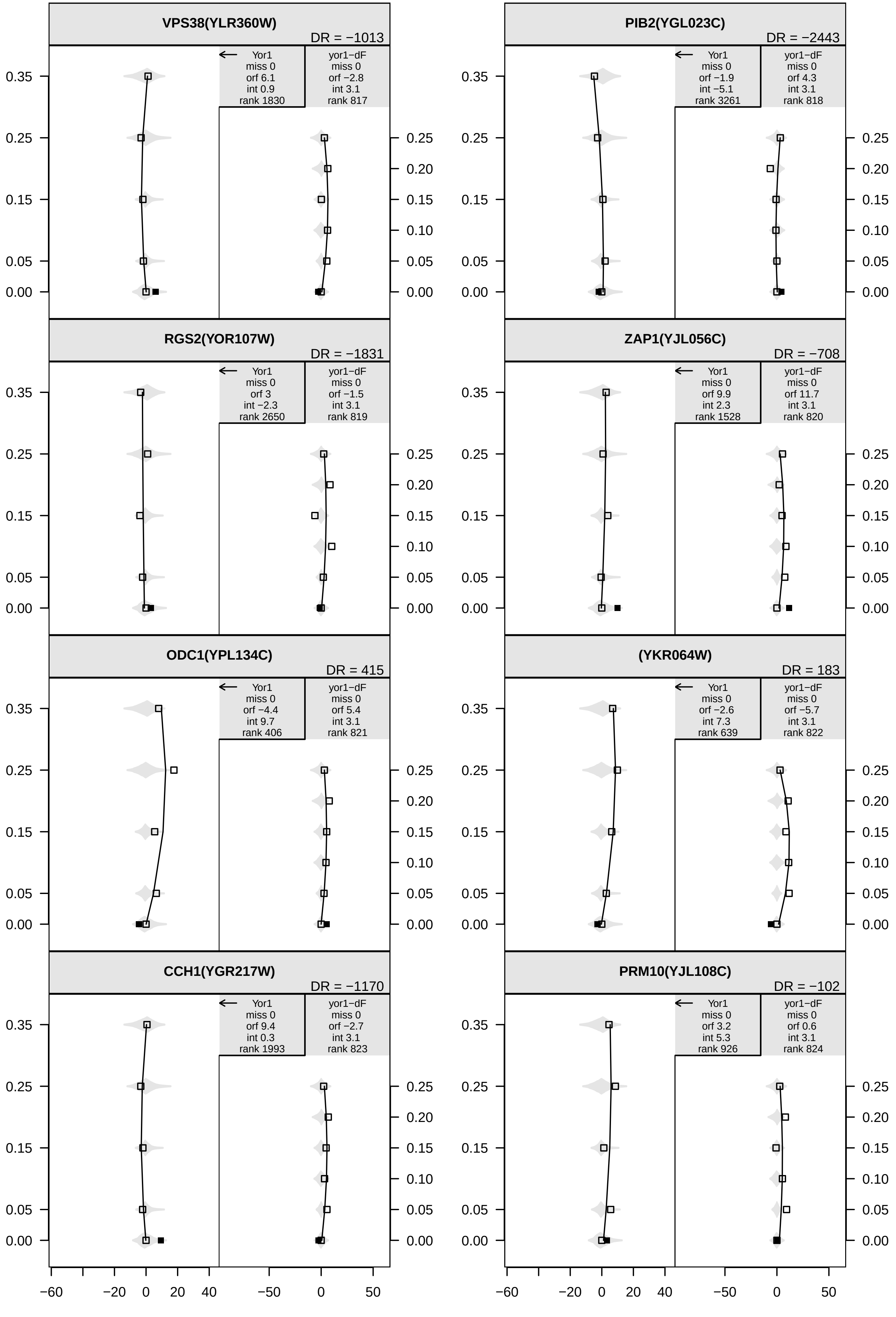

VPS38(YLR360W)
DR = −1013
Yor1 miss 0 orf 6.1 int 0.9 rank 1830
yor1−dF miss 0 orf −2.8 int 3.1 rank 817
PIB2(YGL023C)
DR = −2443
Yor1 miss 0 orf −1.9 int −5.1 rank 3261
yor1−dF miss 0 orf 4.3 int 3.1 rank 818
RGS2(YOR107W)
DR = −1831
Yor1 miss 0 orf 3 int −2.3 rank 2650
yor1−dF miss 0 orf −1.5 int 3.1 rank 819
ZAP1(YJL056C)
DR = −708
Yor1 miss 0 orf 9.9 int 2.3 rank 1528
yor1−dF miss 0 orf 11.7 int 3.1 rank 820
ODC1(YPL134C)
DR = 415
Yor1 miss 0 orf −4.4 int 9.7 rank 406
yor1−dF miss 0 orf 5.4 int 3.1 rank 821
(YKR064W)
DR = 183
Yor1 miss 0 orf −2.6 int 7.3 rank 639
yor1−dF miss 0 orf −5.7 int 3.1 rank 822
CCH1(YGR217W)
DR = −1170
Yor1 miss 0 orf 9.4 int 0.3 rank 1993
yor1−dF miss 0 orf −2.7 int 3.1 rank 823
PRM10(YJL108C)
DR = −102
Yor1 miss 0 orf 3.2 int 5.3 rank 926
yor1−dF miss 0 orf 0.6 int 3.1 rank 824
0.35
0.25
0.20
0.15
0.10
0.05
0.00
−60
−20
0
20
40
−50
0
50

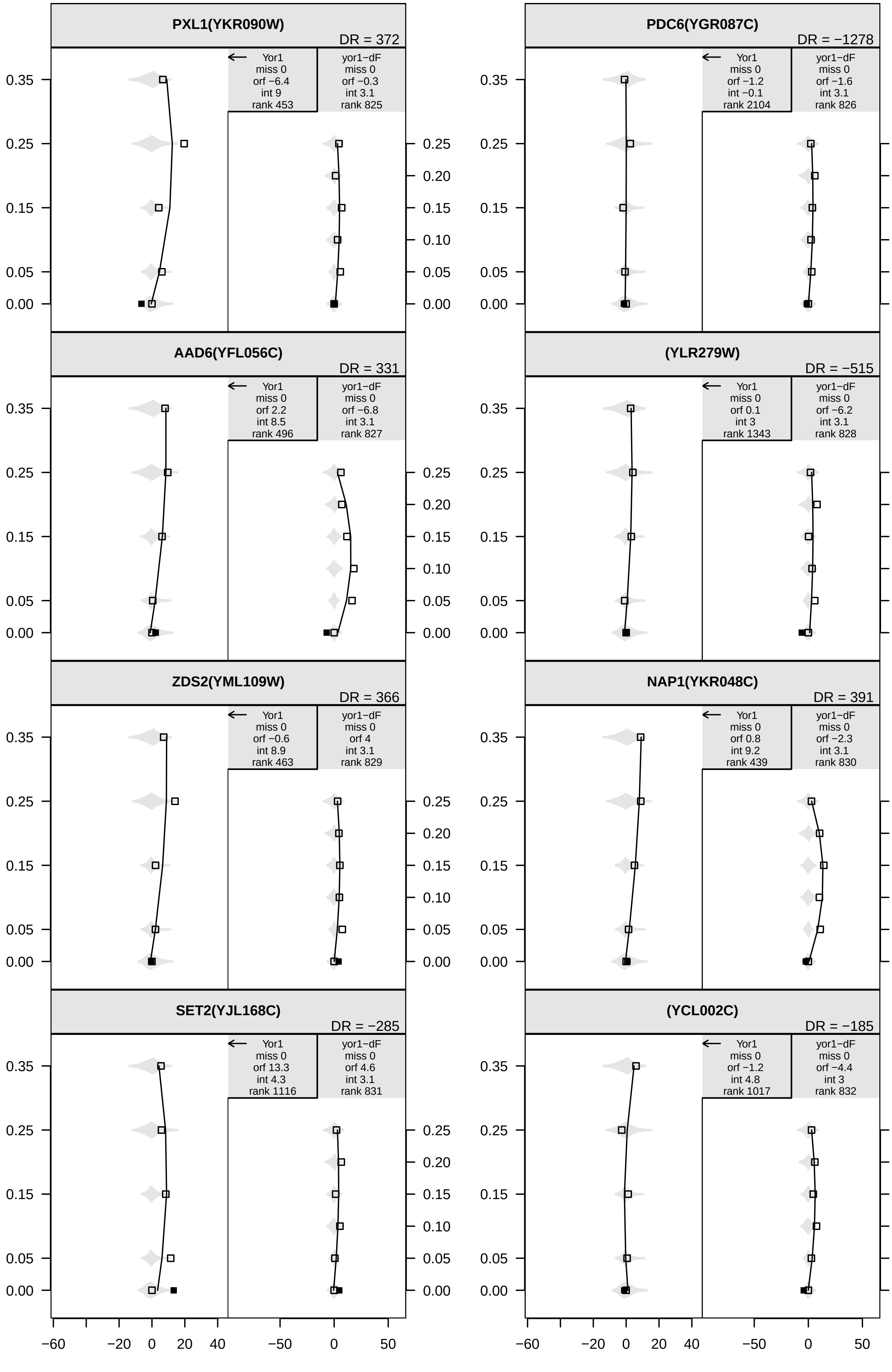
PXL1(YKR090W)
DR = 372
Yor1 miss 0 orf −6.4 int 9 rank 453
yor1−dF miss 0 orf −0.3 int 3.1 rank 825
PDC6(YGR087C)
DR = −1278
Yor1 miss 0 orf −1.2 int −0.1 rank 2104
yor1−dF miss 0 orf −1.6 int 3.1 rank 826
AAD6(YFL056C)
DR = 331
Yor1 miss 0 orf 2.2 int 8.5 rank 496
yor1−dF miss 0 orf −6.8 int 3.1 rank 827
(YLR279W)
DR = −515
Yor1 miss 0 orf 0.1 int 3 rank 1343
yor1−dF miss 0 orf −6.2 int 3.1 rank 828
ZDS2(YML109W)
DR = 366
Yor1 miss 0 orf −0.6 int 8.9 rank 463
yor1−dF miss 0 orf 4 int 3.1 rank 829
NAP1(YKR048C)
DR = 391
Yor1 miss 0 orf 0.8 int 9.2 rank 439
yor1−dF miss 0 orf −2.3 int 3.1 rank 830
SET2(YJL168C)
DR = −285
Yor1 miss 0 orf 13.3 int 4.3 rank 1116
yor1−dF miss 0 orf 4.6 int 3.1 rank 831
(YCL002C)
DR = −185
Yor1 miss 0 orf −1.2 int 4.8 rank 1017
yor1−dF miss 0 orf −4.4 int 3 rank 832
0.35 0.25 0.20 0.15 0.10 0.05 0.00
−60 −20 0 20 40 −50 0 50

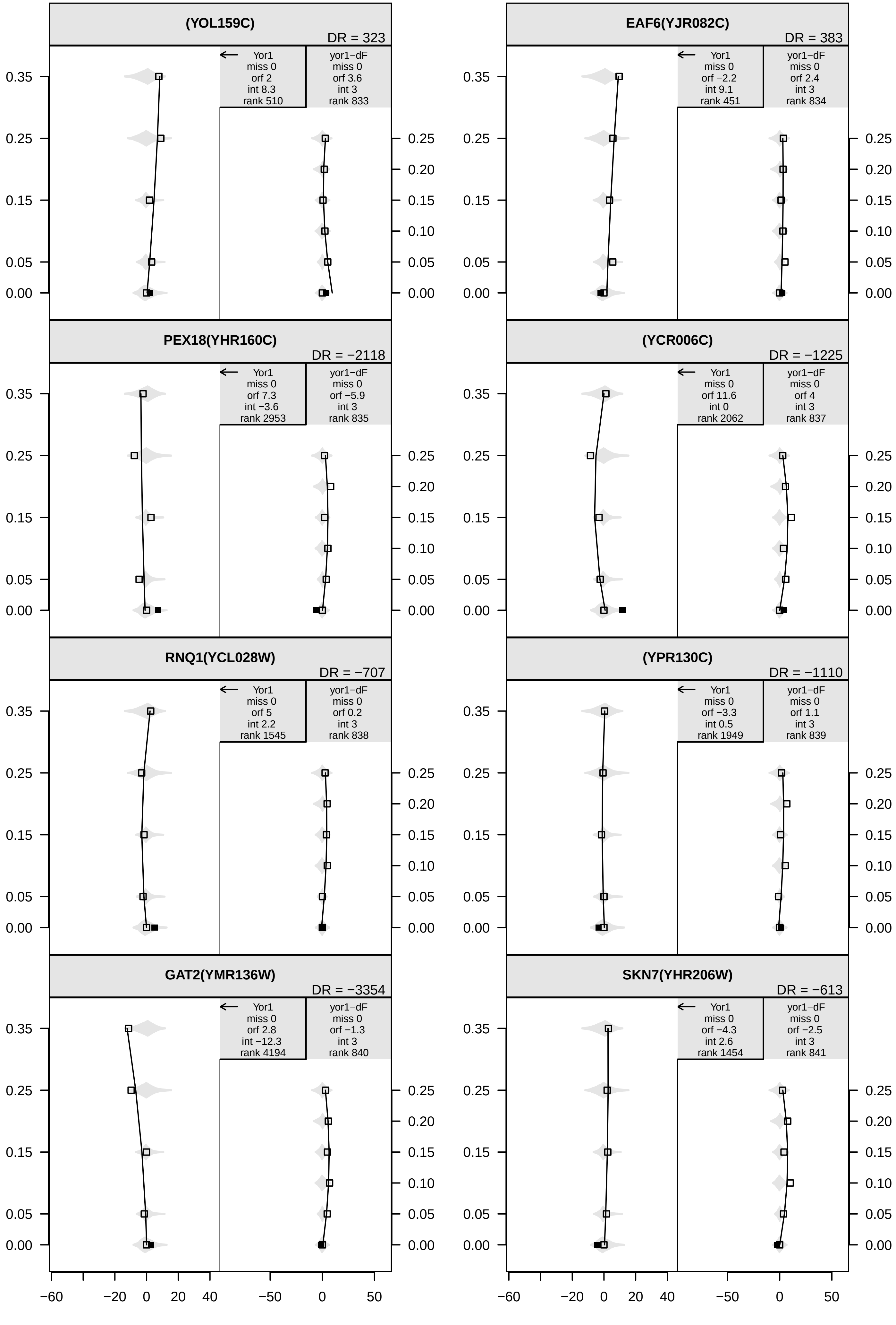

(YOL159C)
DR = 323
Yor1
miss 0
orf 2
int 8.3
rank 510
yor1−dF
miss 0
orf 3.6
int 3
rank 833
EAF6(YJR082C)
DR = 383
Yor1
miss 0
orf −2.2
int 9.1
rank 451
yor1−dF
miss 0
orf 2.4
int 3
rank 834
PEX18(YHR160C)
DR = −2118
Yor1
miss 0
orf 7.3
int −3.6
rank 2953
yor1−dF
miss 0
orf −5.9
int 3
rank 835
(YCR006C)
DR = −1225
Yor1
miss 0
orf 11.6
int 0
rank 2062
yor1−dF
miss 0
orf 4
int 3
rank 837
RNQ1(YCL028W)
DR = −707
Yor1
miss 0
orf 5
int 2.2
rank 1545
yor1−dF
miss 0
orf 0.2
int 3
rank 838
(YPR130C)
DR = −1110
Yor1
miss 0
orf −3.3
int 0.5
rank 1949
yor1−dF
miss 0
orf 1.1
int 3
rank 839
GAT2(YMR136W)
DR = −3354
Yor1
miss 0
orf 2.8
int −12.3
rank 4194
yor1−dF
miss 0
orf −1.3
int 3
rank 840
SKN7(YHR206W)
DR = −613
Yor1
miss 0
orf −4.3
int 2.6
rank 1454
yor1−dF
miss 0
orf −2.5
int 3
rank 841
0.35
0.25
0.20
0.15
0.10
0.05
0.00
−60
−20
0
20
40
−50
0
50

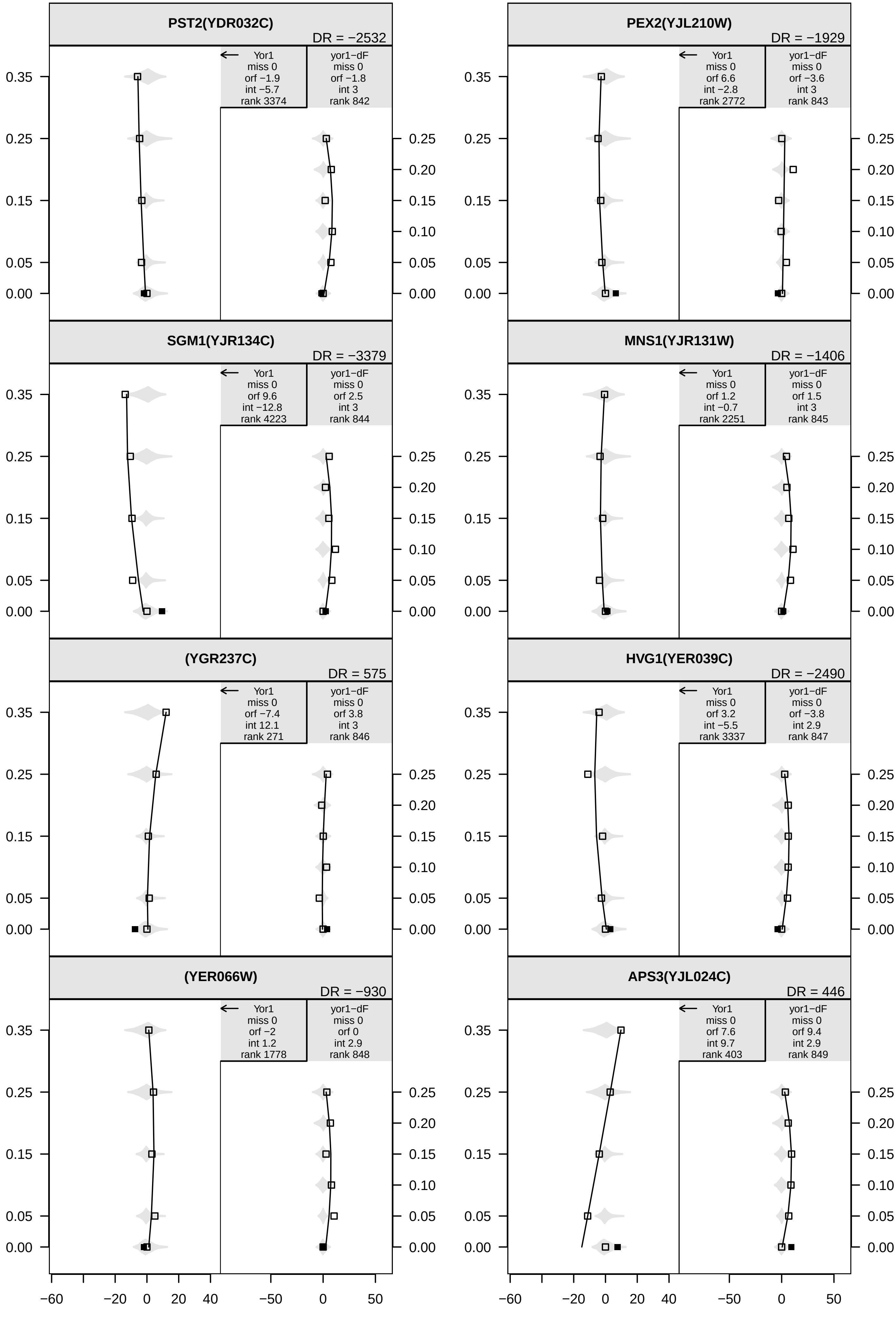
PST2(YDR032C)
DR = −2532
Yor1
miss 0
orf −1.9
int −5.7
rank 3374
yor1−dF
miss 0
orf −1.8
int 3
rank 842
PEX2(YJL210W)
DR = −1929
Yor1
miss 0
orf 6.6
int −2.8
rank 2772
yor1−dF
miss 0
orf −3.6
int 3
rank 843
SGM1(YJR134C)
DR = −3379
Yor1
miss 0
orf 9.6
int −12.8
rank 4223
yor1−dF
miss 0
orf 2.5
int 3
rank 844
MNS1(YJR131W)
DR = −1406
Yor1
miss 0
orf 1.2
int −0.7
rank 2251
yor1−dF
miss 0
orf 1.5
int 3
rank 845
(YGR237C)
DR = 575
Yor1
miss 0
orf −7.4
int 12.1
rank 271
yor1−dF
miss 0
orf 3.8
int 3
rank 846
HVG1(YER039C)
DR = −2490
Yor1
miss 0
orf 3.2
int −5.5
rank 3337
yor1−dF
miss 0
orf −3.8
int 2.9
rank 847
(YER066W)
DR = −930
Yor1
miss 0
orf −2
int 1.2
rank 1778
yor1−dF
miss 0
orf 0
int 2.9
rank 848
APS3(YJL024C)
DR = 446
Yor1
miss 0
orf 7.6
int 9.7
rank 403
yor1−dF
miss 0
orf 9.4
int 2.9
rank 849
0.35
0.25
0.20
0.15
0.10
0.05
0.00
−60
−20
0
20
40
−50
0
50

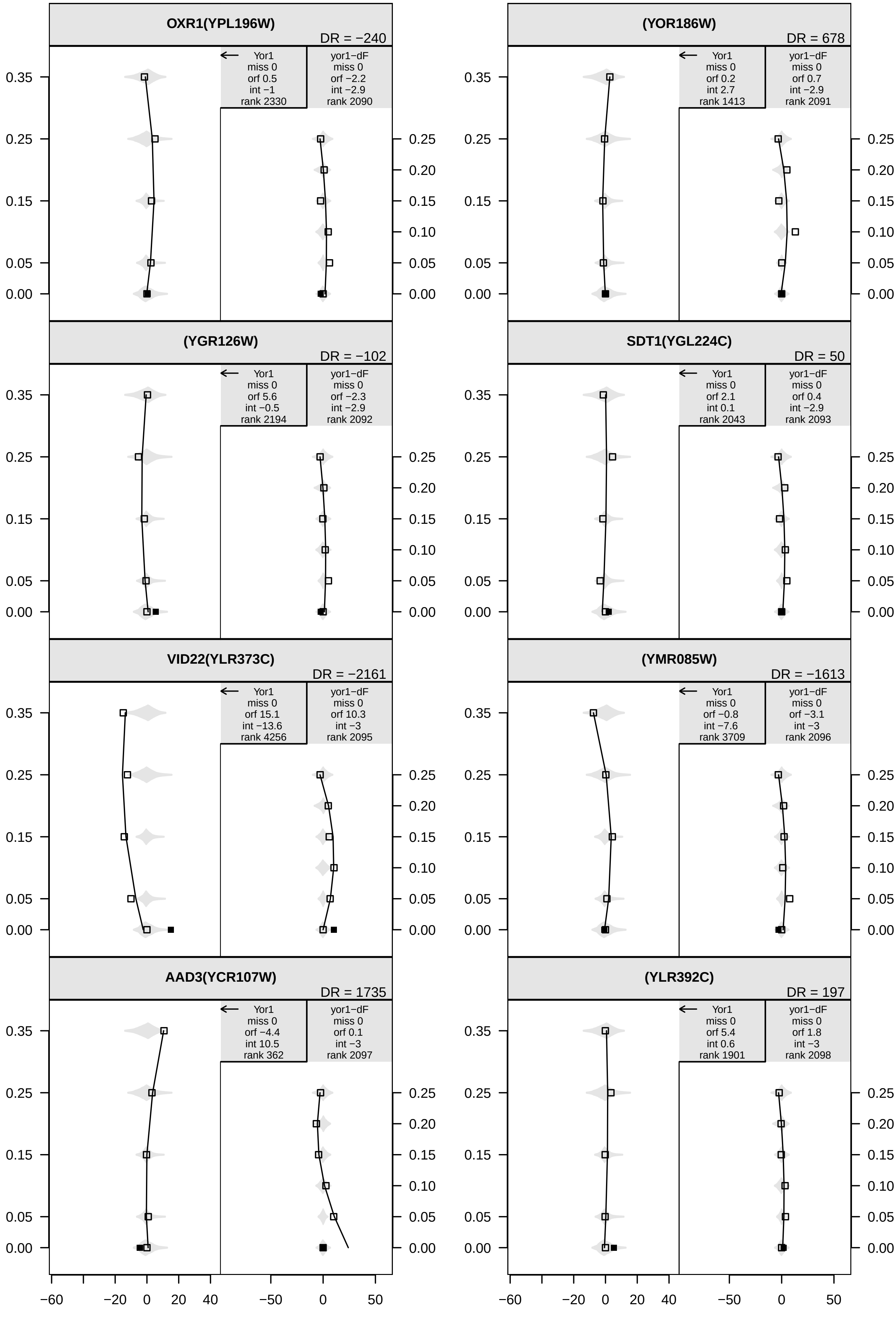
OXR1(YPL196W)
DR = −240
Yor1
miss 0
orf 0.5
int −1
rank 2330
yor1−dF
miss 0
orf −2.2
int −2.9
rank 2090
(YOR186W)
DR = 678
Yor1
miss 0
orf 0.2
int 2.7
rank 1413
yor1−dF
miss 0
orf 0.7
int −2.9
rank 2091
(YGR126W)
DR = −102
Yor1
miss 0
orf 5.6
int −0.5
rank 2194
yor1−dF
miss 0
orf −2.3
int −2.9
rank 2092
SDT1(YGL224C)
DR = 50
Yor1
miss 0
orf 2.1
int 0.1
rank 2043
yor1−dF
miss 0
orf 0.4
int −2.9
rank 2093
VID22(YLR373C)
DR = −2161
Yor1
miss 0
orf 15.1
int −13.6
rank 4256
yor1−dF
miss 0
orf 10.3
int −3
rank 2095
(YMR085W)
DR = −1613
Yor1
miss 0
orf −0.8
int −7.6
rank 3709
yor1−dF
miss 0
orf −3.1
int −3
rank 2096
AAD3(YCR107W)
DR = 1735
Yor1
miss 0
orf −4.4
int 10.5
rank 362
yor1−dF
miss 0
orf 0.1
int −3
rank 2097
(YLR392C)
DR = 197
Yor1
miss 0
orf 5.4
int 0.6
rank 1901
yor1−dF
miss 0
orf 1.8
int −3
rank 2098
0.35
0.25
0.20
0.15
0.10
0.05
0.00
−60
−20
0
20
40
−50
0
50

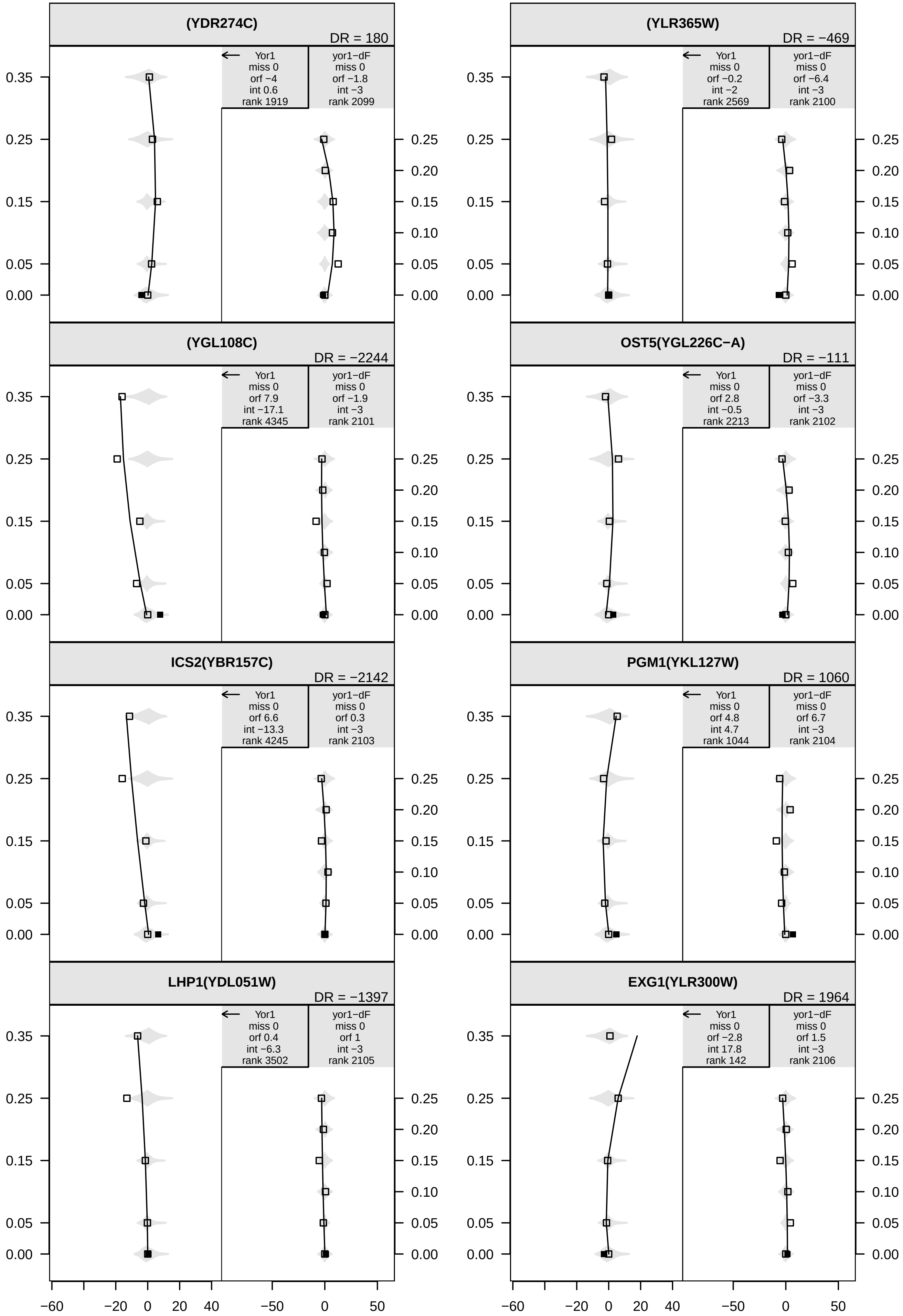

(YDR274C)
DR = 180
Yor1 miss 0 orf −4 int 0.6 rank 1919
yor1−dF miss 0 orf −1.8 int −3 rank 2099
(YLR365W)
DR = −469
Yor1 miss 0 orf −0.2 int −2 rank 2569
yor1−dF miss 0 orf −6.4 int −3 rank 2100
(YGL108C)
DR = −2244
Yor1 miss 0 orf 7.9 int −17.1 rank 4345
yor1−dF miss 0 orf −1.9 int −3 rank 2101
OST5(YGL226C−A)
DR = −111
Yor1 miss 0 orf 2.8 int −0.5 rank 2213
yor1−dF miss 0 orf −3.3 int −3 rank 2102
ICS2(YBR157C)
DR = −2142
Yor1 miss 0 orf 6.6 int −13.3 rank 4245
yor1−dF miss 0 orf 0.3 int −3 rank 2103
PGM1(YKL127W)
DR = 1060
Yor1 miss 0 orf 4.8 int 4.7 rank 1044
yor1−dF miss 0 orf 6.7 int −3 rank 2104
LHP1(YDL051W)
DR = −1397
Yor1 miss 0 orf 0.4 int −6.3 rank 3502
yor1−dF miss 0 orf 1 int −3 rank 2105
EXG1(YLR300W)
DR = 1964
Yor1 miss 0 orf −2.8 int 17.8 rank 142
yor1−dF miss 0 orf 1.5 int −3 rank 2106

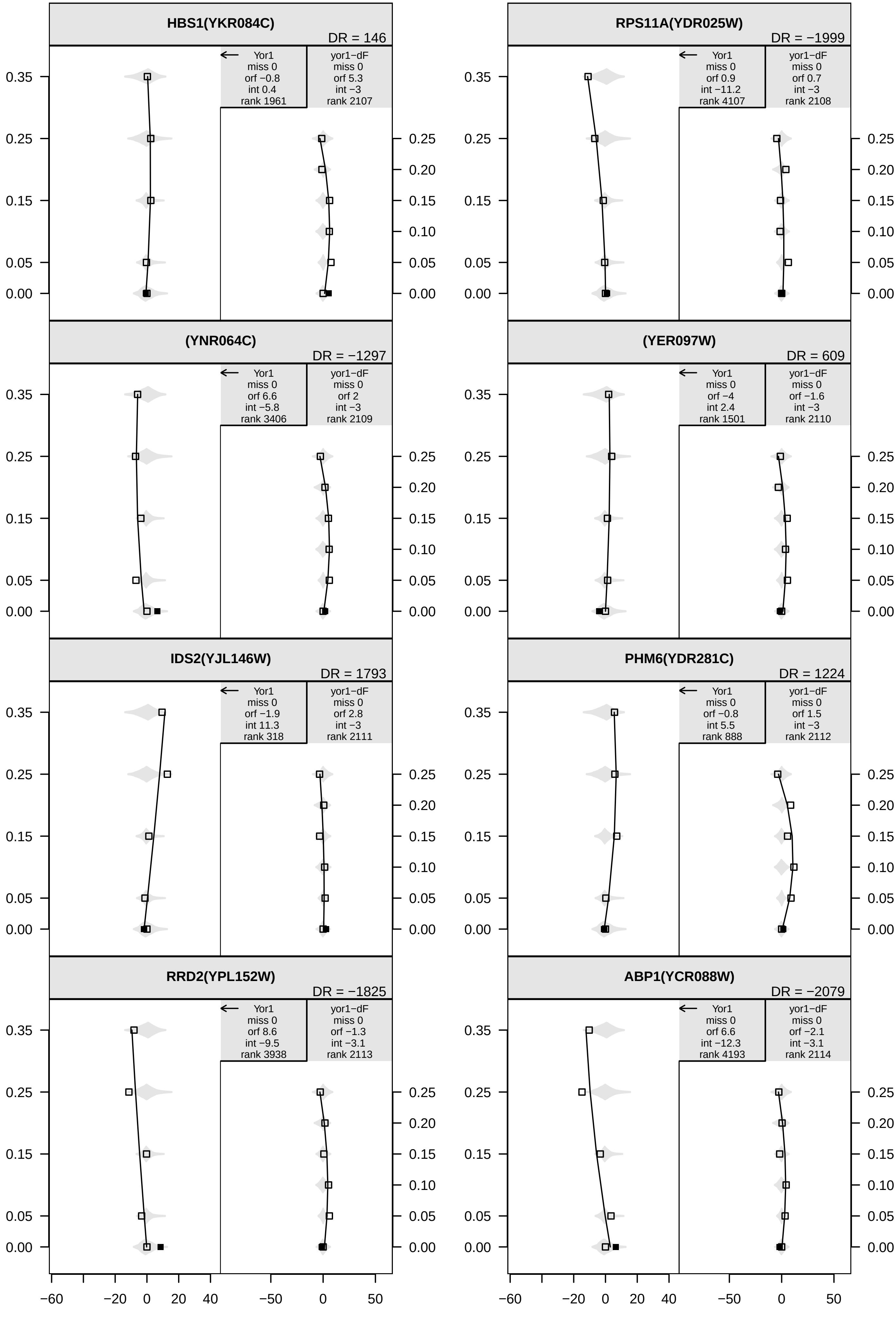

HBS1(YKR084C)
DR = 146
Yor1
miss 0
orf −0.8
int 0.4
rank 1961
yor1−dF
miss 0
orf 5.3
int −3
rank 2107
RPS11A(YDR025W)
DR = −1999
Yor1
miss 0
orf 0.9
int −11.2
rank 4107
yor1−dF
miss 0
orf 0.7
int −3
rank 2108
(YNR064C)
DR = −1297
Yor1
miss 0
orf 6.6
int −5.8
rank 3406
yor1−dF
miss 0
orf 2
int −3
rank 2109
(YER097W)
DR = 609
Yor1
miss 0
orf −4
int 2.4
rank 1501
yor1−dF
miss 0
orf −1.6
int −3
rank 2110
IDS2(YJL146W)
DR = 1793
Yor1
miss 0
orf −1.9
int 11.3
rank 318
yor1−dF
miss 0
orf 2.8
int −3
rank 2111
PHM6(YDR281C)
DR = 1224
Yor1
miss 0
orf −0.8
int 5.5
rank 888
yor1−dF
miss 0
orf 1.5
int −3
rank 2112
RRD2(YPL152W)
DR = −1825
Yor1
miss 0
orf 8.6
int −9.5
rank 3938
yor1−dF
miss 0
orf −1.3
int −3.1
rank 2113
ABP1(YCR088W)
DR = −2079
Yor1
miss 0
orf 6.6
int −12.3
rank 4193
yor1−dF
miss 0
orf −2.1
int −3.1
rank 2114
0.35
0.25
0.20
0.15
0.10
0.05
0.00
−60
−20
0
20
40
−50
0
50

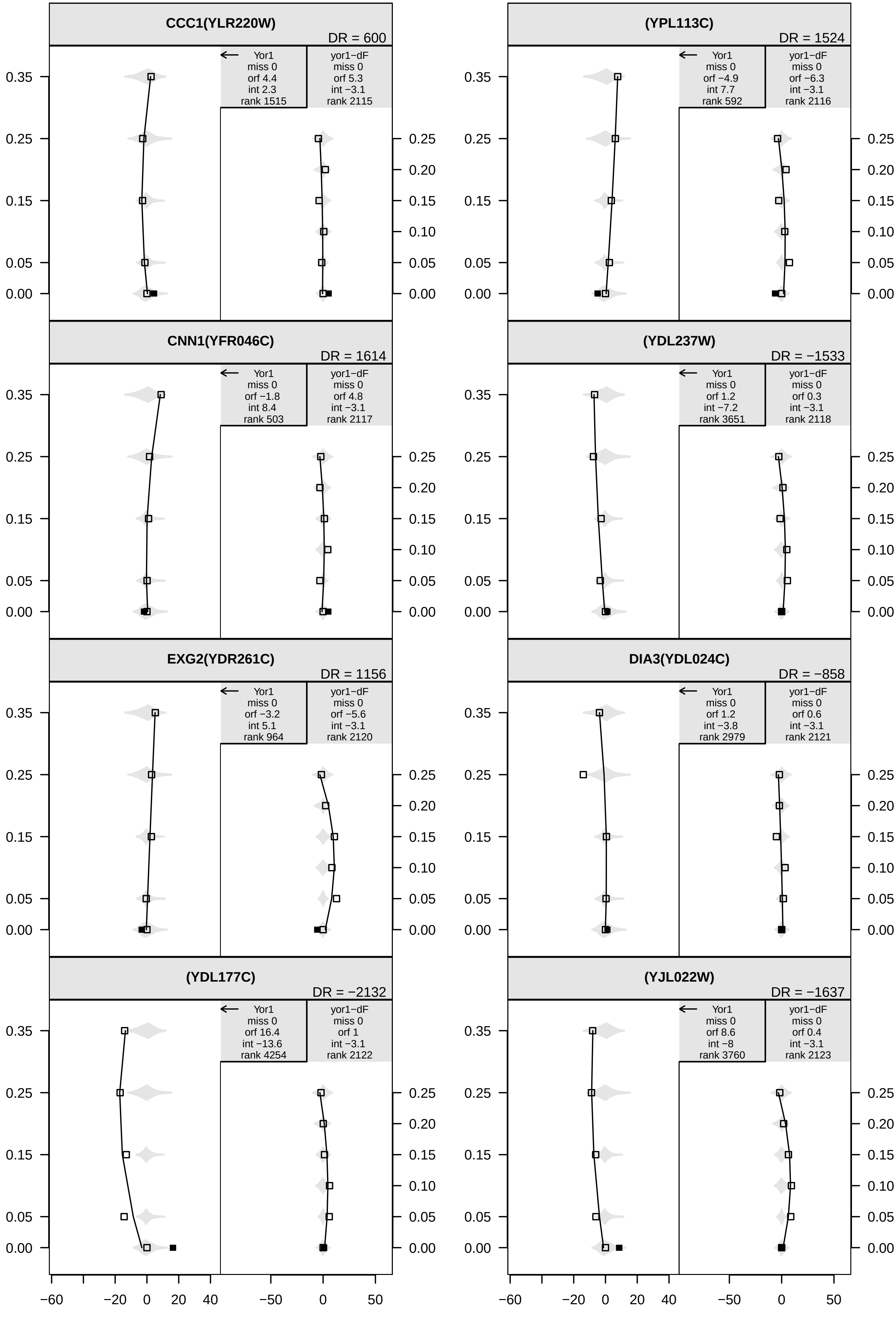

CCC1(YLR220W)
DR = 600
Yor1 miss 0 orf 4.4 int 2.3 rank 1515
yor1−dF miss 0 orf 5.3 int −3.1 rank 2115
(YPL113C)
DR = 1524
Yor1 miss 0 orf −4.9 int 7.7 rank 592
yor1−dF miss 0 orf −6.3 int −3.1 rank 2116
CNN1(YFR046C)
DR = 1614
Yor1 miss 0 orf −1.8 int 8.4 rank 503
yor1−dF miss 0 orf 4.8 int −3.1 rank 2117
(YDL237W)
DR = −1533
Yor1 miss 0 orf 1.2 int −7.2 rank 3651
yor1−dF miss 0 orf 0.3 int −3.1 rank 2118
EXG2(YDR261C)
DR = 1156
Yor1 miss 0 orf −3.2 int 5.1 rank 964
yor1−dF miss 0 orf −5.6 int −3.1 rank 2120
DIA3(YDL024C)
DR = −858
Yor1 miss 0 orf 1.2 int −3.8 rank 2979
yor1−dF miss 0 orf 0.6 int −3.1 rank 2121
(YDL177C)
DR = −2132
Yor1 miss 0 orf 16.4 int −13.6 rank 4254
yor1−dF miss 0 orf 1 int −3.1 rank 2122
(YJL022W)
DR = −1637
Yor1 miss 0 orf 8.6 int −8 rank 3760
yor1−dF miss 0 orf 0.4 int −3.1 rank 2123
0.00 0.05 0.10 0.15 0.20 0.25 0.35
−60 −20 0 20 40 −50 0 50

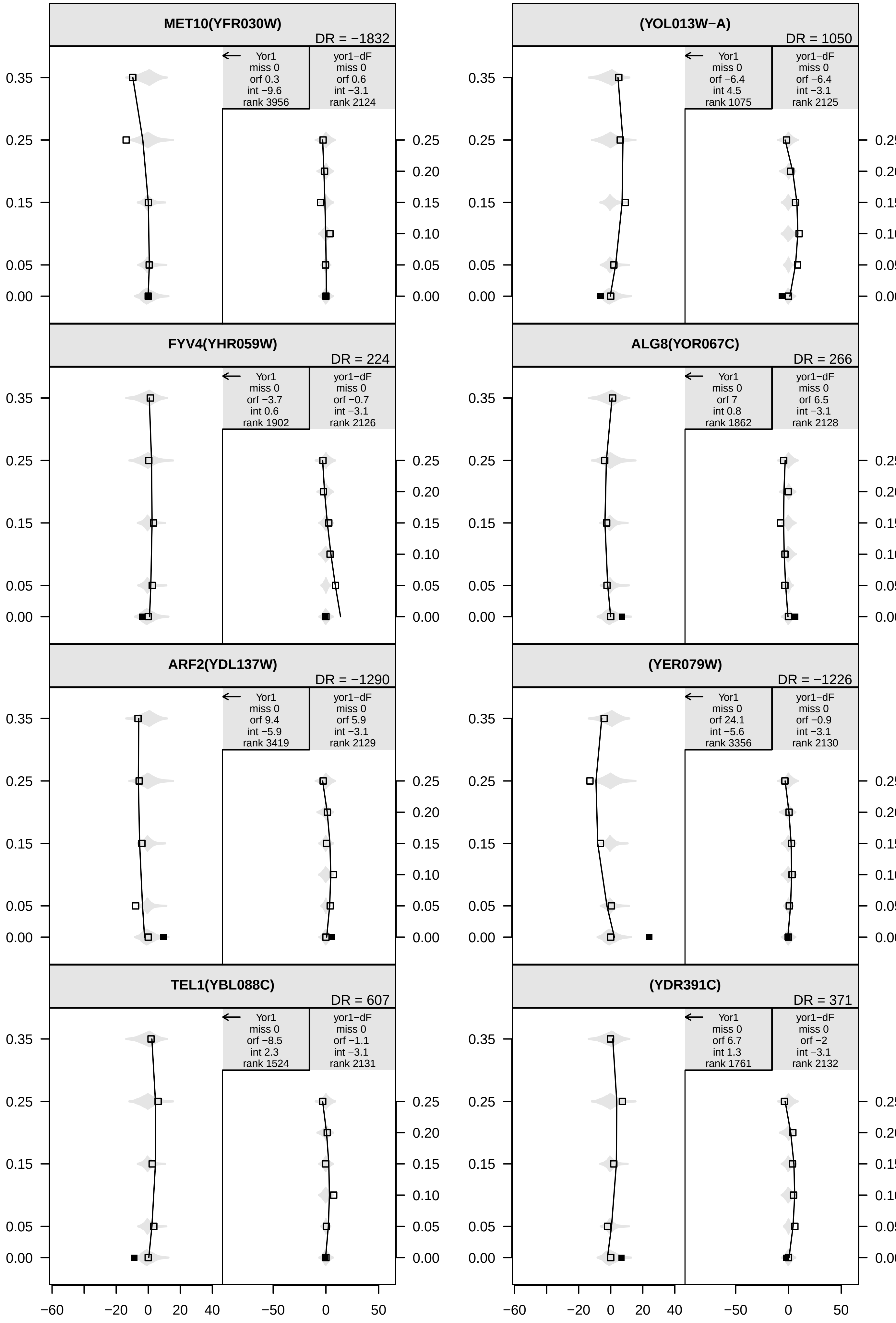

MET10(YFR030W)
DR = −1832
Yor1 miss 0 orf 0.3 int −9.6 rank 3956
yor1−dF miss 0 orf 0.6 int −3.1 rank 2124
(YOL013W−A)
DR = 1050
Yor1 miss 0 orf −6.4 int 4.5 rank 1075
yor1−dF miss 0 orf −6.4 int −3.1 rank 2125
FYV4(YHR059W)
DR = 224
Yor1 miss 0 orf −3.7 int 0.6 rank 1902
yor1−dF miss 0 orf −0.7 int −3.1 rank 2126
ALG8(YOR067C)
DR = 266
Yor1 miss 0 orf 7 int 0.8 rank 1862
yor1−dF miss 0 orf 6.5 int −3.1 rank 2128
ARF2(YDL137W)
DR = −1290
Yor1 miss 0 orf 9.4 int −5.9 rank 3419
yor1−dF miss 0 orf 5.9 int −3.1 rank 2129
(YER079W)
DR = −1226
Yor1 miss 0 orf 24.1 int −5.6 rank 3356
yor1−dF miss 0 orf −0.9 int −3.1 rank 2130
TEL1(YBL088C)
DR = 607
Yor1 miss 0 orf −8.5 int 2.3 rank 1524
yor1−dF miss 0 orf −1.1 int −3.1 rank 2131
(YDR391C)
DR = 371
Yor1 miss 0 orf 6.7 int 1.3 rank 1761
yor1−dF miss 0 orf −2 int −3.1 rank 2132
0.35
0.25
0.20
0.15
0.10
0.05
0.00
−60
−20
0
20
40
−50
0
50

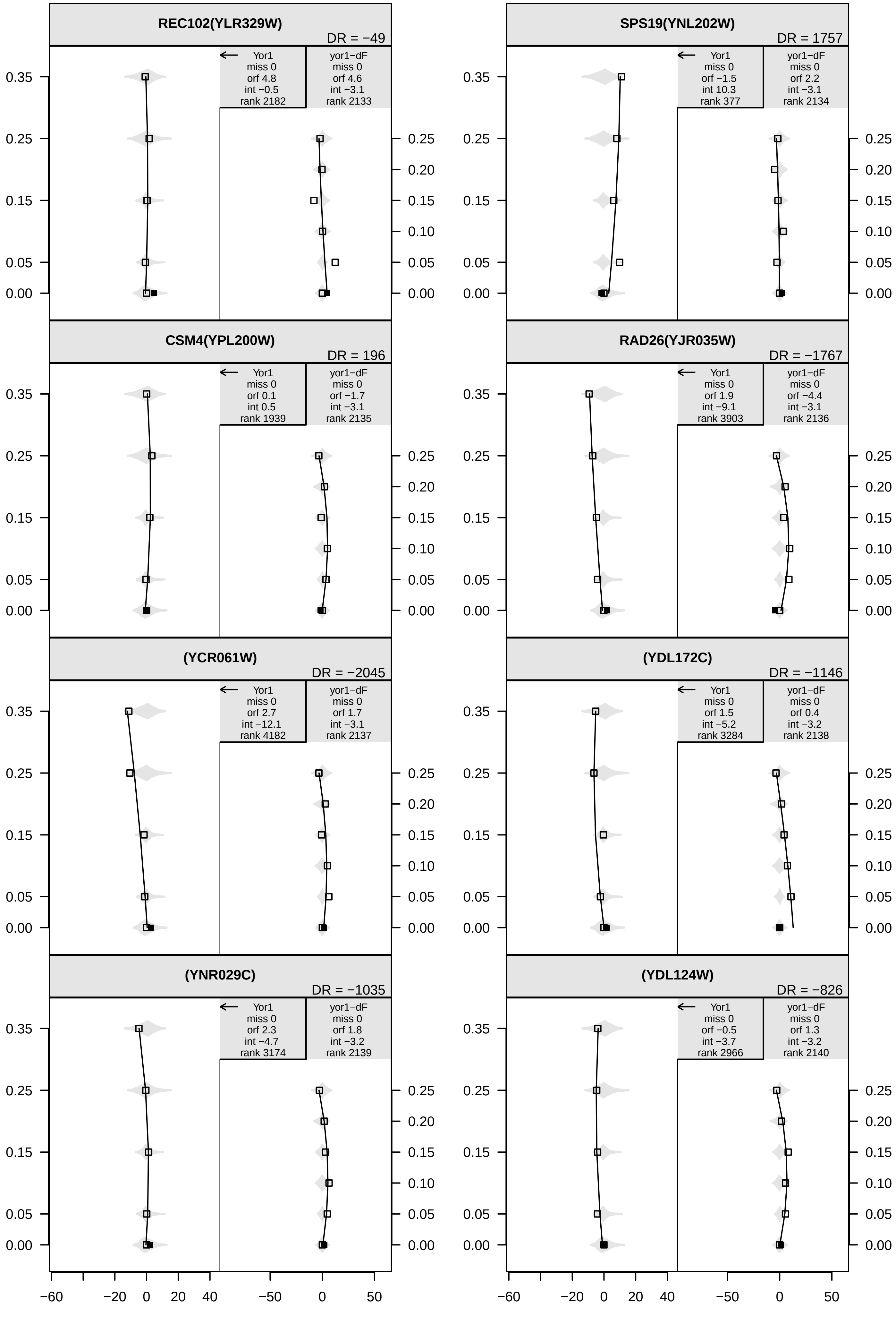
REC102(YLR329W)
DR = −49
Yor1 miss 0 orf 4.8 int −0.5 rank 2182
yor1−dF miss 0 orf 4.6 int −3.1 rank 2133
SPS19(YNL202W)
DR = 1757
Yor1 miss 0 orf −1.5 int 10.3 rank 377
yor1−dF miss 0 orf 2.2 int −3.1 rank 2134
CSM4(YPL200W)
DR = 196
Yor1 miss 0 orf 0.1 int 0.5 rank 1939
yor1−dF miss 0 orf −1.7 int −3.1 rank 2135
RAD26(YJR035W)
DR = −1767
Yor1 miss 0 orf 1.9 int −9.1 rank 3903
yor1−dF miss 0 orf −4.4 int −3.1 rank 2136
(YCR061W)
DR = −2045
Yor1 miss 0 orf 2.7 int −12.1 rank 4182
yor1−dF miss 0 orf 1.7 int −3.1 rank 2137
(YDL172C)
DR = −1146
Yor1 miss 0 orf 1.5 int −5.2 rank 3284
yor1−dF miss 0 orf 0.4 int −3.2 rank 2138
(YNR029C)
DR = −1035
Yor1 miss 0 orf 2.3 int −4.7 rank 3174
yor1−dF miss 0 orf 1.8 int −3.2 rank 2139
(YDL124W)
DR = −826
Yor1 miss 0 orf −0.5 int −3.7 rank 2966
yor1−dF miss 0 orf 1.3 int −3.2 rank 2140
0.35 0.25 0.20 0.15 0.10 0.05 0.00
−60 −20 0 20 40 −50 0 50

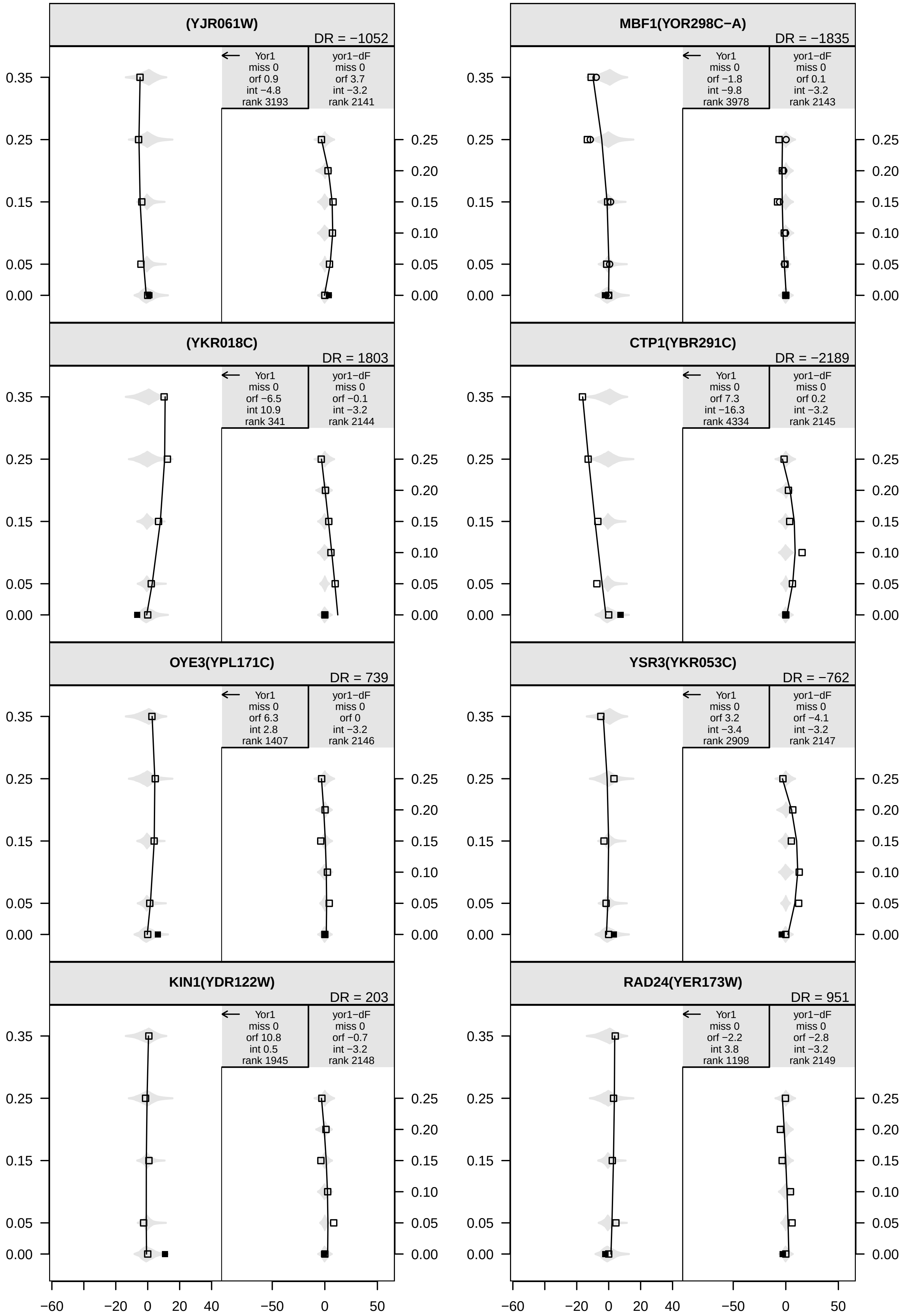

(YJR061W)
DR = −1052
Yor1 miss 0 orf 0.9 int −4.8 rank 3193
yor1−dF miss 0 orf 3.7 int −3.2 rank 2141
MBF1(YOR298C−A)
DR = −1835
Yor1 miss 0 orf −1.8 int −9.8 rank 3978
yor1−dF miss 0 orf 0.1 int −3.2 rank 2143
(YKR018C)
DR = 1803
Yor1 miss 0 orf −6.5 int 10.9 rank 341
yor1−dF miss 0 orf −0.1 int −3.2 rank 2144
CTP1(YBR291C)
DR = −2189
Yor1 miss 0 orf 7.3 int −16.3 rank 4334
yor1−dF miss 0 orf 0.2 int −3.2 rank 2145
OYE3(YPL171C)
DR = 739
Yor1 miss 0 orf 6.3 int 2.8 rank 1407
yor1−dF miss 0 orf 0 int −3.2 rank 2146
YSR3(YKR053C)
DR = −762
Yor1 miss 0 orf 3.2 int −3.4 rank 2909
yor1−dF miss 0 orf −4.1 int −3.2 rank 2147
KIN1(YDR122W)
DR = 203
Yor1 miss 0 orf 10.8 int 0.5 rank 1945
yor1−dF miss 0 orf −0.7 int −3.2 rank 2148
RAD24(YER173W)
DR = 951
Yor1 miss 0 orf −2.2 int 3.8 rank 1198
yor1−dF miss 0 orf −2.8 int −3.2 rank 2149
0.35 0.25 0.15 0.05 0.00
0.25 0.20 0.15 0.10 0.05 0.00
−60 −20 0 20 40 −50 0 50

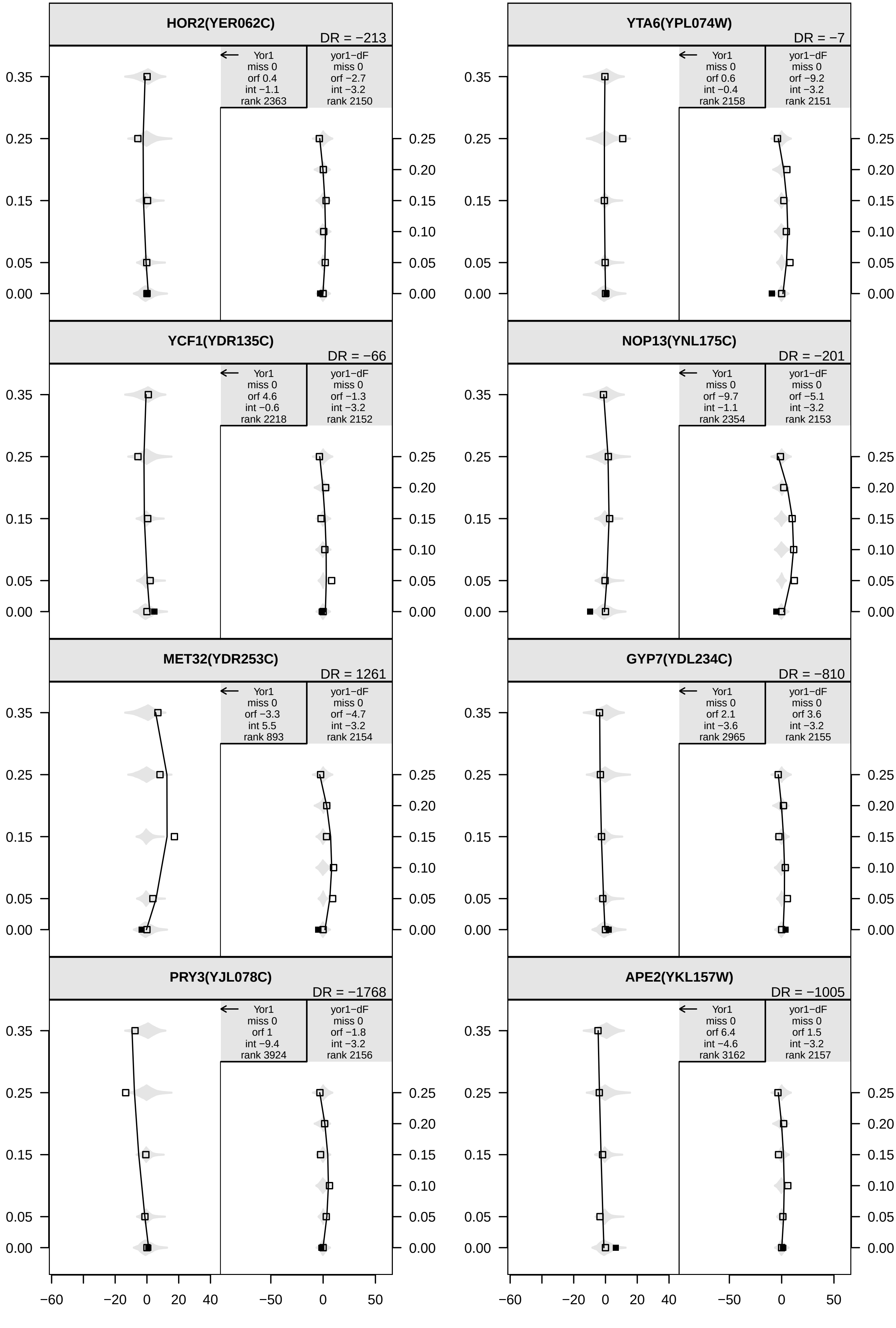

HOR2(YER062C)
DR = −213
Yor1 miss 0 orf 0.4 int −1.1 rank 2363
yor1−dF miss 0 orf −2.7 int −3.2 rank 2150
YTA6(YPL074W)
DR = −7
Yor1 miss 0 orf 0.6 int −0.4 rank 2158
yor1−dF miss 0 orf −9.2 int −3.2 rank 2151
YCF1(YDR135C)
DR = −66
Yor1 miss 0 orf 4.6 int −0.6 rank 2218
yor1−dF miss 0 orf −1.3 int −3.2 rank 2152
NOP13(YNL175C)
DR = −201
Yor1 miss 0 orf −9.7 int −1.1 rank 2354
yor1−dF miss 0 orf −5.1 int −3.2 rank 2153
MET32(YDR253C)
DR = 1261
Yor1 miss 0 orf −3.3 int 5.5 rank 893
yor1−dF miss 0 orf −4.7 int −3.2 rank 2154
GYP7(YDL234C)
DR = −810
Yor1 miss 0 orf 2.1 int −3.6 rank 2965
yor1−dF miss 0 orf 3.6 int −3.2 rank 2155
PRY3(YJL078C)
DR = −1768
Yor1 miss 0 orf 1 int −9.4 rank 3924
yor1−dF miss 0 orf −1.8 int −3.2 rank 2156
APE2(YKL157W)
DR = −1005
Yor1 miss 0 orf 6.4 int −4.6 rank 3162
yor1−dF miss 0 orf 1.5 int −3.2 rank 2157

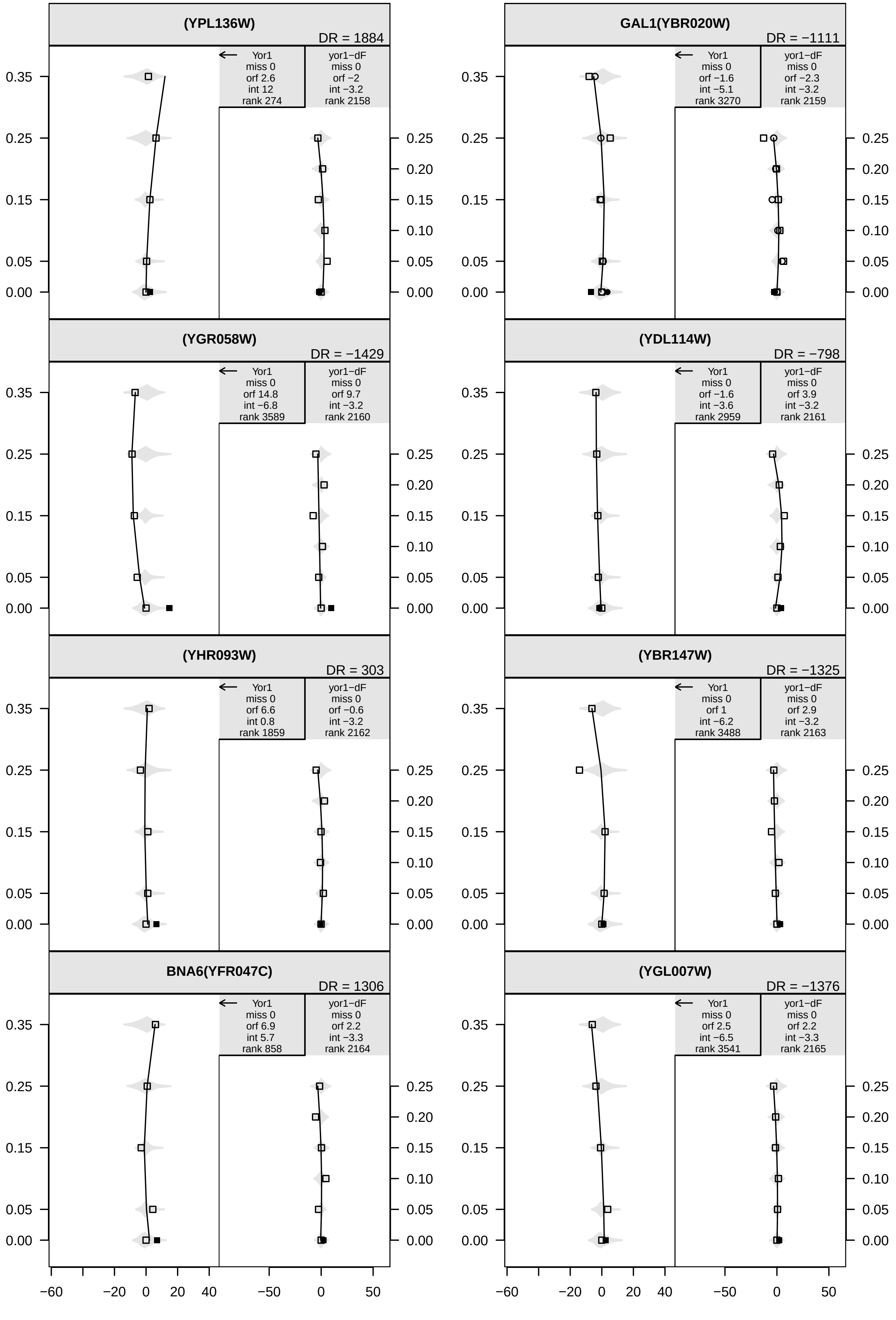
(YPL136W)
DR = 1884
Yor1 miss 0 orf 2.6 int 12 rank 274
yor1−dF miss 0 orf −2 int −3.2 rank 2158
GAL1(YBR020W)
DR = −1111
Yor1 miss 0 orf −1.6 int −5.1 rank 3270
yor1−dF miss 0 orf −2.3 int −3.2 rank 2159
(YGR058W)
DR = −1429
Yor1 miss 0 orf 14.8 int −6.8 rank 3589
yor1−dF miss 0 orf 9.7 int −3.2 rank 2160
(YDL114W)
DR = −798
Yor1 miss 0 orf −1.6 int −3.6 rank 2959
yor1−dF miss 0 orf 3.9 int −3.2 rank 2161
(YHR093W)
DR = 303
Yor1 miss 0 orf 6.6 int 0.8 rank 1859
yor1−dF miss 0 orf −0.6 int −3.2 rank 2162
(YBR147W)
DR = −1325
Yor1 miss 0 orf 1 int −6.2 rank 3488
yor1−dF miss 0 orf 2.9 int −3.2 rank 2163
BNA6(YFR047C)
DR = 1306
Yor1 miss 0 orf 6.9 int 5.7 rank 858
yor1−dF miss 0 orf 2.2 int −3.3 rank 2164
(YGL007W)
DR = −1376
Yor1 miss 0 orf 2.5 int −6.5 rank 3541
yor1−dF miss 0 orf 2.2 int −3.3 rank 2165
0.00 0.05 0.10 0.15 0.20 0.25 0.35
−60 −20 0 20 40 −50 0 50

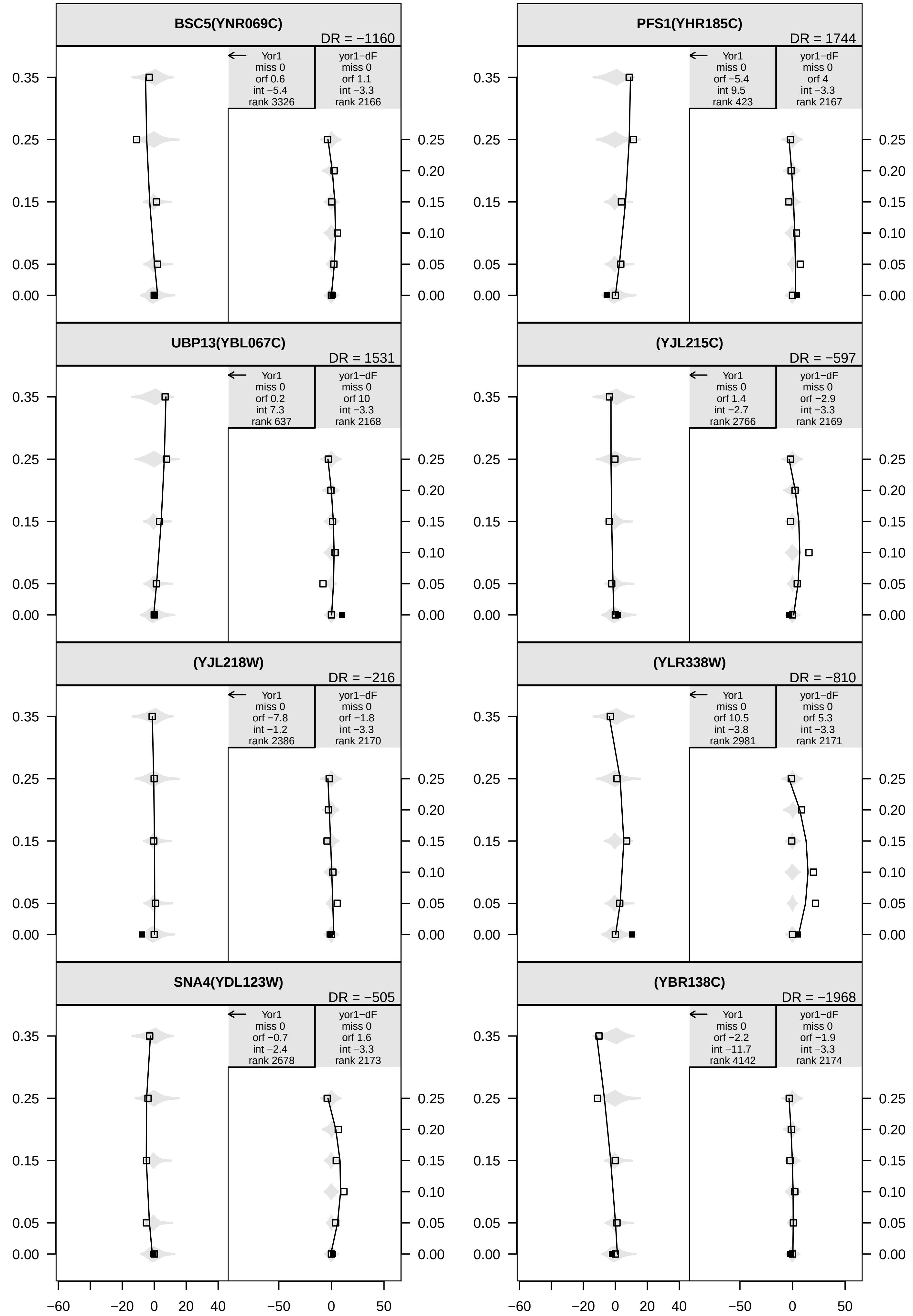

BSC5(YNR069C)
DR = −1160
Yor1 miss 0 orf 0.6 int −5.4 rank 3326
yor1−dF miss 0 orf 1.1 int −3.3 rank 2166
PFS1(YHR185C)
DR = 1744
Yor1 miss 0 orf −5.4 int 9.5 rank 423
yor1−dF miss 0 orf 4 int −3.3 rank 2167
UBP13(YBL067C)
DR = 1531
Yor1 miss 0 orf 0.2 int 7.3 rank 637
yor1−dF miss 0 orf 10 int −3.3 rank 2168
(YJL215C)
DR = −597
Yor1 miss 0 orf 1.4 int −2.7 rank 2766
yor1−dF miss 0 orf −2.9 int −3.3 rank 2169
(YJL218W)
DR = −216
Yor1 miss 0 orf −7.8 int −1.2 rank 2386
yor1−dF miss 0 orf −1.8 int −3.3 rank 2170
(YLR338W)
DR = −810
Yor1 miss 0 orf 10.5 int −3.8 rank 2981
yor1−dF miss 0 orf 5.3 int −3.3 rank 2171
SNA4(YDL123W)
DR = −505
Yor1 miss 0 orf −0.7 int −2.4 rank 2678
yor1−dF miss 0 orf 1.6 int −3.3 rank 2173
(YBR138C)
DR = −1968
Yor1 miss 0 orf −2.2 int −11.7 rank 4142
yor1−dF miss 0 orf −1.9 int −3.3 rank 2174
0.00 0.05 0.10 0.15 0.20 0.25 0.35
−60 −20 0 20 40 −50 0 50

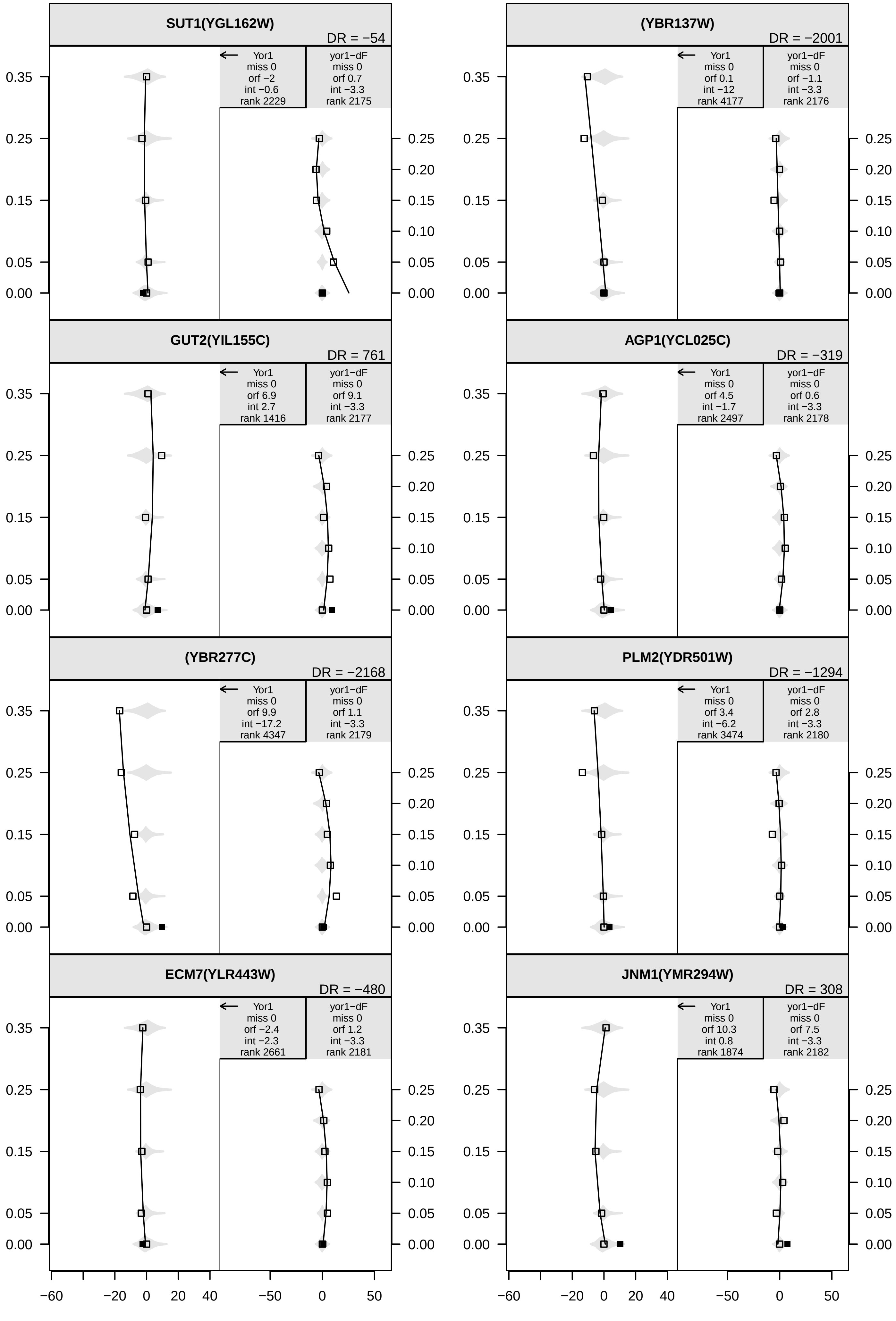

SUT1(YGL162W)
DR = −54
Yor1 miss 0 orf −2 int −0.6 rank 2229
yor1−dF miss 0 orf 0.7 int −3.3 rank 2175
(YBR137W)
DR = −2001
Yor1 miss 0 orf 0.1 int −12 rank 4177
yor1−dF miss 0 orf −1.1 int −3.3 rank 2176
GUT2(YIL155C)
DR = 761
Yor1 miss 0 orf 6.9 int 2.7 rank 1416
yor1−dF miss 0 orf 9.1 int −3.3 rank 2177
AGP1(YCL025C)
DR = −319
Yor1 miss 0 orf 4.5 int −1.7 rank 2497
yor1−dF miss 0 orf 0.6 int −3.3 rank 2178
(YBR277C)
DR = −2168
Yor1 miss 0 orf 9.9 int −17.2 rank 4347
yor1−dF miss 0 orf 1.1 int −3.3 rank 2179
PLM2(YDR501W)
DR = −1294
Yor1 miss 0 orf 3.4 int −6.2 rank 3474
yor1−dF miss 0 orf 2.8 int −3.3 rank 2180
ECM7(YLR443W)
DR = −480
Yor1 miss 0 orf −2.4 int −2.3 rank 2661
yor1−dF miss 0 orf 1.2 int −3.3 rank 2181
JNM1(YMR294W)
DR = 308
Yor1 miss 0 orf 10.3 int 0.8 rank 1874
yor1−dF miss 0 orf 7.5 int −3.3 rank 2182

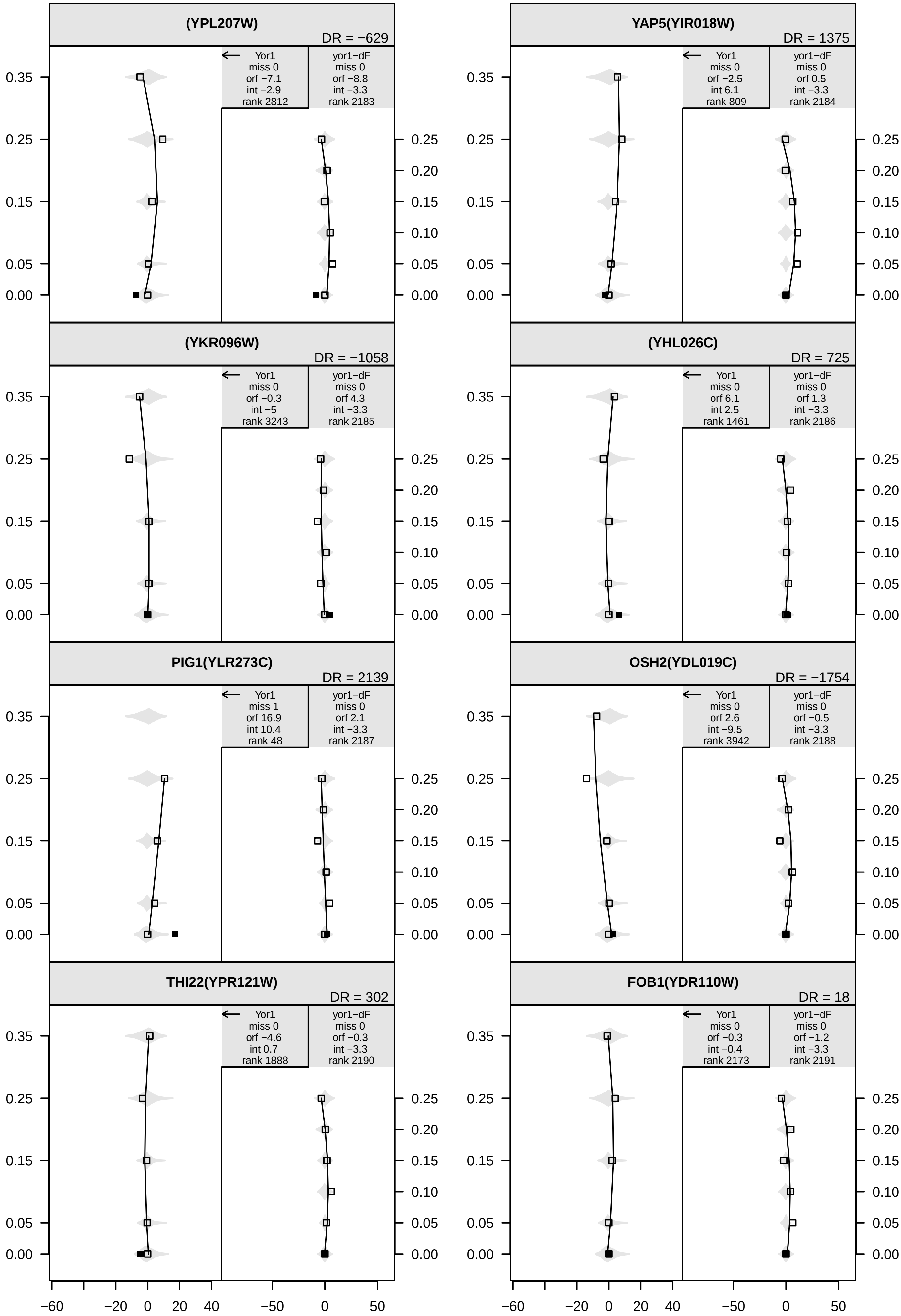

(YPL207W)
DR = −629
Yor1 miss 0 orf −7.1 int −2.9 rank 2812
yor1−dF miss 0 orf −8.8 int −3.3 rank 2183
YAP5(YIR018W)
DR = 1375
Yor1 miss 0 orf −2.5 int 6.1 rank 809
yor1−dF miss 0 orf 0.5 int −3.3 rank 2184
(YKR096W)
DR = −1058
Yor1 miss 0 orf −0.3 int −5 rank 3243
yor1−dF miss 0 orf 4.3 int −3.3 rank 2185
(YHL026C)
DR = 725
Yor1 miss 0 orf 6.1 int 2.5 rank 1461
yor1−dF miss 0 orf 1.3 int −3.3 rank 2186
PIG1(YLR273C)
DR = 2139
Yor1 miss 1 orf 16.9 int 10.4 rank 48
yor1−dF miss 0 orf 2.1 int −3.3 rank 2187
OSH2(YDL019C)
DR = −1754
Yor1 miss 0 orf 2.6 int −9.5 rank 3942
yor1−dF miss 0 orf −0.5 int −3.3 rank 2188
THI22(YPR121W)
DR = 302
Yor1 miss 0 orf −4.6 int 0.7 rank 1888
yor1−dF miss 0 orf −0.3 int −3.3 rank 2190
FOB1(YDR110W)
DR = 18
Yor1 miss 0 orf −0.3 int −0.4 rank 2173
yor1−dF miss 0 orf −1.2 int −3.3 rank 2191

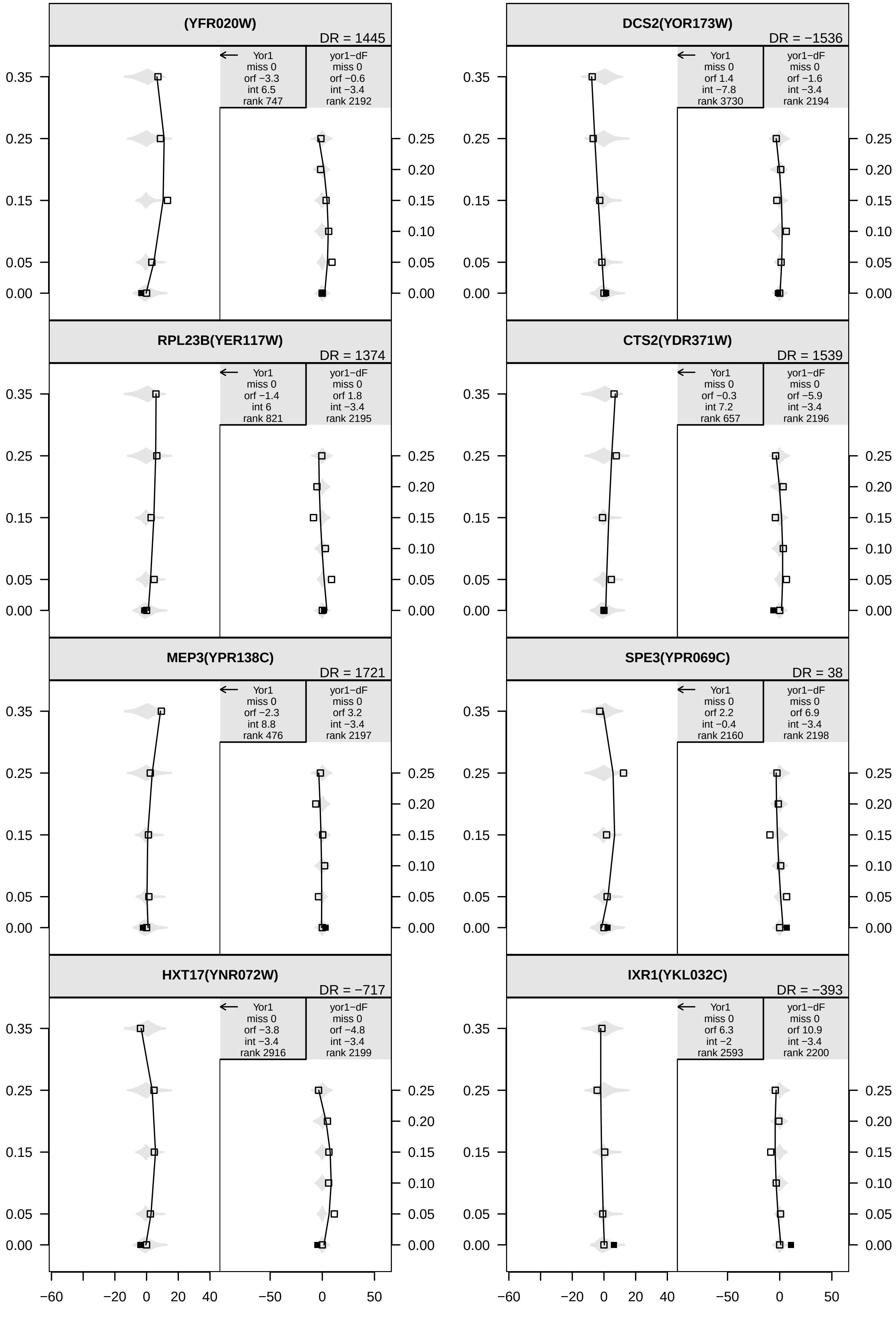

(YFR020W)
DR = 1445
Yor1 miss 0 orf −3.3 int 6.5 rank 747
yor1−dF miss 0 orf −0.6 int −3.4 rank 2192
DCS2(YOR173W)
DR = −1536
Yor1 miss 0 orf 1.4 int −7.8 rank 3730
yor1−dF miss 0 orf −1.6 int −3.4 rank 2194
RPL23B(YER117W)
DR = 1374
Yor1 miss 0 orf −1.4 int 6 rank 821
yor1−dF miss 0 orf 1.8 int −3.4 rank 2195
CTS2(YDR371W)
DR = 1539
Yor1 miss 0 orf −0.3 int 7.2 rank 657
yor1−dF miss 0 orf −5.9 int −3.4 rank 2196
MEP3(YPR138C)
DR = 1721
Yor1 miss 0 orf −2.3 int 8.8 rank 476
yor1−dF miss 0 orf 3.2 int −3.4 rank 2197
SPE3(YPR069C)
DR = 38
Yor1 miss 0 orf 2.2 int −0.4 rank 2160
yor1−dF miss 0 orf 6.9 int −3.4 rank 2198
HXT17(YNR072W)
DR = −717
Yor1 miss 0 orf −3.8 int −3.4 rank 2916
yor1−dF miss 0 orf −4.8 int −3.4 rank 2199
IXR1(YKL032C)
DR = −393
Yor1 miss 0 orf 6.3 int −2 rank 2593
yor1−dF miss 0 orf 10.9 int −3.4 rank 2200

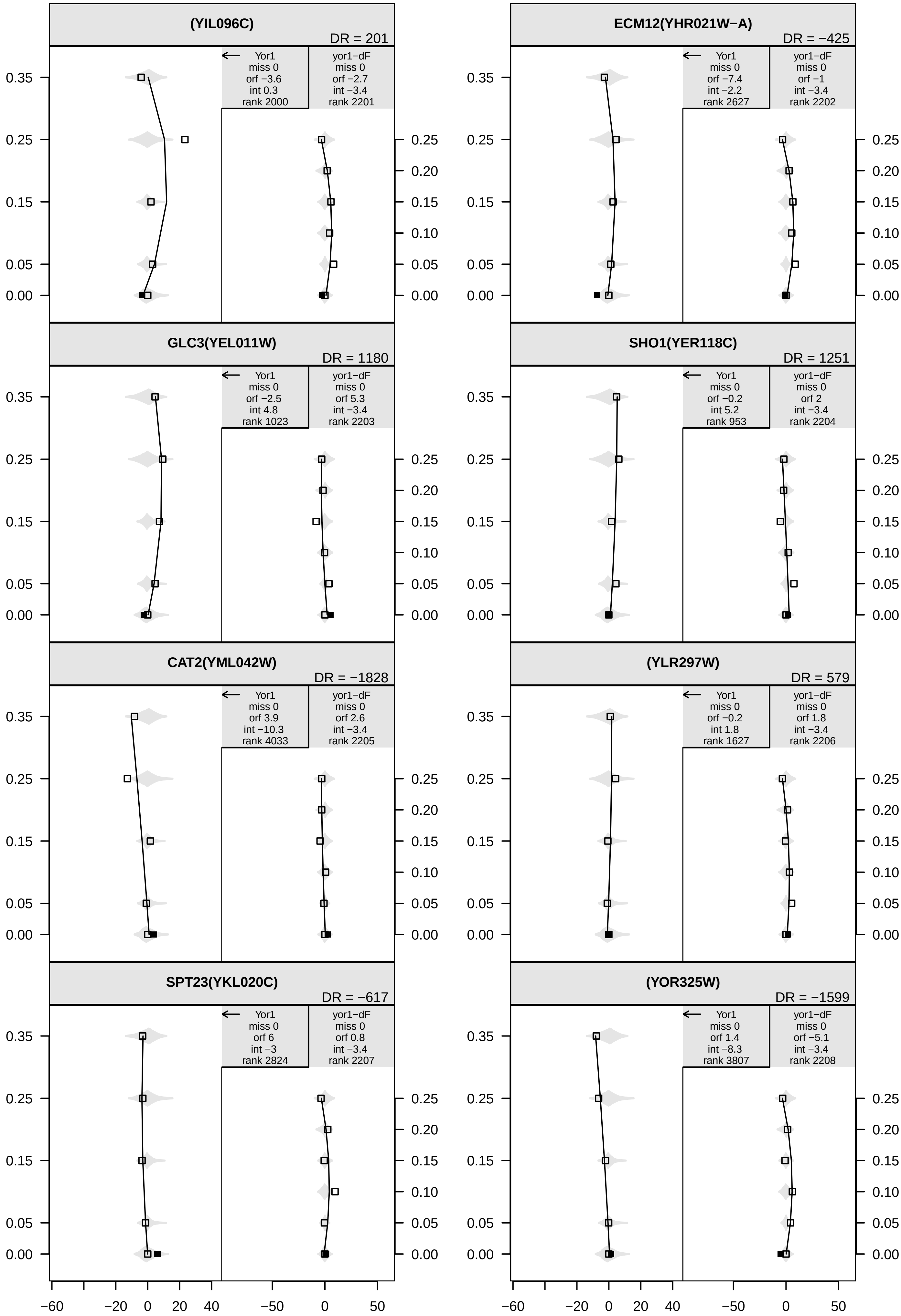

(YIL096C)
DR = 201
Yor1
miss 0
orf −3.6
int 0.3
rank 2000
yor1−dF
miss 0
orf −2.7
int −3.4
rank 2201
ECM12(YHR021W−A)
DR = −425
Yor1
miss 0
orf −7.4
int −2.2
rank 2627
yor1−dF
miss 0
orf −1
int −3.4
rank 2202
GLC3(YEL011W)
DR = 1180
Yor1
miss 0
orf −2.5
int 4.8
rank 1023
yor1−dF
miss 0
orf 5.3
int −3.4
rank 2203
SHO1(YER118C)
DR = 1251
Yor1
miss 0
orf −0.2
int 5.2
rank 953
yor1−dF
miss 0
orf 2
int −3.4
rank 2204
CAT2(YML042W)
DR = −1828
Yor1
miss 0
orf 3.9
int −10.3
rank 4033
yor1−dF
miss 0
orf 2.6
int −3.4
rank 2205
(YLR297W)
DR = 579
Yor1
miss 0
orf −0.2
int 1.8
rank 1627
yor1−dF
miss 0
orf 1.8
int −3.4
rank 2206
SPT23(YKL020C)
DR = −617
Yor1
miss 0
orf 6
int −3
rank 2824
yor1−dF
miss 0
orf 0.8
int −3.4
rank 2207
(YOR325W)
DR = −1599
Yor1
miss 0
orf 1.4
int −8.3
rank 3807
yor1−dF
miss 0
orf −5.1
int −3.4
rank 2208
0.35
0.25
0.20
0.15
0.10
0.05
0.00
−60
−20
0
20
40
−50
0
50

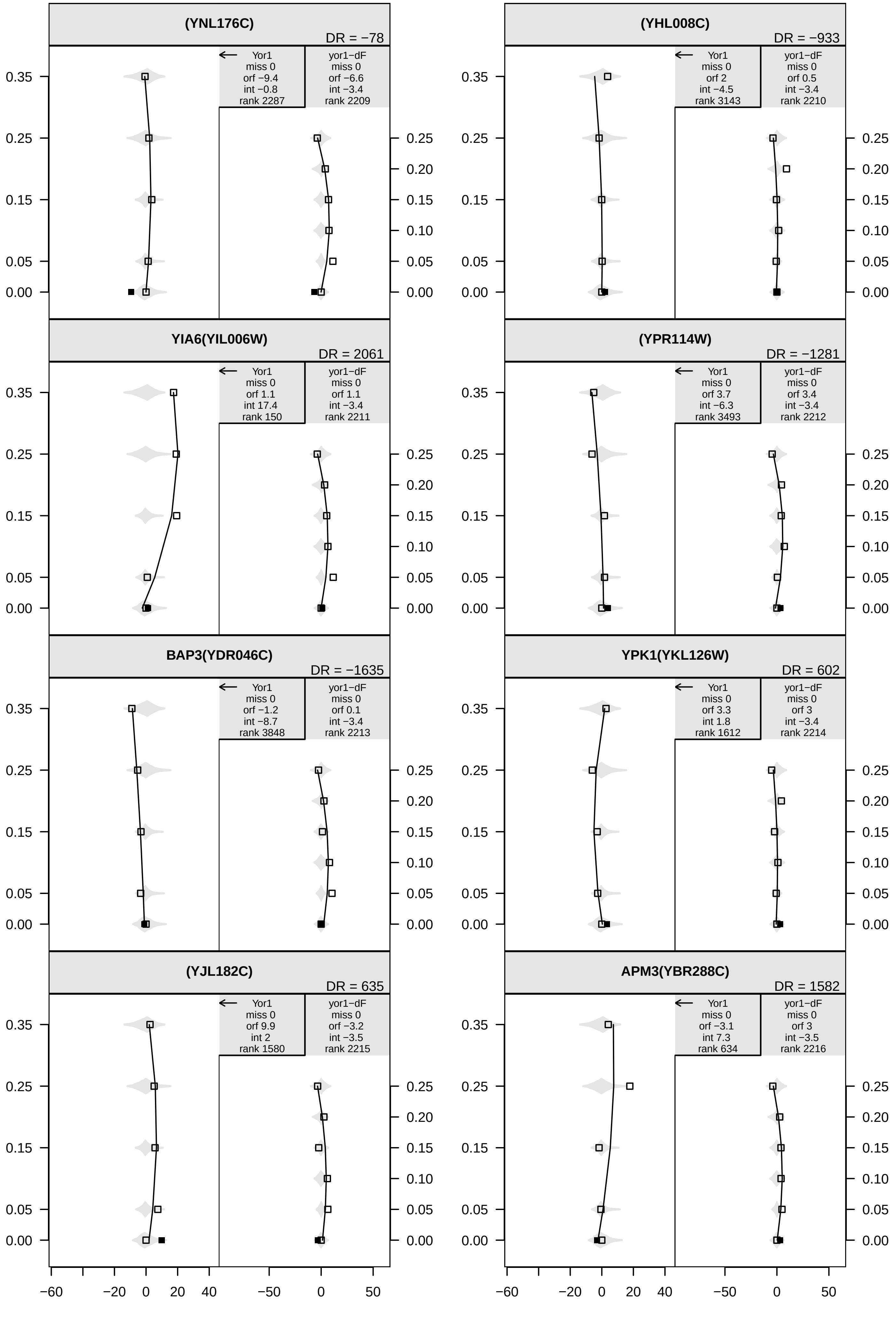
(YNL176C)
DR = −78
Yor1 miss 0 orf −9.4 int −0.8 rank 2287
yor1−dF miss 0 orf −6.6 int −3.4 rank 2209
(YHL008C)
DR = −933
Yor1 miss 0 orf 2 int −4.5 rank 3143
yor1−dF miss 0 orf 0.5 int −3.4 rank 2210
YIA6(YIL006W)
DR = 2061
Yor1 miss 0 orf 1.1 int 17.4 rank 150
yor1−dF miss 0 orf 1.1 int −3.4 rank 2211
(YPR114W)
DR = −1281
Yor1 miss 0 orf 3.7 int −6.3 rank 3493
yor1−dF miss 0 orf 3.4 int −3.4 rank 2212
BAP3(YDR046C)
DR = −1635
Yor1 miss 0 orf −1.2 int −8.7 rank 3848
yor1−dF miss 0 orf 0.1 int −3.4 rank 2213
YPK1(YKL126W)
DR = 602
Yor1 miss 0 orf 3.3 int 1.8 rank 1612
yor1−dF miss 0 orf 3 int −3.4 rank 2214
(YJL182C)
DR = 635
Yor1 miss 0 orf 9.9 int 2 rank 1580
yor1−dF miss 0 orf −3.2 int −3.5 rank 2215
APM3(YBR288C)
DR = 1582
Yor1 miss 0 orf −3.1 int 7.3 rank 634
yor1−dF miss 0 orf 3 int −3.5 rank 2216
0.00 0.05 0.10 0.15 0.20 0.25 0.35
−60 −20 0 20 40 −50 0 50

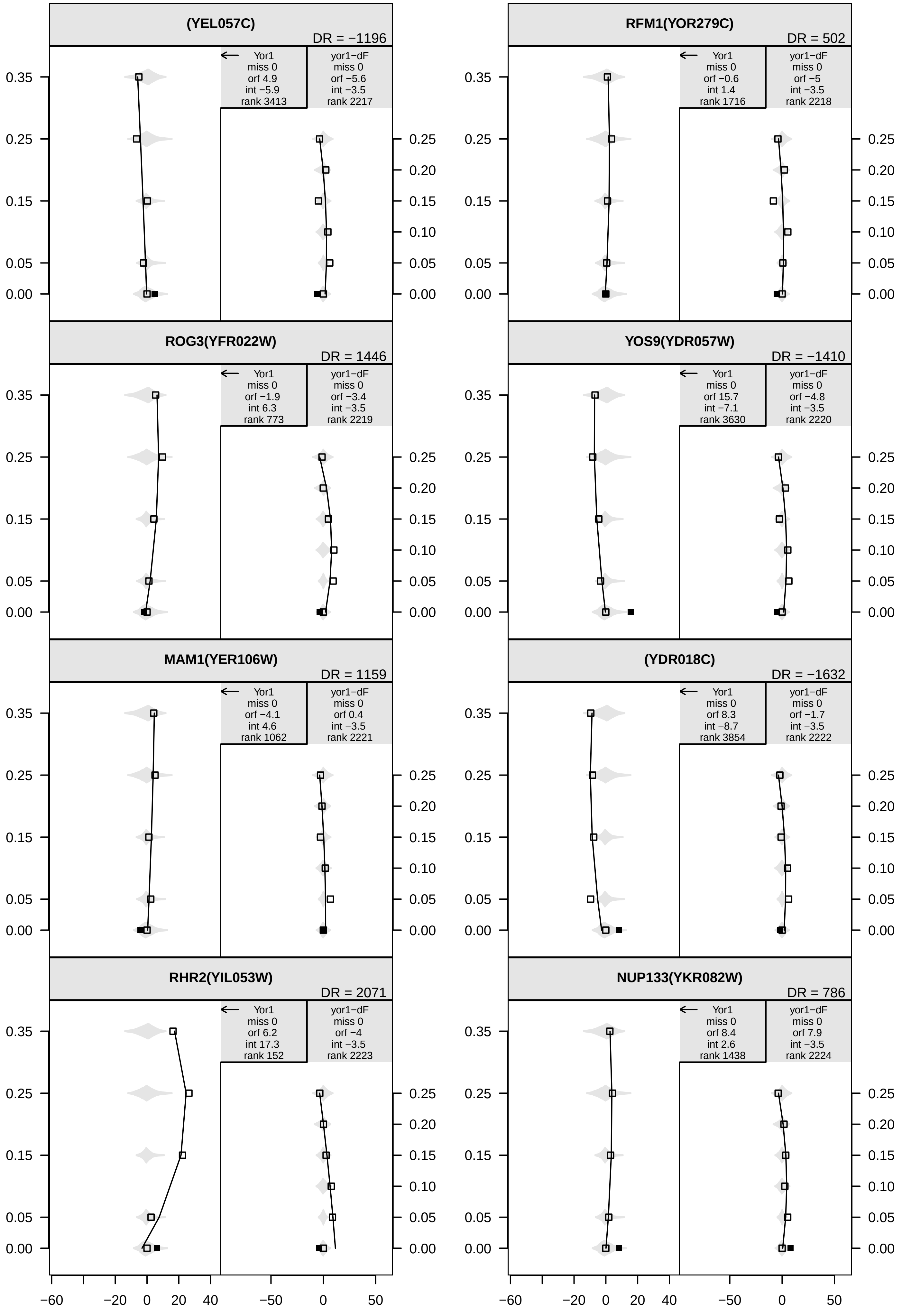

(YEL057C)
DR = −1196
Yor1 miss 0 orf 4.9 int −5.9 rank 3413
yor1−dF miss 0 orf −5.6 int −3.5 rank 2217
RFM1(YOR279C)
DR = 502
Yor1 miss 0 orf −0.6 int 1.4 rank 1716
yor1−dF miss 0 orf −5 int −3.5 rank 2218
ROG3(YFR022W)
DR = 1446
Yor1 miss 0 orf −1.9 int 6.3 rank 773
yor1−dF miss 0 orf −3.4 int −3.5 rank 2219
YOS9(YDR057W)
DR = −1410
Yor1 miss 0 orf 15.7 int −7.1 rank 3630
yor1−dF miss 0 orf −4.8 int −3.5 rank 2220
MAM1(YER106W)
DR = 1159
Yor1 miss 0 orf −4.1 int 4.6 rank 1062
yor1−dF miss 0 orf 0.4 int −3.5 rank 2221
(YDR018C)
DR = −1632
Yor1 miss 0 orf 8.3 int −8.7 rank 3854
yor1−dF miss 0 orf −1.7 int −3.5 rank 2222
RHR2(YIL053W)
DR = 2071
Yor1 miss 0 orf 6.2 int 17.3 rank 152
yor1−dF miss 0 orf −4 int −3.5 rank 2223
NUP133(YKR082W)
DR = 786
Yor1 miss 0 orf 8.4 int 2.6 rank 1438
yor1−dF miss 0 orf 7.9 int −3.5 rank 2224
0.35 0.25 0.15 0.05 0.00
0.25 0.20 0.15 0.10 0.05 0.00
−60 −20 0 20 40
−50 0 50

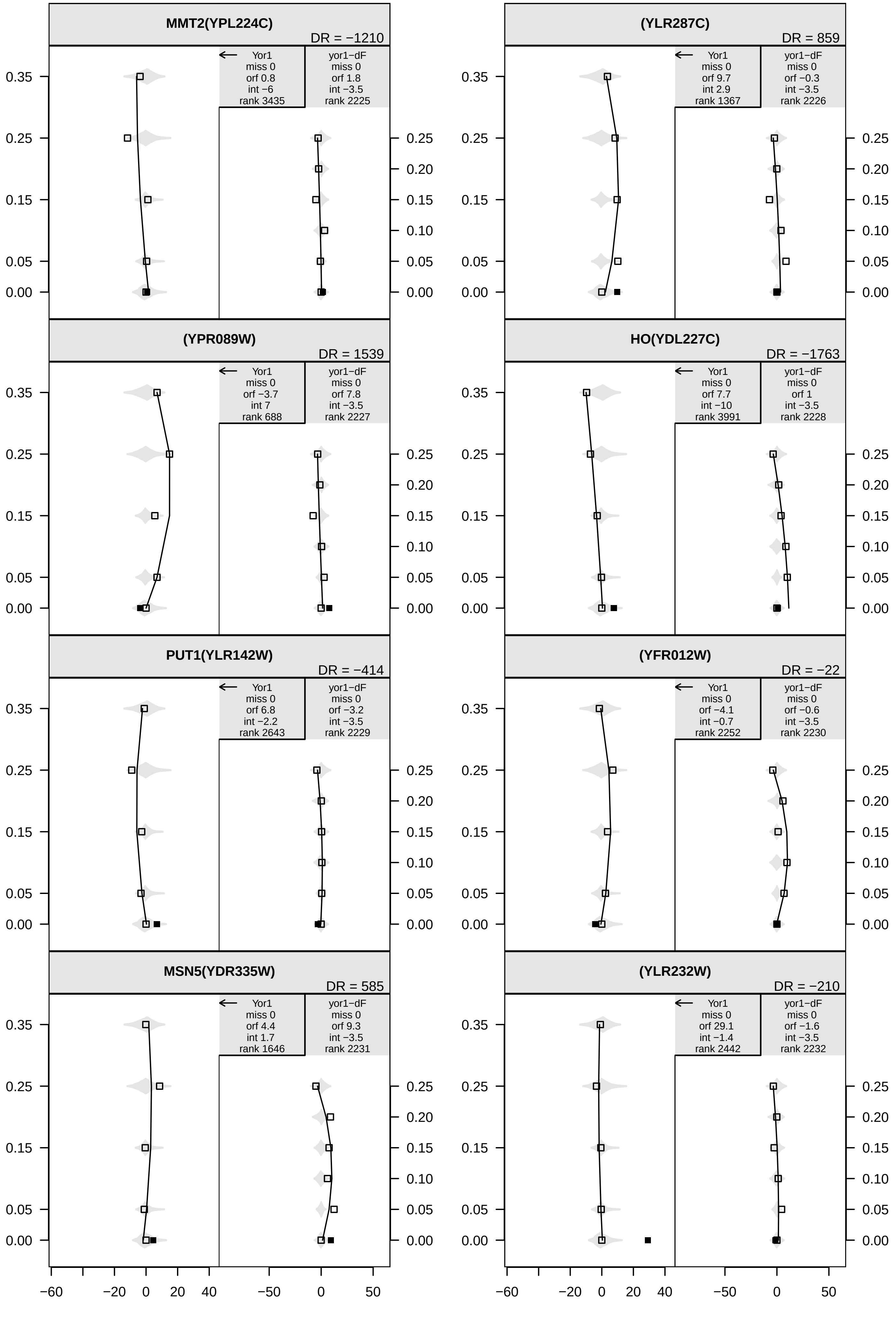

MMT2(YPL224C)
DR = −1210
Yor1 miss 0 orf 0.8 int −6 rank 3435
yor1−dF miss 0 orf 1.8 int −3.5 rank 2225
(YLR287C)
DR = 859
Yor1 miss 0 orf 9.7 int 2.9 rank 1367
yor1−dF miss 0 orf −0.3 int −3.5 rank 2226
(YPR089W)
DR = 1539
Yor1 miss 0 orf −3.7 int 7 rank 688
yor1−dF miss 0 orf 7.8 int −3.5 rank 2227
HO(YDL227C)
DR = −1763
Yor1 miss 0 orf 7.7 int −10 rank 3991
yor1−dF miss 0 orf 1 int −3.5 rank 2228
PUT1(YLR142W)
DR = −414
Yor1 miss 0 orf 6.8 int −2.2 rank 2643
yor1−dF miss 0 orf −3.2 int −3.5 rank 2229
(YFR012W)
DR = −22
Yor1 miss 0 orf −4.1 int −0.7 rank 2252
yor1−dF miss 0 orf −0.6 int −3.5 rank 2230
MSN5(YDR335W)
DR = 585
Yor1 miss 0 orf 4.4 int 1.7 rank 1646
yor1−dF miss 0 orf 9.3 int −3.5 rank 2231
(YLR232W)
DR = −210
Yor1 miss 0 orf 29.1 int −1.4 rank 2442
yor1−dF miss 0 orf −1.6 int −3.5 rank 2232
0.00 0.05 0.10 0.15 0.20 0.25 0.35
−60 −20 0 20 40 −50 0 50

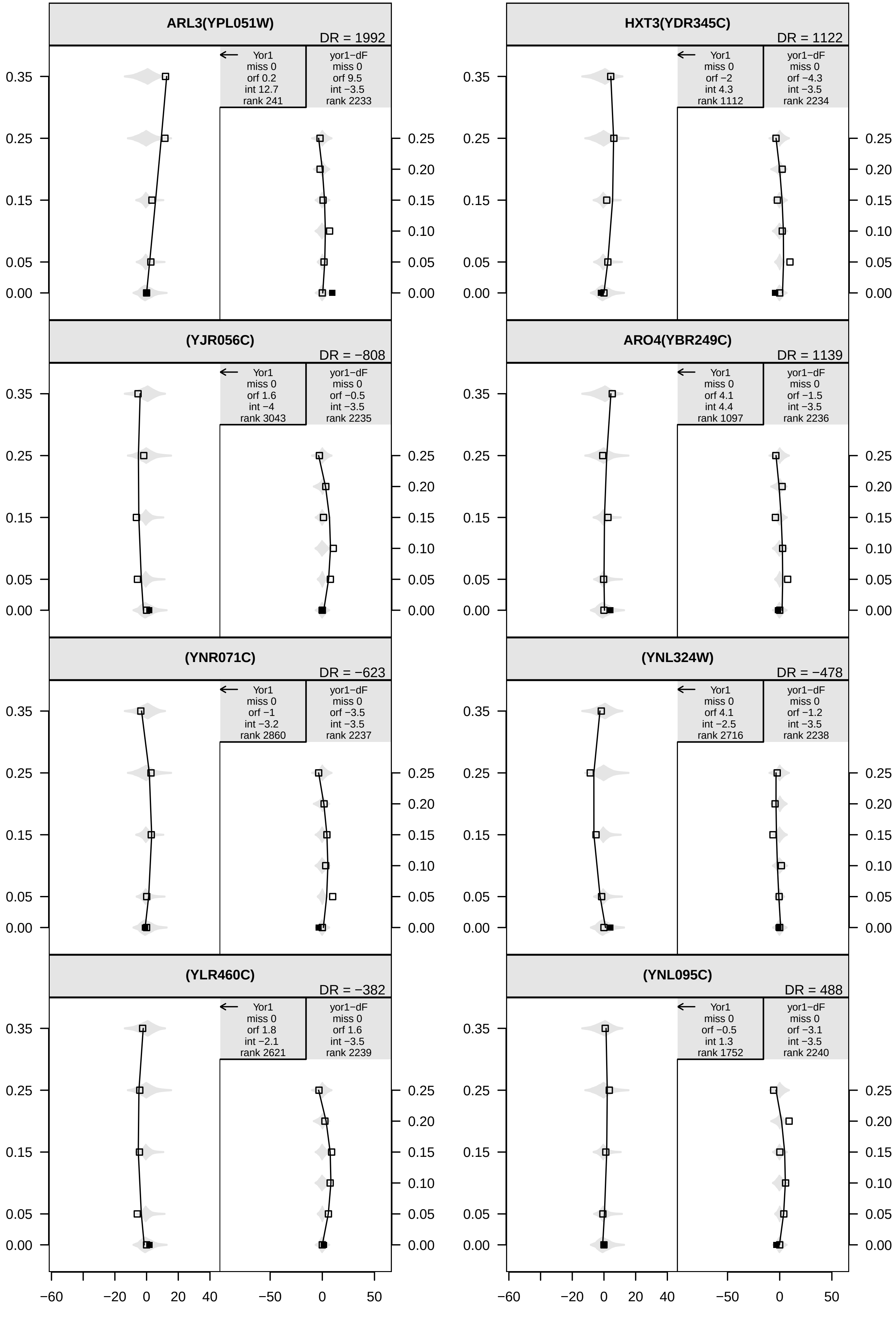
ARL3(YPL051W)
DR = 1992
Yor1
miss 0
orf 0.2
int 12.7
rank 241
yor1−dF
miss 0
orf 9.5
int −3.5
rank 2233
HXT3(YDR345C)
DR = 1122
Yor1
miss 0
orf −2
int 4.3
rank 1112
yor1−dF
miss 0
orf −4.3
int −3.5
rank 2234
(YJR056C)
DR = −808
Yor1
miss 0
orf 1.6
int −4
rank 3043
yor1−dF
miss 0
orf −0.5
int −3.5
rank 2235
ARO4(YBR249C)
DR = 1139
Yor1
miss 0
orf 4.1
int 4.4
rank 1097
yor1−dF
miss 0
orf −1.5
int −3.5
rank 2236
(YNR071C)
DR = −623
Yor1
miss 0
orf −1
int −3.2
rank 2860
yor1−dF
miss 0
orf −3.5
int −3.5
rank 2237
(YNL324W)
DR = −478
Yor1
miss 0
orf 4.1
int −2.5
rank 2716
yor1−dF
miss 0
orf −1.2
int −3.5
rank 2238
(YLR460C)
DR = −382
Yor1
miss 0
orf 1.8
int −2.1
rank 2621
yor1−dF
miss 0
orf 1.6
int −3.5
rank 2239
(YNL095C)
DR = 488
Yor1
miss 0
orf −0.5
int 1.3
rank 1752
yor1−dF
miss 0
orf −3.1
int −3.5
rank 2240
0.35
0.25
0.20
0.15
0.10
0.05
0.00
−60
−20
0
20
40
−50
0
50

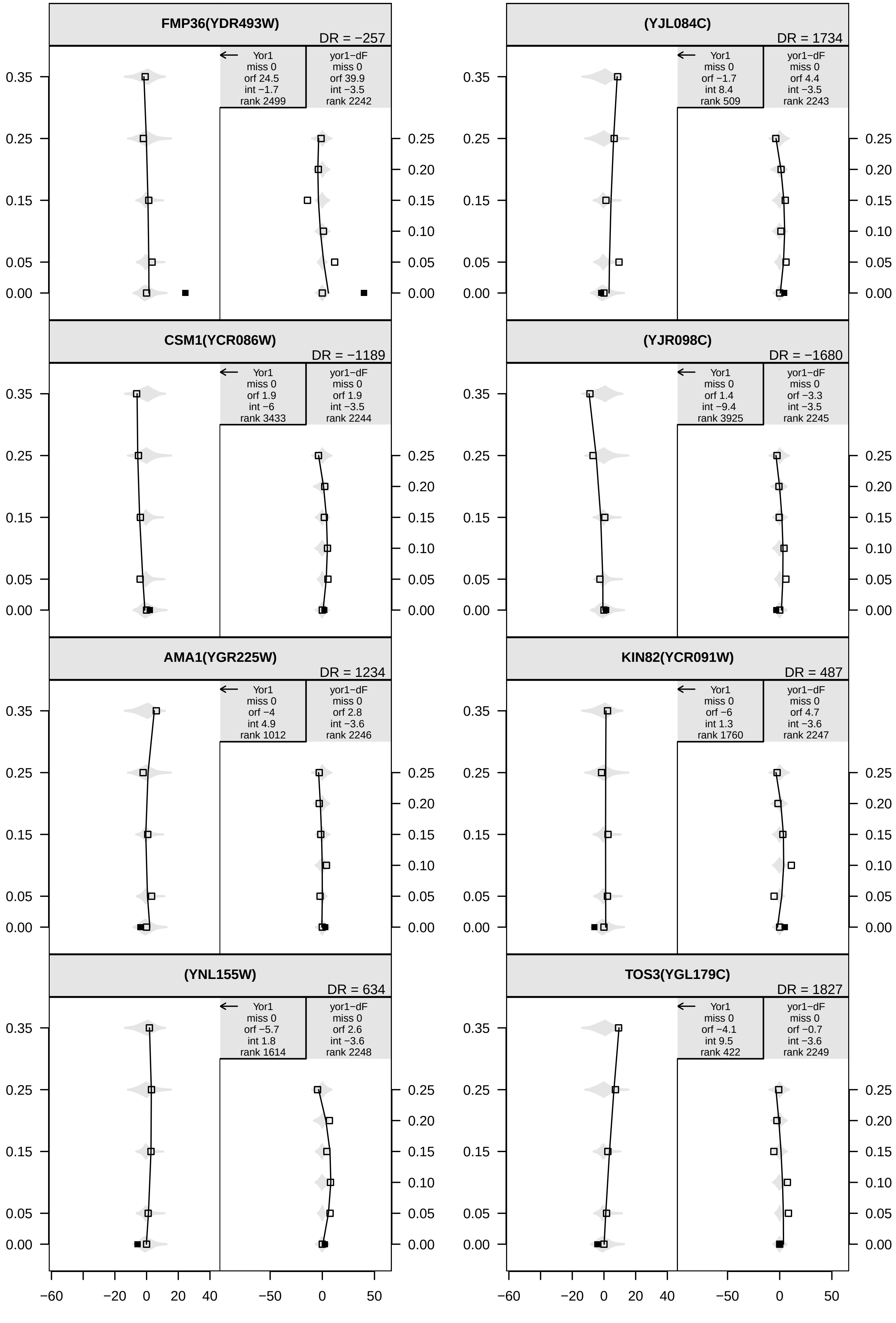

FMP36(YDR493W)
DR = −257
Yor1 miss 0 orf 24.5 int −1.7 rank 2499
yor1−dF miss 0 orf 39.9 int −3.5 rank 2242
(YJL084C)
DR = 1734
Yor1 miss 0 orf −1.7 int 8.4 rank 509
yor1−dF miss 0 orf 4.4 int −3.5 rank 2243
CSM1(YCR086W)
DR = −1189
Yor1 miss 0 orf 1.9 int −6 rank 3433
yor1−dF miss 0 orf 1.9 int −3.5 rank 2244
(YJR098C)
DR = −1680
Yor1 miss 0 orf 1.4 int −9.4 rank 3925
yor1−dF miss 0 orf −3.3 int −3.5 rank 2245
AMA1(YGR225W)
DR = 1234
Yor1 miss 0 orf −4 int 4.9 rank 1012
yor1−dF miss 0 orf 2.8 int −3.6 rank 2246
KIN82(YCR091W)
DR = 487
Yor1 miss 0 orf −6 int 1.3 rank 1760
yor1−dF miss 0 orf 4.7 int −3.6 rank 2247
(YNL155W)
DR = 634
Yor1 miss 0 orf −5.7 int 1.8 rank 1614
yor1−dF miss 0 orf 2.6 int −3.6 rank 2248
TOS3(YGL179C)
DR = 1827
Yor1 miss 0 orf −4.1 int 9.5 rank 422
yor1−dF miss 0 orf −0.7 int −3.6 rank 2249
0.00 0.05 0.10 0.15 0.20 0.25 0.35
−60 −20 0 20 40 −50 0 50

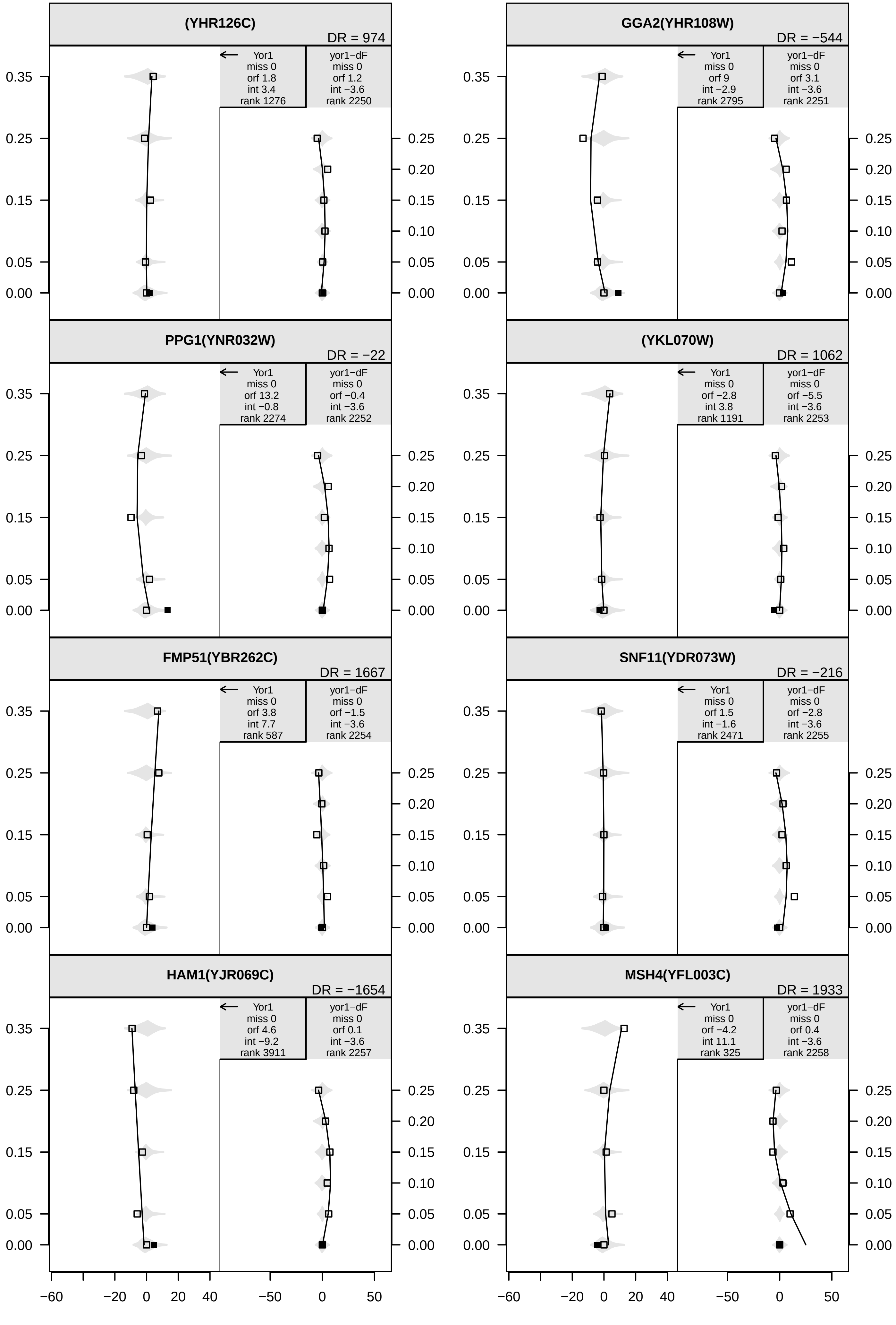

(YHR126C)
DR = 974
Yor1 miss 0 orf 1.8 int 3.4 rank 1276
yor1−dF miss 0 orf 1.2 int −3.6 rank 2250
GGA2(YHR108W)
DR = −544
Yor1 miss 0 orf 9 int −2.9 rank 2795
yor1−dF miss 0 orf 3.1 int −3.6 rank 2251
PPG1(YNR032W)
DR = −22
Yor1 miss 0 orf 13.2 int −0.8 rank 2274
yor1−dF miss 0 orf −0.4 int −3.6 rank 2252
(YKL070W)
DR = 1062
Yor1 miss 0 orf −2.8 int 3.8 rank 1191
yor1−dF miss 0 orf −5.5 int −3.6 rank 2253
FMP51(YBR262C)
DR = 1667
Yor1 miss 0 orf 3.8 int 7.7 rank 587
yor1−dF miss 0 orf −1.5 int −3.6 rank 2254
SNF11(YDR073W)
DR = −216
Yor1 miss 0 orf 1.5 int −1.6 rank 2471
yor1−dF miss 0 orf −2.8 int −3.6 rank 2255
HAM1(YJR069C)
DR = −1654
Yor1 miss 0 orf 4.6 int −9.2 rank 3911
yor1−dF miss 0 orf 0.1 int −3.6 rank 2257
MSH4(YFL003C)
DR = 1933
Yor1 miss 0 orf −4.2 int 11.1 rank 325
yor1−dF miss 0 orf 0.4 int −3.6 rank 2258
0.35
0.25
0.20
0.15
0.10
0.05
0.00
−60
−20
0
20
40
−50
0
50

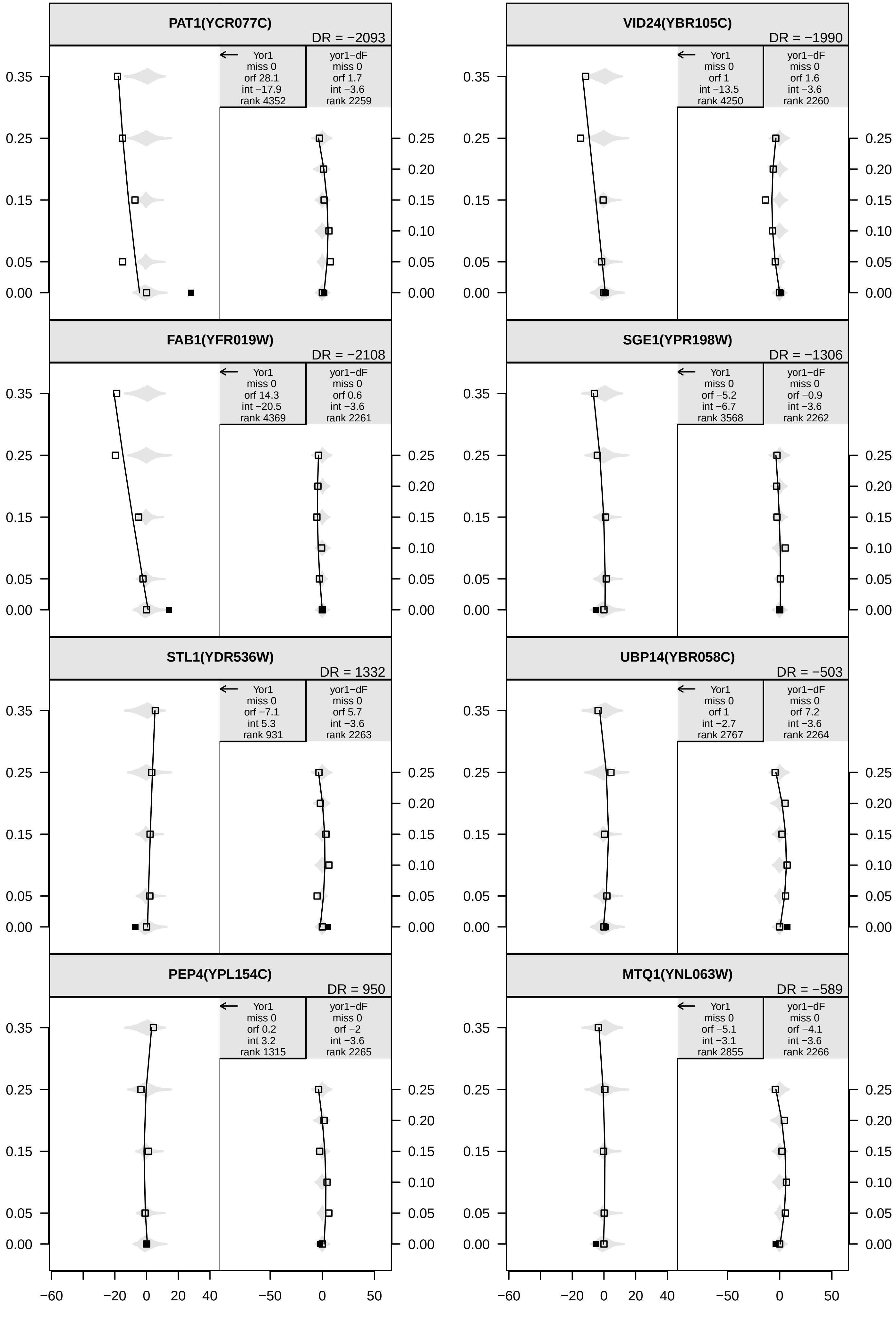
PAT1(YCR077C)
DR = −2093
Yor1 miss 0 orf 28.1 int −17.9 rank 4352
yor1−dF miss 0 orf 1.7 int −3.6 rank 2259
VID24(YBR105C)
DR = −1990
Yor1 miss 0 orf 1 int −13.5 rank 4250
yor1−dF miss 0 orf 1.6 int −3.6 rank 2260
FAB1(YFR019W)
DR = −2108
Yor1 miss 0 orf 14.3 int −20.5 rank 4369
yor1−dF miss 0 orf 0.6 int −3.6 rank 2261
SGE1(YPR198W)
DR = −1306
Yor1 miss 0 orf −5.2 int −6.7 rank 3568
yor1−dF miss 0 orf −0.9 int −3.6 rank 2262
STL1(YDR536W)
DR = 1332
Yor1 miss 0 orf −7.1 int 5.3 rank 931
yor1−dF miss 0 orf 5.7 int −3.6 rank 2263
UBP14(YBR058C)
DR = −503
Yor1 miss 0 orf 1 int −2.7 rank 2767
yor1−dF miss 0 orf 7.2 int −3.6 rank 2264
PEP4(YPL154C)
DR = 950
Yor1 miss 0 orf 0.2 int 3.2 rank 1315
yor1−dF miss 0 orf −2 int −3.6 rank 2265
MTQ1(YNL063W)
DR = −589
Yor1 miss 0 orf −5.1 int −3.1 rank 2855
yor1−dF miss 0 orf −4.1 int −3.6 rank 2266
0.35 0.25 0.15 0.05 0.00
0.25 0.20 0.15 0.10 0.05 0.00
−60 −20 0 20 40 −50 0 50

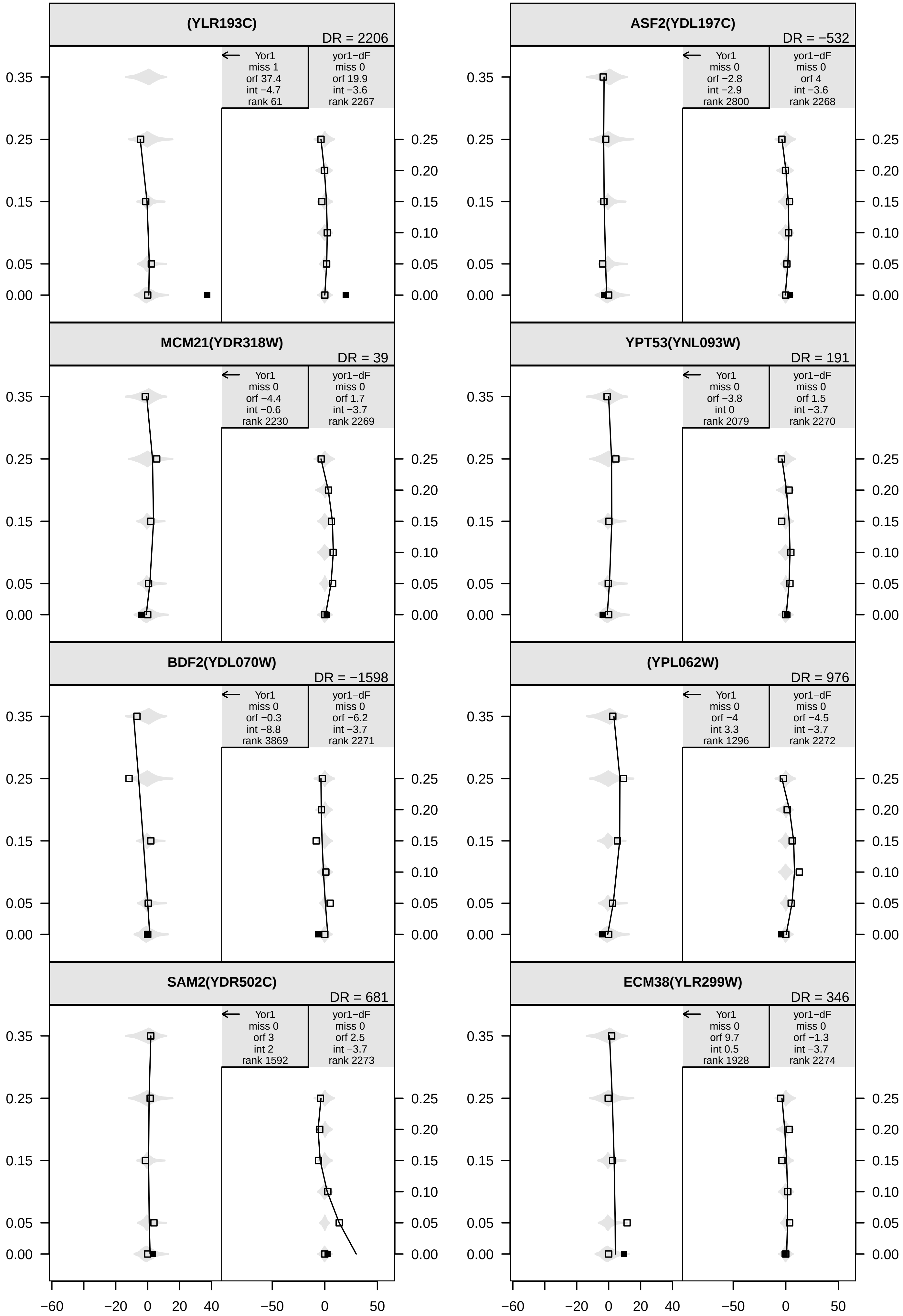
(YLR193C)
DR = 2206
Yor1
miss 1
orf 37.4
int −4.7
rank 61
yor1−dF
miss 0
orf 19.9
int −3.6
rank 2267
ASF2(YDL197C)
DR = −532
Yor1
miss 0
orf −2.8
int −2.9
rank 2800
yor1−dF
miss 0
orf 4
int −3.6
rank 2268
MCM21(YDR318W)
DR = 39
Yor1
miss 0
orf −4.4
int −0.6
rank 2230
yor1−dF
miss 0
orf 1.7
int −3.7
rank 2269
YPT53(YNL093W)
DR = 191
Yor1
miss 0
orf −3.8
int 0
rank 2079
yor1−dF
miss 0
orf 1.5
int −3.7
rank 2270
BDF2(YDL070W)
DR = −1598
Yor1
miss 0
orf −0.3
int −8.8
rank 3869
yor1−dF
miss 0
orf −6.2
int −3.7
rank 2271
(YPL062W)
DR = 976
Yor1
miss 0
orf −4
int 3.3
rank 1296
yor1−dF
miss 0
orf −4.5
int −3.7
rank 2272
SAM2(YDR502C)
DR = 681
Yor1
miss 0
orf 3
int 2
rank 1592
yor1−dF
miss 0
orf 2.5
int −3.7
rank 2273
ECM38(YLR299W)
DR = 346
Yor1
miss 0
orf 9.7
int 0.5
rank 1928
yor1−dF
miss 0
orf −1.3
int −3.7
rank 2274
0.35 0.25 0.20 0.15 0.10 0.05 0.00
−60 −20 0 20 40 −50 0 50

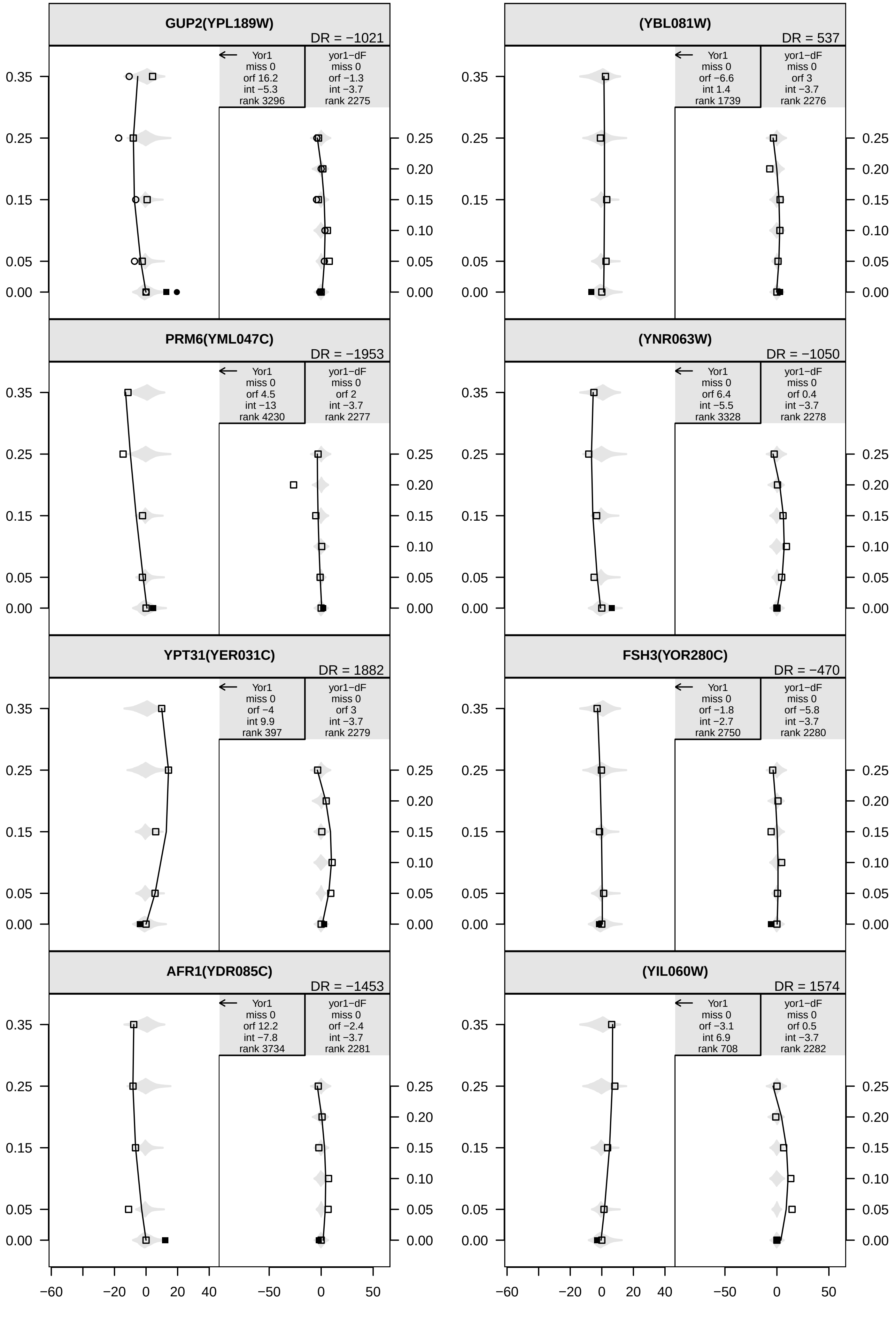
GUP2(YPL189W)
DR = −1021
Yor1 miss 0 orf 16.2 int −5.3 rank 3296
yor1−dF miss 0 orf −1.3 int −3.7 rank 2275
(YBL081W)
DR = 537
Yor1 miss 0 orf −6.6 int 1.4 rank 1739
yor1−dF miss 0 orf 3 int −3.7 rank 2276
PRM6(YML047C)
DR = −1953
Yor1 miss 0 orf 4.5 int −13 rank 4230
yor1−dF miss 0 orf 2 int −3.7 rank 2277
(YNR063W)
DR = −1050
Yor1 miss 0 orf 6.4 int −5.5 rank 3328
yor1−dF miss 0 orf 0.4 int −3.7 rank 2278
YPT31(YER031C)
DR = 1882
Yor1 miss 0 orf −4 int 9.9 rank 397
yor1−dF miss 0 orf 3 int −3.7 rank 2279
FSH3(YOR280C)
DR = −470
Yor1 miss 0 orf −1.8 int −2.7 rank 2750
yor1−dF miss 0 orf −5.8 int −3.7 rank 2280
AFR1(YDR085C)
DR = −1453
Yor1 miss 0 orf 12.2 int −7.8 rank 3734
yor1−dF miss 0 orf −2.4 int −3.7 rank 2281
(YIL060W)
DR = 1574
Yor1 miss 0 orf −3.1 int 6.9 rank 708
yor1−dF miss 0 orf 0.5 int −3.7 rank 2282
0.35
0.25
0.20
0.15
0.10
0.05
0.00
−60
−20
0
20
40
−50
0
50

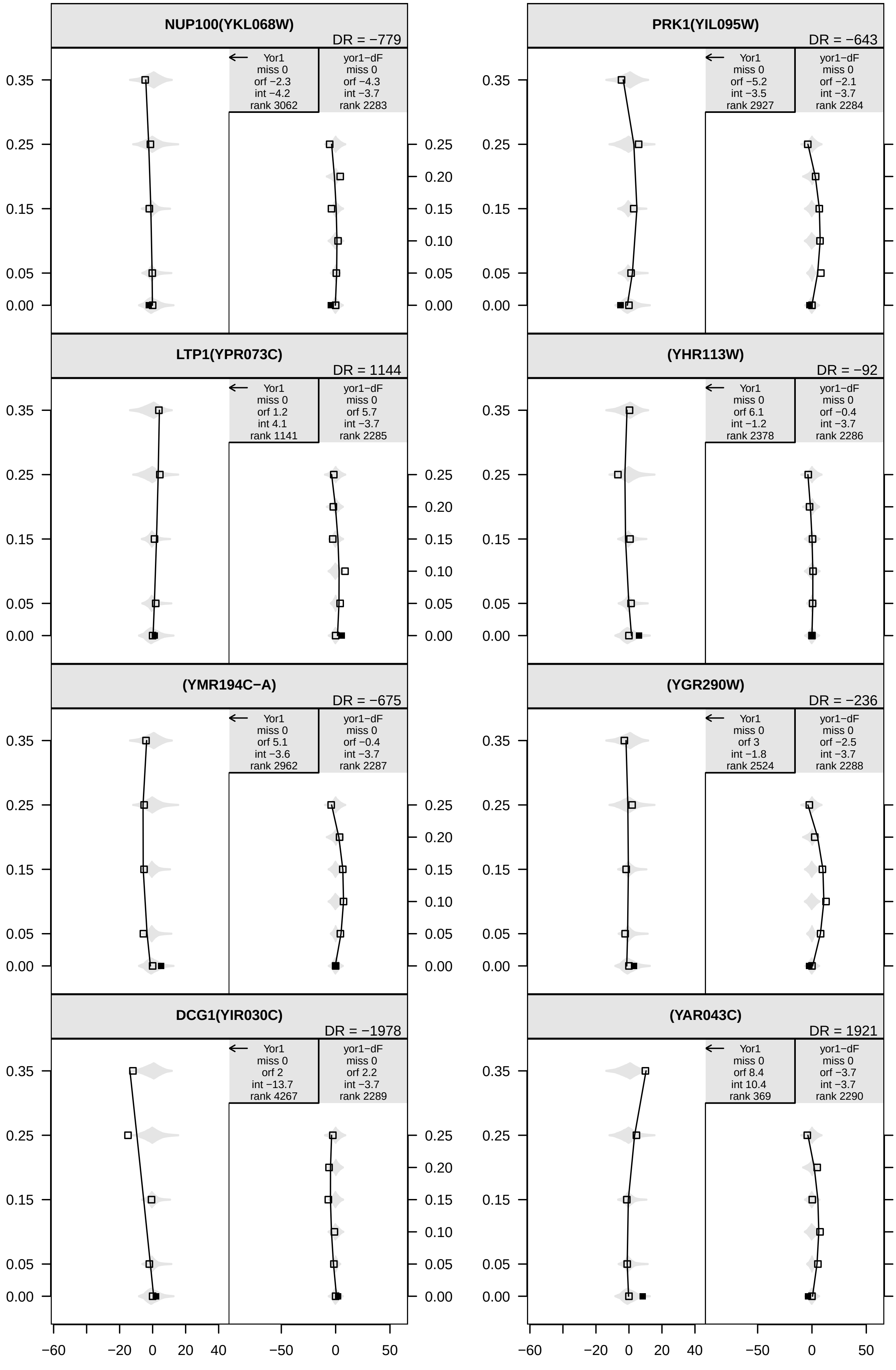
NUP100(YKL068W)
DR = −779
Yor1 miss 0 orf −2.3 int −4.2 rank 3062
yor1−dF miss 0 orf −4.3 int −3.7 rank 2283
PRK1(YIL095W)
DR = −643
Yor1 miss 0 orf −5.2 int −3.5 rank 2927
yor1−dF miss 0 orf −2.1 int −3.7 rank 2284
LTP1(YPR073C)
DR = 1144
Yor1 miss 0 orf 1.2 int 4.1 rank 1141
yor1−dF miss 0 orf 5.7 int −3.7 rank 2285
(YHR113W)
DR = −92
Yor1 miss 0 orf 6.1 int −1.2 rank 2378
yor1−dF miss 0 orf −0.4 int −3.7 rank 2286
(YMR194C−A)
DR = −675
Yor1 miss 0 orf 5.1 int −3.6 rank 2962
yor1−dF miss 0 orf −0.4 int −3.7 rank 2287
(YGR290W)
DR = −236
Yor1 miss 0 orf 3 int −1.8 rank 2524
yor1−dF miss 0 orf −2.5 int −3.7 rank 2288
DCG1(YIR030C)
DR = −1978
Yor1 miss 0 orf 2 int −13.7 rank 4267
yor1−dF miss 0 orf 2.2 int −3.7 rank 2289
(YAR043C)
DR = 1921
Yor1 miss 0 orf 8.4 int 10.4 rank 369
yor1−dF miss 0 orf −3.7 int −3.7 rank 2290

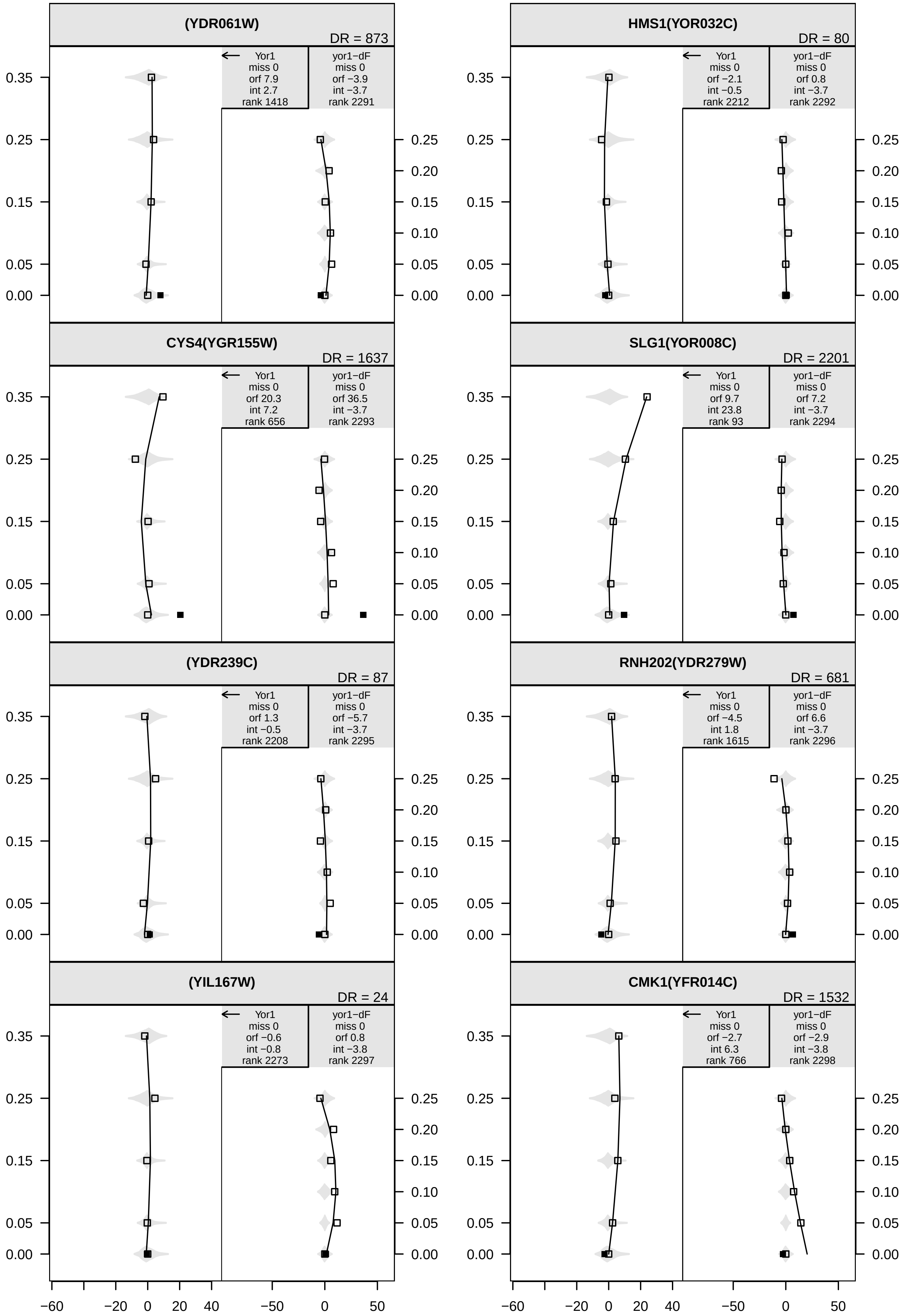

(YDR061W)
DR = 873
Yor1
miss 0
orf 7.9
int 2.7
rank 1418
yor1−dF
miss 0
orf −3.9
int −3.7
rank 2291
HMS1(YOR032C)
DR = 80
Yor1
miss 0
orf −2.1
int −0.5
rank 2212
yor1−dF
miss 0
orf 0.8
int −3.7
rank 2292
CYS4(YGR155W)
DR = 1637
Yor1
miss 0
orf 20.3
int 7.2
rank 656
yor1−dF
miss 0
orf 36.5
int −3.7
rank 2293
SLG1(YOR008C)
DR = 2201
Yor1
miss 0
orf 9.7
int 23.8
rank 93
yor1−dF
miss 0
orf 7.2
int −3.7
rank 2294
(YDR239C)
DR = 87
Yor1
miss 0
orf 1.3
int −0.5
rank 2208
yor1−dF
miss 0
orf −5.7
int −3.7
rank 2295
RNH202(YDR279W)
DR = 681
Yor1
miss 0
orf −4.5
int 1.8
rank 1615
yor1−dF
miss 0
orf 6.6
int −3.7
rank 2296
(YIL167W)
DR = 24
Yor1
miss 0
orf −0.6
int −0.8
rank 2273
yor1−dF
miss 0
orf 0.8
int −3.8
rank 2297
CMK1(YFR014C)
DR = 1532
Yor1
miss 0
orf −2.7
int 6.3
rank 766
yor1−dF
miss 0
orf −2.9
int −3.8
rank 2298
0.35
0.25
0.20
0.15
0.10
0.05
0.00
−60
−20
0
20
40
−50
0
50

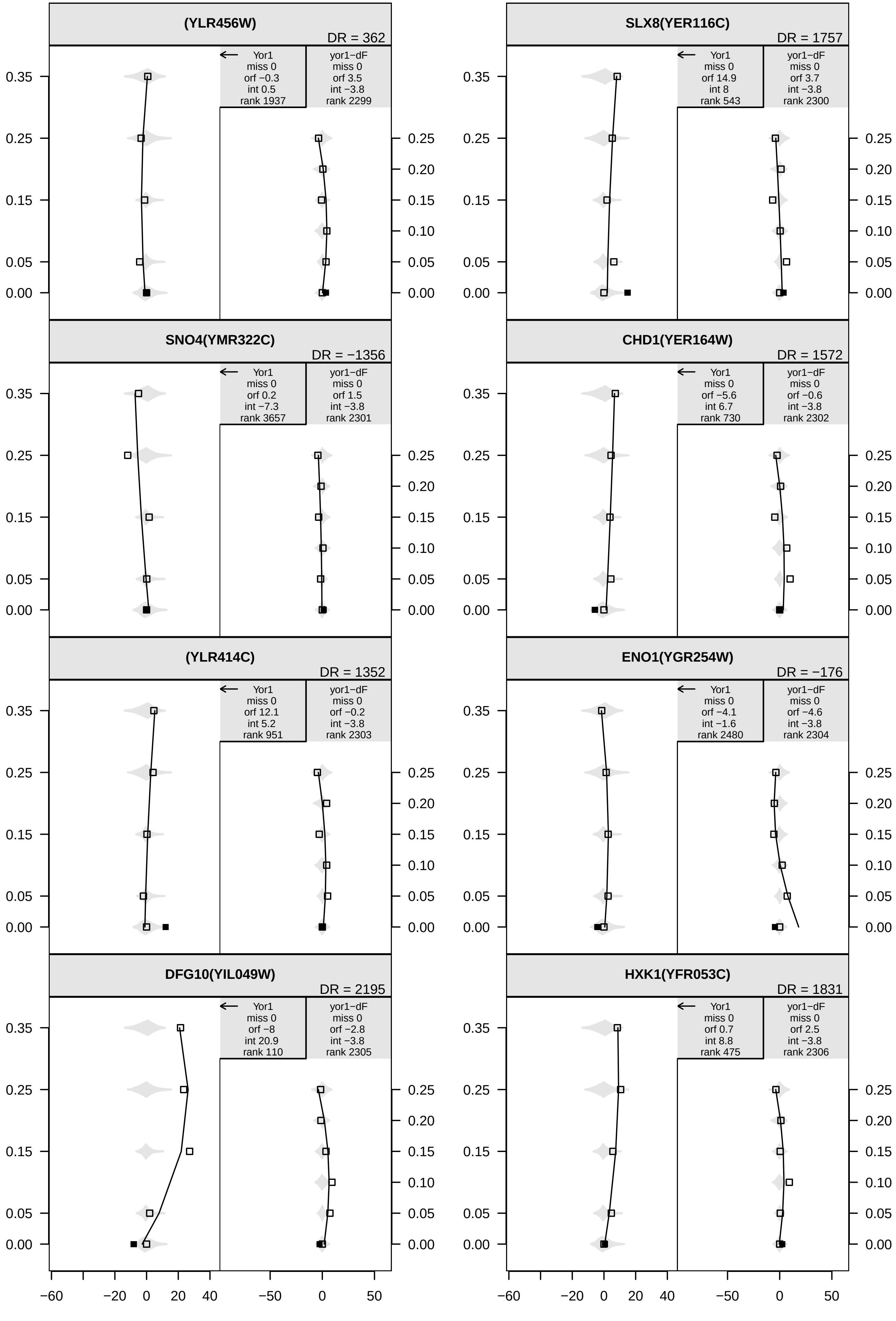
(YLR456W)
DR = 362
Yor1 miss 0 orf −0.3 int 0.5 rank 1937
yor1−dF miss 0 orf 3.5 int −3.8 rank 2299
SLX8(YER116C)
DR = 1757
Yor1 miss 0 orf 14.9 int 8 rank 543
yor1−dF miss 0 orf 3.7 int −3.8 rank 2300
SNO4(YMR322C)
DR = −1356
Yor1 miss 0 orf 0.2 int −7.3 rank 3657
yor1−dF miss 0 orf 1.5 int −3.8 rank 2301
CHD1(YER164W)
DR = 1572
Yor1 miss 0 orf −5.6 int 6.7 rank 730
yor1−dF miss 0 orf −0.6 int −3.8 rank 2302
(YLR414C)
DR = 1352
Yor1 miss 0 orf 12.1 int 5.2 rank 951
yor1−dF miss 0 orf −0.2 int −3.8 rank 2303
ENO1(YGR254W)
DR = −176
Yor1 miss 0 orf −4.1 int −1.6 rank 2480
yor1−dF miss 0 orf −4.6 int −3.8 rank 2304
DFG10(YIL049W)
DR = 2195
Yor1 miss 0 orf −8 int 20.9 rank 110
yor1−dF miss 0 orf −2.8 int −3.8 rank 2305
HXK1(YFR053C)
DR = 1831
Yor1 miss 0 orf 0.7 int 8.8 rank 475
yor1−dF miss 0 orf 2.5 int −3.8 rank 2306
0.35 0.25 0.15 0.05 0.00
0.25 0.20 0.15 0.10 0.05 0.00
−60 −20 0 20 40
−50 0 50

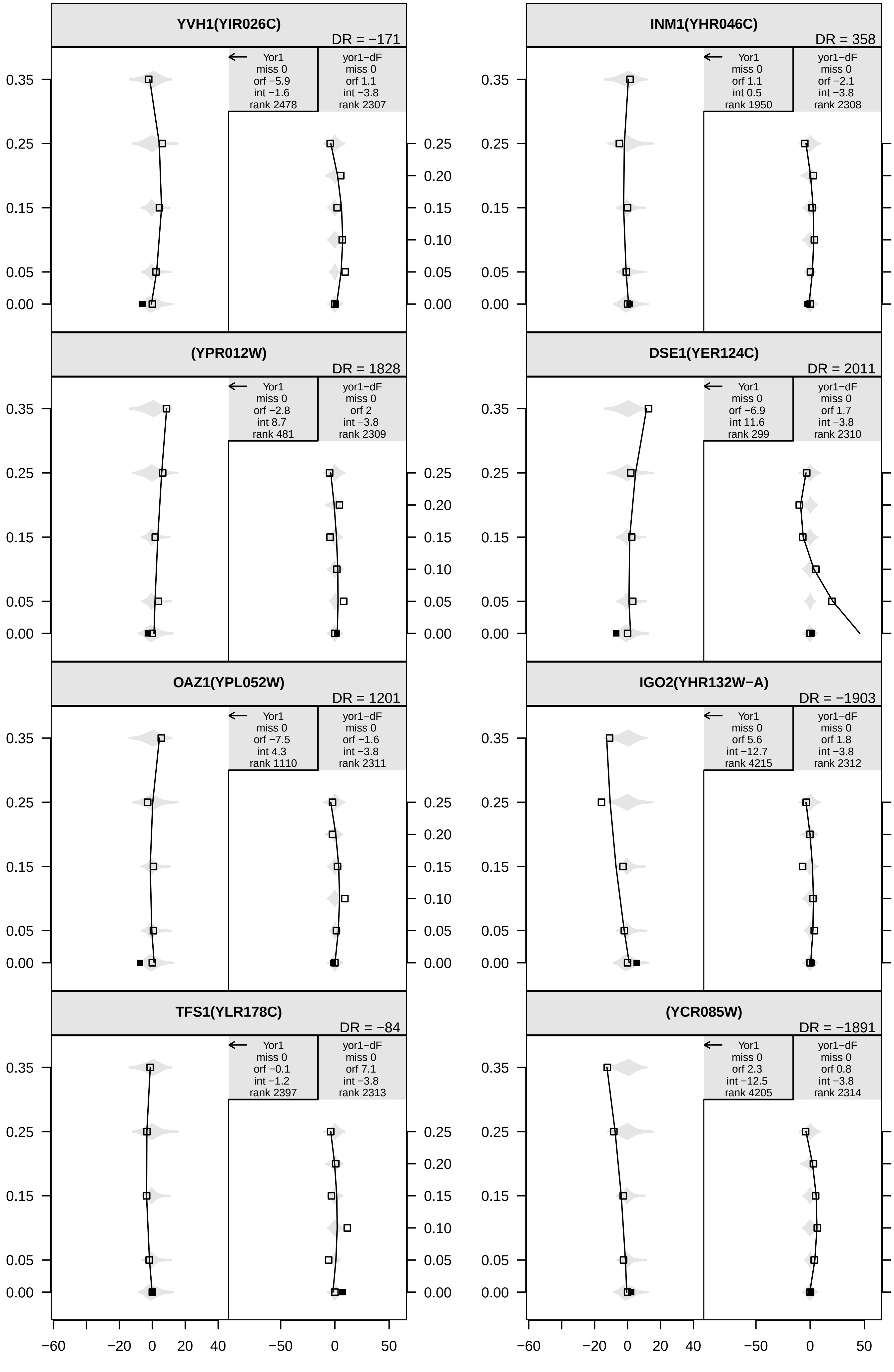
YVH1(YIR026C)
DR = −171
Yor1 miss 0 orf −5.9 int −1.6 rank 2478
yor1−dF miss 0 orf 1.1 int −3.8 rank 2307
INM1(YHR046C)
DR = 358
Yor1 miss 0 orf 1.1 int 0.5 rank 1950
yor1−dF miss 0 orf −2.1 int −3.8 rank 2308
(YPR012W)
DR = 1828
Yor1 miss 0 orf −2.8 int 8.7 rank 481
yor1−dF miss 0 orf 2 int −3.8 rank 2309
DSE1(YER124C)
DR = 2011
Yor1 miss 0 orf −6.9 int 11.6 rank 299
yor1−dF miss 0 orf 1.7 int −3.8 rank 2310
OAZ1(YPL052W)
DR = 1201
Yor1 miss 0 orf −7.5 int 4.3 rank 1110
yor1−dF miss 0 orf −1.6 int −3.8 rank 2311
IGO2(YHR132W−A)
DR = −1903
Yor1 miss 0 orf 5.6 int −12.7 rank 4215
yor1−dF miss 0 orf 1.8 int −3.8 rank 2312
TFS1(YLR178C)
DR = −84
Yor1 miss 0 orf −0.1 int −1.2 rank 2397
yor1−dF miss 0 orf 7.1 int −3.8 rank 2313
(YCR085W)
DR = −1891
Yor1 miss 0 orf 2.3 int −12.5 rank 4205
yor1−dF miss 0 orf 0.8 int −3.8 rank 2314
0.35
0.25
0.20
0.15
0.10
0.05
0.00
−60
−20
0
20
40
−50
0
50

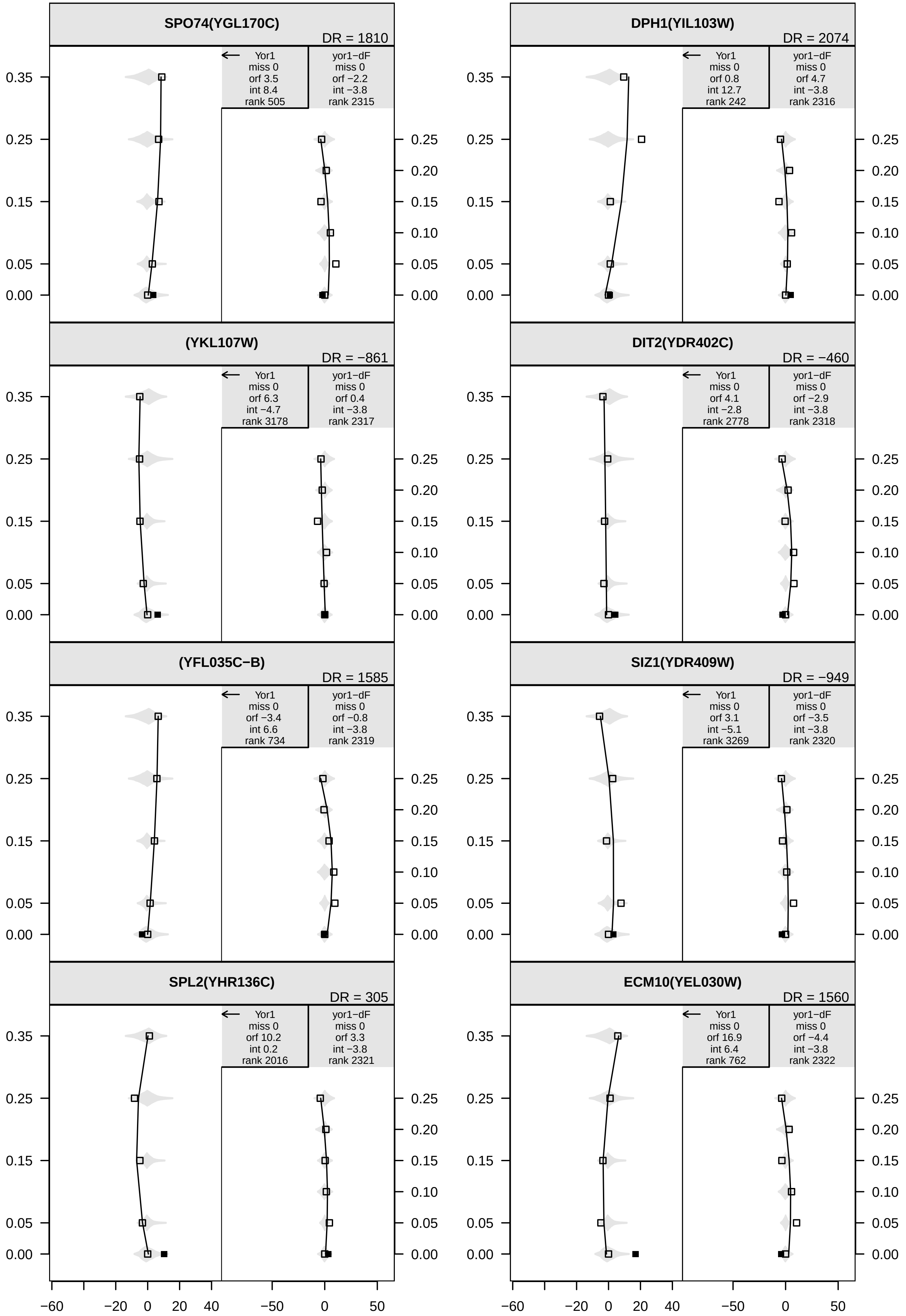
SPO74(YGL170C)
DR = 1810
Yor1
miss 0
orf 3.5
int 8.4
rank 505
yor1−dF
miss 0
orf −2.2
int −3.8
rank 2315
DPH1(YIL103W)
DR = 2074
Yor1
miss 0
orf 0.8
int 12.7
rank 242
yor1−dF
miss 0
orf 4.7
int −3.8
rank 2316
(YKL107W)
DR = −861
Yor1
miss 0
orf 6.3
int −4.7
rank 3178
yor1−dF
miss 0
orf 0.4
int −3.8
rank 2317
DIT2(YDR402C)
DR = −460
Yor1
miss 0
orf 4.1
int −2.8
rank 2778
yor1−dF
miss 0
orf −2.9
int −3.8
rank 2318
(YFL035C−B)
DR = 1585
Yor1
miss 0
orf −3.4
int 6.6
rank 734
yor1−dF
miss 0
orf −0.8
int −3.8
rank 2319
SIZ1(YDR409W)
DR = −949
Yor1
miss 0
orf 3.1
int −5.1
rank 3269
yor1−dF
miss 0
orf −3.5
int −3.8
rank 2320
SPL2(YHR136C)
DR = 305
Yor1
miss 0
orf 10.2
int 0.2
rank 2016
yor1−dF
miss 0
orf 3.3
int −3.8
rank 2321
ECM10(YEL030W)
DR = 1560
Yor1
miss 0
orf 16.9
int 6.4
rank 762
yor1−dF
miss 0
orf −4.4
int −3.8
rank 2322
0.35
0.25
0.20
0.15
0.10
0.05
0.00
−60
−20
0
20
40
−50
0
50

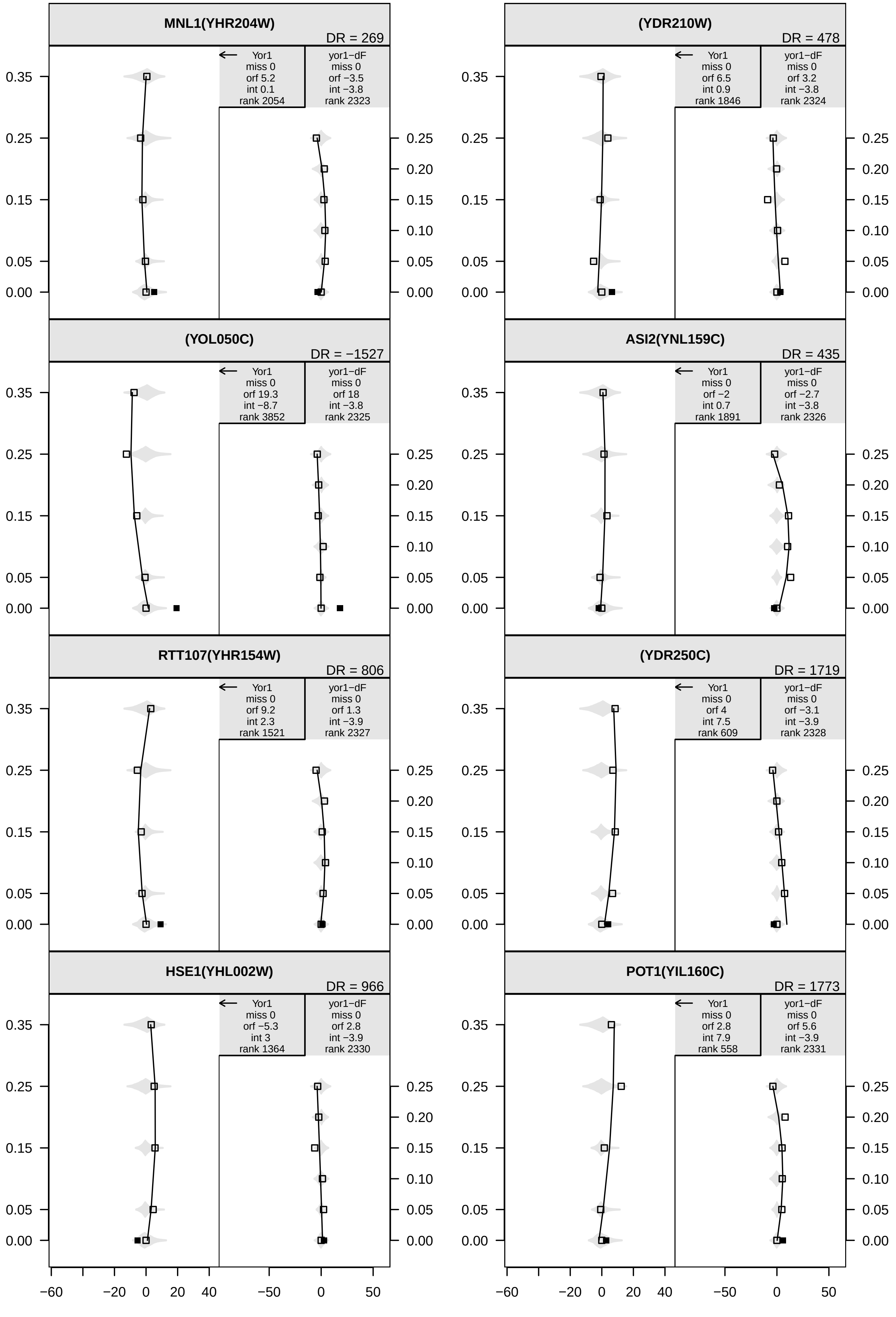

MNL1(YHR204W)
DR = 269
Yor1 miss 0 orf 5.2 int 0.1 rank 2054
yor1−dF miss 0 orf −3.5 int −3.8 rank 2323
(YDR210W)
DR = 478
Yor1 miss 0 orf 6.5 int 0.9 rank 1846
yor1−dF miss 0 orf 3.2 int −3.8 rank 2324
(YOL050C)
DR = −1527
Yor1 miss 0 orf 19.3 int −8.7 rank 3852
yor1−dF miss 0 orf 18 int −3.8 rank 2325
ASI2(YNL159C)
DR = 435
Yor1 miss 0 orf −2 int 0.7 rank 1891
yor1−dF miss 0 orf −2.7 int −3.8 rank 2326
RTT107(YHR154W)
DR = 806
Yor1 miss 0 orf 9.2 int 2.3 rank 1521
yor1−dF miss 0 orf 1.3 int −3.9 rank 2327
(YDR250C)
DR = 1719
Yor1 miss 0 orf 4 int 7.5 rank 609
yor1−dF miss 0 orf −3.1 int −3.9 rank 2328
HSE1(YHL002W)
DR = 966
Yor1 miss 0 orf −5.3 int 3 rank 1364
yor1−dF miss 0 orf 2.8 int −3.9 rank 2330
POT1(YIL160C)
DR = 1773
Yor1 miss 0 orf 2.8 int 7.9 rank 558
yor1−dF miss 0 orf 5.6 int −3.9 rank 2331
0.35 0.25 0.15 0.05 0.00
0.25 0.20 0.15 0.10 0.05 0.00
−60 −20 0 20 40
−50 0 50

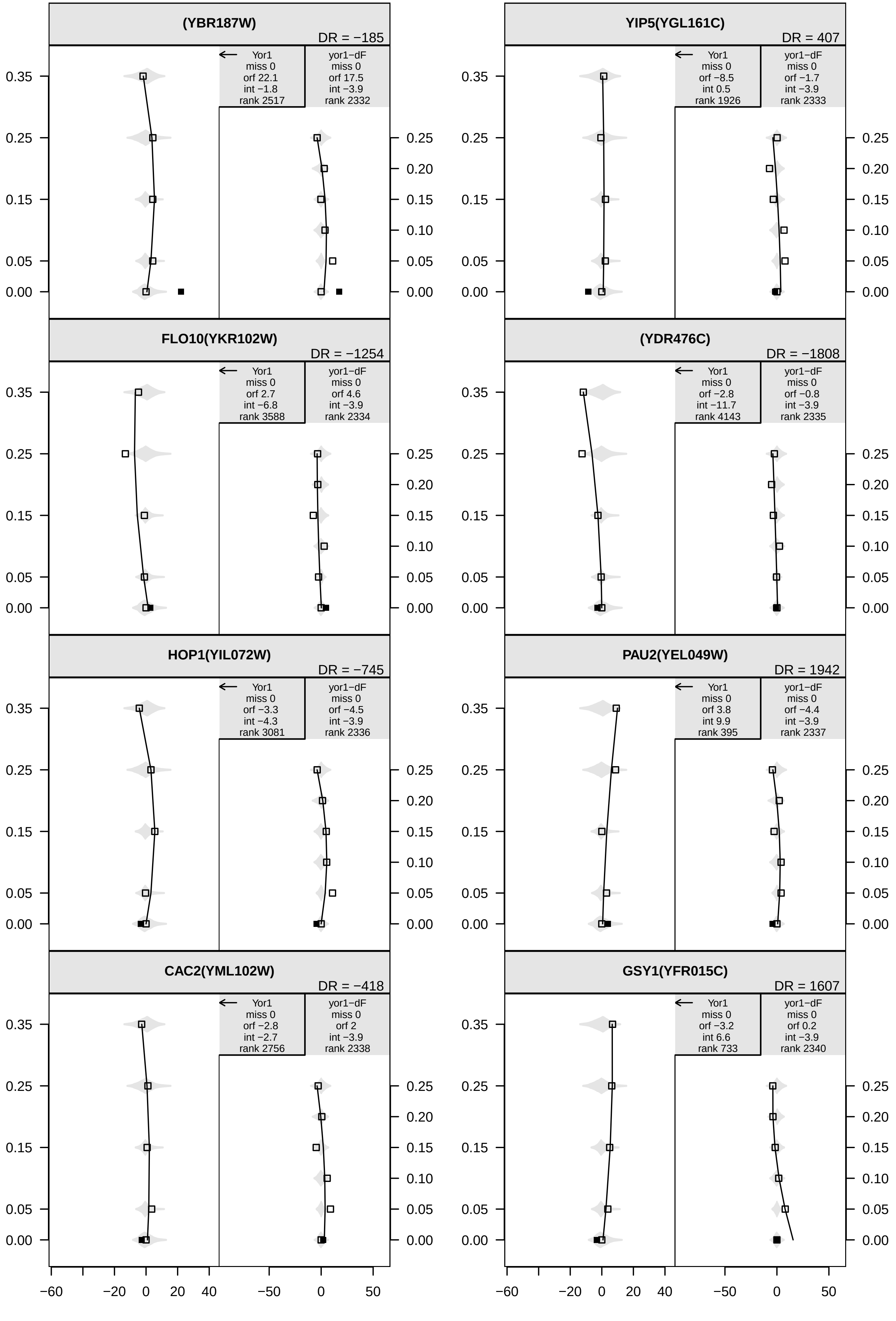

(YBR187W)
DR = −185
Yor1
miss 0
orf 22.1
int −1.8
rank 2517
yor1−dF
miss 0
orf 17.5
int −3.9
rank 2332
YIP5(YGL161C)
DR = 407
Yor1
miss 0
orf −8.5
int 0.5
rank 1926
yor1−dF
miss 0
orf −1.7
int −3.9
rank 2333
FLO10(YKR102W)
DR = −1254
Yor1
miss 0
orf 2.7
int −6.8
rank 3588
yor1−dF
miss 0
orf 4.6
int −3.9
rank 2334
(YDR476C)
DR = −1808
Yor1
miss 0
orf −2.8
int −11.7
rank 4143
yor1−dF
miss 0
orf −0.8
int −3.9
rank 2335
HOP1(YIL072W)
DR = −745
Yor1
miss 0
orf −3.3
int −4.3
rank 3081
yor1−dF
miss 0
orf −4.5
int −3.9
rank 2336
PAU2(YEL049W)
DR = 1942
Yor1
miss 0
orf 3.8
int 9.9
rank 395
yor1−dF
miss 0
orf −4.4
int −3.9
rank 2337
CAC2(YML102W)
DR = −418
Yor1
miss 0
orf −2.8
int −2.7
rank 2756
yor1−dF
miss 0
orf 2
int −3.9
rank 2338
GSY1(YFR015C)
DR = 1607
Yor1
miss 0
orf −3.2
int 6.6
rank 733
yor1−dF
miss 0
orf 0.2
int −3.9
rank 2340
0.35
0.25
0.20
0.15
0.10
0.05
0.00
−60
−20
0
20
40
−50
0
50

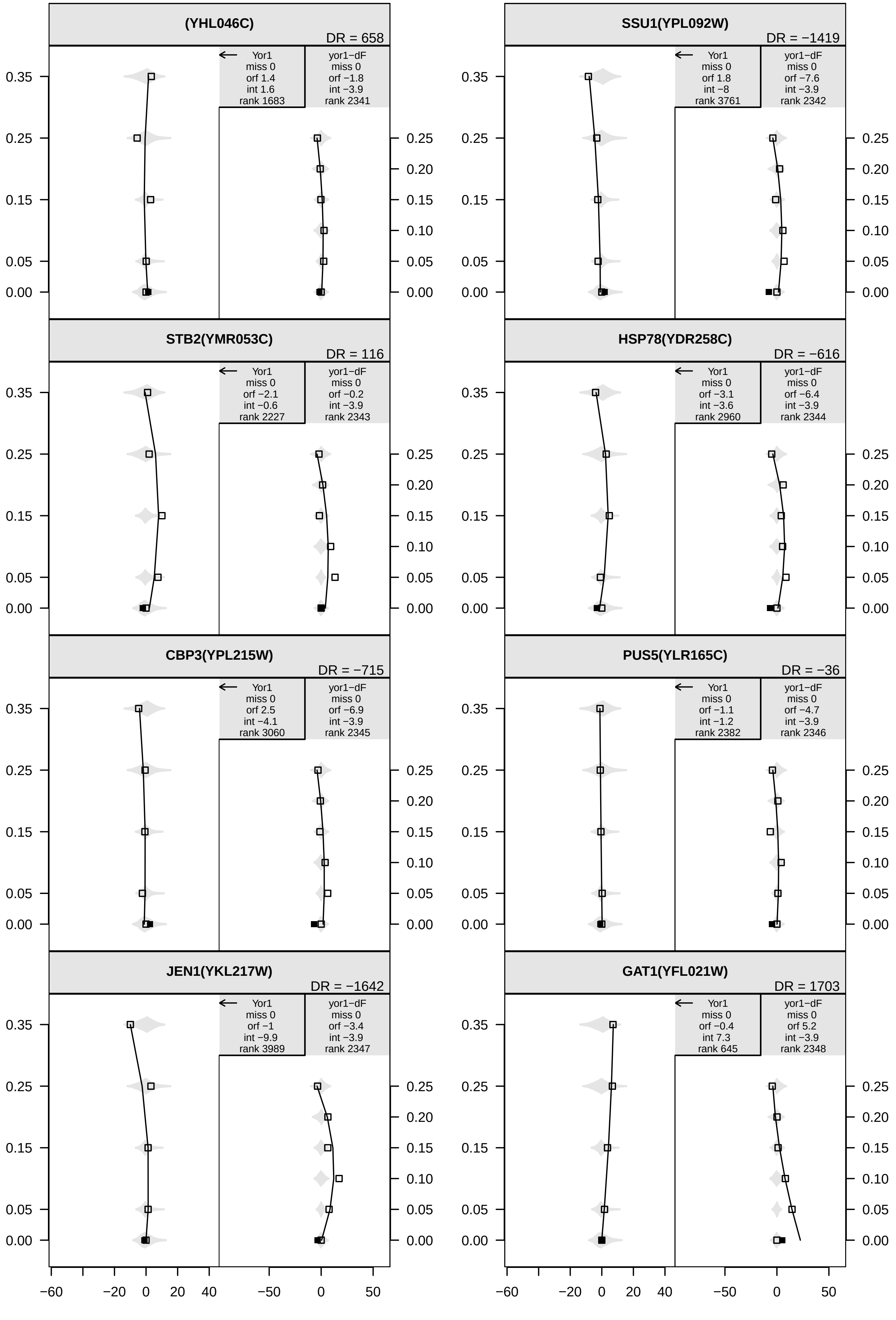

(YHL046C)
DR = 658
Yor1
miss 0
orf 1.4
int 1.6
rank 1683
yor1−dF
miss 0
orf −1.8
int −3.9
rank 2341
SSU1(YPL092W)
DR = −1419
Yor1
miss 0
orf 1.8
int −8
rank 3761
yor1−dF
miss 0
orf −7.6
int −3.9
rank 2342
STB2(YMR053C)
DR = 116
Yor1
miss 0
orf −2.1
int −0.6
rank 2227
yor1−dF
miss 0
orf −0.2
int −3.9
rank 2343
HSP78(YDR258C)
DR = −616
Yor1
miss 0
orf −3.1
int −3.6
rank 2960
yor1−dF
miss 0
orf −6.4
int −3.9
rank 2344
CBP3(YPL215W)
DR = −715
Yor1
miss 0
orf 2.5
int −4.1
rank 3060
yor1−dF
miss 0
orf −6.9
int −3.9
rank 2345
PUS5(YLR165C)
DR = −36
Yor1
miss 0
orf −1.1
int −1.2
rank 2382
yor1−dF
miss 0
orf −4.7
int −3.9
rank 2346
JEN1(YKL217W)
DR = −1642
Yor1
miss 0
orf −1
int −9.9
rank 3989
yor1−dF
miss 0
orf −3.4
int −3.9
rank 2347
GAT1(YFL021W)
DR = 1703
Yor1
miss 0
orf −0.4
int 7.3
rank 645
yor1−dF
miss 0
orf 5.2
int −3.9
rank 2348
0.35
0.25
0.20
0.15
0.10
0.05
0.00
−60
−20
0
20
40
−50
0
50

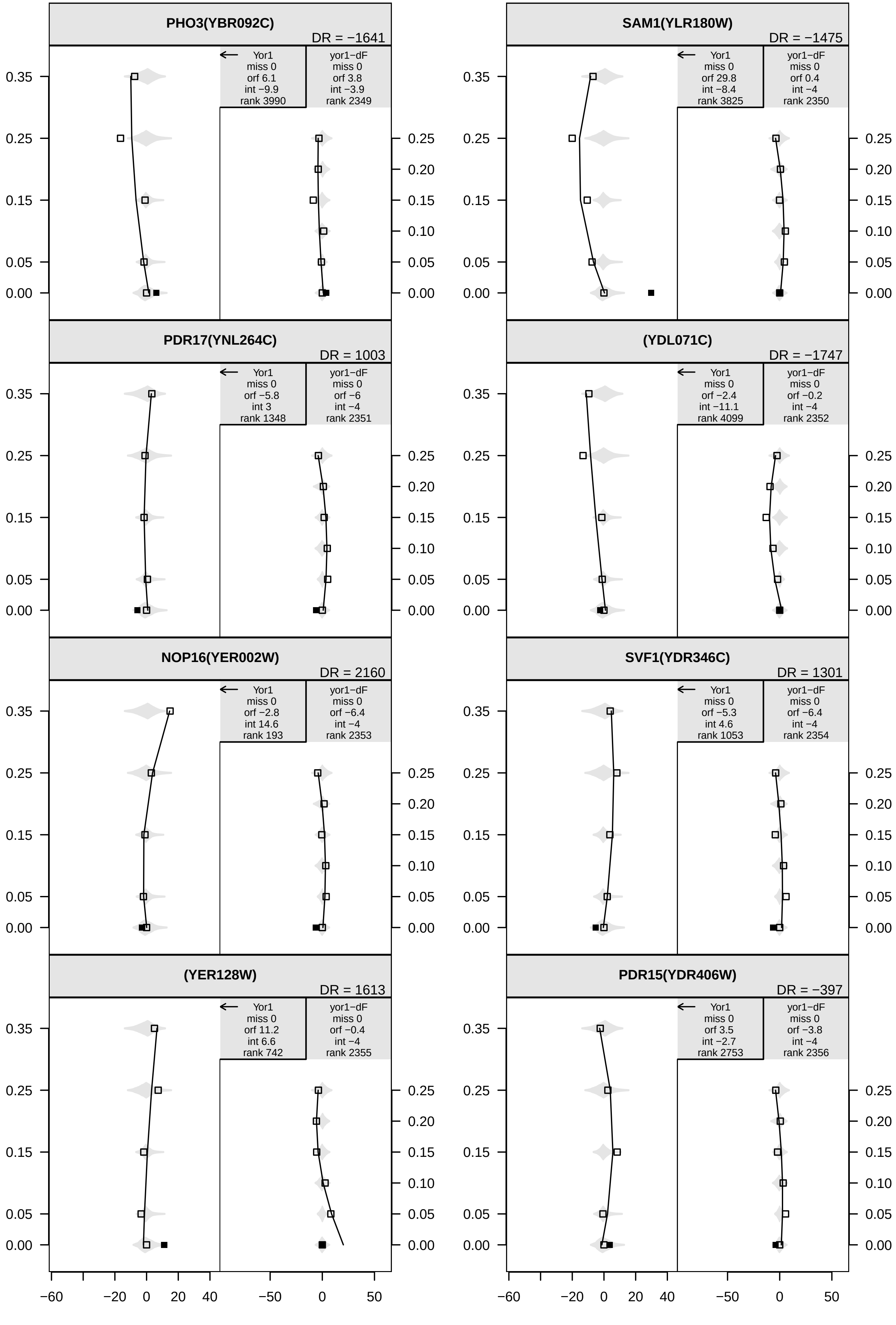

PHO3(YBR092C)
DR = −1641
Yor1 miss 0 orf 6.1 int −9.9 rank 3990
yor1−dF miss 0 orf 3.8 int −3.9 rank 2349
SAM1(YLR180W)
DR = −1475
Yor1 miss 0 orf 29.8 int −8.4 rank 3825
yor1−dF miss 0 orf 0.4 int −4 rank 2350
PDR17(YNL264C)
DR = 1003
Yor1 miss 0 orf −5.8 int 3 rank 1348
yor1−dF miss 0 orf −6 int −4 rank 2351
(YDL071C)
DR = −1747
Yor1 miss 0 orf −2.4 int −11.1 rank 4099
yor1−dF miss 0 orf −0.2 int −4 rank 2352
NOP16(YER002W)
DR = 2160
Yor1 miss 0 orf −2.8 int 14.6 rank 193
yor1−dF miss 0 orf −6.4 int −4 rank 2353
SVF1(YDR346C)
DR = 1301
Yor1 miss 0 orf −5.3 int 4.6 rank 1053
yor1−dF miss 0 orf −6.4 int −4 rank 2354
(YER128W)
DR = 1613
Yor1 miss 0 orf 11.2 int 6.6 rank 742
yor1−dF miss 0 orf −0.4 int −4 rank 2355
PDR15(YDR406W)
DR = −397
Yor1 miss 0 orf 3.5 int −2.7 rank 2753
yor1−dF miss 0 orf −3.8 int −4 rank 2356

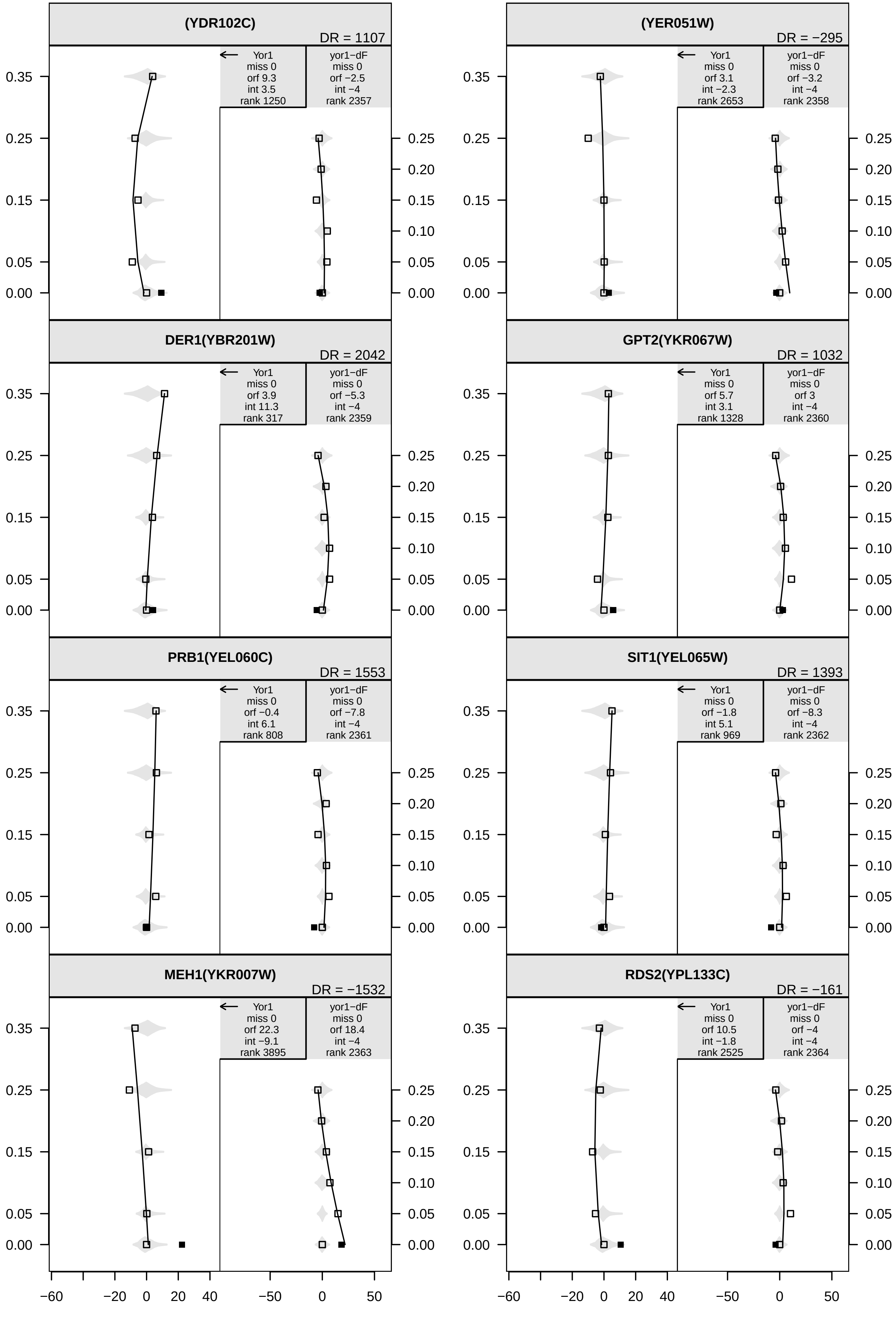
(YDR102C)
DR = 1107
Yor1
miss 0
orf 9.3
int 3.5
rank 1250
yor1−dF
miss 0
orf −2.5
int −4
rank 2357
(YER051W)
DR = −295
Yor1
miss 0
orf 3.1
int −2.3
rank 2653
yor1−dF
miss 0
orf −3.2
int −4
rank 2358
DER1(YBR201W)
DR = 2042
Yor1
miss 0
orf 3.9
int 11.3
rank 317
yor1−dF
miss 0
orf −5.3
int −4
rank 2359
GPT2(YKR067W)
DR = 1032
Yor1
miss 0
orf 5.7
int 3.1
rank 1328
yor1−dF
miss 0
orf 3
int −4
rank 2360
PRB1(YEL060C)
DR = 1553
Yor1
miss 0
orf −0.4
int 6.1
rank 808
yor1−dF
miss 0
orf −7.8
int −4
rank 2361
SIT1(YEL065W)
DR = 1393
Yor1
miss 0
orf −1.8
int 5.1
rank 969
yor1−dF
miss 0
orf −8.3
int −4
rank 2362
MEH1(YKR007W)
DR = −1532
Yor1
miss 0
orf 22.3
int −9.1
rank 3895
yor1−dF
miss 0
orf 18.4
int −4
rank 2363
RDS2(YPL133C)
DR = −161
Yor1
miss 0
orf 10.5
int −1.8
rank 2525
yor1−dF
miss 0
orf −4
int −4
rank 2364
0.35
0.25
0.20
0.15
0.10
0.05
0.00
−60
−20
0
20
40
−50
0
50

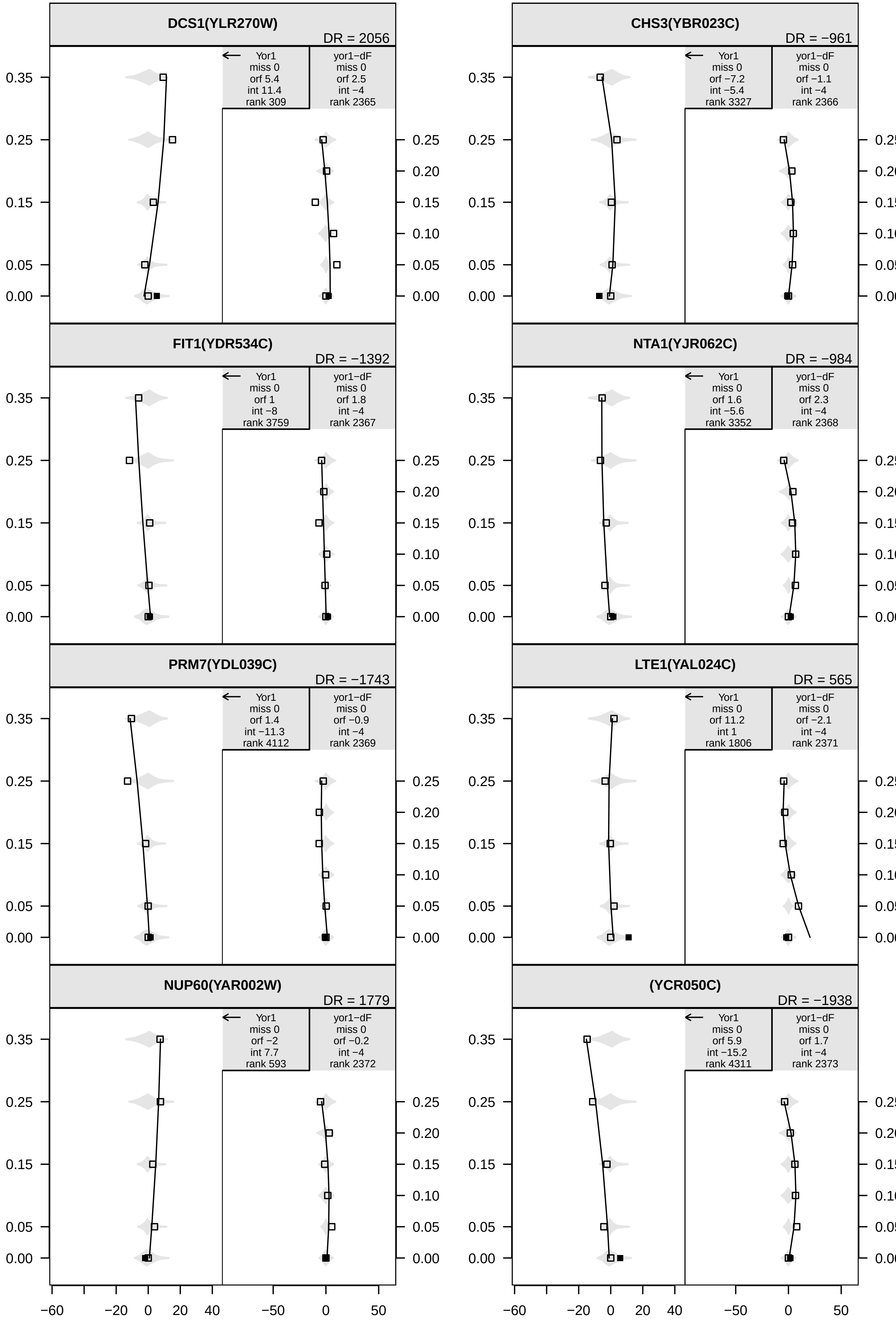

DCS1(YLR270W)
DR = 2056
Yor1
miss 0
orf 5.4
int 11.4
rank 309
yor1−dF
miss 0
orf 2.5
int −4
rank 2365
CHS3(YBR023C)
DR = −961
Yor1
miss 0
orf −7.2
int −5.4
rank 3327
yor1−dF
miss 0
orf −1.1
int −4
rank 2366
FIT1(YDR534C)
DR = −1392
Yor1
miss 0
orf 1
int −8
rank 3759
yor1−dF
miss 0
orf 1.8
int −4
rank 2367
NTA1(YJR062C)
DR = −984
Yor1
miss 0
orf 1.6
int −5.6
rank 3352
yor1−dF
miss 0
orf 2.3
int −4
rank 2368
PRM7(YDL039C)
DR = −1743
Yor1
miss 0
orf 1.4
int −11.3
rank 4112
yor1−dF
miss 0
orf −0.9
int −4
rank 2369
LTE1(YAL024C)
DR = 565
Yor1
miss 0
orf 11.2
int 1
rank 1806
yor1−dF
miss 0
orf −2.1
int −4
rank 2371
NUP60(YAR002W)
DR = 1779
Yor1
miss 0
orf −2
int 7.7
rank 593
yor1−dF
miss 0
orf −0.2
int −4
rank 2372
(YCR050C)
DR = −1938
Yor1
miss 0
orf 5.9
int −15.2
rank 4311
yor1−dF
miss 0
orf 1.7
int −4
rank 2373
0.35
0.25
0.20
0.15
0.10
0.05
0.00
−60
−20
0
20
40
−50
0
50

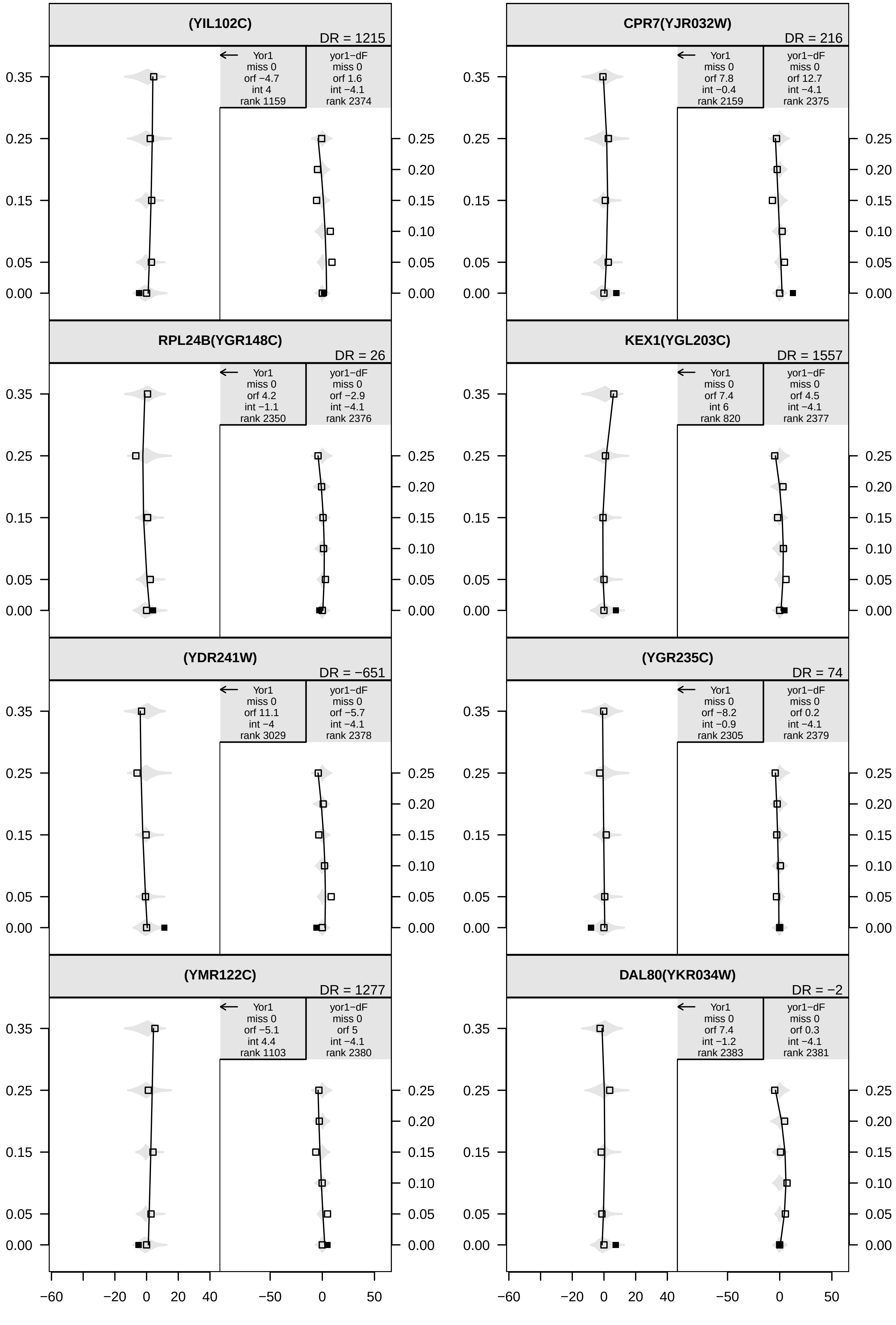

(YIL102C)
DR = 1215
Yor1 miss 0 orf −4.7 int 4 rank 1159
yor1−dF miss 0 orf 1.6 int −4.1 rank 2374
CPR7(YJR032W)
DR = 216
Yor1 miss 0 orf 7.8 int −0.4 rank 2159
yor1−dF miss 0 orf 12.7 int −4.1 rank 2375
RPL24B(YGR148C)
DR = 26
Yor1 miss 0 orf 4.2 int −1.1 rank 2350
yor1−dF miss 0 orf −2.9 int −4.1 rank 2376
KEX1(YGL203C)
DR = 1557
Yor1 miss 0 orf 7.4 int 6 rank 820
yor1−dF miss 0 orf 4.5 int −4.1 rank 2377
(YDR241W)
DR = −651
Yor1 miss 0 orf 11.1 int −4 rank 3029
yor1−dF miss 0 orf −5.7 int −4.1 rank 2378
(YGR235C)
DR = 74
Yor1 miss 0 orf −8.2 int −0.9 rank 2305
yor1−dF miss 0 orf 0.2 int −4.1 rank 2379
(YMR122C)
DR = 1277
Yor1 miss 0 orf −5.1 int 4.4 rank 1103
yor1−dF miss 0 orf 5 int −4.1 rank 2380
DAL80(YKR034W)
DR = −2
Yor1 miss 0 orf 7.4 int −1.2 rank 2383
yor1−dF miss 0 orf 0.3 int −4.1 rank 2381

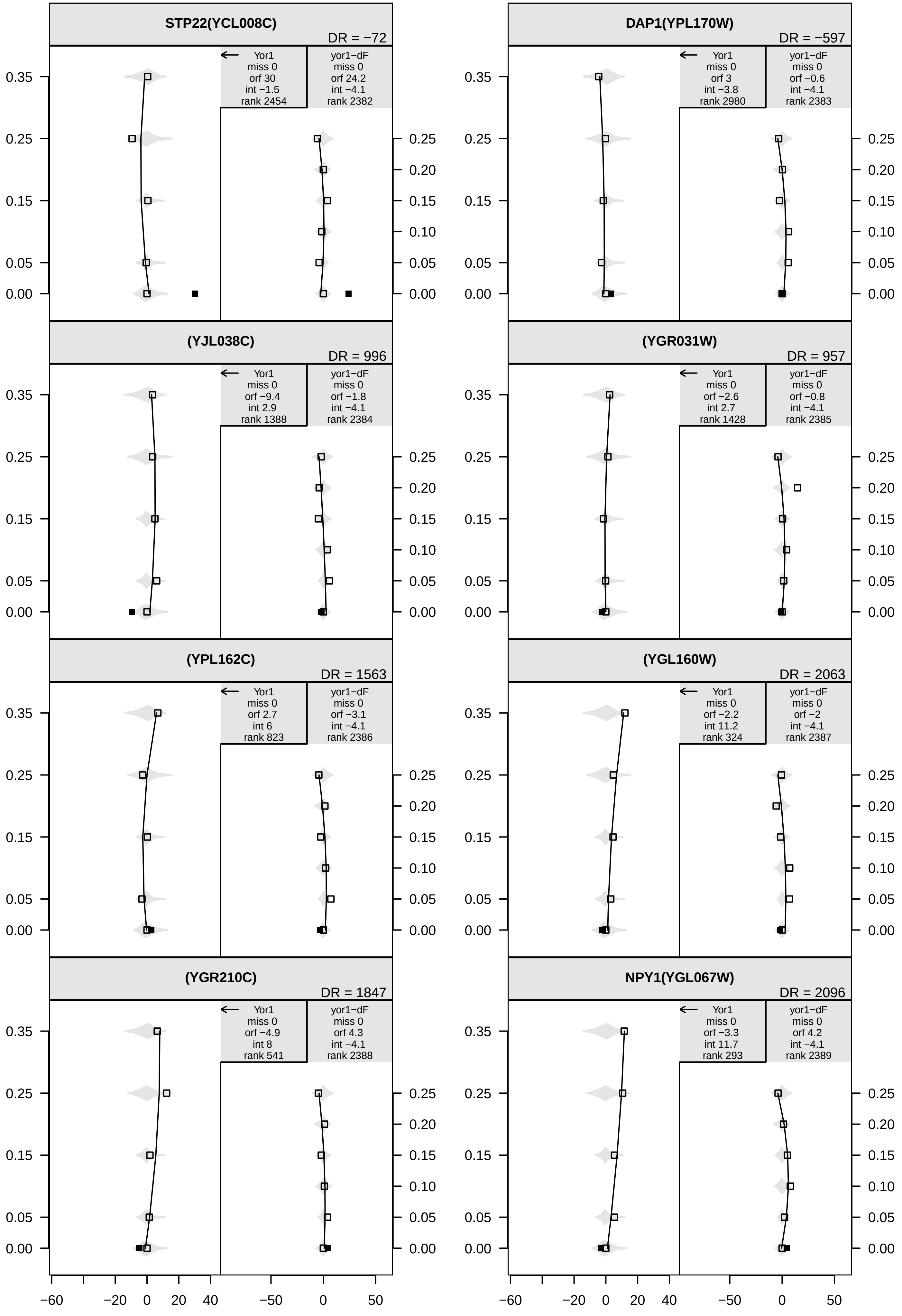
STP22(YCL008C)
DR = −72
Yor1 miss 0 orf 30 int −1.5 rank 2454
yor1−dF miss 0 orf 24.2 int −4.1 rank 2382
DAP1(YPL170W)
DR = −597
Yor1 miss 0 orf 3 int −3.8 rank 2980
yor1−dF miss 0 orf −0.6 int −4.1 rank 2383
(YJL038C)
DR = 996
Yor1 miss 0 orf −9.4 int 2.9 rank 1388
yor1−dF miss 0 orf −1.8 int −4.1 rank 2384
(YGR031W)
DR = 957
Yor1 miss 0 orf −2.6 int 2.7 rank 1428
yor1−dF miss 0 orf −0.8 int −4.1 rank 2385
(YPL162C)
DR = 1563
Yor1 miss 0 orf 2.7 int 6 rank 823
yor1−dF miss 0 orf −3.1 int −4.1 rank 2386
(YGL160W)
DR = 2063
Yor1 miss 0 orf −2.2 int 11.2 rank 324
yor1−dF miss 0 orf −2 int −4.1 rank 2387
(YGR210C)
DR = 1847
Yor1 miss 0 orf −4.9 int 8 rank 541
yor1−dF miss 0 orf 4.3 int −4.1 rank 2388
NPY1(YGL067W)
DR = 2096
Yor1 miss 0 orf −3.3 int 11.7 rank 293
yor1−dF miss 0 orf 4.2 int −4.1 rank 2389
0.35
0.25
0.20
0.15
0.10
0.05
0.00
−60
−20
0
20
40
−50
0
50

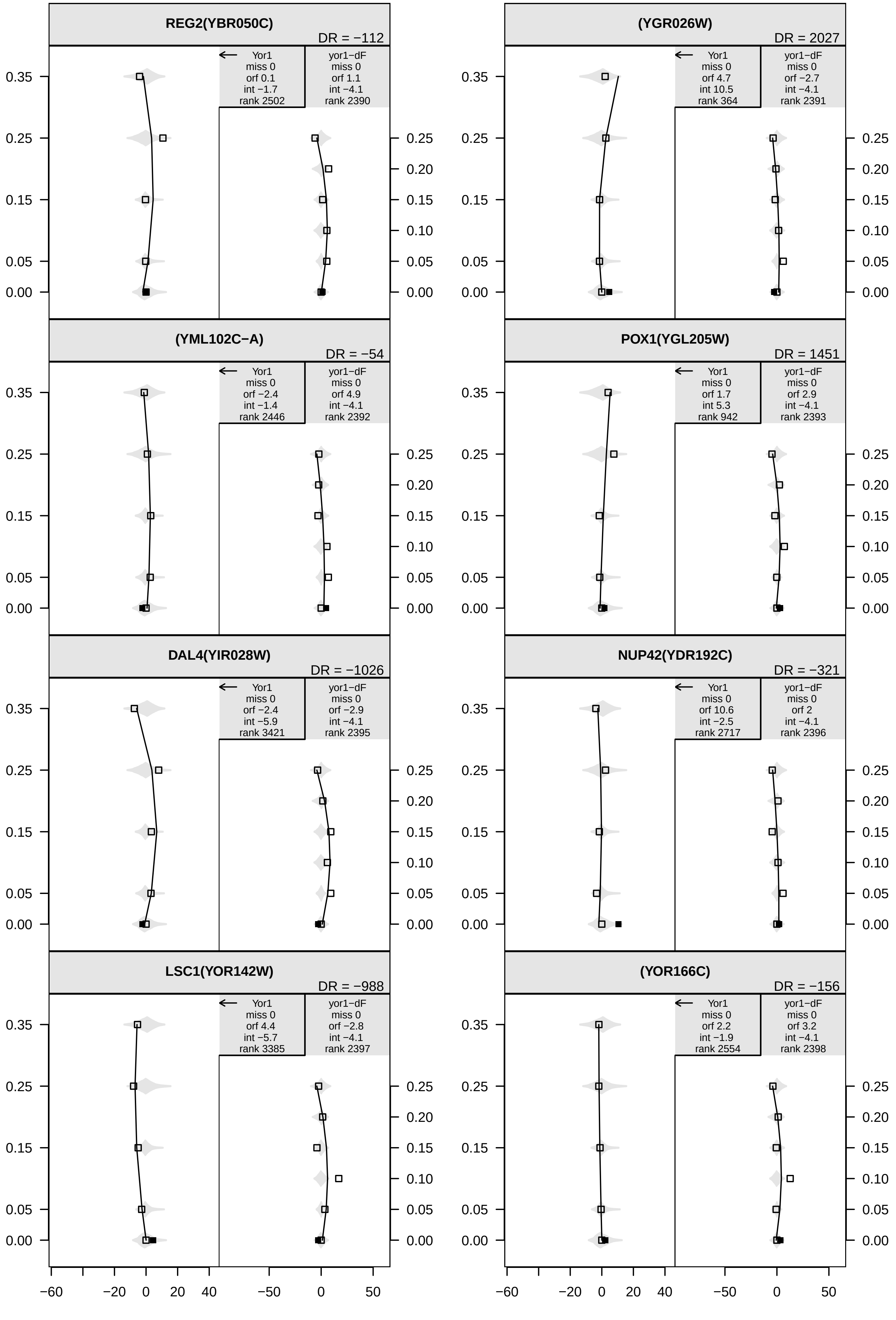

REG2(YBR050C)
DR = −112
Yor1
miss 0
orf 0.1
int −1.7
rank 2502
yor1−dF
miss 0
orf 1.1
int −4.1
rank 2390
(YGR026W)
DR = 2027
Yor1
miss 0
orf 4.7
int 10.5
rank 364
yor1−dF
miss 0
orf −2.7
int −4.1
rank 2391
(YML102C−A)
DR = −54
Yor1
miss 0
orf −2.4
int −1.4
rank 2446
yor1−dF
miss 0
orf 4.9
int −4.1
rank 2392
POX1(YGL205W)
DR = 1451
Yor1
miss 0
orf 1.7
int 5.3
rank 942
yor1−dF
miss 0
orf 2.9
int −4.1
rank 2393
DAL4(YIR028W)
DR = −1026
Yor1
miss 0
orf −2.4
int −5.9
rank 3421
yor1−dF
miss 0
orf −2.9
int −4.1
rank 2395
NUP42(YDR192C)
DR = −321
Yor1
miss 0
orf 10.6
int −2.5
rank 2717
yor1−dF
miss 0
orf 2
int −4.1
rank 2396
LSC1(YOR142W)
DR = −988
Yor1
miss 0
orf 4.4
int −5.7
rank 3385
yor1−dF
miss 0
orf −2.8
int −4.1
rank 2397
(YOR166C)
DR = −156
Yor1
miss 0
orf 2.2
int −1.9
rank 2554
yor1−dF
miss 0
orf 3.2
int −4.1
rank 2398
0.35
0.25
0.20
0.15
0.10
0.05
0.00
−60
−20
0
20
40
−50
0
50

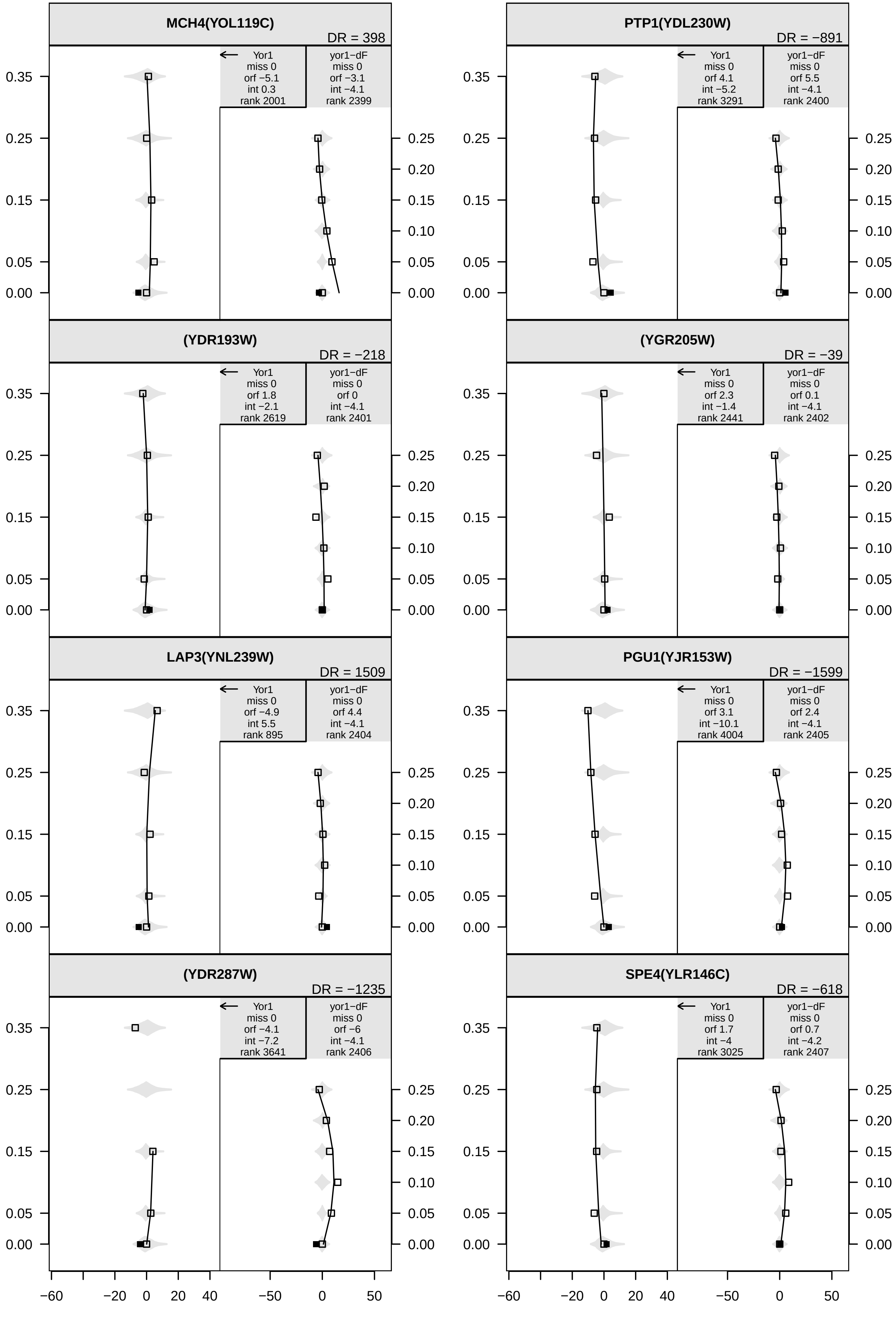
MCH4(YOL119C)
DR = 398
Yor1 miss 0 orf −5.1 int 0.3 rank 2001
yor1−dF miss 0 orf −3.1 int −4.1 rank 2399
PTP1(YDL230W)
DR = −891
Yor1 miss 0 orf 4.1 int −5.2 rank 3291
yor1−dF miss 0 orf 5.5 int −4.1 rank 2400
(YDR193W)
DR = −218
Yor1 miss 0 orf 1.8 int −2.1 rank 2619
yor1−dF miss 0 orf 0 int −4.1 rank 2401
(YGR205W)
DR = −39
Yor1 miss 0 orf 2.3 int −1.4 rank 2441
yor1−dF miss 0 orf 0.1 int −4.1 rank 2402
LAP3(YNL239W)
DR = 1509
Yor1 miss 0 orf −4.9 int 5.5 rank 895
yor1−dF miss 0 orf 4.4 int −4.1 rank 2404
PGU1(YJR153W)
DR = −1599
Yor1 miss 0 orf 3.1 int −10.1 rank 4004
yor1−dF miss 0 orf 2.4 int −4.1 rank 2405
(YDR287W)
DR = −1235
Yor1 miss 0 orf −4.1 int −7.2 rank 3641
yor1−dF miss 0 orf −6 int −4.1 rank 2406
SPE4(YLR146C)
DR = −618
Yor1 miss 0 orf 1.7 int −4 rank 3025
yor1−dF miss 0 orf 0.7 int −4.2 rank 2407
0.00 0.05 0.15 0.25 0.35
0.00 0.05 0.10 0.15 0.20 0.25
−60 −20 0 20 40
−50 0 50

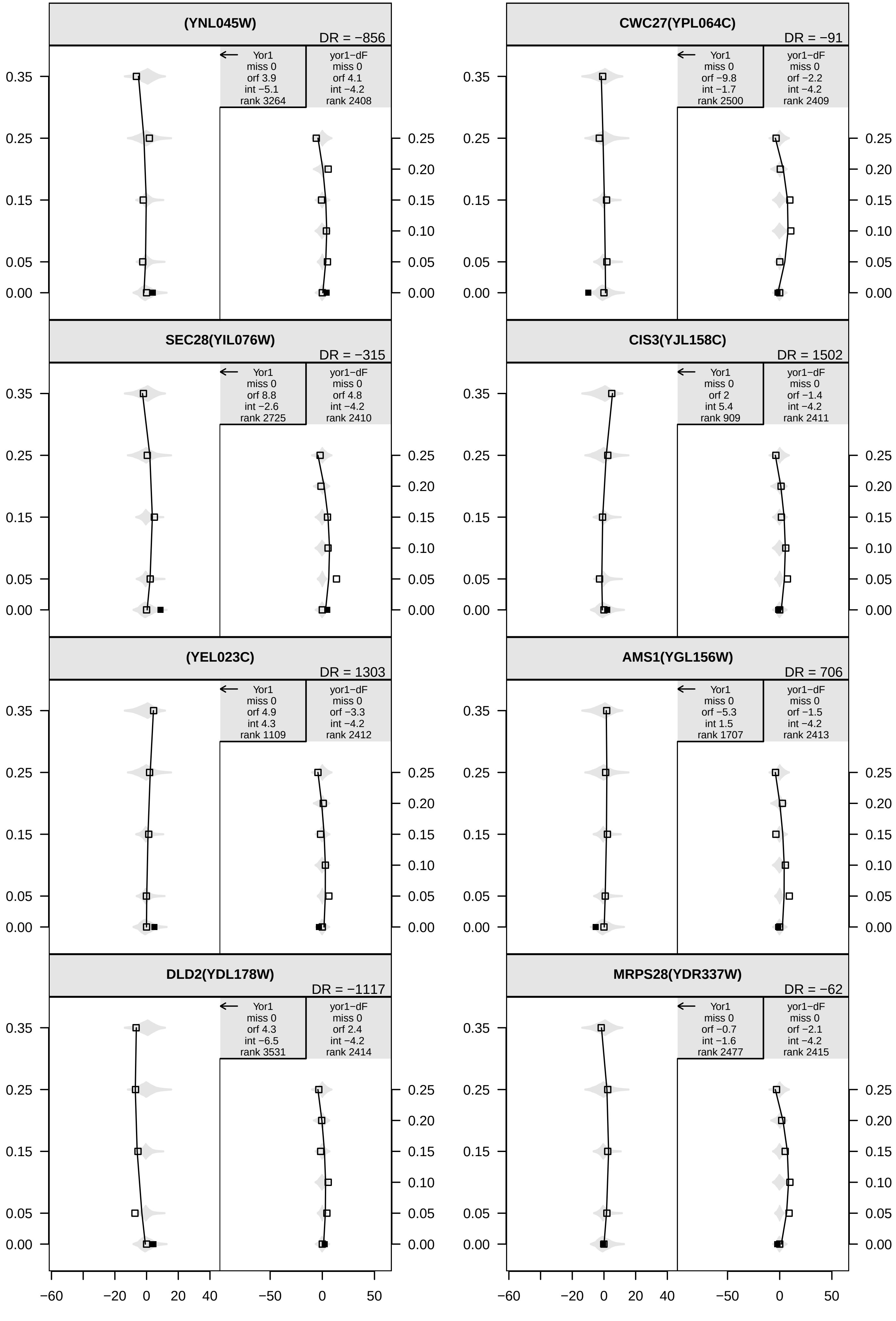

(YNL045W)
DR = −856
Yor1 miss 0 orf 3.9 int −5.1 rank 3264
yor1−dF miss 0 orf 4.1 int −4.2 rank 2408
CWC27(YPL064C)
DR = −91
Yor1 miss 0 orf −9.8 int −1.7 rank 2500
yor1−dF miss 0 orf −2.2 int −4.2 rank 2409
SEC28(YIL076W)
DR = −315
Yor1 miss 0 orf 8.8 int −2.6 rank 2725
yor1−dF miss 0 orf 4.8 int −4.2 rank 2410
CIS3(YJL158C)
DR = 1502
Yor1 miss 0 orf 2 int 5.4 rank 909
yor1−dF miss 0 orf −1.4 int −4.2 rank 2411
(YEL023C)
DR = 1303
Yor1 miss 0 orf 4.9 int 4.3 rank 1109
yor1−dF miss 0 orf −3.3 int −4.2 rank 2412
AMS1(YGL156W)
DR = 706
Yor1 miss 0 orf −5.3 int 1.5 rank 1707
yor1−dF miss 0 orf −1.5 int −4.2 rank 2413
DLD2(YDL178W)
DR = −1117
Yor1 miss 0 orf 4.3 int −6.5 rank 3531
yor1−dF miss 0 orf 2.4 int −4.2 rank 2414
MRPS28(YDR337W)
DR = −62
Yor1 miss 0 orf −0.7 int −1.6 rank 2477
yor1−dF miss 0 orf −2.1 int −4.2 rank 2415
0.35 0.25 0.20 0.15 0.10 0.05 0.00
−60 −20 0 20 40 −50 0 50

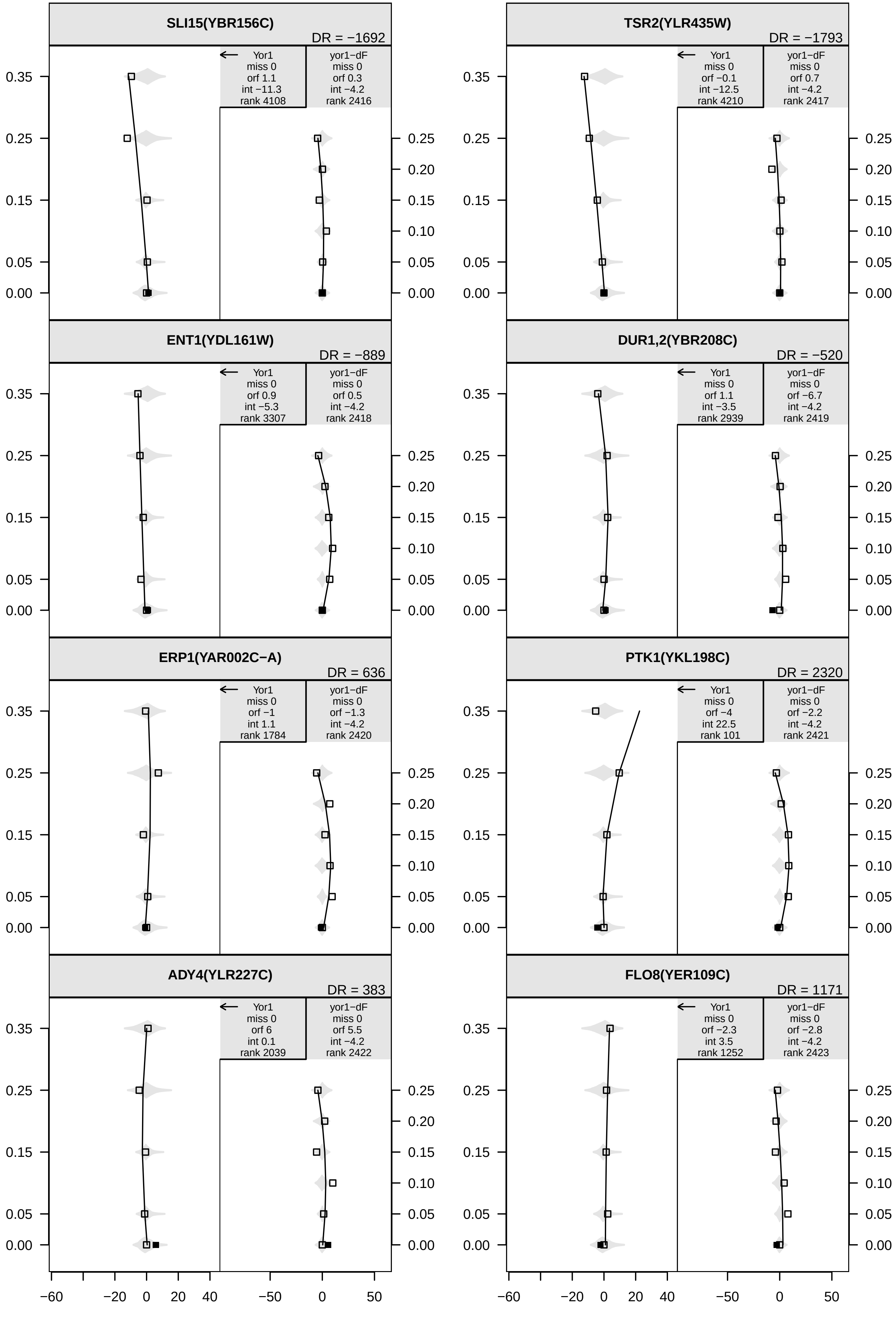

SLI15(YBR156C)
DR = −1692
Yor1 miss 0 orf 1.1 int −11.3 rank 4108
yor1−dF miss 0 orf 0.3 int −4.2 rank 2416
TSR2(YLR435W)
DR = −1793
Yor1 miss 0 orf −0.1 int −12.5 rank 4210
yor1−dF miss 0 orf 0.7 int −4.2 rank 2417
ENT1(YDL161W)
DR = −889
Yor1 miss 0 orf 0.9 int −5.3 rank 3307
yor1−dF miss 0 orf 0.5 int −4.2 rank 2418
DUR1,2(YBR208C)
DR = −520
Yor1 miss 0 orf 1.1 int −3.5 rank 2939
yor1−dF miss 0 orf −6.7 int −4.2 rank 2419
ERP1(YAR002C−A)
DR = 636
Yor1 miss 0 orf −1 int 1.1 rank 1784
yor1−dF miss 0 orf −1.3 int −4.2 rank 2420
PTK1(YKL198C)
DR = 2320
Yor1 miss 0 orf −4 int 22.5 rank 101
yor1−dF miss 0 orf −2.2 int −4.2 rank 2421
ADY4(YLR227C)
DR = 383
Yor1 miss 0 orf 6 int 0.1 rank 2039
yor1−dF miss 0 orf 5.5 int −4.2 rank 2422
FLO8(YER109C)
DR = 1171
Yor1 miss 0 orf −2.3 int 3.5 rank 1252
yor1−dF miss 0 orf −2.8 int −4.2 rank 2423

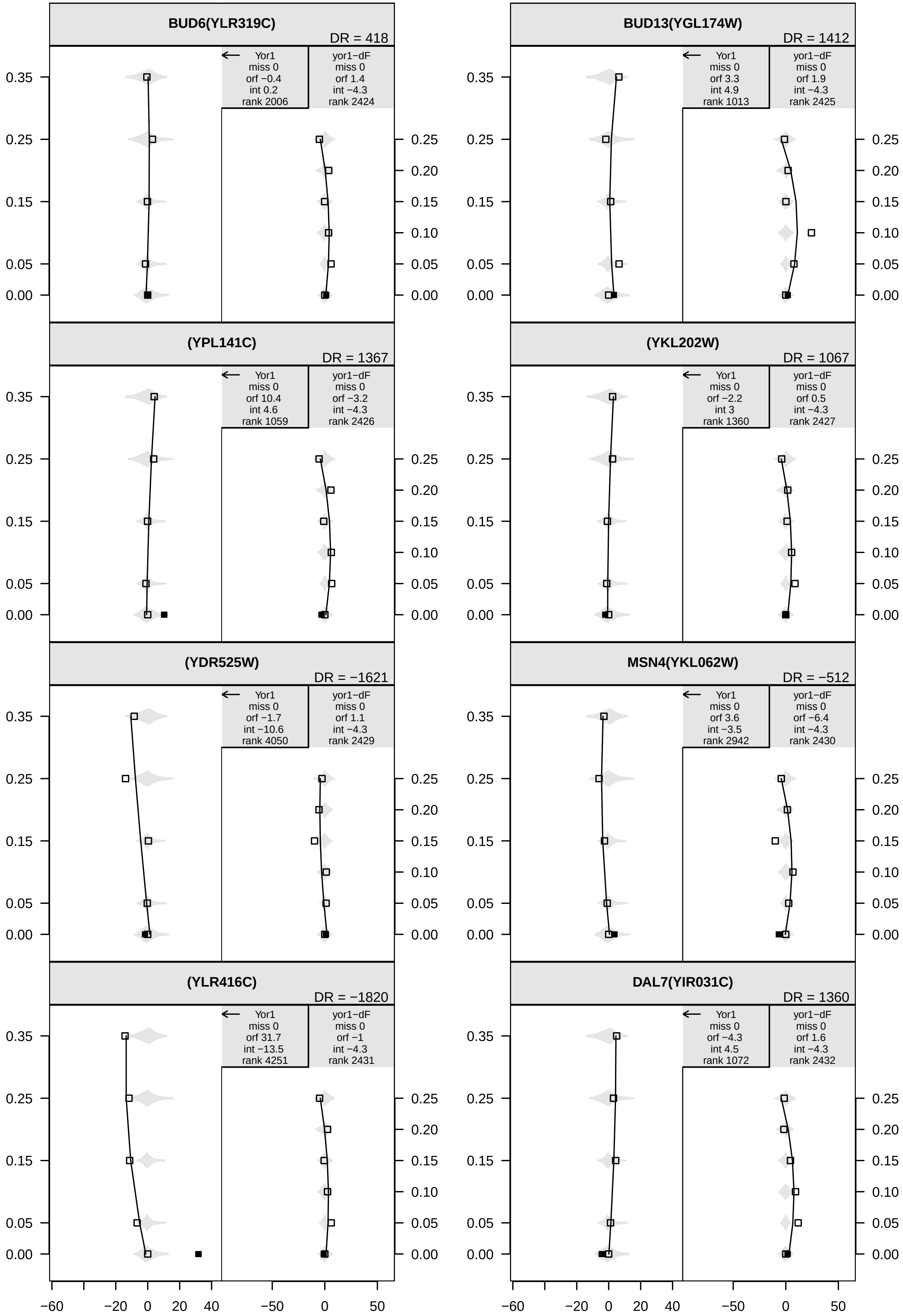

BUD6(YLR319C)
DR = 418
Yor1 miss 0 orf −0.4 int 0.2 rank 2006
yor1−dF miss 0 orf 1.4 int −4.3 rank 2424
BUD13(YGL174W)
DR = 1412
Yor1 miss 0 orf 3.3 int 4.9 rank 1013
yor1−dF miss 0 orf 1.9 int −4.3 rank 2425
(YPL141C)
DR = 1367
Yor1 miss 0 orf 10.4 int 4.6 rank 1059
yor1−dF miss 0 orf −3.2 int −4.3 rank 2426
(YKL202W)
DR = 1067
Yor1 miss 0 orf −2.2 int 3 rank 1360
yor1−dF miss 0 orf 0.5 int −4.3 rank 2427
(YDR525W)
DR = −1621
Yor1 miss 0 orf −1.7 int −10.6 rank 4050
yor1−dF miss 0 orf 1.1 int −4.3 rank 2429
MSN4(YKL062W)
DR = −512
Yor1 miss 0 orf 3.6 int −3.5 rank 2942
yor1−dF miss 0 orf −6.4 int −4.3 rank 2430
(YLR416C)
DR = −1820
Yor1 miss 0 orf 31.7 int −13.5 rank 4251
yor1−dF miss 0 orf −1 int −4.3 rank 2431
DAL7(YIR031C)
DR = 1360
Yor1 miss 0 orf −4.3 int 4.5 rank 1072
yor1−dF miss 0 orf 1.6 int −4.3 rank 2432
0.00 0.05 0.10 0.15 0.20 0.25 0.35
−60 −20 0 20 40 −50 0 50

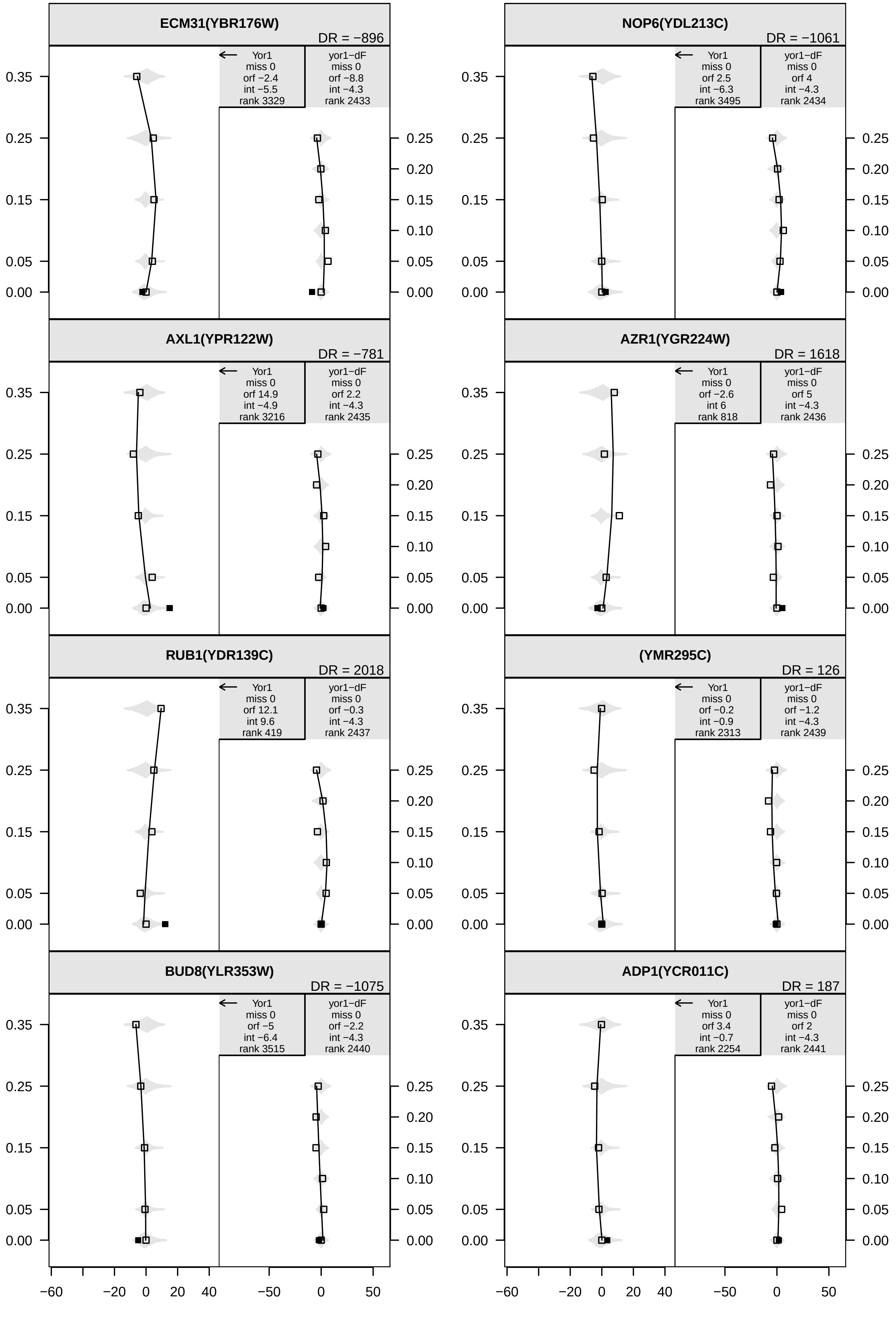

ECM31(YBR176W)
DR = −896
Yor1 miss 0 orf −2.4 int −5.5 rank 3329
yor1−dF miss 0 orf −8.8 int −4.3 rank 2433
NOP6(YDL213C)
DR = −1061
Yor1 miss 0 orf 2.5 int −6.3 rank 3495
yor1−dF miss 0 orf 4 int −4.3 rank 2434
AXL1(YPR122W)
DR = −781
Yor1 miss 0 orf 14.9 int −4.9 rank 3216
yor1−dF miss 0 orf 2.2 int −4.3 rank 2435
AZR1(YGR224W)
DR = 1618
Yor1 miss 0 orf −2.6 int 6 rank 818
yor1−dF miss 0 orf 5 int −4.3 rank 2436
RUB1(YDR139C)
DR = 2018
Yor1 miss 0 orf 12.1 int 9.6 rank 419
yor1−dF miss 0 orf −0.3 int −4.3 rank 2437
(YMR295C)
DR = 126
Yor1 miss 0 orf −0.2 int −0.9 rank 2313
yor1−dF miss 0 orf −1.2 int −4.3 rank 2439
BUD8(YLR353W)
DR = −1075
Yor1 miss 0 orf −5 int −6.4 rank 3515
yor1−dF miss 0 orf −2.2 int −4.3 rank 2440
ADP1(YCR011C)
DR = 187
Yor1 miss 0 orf 3.4 int −0.7 rank 2254
yor1−dF miss 0 orf 2 int −4.3 rank 2441
0.00 0.05 0.10 0.15 0.20 0.25 0.35
−60 −20 0 20 40
−50 0 50

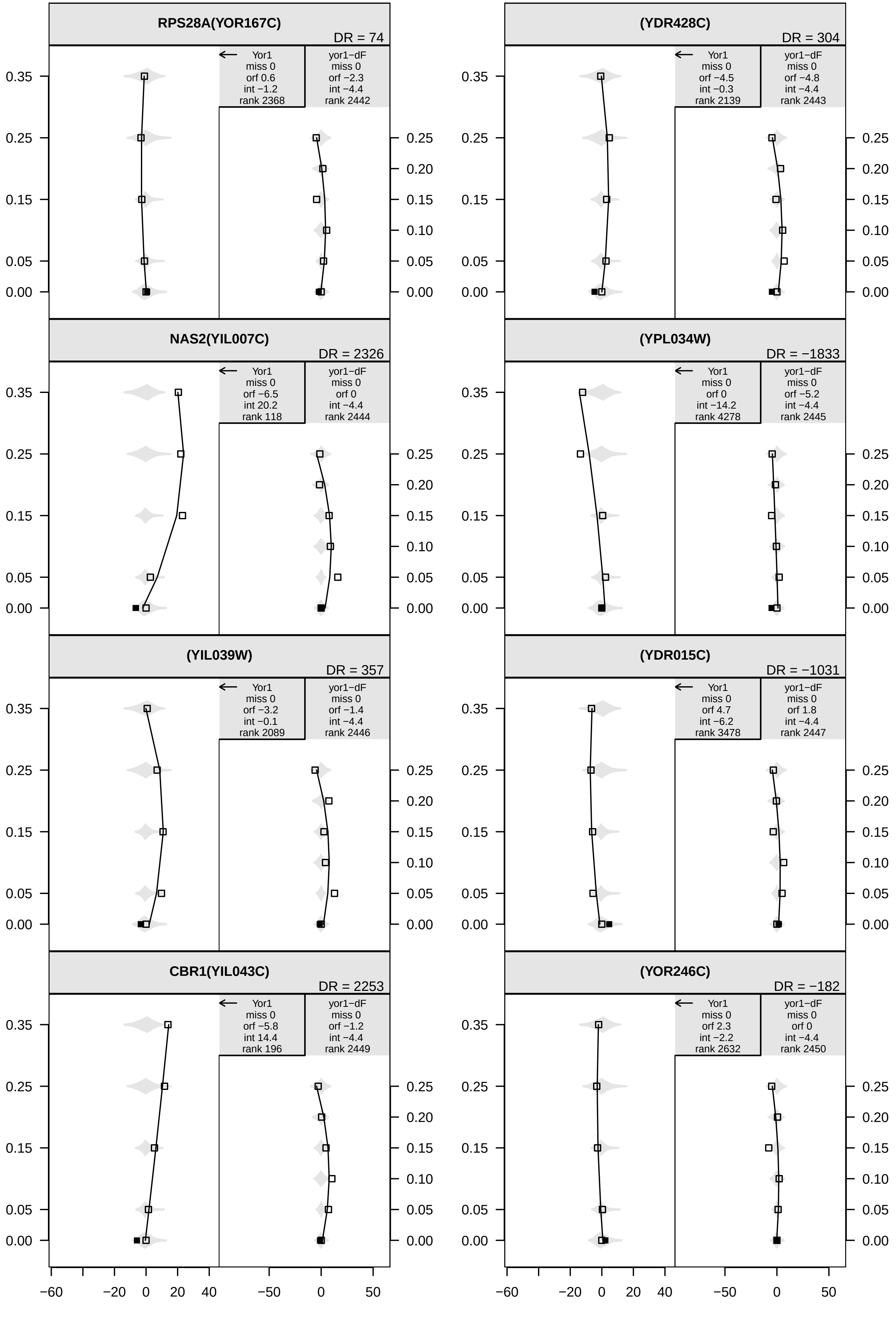
RPS28A(YOR167C)
DR = 74
Yor1 miss 0 orf 0.6 int −1.2 rank 2368
yor1−dF miss 0 orf −2.3 int −4.4 rank 2442
(YDR428C)
DR = 304
Yor1 miss 0 orf −4.5 int −0.3 rank 2139
yor1−dF miss 0 orf −4.8 int −4.4 rank 2443
NAS2(YIL007C)
DR = 2326
Yor1 miss 0 orf −6.5 int 20.2 rank 118
yor1−dF miss 0 orf 0 int −4.4 rank 2444
(YPL034W)
DR = −1833
Yor1 miss 0 orf 0 int −14.2 rank 4278
yor1−dF miss 0 orf −5.2 int −4.4 rank 2445
(YIL039W)
DR = 357
Yor1 miss 0 orf −3.2 int −0.1 rank 2089
yor1−dF miss 0 orf −1.4 int −4.4 rank 2446
(YDR015C)
DR = −1031
Yor1 miss 0 orf 4.7 int −6.2 rank 3478
yor1−dF miss 0 orf 1.8 int −4.4 rank 2447
CBR1(YIL043C)
DR = 2253
Yor1 miss 0 orf −5.8 int 14.4 rank 196
yor1−dF miss 0 orf −1.2 int −4.4 rank 2449
(YOR246C)
DR = −182
Yor1 miss 0 orf 2.3 int −2.2 rank 2632
yor1−dF miss 0 orf 0 int −4.4 rank 2450
0.00 0.05 0.10 0.15 0.20 0.25 0.35
−60 −20 0 20 40 −50 0 50

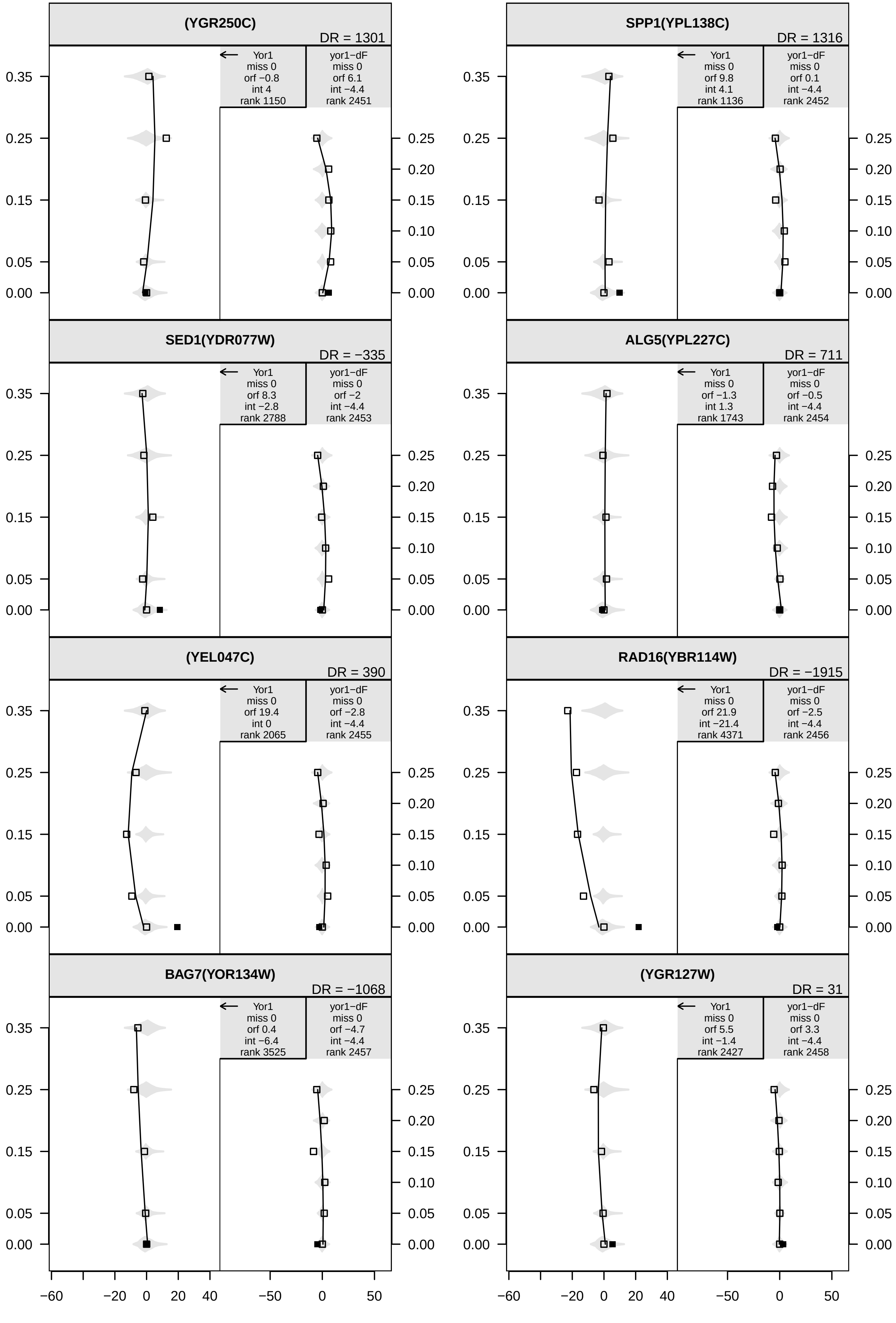
(YGR250C)
DR = 1301
Yor1
miss 0
orf −0.8
int 4
rank 1150
yor1−dF
miss 0
orf 6.1
int −4.4
rank 2451
SPP1(YPL138C)
DR = 1316
Yor1
miss 0
orf 9.8
int 4.1
rank 1136
yor1−dF
miss 0
orf 0.1
int −4.4
rank 2452
SED1(YDR077W)
DR = −335
Yor1
miss 0
orf 8.3
int −2.8
rank 2788
yor1−dF
miss 0
orf −2
int −4.4
rank 2453
ALG5(YPL227C)
DR = 711
Yor1
miss 0
orf −1.3
int 1.3
rank 1743
yor1−dF
miss 0
orf −0.5
int −4.4
rank 2454
(YEL047C)
DR = 390
Yor1
miss 0
orf 19.4
int 0
rank 2065
yor1−dF
miss 0
orf −2.8
int −4.4
rank 2455
RAD16(YBR114W)
DR = −1915
Yor1
miss 0
orf 21.9
int −21.4
rank 4371
yor1−dF
miss 0
orf −2.5
int −4.4
rank 2456
BAG7(YOR134W)
DR = −1068
Yor1
miss 0
orf 0.4
int −6.4
rank 3525
yor1−dF
miss 0
orf −4.7
int −4.4
rank 2457
(YGR127W)
DR = 31
Yor1
miss 0
orf 5.5
int −1.4
rank 2427
yor1−dF
miss 0
orf 3.3
int −4.4
rank 2458
0.35
0.25
0.20
0.15
0.10
0.05
0.00
−60
−20
0
20
40
−50
0
50

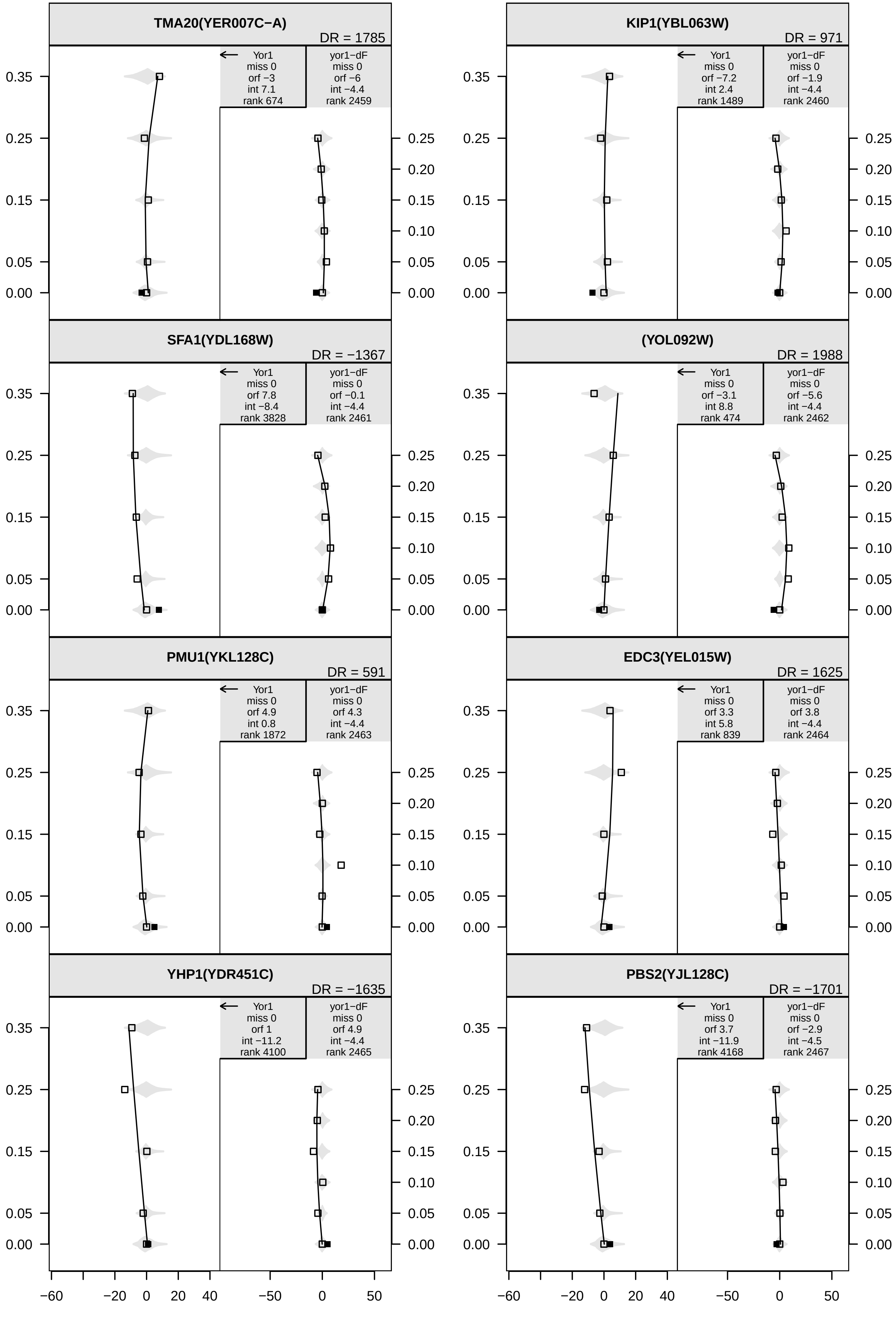
TMA20(YER007C−A)
DR = 1785
Yor1 miss 0 orf −3 int 7.1 rank 674
yor1−dF miss 0 orf −6 int −4.4 rank 2459
KIP1(YBL063W)
DR = 971
Yor1 miss 0 orf −7.2 int 2.4 rank 1489
yor1−dF miss 0 orf −1.9 int −4.4 rank 2460
SFA1(YDL168W)
DR = −1367
Yor1 miss 0 orf 7.8 int −8.4 rank 3828
yor1−dF miss 0 orf −0.1 int −4.4 rank 2461
(YOL092W)
DR = 1988
Yor1 miss 0 orf −3.1 int 8.8 rank 474
yor1−dF miss 0 orf −5.6 int −4.4 rank 2462
PMU1(YKL128C)
DR = 591
Yor1 miss 0 orf 4.9 int 0.8 rank 1872
yor1−dF miss 0 orf 4.3 int −4.4 rank 2463
EDC3(YEL015W)
DR = 1625
Yor1 miss 0 orf 3.3 int 5.8 rank 839
yor1−dF miss 0 orf 3.8 int −4.4 rank 2464
YHP1(YDR451C)
DR = −1635
Yor1 miss 0 orf 1 int −11.2 rank 4100
yor1−dF miss 0 orf 4.9 int −4.4 rank 2465
PBS2(YJL128C)
DR = −1701
Yor1 miss 0 orf 3.7 int −11.9 rank 4168
yor1−dF miss 0 orf −2.9 int −4.5 rank 2467
0.35 0.25 0.15 0.05 0.00
0.25 0.20 0.15 0.10 0.05 0.00
−60 −20 0 20 40 −50 0 50

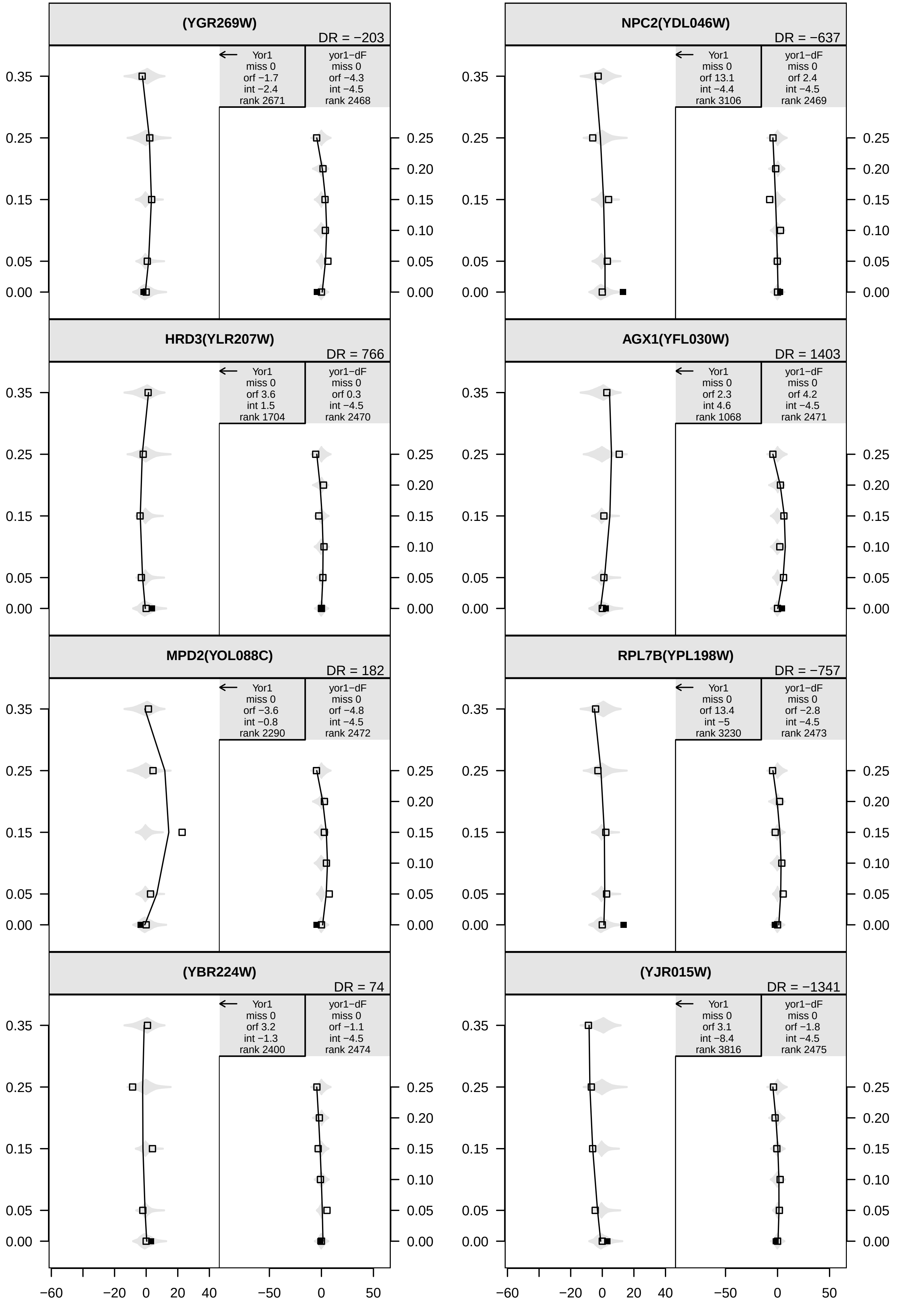
(YGR269W)
DR = −203
Yor1
miss 0
orf −1.7
int −2.4
rank 2671
yor1−dF
miss 0
orf −4.3
int −4.5
rank 2468
NPC2(YDL046W)
DR = −637
Yor1
miss 0
orf 13.1
int −4.4
rank 3106
yor1−dF
miss 0
orf 2.4
int −4.5
rank 2469
HRD3(YLR207W)
DR = 766
Yor1
miss 0
orf 3.6
int 1.5
rank 1704
yor1−dF
miss 0
orf 0.3
int −4.5
rank 2470
AGX1(YFL030W)
DR = 1403
Yor1
miss 0
orf 2.3
int 4.6
rank 1068
yor1−dF
miss 0
orf 4.2
int −4.5
rank 2471
MPD2(YOL088C)
DR = 182
Yor1
miss 0
orf −3.6
int −0.8
rank 2290
yor1−dF
miss 0
orf −4.8
int −4.5
rank 2472
RPL7B(YPL198W)
DR = −757
Yor1
miss 0
orf 13.4
int −5
rank 3230
yor1−dF
miss 0
orf −2.8
int −4.5
rank 2473
(YBR224W)
DR = 74
Yor1
miss 0
orf 3.2
int −1.3
rank 2400
yor1−dF
miss 0
orf −1.1
int −4.5
rank 2474
(YJR015W)
DR = −1341
Yor1
miss 0
orf 3.1
int −8.4
rank 3816
yor1−dF
miss 0
orf −1.8
int −4.5
rank 2475
0.35
0.25
0.20
0.15
0.10
0.05
0.00
−60
−20
0
20
40
−50
0
50

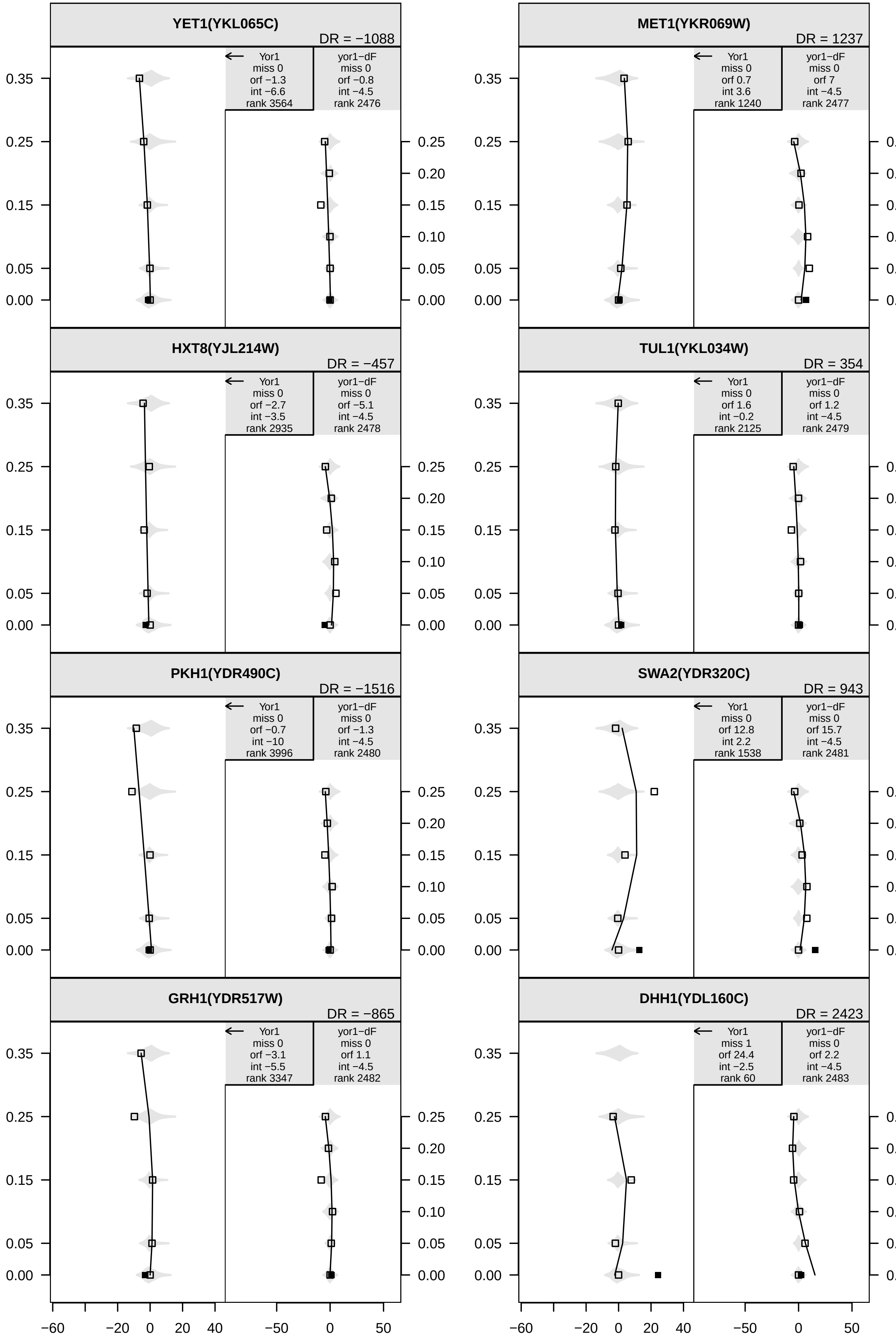
YET1(YKL065C)
DR = −1088
Yor1
miss 0
orf −1.3
int −6.6
rank 3564
yor1−dF
miss 0
orf −0.8
int −4.5
rank 2476
MET1(YKR069W)
DR = 1237
Yor1
miss 0
orf 0.7
int 3.6
rank 1240
yor1−dF
miss 0
orf 7
int −4.5
rank 2477
HXT8(YJL214W)
DR = −457
Yor1
miss 0
orf −2.7
int −3.5
rank 2935
yor1−dF
miss 0
orf −5.1
int −4.5
rank 2478
TUL1(YKL034W)
DR = 354
Yor1
miss 0
orf 1.6
int −0.2
rank 2125
yor1−dF
miss 0
orf 1.2
int −4.5
rank 2479
PKH1(YDR490C)
DR = −1516
Yor1
miss 0
orf −0.7
int −10
rank 3996
yor1−dF
miss 0
orf −1.3
int −4.5
rank 2480
SWA2(YDR320C)
DR = 943
Yor1
miss 0
orf 12.8
int 2.2
rank 1538
yor1−dF
miss 0
orf 15.7
int −4.5
rank 2481
GRH1(YDR517W)
DR = −865
Yor1
miss 0
orf −3.1
int −5.5
rank 3347
yor1−dF
miss 0
orf 1.1
int −4.5
rank 2482
DHH1(YDL160C)
DR = 2423
Yor1
miss 1
orf 24.4
int −2.5
rank 60
yor1−dF
miss 0
orf 2.2
int −4.5
rank 2483
0.35
0.25
0.20
0.15
0.10
0.05
0.00
−60
−20
0
20
40
−50
0
50

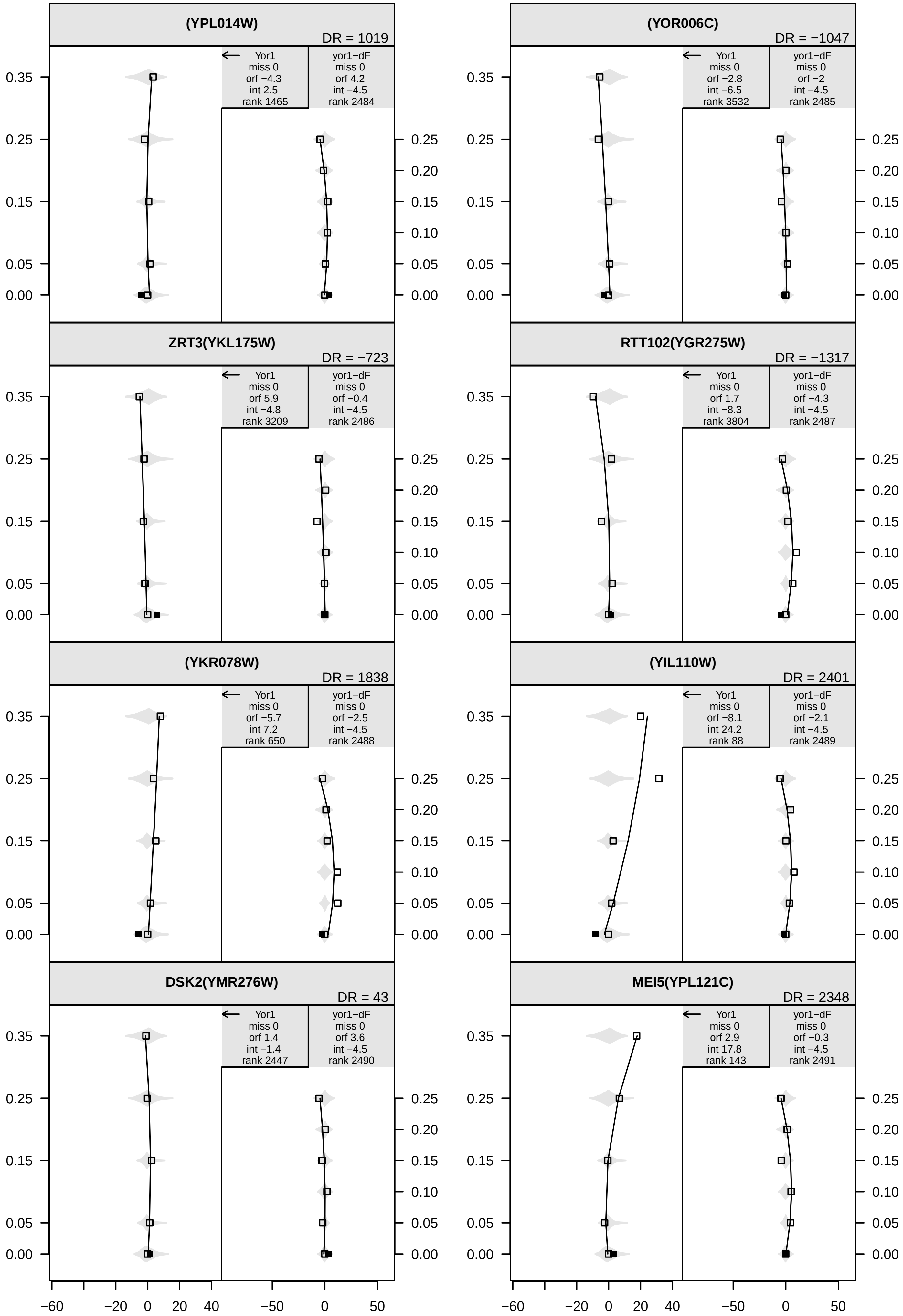

(YPL014W)
DR = 1019
Yor1 miss 0 orf −4.3 int 2.5 rank 1465
yor1−dF miss 0 orf 4.2 int −4.5 rank 2484
(YOR006C)
DR = −1047
Yor1 miss 0 orf −2.8 int −6.5 rank 3532
yor1−dF miss 0 orf −2 int −4.5 rank 2485
ZRT3(YKL175W)
DR = −723
Yor1 miss 0 orf 5.9 int −4.8 rank 3209
yor1−dF miss 0 orf −0.4 int −4.5 rank 2486
RTT102(YGR275W)
DR = −1317
Yor1 miss 0 orf 1.7 int −8.3 rank 3804
yor1−dF miss 0 orf −4.3 int −4.5 rank 2487
(YKR078W)
DR = 1838
Yor1 miss 0 orf −5.7 int 7.2 rank 650
yor1−dF miss 0 orf −2.5 int −4.5 rank 2488
(YIL110W)
DR = 2401
Yor1 miss 0 orf −8.1 int 24.2 rank 88
yor1−dF miss 0 orf −2.1 int −4.5 rank 2489
DSK2(YMR276W)
DR = 43
Yor1 miss 0 orf 1.4 int −1.4 rank 2447
yor1−dF miss 0 orf 3.6 int −4.5 rank 2490
MEI5(YPL121C)
DR = 2348
Yor1 miss 0 orf 2.9 int 17.8 rank 143
yor1−dF miss 0 orf −0.3 int −4.5 rank 2491
0.35 0.25 0.20 0.15 0.10 0.05 0.00
−60 −20 0 20 40 −50 0 50

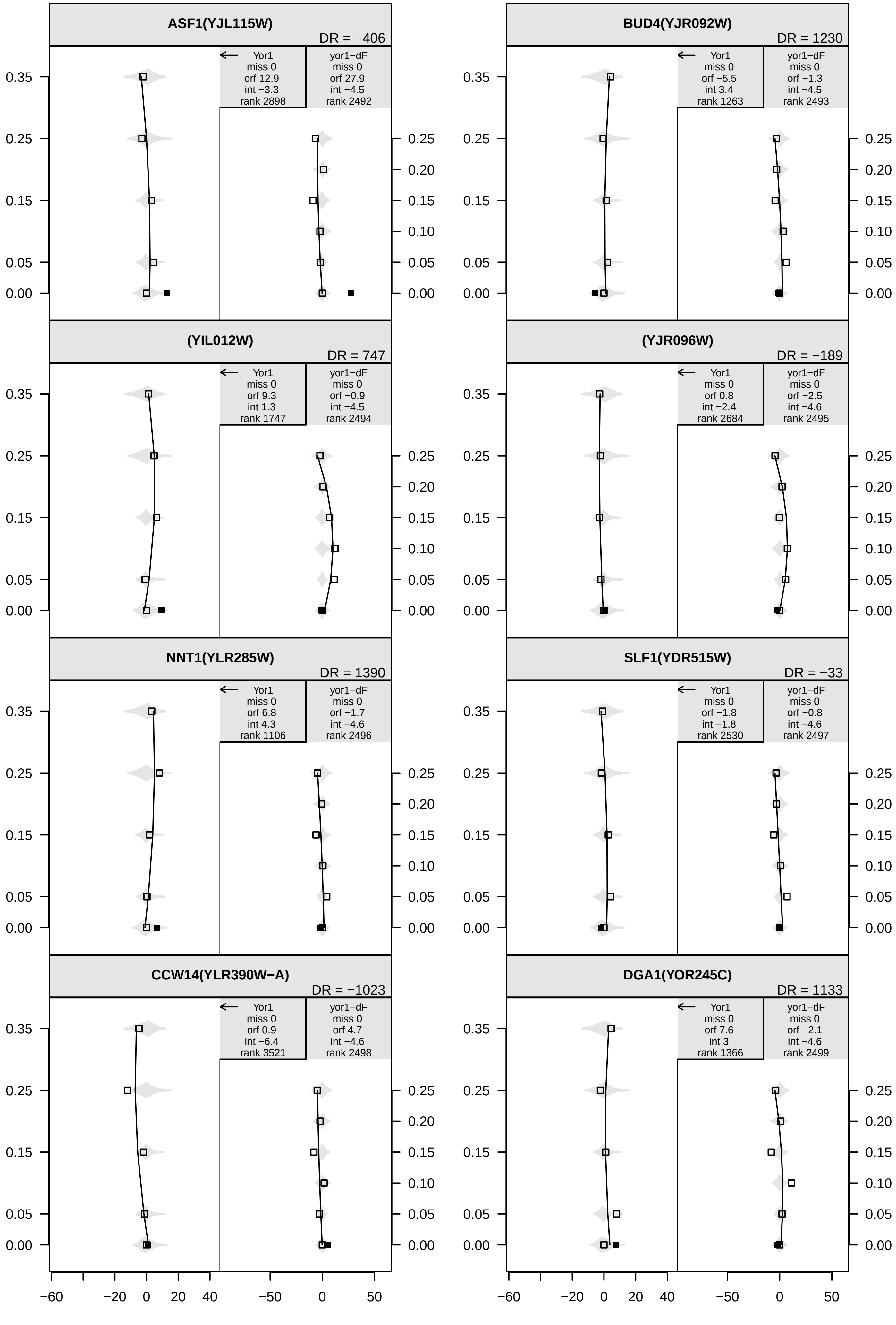
ASF1(YJL115W)
DR = −406
Yor1 miss 0 orf 12.9 int −3.3 rank 2898
yor1−dF miss 0 orf 27.9 int −4.5 rank 2492
BUD4(YJR092W)
DR = 1230
Yor1 miss 0 orf −5.5 int 3.4 rank 1263
yor1−dF miss 0 orf −1.3 int −4.5 rank 2493
(YIL012W)
DR = 747
Yor1 miss 0 orf 9.3 int 1.3 rank 1747
yor1−dF miss 0 orf −0.9 int −4.5 rank 2494
(YJR096W)
DR = −189
Yor1 miss 0 orf 0.8 int −2.4 rank 2684
yor1−dF miss 0 orf −2.5 int −4.6 rank 2495
NNT1(YLR285W)
DR = 1390
Yor1 miss 0 orf 6.8 int 4.3 rank 1106
yor1−dF miss 0 orf −1.7 int −4.6 rank 2496
SLF1(YDR515W)
DR = −33
Yor1 miss 0 orf −1.8 int −1.8 rank 2530
yor1−dF miss 0 orf −0.8 int −4.6 rank 2497
CCW14(YLR390W−A)
DR = −1023
Yor1 miss 0 orf 0.9 int −6.4 rank 3521
yor1−dF miss 0 orf 4.7 int −4.6 rank 2498
DGA1(YOR245C)
DR = 1133
Yor1 miss 0 orf 7.6 int 3 rank 1366
yor1−dF miss 0 orf −2.1 int −4.6 rank 2499
0.35
0.25
0.20
0.15
0.10
0.05
0.00
−60
−20
0
20
40
−50
50

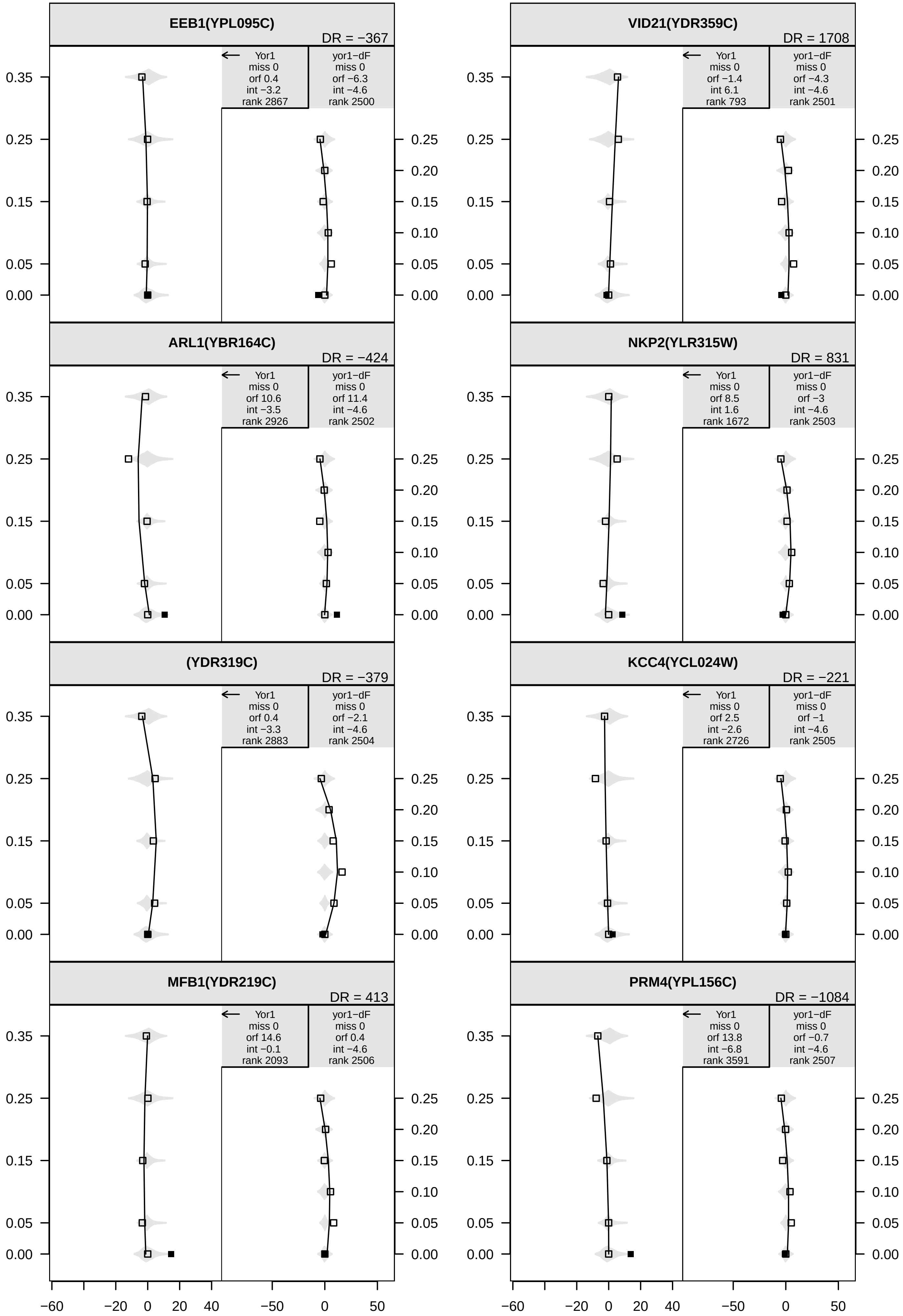

EEB1(YPL095C)
DR = −367
Yor1 miss 0 orf 0.4 int −3.2 rank 2867
yor1−dF miss 0 orf −6.3 int −4.6 rank 2500
VID21(YDR359C)
DR = 1708
Yor1 miss 0 orf −1.4 int 6.1 rank 793
yor1−dF miss 0 orf −4.3 int −4.6 rank 2501
ARL1(YBR164C)
DR = −424
Yor1 miss 0 orf 10.6 int −3.5 rank 2926
yor1−dF miss 0 orf 11.4 int −4.6 rank 2502
NKP2(YLR315W)
DR = 831
Yor1 miss 0 orf 8.5 int 1.6 rank 1672
yor1−dF miss 0 orf −3 int −4.6 rank 2503
(YDR319C)
DR = −379
Yor1 miss 0 orf 0.4 int −3.3 rank 2883
yor1−dF miss 0 orf −2.1 int −4.6 rank 2504
KCC4(YCL024W)
DR = −221
Yor1 miss 0 orf 2.5 int −2.6 rank 2726
yor1−dF miss 0 orf −1 int −4.6 rank 2505
MFB1(YDR219C)
DR = 413
Yor1 miss 0 orf 14.6 int −0.1 rank 2093
yor1−dF miss 0 orf 0.4 int −4.6 rank 2506
PRM4(YPL156C)
DR = −1084
Yor1 miss 0 orf 13.8 int −6.8 rank 3591
yor1−dF miss 0 orf −0.7 int −4.6 rank 2507
0.35 0.25 0.15 0.05 0.00
0.25 0.20 0.15 0.10 0.05 0.00
−60 −20 0 20 40 −50 0 50

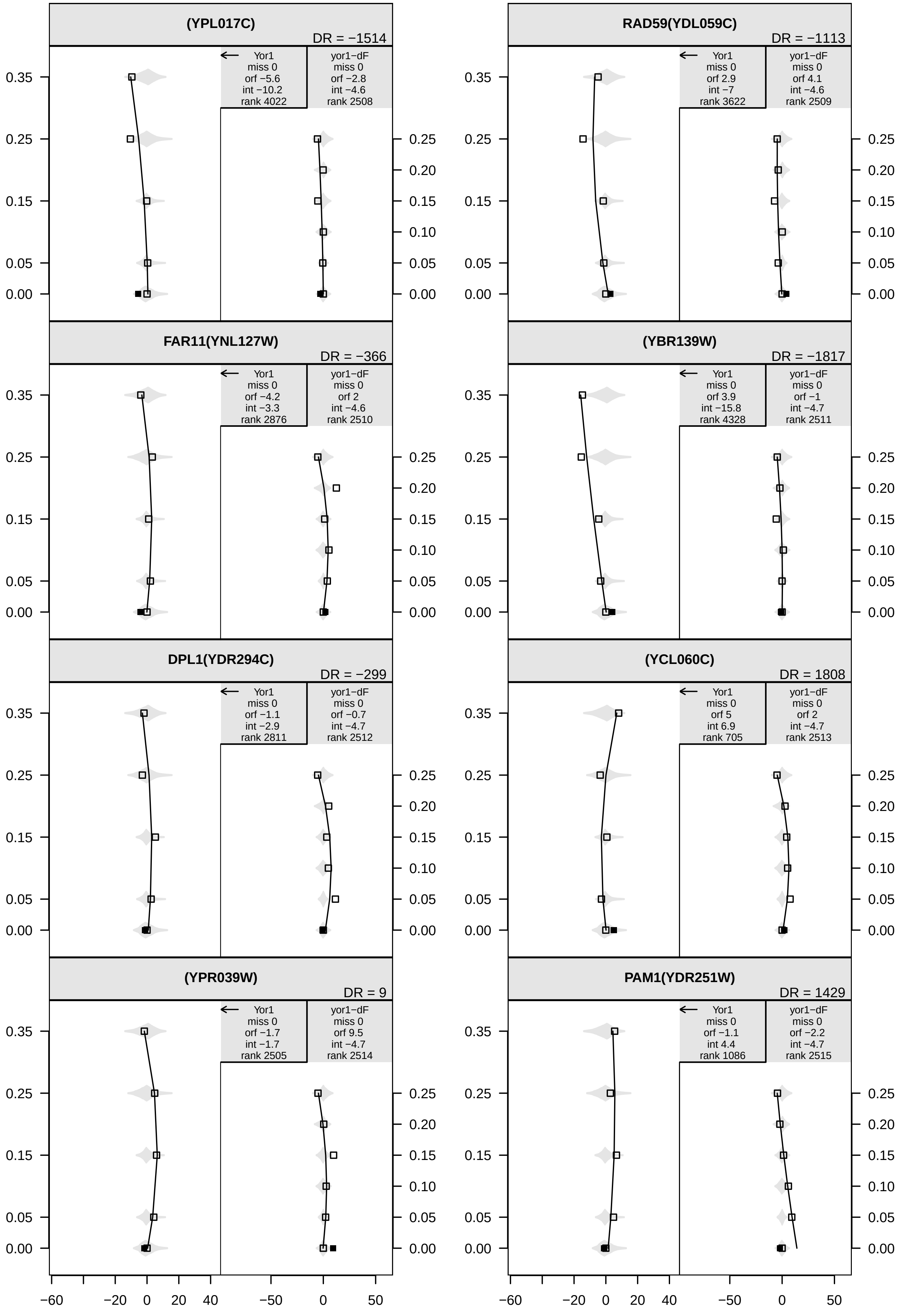
(YPL017C)
DR = −1514
Yor1
miss 0
orf −5.6
int −10.2
rank 4022
yor1−dF
miss 0
orf −2.8
int −4.6
rank 2508
RAD59(YDL059C)
DR = −1113
Yor1
miss 0
orf 2.9
int −7
rank 3622
yor1−dF
miss 0
orf 4.1
int −4.6
rank 2509
FAR11(YNL127W)
DR = −366
Yor1
miss 0
orf −4.2
int −3.3
rank 2876
yor1−dF
miss 0
orf 2
int −4.6
rank 2510
(YBR139W)
DR = −1817
Yor1
miss 0
orf 3.9
int −15.8
rank 4328
yor1−dF
miss 0
orf −1
int −4.7
rank 2511
DPL1(YDR294C)
DR = −299
Yor1
miss 0
orf −1.1
int −2.9
rank 2811
yor1−dF
miss 0
orf −0.7
int −4.7
rank 2512
(YCL060C)
DR = 1808
Yor1
miss 0
orf 5
int 6.9
rank 705
yor1−dF
miss 0
orf 2
int −4.7
rank 2513
(YPR039W)
DR = 9
Yor1
miss 0
orf −1.7
int −1.7
rank 2505
yor1−dF
miss 0
orf 9.5
int −4.7
rank 2514
PAM1(YDR251W)
DR = 1429
Yor1
miss 0
orf −1.1
int 4.4
rank 1086
yor1−dF
miss 0
orf −2.2
int −4.7
rank 2515
0.35
0.25
0.20
0.15
0.10
0.05
0.00
−60
−20
0
20
40
−50
0
50

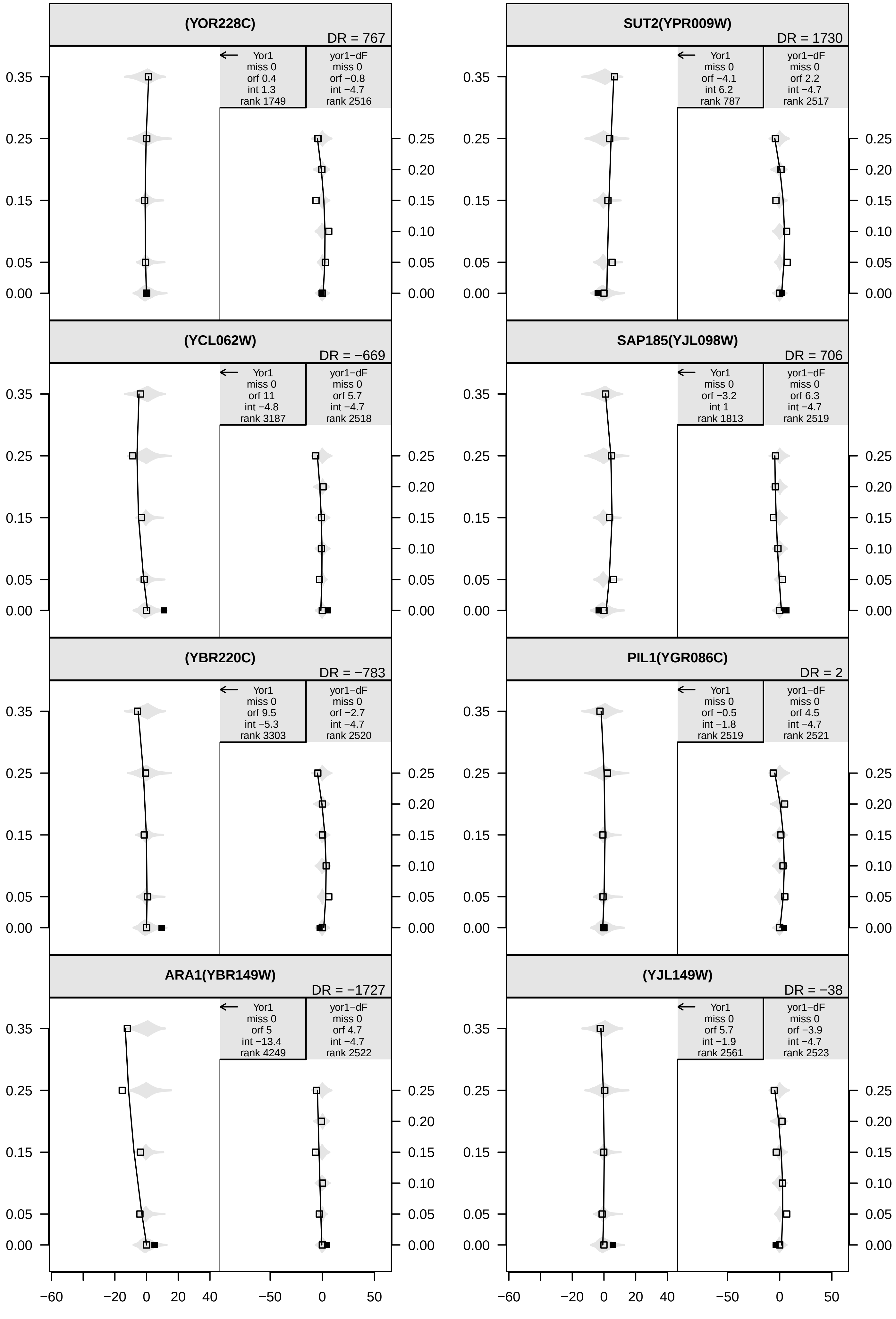
(YOR228C)
DR = 767
Yor1 miss 0 orf 0.4 int 1.3 rank 1749
yor1−dF miss 0 orf −0.8 int −4.7 rank 2516
SUT2(YPR009W)
DR = 1730
Yor1 miss 0 orf −4.1 int 6.2 rank 787
yor1−dF miss 0 orf 2.2 int −4.7 rank 2517
(YCL062W)
DR = −669
Yor1 miss 0 orf 11 int −4.8 rank 3187
yor1−dF miss 0 orf 5.7 int −4.7 rank 2518
SAP185(YJL098W)
DR = 706
Yor1 miss 0 orf −3.2 int 1 rank 1813
yor1−dF miss 0 orf 6.3 int −4.7 rank 2519
(YBR220C)
DR = −783
Yor1 miss 0 orf 9.5 int −5.3 rank 3303
yor1−dF miss 0 orf −2.7 int −4.7 rank 2520
PIL1(YGR086C)
DR = 2
Yor1 miss 0 orf −0.5 int −1.8 rank 2519
yor1−dF miss 0 orf 4.5 int −4.7 rank 2521
ARA1(YBR149W)
DR = −1727
Yor1 miss 0 orf 5 int −13.4 rank 4249
yor1−dF miss 0 orf 4.7 int −4.7 rank 2522
(YJL149W)
DR = −38
Yor1 miss 0 orf 5.7 int −1.9 rank 2561
yor1−dF miss 0 orf −3.9 int −4.7 rank 2523
0.35 0.25 0.20 0.15 0.10 0.05 0.00
−60 −20 0 20 40 −50 0 50

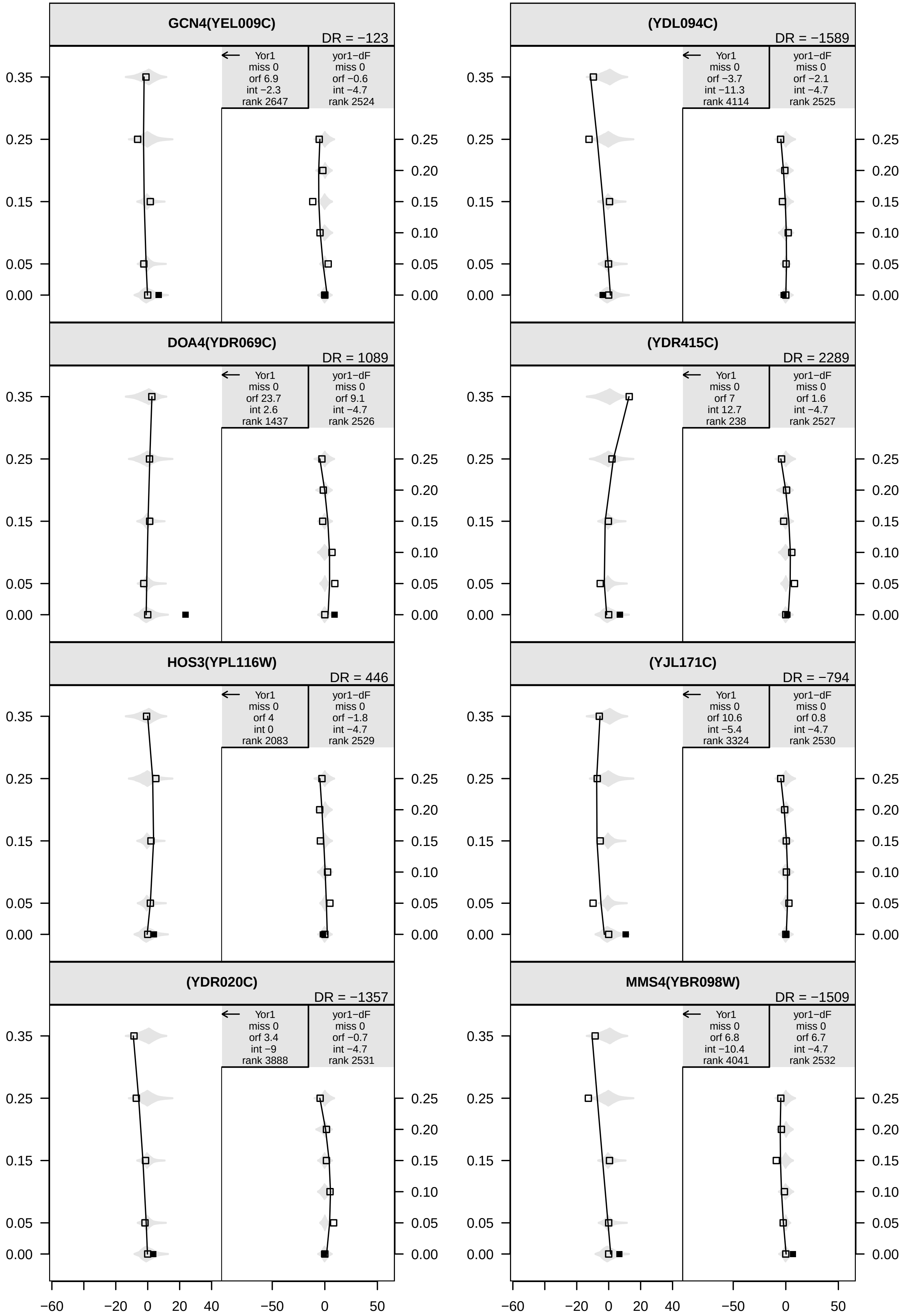
GCN4(YEL009C)
DR = −123
Yor1
miss 0
orf 6.9
int −2.3
rank 2647
yor1−dF
miss 0
orf −0.6
int −4.7
rank 2524
(YDL094C)
DR = −1589
Yor1
miss 0
orf −3.7
int −11.3
rank 4114
yor1−dF
miss 0
orf −2.1
int −4.7
rank 2525
DOA4(YDR069C)
DR = 1089
Yor1
miss 0
orf 23.7
int 2.6
rank 1437
yor1−dF
miss 0
orf 9.1
int −4.7
rank 2526
(YDR415C)
DR = 2289
Yor1
miss 0
orf 7
int 12.7
rank 238
yor1−dF
miss 0
orf 1.6
int −4.7
rank 2527
HOS3(YPL116W)
DR = 446
Yor1
miss 0
orf 4
int 0
rank 2083
yor1−dF
miss 0
orf −1.8
int −4.7
rank 2529
(YJL171C)
DR = −794
Yor1
miss 0
orf 10.6
int −5.4
rank 3324
yor1−dF
miss 0
orf 0.8
int −4.7
rank 2530
(YDR020C)
DR = −1357
Yor1
miss 0
orf 3.4
int −9
rank 3888
yor1−dF
miss 0
orf −0.7
int −4.7
rank 2531
MMS4(YBR098W)
DR = −1509
Yor1
miss 0
orf 6.8
int −10.4
rank 4041
yor1−dF
miss 0
orf 6.7
int −4.7
rank 2532
0.35
0.25
0.20
0.15
0.10
0.05
0.00
−60
−20
0
20
40
−50
0
50

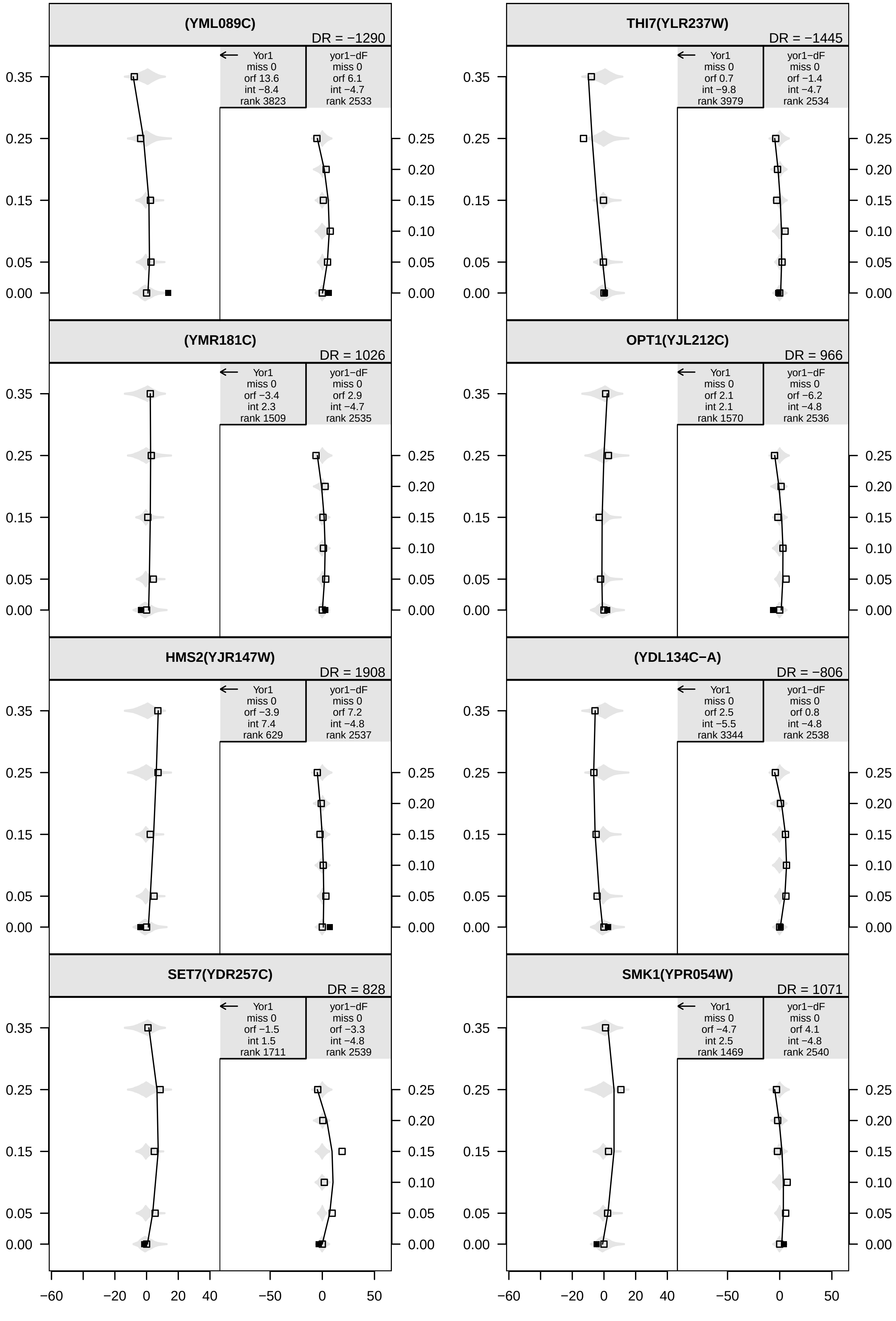
(YML089C)
DR = −1290
Yor1 miss 0 orf 13.6 int −8.4 rank 3823
yor1−dF miss 0 orf 6.1 int −4.7 rank 2533
THI7(YLR237W)
DR = −1445
Yor1 miss 0 orf 0.7 int −9.8 rank 3979
yor1−dF miss 0 orf −1.4 int −4.7 rank 2534
(YMR181C)
DR = 1026
Yor1 miss 0 orf −3.4 int 2.3 rank 1509
yor1−dF miss 0 orf 2.9 int −4.7 rank 2535
OPT1(YJL212C)
DR = 966
Yor1 miss 0 orf 2.1 int 2.1 rank 1570
yor1−dF miss 0 orf −6.2 int −4.8 rank 2536
HMS2(YJR147W)
DR = 1908
Yor1 miss 0 orf −3.9 int 7.4 rank 629
yor1−dF miss 0 orf 7.2 int −4.8 rank 2537
(YDL134C−A)
DR = −806
Yor1 miss 0 orf 2.5 int −5.5 rank 3344
yor1−dF miss 0 orf 0.8 int −4.8 rank 2538
SET7(YDR257C)
DR = 828
Yor1 miss 0 orf −1.5 int 1.5 rank 1711
yor1−dF miss 0 orf −3.3 int −4.8 rank 2539
SMK1(YPR054W)
DR = 1071
Yor1 miss 0 orf −4.7 int 2.5 rank 1469
yor1−dF miss 0 orf 4.1 int −4.8 rank 2540

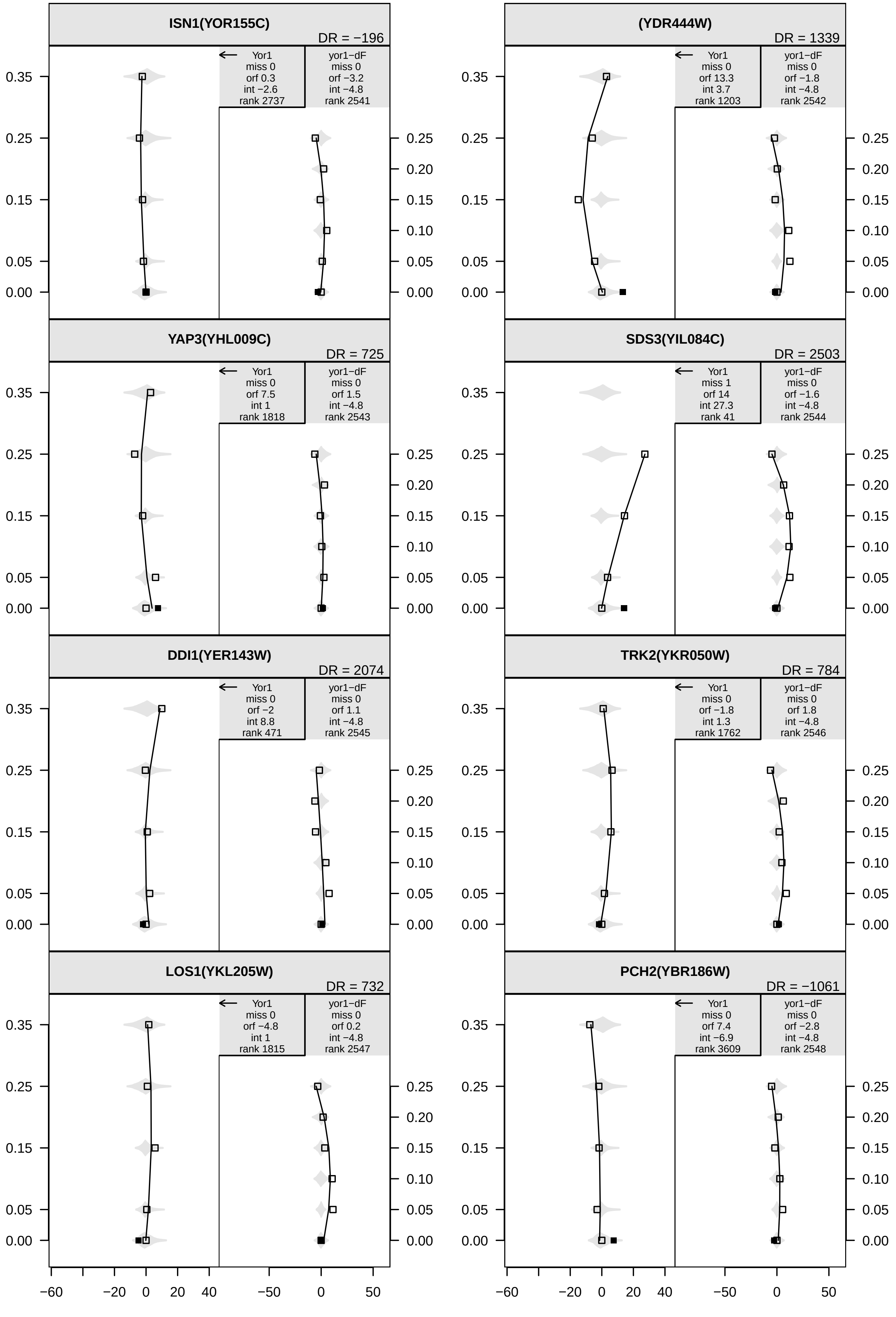

ISN1(YOR155C)
DR = −196
Yor1 miss 0 orf 0.3 int −2.6 rank 2737
yor1−dF miss 0 orf −3.2 int −4.8 rank 2541
(YDR444W)
DR = 1339
Yor1 miss 0 orf 13.3 int 3.7 rank 1203
yor1−dF miss 0 orf −1.8 int −4.8 rank 2542
YAP3(YHL009C)
DR = 725
Yor1 miss 0 orf 7.5 int 1 rank 1818
yor1−dF miss 0 orf 1.5 int −4.8 rank 2543
SDS3(YIL084C)
DR = 2503
Yor1 miss 1 orf 14 int 27.3 rank 41
yor1−dF miss 0 orf −1.6 int −4.8 rank 2544
DDI1(YER143W)
DR = 2074
Yor1 miss 0 orf −2 int 8.8 rank 471
yor1−dF miss 0 orf 1.1 int −4.8 rank 2545
TRK2(YKR050W)
DR = 784
Yor1 miss 0 orf −1.8 int 1.3 rank 1762
yor1−dF miss 0 orf 1.8 int −4.8 rank 2546
LOS1(YKL205W)
DR = 732
Yor1 miss 0 orf −4.8 int 1 rank 1815
yor1−dF miss 0 orf 0.2 int −4.8 rank 2547
PCH2(YBR186W)
DR = −1061
Yor1 miss 0 orf 7.4 int −6.9 rank 3609
yor1−dF miss 0 orf −2.8 int −4.8 rank 2548
0.35
0.25
0.20
0.15
0.10
0.05
0.00
−60
−20
0
20
40
−50
0
50

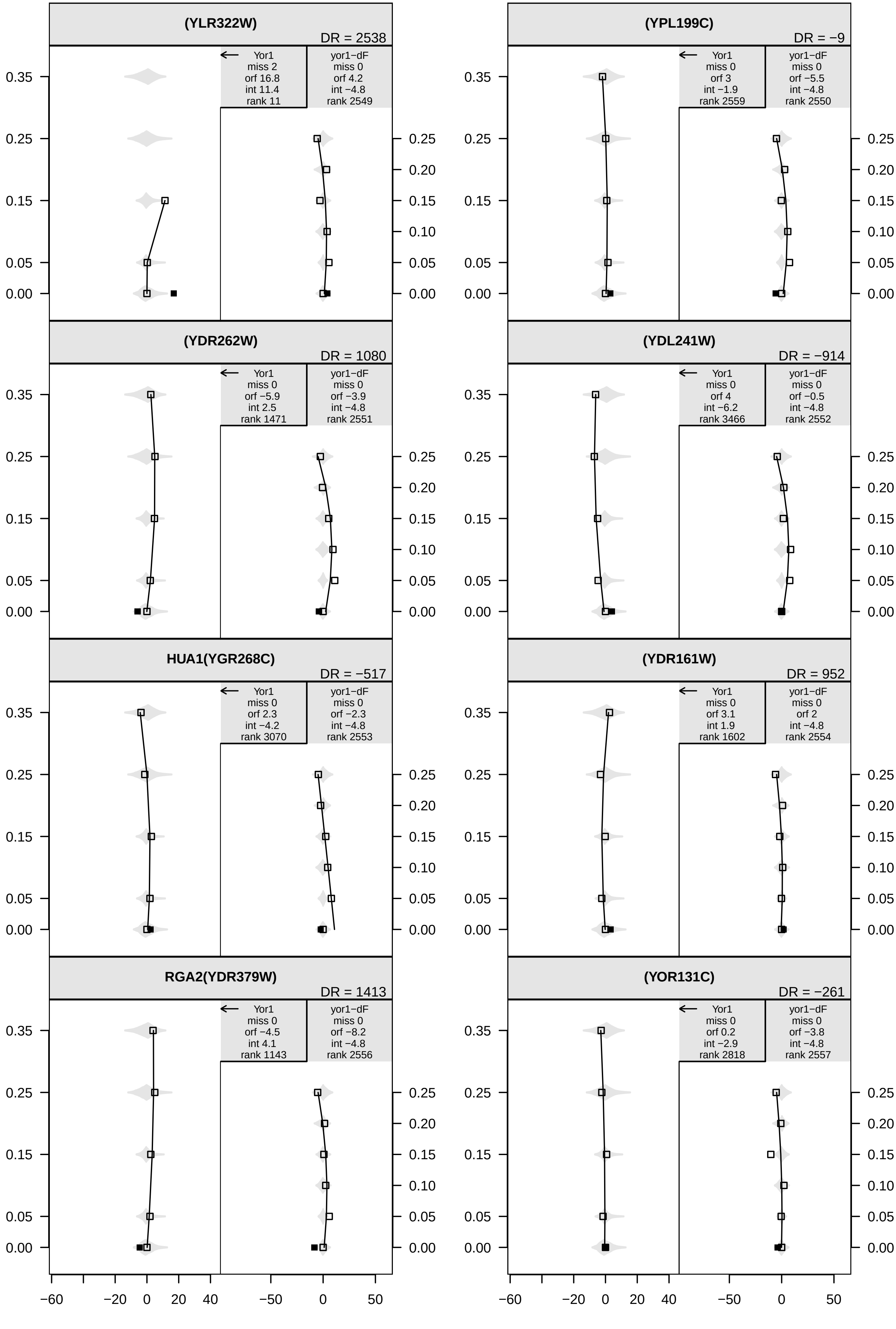

(YLR322W)
DR = 2538
Yor1
miss 2
orf 16.8
int 11.4
rank 11
yor1−dF
miss 0
orf 4.2
int −4.8
rank 2549
(YPL199C)
DR = −9
Yor1
miss 0
orf 3
int −1.9
rank 2559
yor1−dF
miss 0
orf −5.5
int −4.8
rank 2550
(YDR262W)
DR = 1080
Yor1
miss 0
orf −5.9
int 2.5
rank 1471
yor1−dF
miss 0
orf −3.9
int −4.8
rank 2551
(YDL241W)
DR = −914
Yor1
miss 0
orf 4
int −6.2
rank 3466
yor1−dF
miss 0
orf −0.5
int −4.8
rank 2552
HUA1(YGR268C)
DR = −517
Yor1
miss 0
orf 2.3
int −4.2
rank 3070
yor1−dF
miss 0
orf −2.3
int −4.8
rank 2553
(YDR161W)
DR = 952
Yor1
miss 0
orf 3.1
int 1.9
rank 1602
yor1−dF
miss 0
orf 2
int −4.8
rank 2554
RGA2(YDR379W)
DR = 1413
Yor1
miss 0
orf −4.5
int 4.1
rank 1143
yor1−dF
miss 0
orf −8.2
int −4.8
rank 2556
(YOR131C)
DR = −261
Yor1
miss 0
orf 0.2
int −2.9
rank 2818
yor1−dF
miss 0
orf −3.8
int −4.8
rank 2557
0.35
0.25
0.20
0.15
0.10
0.05
0.00
−60
−20
0
20
40
−50
0
50

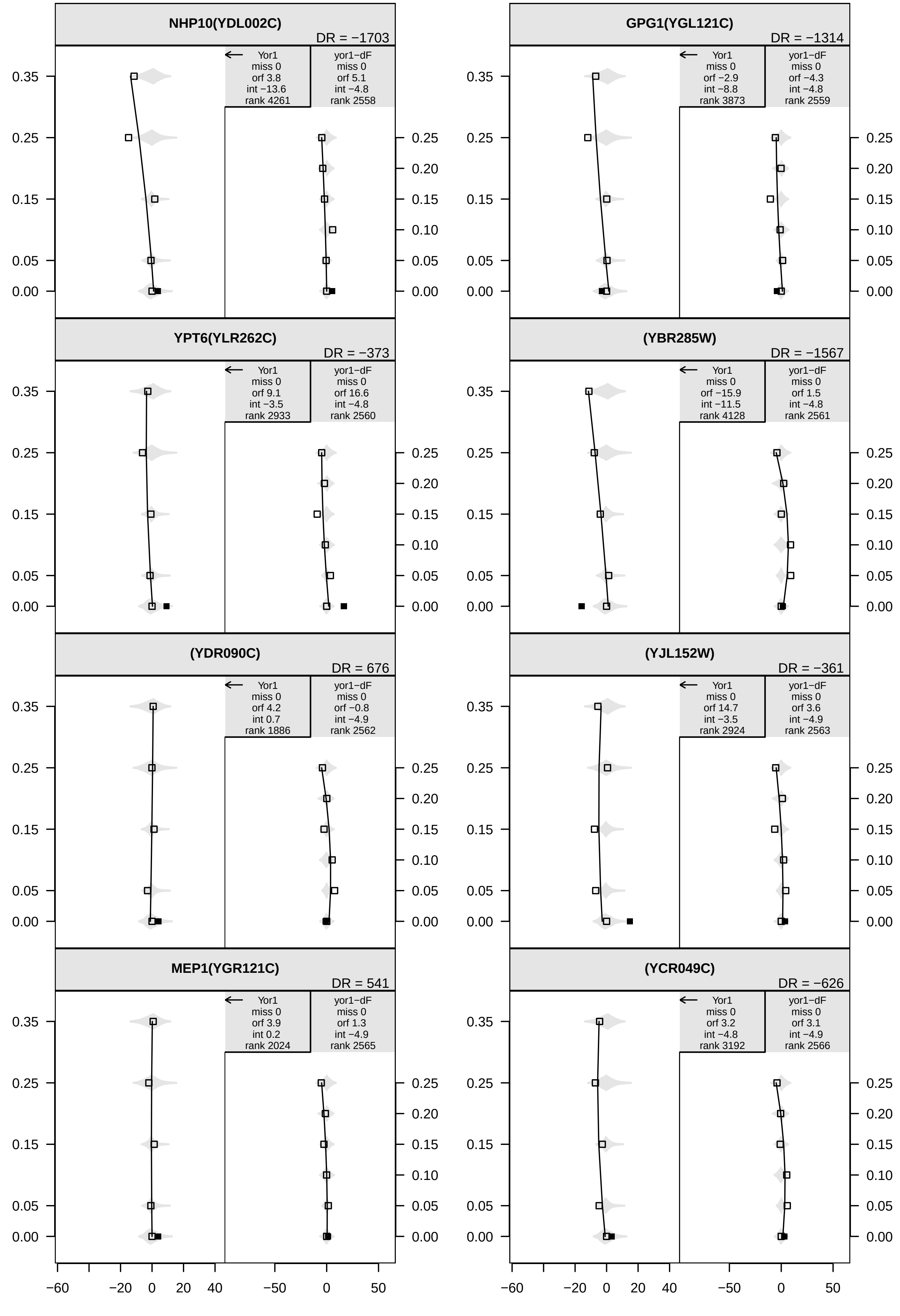

NHP10(YDL002C)
DR = −1703
Yor1 miss 0 orf 3.8 int −13.6 rank 4261
yor1−dF miss 0 orf 5.1 int −4.8 rank 2558
GPG1(YGL121C)
DR = −1314
Yor1 miss 0 orf −2.9 int −8.8 rank 3873
yor1−dF miss 0 orf −4.3 int −4.8 rank 2559
YPT6(YLR262C)
DR = −373
Yor1 miss 0 orf 9.1 int −3.5 rank 2933
yor1−dF miss 0 orf 16.6 int −4.8 rank 2560
(YBR285W)
DR = −1567
Yor1 miss 0 orf −15.9 int −11.5 rank 4128
yor1−dF miss 0 orf 1.5 int −4.8 rank 2561
(YDR090C)
DR = 676
Yor1 miss 0 orf 4.2 int 0.7 rank 1886
yor1−dF miss 0 orf −0.8 int −4.9 rank 2562
(YJL152W)
DR = −361
Yor1 miss 0 orf 14.7 int −3.5 rank 2924
yor1−dF miss 0 orf 3.6 int −4.9 rank 2563
MEP1(YGR121C)
DR = 541
Yor1 miss 0 orf 3.9 int 0.2 rank 2024
yor1−dF miss 0 orf 1.3 int −4.9 rank 2565
(YCR049C)
DR = −626
Yor1 miss 0 orf 3.2 int −4.8 rank 3192
yor1−dF miss 0 orf 3.1 int −4.9 rank 2566

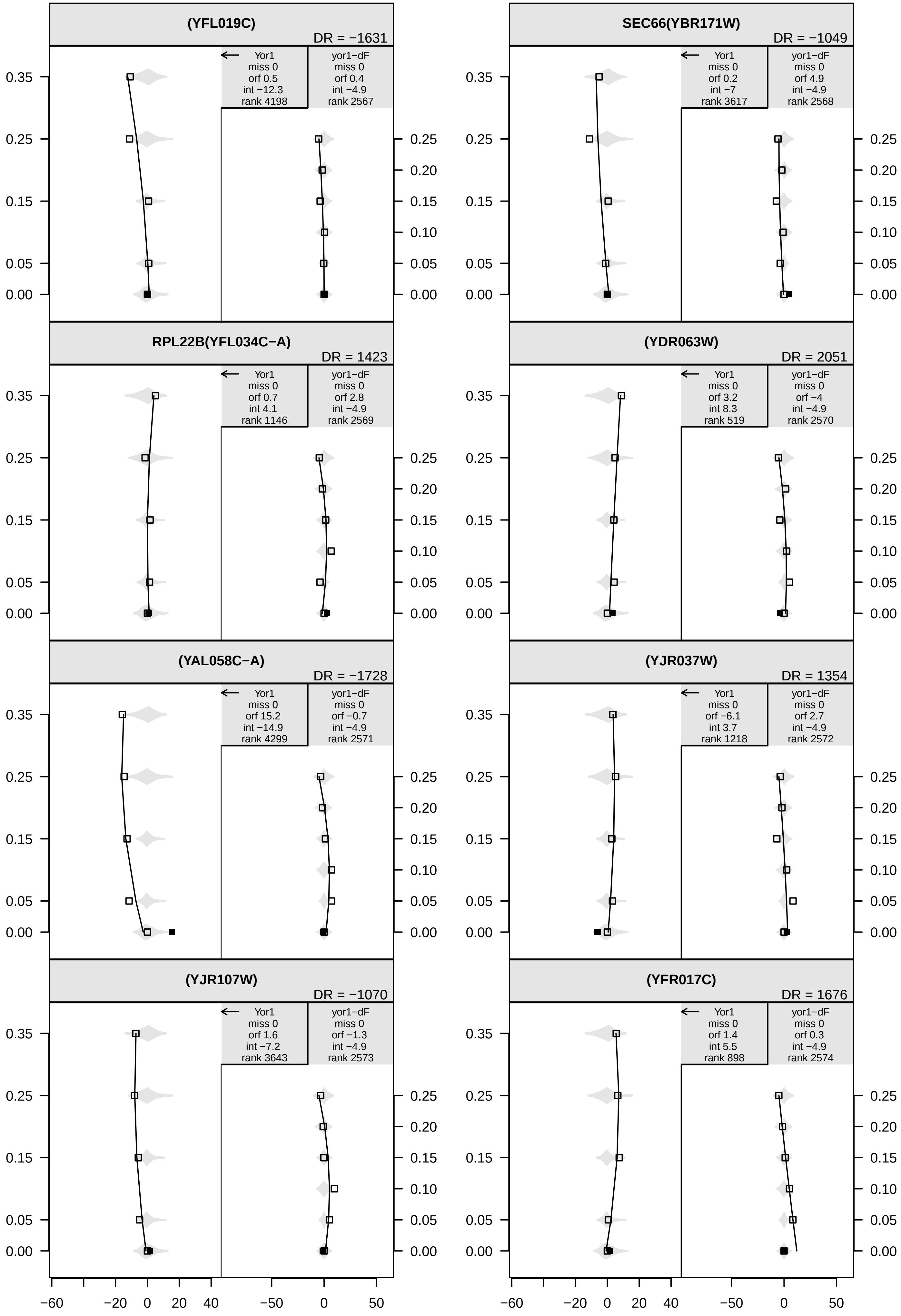
(YFL019C)
DR = −1631
Yor1
miss 0
orf 0.5
int −12.3
rank 4198
yor1−dF
miss 0
orf 0.4
int −4.9
rank 2567
SEC66(YBR171W)
DR = −1049
Yor1
miss 0
orf 0.2
int −7
rank 3617
yor1−dF
miss 0
orf 4.9
int −4.9
rank 2568
RPL22B(YFL034C−A)
DR = 1423
Yor1
miss 0
orf 0.7
int 4.1
rank 1146
yor1−dF
miss 0
orf 2.8
int −4.9
rank 2569
(YDR063W)
DR = 2051
Yor1
miss 0
orf 3.2
int 8.3
rank 519
yor1−dF
miss 0
orf −4
int −4.9
rank 2570
(YAL058C−A)
DR = −1728
Yor1
miss 0
orf 15.2
int −14.9
rank 4299
yor1−dF
miss 0
orf −0.7
int −4.9
rank 2571
(YJR037W)
DR = 1354
Yor1
miss 0
orf −6.1
int 3.7
rank 1218
yor1−dF
miss 0
orf 2.7
int −4.9
rank 2572
(YJR107W)
DR = −1070
Yor1
miss 0
orf 1.6
int −7.2
rank 3643
yor1−dF
miss 0
orf −1.3
int −4.9
rank 2573
(YFR017C)
DR = 1676
Yor1
miss 0
orf 1.4
int 5.5
rank 898
yor1−dF
miss 0
orf 0.3
int −4.9
rank 2574
0.35
0.25
0.20
0.15
0.10
0.05
0.00
−60
−20
0
20
40
−50
0
50

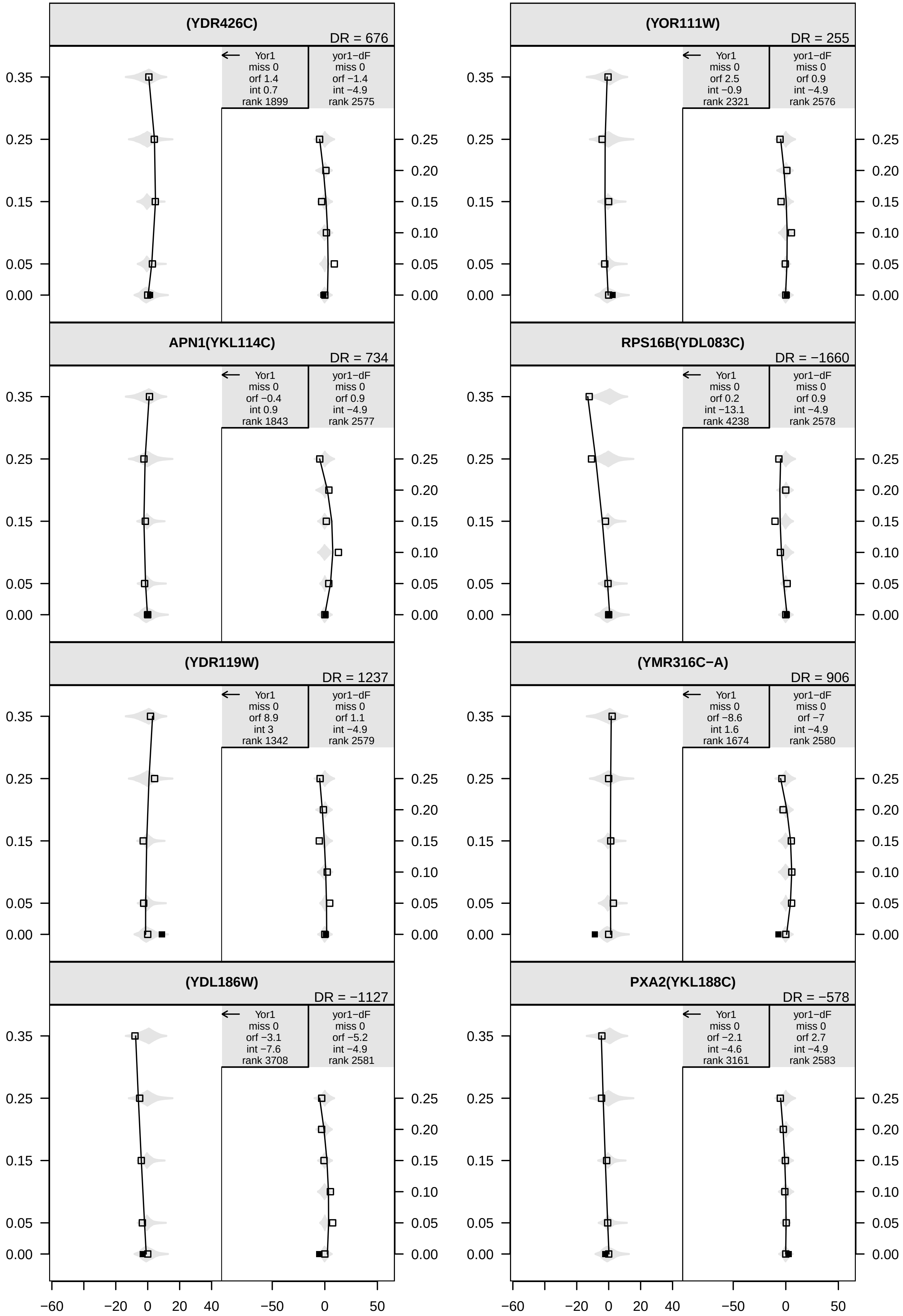
(YDR426C)
DR = 676
Yor1 miss 0 orf 1.4 int 0.7 rank 1899
yor1−dF miss 0 orf −1.4 int −4.9 rank 2575
(YOR111W)
DR = 255
Yor1 miss 0 orf 2.5 int −0.9 rank 2321
yor1−dF miss 0 orf 0.9 int −4.9 rank 2576
APN1(YKL114C)
DR = 734
Yor1 miss 0 orf −0.4 int 0.9 rank 1843
yor1−dF miss 0 orf 0.9 int −4.9 rank 2577
RPS16B(YDL083C)
DR = −1660
Yor1 miss 0 orf 0.2 int −13.1 rank 4238
yor1−dF miss 0 orf 0.9 int −4.9 rank 2578
(YDR119W)
DR = 1237
Yor1 miss 0 orf 8.9 int 3 rank 1342
yor1−dF miss 0 orf 1.1 int −4.9 rank 2579
(YMR316C−A)
DR = 906
Yor1 miss 0 orf −8.6 int 1.6 rank 1674
yor1−dF miss 0 orf −7 int −4.9 rank 2580
(YDL186W)
DR = −1127
Yor1 miss 0 orf −3.1 int −7.6 rank 3708
yor1−dF miss 0 orf −5.2 int −4.9 rank 2581
PXA2(YKL188C)
DR = −578
Yor1 miss 0 orf −2.1 int −4.6 rank 3161
yor1−dF miss 0 orf 2.7 int −4.9 rank 2583
0.35 0.25 0.15 0.05 0.00
0.25 0.20 0.15 0.10 0.05 0.00
−60 −20 0 20 40 −50 0 50

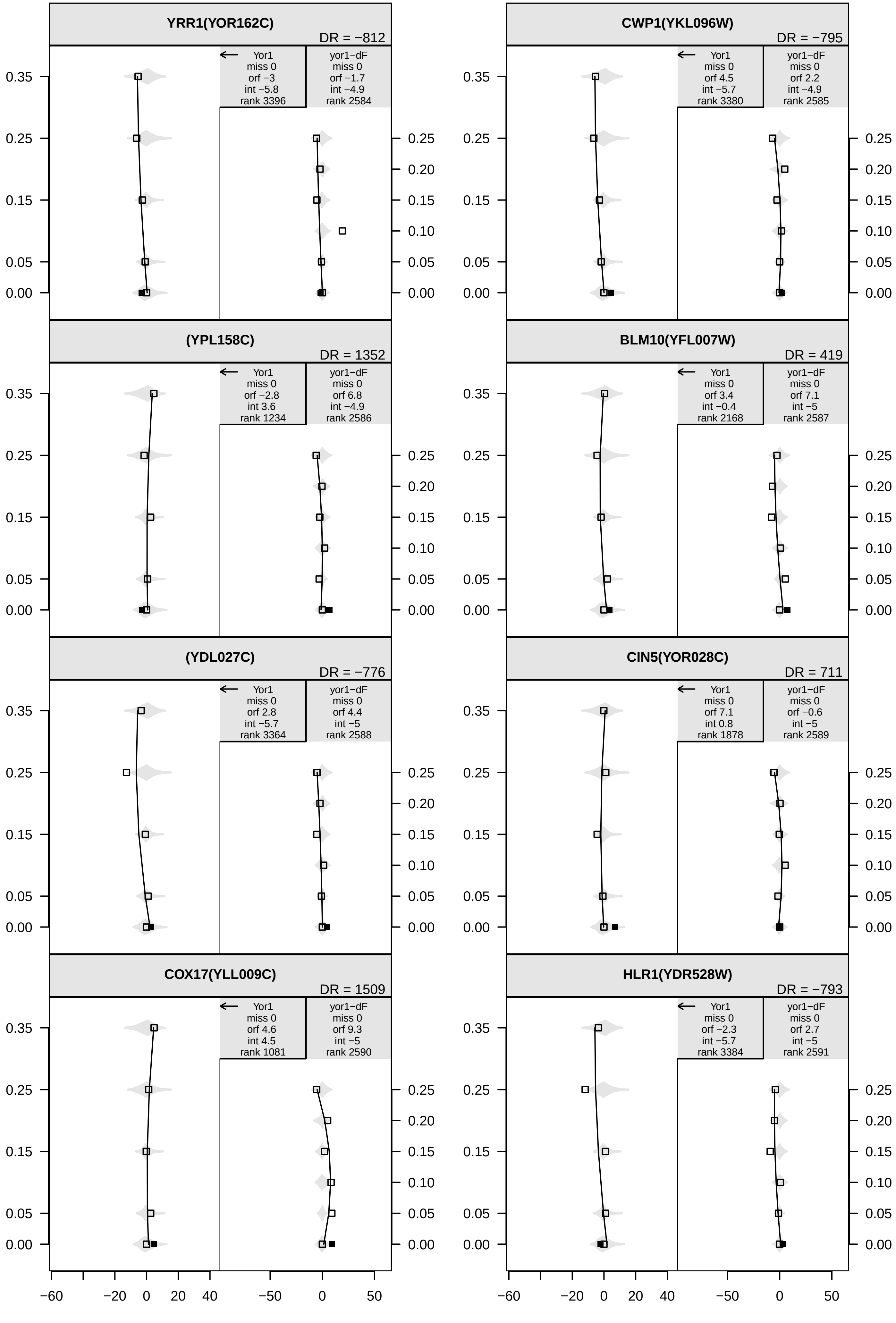

YRR1(YOR162C)
DR = −812
Yor1 miss 0 orf −3 int −5.8 rank 3396
yor1−dF miss 0 orf −1.7 int −4.9 rank 2584
CWP1(YKL096W)
DR = −795
Yor1 miss 0 orf 4.5 int −5.7 rank 3380
yor1−dF miss 0 orf 2.2 int −4.9 rank 2585
(YPL158C)
DR = 1352
Yor1 miss 0 orf −2.8 int 3.6 rank 1234
yor1−dF miss 0 orf 6.8 int −4.9 rank 2586
BLM10(YFL007W)
DR = 419
Yor1 miss 0 orf 3.4 int −0.4 rank 2168
yor1−dF miss 0 orf 7.1 int −5 rank 2587
(YDL027C)
DR = −776
Yor1 miss 0 orf 2.8 int −5.7 rank 3364
yor1−dF miss 0 orf 4.4 int −5 rank 2588
CIN5(YOR028C)
DR = 711
Yor1 miss 0 orf 7.1 int 0.8 rank 1878
yor1−dF miss 0 orf −0.6 int −5 rank 2589
COX17(YLL009C)
DR = 1509
Yor1 miss 0 orf 4.6 int 4.5 rank 1081
yor1−dF miss 0 orf 9.3 int −5 rank 2590
HLR1(YDR528W)
DR = −793
Yor1 miss 0 orf −2.3 int −5.7 rank 3384
yor1−dF miss 0 orf 2.7 int −5 rank 2591
0.35
0.25
0.20
0.15
0.10
0.05
0.00
−60
−20
0
20
40
−50
0
50

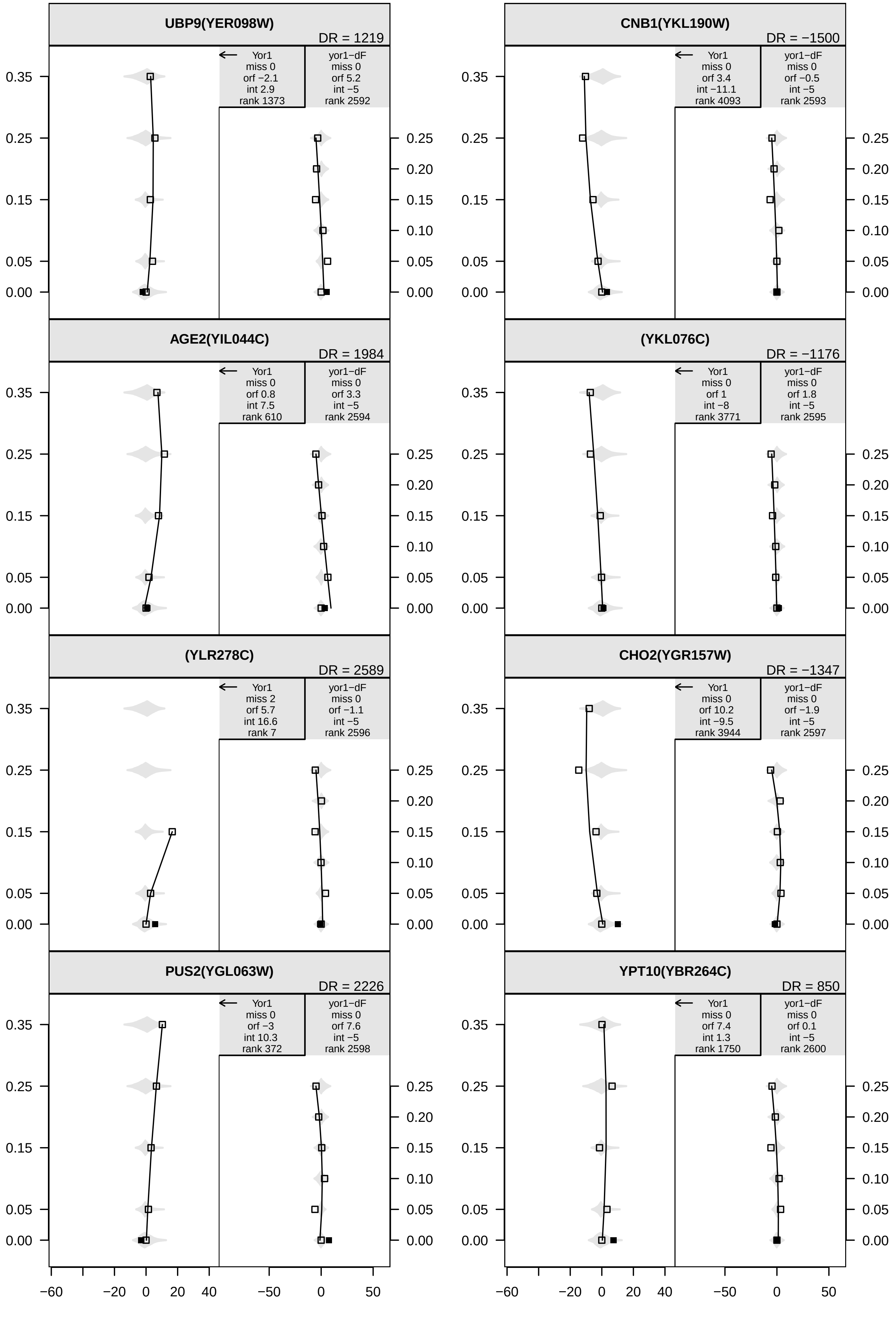
UBP9(YER098W)
DR = 1219
Yor1
miss 0
orf −2.1
int 2.9
rank 1373
yor1−dF
miss 0
orf 5.2
int −5
rank 2592
CNB1(YKL190W)
DR = −1500
Yor1
miss 0
orf 3.4
int −11.1
rank 4093
yor1−dF
miss 0
orf −0.5
int −5
rank 2593
AGE2(YIL044C)
DR = 1984
Yor1
miss 0
orf 0.8
int 7.5
rank 610
yor1−dF
miss 0
orf 3.3
int −5
rank 2594
(YKL076C)
DR = −1176
Yor1
miss 0
orf 1
int −8
rank 3771
yor1−dF
miss 0
orf 1.8
int −5
rank 2595
(YLR278C)
DR = 2589
Yor1
miss 2
orf 5.7
int 16.6
rank 7
yor1−dF
miss 0
orf −1.1
int −5
rank 2596
CHO2(YGR157W)
DR = −1347
Yor1
miss 0
orf 10.2
int −9.5
rank 3944
yor1−dF
miss 0
orf −1.9
int −5
rank 2597
PUS2(YGL063W)
DR = 2226
Yor1
miss 0
orf −3
int 10.3
rank 372
yor1−dF
miss 0
orf 7.6
int −5
rank 2598
YPT10(YBR264C)
DR = 850
Yor1
miss 0
orf 7.4
int 1.3
rank 1750
yor1−dF
miss 0
orf 0.1
int −5
rank 2600
0.35
0.25
0.20
0.15
0.10
0.05
0.00
−60
−20
0
20
40
−50
0
50

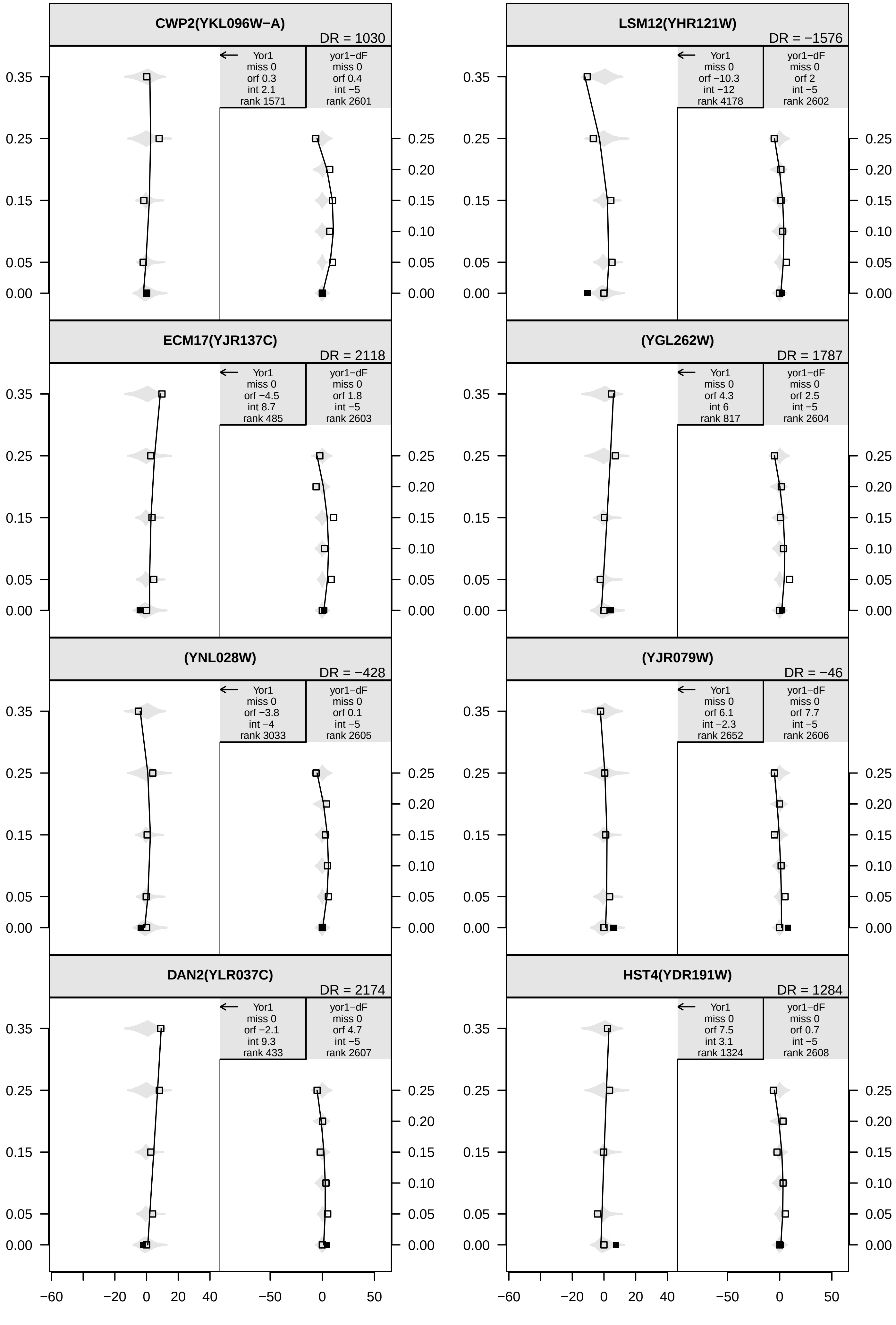
CWP2(YKL096W−A)
DR = 1030
Yor1 miss 0 orf 0.3 int 2.1 rank 1571
yor1−dF miss 0 orf 0.4 int −5 rank 2601
LSM12(YHR121W)
DR = −1576
Yor1 miss 0 orf −10.3 int −12 rank 4178
yor1−dF miss 0 orf 2 int −5 rank 2602
ECM17(YJR137C)
DR = 2118
Yor1 miss 0 orf −4.5 int 8.7 rank 485
yor1−dF miss 0 orf 1.8 int −5 rank 2603
(YGL262W)
DR = 1787
Yor1 miss 0 orf 4.3 int 6 rank 817
yor1−dF miss 0 orf 2.5 int −5 rank 2604
(YNL028W)
DR = −428
Yor1 miss 0 orf −3.8 int −4 rank 3033
yor1−dF miss 0 orf 0.1 int −5 rank 2605
(YJR079W)
DR = −46
Yor1 miss 0 orf 6.1 int −2.3 rank 2652
yor1−dF miss 0 orf 7.7 int −5 rank 2606
DAN2(YLR037C)
DR = 2174
Yor1 miss 0 orf −2.1 int 9.3 rank 433
yor1−dF miss 0 orf 4.7 int −5 rank 2607
HST4(YDR191W)
DR = 1284
Yor1 miss 0 orf 7.5 int 3.1 rank 1324
yor1−dF miss 0 orf 0.7 int −5 rank 2608

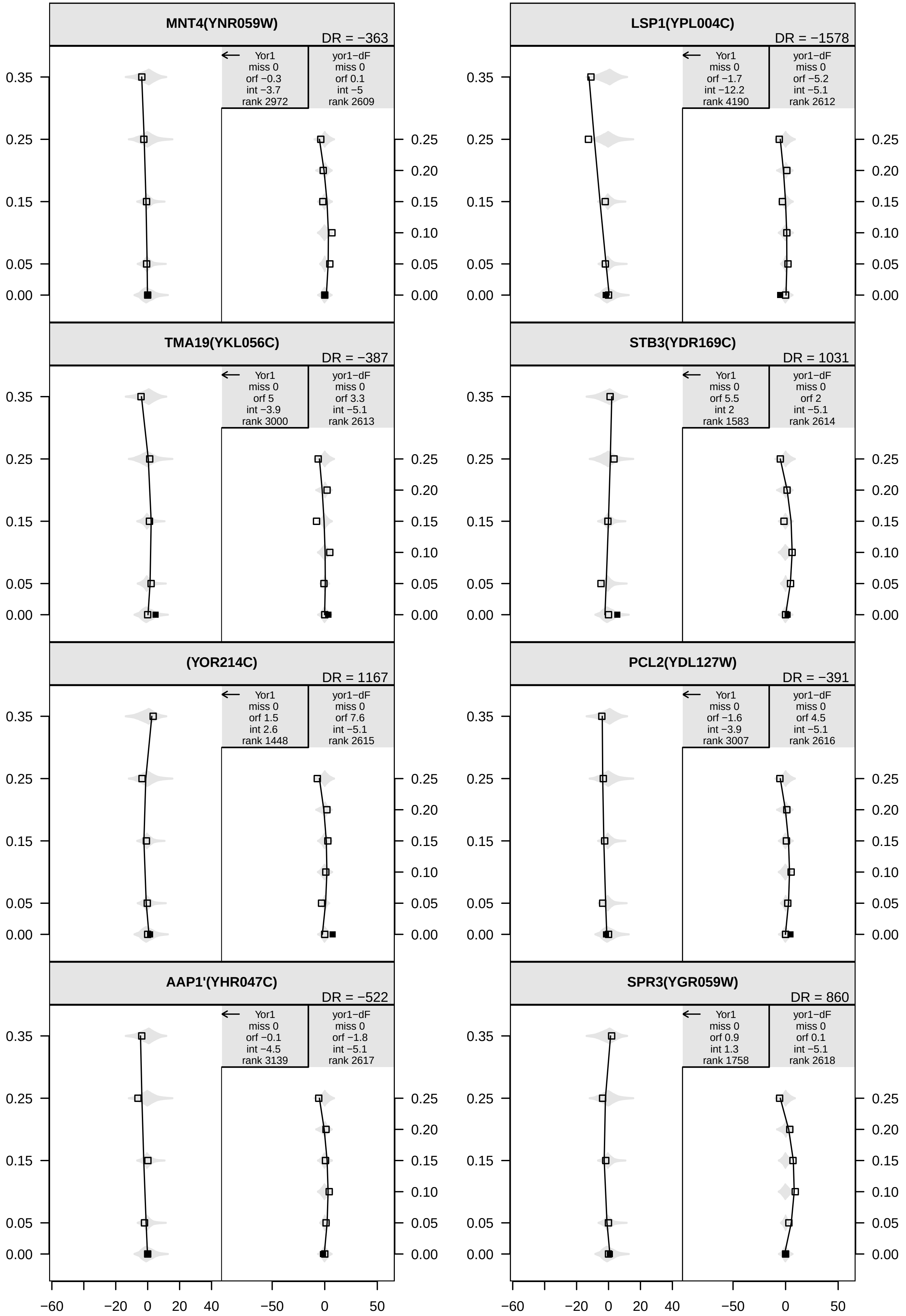
MNT4(YNR059W)
DR = −363
Yor1
miss 0
orf −0.3
int −3.7
rank 2972
yor1−dF
miss 0
orf 0.1
int −5
rank 2609
LSP1(YPL004C)
DR = −1578
Yor1
miss 0
orf −1.7
int −12.2
rank 4190
yor1−dF
miss 0
orf −5.2
int −5.1
rank 2612
TMA19(YKL056C)
DR = −387
Yor1
miss 0
orf 5
int −3.9
rank 3000
yor1−dF
miss 0
orf 3.3
int −5.1
rank 2613
STB3(YDR169C)
DR = 1031
Yor1
miss 0
orf 5.5
int 2
rank 1583
yor1−dF
miss 0
orf 2
int −5.1
rank 2614
(YOR214C)
DR = 1167
Yor1
miss 0
orf 1.5
int 2.6
rank 1448
yor1−dF
miss 0
orf 7.6
int −5.1
rank 2615
PCL2(YDL127W)
DR = −391
Yor1
miss 0
orf −1.6
int −3.9
rank 3007
yor1−dF
miss 0
orf 4.5
int −5.1
rank 2616
AAP1'(YHR047C)
DR = −522
Yor1
miss 0
orf −0.1
int −4.5
rank 3139
yor1−dF
miss 0
orf −1.8
int −5.1
rank 2617
SPR3(YGR059W)
DR = 860
Yor1
miss 0
orf 0.9
int 1.3
rank 1758
yor1−dF
miss 0
orf 0.1
int −5.1
rank 2618
0.35
0.25
0.20
0.15
0.10
0.05
0.00
−60
−20
0
20
40
−50
0
50

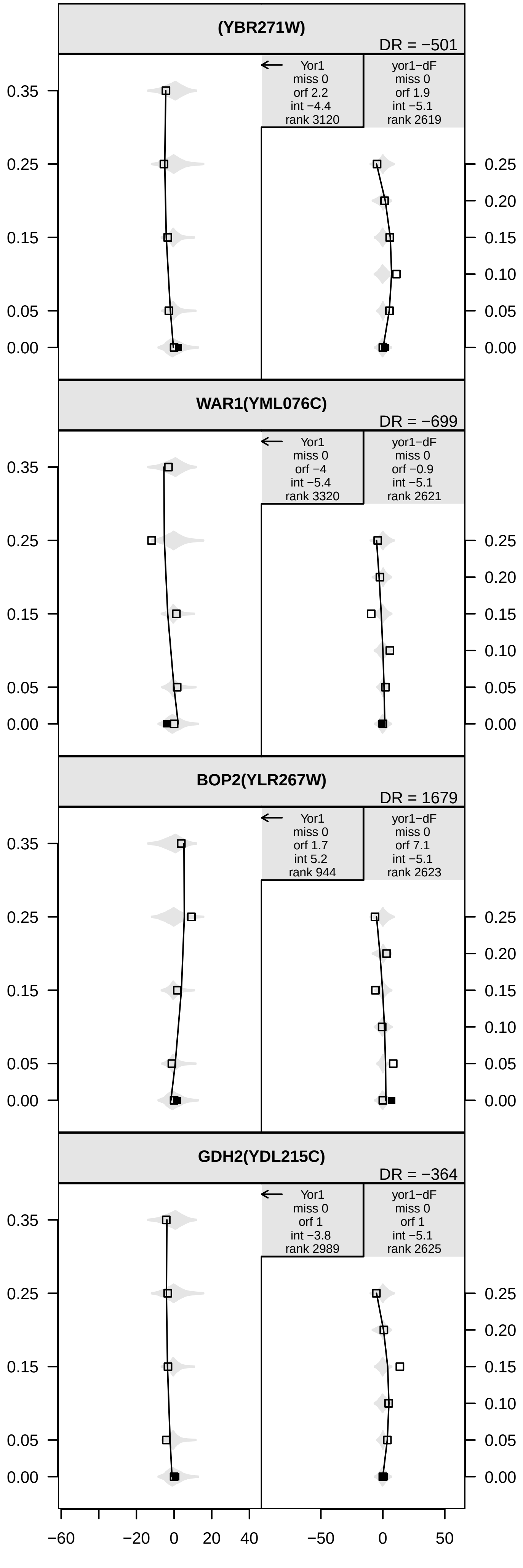

(YBR271W)
DR = −501
Yor1
miss 0
orf 2.2
int −4.4
rank 3120
yor1−dF
miss 0
orf 1.9
int −5.1
rank 2619
CAF16(YFL028C)
DR = −699
Yor1
miss 0
orf 4.9
int −5.4
rank 3319
yor1−dF
miss 0
orf 13.2
int −5.1
rank 2620
WAR1(YML076C)
DR = −699
Yor1
miss 0
orf −4
int −5.4
rank 3320
yor1−dF
miss 0
orf −0.9
int −5.1
rank 2621
GAL80(YML051W)
DR = 1639
Yor1
miss 0
orf 3.8
int 5
rank 983
yor1−dF
miss 0
orf 2.9
int −5.1
rank 2622
BOP2(YLR267W)
DR = 1679
Yor1
miss 0
orf 1.7
int 5.2
rank 944
yor1−dF
miss 0
orf 7.1
int −5.1
rank 2623
SPR28(YDR218C)
DR = −219
Yor1
miss 0
orf 8.7
int −3
rank 2843
yor1−dF
miss 0
orf −1.6
int −5.1
rank 2624
GDH2(YDL215C)
DR = −364
Yor1
miss 0
orf 1
int −3.8
rank 2989
yor1−dF
miss 0
orf 1
int −5.1
rank 2625
STE7(YDL159W)
DR = −342
Yor1
miss 0
orf 4.3
int −3.7
rank 2968
yor1−dF
miss 0
orf −1.1
int −5.1
rank 2626
0.35
0.25
0.20
0.15
0.10
0.05
0.00
−60
−20
0
20
40
−50
0
50

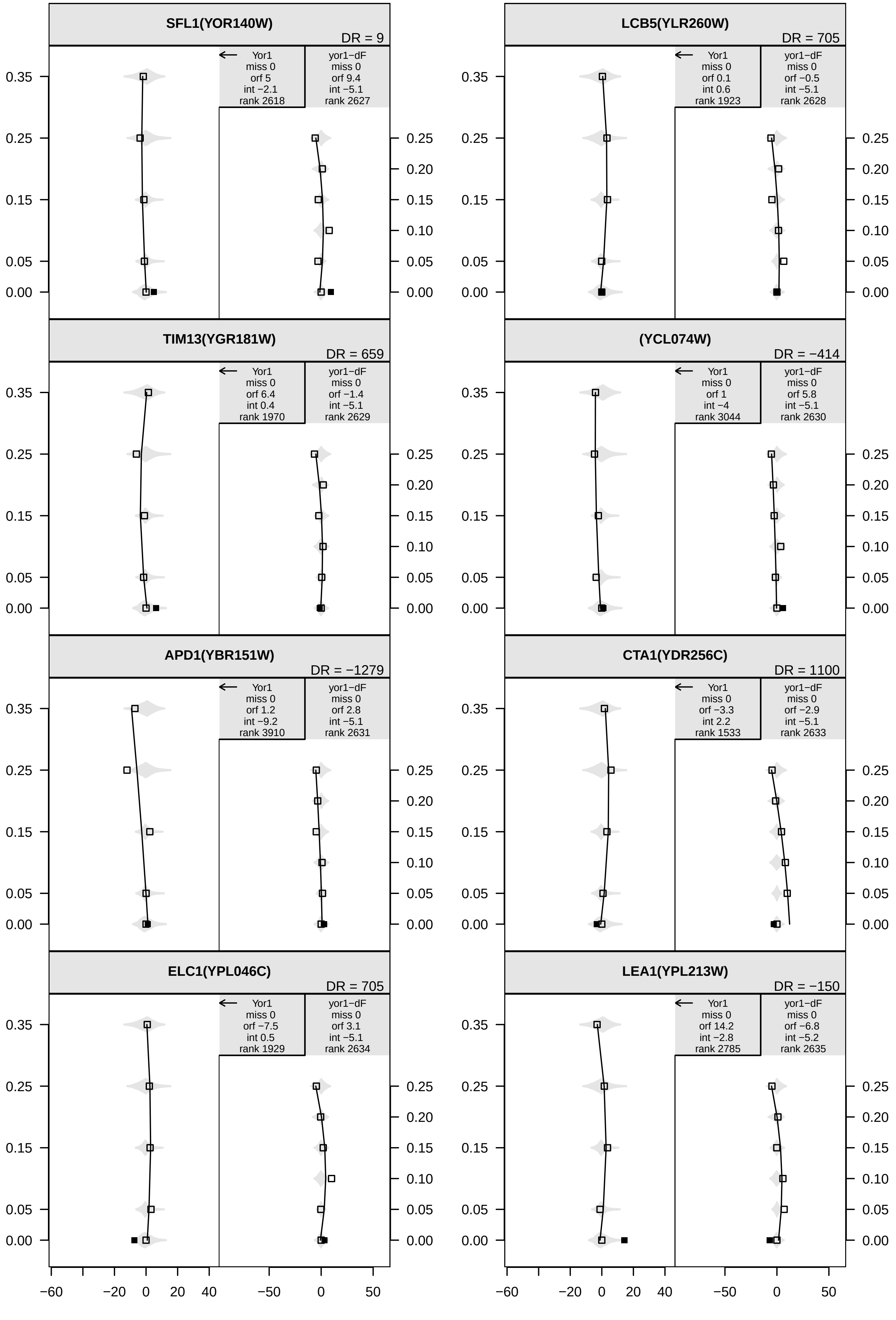
SFL1(YOR140W)
DR = 9
Yor1 miss 0 orf 5 int −2.1 rank 2618
yor1−dF miss 0 orf 9.4 int −5.1 rank 2627
LCB5(YLR260W)
DR = 705
Yor1 miss 0 orf 0.1 int 0.6 rank 1923
yor1−dF miss 0 orf −0.5 int −5.1 rank 2628
TIM13(YGR181W)
DR = 659
Yor1 miss 0 orf 6.4 int 0.4 rank 1970
yor1−dF miss 0 orf −1.4 int −5.1 rank 2629
(YCL074W)
DR = −414
Yor1 miss 0 orf 1 int −4 rank 3044
yor1−dF miss 0 orf 5.8 int −5.1 rank 2630
APD1(YBR151W)
DR = −1279
Yor1 miss 0 orf 1.2 int −9.2 rank 3910
yor1−dF miss 0 orf 2.8 int −5.1 rank 2631
CTA1(YDR256C)
DR = 1100
Yor1 miss 0 orf −3.3 int 2.2 rank 1533
yor1−dF miss 0 orf −2.9 int −5.1 rank 2633
ELC1(YPL046C)
DR = 705
Yor1 miss 0 orf −7.5 int 0.5 rank 1929
yor1−dF miss 0 orf 3.1 int −5.1 rank 2634
LEA1(YPL213W)
DR = −150
Yor1 miss 0 orf 14.2 int −2.8 rank 2785
yor1−dF miss 0 orf −6.8 int −5.2 rank 2635

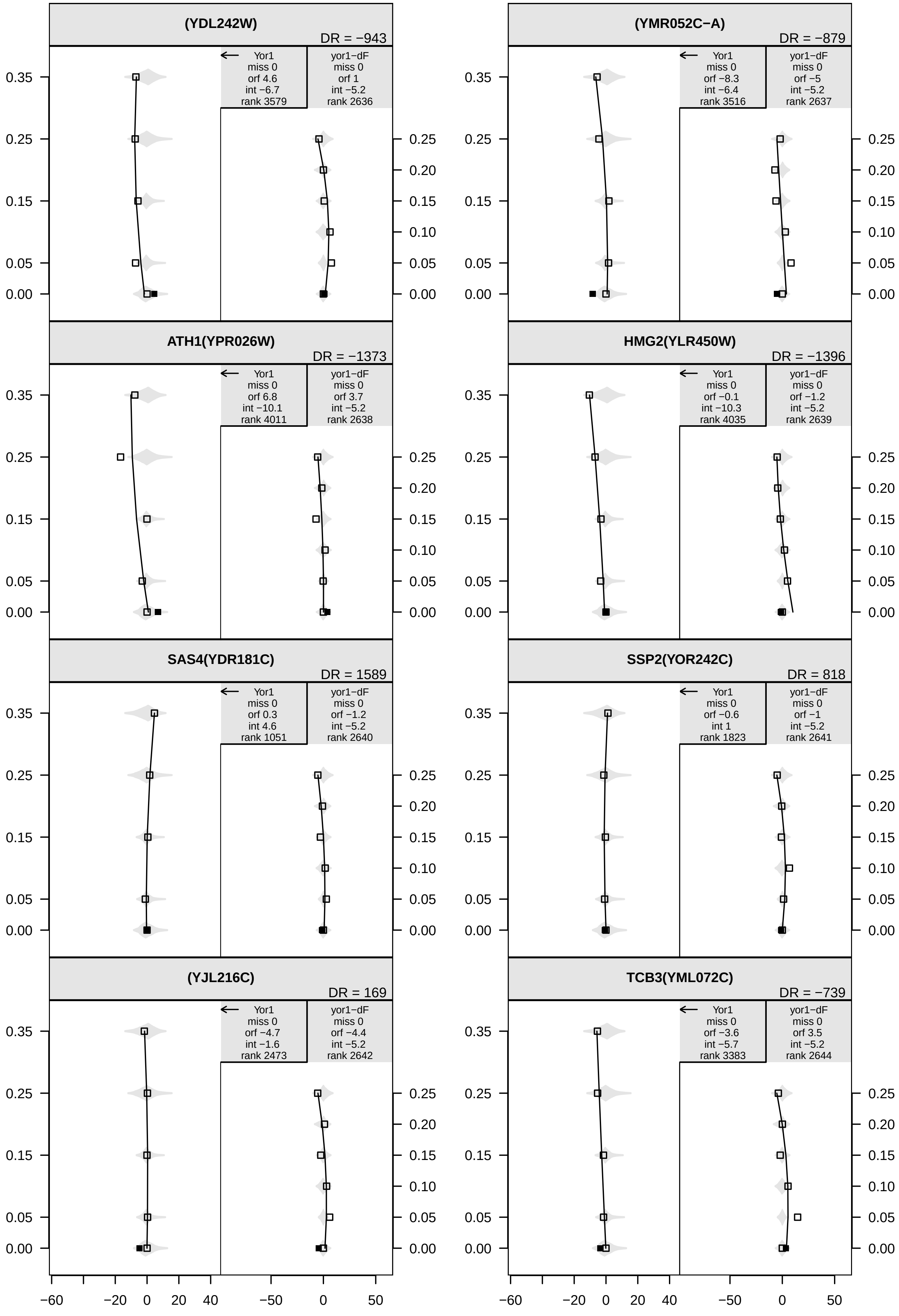

(YDL242W)
DR = −943
Yor1 miss 0 orf 4.6 int −6.7 rank 3579
yor1−dF miss 0 orf 1 int −5.2 rank 2636
(YMR052C−A)
DR = −879
Yor1 miss 0 orf −8.3 int −6.4 rank 3516
yor1−dF miss 0 orf −5 int −5.2 rank 2637
ATH1(YPR026W)
DR = −1373
Yor1 miss 0 orf 6.8 int −10.1 rank 4011
yor1−dF miss 0 orf 3.7 int −5.2 rank 2638
HMG2(YLR450W)
DR = −1396
Yor1 miss 0 orf −0.1 int −10.3 rank 4035
yor1−dF miss 0 orf −1.2 int −5.2 rank 2639
SAS4(YDR181C)
DR = 1589
Yor1 miss 0 orf 0.3 int 4.6 rank 1051
yor1−dF miss 0 orf −1.2 int −5.2 rank 2640
SSP2(YOR242C)
DR = 818
Yor1 miss 0 orf −0.6 int 1 rank 1823
yor1−dF miss 0 orf −1 int −5.2 rank 2641
(YJL216C)
DR = 169
Yor1 miss 0 orf −4.7 int −1.6 rank 2473
yor1−dF miss 0 orf −4.4 int −5.2 rank 2642
TCB3(YML072C)
DR = −739
Yor1 miss 0 orf −3.6 int −5.7 rank 3383
yor1−dF miss 0 orf 3.5 int −5.2 rank 2644
0.00 0.05 0.15 0.25 0.35
0.00 0.05 0.10 0.15 0.20 0.25
−60 −20 0 20 40
−50 0 50

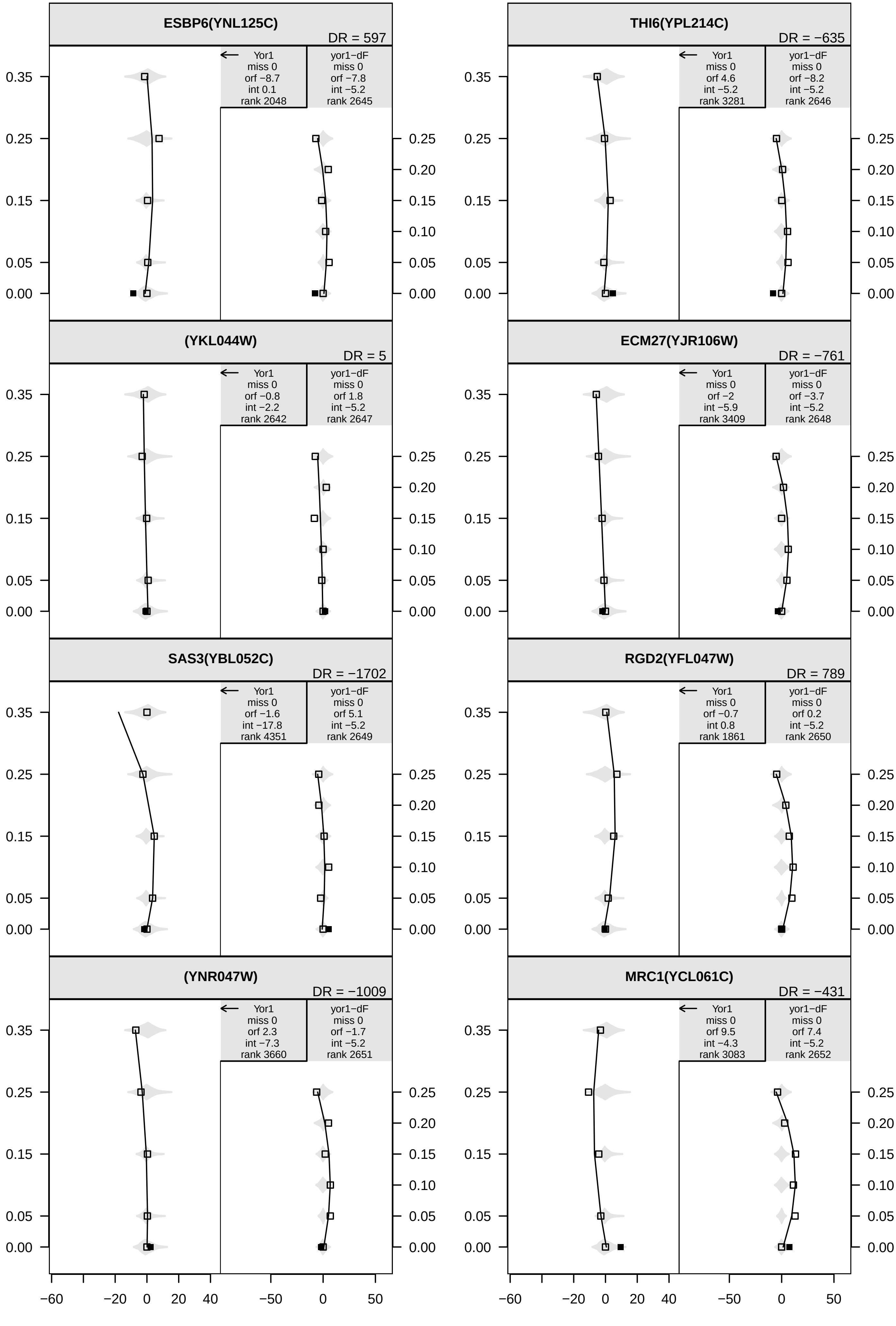

ESBP6(YNL125C)
DR = 597
Yor1
miss 0
orf −8.7
int 0.1
rank 2048
yor1−dF
miss 0
orf −7.8
int −5.2
rank 2645
THI6(YPL214C)
DR = −635
Yor1
miss 0
orf 4.6
int −5.2
rank 3281
yor1−dF
miss 0
orf −8.2
int −5.2
rank 2646
(YKL044W)
DR = 5
Yor1
miss 0
orf −0.8
int −2.2
rank 2642
yor1−dF
miss 0
orf 1.8
int −5.2
rank 2647
ECM27(YJR106W)
DR = −761
Yor1
miss 0
orf −2
int −5.9
rank 3409
yor1−dF
miss 0
orf −3.7
int −5.2
rank 2648
SAS3(YBL052C)
DR = −1702
Yor1
miss 0
orf −1.6
int −17.8
rank 4351
yor1−dF
miss 0
orf 5.1
int −5.2
rank 2649
RGD2(YFL047W)
DR = 789
Yor1
miss 0
orf −0.7
int 0.8
rank 1861
yor1−dF
miss 0
orf 0.2
int −5.2
rank 2650
(YNR047W)
DR = −1009
Yor1
miss 0
orf 2.3
int −7.3
rank 3660
yor1−dF
miss 0
orf −1.7
int −5.2
rank 2651
MRC1(YCL061C)
DR = −431
Yor1
miss 0
orf 9.5
int −4.3
rank 3083
yor1−dF
miss 0
orf 7.4
int −5.2
rank 2652
0.35
0.25
0.20
0.15
0.10
0.05
0.00
−60
−20
0
20
40
−50
0
50

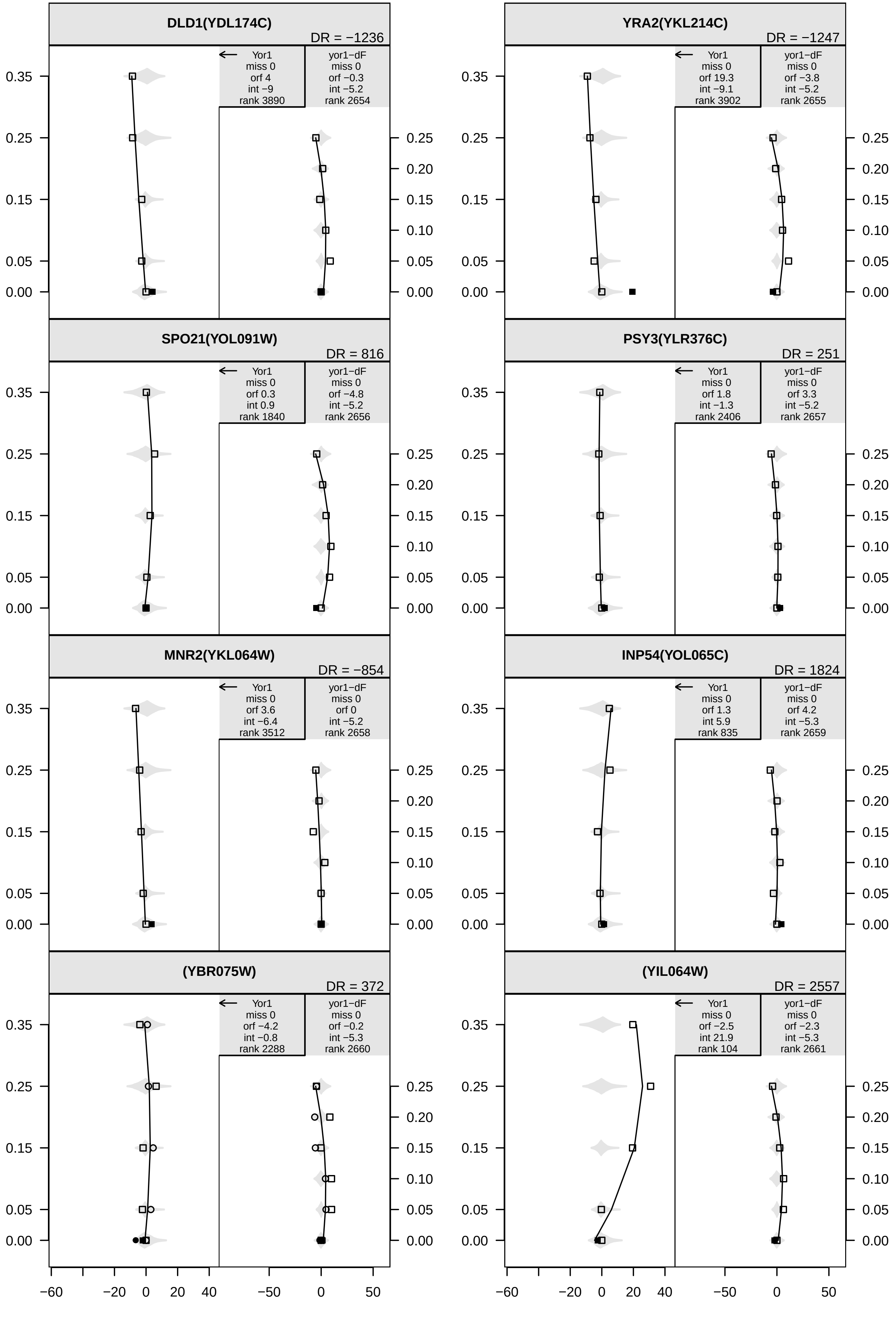

DLD1(YDL174C)
DR = −1236
Yor1 miss 0 orf 4 int −9 rank 3890
yor1−dF miss 0 orf −0.3 int −5.2 rank 2654
YRA2(YKL214C)
DR = −1247
Yor1 miss 0 orf 19.3 int −9.1 rank 3902
yor1−dF miss 0 orf −3.8 int −5.2 rank 2655
SPO21(YOL091W)
DR = 816
Yor1 miss 0 orf 0.3 int 0.9 rank 1840
yor1−dF miss 0 orf −4.8 int −5.2 rank 2656
PSY3(YLR376C)
DR = 251
Yor1 miss 0 orf 1.8 int −1.3 rank 2406
yor1−dF miss 0 orf 3.3 int −5.2 rank 2657
MNR2(YKL064W)
DR = −854
Yor1 miss 0 orf 3.6 int −6.4 rank 3512
yor1−dF miss 0 orf 0 int −5.2 rank 2658
INP54(YOL065C)
DR = 1824
Yor1 miss 0 orf 1.3 int 5.9 rank 835
yor1−dF miss 0 orf 4.2 int −5.3 rank 2659
(YBR075W)
DR = 372
Yor1 miss 0 orf −4.2 int −0.8 rank 2288
yor1−dF miss 0 orf −0.2 int −5.3 rank 2660
(YIL064W)
DR = 2557
Yor1 miss 0 orf −2.5 int 21.9 rank 104
yor1−dF miss 0 orf −2.3 int −5.3 rank 2661
0.35 0.25 0.15 0.05 0.00
0.25 0.20 0.15 0.10 0.05 0.00
−60 −20 0 20 40
−50 0 50

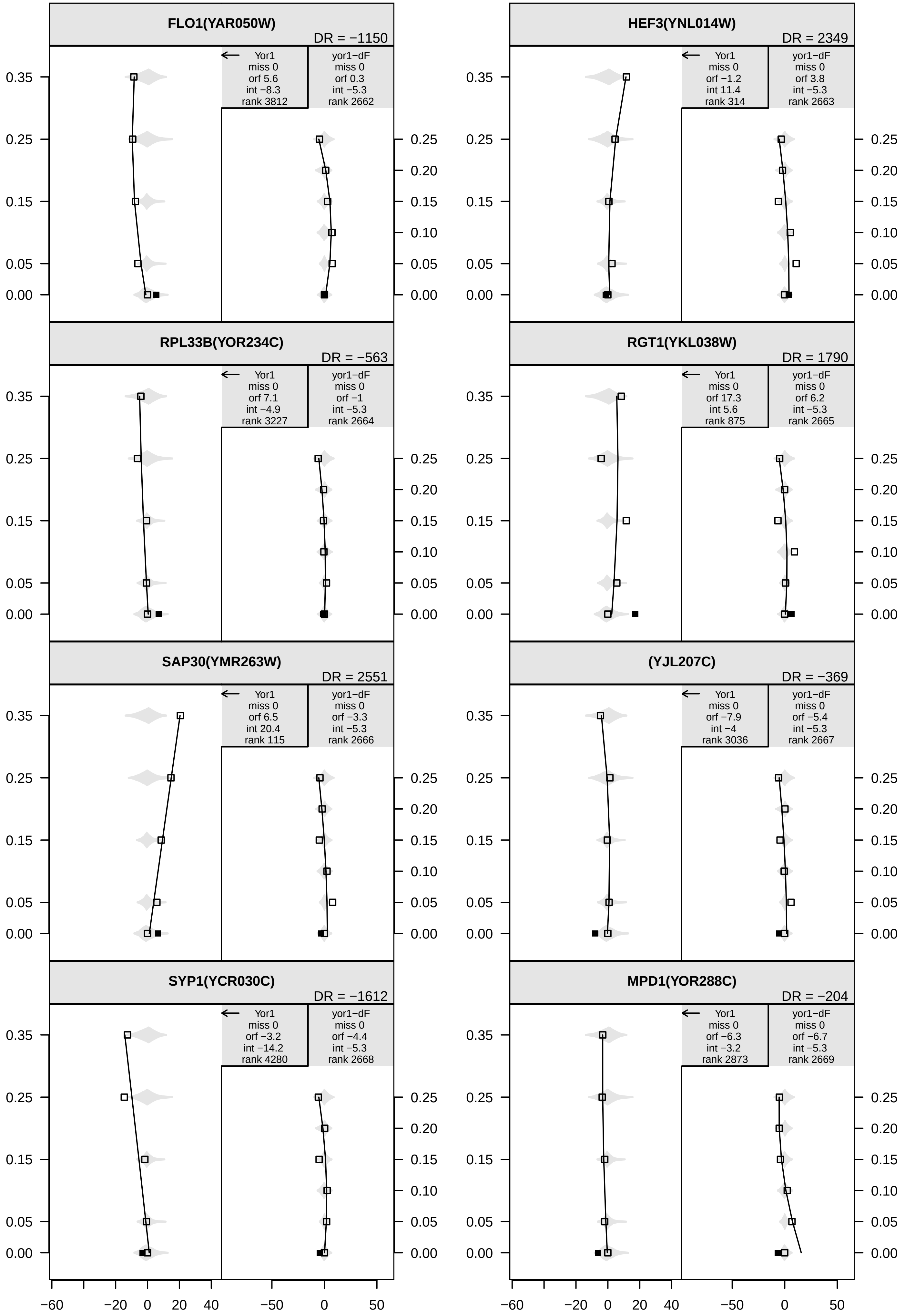

FLO1(YAR050W)
DR = −1150
Yor1
miss 0
orf 5.6
int −8.3
rank 3812
yor1−dF
miss 0
orf 0.3
int −5.3
rank 2662
HEF3(YNL014W)
DR = 2349
Yor1
miss 0
orf −1.2
int 11.4
rank 314
yor1−dF
miss 0
orf 3.8
int −5.3
rank 2663
RPL33B(YOR234C)
DR = −563
Yor1
miss 0
orf 7.1
int −4.9
rank 3227
yor1−dF
miss 0
orf −1
int −5.3
rank 2664
RGT1(YKL038W)
DR = 1790
Yor1
miss 0
orf 17.3
int 5.6
rank 875
yor1−dF
miss 0
orf 6.2
int −5.3
rank 2665
SAP30(YMR263W)
DR = 2551
Yor1
miss 0
orf 6.5
int 20.4
rank 115
yor1−dF
miss 0
orf −3.3
int −5.3
rank 2666
(YJL207C)
DR = −369
Yor1
miss 0
orf −7.9
int −4
rank 3036
yor1−dF
miss 0
orf −5.4
int −5.3
rank 2667
SYP1(YCR030C)
DR = −1612
Yor1
miss 0
orf −3.2
int −14.2
rank 4280
yor1−dF
miss 0
orf −4.4
int −5.3
rank 2668
MPD1(YOR288C)
DR = −204
Yor1
miss 0
orf −6.3
int −3.2
rank 2873
yor1−dF
miss 0
orf −6.7
int −5.3
rank 2669
0.35
0.25
0.20
0.15
0.10
0.05
0.00
−60
−20
0
20
40
−50
0
50

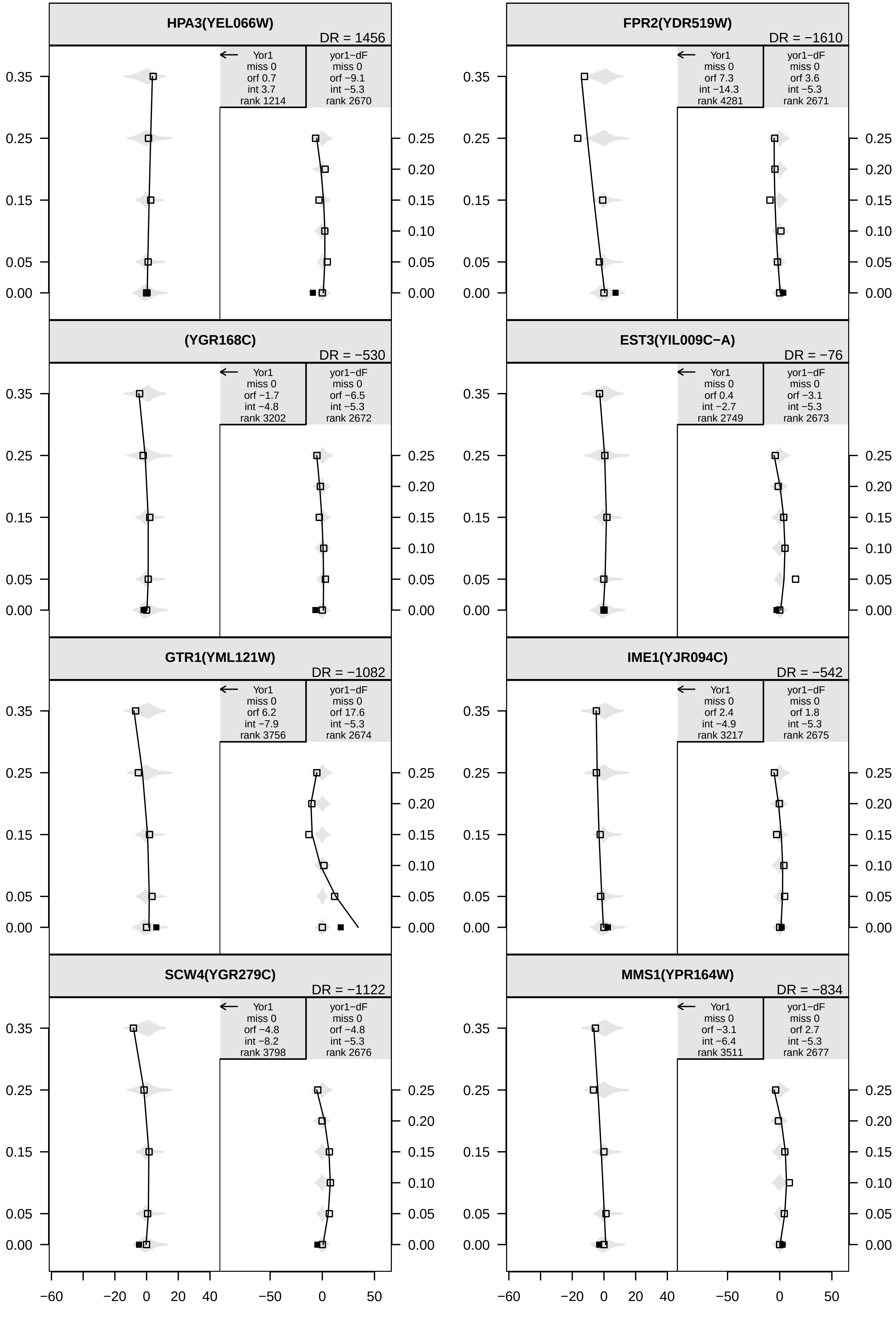

HPA3(YEL066W)
DR = 1456
Yor1 miss 0 orf 0.7 int 3.7 rank 1214
yor1−dF miss 0 orf −9.1 int −5.3 rank 2670
FPR2(YDR519W)
DR = −1610
Yor1 miss 0 orf 7.3 int −14.3 rank 4281
yor1−dF miss 0 orf 3.6 int −5.3 rank 2671
(YGR168C)
DR = −530
Yor1 miss 0 orf −1.7 int −4.8 rank 3202
yor1−dF miss 0 orf −6.5 int −5.3 rank 2672
EST3(YIL009C−A)
DR = −76
Yor1 miss 0 orf 0.4 int −2.7 rank 2749
yor1−dF miss 0 orf −3.1 int −5.3 rank 2673
GTR1(YML121W)
DR = −1082
Yor1 miss 0 orf 6.2 int −7.9 rank 3756
yor1−dF miss 0 orf 17.6 int −5.3 rank 2674
IME1(YJR094C)
DR = −542
Yor1 miss 0 orf 2.4 int −4.9 rank 3217
yor1−dF miss 0 orf 1.8 int −5.3 rank 2675
SCW4(YGR279C)
DR = −1122
Yor1 miss 0 orf −4.8 int −8.2 rank 3798
yor1−dF miss 0 orf −4.8 int −5.3 rank 2676
MMS1(YPR164W)
DR = −834
Yor1 miss 0 orf −3.1 int −6.4 rank 3511
yor1−dF miss 0 orf 2.7 int −5.3 rank 2677
0.35 0.25 0.15 0.05 0.00
0.25 0.20 0.15 0.10 0.05 0.00
−60 −20 0 20 40
−50 0 50

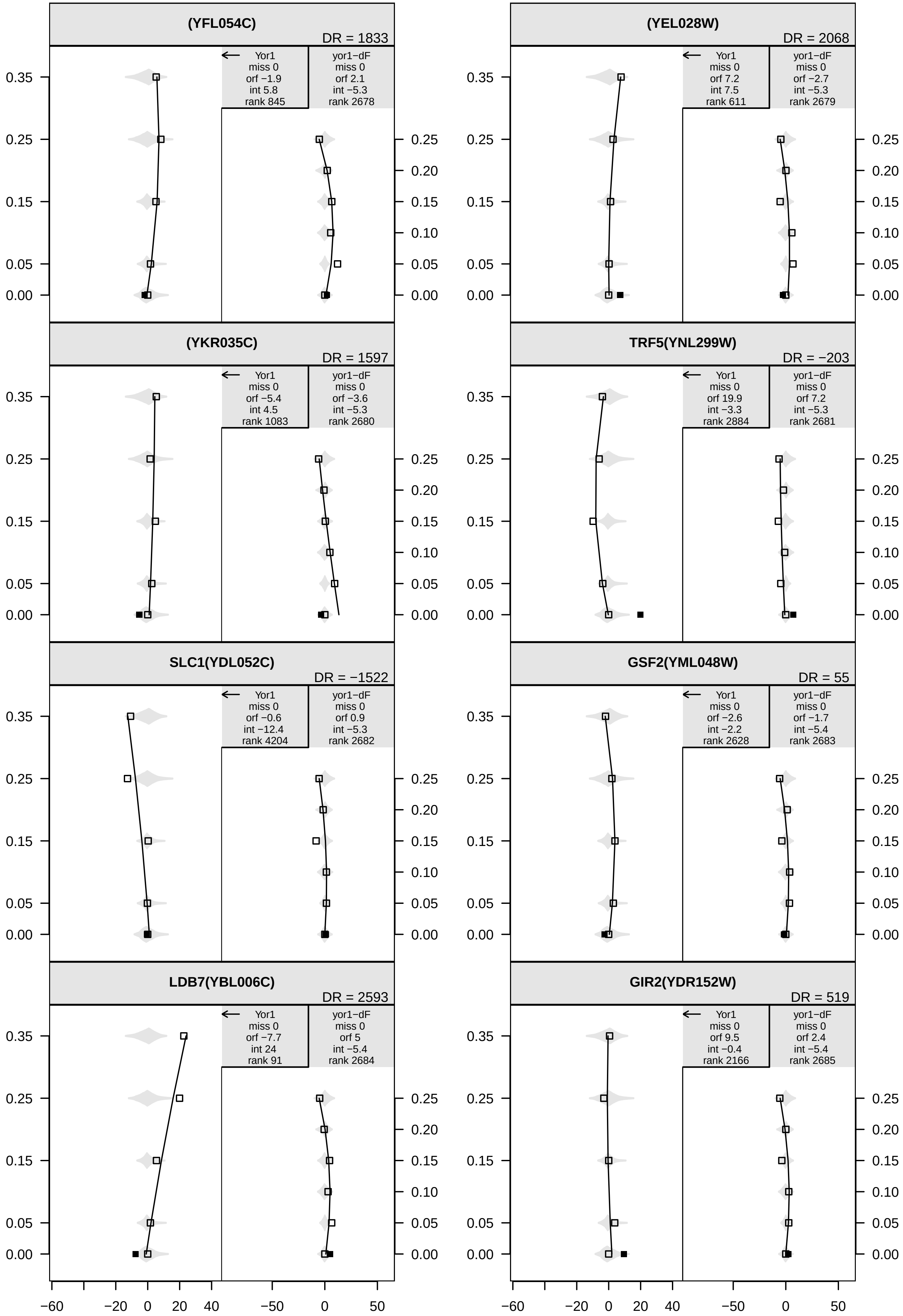

(YFL054C)
DR = 1833
Yor1 miss 0 orf −1.9 int 5.8 rank 845
yor1−dF miss 0 orf 2.1 int −5.3 rank 2678
(YEL028W)
DR = 2068
Yor1 miss 0 orf 7.2 int 7.5 rank 611
yor1−dF miss 0 orf −2.7 int −5.3 rank 2679
(YKR035C)
DR = 1597
Yor1 miss 0 orf −5.4 int 4.5 rank 1083
yor1−dF miss 0 orf −3.6 int −5.3 rank 2680
TRF5(YNL299W)
DR = −203
Yor1 miss 0 orf 19.9 int −3.3 rank 2884
yor1−dF miss 0 orf 7.2 int −5.3 rank 2681
SLC1(YDL052C)
DR = −1522
Yor1 miss 0 orf −0.6 int −12.4 rank 4204
yor1−dF miss 0 orf 0.9 int −5.3 rank 2682
GSF2(YML048W)
DR = 55
Yor1 miss 0 orf −2.6 int −2.2 rank 2628
yor1−dF miss 0 orf −1.7 int −5.4 rank 2683
LDB7(YBL006C)
DR = 2593
Yor1 miss 0 orf −7.7 int 24 rank 91
yor1−dF miss 0 orf 5 int −5.4 rank 2684
GIR2(YDR152W)
DR = 519
Yor1 miss 0 orf 9.5 int −0.4 rank 2166
yor1−dF miss 0 orf 2.4 int −5.4 rank 2685
0.35
0.25
0.20
0.15
0.10
0.05
0.00
−60
−20
0
20
40
−50
0
50

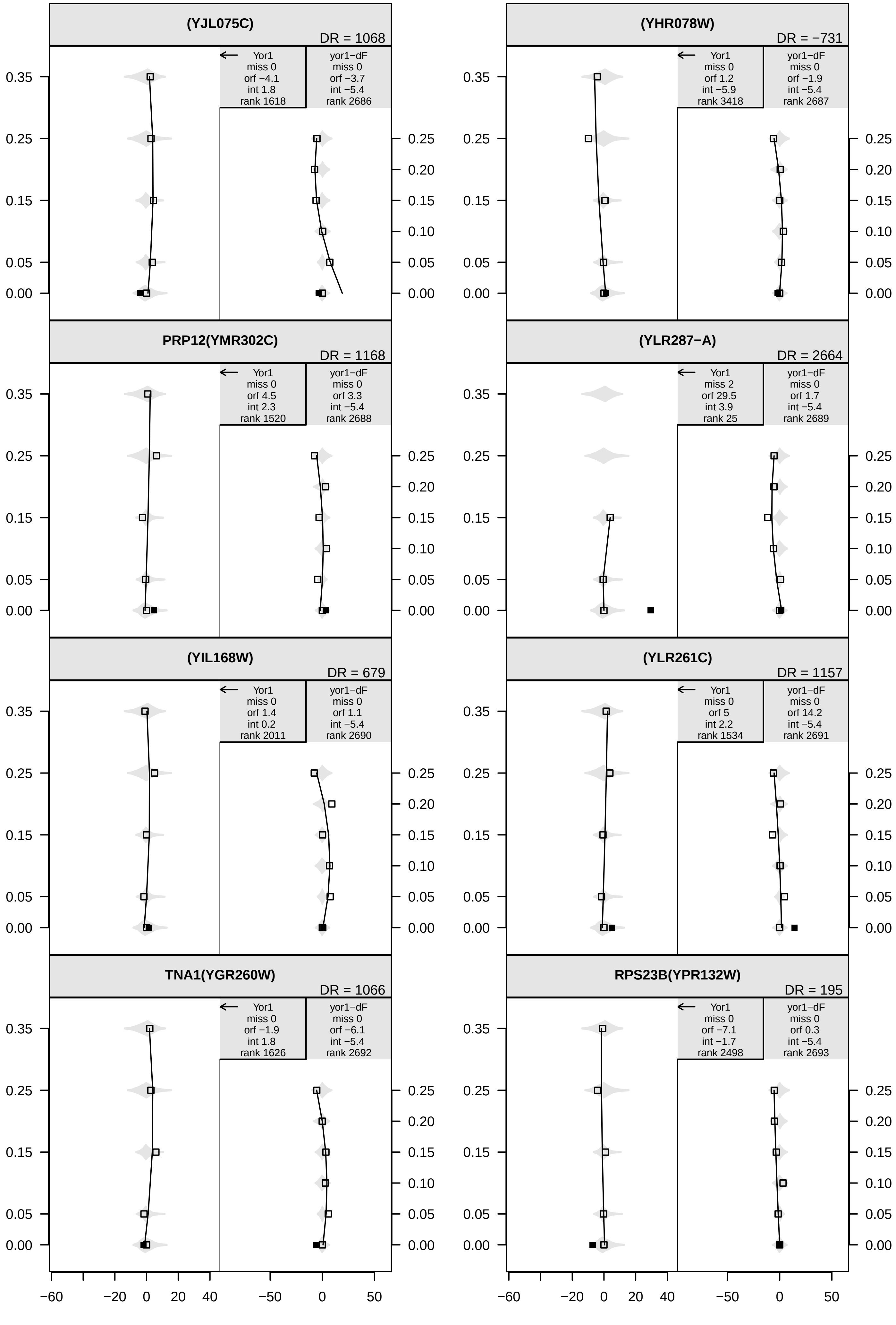
(YJL075C)
DR = 1068
Yor1 miss 0 orf −4.1 int 1.8 rank 1618
yor1−dF miss 0 orf −3.7 int −5.4 rank 2686
(YHR078W)
DR = −731
Yor1 miss 0 orf 1.2 int −5.9 rank 3418
yor1−dF miss 0 orf −1.9 int −5.4 rank 2687
PRP12(YMR302C)
DR = 1168
Yor1 miss 0 orf 4.5 int 2.3 rank 1520
yor1−dF miss 0 orf 3.3 int −5.4 rank 2688
(YLR287−A)
DR = 2664
Yor1 miss 2 orf 29.5 int 3.9 rank 25
yor1−dF miss 0 orf 1.7 int −5.4 rank 2689
(YIL168W)
DR = 679
Yor1 miss 0 orf 1.4 int 0.2 rank 2011
yor1−dF miss 0 orf 1.1 int −5.4 rank 2690
(YLR261C)
DR = 1157
Yor1 miss 0 orf 5 int 2.2 rank 1534
yor1−dF miss 0 orf 14.2 int −5.4 rank 2691
TNA1(YGR260W)
DR = 1066
Yor1 miss 0 orf −1.9 int 1.8 rank 1626
yor1−dF miss 0 orf −6.1 int −5.4 rank 2692
RPS23B(YPR132W)
DR = 195
Yor1 miss 0 orf −7.1 int −1.7 rank 2498
yor1−dF miss 0 orf 0.3 int −5.4 rank 2693

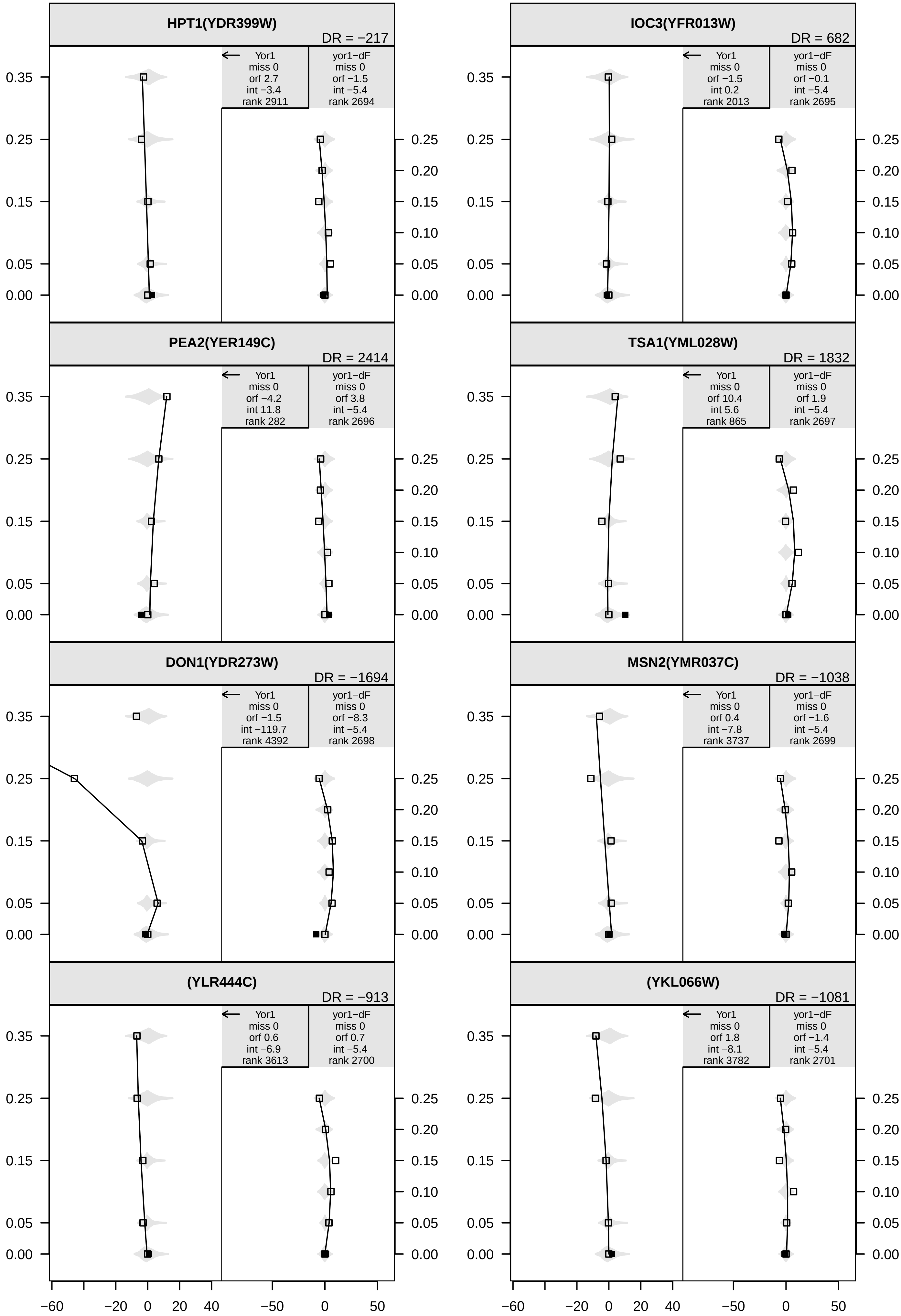
HPT1(YDR399W)
DR = −217
Yor1
miss 0
orf 2.7
int −3.4
rank 2911
yor1−dF
miss 0
orf −1.5
int −5.4
rank 2694
IOC3(YFR013W)
DR = 682
Yor1
miss 0
orf −1.5
int 0.2
rank 2013
yor1−dF
miss 0
orf −0.1
int −5.4
rank 2695
PEA2(YER149C)
DR = 2414
Yor1
miss 0
orf −4.2
int 11.8
rank 282
yor1−dF
miss 0
orf 3.8
int −5.4
rank 2696
TSA1(YML028W)
DR = 1832
Yor1
miss 0
orf 10.4
int 5.6
rank 865
yor1−dF
miss 0
orf 1.9
int −5.4
rank 2697
DON1(YDR273W)
DR = −1694
Yor1
miss 0
orf −1.5
int −119.7
rank 4392
yor1−dF
miss 0
orf −8.3
int −5.4
rank 2698
MSN2(YMR037C)
DR = −1038
Yor1
miss 0
orf 0.4
int −7.8
rank 3737
yor1−dF
miss 0
orf −1.6
int −5.4
rank 2699
(YLR444C)
DR = −913
Yor1
miss 0
orf 0.6
int −6.9
rank 3613
yor1−dF
miss 0
orf 0.7
int −5.4
rank 2700
(YKL066W)
DR = −1081
Yor1
miss 0
orf 1.8
int −8.1
rank 3782
yor1−dF
miss 0
orf −1.4
int −5.4
rank 2701
0.35
0.25
0.20
0.15
0.10
0.05
0.00
−60
−20
0
20
40
−50
0
50

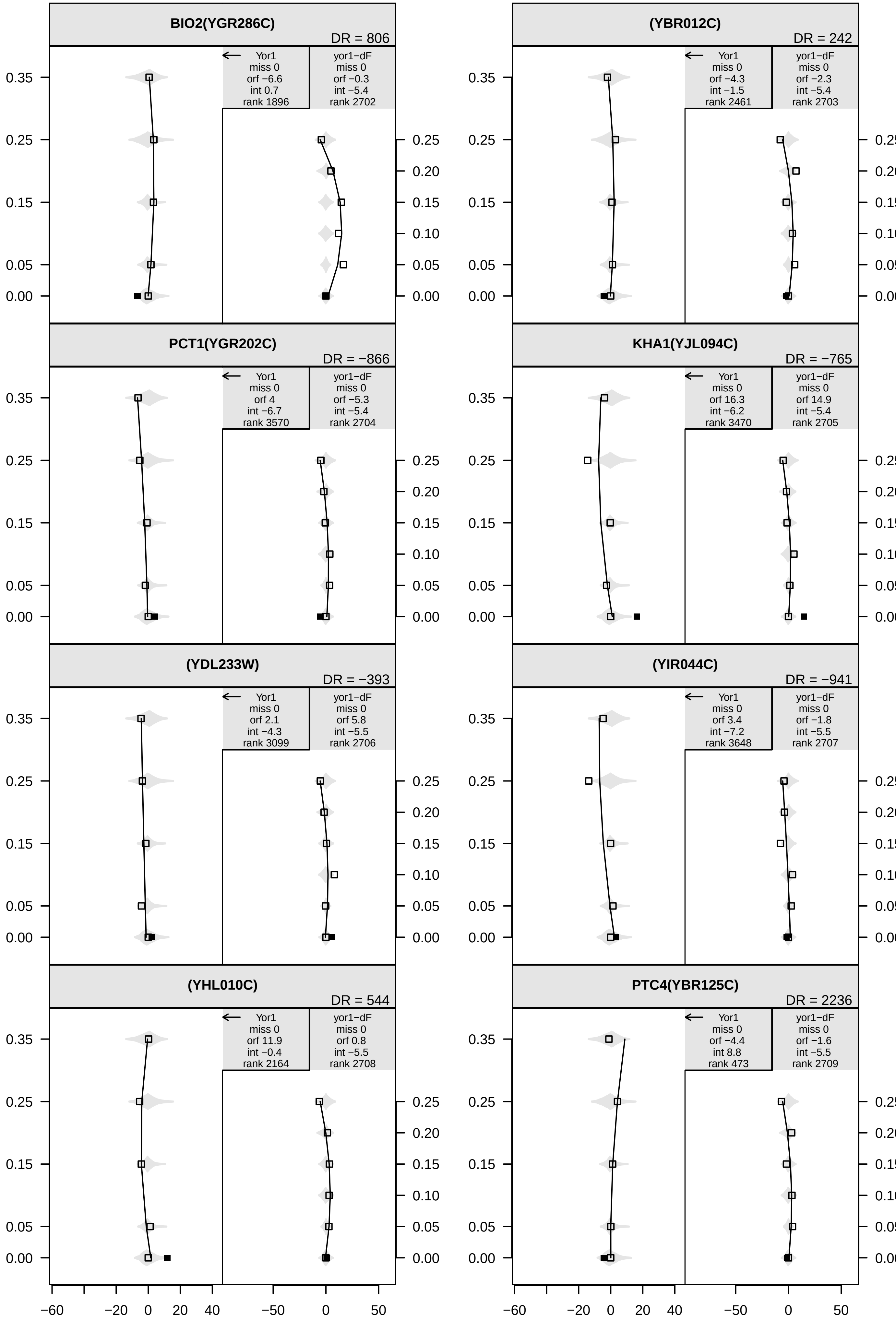

BIO2(YGR286C)
DR = 806
Yor1 miss 0 orf −6.6 int 0.7 rank 1896
yor1−dF miss 0 orf −0.3 int −5.4 rank 2702
(YBR012C)
DR = 242
Yor1 miss 0 orf −4.3 int −1.5 rank 2461
yor1−dF miss 0 orf −2.3 int −5.4 rank 2703
PCT1(YGR202C)
DR = −866
Yor1 miss 0 orf 4 int −6.7 rank 3570
yor1−dF miss 0 orf −5.3 int −5.4 rank 2704
KHA1(YJL094C)
DR = −765
Yor1 miss 0 orf 16.3 int −6.2 rank 3470
yor1−dF miss 0 orf 14.9 int −5.4 rank 2705
(YDL233W)
DR = −393
Yor1 miss 0 orf 2.1 int −4.3 rank 3099
yor1−dF miss 0 orf 5.8 int −5.5 rank 2706
(YIR044C)
DR = −941
Yor1 miss 0 orf 3.4 int −7.2 rank 3648
yor1−dF miss 0 orf −1.8 int −5.5 rank 2707
(YHL010C)
DR = 544
Yor1 miss 0 orf 11.9 int −0.4 rank 2164
yor1−dF miss 0 orf 0.8 int −5.5 rank 2708
PTC4(YBR125C)
DR = 2236
Yor1 miss 0 orf −4.4 int 8.8 rank 473
yor1−dF miss 0 orf −1.6 int −5.5 rank 2709
0.00 0.05 0.10 0.15 0.20 0.25 0.35
−60 −20 0 20 40 −50 0 50

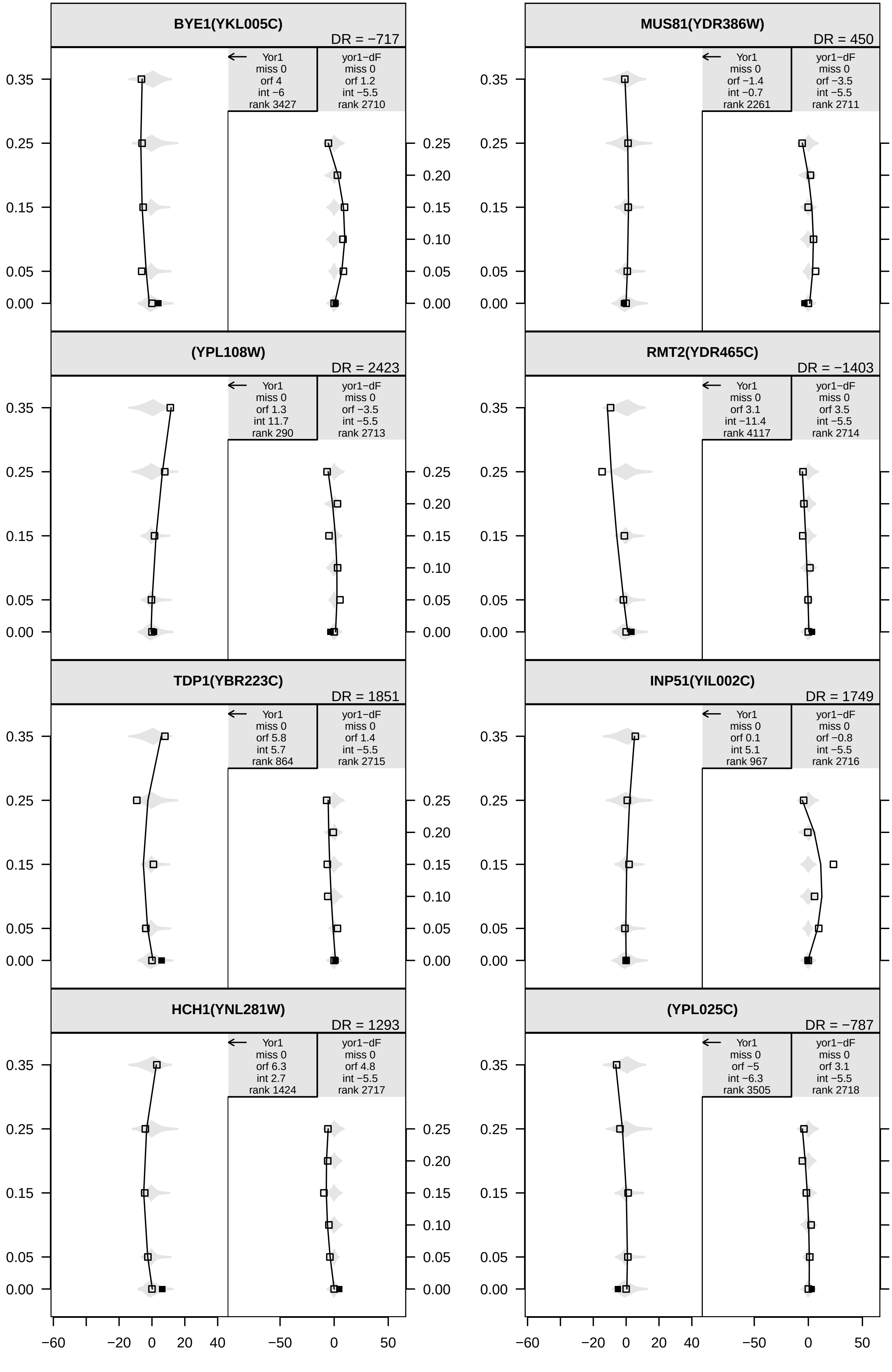
BYE1(YKL005C)
DR = −717
Yor1 miss 0 orf 4 int −6 rank 3427
yor1−dF miss 0 orf 1.2 int −5.5 rank 2710
MUS81(YDR386W)
DR = 450
Yor1 miss 0 orf −1.4 int −0.7 rank 2261
yor1−dF miss 0 orf −3.5 int −5.5 rank 2711
(YPL108W)
DR = 2423
Yor1 miss 0 orf 1.3 int 11.7 rank 290
yor1−dF miss 0 orf −3.5 int −5.5 rank 2713
RMT2(YDR465C)
DR = −1403
Yor1 miss 0 orf 3.1 int −11.4 rank 4117
yor1−dF miss 0 orf 3.5 int −5.5 rank 2714
TDP1(YBR223C)
DR = 1851
Yor1 miss 0 orf 5.8 int 5.7 rank 864
yor1−dF miss 0 orf 1.4 int −5.5 rank 2715
INP51(YIL002C)
DR = 1749
Yor1 miss 0 orf 0.1 int 5.1 rank 967
yor1−dF miss 0 orf −0.8 int −5.5 rank 2716
HCH1(YNL281W)
DR = 1293
Yor1 miss 0 orf 6.3 int 2.7 rank 1424
yor1−dF miss 0 orf 4.8 int −5.5 rank 2717
(YPL025C)
DR = −787
Yor1 miss 0 orf −5 int −6.3 rank 3505
yor1−dF miss 0 orf 3.1 int −5.5 rank 2718
0.00 0.05 0.15 0.25 0.35
0.00 0.05 0.10 0.15 0.20 0.25
−60 −20 0 20 40
−50 0 50

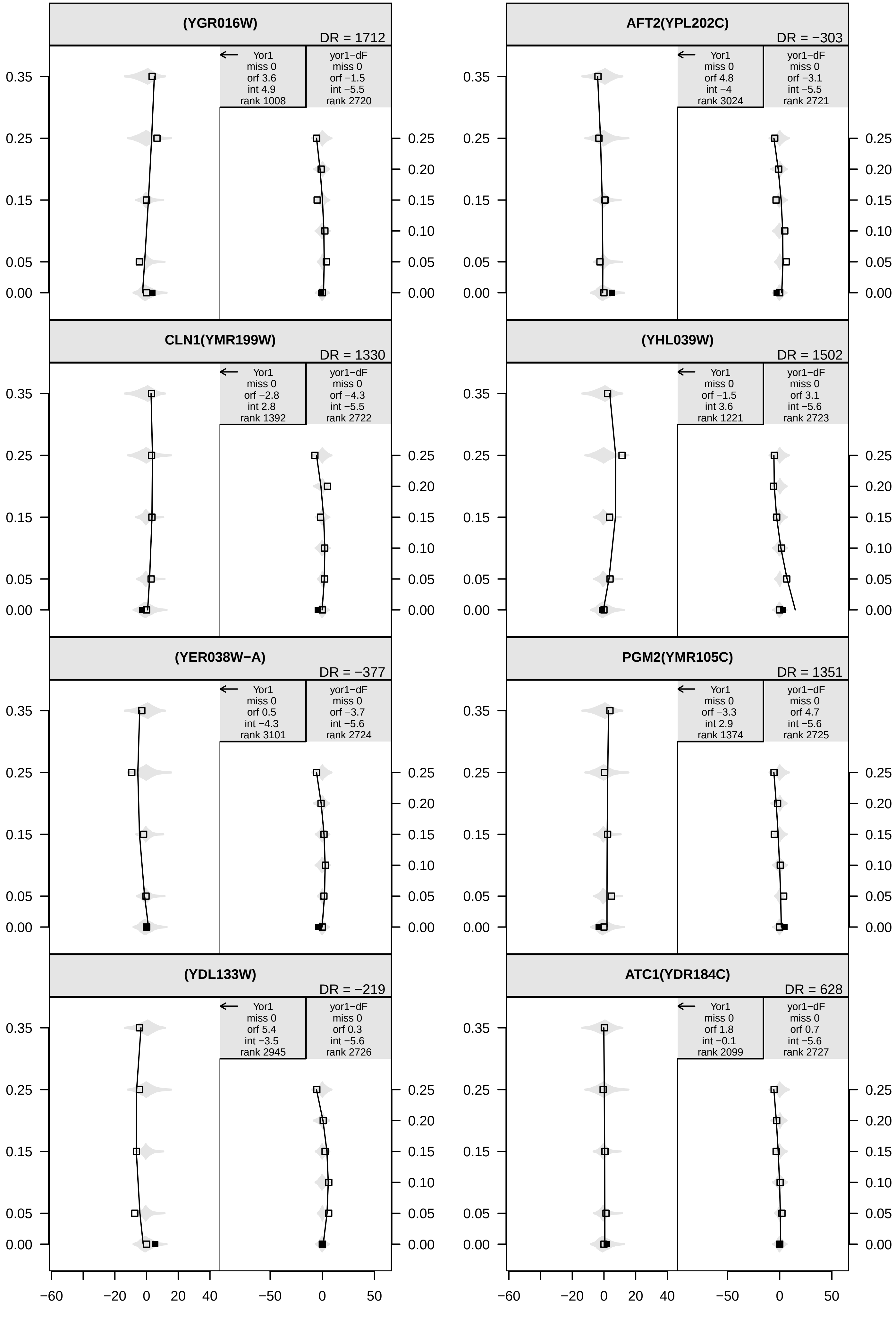

(YGR016W)
DR = 1712
Yor1 miss 0 orf 3.6 int 4.9 rank 1008
yor1−dF miss 0 orf −1.5 int −5.5 rank 2720
AFT2(YPL202C)
DR = −303
Yor1 miss 0 orf 4.8 int −4 rank 3024
yor1−dF miss 0 orf −3.1 int −5.5 rank 2721
CLN1(YMR199W)
DR = 1330
Yor1 miss 0 orf −2.8 int 2.8 rank 1392
yor1−dF miss 0 orf −4.3 int −5.5 rank 2722
(YHL039W)
DR = 1502
Yor1 miss 0 orf −1.5 int 3.6 rank 1221
yor1−dF miss 0 orf 3.1 int −5.6 rank 2723
(YER038W−A)
DR = −377
Yor1 miss 0 orf 0.5 int −4.3 rank 3101
yor1−dF miss 0 orf −3.7 int −5.6 rank 2724
PGM2(YMR105C)
DR = 1351
Yor1 miss 0 orf −3.3 int 2.9 rank 1374
yor1−dF miss 0 orf 4.7 int −5.6 rank 2725
(YDL133W)
DR = −219
Yor1 miss 0 orf 5.4 int −3.5 rank 2945
yor1−dF miss 0 orf 0.3 int −5.6 rank 2726
ATC1(YDR184C)
DR = 628
Yor1 miss 0 orf 1.8 int −0.1 rank 2099
yor1−dF miss 0 orf 0.7 int −5.6 rank 2727

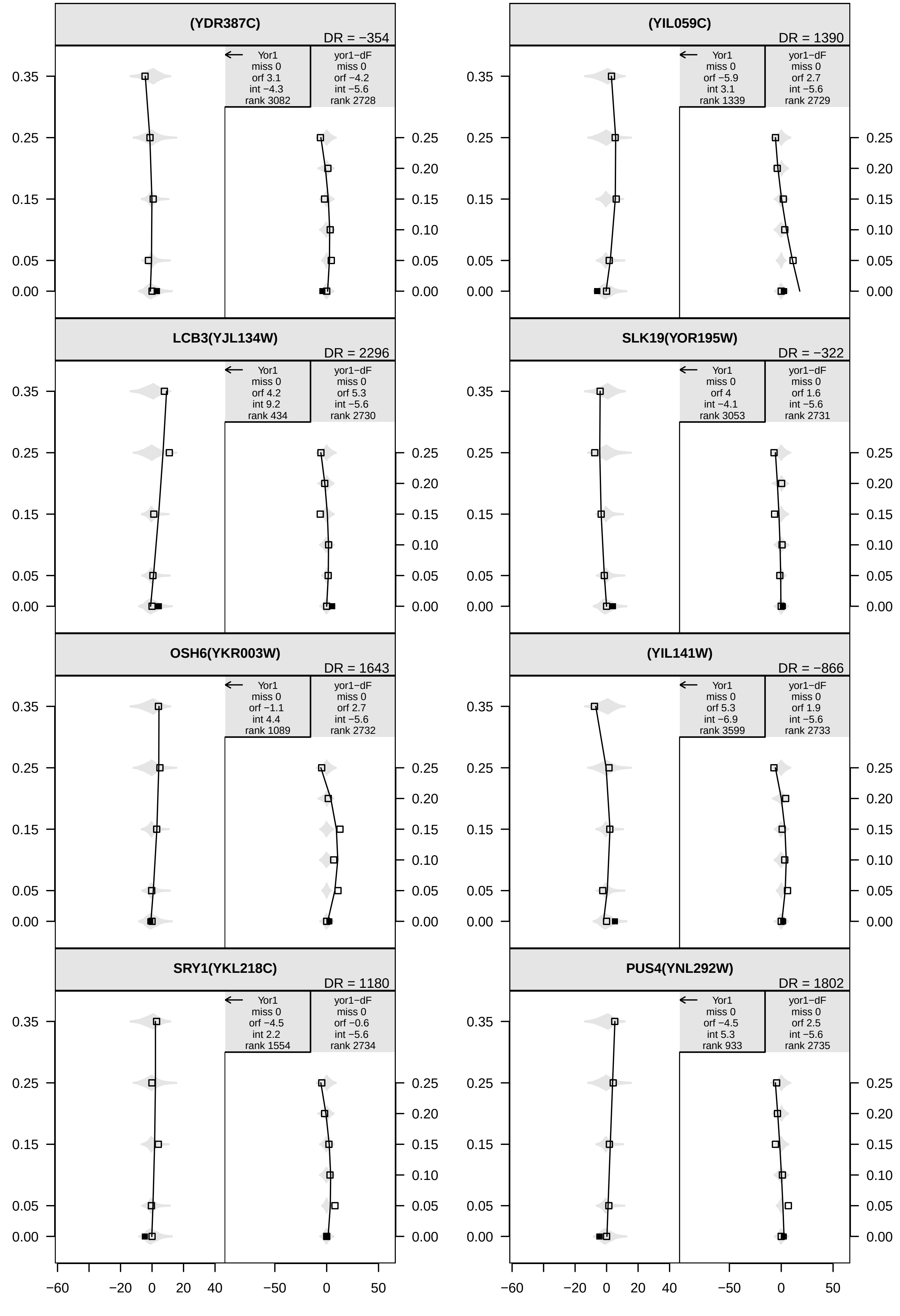
(YDR387C)
DR = −354
Yor1 miss 0 orf 3.1 int −4.3 rank 3082
yor1−dF miss 0 orf −4.2 int −5.6 rank 2728
(YIL059C)
DR = 1390
Yor1 miss 0 orf −5.9 int 3.1 rank 1339
yor1−dF miss 0 orf 2.7 int −5.6 rank 2729
LCB3(YJL134W)
DR = 2296
Yor1 miss 0 orf 4.2 int 9.2 rank 434
yor1−dF miss 0 orf 5.3 int −5.6 rank 2730
SLK19(YOR195W)
DR = −322
Yor1 miss 0 orf 4 int −4.1 rank 3053
yor1−dF miss 0 orf 1.6 int −5.6 rank 2731
OSH6(YKR003W)
DR = 1643
Yor1 miss 0 orf −1.1 int 4.4 rank 1089
yor1−dF miss 0 orf 2.7 int −5.6 rank 2732
(YIL141W)
DR = −866
Yor1 miss 0 orf 5.3 int −6.9 rank 3599
yor1−dF miss 0 orf 1.9 int −5.6 rank 2733
SRY1(YKL218C)
DR = 1180
Yor1 miss 0 orf −4.5 int 2.2 rank 1554
yor1−dF miss 0 orf −0.6 int −5.6 rank 2734
PUS4(YNL292W)
DR = 1802
Yor1 miss 0 orf −4.5 int 5.3 rank 933
yor1−dF miss 0 orf 2.5 int −5.6 rank 2735
0.35 0.25 0.15 0.05 0.00
0.25 0.20 0.15 0.10 0.05 0.00
−60 −20 0 20 40
−50 0 50

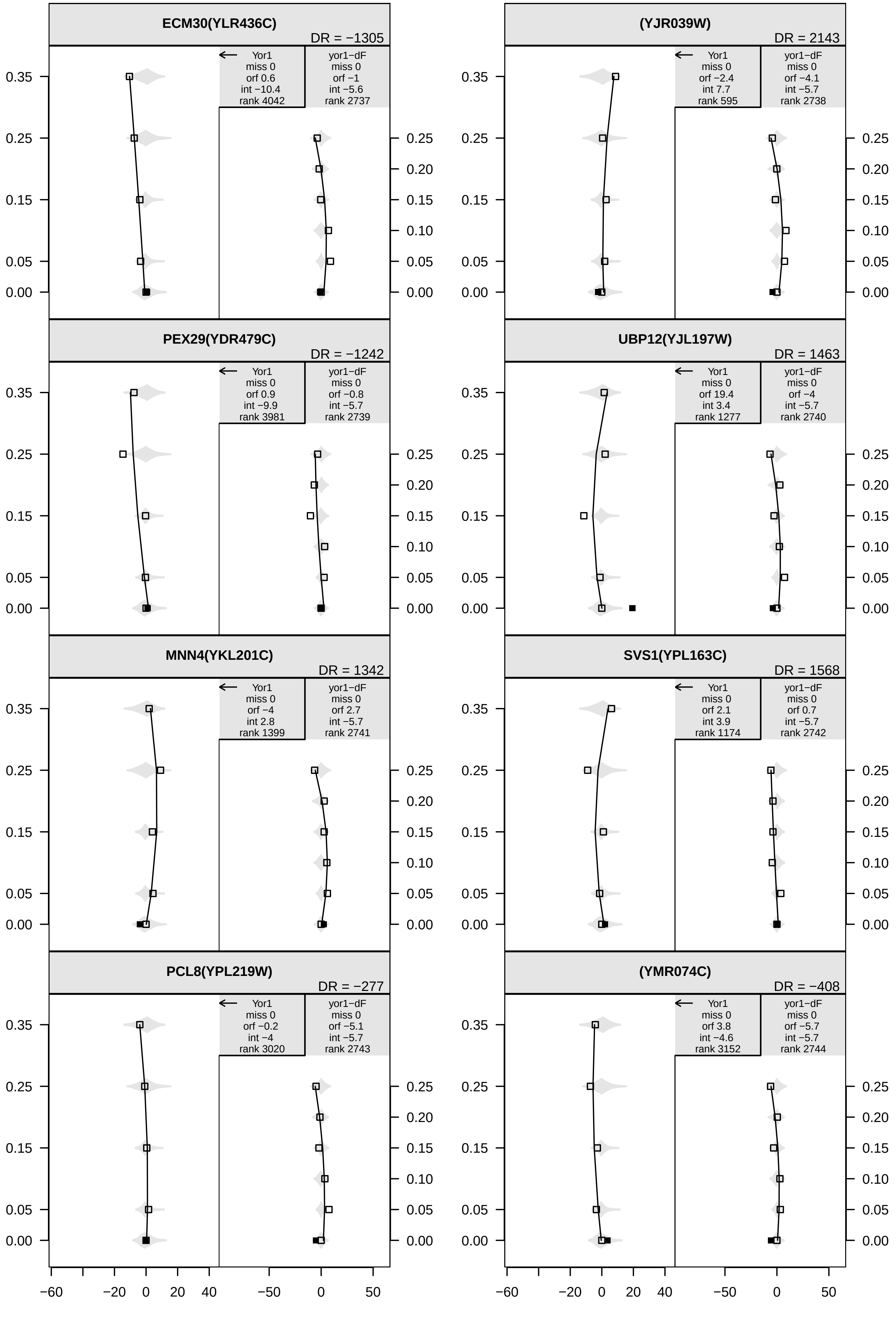

ECM30(YLR436C)
DR = −1305
Yor1 miss 0 orf 0.6 int −10.4 rank 4042
yor1−dF miss 0 orf −1 int −5.6 rank 2737
(YJR039W)
DR = 2143
Yor1 miss 0 orf −2.4 int 7.7 rank 595
yor1−dF miss 0 orf −4.1 int −5.7 rank 2738
PEX29(YDR479C)
DR = −1242
Yor1 miss 0 orf 0.9 int −9.9 rank 3981
yor1−dF miss 0 orf −0.8 int −5.7 rank 2739
UBP12(YJL197W)
DR = 1463
Yor1 miss 0 orf 19.4 int 3.4 rank 1277
yor1−dF miss 0 orf −4 int −5.7 rank 2740
MNN4(YKL201C)
DR = 1342
Yor1 miss 0 orf −4 int 2.8 rank 1399
yor1−dF miss 0 orf 2.7 int −5.7 rank 2741
SVS1(YPL163C)
DR = 1568
Yor1 miss 0 orf 2.1 int 3.9 rank 1174
yor1−dF miss 0 orf 0.7 int −5.7 rank 2742
PCL8(YPL219W)
DR = −277
Yor1 miss 0 orf −0.2 int −4 rank 3020
yor1−dF miss 0 orf −5.1 int −5.7 rank 2743
(YMR074C)
DR = −408
Yor1 miss 0 orf 3.8 int −4.6 rank 3152
yor1−dF miss 0 orf −5.7 int −5.7 rank 2744
0.35 0.25 0.15 0.05 0.00
0.25 0.20 0.15 0.10 0.05 0.00
−60 −20 0 20 40
−50 0 50

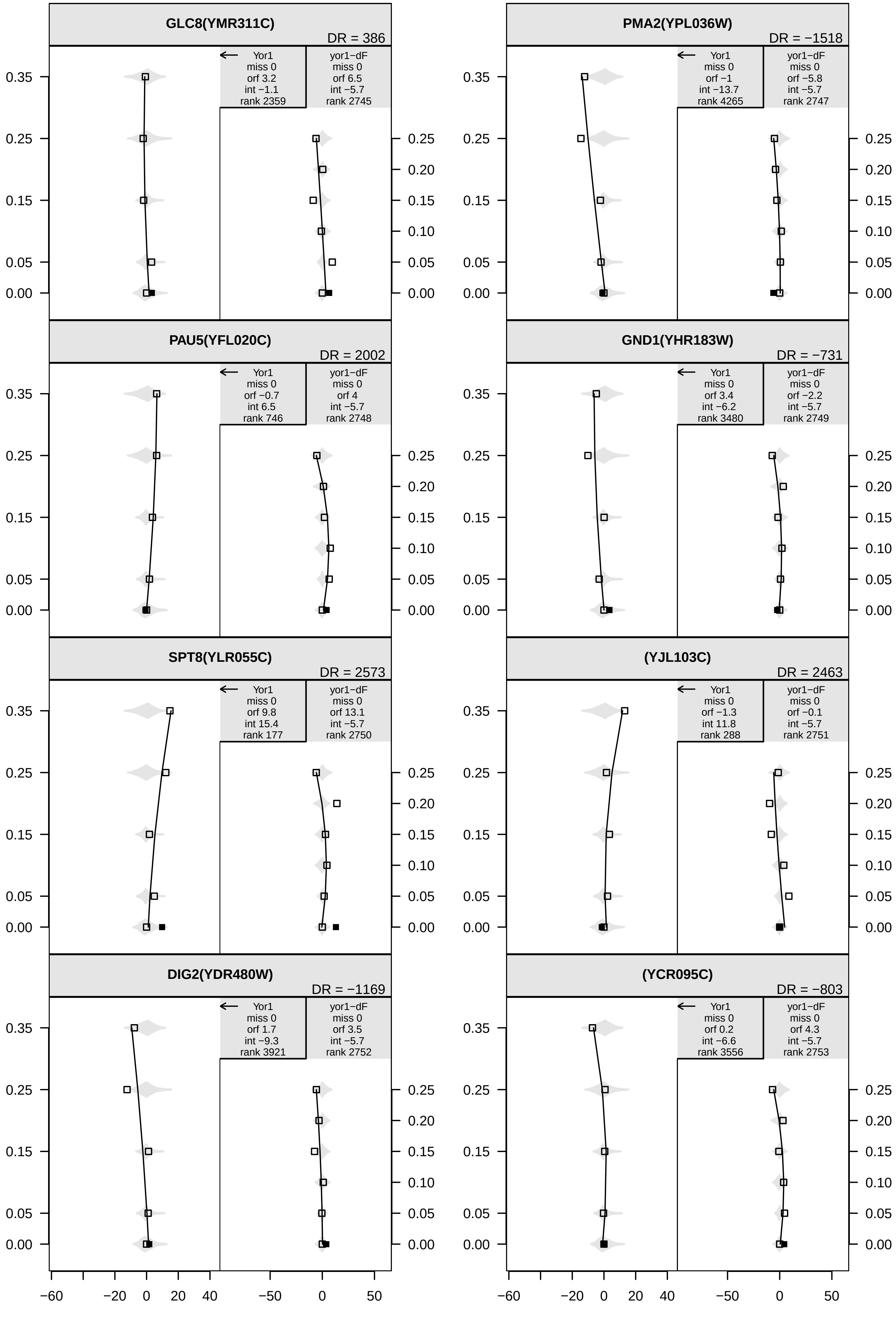
GLC8(YMR311C)
DR = 386
Yor1
miss 0
orf 3.2
int −1.1
rank 2359
yor1−dF
miss 0
orf 6.5
int −5.7
rank 2745
PMA2(YPL036W)
DR = −1518
Yor1
miss 0
orf −1
int −13.7
rank 4265
yor1−dF
miss 0
orf −5.8
int −5.7
rank 2747
PAU5(YFL020C)
DR = 2002
Yor1
miss 0
orf −0.7
int 6.5
rank 746
yor1−dF
miss 0
orf 4
int −5.7
rank 2748
GND1(YHR183W)
DR = −731
Yor1
miss 0
orf 3.4
int −6.2
rank 3480
yor1−dF
miss 0
orf −2.2
int −5.7
rank 2749
SPT8(YLR055C)
DR = 2573
Yor1
miss 0
orf 9.8
int 15.4
rank 177
yor1−dF
miss 0
orf 13.1
int −5.7
rank 2750
(YJL103C)
DR = 2463
Yor1
miss 0
orf −1.3
int 11.8
rank 288
yor1−dF
miss 0
orf −0.1
int −5.7
rank 2751
DIG2(YDR480W)
DR = −1169
Yor1
miss 0
orf 1.7
int −9.3
rank 3921
yor1−dF
miss 0
orf 3.5
int −5.7
rank 2752
(YCR095C)
DR = −803
Yor1
miss 0
orf 0.2
int −6.6
rank 3556
yor1−dF
miss 0
orf 4.3
int −5.7
rank 2753
0.35
0.25
0.20
0.15
0.10
0.05
0.00
−60
−20
0
20
40
−50
0
50

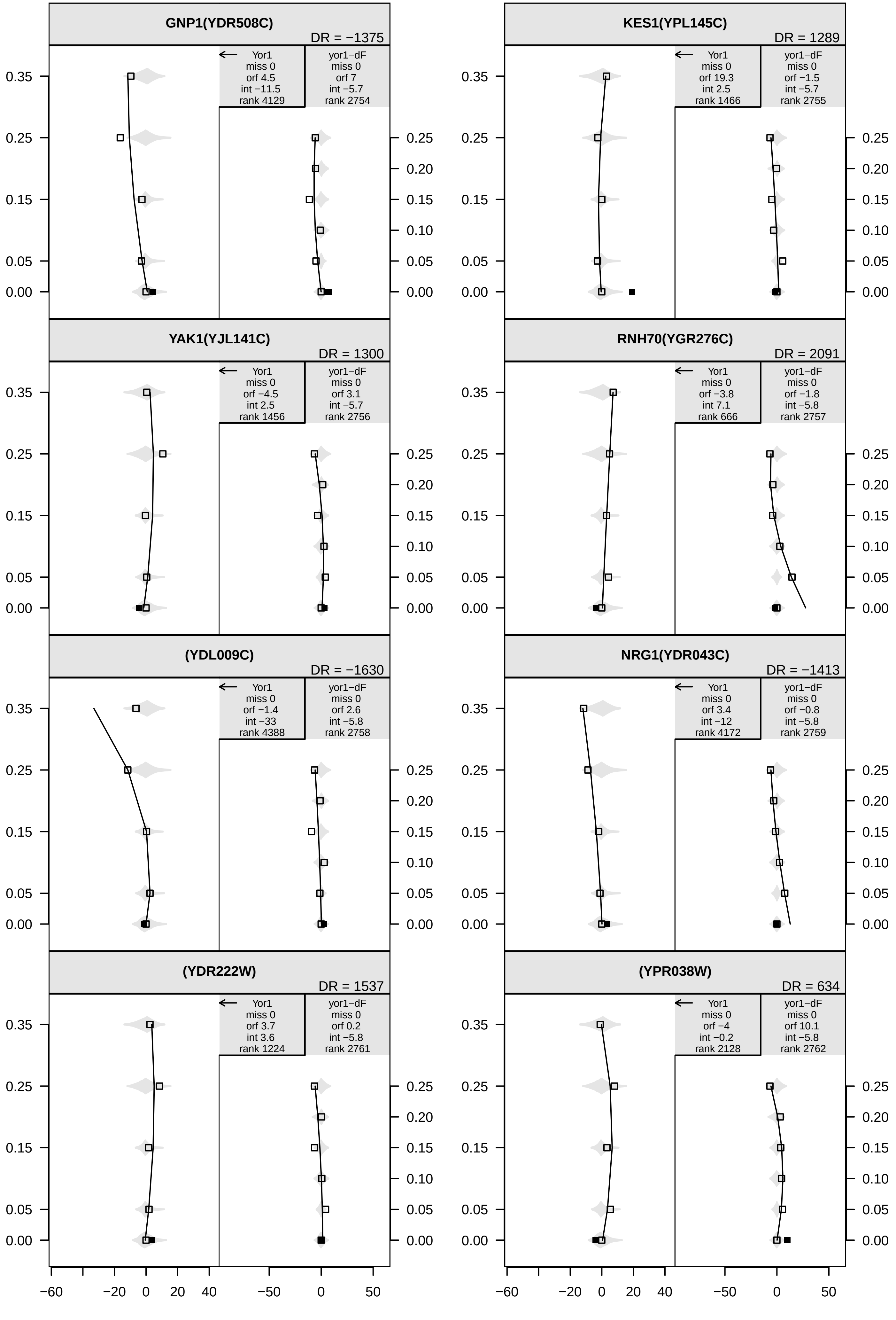
GNP1(YDR508C)
DR = −1375
Yor1
miss 0
orf 4.5
int −11.5
rank 4129
yor1−dF
miss 0
orf 7
int −5.7
rank 2754
KES1(YPL145C)
DR = 1289
Yor1
miss 0
orf 19.3
int 2.5
rank 1466
yor1−dF
miss 0
orf −1.5
int −5.7
rank 2755
YAK1(YJL141C)
DR = 1300
Yor1
miss 0
orf −4.5
int 2.5
rank 1456
yor1−dF
miss 0
orf 3.1
int −5.7
rank 2756
RNH70(YGR276C)
DR = 2091
Yor1
miss 0
orf −3.8
int 7.1
rank 666
yor1−dF
miss 0
orf −1.8
int −5.8
rank 2757
(YDL009C)
DR = −1630
Yor1
miss 0
orf −1.4
int −33
rank 4388
yor1−dF
miss 0
orf 2.6
int −5.8
rank 2758
NRG1(YDR043C)
DR = −1413
Yor1
miss 0
orf 3.4
int −12
rank 4172
yor1−dF
miss 0
orf −0.8
int −5.8
rank 2759
(YDR222W)
DR = 1537
Yor1
miss 0
orf 3.7
int 3.6
rank 1224
yor1−dF
miss 0
orf 0.2
int −5.8
rank 2761
(YPR038W)
DR = 634
Yor1
miss 0
orf −4
int −0.2
rank 2128
yor1−dF
miss 0
orf 10.1
int −5.8
rank 2762
0.35
0.25
0.20
0.15
0.10
0.05
0.00
−60
−20
0
20
40
−50
0
50

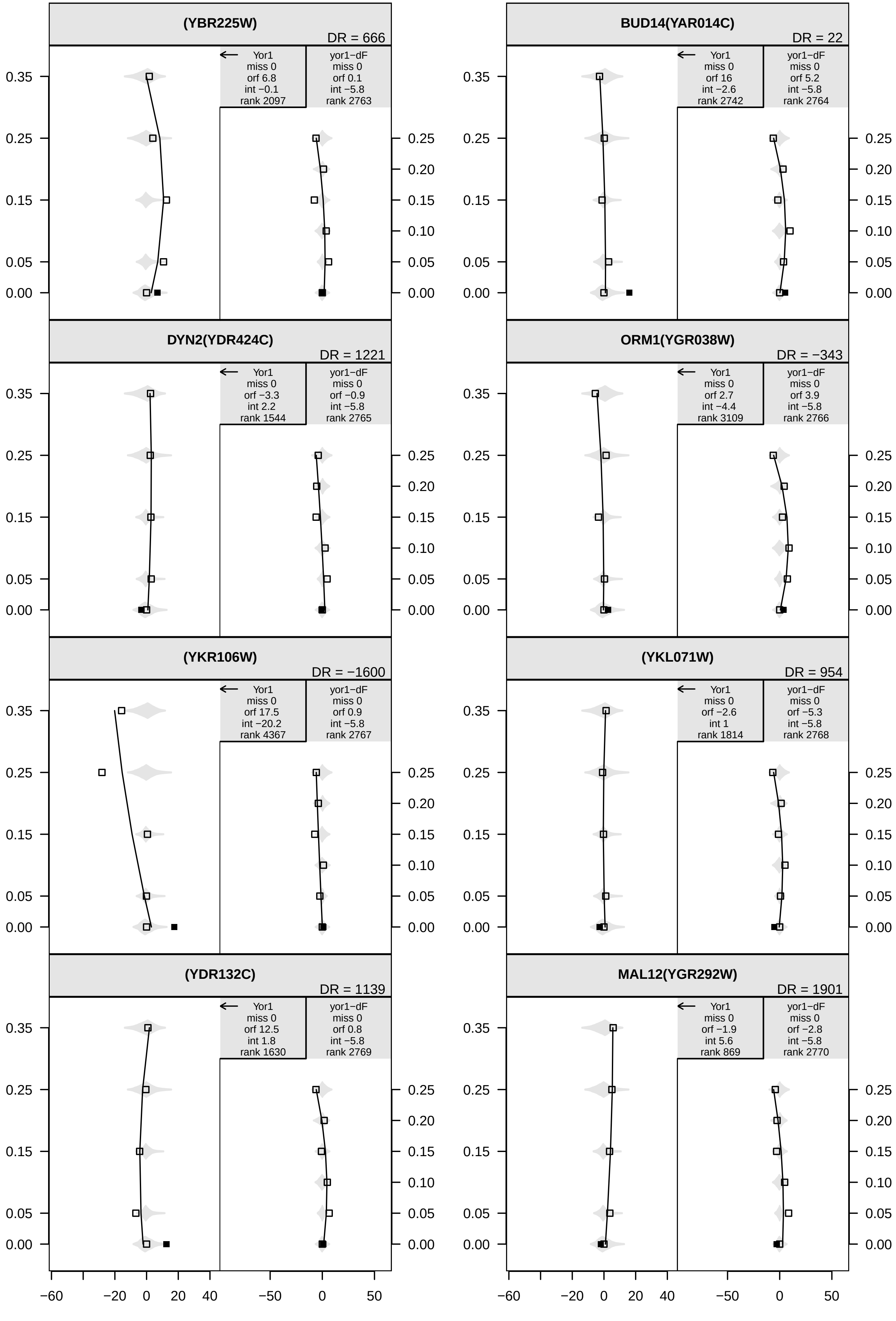

(YBR225W)
DR = 666
Yor1 miss 0 orf 6.8 int −0.1 rank 2097
yor1−dF miss 0 orf 0.1 int −5.8 rank 2763
BUD14(YAR014C)
DR = 22
Yor1 miss 0 orf 16 int −2.6 rank 2742
yor1−dF miss 0 orf 5.2 int −5.8 rank 2764
DYN2(YDR424C)
DR = 1221
Yor1 miss 0 orf −3.3 int 2.2 rank 1544
yor1−dF miss 0 orf −0.9 int −5.8 rank 2765
ORM1(YGR038W)
DR = −343
Yor1 miss 0 orf 2.7 int −4.4 rank 3109
yor1−dF miss 0 orf 3.9 int −5.8 rank 2766
(YKR106W)
DR = −1600
Yor1 miss 0 orf 17.5 int −20.2 rank 4367
yor1−dF miss 0 orf 0.9 int −5.8 rank 2767
(YKL071W)
DR = 954
Yor1 miss 0 orf −2.6 int 1 rank 1814
yor1−dF miss 0 orf −5.3 int −5.8 rank 2768
(YDR132C)
DR = 1139
Yor1 miss 0 orf 12.5 int 1.8 rank 1630
yor1−dF miss 0 orf 0.8 int −5.8 rank 2769
MAL12(YGR292W)
DR = 1901
Yor1 miss 0 orf −1.9 int 5.6 rank 869
yor1−dF miss 0 orf −2.8 int −5.8 rank 2770
0.00 0.05 0.10 0.15 0.20 0.25 0.35
−60 −20 0 20 40 −50 0 50

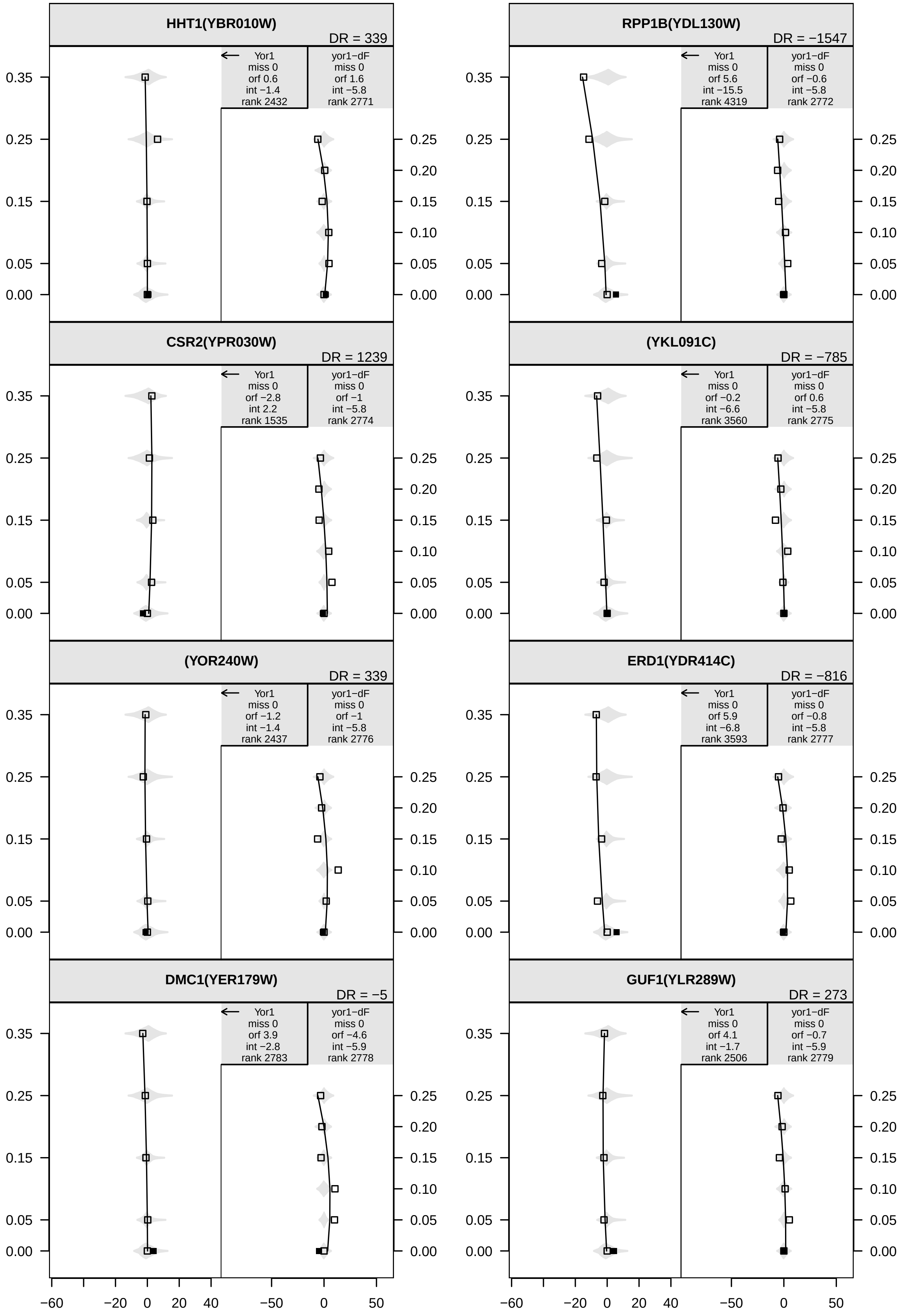

HHT1(YBR010W)
DR = 339
Yor1 miss 0 orf 0.6 int −1.4 rank 2432
yor1−dF miss 0 orf 1.6 int −5.8 rank 2771
RPP1B(YDL130W)
DR = −1547
Yor1 miss 0 orf 5.6 int −15.5 rank 4319
yor1−dF miss 0 orf −0.6 int −5.8 rank 2772
CSR2(YPR030W)
DR = 1239
Yor1 miss 0 orf −2.8 int 2.2 rank 1535
yor1−dF miss 0 orf −1 int −5.8 rank 2774
(YKL091C)
DR = −785
Yor1 miss 0 orf −0.2 int −6.6 rank 3560
yor1−dF miss 0 orf 0.6 int −5.8 rank 2775
(YOR240W)
DR = 339
Yor1 miss 0 orf −1.2 int −1.4 rank 2437
yor1−dF miss 0 orf −1 int −5.8 rank 2776
ERD1(YDR414C)
DR = −816
Yor1 miss 0 orf 5.9 int −6.8 rank 3593
yor1−dF miss 0 orf −0.8 int −5.8 rank 2777
DMC1(YER179W)
DR = −5
Yor1 miss 0 orf 3.9 int −2.8 rank 2783
yor1−dF miss 0 orf −4.6 int −5.9 rank 2778
GUF1(YLR289W)
DR = 273
Yor1 miss 0 orf 4.1 int −1.7 rank 2506
yor1−dF miss 0 orf −0.7 int −5.9 rank 2779
0.35 0.25 0.15 0.05 0.00
0.25 0.20 0.15 0.10 0.05 0.00
−60 −20 0 20 40
−50 0 50

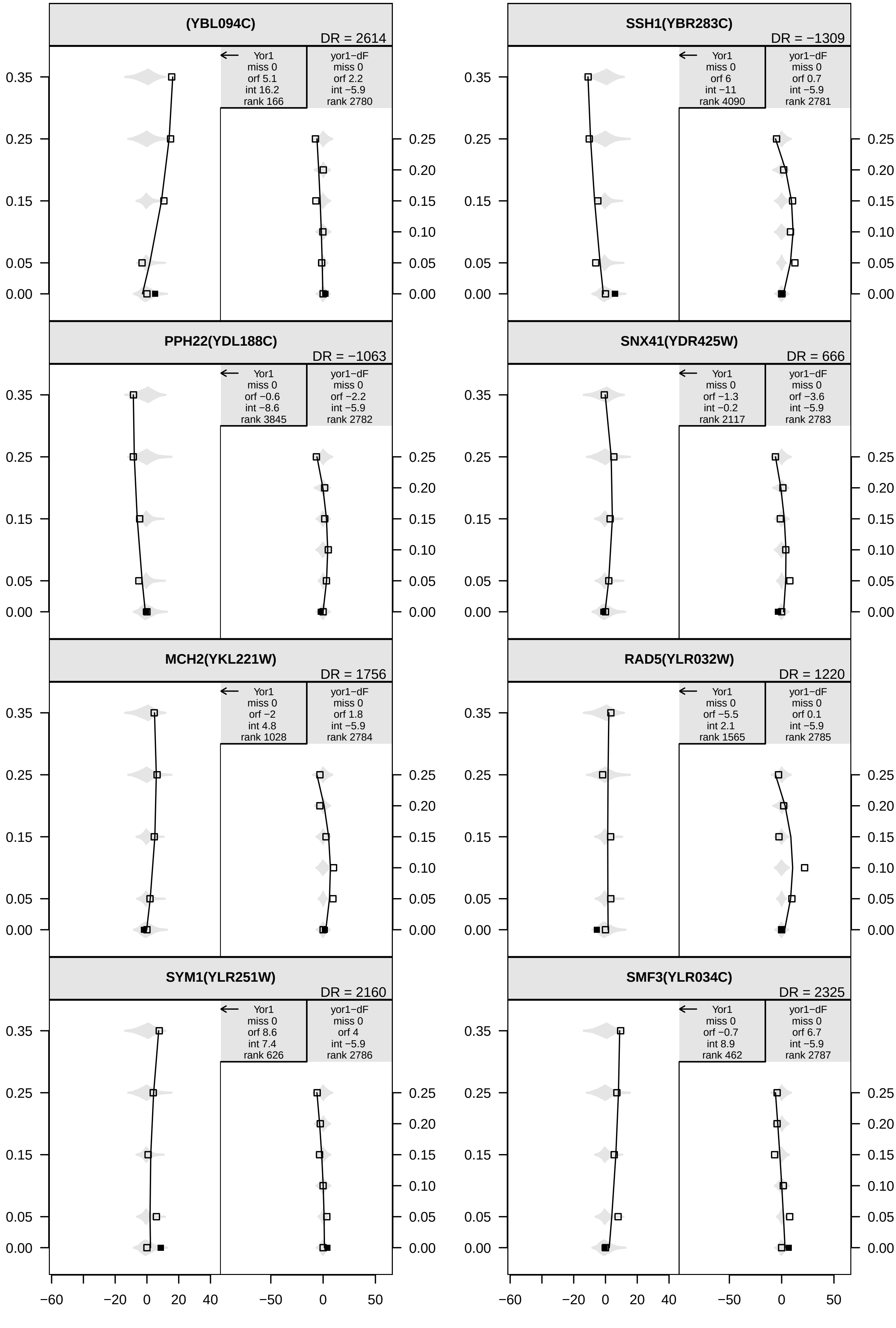

(YBL094C)
DR = 2614
Yor1
miss 0
orf 5.1
int 16.2
rank 166
yor1−dF
miss 0
orf 2.2
int −5.9
rank 2780
SSH1(YBR283C)
DR = −1309
Yor1
miss 0
orf 6
int −11
rank 4090
yor1−dF
miss 0
orf 0.7
int −5.9
rank 2781
PPH22(YDL188C)
DR = −1063
Yor1
miss 0
orf −0.6
int −8.6
rank 3845
yor1−dF
miss 0
orf −2.2
int −5.9
rank 2782
SNX41(YDR425W)
DR = 666
Yor1
miss 0
orf −1.3
int −0.2
rank 2117
yor1−dF
miss 0
orf −3.6
int −5.9
rank 2783
MCH2(YKL221W)
DR = 1756
Yor1
miss 0
orf −2
int 4.8
rank 1028
yor1−dF
miss 0
orf 1.8
int −5.9
rank 2784
RAD5(YLR032W)
DR = 1220
Yor1
miss 0
orf −5.5
int 2.1
rank 1565
yor1−dF
miss 0
orf 0.1
int −5.9
rank 2785
SYM1(YLR251W)
DR = 2160
Yor1
miss 0
orf 8.6
int 7.4
rank 626
yor1−dF
miss 0
orf 4
int −5.9
rank 2786
SMF3(YLR034C)
DR = 2325
Yor1
miss 0
orf −0.7
int 8.9
rank 462
yor1−dF
miss 0
orf 6.7
int −5.9
rank 2787
0.35
0.25
0.20
0.15
0.10
0.05
0.00
−60
−20
0
20
40
−50
0
50

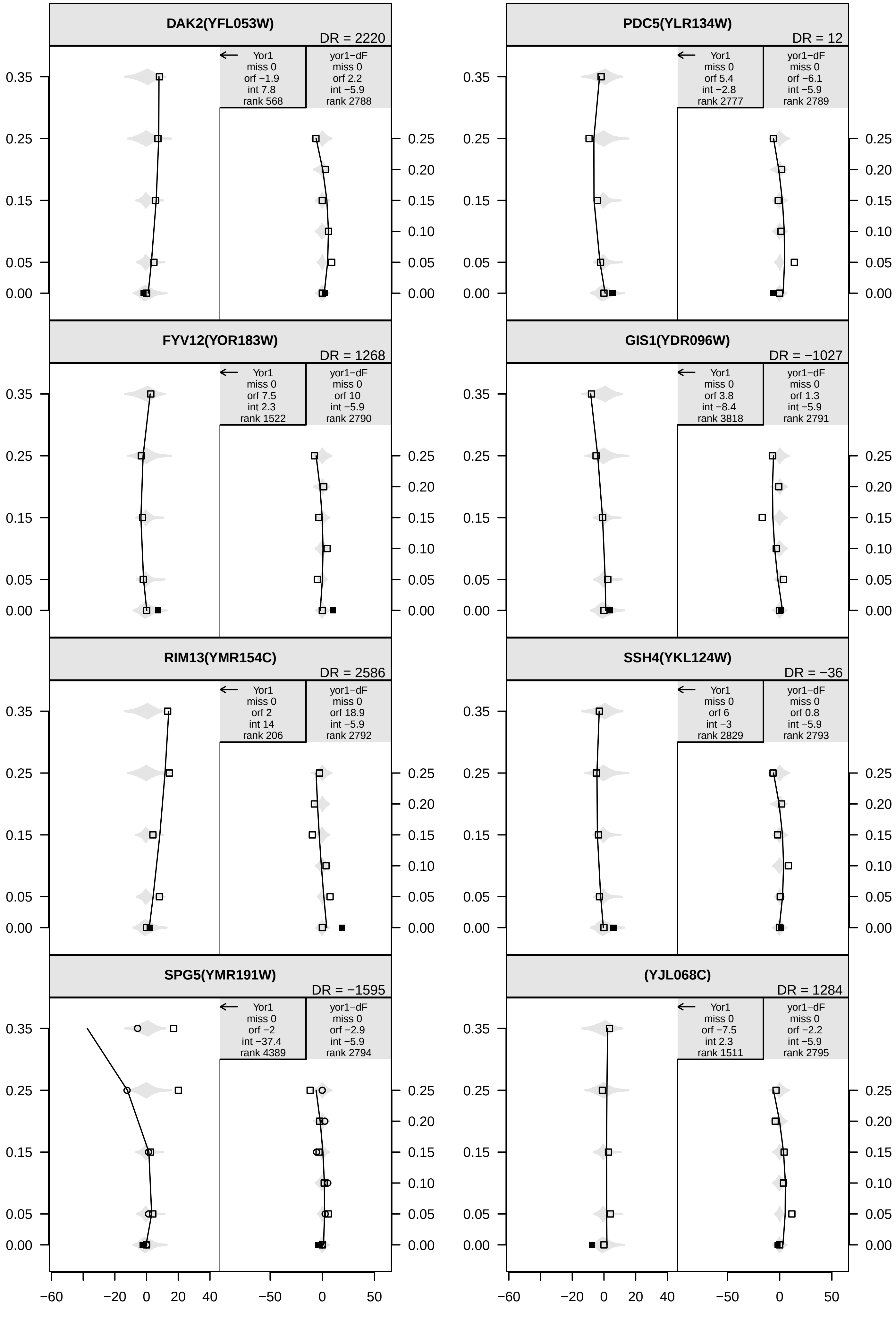

DAK2(YFL053W)
DR = 2220
Yor1 miss 0 orf −1.9 int 7.8 rank 568
yor1−dF miss 0 orf 2.2 int −5.9 rank 2788
PDC5(YLR134W)
DR = 12
Yor1 miss 0 orf 5.4 int −2.8 rank 2777
yor1−dF miss 0 orf −6.1 int −5.9 rank 2789
FYV12(YOR183W)
DR = 1268
Yor1 miss 0 orf 7.5 int 2.3 rank 1522
yor1−dF miss 0 orf 10 int −5.9 rank 2790
GIS1(YDR096W)
DR = −1027
Yor1 miss 0 orf 3.8 int −8.4 rank 3818
yor1−dF miss 0 orf 1.3 int −5.9 rank 2791
RIM13(YMR154C)
DR = 2586
Yor1 miss 0 orf 2 int 14 rank 206
yor1−dF miss 0 orf 18.9 int −5.9 rank 2792
SSH4(YKL124W)
DR = −36
Yor1 miss 0 orf 6 int −3 rank 2829
yor1−dF miss 0 orf 0.8 int −5.9 rank 2793
SPG5(YMR191W)
DR = −1595
Yor1 miss 0 orf −2 int −37.4 rank 4389
yor1−dF miss 0 orf −2.9 int −5.9 rank 2794
(YJL068C)
DR = 1284
Yor1 miss 0 orf −7.5 int 2.3 rank 1511
yor1−dF miss 0 orf −2.2 int −5.9 rank 2795
0.35
0.25
0.20
0.15
0.10
0.05
0.00
−60
−20
0
20
40
−50
0
50

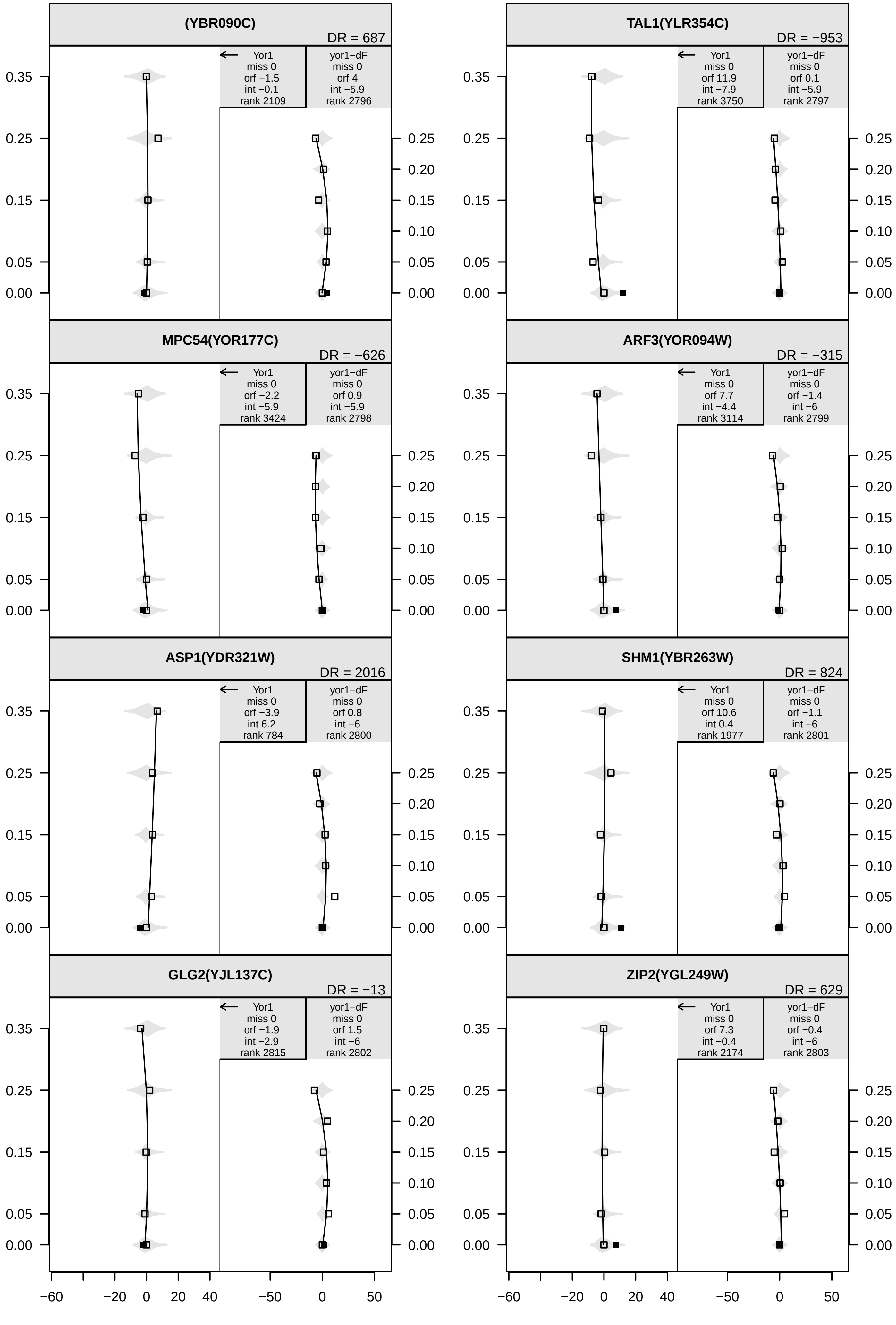
(YBR090C)
DR = 687
Yor1 miss 0 orf −1.5 int −0.1 rank 2109
yor1−dF miss 0 orf 4 int −5.9 rank 2796
TAL1(YLR354C)
DR = −953
Yor1 miss 0 orf 11.9 int −7.9 rank 3750
yor1−dF miss 0 orf 0.1 int −5.9 rank 2797
MPC54(YOR177C)
DR = −626
Yor1 miss 0 orf −2.2 int −5.9 rank 3424
yor1−dF miss 0 orf 0.9 int −5.9 rank 2798
ARF3(YOR094W)
DR = −315
Yor1 miss 0 orf 7.7 int −4.4 rank 3114
yor1−dF miss 0 orf −1.4 int −6 rank 2799
ASP1(YDR321W)
DR = 2016
Yor1 miss 0 orf −3.9 int 6.2 rank 784
yor1−dF miss 0 orf 0.8 int −6 rank 2800
SHM1(YBR263W)
DR = 824
Yor1 miss 0 orf 10.6 int 0.4 rank 1977
yor1−dF miss 0 orf −1.1 int −6 rank 2801
GLG2(YJL137C)
DR = −13
Yor1 miss 0 orf −1.9 int −2.9 rank 2815
yor1−dF miss 0 orf 1.5 int −6 rank 2802
ZIP2(YGL249W)
DR = 629
Yor1 miss 0 orf 7.3 int −0.4 rank 2174
yor1−dF miss 0 orf −0.4 int −6 rank 2803
0.35 0.25 0.20 0.15 0.10 0.05 0.00
−60 −20 0 20 40 −50 0 50

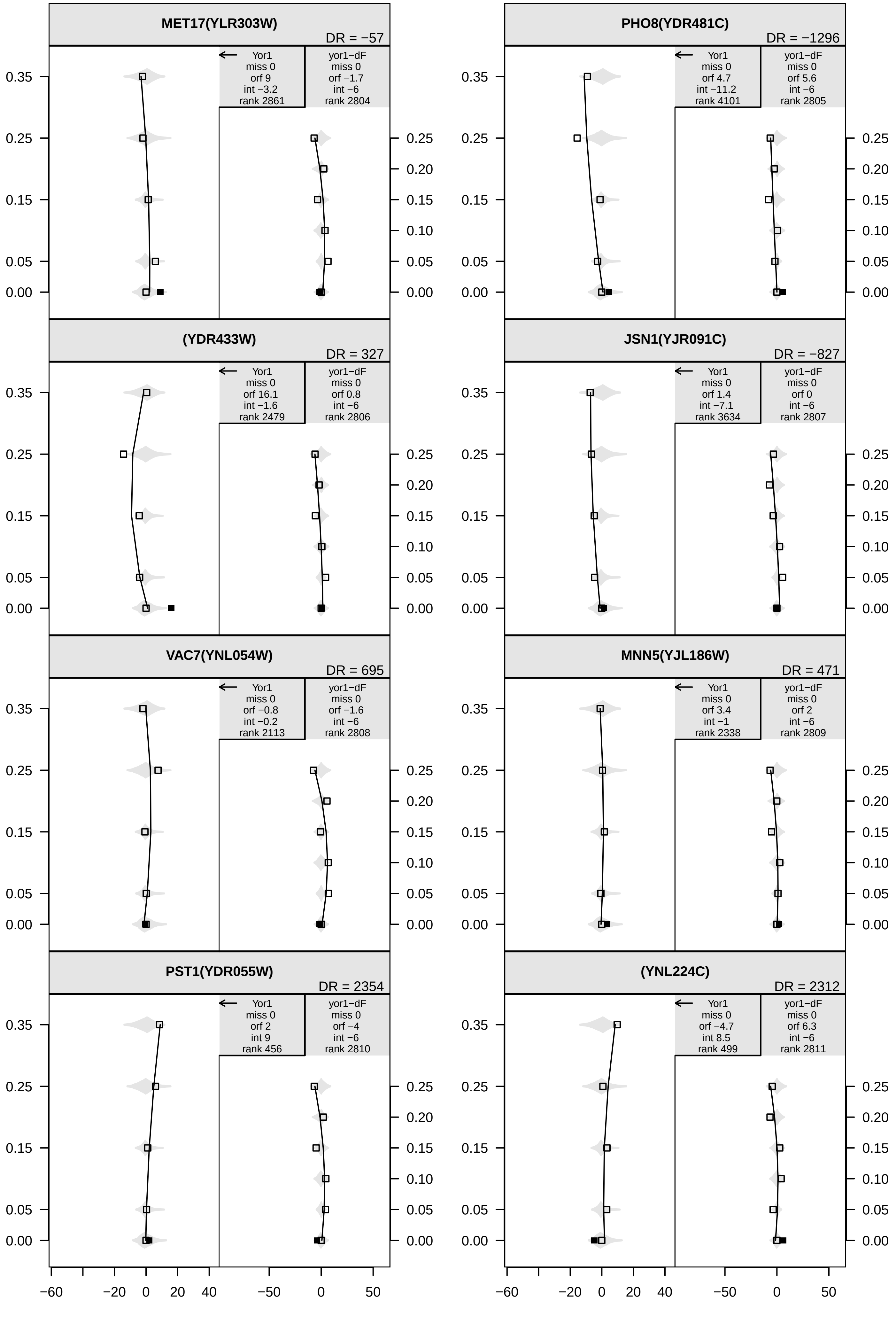
MET17(YLR303W)
DR = −57
Yor1 miss 0 orf 9 int −3.2 rank 2861
yor1−dF miss 0 orf −1.7 int −6 rank 2804
PHO8(YDR481C)
DR = −1296
Yor1 miss 0 orf 4.7 int −11.2 rank 4101
yor1−dF miss 0 orf 5.6 int −6 rank 2805
(YDR433W)
DR = 327
Yor1 miss 0 orf 16.1 int −1.6 rank 2479
yor1−dF miss 0 orf 0.8 int −6 rank 2806
JSN1(YJR091C)
DR = −827
Yor1 miss 0 orf 1.4 int −7.1 rank 3634
yor1−dF miss 0 orf 0 int −6 rank 2807
VAC7(YNL054W)
DR = 695
Yor1 miss 0 orf −0.8 int −0.2 rank 2113
yor1−dF miss 0 orf −1.6 int −6 rank 2808
MNN5(YJL186W)
DR = 471
Yor1 miss 0 orf 3.4 int −1 rank 2338
yor1−dF miss 0 orf 2 int −6 rank 2809
PST1(YDR055W)
DR = 2354
Yor1 miss 0 orf 2 int 9 rank 456
yor1−dF miss 0 orf −4 int −6 rank 2810
(YNL224C)
DR = 2312
Yor1 miss 0 orf −4.7 int 8.5 rank 499
yor1−dF miss 0 orf 6.3 int −6 rank 2811
0.00 0.05 0.10 0.15 0.20 0.25 0.35
−60 −20 0 20 40 −50 0 50

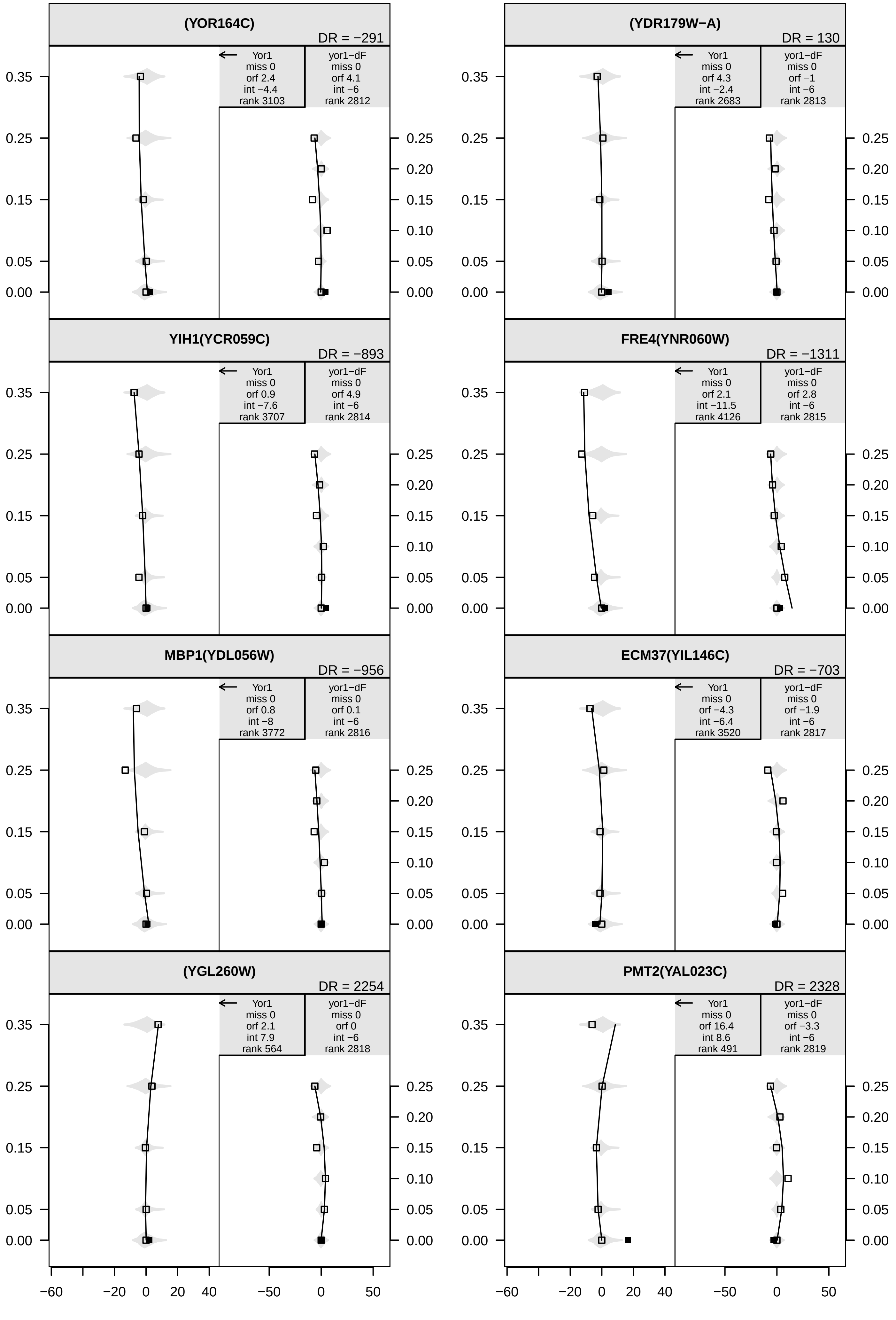

(YOR164C)
DR = −291
Yor1
miss 0
orf 2.4
int −4.4
rank 3103
yor1−dF
miss 0
orf 4.1
int −6
rank 2812
(YDR179W−A)
DR = 130
Yor1
miss 0
orf 4.3
int −2.4
rank 2683
yor1−dF
miss 0
orf −1
int −6
rank 2813
YIH1(YCR059C)
DR = −893
Yor1
miss 0
orf 0.9
int −7.6
rank 3707
yor1−dF
miss 0
orf 4.9
int −6
rank 2814
FRE4(YNR060W)
DR = −1311
Yor1
miss 0
orf 2.1
int −11.5
rank 4126
yor1−dF
miss 0
orf 2.8
int −6
rank 2815
MBP1(YDL056W)
DR = −956
Yor1
miss 0
orf 0.8
int −8
rank 3772
yor1−dF
miss 0
orf 0.1
int −6
rank 2816
ECM37(YIL146C)
DR = −703
Yor1
miss 0
orf −4.3
int −6.4
rank 3520
yor1−dF
miss 0
orf −1.9
int −6
rank 2817
(YGL260W)
DR = 2254
Yor1
miss 0
orf 2.1
int 7.9
rank 564
yor1−dF
miss 0
orf 0
int −6
rank 2818
PMT2(YAL023C)
DR = 2328
Yor1
miss 0
orf 16.4
int 8.6
rank 491
yor1−dF
miss 0
orf −3.3
int −6
rank 2819
0.35
0.25
0.20
0.15
0.10
0.05
0.00
−60
−20
0
20
40
−50
0
50

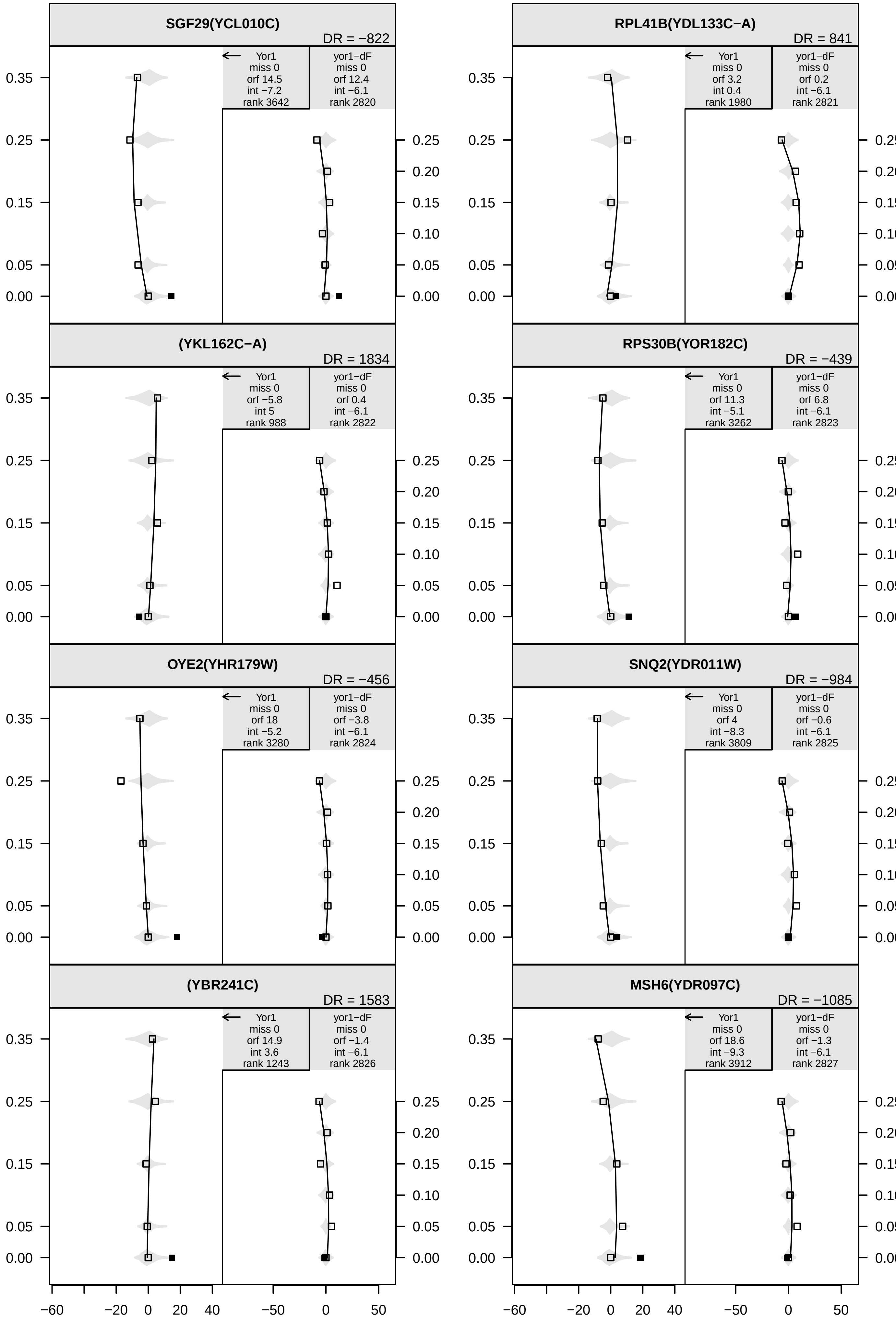
SGF29(YCL010C)
DR = −822
Yor1
miss 0
orf 14.5
int −7.2
rank 3642
yor1−dF
miss 0
orf 12.4
int −6.1
rank 2820
RPL41B(YDL133C−A)
DR = 841
Yor1
miss 0
orf 3.2
int 0.4
rank 1980
yor1−dF
miss 0
orf 0.2
int −6.1
rank 2821
(YKL162C−A)
DR = 1834
Yor1
miss 0
orf −5.8
int 5
rank 988
yor1−dF
miss 0
orf 0.4
int −6.1
rank 2822
RPS30B(YOR182C)
DR = −439
Yor1
miss 0
orf 11.3
int −5.1
rank 3262
yor1−dF
miss 0
orf 6.8
int −6.1
rank 2823
OYE2(YHR179W)
DR = −456
Yor1
miss 0
orf 18
int −5.2
rank 3280
yor1−dF
miss 0
orf −3.8
int −6.1
rank 2824
SNQ2(YDR011W)
DR = −984
Yor1
miss 0
orf 4
int −8.3
rank 3809
yor1−dF
miss 0
orf −0.6
int −6.1
rank 2825
(YBR241C)
DR = 1583
Yor1
miss 0
orf 14.9
int 3.6
rank 1243
yor1−dF
miss 0
orf −1.4
int −6.1
rank 2826
MSH6(YDR097C)
DR = −1085
Yor1
miss 0
orf 18.6
int −9.3
rank 3912
yor1−dF
miss 0
orf −1.3
int −6.1
rank 2827
0.35
0.25
0.20
0.15
0.10
0.05
0.00
−60
−20
0
20
40
−50
0
50

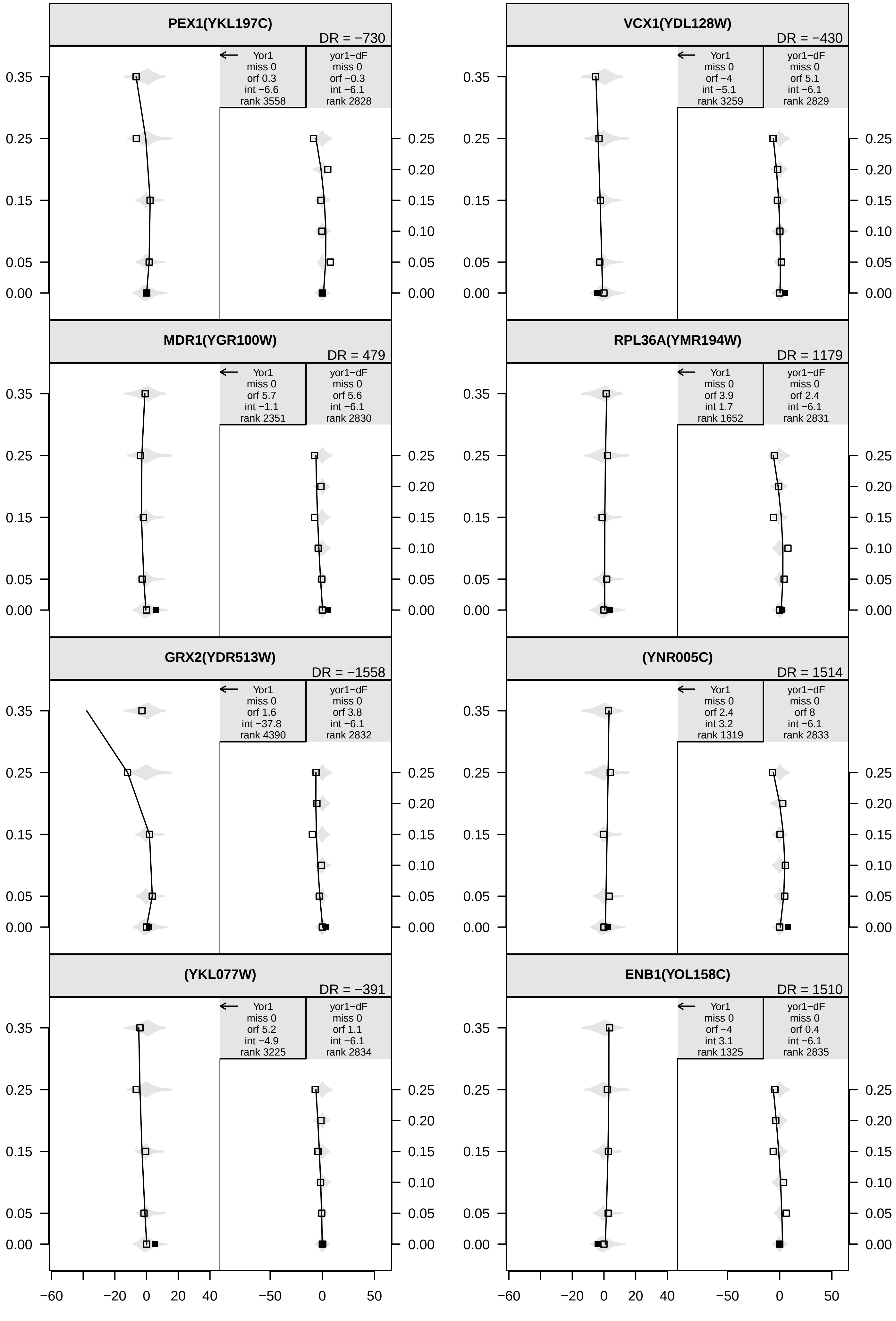

PEX1(YKL197C)
DR = −730
Yor1 miss 0 orf 0.3 int −6.6 rank 3558
yor1−dF miss 0 orf −0.3 int −6.1 rank 2828
VCX1(YDL128W)
DR = −430
Yor1 miss 0 orf −4 int −5.1 rank 3259
yor1−dF miss 0 orf 5.1 int −6.1 rank 2829
MDR1(YGR100W)
DR = 479
Yor1 miss 0 orf 5.7 int −1.1 rank 2351
yor1−dF miss 0 orf 5.6 int −6.1 rank 2830
RPL36A(YMR194W)
DR = 1179
Yor1 miss 0 orf 3.9 int 1.7 rank 1652
yor1−dF miss 0 orf 2.4 int −6.1 rank 2831
GRX2(YDR513W)
DR = −1558
Yor1 miss 0 orf 1.6 int −37.8 rank 4390
yor1−dF miss 0 orf 3.8 int −6.1 rank 2832
(YNR005C)
DR = 1514
Yor1 miss 0 orf 2.4 int 3.2 rank 1319
yor1−dF miss 0 orf 8 int −6.1 rank 2833
(YKL077W)
DR = −391
Yor1 miss 0 orf 5.2 int −4.9 rank 3225
yor1−dF miss 0 orf 1.1 int −6.1 rank 2834
ENB1(YOL158C)
DR = 1510
Yor1 miss 0 orf −4 int 3.1 rank 1325
yor1−dF miss 0 orf 0.4 int −6.1 rank 2835
0.00 0.05 0.10 0.15 0.20 0.25 0.35
−60 −20 0 20 40
−50 0 50

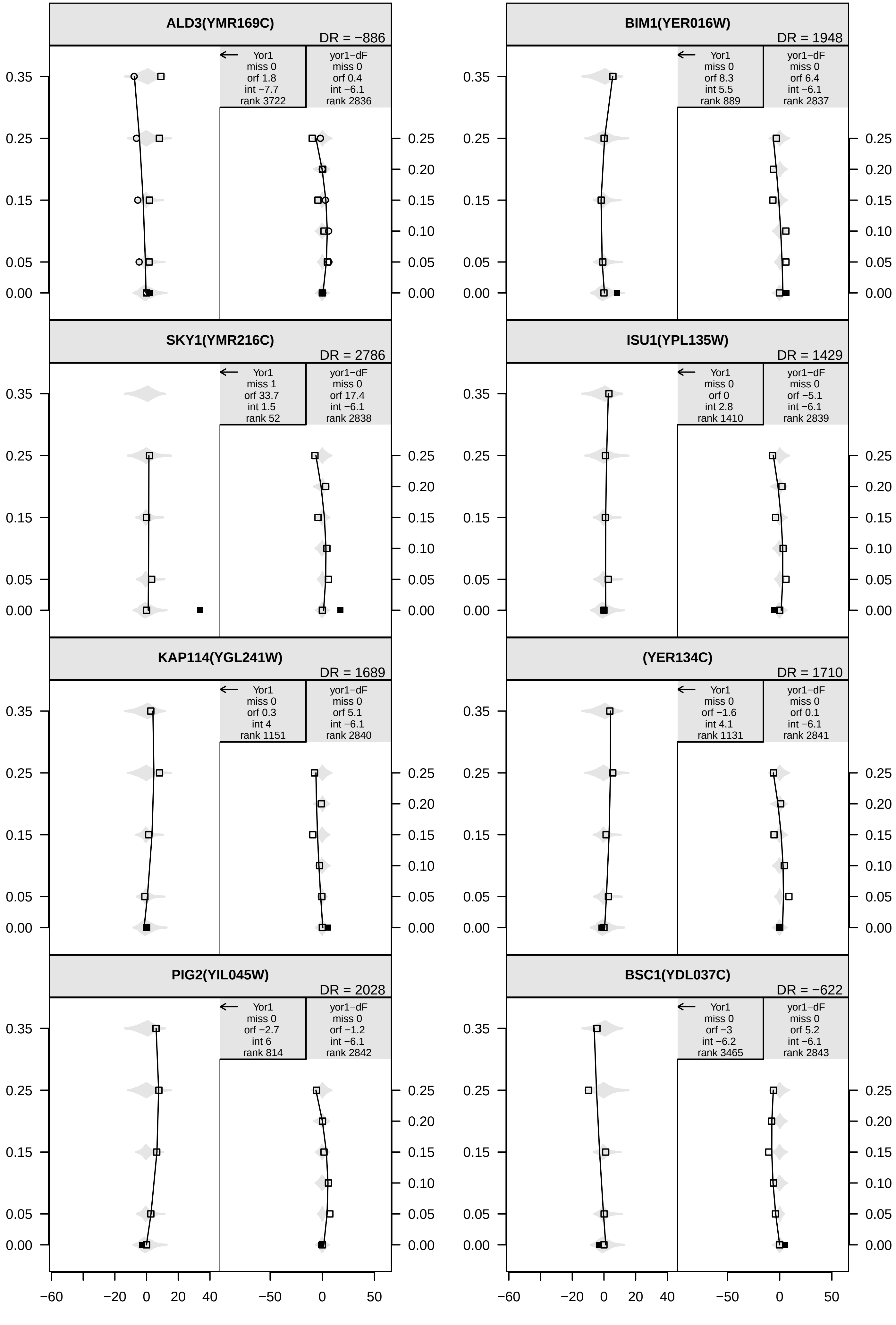

ALD3(YMR169C)
DR = −886
Yor1 miss 0 orf 1.8 int −7.7 rank 3722
yor1−dF miss 0 orf 0.4 int −6.1 rank 2836
BIM1(YER016W)
DR = 1948
Yor1 miss 0 orf 8.3 int 5.5 rank 889
yor1−dF miss 0 orf 6.4 int −6.1 rank 2837
SKY1(YMR216C)
DR = 2786
Yor1 miss 1 orf 33.7 int 1.5 rank 52
yor1−dF miss 0 orf 17.4 int −6.1 rank 2838
ISU1(YPL135W)
DR = 1429
Yor1 miss 0 orf 0 int 2.8 rank 1410
yor1−dF miss 0 orf −5.1 int −6.1 rank 2839
KAP114(YGL241W)
DR = 1689
Yor1 miss 0 orf 0.3 int 4 rank 1151
yor1−dF miss 0 orf 5.1 int −6.1 rank 2840
(YER134C)
DR = 1710
Yor1 miss 0 orf −1.6 int 4.1 rank 1131
yor1−dF miss 0 orf 0.1 int −6.1 rank 2841
PIG2(YIL045W)
DR = 2028
Yor1 miss 0 orf −2.7 int 6 rank 814
yor1−dF miss 0 orf −1.2 int −6.1 rank 2842
BSC1(YDL037C)
DR = −622
Yor1 miss 0 orf −3 int −6.2 rank 3465
yor1−dF miss 0 orf 5.2 int −6.1 rank 2843
0.35 0.25 0.15 0.05 0.00
0.25 0.20 0.15 0.10 0.05 0.00
−60 −20 0 20 40
−50 0 50

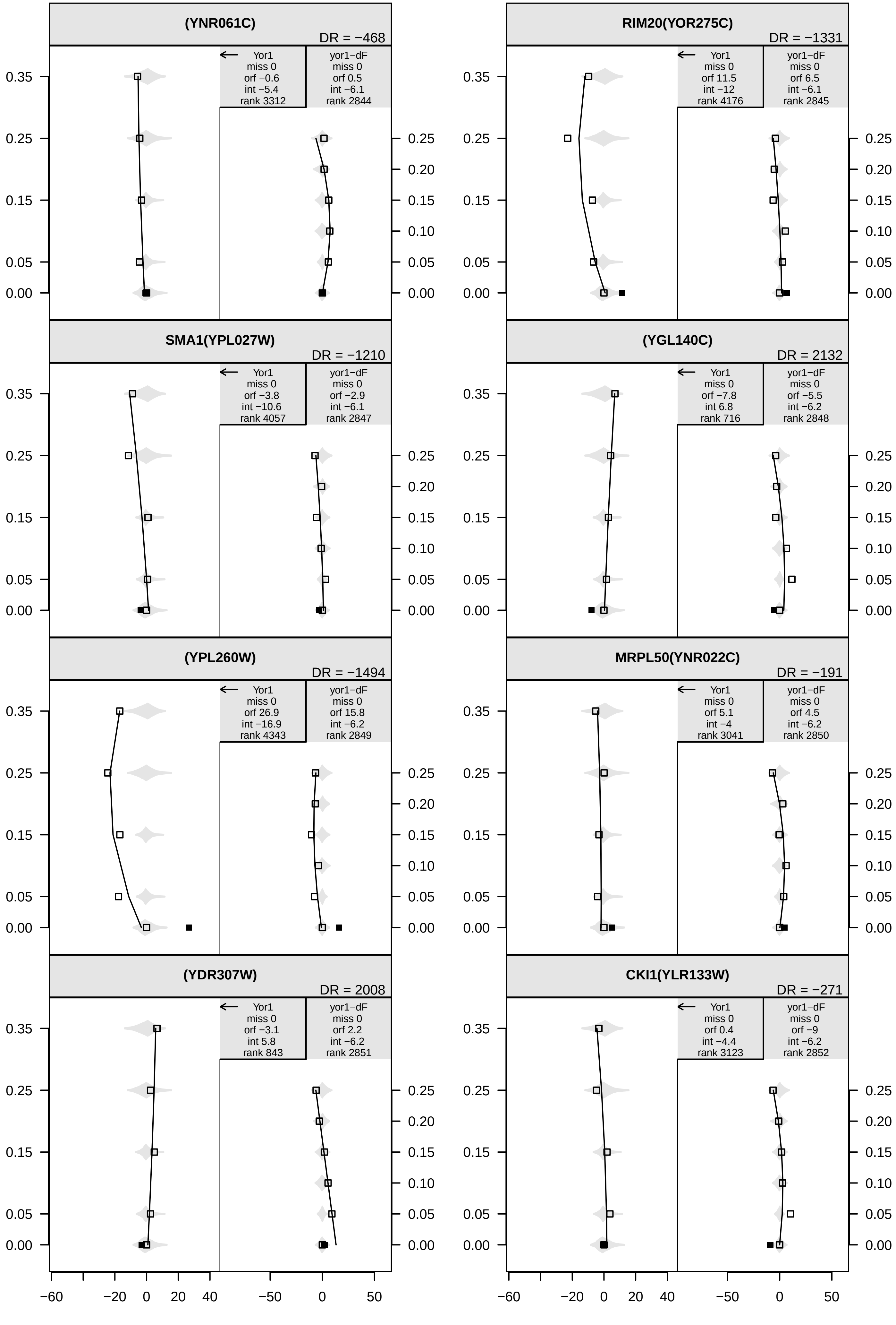
(YNR061C)
DR = −468
Yor1 miss 0 orf −0.6 int −5.4 rank 3312
yor1−dF miss 0 orf 0.5 int −6.1 rank 2844
RIM20(YOR275C)
DR = −1331
Yor1 miss 0 orf 11.5 int −12 rank 4176
yor1−dF miss 0 orf 6.5 int −6.1 rank 2845
SMA1(YPL027W)
DR = −1210
Yor1 miss 0 orf −3.8 int −10.6 rank 4057
yor1−dF miss 0 orf −2.9 int −6.1 rank 2847
(YGL140C)
DR = 2132
Yor1 miss 0 orf −7.8 int 6.8 rank 716
yor1−dF miss 0 orf −5.5 int −6.2 rank 2848
(YPL260W)
DR = −1494
Yor1 miss 0 orf 26.9 int −16.9 rank 4343
yor1−dF miss 0 orf 15.8 int −6.2 rank 2849
MRPL50(YNR022C)
DR = −191
Yor1 miss 0 orf 5.1 int −4 rank 3041
yor1−dF miss 0 orf 4.5 int −6.2 rank 2850
(YDR307W)
DR = 2008
Yor1 miss 0 orf −3.1 int 5.8 rank 843
yor1−dF miss 0 orf 2.2 int −6.2 rank 2851
CKI1(YLR133W)
DR = −271
Yor1 miss 0 orf 0.4 int −4.4 rank 3123
yor1−dF miss 0 orf −9 int −6.2 rank 2852
0.35 0.25 0.15 0.05 0.00
0.25 0.20 0.15 0.10 0.05 0.00
−60 −20 0 20 40 −50 0 50

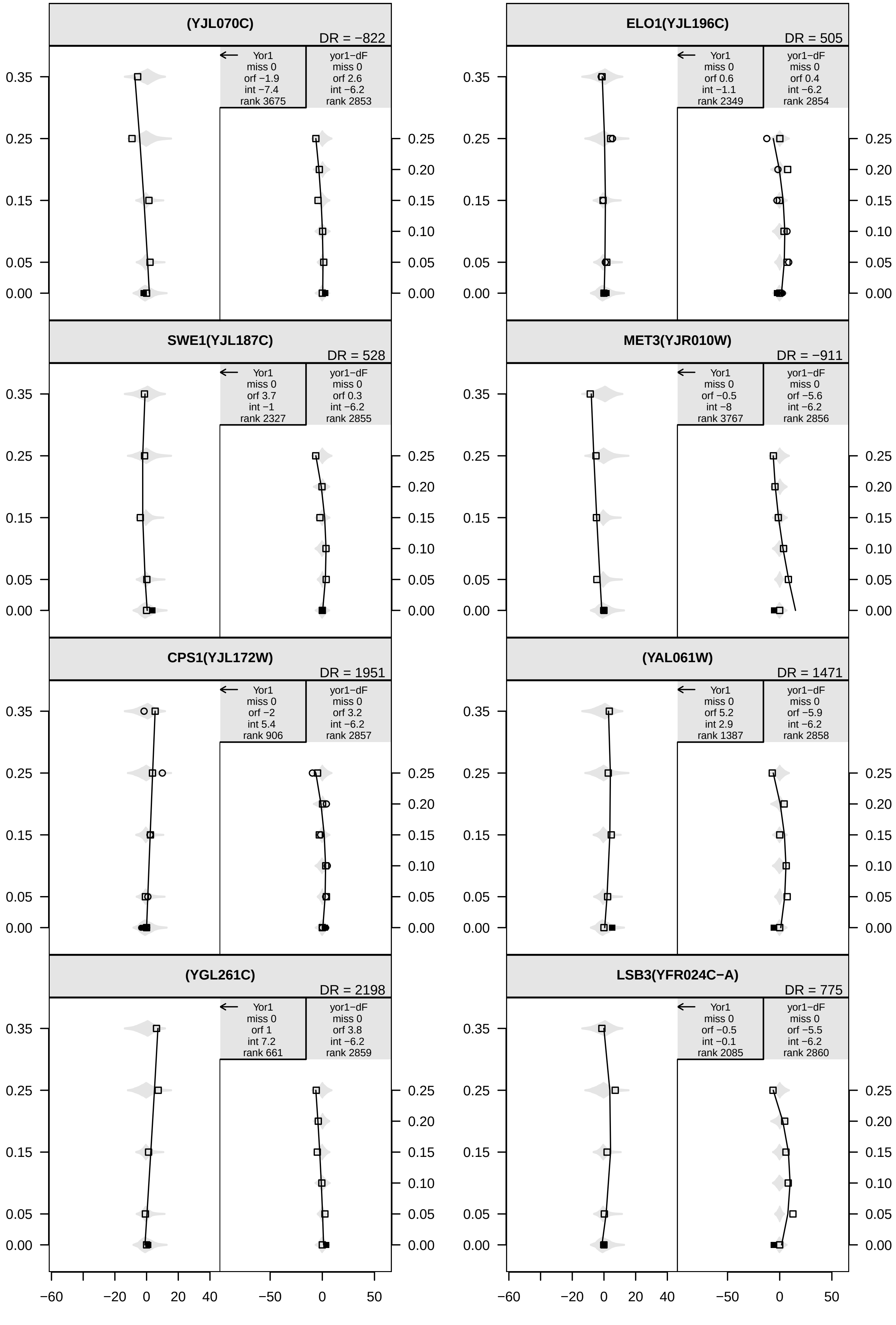
(YJL070C)
DR = −822
Yor1 miss 0 orf −1.9 int −7.4 rank 3675
yor1−dF miss 0 orf 2.6 int −6.2 rank 2853
ELO1(YJL196C)
DR = 505
Yor1 miss 0 orf 0.6 int −1.1 rank 2349
yor1−dF miss 0 orf 0.4 int −6.2 rank 2854
SWE1(YJL187C)
DR = 528
Yor1 miss 0 orf 3.7 int −1 rank 2327
yor1−dF miss 0 orf 0.3 int −6.2 rank 2855
MET3(YJR010W)
DR = −911
Yor1 miss 0 orf −0.5 int −8 rank 3767
yor1−dF miss 0 orf −5.6 int −6.2 rank 2856
CPS1(YJL172W)
DR = 1951
Yor1 miss 0 orf −2 int 5.4 rank 906
yor1−dF miss 0 orf 3.2 int −6.2 rank 2857
(YAL061W)
DR = 1471
Yor1 miss 0 orf 5.2 int 2.9 rank 1387
yor1−dF miss 0 orf −5.9 int −6.2 rank 2858
(YGL261C)
DR = 2198
Yor1 miss 0 orf 1 int 7.2 rank 661
yor1−dF miss 0 orf 3.8 int −6.2 rank 2859
LSB3(YFR024C−A)
DR = 775
Yor1 miss 0 orf −0.5 int −0.1 rank 2085
yor1−dF miss 0 orf −5.5 int −6.2 rank 2860
0.00 0.05 0.10 0.15 0.20 0.25 0.35
−60 −20 0 20 40 −50 0 50

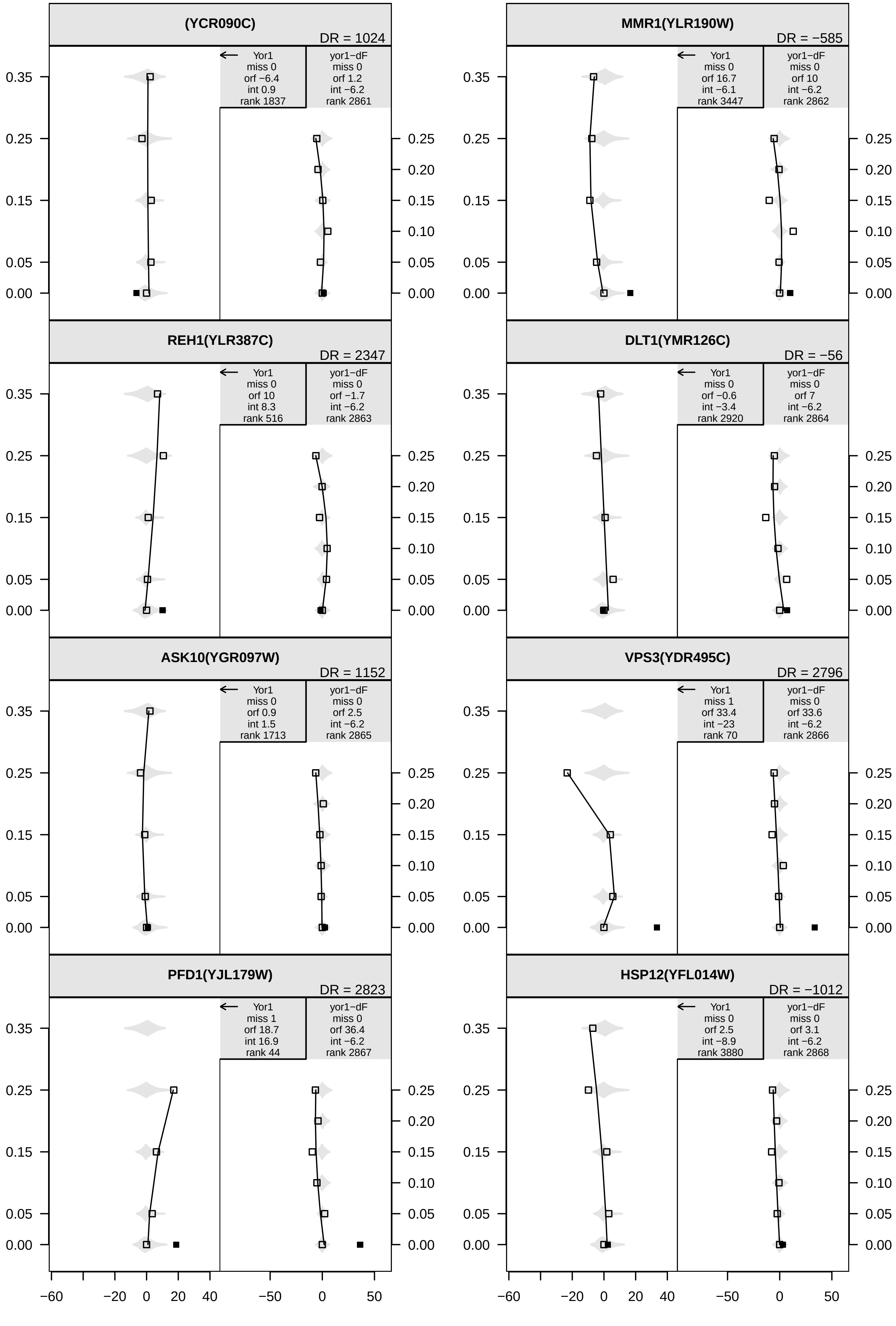

(YCR090C)
DR = 1024
Yor1 miss 0 orf −6.4 int 0.9 rank 1837
yor1−dF miss 0 orf 1.2 int −6.2 rank 2861
MMR1(YLR190W)
DR = −585
Yor1 miss 0 orf 16.7 int −6.1 rank 3447
yor1−dF miss 0 orf 10 int −6.2 rank 2862
REH1(YLR387C)
DR = 2347
Yor1 miss 0 orf 10 int 8.3 rank 516
yor1−dF miss 0 orf −1.7 int −6.2 rank 2863
DLT1(YMR126C)
DR = −56
Yor1 miss 0 orf −0.6 int −3.4 rank 2920
yor1−dF miss 0 orf 7 int −6.2 rank 2864
ASK10(YGR097W)
DR = 1152
Yor1 miss 0 orf 0.9 int 1.5 rank 1713
yor1−dF miss 0 orf 2.5 int −6.2 rank 2865
VPS3(YDR495C)
DR = 2796
Yor1 miss 1 orf 33.4 int −23 rank 70
yor1−dF miss 0 orf 33.6 int −6.2 rank 2866
PFD1(YJL179W)
DR = 2823
Yor1 miss 1 orf 18.7 int 16.9 rank 44
yor1−dF miss 0 orf 36.4 int −6.2 rank 2867
HSP12(YFL014W)
DR = −1012
Yor1 miss 0 orf 2.5 int −8.9 rank 3880
yor1−dF miss 0 orf 3.1 int −6.2 rank 2868
0.35 0.25 0.15 0.05 0.00
0.25 0.20 0.15 0.10 0.05 0.00
−60 −20 0 20 40
−50 0 50

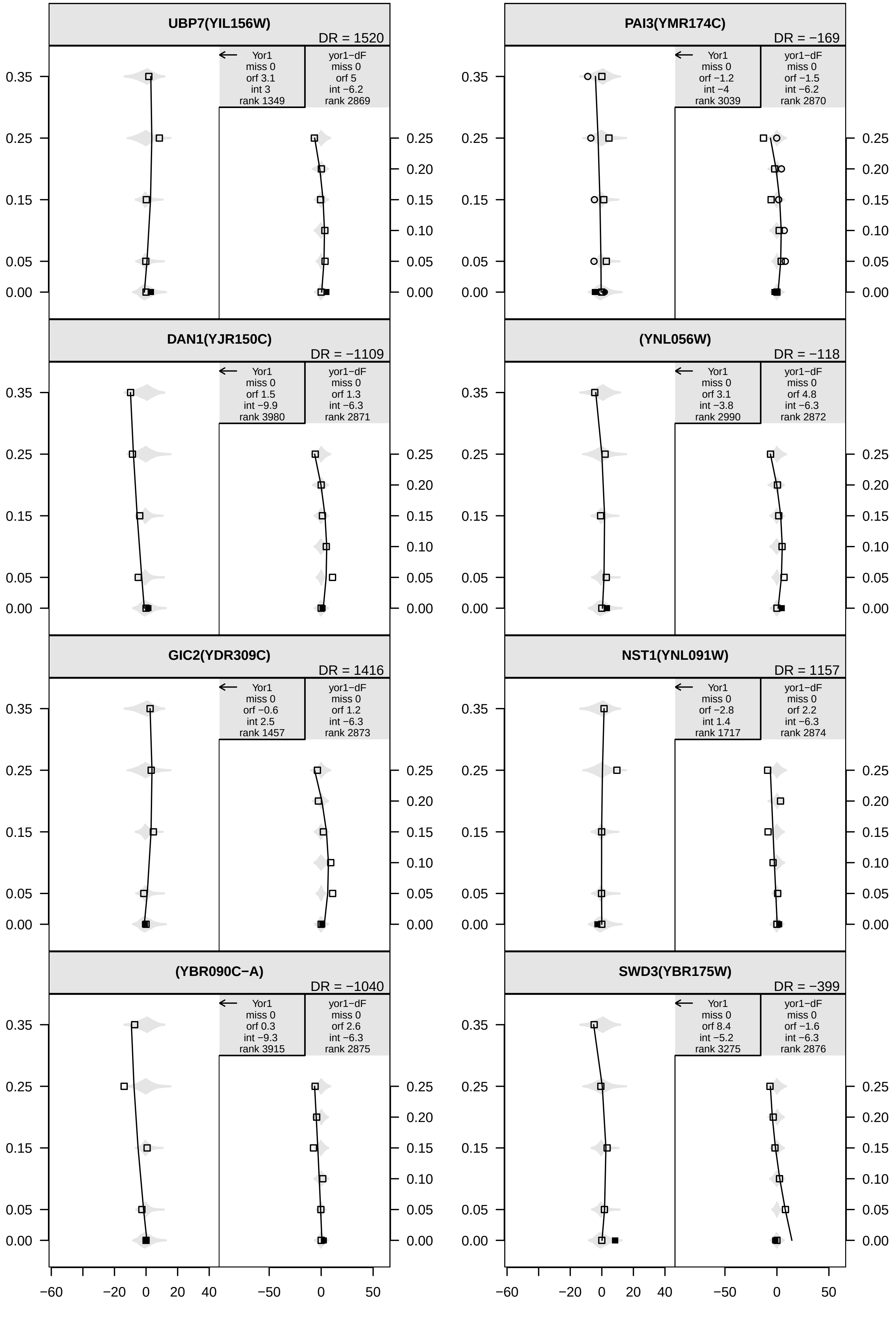
UBP7(YIL156W)
DR = 1520
Yor1 miss 0 orf 3.1 int 3 rank 1349
yor1−dF miss 0 orf 5 int −6.2 rank 2869
PAI3(YMR174C)
DR = −169
Yor1 miss 0 orf −1.2 int −4 rank 3039
yor1−dF miss 0 orf −1.5 int −6.2 rank 2870
DAN1(YJR150C)
DR = −1109
Yor1 miss 0 orf 1.5 int −9.9 rank 3980
yor1−dF miss 0 orf 1.3 int −6.3 rank 2871
(YNL056W)
DR = −118
Yor1 miss 0 orf 3.1 int −3.8 rank 2990
yor1−dF miss 0 orf 4.8 int −6.3 rank 2872
GIC2(YDR309C)
DR = 1416
Yor1 miss 0 orf −0.6 int 2.5 rank 1457
yor1−dF miss 0 orf 1.2 int −6.3 rank 2873
NST1(YNL091W)
DR = 1157
Yor1 miss 0 orf −2.8 int 1.4 rank 1717
yor1−dF miss 0 orf 2.2 int −6.3 rank 2874
(YBR090C−A)
DR = −1040
Yor1 miss 0 orf 0.3 int −9.3 rank 3915
yor1−dF miss 0 orf 2.6 int −6.3 rank 2875
SWD3(YBR175W)
DR = −399
Yor1 miss 0 orf 8.4 int −5.2 rank 3275
yor1−dF miss 0 orf −1.6 int −6.3 rank 2876
0.00 0.05 0.10 0.15 0.20 0.25 0.35
−60 −20 0 20 40 −50 0 50

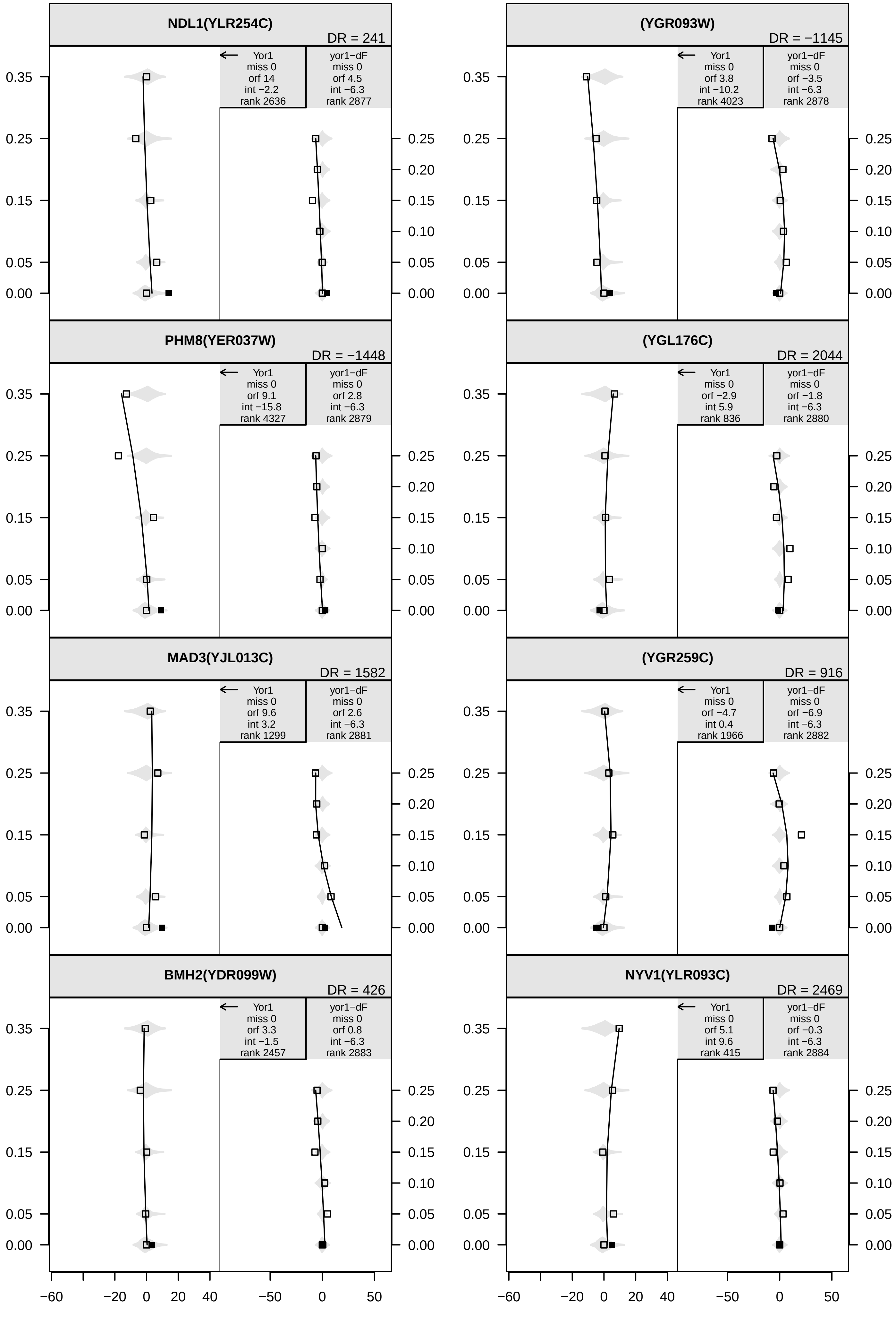
NDL1(YLR254C)
DR = 241
Yor1 miss 0 orf 14 int −2.2 rank 2636
yor1−dF miss 0 orf 4.5 int −6.3 rank 2877
(YGR093W)
DR = −1145
Yor1 miss 0 orf 3.8 int −10.2 rank 4023
yor1−dF miss 0 orf −3.5 int −6.3 rank 2878
PHM8(YER037W)
DR = −1448
Yor1 miss 0 orf 9.1 int −15.8 rank 4327
yor1−dF miss 0 orf 2.8 int −6.3 rank 2879
(YGL176C)
DR = 2044
Yor1 miss 0 orf −2.9 int 5.9 rank 836
yor1−dF miss 0 orf −1.8 int −6.3 rank 2880
MAD3(YJL013C)
DR = 1582
Yor1 miss 0 orf 9.6 int 3.2 rank 1299
yor1−dF miss 0 orf 2.6 int −6.3 rank 2881
(YGR259C)
DR = 916
Yor1 miss 0 orf −4.7 int 0.4 rank 1966
yor1−dF miss 0 orf −6.9 int −6.3 rank 2882
BMH2(YDR099W)
DR = 426
Yor1 miss 0 orf 3.3 int −1.5 rank 2457
yor1−dF miss 0 orf 0.8 int −6.3 rank 2883
NYV1(YLR093C)
DR = 2469
Yor1 miss 0 orf 5.1 int 9.6 rank 415
yor1−dF miss 0 orf −0.3 int −6.3 rank 2884

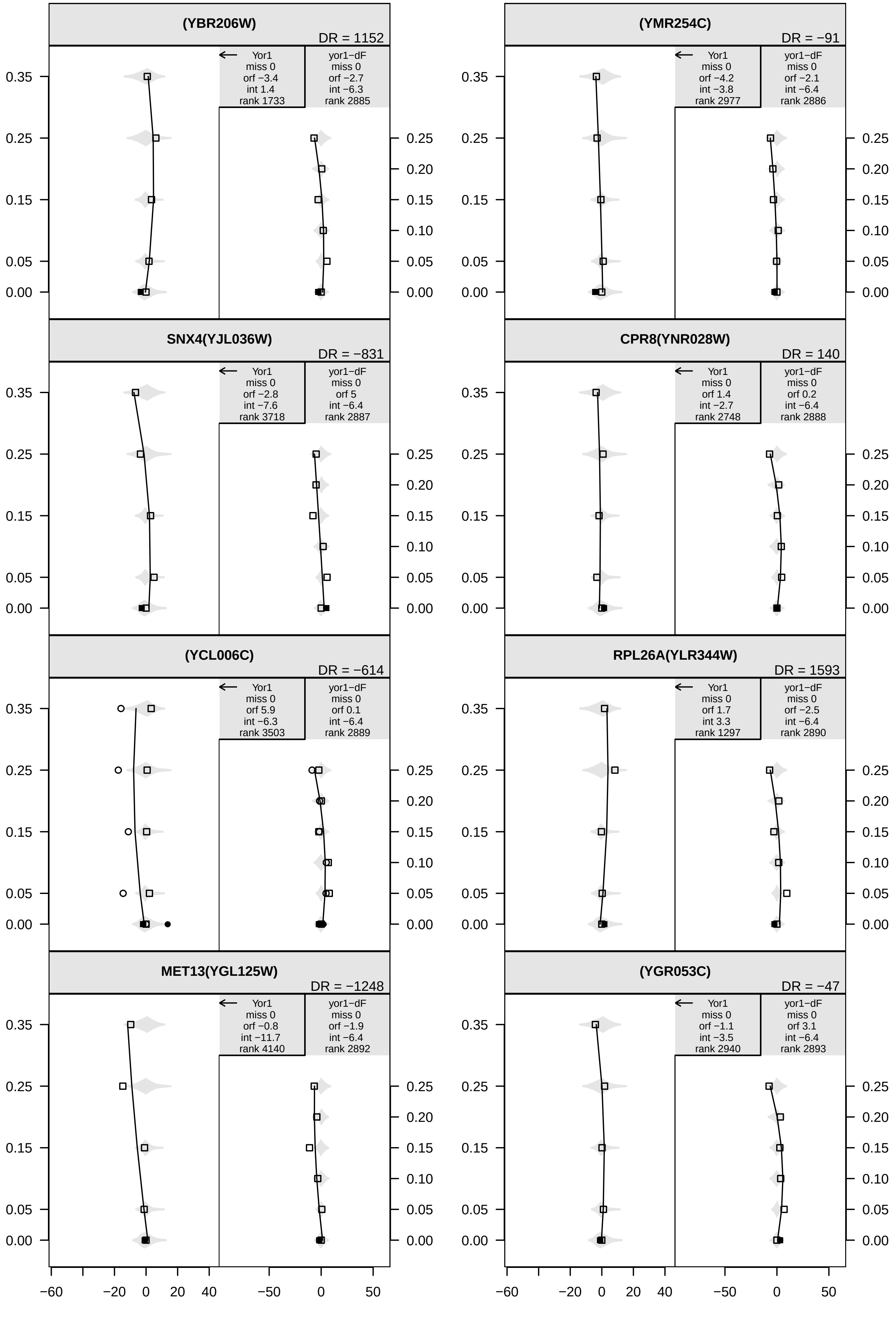
(YBR206W)
DR = 1152
Yor1 miss 0 orf −3.4 int 1.4 rank 1733
yor1−dF miss 0 orf −2.7 int −6.3 rank 2885
(YMR254C)
DR = −91
Yor1 miss 0 orf −4.2 int −3.8 rank 2977
yor1−dF miss 0 orf −2.1 int −6.4 rank 2886
SNX4(YJL036W)
DR = −831
Yor1 miss 0 orf −2.8 int −7.6 rank 3718
yor1−dF miss 0 orf 5 int −6.4 rank 2887
CPR8(YNR028W)
DR = 140
Yor1 miss 0 orf 1.4 int −2.7 rank 2748
yor1−dF miss 0 orf 0.2 int −6.4 rank 2888
(YCL006C)
DR = −614
Yor1 miss 0 orf 5.9 int −6.3 rank 3503
yor1−dF miss 0 orf 0.1 int −6.4 rank 2889
RPL26A(YLR344W)
DR = 1593
Yor1 miss 0 orf 1.7 int 3.3 rank 1297
yor1−dF miss 0 orf −2.5 int −6.4 rank 2890
MET13(YGL125W)
DR = −1248
Yor1 miss 0 orf −0.8 int −11.7 rank 4140
yor1−dF miss 0 orf −1.9 int −6.4 rank 2892
(YGR053C)
DR = −47
Yor1 miss 0 orf −1.1 int −3.5 rank 2940
yor1−dF miss 0 orf 3.1 int −6.4 rank 2893

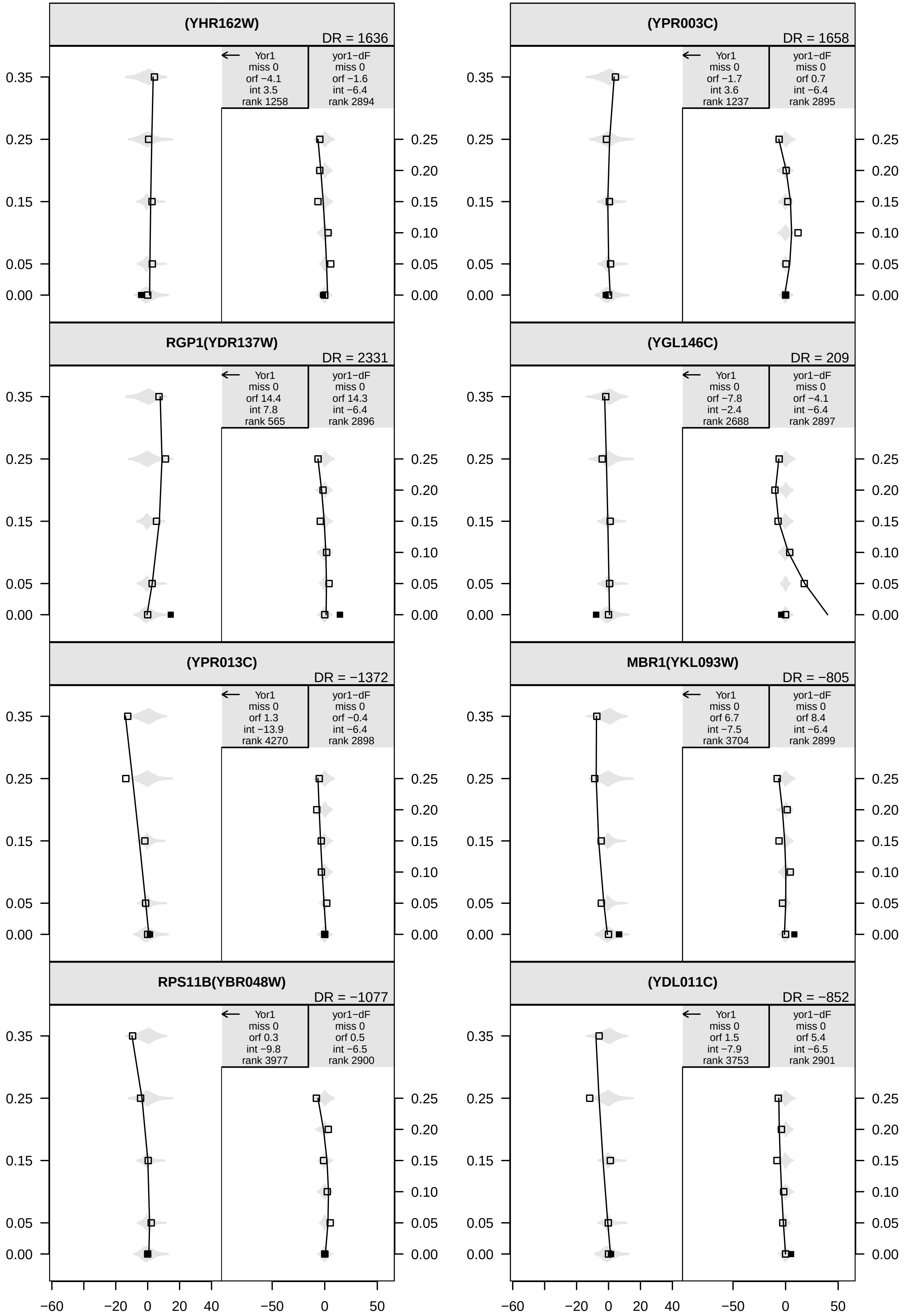
(YHR162W)
DR = 1636
Yor1
miss 0
orf −4.1
int 3.5
rank 1258
yor1−dF
miss 0
orf −1.6
int −6.4
rank 2894
(YPR003C)
DR = 1658
Yor1
miss 0
orf −1.7
int 3.6
rank 1237
yor1−dF
miss 0
orf 0.7
int −6.4
rank 2895
RGP1(YDR137W)
DR = 2331
Yor1
miss 0
orf 14.4
int 7.8
rank 565
yor1−dF
miss 0
orf 14.3
int −6.4
rank 2896
(YGL146C)
DR = 209
Yor1
miss 0
orf −7.8
int −2.4
rank 2688
yor1−dF
miss 0
orf −4.1
int −6.4
rank 2897
(YPR013C)
DR = −1372
Yor1
miss 0
orf 1.3
int −13.9
rank 4270
yor1−dF
miss 0
orf −0.4
int −6.4
rank 2898
MBR1(YKL093W)
DR = −805
Yor1
miss 0
orf 6.7
int −7.5
rank 3704
yor1−dF
miss 0
orf 8.4
int −6.4
rank 2899
RPS11B(YBR048W)
DR = −1077
Yor1
miss 0
orf 0.3
int −9.8
rank 3977
yor1−dF
miss 0
orf 0.5
int −6.5
rank 2900
(YDL011C)
DR = −852
Yor1
miss 0
orf 1.5
int −7.9
rank 3753
yor1−dF
miss 0
orf 5.4
int −6.5
rank 2901
0.35
0.25
0.20
0.15
0.10
0.05
0.00
−60
−20
0
20
40
−50
0
50

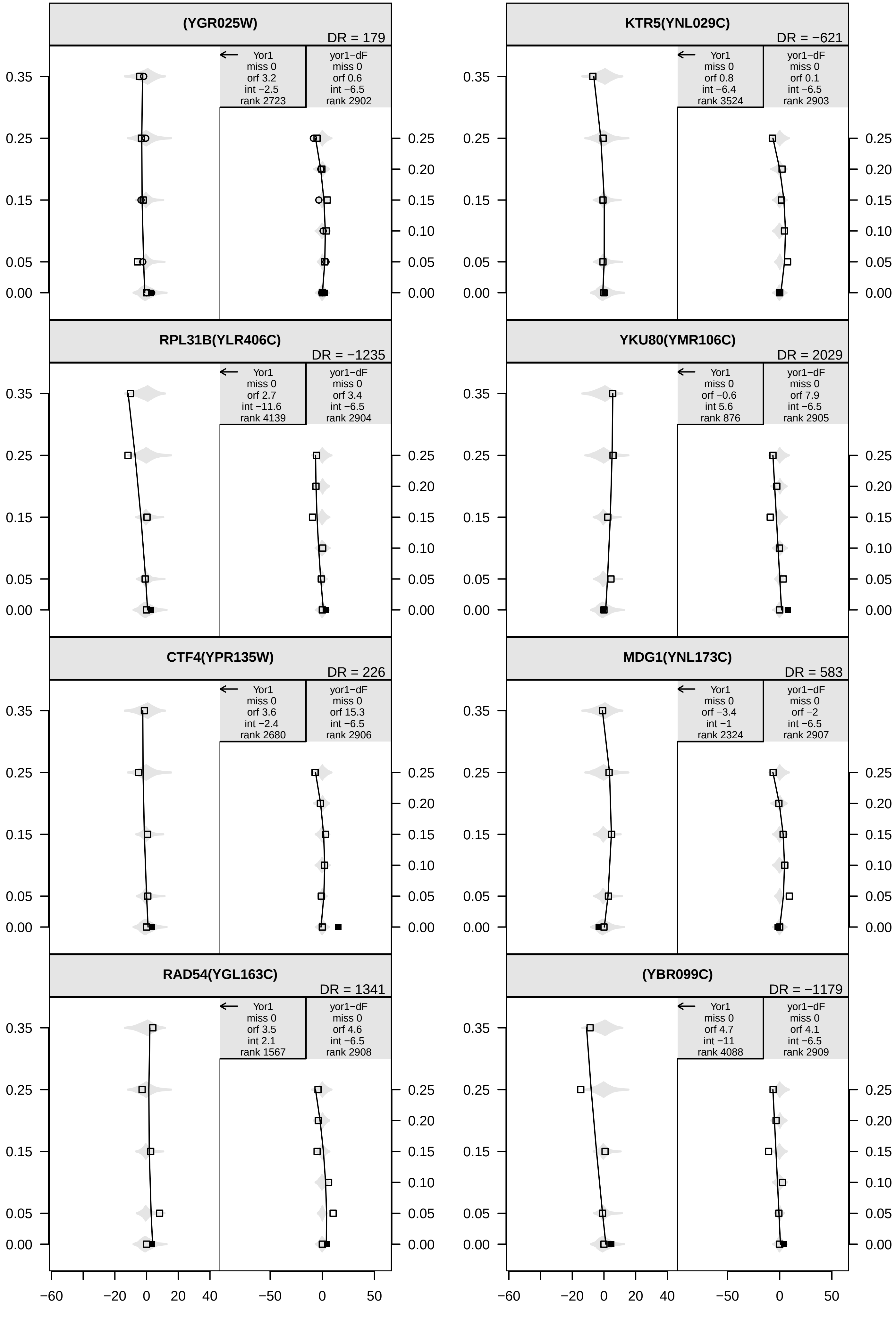

(YGR025W)
DR = 179
Yor1
miss 0
orf 3.2
int −2.5
rank 2723
yor1−dF
miss 0
orf 0.6
int −6.5
rank 2902
KTR5(YNL029C)
DR = −621
Yor1
miss 0
orf 0.8
int −6.4
rank 3524
yor1−dF
miss 0
orf 0.1
int −6.5
rank 2903
RPL31B(YLR406C)
DR = −1235
Yor1
miss 0
orf 2.7
int −11.6
rank 4139
yor1−dF
miss 0
orf 3.4
int −6.5
rank 2904
YKU80(YMR106C)
DR = 2029
Yor1
miss 0
orf −0.6
int 5.6
rank 876
yor1−dF
miss 0
orf 7.9
int −6.5
rank 2905
CTF4(YPR135W)
DR = 226
Yor1
miss 0
orf 3.6
int −2.4
rank 2680
yor1−dF
miss 0
orf 15.3
int −6.5
rank 2906
MDG1(YNL173C)
DR = 583
Yor1
miss 0
orf −3.4
int −1
rank 2324
yor1−dF
miss 0
orf −2
int −6.5
rank 2907
RAD54(YGL163C)
DR = 1341
Yor1
miss 0
orf 3.5
int 2.1
rank 1567
yor1−dF
miss 0
orf 4.6
int −6.5
rank 2908
(YBR099C)
DR = −1179
Yor1
miss 0
orf 4.7
int −11
rank 4088
yor1−dF
miss 0
orf 4.1
int −6.5
rank 2909
0.35
0.25
0.20
0.15
0.10
0.05
0.00
−60
−20
0
20
40
−50
0
50

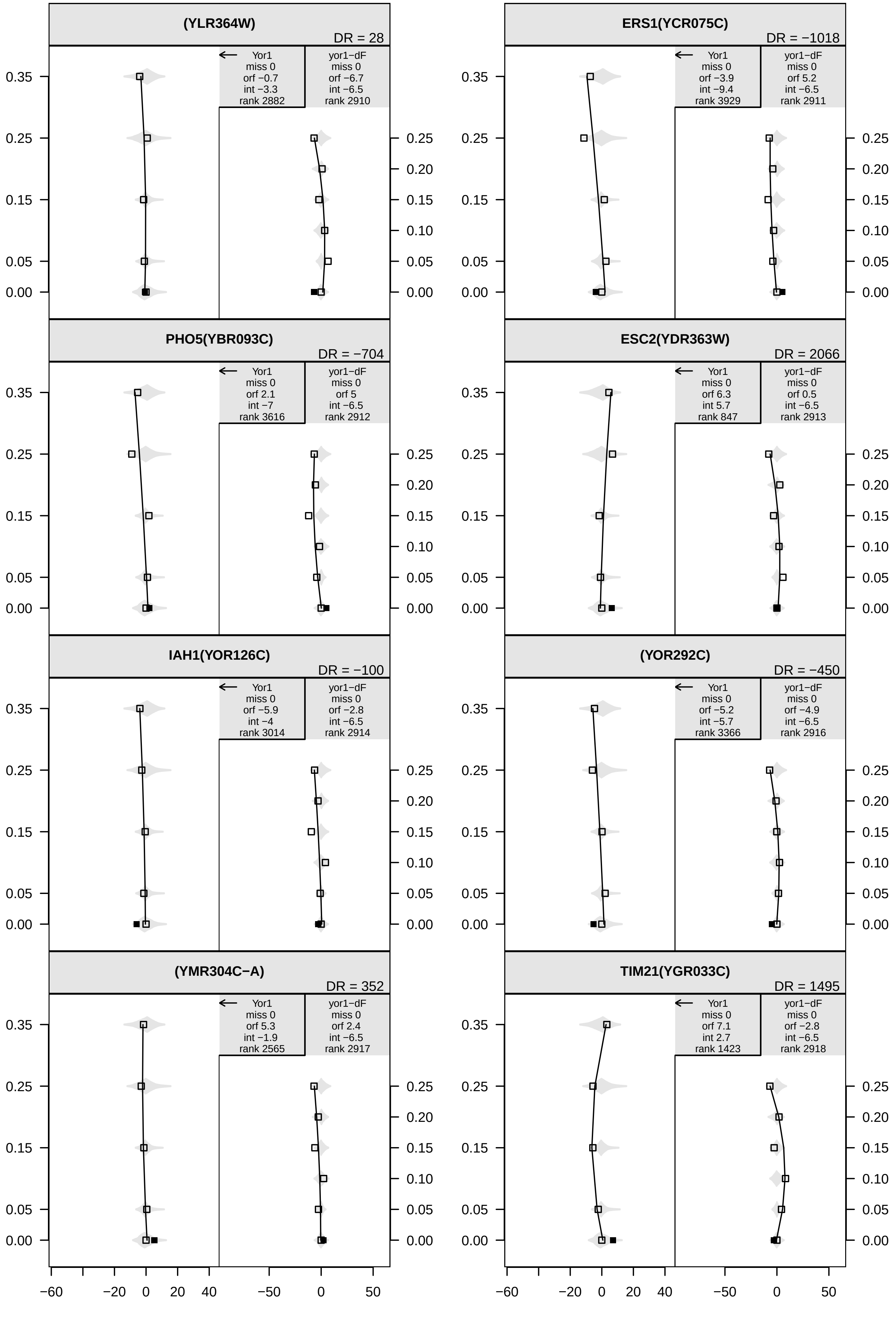

(YLR364W)
DR = 28
Yor1 miss 0 orf −0.7 int −3.3 rank 2882
yor1−dF miss 0 orf −6.7 int −6.5 rank 2910
ERS1(YCR075C)
DR = −1018
Yor1 miss 0 orf −3.9 int −9.4 rank 3929
yor1−dF miss 0 orf 5.2 int −6.5 rank 2911
PHO5(YBR093C)
DR = −704
Yor1 miss 0 orf 2.1 int −7 rank 3616
yor1−dF miss 0 orf 5 int −6.5 rank 2912
ESC2(YDR363W)
DR = 2066
Yor1 miss 0 orf 6.3 int 5.7 rank 847
yor1−dF miss 0 orf 0.5 int −6.5 rank 2913
IAH1(YOR126C)
DR = −100
Yor1 miss 0 orf −5.9 int −4 rank 3014
yor1−dF miss 0 orf −2.8 int −6.5 rank 2914
(YOR292C)
DR = −450
Yor1 miss 0 orf −5.2 int −5.7 rank 3366
yor1−dF miss 0 orf −4.9 int −6.5 rank 2916
(YMR304C−A)
DR = 352
Yor1 miss 0 orf 5.3 int −1.9 rank 2565
yor1−dF miss 0 orf 2.4 int −6.5 rank 2917
TIM21(YGR033C)
DR = 1495
Yor1 miss 0 orf 7.1 int 2.7 rank 1423
yor1−dF miss 0 orf −2.8 int −6.5 rank 2918

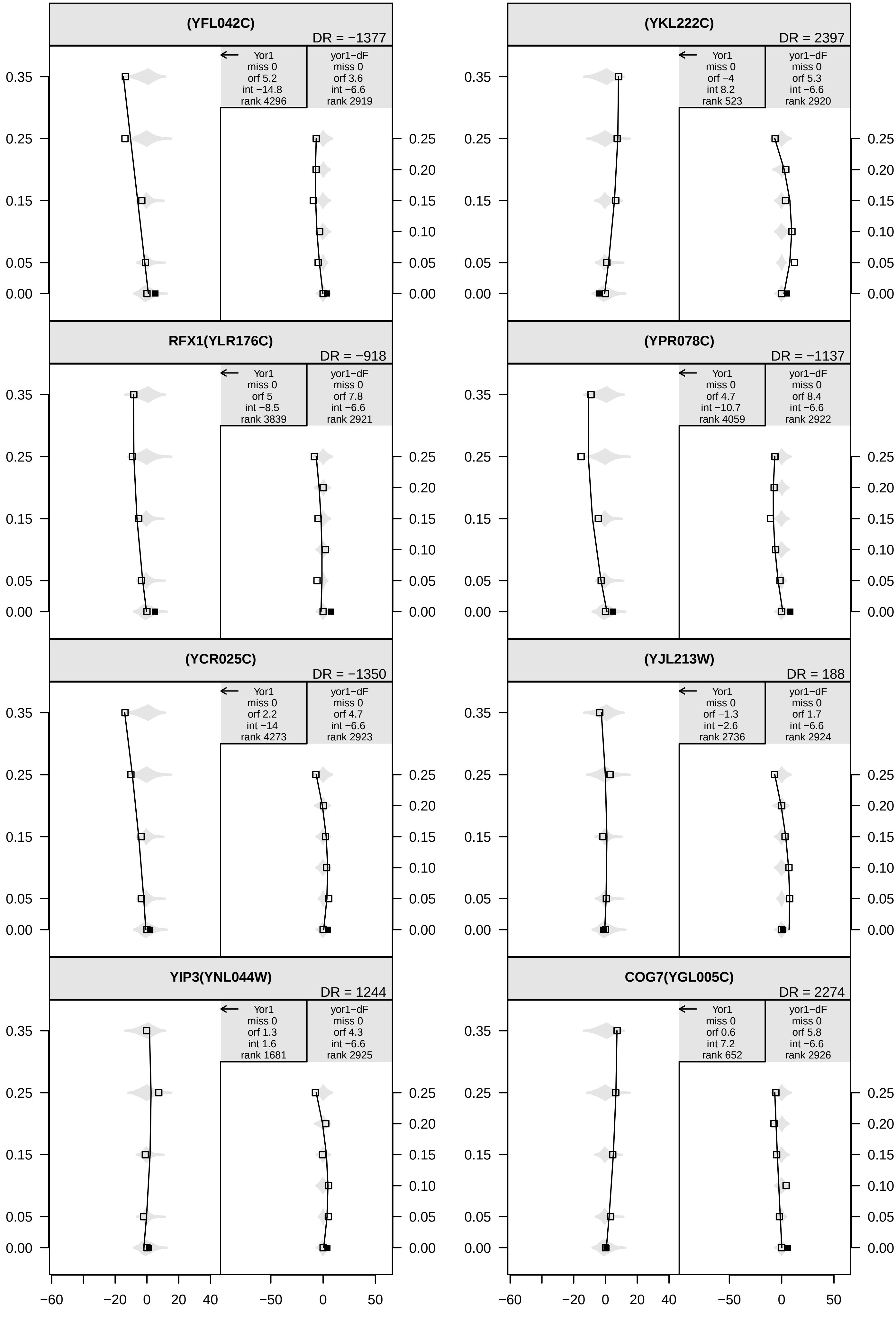
(YFL042C)
DR = −1377
Yor1 miss 0 orf 5.2 int −14.8 rank 4296
yor1−dF miss 0 orf 3.6 int −6.6 rank 2919
(YKL222C)
DR = 2397
Yor1 miss 0 orf −4 int 8.2 rank 523
yor1−dF miss 0 orf 5.3 int −6.6 rank 2920
RFX1(YLR176C)
DR = −918
Yor1 miss 0 orf 5 int −8.5 rank 3839
yor1−dF miss 0 orf 7.8 int −6.6 rank 2921
(YPR078C)
DR = −1137
Yor1 miss 0 orf 4.7 int −10.7 rank 4059
yor1−dF miss 0 orf 8.4 int −6.6 rank 2922
(YCR025C)
DR = −1350
Yor1 miss 0 orf 2.2 int −14 rank 4273
yor1−dF miss 0 orf 4.7 int −6.6 rank 2923
(YJL213W)
DR = 188
Yor1 miss 0 orf −1.3 int −2.6 rank 2736
yor1−dF miss 0 orf 1.7 int −6.6 rank 2924
YIP3(YNL044W)
DR = 1244
Yor1 miss 0 orf 1.3 int 1.6 rank 1681
yor1−dF miss 0 orf 4.3 int −6.6 rank 2925
COG7(YGL005C)
DR = 2274
Yor1 miss 0 orf 0.6 int 7.2 rank 652
yor1−dF miss 0 orf 5.8 int −6.6 rank 2926
0.35 0.25 0.20 0.15 0.10 0.05 0.00
−60 −20 0 20 40 −50 0 50

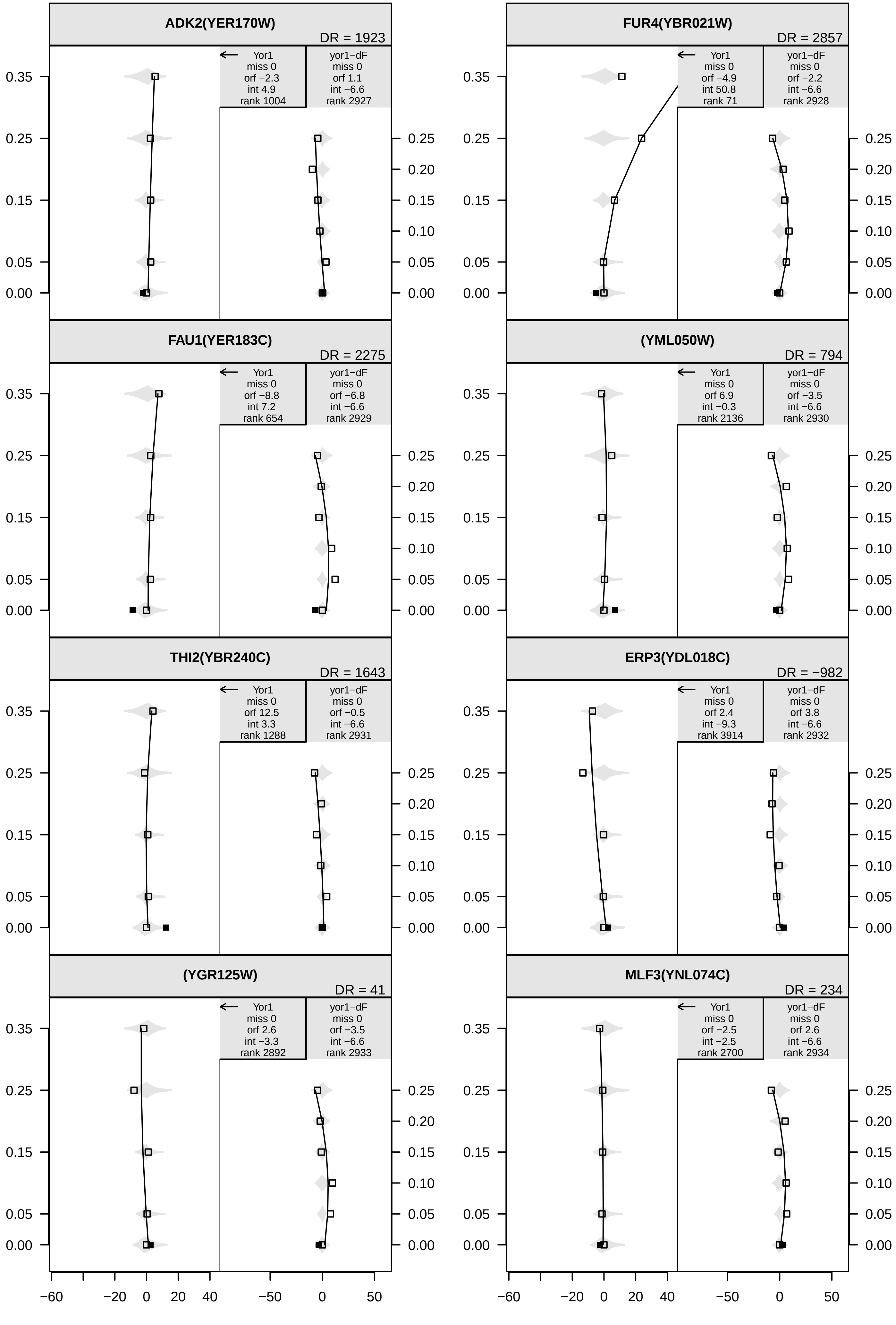

ADK2(YER170W)
DR = 1923
Yor1 miss 0 orf −2.3 int 4.9 rank 1004
yor1−dF miss 0 orf 1.1 int −6.6 rank 2927
FUR4(YBR021W)
DR = 2857
Yor1 miss 0 orf −4.9 int 50.8 rank 71
yor1−dF miss 0 orf −2.2 int −6.6 rank 2928
FAU1(YER183C)
DR = 2275
Yor1 miss 0 orf −8.8 int 7.2 rank 654
yor1−dF miss 0 orf −6.8 int −6.6 rank 2929
(YML050W)
DR = 794
Yor1 miss 0 orf 6.9 int −0.3 rank 2136
yor1−dF miss 0 orf −3.5 int −6.6 rank 2930
THI2(YBR240C)
DR = 1643
Yor1 miss 0 orf 12.5 int 3.3 rank 1288
yor1−dF miss 0 orf −0.5 int −6.6 rank 2931
ERP3(YDL018C)
DR = −982
Yor1 miss 0 orf 2.4 int −9.3 rank 3914
yor1−dF miss 0 orf 3.8 int −6.6 rank 2932
(YGR125W)
DR = 41
Yor1 miss 0 orf 2.6 int −3.3 rank 2892
yor1−dF miss 0 orf −3.5 int −6.6 rank 2933
MLF3(YNL074C)
DR = 234
Yor1 miss 0 orf −2.5 int −2.5 rank 2700
yor1−dF miss 0 orf 2.6 int −6.6 rank 2934
0.35 0.25 0.20 0.15 0.10 0.05 0.00
−60 −20 0 20 40 −50 0 50

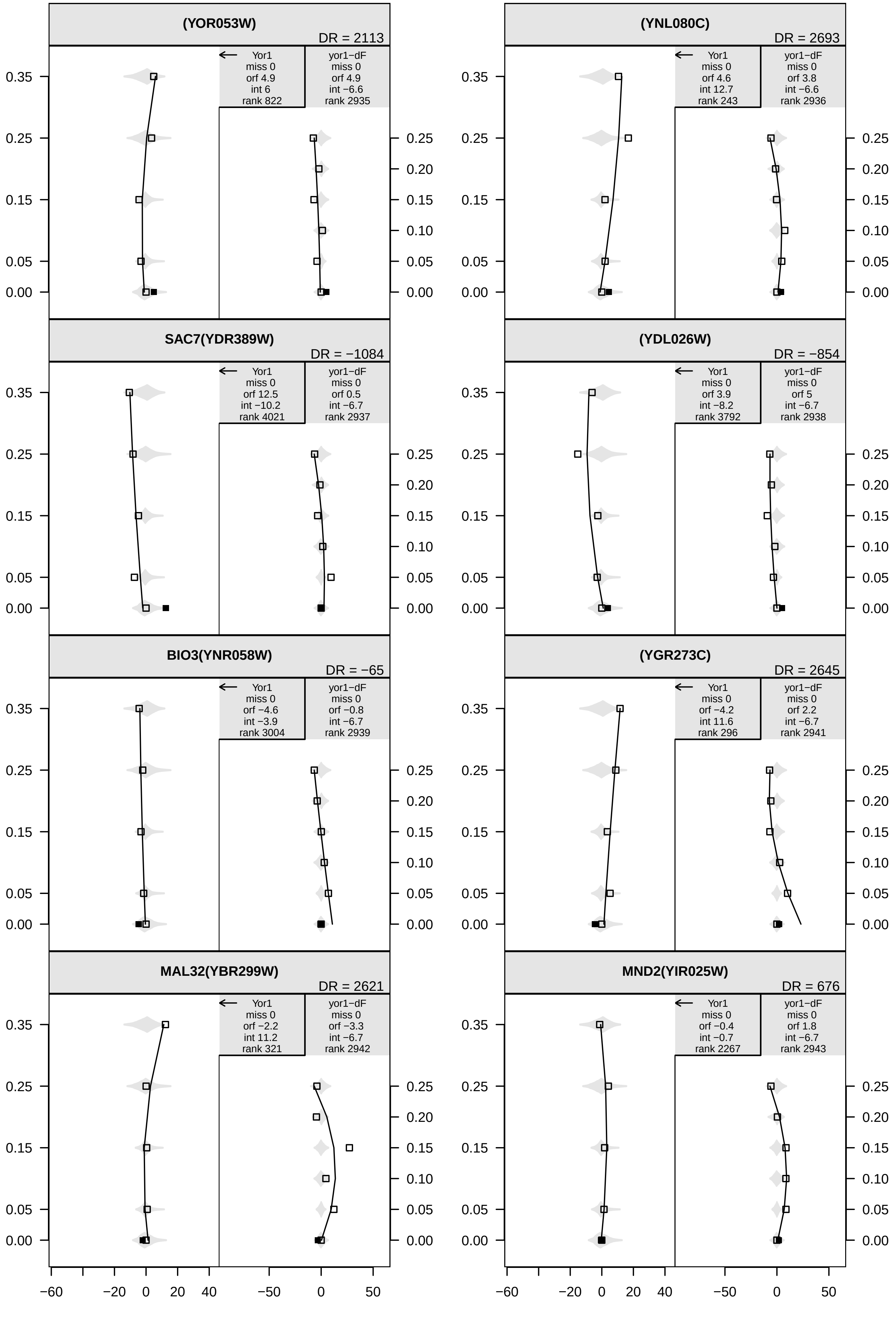
(YOR053W)
DR = 2113
Yor1 miss 0 orf 4.9 int 6 rank 822
yor1−dF miss 0 orf 4.9 int −6.6 rank 2935
(YNL080C)
DR = 2693
Yor1 miss 0 orf 4.6 int 12.7 rank 243
yor1−dF miss 0 orf 3.8 int −6.6 rank 2936
SAC7(YDR389W)
DR = −1084
Yor1 miss 0 orf 12.5 int −10.2 rank 4021
yor1−dF miss 0 orf 0.5 int −6.7 rank 2937
(YDL026W)
DR = −854
Yor1 miss 0 orf 3.9 int −8.2 rank 3792
yor1−dF miss 0 orf 5 int −6.7 rank 2938
BIO3(YNR058W)
DR = −65
Yor1 miss 0 orf −4.6 int −3.9 rank 3004
yor1−dF miss 0 orf −0.8 int −6.7 rank 2939
(YGR273C)
DR = 2645
Yor1 miss 0 orf −4.2 int 11.6 rank 296
yor1−dF miss 0 orf 2.2 int −6.7 rank 2941
MAL32(YBR299W)
DR = 2621
Yor1 miss 0 orf −2.2 int 11.2 rank 321
yor1−dF miss 0 orf −3.3 int −6.7 rank 2942
MND2(YIR025W)
DR = 676
Yor1 miss 0 orf −0.4 int −0.7 rank 2267
yor1−dF miss 0 orf 1.8 int −6.7 rank 2943

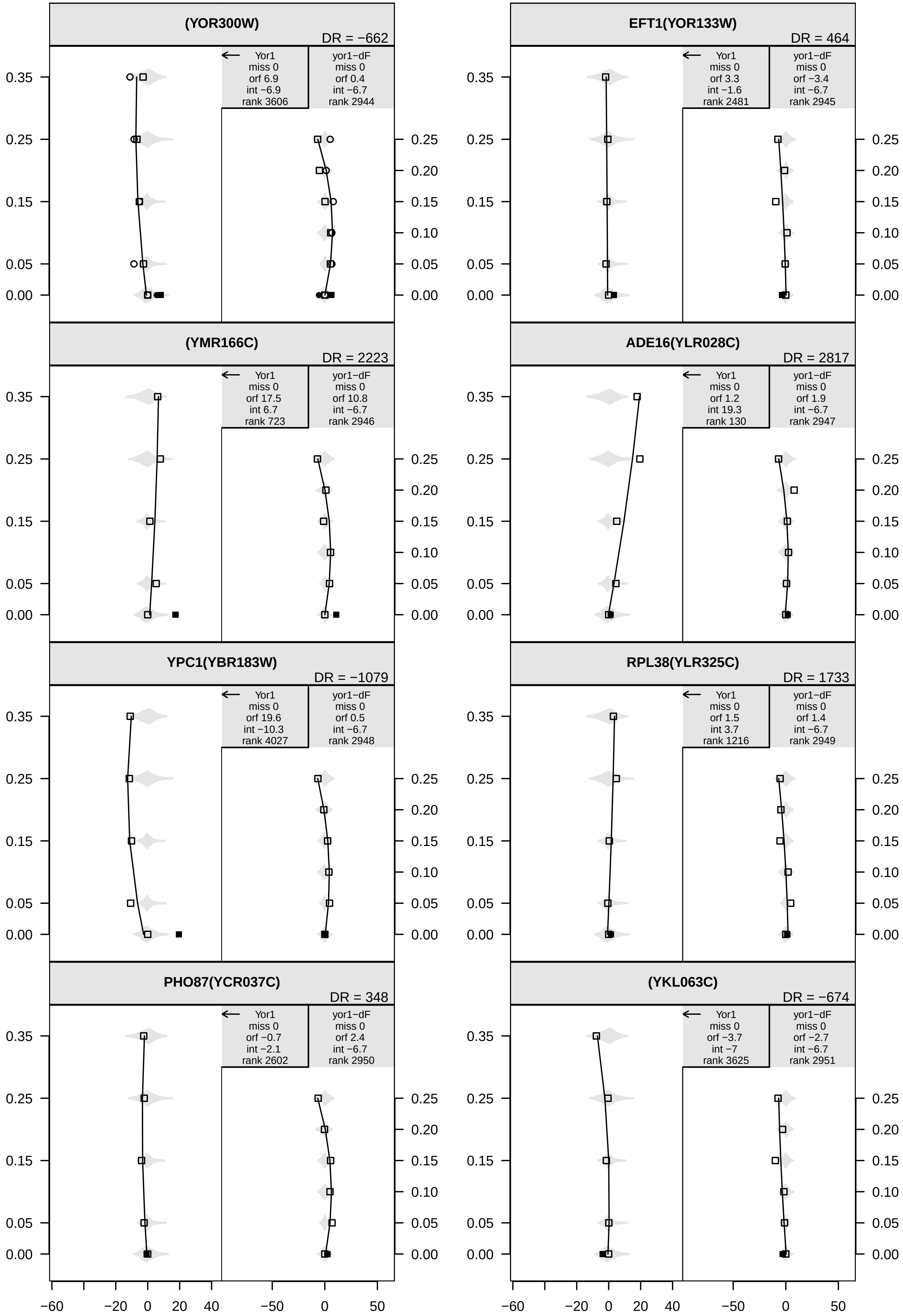

(YOR300W)
DR = −662
Yor1 miss 0 orf 6.9 int −6.9 rank 3606
yor1−dF miss 0 orf 0.4 int −6.7 rank 2944
EFT1(YOR133W)
DR = 464
Yor1 miss 0 orf 3.3 int −1.6 rank 2481
yor1−dF miss 0 orf −3.4 int −6.7 rank 2945
(YMR166C)
DR = 2223
Yor1 miss 0 orf 17.5 int 6.7 rank 723
yor1−dF miss 0 orf 10.8 int −6.7 rank 2946
ADE16(YLR028C)
DR = 2817
Yor1 miss 0 orf 1.2 int 19.3 rank 130
yor1−dF miss 0 orf 1.9 int −6.7 rank 2947
YPC1(YBR183W)
DR = −1079
Yor1 miss 0 orf 19.6 int −10.3 rank 4027
yor1−dF miss 0 orf 0.5 int −6.7 rank 2948
RPL38(YLR325C)
DR = 1733
Yor1 miss 0 orf 1.5 int 3.7 rank 1216
yor1−dF miss 0 orf 1.4 int −6.7 rank 2949
PHO87(YCR037C)
DR = 348
Yor1 miss 0 orf −0.7 int −2.1 rank 2602
yor1−dF miss 0 orf 2.4 int −6.7 rank 2950
(YKL063C)
DR = −674
Yor1 miss 0 orf −3.7 int −7 rank 3625
yor1−dF miss 0 orf −2.7 int −6.7 rank 2951
0.35 0.25 0.15 0.05 0.00
0.25 0.20 0.15 0.10 0.05 0.00
−60 −20 0 20 40 −50 0 50

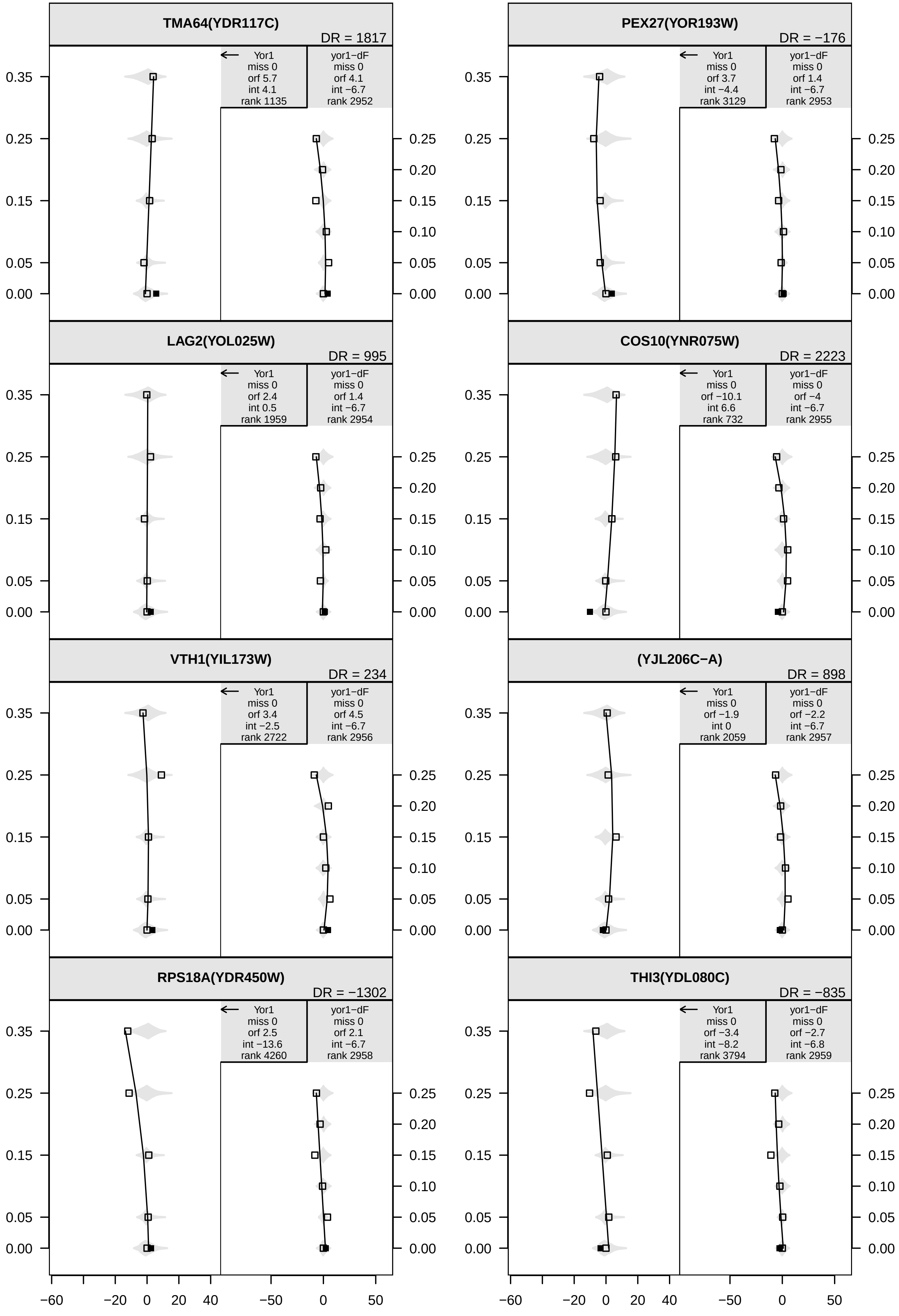

TMA64(YDR117C)
DR = 1817
Yor1 miss 0 orf 5.7 int 4.1 rank 1135
yor1−dF miss 0 orf 4.1 int −6.7 rank 2952
PEX27(YOR193W)
DR = −176
Yor1 miss 0 orf 3.7 int −4.4 rank 3129
yor1−dF miss 0 orf 1.4 int −6.7 rank 2953
LAG2(YOL025W)
DR = 995
Yor1 miss 0 orf 2.4 int 0.5 rank 1959
yor1−dF miss 0 orf 1.4 int −6.7 rank 2954
COS10(YNR075W)
DR = 2223
Yor1 miss 0 orf −10.1 int 6.6 rank 732
yor1−dF miss 0 orf −4 int −6.7 rank 2955
VTH1(YIL173W)
DR = 234
Yor1 miss 0 orf 3.4 int −2.5 rank 2722
yor1−dF miss 0 orf 4.5 int −6.7 rank 2956
(YJL206C−A)
DR = 898
Yor1 miss 0 orf −1.9 int 0 rank 2059
yor1−dF miss 0 orf −2.2 int −6.7 rank 2957
RPS18A(YDR450W)
DR = −1302
Yor1 miss 0 orf 2.5 int −13.6 rank 4260
yor1−dF miss 0 orf 2.1 int −6.7 rank 2958
THI3(YDL080C)
DR = −835
Yor1 miss 0 orf −3.4 int −8.2 rank 3794
yor1−dF miss 0 orf −2.7 int −6.8 rank 2959
0.35 0.25 0.15 0.05 0.00
0.25 0.20 0.15 0.10 0.05 0.00
−60 −20 0 20 40
−50 0 50

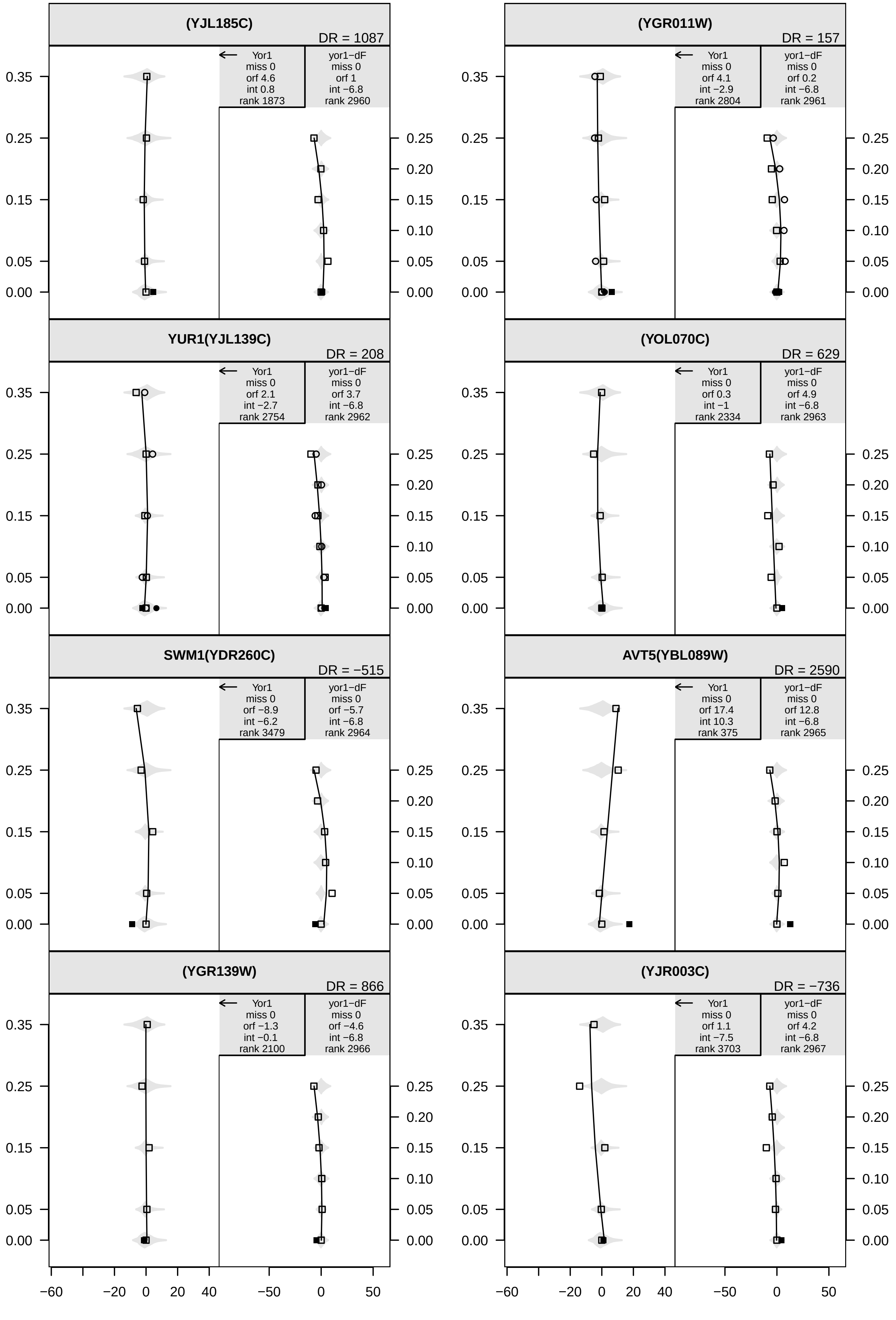

(YJL185C)
DR = 1087
Yor1
miss 0
orf 4.6
int 0.8
rank 1873
yor1−dF
miss 0
orf 1
int −6.8
rank 2960
(YGR011W)
DR = 157
Yor1
miss 0
orf 4.1
int −2.9
rank 2804
yor1−dF
miss 0
orf 0.2
int −6.8
rank 2961
YUR1(YJL139C)
DR = 208
Yor1
miss 0
orf 2.1
int −2.7
rank 2754
yor1−dF
miss 0
orf 3.7
int −6.8
rank 2962
(YOL070C)
DR = 629
Yor1
miss 0
orf 0.3
int −1
rank 2334
yor1−dF
miss 0
orf 4.9
int −6.8
rank 2963
SWM1(YDR260C)
DR = −515
Yor1
miss 0
orf −8.9
int −6.2
rank 3479
yor1−dF
miss 0
orf −5.7
int −6.8
rank 2964
AVT5(YBL089W)
DR = 2590
Yor1
miss 0
orf 17.4
int 10.3
rank 375
yor1−dF
miss 0
orf 12.8
int −6.8
rank 2965
(YGR139W)
DR = 866
Yor1
miss 0
orf −1.3
int −0.1
rank 2100
yor1−dF
miss 0
orf −4.6
int −6.8
rank 2966
(YJR003C)
DR = −736
Yor1
miss 0
orf 1.1
int −7.5
rank 3703
yor1−dF
miss 0
orf 4.2
int −6.8
rank 2967
0.35
0.25
0.20
0.15
0.10
0.05
0.00
−60
−20
0
20
40
−50
0
50

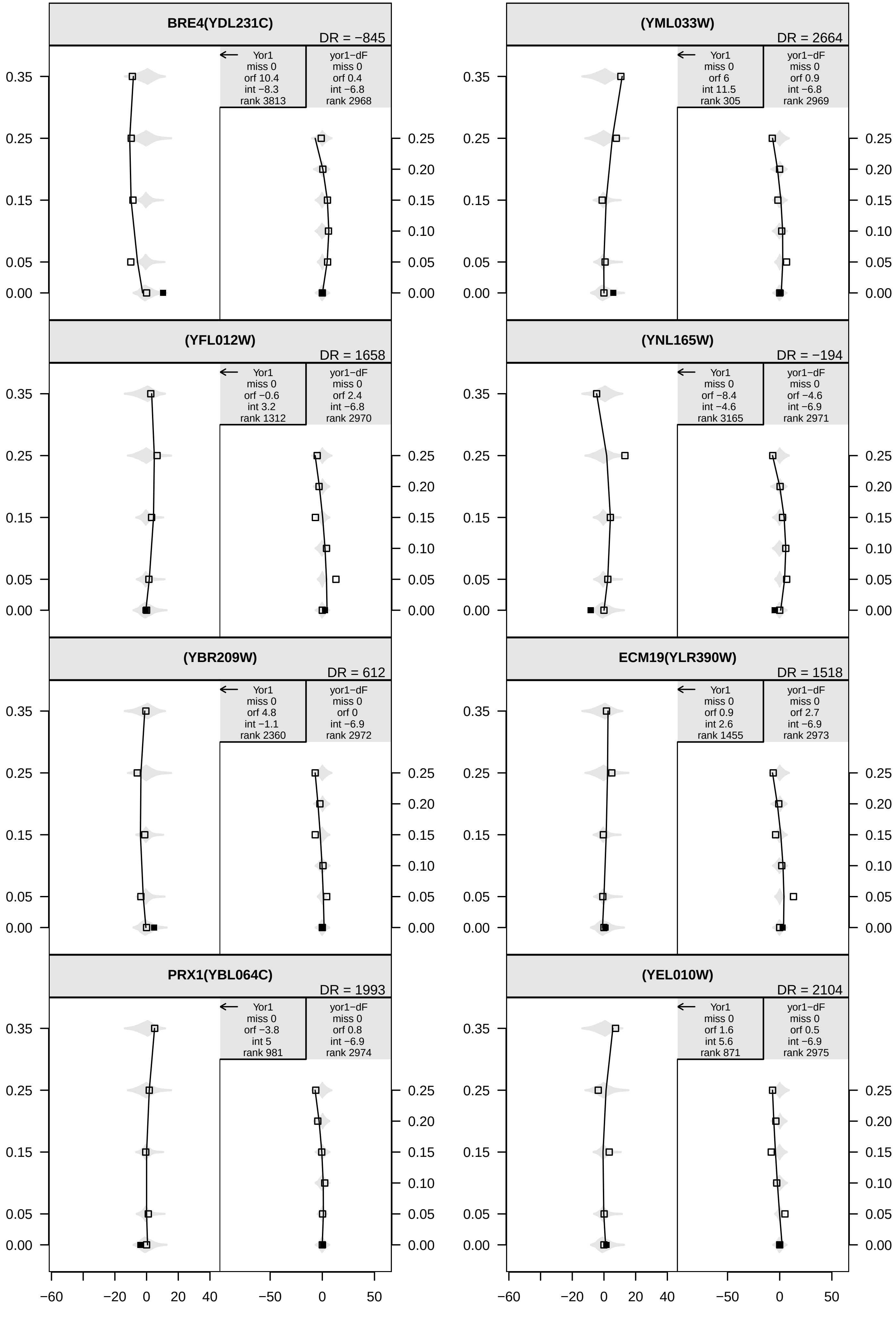
BRE4(YDL231C)
DR = −845
Yor1
miss 0
orf 10.4
int −8.3
rank 3813
yor1−dF
miss 0
orf 0.4
int −6.8
rank 2968
(YML033W)
DR = 2664
Yor1
miss 0
orf 6
int 11.5
rank 305
yor1−dF
miss 0
orf 0.9
int −6.8
rank 2969
(YFL012W)
DR = 1658
Yor1
miss 0
orf −0.6
int 3.2
rank 1312
yor1−dF
miss 0
orf 2.4
int −6.8
rank 2970
(YNL165W)
DR = −194
Yor1
miss 0
orf −8.4
int −4.6
rank 3165
yor1−dF
miss 0
orf −4.6
int −6.9
rank 2971
(YBR209W)
DR = 612
Yor1
miss 0
orf 4.8
int −1.1
rank 2360
yor1−dF
miss 0
orf 0
int −6.9
rank 2972
ECM19(YLR390W)
DR = 1518
Yor1
miss 0
orf 0.9
int 2.6
rank 1455
yor1−dF
miss 0
orf 2.7
int −6.9
rank 2973
PRX1(YBL064C)
DR = 1993
Yor1
miss 0
orf −3.8
int 5
rank 981
yor1−dF
miss 0
orf 0.8
int −6.9
rank 2974
(YEL010W)
DR = 2104
Yor1
miss 0
orf 1.6
int 5.6
rank 871
yor1−dF
miss 0
orf 0.5
int −6.9
rank 2975
0.35
0.25
0.20
0.15
0.10
0.05
0.00
−60
−20
0
20
40
−50
0
50

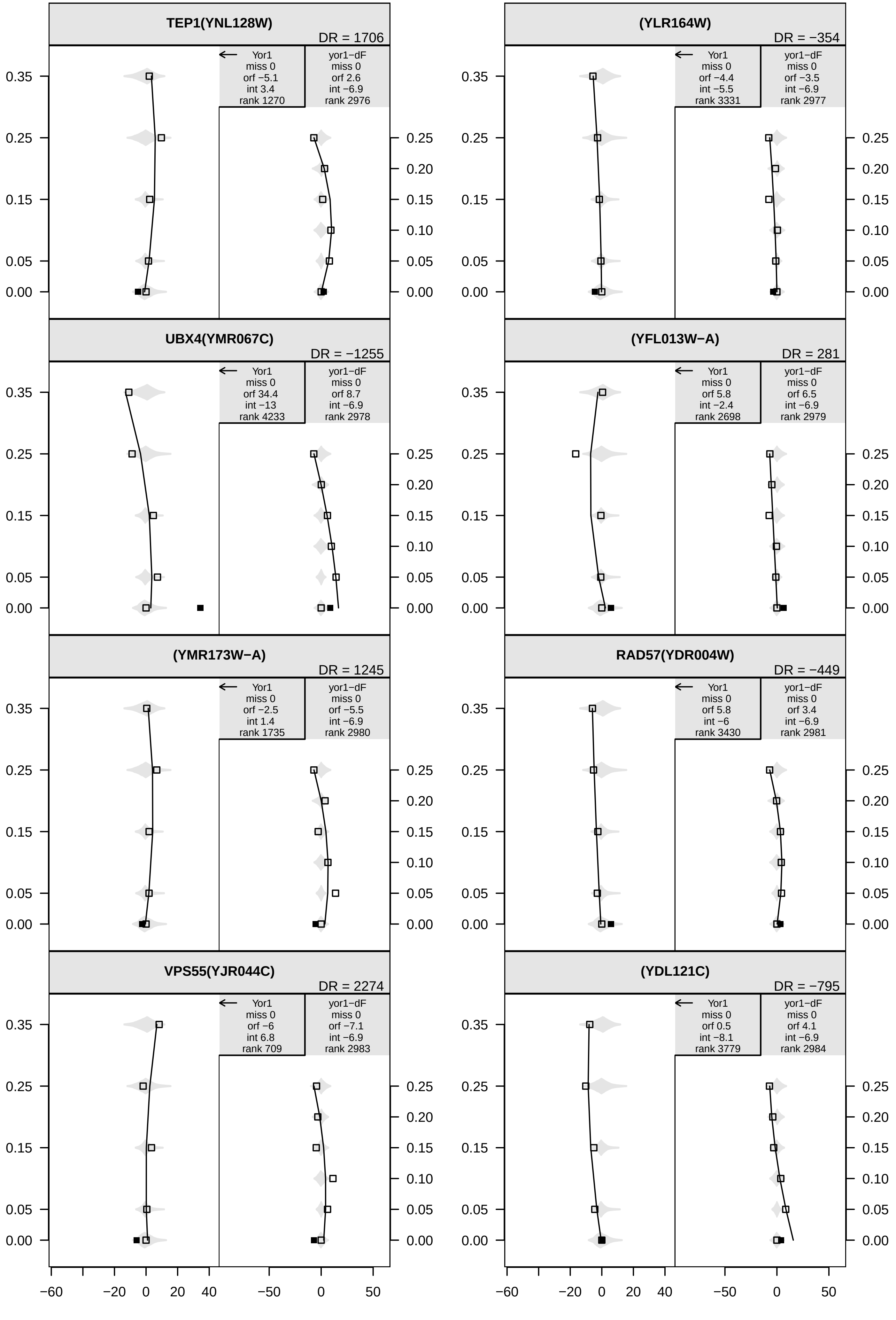

TEP1(YNL128W)
DR = 1706
Yor1 miss 0 orf −5.1 int 3.4 rank 1270
yor1−dF miss 0 orf 2.6 int −6.9 rank 2976
(YLR164W)
DR = −354
Yor1 miss 0 orf −4.4 int −5.5 rank 3331
yor1−dF miss 0 orf −3.5 int −6.9 rank 2977
UBX4(YMR067C)
DR = −1255
Yor1 miss 0 orf 34.4 int −13 rank 4233
yor1−dF miss 0 orf 8.7 int −6.9 rank 2978
(YFL013W−A)
DR = 281
Yor1 miss 0 orf 5.8 int −2.4 rank 2698
yor1−dF miss 0 orf 6.5 int −6.9 rank 2979
(YMR173W−A)
DR = 1245
Yor1 miss 0 orf −2.5 int 1.4 rank 1735
yor1−dF miss 0 orf −5.5 int −6.9 rank 2980
RAD57(YDR004W)
DR = −449
Yor1 miss 0 orf 5.8 int −6 rank 3430
yor1−dF miss 0 orf 3.4 int −6.9 rank 2981
VPS55(YJR044C)
DR = 2274
Yor1 miss 0 orf −6 int 6.8 rank 709
yor1−dF miss 0 orf −7.1 int −6.9 rank 2983
(YDL121C)
DR = −795
Yor1 miss 0 orf 0.5 int −8.1 rank 3779
yor1−dF miss 0 orf 4.1 int −6.9 rank 2984
0.00 0.05 0.10 0.15 0.20 0.25 0.35
−60 −20 0 20 40 −50 0 50

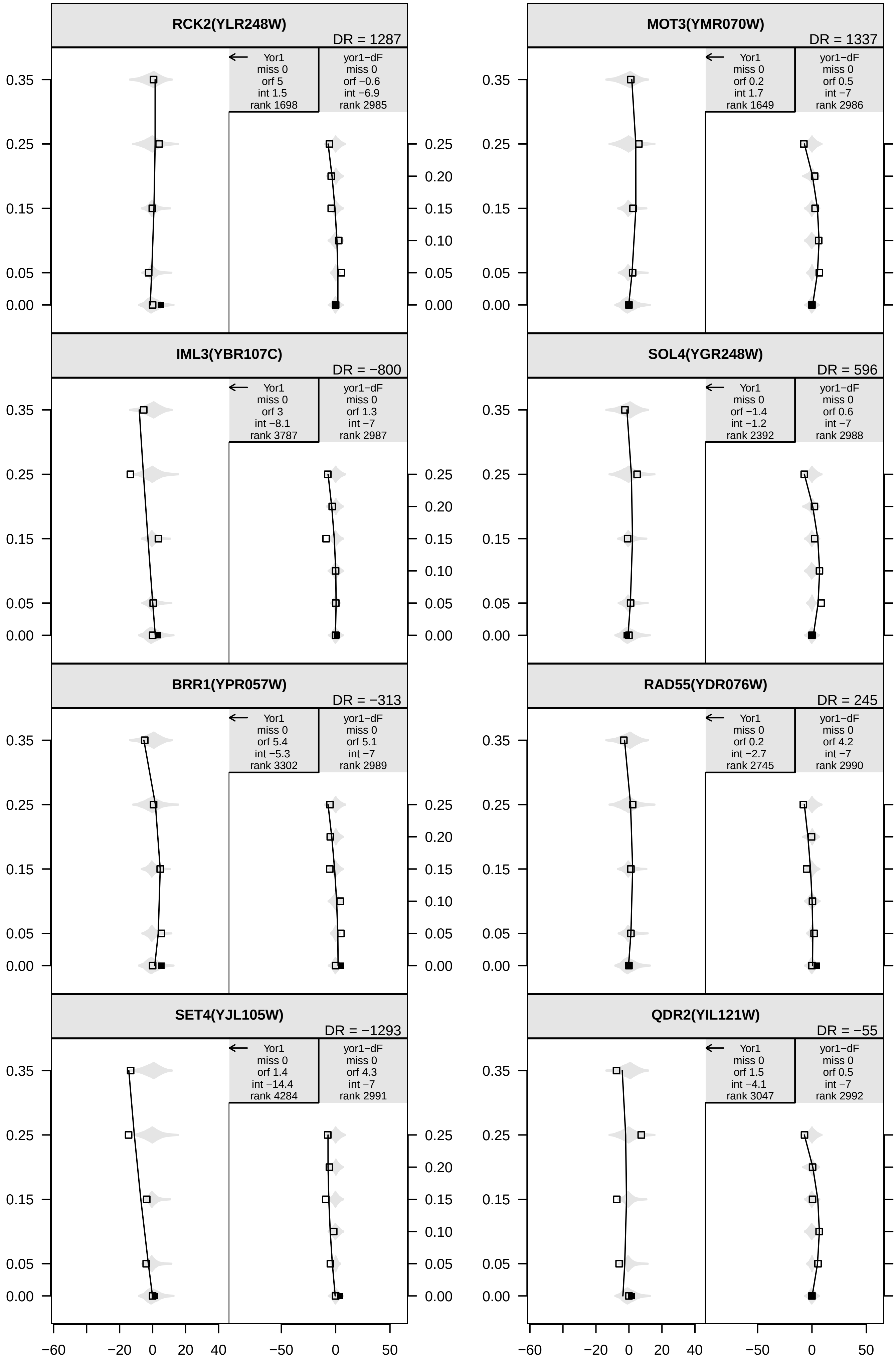
RCK2(YLR248W)
DR = 1287
Yor1 miss 0 orf 5 int 1.5 rank 1698
yor1−dF miss 0 orf −0.6 int −6.9 rank 2985
MOT3(YMR070W)
DR = 1337
Yor1 miss 0 orf 0.2 int 1.7 rank 1649
yor1−dF miss 0 orf 0.5 int −7 rank 2986
IML3(YBR107C)
DR = −800
Yor1 miss 0 orf 3 int −8.1 rank 3787
yor1−dF miss 0 orf 1.3 int −7 rank 2987
SOL4(YGR248W)
DR = 596
Yor1 miss 0 orf −1.4 int −1.2 rank 2392
yor1−dF miss 0 orf 0.6 int −7 rank 2988
BRR1(YPR057W)
DR = −313
Yor1 miss 0 orf 5.4 int −5.3 rank 3302
yor1−dF miss 0 orf 5.1 int −7 rank 2989
RAD55(YDR076W)
DR = 245
Yor1 miss 0 orf 0.2 int −2.7 rank 2745
yor1−dF miss 0 orf 4.2 int −7 rank 2990
SET4(YJL105W)
DR = −1293
Yor1 miss 0 orf 1.4 int −14.4 rank 4284
yor1−dF miss 0 orf 4.3 int −7 rank 2991
QDR2(YIL121W)
DR = −55
Yor1 miss 0 orf 1.5 int −4.1 rank 3047
yor1−dF miss 0 orf 0.5 int −7 rank 2992
0.35 0.25 0.20 0.15 0.10 0.05 0.00
−60 −20 0 20 40 −50 0 50

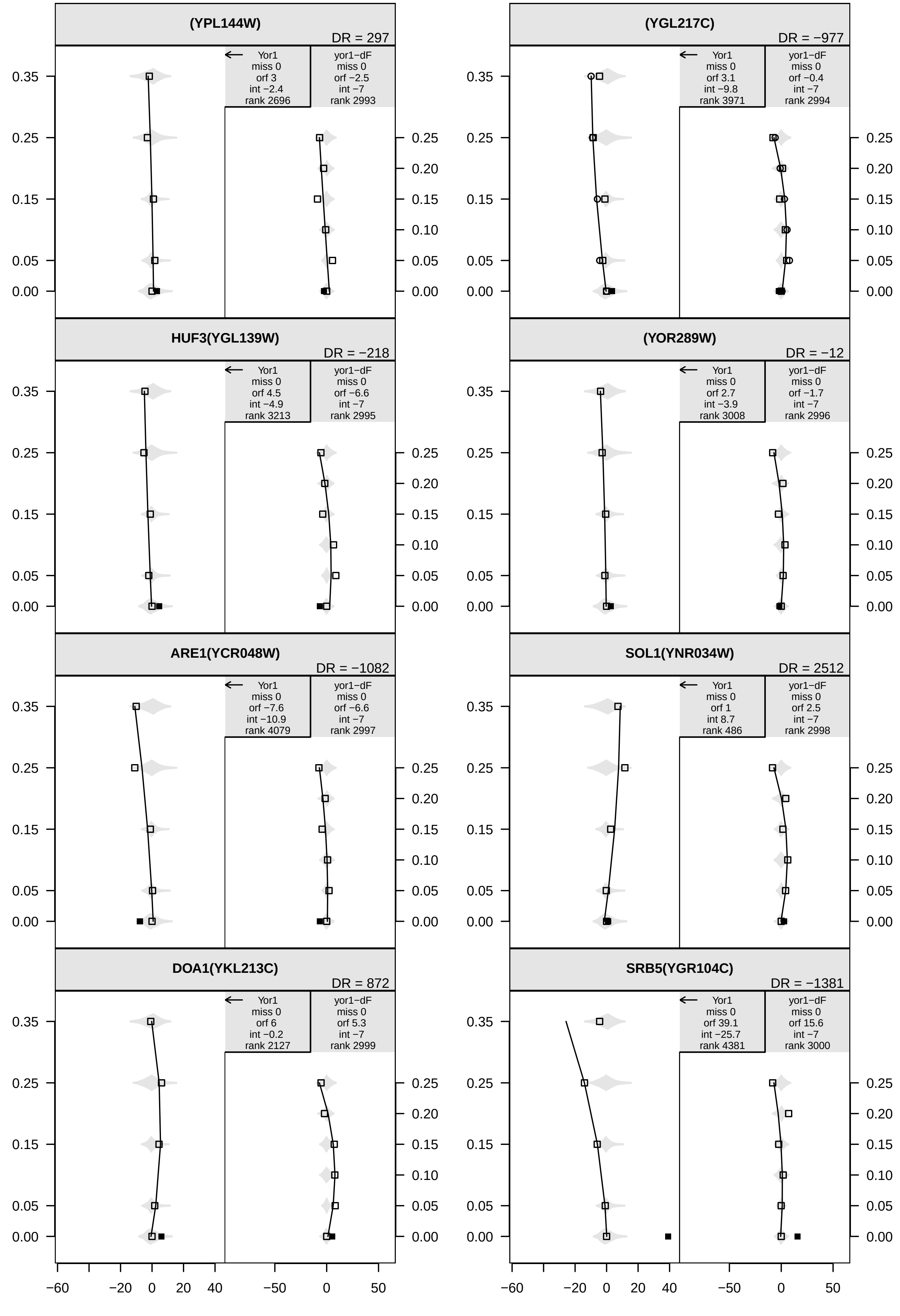

(YPL144W)
DR = 297
Yor1 miss 0 orf 3 int −2.4 rank 2696
yor1−dF miss 0 orf −2.5 int −7 rank 2993
(YGL217C)
DR = −977
Yor1 miss 0 orf 3.1 int −9.8 rank 3971
yor1−dF miss 0 orf −0.4 int −7 rank 2994
HUF3(YGL139W)
DR = −218
Yor1 miss 0 orf 4.5 int −4.9 rank 3213
yor1−dF miss 0 orf −6.6 int −7 rank 2995
(YOR289W)
DR = −12
Yor1 miss 0 orf 2.7 int −3.9 rank 3008
yor1−dF miss 0 orf −1.7 int −7 rank 2996
ARE1(YCR048W)
DR = −1082
Yor1 miss 0 orf −7.6 int −10.9 rank 4079
yor1−dF miss 0 orf −6.6 int −7 rank 2997
SOL1(YNR034W)
DR = 2512
Yor1 miss 0 orf 1 int 8.7 rank 486
yor1−dF miss 0 orf 2.5 int −7 rank 2998
DOA1(YKL213C)
DR = 872
Yor1 miss 0 orf 6 int −0.2 rank 2127
yor1−dF miss 0 orf 5.3 int −7 rank 2999
SRB5(YGR104C)
DR = −1381
Yor1 miss 0 orf 39.1 int −25.7 rank 4381
yor1−dF miss 0 orf 15.6 int −7 rank 3000

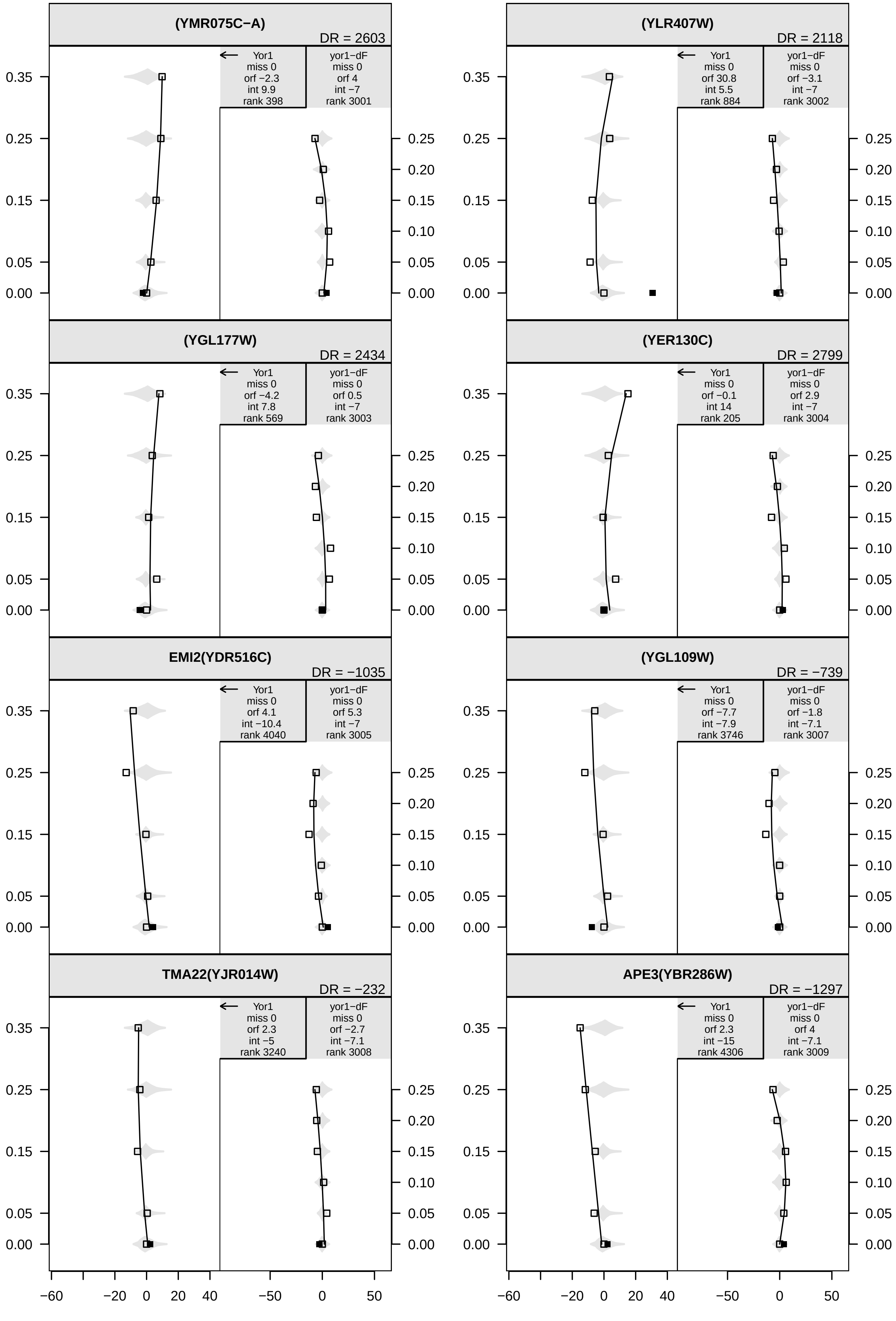
(YMR075C−A)
DR = 2603
Yor1 miss 0 orf −2.3 int 9.9 rank 398
yor1−dF miss 0 orf 4 int −7 rank 3001
(YLR407W)
DR = 2118
Yor1 miss 0 orf 30.8 int 5.5 rank 884
yor1−dF miss 0 orf −3.1 int −7 rank 3002
(YGL177W)
DR = 2434
Yor1 miss 0 orf −4.2 int 7.8 rank 569
yor1−dF miss 0 orf 0.5 int −7 rank 3003
(YER130C)
DR = 2799
Yor1 miss 0 orf −0.1 int 14 rank 205
yor1−dF miss 0 orf 2.9 int −7 rank 3004
EMI2(YDR516C)
DR = −1035
Yor1 miss 0 orf 4.1 int −10.4 rank 4040
yor1−dF miss 0 orf 5.3 int −7 rank 3005
(YGL109W)
DR = −739
Yor1 miss 0 orf −7.7 int −7.9 rank 3746
yor1−dF miss 0 orf −1.8 int −7.1 rank 3007
TMA22(YJR014W)
DR = −232
Yor1 miss 0 orf 2.3 int −5 rank 3240
yor1−dF miss 0 orf −2.7 int −7.1 rank 3008
APE3(YBR286W)
DR = −1297
Yor1 miss 0 orf 2.3 int −15 rank 4306
yor1−dF miss 0 orf 4 int −7.1 rank 3009
0.35 0.25 0.20 0.15 0.10 0.05 0.00
−60 −20 0 20 40 −50 0 50

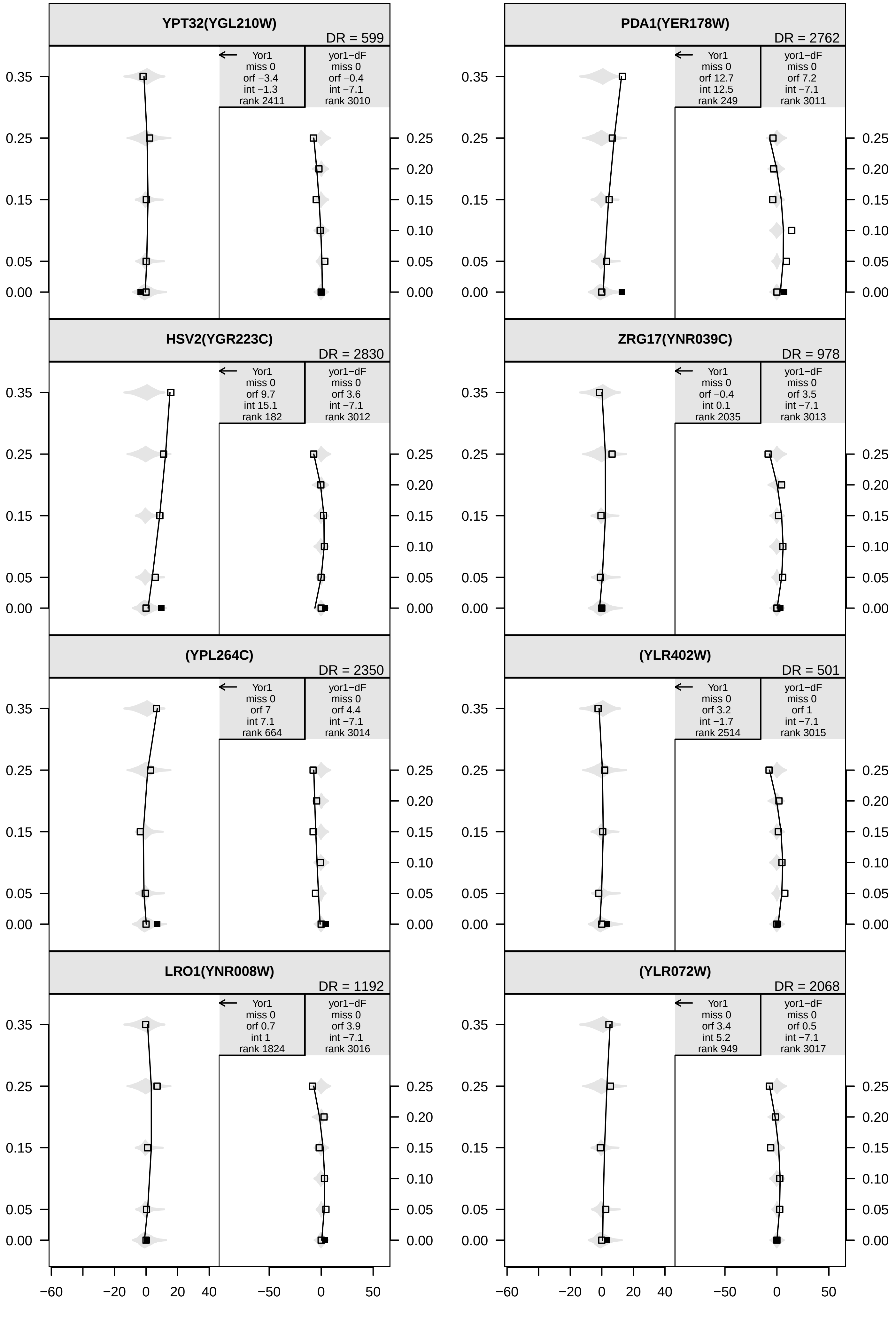
YPT32(YGL210W)
DR = 599
Yor1
miss 0
orf −3.4
int −1.3
rank 2411
yor1−dF
miss 0
orf −0.4
int −7.1
rank 3010
PDA1(YER178W)
DR = 2762
Yor1
miss 0
orf 12.7
int 12.5
rank 249
yor1−dF
miss 0
orf 7.2
int −7.1
rank 3011
HSV2(YGR223C)
DR = 2830
Yor1
miss 0
orf 9.7
int 15.1
rank 182
yor1−dF
miss 0
orf 3.6
int −7.1
rank 3012
ZRG17(YNR039C)
DR = 978
Yor1
miss 0
orf −0.4
int 0.1
rank 2035
yor1−dF
miss 0
orf 3.5
int −7.1
rank 3013
(YPL264C)
DR = 2350
Yor1
miss 0
orf 7
int 7.1
rank 664
yor1−dF
miss 0
orf 4.4
int −7.1
rank 3014
(YLR402W)
DR = 501
Yor1
miss 0
orf 3.2
int −1.7
rank 2514
yor1−dF
miss 0
orf 1
int −7.1
rank 3015
LRO1(YNR008W)
DR = 1192
Yor1
miss 0
orf 0.7
int 1
rank 1824
yor1−dF
miss 0
orf 3.9
int −7.1
rank 3016
(YLR072W)
DR = 2068
Yor1
miss 0
orf 3.4
int 5.2
rank 949
yor1−dF
miss 0
orf 0.5
int −7.1
rank 3017
0.35
0.25
0.20
0.15
0.10
0.05
0.00
−60
−20
0
20
40
−50
0
50

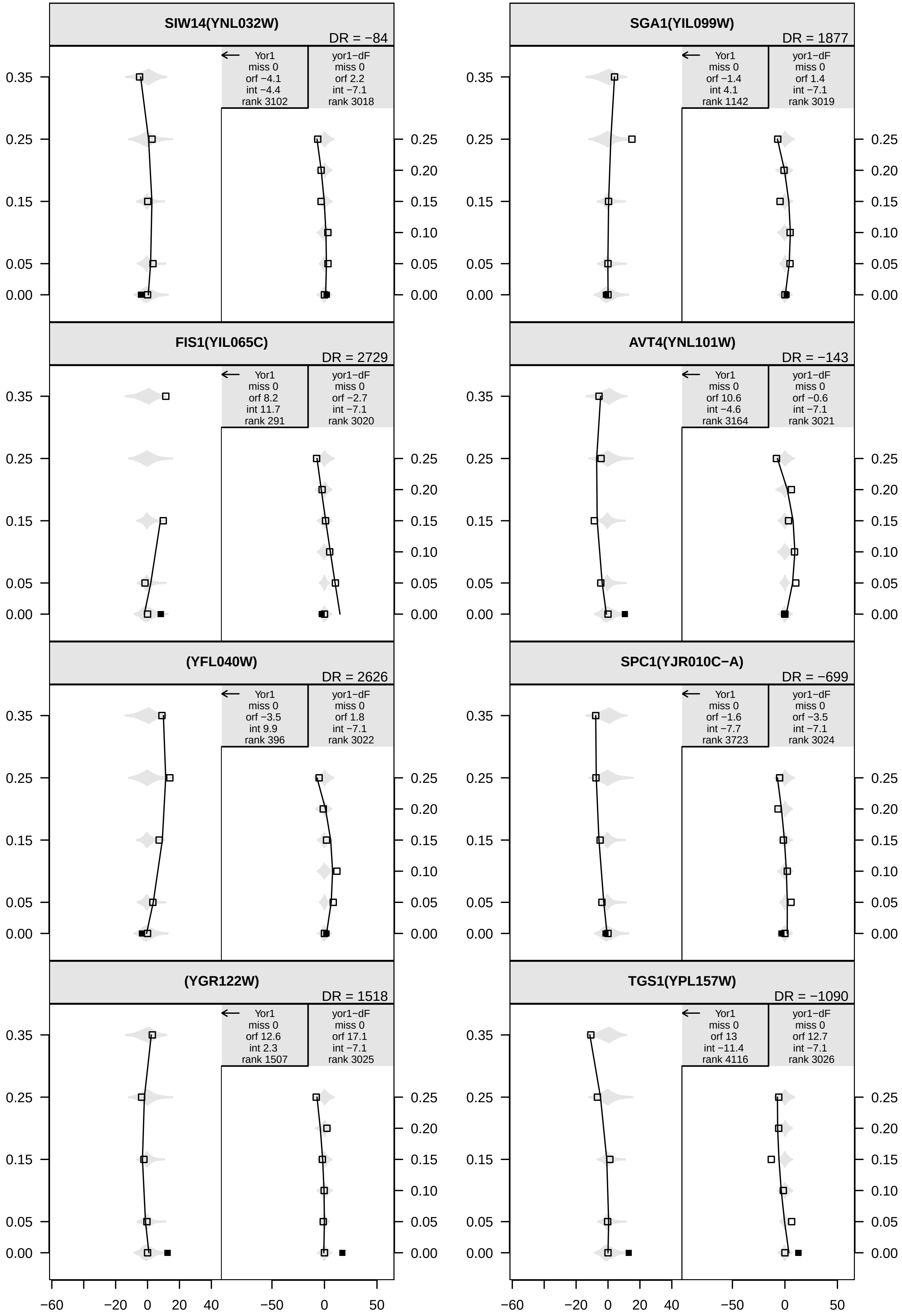
SIW14(YNL032W)
DR = −84
Yor1
miss 0
orf −4.1
int −4.4
rank 3102
yor1−dF
miss 0
orf 2.2
int −7.1
rank 3018
SGA1(YIL099W)
DR = 1877
Yor1
miss 0
orf −1.4
int 4.1
rank 1142
yor1−dF
miss 0
orf 1.4
int −7.1
rank 3019
FIS1(YIL065C)
DR = 2729
Yor1
miss 0
orf 8.2
int 11.7
rank 291
yor1−dF
miss 0
orf −2.7
int −7.1
rank 3020
AVT4(YNL101W)
DR = −143
Yor1
miss 0
orf 10.6
int −4.6
rank 3164
yor1−dF
miss 0
orf −0.6
int −7.1
rank 3021
(YFL040W)
DR = 2626
Yor1
miss 0
orf −3.5
int 9.9
rank 396
yor1−dF
miss 0
orf 1.8
int −7.1
rank 3022
SPC1(YJR010C−A)
DR = −699
Yor1
miss 0
orf −1.6
int −7.7
rank 3723
yor1−dF
miss 0
orf −3.5
int −7.1
rank 3024
(YGR122W)
DR = 1518
Yor1
miss 0
orf 12.6
int 2.3
rank 1507
yor1−dF
miss 0
orf 17.1
int −7.1
rank 3025
TGS1(YPL157W)
DR = −1090
Yor1
miss 0
orf 13
int −11.4
rank 4116
yor1−dF
miss 0
orf 12.7
int −7.1
rank 3026
0.35
0.25
0.20
0.15
0.10
0.05
0.00
−60
−20
0
20
40
−50
0
50

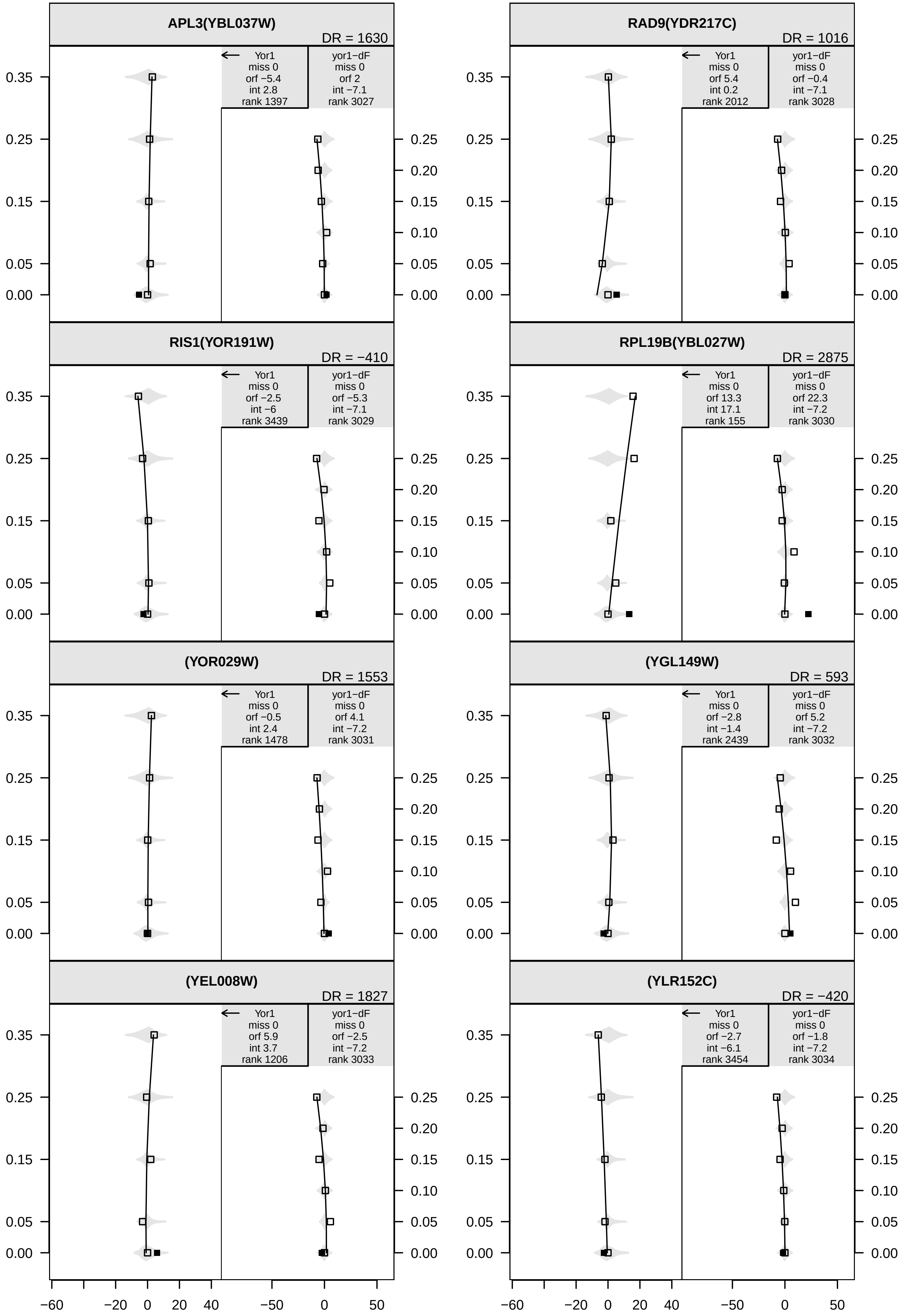
APL3(YBL037W)
DR = 1630
Yor1 miss 0 orf −5.4 int 2.8 rank 1397
yor1−dF miss 0 orf 2 int −7.1 rank 3027
RAD9(YDR217C)
DR = 1016
Yor1 miss 0 orf 5.4 int 0.2 rank 2012
yor1−dF miss 0 orf −0.4 int −7.1 rank 3028
RIS1(YOR191W)
DR = −410
Yor1 miss 0 orf −2.5 int −6 rank 3439
yor1−dF miss 0 orf −5.3 int −7.1 rank 3029
RPL19B(YBL027W)
DR = 2875
Yor1 miss 0 orf 13.3 int 17.1 rank 155
yor1−dF miss 0 orf 22.3 int −7.2 rank 3030
(YOR029W)
DR = 1553
Yor1 miss 0 orf −0.5 int 2.4 rank 1478
yor1−dF miss 0 orf 4.1 int −7.2 rank 3031
(YGL149W)
DR = 593
Yor1 miss 0 orf −2.8 int −1.4 rank 2439
yor1−dF miss 0 orf 5.2 int −7.2 rank 3032
(YEL008W)
DR = 1827
Yor1 miss 0 orf 5.9 int 3.7 rank 1206
yor1−dF miss 0 orf −2.5 int −7.2 rank 3033
(YLR152C)
DR = −420
Yor1 miss 0 orf −2.7 int −6.1 rank 3454
yor1−dF miss 0 orf −1.8 int −7.2 rank 3034
0.35
0.25
0.20
0.15
0.10
0.05
0.00
−60
−20
0
20
40
−50
0
50

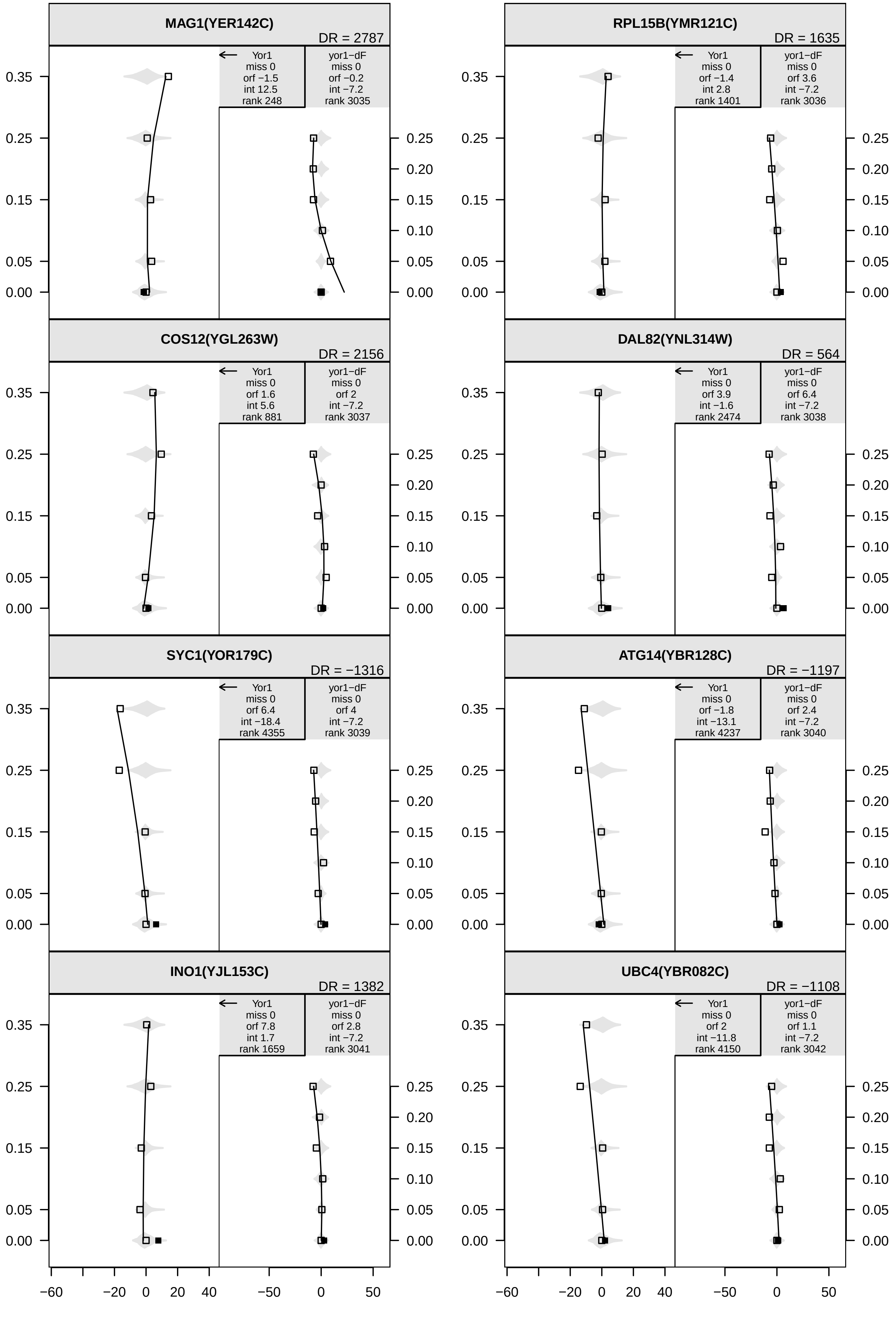
MAG1(YER142C)
DR = 2787
Yor1
miss 0
orf −1.5
int 12.5
rank 248
yor1−dF
miss 0
orf −0.2
int −7.2
rank 3035
RPL15B(YMR121C)
DR = 1635
Yor1
miss 0
orf −1.4
int 2.8
rank 1401
yor1−dF
miss 0
orf 3.6
int −7.2
rank 3036
COS12(YGL263W)
DR = 2156
Yor1
miss 0
orf 1.6
int 5.6
rank 881
yor1−dF
miss 0
orf 2
int −7.2
rank 3037
DAL82(YNL314W)
DR = 564
Yor1
miss 0
orf 3.9
int −1.6
rank 2474
yor1−dF
miss 0
orf 6.4
int −7.2
rank 3038
SYC1(YOR179C)
DR = −1316
Yor1
miss 0
orf 6.4
int −18.4
rank 4355
yor1−dF
miss 0
orf 4
int −7.2
rank 3039
ATG14(YBR128C)
DR = −1197
Yor1
miss 0
orf −1.8
int −13.1
rank 4237
yor1−dF
miss 0
orf 2.4
int −7.2
rank 3040
INO1(YJL153C)
DR = 1382
Yor1
miss 0
orf 7.8
int 1.7
rank 1659
yor1−dF
miss 0
orf 2.8
int −7.2
rank 3041
UBC4(YBR082C)
DR = −1108
Yor1
miss 0
orf 2
int −11.8
rank 4150
yor1−dF
miss 0
orf 1.1
int −7.2
rank 3042
0.35
0.25
0.20
0.15
0.10
0.05
0.00
−60
−20
0
20
40
−50
0
50

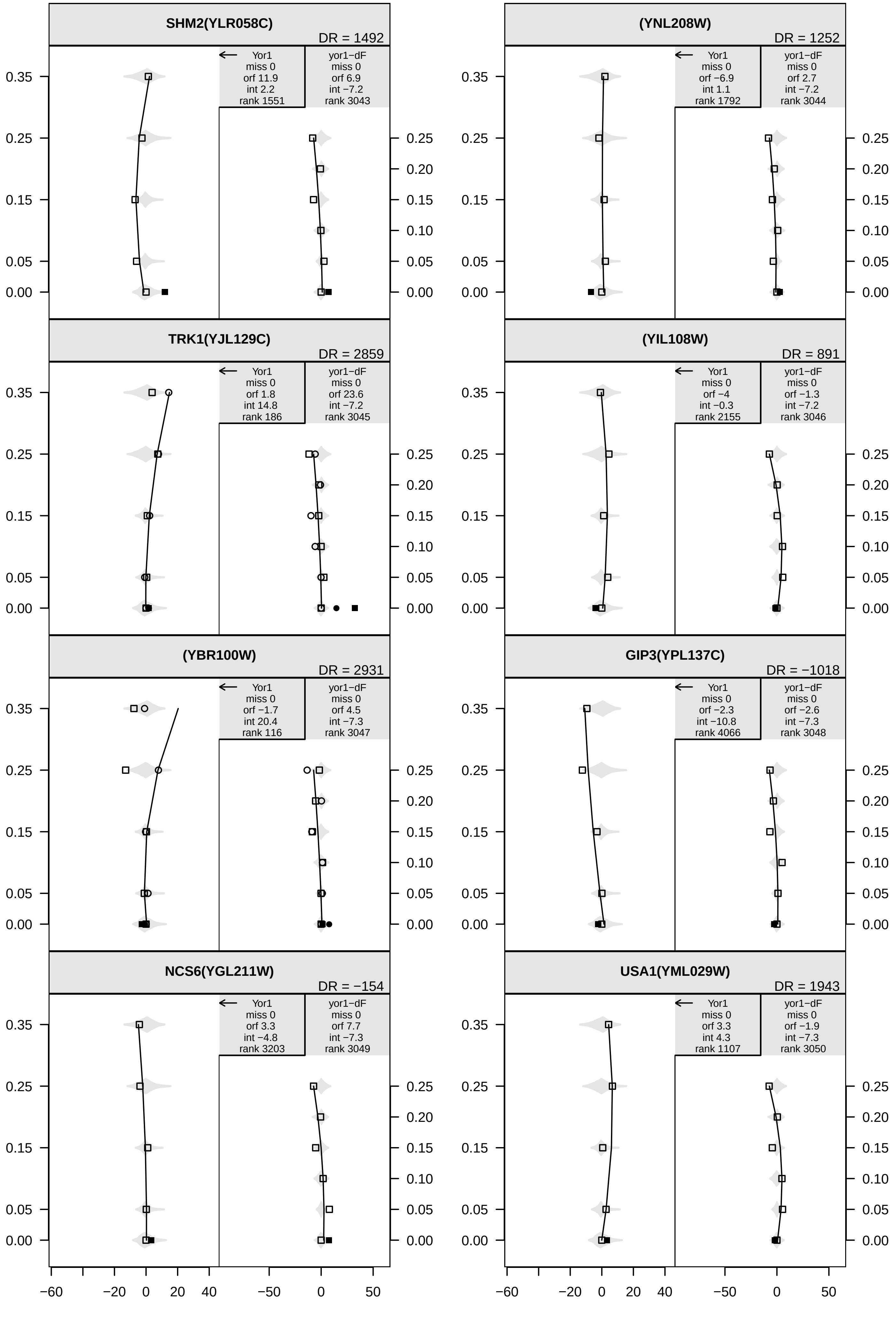

SHM2(YLR058C)
DR = 1492
Yor1 miss 0 orf 11.9 int 2.2 rank 1551
yor1−dF miss 0 orf 6.9 int −7.2 rank 3043
(YNL208W)
DR = 1252
Yor1 miss 0 orf −6.9 int 1.1 rank 1792
yor1−dF miss 0 orf 2.7 int −7.2 rank 3044
TRK1(YJL129C)
DR = 2859
Yor1 miss 0 orf 1.8 int 14.8 rank 186
yor1−dF miss 0 orf 23.6 int −7.2 rank 3045
(YIL108W)
DR = 891
Yor1 miss 0 orf −4 int −0.3 rank 2155
yor1−dF miss 0 orf −1.3 int −7.2 rank 3046
(YBR100W)
DR = 2931
Yor1 miss 0 orf −1.7 int 20.4 rank 116
yor1−dF miss 0 orf 4.5 int −7.3 rank 3047
GIP3(YPL137C)
DR = −1018
Yor1 miss 0 orf −2.3 int −10.8 rank 4066
yor1−dF miss 0 orf −2.6 int −7.3 rank 3048
NCS6(YGL211W)
DR = −154
Yor1 miss 0 orf 3.3 int −4.8 rank 3203
yor1−dF miss 0 orf 7.7 int −7.3 rank 3049
USA1(YML029W)
DR = 1943
Yor1 miss 0 orf 3.3 int 4.3 rank 1107
yor1−dF miss 0 orf −1.9 int −7.3 rank 3050
0.35 0.25 0.20 0.15 0.10 0.05 0.00
−60 −20 0 20 40 −50 0 50

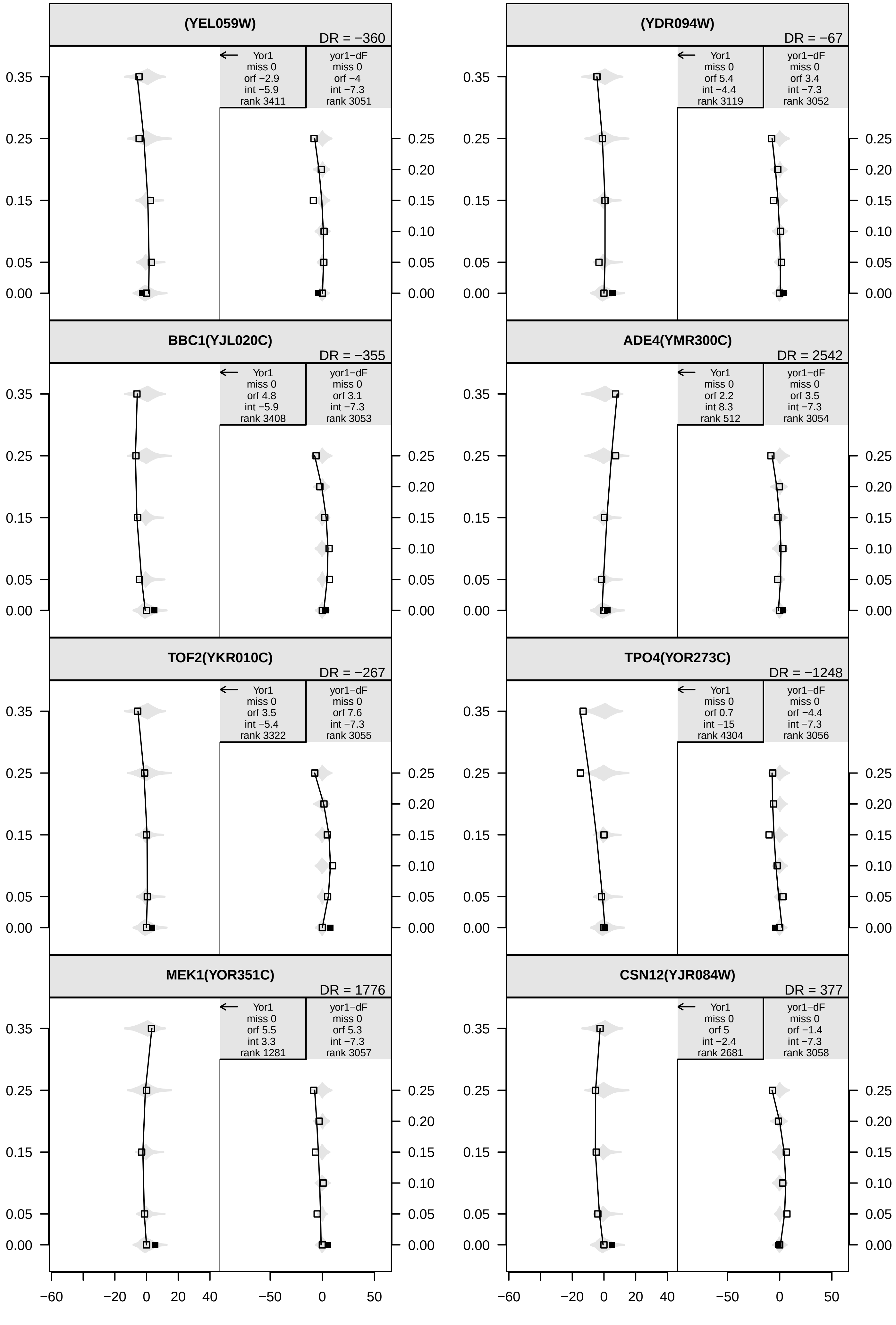

(YEL059W)
DR = −360
Yor1 miss 0 orf −2.9 int −5.9 rank 3411
yor1−dF miss 0 orf −4 int −7.3 rank 3051
(YDR094W)
DR = −67
Yor1 miss 0 orf 5.4 int −4.4 rank 3119
yor1−dF miss 0 orf 3.4 int −7.3 rank 3052
BBC1(YJL020C)
DR = −355
Yor1 miss 0 orf 4.8 int −5.9 rank 3408
yor1−dF miss 0 orf 3.1 int −7.3 rank 3053
ADE4(YMR300C)
DR = 2542
Yor1 miss 0 orf 2.2 int 8.3 rank 512
yor1−dF miss 0 orf 3.5 int −7.3 rank 3054
TOF2(YKR010C)
DR = −267
Yor1 miss 0 orf 3.5 int −5.4 rank 3322
yor1−dF miss 0 orf 7.6 int −7.3 rank 3055
TPO4(YOR273C)
DR = −1248
Yor1 miss 0 orf 0.7 int −15 rank 4304
yor1−dF miss 0 orf −4.4 int −7.3 rank 3056
MEK1(YOR351C)
DR = 1776
Yor1 miss 0 orf 5.5 int 3.3 rank 1281
yor1−dF miss 0 orf 5.3 int −7.3 rank 3057
CSN12(YJR084W)
DR = 377
Yor1 miss 0 orf 5 int −2.4 rank 2681
yor1−dF miss 0 orf −1.4 int −7.3 rank 3058

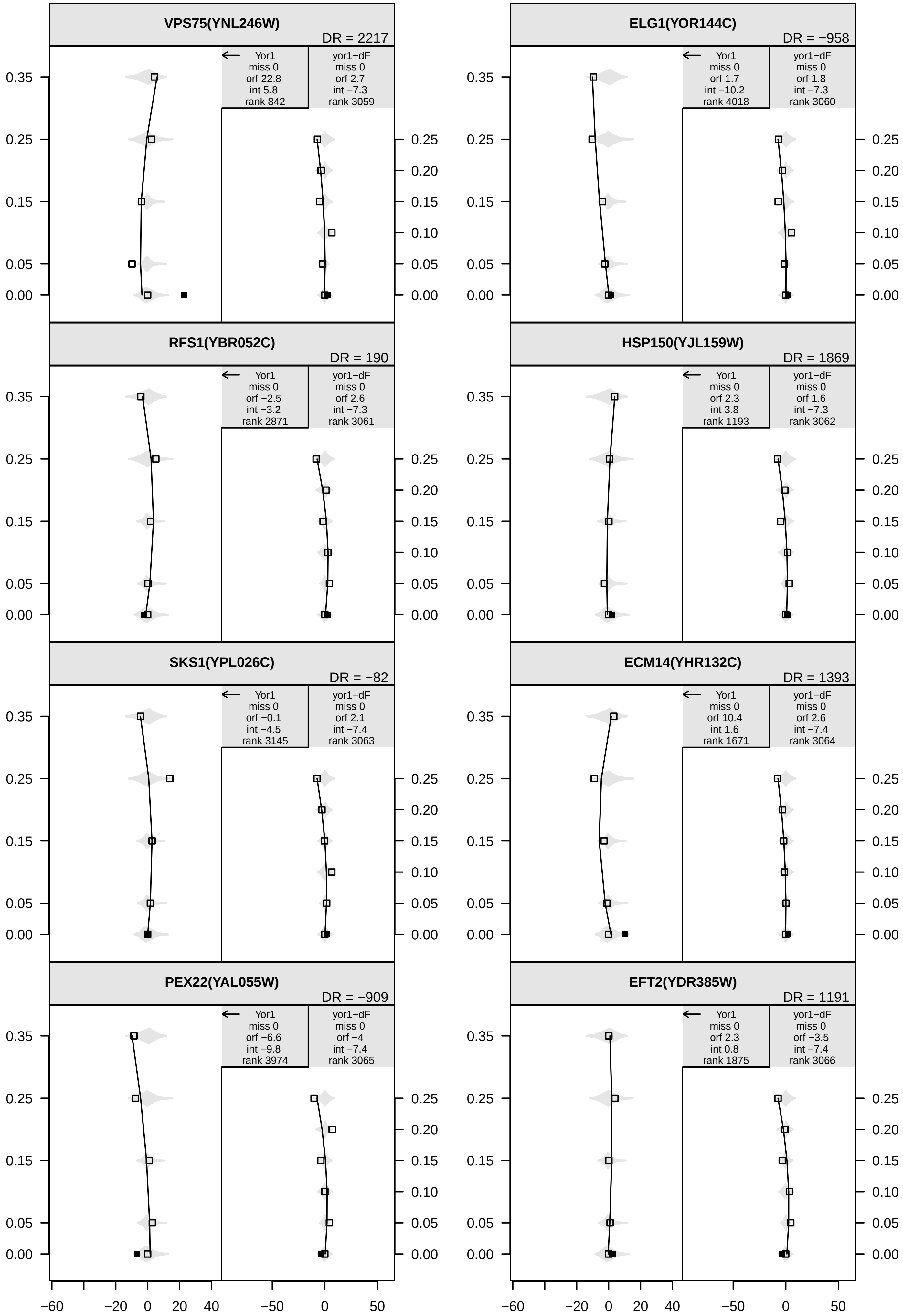

VPS75(YNL246W)
DR = 2217
Yor1
miss 0
orf 22.8
int 5.8
rank 842
yor1−dF
miss 0
orf 2.7
int −7.3
rank 3059
ELG1(YOR144C)
DR = −958
Yor1
miss 0
orf 1.7
int −10.2
rank 4018
yor1−dF
miss 0
orf 1.8
int −7.3
rank 3060
RFS1(YBR052C)
DR = 190
Yor1
miss 0
orf −2.5
int −3.2
rank 2871
yor1−dF
miss 0
orf 2.6
int −7.3
rank 3061
HSP150(YJL159W)
DR = 1869
Yor1
miss 0
orf 2.3
int 3.8
rank 1193
yor1−dF
miss 0
orf 1.6
int −7.3
rank 3062
SKS1(YPL026C)
DR = −82
Yor1
miss 0
orf −0.1
int −4.5
rank 3145
yor1−dF
miss 0
orf 2.1
int −7.4
rank 3063
ECM14(YHR132C)
DR = 1393
Yor1
miss 0
orf 10.4
int 1.6
rank 1671
yor1−dF
miss 0
orf 2.6
int −7.4
rank 3064
PEX22(YAL055W)
DR = −909
Yor1
miss 0
orf −6.6
int −9.8
rank 3974
yor1−dF
miss 0
orf −4
int −7.4
rank 3065
EFT2(YDR385W)
DR = 1191
Yor1
miss 0
orf 2.3
int 0.8
rank 1875
yor1−dF
miss 0
orf −3.5
int −7.4
rank 3066
0.35
0.25
0.20
0.15
0.10
0.05
0.00
−60
−20
0
20
40
−50
0
50

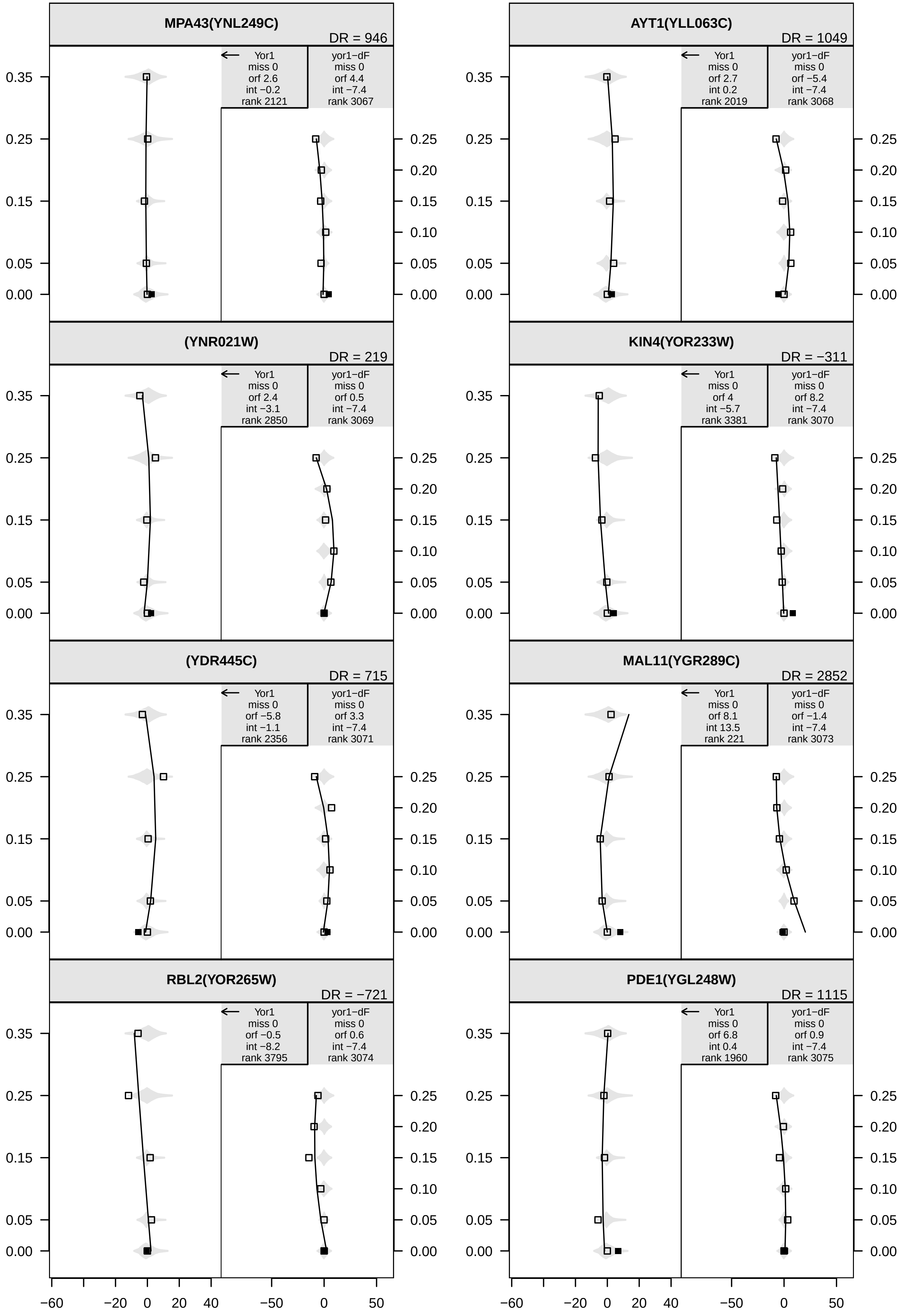
MPA43(YNL249C)
DR = 946
Yor1 miss 0 orf 2.6 int −0.2 rank 2121
yor1−dF miss 0 orf 4.4 int −7.4 rank 3067
AYT1(YLL063C)
DR = 1049
Yor1 miss 0 orf 2.7 int 0.2 rank 2019
yor1−dF miss 0 orf −5.4 int −7.4 rank 3068
(YNR021W)
DR = 219
Yor1 miss 0 orf 2.4 int −3.1 rank 2850
yor1−dF miss 0 orf 0.5 int −7.4 rank 3069
KIN4(YOR233W)
DR = −311
Yor1 miss 0 orf 4 int −5.7 rank 3381
yor1−dF miss 0 orf 8.2 int −7.4 rank 3070
(YDR445C)
DR = 715
Yor1 miss 0 orf −5.8 int −1.1 rank 2356
yor1−dF miss 0 orf 3.3 int −7.4 rank 3071
MAL11(YGR289C)
DR = 2852
Yor1 miss 0 orf 8.1 int 13.5 rank 221
yor1−dF miss 0 orf −1.4 int −7.4 rank 3073
RBL2(YOR265W)
DR = −721
Yor1 miss 0 orf −0.5 int −8.2 rank 3795
yor1−dF miss 0 orf 0.6 int −7.4 rank 3074
PDE1(YGL248W)
DR = 1115
Yor1 miss 0 orf 6.8 int 0.4 rank 1960
yor1−dF miss 0 orf 0.9 int −7.4 rank 3075

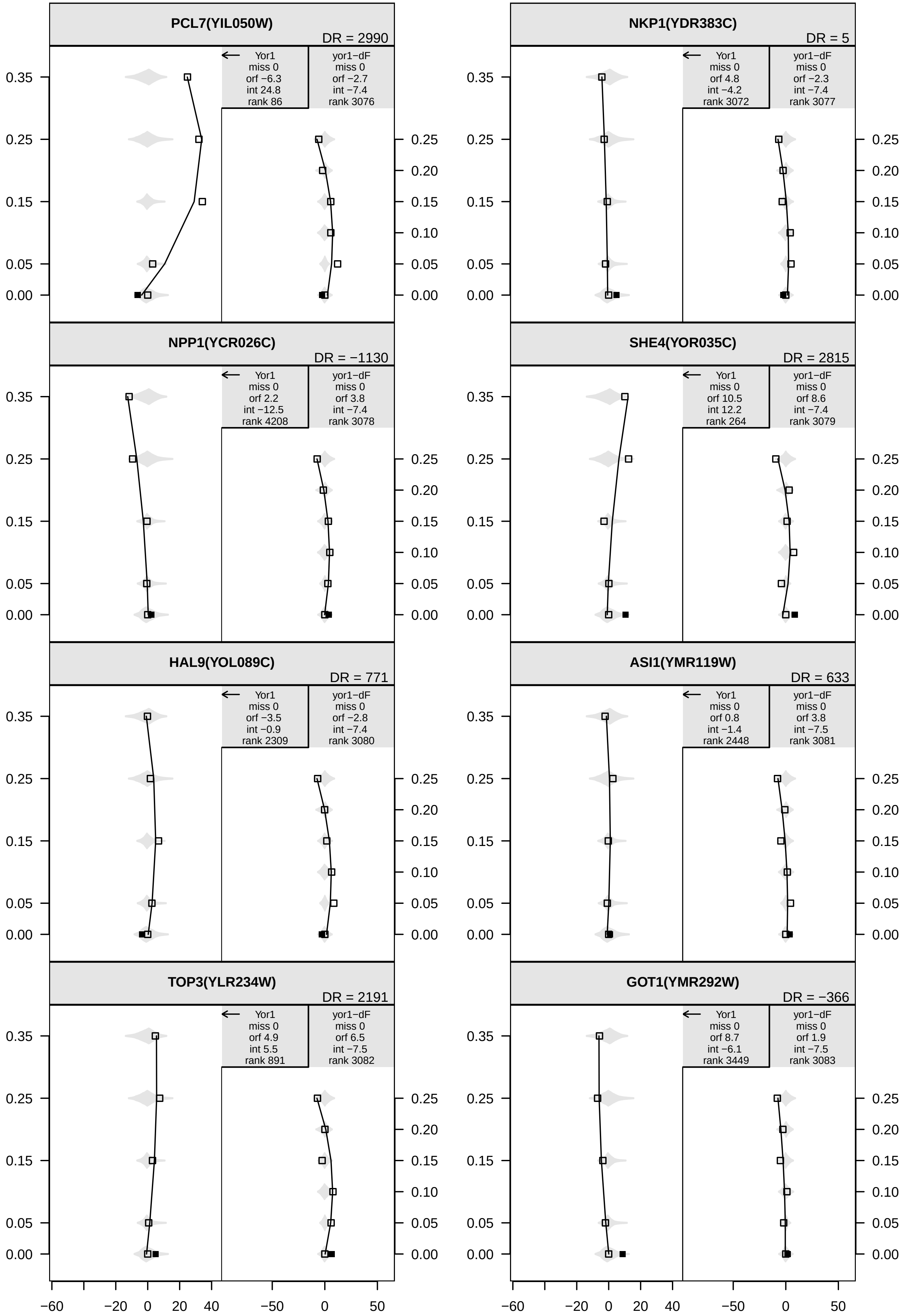

PCL7(YIL050W)
DR = 2990
Yor1 miss 0 orf −6.3 int 24.8 rank 86
yor1−dF miss 0 orf −2.7 int −7.4 rank 3076
NKP1(YDR383C)
DR = 5
Yor1 miss 0 orf 4.8 int −4.2 rank 3072
yor1−dF miss 0 orf −2.3 int −7.4 rank 3077
NPP1(YCR026C)
DR = −1130
Yor1 miss 0 orf 2.2 int −12.5 rank 4208
yor1−dF miss 0 orf 3.8 int −7.4 rank 3078
SHE4(YOR035C)
DR = 2815
Yor1 miss 0 orf 10.5 int 12.2 rank 264
yor1−dF miss 0 orf 8.6 int −7.4 rank 3079
HAL9(YOL089C)
DR = 771
Yor1 miss 0 orf −3.5 int −0.9 rank 2309
yor1−dF miss 0 orf −2.8 int −7.4 rank 3080
ASI1(YMR119W)
DR = 633
Yor1 miss 0 orf 0.8 int −1.4 rank 2448
yor1−dF miss 0 orf 3.8 int −7.5 rank 3081
TOP3(YLR234W)
DR = 2191
Yor1 miss 0 orf 4.9 int 5.5 rank 891
yor1−dF miss 0 orf 6.5 int −7.5 rank 3082
GOT1(YMR292W)
DR = −366
Yor1 miss 0 orf 8.7 int −6.1 rank 3449
yor1−dF miss 0 orf 1.9 int −7.5 rank 3083

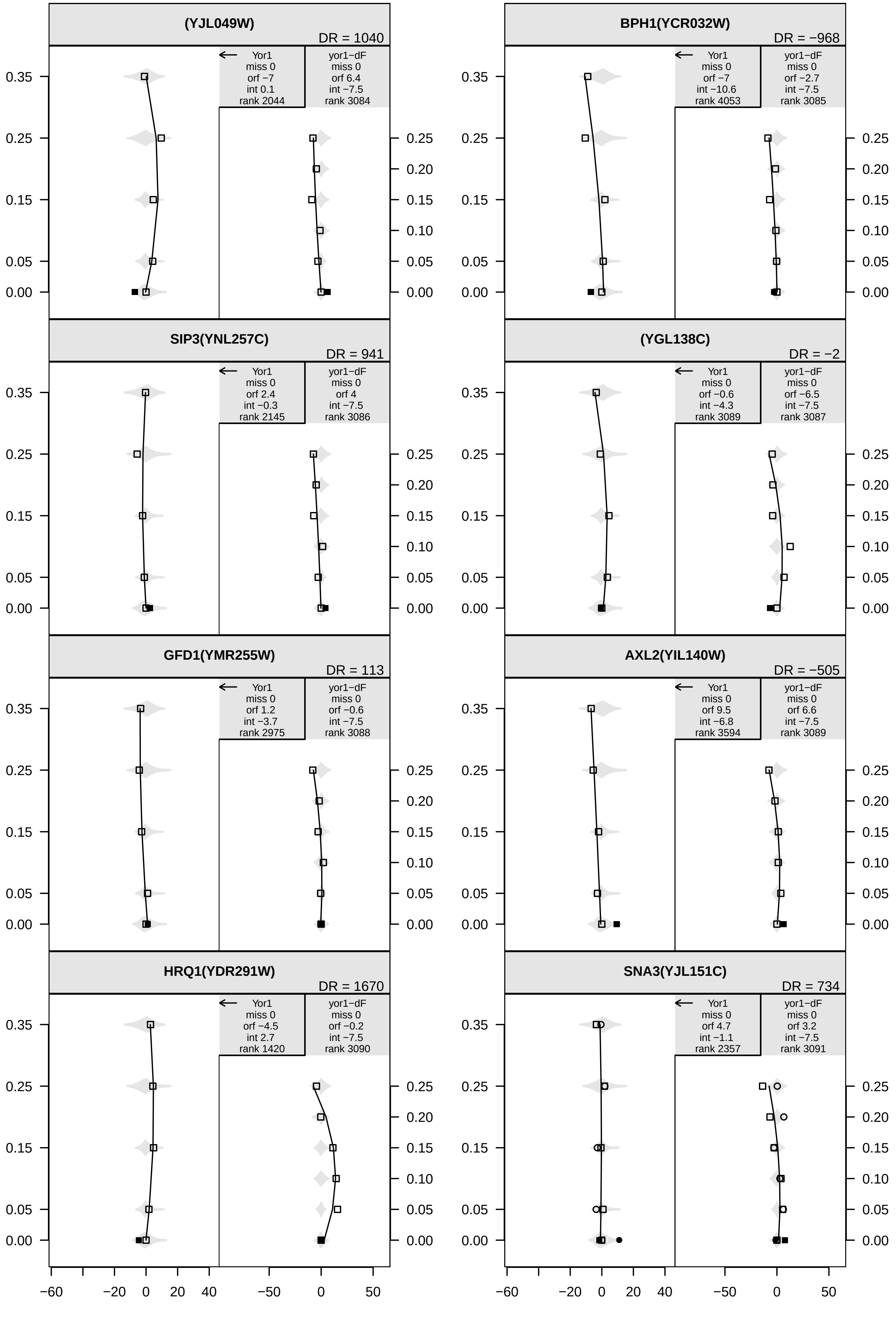

(YJL049W)
DR = 1040
Yor1 miss 0 orf −7 int 0.1 rank 2044
yor1−dF miss 0 orf 6.4 int −7.5 rank 3084
BPH1(YCR032W)
DR = −968
Yor1 miss 0 orf −7 int −10.6 rank 4053
yor1−dF miss 0 orf −2.7 int −7.5 rank 3085
SIP3(YNL257C)
DR = 941
Yor1 miss 0 orf 2.4 int −0.3 rank 2145
yor1−dF miss 0 orf 4 int −7.5 rank 3086
(YGL138C)
DR = −2
Yor1 miss 0 orf −0.6 int −4.3 rank 3089
yor1−dF miss 0 orf −6.5 int −7.5 rank 3087
GFD1(YMR255W)
DR = 113
Yor1 miss 0 orf 1.2 int −3.7 rank 2975
yor1−dF miss 0 orf −0.6 int −7.5 rank 3088
AXL2(YIL140W)
DR = −505
Yor1 miss 0 orf 9.5 int −6.8 rank 3594
yor1−dF miss 0 orf 6.6 int −7.5 rank 3089
HRQ1(YDR291W)
DR = 1670
Yor1 miss 0 orf −4.5 int 2.7 rank 1420
yor1−dF miss 0 orf −0.2 int −7.5 rank 3090
SNA3(YJL151C)
DR = 734
Yor1 miss 0 orf 4.7 int −1.1 rank 2357
yor1−dF miss 0 orf 3.2 int −7.5 rank 3091
0.35 0.25 0.20 0.15 0.10 0.05 0.00
−60 −20 0 20 40 −50 0 50

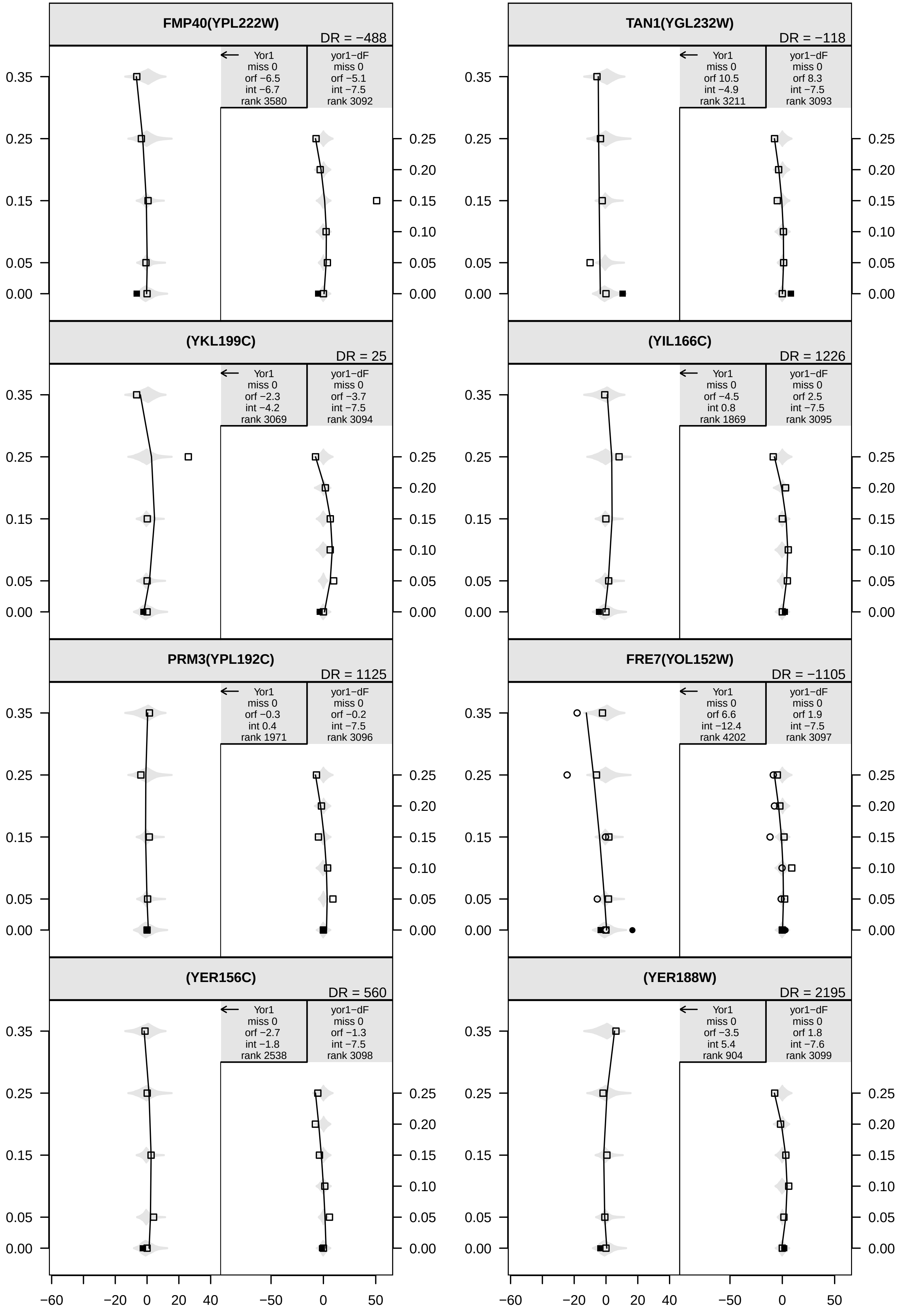
FMP40(YPL222W)
DR = −488
Yor1 miss 0 orf −6.5 int −6.7 rank 3580
yor1−dF miss 0 orf −5.1 int −7.5 rank 3092
TAN1(YGL232W)
DR = −118
Yor1 miss 0 orf 10.5 int −4.9 rank 3211
yor1−dF miss 0 orf 8.3 int −7.5 rank 3093
(YKL199C)
DR = 25
Yor1 miss 0 orf −2.3 int −4.2 rank 3069
yor1−dF miss 0 orf −3.7 int −7.5 rank 3094
(YIL166C)
DR = 1226
Yor1 miss 0 orf −4.5 int 0.8 rank 1869
yor1−dF miss 0 orf 2.5 int −7.5 rank 3095
PRM3(YPL192C)
DR = 1125
Yor1 miss 0 orf −0.3 int 0.4 rank 1971
yor1−dF miss 0 orf −0.2 int −7.5 rank 3096
FRE7(YOL152W)
DR = −1105
Yor1 miss 0 orf 6.6 int −12.4 rank 4202
yor1−dF miss 0 orf 1.9 int −7.5 rank 3097
(YER156C)
DR = 560
Yor1 miss 0 orf −2.7 int −1.8 rank 2538
yor1−dF miss 0 orf −1.3 int −7.5 rank 3098
(YER188W)
DR = 2195
Yor1 miss 0 orf −3.5 int 5.4 rank 904
yor1−dF miss 0 orf 1.8 int −7.6 rank 3099
0.35 0.25 0.20 0.15 0.10 0.05 0.00
−60 −20 0 20 40 −50 0 50

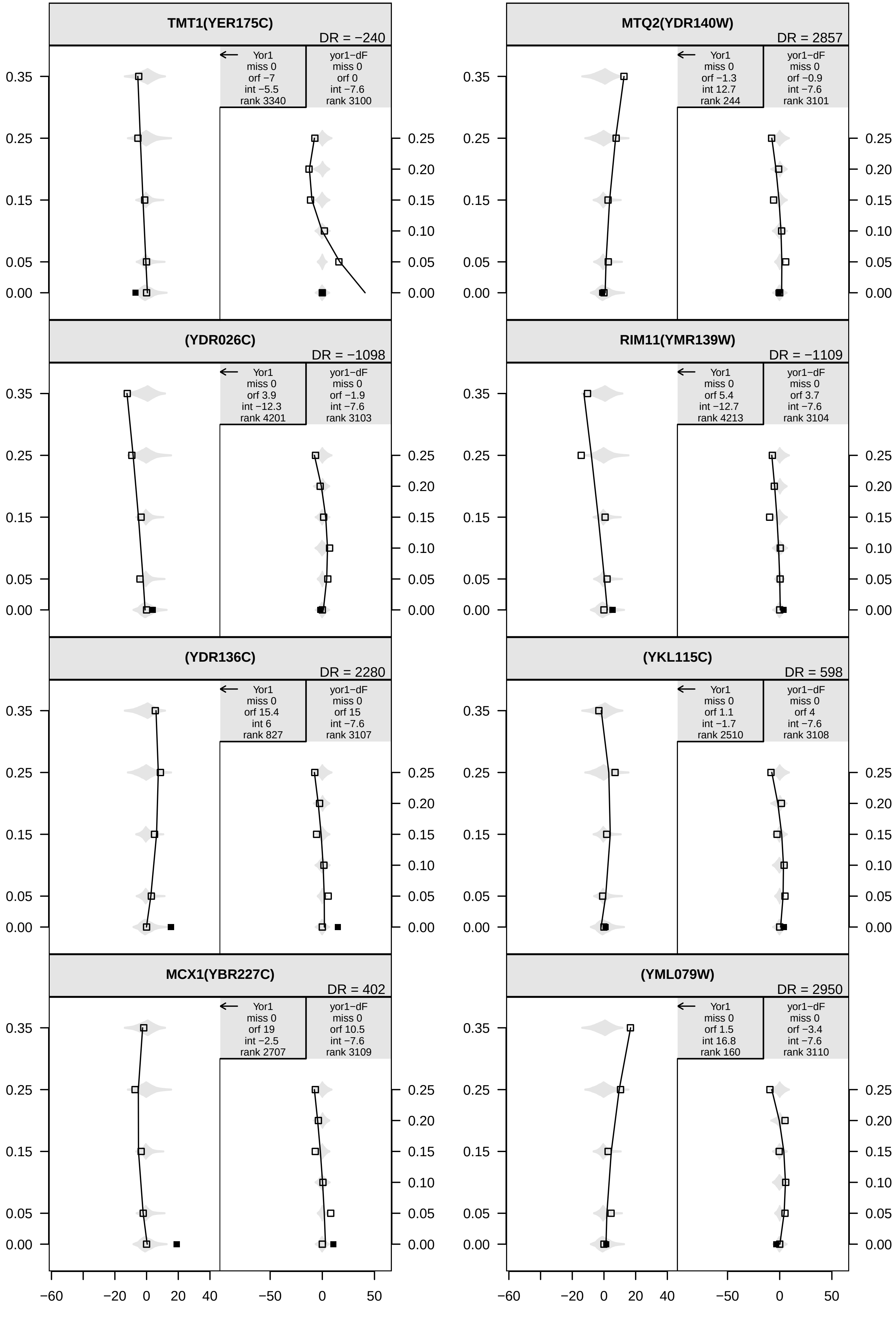
TMT1(YER175C)
DR = −240
Yor1
miss 0
orf −7
int −5.5
rank 3340
yor1−dF
miss 0
orf 0
int −7.6
rank 3100
MTQ2(YDR140W)
DR = 2857
Yor1
miss 0
orf −1.3
int 12.7
rank 244
yor1−dF
miss 0
orf −0.9
int −7.6
rank 3101
(YDR026C)
DR = −1098
Yor1
miss 0
orf 3.9
int −12.3
rank 4201
yor1−dF
miss 0
orf −1.9
int −7.6
rank 3103
RIM11(YMR139W)
DR = −1109
Yor1
miss 0
orf 5.4
int −12.7
rank 4213
yor1−dF
miss 0
orf 3.7
int −7.6
rank 3104
(YDR136C)
DR = 2280
Yor1
miss 0
orf 15.4
int 6
rank 827
yor1−dF
miss 0
orf 15
int −7.6
rank 3107
(YKL115C)
DR = 598
Yor1
miss 0
orf 1.1
int −1.7
rank 2510
yor1−dF
miss 0
orf 4
int −7.6
rank 3108
MCX1(YBR227C)
DR = 402
Yor1
miss 0
orf 19
int −2.5
rank 2707
yor1−dF
miss 0
orf 10.5
int −7.6
rank 3109
(YML079W)
DR = 2950
Yor1
miss 0
orf 1.5
int 16.8
rank 160
yor1−dF
miss 0
orf −3.4
int −7.6
rank 3110
0.00
0.05
0.10
0.15
0.20
0.25
0.35
−60
−20
0
20
40
−50
0
50

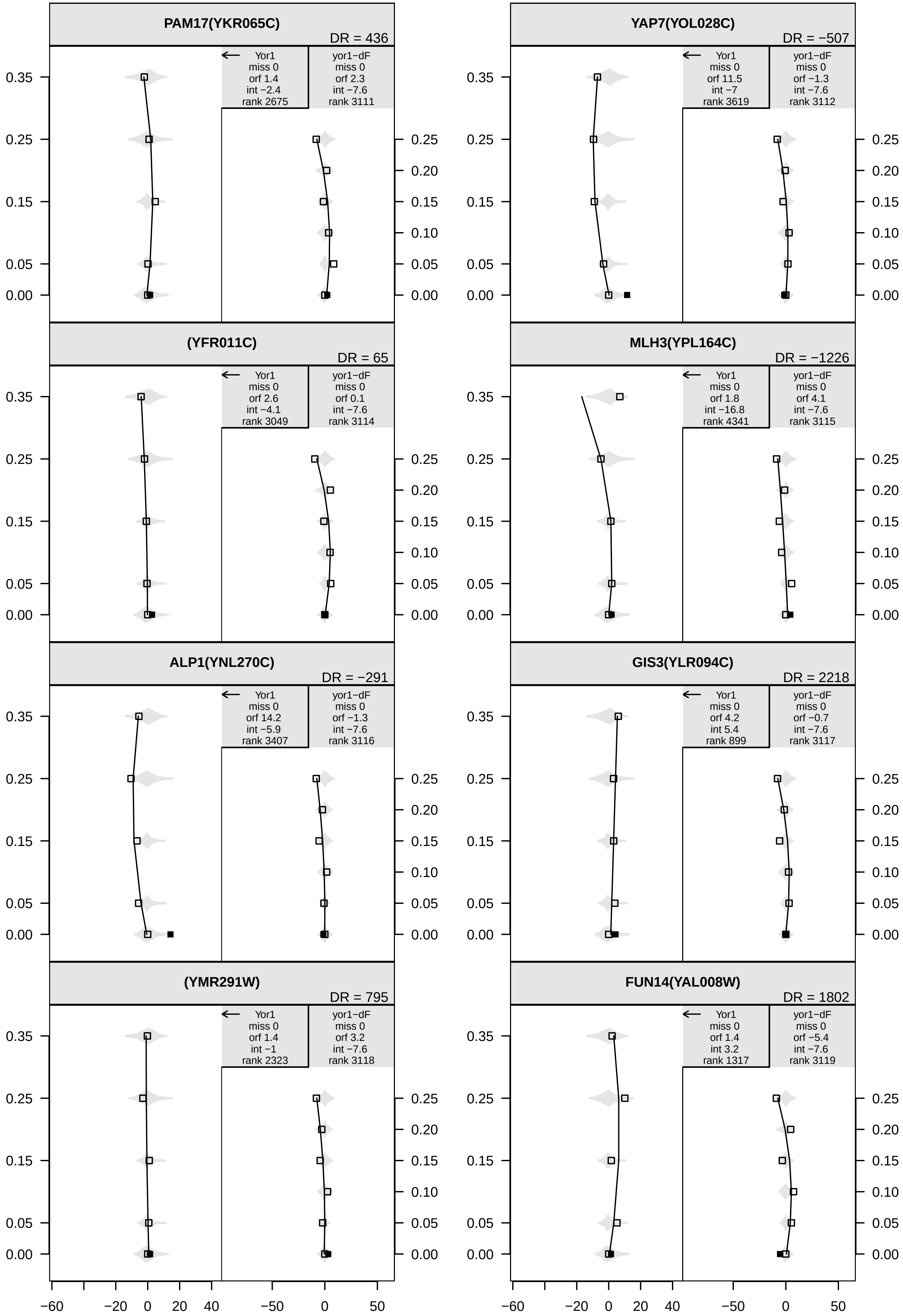
PAM17(YKR065C)
DR = 436
Yor1 miss 0 orf 1.4 int −2.4 rank 2675
yor1−dF miss 0 orf 2.3 int −7.6 rank 3111
YAP7(YOL028C)
DR = −507
Yor1 miss 0 orf 11.5 int −7 rank 3619
yor1−dF miss 0 orf −1.3 int −7.6 rank 3112
(YFR011C)
DR = 65
Yor1 miss 0 orf 2.6 int −4.1 rank 3049
yor1−dF miss 0 orf 0.1 int −7.6 rank 3114
MLH3(YPL164C)
DR = −1226
Yor1 miss 0 orf 1.8 int −16.8 rank 4341
yor1−dF miss 0 orf 4.1 int −7.6 rank 3115
ALP1(YNL270C)
DR = −291
Yor1 miss 0 orf 14.2 int −5.9 rank 3407
yor1−dF miss 0 orf −1.3 int −7.6 rank 3116
GIS3(YLR094C)
DR = 2218
Yor1 miss 0 orf 4.2 int 5.4 rank 899
yor1−dF miss 0 orf −0.7 int −7.6 rank 3117
(YMR291W)
DR = 795
Yor1 miss 0 orf 1.4 int −1 rank 2323
yor1−dF miss 0 orf 3.2 int −7.6 rank 3118
FUN14(YAL008W)
DR = 1802
Yor1 miss 0 orf 1.4 int 3.2 rank 1317
yor1−dF miss 0 orf −5.4 int −7.6 rank 3119
0.00 0.05 0.10 0.15 0.20 0.25 0.35
−60 −20 0 20 40 −50 0 50

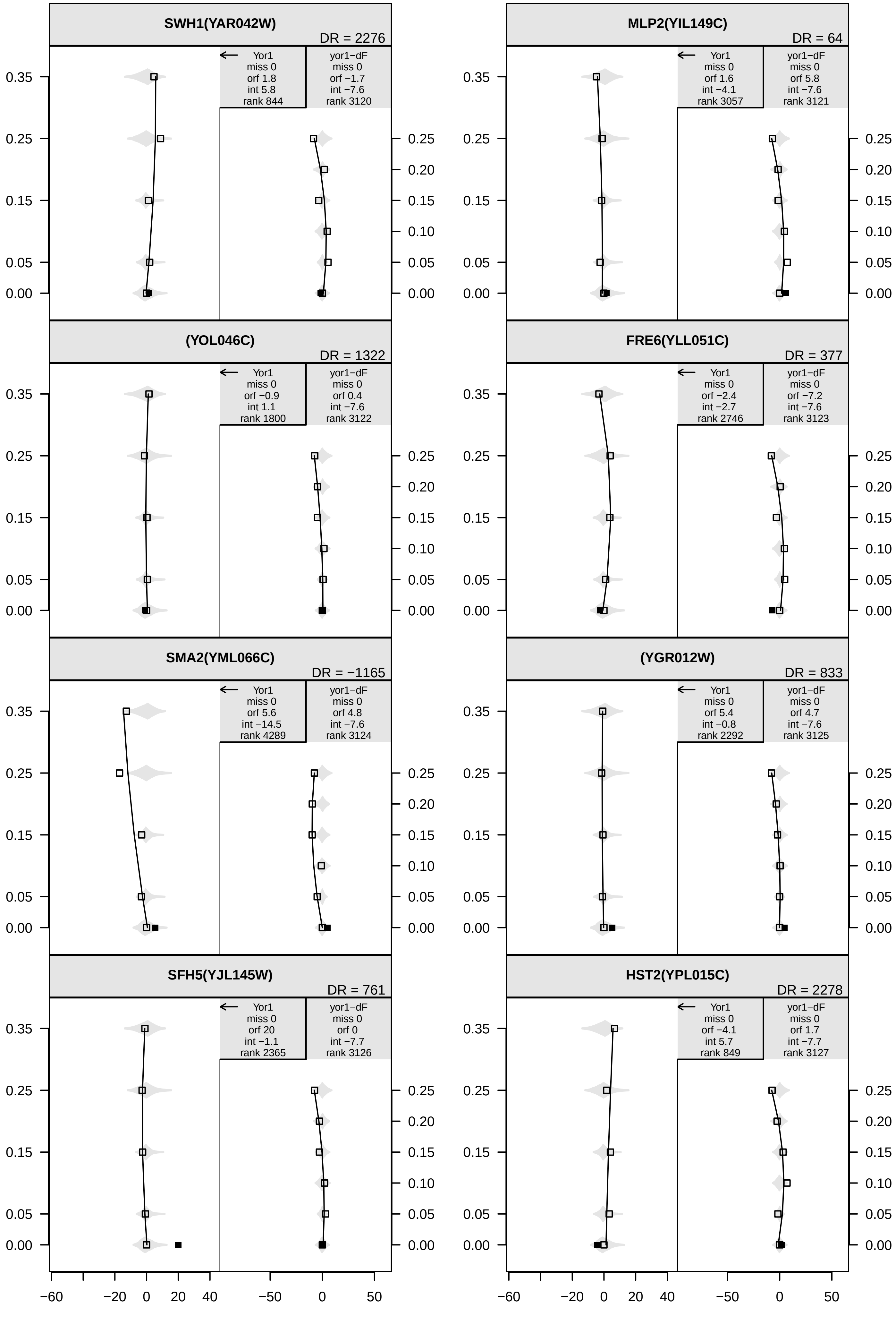

SWH1(YAR042W)
DR = 2276
Yor1 miss 0 orf 1.8 int 5.8 rank 844
yor1−dF miss 0 orf −1.7 int −7.6 rank 3120
MLP2(YIL149C)
DR = 64
Yor1 miss 0 orf 1.6 int −4.1 rank 3057
yor1−dF miss 0 orf 5.8 int −7.6 rank 3121
(YOL046C)
DR = 1322
Yor1 miss 0 orf −0.9 int 1.1 rank 1800
yor1−dF miss 0 orf 0.4 int −7.6 rank 3122
FRE6(YLL051C)
DR = 377
Yor1 miss 0 orf −2.4 int −2.7 rank 2746
yor1−dF miss 0 orf −7.2 int −7.6 rank 3123
SMA2(YML066C)
DR = −1165
Yor1 miss 0 orf 5.6 int −14.5 rank 4289
yor1−dF miss 0 orf 4.8 int −7.6 rank 3124
(YGR012W)
DR = 833
Yor1 miss 0 orf 5.4 int −0.8 rank 2292
yor1−dF miss 0 orf 4.7 int −7.6 rank 3125
SFH5(YJL145W)
DR = 761
Yor1 miss 0 orf 20 int −1.1 rank 2365
yor1−dF miss 0 orf 0 int −7.7 rank 3126
HST2(YPL015C)
DR = 2278
Yor1 miss 0 orf −4.1 int 5.7 rank 849
yor1−dF miss 0 orf 1.7 int −7.7 rank 3127
0.35 0.25 0.20 0.15 0.10 0.05 0.00
−60 −20 0 20 40 −50 0 50

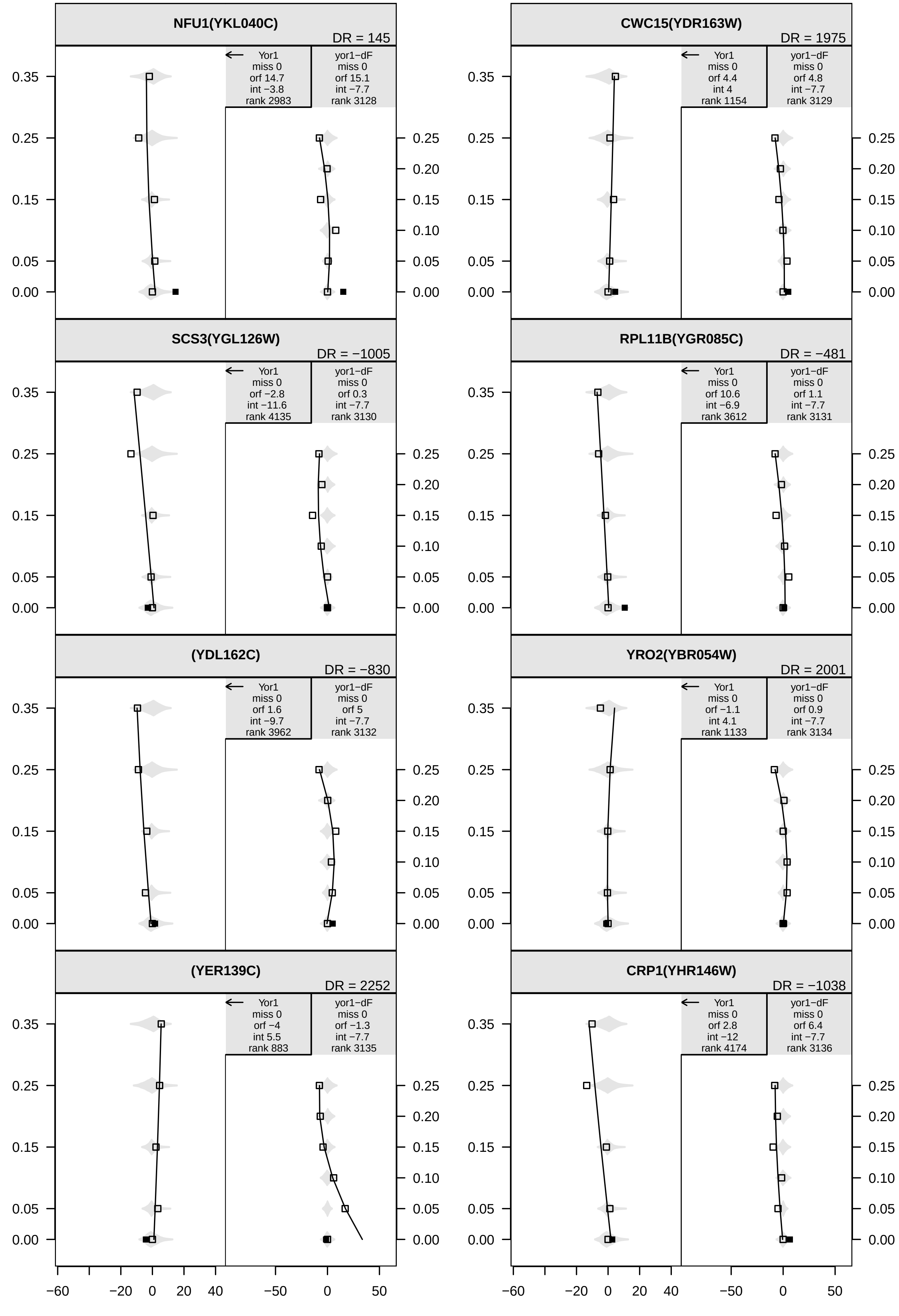

NFU1(YKL040C)
DR = 145
Yor1 miss 0 orf 14.7 int −3.8 rank 2983
yor1−dF miss 0 orf 15.1 int −7.7 rank 3128
CWC15(YDR163W)
DR = 1975
Yor1 miss 0 orf 4.4 int 4 rank 1154
yor1−dF miss 0 orf 4.8 int −7.7 rank 3129
SCS3(YGL126W)
DR = −1005
Yor1 miss 0 orf −2.8 int −11.6 rank 4135
yor1−dF miss 0 orf 0.3 int −7.7 rank 3130
RPL11B(YGR085C)
DR = −481
Yor1 miss 0 orf 10.6 int −6.9 rank 3612
yor1−dF miss 0 orf 1.1 int −7.7 rank 3131
(YDL162C)
DR = −830
Yor1 miss 0 orf 1.6 int −9.7 rank 3962
yor1−dF miss 0 orf 5 int −7.7 rank 3132
YRO2(YBR054W)
DR = 2001
Yor1 miss 0 orf −1.1 int 4.1 rank 1133
yor1−dF miss 0 orf 0.9 int −7.7 rank 3134
(YER139C)
DR = 2252
Yor1 miss 0 orf −4 int 5.5 rank 883
yor1−dF miss 0 orf −1.3 int −7.7 rank 3135
CRP1(YHR146W)
DR = −1038
Yor1 miss 0 orf 2.8 int −12 rank 4174
yor1−dF miss 0 orf 6.4 int −7.7 rank 3136
0.00 0.05 0.10 0.15 0.20 0.25 0.35
−60 −20 0 20 40 −50 0 50

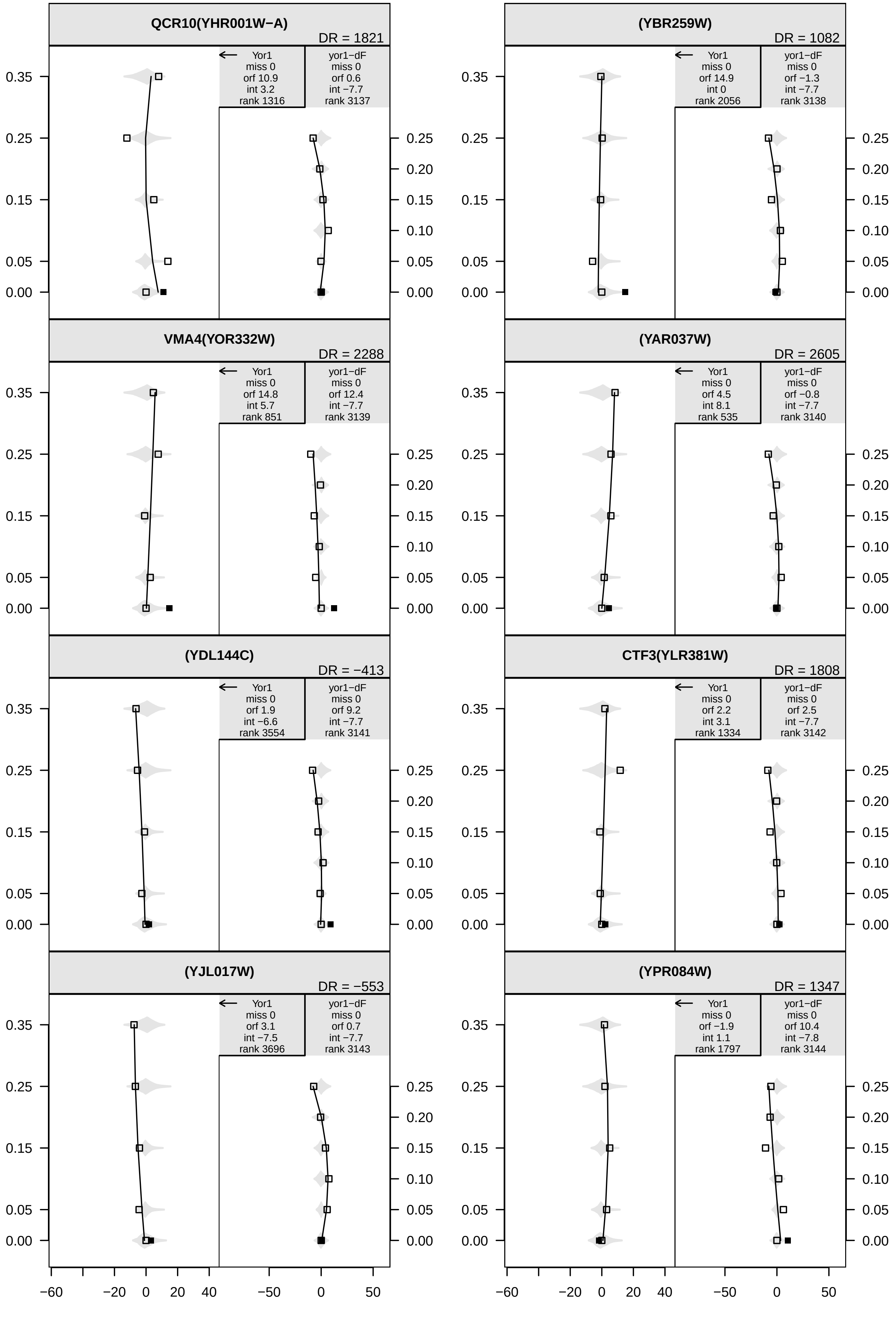
QCR10(YHR001W−A)
DR = 1821
Yor1 miss 0 orf 10.9 int 3.2 rank 1316
yor1−dF miss 0 orf 0.6 int −7.7 rank 3137
(YBR259W)
DR = 1082
Yor1 miss 0 orf 14.9 int 0 rank 2056
yor1−dF miss 0 orf −1.3 int −7.7 rank 3138
VMA4(YOR332W)
DR = 2288
Yor1 miss 0 orf 14.8 int 5.7 rank 851
yor1−dF miss 0 orf 12.4 int −7.7 rank 3139
(YAR037W)
DR = 2605
Yor1 miss 0 orf 4.5 int 8.1 rank 535
yor1−dF miss 0 orf −0.8 int −7.7 rank 3140
(YDL144C)
DR = −413
Yor1 miss 0 orf 1.9 int −6.6 rank 3554
yor1−dF miss 0 orf 9.2 int −7.7 rank 3141
CTF3(YLR381W)
DR = 1808
Yor1 miss 0 orf 2.2 int 3.1 rank 1334
yor1−dF miss 0 orf 2.5 int −7.7 rank 3142
(YJL017W)
DR = −553
Yor1 miss 0 orf 3.1 int −7.5 rank 3696
yor1−dF miss 0 orf 0.7 int −7.7 rank 3143
(YPR084W)
DR = 1347
Yor1 miss 0 orf −1.9 int 1.1 rank 1797
yor1−dF miss 0 orf 10.4 int −7.8 rank 3144

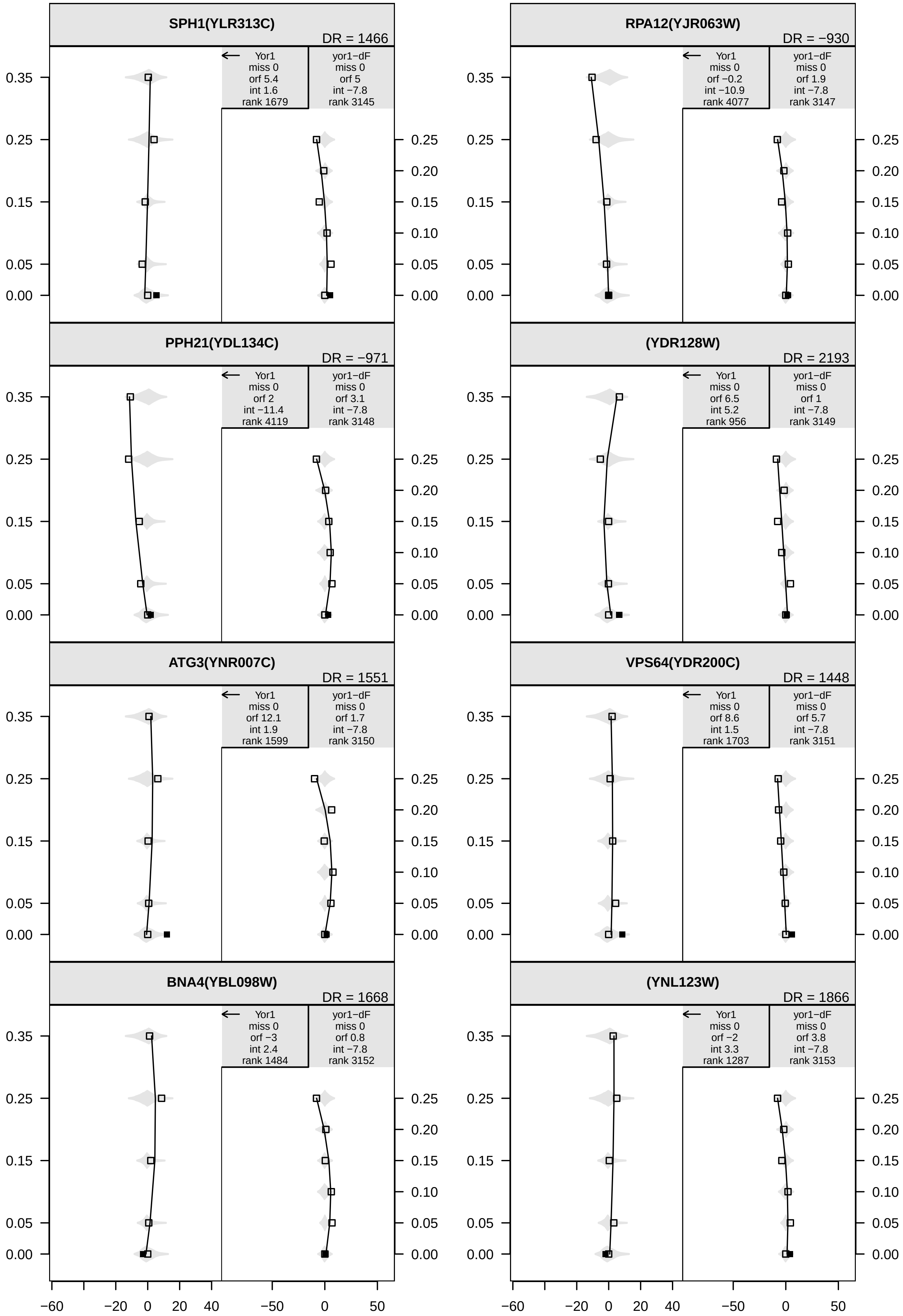

SPH1(YLR313C)
DR = 1466
Yor1 miss 0 orf 5.4 int 1.6 rank 1679
yor1−dF miss 0 orf 5 int −7.8 rank 3145
RPA12(YJR063W)
DR = −930
Yor1 miss 0 orf −0.2 int −10.9 rank 4077
yor1−dF miss 0 orf 1.9 int −7.8 rank 3147
PPH21(YDL134C)
DR = −971
Yor1 miss 0 orf 2 int −11.4 rank 4119
yor1−dF miss 0 orf 3.1 int −7.8 rank 3148
(YDR128W)
DR = 2193
Yor1 miss 0 orf 6.5 int 5.2 rank 956
yor1−dF miss 0 orf 1 int −7.8 rank 3149
ATG3(YNR007C)
DR = 1551
Yor1 miss 0 orf 12.1 int 1.9 rank 1599
yor1−dF miss 0 orf 1.7 int −7.8 rank 3150
VPS64(YDR200C)
DR = 1448
Yor1 miss 0 orf 8.6 int 1.5 rank 1703
yor1−dF miss 0 orf 5.7 int −7.8 rank 3151
BNA4(YBL098W)
DR = 1668
Yor1 miss 0 orf −3 int 2.4 rank 1484
yor1−dF miss 0 orf 0.8 int −7.8 rank 3152
(YNL123W)
DR = 1866
Yor1 miss 0 orf −2 int 3.3 rank 1287
yor1−dF miss 0 orf 3.8 int −7.8 rank 3153
0.35
0.25
0.20
0.15
0.10
0.05
0.00
−60
−20
0
20
40
−50
0
50

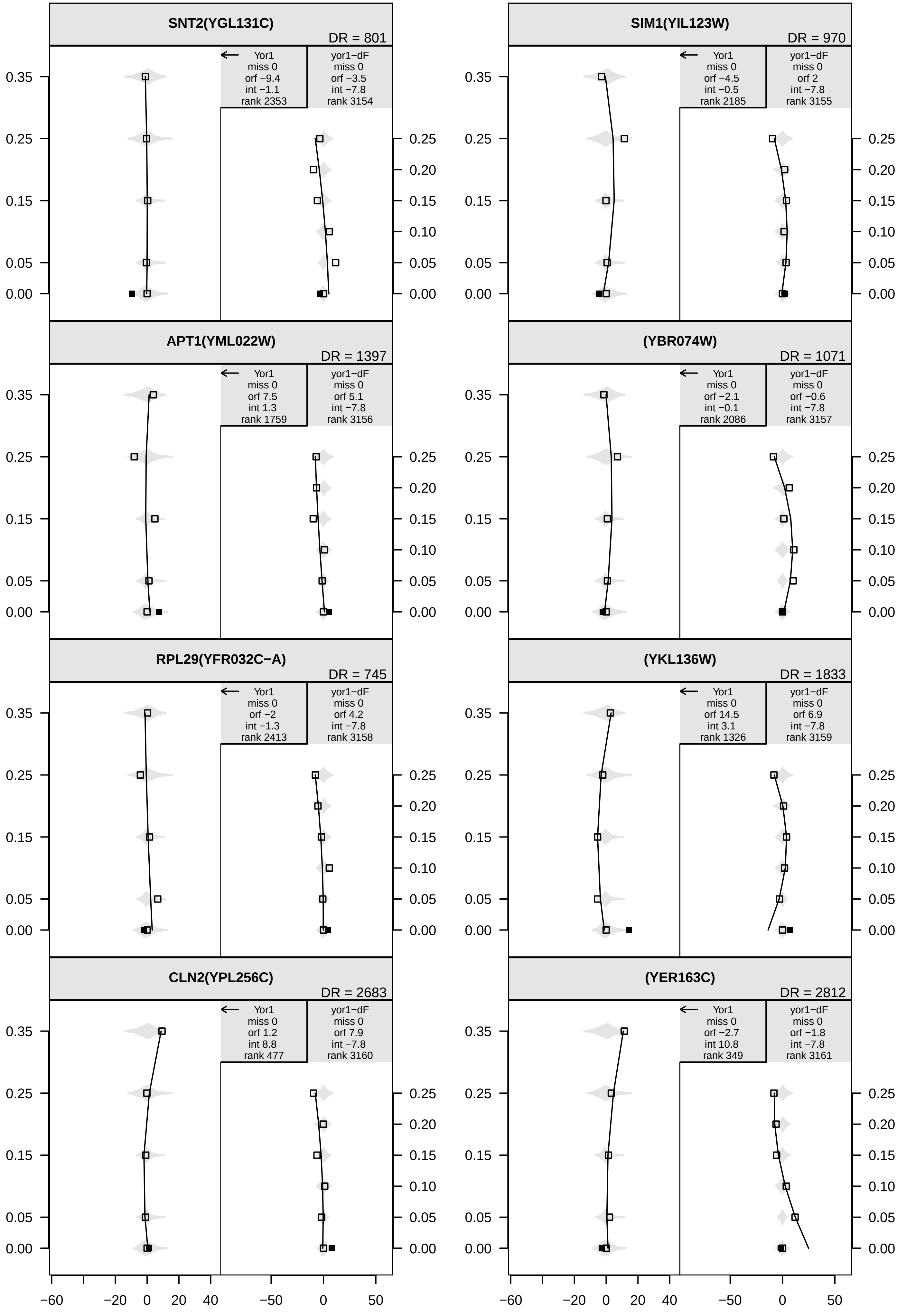
SNT2(YGL131C)
DR = 801
Yor1
miss 0
orf −9.4
int −1.1
rank 2353
yor1−dF
miss 0
orf −3.5
int −7.8
rank 3154
SIM1(YIL123W)
DR = 970
Yor1
miss 0
orf −4.5
int −0.5
rank 2185
yor1−dF
miss 0
orf 2
int −7.8
rank 3155
APT1(YML022W)
DR = 1397
Yor1
miss 0
orf 7.5
int 1.3
rank 1759
yor1−dF
miss 0
orf 5.1
int −7.8
rank 3156
(YBR074W)
DR = 1071
Yor1
miss 0
orf −2.1
int −0.1
rank 2086
yor1−dF
miss 0
orf −0.6
int −7.8
rank 3157
RPL29(YFR032C−A)
DR = 745
Yor1
miss 0
orf −2
int −1.3
rank 2413
yor1−dF
miss 0
orf 4.2
int −7.8
rank 3158
(YKL136W)
DR = 1833
Yor1
miss 0
orf 14.5
int 3.1
rank 1326
yor1−dF
miss 0
orf 6.9
int −7.8
rank 3159
CLN2(YPL256C)
DR = 2683
Yor1
miss 0
orf 1.2
int 8.8
rank 477
yor1−dF
miss 0
orf 7.9
int −7.8
rank 3160
(YER163C)
DR = 2812
Yor1
miss 0
orf −2.7
int 10.8
rank 349
yor1−dF
miss 0
orf −1.8
int −7.8
rank 3161
0.35
0.25
0.20
0.15
0.10
0.05
0.00
−60
−20
0
20
40
−50
0
50

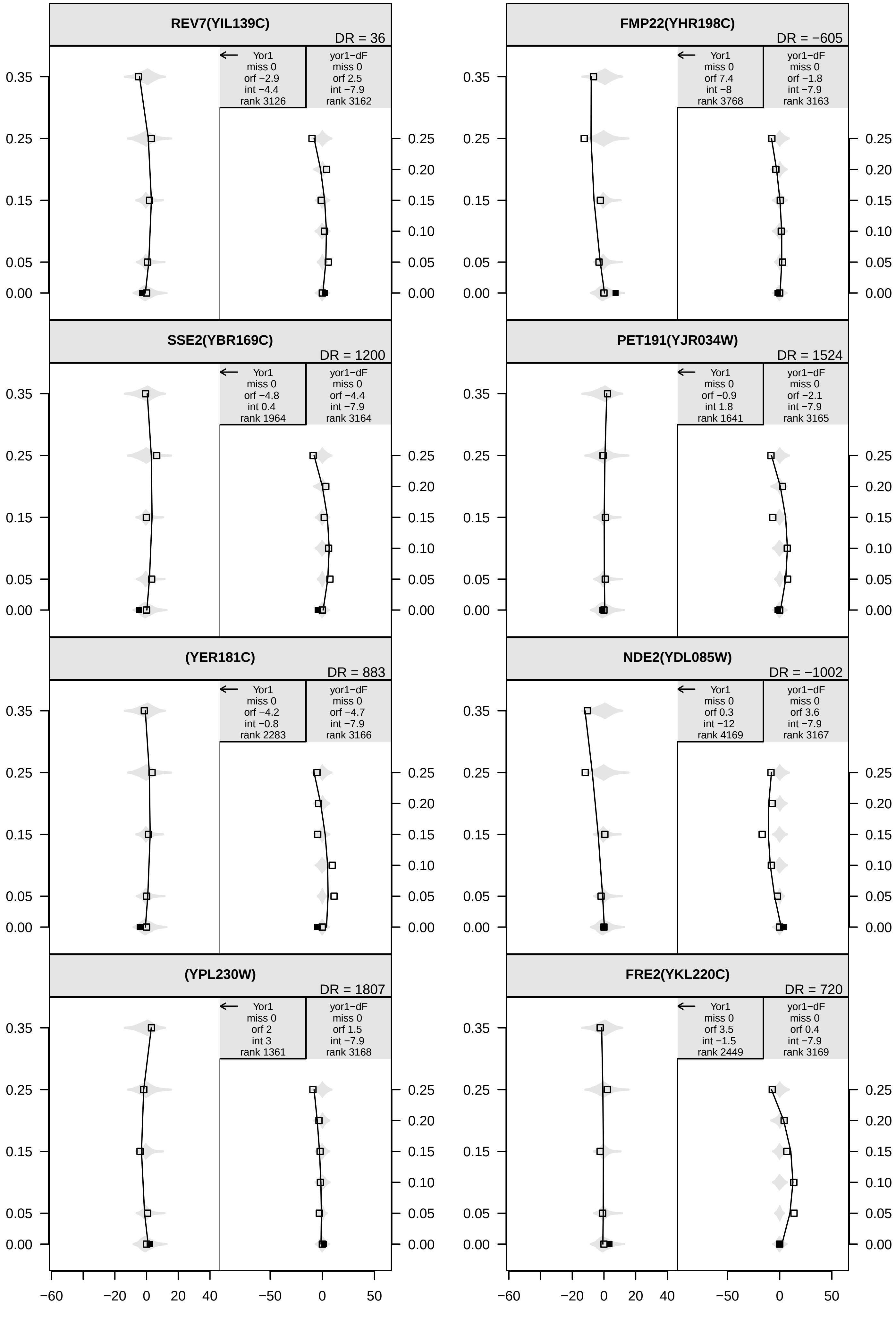

REV7(YIL139C)
DR = 36
Yor1 miss 0 orf −2.9 int −4.4 rank 3126
yor1−dF miss 0 orf 2.5 int −7.9 rank 3162
FMP22(YHR198C)
DR = −605
Yor1 miss 0 orf 7.4 int −8 rank 3768
yor1−dF miss 0 orf −1.8 int −7.9 rank 3163
SSE2(YBR169C)
DR = 1200
Yor1 miss 0 orf −4.8 int 0.4 rank 1964
yor1−dF miss 0 orf −4.4 int −7.9 rank 3164
PET191(YJR034W)
DR = 1524
Yor1 miss 0 orf −0.9 int 1.8 rank 1641
yor1−dF miss 0 orf −2.1 int −7.9 rank 3165
(YER181C)
DR = 883
Yor1 miss 0 orf −4.2 int −0.8 rank 2283
yor1−dF miss 0 orf −4.7 int −7.9 rank 3166
NDE2(YDL085W)
DR = −1002
Yor1 miss 0 orf 0.3 int −12 rank 4169
yor1−dF miss 0 orf 3.6 int −7.9 rank 3167
(YPL230W)
DR = 1807
Yor1 miss 0 orf 2 int 3 rank 1361
yor1−dF miss 0 orf 1.5 int −7.9 rank 3168
FRE2(YKL220C)
DR = 720
Yor1 miss 0 orf 3.5 int −1.5 rank 2449
yor1−dF miss 0 orf 0.4 int −7.9 rank 3169

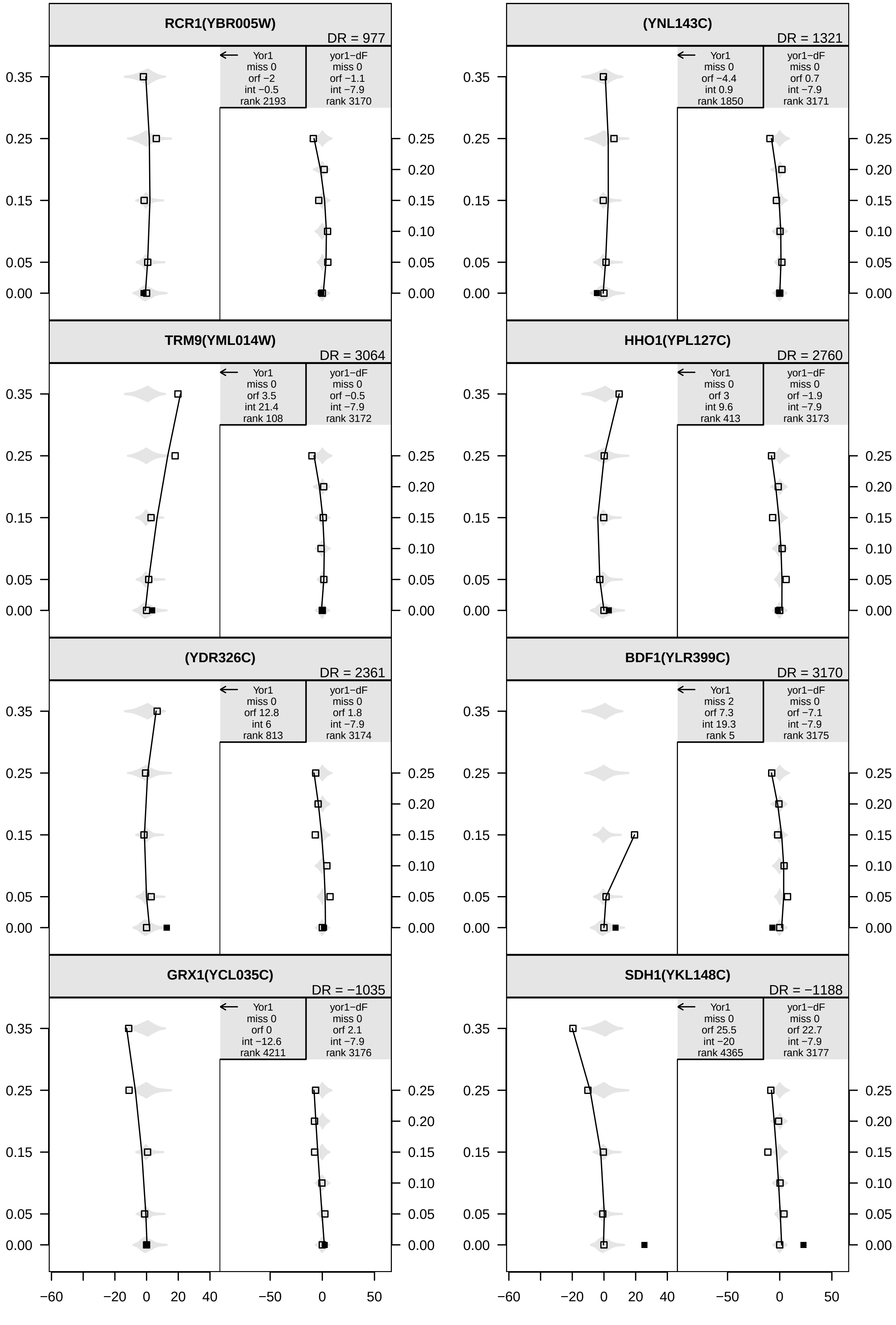

RCR1(YBR005W)
DR = 977
Yor1 miss 0 orf −2 int −0.5 rank 2193
yor1−dF miss 0 orf −1.1 int −7.9 rank 3170
(YNL143C)
DR = 1321
Yor1 miss 0 orf −4.4 int 0.9 rank 1850
yor1−dF miss 0 orf 0.7 int −7.9 rank 3171
TRM9(YML014W)
DR = 3064
Yor1 miss 0 orf 3.5 int 21.4 rank 108
yor1−dF miss 0 orf −0.5 int −7.9 rank 3172
HHO1(YPL127C)
DR = 2760
Yor1 miss 0 orf 3 int 9.6 rank 413
yor1−dF miss 0 orf −1.9 int −7.9 rank 3173
(YDR326C)
DR = 2361
Yor1 miss 0 orf 12.8 int 6 rank 813
yor1−dF miss 0 orf 1.8 int −7.9 rank 3174
BDF1(YLR399C)
DR = 3170
Yor1 miss 2 orf 7.3 int 19.3 rank 5
yor1−dF miss 0 orf −7.1 int −7.9 rank 3175
GRX1(YCL035C)
DR = −1035
Yor1 miss 0 orf 0 int −12.6 rank 4211
yor1−dF miss 0 orf 2.1 int −7.9 rank 3176
SDH1(YKL148C)
DR = −1188
Yor1 miss 0 orf 25.5 int −20 rank 4365
yor1−dF miss 0 orf 22.7 int −7.9 rank 3177
0.35 0.25 0.15 0.05 0.00
0.25 0.20 0.15 0.10 0.05 0.00
−60 −20 0 20 40 −50 0 50

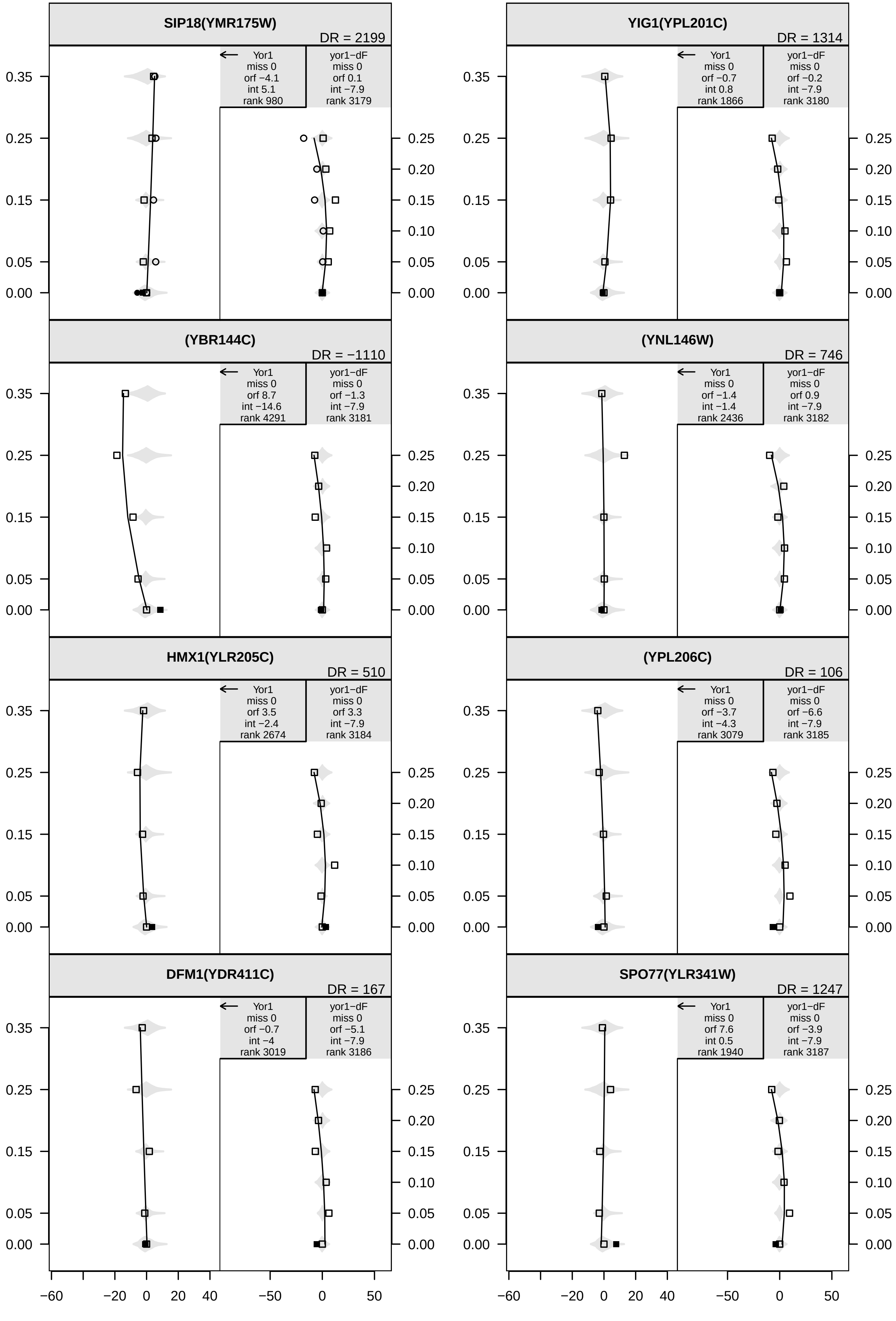

SIP18(YMR175W)
DR = 2199
Yor1 miss 0 orf −4.1 int 5.1 rank 980
yor1−dF miss 0 orf 0.1 int −7.9 rank 3179
YIG1(YPL201C)
DR = 1314
Yor1 miss 0 orf −0.7 int 0.8 rank 1866
yor1−dF miss 0 orf −0.2 int −7.9 rank 3180
(YBR144C)
DR = −1110
Yor1 miss 0 orf 8.7 int −14.6 rank 4291
yor1−dF miss 0 orf −1.3 int −7.9 rank 3181
(YNL146W)
DR = 746
Yor1 miss 0 orf −1.4 int −1.4 rank 2436
yor1−dF miss 0 orf 0.9 int −7.9 rank 3182
HMX1(YLR205C)
DR = 510
Yor1 miss 0 orf 3.5 int −2.4 rank 2674
yor1−dF miss 0 orf 3.3 int −7.9 rank 3184
(YPL206C)
DR = 106
Yor1 miss 0 orf −3.7 int −4.3 rank 3079
yor1−dF miss 0 orf −6.6 int −7.9 rank 3185
DFM1(YDR411C)
DR = 167
Yor1 miss 0 orf −0.7 int −4 rank 3019
yor1−dF miss 0 orf −5.1 int −7.9 rank 3186
SPO77(YLR341W)
DR = 1247
Yor1 miss 0 orf 7.6 int 0.5 rank 1940
yor1−dF miss 0 orf −3.9 int −7.9 rank 3187
0.00 0.05 0.10 0.15 0.20 0.25 0.35
−60 −20 0 20 40 −50 0 50

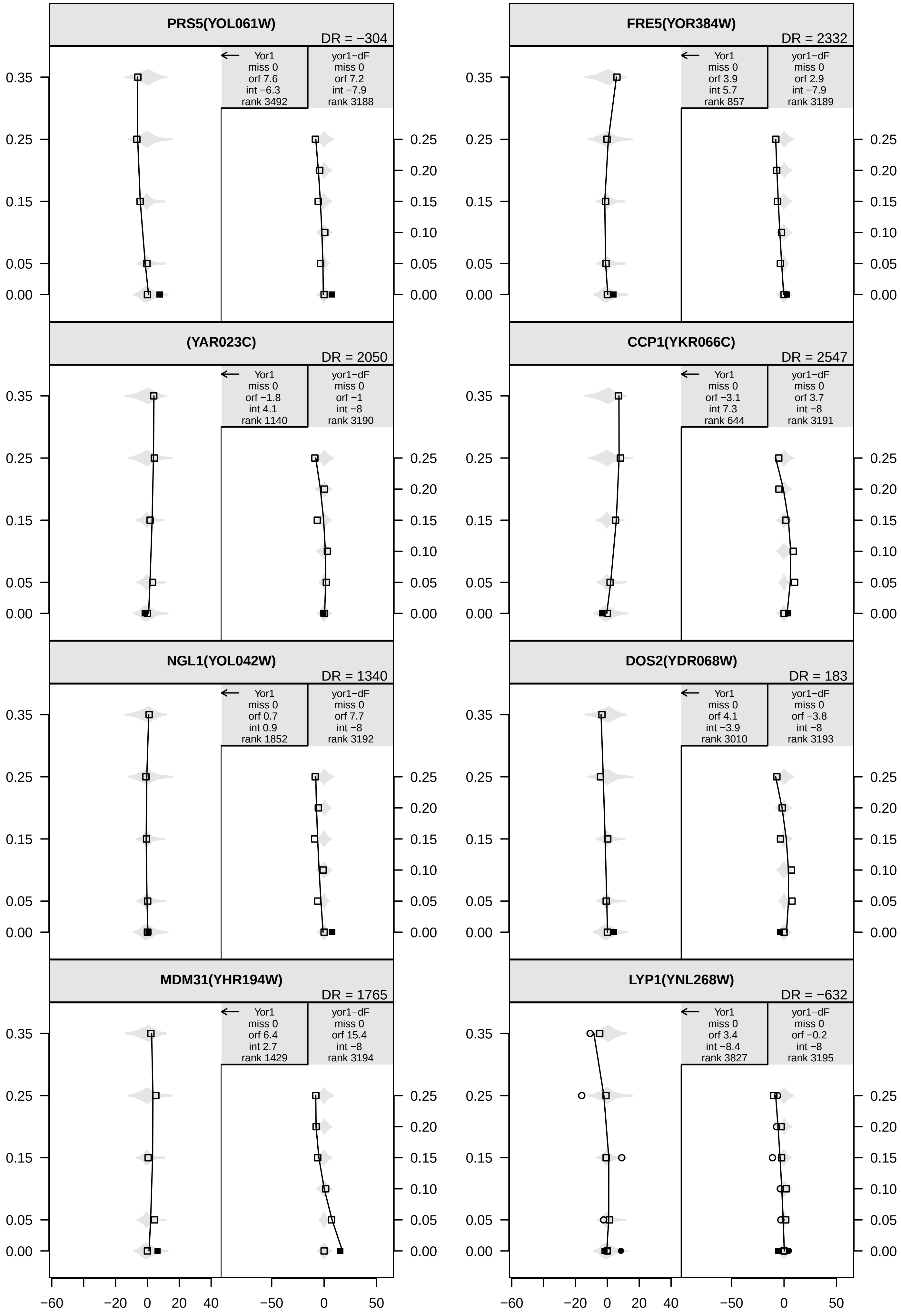

PRS5(YOL061W)
DR = −304
Yor1 miss 0 orf 7.6 int −6.3 rank 3492
yor1−dF miss 0 orf 7.2 int −7.9 rank 3188
FRE5(YOR384W)
DR = 2332
Yor1 miss 0 orf 3.9 int 5.7 rank 857
yor1−dF miss 0 orf 2.9 int −7.9 rank 3189
(YAR023C)
DR = 2050
Yor1 miss 0 orf −1.8 int 4.1 rank 1140
yor1−dF miss 0 orf −1 int −8 rank 3190
CCP1(YKR066C)
DR = 2547
Yor1 miss 0 orf −3.1 int 7.3 rank 644
yor1−dF miss 0 orf 3.7 int −8 rank 3191
NGL1(YOL042W)
DR = 1340
Yor1 miss 0 orf 0.7 int 0.9 rank 1852
yor1−dF miss 0 orf 7.7 int −8 rank 3192
DOS2(YDR068W)
DR = 183
Yor1 miss 0 orf 4.1 int −3.9 rank 3010
yor1−dF miss 0 orf −3.8 int −8 rank 3193
MDM31(YHR194W)
DR = 1765
Yor1 miss 0 orf 6.4 int 2.7 rank 1429
yor1−dF miss 0 orf 15.4 int −8 rank 3194
LYP1(YNL268W)
DR = −632
Yor1 miss 0 orf 3.4 int −8.4 rank 3827
yor1−dF miss 0 orf −0.2 int −8 rank 3195
0.35 0.25 0.20 0.15 0.10 0.05 0.00
−60 −20 0 20 40 −50 0 50

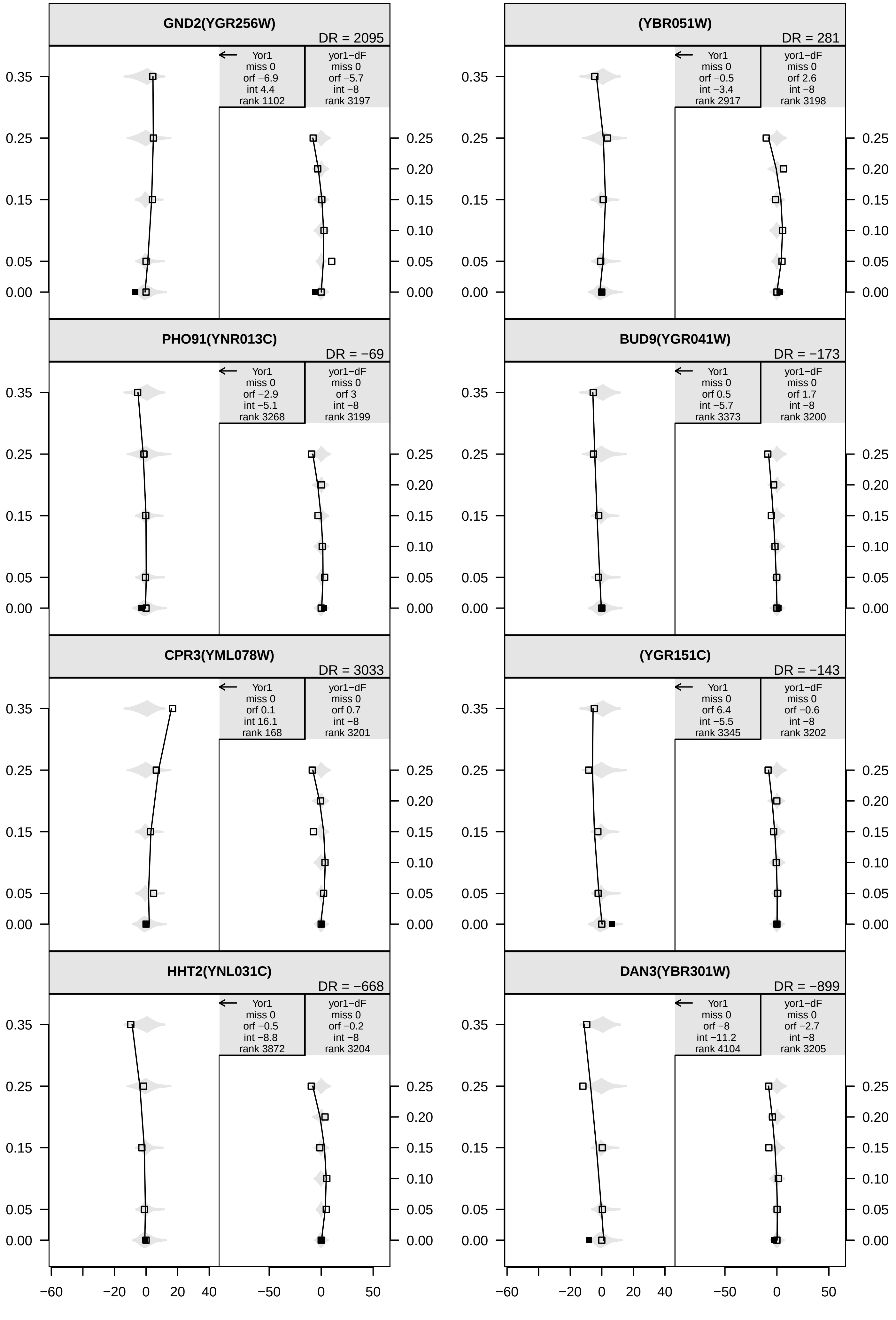
GND2(YGR256W)
DR = 2095
Yor1
miss 0
orf −6.9
int 4.4
rank 1102
yor1−dF
miss 0
orf −5.7
int −8
rank 3197
(YBR051W)
DR = 281
Yor1
miss 0
orf −0.5
int −3.4
rank 2917
yor1−dF
miss 0
orf 2.6
int −8
rank 3198
PHO91(YNR013C)
DR = −69
Yor1
miss 0
orf −2.9
int −5.1
rank 3268
yor1−dF
miss 0
orf 3
int −8
rank 3199
BUD9(YGR041W)
DR = −173
Yor1
miss 0
orf 0.5
int −5.7
rank 3373
yor1−dF
miss 0
orf 1.7
int −8
rank 3200
CPR3(YML078W)
DR = 3033
Yor1
miss 0
orf 0.1
int 16.1
rank 168
yor1−dF
miss 0
orf 0.7
int −8
rank 3201
(YGR151C)
DR = −143
Yor1
miss 0
orf 6.4
int −5.5
rank 3345
yor1−dF
miss 0
orf −0.6
int −8
rank 3202
HHT2(YNL031C)
DR = −668
Yor1
miss 0
orf −0.5
int −8.8
rank 3872
yor1−dF
miss 0
orf −0.2
int −8
rank 3204
DAN3(YBR301W)
DR = −899
Yor1
miss 0
orf −8
int −11.2
rank 4104
yor1−dF
miss 0
orf −2.7
int −8
rank 3205
0.35
0.25
0.20
0.15
0.10
0.05
0.00
−60
−20
0
20
40
−50
0
50

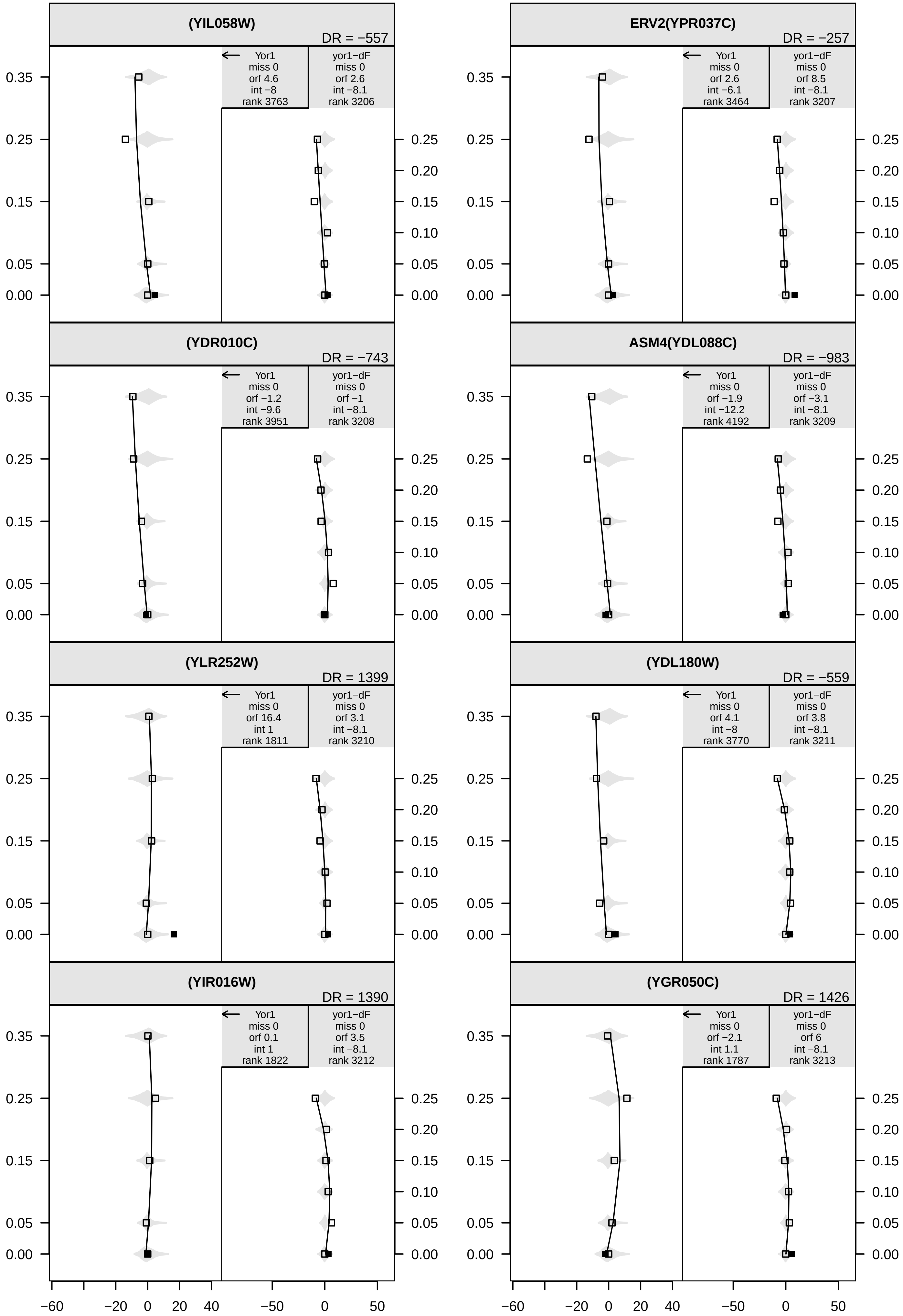
(YIL058W)
DR = −557
Yor1
miss 0
orf 4.6
int −8
rank 3763
yor1−dF
miss 0
orf 2.6
int −8.1
rank 3206
ERV2(YPR037C)
DR = −257
Yor1
miss 0
orf 2.6
int −6.1
rank 3464
yor1−dF
miss 0
orf 8.5
int −8.1
rank 3207
(YDR010C)
DR = −743
Yor1
miss 0
orf −1.2
int −9.6
rank 3951
yor1−dF
miss 0
orf −1
int −8.1
rank 3208
ASM4(YDL088C)
DR = −983
Yor1
miss 0
orf −1.9
int −12.2
rank 4192
yor1−dF
miss 0
orf −3.1
int −8.1
rank 3209
(YLR252W)
DR = 1399
Yor1
miss 0
orf 16.4
int 1
rank 1811
yor1−dF
miss 0
orf 3.1
int −8.1
rank 3210
(YDL180W)
DR = −559
Yor1
miss 0
orf 4.1
int −8
rank 3770
yor1−dF
miss 0
orf 3.8
int −8.1
rank 3211
(YIR016W)
DR = 1390
Yor1
miss 0
orf 0.1
int 1
rank 1822
yor1−dF
miss 0
orf 3.5
int −8.1
rank 3212
(YGR050C)
DR = 1426
Yor1
miss 0
orf −2.1
int 1.1
rank 1787
yor1−dF
miss 0
orf 6
int −8.1
rank 3213
0.35
0.25
0.20
0.15
0.10
0.05
0.00
−60
−20
0
20
40
−50
0
50

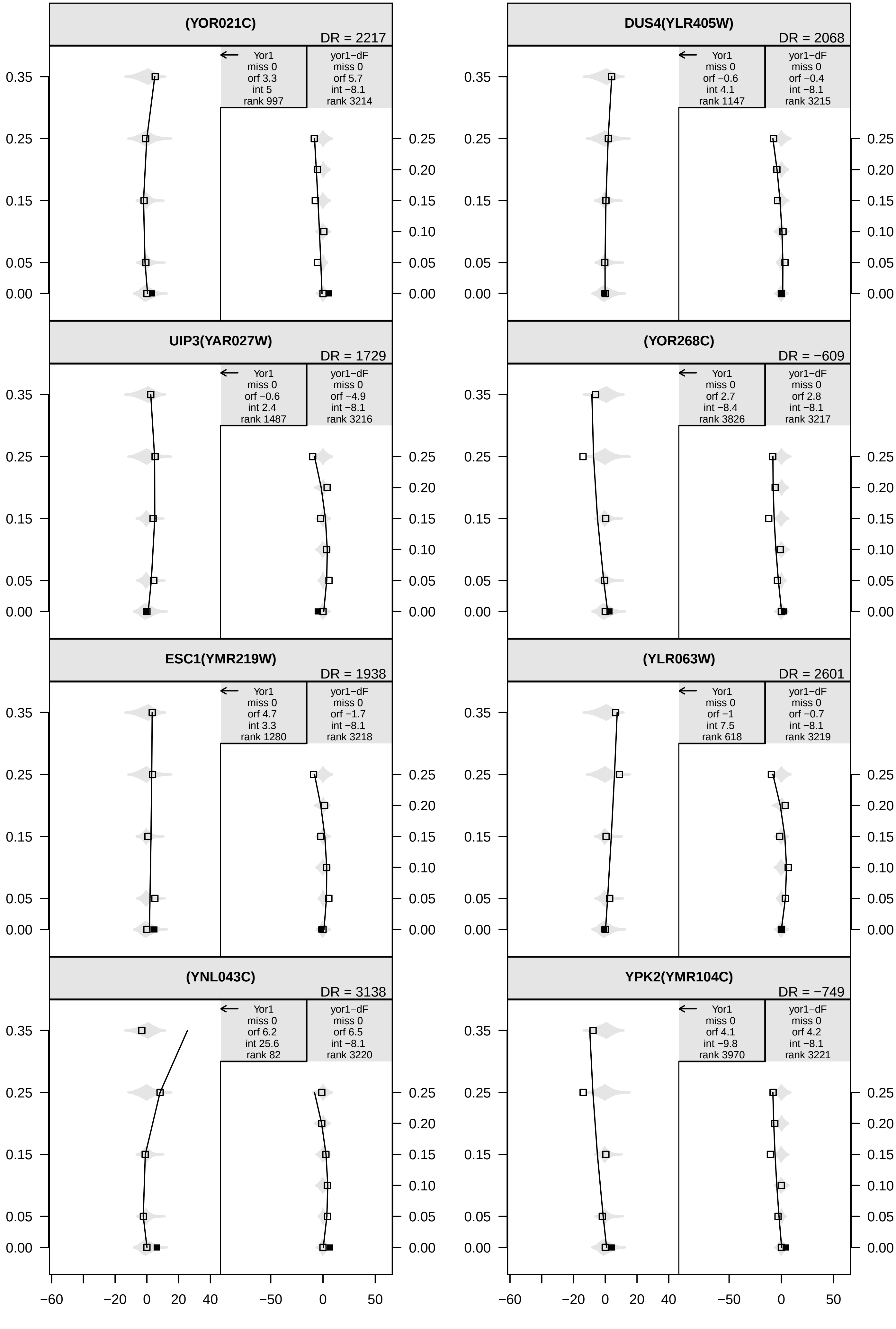

(YOR021C)
DR = 2217
Yor1 miss 0 orf 3.3 int 5 rank 997
yor1−dF miss 0 orf 5.7 int −8.1 rank 3214
DUS4(YLR405W)
DR = 2068
Yor1 miss 0 orf −0.6 int 4.1 rank 1147
yor1−dF miss 0 orf −0.4 int −8.1 rank 3215
UIP3(YAR027W)
DR = 1729
Yor1 miss 0 orf −0.6 int 2.4 rank 1487
yor1−dF miss 0 orf −4.9 int −8.1 rank 3216
(YOR268C)
DR = −609
Yor1 miss 0 orf 2.7 int −8.4 rank 3826
yor1−dF miss 0 orf 2.8 int −8.1 rank 3217
ESC1(YMR219W)
DR = 1938
Yor1 miss 0 orf 4.7 int 3.3 rank 1280
yor1−dF miss 0 orf −1.7 int −8.1 rank 3218
(YLR063W)
DR = 2601
Yor1 miss 0 orf −1 int 7.5 rank 618
yor1−dF miss 0 orf −0.7 int −8.1 rank 3219
(YNL043C)
DR = 3138
Yor1 miss 0 orf 6.2 int 25.6 rank 82
yor1−dF miss 0 orf 6.5 int −8.1 rank 3220
YPK2(YMR104C)
DR = −749
Yor1 miss 0 orf 4.1 int −9.8 rank 3970
yor1−dF miss 0 orf 4.2 int −8.1 rank 3221
0.35 0.25 0.15 0.05 0.00
0.25 0.20 0.15 0.10 0.05 0.00
−60 −20 0 20 40
−50 0 50

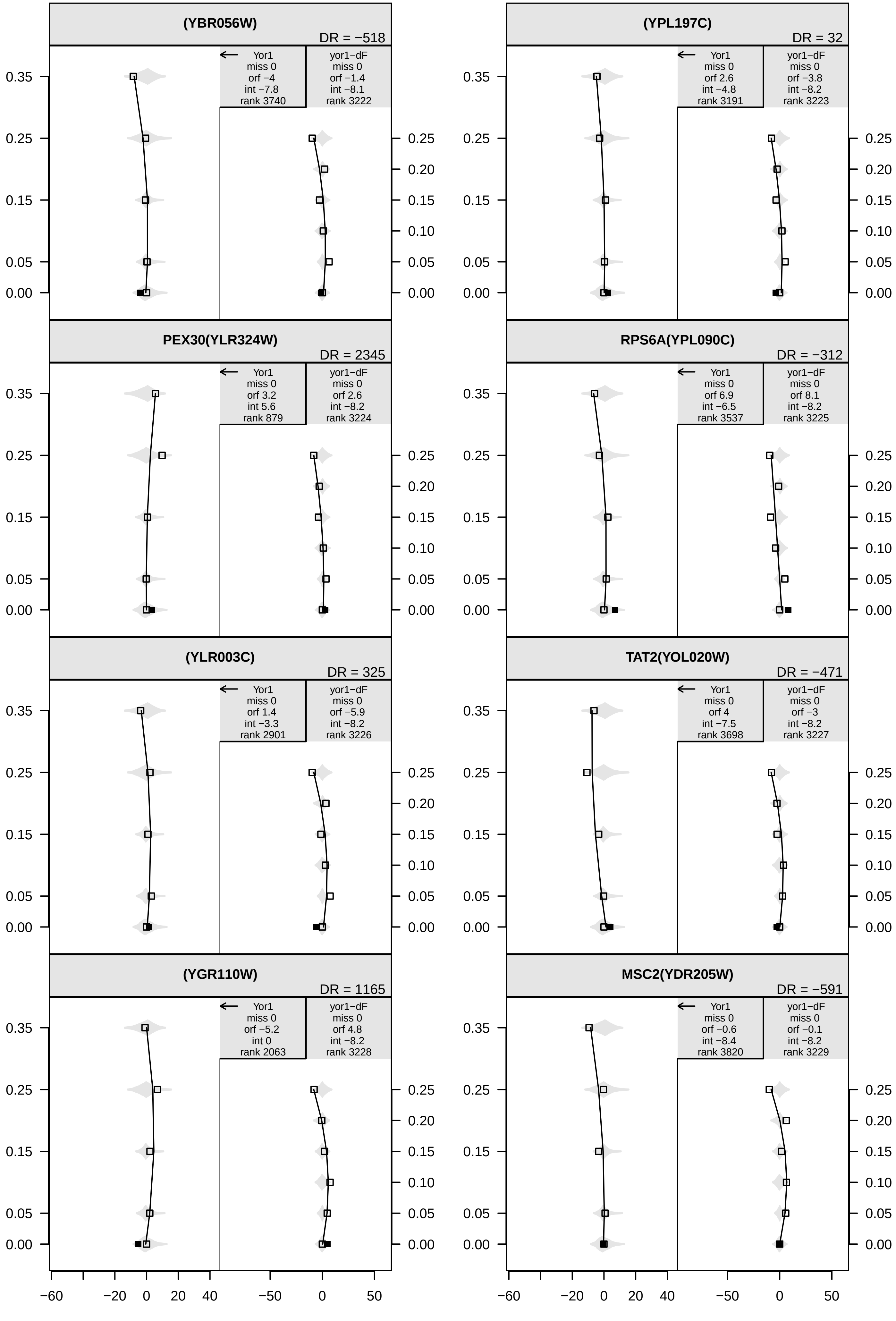
(YBR056W)
DR = −518
Yor1
miss 0
orf −4
int −7.8
rank 3740
yor1−dF
miss 0
orf −1.4
int −8.1
rank 3222
(YPL197C)
DR = 32
Yor1
miss 0
orf 2.6
int −4.8
rank 3191
yor1−dF
miss 0
orf −3.8
int −8.2
rank 3223
PEX30(YLR324W)
DR = 2345
Yor1
miss 0
orf 3.2
int 5.6
rank 879
yor1−dF
miss 0
orf 2.6
int −8.2
rank 3224
RPS6A(YPL090C)
DR = −312
Yor1
miss 0
orf 6.9
int −6.5
rank 3537
yor1−dF
miss 0
orf 8.1
int −8.2
rank 3225
(YLR003C)
DR = 325
Yor1
miss 0
orf 1.4
int −3.3
rank 2901
yor1−dF
miss 0
orf −5.9
int −8.2
rank 3226
TAT2(YOL020W)
DR = −471
Yor1
miss 0
orf 4
int −7.5
rank 3698
yor1−dF
miss 0
orf −3
int −8.2
rank 3227
(YGR110W)
DR = 1165
Yor1
miss 0
orf −5.2
int 0
rank 2063
yor1−dF
miss 0
orf 4.8
int −8.2
rank 3228
MSC2(YDR205W)
DR = −591
Yor1
miss 0
orf −0.6
int −8.4
rank 3820
yor1−dF
miss 0
orf −0.1
int −8.2
rank 3229
0.35
0.25
0.20
0.15
0.10
0.05
0.00
−60
−20
0
20
40
−50
0
50

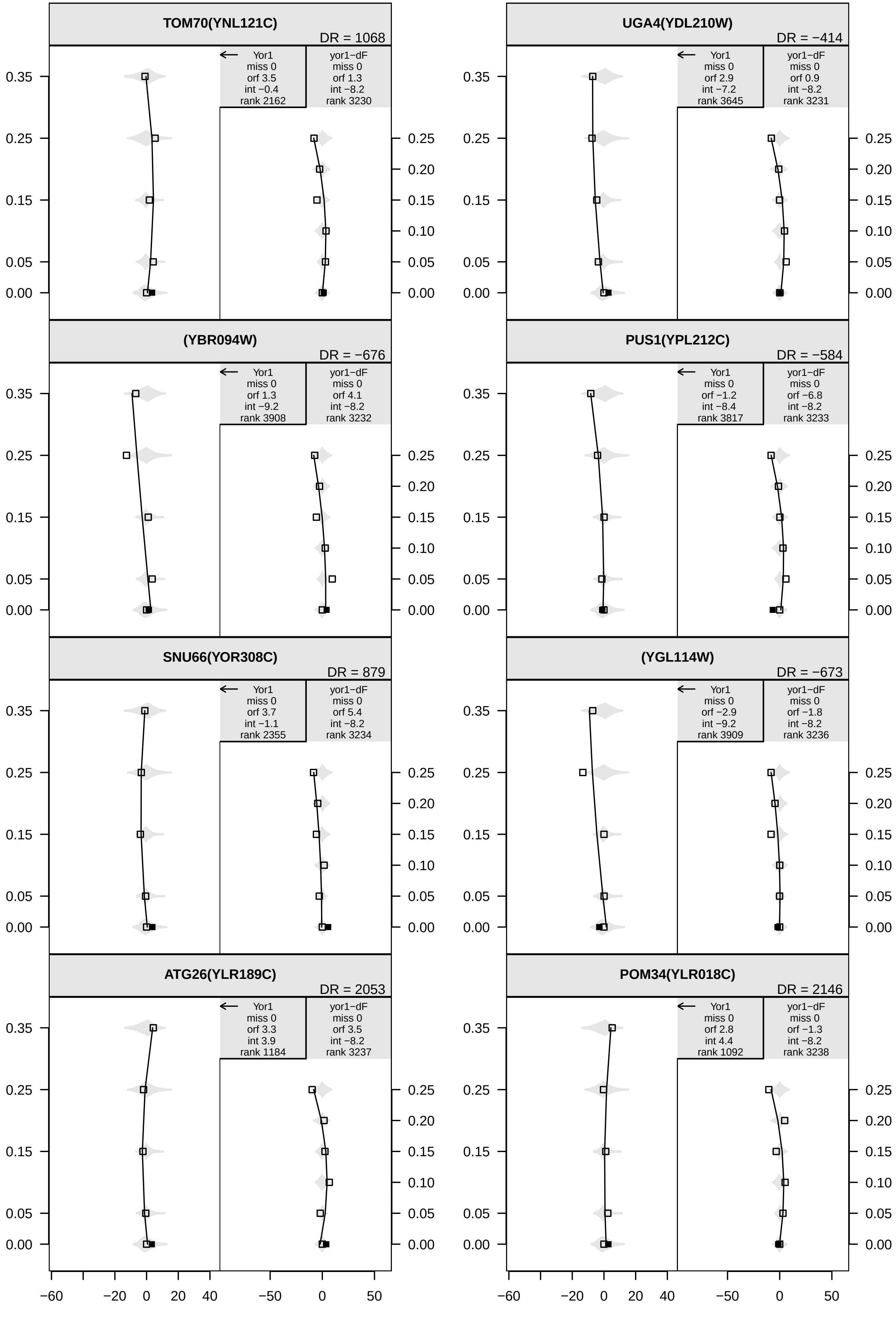
TOM70(YNL121C)
DR = 1068
Yor1
miss 0
orf 3.5
int −0.4
rank 2162
yor1−dF
miss 0
orf 1.3
int −8.2
rank 3230
UGA4(YDL210W)
DR = −414
Yor1
miss 0
orf 2.9
int −7.2
rank 3645
yor1−dF
miss 0
orf 0.9
int −8.2
rank 3231
(YBR094W)
DR = −676
Yor1
miss 0
orf 1.3
int −9.2
rank 3908
yor1−dF
miss 0
orf 4.1
int −8.2
rank 3232
PUS1(YPL212C)
DR = −584
Yor1
miss 0
orf −1.2
int −8.4
rank 3817
yor1−dF
miss 0
orf −6.8
int −8.2
rank 3233
SNU66(YOR308C)
DR = 879
Yor1
miss 0
orf 3.7
int −1.1
rank 2355
yor1−dF
miss 0
orf 5.4
int −8.2
rank 3234
(YGL114W)
DR = −673
Yor1
miss 0
orf −2.9
int −9.2
rank 3909
yor1−dF
miss 0
orf −1.8
int −8.2
rank 3236
ATG26(YLR189C)
DR = 2053
Yor1
miss 0
orf 3.3
int 3.9
rank 1184
yor1−dF
miss 0
orf 3.5
int −8.2
rank 3237
POM34(YLR018C)
DR = 2146
Yor1
miss 0
orf 2.8
int 4.4
rank 1092
yor1−dF
miss 0
orf −1.3
int −8.2
rank 3238
0.35
0.25
0.20
0.15
0.10
0.05
0.00
−60
−20
0
20
40
−50
0
50

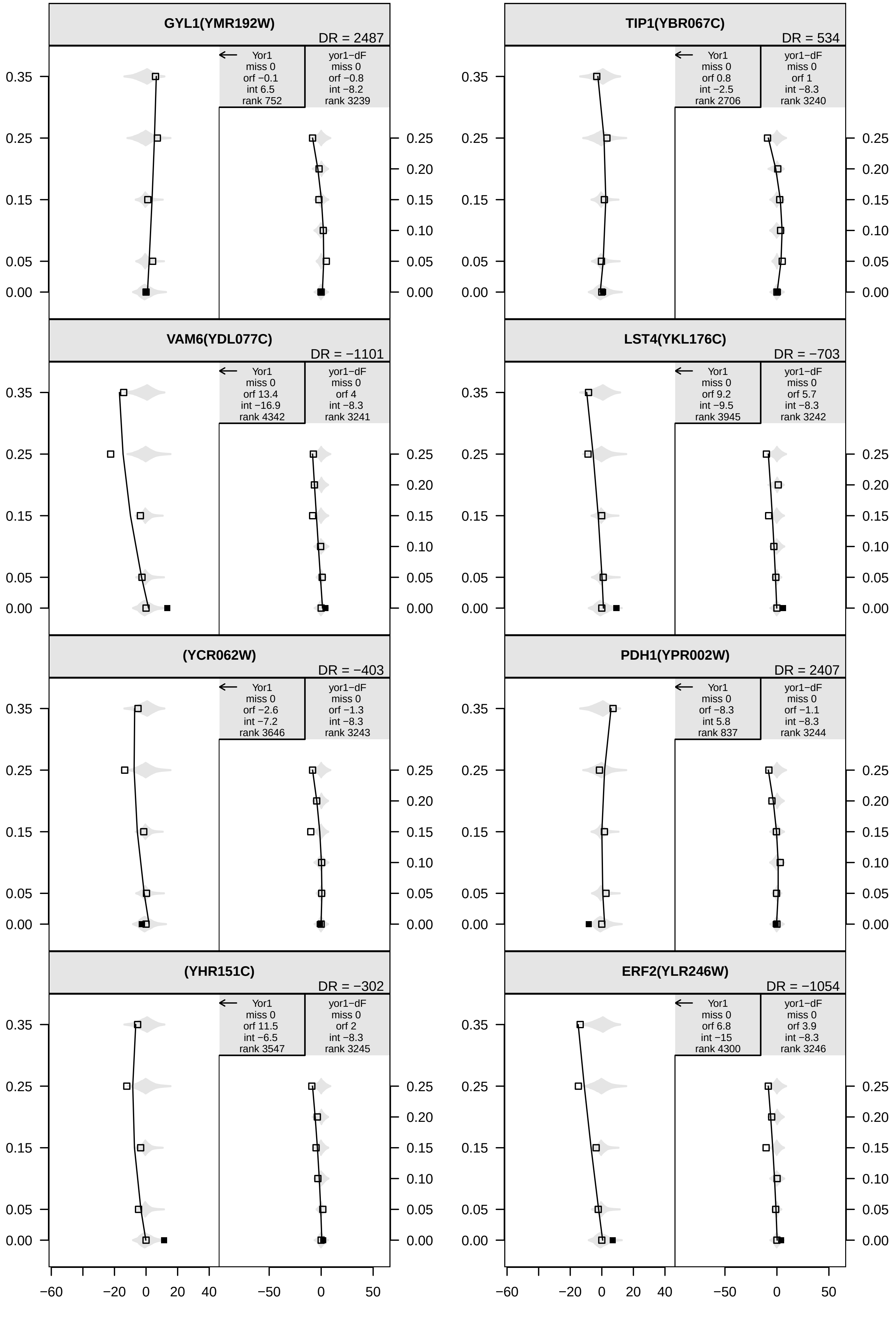

GYL1(YMR192W)
DR = 2487
Yor1 miss 0 orf −0.1 int 6.5 rank 752
yor1−dF miss 0 orf −0.8 int −8.2 rank 3239
TIP1(YBR067C)
DR = 534
Yor1 miss 0 orf 0.8 int −2.5 rank 2706
yor1−dF miss 0 orf 1 int −8.3 rank 3240
VAM6(YDL077C)
DR = −1101
Yor1 miss 0 orf 13.4 int −16.9 rank 4342
yor1−dF miss 0 orf 4 int −8.3 rank 3241
LST4(YKL176C)
DR = −703
Yor1 miss 0 orf 9.2 int −9.5 rank 3945
yor1−dF miss 0 orf 5.7 int −8.3 rank 3242
(YCR062W)
DR = −403
Yor1 miss 0 orf −2.6 int −7.2 rank 3646
yor1−dF miss 0 orf −1.3 int −8.3 rank 3243
PDH1(YPR002W)
DR = 2407
Yor1 miss 0 orf −8.3 int 5.8 rank 837
yor1−dF miss 0 orf −1.1 int −8.3 rank 3244
(YHR151C)
DR = −302
Yor1 miss 0 orf 11.5 int −6.5 rank 3547
yor1−dF miss 0 orf 2 int −8.3 rank 3245
ERF2(YLR246W)
DR = −1054
Yor1 miss 0 orf 6.8 int −15 rank 4300
yor1−dF miss 0 orf 3.9 int −8.3 rank 3246
0.00 0.05 0.10 0.15 0.20 0.25 0.35
−60 −20 0 20 40 −50 0 50

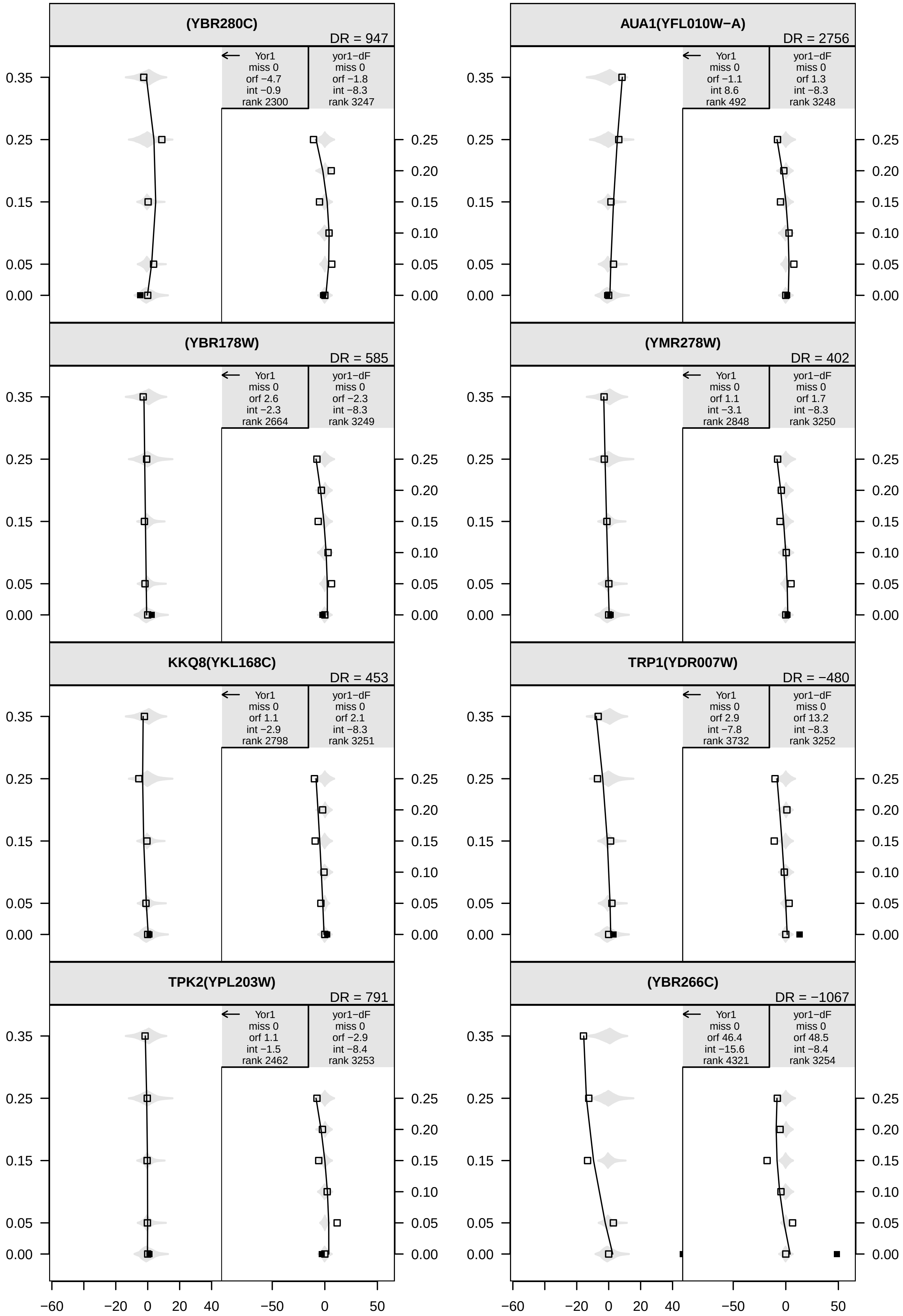
(YBR280C)
DR = 947
Yor1
miss 0
orf −4.7
int −0.9
rank 2300
yor1−dF
miss 0
orf −1.8
int −8.3
rank 3247
AUA1(YFL010W−A)
DR = 2756
Yor1
miss 0
orf −1.1
int 8.6
rank 492
yor1−dF
miss 0
orf 1.3
int −8.3
rank 3248
(YBR178W)
DR = 585
Yor1
miss 0
orf 2.6
int −2.3
rank 2664
yor1−dF
miss 0
orf −2.3
int −8.3
rank 3249
(YMR278W)
DR = 402
Yor1
miss 0
orf 1.1
int −3.1
rank 2848
yor1−dF
miss 0
orf 1.7
int −8.3
rank 3250
KKQ8(YKL168C)
DR = 453
Yor1
miss 0
orf 1.1
int −2.9
rank 2798
yor1−dF
miss 0
orf 2.1
int −8.3
rank 3251
TRP1(YDR007W)
DR = −480
Yor1
miss 0
orf 2.9
int −7.8
rank 3732
yor1−dF
miss 0
orf 13.2
int −8.3
rank 3252
TPK2(YPL203W)
DR = 791
Yor1
miss 0
orf 1.1
int −1.5
rank 2462
yor1−dF
miss 0
orf −2.9
int −8.4
rank 3253
(YBR266C)
DR = −1067
Yor1
miss 0
orf 46.4
int −15.6
rank 4321
yor1−dF
miss 0
orf 48.5
int −8.4
rank 3254
0.35
0.25
0.20
0.15
0.10
0.05
0.00
−60
−20
0
20
40
−50
0
50

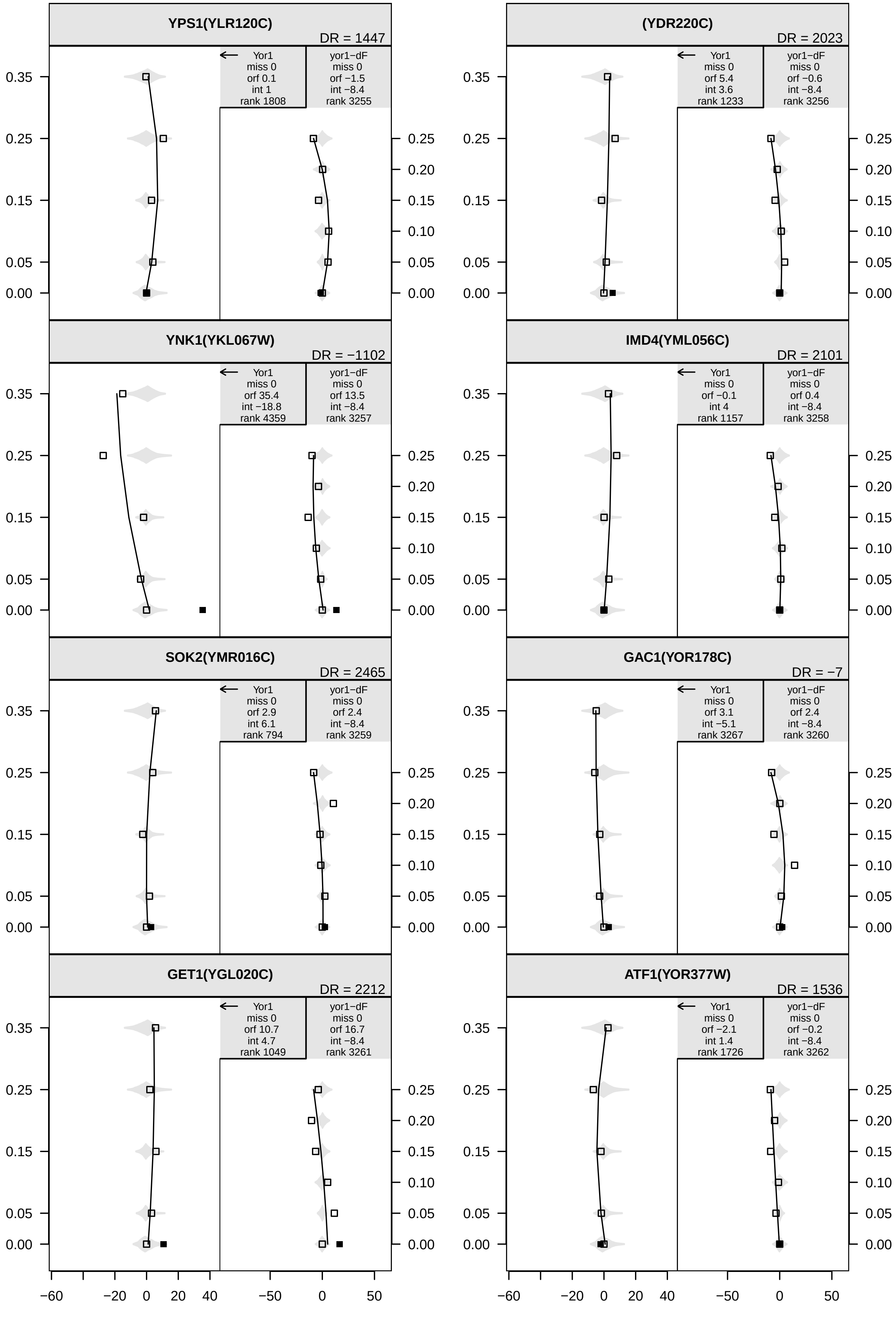

YPS1(YLR120C)
DR = 1447
Yor1 miss 0 orf 0.1 int 1 rank 1808
yor1−dF miss 0 orf −1.5 int −8.4 rank 3255
(YDR220C)
DR = 2023
Yor1 miss 0 orf 5.4 int 3.6 rank 1233
yor1−dF miss 0 orf −0.6 int −8.4 rank 3256
YNK1(YKL067W)
DR = −1102
Yor1 miss 0 orf 35.4 int −18.8 rank 4359
yor1−dF miss 0 orf 13.5 int −8.4 rank 3257
IMD4(YML056C)
DR = 2101
Yor1 miss 0 orf −0.1 int 4 rank 1157
yor1−dF miss 0 orf 0.4 int −8.4 rank 3258
SOK2(YMR016C)
DR = 2465
Yor1 miss 0 orf 2.9 int 6.1 rank 794
yor1−dF miss 0 orf 2.4 int −8.4 rank 3259
GAC1(YOR178C)
DR = −7
Yor1 miss 0 orf 3.1 int −5.1 rank 3267
yor1−dF miss 0 orf 2.4 int −8.4 rank 3260
GET1(YGL020C)
DR = 2212
Yor1 miss 0 orf 10.7 int 4.7 rank 1049
yor1−dF miss 0 orf 16.7 int −8.4 rank 3261
ATF1(YOR377W)
DR = 1536
Yor1 miss 0 orf −2.1 int 1.4 rank 1726
yor1−dF miss 0 orf −0.2 int −8.4 rank 3262

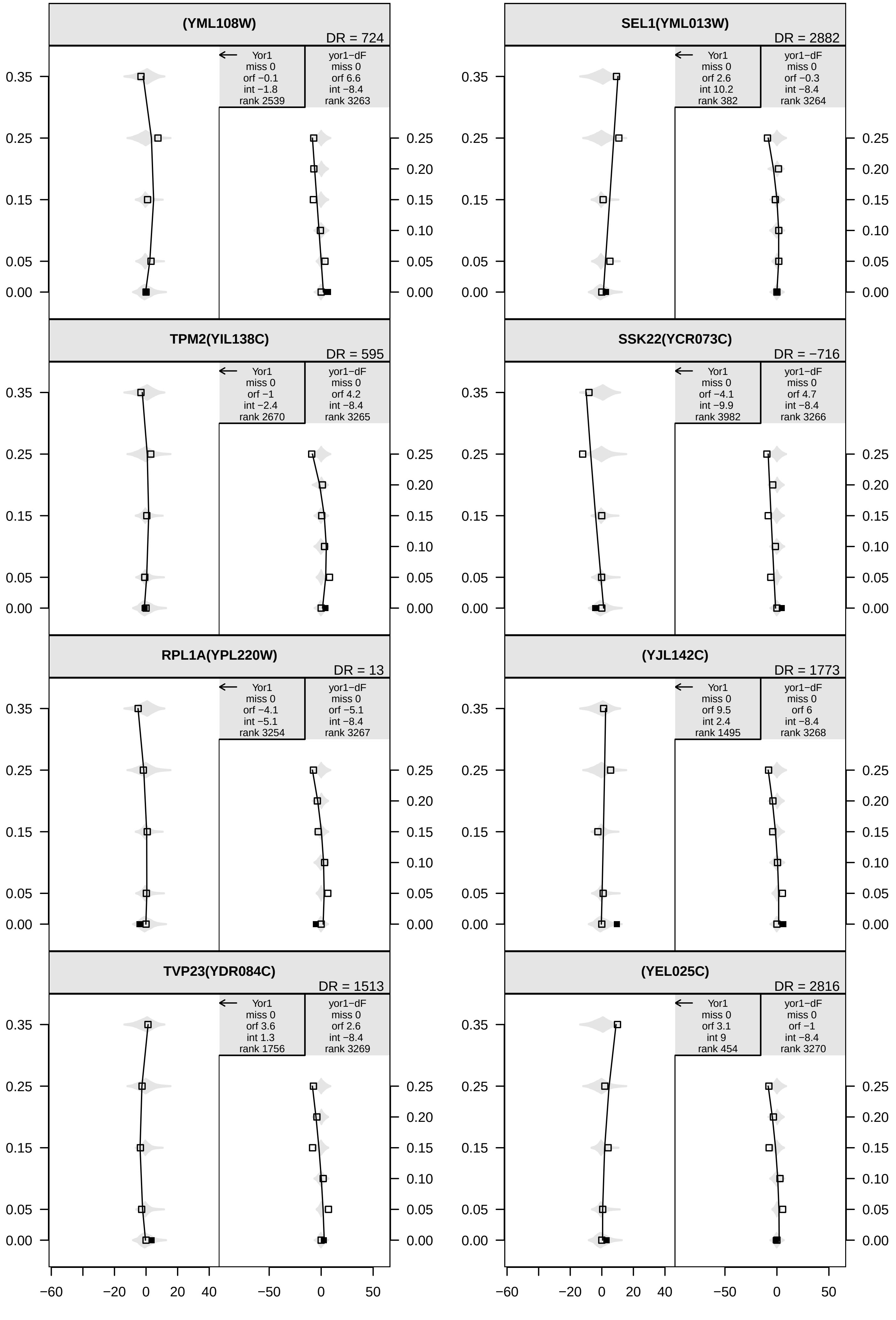

(YML108W)
DR = 724
Yor1
miss 0
orf −0.1
int −1.8
rank 2539
yor1−dF
miss 0
orf 6.6
int −8.4
rank 3263
SEL1(YML013W)
DR = 2882
Yor1
miss 0
orf 2.6
int 10.2
rank 382
yor1−dF
miss 0
orf −0.3
int −8.4
rank 3264
TPM2(YIL138C)
DR = 595
Yor1
miss 0
orf −1
int −2.4
rank 2670
yor1−dF
miss 0
orf 4.2
int −8.4
rank 3265
SSK22(YCR073C)
DR = −716
Yor1
miss 0
orf −4.1
int −9.9
rank 3982
yor1−dF
miss 0
orf 4.7
int −8.4
rank 3266
RPL1A(YPL220W)
DR = 13
Yor1
miss 0
orf −4.1
int −5.1
rank 3254
yor1−dF
miss 0
orf −5.1
int −8.4
rank 3267
(YJL142C)
DR = 1773
Yor1
miss 0
orf 9.5
int 2.4
rank 1495
yor1−dF
miss 0
orf 6
int −8.4
rank 3268
TVP23(YDR084C)
DR = 1513
Yor1
miss 0
orf 3.6
int 1.3
rank 1756
yor1−dF
miss 0
orf 2.6
int −8.4
rank 3269
(YEL025C)
DR = 2816
Yor1
miss 0
orf 3.1
int 9
rank 454
yor1−dF
miss 0
orf −1
int −8.4
rank 3270

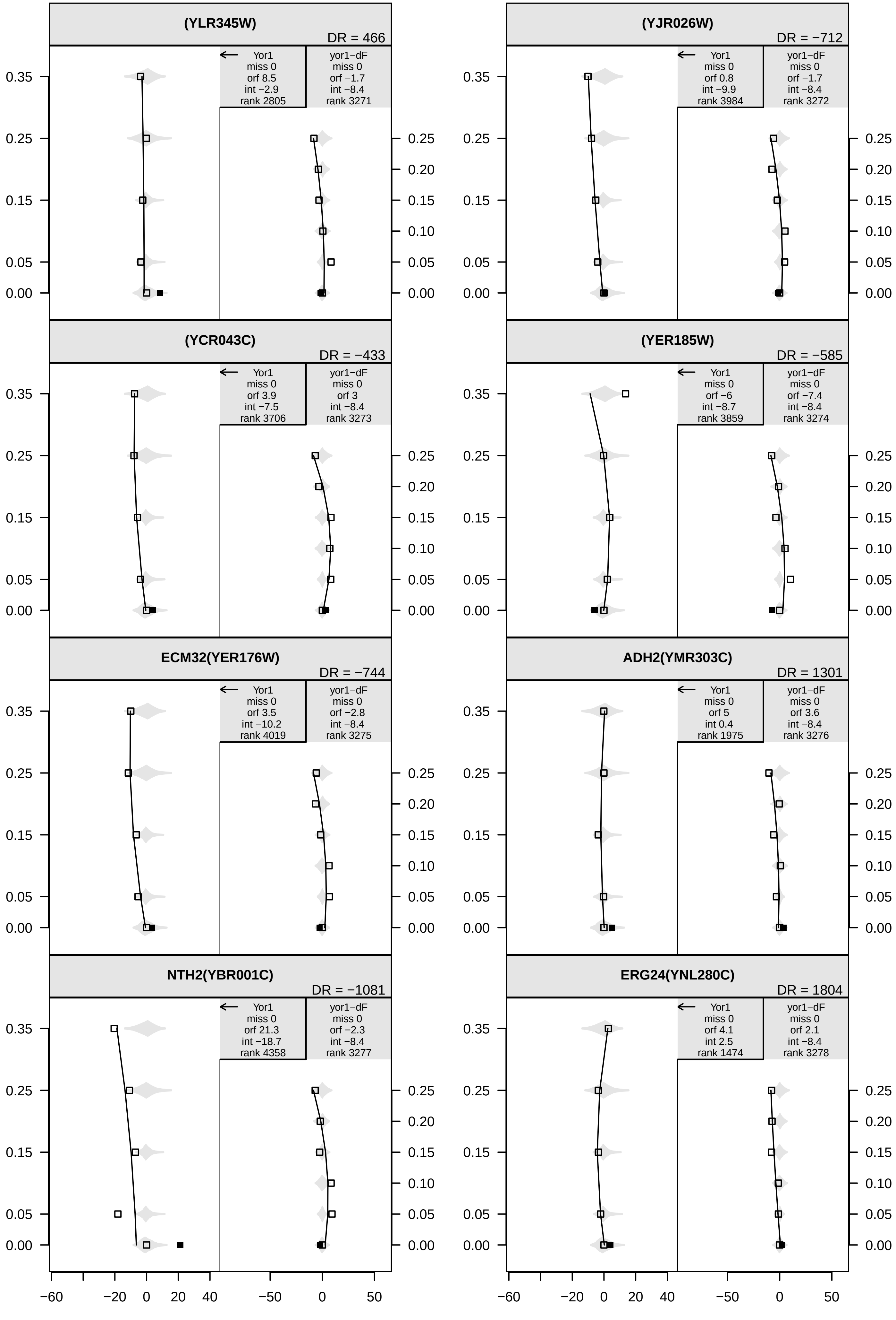
(YLR345W)
DR = 466
Yor1 miss 0 orf 8.5 int −2.9 rank 2805
yor1−dF miss 0 orf −1.7 int −8.4 rank 3271
(YJR026W)
DR = −712
Yor1 miss 0 orf 0.8 int −9.9 rank 3984
yor1−dF miss 0 orf −1.7 int −8.4 rank 3272
(YCR043C)
DR = −433
Yor1 miss 0 orf 3.9 int −7.5 rank 3706
yor1−dF miss 0 orf 3 int −8.4 rank 3273
(YER185W)
DR = −585
Yor1 miss 0 orf −6 int −8.7 rank 3859
yor1−dF miss 0 orf −7.4 int −8.4 rank 3274
ECM32(YER176W)
DR = −744
Yor1 miss 0 orf 3.5 int −10.2 rank 4019
yor1−dF miss 0 orf −2.8 int −8.4 rank 3275
ADH2(YMR303C)
DR = 1301
Yor1 miss 0 orf 5 int 0.4 rank 1975
yor1−dF miss 0 orf 3.6 int −8.4 rank 3276
NTH2(YBR001C)
DR = −1081
Yor1 miss 0 orf 21.3 int −18.7 rank 4358
yor1−dF miss 0 orf −2.3 int −8.4 rank 3277
ERG24(YNL280C)
DR = 1804
Yor1 miss 0 orf 4.1 int 2.5 rank 1474
yor1−dF miss 0 orf 2.1 int −8.4 rank 3278
0.35 0.25 0.15 0.05 0.00
0.25 0.20 0.15 0.10 0.05 0.00
−60 −20 0 20 40 −50 0 50

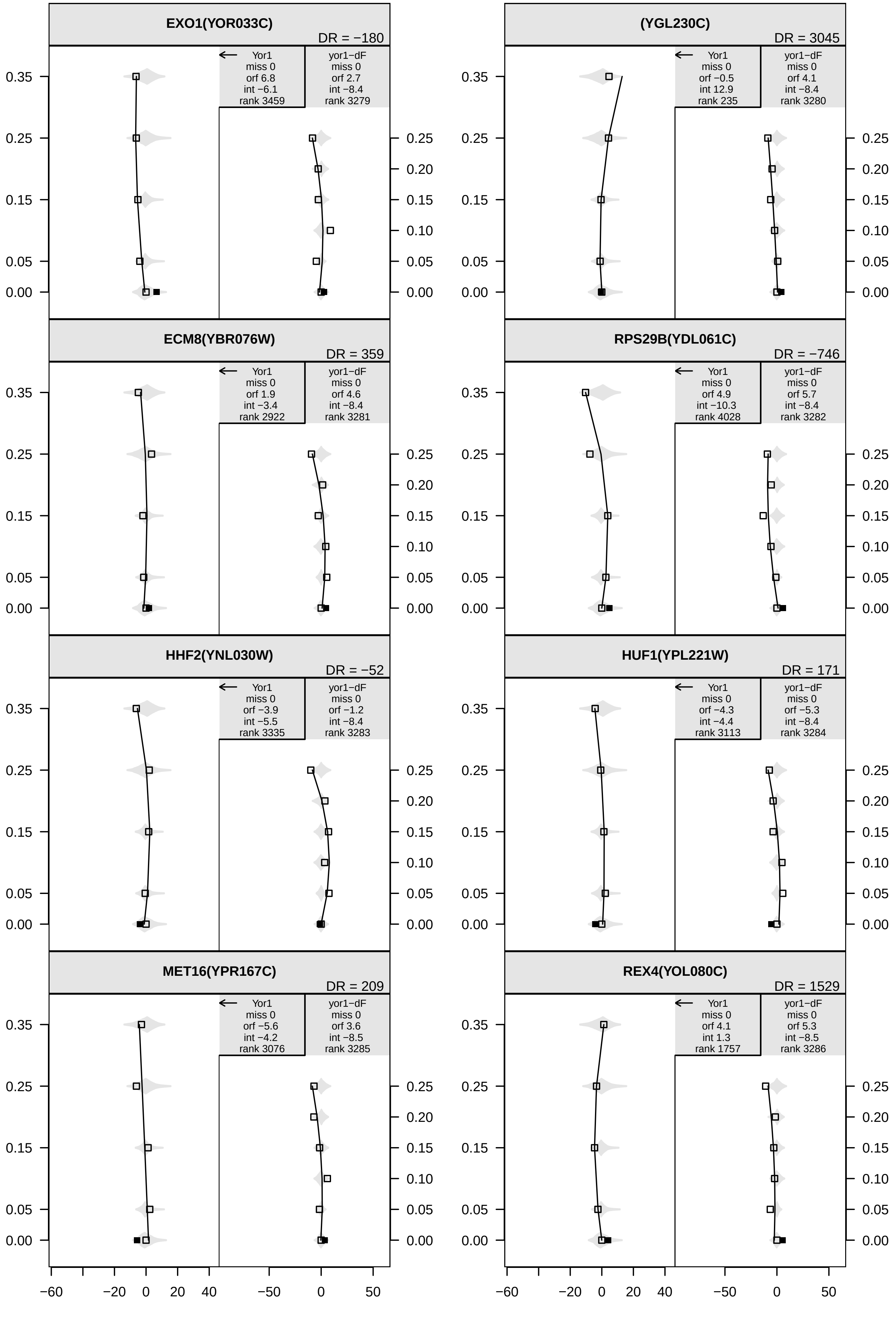
EXO1(YOR033C)
DR = −180
Yor1
miss 0
orf 6.8
int −6.1
rank 3459
yor1−dF
miss 0
orf 2.7
int −8.4
rank 3279
(YGL230C)
DR = 3045
Yor1
miss 0
orf −0.5
int 12.9
rank 235
yor1−dF
miss 0
orf 4.1
int −8.4
rank 3280
ECM8(YBR076W)
DR = 359
Yor1
miss 0
orf 1.9
int −3.4
rank 2922
yor1−dF
miss 0
orf 4.6
int −8.4
rank 3281
RPS29B(YDL061C)
DR = −746
Yor1
miss 0
orf 4.9
int −10.3
rank 4028
yor1−dF
miss 0
orf 5.7
int −8.4
rank 3282
HHF2(YNL030W)
DR = −52
Yor1
miss 0
orf −3.9
int −5.5
rank 3335
yor1−dF
miss 0
orf −1.2
int −8.4
rank 3283
HUF1(YPL221W)
DR = 171
Yor1
miss 0
orf −4.3
int −4.4
rank 3113
yor1−dF
miss 0
orf −5.3
int −8.4
rank 3284
MET16(YPR167C)
DR = 209
Yor1
miss 0
orf −5.6
int −4.2
rank 3076
yor1−dF
miss 0
orf 3.6
int −8.5
rank 3285
REX4(YOL080C)
DR = 1529
Yor1
miss 0
orf 4.1
int 1.3
rank 1757
yor1−dF
miss 0
orf 5.3
int −8.5
rank 3286
0.35
0.25
0.20
0.15
0.10
0.05
0.00
−60
−20
0
20
40
−50
0
50

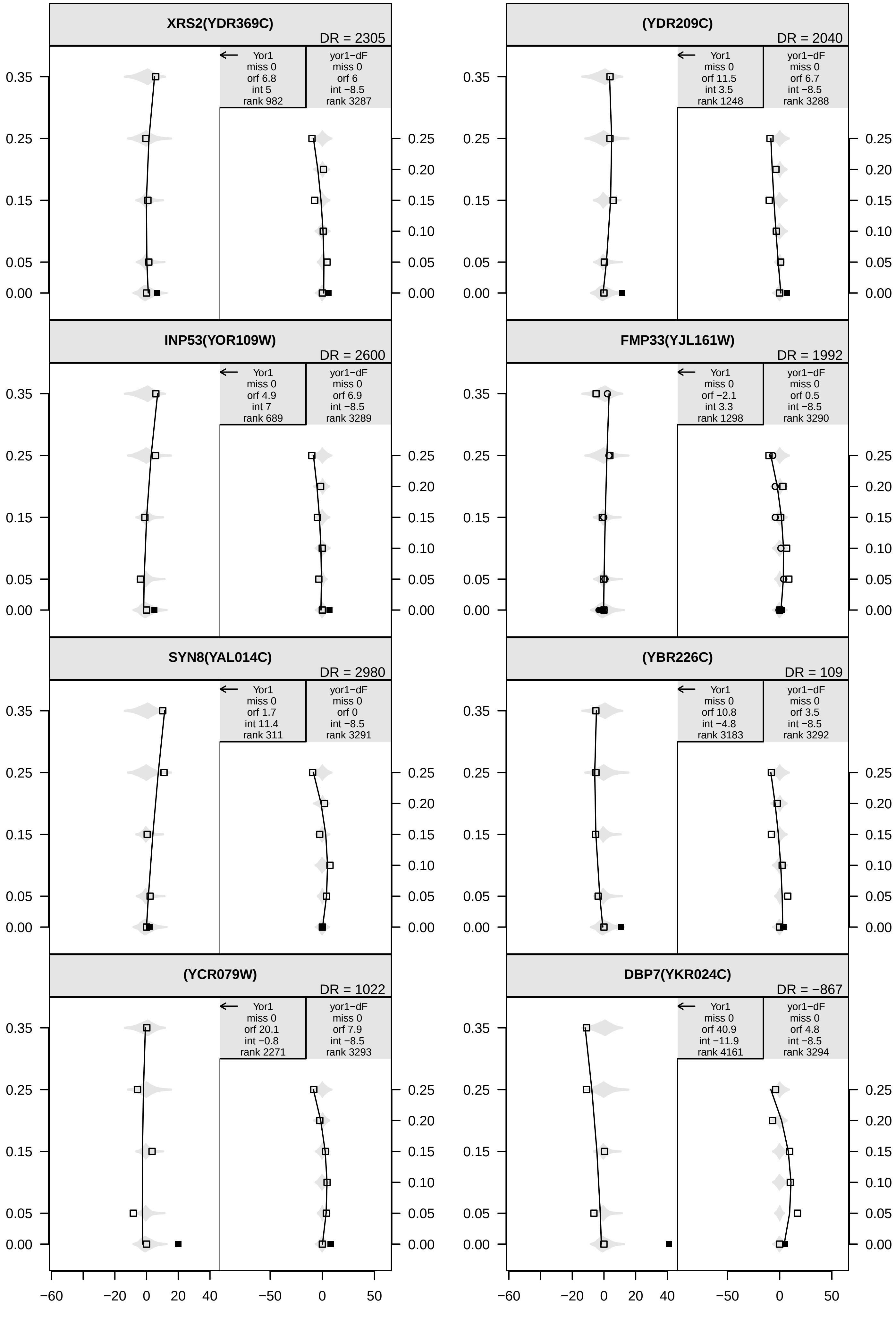

XRS2(YDR369C)
DR = 2305
Yor1 miss 0 orf 6.8 int 5 rank 982
yor1−dF miss 0 orf 6 int −8.5 rank 3287
(YDR209C)
DR = 2040
Yor1 miss 0 orf 11.5 int 3.5 rank 1248
yor1−dF miss 0 orf 6.7 int −8.5 rank 3288
INP53(YOR109W)
DR = 2600
Yor1 miss 0 orf 4.9 int 7 rank 689
yor1−dF miss 0 orf 6.9 int −8.5 rank 3289
FMP33(YJL161W)
DR = 1992
Yor1 miss 0 orf −2.1 int 3.3 rank 1298
yor1−dF miss 0 orf 0.5 int −8.5 rank 3290
SYN8(YAL014C)
DR = 2980
Yor1 miss 0 orf 1.7 int 11.4 rank 311
yor1−dF miss 0 orf 0 int −8.5 rank 3291
(YBR226C)
DR = 109
Yor1 miss 0 orf 10.8 int −4.8 rank 3183
yor1−dF miss 0 orf 3.5 int −8.5 rank 3292
(YCR079W)
DR = 1022
Yor1 miss 0 orf 20.1 int −0.8 rank 2271
yor1−dF miss 0 orf 7.9 int −8.5 rank 3293
DBP7(YKR024C)
DR = −867
Yor1 miss 0 orf 40.9 int −11.9 rank 4161
yor1−dF miss 0 orf 4.8 int −8.5 rank 3294
0.35 0.25 0.15 0.05 0.00
0.25 0.20 0.15 0.10 0.05 0.00
−60 −20 0 20 40 −50 0 50

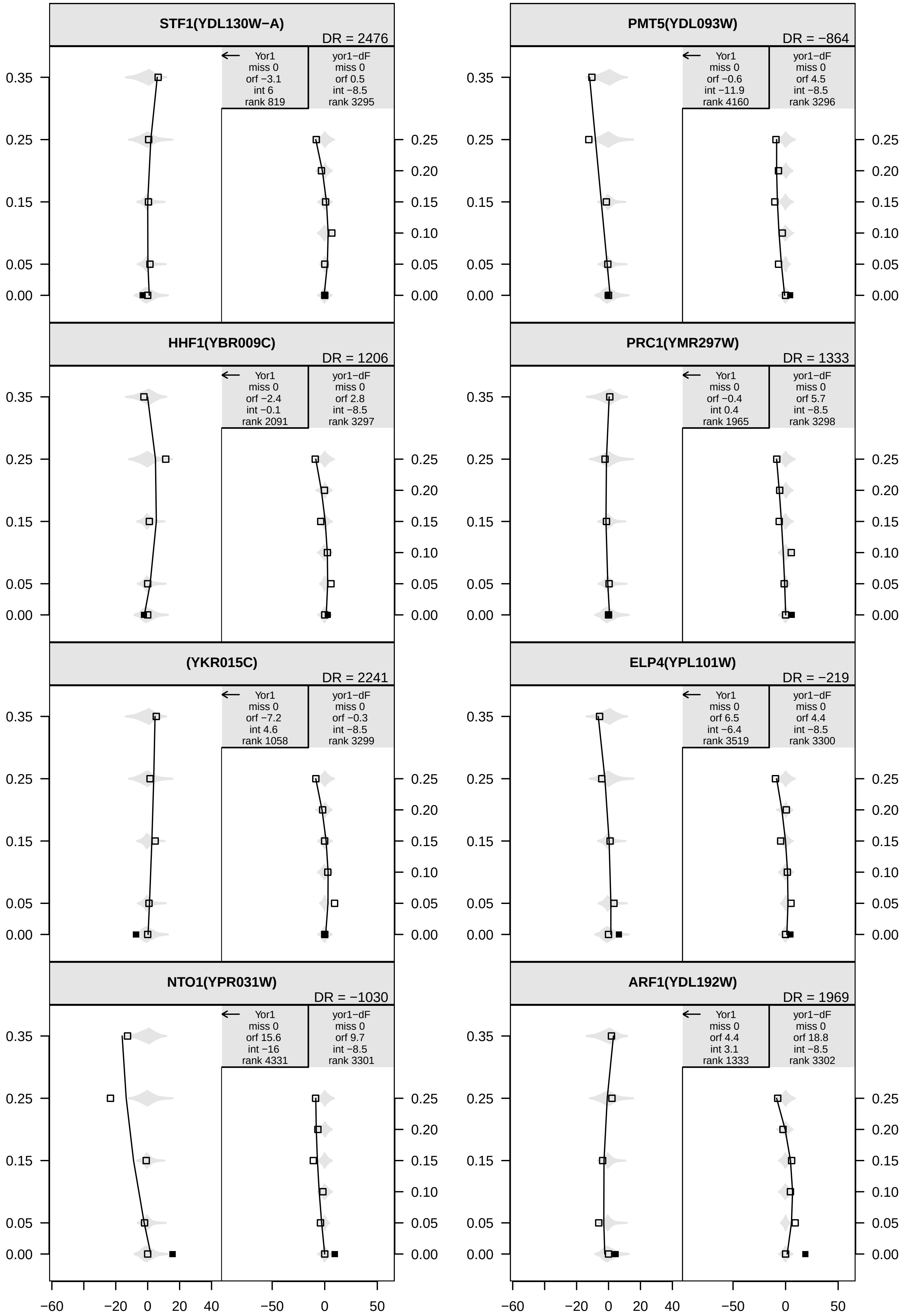
STF1(YDL130W−A)
DR = 2476
Yor1 miss 0 orf −3.1 int 6 rank 819
yor1−dF miss 0 orf 0.5 int −8.5 rank 3295
PMT5(YDL093W)
DR = −864
Yor1 miss 0 orf −0.6 int −11.9 rank 4160
yor1−dF miss 0 orf 4.5 int −8.5 rank 3296
HHF1(YBR009C)
DR = 1206
Yor1 miss 0 orf −2.4 int −0.1 rank 2091
yor1−dF miss 0 orf 2.8 int −8.5 rank 3297
PRC1(YMR297W)
DR = 1333
Yor1 miss 0 orf −0.4 int 0.4 rank 1965
yor1−dF miss 0 orf 5.7 int −8.5 rank 3298
(YKR015C)
DR = 2241
Yor1 miss 0 orf −7.2 int 4.6 rank 1058
yor1−dF miss 0 orf −0.3 int −8.5 rank 3299
ELP4(YPL101W)
DR = −219
Yor1 miss 0 orf 6.5 int −6.4 rank 3519
yor1−dF miss 0 orf 4.4 int −8.5 rank 3300
NTO1(YPR031W)
DR = −1030
Yor1 miss 0 orf 15.6 int −16 rank 4331
yor1−dF miss 0 orf 9.7 int −8.5 rank 3301
ARF1(YDL192W)
DR = 1969
Yor1 miss 0 orf 4.4 int 3.1 rank 1333
yor1−dF miss 0 orf 18.8 int −8.5 rank 3302

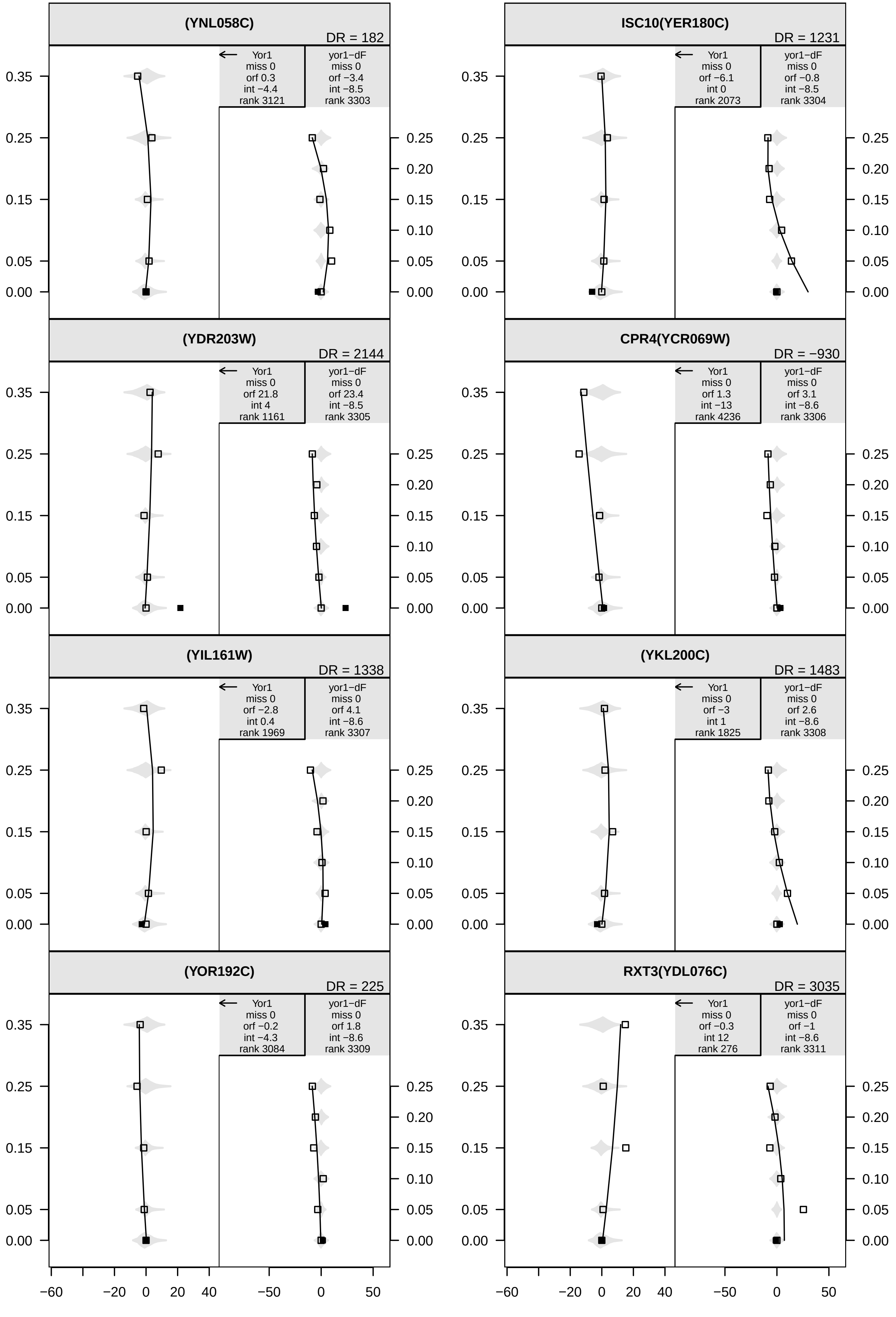

(YNL058C)
DR = 182
Yor1
miss 0
orf 0.3
int −4.4
rank 3121
yor1−dF
miss 0
orf −3.4
int −8.5
rank 3303
ISC10(YER180C)
DR = 1231
Yor1
miss 0
orf −6.1
int 0
rank 2073
yor1−dF
miss 0
orf −0.8
int −8.5
rank 3304
(YDR203W)
DR = 2144
Yor1
miss 0
orf 21.8
int 4
rank 1161
yor1−dF
miss 0
orf 23.4
int −8.5
rank 3305
CPR4(YCR069W)
DR = −930
Yor1
miss 0
orf 1.3
int −13
rank 4236
yor1−dF
miss 0
orf 3.1
int −8.6
rank 3306
(YIL161W)
DR = 1338
Yor1
miss 0
orf −2.8
int 0.4
rank 1969
yor1−dF
miss 0
orf 4.1
int −8.6
rank 3307
(YKL200C)
DR = 1483
Yor1
miss 0
orf −3
int 1
rank 1825
yor1−dF
miss 0
orf 2.6
int −8.6
rank 3308
(YOR192C)
DR = 225
Yor1
miss 0
orf −0.2
int −4.3
rank 3084
yor1−dF
miss 0
orf 1.8
int −8.6
rank 3309
RXT3(YDL076C)
DR = 3035
Yor1
miss 0
orf −0.3
int 12
rank 276
yor1−dF
miss 0
orf −1
int −8.6
rank 3311
0.00
0.05
0.10
0.15
0.20
0.25
0.35
−60
−20
0
20
40
−50
0
50

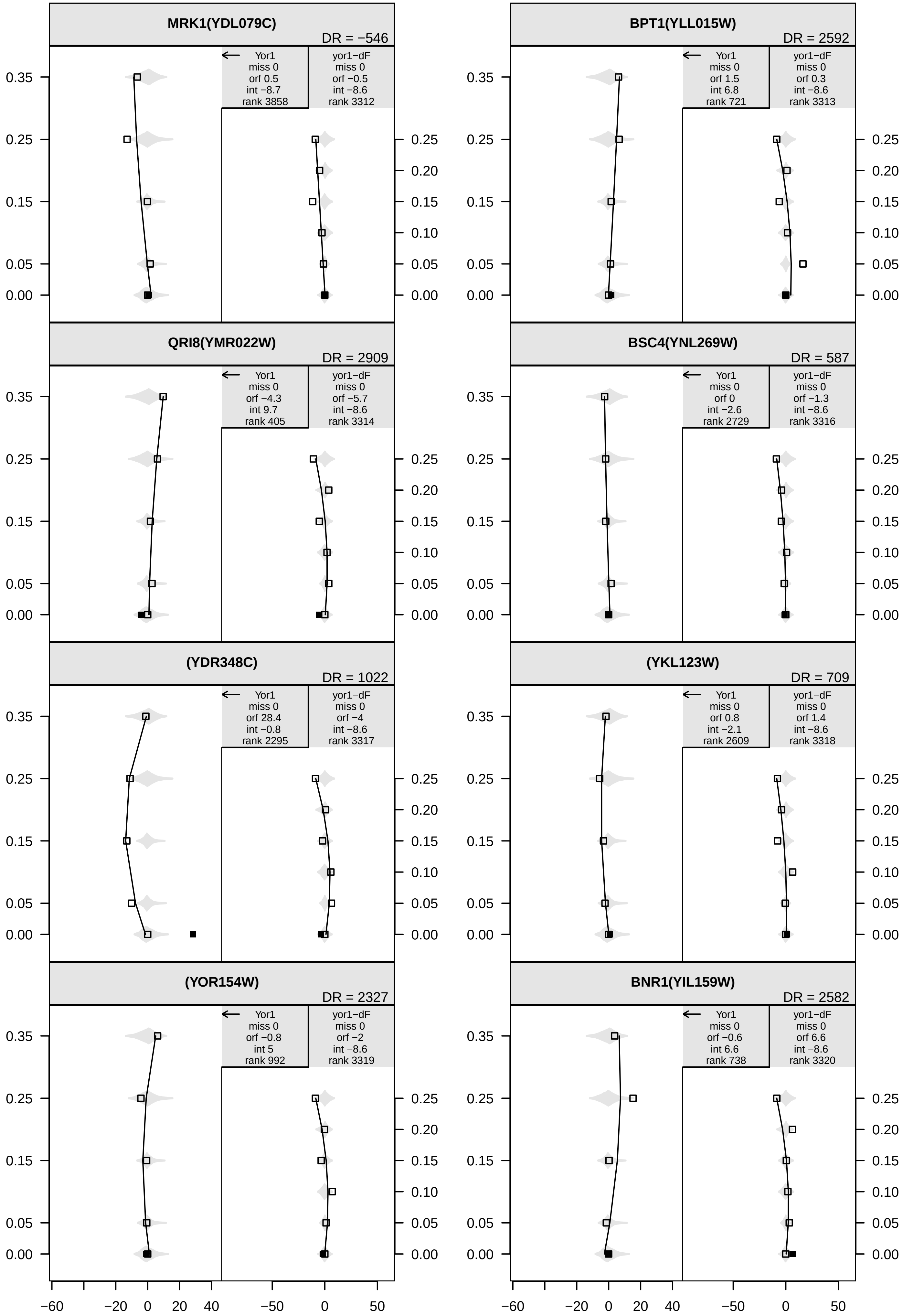
MRK1(YDL079C)
DR = −546
Yor1 miss 0 orf 0.5 int −8.7 rank 3858
yor1−dF miss 0 orf −0.5 int −8.6 rank 3312
BPT1(YLL015W)
DR = 2592
Yor1 miss 0 orf 1.5 int 6.8 rank 721
yor1−dF miss 0 orf 0.3 int −8.6 rank 3313
QRI8(YMR022W)
DR = 2909
Yor1 miss 0 orf −4.3 int 9.7 rank 405
yor1−dF miss 0 orf −5.7 int −8.6 rank 3314
BSC4(YNL269W)
DR = 587
Yor1 miss 0 orf 0 int −2.6 rank 2729
yor1−dF miss 0 orf −1.3 int −8.6 rank 3316
(YDR348C)
DR = 1022
Yor1 miss 0 orf 28.4 int −0.8 rank 2295
yor1−dF miss 0 orf −4 int −8.6 rank 3317
(YKL123W)
DR = 709
Yor1 miss 0 orf 0.8 int −2.1 rank 2609
yor1−dF miss 0 orf 1.4 int −8.6 rank 3318
(YOR154W)
DR = 2327
Yor1 miss 0 orf −0.8 int 5 rank 992
yor1−dF miss 0 orf −2 int −8.6 rank 3319
BNR1(YIL159W)
DR = 2582
Yor1 miss 0 orf −0.6 int 6.6 rank 738
yor1−dF miss 0 orf 6.6 int −8.6 rank 3320

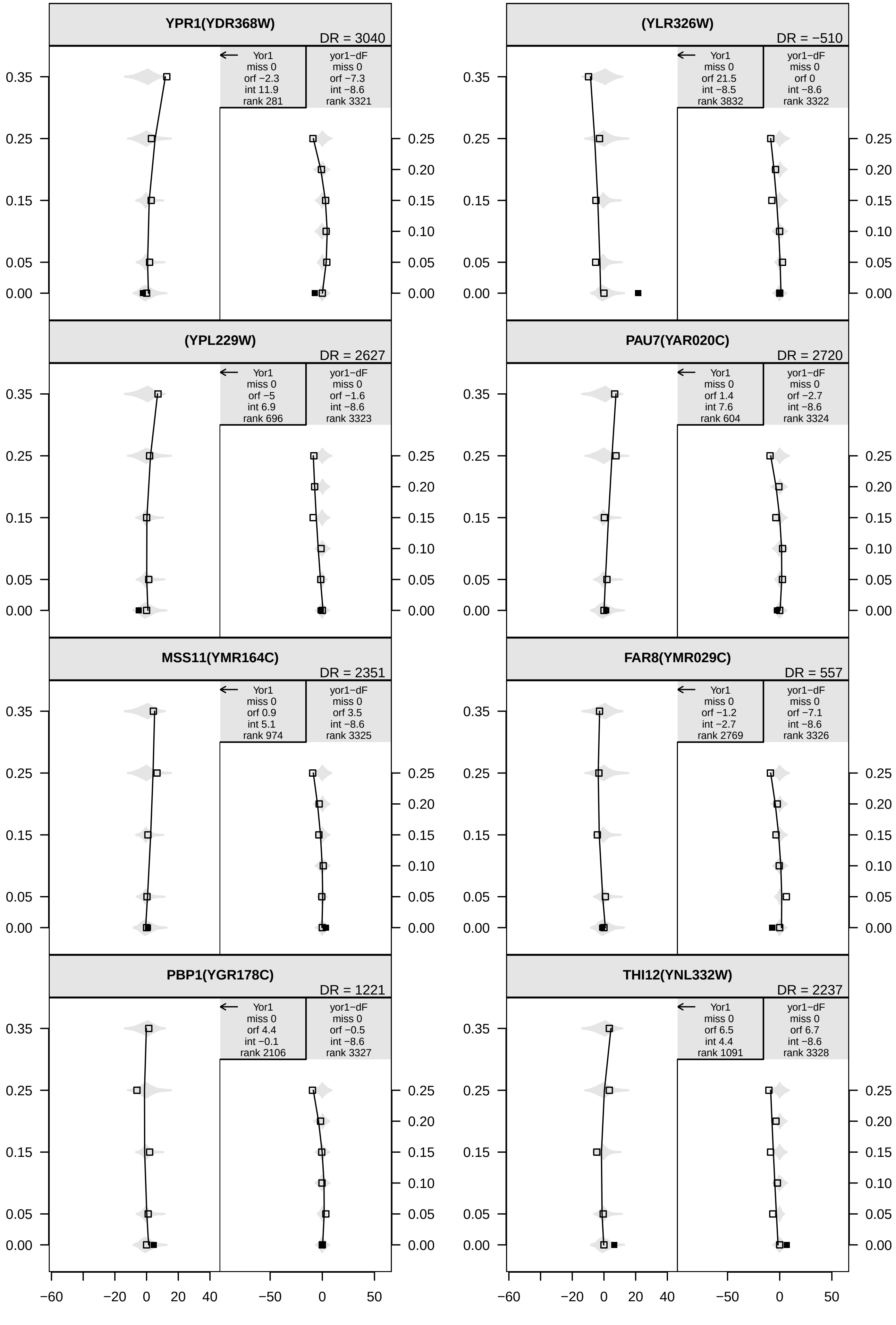

YPR1(YDR368W)
DR = 3040
Yor1 miss 0 orf −2.3 int 11.9 rank 281
yor1−dF miss 0 orf −7.3 int −8.6 rank 3321
(YLR326W)
DR = −510
Yor1 miss 0 orf 21.5 int −8.5 rank 3832
yor1−dF miss 0 orf 0 int −8.6 rank 3322
(YPL229W)
DR = 2627
Yor1 miss 0 orf −5 int 6.9 rank 696
yor1−dF miss 0 orf −1.6 int −8.6 rank 3323
PAU7(YAR020C)
DR = 2720
Yor1 miss 0 orf 1.4 int 7.6 rank 604
yor1−dF miss 0 orf −2.7 int −8.6 rank 3324
MSS11(YMR164C)
DR = 2351
Yor1 miss 0 orf 0.9 int 5.1 rank 974
yor1−dF miss 0 orf 3.5 int −8.6 rank 3325
FAR8(YMR029C)
DR = 557
Yor1 miss 0 orf −1.2 int −2.7 rank 2769
yor1−dF miss 0 orf −7.1 int −8.6 rank 3326
PBP1(YGR178C)
DR = 1221
Yor1 miss 0 orf 4.4 int −0.1 rank 2106
yor1−dF miss 0 orf −0.5 int −8.6 rank 3327
THI12(YNL332W)
DR = 2237
Yor1 miss 0 orf 6.5 int 4.4 rank 1091
yor1−dF miss 0 orf 6.7 int −8.6 rank 3328
0.00 0.05 0.10 0.15 0.20 0.25 0.35
−60 −20 0 20 40 −50 0 50

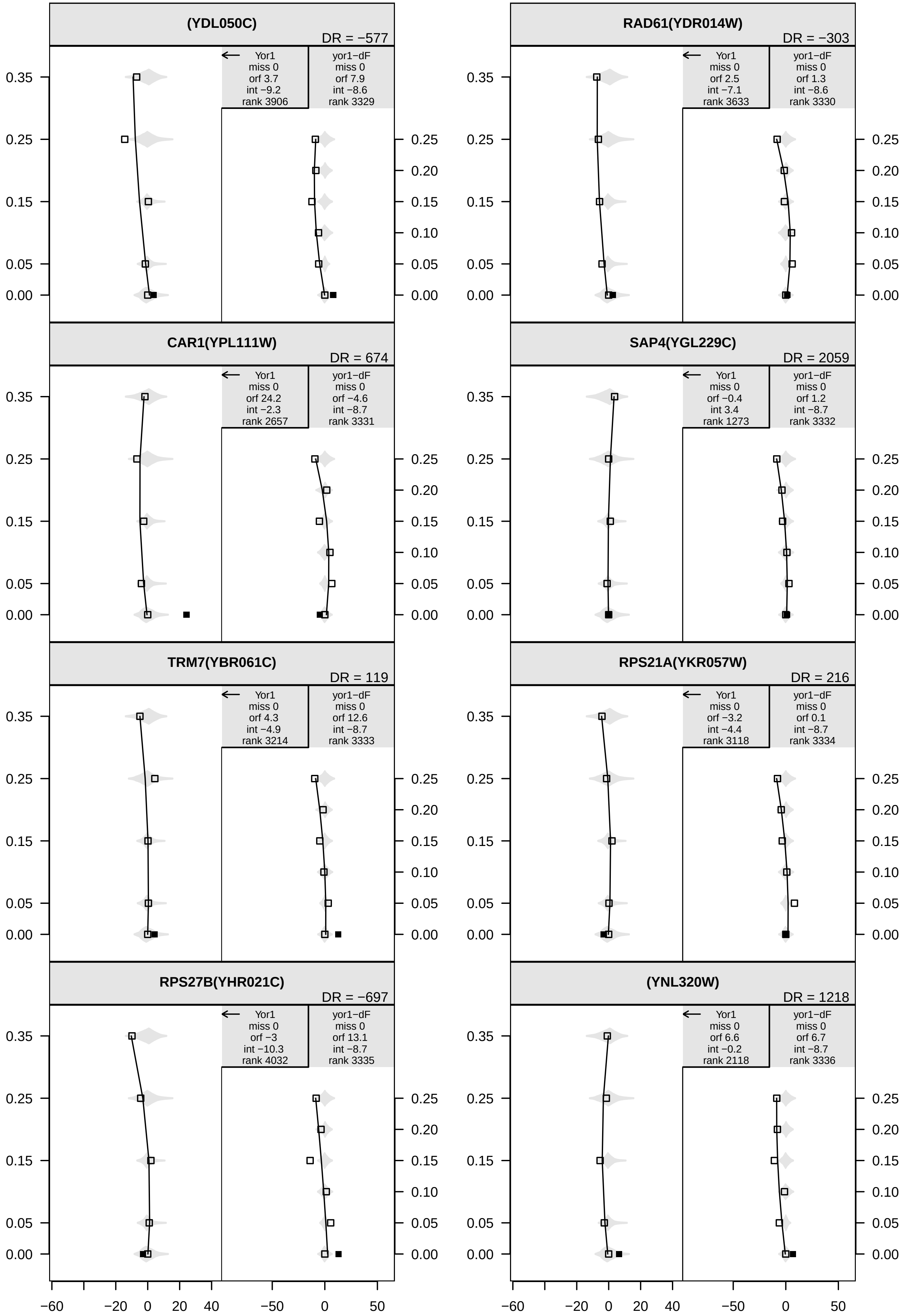
(YDL050C)
DR = −577
Yor1 miss 0 orf 3.7 int −9.2 rank 3906
yor1−dF miss 0 orf 7.9 int −8.6 rank 3329
RAD61(YDR014W)
DR = −303
Yor1 miss 0 orf 2.5 int −7.1 rank 3633
yor1−dF miss 0 orf 1.3 int −8.6 rank 3330
CAR1(YPL111W)
DR = 674
Yor1 miss 0 orf 24.2 int −2.3 rank 2657
yor1−dF miss 0 orf −4.6 int −8.7 rank 3331
SAP4(YGL229C)
DR = 2059
Yor1 miss 0 orf −0.4 int 3.4 rank 1273
yor1−dF miss 0 orf 1.2 int −8.7 rank 3332
TRM7(YBR061C)
DR = 119
Yor1 miss 0 orf 4.3 int −4.9 rank 3214
yor1−dF miss 0 orf 12.6 int −8.7 rank 3333
RPS21A(YKR057W)
DR = 216
Yor1 miss 0 orf −3.2 int −4.4 rank 3118
yor1−dF miss 0 orf 0.1 int −8.7 rank 3334
RPS27B(YHR021C)
DR = −697
Yor1 miss 0 orf −3 int −10.3 rank 4032
yor1−dF miss 0 orf 13.1 int −8.7 rank 3335
(YNL320W)
DR = 1218
Yor1 miss 0 orf 6.6 int −0.2 rank 2118
yor1−dF miss 0 orf 6.7 int −8.7 rank 3336
0.35
0.25
0.20
0.15
0.10
0.05
0.00
−60
−20
0
20
40
−50
0
50

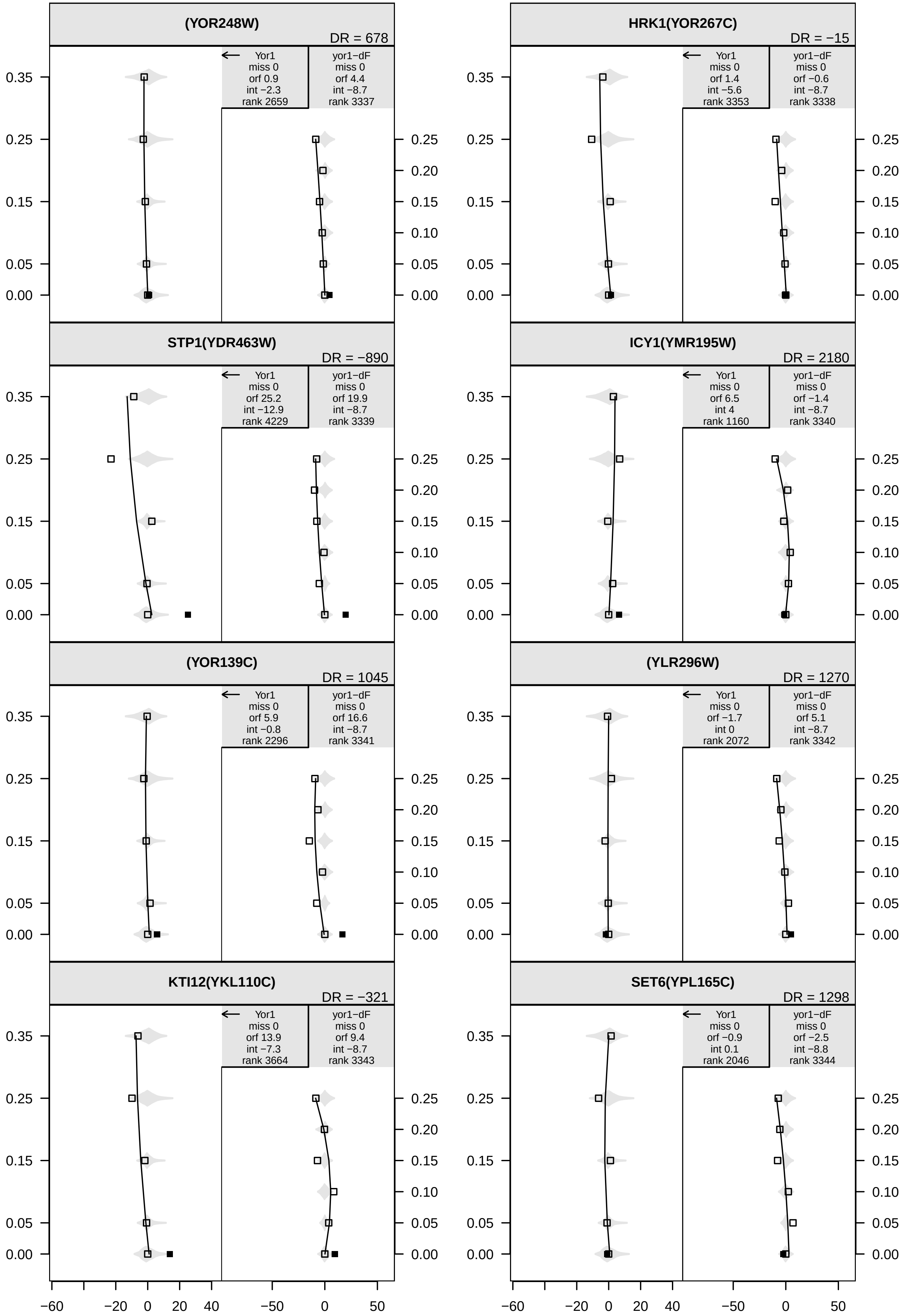

(YOR248W)
DR = 678
Yor1
miss 0
orf 0.9
int −2.3
rank 2659
yor1−dF
miss 0
orf 4.4
int −8.7
rank 3337
HRK1(YOR267C)
DR = −15
Yor1
miss 0
orf 1.4
int −5.6
rank 3353
yor1−dF
miss 0
orf −0.6
int −8.7
rank 3338
STP1(YDR463W)
DR = −890
Yor1
miss 0
orf 25.2
int −12.9
rank 4229
yor1−dF
miss 0
orf 19.9
int −8.7
rank 3339
ICY1(YMR195W)
DR = 2180
Yor1
miss 0
orf 6.5
int 4
rank 1160
yor1−dF
miss 0
orf −1.4
int −8.7
rank 3340
(YOR139C)
DR = 1045
Yor1
miss 0
orf 5.9
int −0.8
rank 2296
yor1−dF
miss 0
orf 16.6
int −8.7
rank 3341
(YLR296W)
DR = 1270
Yor1
miss 0
orf −1.7
int 0
rank 2072
yor1−dF
miss 0
orf 5.1
int −8.7
rank 3342
KTI12(YKL110C)
DR = −321
Yor1
miss 0
orf 13.9
int −7.3
rank 3664
yor1−dF
miss 0
orf 9.4
int −8.7
rank 3343
SET6(YPL165C)
DR = 1298
Yor1
miss 0
orf −0.9
int 0.1
rank 2046
yor1−dF
miss 0
orf −2.5
int −8.8
rank 3344
0.35
0.25
0.20
0.15
0.10
0.05
0.00
−60
−20
0
20
40
−50
0
50

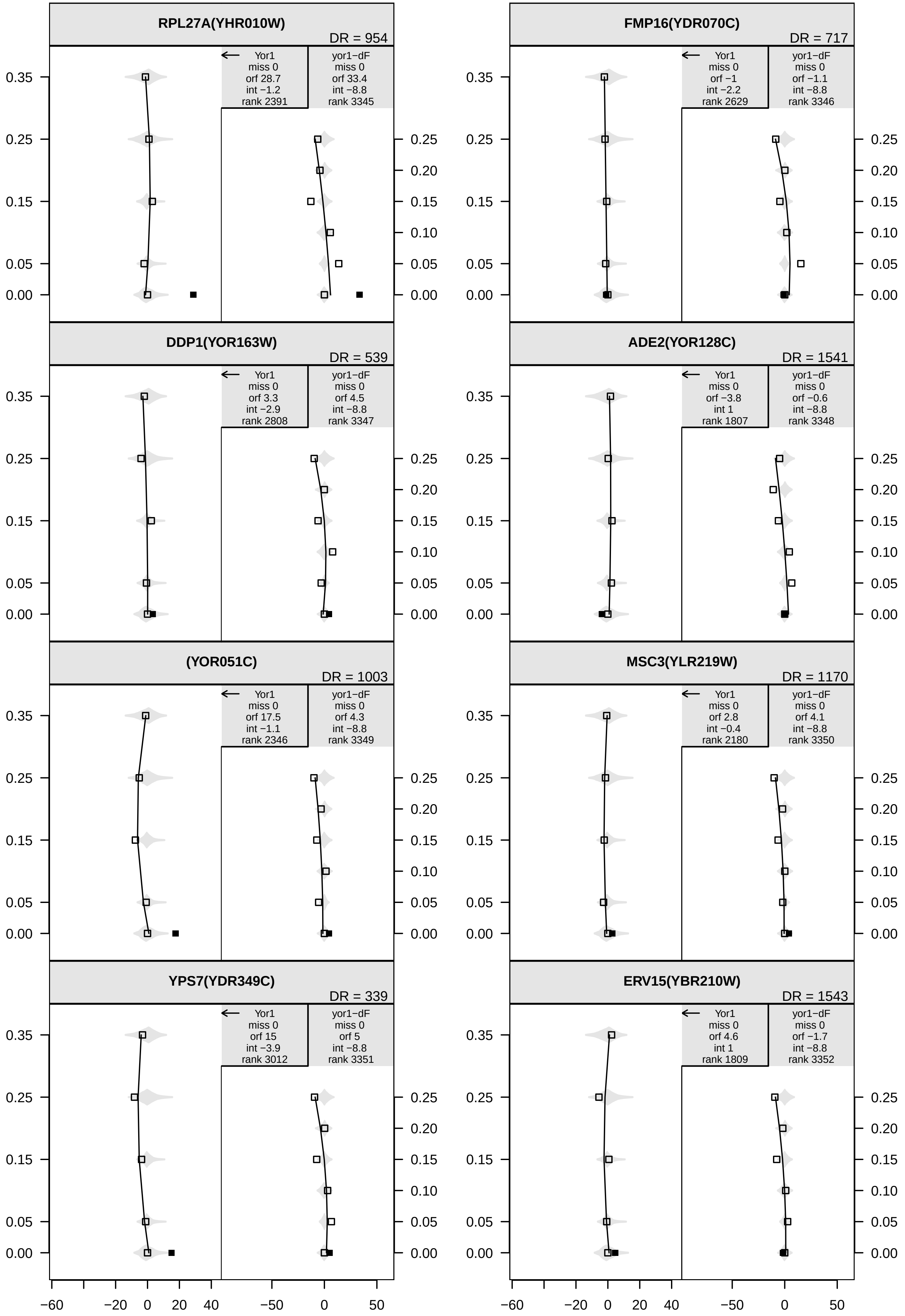
RPL27A(YHR010W)
DR = 954
Yor1
miss 0
orf 28.7
int −1.2
rank 2391
yor1−dF
miss 0
orf 33.4
int −8.8
rank 3345
FMP16(YDR070C)
DR = 717
Yor1
miss 0
orf −1
int −2.2
rank 2629
yor1−dF
miss 0
orf −1.1
int −8.8
rank 3346
DDP1(YOR163W)
DR = 539
Yor1
miss 0
orf 3.3
int −2.9
rank 2808
yor1−dF
miss 0
orf 4.5
int −8.8
rank 3347
ADE2(YOR128C)
DR = 1541
Yor1
miss 0
orf −3.8
int 1
rank 1807
yor1−dF
miss 0
orf −0.6
int −8.8
rank 3348
(YOR051C)
DR = 1003
Yor1
miss 0
orf 17.5
int −1.1
rank 2346
yor1−dF
miss 0
orf 4.3
int −8.8
rank 3349
MSC3(YLR219W)
DR = 1170
Yor1
miss 0
orf 2.8
int −0.4
rank 2180
yor1−dF
miss 0
orf 4.1
int −8.8
rank 3350
YPS7(YDR349C)
DR = 339
Yor1
miss 0
orf 15
int −3.9
rank 3012
yor1−dF
miss 0
orf 5
int −8.8
rank 3351
ERV15(YBR210W)
DR = 1543
Yor1
miss 0
orf 4.6
int 1
rank 1809
yor1−dF
miss 0
orf −1.7
int −8.8
rank 3352
0.35
0.25
0.20
0.15
0.10
0.05
0.00
−60
−20
0
20
40
−50
0
50

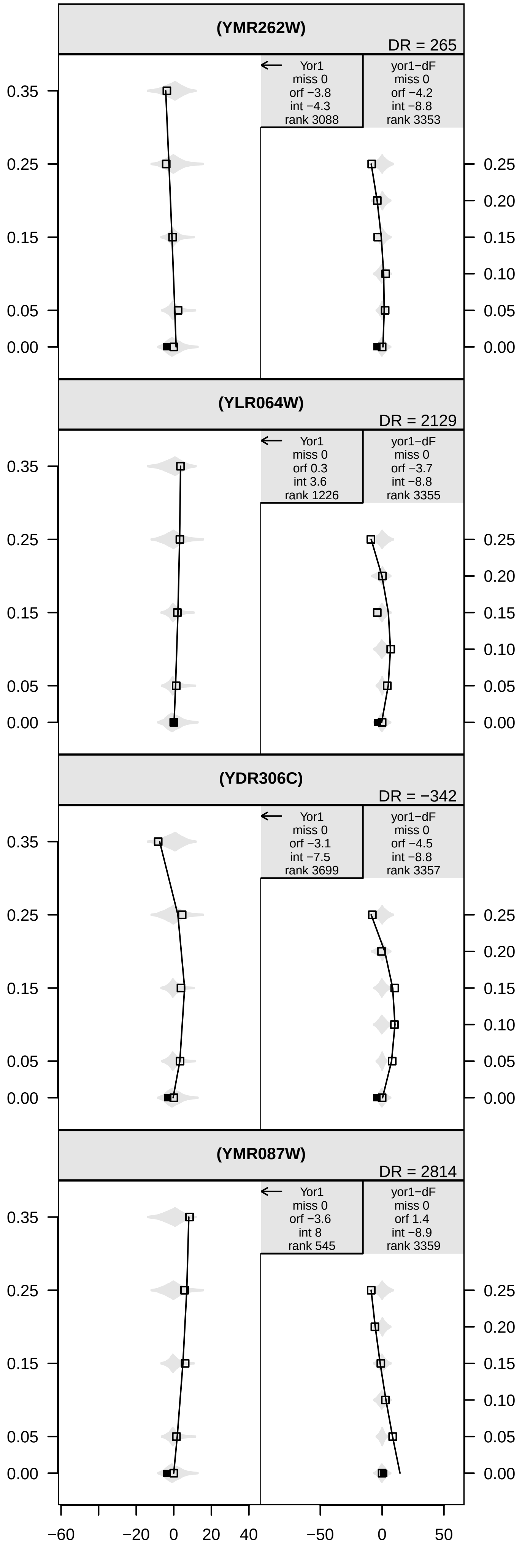

(YMR262W)
DR = 265
Yor1 miss 0 orf −3.8 int −4.3 rank 3088
yor1−dF miss 0 orf −4.2 int −8.8 rank 3353
ECM21(YBL101C)
DR = 1407
Yor1 miss 0 orf −0.5 int 0.5 rank 1947
yor1−dF miss 0 orf 5.1 int −8.8 rank 3354
(YLR064W)
DR = 2129
Yor1 miss 0 orf 0.3 int 3.6 rank 1226
yor1−dF miss 0 orf −3.7 int −8.8 rank 3355
(YJL135W)
DR = 2111
Yor1 miss 0 orf 4.3 int 3.5 rank 1245
yor1−dF miss 0 orf 2.9 int −8.8 rank 3356
(YDR306C)
DR = −342
Yor1 miss 0 orf −3.1 int −7.5 rank 3699
yor1−dF miss 0 orf −4.5 int −8.8 rank 3357
AMD2(YDR242W)
DR = 2982
Yor1 miss 0 orf −0.7 int 10.3 rank 376
yor1−dF miss 0 orf 5.8 int −8.9 rank 3358
(YMR087W)
DR = 2814
Yor1 miss 0 orf −3.6 int 8 rank 545
yor1−dF miss 0 orf 1.4 int −8.9 rank 3359
(YNL040W)
DR = −505
Yor1 miss 0 orf 7.2 int −8.8 rank 3865
yor1−dF miss 0 orf 3.5 int −8.9 rank 3360
0.35 0.25 0.20 0.15 0.10 0.05 0.00
−60 −20 0 20 40 −50 0 50

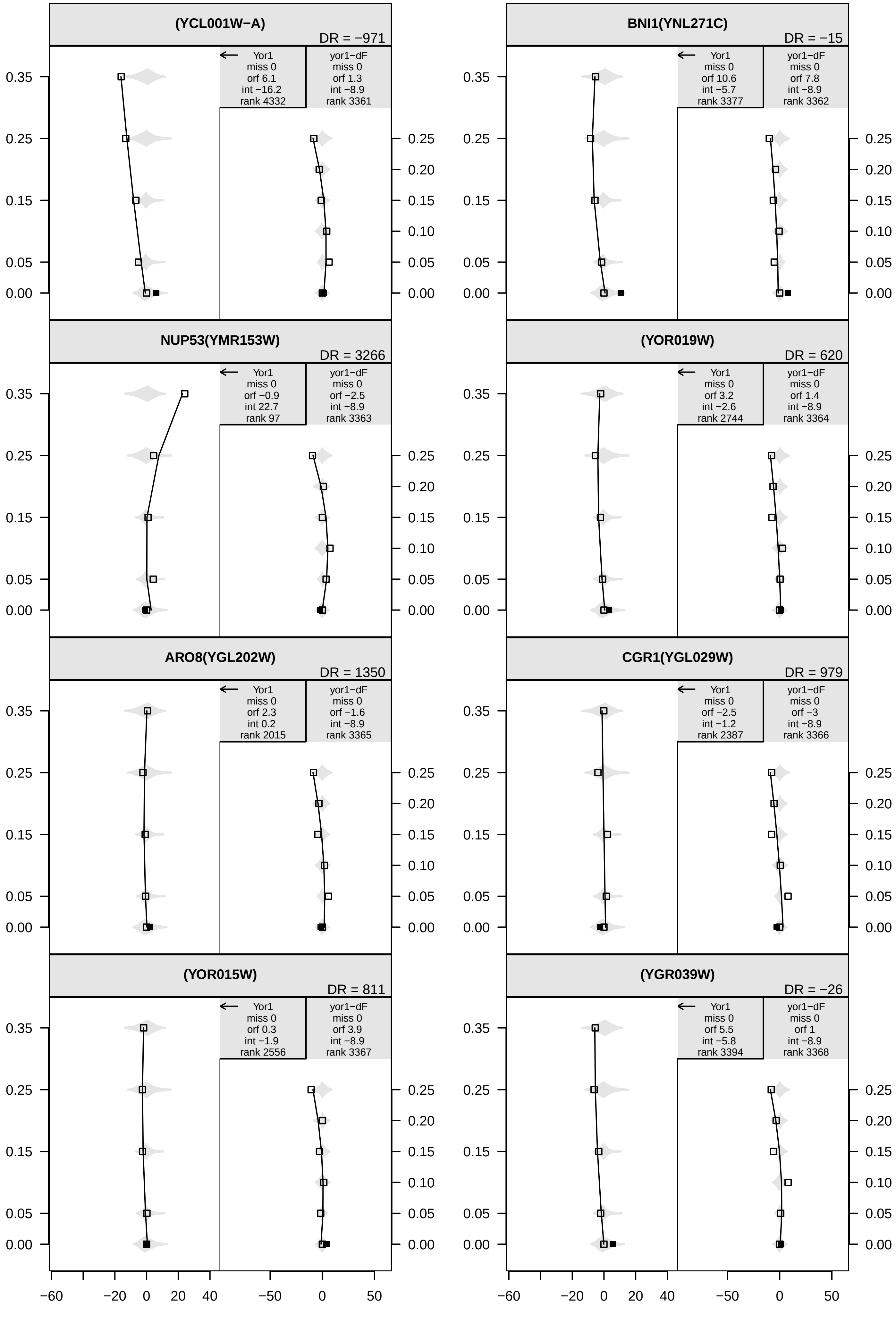
(YCL001W−A)
DR = −971
Yor1
miss 0
orf 6.1
int −16.2
rank 4332
yor1−dF
miss 0
orf 1.3
int −8.9
rank 3361
BNI1(YNL271C)
DR = −15
Yor1
miss 0
orf 10.6
int −5.7
rank 3377
yor1−dF
miss 0
orf 7.8
int −8.9
rank 3362
NUP53(YMR153W)
DR = 3266
Yor1
miss 0
orf −0.9
int 22.7
rank 97
yor1−dF
miss 0
orf −2.5
int −8.9
rank 3363
(YOR019W)
DR = 620
Yor1
miss 0
orf 3.2
int −2.6
rank 2744
yor1−dF
miss 0
orf 1.4
int −8.9
rank 3364
ARO8(YGL202W)
DR = 1350
Yor1
miss 0
orf 2.3
int 0.2
rank 2015
yor1−dF
miss 0
orf −1.6
int −8.9
rank 3365
CGR1(YGL029W)
DR = 979
Yor1
miss 0
orf −2.5
int −1.2
rank 2387
yor1−dF
miss 0
orf −3
int −8.9
rank 3366
(YOR015W)
DR = 811
Yor1
miss 0
orf 0.3
int −1.9
rank 2556
yor1−dF
miss 0
orf 3.9
int −8.9
rank 3367
(YGR039W)
DR = −26
Yor1
miss 0
orf 5.5
int −5.8
rank 3394
yor1−dF
miss 0
orf 1
int −8.9
rank 3368
0.35
0.25
0.20
0.15
0.10
0.05
0.00
−60
−20
0
20
40
−50
0
50

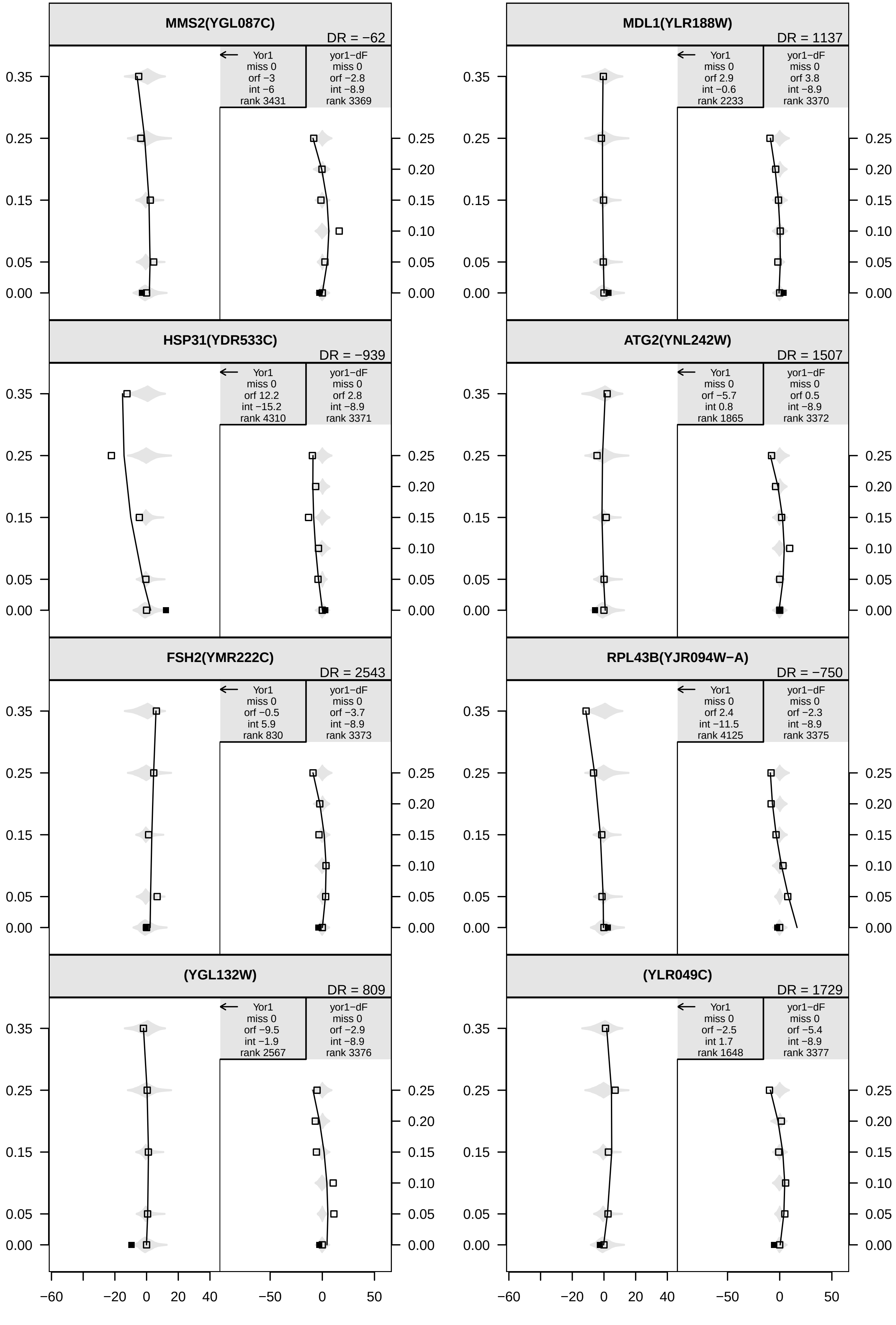
MMS2(YGL087C)
DR = −62
Yor1 miss 0 orf −3 int −6 rank 3431
yor1−dF miss 0 orf −2.8 int −8.9 rank 3369
MDL1(YLR188W)
DR = 1137
Yor1 miss 0 orf 2.9 int −0.6 rank 2233
yor1−dF miss 0 orf 3.8 int −8.9 rank 3370
HSP31(YDR533C)
DR = −939
Yor1 miss 0 orf 12.2 int −15.2 rank 4310
yor1−dF miss 0 orf 2.8 int −8.9 rank 3371
ATG2(YNL242W)
DR = 1507
Yor1 miss 0 orf −5.7 int 0.8 rank 1865
yor1−dF miss 0 orf 0.5 int −8.9 rank 3372
FSH2(YMR222C)
DR = 2543
Yor1 miss 0 orf −0.5 int 5.9 rank 830
yor1−dF miss 0 orf −3.7 int −8.9 rank 3373
RPL43B(YJR094W−A)
DR = −750
Yor1 miss 0 orf 2.4 int −11.5 rank 4125
yor1−dF miss 0 orf −2.3 int −8.9 rank 3375
(YGL132W)
DR = 809
Yor1 miss 0 orf −9.5 int −1.9 rank 2567
yor1−dF miss 0 orf −2.9 int −8.9 rank 3376
(YLR049C)
DR = 1729
Yor1 miss 0 orf −2.5 int 1.7 rank 1648
yor1−dF miss 0 orf −5.4 int −8.9 rank 3377

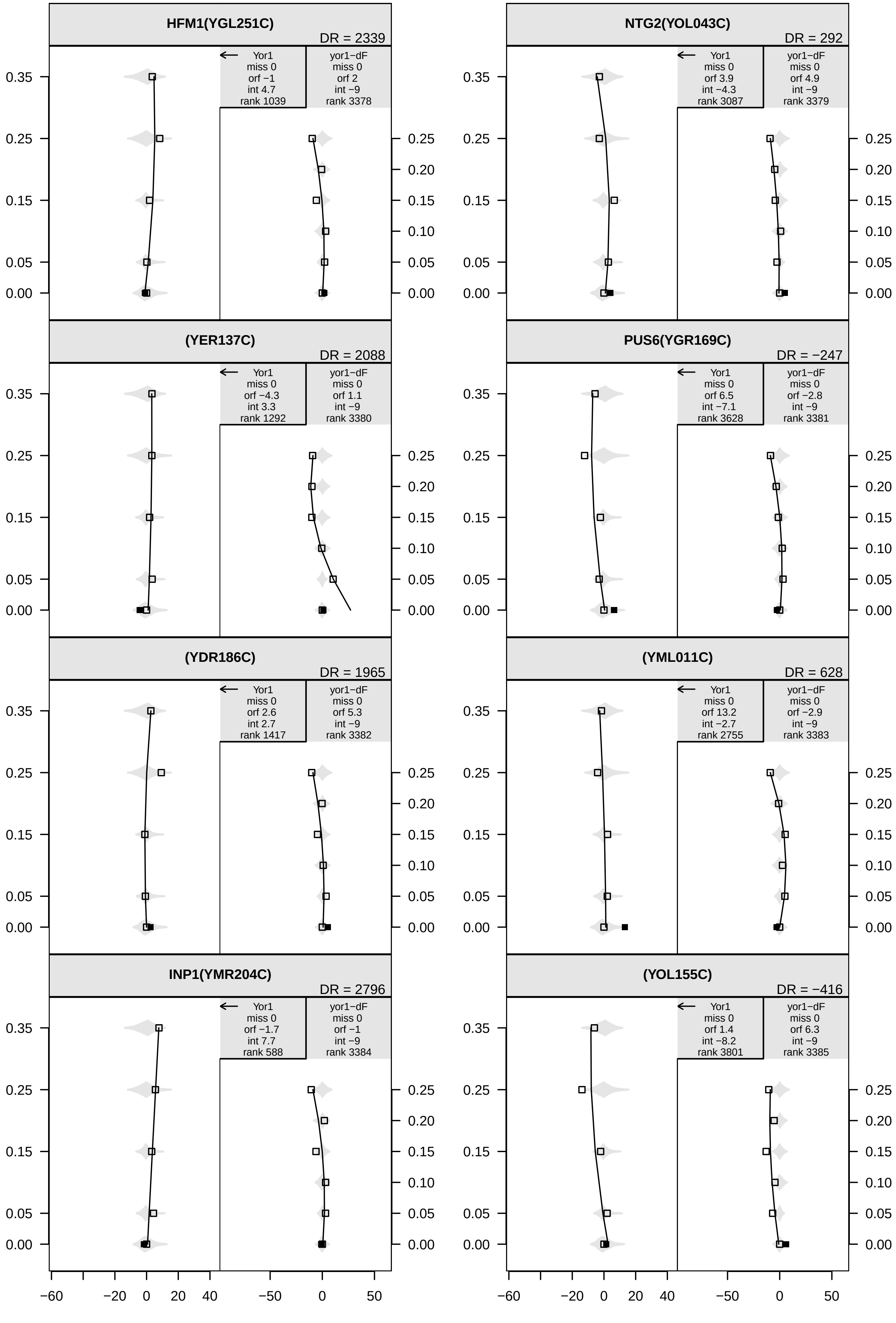

HFM1(YGL251C)
DR = 2339
Yor1 miss 0 orf −1 int 4.7 rank 1039
yor1−dF miss 0 orf 2 int −9 rank 3378
NTG2(YOL043C)
DR = 292
Yor1 miss 0 orf 3.9 int −4.3 rank 3087
yor1−dF miss 0 orf 4.9 int −9 rank 3379
(YER137C)
DR = 2088
Yor1 miss 0 orf −4.3 int 3.3 rank 1292
yor1−dF miss 0 orf 1.1 int −9 rank 3380
PUS6(YGR169C)
DR = −247
Yor1 miss 0 orf 6.5 int −7.1 rank 3628
yor1−dF miss 0 orf −2.8 int −9 rank 3381
(YDR186C)
DR = 1965
Yor1 miss 0 orf 2.6 int 2.7 rank 1417
yor1−dF miss 0 orf 5.3 int −9 rank 3382
(YML011C)
DR = 628
Yor1 miss 0 orf 13.2 int −2.7 rank 2755
yor1−dF miss 0 orf −2.9 int −9 rank 3383
INP1(YMR204C)
DR = 2796
Yor1 miss 0 orf −1.7 int 7.7 rank 588
yor1−dF miss 0 orf −1 int −9 rank 3384
(YOL155C)
DR = −416
Yor1 miss 0 orf 1.4 int −8.2 rank 3801
yor1−dF miss 0 orf 6.3 int −9 rank 3385
0.00 0.05 0.10 0.15 0.20 0.25 0.35
−60 −20 0 20 40 −50 0 50

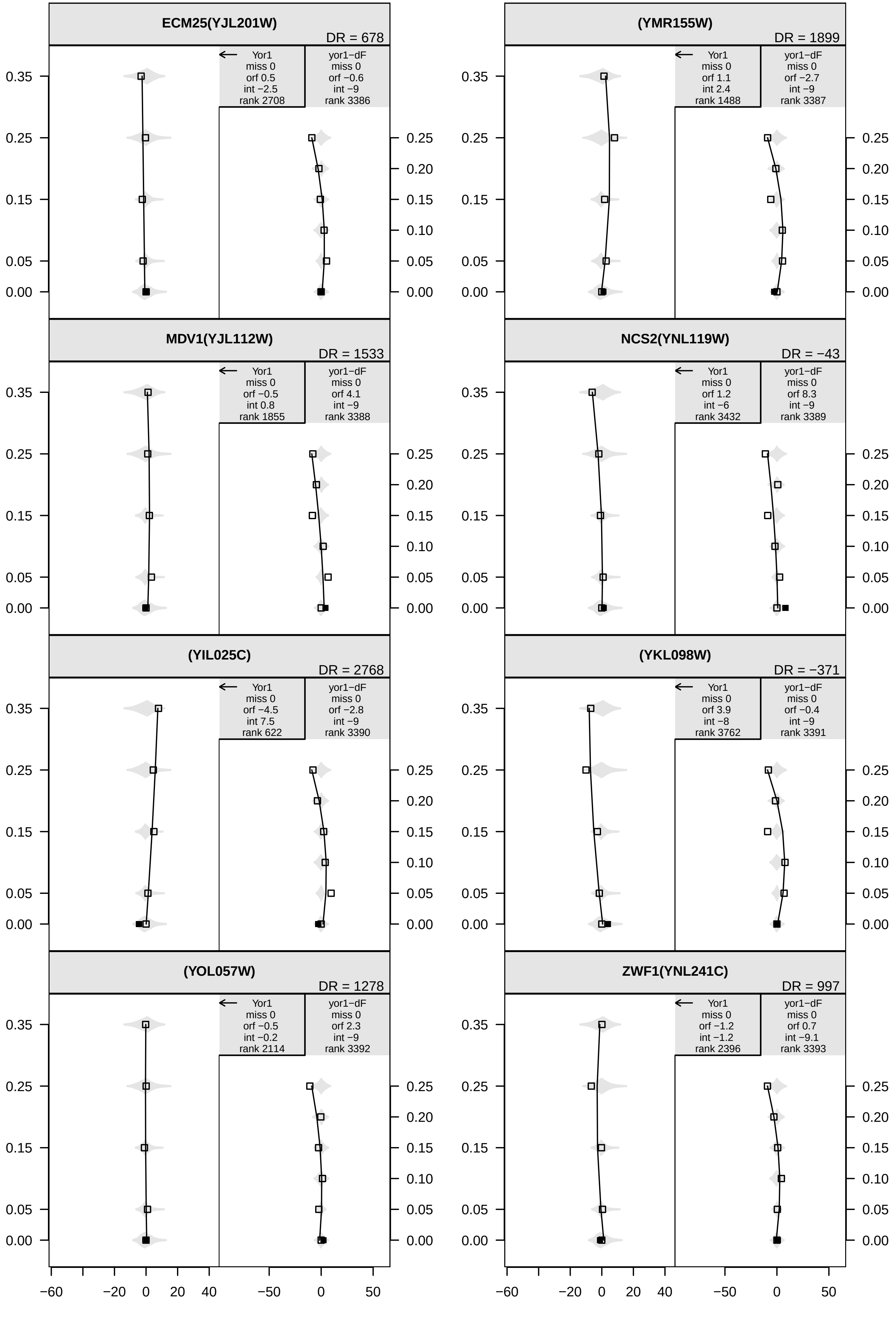

ECM25(YJL201W)
DR = 678
Yor1 miss 0 orf 0.5 int −2.5 rank 2708
yor1−dF miss 0 orf −0.6 int −9 rank 3386
(YMR155W)
DR = 1899
Yor1 miss 0 orf 1.1 int 2.4 rank 1488
yor1−dF miss 0 orf −2.7 int −9 rank 3387
MDV1(YJL112W)
DR = 1533
Yor1 miss 0 orf −0.5 int 0.8 rank 1855
yor1−dF miss 0 orf 4.1 int −9 rank 3388
NCS2(YNL119W)
DR = −43
Yor1 miss 0 orf 1.2 int −6 rank 3432
yor1−dF miss 0 orf 8.3 int −9 rank 3389
(YIL025C)
DR = 2768
Yor1 miss 0 orf −4.5 int 7.5 rank 622
yor1−dF miss 0 orf −2.8 int −9 rank 3390
(YKL098W)
DR = −371
Yor1 miss 0 orf 3.9 int −8 rank 3762
yor1−dF miss 0 orf −0.4 int −9 rank 3391
(YOL057W)
DR = 1278
Yor1 miss 0 orf −0.5 int −0.2 rank 2114
yor1−dF miss 0 orf 2.3 int −9 rank 3392
ZWF1(YNL241C)
DR = 997
Yor1 miss 0 orf −1.2 int −1.2 rank 2396
yor1−dF miss 0 orf 0.7 int −9.1 rank 3393
0.35 0.25 0.15 0.05 0.00
0.25 0.20 0.15 0.10 0.05 0.00
−60 −20 0 20 40
−50 0 50

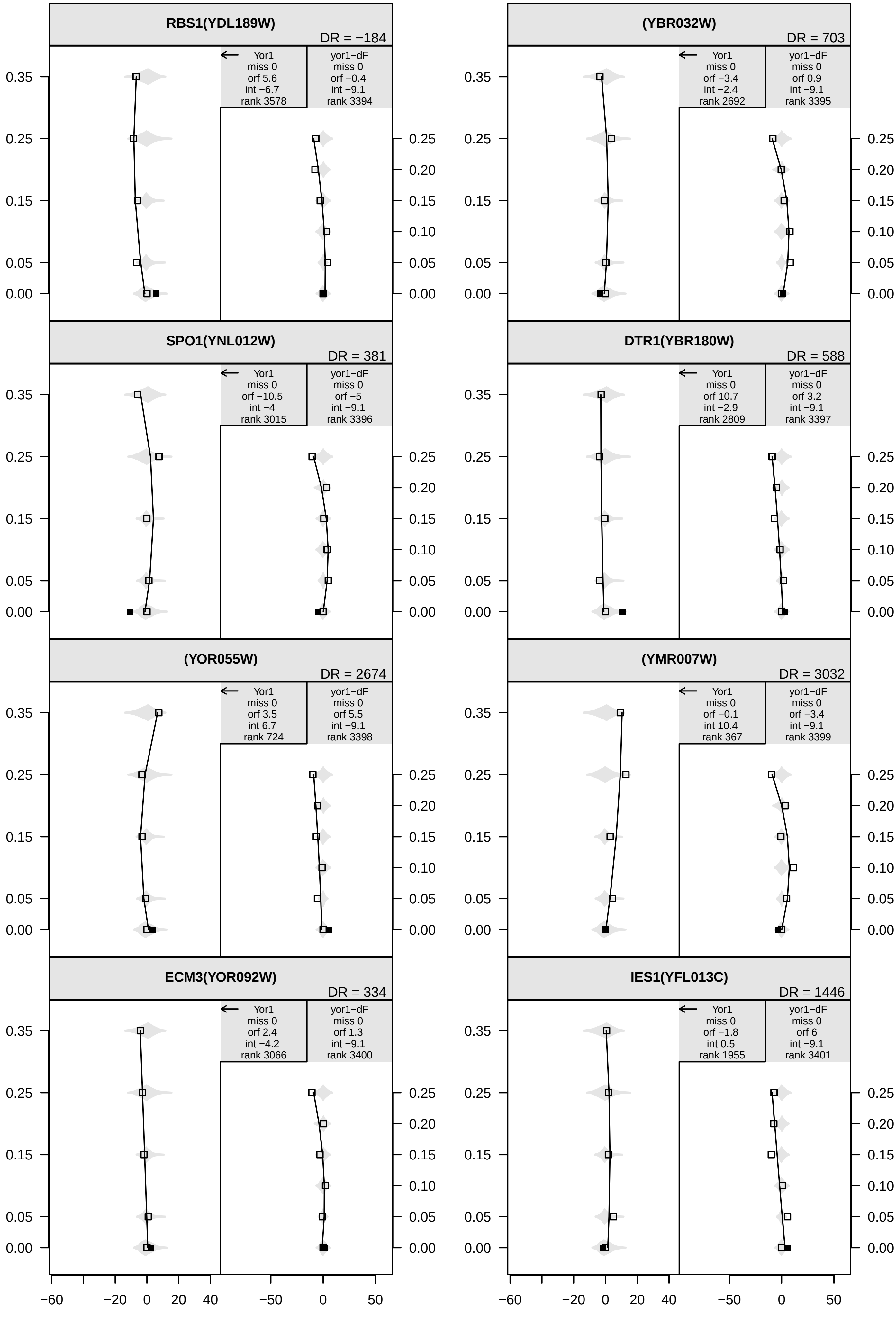
RBS1(YDL189W)
DR = −184
Yor1 miss 0 orf 5.6 int −6.7 rank 3578
yor1−dF miss 0 orf −0.4 int −9.1 rank 3394
(YBR032W)
DR = 703
Yor1 miss 0 orf −3.4 int −2.4 rank 2692
yor1−dF miss 0 orf 0.9 int −9.1 rank 3395
SPO1(YNL012W)
DR = 381
Yor1 miss 0 orf −10.5 int −4 rank 3015
yor1−dF miss 0 orf −5 int −9.1 rank 3396
DTR1(YBR180W)
DR = 588
Yor1 miss 0 orf 10.7 int −2.9 rank 2809
yor1−dF miss 0 orf 3.2 int −9.1 rank 3397
(YOR055W)
DR = 2674
Yor1 miss 0 orf 3.5 int 6.7 rank 724
yor1−dF miss 0 orf 5.5 int −9.1 rank 3398
(YMR007W)
DR = 3032
Yor1 miss 0 orf −0.1 int 10.4 rank 367
yor1−dF miss 0 orf −3.4 int −9.1 rank 3399
ECM3(YOR092W)
DR = 334
Yor1 miss 0 orf 2.4 int −4.2 rank 3066
yor1−dF miss 0 orf 1.3 int −9.1 rank 3400
IES1(YFL013C)
DR = 1446
Yor1 miss 0 orf −1.8 int 0.5 rank 1955
yor1−dF miss 0 orf 6 int −9.1 rank 3401
0.35 0.25 0.15 0.05 0.00
0.25 0.20 0.15 0.10 0.05 0.00
−60 −20 0 20 40 −50 0 50

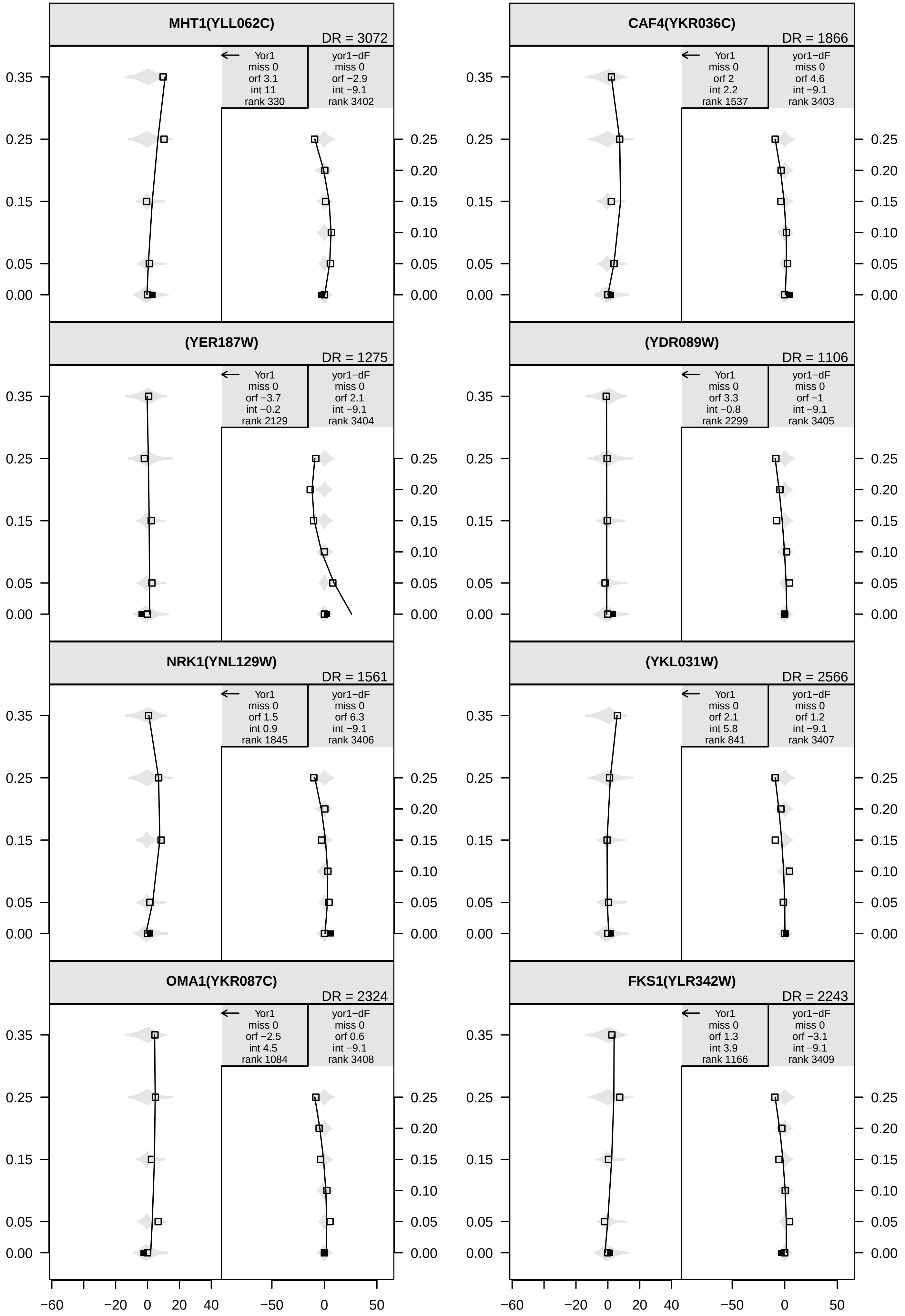
MHT1(YLL062C)
DR = 3072
Yor1
miss 0
orf 3.1
int 11
rank 330
yor1−dF
miss 0
orf −2.9
int −9.1
rank 3402
CAF4(YKR036C)
DR = 1866
Yor1
miss 0
orf 2
int 2.2
rank 1537
yor1−dF
miss 0
orf 4.6
int −9.1
rank 3403
(YER187W)
DR = 1275
Yor1
miss 0
orf −3.7
int −0.2
rank 2129
yor1−dF
miss 0
orf 2.1
int −9.1
rank 3404
(YDR089W)
DR = 1106
Yor1
miss 0
orf 3.3
int −0.8
rank 2299
yor1−dF
miss 0
orf −1
int −9.1
rank 3405
NRK1(YNL129W)
DR = 1561
Yor1
miss 0
orf 1.5
int 0.9
rank 1845
yor1−dF
miss 0
orf 6.3
int −9.1
rank 3406
(YKL031W)
DR = 2566
Yor1
miss 0
orf 2.1
int 5.8
rank 841
yor1−dF
miss 0
orf 1.2
int −9.1
rank 3407
OMA1(YKR087C)
DR = 2324
Yor1
miss 0
orf −2.5
int 4.5
rank 1084
yor1−dF
miss 0
orf 0.6
int −9.1
rank 3408
FKS1(YLR342W)
DR = 2243
Yor1
miss 0
orf 1.3
int 3.9
rank 1166
yor1−dF
miss 0
orf −3.1
int −9.1
rank 3409
0.00
0.05
0.10
0.15
0.20
0.25
0.35
−60
−20
0
20
40
−50
0
50

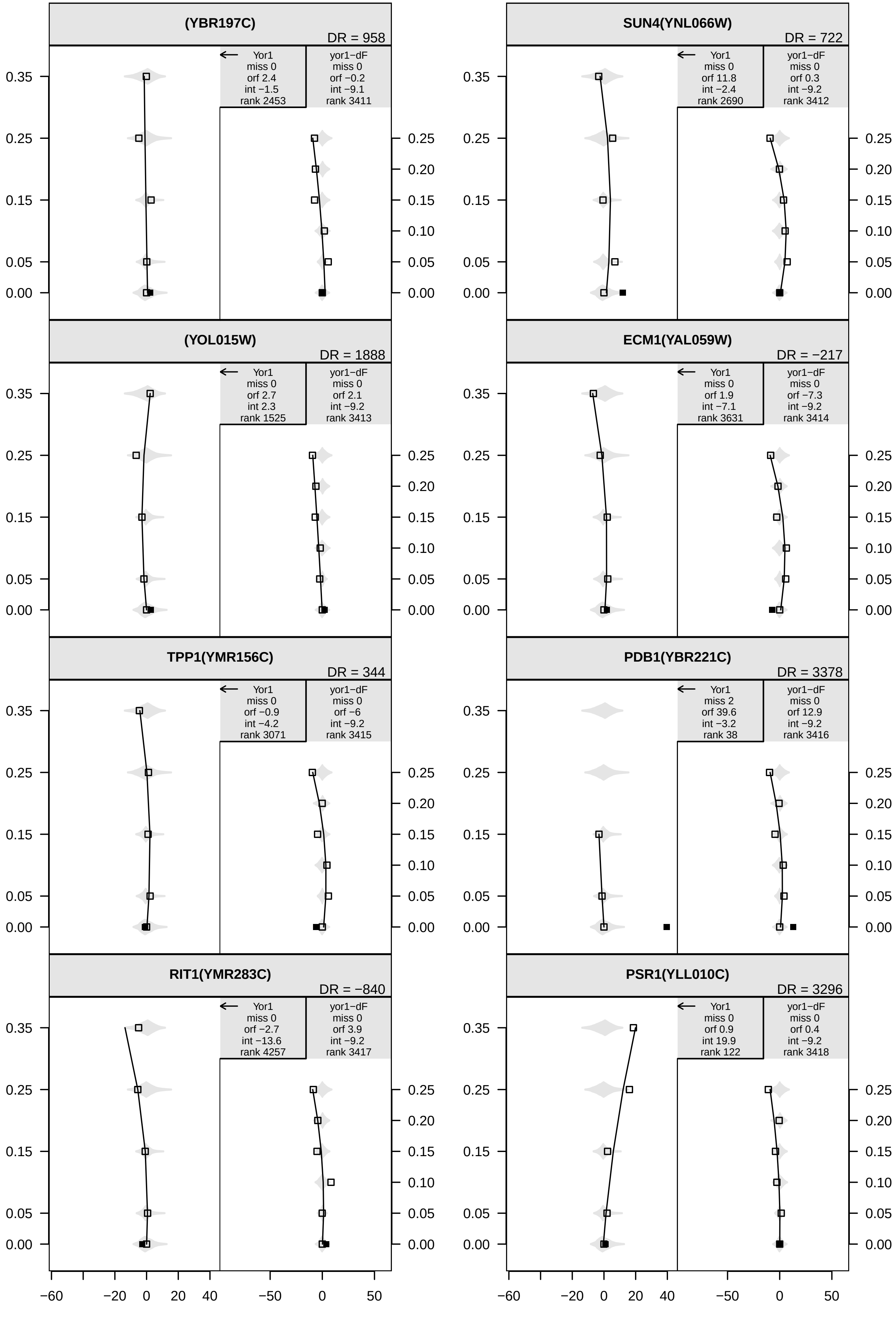

(YBR197C)
DR = 958
Yor1 miss 0 orf 2.4 int −1.5 rank 2453
yor1−dF miss 0 orf −0.2 int −9.1 rank 3411
SUN4(YNL066W)
DR = 722
Yor1 miss 0 orf 11.8 int −2.4 rank 2690
yor1−dF miss 0 orf 0.3 int −9.2 rank 3412
(YOL015W)
DR = 1888
Yor1 miss 0 orf 2.7 int 2.3 rank 1525
yor1−dF miss 0 orf 2.1 int −9.2 rank 3413
ECM1(YAL059W)
DR = −217
Yor1 miss 0 orf 1.9 int −7.1 rank 3631
yor1−dF miss 0 orf −7.3 int −9.2 rank 3414
TPP1(YMR156C)
DR = 344
Yor1 miss 0 orf −0.9 int −4.2 rank 3071
yor1−dF miss 0 orf −6 int −9.2 rank 3415
PDB1(YBR221C)
DR = 3378
Yor1 miss 2 orf 39.6 int −3.2 rank 38
yor1−dF miss 0 orf 12.9 int −9.2 rank 3416
RIT1(YMR283C)
DR = −840
Yor1 miss 0 orf −2.7 int −13.6 rank 4257
yor1−dF miss 0 orf 3.9 int −9.2 rank 3417
PSR1(YLL010C)
DR = 3296
Yor1 miss 0 orf 0.9 int 19.9 rank 122
yor1−dF miss 0 orf 0.4 int −9.2 rank 3418
0.35 0.25 0.20 0.15 0.10 0.05 0.00
−60 −20 0 20 40 −50 0 50

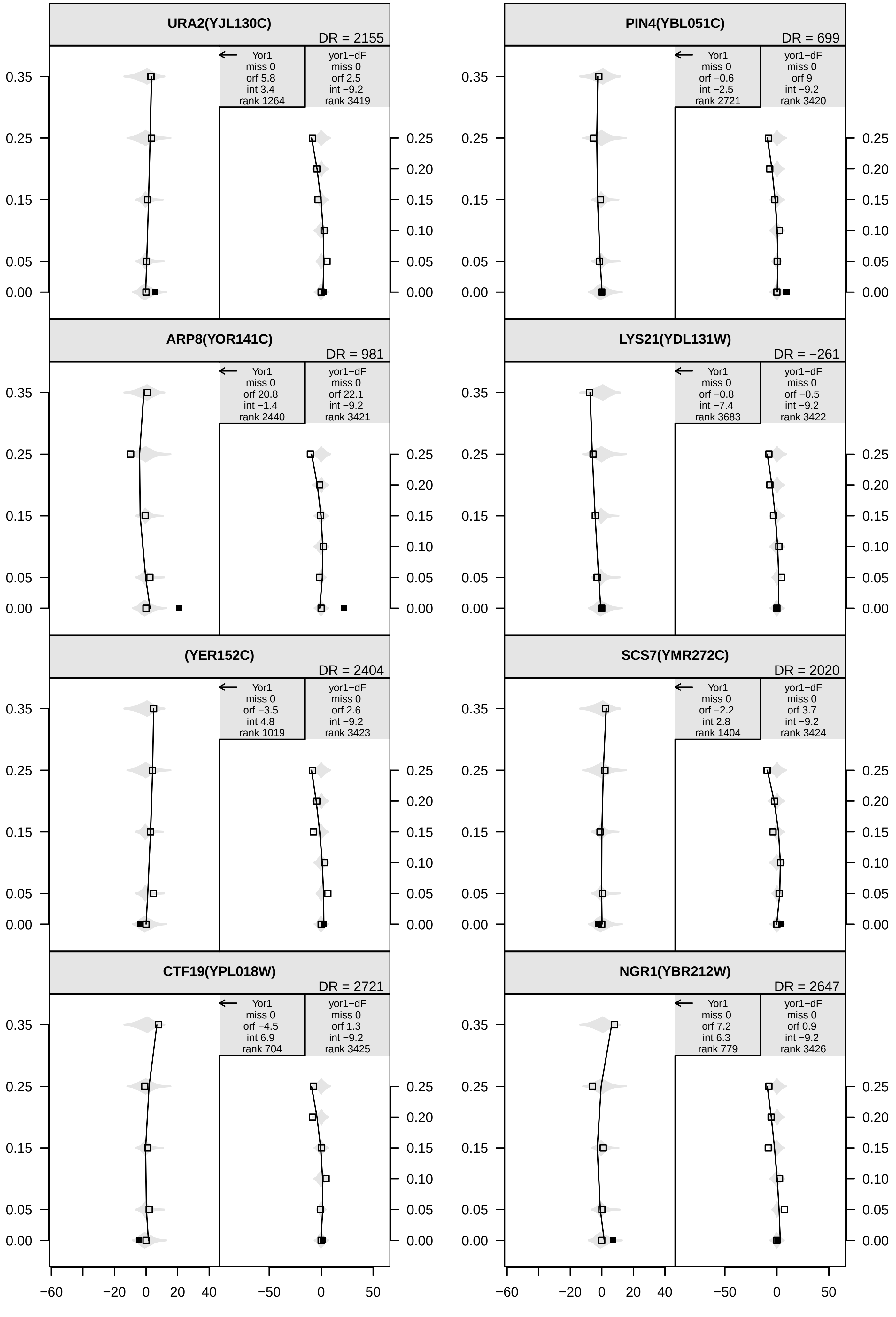

URA2(YJL130C)
DR = 2155
Yor1 miss 0 orf 5.8 int 3.4 rank 1264
yor1−dF miss 0 orf 2.5 int −9.2 rank 3419
PIN4(YBL051C)
DR = 699
Yor1 miss 0 orf −0.6 int −2.5 rank 2721
yor1−dF miss 0 orf 9 int −9.2 rank 3420
ARP8(YOR141C)
DR = 981
Yor1 miss 0 orf 20.8 int −1.4 rank 2440
yor1−dF miss 0 orf 22.1 int −9.2 rank 3421
LYS21(YDL131W)
DR = −261
Yor1 miss 0 orf −0.8 int −7.4 rank 3683
yor1−dF miss 0 orf −0.5 int −9.2 rank 3422
(YER152C)
DR = 2404
Yor1 miss 0 orf −3.5 int 4.8 rank 1019
yor1−dF miss 0 orf 2.6 int −9.2 rank 3423
SCS7(YMR272C)
DR = 2020
Yor1 miss 0 orf −2.2 int 2.8 rank 1404
yor1−dF miss 0 orf 3.7 int −9.2 rank 3424
CTF19(YPL018W)
DR = 2721
Yor1 miss 0 orf −4.5 int 6.9 rank 704
yor1−dF miss 0 orf 1.3 int −9.2 rank 3425
NGR1(YBR212W)
DR = 2647
Yor1 miss 0 orf 7.2 int 6.3 rank 779
yor1−dF miss 0 orf 0.9 int −9.2 rank 3426
0.35 0.25 0.20 0.15 0.10 0.05 0.00
−60 −20 0 20 40 −50 0 50

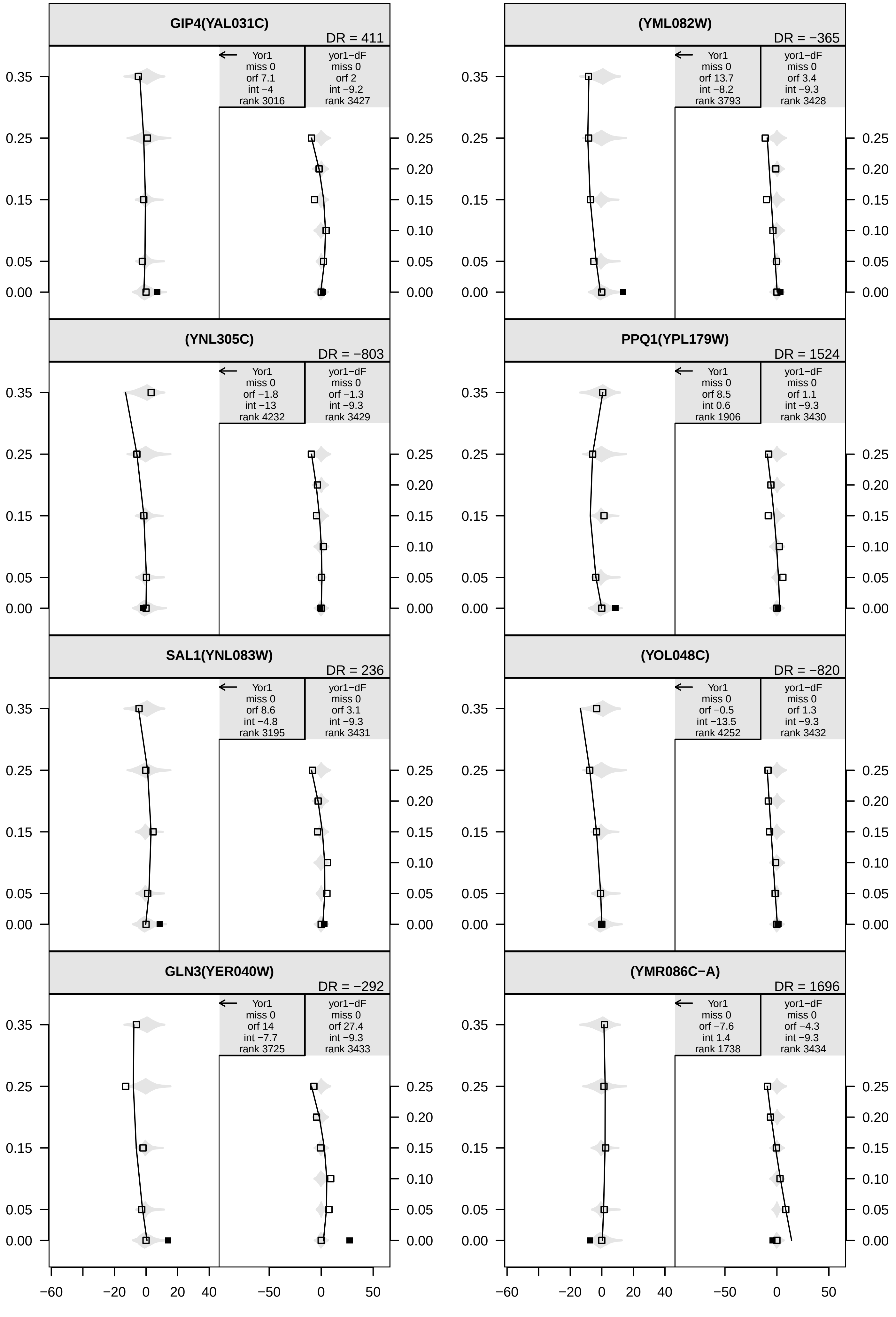
GIP4(YAL031C)
DR = 411
Yor1 miss 0 orf 7.1 int −4 rank 3016
yor1−dF miss 0 orf 2 int −9.2 rank 3427
(YML082W)
DR = −365
Yor1 miss 0 orf 13.7 int −8.2 rank 3793
yor1−dF miss 0 orf 3.4 int −9.3 rank 3428
(YNL305C)
DR = −803
Yor1 miss 0 orf −1.8 int −13 rank 4232
yor1−dF miss 0 orf −1.3 int −9.3 rank 3429
PPQ1(YPL179W)
DR = 1524
Yor1 miss 0 orf 8.5 int 0.6 rank 1906
yor1−dF miss 0 orf 1.1 int −9.3 rank 3430
SAL1(YNL083W)
DR = 236
Yor1 miss 0 orf 8.6 int −4.8 rank 3195
yor1−dF miss 0 orf 3.1 int −9.3 rank 3431
(YOL048C)
DR = −820
Yor1 miss 0 orf −0.5 int −13.5 rank 4252
yor1−dF miss 0 orf 1.3 int −9.3 rank 3432
GLN3(YER040W)
DR = −292
Yor1 miss 0 orf 14 int −7.7 rank 3725
yor1−dF miss 0 orf 27.4 int −9.3 rank 3433
(YMR086C−A)
DR = 1696
Yor1 miss 0 orf −7.6 int 1.4 rank 1738
yor1−dF miss 0 orf −4.3 int −9.3 rank 3434
0.35 0.25 0.20 0.15 0.10 0.05 0.00
−60 −20 0 20 40 −50 0 50

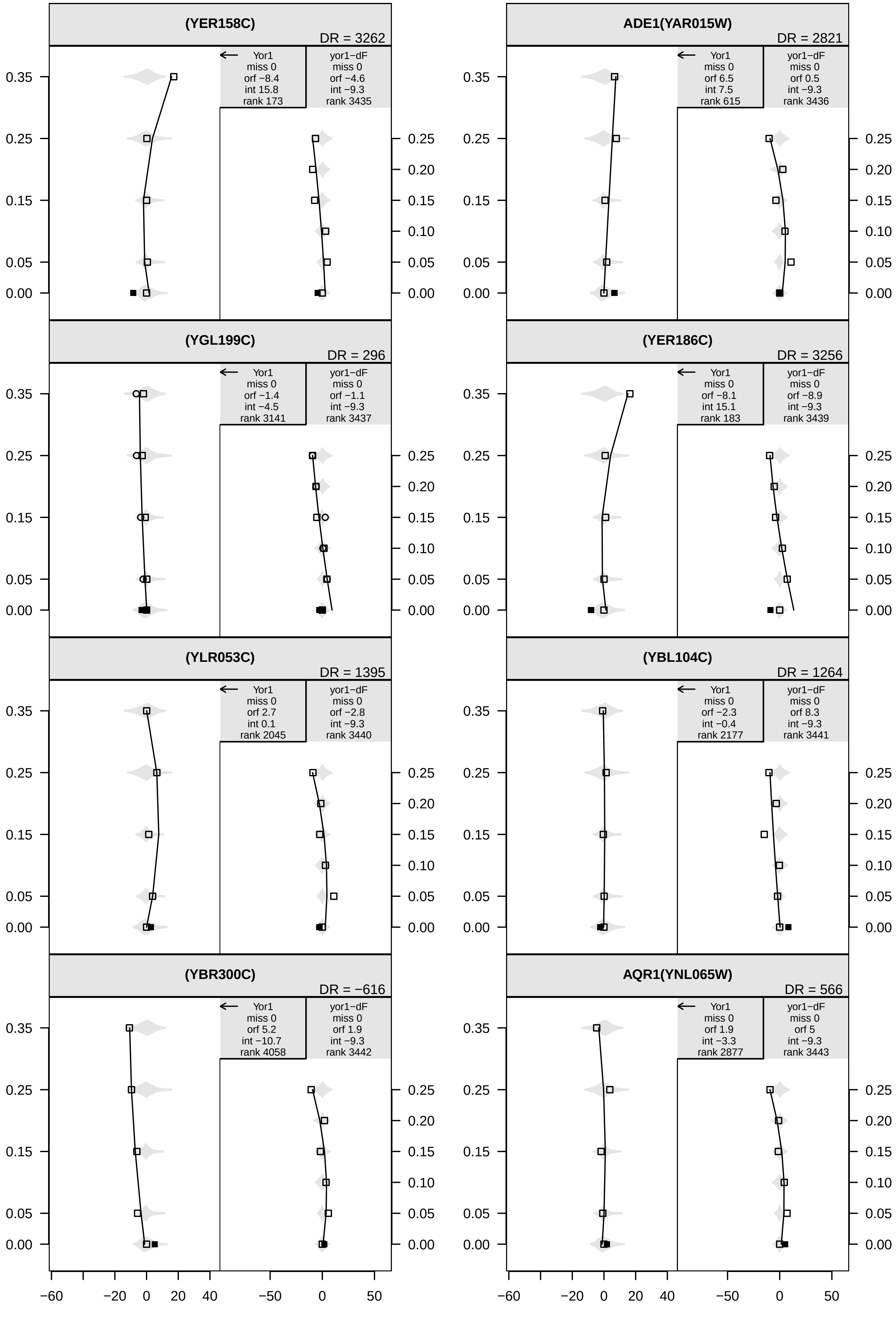
(YER158C)
DR = 3262
Yor1
miss 0
orf −8.4
int 15.8
rank 173
yor1−dF
miss 0
orf −4.6
int −9.3
rank 3435
ADE1(YAR015W)
DR = 2821
Yor1
miss 0
orf 6.5
int 7.5
rank 615
yor1−dF
miss 0
orf 0.5
int −9.3
rank 3436
(YGL199C)
DR = 296
Yor1
miss 0
orf −1.4
int −4.5
rank 3141
yor1−dF
miss 0
orf −1.1
int −9.3
rank 3437
(YER186C)
DR = 3256
Yor1
miss 0
orf −8.1
int 15.1
rank 183
yor1−dF
miss 0
orf −8.9
int −9.3
rank 3439
(YLR053C)
DR = 1395
Yor1
miss 0
orf 2.7
int 0.1
rank 2045
yor1−dF
miss 0
orf −2.8
int −9.3
rank 3440
(YBL104C)
DR = 1264
Yor1
miss 0
orf −2.3
int −0.4
rank 2177
yor1−dF
miss 0
orf 8.3
int −9.3
rank 3441
(YBR300C)
DR = −616
Yor1
miss 0
orf 5.2
int −10.7
rank 4058
yor1−dF
miss 0
orf 1.9
int −9.3
rank 3442
AQR1(YNL065W)
DR = 566
Yor1
miss 0
orf 1.9
int −3.3
rank 2877
yor1−dF
miss 0
orf 5
int −9.3
rank 3443
0.35
0.25
0.20
0.15
0.10
0.05
0.00
−60
−20
0
20
40
−50
0
50

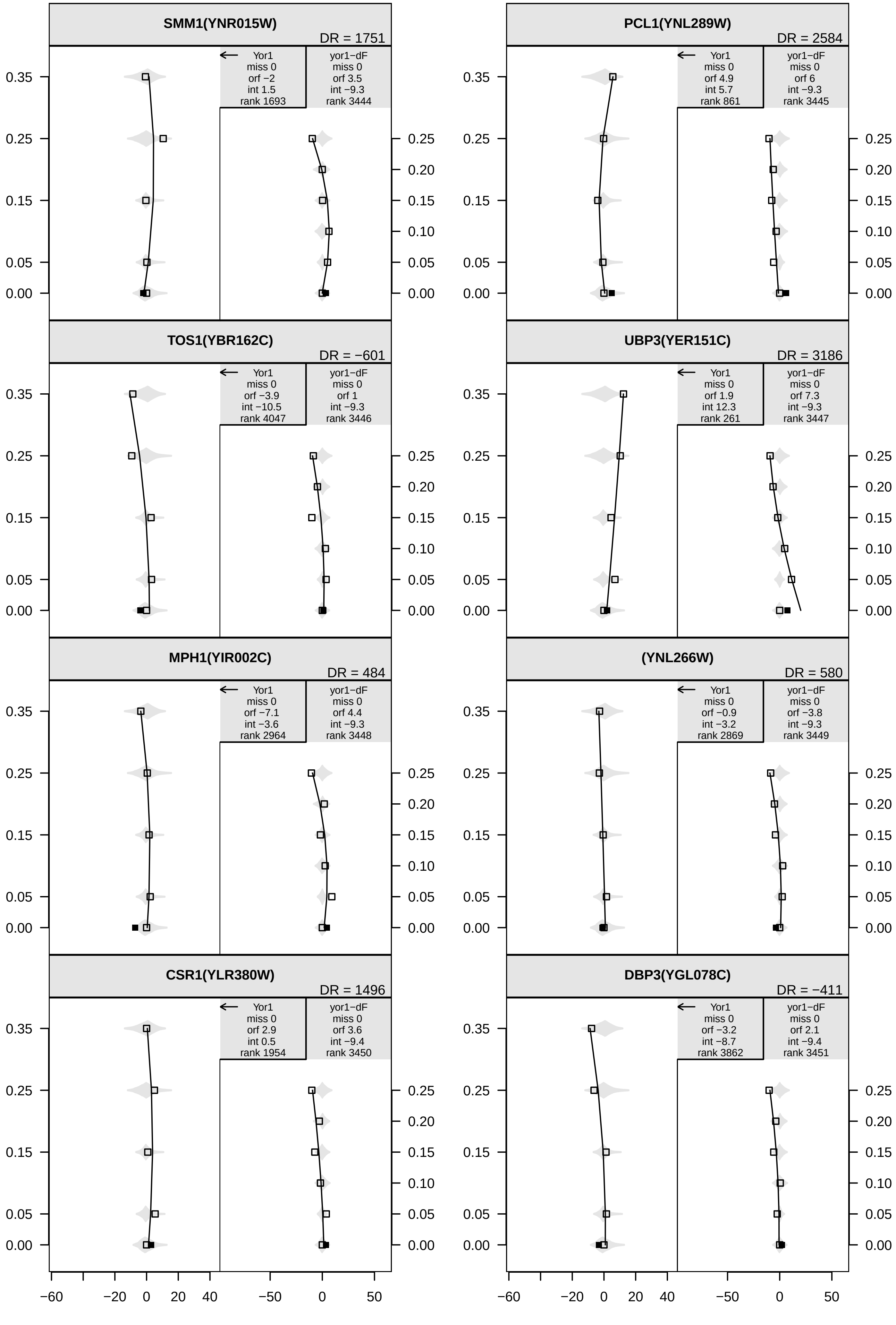
SMM1(YNR015W)
DR = 1751
Yor1 miss 0 orf −2 int 1.5 rank 1693
yor1−dF miss 0 orf 3.5 int −9.3 rank 3444
PCL1(YNL289W)
DR = 2584
Yor1 miss 0 orf 4.9 int 5.7 rank 861
yor1−dF miss 0 orf 6 int −9.3 rank 3445
TOS1(YBR162C)
DR = −601
Yor1 miss 0 orf −3.9 int −10.5 rank 4047
yor1−dF miss 0 orf 1 int −9.3 rank 3446
UBP3(YER151C)
DR = 3186
Yor1 miss 0 orf 1.9 int 12.3 rank 261
yor1−dF miss 0 orf 7.3 int −9.3 rank 3447
MPH1(YIR002C)
DR = 484
Yor1 miss 0 orf −7.1 int −3.6 rank 2964
yor1−dF miss 0 orf 4.4 int −9.3 rank 3448
(YNL266W)
DR = 580
Yor1 miss 0 orf −0.9 int −3.2 rank 2869
yor1−dF miss 0 orf −3.8 int −9.3 rank 3449
CSR1(YLR380W)
DR = 1496
Yor1 miss 0 orf 2.9 int 0.5 rank 1954
yor1−dF miss 0 orf 3.6 int −9.4 rank 3450
DBP3(YGL078C)
DR = −411
Yor1 miss 0 orf −3.2 int −8.7 rank 3862
yor1−dF miss 0 orf 2.1 int −9.4 rank 3451
0.35 0.25 0.20 0.15 0.10 0.05 0.00
−60 −20 0 20 40 −50 0 50

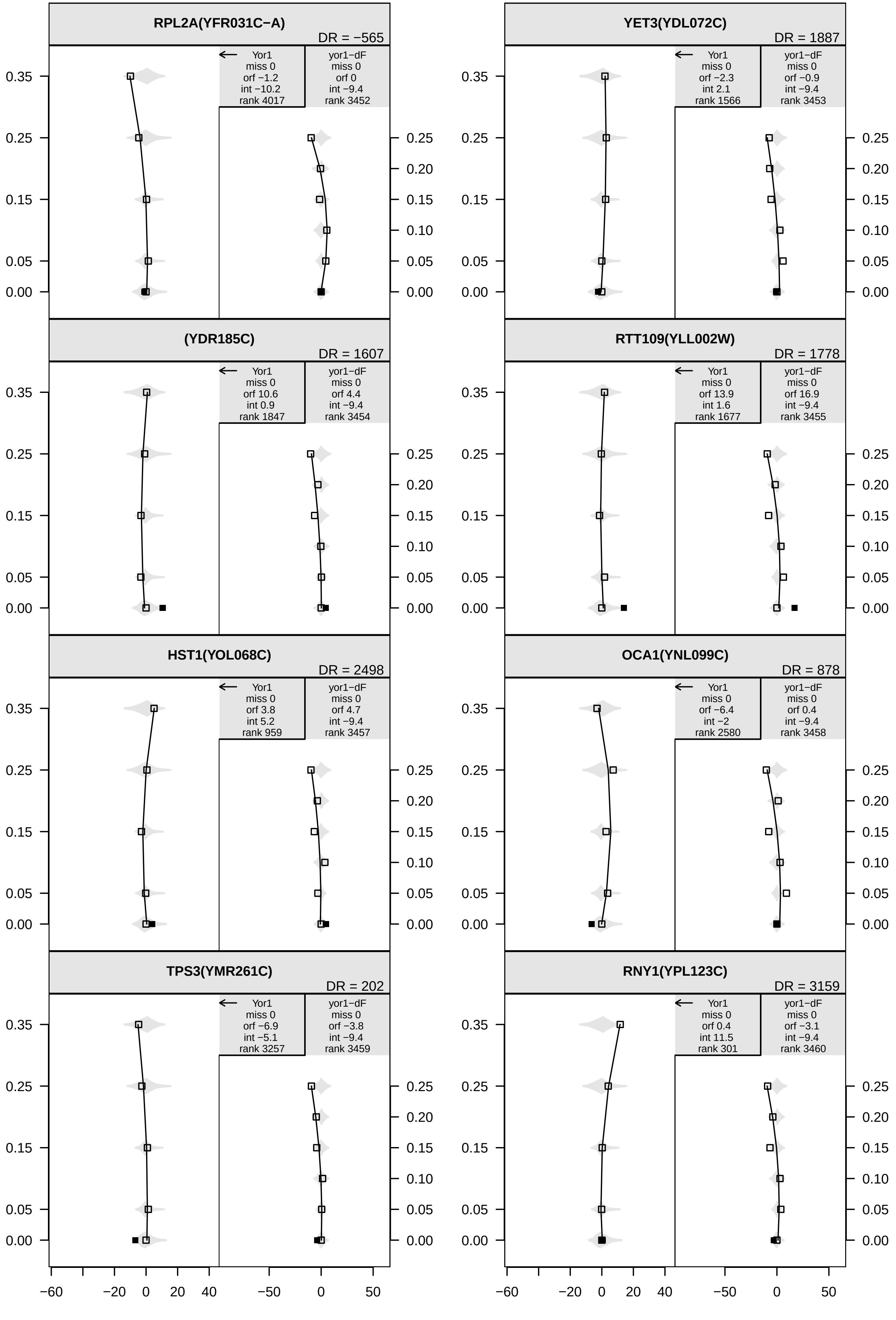
RPL2A(YFR031C−A)
DR = −565
Yor1
miss 0
orf −1.2
int −10.2
rank 4017
yor1−dF
miss 0
orf 0
int −9.4
rank 3452
YET3(YDL072C)
DR = 1887
Yor1
miss 0
orf −2.3
int 2.1
rank 1566
yor1−dF
miss 0
orf −0.9
int −9.4
rank 3453
(YDR185C)
DR = 1607
Yor1
miss 0
orf 10.6
int 0.9
rank 1847
yor1−dF
miss 0
orf 4.4
int −9.4
rank 3454
RTT109(YLL002W)
DR = 1778
Yor1
miss 0
orf 13.9
int 1.6
rank 1677
yor1−dF
miss 0
orf 16.9
int −9.4
rank 3455
HST1(YOL068C)
DR = 2498
Yor1
miss 0
orf 3.8
int 5.2
rank 959
yor1−dF
miss 0
orf 4.7
int −9.4
rank 3457
OCA1(YNL099C)
DR = 878
Yor1
miss 0
orf −6.4
int −2
rank 2580
yor1−dF
miss 0
orf 0.4
int −9.4
rank 3458
TPS3(YMR261C)
DR = 202
Yor1
miss 0
orf −6.9
int −5.1
rank 3257
yor1−dF
miss 0
orf −3.8
int −9.4
rank 3459
RNY1(YPL123C)
DR = 3159
Yor1
miss 0
orf 0.4
int 11.5
rank 301
yor1−dF
miss 0
orf −3.1
int −9.4
rank 3460
0.35
0.25
0.20
0.15
0.10
0.05
0.00
−60
−20
0
20
40
−50
0
50

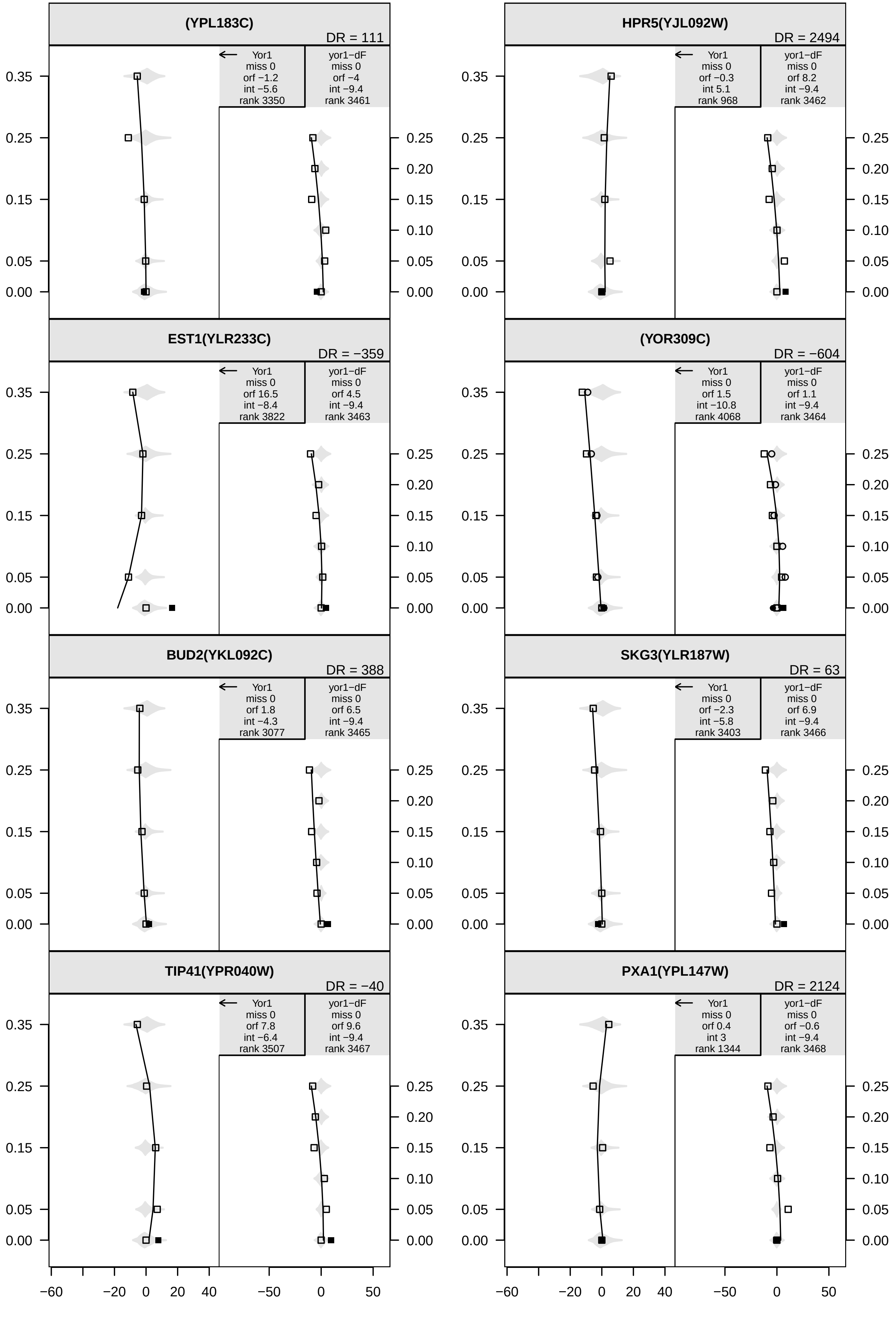

(YPL183C)
DR = 111
Yor1 miss 0 orf −1.2 int −5.6 rank 3350
yor1−dF miss 0 orf −4 int −9.4 rank 3461
HPR5(YJL092W)
DR = 2494
Yor1 miss 0 orf −0.3 int 5.1 rank 968
yor1−dF miss 0 orf 8.2 int −9.4 rank 3462
EST1(YLR233C)
DR = −359
Yor1 miss 0 orf 16.5 int −8.4 rank 3822
yor1−dF miss 0 orf 4.5 int −9.4 rank 3463
(YOR309C)
DR = −604
Yor1 miss 0 orf 1.5 int −10.8 rank 4068
yor1−dF miss 0 orf 1.1 int −9.4 rank 3464
BUD2(YKL092C)
DR = 388
Yor1 miss 0 orf 1.8 int −4.3 rank 3077
yor1−dF miss 0 orf 6.5 int −9.4 rank 3465
SKG3(YLR187W)
DR = 63
Yor1 miss 0 orf −2.3 int −5.8 rank 3403
yor1−dF miss 0 orf 6.9 int −9.4 rank 3466
TIP41(YPR040W)
DR = −40
Yor1 miss 0 orf 7.8 int −6.4 rank 3507
yor1−dF miss 0 orf 9.6 int −9.4 rank 3467
PXA1(YPL147W)
DR = 2124
Yor1 miss 0 orf 0.4 int 3 rank 1344
yor1−dF miss 0 orf −0.6 int −9.4 rank 3468
0.35 0.25 0.15 0.05 0.00
0.25 0.20 0.15 0.10 0.05 0.00
−60 −20 0 20 40
−50 0 50

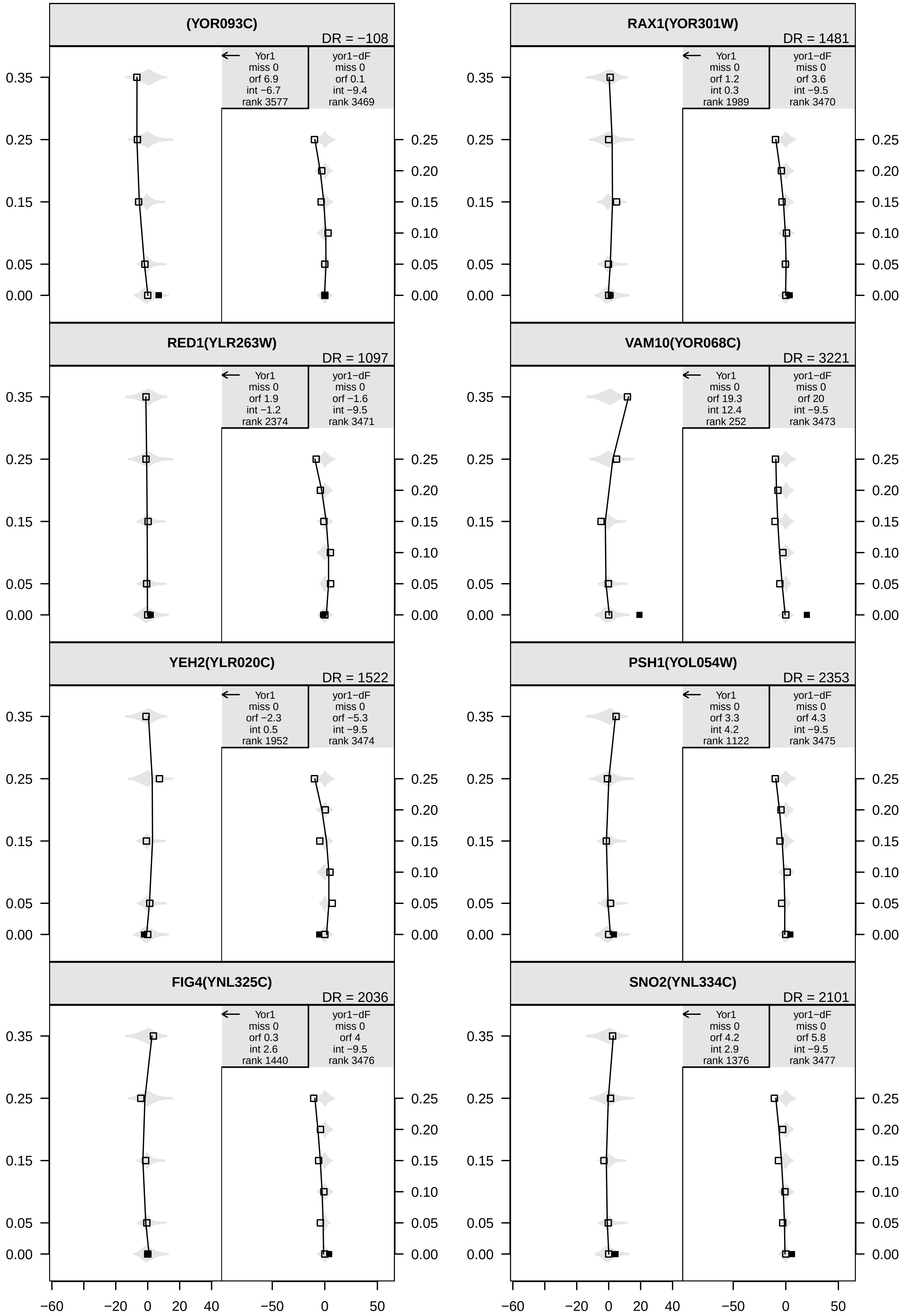

(YOR093C)
DR = −108
Yor1
miss 0
orf 6.9
int −6.7
rank 3577
yor1−dF
miss 0
orf 0.1
int −9.4
rank 3469
RAX1(YOR301W)
DR = 1481
Yor1
miss 0
orf 1.2
int 0.3
rank 1989
yor1−dF
miss 0
orf 3.6
int −9.5
rank 3470
RED1(YLR263W)
DR = 1097
Yor1
miss 0
orf 1.9
int −1.2
rank 2374
yor1−dF
miss 0
orf −1.6
int −9.5
rank 3471
VAM10(YOR068C)
DR = 3221
Yor1
miss 0
orf 19.3
int 12.4
rank 252
yor1−dF
miss 0
orf 20
int −9.5
rank 3473
YEH2(YLR020C)
DR = 1522
Yor1
miss 0
orf −2.3
int 0.5
rank 1952
yor1−dF
miss 0
orf −5.3
int −9.5
rank 3474
PSH1(YOL054W)
DR = 2353
Yor1
miss 0
orf 3.3
int 4.2
rank 1122
yor1−dF
miss 0
orf 4.3
int −9.5
rank 3475
FIG4(YNL325C)
DR = 2036
Yor1
miss 0
orf 0.3
int 2.6
rank 1440
yor1−dF
miss 0
orf 4
int −9.5
rank 3476
SNO2(YNL334C)
DR = 2101
Yor1
miss 0
orf 4.2
int 2.9
rank 1376
yor1−dF
miss 0
orf 5.8
int −9.5
rank 3477
0.35
0.25
0.20
0.15
0.10
0.05
0.00
−60
−20
0
20
40
−50
0
50

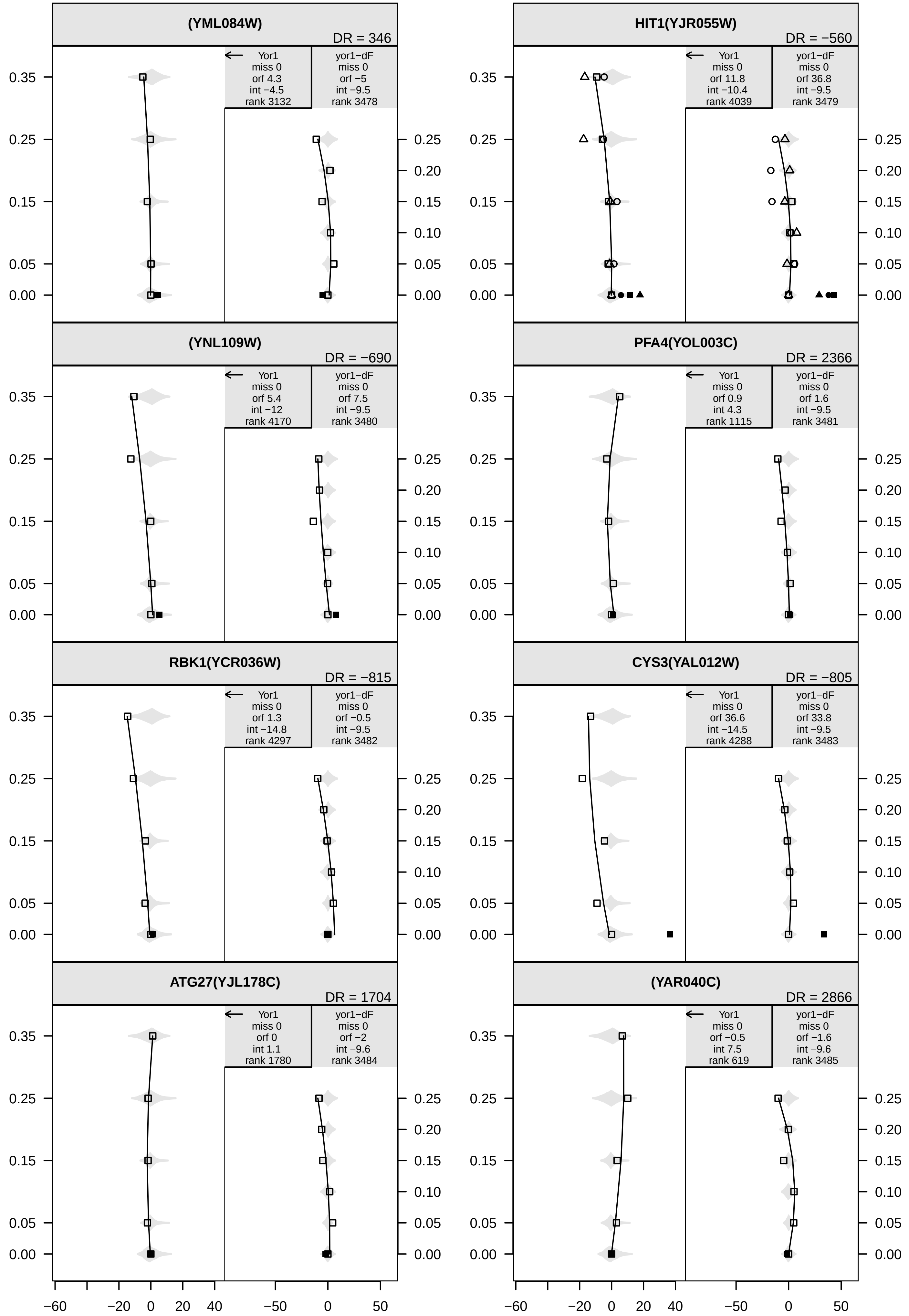

(YML084W)
DR = 346
Yor1 miss 0 orf 4.3 int −4.5 rank 3132
yor1−dF miss 0 orf −5 int −9.5 rank 3478
HIT1(YJR055W)
DR = −560
Yor1 miss 0 orf 11.8 int −10.4 rank 4039
yor1−dF miss 0 orf 36.8 int −9.5 rank 3479
(YNL109W)
DR = −690
Yor1 miss 0 orf 5.4 int −12 rank 4170
yor1−dF miss 0 orf 7.5 int −9.5 rank 3480
PFA4(YOL003C)
DR = 2366
Yor1 miss 0 orf 0.9 int 4.3 rank 1115
yor1−dF miss 0 orf 1.6 int −9.5 rank 3481
RBK1(YCR036W)
DR = −815
Yor1 miss 0 orf 1.3 int −14.8 rank 4297
yor1−dF miss 0 orf −0.5 int −9.5 rank 3482
CYS3(YAL012W)
DR = −805
Yor1 miss 0 orf 36.6 int −14.5 rank 4288
yor1−dF miss 0 orf 33.8 int −9.5 rank 3483
ATG27(YJL178C)
DR = 1704
Yor1 miss 0 orf 0 int 1.1 rank 1780
yor1−dF miss 0 orf −2 int −9.6 rank 3484
(YAR040C)
DR = 2866
Yor1 miss 0 orf −0.5 int 7.5 rank 619
yor1−dF miss 0 orf −1.6 int −9.6 rank 3485
0.00 0.05 0.10 0.15 0.20 0.25 0.35
−60 −20 0 20 40
−50 0 50

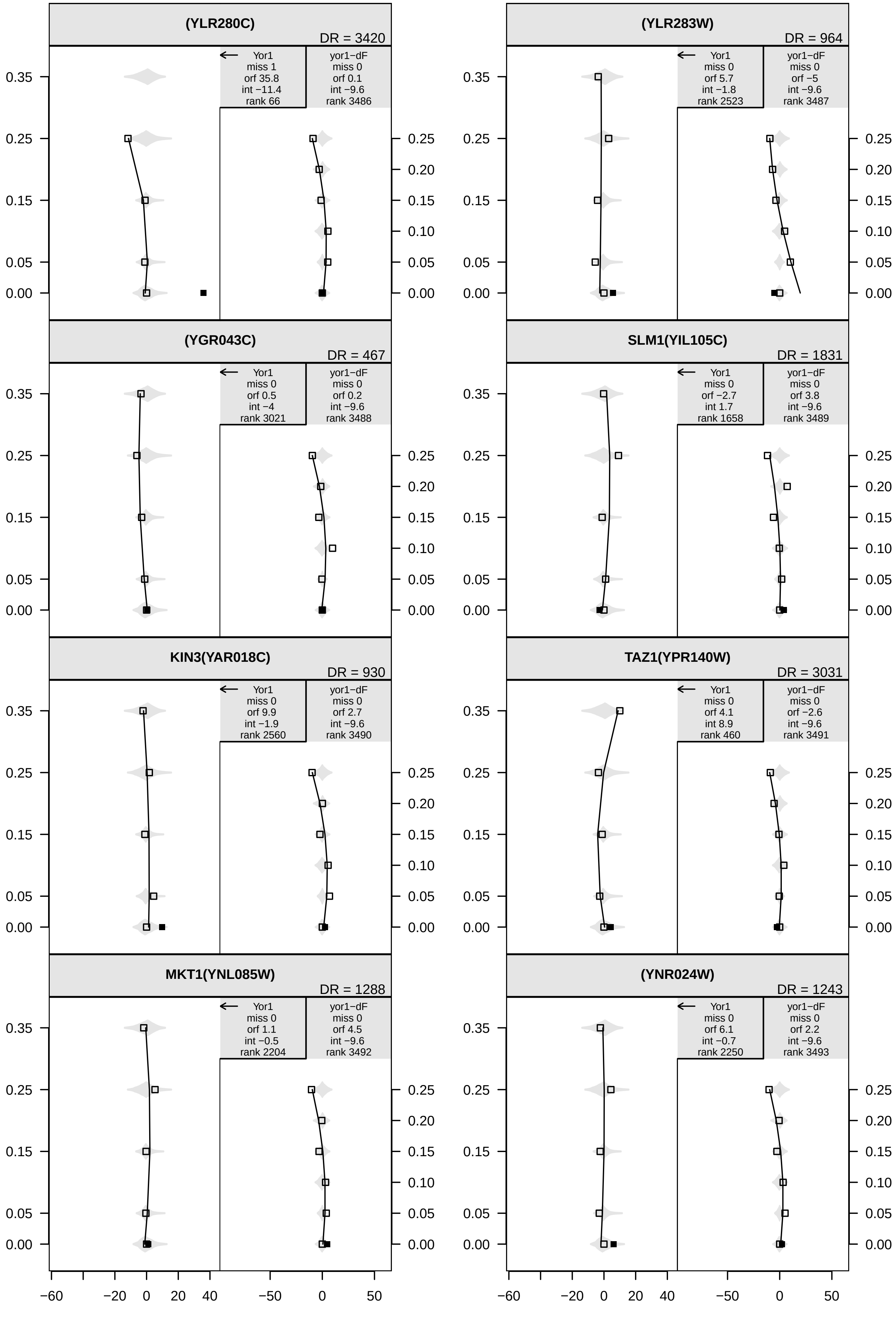
(YLR280C)
DR = 3420
Yor1
miss 1
orf 35.8
int −11.4
rank 66
yor1−dF
miss 0
orf 0.1
int −9.6
rank 3486
(YLR283W)
DR = 964
Yor1
miss 0
orf 5.7
int −1.8
rank 2523
yor1−dF
miss 0
orf −5
int −9.6
rank 3487
(YGR043C)
DR = 467
Yor1
miss 0
orf 0.5
int −4
rank 3021
yor1−dF
miss 0
orf 0.2
int −9.6
rank 3488
SLM1(YIL105C)
DR = 1831
Yor1
miss 0
orf −2.7
int 1.7
rank 1658
yor1−dF
miss 0
orf 3.8
int −9.6
rank 3489
KIN3(YAR018C)
DR = 930
Yor1
miss 0
orf 9.9
int −1.9
rank 2560
yor1−dF
miss 0
orf 2.7
int −9.6
rank 3490
TAZ1(YPR140W)
DR = 3031
Yor1
miss 0
orf 4.1
int 8.9
rank 460
yor1−dF
miss 0
orf −2.6
int −9.6
rank 3491
MKT1(YNL085W)
DR = 1288
Yor1
miss 0
orf 1.1
int −0.5
rank 2204
yor1−dF
miss 0
orf 4.5
int −9.6
rank 3492
(YNR024W)
DR = 1243
Yor1
miss 0
orf 6.1
int −0.7
rank 2250
yor1−dF
miss 0
orf 2.2
int −9.6
rank 3493
0.35
0.25
0.20
0.15
0.10
0.05
0.00
−60
−20
0
20
40
−50
0
50

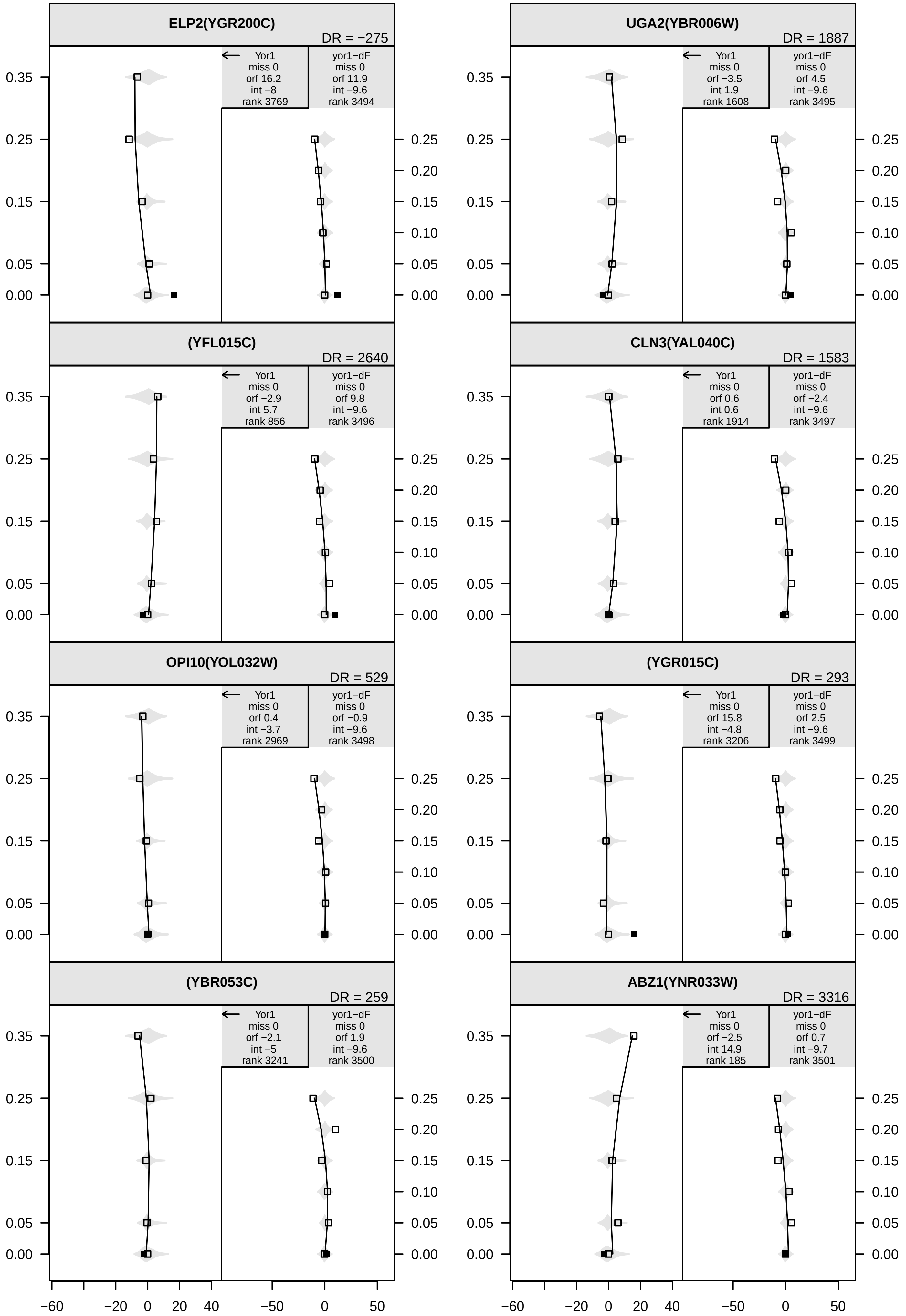
ELP2(YGR200C)
DR = −275
Yor1
miss 0
orf 16.2
int −8
rank 3769
yor1−dF
miss 0
orf 11.9
int −9.6
rank 3494
UGA2(YBR006W)
DR = 1887
Yor1
miss 0
orf −3.5
int 1.9
rank 1608
yor1−dF
miss 0
orf 4.5
int −9.6
rank 3495
(YFL015C)
DR = 2640
Yor1
miss 0
orf −2.9
int 5.7
rank 856
yor1−dF
miss 0
orf 9.8
int −9.6
rank 3496
CLN3(YAL040C)
DR = 1583
Yor1
miss 0
orf 0.6
int 0.6
rank 1914
yor1−dF
miss 0
orf −2.4
int −9.6
rank 3497
OPI10(YOL032W)
DR = 529
Yor1
miss 0
orf 0.4
int −3.7
rank 2969
yor1−dF
miss 0
orf −0.9
int −9.6
rank 3498
(YGR015C)
DR = 293
Yor1
miss 0
orf 15.8
int −4.8
rank 3206
yor1−dF
miss 0
orf 2.5
int −9.6
rank 3499
(YBR053C)
DR = 259
Yor1
miss 0
orf −2.1
int −5
rank 3241
yor1−dF
miss 0
orf 1.9
int −9.6
rank 3500
ABZ1(YNR033W)
DR = 3316
Yor1
miss 0
orf −2.5
int 14.9
rank 185
yor1−dF
miss 0
orf 0.7
int −9.7
rank 3501
0.35
0.25
0.20
0.15
0.10
0.05
0.00
−60
−20
0
20
40
−50
0
50

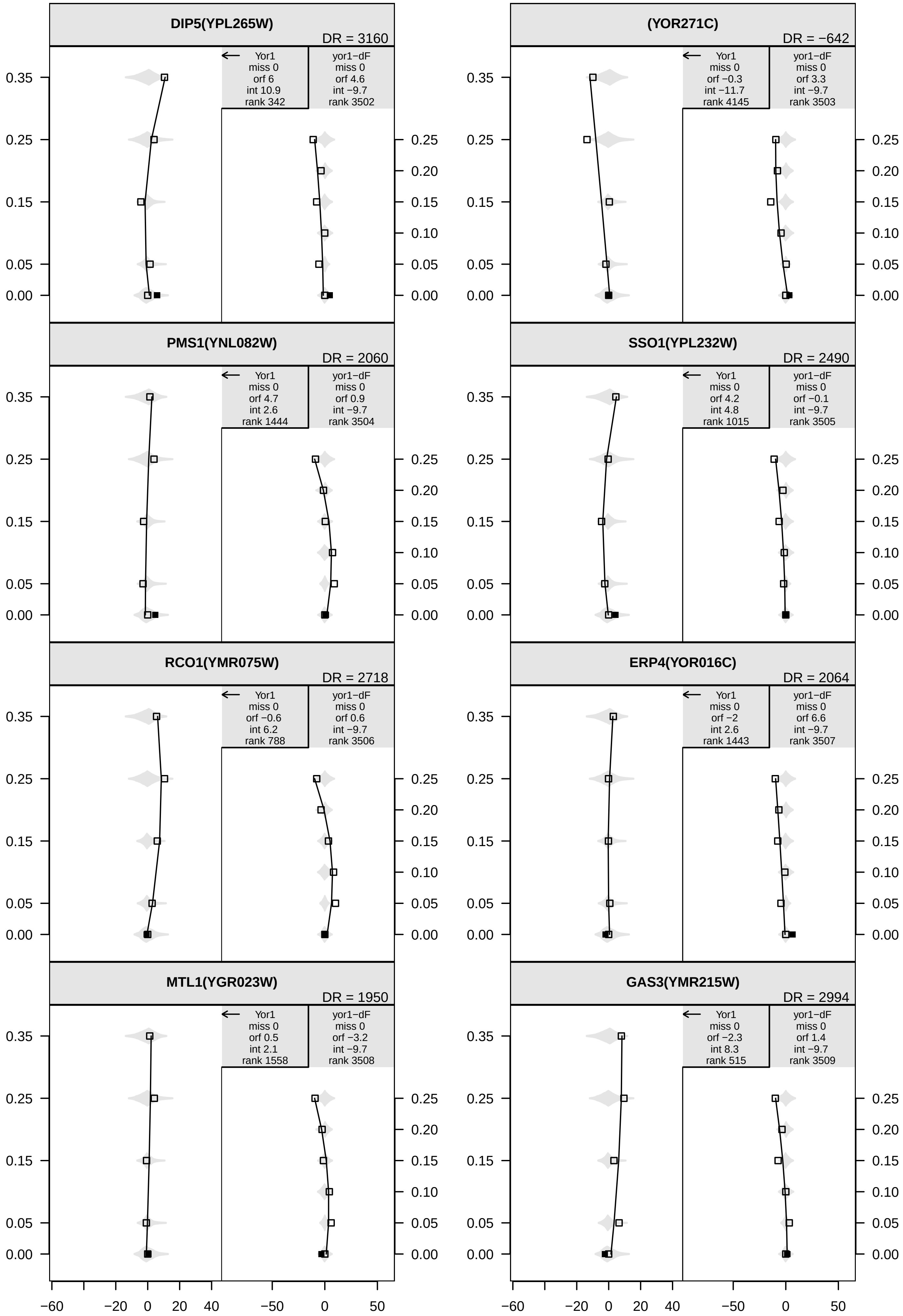
DIP5(YPL265W)
DR = 3160
Yor1 miss 0 orf 6 int 10.9 rank 342
yor1−dF miss 0 orf 4.6 int −9.7 rank 3502
(YOR271C)
DR = −642
Yor1 miss 0 orf −0.3 int −11.7 rank 4145
yor1−dF miss 0 orf 3.3 int −9.7 rank 3503
PMS1(YNL082W)
DR = 2060
Yor1 miss 0 orf 4.7 int 2.6 rank 1444
yor1−dF miss 0 orf 0.9 int −9.7 rank 3504
SSO1(YPL232W)
DR = 2490
Yor1 miss 0 orf 4.2 int 4.8 rank 1015
yor1−dF miss 0 orf −0.1 int −9.7 rank 3505
RCO1(YMR075W)
DR = 2718
Yor1 miss 0 orf −0.6 int 6.2 rank 788
yor1−dF miss 0 orf 0.6 int −9.7 rank 3506
ERP4(YOR016C)
DR = 2064
Yor1 miss 0 orf −2 int 2.6 rank 1443
yor1−dF miss 0 orf 6.6 int −9.7 rank 3507
MTL1(YGR023W)
DR = 1950
Yor1 miss 0 orf 0.5 int 2.1 rank 1558
yor1−dF miss 0 orf −3.2 int −9.7 rank 3508
GAS3(YMR215W)
DR = 2994
Yor1 miss 0 orf −2.3 int 8.3 rank 515
yor1−dF miss 0 orf 1.4 int −9.7 rank 3509
0.35
0.25
0.20
0.15
0.10
0.05
0.00
−60 −20 0 20 40
−50 0 50

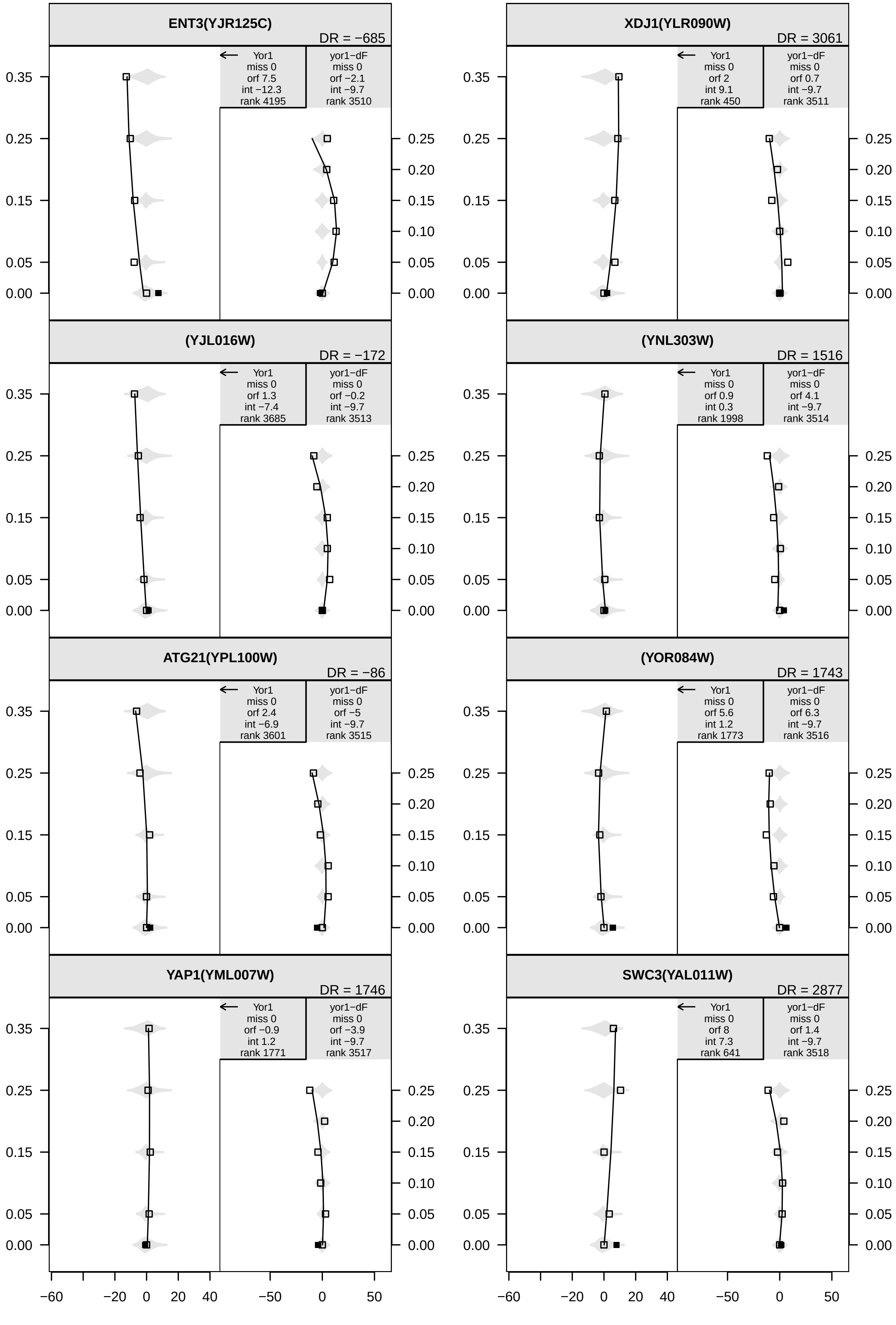
ENT3(YJR125C)
DR = −685
Yor1 miss 0 orf 7.5 int −12.3 rank 4195
yor1−dF miss 0 orf −2.1 int −9.7 rank 3510
XDJ1(YLR090W)
DR = 3061
Yor1 miss 0 orf 2 int 9.1 rank 450
yor1−dF miss 0 orf 0.7 int −9.7 rank 3511
(YJL016W)
DR = −172
Yor1 miss 0 orf 1.3 int −7.4 rank 3685
yor1−dF miss 0 orf −0.2 int −9.7 rank 3513
(YNL303W)
DR = 1516
Yor1 miss 0 orf 0.9 int 0.3 rank 1998
yor1−dF miss 0 orf 4.1 int −9.7 rank 3514
ATG21(YPL100W)
DR = −86
Yor1 miss 0 orf 2.4 int −6.9 rank 3601
yor1−dF miss 0 orf −5 int −9.7 rank 3515
(YOR084W)
DR = 1743
Yor1 miss 0 orf 5.6 int 1.2 rank 1773
yor1−dF miss 0 orf 6.3 int −9.7 rank 3516
YAP1(YML007W)
DR = 1746
Yor1 miss 0 orf −0.9 int 1.2 rank 1771
yor1−dF miss 0 orf −3.9 int −9.7 rank 3517
SWC3(YAL011W)
DR = 2877
Yor1 miss 0 orf 8 int 7.3 rank 641
yor1−dF miss 0 orf 1.4 int −9.7 rank 3518
0.00 0.05 0.10 0.15 0.20 0.25 0.35
−60 −20 0 20 40 −50 0 50

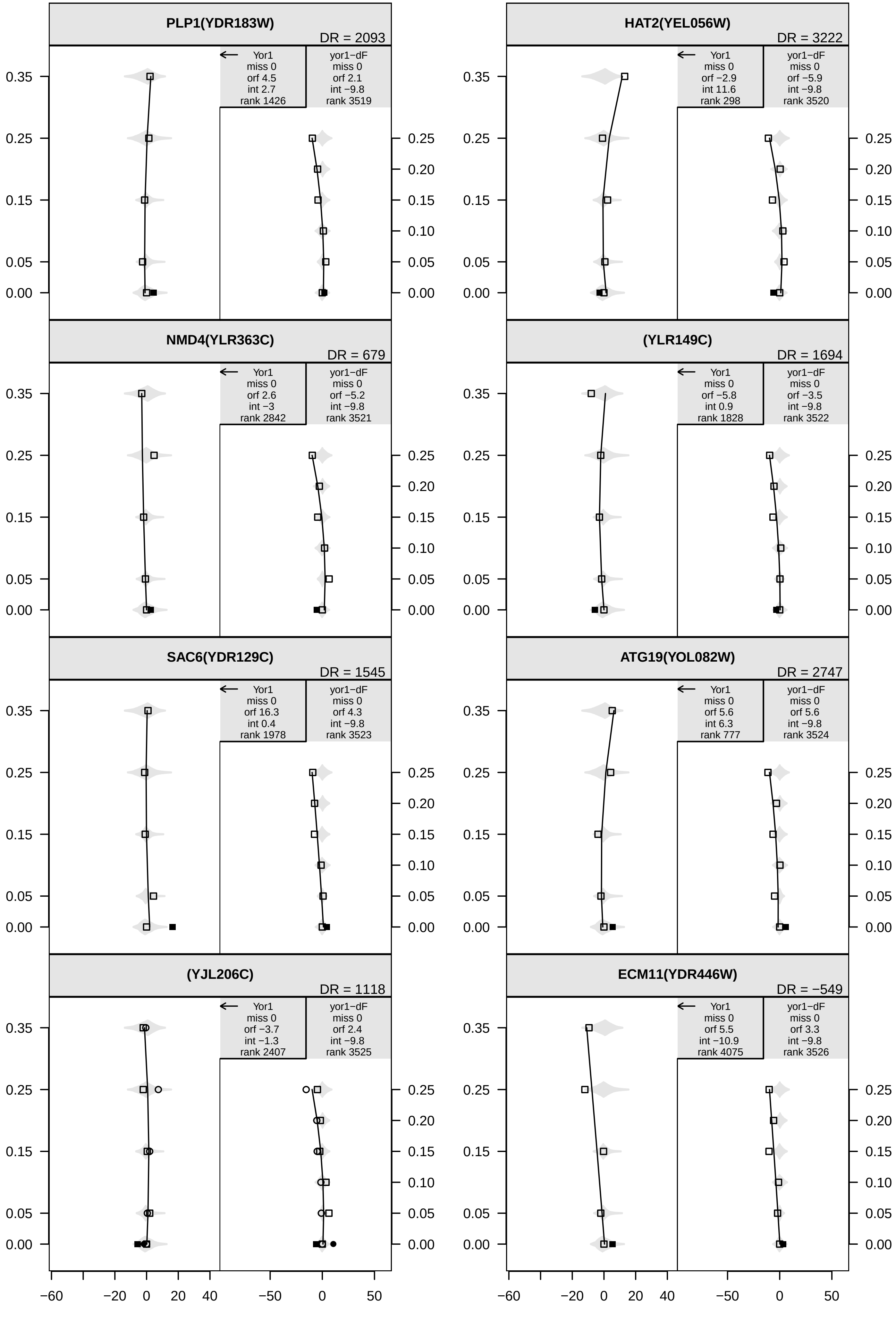

PLP1(YDR183W)
DR = 2093
Yor1
miss 0
orf 4.5
int 2.7
rank 1426
yor1−dF
miss 0
orf 2.1
int −9.8
rank 3519
HAT2(YEL056W)
DR = 3222
Yor1
miss 0
orf −2.9
int 11.6
rank 298
yor1−dF
miss 0
orf −5.9
int −9.8
rank 3520
NMD4(YLR363C)
DR = 679
Yor1
miss 0
orf 2.6
int −3
rank 2842
yor1−dF
miss 0
orf −5.2
int −9.8
rank 3521
(YLR149C)
DR = 1694
Yor1
miss 0
orf −5.8
int 0.9
rank 1828
yor1−dF
miss 0
orf −3.5
int −9.8
rank 3522
SAC6(YDR129C)
DR = 1545
Yor1
miss 0
orf 16.3
int 0.4
rank 1978
yor1−dF
miss 0
orf 4.3
int −9.8
rank 3523
ATG19(YOL082W)
DR = 2747
Yor1
miss 0
orf 5.6
int 6.3
rank 777
yor1−dF
miss 0
orf 5.6
int −9.8
rank 3524
(YJL206C)
DR = 1118
Yor1
miss 0
orf −3.7
int −1.3
rank 2407
yor1−dF
miss 0
orf 2.4
int −9.8
rank 3525
ECM11(YDR446W)
DR = −549
Yor1
miss 0
orf 5.5
int −10.9
rank 4075
yor1−dF
miss 0
orf 3.3
int −9.8
rank 3526
0.00 0.05 0.10 0.15 0.20 0.25 0.35
−60 −20 0 20 40 −50 0 50

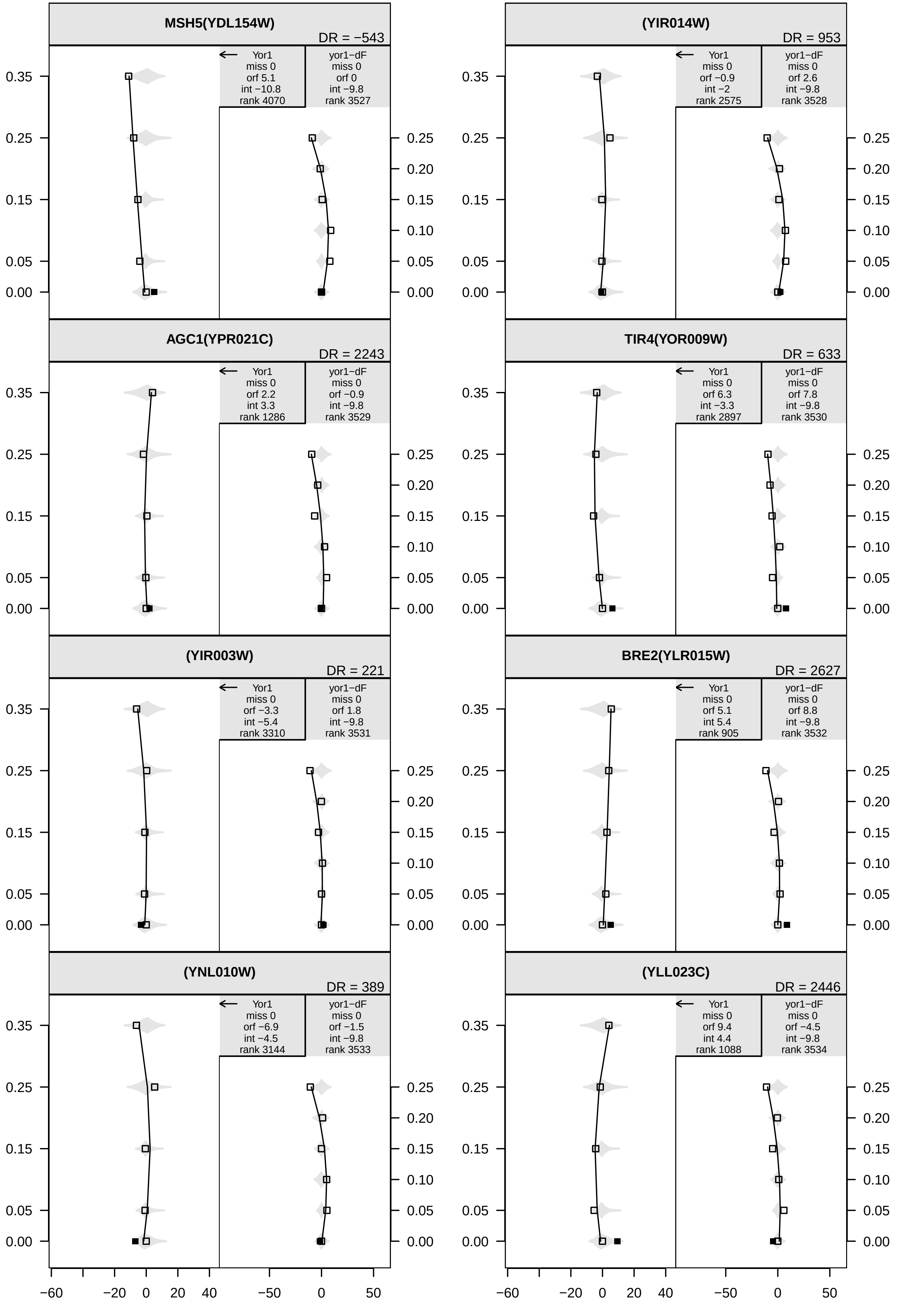

MSH5(YDL154W)
DR = −543
Yor1 miss 0 orf 5.1 int −10.8 rank 4070
yor1−dF miss 0 orf 0 int −9.8 rank 3527
(YIR014W)
DR = 953
Yor1 miss 0 orf −0.9 int −2 rank 2575
yor1−dF miss 0 orf 2.6 int −9.8 rank 3528
AGC1(YPR021C)
DR = 2243
Yor1 miss 0 orf 2.2 int 3.3 rank 1286
yor1−dF miss 0 orf −0.9 int −9.8 rank 3529
TIR4(YOR009W)
DR = 633
Yor1 miss 0 orf 6.3 int −3.3 rank 2897
yor1−dF miss 0 orf 7.8 int −9.8 rank 3530
(YIR003W)
DR = 221
Yor1 miss 0 orf −3.3 int −5.4 rank 3310
yor1−dF miss 0 orf 1.8 int −9.8 rank 3531
BRE2(YLR015W)
DR = 2627
Yor1 miss 0 orf 5.1 int 5.4 rank 905
yor1−dF miss 0 orf 8.8 int −9.8 rank 3532
(YNL010W)
DR = 389
Yor1 miss 0 orf −6.9 int −4.5 rank 3144
yor1−dF miss 0 orf −1.5 int −9.8 rank 3533
(YLL023C)
DR = 2446
Yor1 miss 0 orf 9.4 int 4.4 rank 1088
yor1−dF miss 0 orf −4.5 int −9.8 rank 3534
0.35
0.25
0.20
0.15
0.10
0.05
0.00
−60
−20
0
20
40
−50
0
50

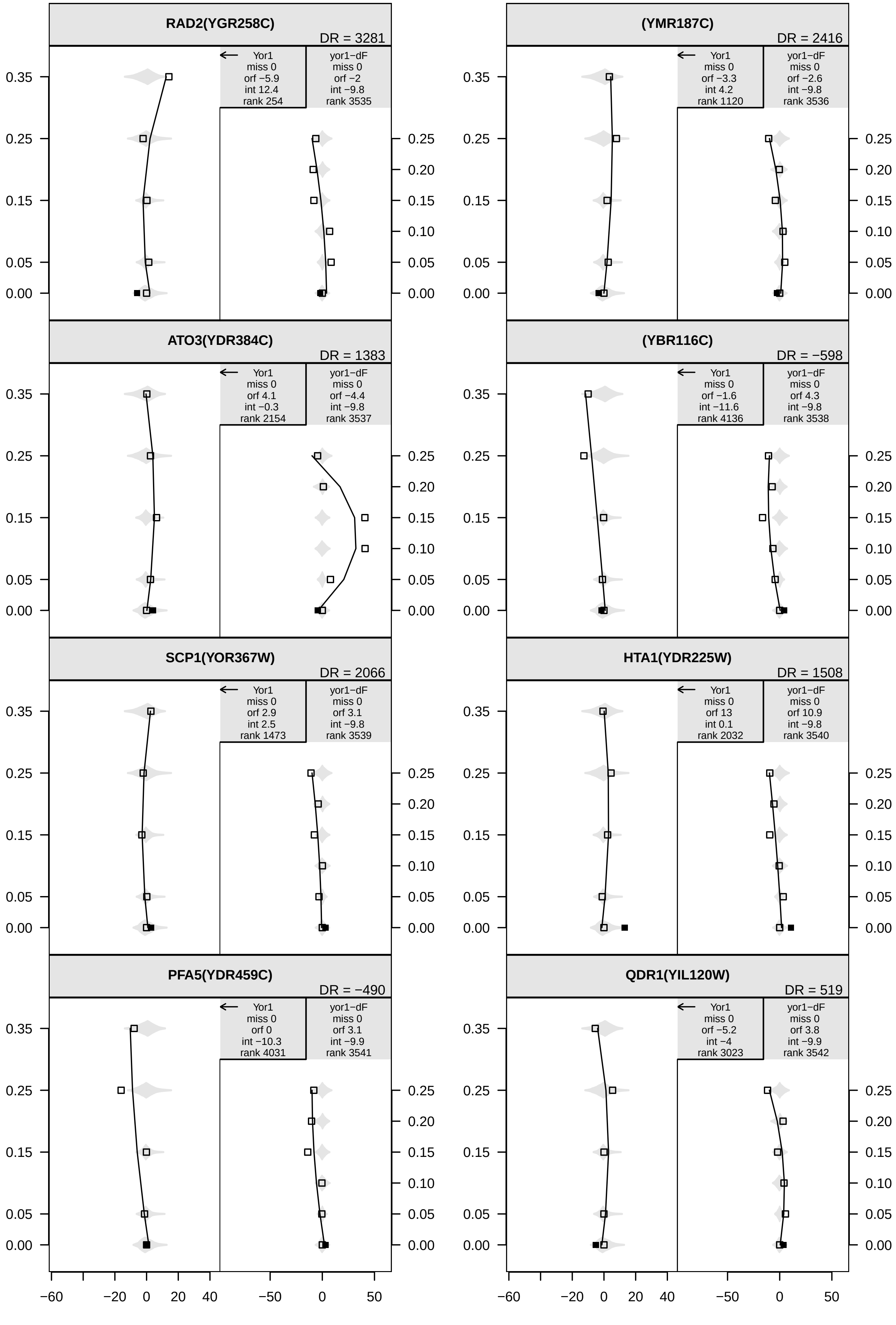
RAD2(YGR258C)
DR = 3281
Yor1 miss 0 orf −5.9 int 12.4 rank 254
yor1−dF miss 0 orf −2 int −9.8 rank 3535
(YMR187C)
DR = 2416
Yor1 miss 0 orf −3.3 int 4.2 rank 1120
yor1−dF miss 0 orf −2.6 int −9.8 rank 3536
ATO3(YDR384C)
DR = 1383
Yor1 miss 0 orf 4.1 int −0.3 rank 2154
yor1−dF miss 0 orf −4.4 int −9.8 rank 3537
(YBR116C)
DR = −598
Yor1 miss 0 orf −1.6 int −11.6 rank 4136
yor1−dF miss 0 orf 4.3 int −9.8 rank 3538
SCP1(YOR367W)
DR = 2066
Yor1 miss 0 orf 2.9 int 2.5 rank 1473
yor1−dF miss 0 orf 3.1 int −9.8 rank 3539
HTA1(YDR225W)
DR = 1508
Yor1 miss 0 orf 13 int 0.1 rank 2032
yor1−dF miss 0 orf 10.9 int −9.8 rank 3540
PFA5(YDR459C)
DR = −490
Yor1 miss 0 orf 0 int −10.3 rank 4031
yor1−dF miss 0 orf 3.1 int −9.9 rank 3541
QDR1(YIL120W)
DR = 519
Yor1 miss 0 orf −5.2 int −4 rank 3023
yor1−dF miss 0 orf 3.8 int −9.9 rank 3542
0.00 0.05 0.10 0.15 0.20 0.25 0.35
−60 −20 0 20 40 −50 0 50

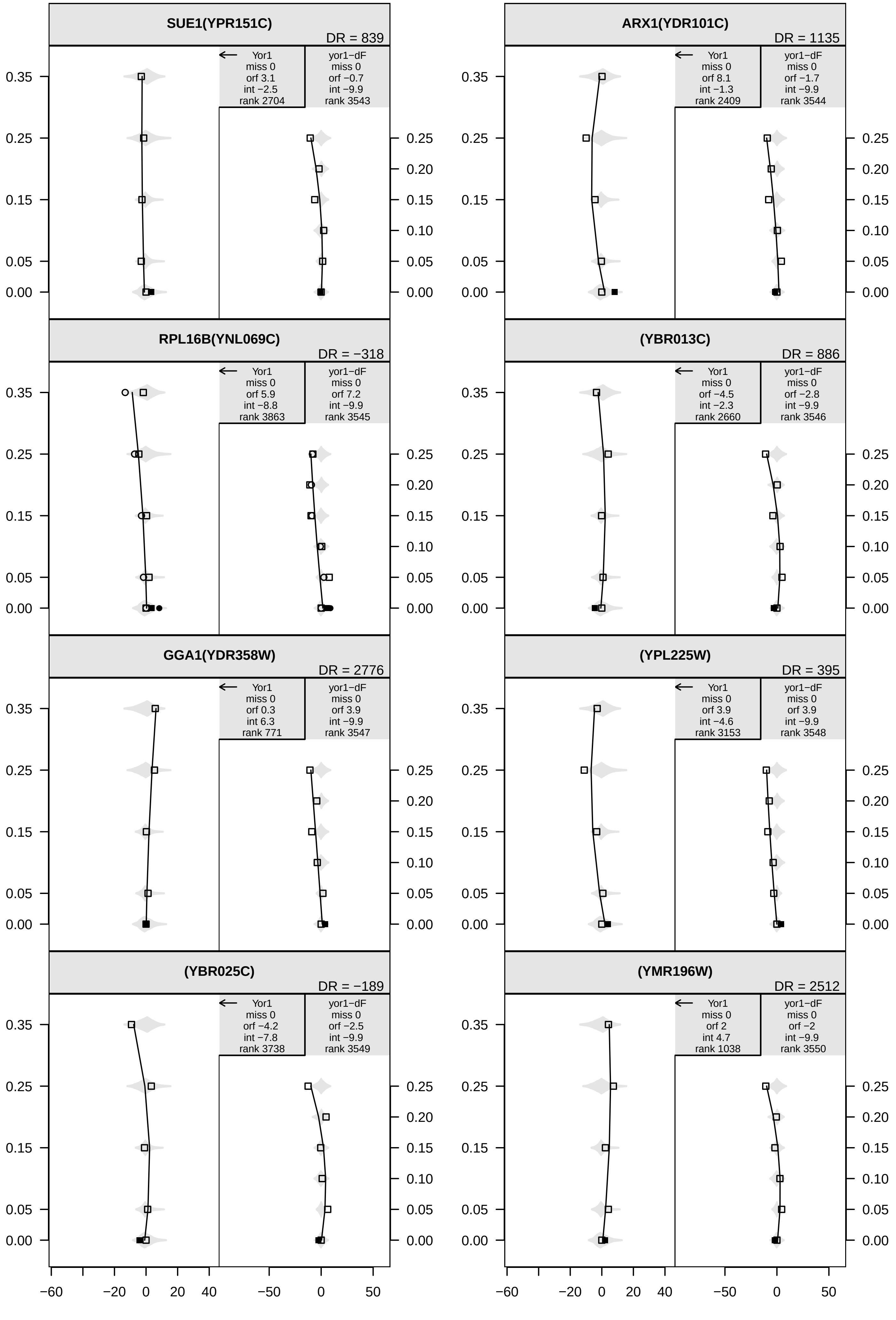

SUE1(YPR151C)
DR = 839
Yor1 miss 0 orf 3.1 int −2.5 rank 2704
yor1−dF miss 0 orf −0.7 int −9.9 rank 3543
ARX1(YDR101C)
DR = 1135
Yor1 miss 0 orf 8.1 int −1.3 rank 2409
yor1−dF miss 0 orf −1.7 int −9.9 rank 3544
RPL16B(YNL069C)
DR = −318
Yor1 miss 0 orf 5.9 int −8.8 rank 3863
yor1−dF miss 0 orf 7.2 int −9.9 rank 3545
(YBR013C)
DR = 886
Yor1 miss 0 orf −4.5 int −2.3 rank 2660
yor1−dF miss 0 orf −2.8 int −9.9 rank 3546
GGA1(YDR358W)
DR = 2776
Yor1 miss 0 orf 0.3 int 6.3 rank 771
yor1−dF miss 0 orf 3.9 int −9.9 rank 3547
(YPL225W)
DR = 395
Yor1 miss 0 orf 3.9 int −4.6 rank 3153
yor1−dF miss 0 orf 3.9 int −9.9 rank 3548
(YBR025C)
DR = −189
Yor1 miss 0 orf −4.2 int −7.8 rank 3738
yor1−dF miss 0 orf −2.5 int −9.9 rank 3549
(YMR196W)
DR = 2512
Yor1 miss 0 orf 2 int 4.7 rank 1038
yor1−dF miss 0 orf −2 int −9.9 rank 3550
0.00 0.05 0.10 0.15 0.20 0.25 0.35
−60 −20 0 20 40 −50 0 50

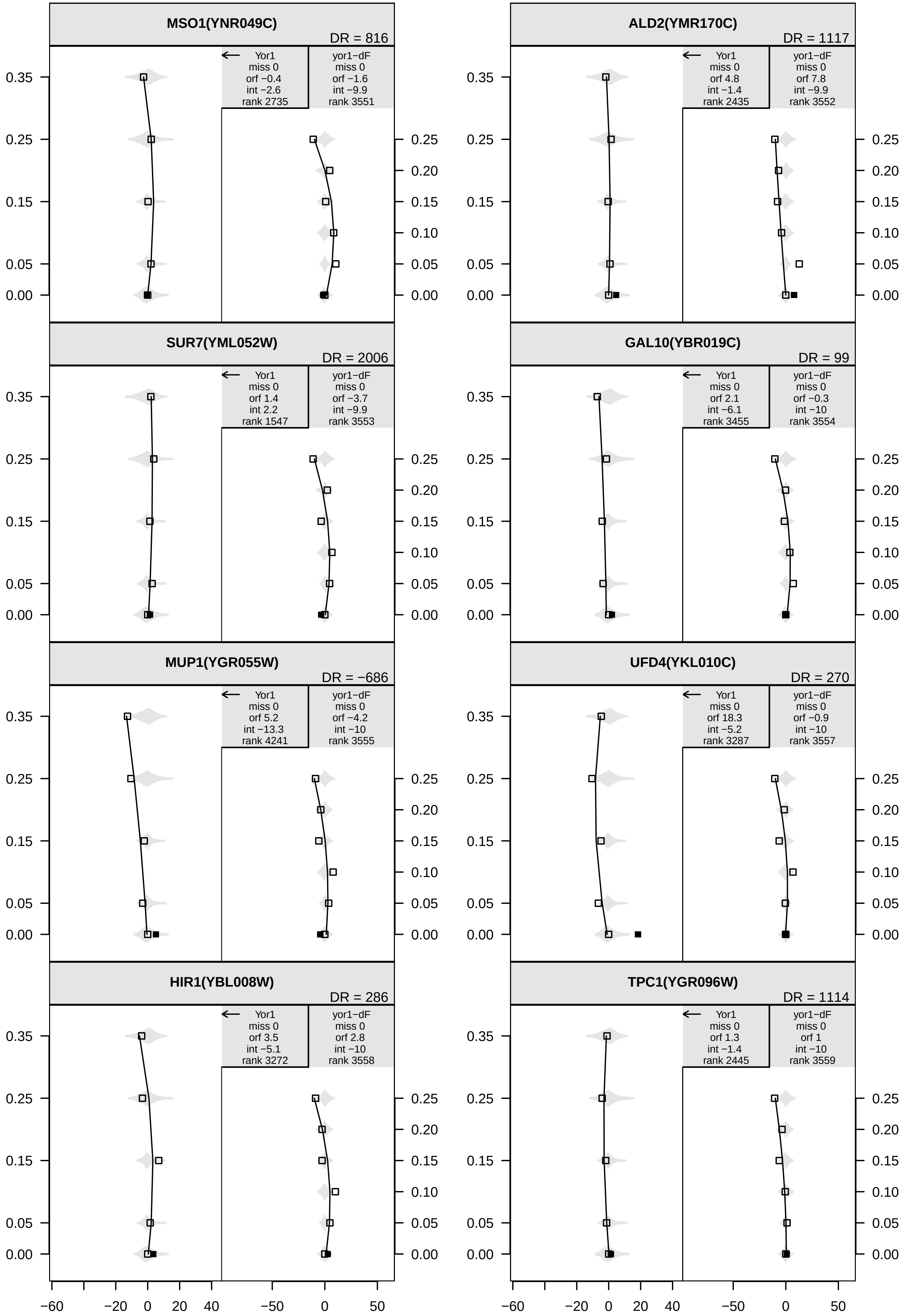

MSO1(YNR049C)
DR = 816
Yor1
miss 0
orf −0.4
int −2.6
rank 2735
yor1−dF
miss 0
orf −1.6
int −9.9
rank 3551
ALD2(YMR170C)
DR = 1117
Yor1
miss 0
orf 4.8
int −1.4
rank 2435
yor1−dF
miss 0
orf 7.8
int −9.9
rank 3552
SUR7(YML052W)
DR = 2006
Yor1
miss 0
orf 1.4
int 2.2
rank 1547
yor1−dF
miss 0
orf −3.7
int −9.9
rank 3553
GAL10(YBR019C)
DR = 99
Yor1
miss 0
orf 2.1
int −6.1
rank 3455
yor1−dF
miss 0
orf −0.3
int −10
rank 3554
MUP1(YGR055W)
DR = −686
Yor1
miss 0
orf 5.2
int −13.3
rank 4241
yor1−dF
miss 0
orf −4.2
int −10
rank 3555
UFD4(YKL010C)
DR = 270
Yor1
miss 0
orf 18.3
int −5.2
rank 3287
yor1−dF
miss 0
orf −0.9
int −10
rank 3557
HIR1(YBL008W)
DR = 286
Yor1
miss 0
orf 3.5
int −5.1
rank 3272
yor1−dF
miss 0
orf 2.8
int −10
rank 3558
TPC1(YGR096W)
DR = 1114
Yor1
miss 0
orf 1.3
int −1.4
rank 2445
yor1−dF
miss 0
orf 1
int −10
rank 3559
0.35
0.25
0.20
0.15
0.10
0.05
0.00
−60
−20
0
20
40
−50
0
50

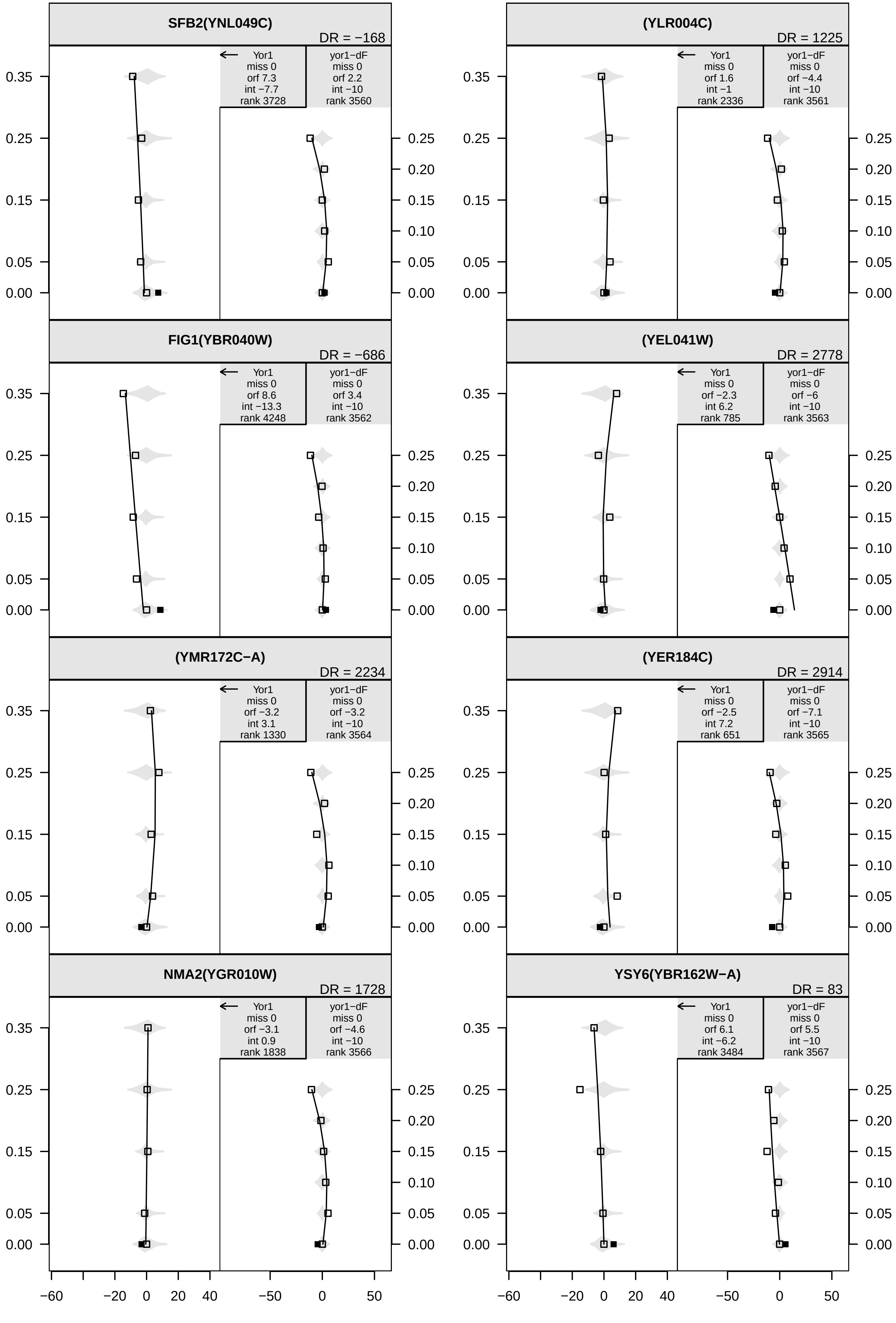
SFB2(YNL049C)
DR = −168
Yor1 miss 0 orf 7.3 int −7.7 rank 3728
yor1−dF miss 0 orf 2.2 int −10 rank 3560
(YLR004C)
DR = 1225
Yor1 miss 0 orf 1.6 int −1 rank 2336
yor1−dF miss 0 orf −4.4 int −10 rank 3561
FIG1(YBR040W)
DR = −686
Yor1 miss 0 orf 8.6 int −13.3 rank 4248
yor1−dF miss 0 orf 3.4 int −10 rank 3562
(YEL041W)
DR = 2778
Yor1 miss 0 orf −2.3 int 6.2 rank 785
yor1−dF miss 0 orf −6 int −10 rank 3563
(YMR172C−A)
DR = 2234
Yor1 miss 0 orf −3.2 int 3.1 rank 1330
yor1−dF miss 0 orf −3.2 int −10 rank 3564
(YER184C)
DR = 2914
Yor1 miss 0 orf −2.5 int 7.2 rank 651
yor1−dF miss 0 orf −7.1 int −10 rank 3565
NMA2(YGR010W)
DR = 1728
Yor1 miss 0 orf −3.1 int 0.9 rank 1838
yor1−dF miss 0 orf −4.6 int −10 rank 3566
YSY6(YBR162W−A)
DR = 83
Yor1 miss 0 orf 6.1 int −6.2 rank 3484
yor1−dF miss 0 orf 5.5 int −10 rank 3567
0.35
0.25
0.20
0.15
0.10
0.05
0.00
−60
−20
0
20
40
−50
0
50

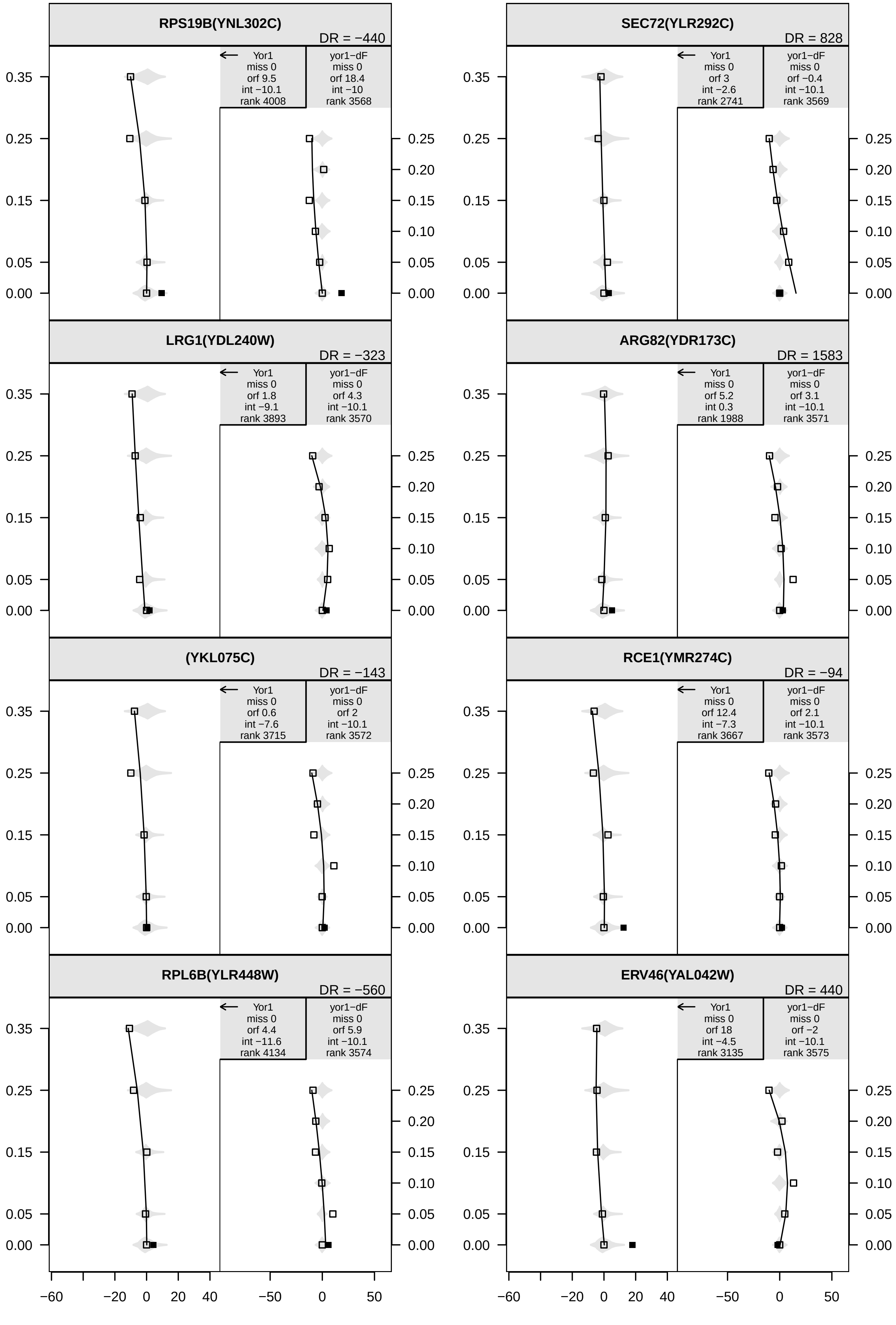

RPS19B(YNL302C)
DR = −440
Yor1 miss 0 orf 9.5 int −10.1 rank 4008
yor1−dF miss 0 orf 18.4 int −10 rank 3568
SEC72(YLR292C)
DR = 828
Yor1 miss 0 orf 3 int −2.6 rank 2741
yor1−dF miss 0 orf −0.4 int −10.1 rank 3569
LRG1(YDL240W)
DR = −323
Yor1 miss 0 orf 1.8 int −9.1 rank 3893
yor1−dF miss 0 orf 4.3 int −10.1 rank 3570
ARG82(YDR173C)
DR = 1583
Yor1 miss 0 orf 5.2 int 0.3 rank 1988
yor1−dF miss 0 orf 3.1 int −10.1 rank 3571
(YKL075C)
DR = −143
Yor1 miss 0 orf 0.6 int −7.6 rank 3715
yor1−dF miss 0 orf 2 int −10.1 rank 3572
RCE1(YMR274C)
DR = −94
Yor1 miss 0 orf 12.4 int −7.3 rank 3667
yor1−dF miss 0 orf 2.1 int −10.1 rank 3573
RPL6B(YLR448W)
DR = −560
Yor1 miss 0 orf 4.4 int −11.6 rank 4134
yor1−dF miss 0 orf 5.9 int −10.1 rank 3574
ERV46(YAL042W)
DR = 440
Yor1 miss 0 orf 18 int −4.5 rank 3135
yor1−dF miss 0 orf −2 int −10.1 rank 3575

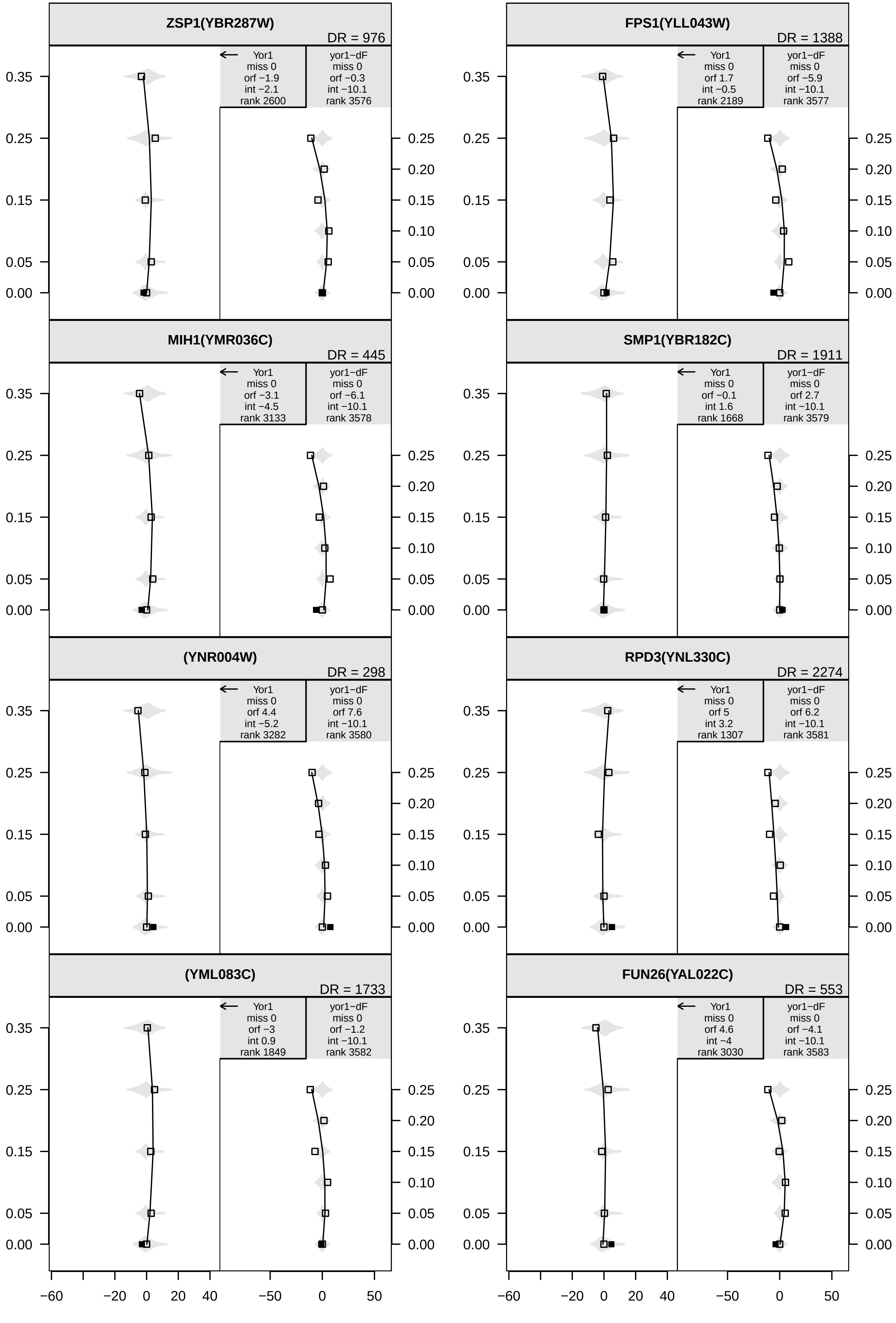
ZSP1(YBR287W)
DR = 976
Yor1 miss 0 orf −1.9 int −2.1 rank 2600
yor1−dF miss 0 orf −0.3 int −10.1 rank 3576
FPS1(YLL043W)
DR = 1388
Yor1 miss 0 orf 1.7 int −0.5 rank 2189
yor1−dF miss 0 orf −5.9 int −10.1 rank 3577
MIH1(YMR036C)
DR = 445
Yor1 miss 0 orf −3.1 int −4.5 rank 3133
yor1−dF miss 0 orf −6.1 int −10.1 rank 3578
SMP1(YBR182C)
DR = 1911
Yor1 miss 0 orf −0.1 int 1.6 rank 1668
yor1−dF miss 0 orf 2.7 int −10.1 rank 3579
(YNR004W)
DR = 298
Yor1 miss 0 orf 4.4 int −5.2 rank 3282
yor1−dF miss 0 orf 7.6 int −10.1 rank 3580
RPD3(YNL330C)
DR = 2274
Yor1 miss 0 orf 5 int 3.2 rank 1307
yor1−dF miss 0 orf 6.2 int −10.1 rank 3581
(YML083C)
DR = 1733
Yor1 miss 0 orf −3 int 0.9 rank 1849
yor1−dF miss 0 orf −1.2 int −10.1 rank 3582
FUN26(YAL022C)
DR = 553
Yor1 miss 0 orf 4.6 int −4 rank 3030
yor1−dF miss 0 orf −4.1 int −10.1 rank 3583
0.35 0.25 0.15 0.05 0.00
0.25 0.20 0.15 0.10 0.05 0.00
−60 −20 0 20 40
−50 0 50

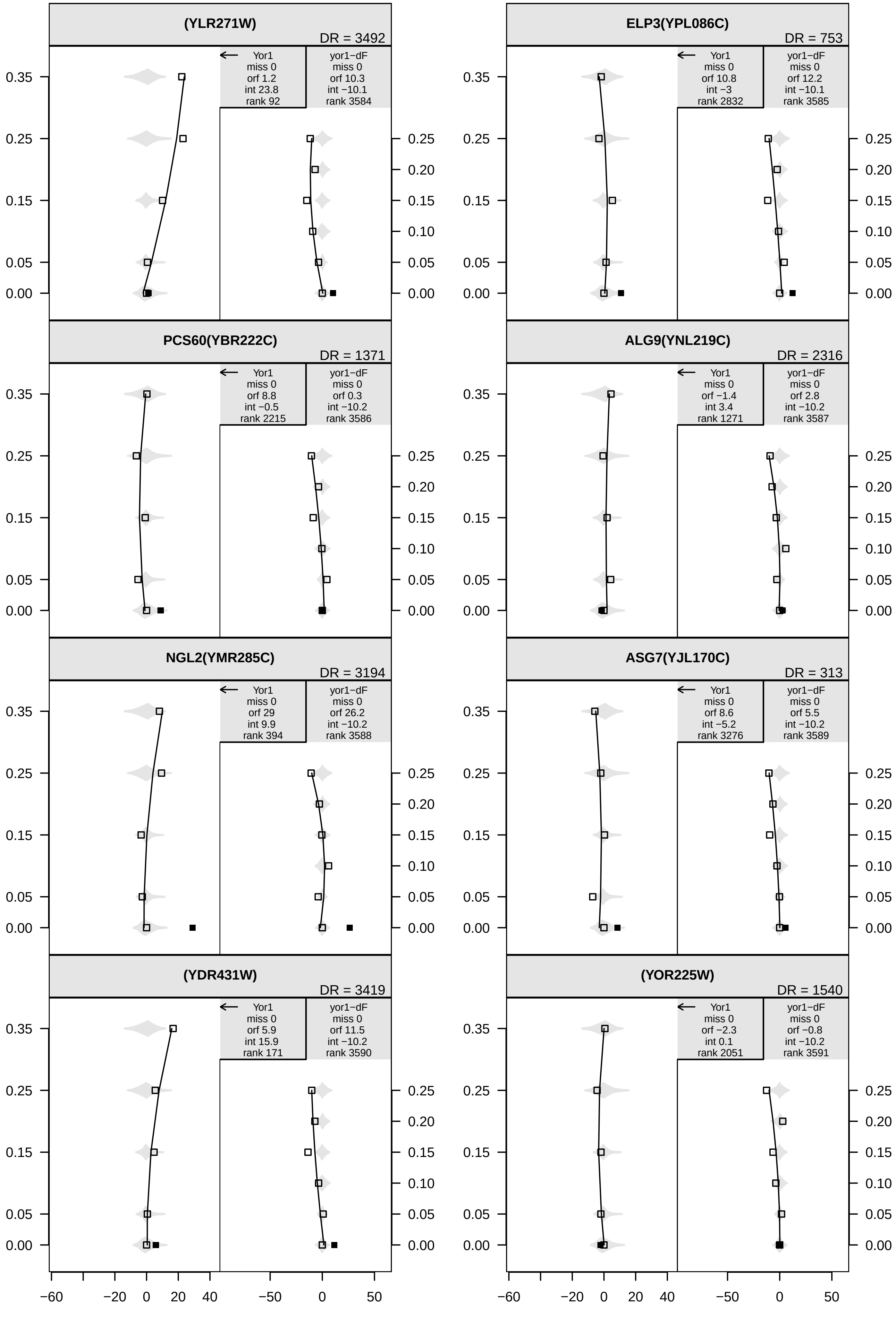
(YLR271W)
DR = 3492
Yor1 miss 0 orf 1.2 int 23.8 rank 92
yor1−dF miss 0 orf 10.3 int −10.1 rank 3584
ELP3(YPL086C)
DR = 753
Yor1 miss 0 orf 10.8 int −3 rank 2832
yor1−dF miss 0 orf 12.2 int −10.1 rank 3585
PCS60(YBR222C)
DR = 1371
Yor1 miss 0 orf 8.8 int −0.5 rank 2215
yor1−dF miss 0 orf 0.3 int −10.2 rank 3586
ALG9(YNL219C)
DR = 2316
Yor1 miss 0 orf −1.4 int 3.4 rank 1271
yor1−dF miss 0 orf 2.8 int −10.2 rank 3587
NGL2(YMR285C)
DR = 3194
Yor1 miss 0 orf 29 int 9.9 rank 394
yor1−dF miss 0 orf 26.2 int −10.2 rank 3588
ASG7(YJL170C)
DR = 313
Yor1 miss 0 orf 8.6 int −5.2 rank 3276
yor1−dF miss 0 orf 5.5 int −10.2 rank 3589
(YDR431W)
DR = 3419
Yor1 miss 0 orf 5.9 int 15.9 rank 171
yor1−dF miss 0 orf 11.5 int −10.2 rank 3590
(YOR225W)
DR = 1540
Yor1 miss 0 orf −2.3 int 0.1 rank 2051
yor1−dF miss 0 orf −0.8 int −10.2 rank 3591
0.35 0.25 0.20 0.15 0.10 0.05 0.00
−60 −20 0 20 40 −50 0 50

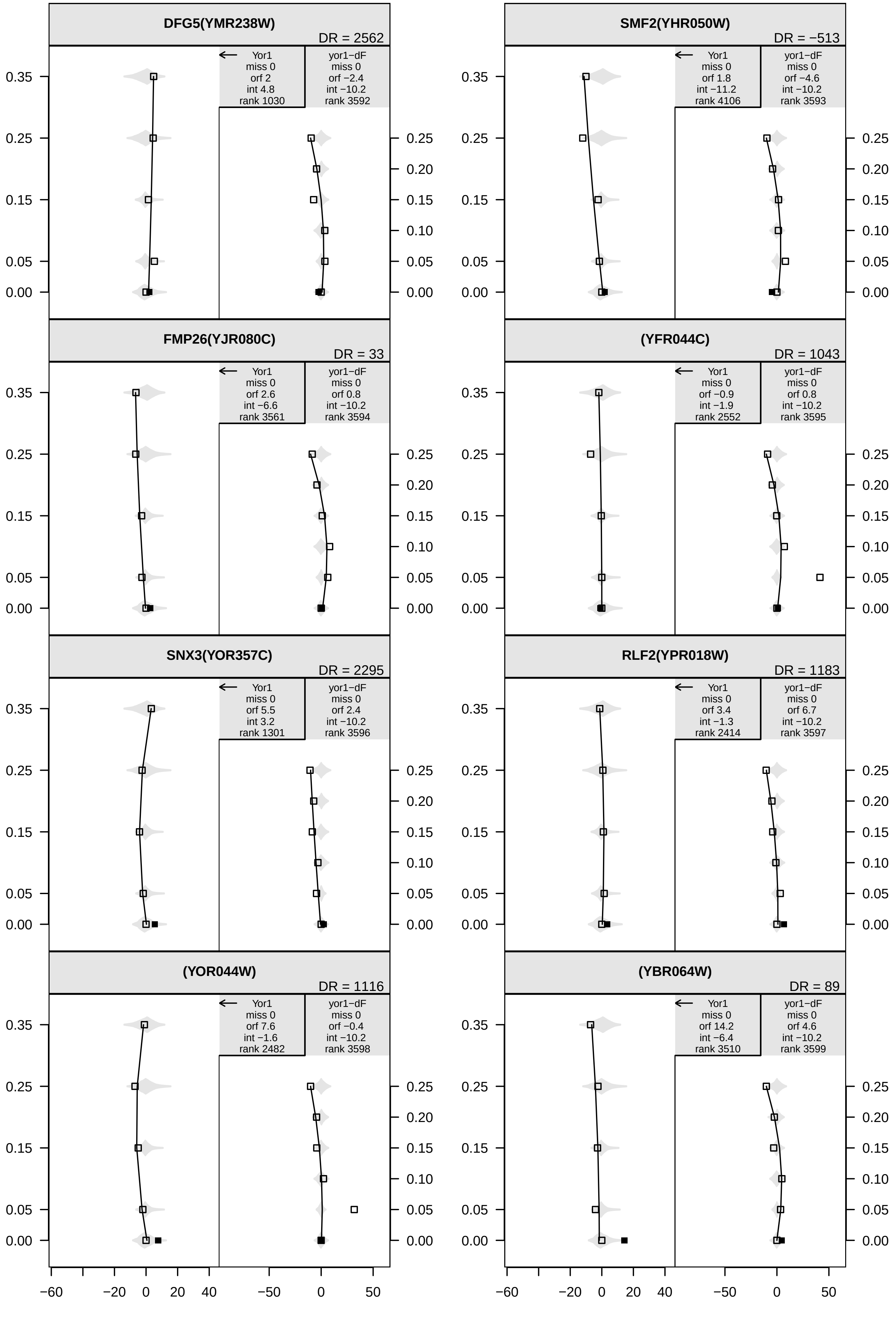

DFG5(YMR238W)
DR = 2562
Yor1 miss 0 orf 2 int 4.8 rank 1030
yor1−dF miss 0 orf −2.4 int −10.2 rank 3592
SMF2(YHR050W)
DR = −513
Yor1 miss 0 orf 1.8 int −11.2 rank 4106
yor1−dF miss 0 orf −4.6 int −10.2 rank 3593
FMP26(YJR080C)
DR = 33
Yor1 miss 0 orf 2.6 int −6.6 rank 3561
yor1−dF miss 0 orf 0.8 int −10.2 rank 3594
(YFR044C)
DR = 1043
Yor1 miss 0 orf −0.9 int −1.9 rank 2552
yor1−dF miss 0 orf 0.8 int −10.2 rank 3595
SNX3(YOR357C)
DR = 2295
Yor1 miss 0 orf 5.5 int 3.2 rank 1301
yor1−dF miss 0 orf 2.4 int −10.2 rank 3596
RLF2(YPR018W)
DR = 1183
Yor1 miss 0 orf 3.4 int −1.3 rank 2414
yor1−dF miss 0 orf 6.7 int −10.2 rank 3597
(YOR044W)
DR = 1116
Yor1 miss 0 orf 7.6 int −1.6 rank 2482
yor1−dF miss 0 orf −0.4 int −10.2 rank 3598
(YBR064W)
DR = 89
Yor1 miss 0 orf 14.2 int −6.4 rank 3510
yor1−dF miss 0 orf 4.6 int −10.2 rank 3599

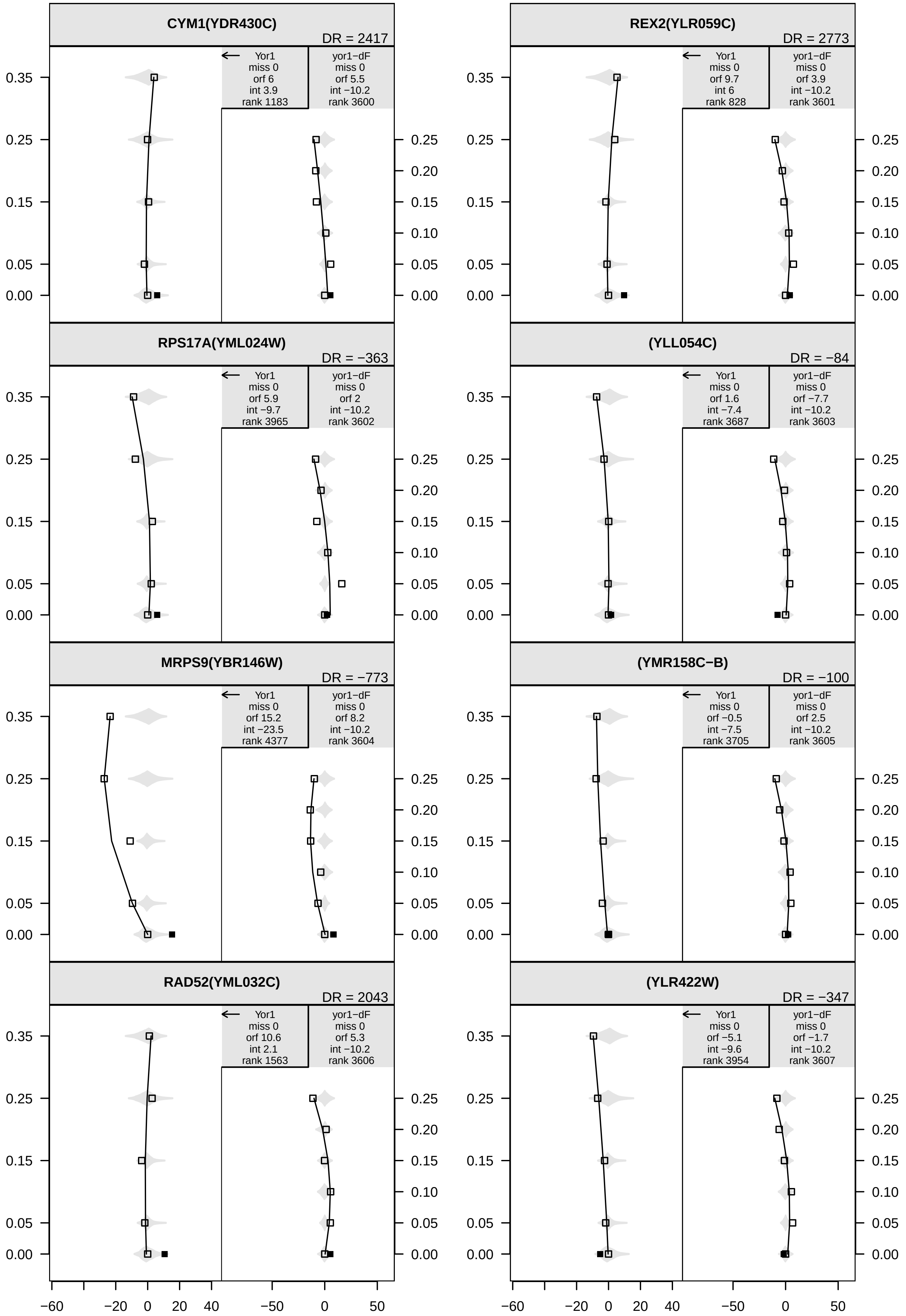
CYM1(YDR430C)
DR = 2417
Yor1
miss 0
orf 6
int 3.9
rank 1183
yor1−dF
miss 0
orf 5.5
int −10.2
rank 3600
REX2(YLR059C)
DR = 2773
Yor1
miss 0
orf 9.7
int 6
rank 828
yor1−dF
miss 0
orf 3.9
int −10.2
rank 3601
RPS17A(YML024W)
DR = −363
Yor1
miss 0
orf 5.9
int −9.7
rank 3965
yor1−dF
miss 0
orf 2
int −10.2
rank 3602
(YLL054C)
DR = −84
Yor1
miss 0
orf 1.6
int −7.4
rank 3687
yor1−dF
miss 0
orf −7.7
int −10.2
rank 3603
MRPS9(YBR146W)
DR = −773
Yor1
miss 0
orf 15.2
int −23.5
rank 4377
yor1−dF
miss 0
orf 8.2
int −10.2
rank 3604
(YMR158C−B)
DR = −100
Yor1
miss 0
orf −0.5
int −7.5
rank 3705
yor1−dF
miss 0
orf 2.5
int −10.2
rank 3605
RAD52(YML032C)
DR = 2043
Yor1
miss 0
orf 10.6
int 2.1
rank 1563
yor1−dF
miss 0
orf 5.3
int −10.2
rank 3606
(YLR422W)
DR = −347
Yor1
miss 0
orf −5.1
int −9.6
rank 3954
yor1−dF
miss 0
orf −1.7
int −10.2
rank 3607
0.35
0.25
0.20
0.15
0.10
0.05
0.00
−60
−20
0
20
40
−50
0
50

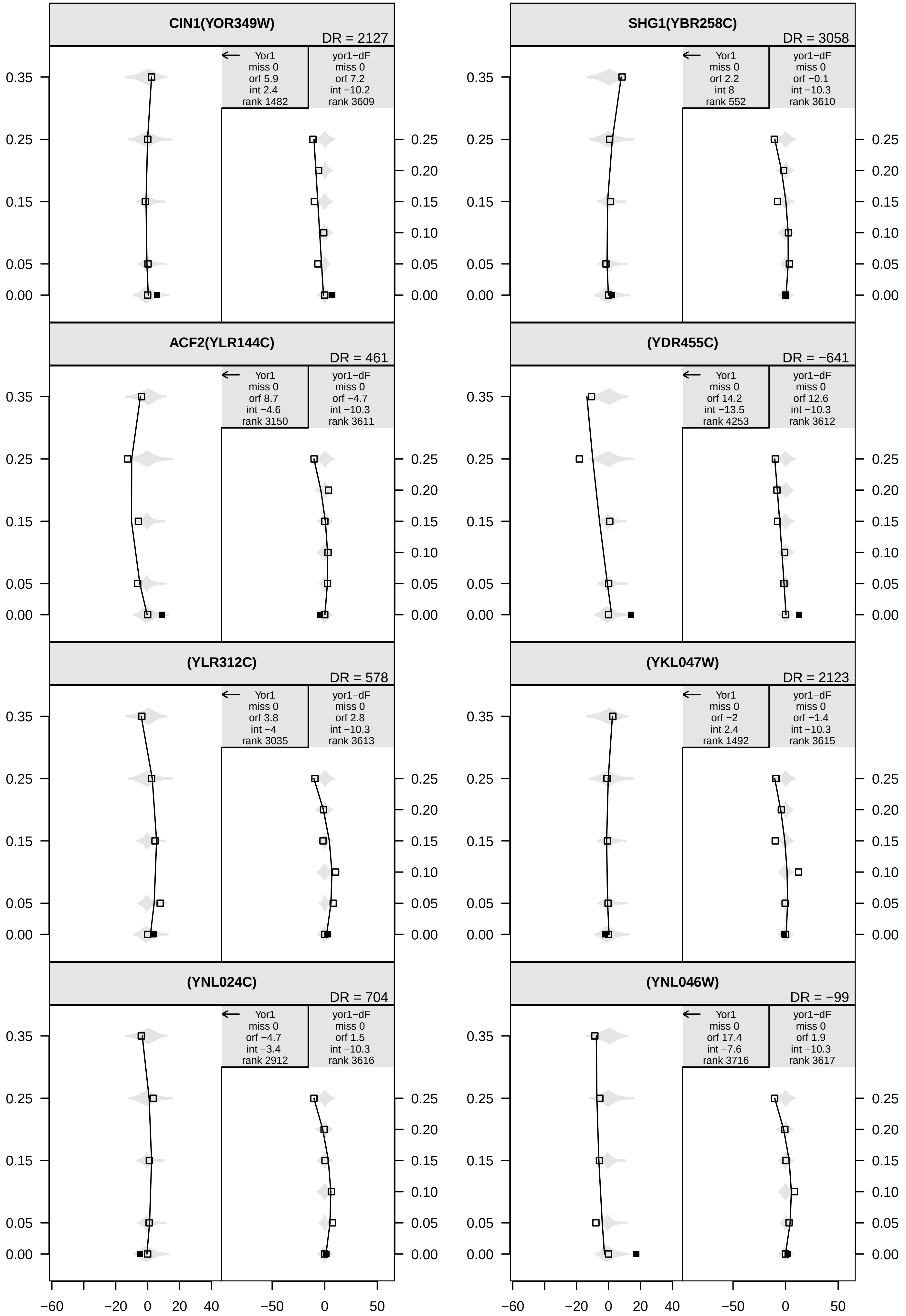

CIN1(YOR349W)
DR = 2127
Yor1 miss 0 orf 5.9 int 2.4 rank 1482
yor1−dF miss 0 orf 7.2 int −10.2 rank 3609
SHG1(YBR258C)
DR = 3058
Yor1 miss 0 orf 2.2 int 8 rank 552
yor1−dF miss 0 orf −0.1 int −10.3 rank 3610
ACF2(YLR144C)
DR = 461
Yor1 miss 0 orf 8.7 int −4.6 rank 3150
yor1−dF miss 0 orf −4.7 int −10.3 rank 3611
(YDR455C)
DR = −641
Yor1 miss 0 orf 14.2 int −13.5 rank 4253
yor1−dF miss 0 orf 12.6 int −10.3 rank 3612
(YLR312C)
DR = 578
Yor1 miss 0 orf 3.8 int −4 rank 3035
yor1−dF miss 0 orf 2.8 int −10.3 rank 3613
(YKL047W)
DR = 2123
Yor1 miss 0 orf −2 int 2.4 rank 1492
yor1−dF miss 0 orf −1.4 int −10.3 rank 3615
(YNL024C)
DR = 704
Yor1 miss 0 orf −4.7 int −3.4 rank 2912
yor1−dF miss 0 orf 1.5 int −10.3 rank 3616
(YNL046W)
DR = −99
Yor1 miss 0 orf 17.4 int −7.6 rank 3716
yor1−dF miss 0 orf 1.9 int −10.3 rank 3617
0.00 0.05 0.10 0.15 0.20 0.25 0.35
−60 −20 0 20 40 −50 0 50

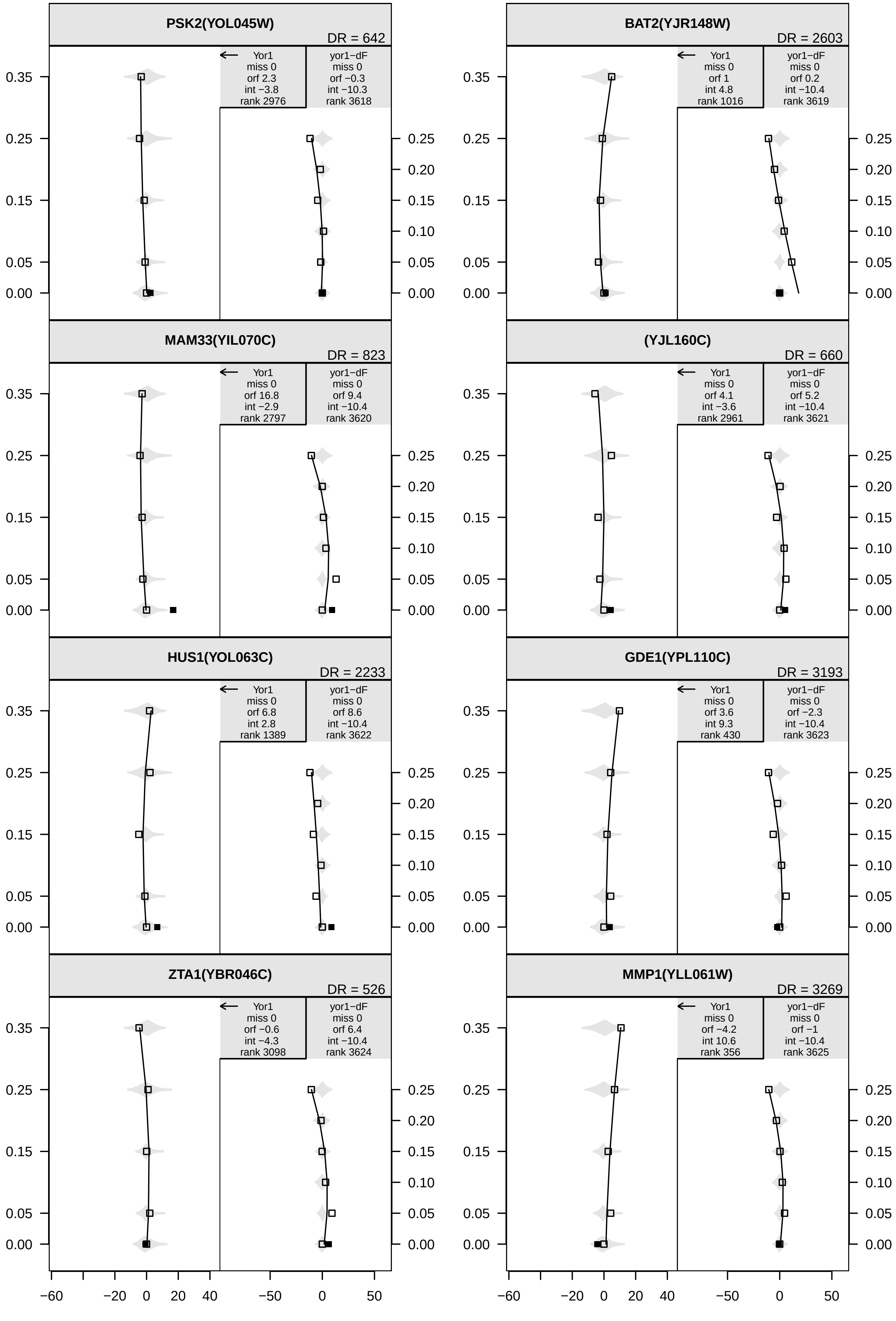
PSK2(YOL045W)
DR = 642
Yor1
miss 0
orf 2.3
int −3.8
rank 2976
yor1−dF
miss 0
orf −0.3
int −10.3
rank 3618
BAT2(YJR148W)
DR = 2603
Yor1
miss 0
orf 1
int 4.8
rank 1016
yor1−dF
miss 0
orf 0.2
int −10.4
rank 3619
MAM33(YIL070C)
DR = 823
Yor1
miss 0
orf 16.8
int −2.9
rank 2797
yor1−dF
miss 0
orf 9.4
int −10.4
rank 3620
(YJL160C)
DR = 660
Yor1
miss 0
orf 4.1
int −3.6
rank 2961
yor1−dF
miss 0
orf 5.2
int −10.4
rank 3621
HUS1(YOL063C)
DR = 2233
Yor1
miss 0
orf 6.8
int 2.8
rank 1389
yor1−dF
miss 0
orf 8.6
int −10.4
rank 3622
GDE1(YPL110C)
DR = 3193
Yor1
miss 0
orf 3.6
int 9.3
rank 430
yor1−dF
miss 0
orf −2.3
int −10.4
rank 3623
ZTA1(YBR046C)
DR = 526
Yor1
miss 0
orf −0.6
int −4.3
rank 3098
yor1−dF
miss 0
orf 6.4
int −10.4
rank 3624
MMP1(YLL061W)
DR = 3269
Yor1
miss 0
orf −4.2
int 10.6
rank 356
yor1−dF
miss 0
orf −1
int −10.4
rank 3625
0.35
0.25
0.20
0.15
0.10
0.05
0.00
−60
−20
0
20
40
−50
0
50

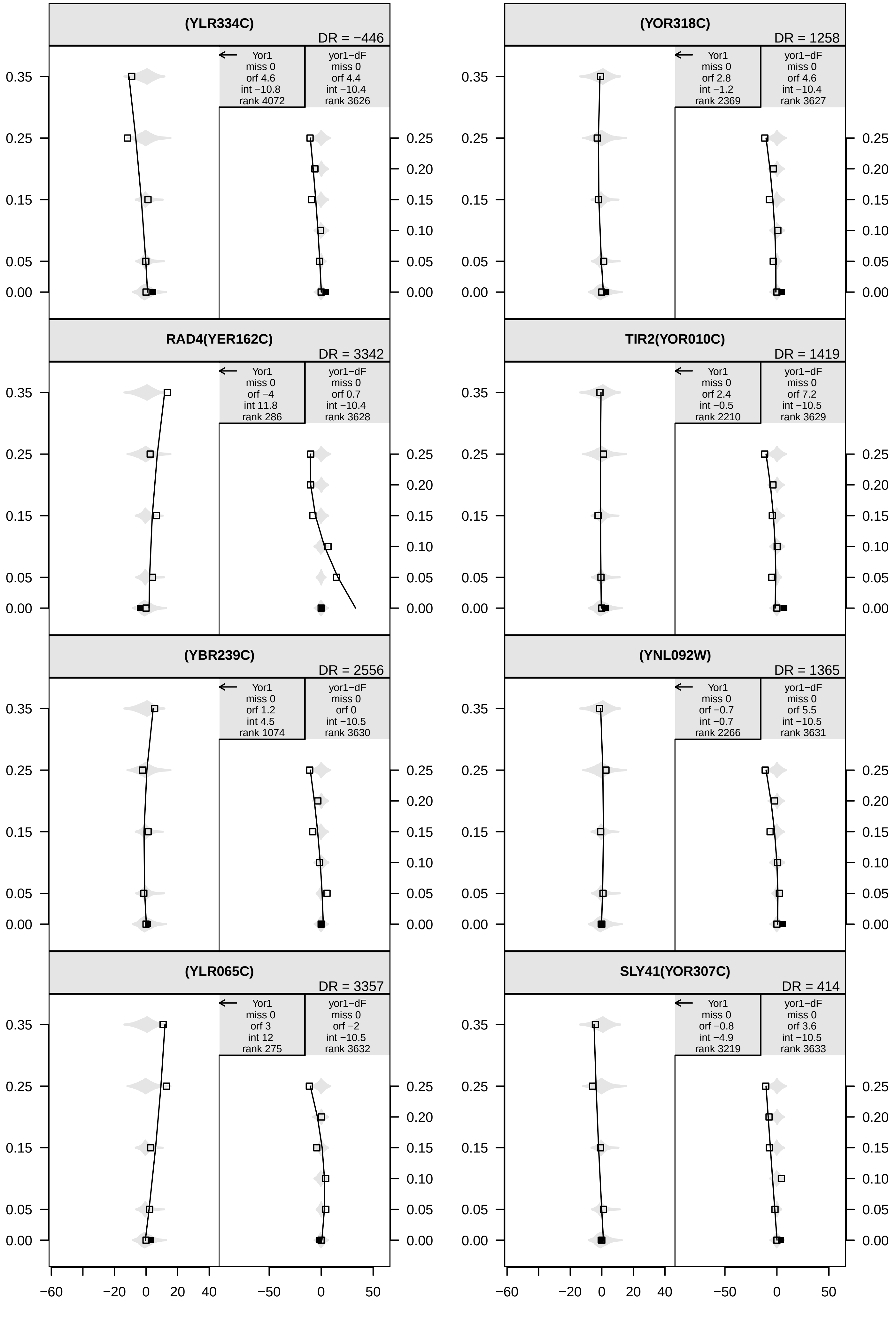

(YLR334C)
DR = −446
Yor1 miss 0 orf 4.6 int −10.8 rank 4072
yor1−dF miss 0 orf 4.4 int −10.4 rank 3626
(YOR318C)
DR = 1258
Yor1 miss 0 orf 2.8 int −1.2 rank 2369
yor1−dF miss 0 orf 4.6 int −10.4 rank 3627
RAD4(YER162C)
DR = 3342
Yor1 miss 0 orf −4 int 11.8 rank 286
yor1−dF miss 0 orf 0.7 int −10.4 rank 3628
TIR2(YOR010C)
DR = 1419
Yor1 miss 0 orf 2.4 int −0.5 rank 2210
yor1−dF miss 0 orf 7.2 int −10.5 rank 3629
(YBR239C)
DR = 2556
Yor1 miss 0 orf 1.2 int 4.5 rank 1074
yor1−dF miss 0 orf 0 int −10.5 rank 3630
(YNL092W)
DR = 1365
Yor1 miss 0 orf −0.7 int −0.7 rank 2266
yor1−dF miss 0 orf 5.5 int −10.5 rank 3631
(YLR065C)
DR = 3357
Yor1 miss 0 orf 3 int 12 rank 275
yor1−dF miss 0 orf −2 int −10.5 rank 3632
SLY41(YOR307C)
DR = 414
Yor1 miss 0 orf −0.8 int −4.9 rank 3219
yor1−dF miss 0 orf 3.6 int −10.5 rank 3633
0.35
0.25
0.20
0.15
0.10
0.05
0.00
−60
−20
0
20
40
−50
0
50

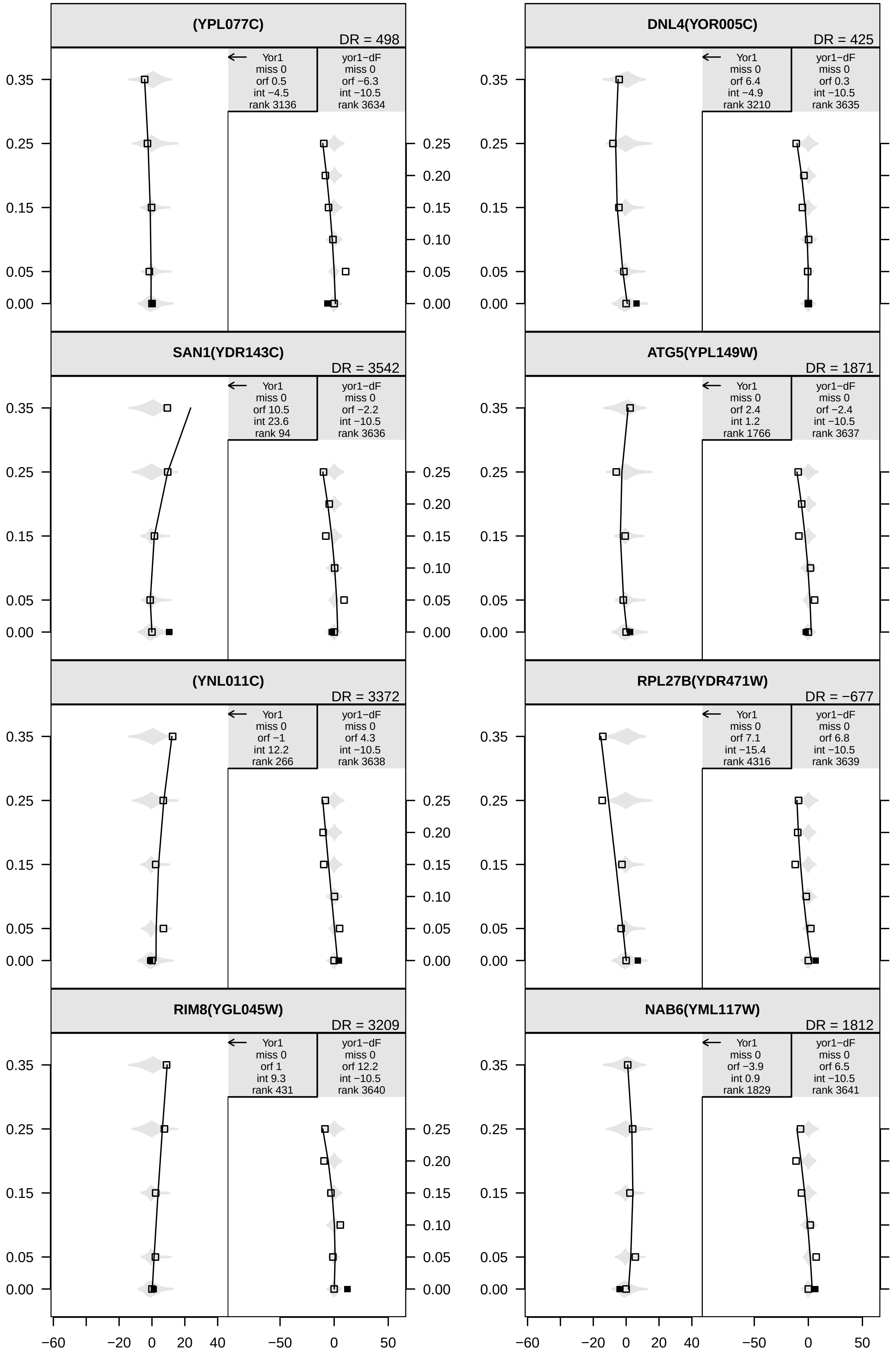
(YPL077C)
DR = 498
Yor1 miss 0 orf 0.5 int −4.5 rank 3136
yor1−dF miss 0 orf −6.3 int −10.5 rank 3634
DNL4(YOR005C)
DR = 425
Yor1 miss 0 orf 6.4 int −4.9 rank 3210
yor1−dF miss 0 orf 0.3 int −10.5 rank 3635
SAN1(YDR143C)
DR = 3542
Yor1 miss 0 orf 10.5 int 23.6 rank 94
yor1−dF miss 0 orf −2.2 int −10.5 rank 3636
ATG5(YPL149W)
DR = 1871
Yor1 miss 0 orf 2.4 int 1.2 rank 1766
yor1−dF miss 0 orf −2.4 int −10.5 rank 3637
(YNL011C)
DR = 3372
Yor1 miss 0 orf −1 int 12.2 rank 266
yor1−dF miss 0 orf 4.3 int −10.5 rank 3638
RPL27B(YDR471W)
DR = −677
Yor1 miss 0 orf 7.1 int −15.4 rank 4316
yor1−dF miss 0 orf 6.8 int −10.5 rank 3639
RIM8(YGL045W)
DR = 3209
Yor1 miss 0 orf 1 int 9.3 rank 431
yor1−dF miss 0 orf 12.2 int −10.5 rank 3640
NAB6(YML117W)
DR = 1812
Yor1 miss 0 orf −3.9 int 0.9 rank 1829
yor1−dF miss 0 orf 6.5 int −10.5 rank 3641
0.35 0.25 0.20 0.15 0.10 0.05 0.00
−60 −20 0 20 40 −50 0 50

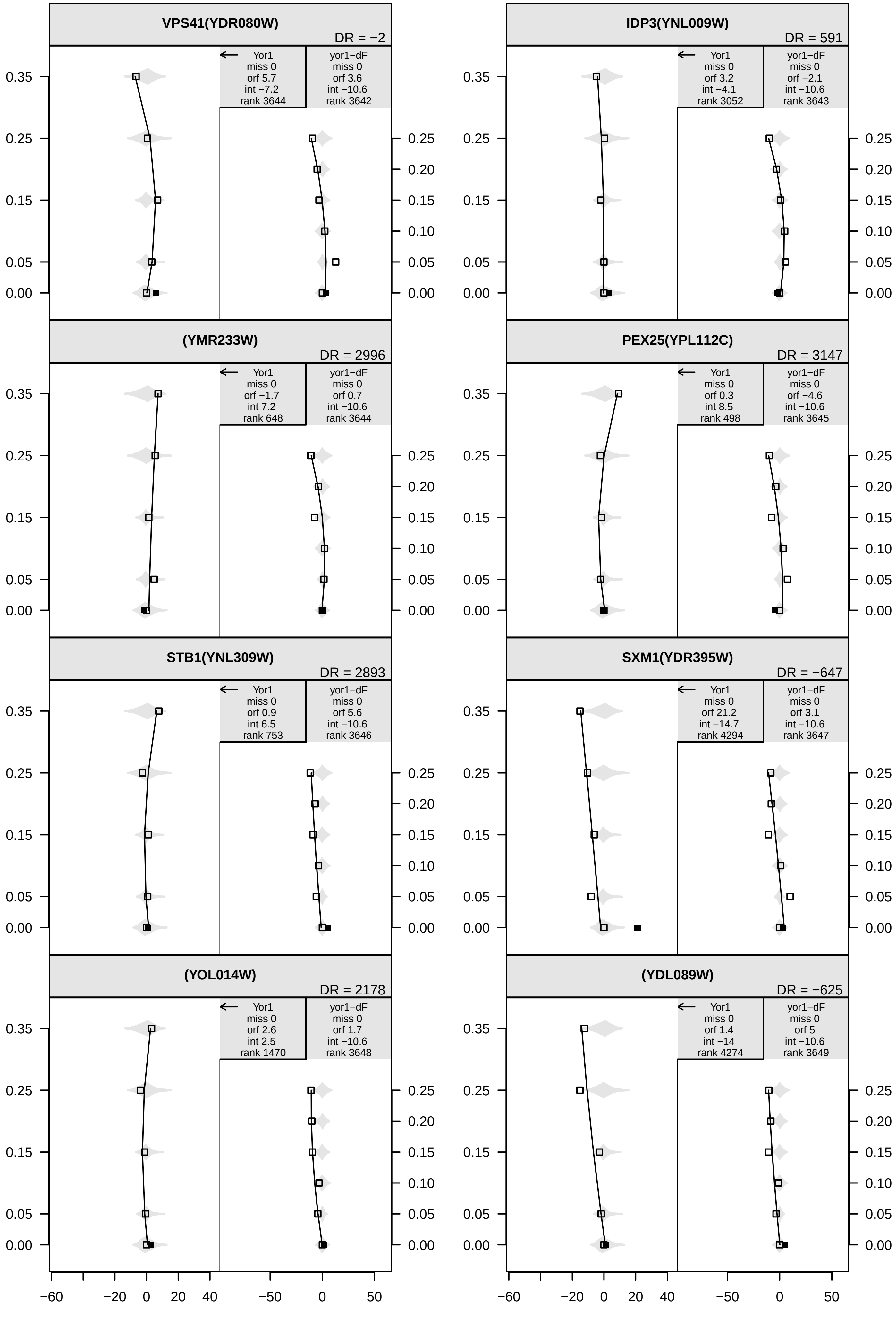
VPS41(YDR080W)
DR = −2
Yor1 miss 0 orf 5.7 int −7.2 rank 3644
yor1−dF miss 0 orf 3.6 int −10.6 rank 3642
IDP3(YNL009W)
DR = 591
Yor1 miss 0 orf 3.2 int −4.1 rank 3052
yor1−dF miss 0 orf −2.1 int −10.6 rank 3643
(YMR233W)
DR = 2996
Yor1 miss 0 orf −1.7 int 7.2 rank 648
yor1−dF miss 0 orf 0.7 int −10.6 rank 3644
PEX25(YPL112C)
DR = 3147
Yor1 miss 0 orf 0.3 int 8.5 rank 498
yor1−dF miss 0 orf −4.6 int −10.6 rank 3645
STB1(YNL309W)
DR = 2893
Yor1 miss 0 orf 0.9 int 6.5 rank 753
yor1−dF miss 0 orf 5.6 int −10.6 rank 3646
SXM1(YDR395W)
DR = −647
Yor1 miss 0 orf 21.2 int −14.7 rank 4294
yor1−dF miss 0 orf 3.1 int −10.6 rank 3647
(YOL014W)
DR = 2178
Yor1 miss 0 orf 2.6 int 2.5 rank 1470
yor1−dF miss 0 orf 1.7 int −10.6 rank 3648
(YDL089W)
DR = −625
Yor1 miss 0 orf 1.4 int −14 rank 4274
yor1−dF miss 0 orf 5 int −10.6 rank 3649
0.00 0.05 0.10 0.15 0.20 0.25 0.35
−60 −20 0 20 40 −50 0 50

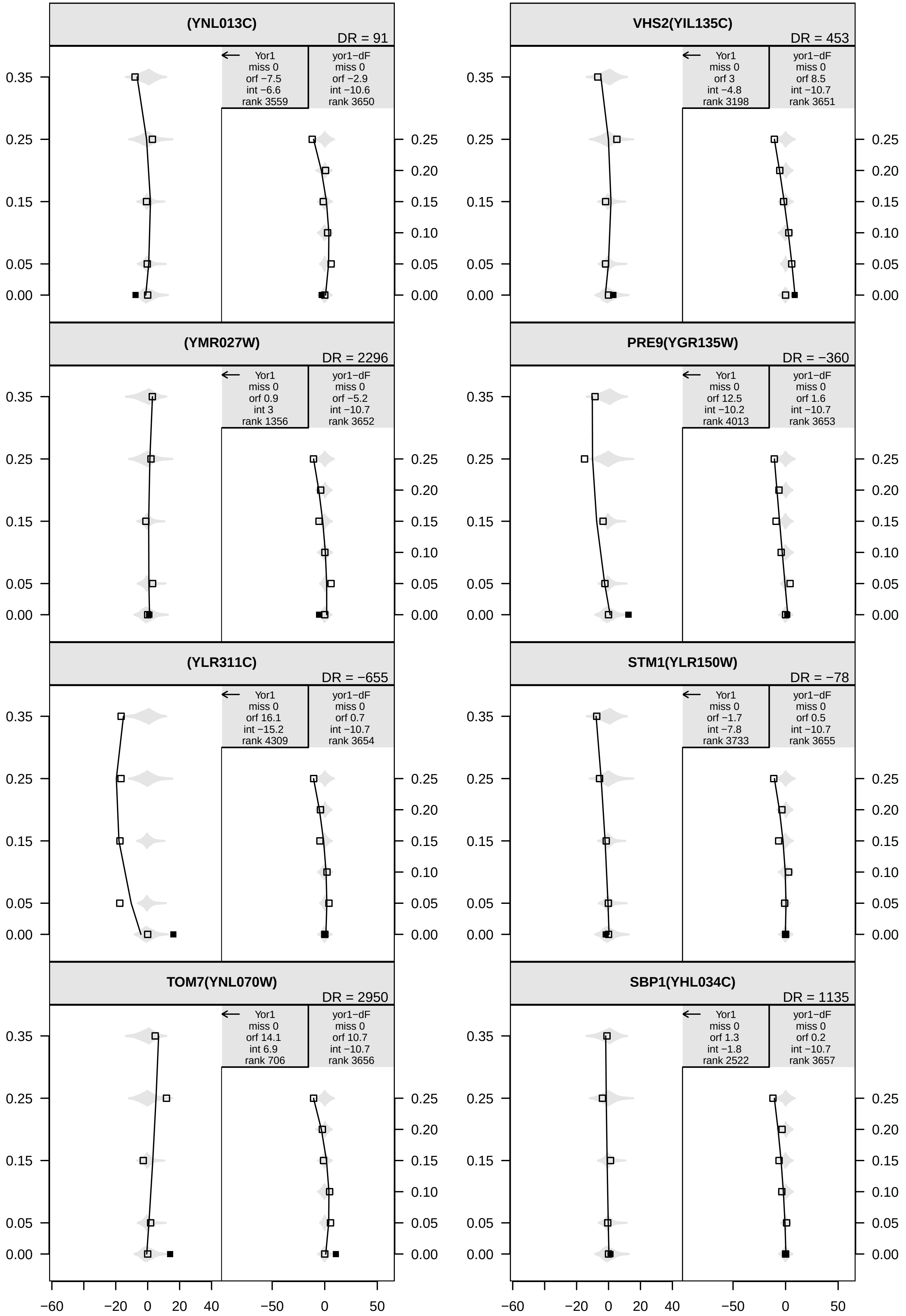

(YNL013C)
DR = 91
Yor1 miss 0 orf −7.5 int −6.6 rank 3559
yor1−dF miss 0 orf −2.9 int −10.6 rank 3650
VHS2(YIL135C)
DR = 453
Yor1 miss 0 orf 3 int −4.8 rank 3198
yor1−dF miss 0 orf 8.5 int −10.7 rank 3651
(YMR027W)
DR = 2296
Yor1 miss 0 orf 0.9 int 3 rank 1356
yor1−dF miss 0 orf −5.2 int −10.7 rank 3652
PRE9(YGR135W)
DR = −360
Yor1 miss 0 orf 12.5 int −10.2 rank 4013
yor1−dF miss 0 orf 1.6 int −10.7 rank 3653
(YLR311C)
DR = −655
Yor1 miss 0 orf 16.1 int −15.2 rank 4309
yor1−dF miss 0 orf 0.7 int −10.7 rank 3654
STM1(YLR150W)
DR = −78
Yor1 miss 0 orf −1.7 int −7.8 rank 3733
yor1−dF miss 0 orf 0.5 int −10.7 rank 3655
TOM7(YNL070W)
DR = 2950
Yor1 miss 0 orf 14.1 int 6.9 rank 706
yor1−dF miss 0 orf 10.7 int −10.7 rank 3656
SBP1(YHL034C)
DR = 1135
Yor1 miss 0 orf 1.3 int −1.8 rank 2522
yor1−dF miss 0 orf 0.2 int −10.7 rank 3657
0.00 0.05 0.15 0.25 0.35
0.00 0.05 0.10 0.15 0.20 0.25
−60 −20 0 20 40
−50 0 50

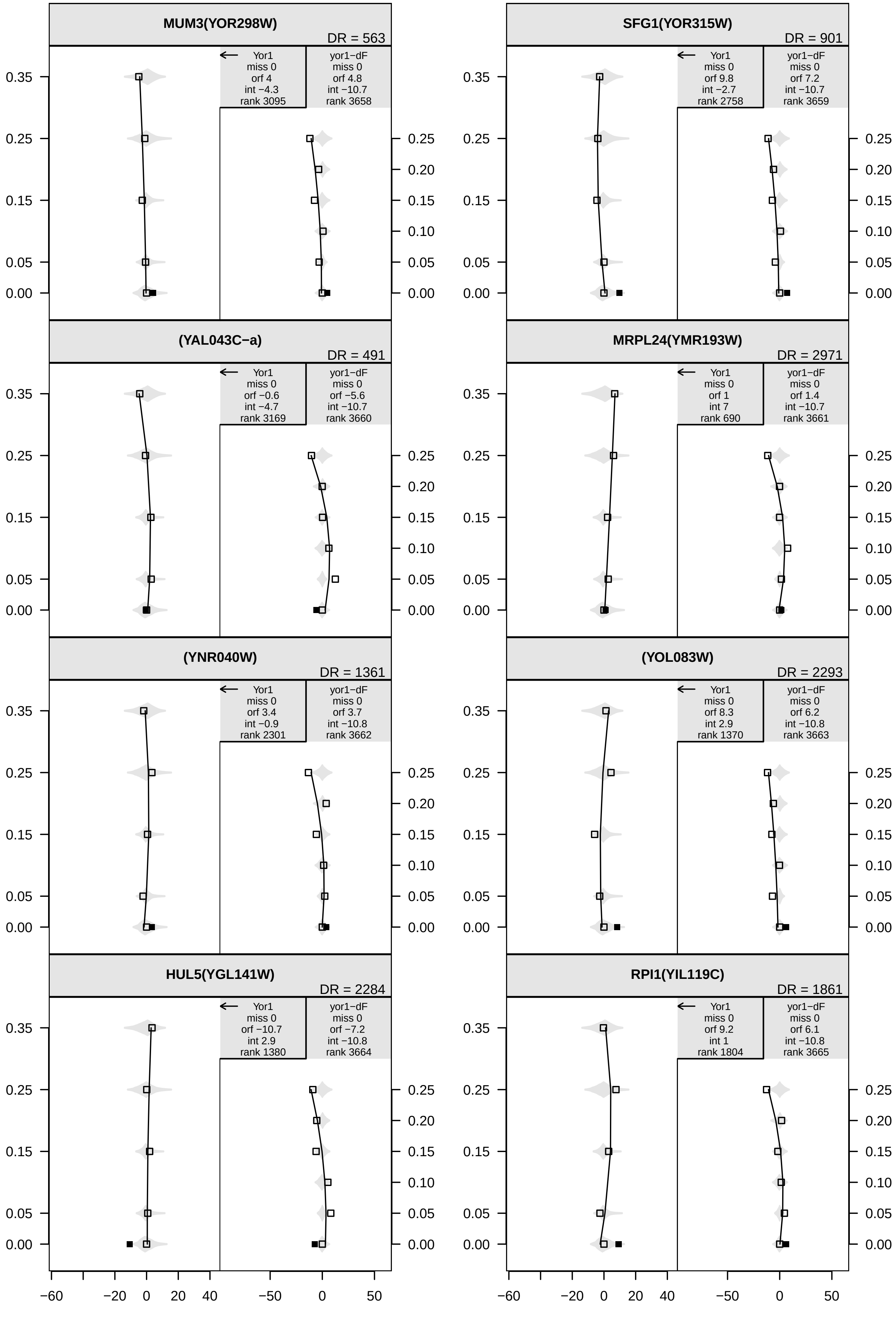

MUM3(YOR298W)
DR = 563
Yor1 miss 0 orf 4 int −4.3 rank 3095
yor1−dF miss 0 orf 4.8 int −10.7 rank 3658
SFG1(YOR315W)
DR = 901
Yor1 miss 0 orf 9.8 int −2.7 rank 2758
yor1−dF miss 0 orf 7.2 int −10.7 rank 3659
(YAL043C−a)
DR = 491
Yor1 miss 0 orf −0.6 int −4.7 rank 3169
yor1−dF miss 0 orf −5.6 int −10.7 rank 3660
MRPL24(YMR193W)
DR = 2971
Yor1 miss 0 orf 1 int 7 rank 690
yor1−dF miss 0 orf 1.4 int −10.7 rank 3661
(YNR040W)
DR = 1361
Yor1 miss 0 orf 3.4 int −0.9 rank 2301
yor1−dF miss 0 orf 3.7 int −10.8 rank 3662
(YOL083W)
DR = 2293
Yor1 miss 0 orf 8.3 int 2.9 rank 1370
yor1−dF miss 0 orf 6.2 int −10.8 rank 3663
HUL5(YGL141W)
DR = 2284
Yor1 miss 0 orf −10.7 int 2.9 rank 1380
yor1−dF miss 0 orf −7.2 int −10.8 rank 3664
RPI1(YIL119C)
DR = 1861
Yor1 miss 0 orf 9.2 int 1 rank 1804
yor1−dF miss 0 orf 6.1 int −10.8 rank 3665
0.35
0.25
0.15
0.05
0.00
0.25
0.20
0.15
0.10
0.05
0.00
−60
−20
0
20
40
−50
0
50

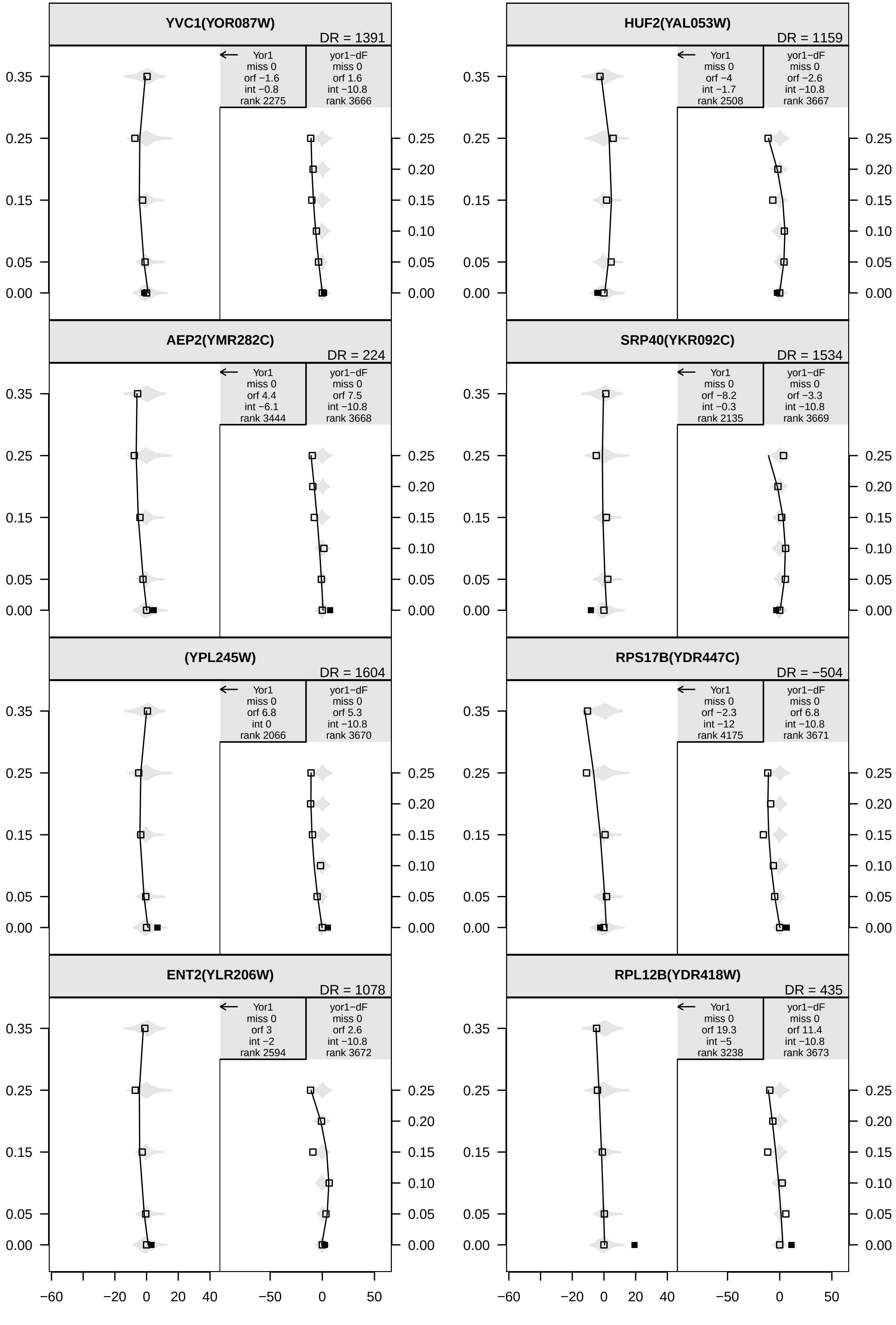

YVC1(YOR087W)
DR = 1391
Yor1 miss 0 orf −1.6 int −0.8 rank 2275
yor1−dF miss 0 orf 1.6 int −10.8 rank 3666
HUF2(YAL053W)
DR = 1159
Yor1 miss 0 orf −4 int −1.7 rank 2508
yor1−dF miss 0 orf −2.6 int −10.8 rank 3667
AEP2(YMR282C)
DR = 224
Yor1 miss 0 orf 4.4 int −6.1 rank 3444
yor1−dF miss 0 orf 7.5 int −10.8 rank 3668
SRP40(YKR092C)
DR = 1534
Yor1 miss 0 orf −8.2 int −0.3 rank 2135
yor1−dF miss 0 orf −3.3 int −10.8 rank 3669
(YPL245W)
DR = 1604
Yor1 miss 0 orf 6.8 int 0 rank 2066
yor1−dF miss 0 orf 5.3 int −10.8 rank 3670
RPS17B(YDR447C)
DR = −504
Yor1 miss 0 orf −2.3 int −12 rank 4175
yor1−dF miss 0 orf 6.8 int −10.8 rank 3671
ENT2(YLR206W)
DR = 1078
Yor1 miss 0 orf 3 int −2 rank 2594
yor1−dF miss 0 orf 2.6 int −10.8 rank 3672
RPL12B(YDR418W)
DR = 435
Yor1 miss 0 orf 19.3 int −5 rank 3238
yor1−dF miss 0 orf 11.4 int −10.8 rank 3673
0.35 0.25 0.20 0.15 0.10 0.05 0.00
−60 −20 0 20 40 −50 0 50

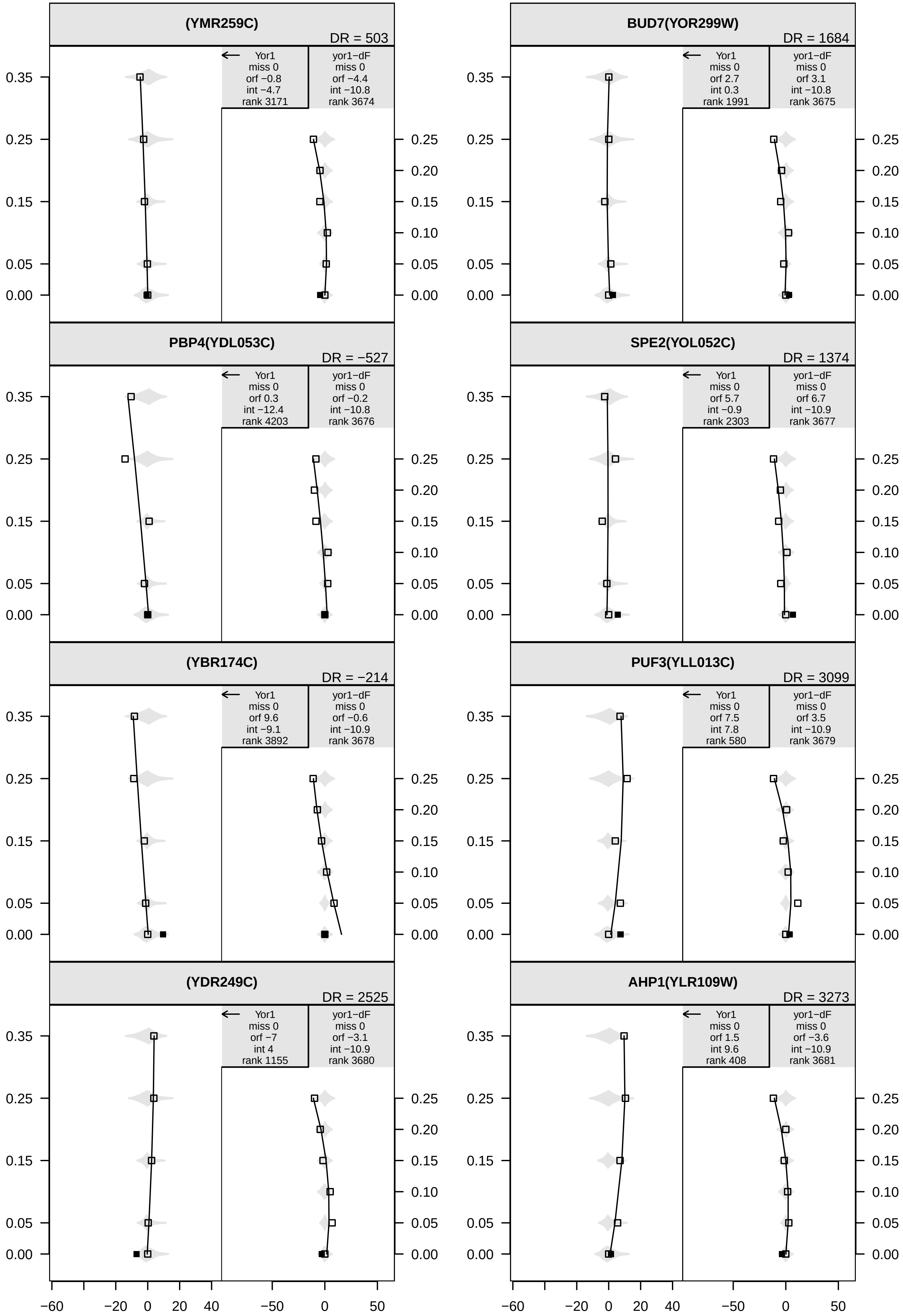

(YMR259C)
DR = 503
Yor1 miss 0 orf −0.8 int −4.7 rank 3171
yor1−dF miss 0 orf −4.4 int −10.8 rank 3674
BUD7(YOR299W)
DR = 1684
Yor1 miss 0 orf 2.7 int 0.3 rank 1991
yor1−dF miss 0 orf 3.1 int −10.8 rank 3675
PBP4(YDL053C)
DR = −527
Yor1 miss 0 orf 0.3 int −12.4 rank 4203
yor1−dF miss 0 orf −0.2 int −10.8 rank 3676
SPE2(YOL052C)
DR = 1374
Yor1 miss 0 orf 5.7 int −0.9 rank 2303
yor1−dF miss 0 orf 6.7 int −10.9 rank 3677
(YBR174C)
DR = −214
Yor1 miss 0 orf 9.6 int −9.1 rank 3892
yor1−dF miss 0 orf −0.6 int −10.9 rank 3678
PUF3(YLL013C)
DR = 3099
Yor1 miss 0 orf 7.5 int 7.8 rank 580
yor1−dF miss 0 orf 3.5 int −10.9 rank 3679
(YDR249C)
DR = 2525
Yor1 miss 0 orf −7 int 4 rank 1155
yor1−dF miss 0 orf −3.1 int −10.9 rank 3680
AHP1(YLR109W)
DR = 3273
Yor1 miss 0 orf 1.5 int 9.6 rank 408
yor1−dF miss 0 orf −3.6 int −10.9 rank 3681
0.35 0.25 0.15 0.05 0.00
0.25 0.20 0.15 0.10 0.05 0.00
−60 −20 0 20 40
−50 0 50

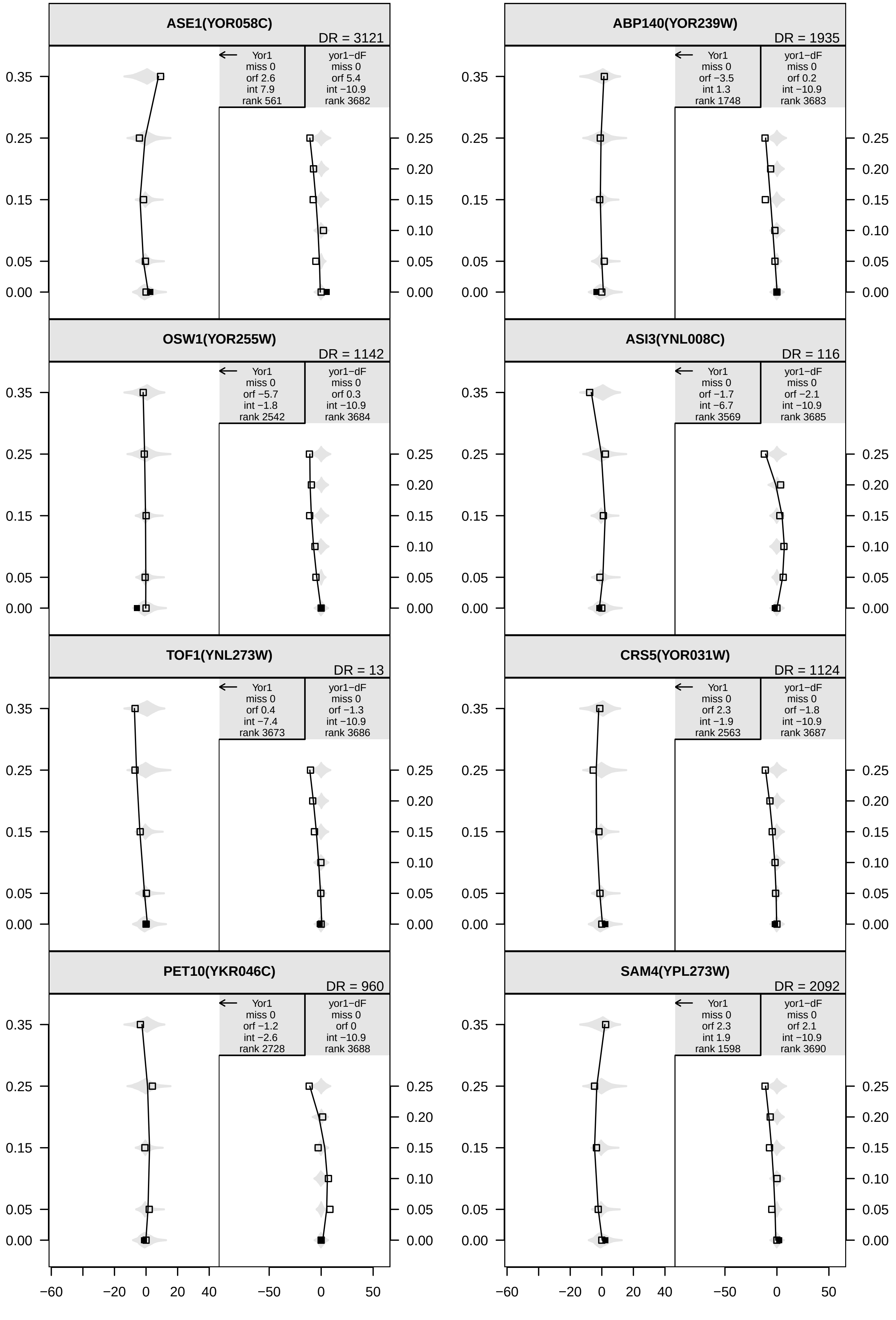

ASE1(YOR058C)
DR = 3121
Yor1 miss 0 orf 2.6 int 7.9 rank 561
yor1−dF miss 0 orf 5.4 int −10.9 rank 3682
ABP140(YOR239W)
DR = 1935
Yor1 miss 0 orf −3.5 int 1.3 rank 1748
yor1−dF miss 0 orf 0.2 int −10.9 rank 3683
OSW1(YOR255W)
DR = 1142
Yor1 miss 0 orf −5.7 int −1.8 rank 2542
yor1−dF miss 0 orf 0.3 int −10.9 rank 3684
ASI3(YNL008C)
DR = 116
Yor1 miss 0 orf −1.7 int −6.7 rank 3569
yor1−dF miss 0 orf −2.1 int −10.9 rank 3685
TOF1(YNL273W)
DR = 13
Yor1 miss 0 orf 0.4 int −7.4 rank 3673
yor1−dF miss 0 orf −1.3 int −10.9 rank 3686
CRS5(YOR031W)
DR = 1124
Yor1 miss 0 orf 2.3 int −1.9 rank 2563
yor1−dF miss 0 orf −1.8 int −10.9 rank 3687
PET10(YKR046C)
DR = 960
Yor1 miss 0 orf −1.2 int −2.6 rank 2728
yor1−dF miss 0 orf 0 int −10.9 rank 3688
SAM4(YPL273W)
DR = 2092
Yor1 miss 0 orf 2.3 int 1.9 rank 1598
yor1−dF miss 0 orf 2.1 int −10.9 rank 3690
0.00 0.05 0.10 0.15 0.20 0.25 0.35
−60 −20 0 20 40
−50 0 50

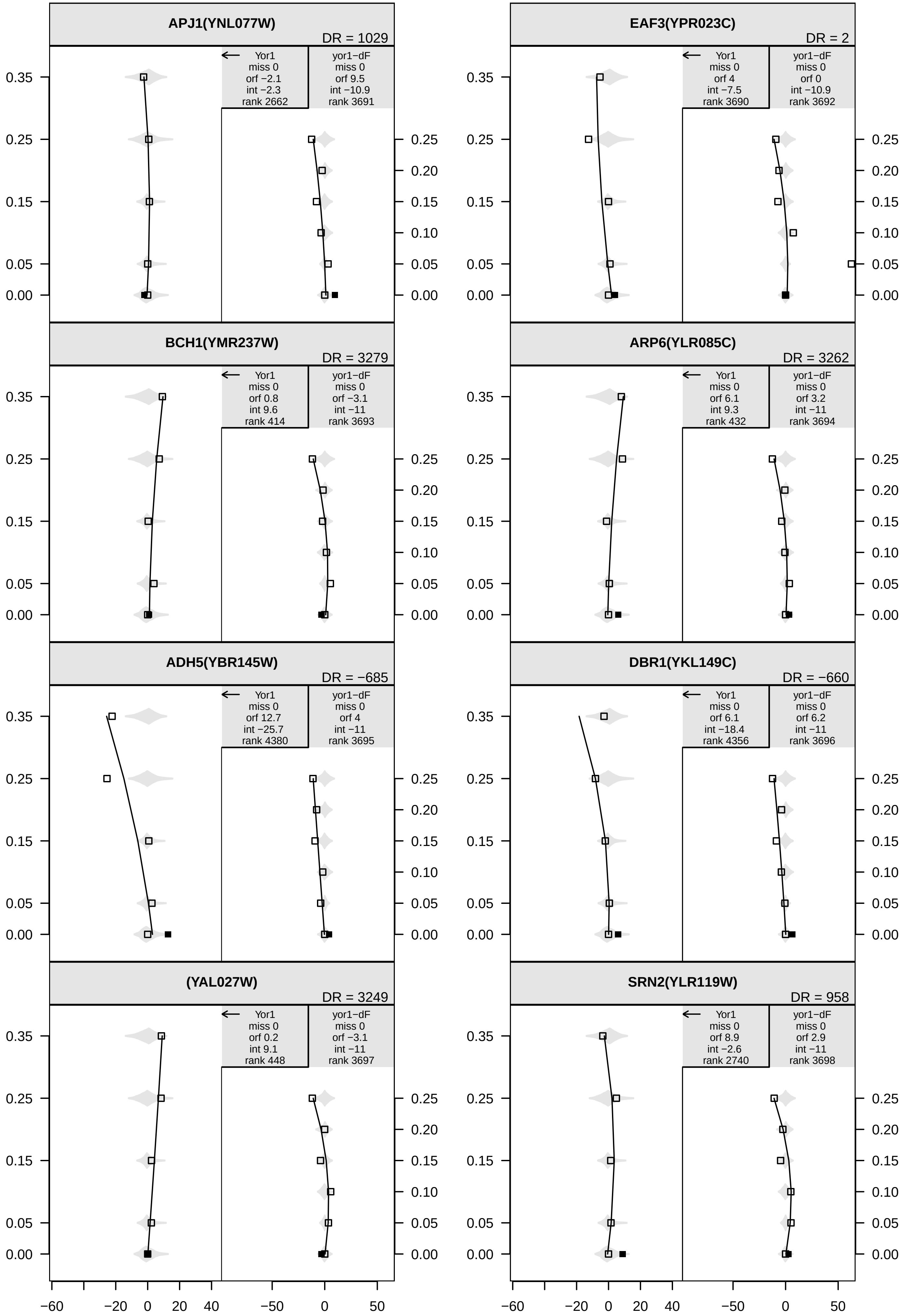

APJ1(YNL077W)
DR = 1029
Yor1
miss 0
orf −2.1
int −2.3
rank 2662
yor1−dF
miss 0
orf 9.5
int −10.9
rank 3691
EAF3(YPR023C)
DR = 2
Yor1
miss 0
orf 4
int −7.5
rank 3690
yor1−dF
miss 0
orf 0
int −10.9
rank 3692
BCH1(YMR237W)
DR = 3279
Yor1
miss 0
orf 0.8
int 9.6
rank 414
yor1−dF
miss 0
orf −3.1
int −11
rank 3693
ARP6(YLR085C)
DR = 3262
Yor1
miss 0
orf 6.1
int 9.3
rank 432
yor1−dF
miss 0
orf 3.2
int −11
rank 3694
ADH5(YBR145W)
DR = −685
Yor1
miss 0
orf 12.7
int −25.7
rank 4380
yor1−dF
miss 0
orf 4
int −11
rank 3695
DBR1(YKL149C)
DR = −660
Yor1
miss 0
orf 6.1
int −18.4
rank 4356
yor1−dF
miss 0
orf 6.2
int −11
rank 3696
(YAL027W)
DR = 3249
Yor1
miss 0
orf 0.2
int 9.1
rank 448
yor1−dF
miss 0
orf −3.1
int −11
rank 3697
SRN2(YLR119W)
DR = 958
Yor1
miss 0
orf 8.9
int −2.6
rank 2740
yor1−dF
miss 0
orf 2.9
int −11
rank 3698
0.35
0.25
0.20
0.15
0.10
0.05
0.00
−60
−20
0
20
40
−50
0
50

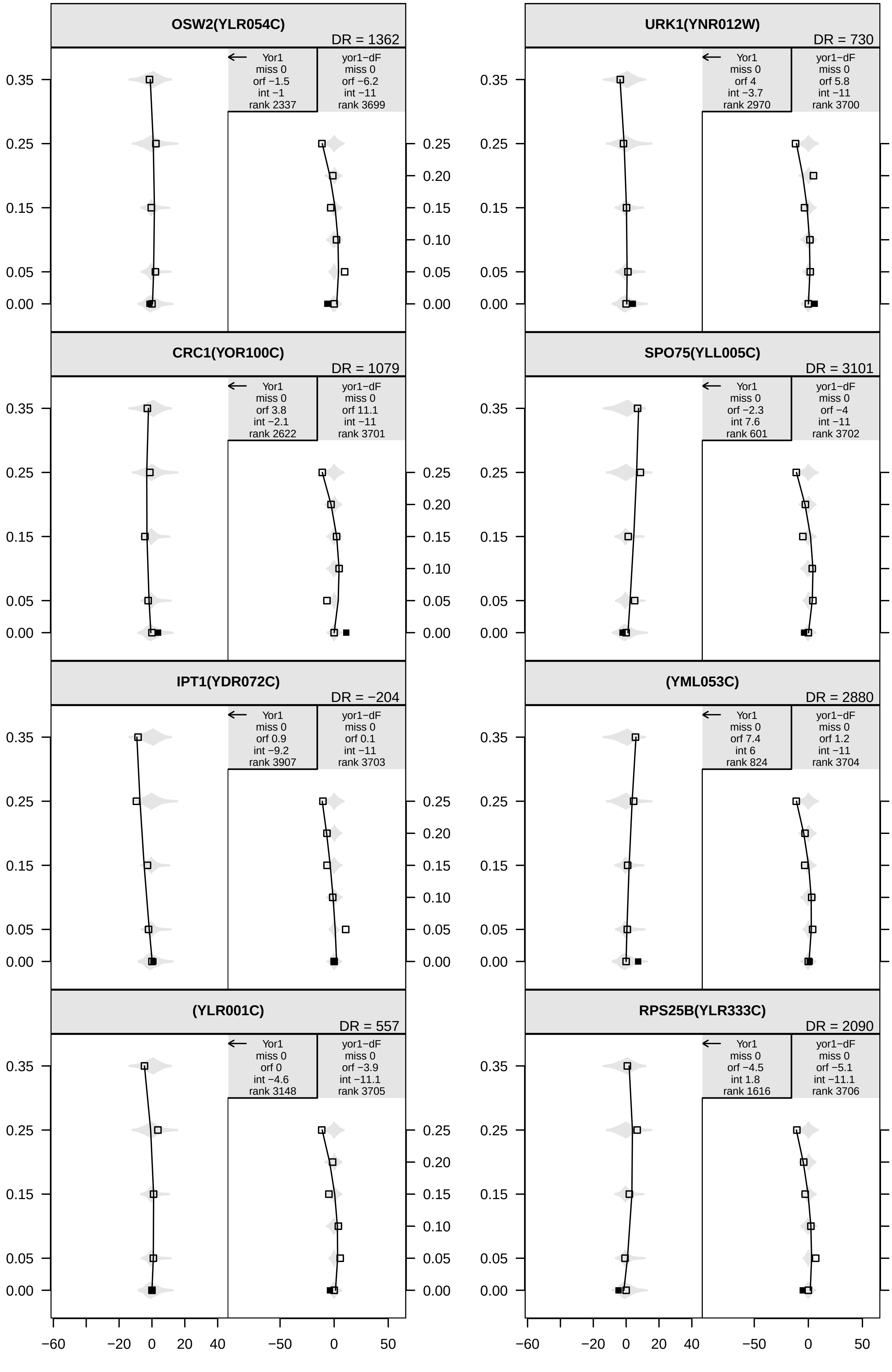
OSW2(YLR054C)
DR = 1362
Yor1
miss 0
orf −1.5
int −1
rank 2337
yor1−dF
miss 0
orf −6.2
int −11
rank 3699
URK1(YNR012W)
DR = 730
Yor1
miss 0
orf 4
int −3.7
rank 2970
yor1−dF
miss 0
orf 5.8
int −11
rank 3700
CRC1(YOR100C)
DR = 1079
Yor1
miss 0
orf 3.8
int −2.1
rank 2622
yor1−dF
miss 0
orf 11.1
int −11
rank 3701
SPO75(YLL005C)
DR = 3101
Yor1
miss 0
orf −2.3
int 7.6
rank 601
yor1−dF
miss 0
orf −4
int −11
rank 3702
IPT1(YDR072C)
DR = −204
Yor1
miss 0
orf 0.9
int −9.2
rank 3907
yor1−dF
miss 0
orf 0.1
int −11
rank 3703
(YML053C)
DR = 2880
Yor1
miss 0
orf 7.4
int 6
rank 824
yor1−dF
miss 0
orf 1.2
int −11
rank 3704
(YLR001C)
DR = 557
Yor1
miss 0
orf 0
int −4.6
rank 3148
yor1−dF
miss 0
orf −3.9
int −11.1
rank 3705
RPS25B(YLR333C)
DR = 2090
Yor1
miss 0
orf −4.5
int 1.8
rank 1616
yor1−dF
miss 0
orf −5.1
int −11.1
rank 3706
0.35
0.25
0.20
0.15
0.10
0.05
0.00
−60
−20
0
20
40
−50
0
50

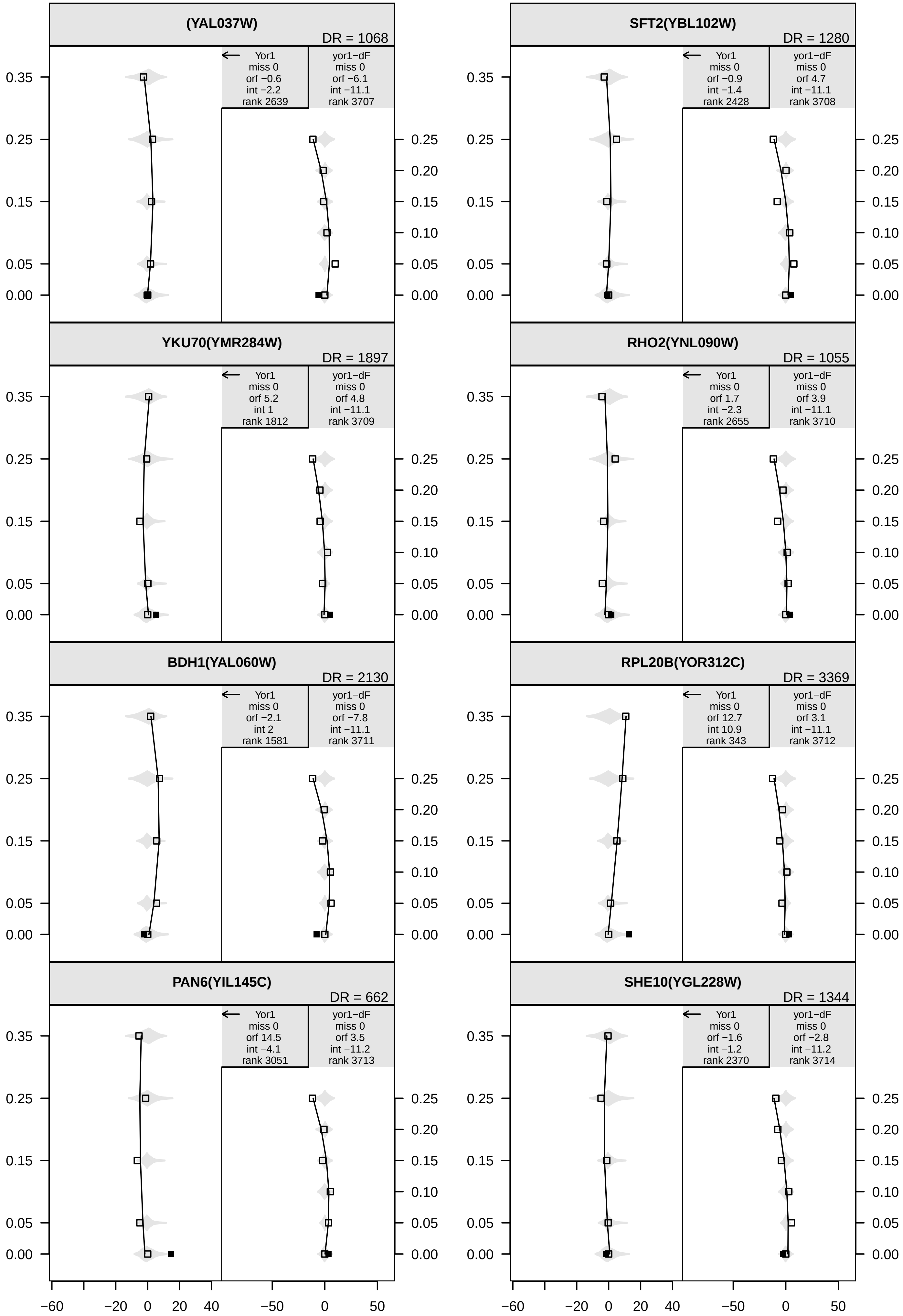
(YAL037W)
DR = 1068
Yor1
miss 0
orf −0.6
int −2.2
rank 2639
yor1−dF
miss 0
orf −6.1
int −11.1
rank 3707
SFT2(YBL102W)
DR = 1280
Yor1
miss 0
orf −0.9
int −1.4
rank 2428
yor1−dF
miss 0
orf 4.7
int −11.1
rank 3708
YKU70(YMR284W)
DR = 1897
Yor1
miss 0
orf 5.2
int 1
rank 1812
yor1−dF
miss 0
orf 4.8
int −11.1
rank 3709
RHO2(YNL090W)
DR = 1055
Yor1
miss 0
orf 1.7
int −2.3
rank 2655
yor1−dF
miss 0
orf 3.9
int −11.1
rank 3710
BDH1(YAL060W)
DR = 2130
Yor1
miss 0
orf −2.1
int 2
rank 1581
yor1−dF
miss 0
orf −7.8
int −11.1
rank 3711
RPL20B(YOR312C)
DR = 3369
Yor1
miss 0
orf 12.7
int 10.9
rank 343
yor1−dF
miss 0
orf 3.1
int −11.1
rank 3712
PAN6(YIL145C)
DR = 662
Yor1
miss 0
orf 14.5
int −4.1
rank 3051
yor1−dF
miss 0
orf 3.5
int −11.2
rank 3713
SHE10(YGL228W)
DR = 1344
Yor1
miss 0
orf −1.6
int −1.2
rank 2370
yor1−dF
miss 0
orf −2.8
int −11.2
rank 3714
0.35
0.25
0.20
0.15
0.10
0.05
0.00
−60
−20
0
20
40
−50
0
50

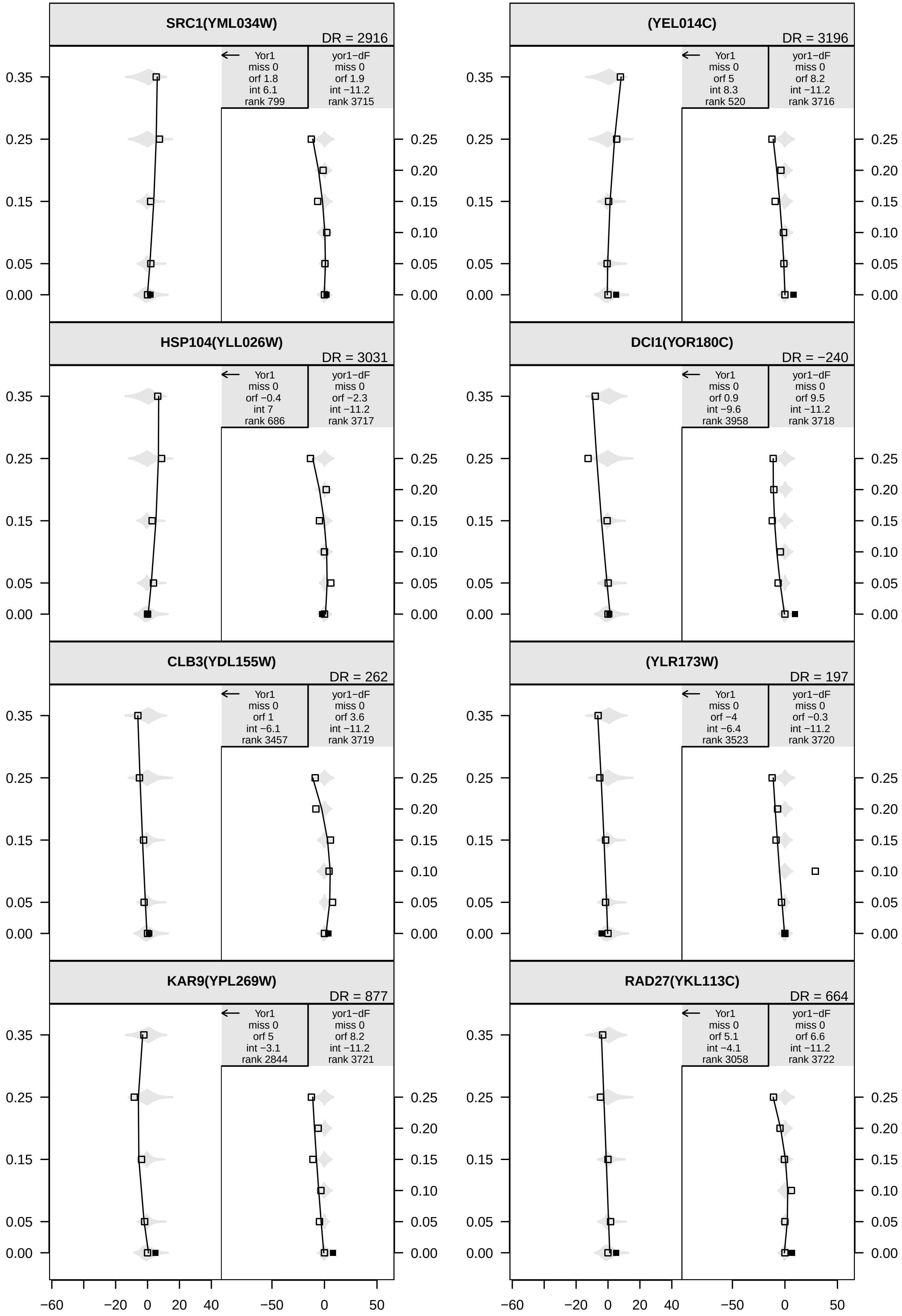
SRC1(YML034W)
DR = 2916
Yor1
miss 0
orf 1.8
int 6.1
rank 799
yor1−dF
miss 0
orf 1.9
int −11.2
rank 3715
(YEL014C)
DR = 3196
Yor1
miss 0
orf 5
int 8.3
rank 520
yor1−dF
miss 0
orf 8.2
int −11.2
rank 3716
HSP104(YLL026W)
DR = 3031
Yor1
miss 0
orf −0.4
int 7
rank 686
yor1−dF
miss 0
orf −2.3
int −11.2
rank 3717
DCI1(YOR180C)
DR = −240
Yor1
miss 0
orf 0.9
int −9.6
rank 3958
yor1−dF
miss 0
orf 9.5
int −11.2
rank 3718
CLB3(YDL155W)
DR = 262
Yor1
miss 0
orf 1
int −6.1
rank 3457
yor1−dF
miss 0
orf 3.6
int −11.2
rank 3719
(YLR173W)
DR = 197
Yor1
miss 0
orf −4
int −6.4
rank 3523
yor1−dF
miss 0
orf −0.3
int −11.2
rank 3720
KAR9(YPL269W)
DR = 877
Yor1
miss 0
orf 5
int −3.1
rank 2844
yor1−dF
miss 0
orf 8.2
int −11.2
rank 3721
RAD27(YKL113C)
DR = 664
Yor1
miss 0
orf 5.1
int −4.1
rank 3058
yor1−dF
miss 0
orf 6.6
int −11.2
rank 3722
0.35
0.25
0.20
0.15
0.10
0.05
0.00
−60
−20
0
20
40
−50
0
50

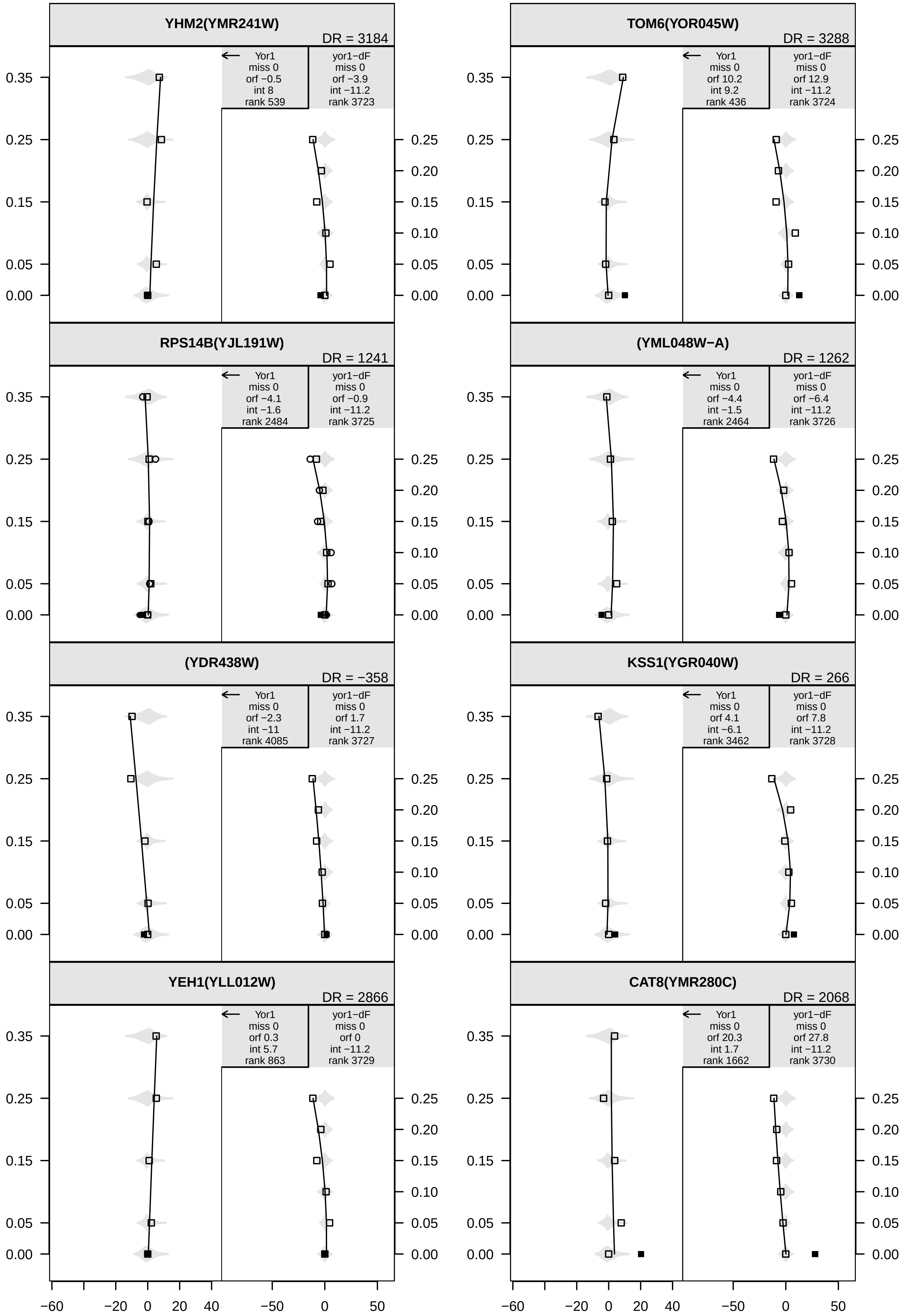

YHM2(YMR241W)
DR = 3184
Yor1 miss 0 orf −0.5 int 8 rank 539
yor1−dF miss 0 orf −3.9 int −11.2 rank 3723
TOM6(YOR045W)
DR = 3288
Yor1 miss 0 orf 10.2 int 9.2 rank 436
yor1−dF miss 0 orf 12.9 int −11.2 rank 3724
RPS14B(YJL191W)
DR = 1241
Yor1 miss 0 orf −4.1 int −1.6 rank 2484
yor1−dF miss 0 orf −0.9 int −11.2 rank 3725
(YML048W−A)
DR = 1262
Yor1 miss 0 orf −4.4 int −1.5 rank 2464
yor1−dF miss 0 orf −6.4 int −11.2 rank 3726
(YDR438W)
DR = −358
Yor1 miss 0 orf −2.3 int −11 rank 4085
yor1−dF miss 0 orf 1.7 int −11.2 rank 3727
KSS1(YGR040W)
DR = 266
Yor1 miss 0 orf 4.1 int −6.1 rank 3462
yor1−dF miss 0 orf 7.8 int −11.2 rank 3728
YEH1(YLL012W)
DR = 2866
Yor1 miss 0 orf 0.3 int 5.7 rank 863
yor1−dF miss 0 orf 0 int −11.2 rank 3729
CAT8(YMR280C)
DR = 2068
Yor1 miss 0 orf 20.3 int 1.7 rank 1662
yor1−dF miss 0 orf 27.8 int −11.2 rank 3730
0.00 0.05 0.10 0.15 0.20 0.25 0.35
−60 −20 0 20 40 −50 0 50

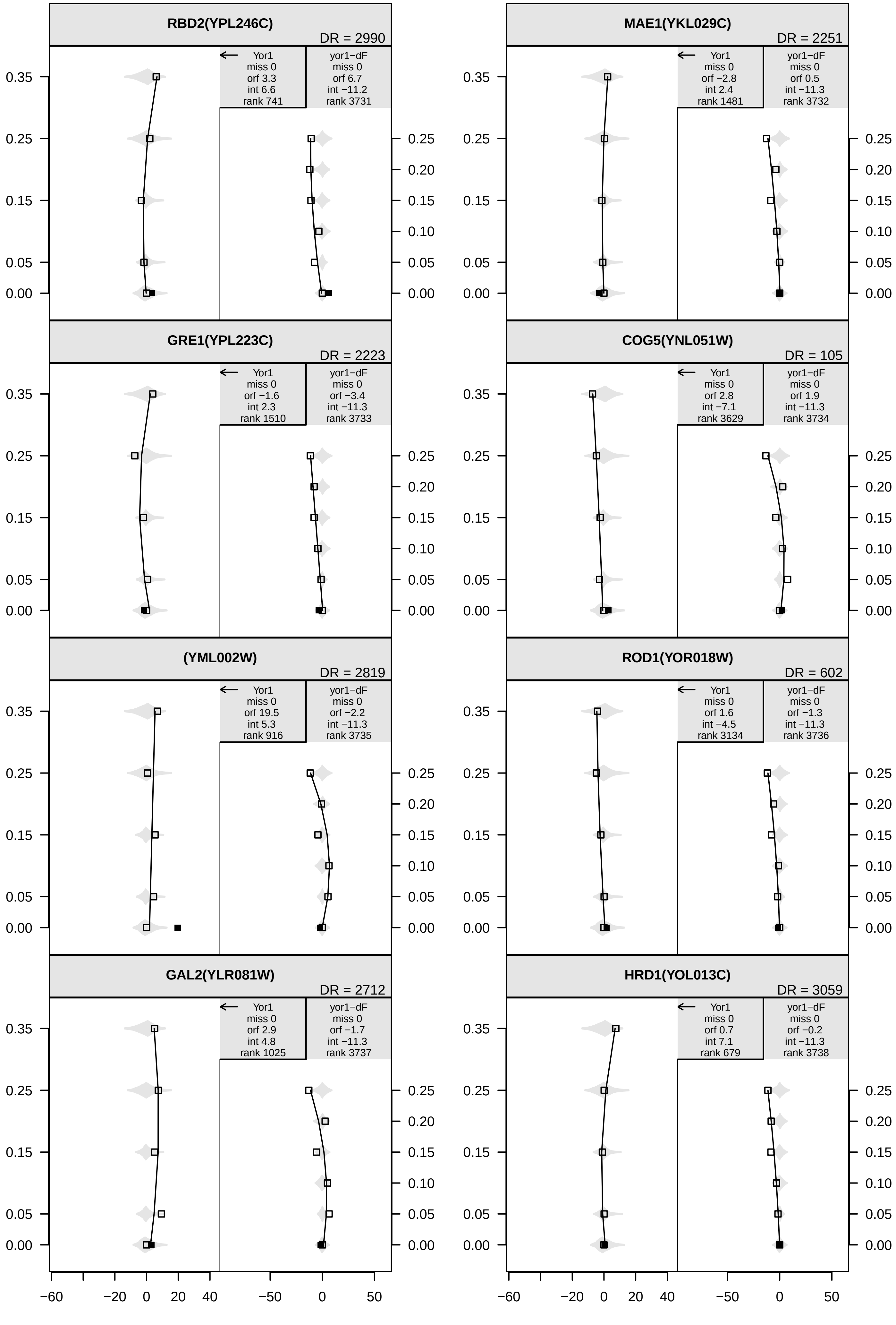

RBD2(YPL246C)
DR = 2990
Yor1 miss 0 orf 3.3 int 6.6 rank 741
yor1−dF miss 0 orf 6.7 int −11.2 rank 3731
MAE1(YKL029C)
DR = 2251
Yor1 miss 0 orf −2.8 int 2.4 rank 1481
yor1−dF miss 0 orf 0.5 int −11.3 rank 3732
GRE1(YPL223C)
DR = 2223
Yor1 miss 0 orf −1.6 int 2.3 rank 1510
yor1−dF miss 0 orf −3.4 int −11.3 rank 3733
COG5(YNL051W)
DR = 105
Yor1 miss 0 orf 2.8 int −7.1 rank 3629
yor1−dF miss 0 orf 1.9 int −11.3 rank 3734
(YML002W)
DR = 2819
Yor1 miss 0 orf 19.5 int 5.3 rank 916
yor1−dF miss 0 orf −2.2 int −11.3 rank 3735
ROD1(YOR018W)
DR = 602
Yor1 miss 0 orf 1.6 int −4.5 rank 3134
yor1−dF miss 0 orf −1.3 int −11.3 rank 3736
GAL2(YLR081W)
DR = 2712
Yor1 miss 0 orf 2.9 int 4.8 rank 1025
yor1−dF miss 0 orf −1.7 int −11.3 rank 3737
HRD1(YOL013C)
DR = 3059
Yor1 miss 0 orf 0.7 int 7.1 rank 679
yor1−dF miss 0 orf −0.2 int −11.3 rank 3738

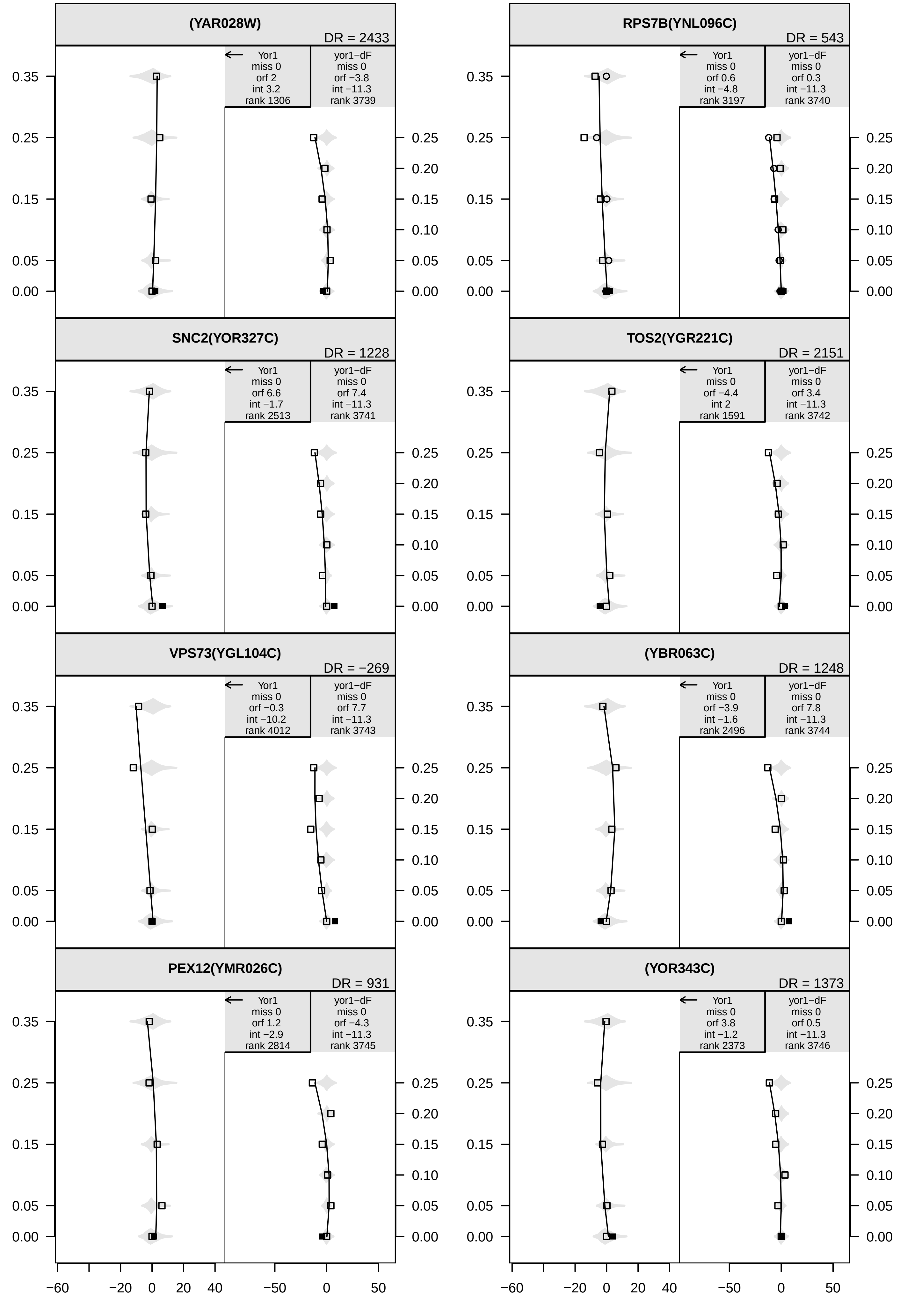

(YAR028W)
DR = 2433
Yor1 miss 0 orf 2 int 3.2 rank 1306
yor1−dF miss 0 orf −3.8 int −11.3 rank 3739
RPS7B(YNL096C)
DR = 543
Yor1 miss 0 orf 0.6 int −4.8 rank 3197
yor1−dF miss 0 orf 0.3 int −11.3 rank 3740
SNC2(YOR327C)
DR = 1228
Yor1 miss 0 orf 6.6 int −1.7 rank 2513
yor1−dF miss 0 orf 7.4 int −11.3 rank 3741
TOS2(YGR221C)
DR = 2151
Yor1 miss 0 orf −4.4 int 2 rank 1591
yor1−dF miss 0 orf 3.4 int −11.3 rank 3742
VPS73(YGL104C)
DR = −269
Yor1 miss 0 orf −0.3 int −10.2 rank 4012
yor1−dF miss 0 orf 7.7 int −11.3 rank 3743
(YBR063C)
DR = 1248
Yor1 miss 0 orf −3.9 int −1.6 rank 2496
yor1−dF miss 0 orf 7.8 int −11.3 rank 3744
PEX12(YMR026C)
DR = 931
Yor1 miss 0 orf 1.2 int −2.9 rank 2814
yor1−dF miss 0 orf −4.3 int −11.3 rank 3745
(YOR343C)
DR = 1373
Yor1 miss 0 orf 3.8 int −1.2 rank 2373
yor1−dF miss 0 orf 0.5 int −11.3 rank 3746
0.35 0.25 0.15 0.05 0.00
0.25 0.20 0.15 0.10 0.05 0.00
−60 −20 0 20 40
−50 0 50

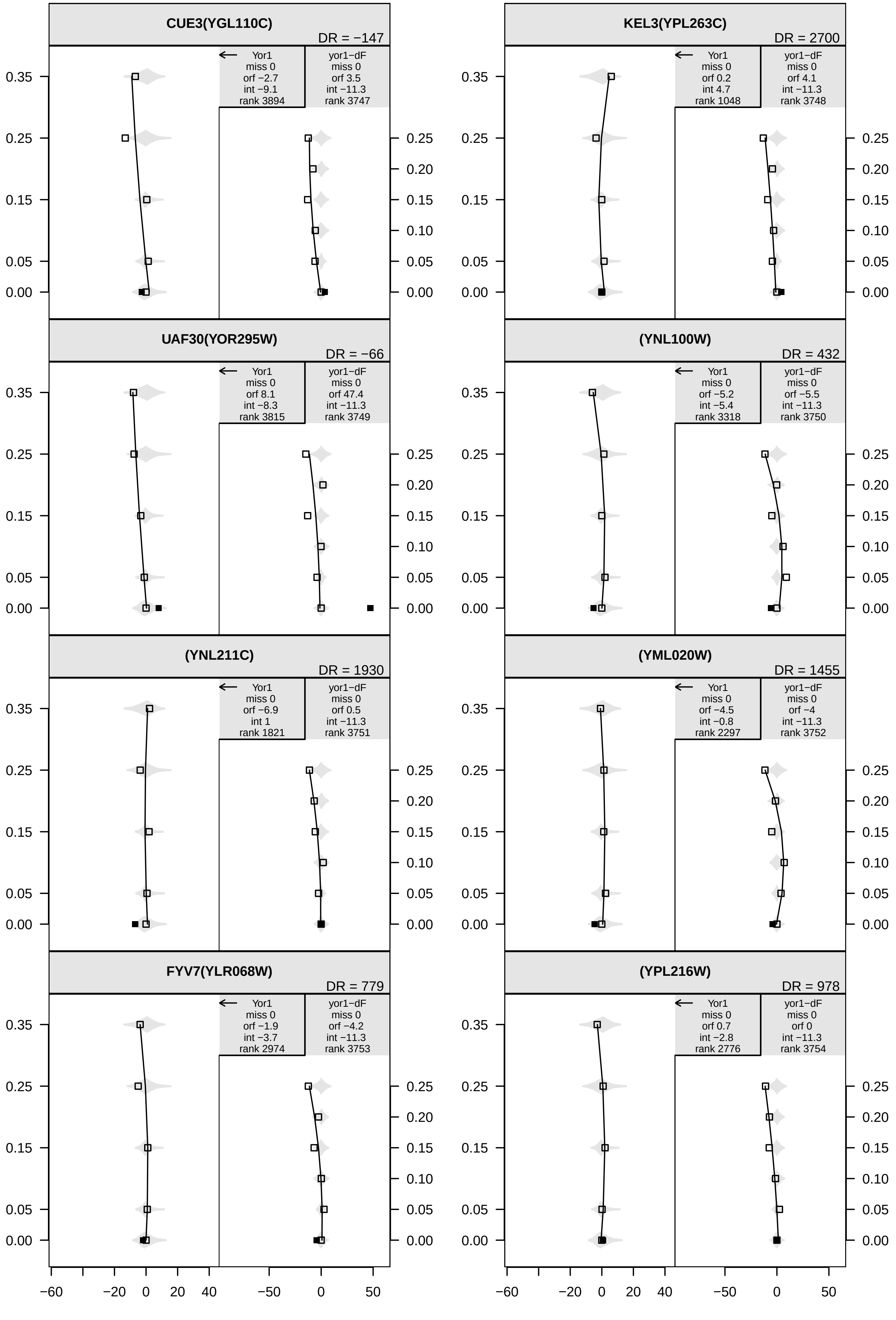

CUE3(YGL110C)
DR = −147
Yor1 miss 0 orf −2.7 int −9.1 rank 3894
yor1−dF miss 0 orf 3.5 int −11.3 rank 3747
KEL3(YPL263C)
DR = 2700
Yor1 miss 0 orf 0.2 int 4.7 rank 1048
yor1−dF miss 0 orf 4.1 int −11.3 rank 3748
UAF30(YOR295W)
DR = −66
Yor1 miss 0 orf 8.1 int −8.3 rank 3815
yor1−dF miss 0 orf 47.4 int −11.3 rank 3749
(YNL100W)
DR = 432
Yor1 miss 0 orf −5.2 int −5.4 rank 3318
yor1−dF miss 0 orf −5.5 int −11.3 rank 3750
(YNL211C)
DR = 1930
Yor1 miss 0 orf −6.9 int 1 rank 1821
yor1−dF miss 0 orf 0.5 int −11.3 rank 3751
(YML020W)
DR = 1455
Yor1 miss 0 orf −4.5 int −0.8 rank 2297
yor1−dF miss 0 orf −4 int −11.3 rank 3752
FYV7(YLR068W)
DR = 779
Yor1 miss 0 orf −1.9 int −3.7 rank 2974
yor1−dF miss 0 orf −4.2 int −11.3 rank 3753
(YPL216W)
DR = 978
Yor1 miss 0 orf 0.7 int −2.8 rank 2776
yor1−dF miss 0 orf 0 int −11.3 rank 3754
0.35 0.25 0.20 0.15 0.10 0.05 0.00
−60 −20 0 20 40 −50 0 50

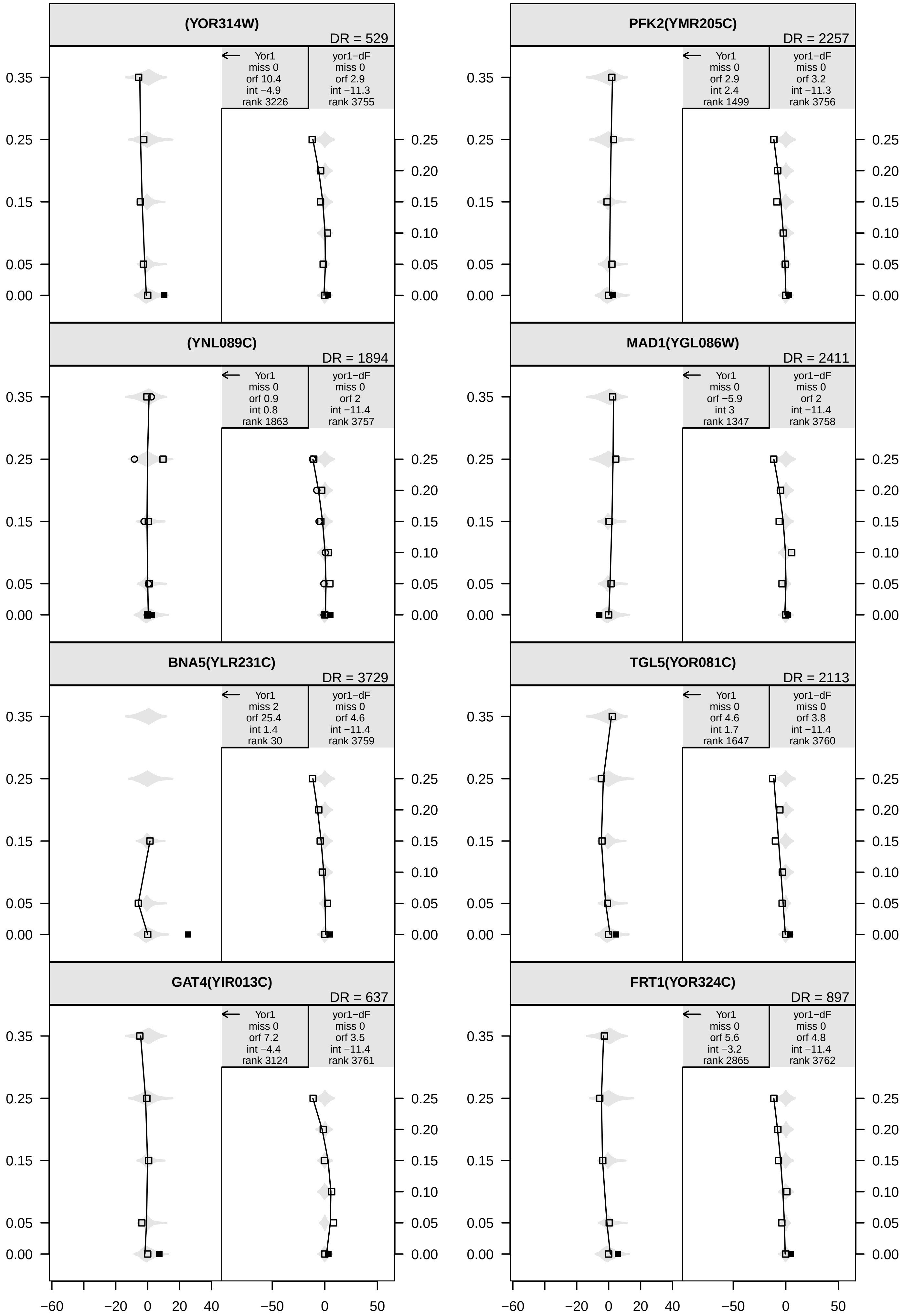

(YOR314W)
DR = 529
Yor1 miss 0 orf 10.4 int −4.9 rank 3226
yor1−dF miss 0 orf 2.9 int −11.3 rank 3755
PFK2(YMR205C)
DR = 2257
Yor1 miss 0 orf 2.9 int 2.4 rank 1499
yor1−dF miss 0 orf 3.2 int −11.3 rank 3756
(YNL089C)
DR = 1894
Yor1 miss 0 orf 0.9 int 0.8 rank 1863
yor1−dF miss 0 orf 2 int −11.4 rank 3757
MAD1(YGL086W)
DR = 2411
Yor1 miss 0 orf −5.9 int 3 rank 1347
yor1−dF miss 0 orf 2 int −11.4 rank 3758
BNA5(YLR231C)
DR = 3729
Yor1 miss 2 orf 25.4 int 1.4 rank 30
yor1−dF miss 0 orf 4.6 int −11.4 rank 3759
TGL5(YOR081C)
DR = 2113
Yor1 miss 0 orf 4.6 int 1.7 rank 1647
yor1−dF miss 0 orf 3.8 int −11.4 rank 3760
GAT4(YIR013C)
DR = 637
Yor1 miss 0 orf 7.2 int −4.4 rank 3124
yor1−dF miss 0 orf 3.5 int −11.4 rank 3761
FRT1(YOR324C)
DR = 897
Yor1 miss 0 orf 5.6 int −3.2 rank 2865
yor1−dF miss 0 orf 4.8 int −11.4 rank 3762
0.35 0.25 0.20 0.15 0.10 0.05 0.00
−60 −20 0 20 40 −50 0 50

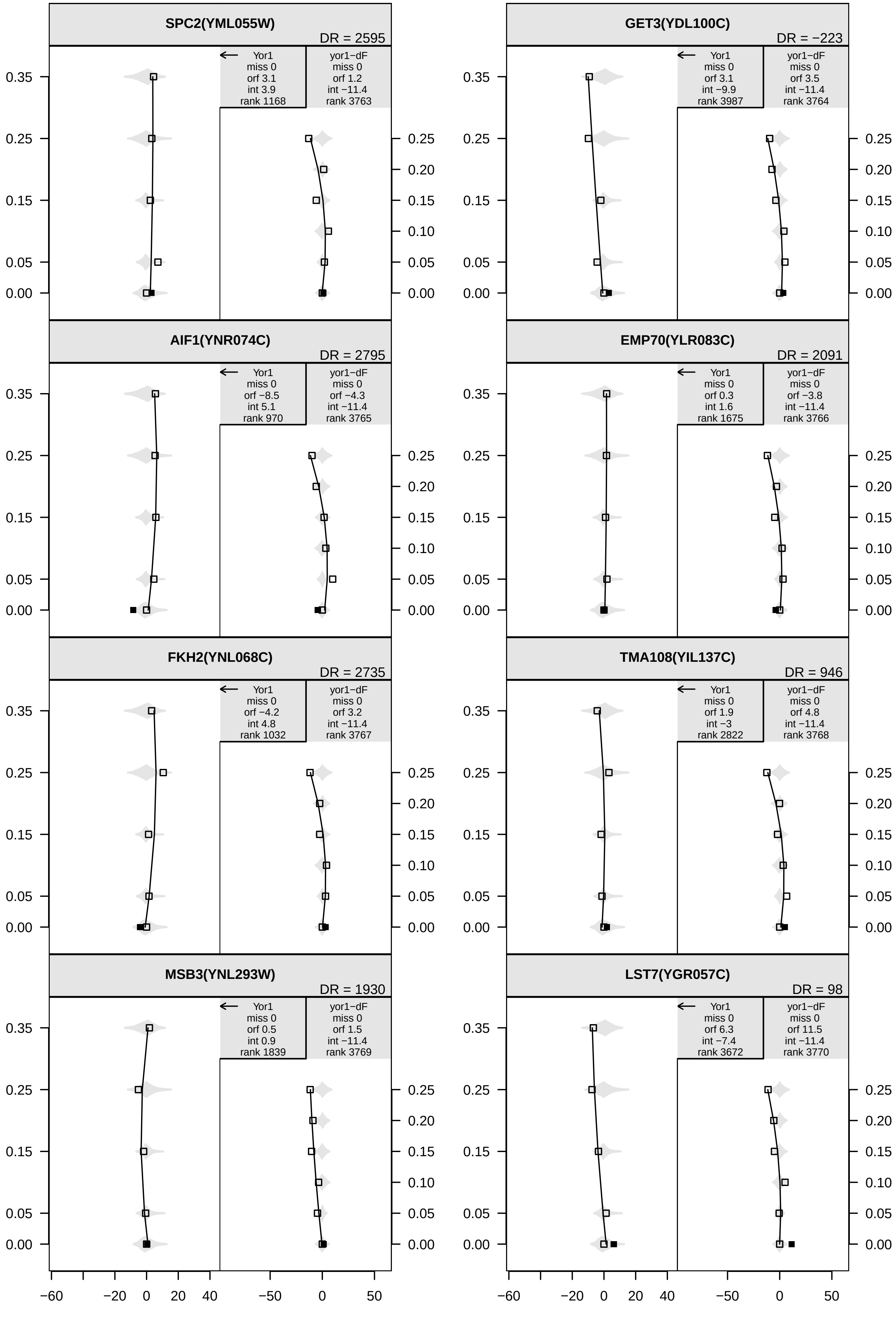

SPC2(YML055W)
DR = 2595
Yor1 miss 0 orf 3.1 int 3.9 rank 1168
yor1−dF miss 0 orf 1.2 int −11.4 rank 3763
GET3(YDL100C)
DR = −223
Yor1 miss 0 orf 3.1 int −9.9 rank 3987
yor1−dF miss 0 orf 3.5 int −11.4 rank 3764
AIF1(YNR074C)
DR = 2795
Yor1 miss 0 orf −8.5 int 5.1 rank 970
yor1−dF miss 0 orf −4.3 int −11.4 rank 3765
EMP70(YLR083C)
DR = 2091
Yor1 miss 0 orf 0.3 int 1.6 rank 1675
yor1−dF miss 0 orf −3.8 int −11.4 rank 3766
FKH2(YNL068C)
DR = 2735
Yor1 miss 0 orf −4.2 int 4.8 rank 1032
yor1−dF miss 0 orf 3.2 int −11.4 rank 3767
TMA108(YIL137C)
DR = 946
Yor1 miss 0 orf 1.9 int −3 rank 2822
yor1−dF miss 0 orf 4.8 int −11.4 rank 3768
MSB3(YNL293W)
DR = 1930
Yor1 miss 0 orf 0.5 int 0.9 rank 1839
yor1−dF miss 0 orf 1.5 int −11.4 rank 3769
LST7(YGR057C)
DR = 98
Yor1 miss 0 orf 6.3 int −7.4 rank 3672
yor1−dF miss 0 orf 11.5 int −11.4 rank 3770
0.35 0.25 0.15 0.05 0.00
0.25 0.20 0.15 0.10 0.05 0.00
−60 −20 0 20 40 −50 0 50

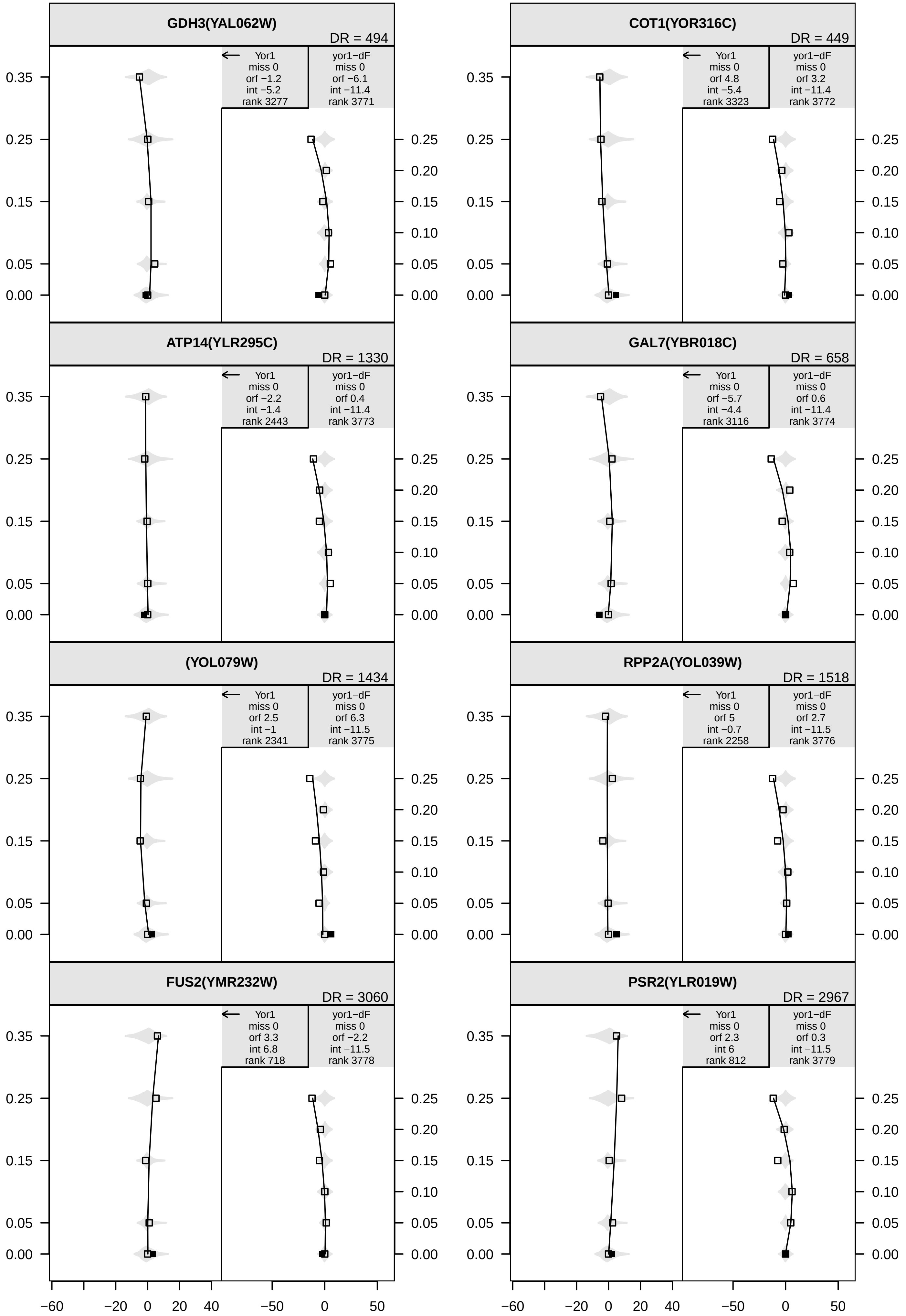

GDH3(YAL062W)
DR = 494
Yor1
miss 0
orf −1.2
int −5.2
rank 3277
yor1−dF
miss 0
orf −6.1
int −11.4
rank 3771
COT1(YOR316C)
DR = 449
Yor1
miss 0
orf 4.8
int −5.4
rank 3323
yor1−dF
miss 0
orf 3.2
int −11.4
rank 3772
ATP14(YLR295C)
DR = 1330
Yor1
miss 0
orf −2.2
int −1.4
rank 2443
yor1−dF
miss 0
orf 0.4
int −11.4
rank 3773
GAL7(YBR018C)
DR = 658
Yor1
miss 0
orf −5.7
int −4.4
rank 3116
yor1−dF
miss 0
orf 0.6
int −11.4
rank 3774
(YOL079W)
DR = 1434
Yor1
miss 0
orf 2.5
int −1
rank 2341
yor1−dF
miss 0
orf 6.3
int −11.5
rank 3775
RPP2A(YOL039W)
DR = 1518
Yor1
miss 0
orf 5
int −0.7
rank 2258
yor1−dF
miss 0
orf 2.7
int −11.5
rank 3776
FUS2(YMR232W)
DR = 3060
Yor1
miss 0
orf 3.3
int 6.8
rank 718
yor1−dF
miss 0
orf −2.2
int −11.5
rank 3778
PSR2(YLR019W)
DR = 2967
Yor1
miss 0
orf 2.3
int 6
rank 812
yor1−dF
miss 0
orf 0.3
int −11.5
rank 3779
0.35
0.25
0.20
0.15
0.10
0.05
0.00
−60
−20
0
20
40
−50
0
50

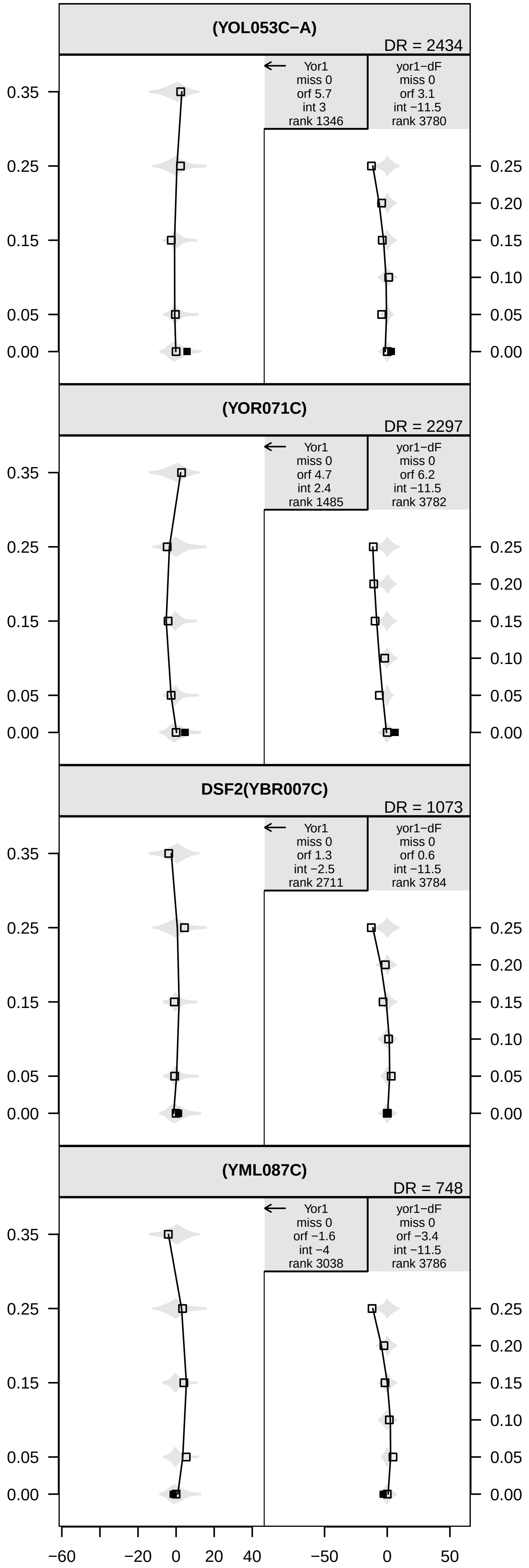

(YOL053C−A)
DR = 2434
Yor1
miss 0
orf 5.7
int 3
rank 1346
yor1−dF
miss 0
orf 3.1
int −11.5
rank 3780
(YLR269C)
DR = 2153
Yor1
miss 0
orf 7.9
int 1.8
rank 1628
yor1−dF
miss 0
orf 5.6
int −11.5
rank 3781
(YOR071C)
DR = 2297
Yor1
miss 0
orf 4.7
int 2.4
rank 1485
yor1−dF
miss 0
orf 6.2
int −11.5
rank 3782
(YLR125W)
DR = 629
Yor1
miss 0
orf 6.7
int −4.6
rank 3154
yor1−dF
miss 0
orf −4.6
int −11.5
rank 3783
DSF2(YBR007C)
DR = 1073
Yor1
miss 0
orf 1.3
int −2.5
rank 2711
yor1−dF
miss 0
orf 0.6
int −11.5
rank 3784
(YLR073C)
DR = 3247
Yor1
miss 0
orf 12.3
int 8
rank 538
yor1−dF
miss 0
orf −0.1
int −11.5
rank 3785
(YML087C)
DR = 748
Yor1
miss 0
orf −1.6
int −4
rank 3038
yor1−dF
miss 0
orf −3.4
int −11.5
rank 3786
(YMR034C)
DR = 2599
Yor1
miss 0
orf −6
int 3.8
rank 1188
yor1−dF
miss 0
orf −7
int −11.5
rank 3787
0.35
0.25
0.20
0.15
0.10
0.05
0.00
−60
−20
0
20
40
−50
0
50

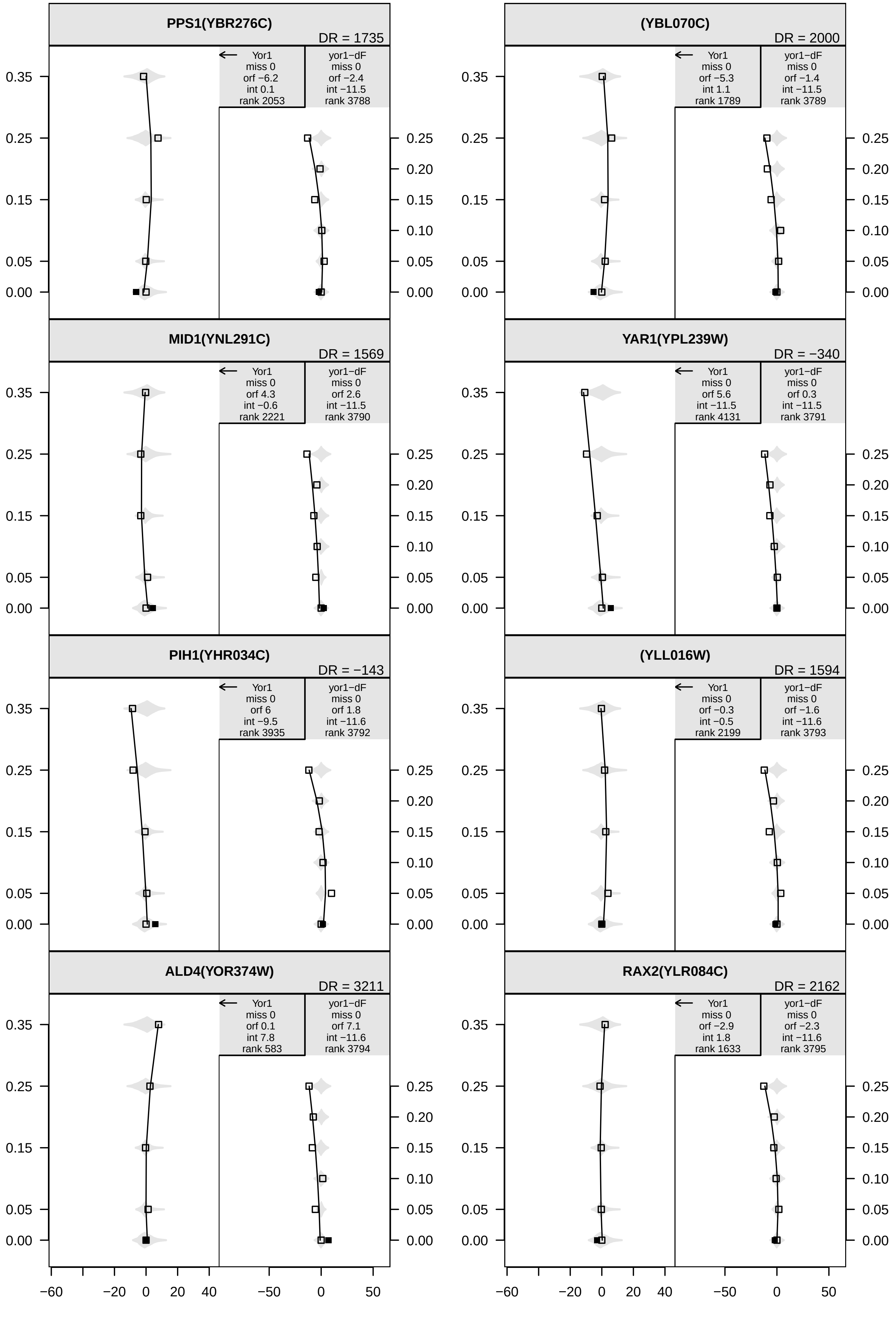

PPS1(YBR276C)
DR = 1735
Yor1 miss 0 orf −6.2 int 0.1 rank 2053
yor1−dF miss 0 orf −2.4 int −11.5 rank 3788
(YBL070C)
DR = 2000
Yor1 miss 0 orf −5.3 int 1.1 rank 1789
yor1−dF miss 0 orf −1.4 int −11.5 rank 3789
MID1(YNL291C)
DR = 1569
Yor1 miss 0 orf 4.3 int −0.6 rank 2221
yor1−dF miss 0 orf 2.6 int −11.5 rank 3790
YAR1(YPL239W)
DR = −340
Yor1 miss 0 orf 5.6 int −11.5 rank 4131
yor1−dF miss 0 orf 0.3 int −11.5 rank 3791
PIH1(YHR034C)
DR = −143
Yor1 miss 0 orf 6 int −9.5 rank 3935
yor1−dF miss 0 orf 1.8 int −11.6 rank 3792
(YLL016W)
DR = 1594
Yor1 miss 0 orf −0.3 int −0.5 rank 2199
yor1−dF miss 0 orf −1.6 int −11.6 rank 3793
ALD4(YOR374W)
DR = 3211
Yor1 miss 0 orf 0.1 int 7.8 rank 583
yor1−dF miss 0 orf 7.1 int −11.6 rank 3794
RAX2(YLR084C)
DR = 2162
Yor1 miss 0 orf −2.9 int 1.8 rank 1633
yor1−dF miss 0 orf −2.3 int −11.6 rank 3795
0.00 0.05 0.10 0.15 0.20 0.25 0.35
−60 −20 0 20 40 −50 0 50

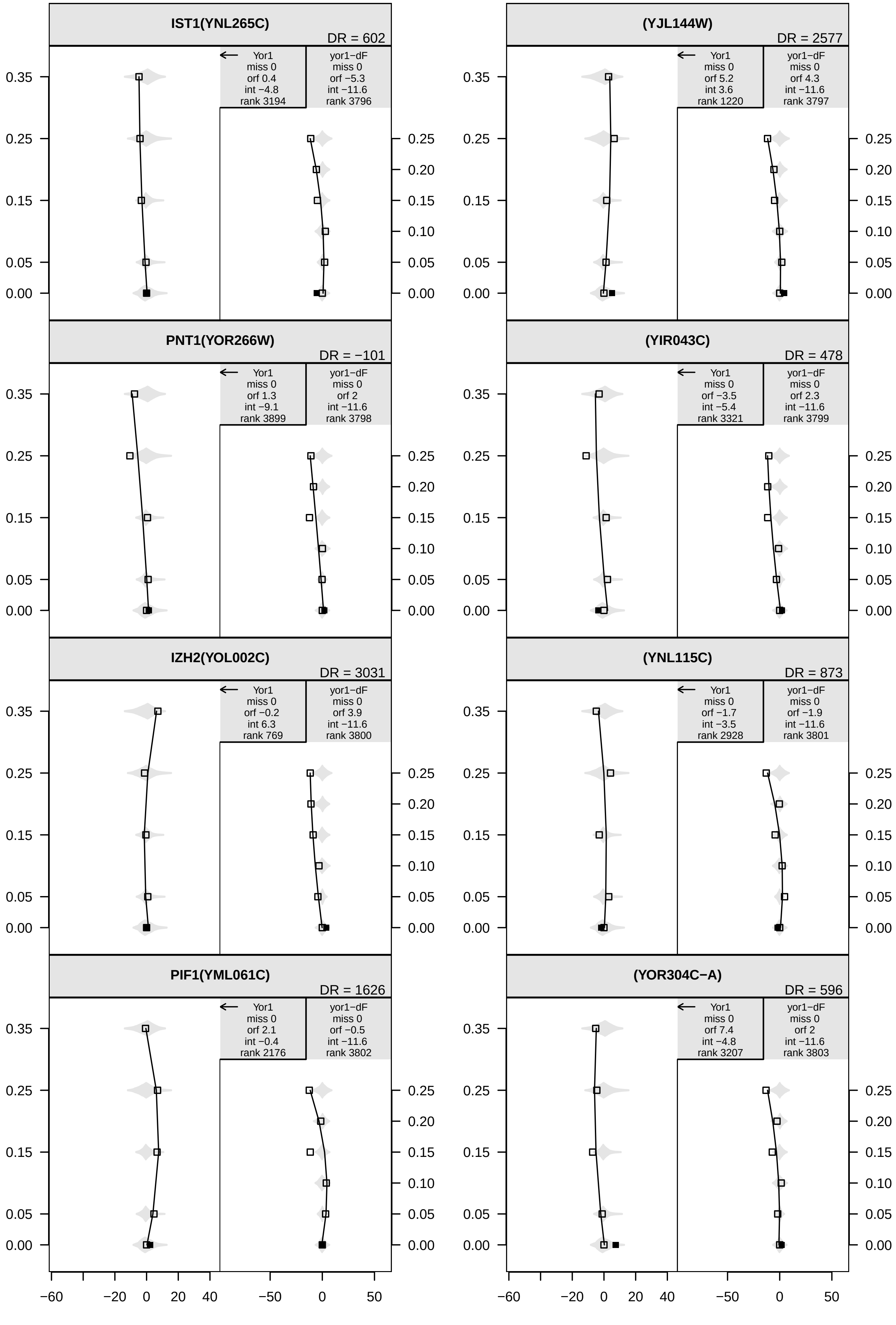
IST1(YNL265C)
DR = 602
Yor1
miss 0
orf 0.4
int −4.8
rank 3194
yor1−dF
miss 0
orf −5.3
int −11.6
rank 3796
(YJL144W)
DR = 2577
Yor1
miss 0
orf 5.2
int 3.6
rank 1220
yor1−dF
miss 0
orf 4.3
int −11.6
rank 3797
PNT1(YOR266W)
DR = −101
Yor1
miss 0
orf 1.3
int −9.1
rank 3899
yor1−dF
miss 0
orf 2
int −11.6
rank 3798
(YIR043C)
DR = 478
Yor1
miss 0
orf −3.5
int −5.4
rank 3321
yor1−dF
miss 0
orf 2.3
int −11.6
rank 3799
IZH2(YOL002C)
DR = 3031
Yor1
miss 0
orf −0.2
int 6.3
rank 769
yor1−dF
miss 0
orf 3.9
int −11.6
rank 3800
(YNL115C)
DR = 873
Yor1
miss 0
orf −1.7
int −3.5
rank 2928
yor1−dF
miss 0
orf −1.9
int −11.6
rank 3801
PIF1(YML061C)
DR = 1626
Yor1
miss 0
orf 2.1
int −0.4
rank 2176
yor1−dF
miss 0
orf −0.5
int −11.6
rank 3802
(YOR304C−A)
DR = 596
Yor1
miss 0
orf 7.4
int −4.8
rank 3207
yor1−dF
miss 0
orf 2
int −11.6
rank 3803
0.35
0.25
0.20
0.15
0.10
0.05
0.00
−60
−20
0
20
40
−50
0
50

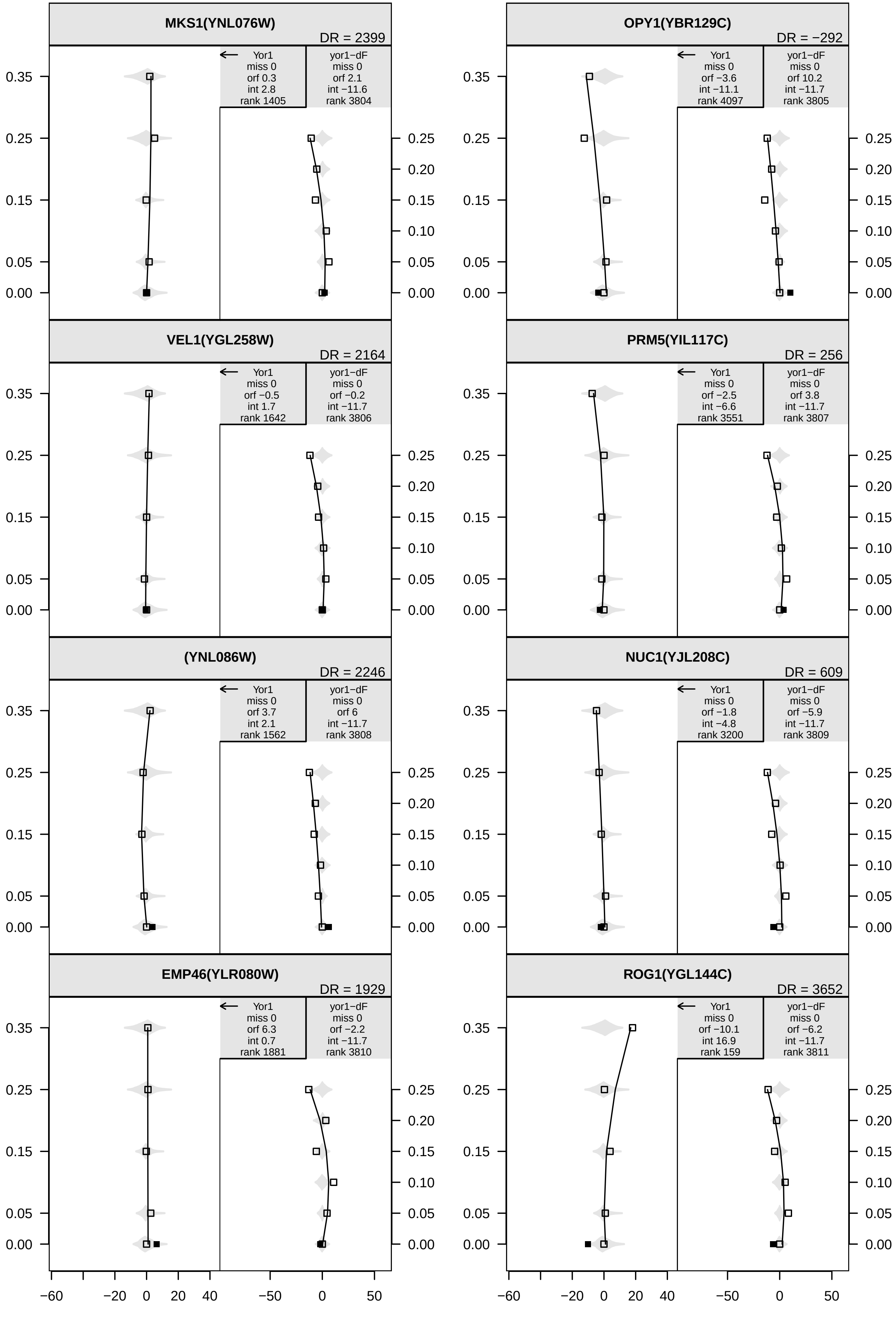
MKS1(YNL076W)
DR = 2399
Yor1
miss 0
orf 0.3
int 2.8
rank 1405
yor1−dF
miss 0
orf 2.1
int −11.6
rank 3804
OPY1(YBR129C)
DR = −292
Yor1
miss 0
orf −3.6
int −11.1
rank 4097
yor1−dF
miss 0
orf 10.2
int −11.7
rank 3805
VEL1(YGL258W)
DR = 2164
Yor1
miss 0
orf −0.5
int 1.7
rank 1642
yor1−dF
miss 0
orf −0.2
int −11.7
rank 3806
PRM5(YIL117C)
DR = 256
Yor1
miss 0
orf −2.5
int −6.6
rank 3551
yor1−dF
miss 0
orf 3.8
int −11.7
rank 3807
(YNL086W)
DR = 2246
Yor1
miss 0
orf 3.7
int 2.1
rank 1562
yor1−dF
miss 0
orf 6
int −11.7
rank 3808
NUC1(YJL208C)
DR = 609
Yor1
miss 0
orf −1.8
int −4.8
rank 3200
yor1−dF
miss 0
orf −5.9
int −11.7
rank 3809
EMP46(YLR080W)
DR = 1929
Yor1
miss 0
orf 6.3
int 0.7
rank 1881
yor1−dF
miss 0
orf −2.2
int −11.7
rank 3810
ROG1(YGL144C)
DR = 3652
Yor1
miss 0
orf −10.1
int 16.9
rank 159
yor1−dF
miss 0
orf −6.2
int −11.7
rank 3811
0.35
0.25
0.20
0.15
0.10
0.05
0.00
−60
−20
0
20
40
−50
0
50

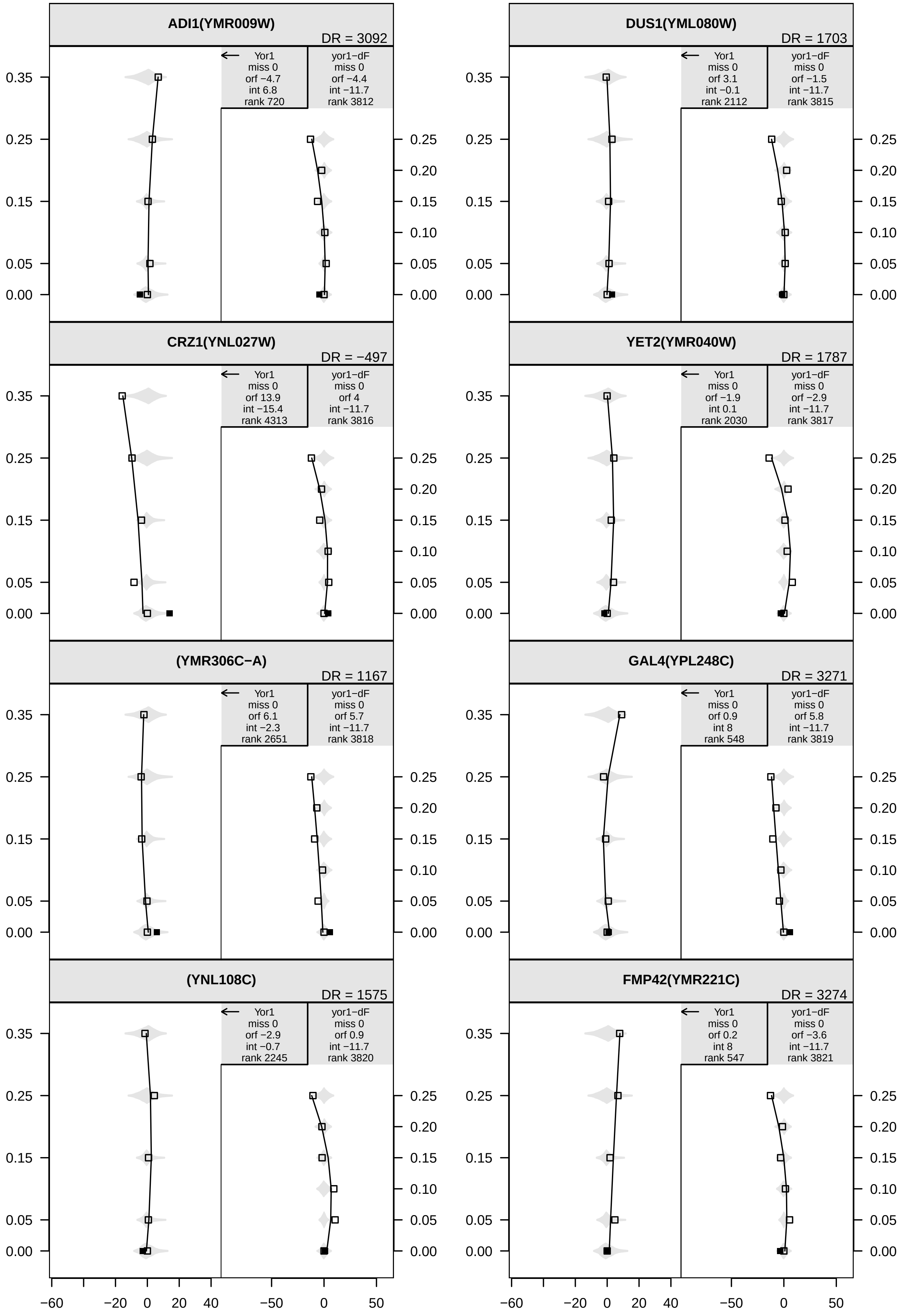
ADI1(YMR009W)
DR = 3092
Yor1 miss 0 orf −4.7 int 6.8 rank 720
yor1−dF miss 0 orf −4.4 int −11.7 rank 3812
DUS1(YML080W)
DR = 1703
Yor1 miss 0 orf 3.1 int −0.1 rank 2112
yor1−dF miss 0 orf −1.5 int −11.7 rank 3815
CRZ1(YNL027W)
DR = −497
Yor1 miss 0 orf 13.9 int −15.4 rank 4313
yor1−dF miss 0 orf 4 int −11.7 rank 3816
YET2(YMR040W)
DR = 1787
Yor1 miss 0 orf −1.9 int 0.1 rank 2030
yor1−dF miss 0 orf −2.9 int −11.7 rank 3817
(YMR306C−A)
DR = 1167
Yor1 miss 0 orf 6.1 int −2.3 rank 2651
yor1−dF miss 0 orf 5.7 int −11.7 rank 3818
GAL4(YPL248C)
DR = 3271
Yor1 miss 0 orf 0.9 int 8 rank 548
yor1−dF miss 0 orf 5.8 int −11.7 rank 3819
(YNL108C)
DR = 1575
Yor1 miss 0 orf −2.9 int −0.7 rank 2245
yor1−dF miss 0 orf 0.9 int −11.7 rank 3820
FMP42(YMR221C)
DR = 3274
Yor1 miss 0 orf 0.2 int 8 rank 547
yor1−dF miss 0 orf −3.6 int −11.7 rank 3821
0.35 0.25 0.20 0.15 0.10 0.05 0.00
−60 −20 0 20 40 −50 0 50

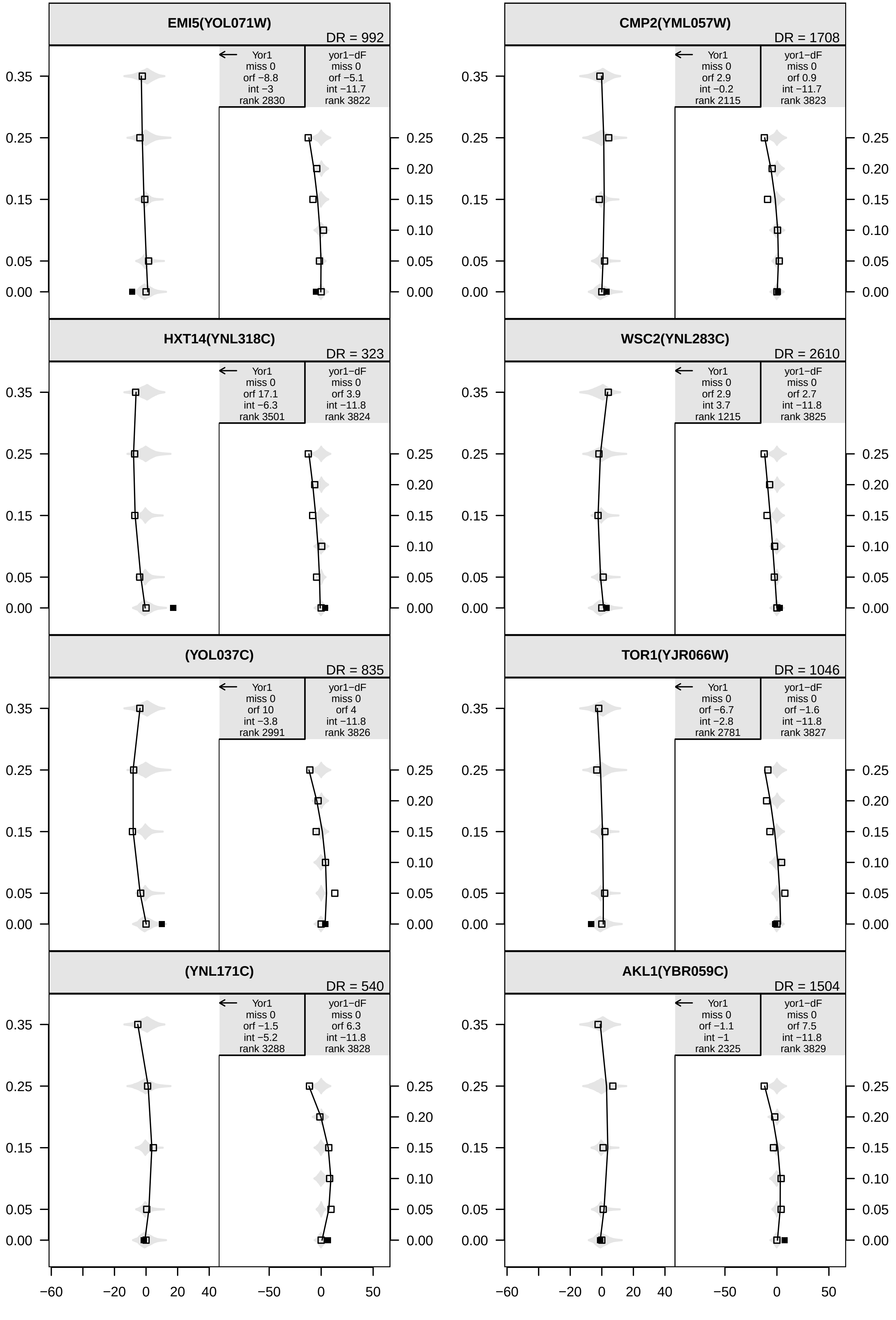
EMI5(YOL071W)
DR = 992
Yor1 miss 0 orf −8.8 int −3 rank 2830
yor1−dF miss 0 orf −5.1 int −11.7 rank 3822
CMP2(YML057W)
DR = 1708
Yor1 miss 0 orf 2.9 int −0.2 rank 2115
yor1−dF miss 0 orf 0.9 int −11.7 rank 3823
HXT14(YNL318C)
DR = 323
Yor1 miss 0 orf 17.1 int −6.3 rank 3501
yor1−dF miss 0 orf 3.9 int −11.8 rank 3824
WSC2(YNL283C)
DR = 2610
Yor1 miss 0 orf 2.9 int 3.7 rank 1215
yor1−dF miss 0 orf 2.7 int −11.8 rank 3825
(YOL037C)
DR = 835
Yor1 miss 0 orf 10 int −3.8 rank 2991
yor1−dF miss 0 orf 4 int −11.8 rank 3826
TOR1(YJR066W)
DR = 1046
Yor1 miss 0 orf −6.7 int −2.8 rank 2781
yor1−dF miss 0 orf −1.6 int −11.8 rank 3827
(YNL171C)
DR = 540
Yor1 miss 0 orf −1.5 int −5.2 rank 3288
yor1−dF miss 0 orf 6.3 int −11.8 rank 3828
AKL1(YBR059C)
DR = 1504
Yor1 miss 0 orf −1.1 int −1 rank 2325
yor1−dF miss 0 orf 7.5 int −11.8 rank 3829
0.00 0.05 0.15 0.25 0.35
0.00 0.05 0.10 0.15 0.20 0.25
−60 −20 0 20 40
−50 0 50

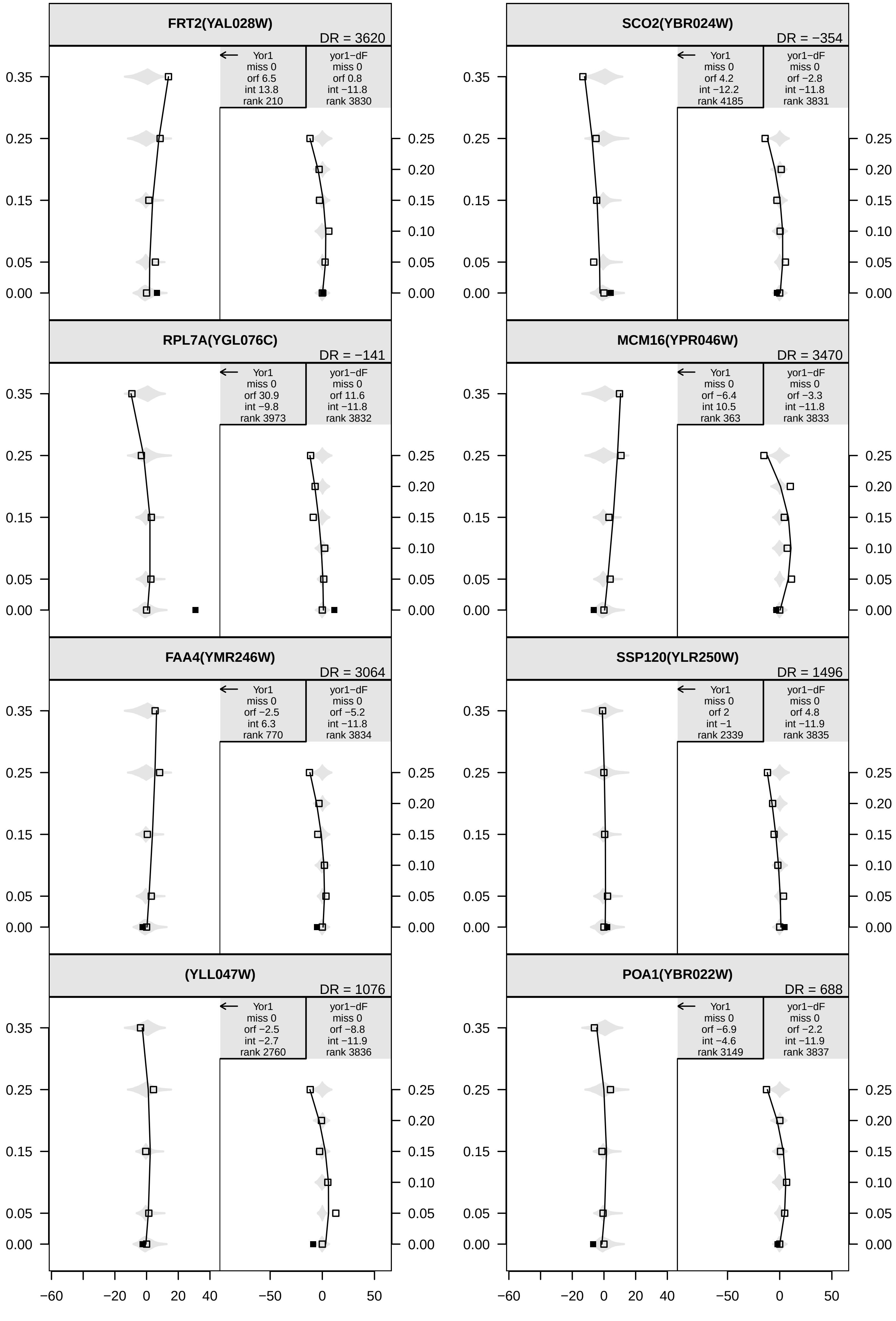

FRT2(YAL028W)
DR = 3620
Yor1
miss 0
orf 6.5
int 13.8
rank 210
yor1−dF
miss 0
orf 0.8
int −11.8
rank 3830
SCO2(YBR024W)
DR = −354
Yor1
miss 0
orf 4.2
int −12.2
rank 4185
yor1−dF
miss 0
orf −2.8
int −11.8
rank 3831
RPL7A(YGL076C)
DR = −141
Yor1
miss 0
orf 30.9
int −9.8
rank 3973
yor1−dF
miss 0
orf 11.6
int −11.8
rank 3832
MCM16(YPR046W)
DR = 3470
Yor1
miss 0
orf −6.4
int 10.5
rank 363
yor1−dF
miss 0
orf −3.3
int −11.8
rank 3833
FAA4(YMR246W)
DR = 3064
Yor1
miss 0
orf −2.5
int 6.3
rank 770
yor1−dF
miss 0
orf −5.2
int −11.8
rank 3834
SSP120(YLR250W)
DR = 1496
Yor1
miss 0
orf 2
int −1
rank 2339
yor1−dF
miss 0
orf 4.8
int −11.9
rank 3835
(YLL047W)
DR = 1076
Yor1
miss 0
orf −2.5
int −2.7
rank 2760
yor1−dF
miss 0
orf −8.8
int −11.9
rank 3836
POA1(YBR022W)
DR = 688
Yor1
miss 0
orf −6.9
int −4.6
rank 3149
yor1−dF
miss 0
orf −2.2
int −11.9
rank 3837
0.35
0.25
0.20
0.15
0.10
0.05
0.00
−60
−20
0
20
40
−50
0
50

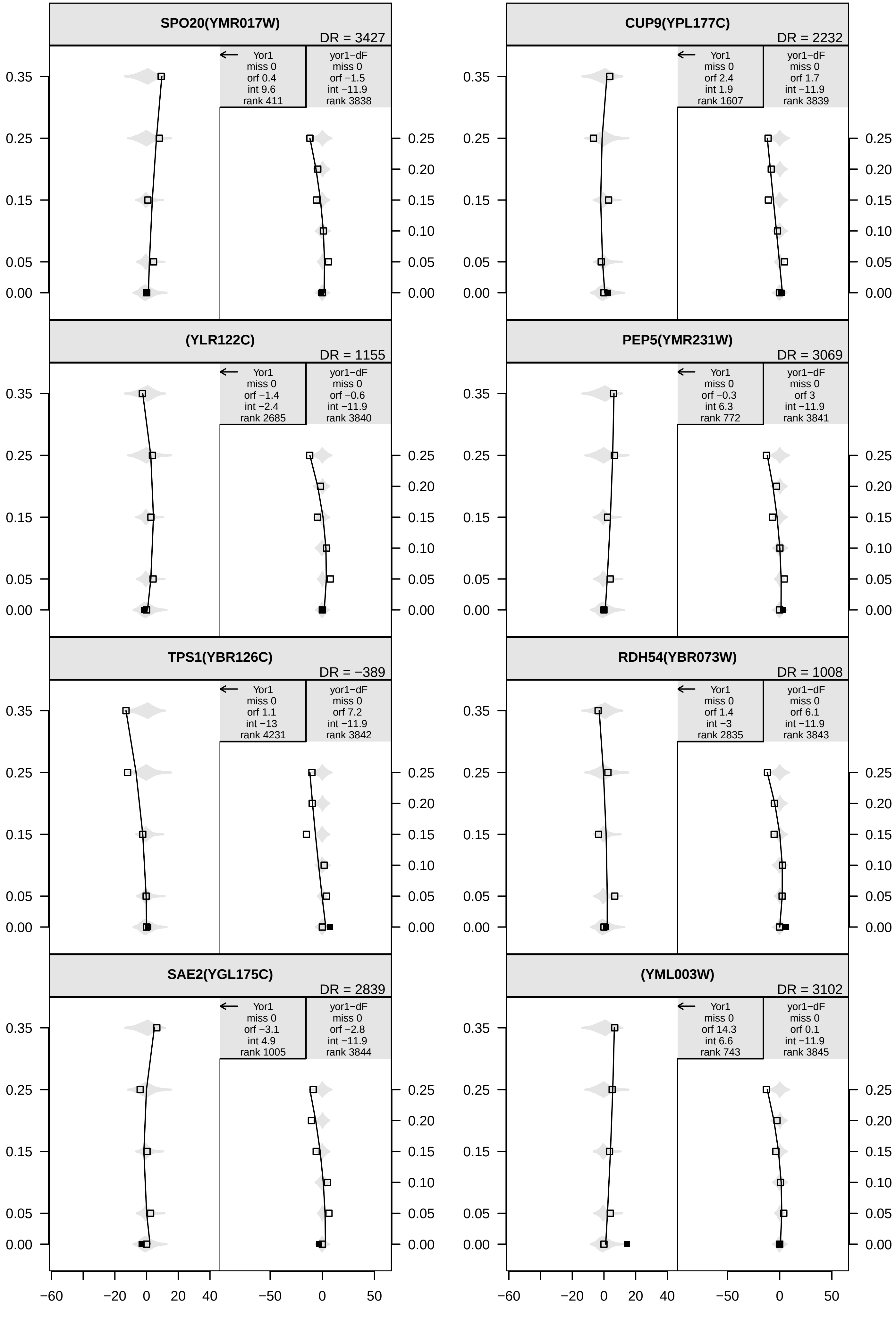
SPO20(YMR017W)
DR = 3427
Yor1
miss 0
orf 0.4
int 9.6
rank 411
yor1−dF
miss 0
orf −1.5
int −11.9
rank 3838
CUP9(YPL177C)
DR = 2232
Yor1
miss 0
orf 2.4
int 1.9
rank 1607
yor1−dF
miss 0
orf 1.7
int −11.9
rank 3839
(YLR122C)
DR = 1155
Yor1
miss 0
orf −1.4
int −2.4
rank 2685
yor1−dF
miss 0
orf −0.6
int −11.9
rank 3840
PEP5(YMR231W)
DR = 3069
Yor1
miss 0
orf −0.3
int 6.3
rank 772
yor1−dF
miss 0
orf 3
int −11.9
rank 3841
TPS1(YBR126C)
DR = −389
Yor1
miss 0
orf 1.1
int −13
rank 4231
yor1−dF
miss 0
orf 7.2
int −11.9
rank 3842
RDH54(YBR073W)
DR = 1008
Yor1
miss 0
orf 1.4
int −3
rank 2835
yor1−dF
miss 0
orf 6.1
int −11.9
rank 3843
SAE2(YGL175C)
DR = 2839
Yor1
miss 0
orf −3.1
int 4.9
rank 1005
yor1−dF
miss 0
orf −2.8
int −11.9
rank 3844
(YML003W)
DR = 3102
Yor1
miss 0
orf 14.3
int 6.6
rank 743
yor1−dF
miss 0
orf 0.1
int −11.9
rank 3845
0.35
0.25
0.20
0.15
0.10
0.05
0.00
−60
−20
0
20
40
−50
0
50

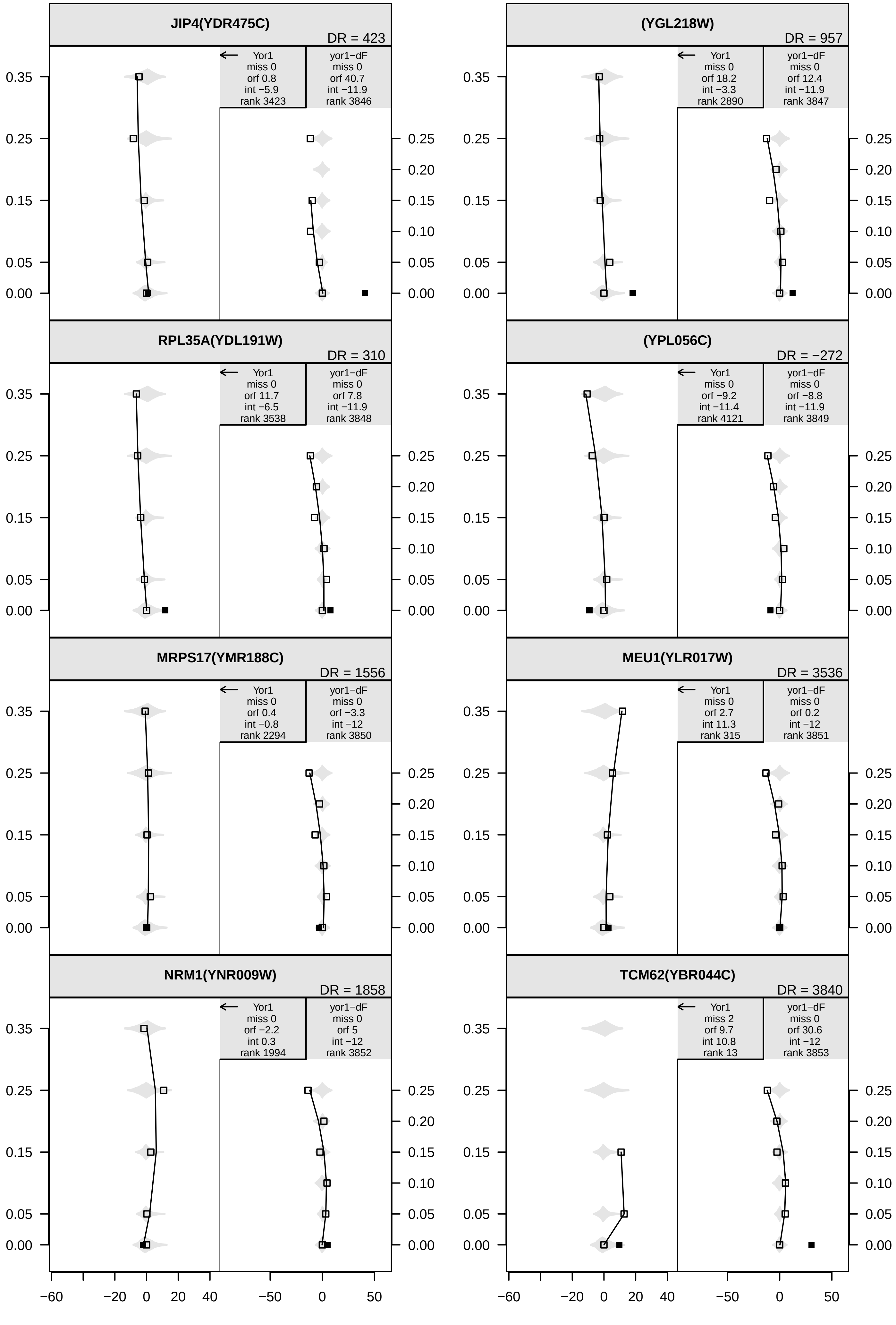
JIP4(YDR475C)
DR = 423
Yor1
miss 0
orf 0.8
int −5.9
rank 3423
yor1−dF
miss 0
orf 40.7
int −11.9
rank 3846
(YGL218W)
DR = 957
Yor1
miss 0
orf 18.2
int −3.3
rank 2890
yor1−dF
miss 0
orf 12.4
int −11.9
rank 3847
RPL35A(YDL191W)
DR = 310
Yor1
miss 0
orf 11.7
int −6.5
rank 3538
yor1−dF
miss 0
orf 7.8
int −11.9
rank 3848
(YPL056C)
DR = −272
Yor1
miss 0
orf −9.2
int −11.4
rank 4121
yor1−dF
miss 0
orf −8.8
int −11.9
rank 3849
MRPS17(YMR188C)
DR = 1556
Yor1
miss 0
orf 0.4
int −0.8
rank 2294
yor1−dF
miss 0
orf −3.3
int −12
rank 3850
MEU1(YLR017W)
DR = 3536
Yor1
miss 0
orf 2.7
int 11.3
rank 315
yor1−dF
miss 0
orf 0.2
int −12
rank 3851
NRM1(YNR009W)
DR = 1858
Yor1
miss 0
orf −2.2
int 0.3
rank 1994
yor1−dF
miss 0
orf 5
int −12
rank 3852
TCM62(YBR044C)
DR = 3840
Yor1
miss 2
orf 9.7
int 10.8
rank 13
yor1−dF
miss 0
orf 30.6
int −12
rank 3853
0.35
0.25
0.20
0.15
0.10
0.05
0.00
−60
−20
0
20
40
−50
0
50

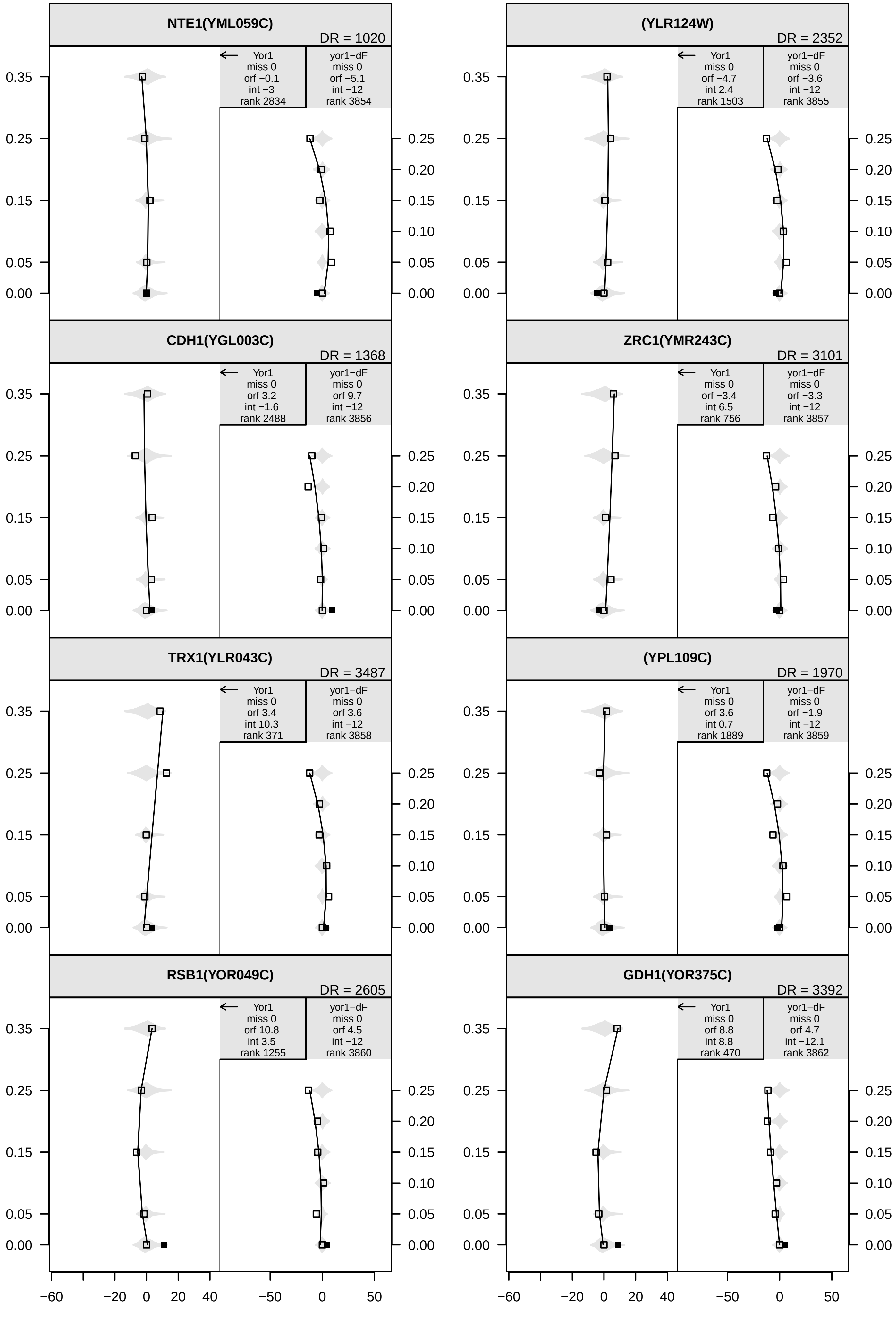
NTE1(YML059C)
DR = 1020
Yor1
miss 0
orf −0.1
int −3
rank 2834
yor1−dF
miss 0
orf −5.1
int −12
rank 3854
(YLR124W)
DR = 2352
Yor1
miss 0
orf −4.7
int 2.4
rank 1503
yor1−dF
miss 0
orf −3.6
int −12
rank 3855
CDH1(YGL003C)
DR = 1368
Yor1
miss 0
orf 3.2
int −1.6
rank 2488
yor1−dF
miss 0
orf 9.7
int −12
rank 3856
ZRC1(YMR243C)
DR = 3101
Yor1
miss 0
orf −3.4
int 6.5
rank 756
yor1−dF
miss 0
orf −3.3
int −12
rank 3857
TRX1(YLR043C)
DR = 3487
Yor1
miss 0
orf 3.4
int 10.3
rank 371
yor1−dF
miss 0
orf 3.6
int −12
rank 3858
(YPL109C)
DR = 1970
Yor1
miss 0
orf 3.6
int 0.7
rank 1889
yor1−dF
miss 0
orf −1.9
int −12
rank 3859
RSB1(YOR049C)
DR = 2605
Yor1
miss 0
orf 10.8
int 3.5
rank 1255
yor1−dF
miss 0
orf 4.5
int −12
rank 3860
GDH1(YOR375C)
DR = 3392
Yor1
miss 0
orf 8.8
int 8.8
rank 470
yor1−dF
miss 0
orf 4.7
int −12.1
rank 3862
0.35
0.25
0.20
0.15
0.10
0.05
0.00
−60
−20
0
20
40
−50
0
50

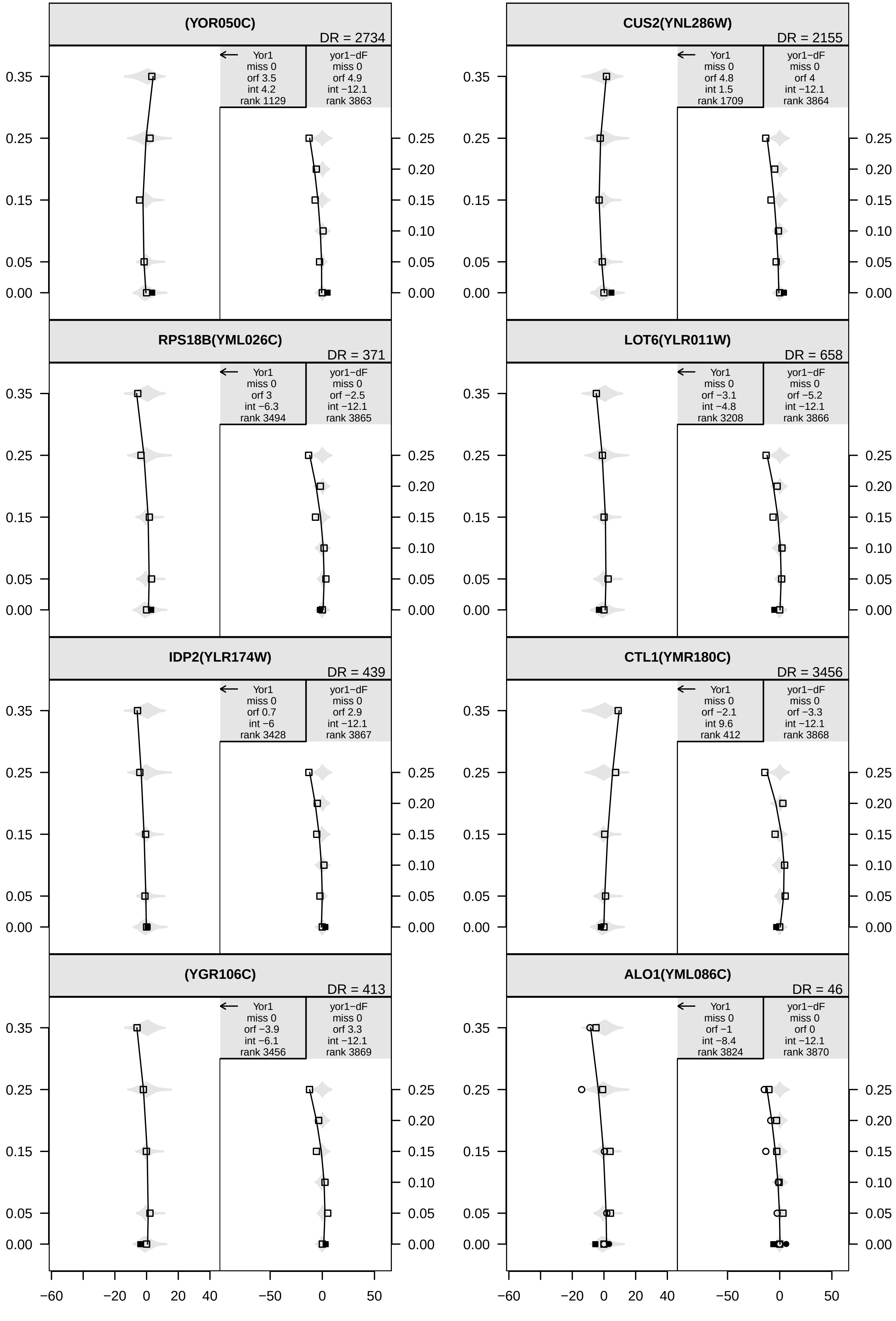
(YOR050C)
DR = 2734
Yor1
miss 0
orf 3.5
int 4.2
rank 1129
yor1−dF
miss 0
orf 4.9
int −12.1
rank 3863
CUS2(YNL286W)
DR = 2155
Yor1
miss 0
orf 4.8
int 1.5
rank 1709
yor1−dF
miss 0
orf 4
int −12.1
rank 3864
RPS18B(YML026C)
DR = 371
Yor1
miss 0
orf 3
int −6.3
rank 3494
yor1−dF
miss 0
orf −2.5
int −12.1
rank 3865
LOT6(YLR011W)
DR = 658
Yor1
miss 0
orf −3.1
int −4.8
rank 3208
yor1−dF
miss 0
orf −5.2
int −12.1
rank 3866
IDP2(YLR174W)
DR = 439
Yor1
miss 0
orf 0.7
int −6
rank 3428
yor1−dF
miss 0
orf 2.9
int −12.1
rank 3867
CTL1(YMR180C)
DR = 3456
Yor1
miss 0
orf −2.1
int 9.6
rank 412
yor1−dF
miss 0
orf −3.3
int −12.1
rank 3868
(YGR106C)
DR = 413
Yor1
miss 0
orf −3.9
int −6.1
rank 3456
yor1−dF
miss 0
orf 3.3
int −12.1
rank 3869
ALO1(YML086C)
DR = 46
Yor1
miss 0
orf −1
int −8.4
rank 3824
yor1−dF
miss 0
orf 0
int −12.1
rank 3870
0.35
0.25
0.20
0.15
0.10
0.05
0.00
−60
−20
0
20
40
−50
0
50

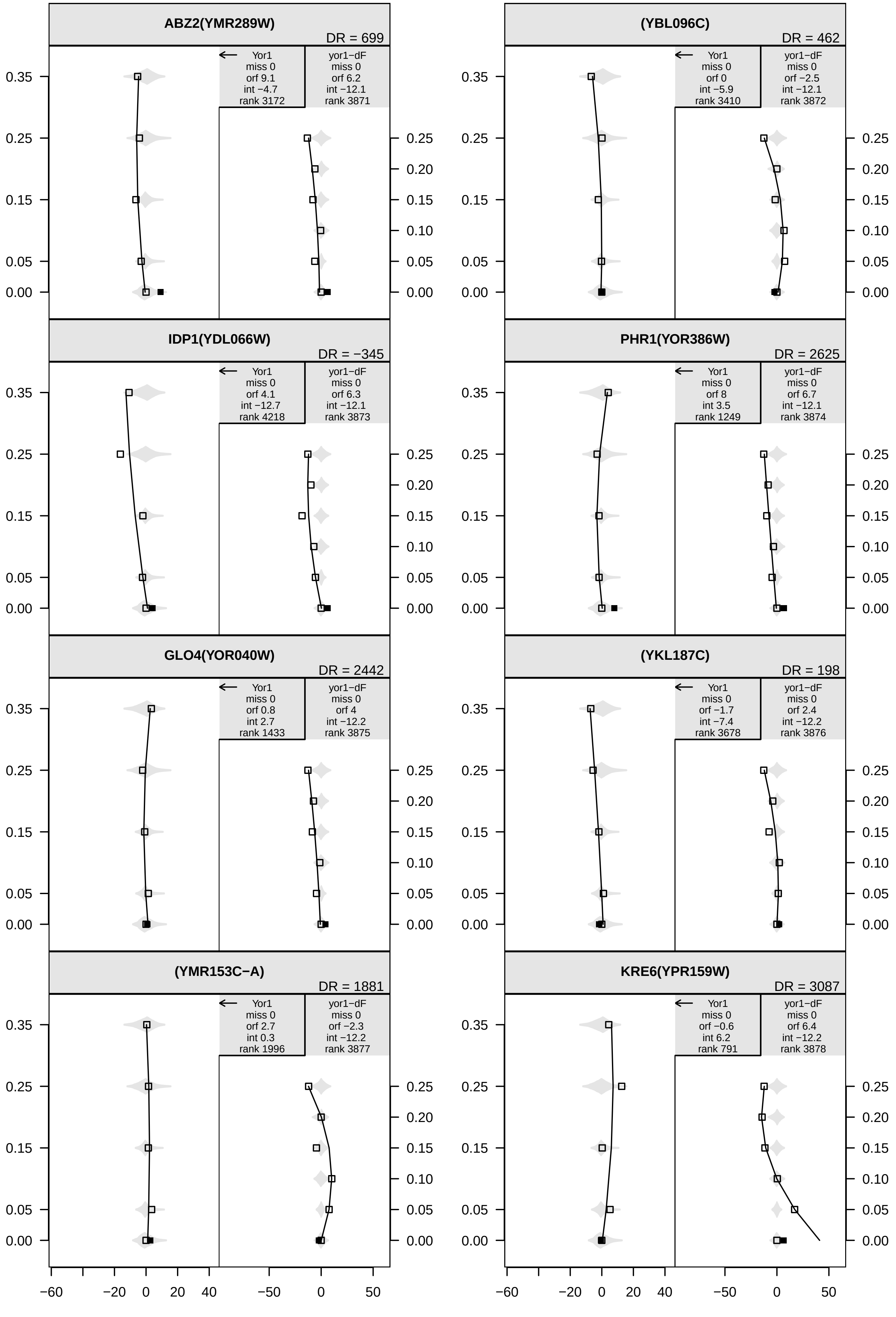
ABZ2(YMR289W)
DR = 699
Yor1
miss 0
orf 9.1
int −4.7
rank 3172
yor1−dF
miss 0
orf 6.2
int −12.1
rank 3871
(YBL096C)
DR = 462
Yor1
miss 0
orf 0
int −5.9
rank 3410
yor1−dF
miss 0
orf −2.5
int −12.1
rank 3872
IDP1(YDL066W)
DR = −345
Yor1
miss 0
orf 4.1
int −12.7
rank 4218
yor1−dF
miss 0
orf 6.3
int −12.1
rank 3873
PHR1(YOR386W)
DR = 2625
Yor1
miss 0
orf 8
int 3.5
rank 1249
yor1−dF
miss 0
orf 6.7
int −12.1
rank 3874
GLO4(YOR040W)
DR = 2442
Yor1
miss 0
orf 0.8
int 2.7
rank 1433
yor1−dF
miss 0
orf 4
int −12.2
rank 3875
(YKL187C)
DR = 198
Yor1
miss 0
orf −1.7
int −7.4
rank 3678
yor1−dF
miss 0
orf 2.4
int −12.2
rank 3876
(YMR153C−A)
DR = 1881
Yor1
miss 0
orf 2.7
int 0.3
rank 1996
yor1−dF
miss 0
orf −2.3
int −12.2
rank 3877
KRE6(YPR159W)
DR = 3087
Yor1
miss 0
orf −0.6
int 6.2
rank 791
yor1−dF
miss 0
orf 6.4
int −12.2
rank 3878
0.35
0.25
0.20
0.15
0.10
0.05
0.00
−60
−20
0
20
40
−50
0
50

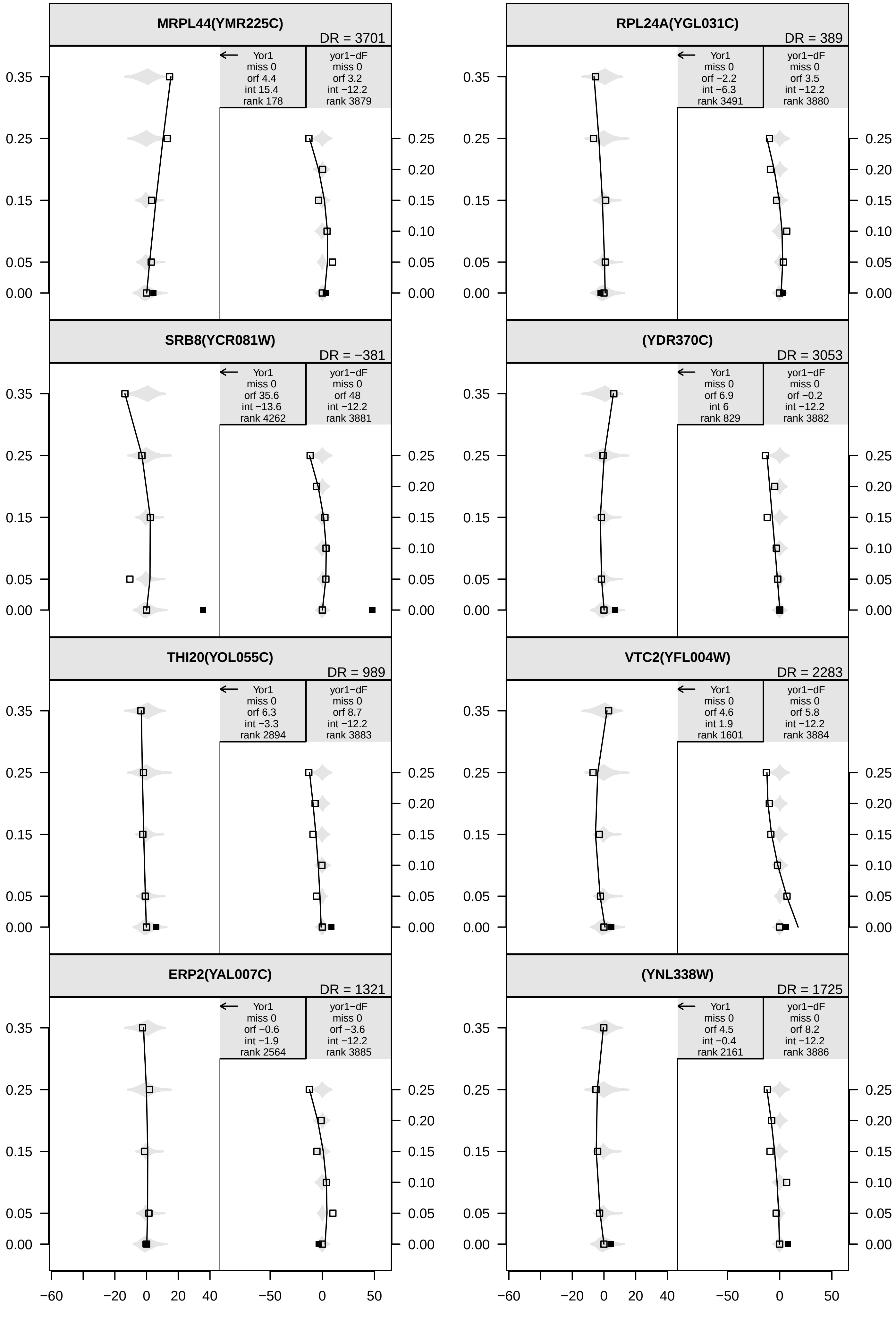

MRPL44(YMR225C)
DR = 3701
Yor1
miss 0
orf 4.4
int 15.4
rank 178
yor1−dF
miss 0
orf 3.2
int −12.2
rank 3879
RPL24A(YGL031C)
DR = 389
Yor1
miss 0
orf −2.2
int −6.3
rank 3491
yor1−dF
miss 0
orf 3.5
int −12.2
rank 3880
SRB8(YCR081W)
DR = −381
Yor1
miss 0
orf 35.6
int −13.6
rank 4262
yor1−dF
miss 0
orf 48
int −12.2
rank 3881
(YDR370C)
DR = 3053
Yor1
miss 0
orf 6.9
int 6
rank 829
yor1−dF
miss 0
orf −0.2
int −12.2
rank 3882
THI20(YOL055C)
DR = 989
Yor1
miss 0
orf 6.3
int −3.3
rank 2894
yor1−dF
miss 0
orf 8.7
int −12.2
rank 3883
VTC2(YFL004W)
DR = 2283
Yor1
miss 0
orf 4.6
int 1.9
rank 1601
yor1−dF
miss 0
orf 5.8
int −12.2
rank 3884
ERP2(YAL007C)
DR = 1321
Yor1
miss 0
orf −0.6
int −1.9
rank 2564
yor1−dF
miss 0
orf −3.6
int −12.2
rank 3885
(YNL338W)
DR = 1725
Yor1
miss 0
orf 4.5
int −0.4
rank 2161
yor1−dF
miss 0
orf 8.2
int −12.2
rank 3886
0.35
0.25
0.20
0.15
0.10
0.05
0.00
−60
−20
0
20
40
−50
0
50

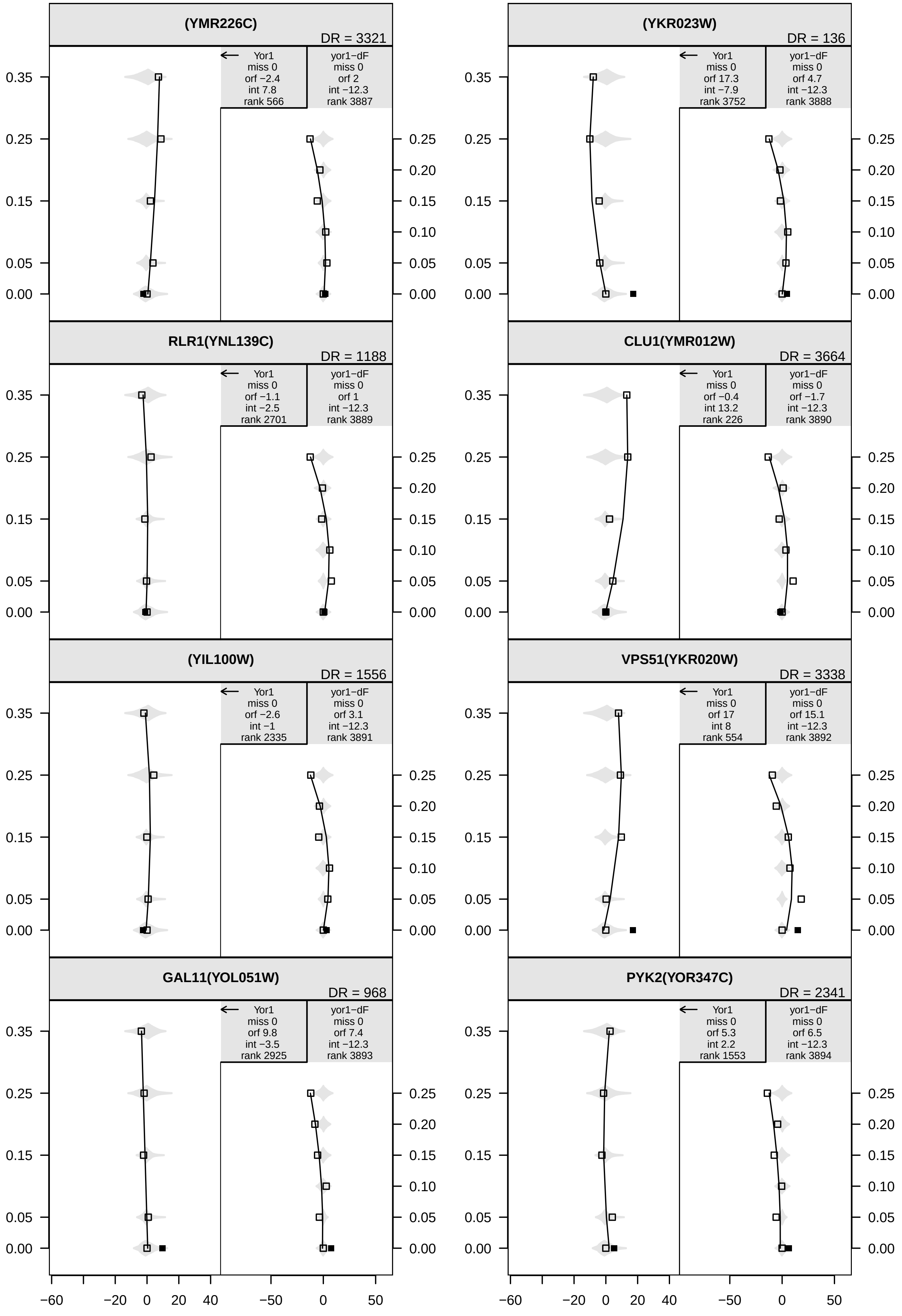

(YMR226C)
DR = 3321
Yor1 miss 0 orf −2.4 int 7.8 rank 566
yor1−dF miss 0 orf 2 int −12.3 rank 3887
(YKR023W)
DR = 136
Yor1 miss 0 orf 17.3 int −7.9 rank 3752
yor1−dF miss 0 orf 4.7 int −12.3 rank 3888
RLR1(YNL139C)
DR = 1188
Yor1 miss 0 orf −1.1 int −2.5 rank 2701
yor1−dF miss 0 orf 1 int −12.3 rank 3889
CLU1(YMR012W)
DR = 3664
Yor1 miss 0 orf −0.4 int 13.2 rank 226
yor1−dF miss 0 orf −1.7 int −12.3 rank 3890
(YIL100W)
DR = 1556
Yor1 miss 0 orf −2.6 int −1 rank 2335
yor1−dF miss 0 orf 3.1 int −12.3 rank 3891
VPS51(YKR020W)
DR = 3338
Yor1 miss 0 orf 17 int 8 rank 554
yor1−dF miss 0 orf 15.1 int −12.3 rank 3892
GAL11(YOL051W)
DR = 968
Yor1 miss 0 orf 9.8 int −3.5 rank 2925
yor1−dF miss 0 orf 7.4 int −12.3 rank 3893
PYK2(YOR347C)
DR = 2341
Yor1 miss 0 orf 5.3 int 2.2 rank 1553
yor1−dF miss 0 orf 6.5 int −12.3 rank 3894
0.00 0.05 0.10 0.15 0.20 0.25 0.35
−60 −20 0 20 40 −50 0 50

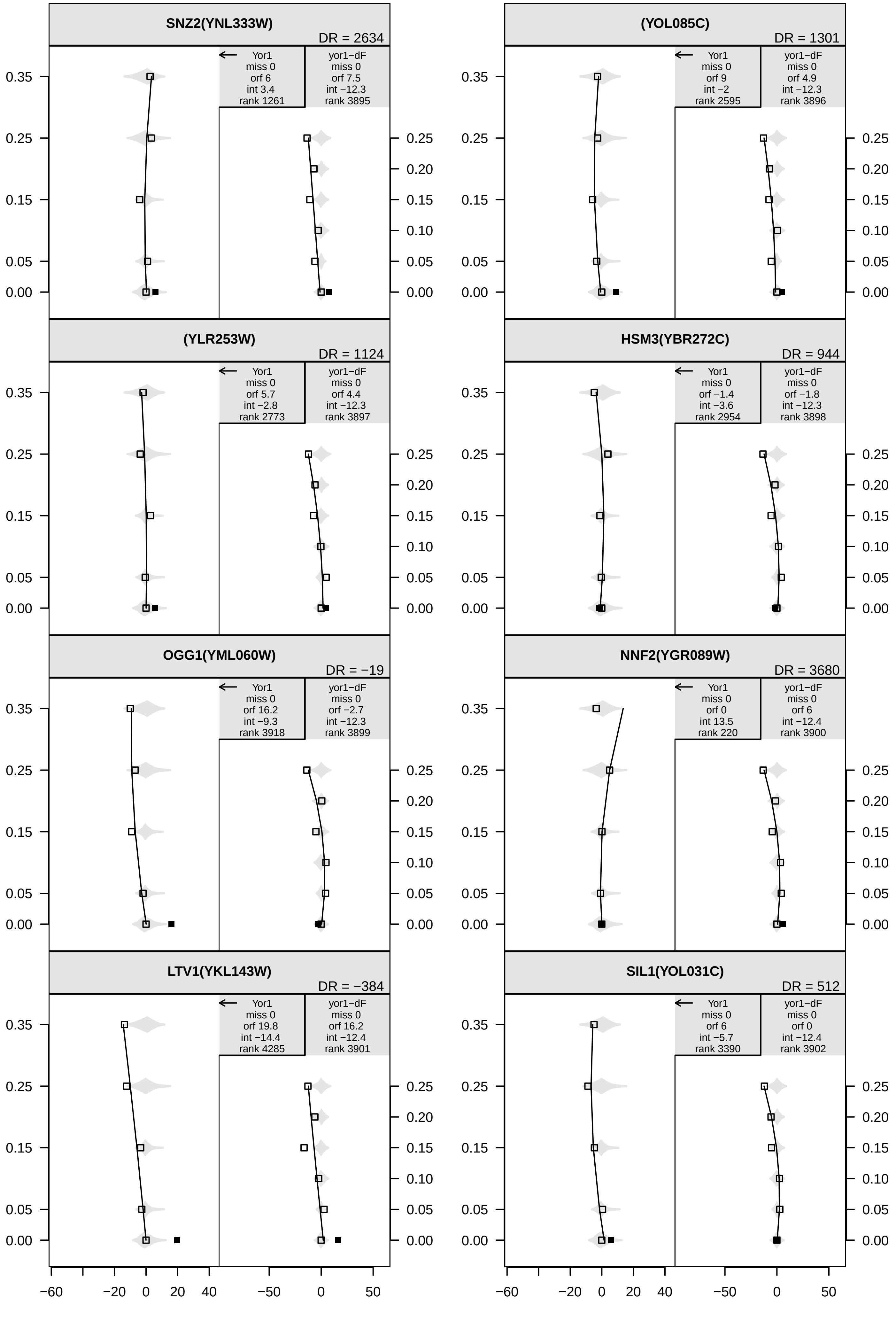
SNZ2(YNL333W)
DR = 2634
Yor1 miss 0 orf 6 int 3.4 rank 1261
yor1−dF miss 0 orf 7.5 int −12.3 rank 3895
(YOL085C)
DR = 1301
Yor1 miss 0 orf 9 int −2 rank 2595
yor1−dF miss 0 orf 4.9 int −12.3 rank 3896
(YLR253W)
DR = 1124
Yor1 miss 0 orf 5.7 int −2.8 rank 2773
yor1−dF miss 0 orf 4.4 int −12.3 rank 3897
HSM3(YBR272C)
DR = 944
Yor1 miss 0 orf −1.4 int −3.6 rank 2954
yor1−dF miss 0 orf −1.8 int −12.3 rank 3898
OGG1(YML060W)
DR = −19
Yor1 miss 0 orf 16.2 int −9.3 rank 3918
yor1−dF miss 0 orf −2.7 int −12.3 rank 3899
NNF2(YGR089W)
DR = 3680
Yor1 miss 0 orf 0 int 13.5 rank 220
yor1−dF miss 0 orf 6 int −12.4 rank 3900
LTV1(YKL143W)
DR = −384
Yor1 miss 0 orf 19.8 int −14.4 rank 4285
yor1−dF miss 0 orf 16.2 int −12.4 rank 3901
SIL1(YOL031C)
DR = 512
Yor1 miss 0 orf 6 int −5.7 rank 3390
yor1−dF miss 0 orf 0 int −12.4 rank 3902
0.35 0.25 0.15 0.05 0.00
0.25 0.20 0.15 0.10 0.05 0.00
−60 −20 0 20 40
−50 0 50

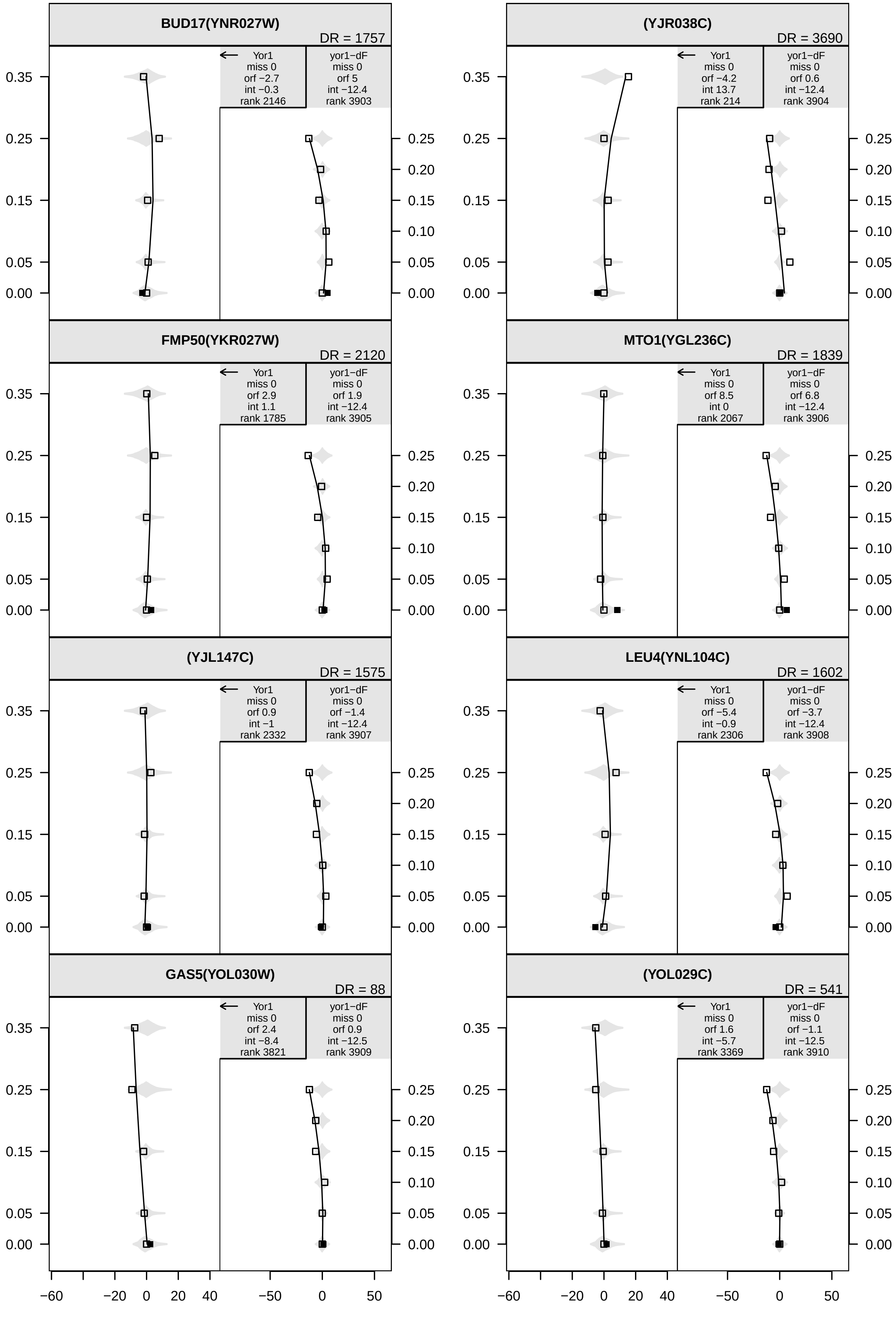
BUD17(YNR027W)
DR = 1757
Yor1 miss 0 orf −2.7 int −0.3 rank 2146
yor1−dF miss 0 orf 5 int −12.4 rank 3903
(YJR038C)
DR = 3690
Yor1 miss 0 orf −4.2 int 13.7 rank 214
yor1−dF miss 0 orf 0.6 int −12.4 rank 3904
FMP50(YKR027W)
DR = 2120
Yor1 miss 0 orf 2.9 int 1.1 rank 1785
yor1−dF miss 0 orf 1.9 int −12.4 rank 3905
MTO1(YGL236C)
DR = 1839
Yor1 miss 0 orf 8.5 int 0 rank 2067
yor1−dF miss 0 orf 6.8 int −12.4 rank 3906
(YJL147C)
DR = 1575
Yor1 miss 0 orf 0.9 int −1 rank 2332
yor1−dF miss 0 orf −1.4 int −12.4 rank 3907
LEU4(YNL104C)
DR = 1602
Yor1 miss 0 orf −5.4 int −0.9 rank 2306
yor1−dF miss 0 orf −3.7 int −12.4 rank 3908
GAS5(YOL030W)
DR = 88
Yor1 miss 0 orf 2.4 int −8.4 rank 3821
yor1−dF miss 0 orf 0.9 int −12.5 rank 3909
(YOL029C)
DR = 541
Yor1 miss 0 orf 1.6 int −5.7 rank 3369
yor1−dF miss 0 orf −1.1 int −12.5 rank 3910
0.35 0.25 0.15 0.05 0.00
0.25 0.20 0.15 0.10 0.05 0.00
−60 −20 0 20 40 −50 0 50

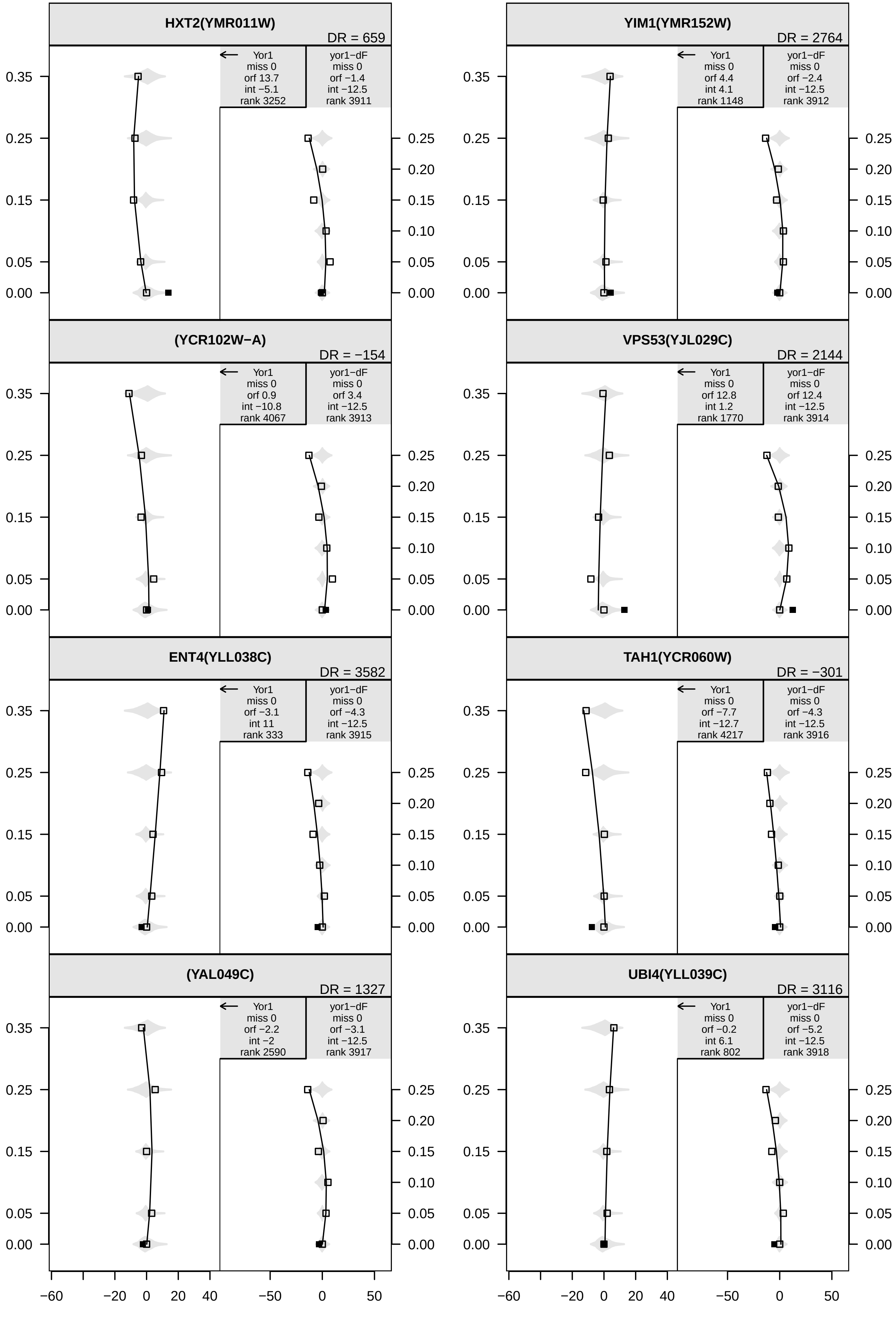
HXT2(YMR011W)
DR = 659
Yor1
miss 0
orf 13.7
int −5.1
rank 3252
yor1−dF
miss 0
orf −1.4
int −12.5
rank 3911
YIM1(YMR152W)
DR = 2764
Yor1
miss 0
orf 4.4
int 4.1
rank 1148
yor1−dF
miss 0
orf −2.4
int −12.5
rank 3912
(YCR102W−A)
DR = −154
Yor1
miss 0
orf 0.9
int −10.8
rank 4067
yor1−dF
miss 0
orf 3.4
int −12.5
rank 3913
VPS53(YJL029C)
DR = 2144
Yor1
miss 0
orf 12.8
int 1.2
rank 1770
yor1−dF
miss 0
orf 12.4
int −12.5
rank 3914
ENT4(YLL038C)
DR = 3582
Yor1
miss 0
orf −3.1
int 11
rank 333
yor1−dF
miss 0
orf −4.3
int −12.5
rank 3915
TAH1(YCR060W)
DR = −301
Yor1
miss 0
orf −7.7
int −12.7
rank 4217
yor1−dF
miss 0
orf −4.3
int −12.5
rank 3916
(YAL049C)
DR = 1327
Yor1
miss 0
orf −2.2
int −2
rank 2590
yor1−dF
miss 0
orf −3.1
int −12.5
rank 3917
UBI4(YLL039C)
DR = 3116
Yor1
miss 0
orf −0.2
int 6.1
rank 802
yor1−dF
miss 0
orf −5.2
int −12.5
rank 3918
0.35
0.25
0.20
0.15
0.10
0.05
0.00
−60
−20
0
20
40
−50
0
50

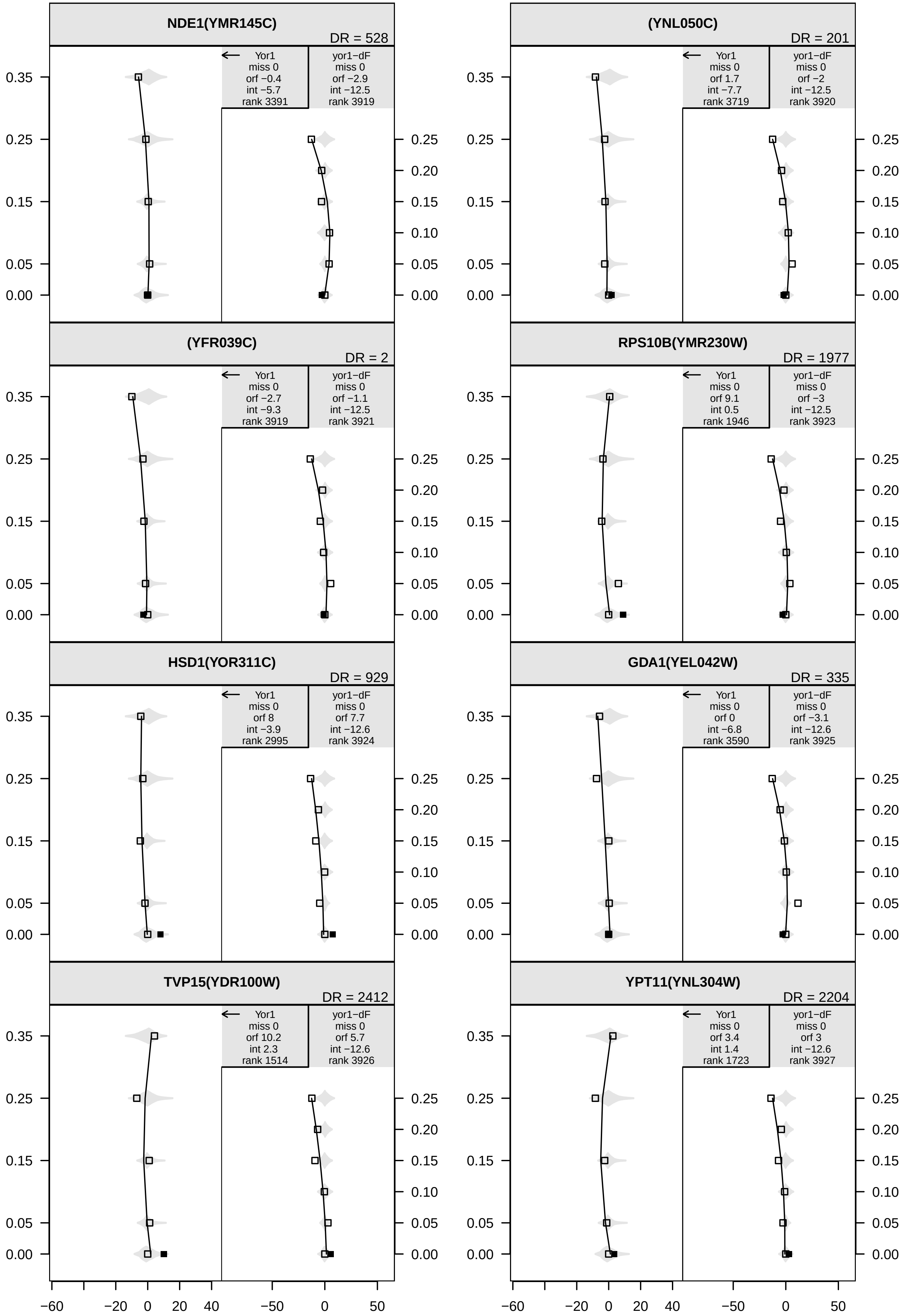
NDE1(YMR145C)
DR = 528
Yor1 miss 0 orf −0.4 int −5.7 rank 3391
yor1−dF miss 0 orf −2.9 int −12.5 rank 3919
(YNL050C)
DR = 201
Yor1 miss 0 orf 1.7 int −7.7 rank 3719
yor1−dF miss 0 orf −2 int −12.5 rank 3920
(YFR039C)
DR = 2
Yor1 miss 0 orf −2.7 int −9.3 rank 3919
yor1−dF miss 0 orf −1.1 int −12.5 rank 3921
RPS10B(YMR230W)
DR = 1977
Yor1 miss 0 orf 9.1 int 0.5 rank 1946
yor1−dF miss 0 orf −3 int −12.5 rank 3923
HSD1(YOR311C)
DR = 929
Yor1 miss 0 orf 8 int −3.9 rank 2995
yor1−dF miss 0 orf 7.7 int −12.6 rank 3924
GDA1(YEL042W)
DR = 335
Yor1 miss 0 orf 0 int −6.8 rank 3590
yor1−dF miss 0 orf −3.1 int −12.6 rank 3925
TVP15(YDR100W)
DR = 2412
Yor1 miss 0 orf 10.2 int 2.3 rank 1514
yor1−dF miss 0 orf 5.7 int −12.6 rank 3926
YPT11(YNL304W)
DR = 2204
Yor1 miss 0 orf 3.4 int 1.4 rank 1723
yor1−dF miss 0 orf 3 int −12.6 rank 3927
0.35 0.25 0.15 0.05 0.00
0.25 0.20 0.15 0.10 0.05 0.00
−60 −20 0 20 40 −50 0 50

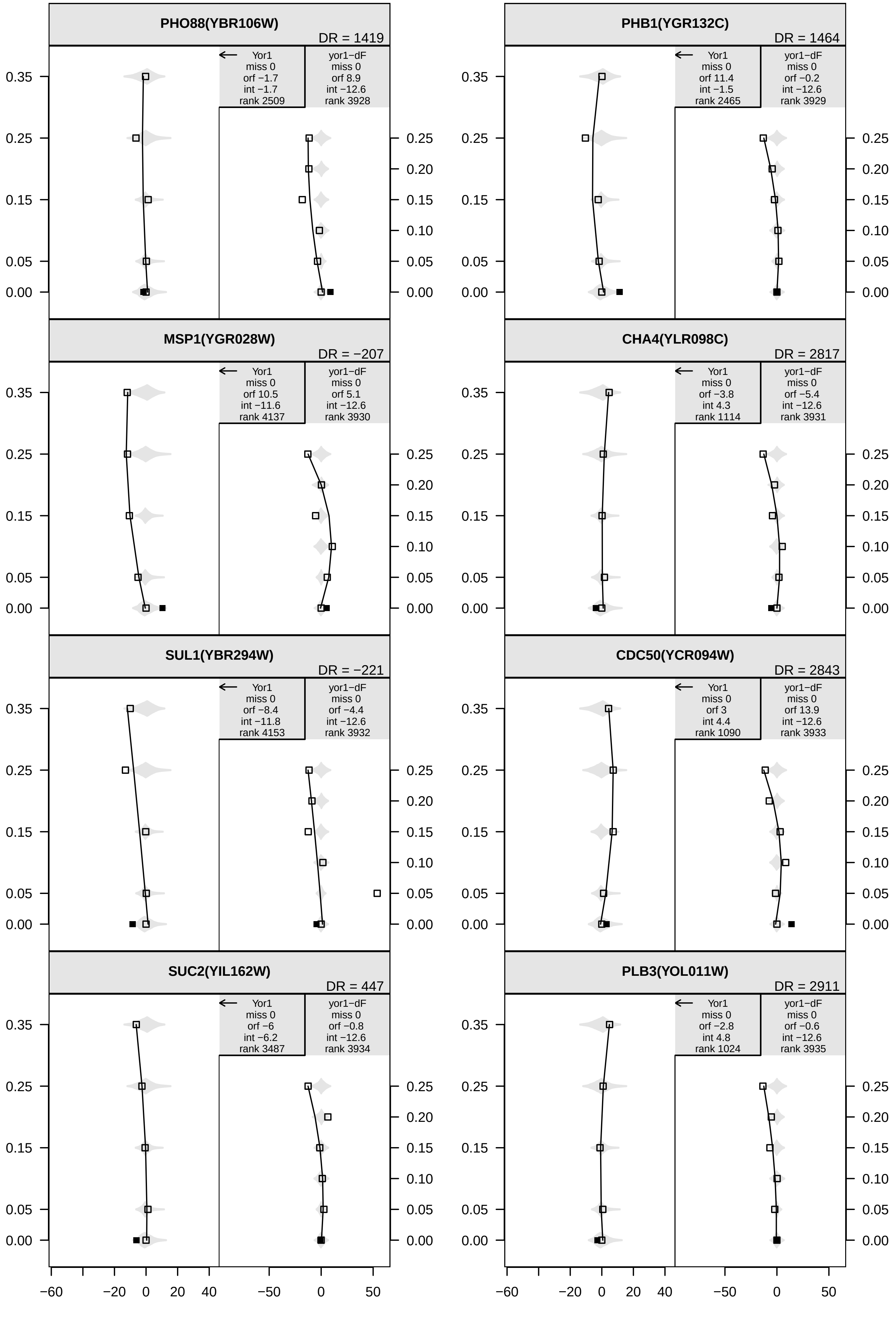

PHO88(YBR106W)
DR = 1419
Yor1 miss 0 orf −1.7 int −1.7 rank 2509
yor1−dF miss 0 orf 8.9 int −12.6 rank 3928
PHB1(YGR132C)
DR = 1464
Yor1 miss 0 orf 11.4 int −1.5 rank 2465
yor1−dF miss 0 orf −0.2 int −12.6 rank 3929
MSP1(YGR028W)
DR = −207
Yor1 miss 0 orf 10.5 int −11.6 rank 4137
yor1−dF miss 0 orf 5.1 int −12.6 rank 3930
CHA4(YLR098C)
DR = 2817
Yor1 miss 0 orf −3.8 int 4.3 rank 1114
yor1−dF miss 0 orf −5.4 int −12.6 rank 3931
SUL1(YBR294W)
DR = −221
Yor1 miss 0 orf −8.4 int −11.8 rank 4153
yor1−dF miss 0 orf −4.4 int −12.6 rank 3932
CDC50(YCR094W)
DR = 2843
Yor1 miss 0 orf 3 int 4.4 rank 1090
yor1−dF miss 0 orf 13.9 int −12.6 rank 3933
SUC2(YIL162W)
DR = 447
Yor1 miss 0 orf −6 int −6.2 rank 3487
yor1−dF miss 0 orf −0.8 int −12.6 rank 3934
PLB3(YOL011W)
DR = 2911
Yor1 miss 0 orf −2.8 int 4.8 rank 1024
yor1−dF miss 0 orf −0.6 int −12.6 rank 3935

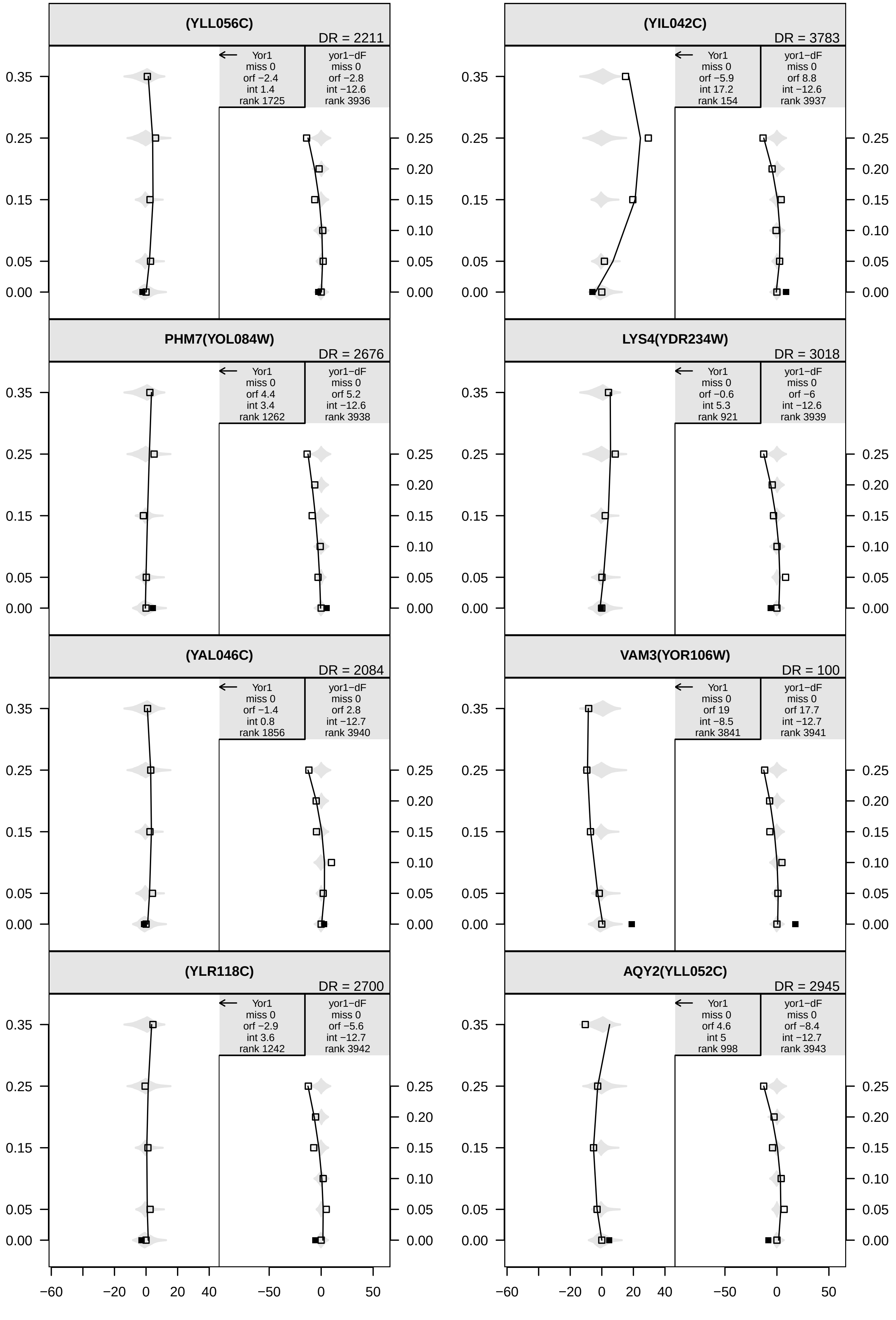

(YLL056C)
DR = 2211
Yor1
miss 0
orf −2.4
int 1.4
rank 1725
yor1−dF
miss 0
orf −2.8
int −12.6
rank 3936
(YIL042C)
DR = 3783
Yor1
miss 0
orf −5.9
int 17.2
rank 154
yor1−dF
miss 0
orf 8.8
int −12.6
rank 3937
PHM7(YOL084W)
DR = 2676
Yor1
miss 0
orf 4.4
int 3.4
rank 1262
yor1−dF
miss 0
orf 5.2
int −12.6
rank 3938
LYS4(YDR234W)
DR = 3018
Yor1
miss 0
orf −0.6
int 5.3
rank 921
yor1−dF
miss 0
orf −6
int −12.6
rank 3939
(YAL046C)
DR = 2084
Yor1
miss 0
orf −1.4
int 0.8
rank 1856
yor1−dF
miss 0
orf 2.8
int −12.7
rank 3940
VAM3(YOR106W)
DR = 100
Yor1
miss 0
orf 19
int −8.5
rank 3841
yor1−dF
miss 0
orf 17.7
int −12.7
rank 3941
(YLR118C)
DR = 2700
Yor1
miss 0
orf −2.9
int 3.6
rank 1242
yor1−dF
miss 0
orf −5.6
int −12.7
rank 3942
AQY2(YLL052C)
DR = 2945
Yor1
miss 0
orf 4.6
int 5
rank 998
yor1−dF
miss 0
orf −8.4
int −12.7
rank 3943
0.35
0.25
0.20
0.15
0.10
0.05
0.00
−60
−20
0
20
40
−50
0
50

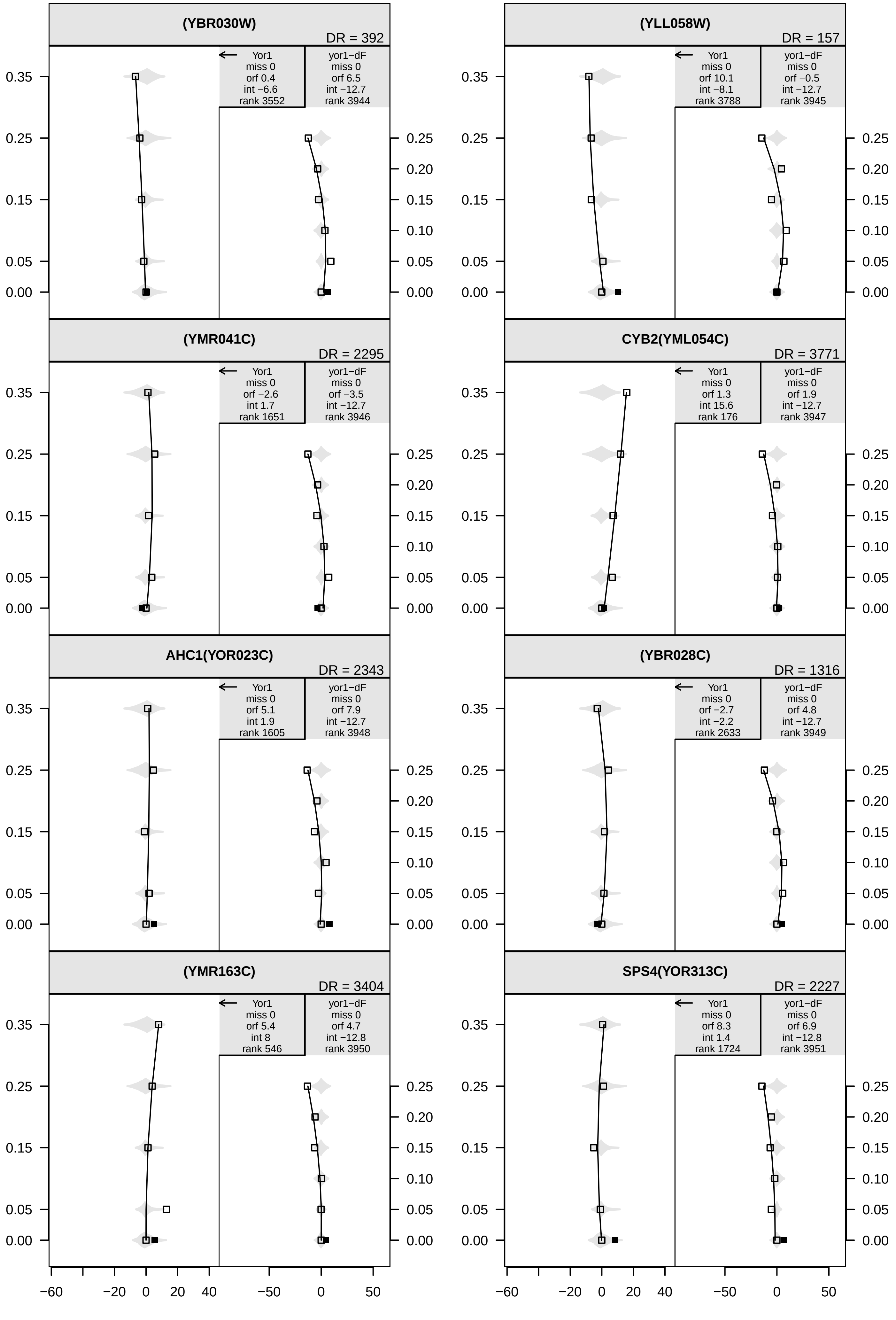

(YBR030W)
DR = 392
Yor1 miss 0 orf 0.4 int −6.6 rank 3552
yor1−dF miss 0 orf 6.5 int −12.7 rank 3944
(YLL058W)
DR = 157
Yor1 miss 0 orf 10.1 int −8.1 rank 3788
yor1−dF miss 0 orf −0.5 int −12.7 rank 3945
(YMR041C)
DR = 2295
Yor1 miss 0 orf −2.6 int 1.7 rank 1651
yor1−dF miss 0 orf −3.5 int −12.7 rank 3946
CYB2(YML054C)
DR = 3771
Yor1 miss 0 orf 1.3 int 15.6 rank 176
yor1−dF miss 0 orf 1.9 int −12.7 rank 3947
AHC1(YOR023C)
DR = 2343
Yor1 miss 0 orf 5.1 int 1.9 rank 1605
yor1−dF miss 0 orf 7.9 int −12.7 rank 3948
(YBR028C)
DR = 1316
Yor1 miss 0 orf −2.7 int −2.2 rank 2633
yor1−dF miss 0 orf 4.8 int −12.7 rank 3949
(YMR163C)
DR = 3404
Yor1 miss 0 orf 5.4 int 8 rank 546
yor1−dF miss 0 orf 4.7 int −12.8 rank 3950
SPS4(YOR313C)
DR = 2227
Yor1 miss 0 orf 8.3 int 1.4 rank 1724
yor1−dF miss 0 orf 6.9 int −12.8 rank 3951
0.35 0.25 0.15 0.05 0.00
0.25 0.20 0.15 0.10 0.05 0.00
−60 −20 0 20 40 −50 0 50

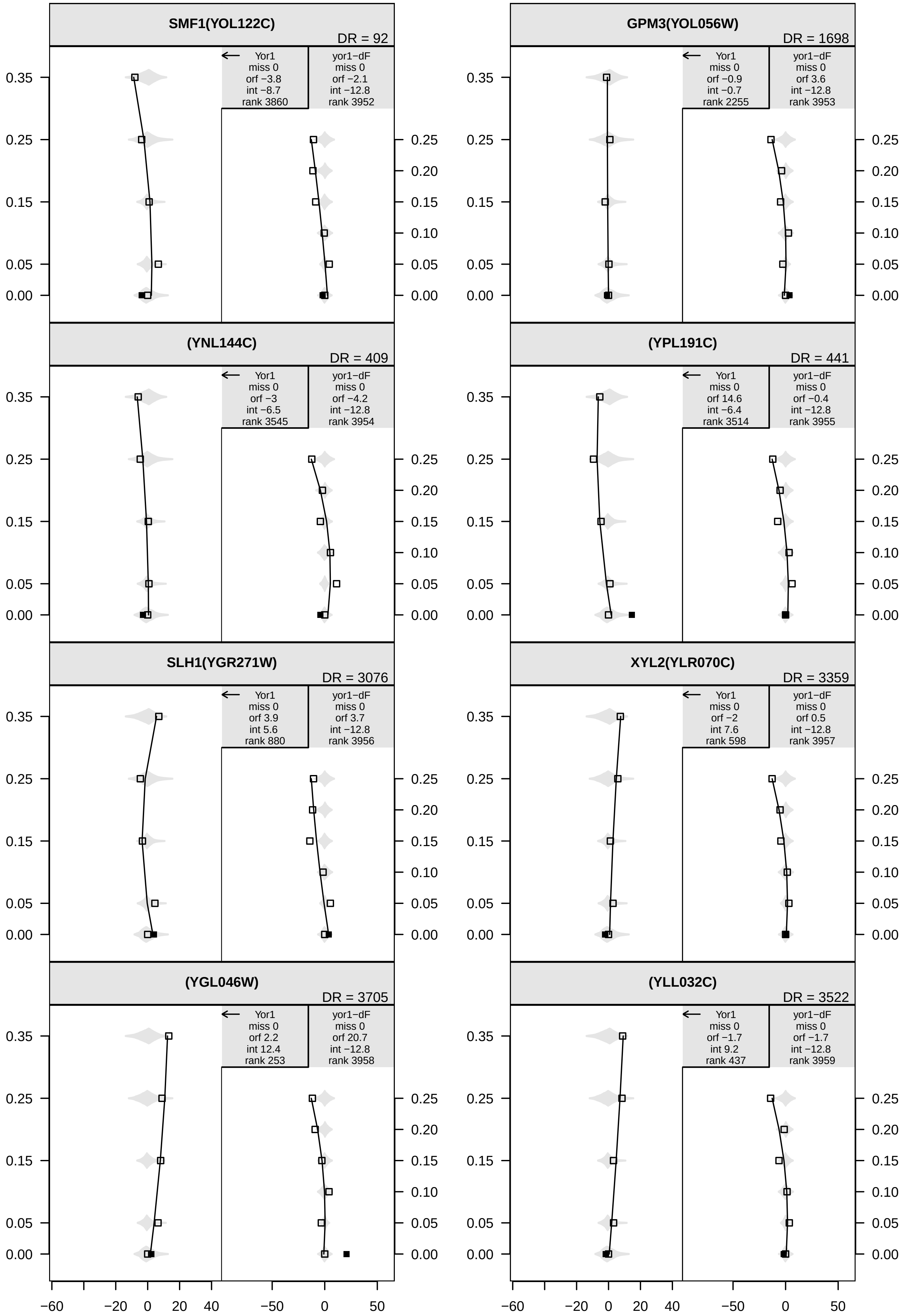
SMF1(YOL122C)
DR = 92
Yor1 miss 0 orf −3.8 int −8.7 rank 3860
yor1−dF miss 0 orf −2.1 int −12.8 rank 3952
GPM3(YOL056W)
DR = 1698
Yor1 miss 0 orf −0.9 int −0.7 rank 2255
yor1−dF miss 0 orf 3.6 int −12.8 rank 3953
(YNL144C)
DR = 409
Yor1 miss 0 orf −3 int −6.5 rank 3545
yor1−dF miss 0 orf −4.2 int −12.8 rank 3954
(YPL191C)
DR = 441
Yor1 miss 0 orf 14.6 int −6.4 rank 3514
yor1−dF miss 0 orf −0.4 int −12.8 rank 3955
SLH1(YGR271W)
DR = 3076
Yor1 miss 0 orf 3.9 int 5.6 rank 880
yor1−dF miss 0 orf 3.7 int −12.8 rank 3956
XYL2(YLR070C)
DR = 3359
Yor1 miss 0 orf −2 int 7.6 rank 598
yor1−dF miss 0 orf 0.5 int −12.8 rank 3957
(YGL046W)
DR = 3705
Yor1 miss 0 orf 2.2 int 12.4 rank 253
yor1−dF miss 0 orf 20.7 int −12.8 rank 3958
(YLL032C)
DR = 3522
Yor1 miss 0 orf −1.7 int 9.2 rank 437
yor1−dF miss 0 orf −1.7 int −12.8 rank 3959

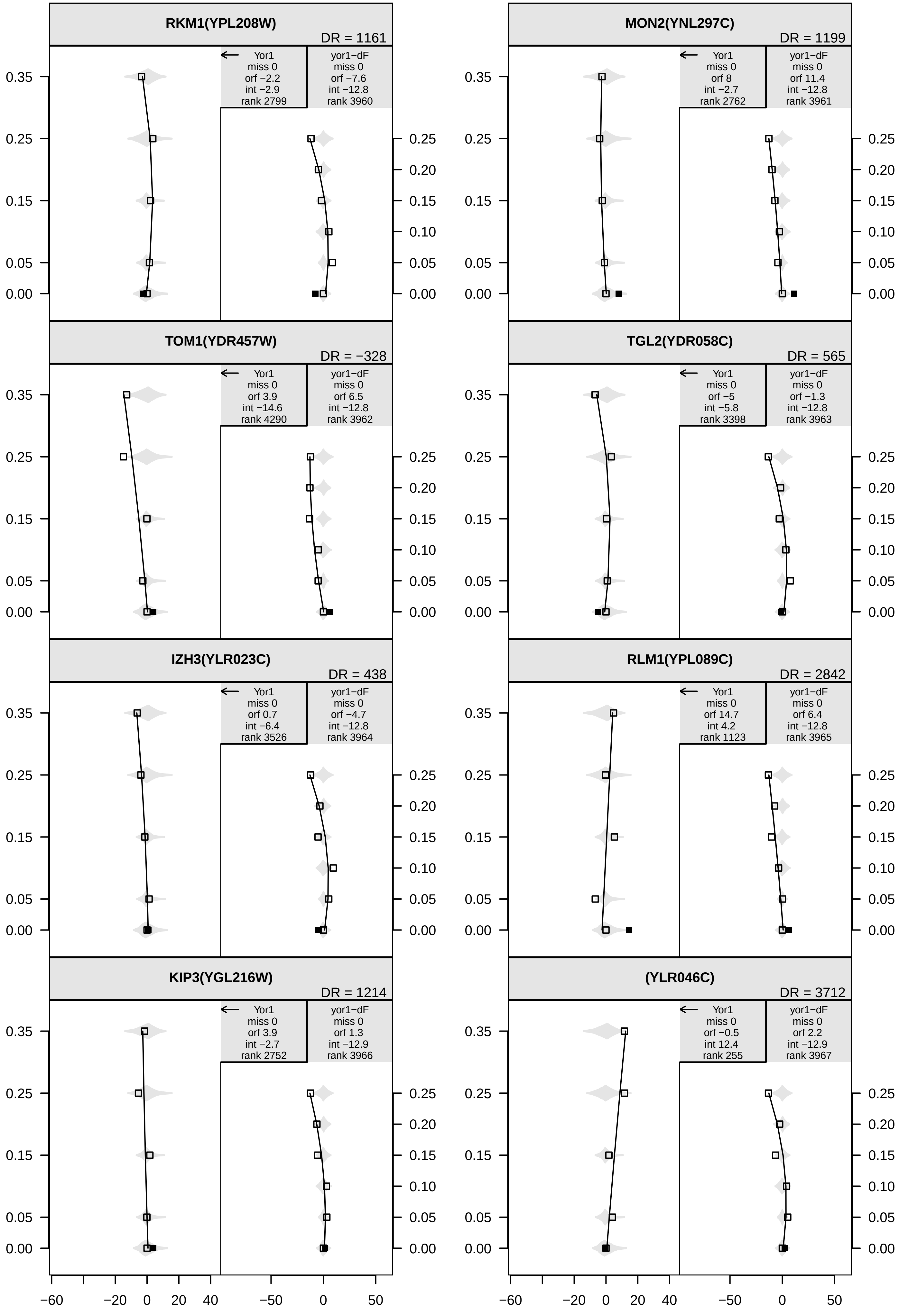
RKM1(YPL208W)
DR = 1161
Yor1
miss 0
orf −2.2
int −2.9
rank 2799
yor1−dF
miss 0
orf −7.6
int −12.8
rank 3960
MON2(YNL297C)
DR = 1199
Yor1
miss 0
orf 8
int −2.7
rank 2762
yor1−dF
miss 0
orf 11.4
int −12.8
rank 3961
TOM1(YDR457W)
DR = −328
Yor1
miss 0
orf 3.9
int −14.6
rank 4290
yor1−dF
miss 0
orf 6.5
int −12.8
rank 3962
TGL2(YDR058C)
DR = 565
Yor1
miss 0
orf −5
int −5.8
rank 3398
yor1−dF
miss 0
orf −1.3
int −12.8
rank 3963
IZH3(YLR023C)
DR = 438
Yor1
miss 0
orf 0.7
int −6.4
rank 3526
yor1−dF
miss 0
orf −4.7
int −12.8
rank 3964
RLM1(YPL089C)
DR = 2842
Yor1
miss 0
orf 14.7
int 4.2
rank 1123
yor1−dF
miss 0
orf 6.4
int −12.8
rank 3965
KIP3(YGL216W)
DR = 1214
Yor1
miss 0
orf 3.9
int −2.7
rank 2752
yor1−dF
miss 0
orf 1.3
int −12.9
rank 3966
(YLR046C)
DR = 3712
Yor1
miss 0
orf −0.5
int 12.4
rank 255
yor1−dF
miss 0
orf 2.2
int −12.9
rank 3967
0.35
0.25
0.20
0.15
0.10
0.05
0.00
−60
−20
0
20
40
−50
0
50

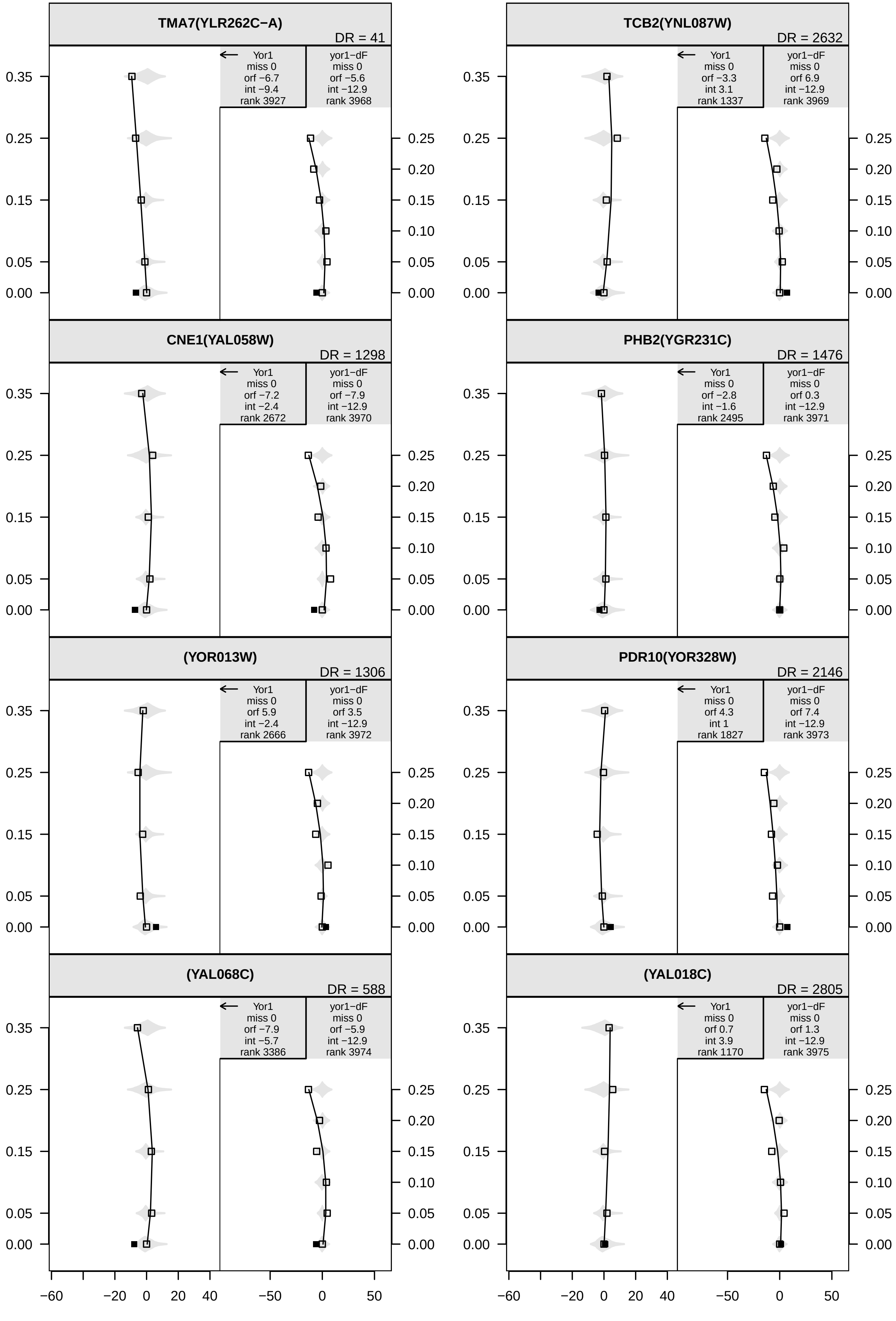

TMA7(YLR262C−A)
DR = 41
Yor1
miss 0
orf −6.7
int −9.4
rank 3927
yor1−dF
miss 0
orf −5.6
int −12.9
rank 3968
TCB2(YNL087W)
DR = 2632
Yor1
miss 0
orf −3.3
int 3.1
rank 1337
yor1−dF
miss 0
orf 6.9
int −12.9
rank 3969
CNE1(YAL058W)
DR = 1298
Yor1
miss 0
orf −7.2
int −2.4
rank 2672
yor1−dF
miss 0
orf −7.9
int −12.9
rank 3970
PHB2(YGR231C)
DR = 1476
Yor1
miss 0
orf −2.8
int −1.6
rank 2495
yor1−dF
miss 0
orf 0.3
int −12.9
rank 3971
(YOR013W)
DR = 1306
Yor1
miss 0
orf 5.9
int −2.4
rank 2666
yor1−dF
miss 0
orf 3.5
int −12.9
rank 3972
PDR10(YOR328W)
DR = 2146
Yor1
miss 0
orf 4.3
int 1
rank 1827
yor1−dF
miss 0
orf 7.4
int −12.9
rank 3973
(YAL068C)
DR = 588
Yor1
miss 0
orf −7.9
int −5.7
rank 3386
yor1−dF
miss 0
orf −5.9
int −12.9
rank 3974
(YAL018C)
DR = 2805
Yor1
miss 0
orf 0.7
int 3.9
rank 1170
yor1−dF
miss 0
orf 1.3
int −12.9
rank 3975
0.35 0.25 0.15 0.05 0.00
0.25 0.20 0.15 0.10 0.05 0.00
−60 −20 0 20 40
−50 0 50

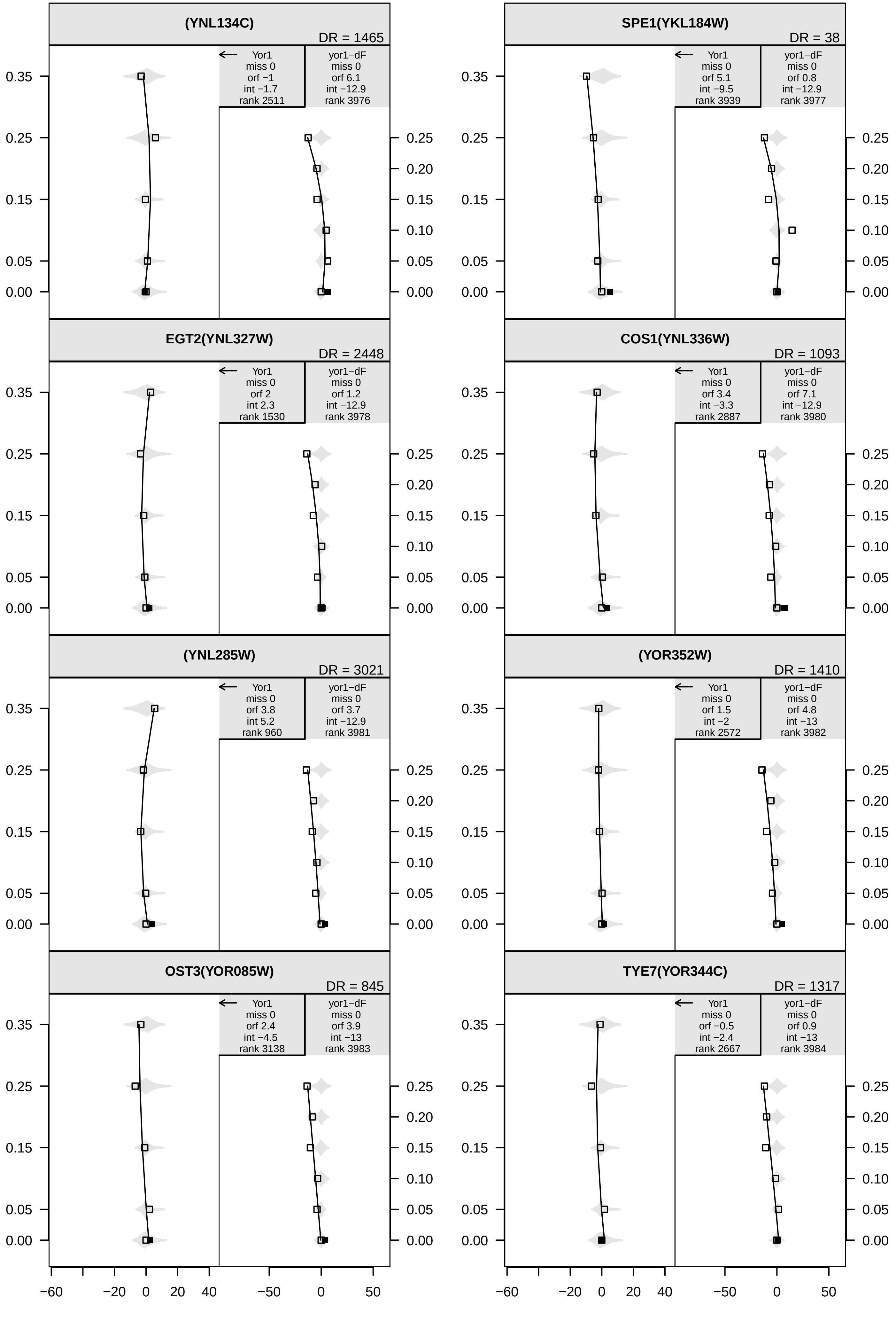

(YNL134C)
DR = 1465
Yor1 miss 0 orf −1 int −1.7 rank 2511
yor1−dF miss 0 orf 6.1 int −12.9 rank 3976
SPE1(YKL184W)
DR = 38
Yor1 miss 0 orf 5.1 int −9.5 rank 3939
yor1−dF miss 0 orf 0.8 int −12.9 rank 3977
EGT2(YNL327W)
DR = 2448
Yor1 miss 0 orf 2 int 2.3 rank 1530
yor1−dF miss 0 orf 1.2 int −12.9 rank 3978
COS1(YNL336W)
DR = 1093
Yor1 miss 0 orf 3.4 int −3.3 rank 2887
yor1−dF miss 0 orf 7.1 int −12.9 rank 3980
(YNL285W)
DR = 3021
Yor1 miss 0 orf 3.8 int 5.2 rank 960
yor1−dF miss 0 orf 3.7 int −12.9 rank 3981
(YOR352W)
DR = 1410
Yor1 miss 0 orf 1.5 int −2 rank 2572
yor1−dF miss 0 orf 4.8 int −13 rank 3982
OST3(YOR085W)
DR = 845
Yor1 miss 0 orf 2.4 int −4.5 rank 3138
yor1−dF miss 0 orf 3.9 int −13 rank 3983
TYE7(YOR344C)
DR = 1317
Yor1 miss 0 orf −0.5 int −2.4 rank 2667
yor1−dF miss 0 orf 0.9 int −13 rank 3984
0.35 0.25 0.15 0.05 0.00
0.25 0.20 0.15 0.10 0.05 0.00
−60 −20 0 20 40
−50 0 50

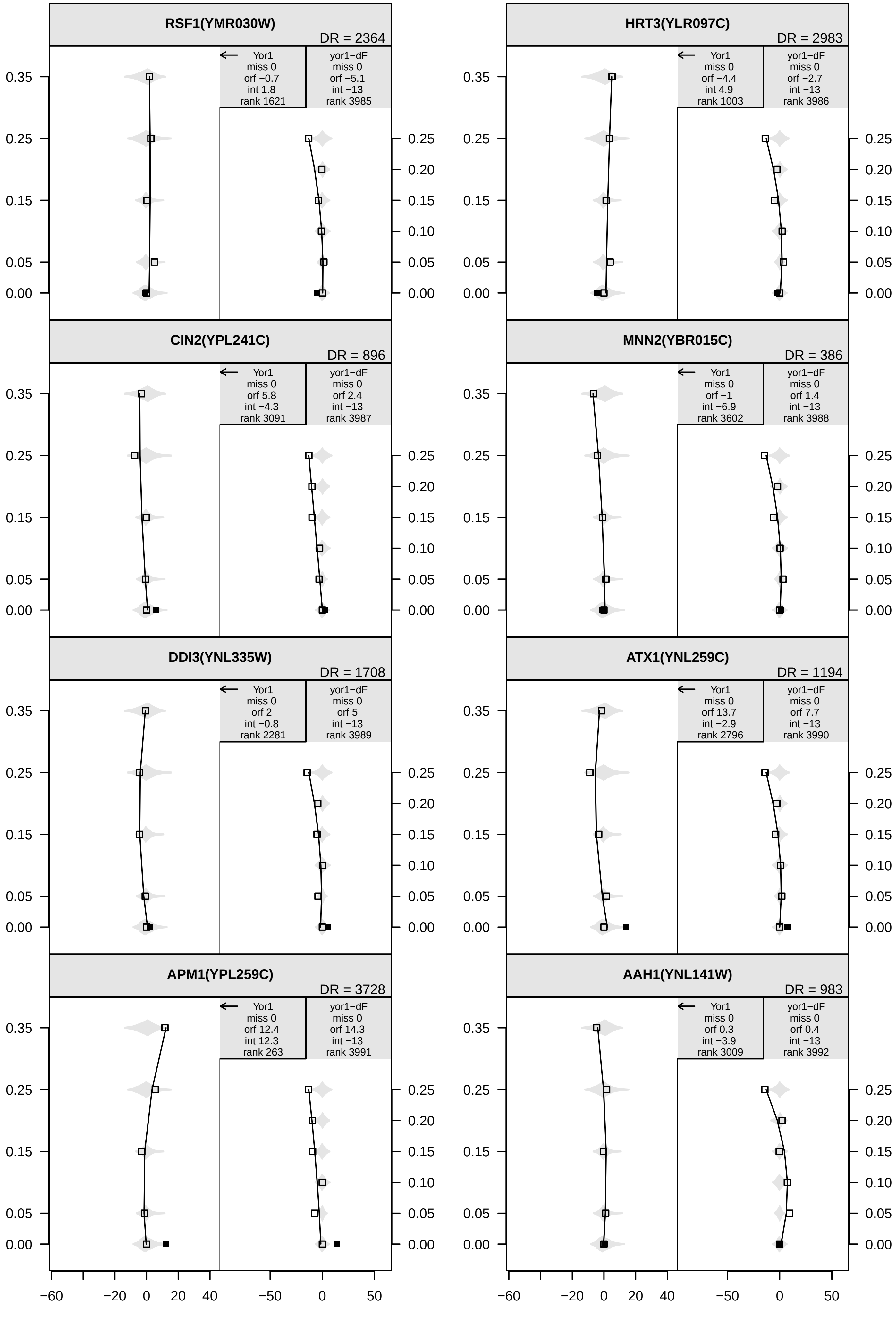

RSF1(YMR030W)
DR = 2364
Yor1 miss 0 orf −0.7 int 1.8 rank 1621
yor1−dF miss 0 orf −5.1 int −13 rank 3985
HRT3(YLR097C)
DR = 2983
Yor1 miss 0 orf −4.4 int 4.9 rank 1003
yor1−dF miss 0 orf −2.7 int −13 rank 3986
CIN2(YPL241C)
DR = 896
Yor1 miss 0 orf 5.8 int −4.3 rank 3091
yor1−dF miss 0 orf 2.4 int −13 rank 3987
MNN2(YBR015C)
DR = 386
Yor1 miss 0 orf −1 int −6.9 rank 3602
yor1−dF miss 0 orf 1.4 int −13 rank 3988
DDI3(YNL335W)
DR = 1708
Yor1 miss 0 orf 2 int −0.8 rank 2281
yor1−dF miss 0 orf 5 int −13 rank 3989
ATX1(YNL259C)
DR = 1194
Yor1 miss 0 orf 13.7 int −2.9 rank 2796
yor1−dF miss 0 orf 7.7 int −13 rank 3990
APM1(YPL259C)
DR = 3728
Yor1 miss 0 orf 12.4 int 12.3 rank 263
yor1−dF miss 0 orf 14.3 int −13 rank 3991
AAH1(YNL141W)
DR = 983
Yor1 miss 0 orf 0.3 int −3.9 rank 3009
yor1−dF miss 0 orf 0.4 int −13 rank 3992
0.35 0.25 0.15 0.05 0.00
0.25 0.20 0.15 0.10 0.05 0.00
−60 −20 0 20 40 −50 0 50

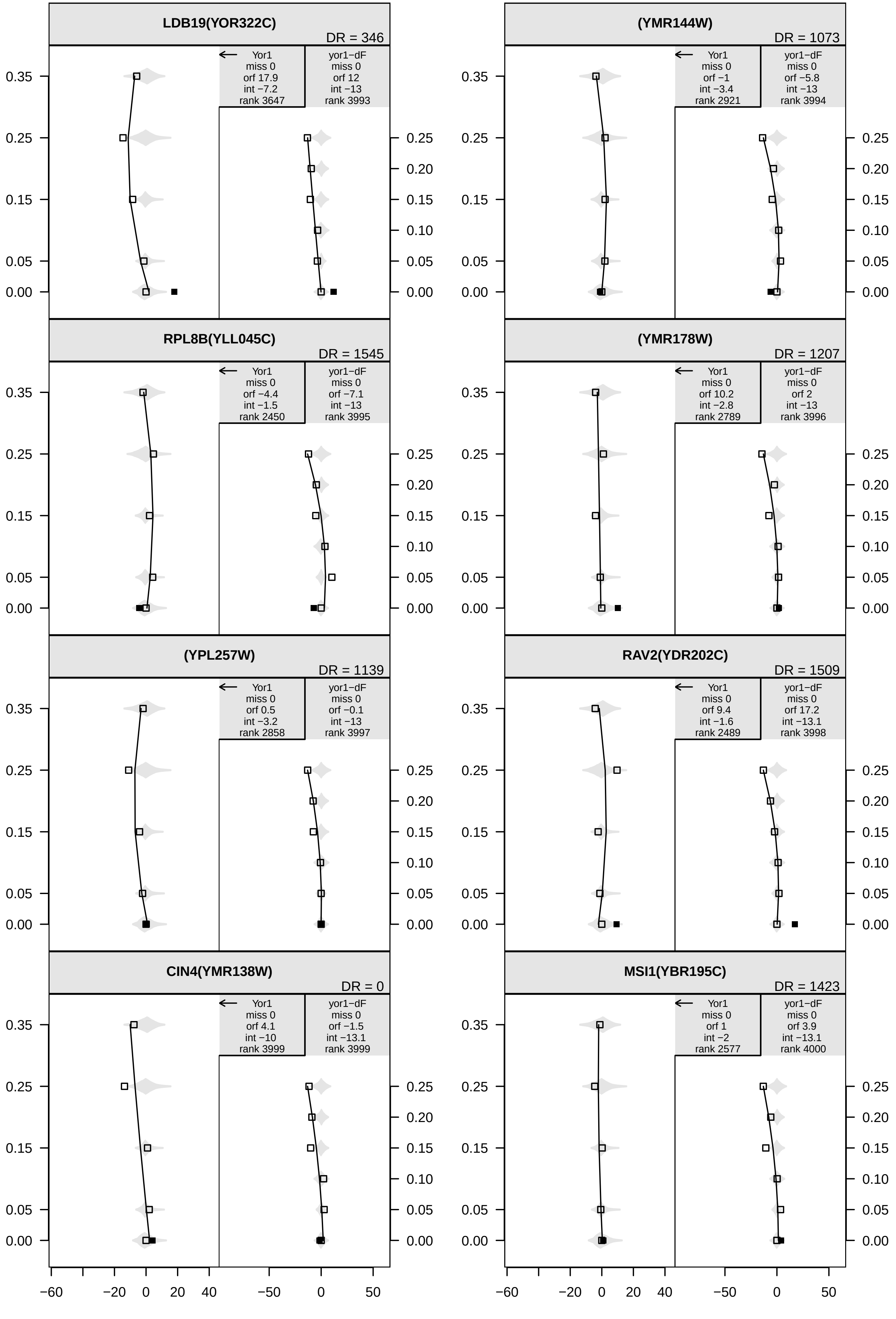
LDB19(YOR322C)
DR = 346
Yor1 miss 0 orf 17.9 int −7.2 rank 3647
yor1−dF miss 0 orf 12 int −13 rank 3993
(YMR144W)
DR = 1073
Yor1 miss 0 orf −1 int −3.4 rank 2921
yor1−dF miss 0 orf −5.8 int −13 rank 3994
RPL8B(YLL045C)
DR = 1545
Yor1 miss 0 orf −4.4 int −1.5 rank 2450
yor1−dF miss 0 orf −7.1 int −13 rank 3995
(YMR178W)
DR = 1207
Yor1 miss 0 orf 10.2 int −2.8 rank 2789
yor1−dF miss 0 orf 2 int −13 rank 3996
(YPL257W)
DR = 1139
Yor1 miss 0 orf 0.5 int −3.2 rank 2858
yor1−dF miss 0 orf −0.1 int −13 rank 3997
RAV2(YDR202C)
DR = 1509
Yor1 miss 0 orf 9.4 int −1.6 rank 2489
yor1−dF miss 0 orf 17.2 int −13.1 rank 3998
CIN4(YMR138W)
DR = 0
Yor1 miss 0 orf 4.1 int −10 rank 3999
yor1−dF miss 0 orf −1.5 int −13.1 rank 3999
MSI1(YBR195C)
DR = 1423
Yor1 miss 0 orf 1 int −2 rank 2577
yor1−dF miss 0 orf 3.9 int −13.1 rank 4000
0.35 0.25 0.15 0.05 0.00
0.25 0.20 0.15 0.10 0.05 0.00
−60 −20 0 20 40 −50 0 50

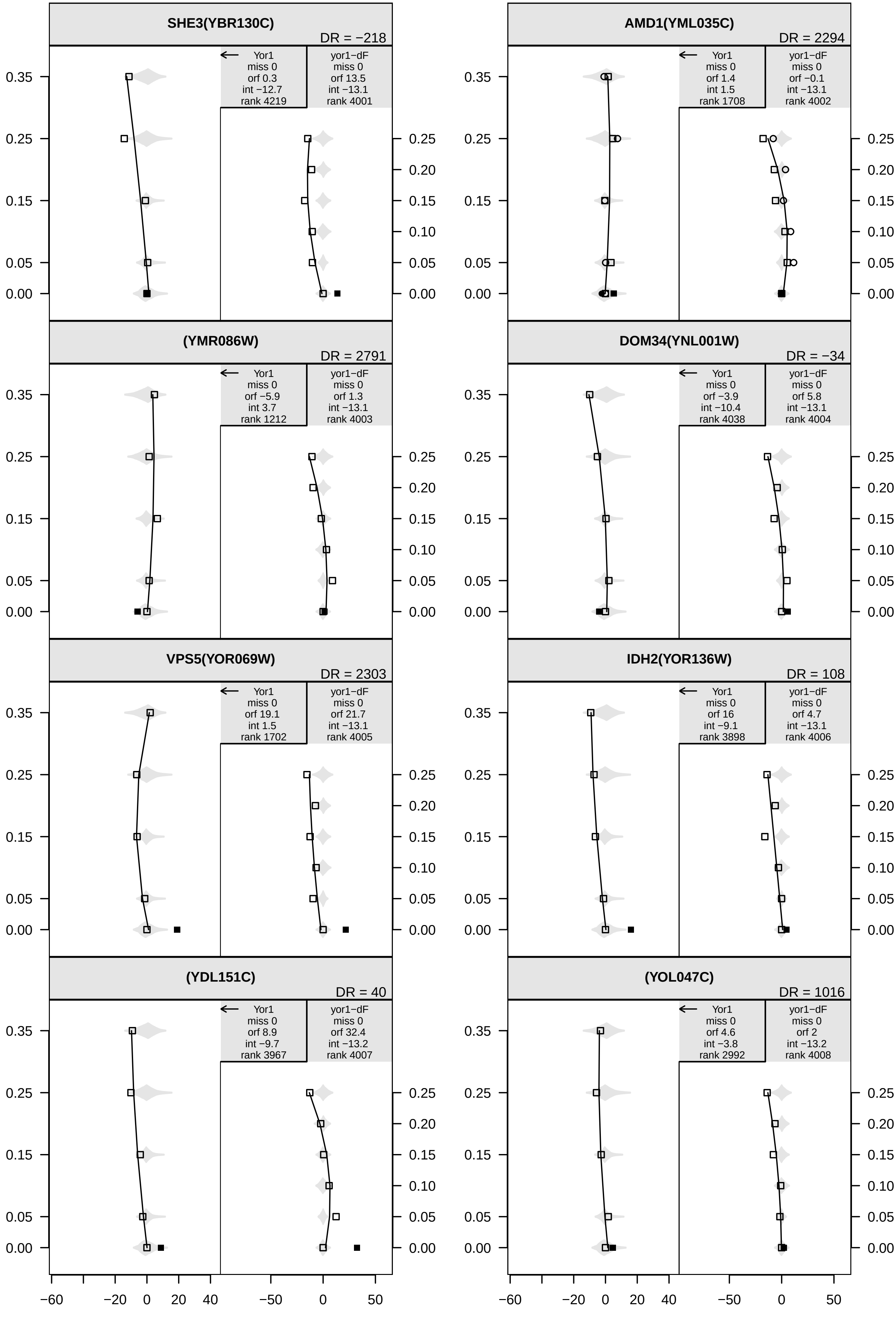

SHE3(YBR130C)
DR = −218
Yor1 miss 0 orf 0.3 int −12.7 rank 4219
yor1−dF miss 0 orf 13.5 int −13.1 rank 4001
AMD1(YML035C)
DR = 2294
Yor1 miss 0 orf 1.4 int 1.5 rank 1708
yor1−dF miss 0 orf −0.1 int −13.1 rank 4002
(YMR086W)
DR = 2791
Yor1 miss 0 orf −5.9 int 3.7 rank 1212
yor1−dF miss 0 orf 1.3 int −13.1 rank 4003
DOM34(YNL001W)
DR = −34
Yor1 miss 0 orf −3.9 int −10.4 rank 4038
yor1−dF miss 0 orf 5.8 int −13.1 rank 4004
VPS5(YOR069W)
DR = 2303
Yor1 miss 0 orf 19.1 int 1.5 rank 1702
yor1−dF miss 0 orf 21.7 int −13.1 rank 4005
IDH2(YOR136W)
DR = 108
Yor1 miss 0 orf 16 int −9.1 rank 3898
yor1−dF miss 0 orf 4.7 int −13.1 rank 4006
(YDL151C)
DR = 40
Yor1 miss 0 orf 8.9 int −9.7 rank 3967
yor1−dF miss 0 orf 32.4 int −13.2 rank 4007
(YOL047C)
DR = 1016
Yor1 miss 0 orf 4.6 int −3.8 rank 2992
yor1−dF miss 0 orf 2 int −13.2 rank 4008
0.35 0.25 0.15 0.05 0.00
0.25 0.20 0.15 0.10 0.05 0.00
−60 −20 0 20 40 −50 0 50

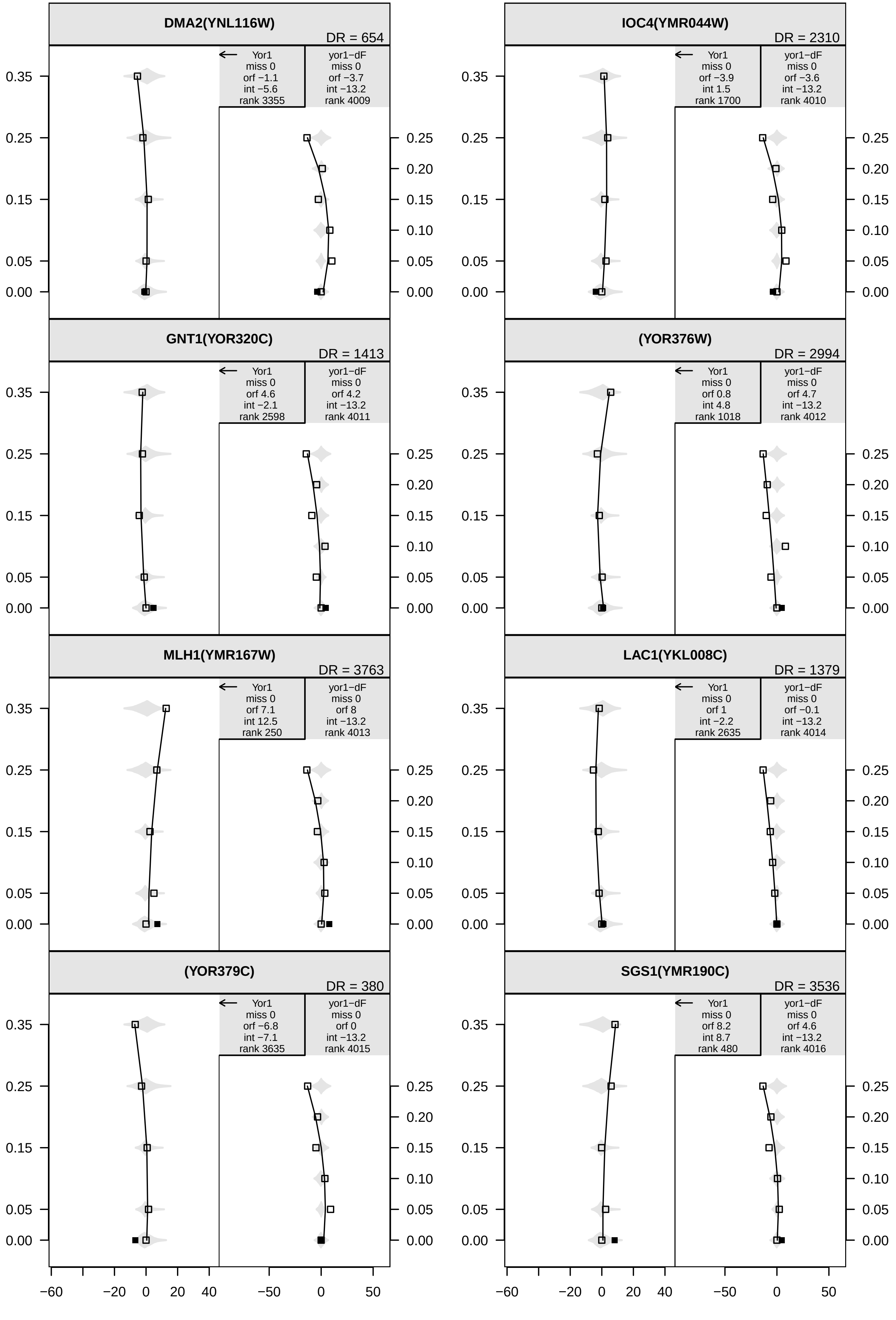

DMA2(YNL116W)
DR = 654
Yor1 miss 0 orf −1.1 int −5.6 rank 3355
yor1−dF miss 0 orf −3.7 int −13.2 rank 4009
IOC4(YMR044W)
DR = 2310
Yor1 miss 0 orf −3.9 int 1.5 rank 1700
yor1−dF miss 0 orf −3.6 int −13.2 rank 4010
GNT1(YOR320C)
DR = 1413
Yor1 miss 0 orf 4.6 int −2.1 rank 2598
yor1−dF miss 0 orf 4.2 int −13.2 rank 4011
(YOR376W)
DR = 2994
Yor1 miss 0 orf 0.8 int 4.8 rank 1018
yor1−dF miss 0 orf 4.7 int −13.2 rank 4012
MLH1(YMR167W)
DR = 3763
Yor1 miss 0 orf 7.1 int 12.5 rank 250
yor1−dF miss 0 orf 8 int −13.2 rank 4013
LAC1(YKL008C)
DR = 1379
Yor1 miss 0 orf 1 int −2.2 rank 2635
yor1−dF miss 0 orf −0.1 int −13.2 rank 4014
(YOR379C)
DR = 380
Yor1 miss 0 orf −6.8 int −7.1 rank 3635
yor1−dF miss 0 orf 0 int −13.2 rank 4015
SGS1(YMR190C)
DR = 3536
Yor1 miss 0 orf 8.2 int 8.7 rank 480
yor1−dF miss 0 orf 4.6 int −13.2 rank 4016
0.35 0.25 0.20 0.15 0.10 0.05 0.00
−60 −20 0 20 40 −50 0 50

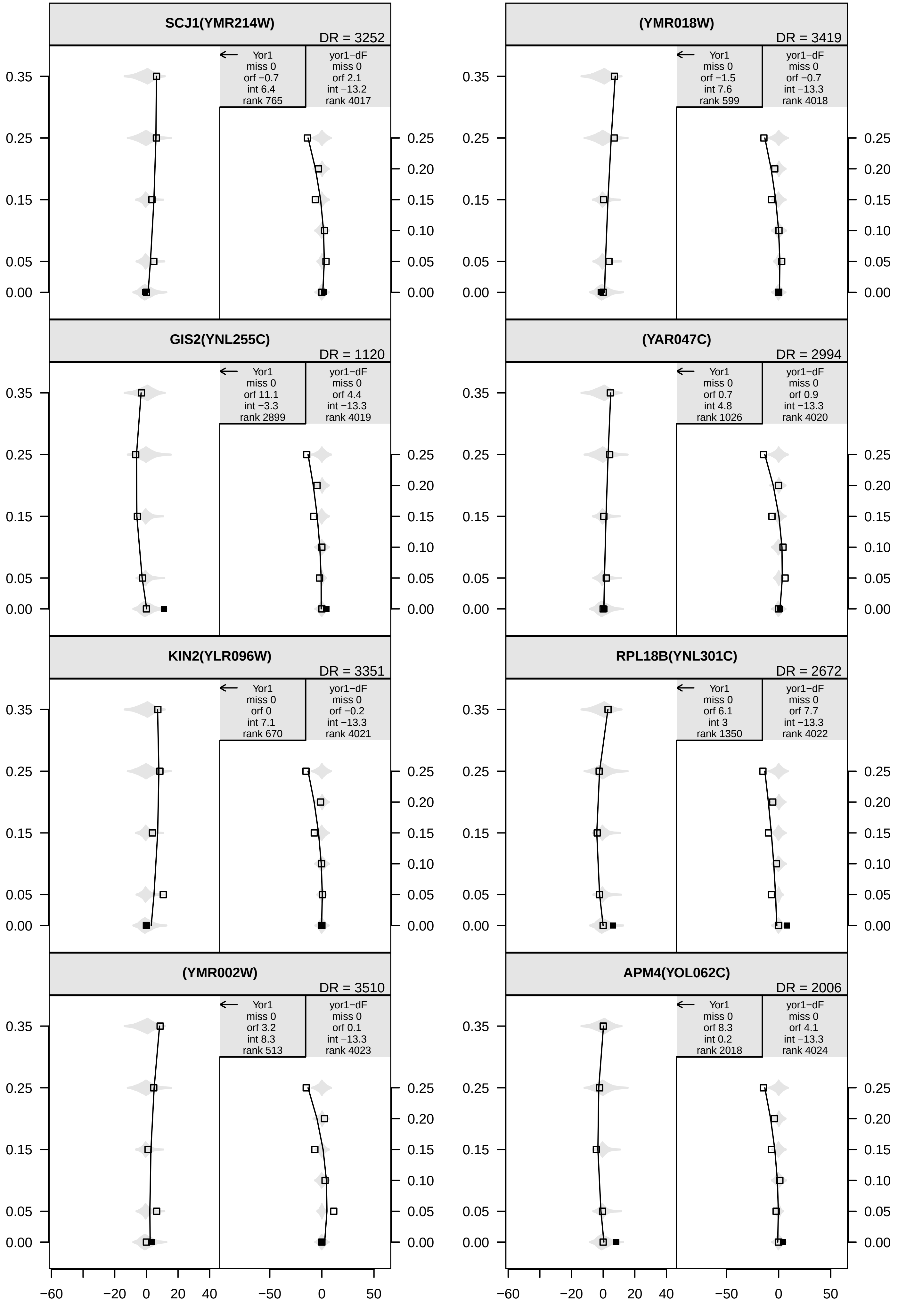
SCJ1(YMR214W)
DR = 3252
Yor1 miss 0 orf −0.7 int 6.4 rank 765
yor1−dF miss 0 orf 2.1 int −13.2 rank 4017
(YMR018W)
DR = 3419
Yor1 miss 0 orf −1.5 int 7.6 rank 599
yor1−dF miss 0 orf −0.7 int −13.3 rank 4018
GIS2(YNL255C)
DR = 1120
Yor1 miss 0 orf 11.1 int −3.3 rank 2899
yor1−dF miss 0 orf 4.4 int −13.3 rank 4019
(YAR047C)
DR = 2994
Yor1 miss 0 orf 0.7 int 4.8 rank 1026
yor1−dF miss 0 orf 0.9 int −13.3 rank 4020
KIN2(YLR096W)
DR = 3351
Yor1 miss 0 orf 0 int 7.1 rank 670
yor1−dF miss 0 orf −0.2 int −13.3 rank 4021
RPL18B(YNL301C)
DR = 2672
Yor1 miss 0 orf 6.1 int 3 rank 1350
yor1−dF miss 0 orf 7.7 int −13.3 rank 4022
(YMR002W)
DR = 3510
Yor1 miss 0 orf 3.2 int 8.3 rank 513
yor1−dF miss 0 orf 0.1 int −13.3 rank 4023
APM4(YOL062C)
DR = 2006
Yor1 miss 0 orf 8.3 int 0.2 rank 2018
yor1−dF miss 0 orf 4.1 int −13.3 rank 4024
0.00 0.05 0.10 0.15 0.20 0.25 0.35
−60 −20 0 20 40 −50 0 50

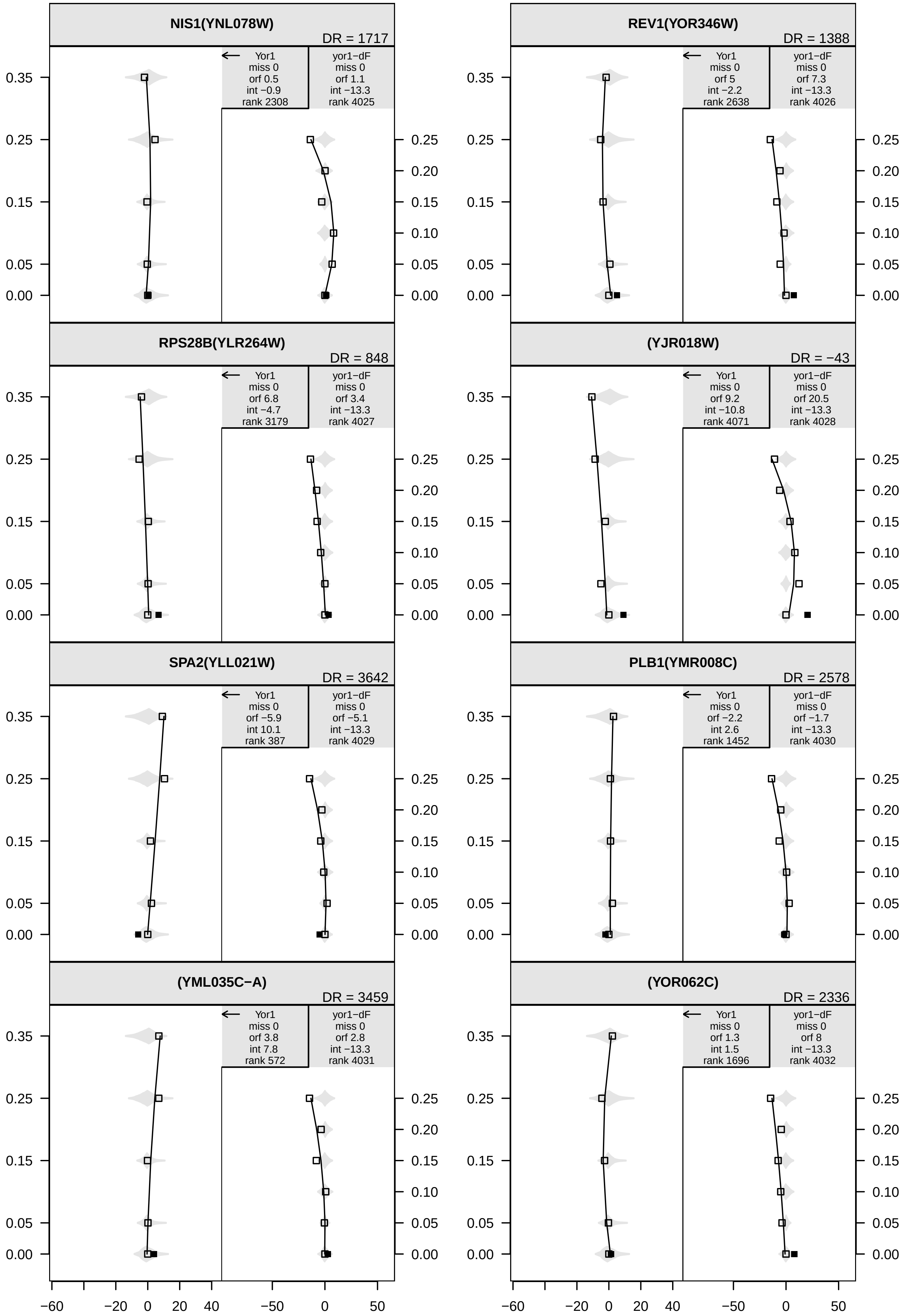

NIS1(YNL078W)
DR = 1717
Yor1 miss 0 orf 0.5 int −0.9 rank 2308
yor1−dF miss 0 orf 1.1 int −13.3 rank 4025
REV1(YOR346W)
DR = 1388
Yor1 miss 0 orf 5 int −2.2 rank 2638
yor1−dF miss 0 orf 7.3 int −13.3 rank 4026
RPS28B(YLR264W)
DR = 848
Yor1 miss 0 orf 6.8 int −4.7 rank 3179
yor1−dF miss 0 orf 3.4 int −13.3 rank 4027
(YJR018W)
DR = −43
Yor1 miss 0 orf 9.2 int −10.8 rank 4071
yor1−dF miss 0 orf 20.5 int −13.3 rank 4028
SPA2(YLL021W)
DR = 3642
Yor1 miss 0 orf −5.9 int 10.1 rank 387
yor1−dF miss 0 orf −5.1 int −13.3 rank 4029
PLB1(YMR008C)
DR = 2578
Yor1 miss 0 orf −2.2 int 2.6 rank 1452
yor1−dF miss 0 orf −1.7 int −13.3 rank 4030
(YML035C−A)
DR = 3459
Yor1 miss 0 orf 3.8 int 7.8 rank 572
yor1−dF miss 0 orf 2.8 int −13.3 rank 4031
(YOR062C)
DR = 2336
Yor1 miss 0 orf 1.3 int 1.5 rank 1696
yor1−dF miss 0 orf 8 int −13.3 rank 4032

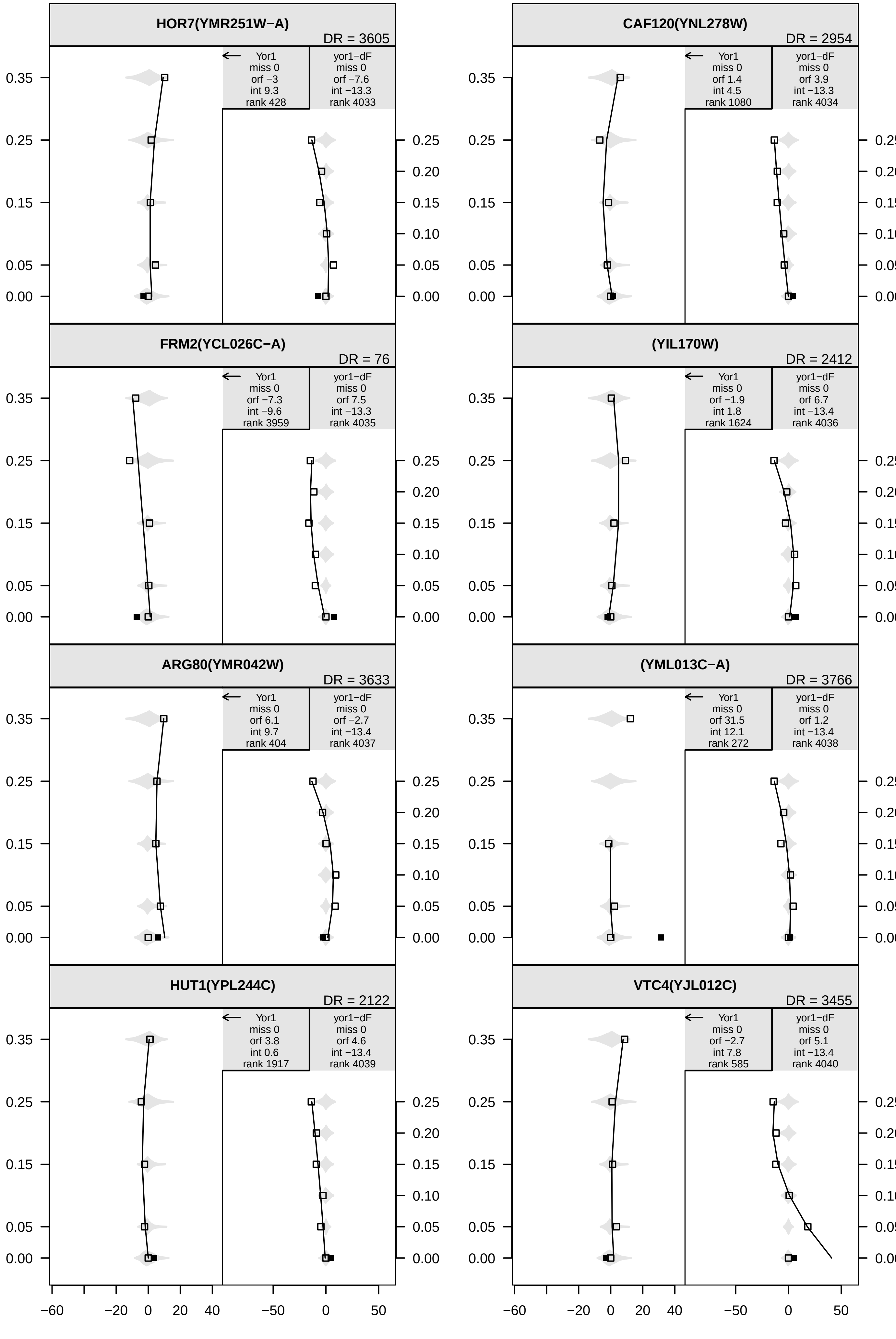

HOR7(YMR251W−A)
DR = 3605
Yor1
miss 0
orf −3
int 9.3
rank 428
yor1−dF
miss 0
orf −7.6
int −13.3
rank 4033
CAF120(YNL278W)
DR = 2954
Yor1
miss 0
orf 1.4
int 4.5
rank 1080
yor1−dF
miss 0
orf 3.9
int −13.3
rank 4034
FRM2(YCL026C−A)
DR = 76
Yor1
miss 0
orf −7.3
int −9.6
rank 3959
yor1−dF
miss 0
orf 7.5
int −13.3
rank 4035
(YIL170W)
DR = 2412
Yor1
miss 0
orf −1.9
int 1.8
rank 1624
yor1−dF
miss 0
orf 6.7
int −13.4
rank 4036
ARG80(YMR042W)
DR = 3633
Yor1
miss 0
orf 6.1
int 9.7
rank 404
yor1−dF
miss 0
orf −2.7
int −13.4
rank 4037
(YML013C−A)
DR = 3766
Yor1
miss 0
orf 31.5
int 12.1
rank 272
yor1−dF
miss 0
orf 1.2
int −13.4
rank 4038
HUT1(YPL244C)
DR = 2122
Yor1
miss 0
orf 3.8
int 0.6
rank 1917
yor1−dF
miss 0
orf 4.6
int −13.4
rank 4039
VTC4(YJL012C)
DR = 3455
Yor1
miss 0
orf −2.7
int 7.8
rank 585
yor1−dF
miss 0
orf 5.1
int −13.4
rank 4040

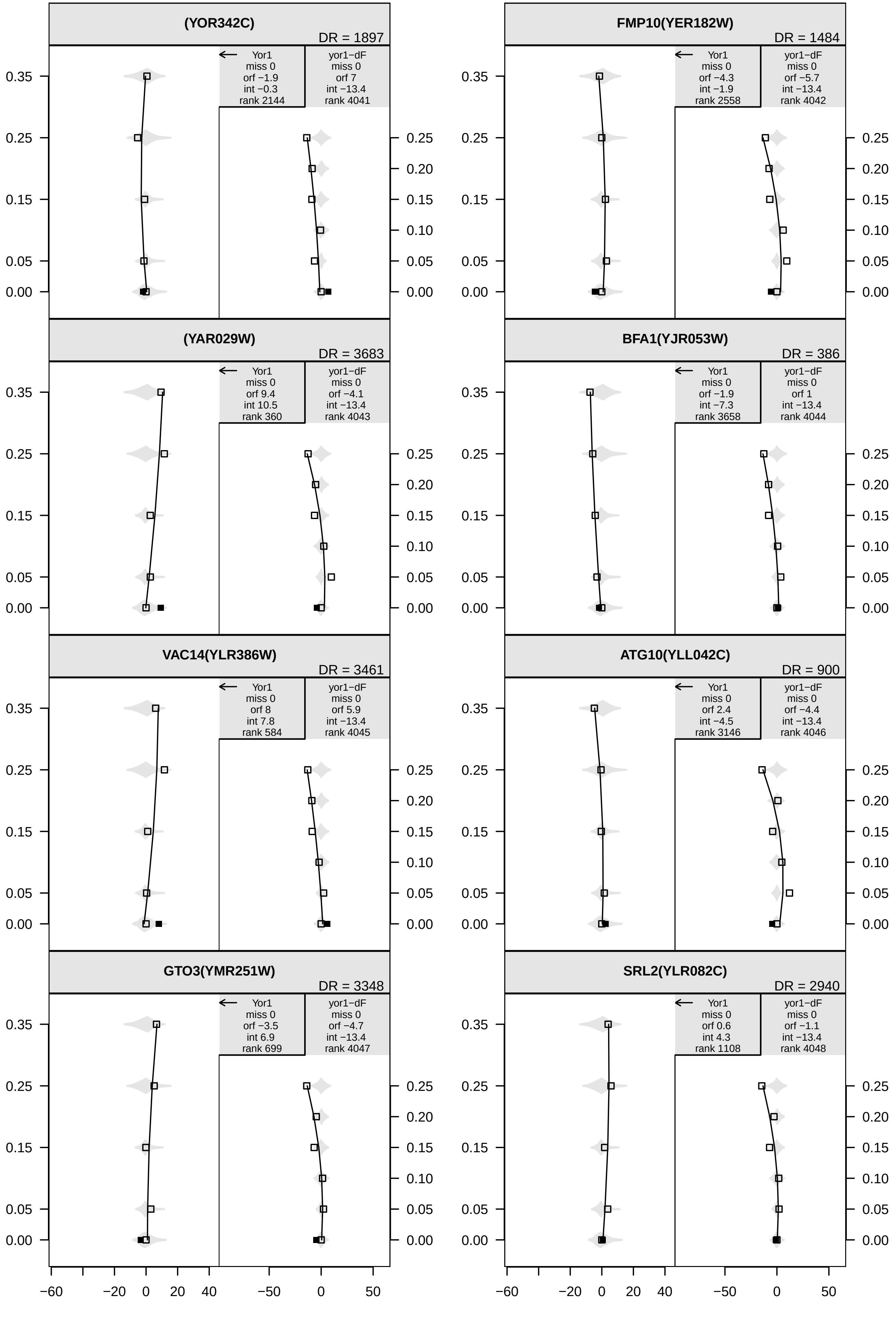

(YOR342C)
DR = 1897
Yor1 miss 0 orf −1.9 int −0.3 rank 2144
yor1−dF miss 0 orf 7 int −13.4 rank 4041
FMP10(YER182W)
DR = 1484
Yor1 miss 0 orf −4.3 int −1.9 rank 2558
yor1−dF miss 0 orf −5.7 int −13.4 rank 4042
(YAR029W)
DR = 3683
Yor1 miss 0 orf 9.4 int 10.5 rank 360
yor1−dF miss 0 orf −4.1 int −13.4 rank 4043
BFA1(YJR053W)
DR = 386
Yor1 miss 0 orf −1.9 int −7.3 rank 3658
yor1−dF miss 0 orf 1 int −13.4 rank 4044
VAC14(YLR386W)
DR = 3461
Yor1 miss 0 orf 8 int 7.8 rank 584
yor1−dF miss 0 orf 5.9 int −13.4 rank 4045
ATG10(YLL042C)
DR = 900
Yor1 miss 0 orf 2.4 int −4.5 rank 3146
yor1−dF miss 0 orf −4.4 int −13.4 rank 4046
GTO3(YMR251W)
DR = 3348
Yor1 miss 0 orf −3.5 int 6.9 rank 699
yor1−dF miss 0 orf −4.7 int −13.4 rank 4047
SRL2(YLR082C)
DR = 2940
Yor1 miss 0 orf 0.6 int 4.3 rank 1108
yor1−dF miss 0 orf −1.1 int −13.4 rank 4048
0.35 0.25 0.15 0.05 0.00
0.25 0.20 0.15 0.10 0.05 0.00
−60 −20 0 20 40 −50 0 50

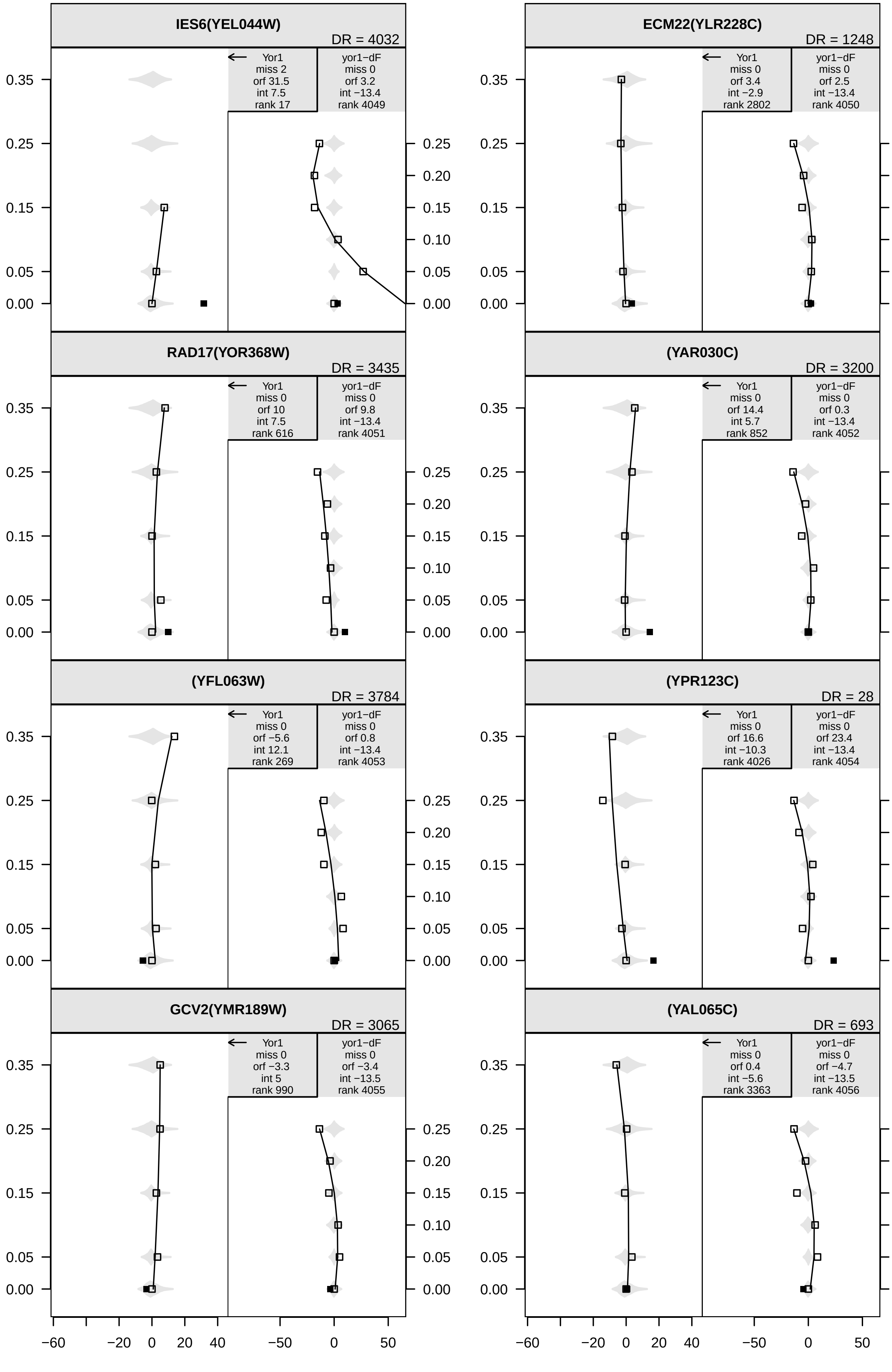
IES6(YEL044W)
DR = 4032
Yor1
miss 2
orf 31.5
int 7.5
rank 17
yor1−dF
miss 0
orf 3.2
int −13.4
rank 4049
ECM22(YLR228C)
DR = 1248
Yor1
miss 0
orf 3.4
int −2.9
rank 2802
yor1−dF
miss 0
orf 2.5
int −13.4
rank 4050
RAD17(YOR368W)
DR = 3435
Yor1
miss 0
orf 10
int 7.5
rank 616
yor1−dF
miss 0
orf 9.8
int −13.4
rank 4051
(YAR030C)
DR = 3200
Yor1
miss 0
orf 14.4
int 5.7
rank 852
yor1−dF
miss 0
orf 0.3
int −13.4
rank 4052
(YFL063W)
DR = 3784
Yor1
miss 0
orf −5.6
int 12.1
rank 269
yor1−dF
miss 0
orf 0.8
int −13.4
rank 4053
(YPR123C)
DR = 28
Yor1
miss 0
orf 16.6
int −10.3
rank 4026
yor1−dF
miss 0
orf 23.4
int −13.4
rank 4054
GCV2(YMR189W)
DR = 3065
Yor1
miss 0
orf −3.3
int 5
rank 990
yor1−dF
miss 0
orf −3.4
int −13.5
rank 4055
(YAL065C)
DR = 693
Yor1
miss 0
orf 0.4
int −5.6
rank 3363
yor1−dF
miss 0
orf −4.7
int −13.5
rank 4056
0.00
0.05
0.10
0.15
0.20
0.25
0.35
−60
−20
0
20
40
−50
0
50

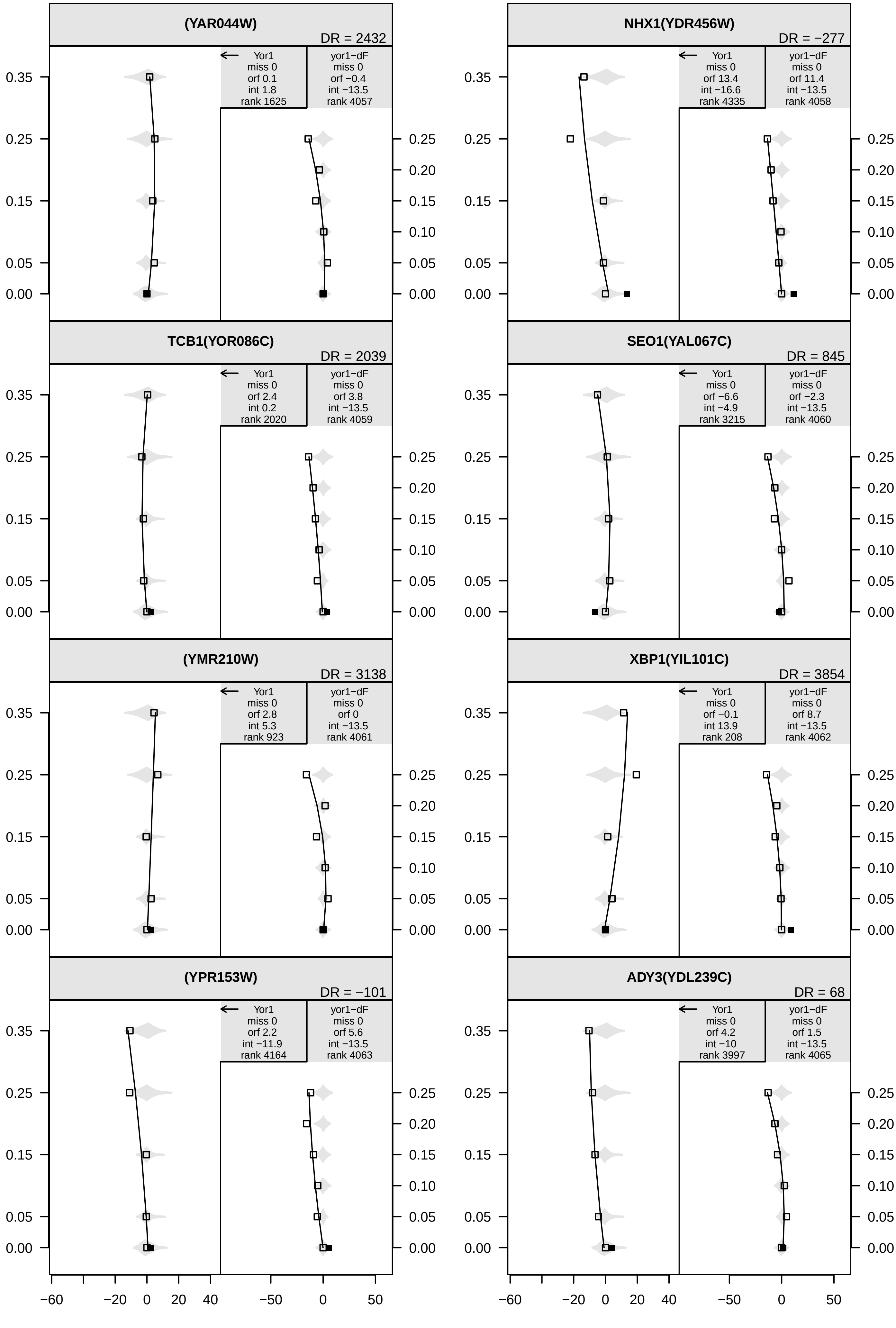

(YAR044W)
DR = 2432
Yor1 miss 0 orf 0.1 int 1.8 rank 1625
yor1−dF miss 0 orf −0.4 int −13.5 rank 4057
NHX1(YDR456W)
DR = −277
Yor1 miss 0 orf 13.4 int −16.6 rank 4335
yor1−dF miss 0 orf 11.4 int −13.5 rank 4058
TCB1(YOR086C)
DR = 2039
Yor1 miss 0 orf 2.4 int 0.2 rank 2020
yor1−dF miss 0 orf 3.8 int −13.5 rank 4059
SEO1(YAL067C)
DR = 845
Yor1 miss 0 orf −6.6 int −4.9 rank 3215
yor1−dF miss 0 orf −2.3 int −13.5 rank 4060
(YMR210W)
DR = 3138
Yor1 miss 0 orf 2.8 int 5.3 rank 923
yor1−dF miss 0 orf 0 int −13.5 rank 4061
XBP1(YIL101C)
DR = 3854
Yor1 miss 0 orf −0.1 int 13.9 rank 208
yor1−dF miss 0 orf 8.7 int −13.5 rank 4062
(YPR153W)
DR = −101
Yor1 miss 0 orf 2.2 int −11.9 rank 4164
yor1−dF miss 0 orf 5.6 int −13.5 rank 4063
ADY3(YDL239C)
DR = 68
Yor1 miss 0 orf 4.2 int −10 rank 3997
yor1−dF miss 0 orf 1.5 int −13.5 rank 4065
0.00 0.05 0.10 0.15 0.20 0.25 0.35
−60 −20 0 20 40
−50 0 50

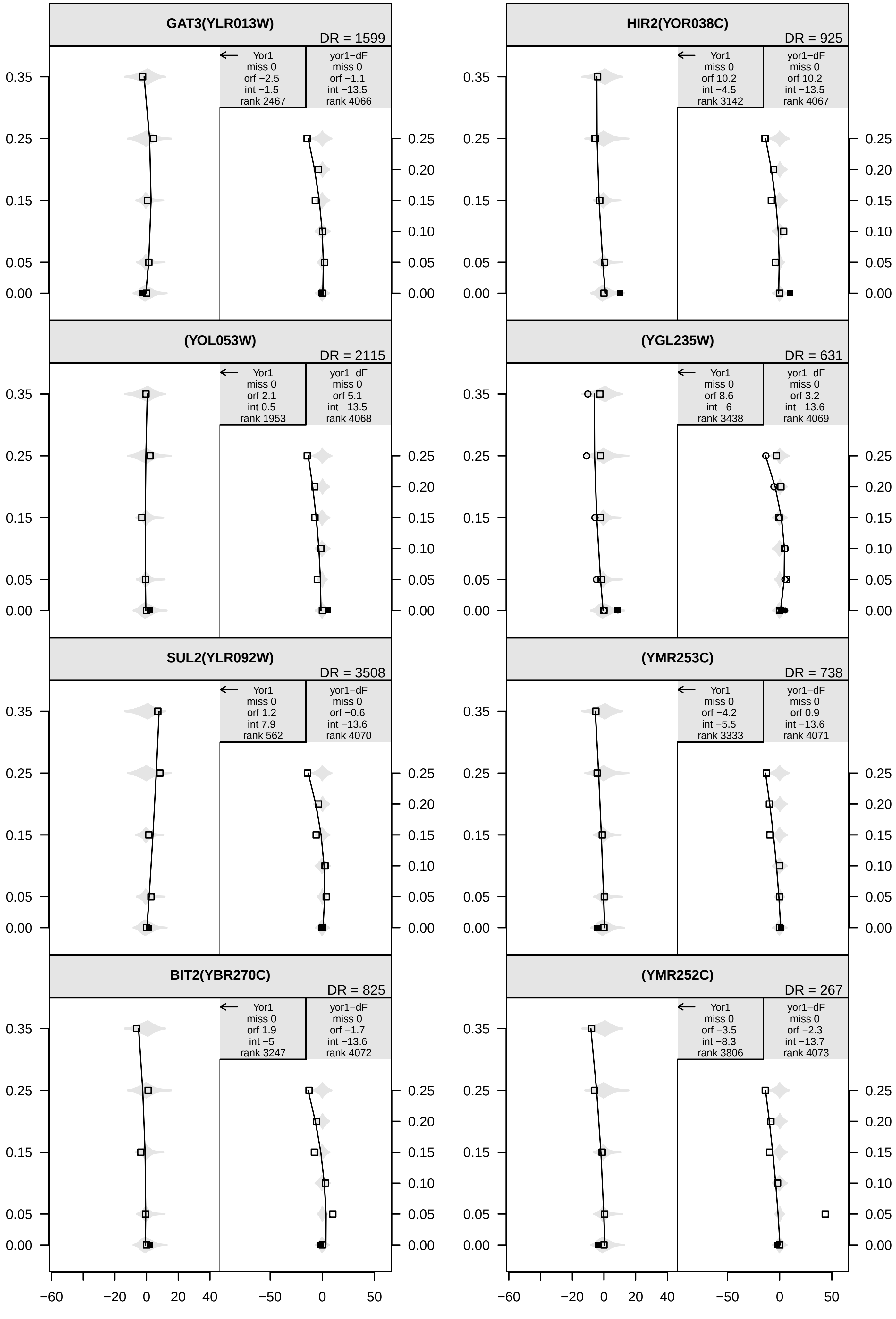
GAT3(YLR013W)
DR = 1599
Yor1 miss 0 orf −2.5 int −1.5 rank 2467
yor1−dF miss 0 orf −1.1 int −13.5 rank 4066
HIR2(YOR038C)
DR = 925
Yor1 miss 0 orf 10.2 int −4.5 rank 3142
yor1−dF miss 0 orf 10.2 int −13.5 rank 4067
(YOL053W)
DR = 2115
Yor1 miss 0 orf 2.1 int 0.5 rank 1953
yor1−dF miss 0 orf 5.1 int −13.5 rank 4068
(YGL235W)
DR = 631
Yor1 miss 0 orf 8.6 int −6 rank 3438
yor1−dF miss 0 orf 3.2 int −13.6 rank 4069
SUL2(YLR092W)
DR = 3508
Yor1 miss 0 orf 1.2 int 7.9 rank 562
yor1−dF miss 0 orf −0.6 int −13.6 rank 4070
(YMR253C)
DR = 738
Yor1 miss 0 orf −4.2 int −5.5 rank 3333
yor1−dF miss 0 orf 0.9 int −13.6 rank 4071
BIT2(YBR270C)
DR = 825
Yor1 miss 0 orf 1.9 int −5 rank 3247
yor1−dF miss 0 orf −1.7 int −13.6 rank 4072
(YMR252C)
DR = 267
Yor1 miss 0 orf −3.5 int −8.3 rank 3806
yor1−dF miss 0 orf −2.3 int −13.7 rank 4073
0.35 0.25 0.20 0.15 0.10 0.05 0.00
−60 −20 0 20 40 −50 0 50

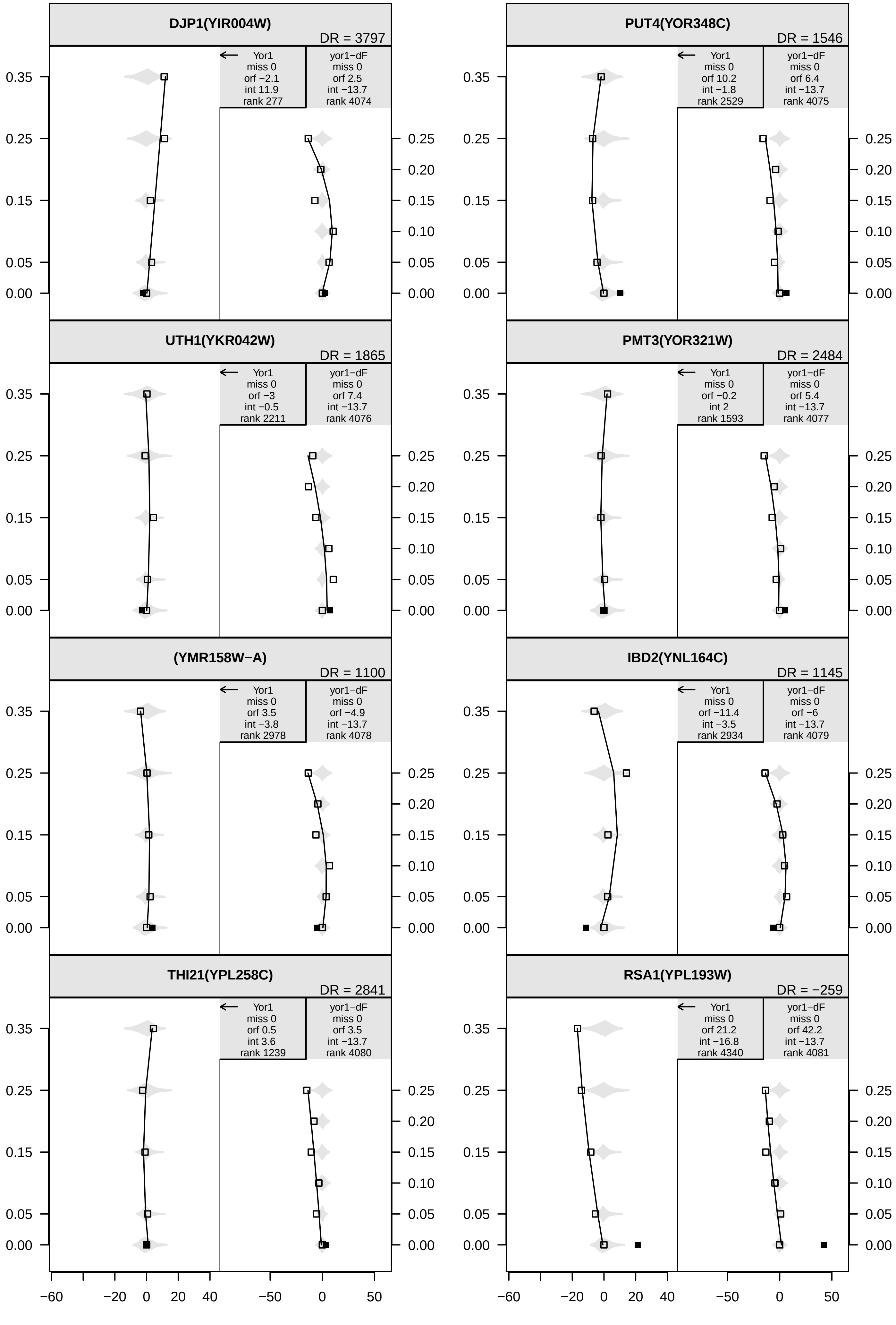

DJP1(YIR004W)
DR = 3797
Yor1
miss 0
orf −2.1
int 11.9
rank 277
yor1−dF
miss 0
orf 2.5
int −13.7
rank 4074
PUT4(YOR348C)
DR = 1546
Yor1
miss 0
orf 10.2
int −1.8
rank 2529
yor1−dF
miss 0
orf 6.4
int −13.7
rank 4075
UTH1(YKR042W)
DR = 1865
Yor1
miss 0
orf −3
int −0.5
rank 2211
yor1−dF
miss 0
orf 7.4
int −13.7
rank 4076
PMT3(YOR321W)
DR = 2484
Yor1
miss 0
orf −0.2
int 2
rank 1593
yor1−dF
miss 0
orf 5.4
int −13.7
rank 4077
(YMR158W−A)
DR = 1100
Yor1
miss 0
orf 3.5
int −3.8
rank 2978
yor1−dF
miss 0
orf −4.9
int −13.7
rank 4078
IBD2(YNL164C)
DR = 1145
Yor1
miss 0
orf −11.4
int −3.5
rank 2934
yor1−dF
miss 0
orf −6
int −13.7
rank 4079
THI21(YPL258C)
DR = 2841
Yor1
miss 0
orf 0.5
int 3.6
rank 1239
yor1−dF
miss 0
orf 3.5
int −13.7
rank 4080
RSA1(YPL193W)
DR = −259
Yor1
miss 0
orf 21.2
int −16.8
rank 4340
yor1−dF
miss 0
orf 42.2
int −13.7
rank 4081
0.35
0.25
0.20
0.15
0.10
0.05
0.00
−60
−20
0
20
40
−50
50

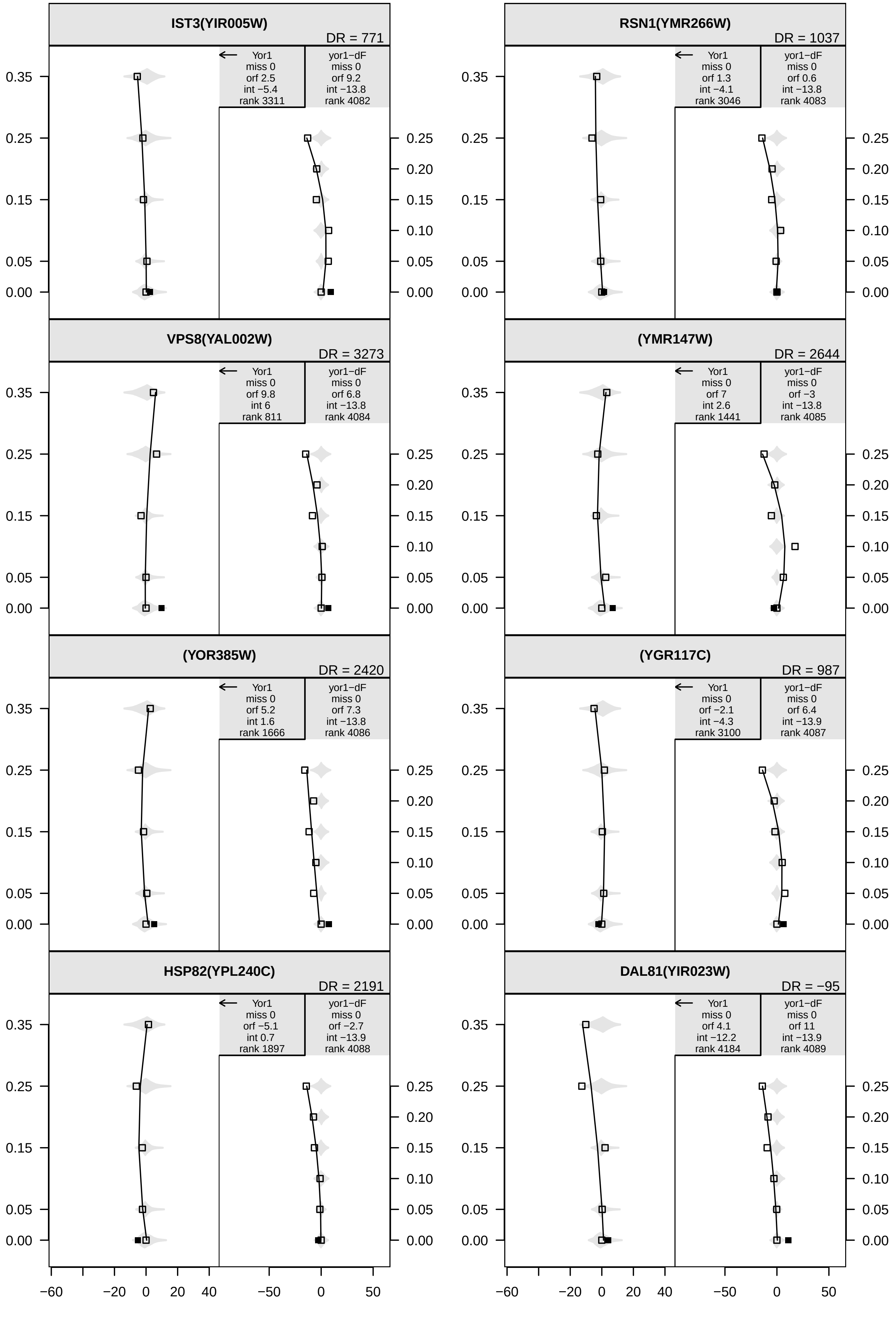

IST3(YIR005W)
DR = 771
Yor1 miss 0 orf 2.5 int −5.4 rank 3311
yor1−dF miss 0 orf 9.2 int −13.8 rank 4082
RSN1(YMR266W)
DR = 1037
Yor1 miss 0 orf 1.3 int −4.1 rank 3046
yor1−dF miss 0 orf 0.6 int −13.8 rank 4083
VPS8(YAL002W)
DR = 3273
Yor1 miss 0 orf 9.8 int 6 rank 811
yor1−dF miss 0 orf 6.8 int −13.8 rank 4084
(YMR147W)
DR = 2644
Yor1 miss 0 orf 7 int 2.6 rank 1441
yor1−dF miss 0 orf −3 int −13.8 rank 4085
(YOR385W)
DR = 2420
Yor1 miss 0 orf 5.2 int 1.6 rank 1666
yor1−dF miss 0 orf 7.3 int −13.8 rank 4086
(YGR117C)
DR = 987
Yor1 miss 0 orf −2.1 int −4.3 rank 3100
yor1−dF miss 0 orf 6.4 int −13.9 rank 4087
HSP82(YPL240C)
DR = 2191
Yor1 miss 0 orf −5.1 int 0.7 rank 1897
yor1−dF miss 0 orf −2.7 int −13.9 rank 4088
DAL81(YIR023W)
DR = −95
Yor1 miss 0 orf 4.1 int −12.2 rank 4184
yor1−dF miss 0 orf 11 int −13.9 rank 4089
0.35 0.25 0.15 0.05 0.00
0.25 0.20 0.15 0.10 0.05 0.00
−60 −20 0 20 40
−50 0 50

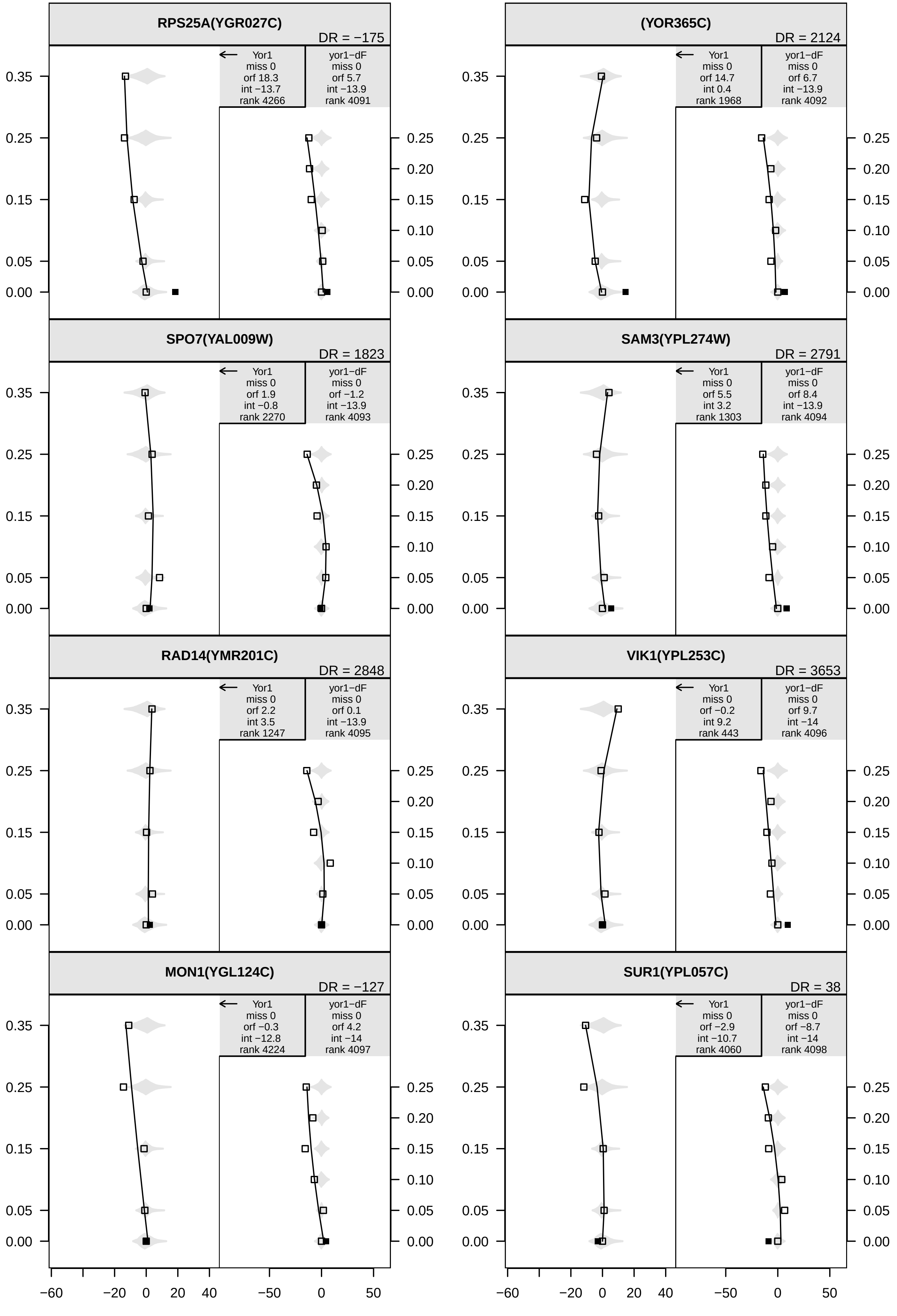
RPS25A(YGR027C)
DR = −175
Yor1
miss 0
orf 18.3
int −13.7
rank 4266
yor1−dF
miss 0
orf 5.7
int −13.9
rank 4091
(YOR365C)
DR = 2124
Yor1
miss 0
orf 14.7
int 0.4
rank 1968
yor1−dF
miss 0
orf 6.7
int −13.9
rank 4092
SPO7(YAL009W)
DR = 1823
Yor1
miss 0
orf 1.9
int −0.8
rank 2270
yor1−dF
miss 0
orf −1.2
int −13.9
rank 4093
SAM3(YPL274W)
DR = 2791
Yor1
miss 0
orf 5.5
int 3.2
rank 1303
yor1−dF
miss 0
orf 8.4
int −13.9
rank 4094
RAD14(YMR201C)
DR = 2848
Yor1
miss 0
orf 2.2
int 3.5
rank 1247
yor1−dF
miss 0
orf 0.1
int −13.9
rank 4095
VIK1(YPL253C)
DR = 3653
Yor1
miss 0
orf −0.2
int 9.2
rank 443
yor1−dF
miss 0
orf 9.7
int −14
rank 4096
MON1(YGL124C)
DR = −127
Yor1
miss 0
orf −0.3
int −12.8
rank 4224
yor1−dF
miss 0
orf 4.2
int −14
rank 4097
SUR1(YPL057C)
DR = 38
Yor1
miss 0
orf −2.9
int −10.7
rank 4060
yor1−dF
miss 0
orf −8.7
int −14
rank 4098
0.35
0.25
0.20
0.15
0.10
0.05
0.00
−60
−20
0
20
40
−50
0
50

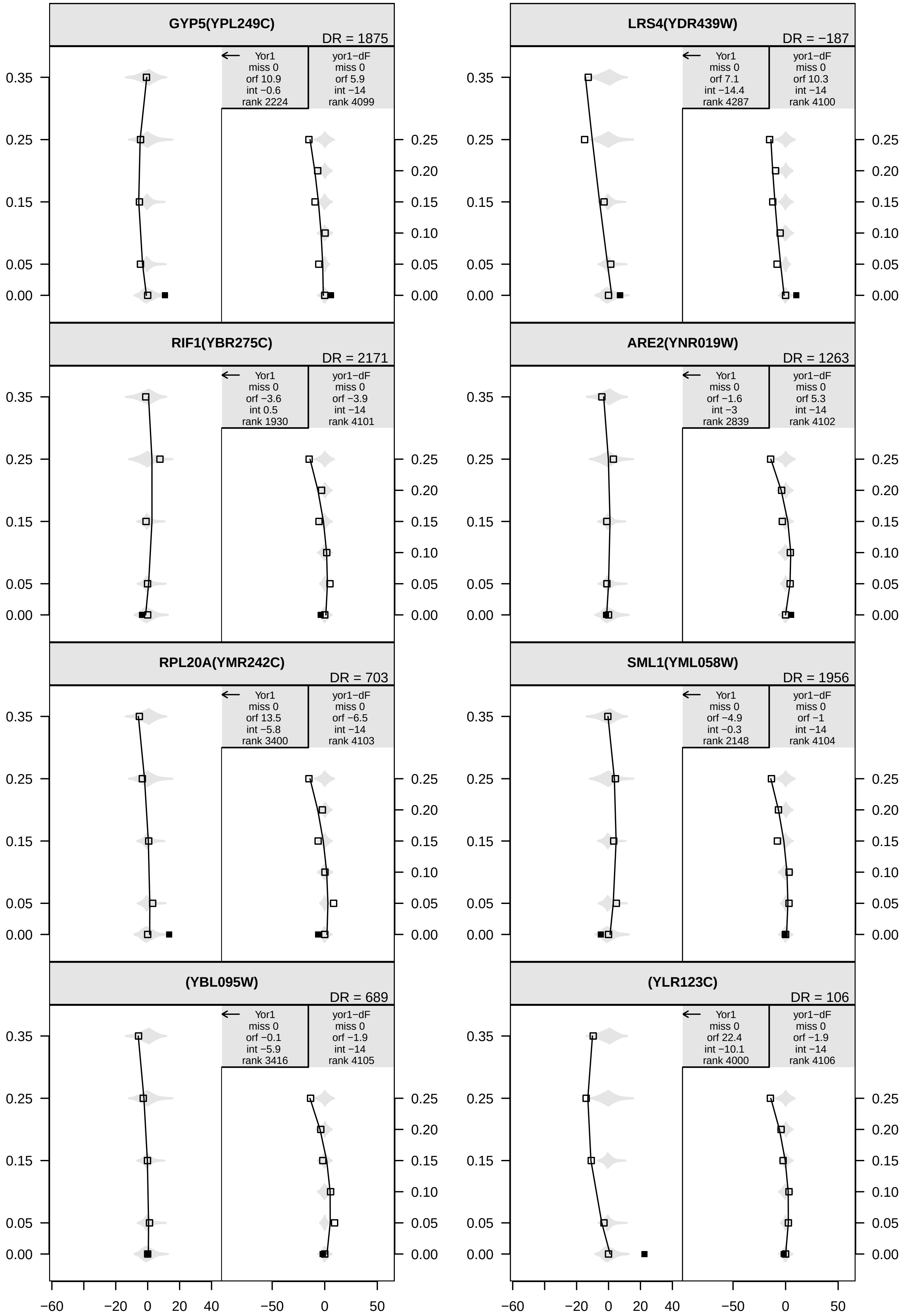

GYP5(YPL249C)
DR = 1875
Yor1
miss 0
orf 10.9
int −0.6
rank 2224
yor1−dF
miss 0
orf 5.9
int −14
rank 4099
LRS4(YDR439W)
DR = −187
Yor1
miss 0
orf 7.1
int −14.4
rank 4287
yor1−dF
miss 0
orf 10.3
int −14
rank 4100
RIF1(YBR275C)
DR = 2171
Yor1
miss 0
orf −3.6
int 0.5
rank 1930
yor1−dF
miss 0
orf −3.9
int −14
rank 4101
ARE2(YNR019W)
DR = 1263
Yor1
miss 0
orf −1.6
int −3
rank 2839
yor1−dF
miss 0
orf 5.3
int −14
rank 4102
RPL20A(YMR242C)
DR = 703
Yor1
miss 0
orf 13.5
int −5.8
rank 3400
yor1−dF
miss 0
orf −6.5
int −14
rank 4103
SML1(YML058W)
DR = 1956
Yor1
miss 0
orf −4.9
int −0.3
rank 2148
yor1−dF
miss 0
orf −1
int −14
rank 4104
(YBL095W)
DR = 689
Yor1
miss 0
orf −0.1
int −5.9
rank 3416
yor1−dF
miss 0
orf −1.9
int −14
rank 4105
(YLR123C)
DR = 106
Yor1
miss 0
orf 22.4
int −10.1
rank 4000
yor1−dF
miss 0
orf −1.9
int −14
rank 4106

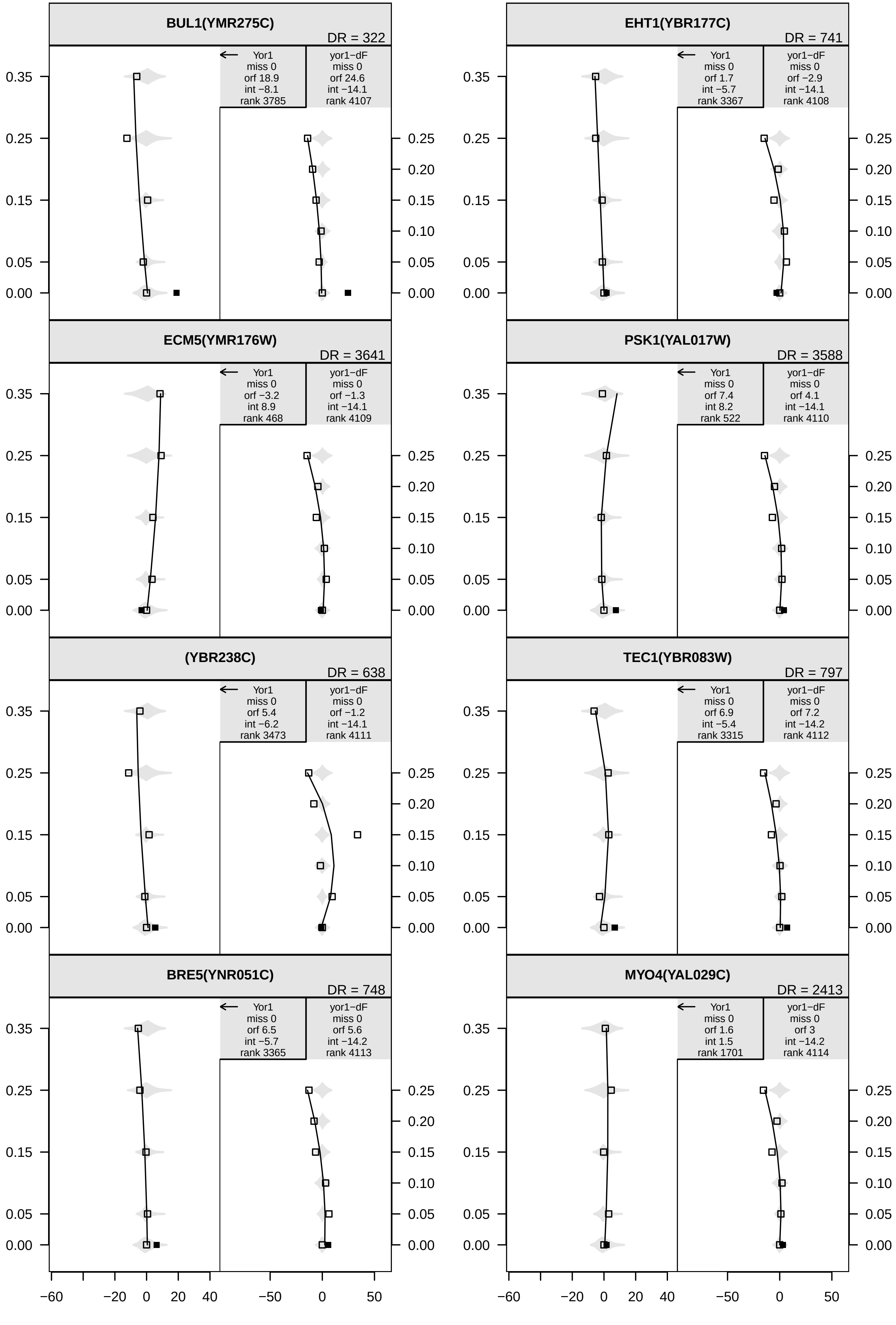
BUL1(YMR275C)
DR = 322
Yor1
miss 0
orf 18.9
int −8.1
rank 3785
yor1−dF
miss 0
orf 24.6
int −14.1
rank 4107
EHT1(YBR177C)
DR = 741
Yor1
miss 0
orf 1.7
int −5.7
rank 3367
yor1−dF
miss 0
orf −2.9
int −14.1
rank 4108
ECM5(YMR176W)
DR = 3641
Yor1
miss 0
orf −3.2
int 8.9
rank 468
yor1−dF
miss 0
orf −1.3
int −14.1
rank 4109
PSK1(YAL017W)
DR = 3588
Yor1
miss 0
orf 7.4
int 8.2
rank 522
yor1−dF
miss 0
orf 4.1
int −14.1
rank 4110
(YBR238C)
DR = 638
Yor1
miss 0
orf 5.4
int −6.2
rank 3473
yor1−dF
miss 0
orf −1.2
int −14.1
rank 4111
TEC1(YBR083W)
DR = 797
Yor1
miss 0
orf 6.9
int −5.4
rank 3315
yor1−dF
miss 0
orf 7.2
int −14.2
rank 4112
BRE5(YNR051C)
DR = 748
Yor1
miss 0
orf 6.5
int −5.7
rank 3365
yor1−dF
miss 0
orf 5.6
int −14.2
rank 4113
MYO4(YAL029C)
DR = 2413
Yor1
miss 0
orf 1.6
int 1.5
rank 1701
yor1−dF
miss 0
orf 3
int −14.2
rank 4114
0.35
0.25
0.20
0.15
0.10
0.05
0.00
−60
−20
0
20
40
−50
0
50

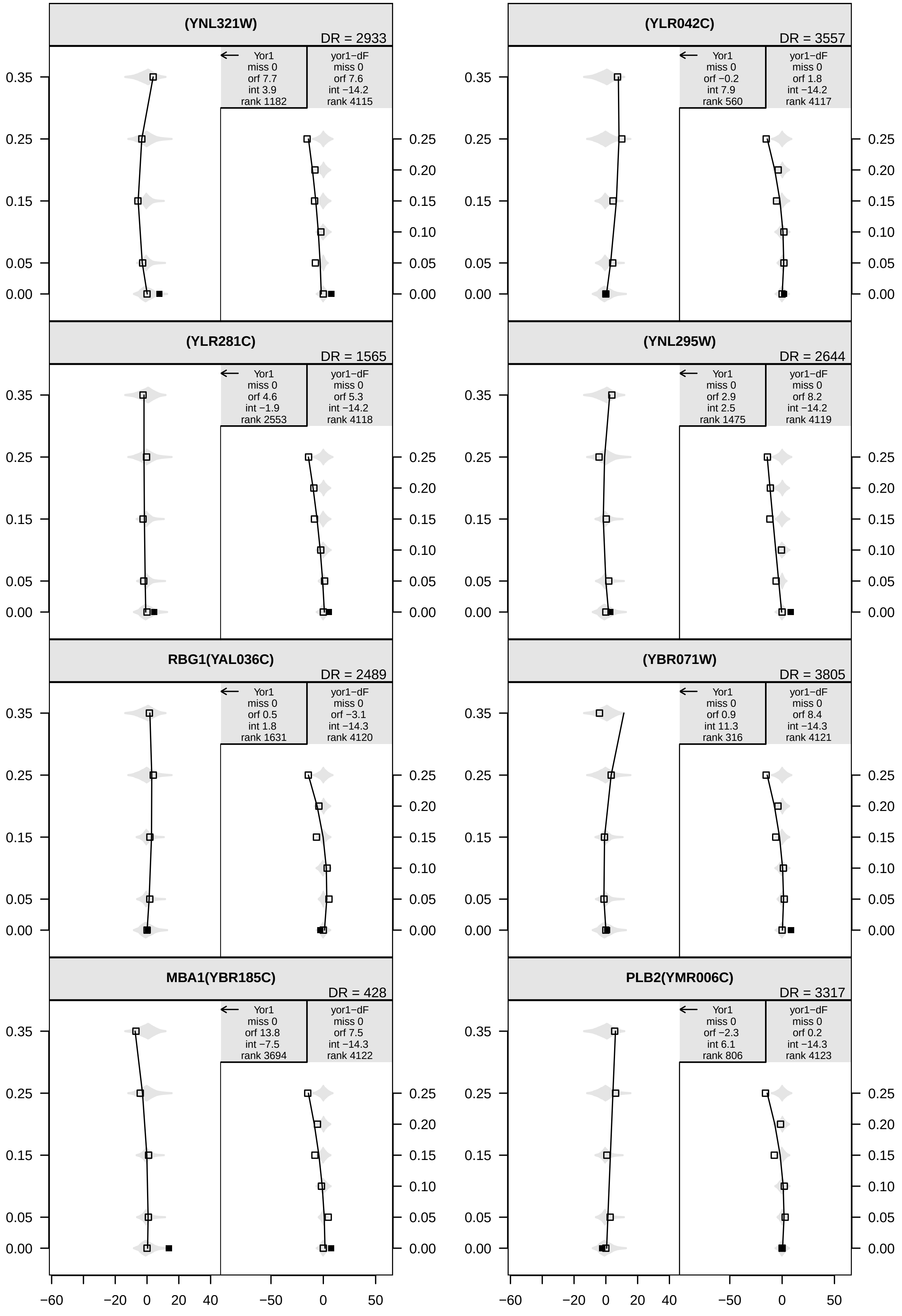

(YNL321W)
DR = 2933
Yor1 miss 0 orf 7.7 int 3.9 rank 1182
yor1−dF miss 0 orf 7.6 int −14.2 rank 4115
(YLR042C)
DR = 3557
Yor1 miss 0 orf −0.2 int 7.9 rank 560
yor1−dF miss 0 orf 1.8 int −14.2 rank 4117
(YLR281C)
DR = 1565
Yor1 miss 0 orf 4.6 int −1.9 rank 2553
yor1−dF miss 0 orf 5.3 int −14.2 rank 4118
(YNL295W)
DR = 2644
Yor1 miss 0 orf 2.9 int 2.5 rank 1475
yor1−dF miss 0 orf 8.2 int −14.2 rank 4119
RBG1(YAL036C)
DR = 2489
Yor1 miss 0 orf 0.5 int 1.8 rank 1631
yor1−dF miss 0 orf −3.1 int −14.3 rank 4120
(YBR071W)
DR = 3805
Yor1 miss 0 orf 0.9 int 11.3 rank 316
yor1−dF miss 0 orf 8.4 int −14.3 rank 4121
MBA1(YBR185C)
DR = 428
Yor1 miss 0 orf 13.8 int −7.5 rank 3694
yor1−dF miss 0 orf 7.5 int −14.3 rank 4122
PLB2(YMR006C)
DR = 3317
Yor1 miss 0 orf −2.3 int 6.1 rank 806
yor1−dF miss 0 orf 0.2 int −14.3 rank 4123
0.35 0.25 0.20 0.15 0.10 0.05 0.00
−60 −20 0 20 40 −50 0 50

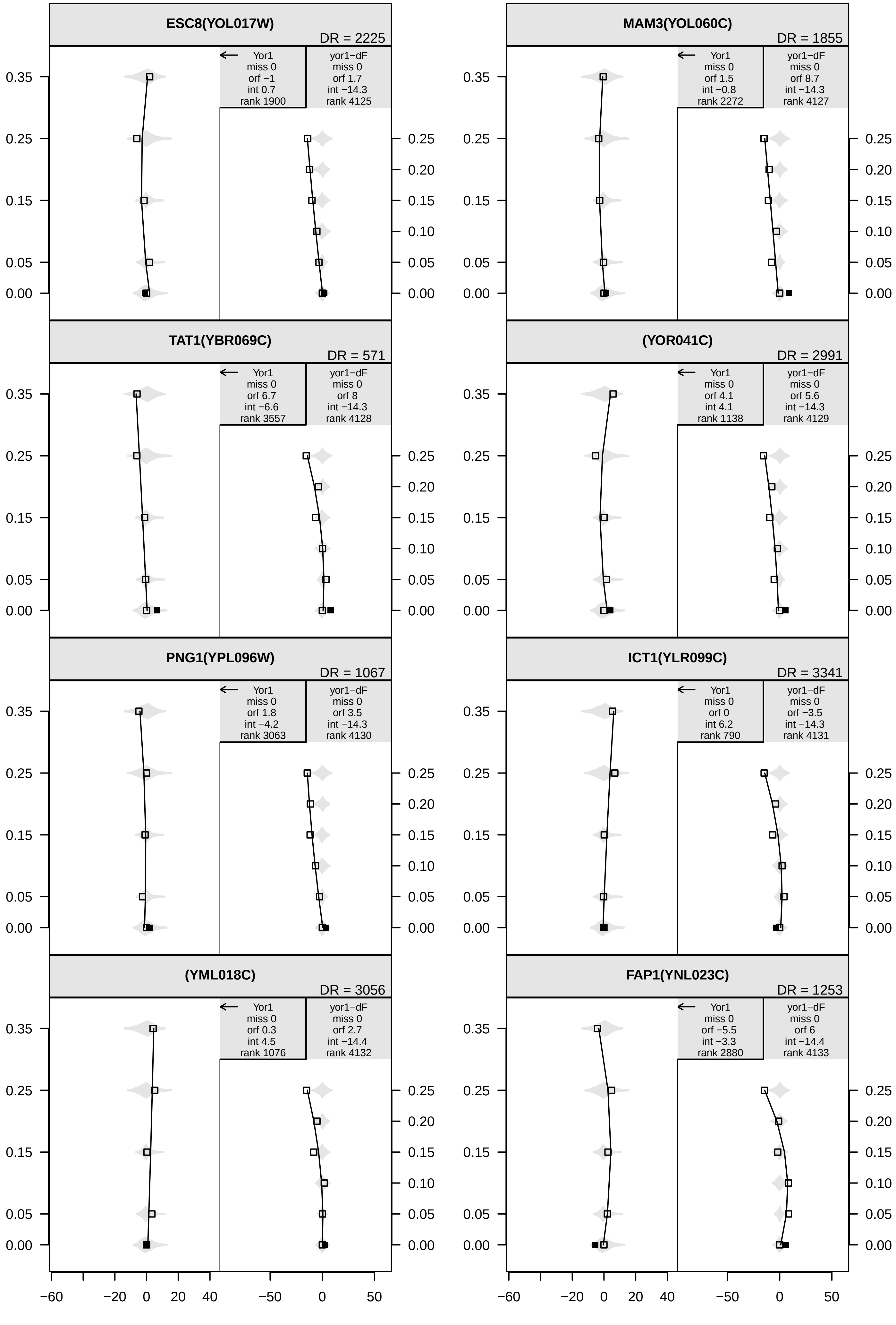

ESC8(YOL017W)
DR = 2225
Yor1 miss 0 orf −1 int 0.7 rank 1900
yor1−dF miss 0 orf 1.7 int −14.3 rank 4125
MAM3(YOL060C)
DR = 1855
Yor1 miss 0 orf 1.5 int −0.8 rank 2272
yor1−dF miss 0 orf 8.7 int −14.3 rank 4127
TAT1(YBR069C)
DR = 571
Yor1 miss 0 orf 6.7 int −6.6 rank 3557
yor1−dF miss 0 orf 8 int −14.3 rank 4128
(YOR041C)
DR = 2991
Yor1 miss 0 orf 4.1 int 4.1 rank 1138
yor1−dF miss 0 orf 5.6 int −14.3 rank 4129
PNG1(YPL096W)
DR = 1067
Yor1 miss 0 orf 1.8 int −4.2 rank 3063
yor1−dF miss 0 orf 3.5 int −14.3 rank 4130
ICT1(YLR099C)
DR = 3341
Yor1 miss 0 orf 0 int 6.2 rank 790
yor1−dF miss 0 orf −3.5 int −14.3 rank 4131
(YML018C)
DR = 3056
Yor1 miss 0 orf 0.3 int 4.5 rank 1076
yor1−dF miss 0 orf 2.7 int −14.4 rank 4132
FAP1(YNL023C)
DR = 1253
Yor1 miss 0 orf −5.5 int −3.3 rank 2880
yor1−dF miss 0 orf 6 int −14.4 rank 4133

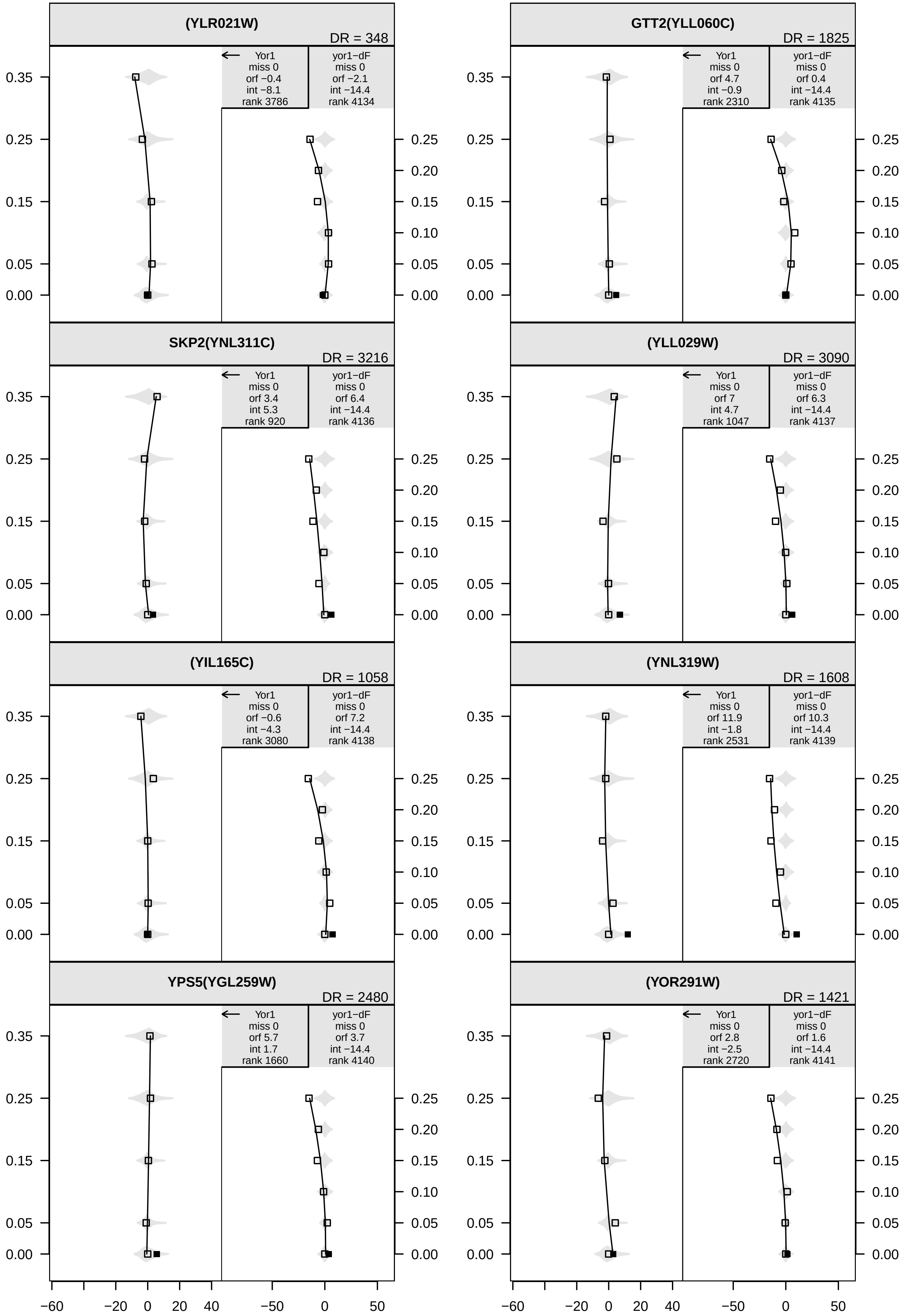

(YLR021W)
DR = 348
Yor1
miss 0
orf −0.4
int −8.1
rank 3786
yor1−dF
miss 0
orf −2.1
int −14.4
rank 4134
GTT2(YLL060C)
DR = 1825
Yor1
miss 0
orf 4.7
int −0.9
rank 2310
yor1−dF
miss 0
orf 0.4
int −14.4
rank 4135
SKP2(YNL311C)
DR = 3216
Yor1
miss 0
orf 3.4
int 5.3
rank 920
yor1−dF
miss 0
orf 6.4
int −14.4
rank 4136
(YLL029W)
DR = 3090
Yor1
miss 0
orf 7
int 4.7
rank 1047
yor1−dF
miss 0
orf 6.3
int −14.4
rank 4137
(YIL165C)
DR = 1058
Yor1
miss 0
orf −0.6
int −4.3
rank 3080
yor1−dF
miss 0
orf 7.2
int −14.4
rank 4138
(YNL319W)
DR = 1608
Yor1
miss 0
orf 11.9
int −1.8
rank 2531
yor1−dF
miss 0
orf 10.3
int −14.4
rank 4139
YPS5(YGL259W)
DR = 2480
Yor1
miss 0
orf 5.7
int 1.7
rank 1660
yor1−dF
miss 0
orf 3.7
int −14.4
rank 4140
(YOR291W)
DR = 1421
Yor1
miss 0
orf 2.8
int −2.5
rank 2720
yor1−dF
miss 0
orf 1.6
int −14.4
rank 4141
0.35
0.25
0.20
0.15
0.10
0.05
0.00
−60
−20
0
20
40
−50
0
50

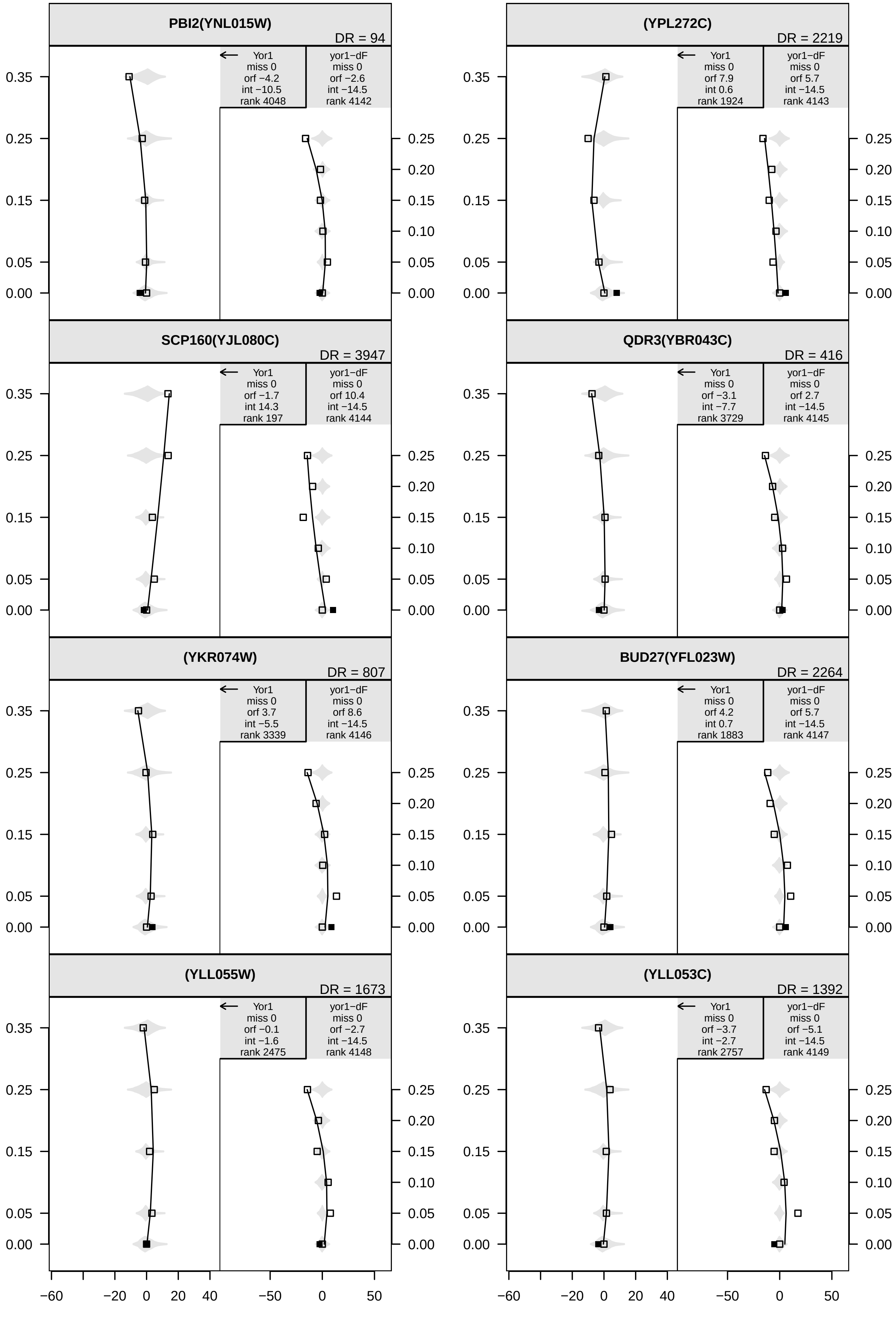

PBI2(YNL015W)
DR = 94
Yor1 miss 0 orf −4.2 int −10.5 rank 4048
yor1−dF miss 0 orf −2.6 int −14.5 rank 4142
(YPL272C)
DR = 2219
Yor1 miss 0 orf 7.9 int 0.6 rank 1924
yor1−dF miss 0 orf 5.7 int −14.5 rank 4143
SCP160(YJL080C)
DR = 3947
Yor1 miss 0 orf −1.7 int 14.3 rank 197
yor1−dF miss 0 orf 10.4 int −14.5 rank 4144
QDR3(YBR043C)
DR = 416
Yor1 miss 0 orf −3.1 int −7.7 rank 3729
yor1−dF miss 0 orf 2.7 int −14.5 rank 4145
(YKR074W)
DR = 807
Yor1 miss 0 orf 3.7 int −5.5 rank 3339
yor1−dF miss 0 orf 8.6 int −14.5 rank 4146
BUD27(YFL023W)
DR = 2264
Yor1 miss 0 orf 4.2 int 0.7 rank 1883
yor1−dF miss 0 orf 5.7 int −14.5 rank 4147
(YLL055W)
DR = 1673
Yor1 miss 0 orf −0.1 int −1.6 rank 2475
yor1−dF miss 0 orf −2.7 int −14.5 rank 4148
(YLL053C)
DR = 1392
Yor1 miss 0 orf −3.7 int −2.7 rank 2757
yor1−dF miss 0 orf −5.1 int −14.5 rank 4149

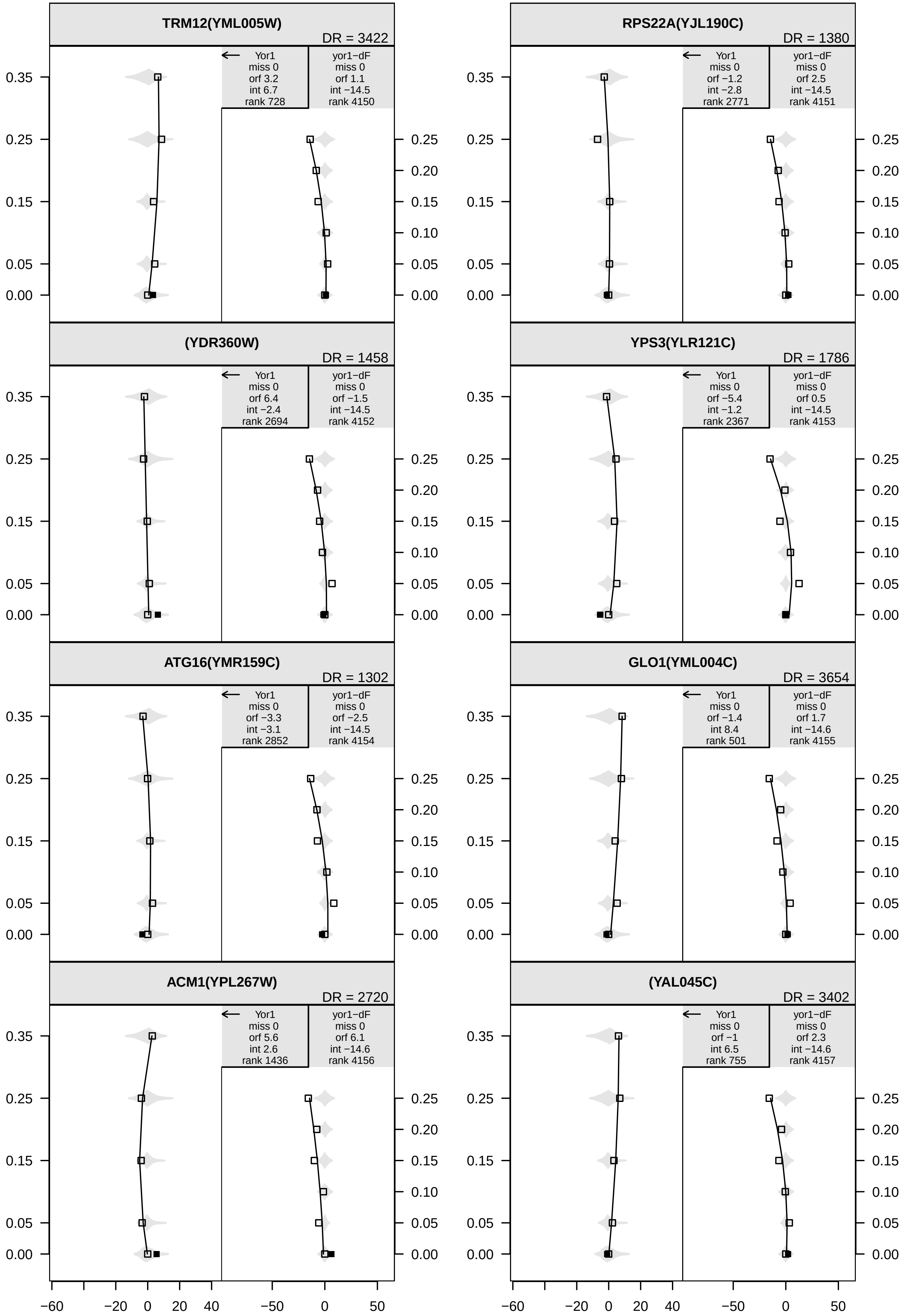
TRM12(YML005W)
DR = 3422
Yor1 miss 0 orf 3.2 int 6.7 rank 728
yor1−dF miss 0 orf 1.1 int −14.5 rank 4150
RPS22A(YJL190C)
DR = 1380
Yor1 miss 0 orf −1.2 int −2.8 rank 2771
yor1−dF miss 0 orf 2.5 int −14.5 rank 4151
(YDR360W)
DR = 1458
Yor1 miss 0 orf 6.4 int −2.4 rank 2694
yor1−dF miss 0 orf −1.5 int −14.5 rank 4152
YPS3(YLR121C)
DR = 1786
Yor1 miss 0 orf −5.4 int −1.2 rank 2367
yor1−dF miss 0 orf 0.5 int −14.5 rank 4153
ATG16(YMR159C)
DR = 1302
Yor1 miss 0 orf −3.3 int −3.1 rank 2852
yor1−dF miss 0 orf −2.5 int −14.5 rank 4154
GLO1(YML004C)
DR = 3654
Yor1 miss 0 orf −1.4 int 8.4 rank 501
yor1−dF miss 0 orf 1.7 int −14.6 rank 4155
ACM1(YPL267W)
DR = 2720
Yor1 miss 0 orf 5.6 int 2.6 rank 1436
yor1−dF miss 0 orf 6.1 int −14.6 rank 4156
(YAL045C)
DR = 3402
Yor1 miss 0 orf −1 int 6.5 rank 755
yor1−dF miss 0 orf 2.3 int −14.6 rank 4157
0.35
0.25
0.20
0.15
0.10
0.05
0.00
−60
−20
0
20
40
−50
0
50

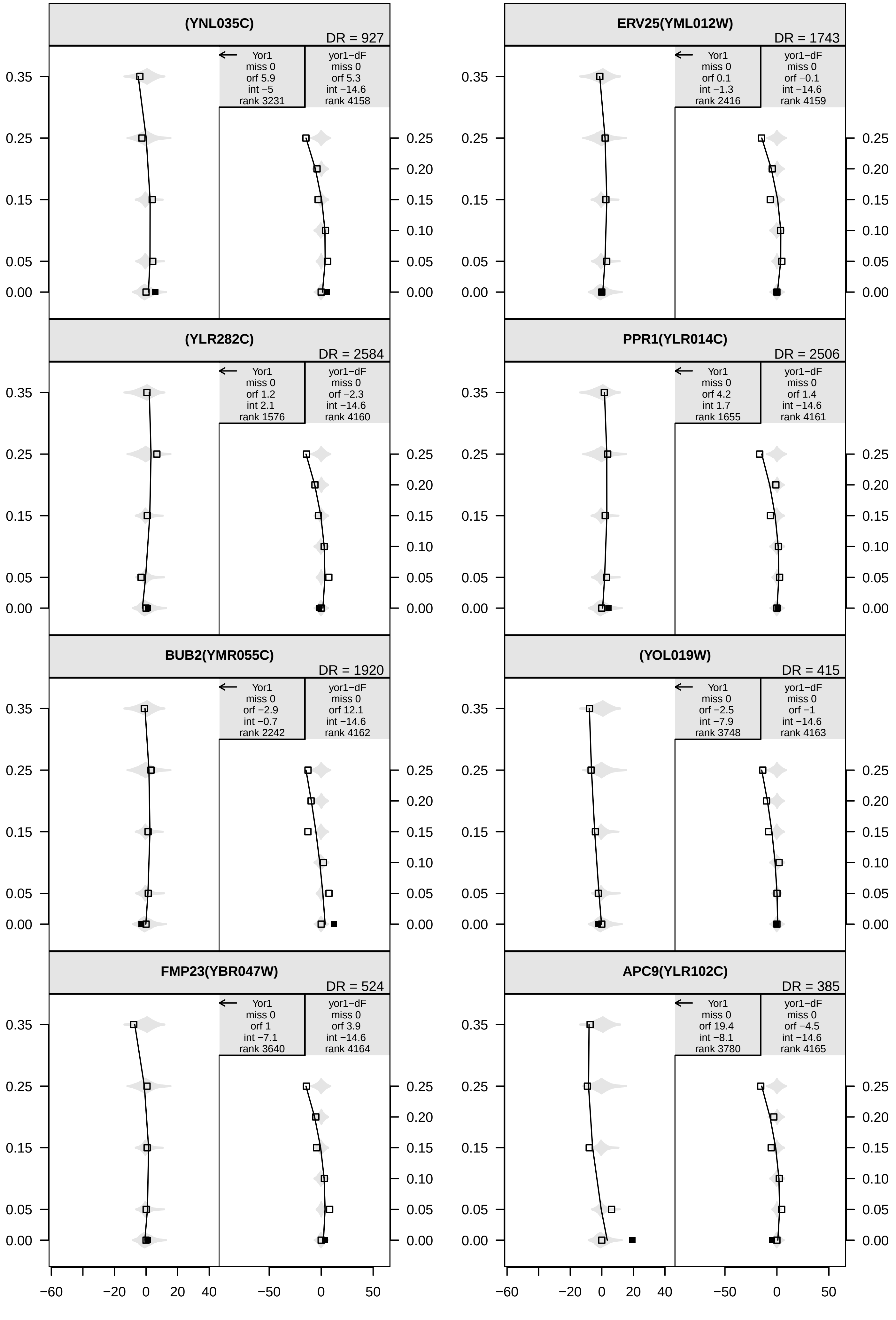
(YNL035C)
DR = 927
Yor1 miss 0 orf 5.9 int −5 rank 3231
yor1−dF miss 0 orf 5.3 int −14.6 rank 4158
ERV25(YML012W)
DR = 1743
Yor1 miss 0 orf 0.1 int −1.3 rank 2416
yor1−dF miss 0 orf −0.1 int −14.6 rank 4159
(YLR282C)
DR = 2584
Yor1 miss 0 orf 1.2 int 2.1 rank 1576
yor1−dF miss 0 orf −2.3 int −14.6 rank 4160
PPR1(YLR014C)
DR = 2506
Yor1 miss 0 orf 4.2 int 1.7 rank 1655
yor1−dF miss 0 orf 1.4 int −14.6 rank 4161
BUB2(YMR055C)
DR = 1920
Yor1 miss 0 orf −2.9 int −0.7 rank 2242
yor1−dF miss 0 orf 12.1 int −14.6 rank 4162
(YOL019W)
DR = 415
Yor1 miss 0 orf −2.5 int −7.9 rank 3748
yor1−dF miss 0 orf −1 int −14.6 rank 4163
FMP23(YBR047W)
DR = 524
Yor1 miss 0 orf 1 int −7.1 rank 3640
yor1−dF miss 0 orf 3.9 int −14.6 rank 4164
APC9(YLR102C)
DR = 385
Yor1 miss 0 orf 19.4 int −8.1 rank 3780
yor1−dF miss 0 orf −4.5 int −14.6 rank 4165
0.35
0.25
0.20
0.15
0.10
0.05
0.00
−60 −20 0 20 40
−50 0 50

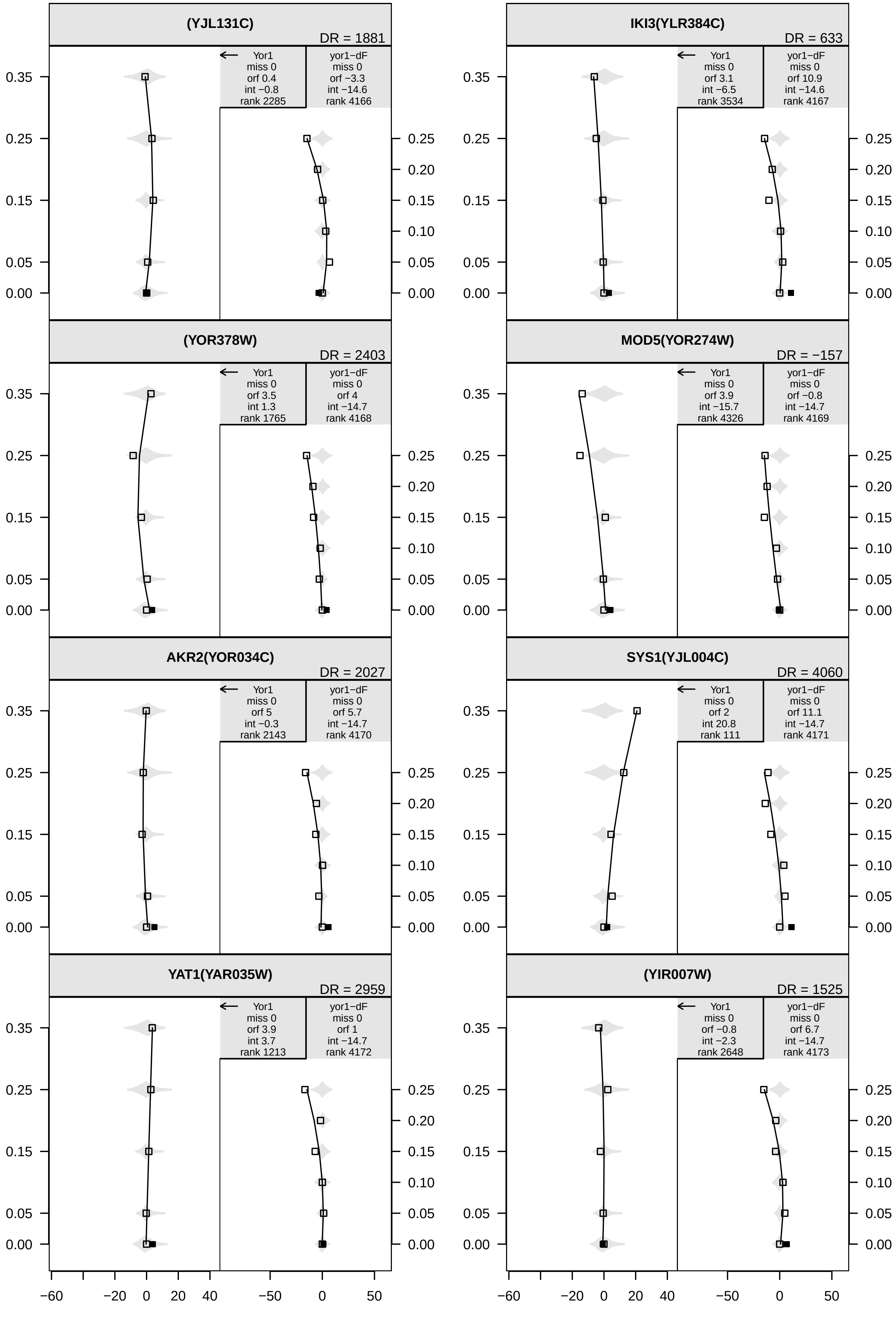
(YJL131C)
DR = 1881
Yor1
miss 0
orf 0.4
int −0.8
rank 2285
yor1−dF
miss 0
orf −3.3
int −14.6
rank 4166
IKI3(YLR384C)
DR = 633
Yor1
miss 0
orf 3.1
int −6.5
rank 3534
yor1−dF
miss 0
orf 10.9
int −14.6
rank 4167
(YOR378W)
DR = 2403
Yor1
miss 0
orf 3.5
int 1.3
rank 1765
yor1−dF
miss 0
orf 4
int −14.7
rank 4168
MOD5(YOR274W)
DR = −157
Yor1
miss 0
orf 3.9
int −15.7
rank 4326
yor1−dF
miss 0
orf −0.8
int −14.7
rank 4169
AKR2(YOR034C)
DR = 2027
Yor1
miss 0
orf 5
int −0.3
rank 2143
yor1−dF
miss 0
orf 5.7
int −14.7
rank 4170
SYS1(YJL004C)
DR = 4060
Yor1
miss 0
orf 2
int 20.8
rank 111
yor1−dF
miss 0
orf 11.1
int −14.7
rank 4171
YAT1(YAR035W)
DR = 2959
Yor1
miss 0
orf 3.9
int 3.7
rank 1213
yor1−dF
miss 0
orf 1
int −14.7
rank 4172
(YIR007W)
DR = 1525
Yor1
miss 0
orf −0.8
int −2.3
rank 2648
yor1−dF
miss 0
orf 6.7
int −14.7
rank 4173
0.35
0.25
0.20
0.15
0.10
0.05
0.00
−60
−20
0
20
40
−50
0
50

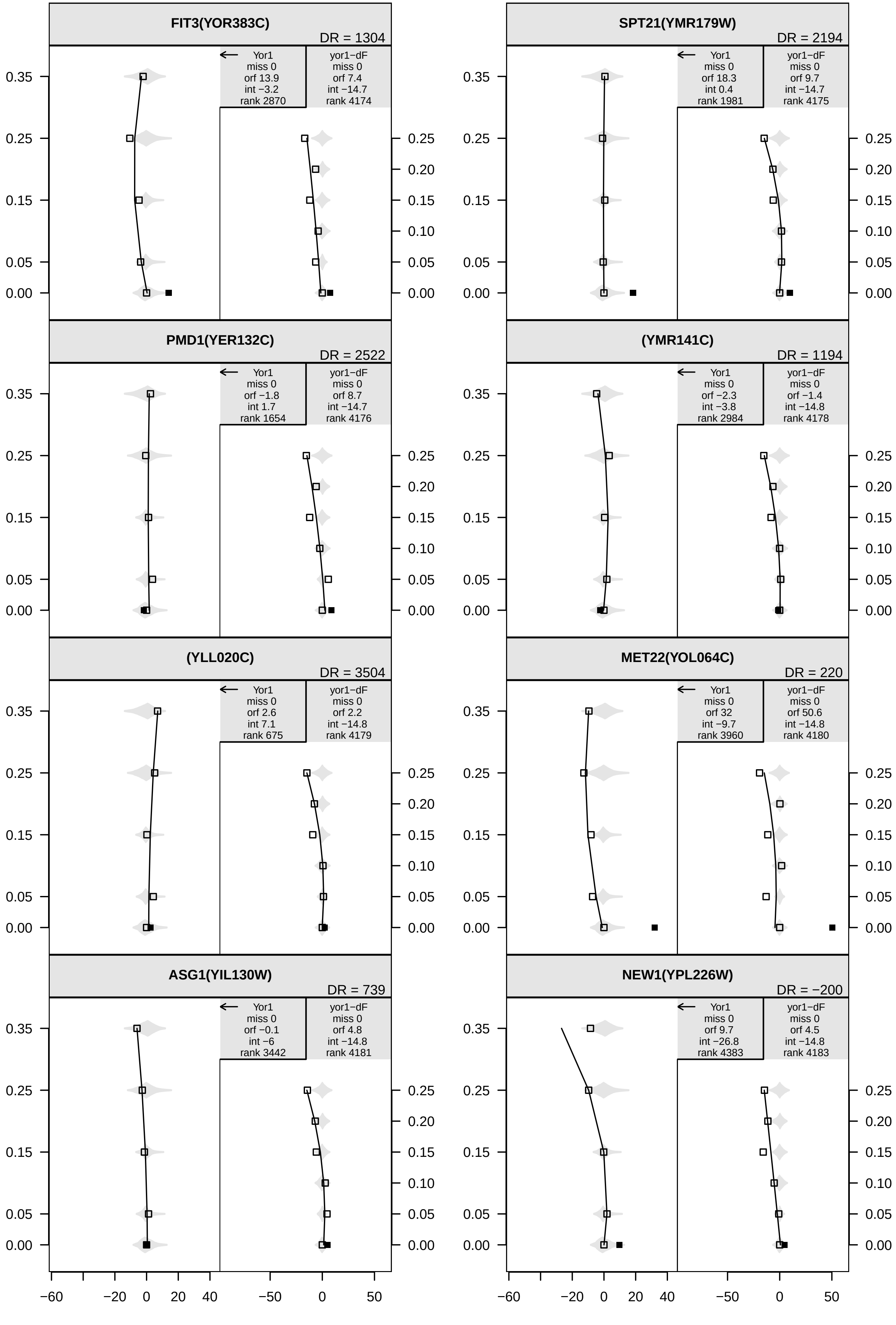
FIT3(YOR383C)
DR = 1304
Yor1 miss 0 orf 13.9 int −3.2 rank 2870
yor1−dF miss 0 orf 7.4 int −14.7 rank 4174
SPT21(YMR179W)
DR = 2194
Yor1 miss 0 orf 18.3 int 0.4 rank 1981
yor1−dF miss 0 orf 9.7 int −14.7 rank 4175
PMD1(YER132C)
DR = 2522
Yor1 miss 0 orf −1.8 int 1.7 rank 1654
yor1−dF miss 0 orf 8.7 int −14.7 rank 4176
(YMR141C)
DR = 1194
Yor1 miss 0 orf −2.3 int −3.8 rank 2984
yor1−dF miss 0 orf −1.4 int −14.8 rank 4178
(YLL020C)
DR = 3504
Yor1 miss 0 orf 2.6 int 7.1 rank 675
yor1−dF miss 0 orf 2.2 int −14.8 rank 4179
MET22(YOL064C)
DR = 220
Yor1 miss 0 orf 32 int −9.7 rank 3960
yor1−dF miss 0 orf 50.6 int −14.8 rank 4180
ASG1(YIL130W)
DR = 739
Yor1 miss 0 orf −0.1 int −6 rank 3442
yor1−dF miss 0 orf 4.8 int −14.8 rank 4181
NEW1(YPL226W)
DR = −200
Yor1 miss 0 orf 9.7 int −26.8 rank 4383
yor1−dF miss 0 orf 4.5 int −14.8 rank 4183
0.35 0.25 0.20 0.15 0.10 0.05 0.00
−60 −20 0 20 40 −50 0 50

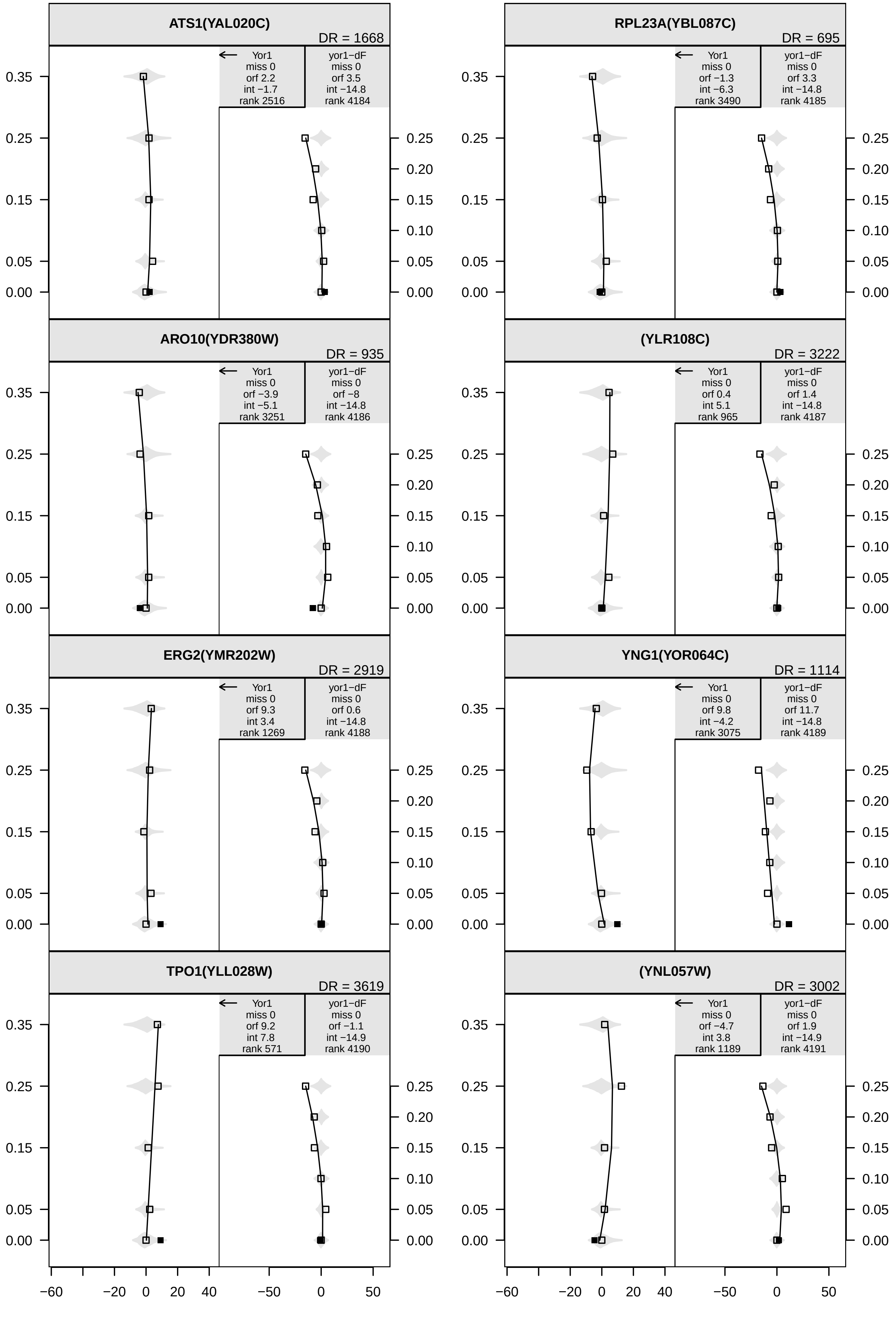

ATS1(YAL020C)
DR = 1668
Yor1 miss 0 orf 2.2 int −1.7 rank 2516
yor1−dF miss 0 orf 3.5 int −14.8 rank 4184
RPL23A(YBL087C)
DR = 695
Yor1 miss 0 orf −1.3 int −6.3 rank 3490
yor1−dF miss 0 orf 3.3 int −14.8 rank 4185
ARO10(YDR380W)
DR = 935
Yor1 miss 0 orf −3.9 int −5.1 rank 3251
yor1−dF miss 0 orf −8 int −14.8 rank 4186
(YLR108C)
DR = 3222
Yor1 miss 0 orf 0.4 int 5.1 rank 965
yor1−dF miss 0 orf 1.4 int −14.8 rank 4187
ERG2(YMR202W)
DR = 2919
Yor1 miss 0 orf 9.3 int 3.4 rank 1269
yor1−dF miss 0 orf 0.6 int −14.8 rank 4188
YNG1(YOR064C)
DR = 1114
Yor1 miss 0 orf 9.8 int −4.2 rank 3075
yor1−dF miss 0 orf 11.7 int −14.8 rank 4189
TPO1(YLL028W)
DR = 3619
Yor1 miss 0 orf 9.2 int 7.8 rank 571
yor1−dF miss 0 orf −1.1 int −14.9 rank 4190
(YNL057W)
DR = 3002
Yor1 miss 0 orf −4.7 int 3.8 rank 1189
yor1−dF miss 0 orf 1.9 int −14.9 rank 4191
0.35 0.25 0.15 0.05 0.00
0.25 0.20 0.15 0.10 0.05 0.00
−60 −20 0 20 40
−50 0 50

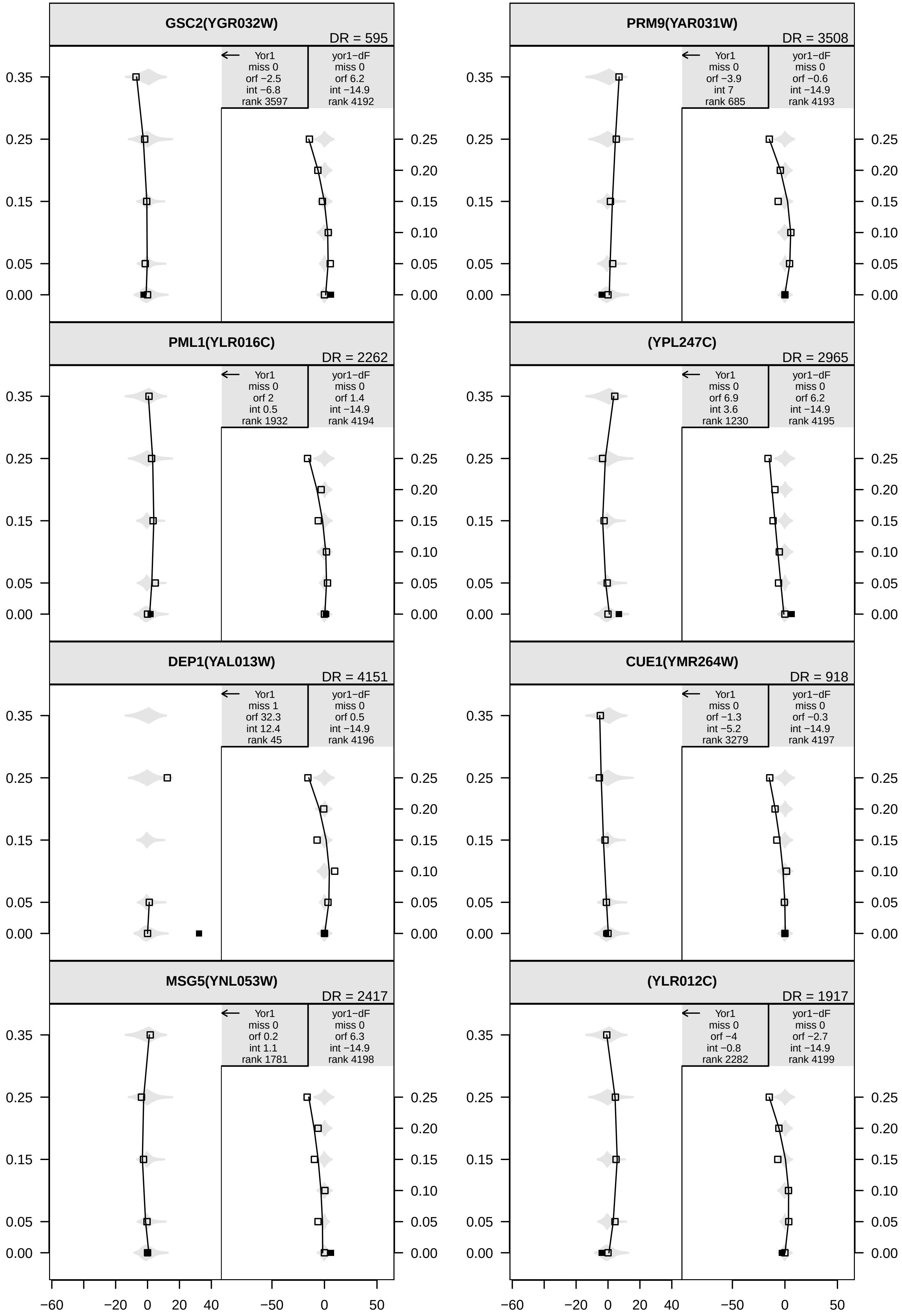
GSC2(YGR032W)
DR = 595
Yor1 miss 0 orf −2.5 int −6.8 rank 3597
yor1−dF miss 0 orf 6.2 int −14.9 rank 4192
PRM9(YAR031W)
DR = 3508
Yor1 miss 0 orf −3.9 int 7 rank 685
yor1−dF miss 0 orf −0.6 int −14.9 rank 4193
PML1(YLR016C)
DR = 2262
Yor1 miss 0 orf 2 int 0.5 rank 1932
yor1−dF miss 0 orf 1.4 int −14.9 rank 4194
(YPL247C)
DR = 2965
Yor1 miss 0 orf 6.9 int 3.6 rank 1230
yor1−dF miss 0 orf 6.2 int −14.9 rank 4195
DEP1(YAL013W)
DR = 4151
Yor1 miss 1 orf 32.3 int 12.4 rank 45
yor1−dF miss 0 orf 0.5 int −14.9 rank 4196
CUE1(YMR264W)
DR = 918
Yor1 miss 0 orf −1.3 int −5.2 rank 3279
yor1−dF miss 0 orf −0.3 int −14.9 rank 4197
MSG5(YNL053W)
DR = 2417
Yor1 miss 0 orf 0.2 int 1.1 rank 1781
yor1−dF miss 0 orf 6.3 int −14.9 rank 4198
(YLR012C)
DR = 1917
Yor1 miss 0 orf −4 int −0.8 rank 2282
yor1−dF miss 0 orf −2.7 int −14.9 rank 4199
0.35 0.25 0.20 0.15 0.10 0.05 0.00
−60 −20 0 20 40 −50 0 50

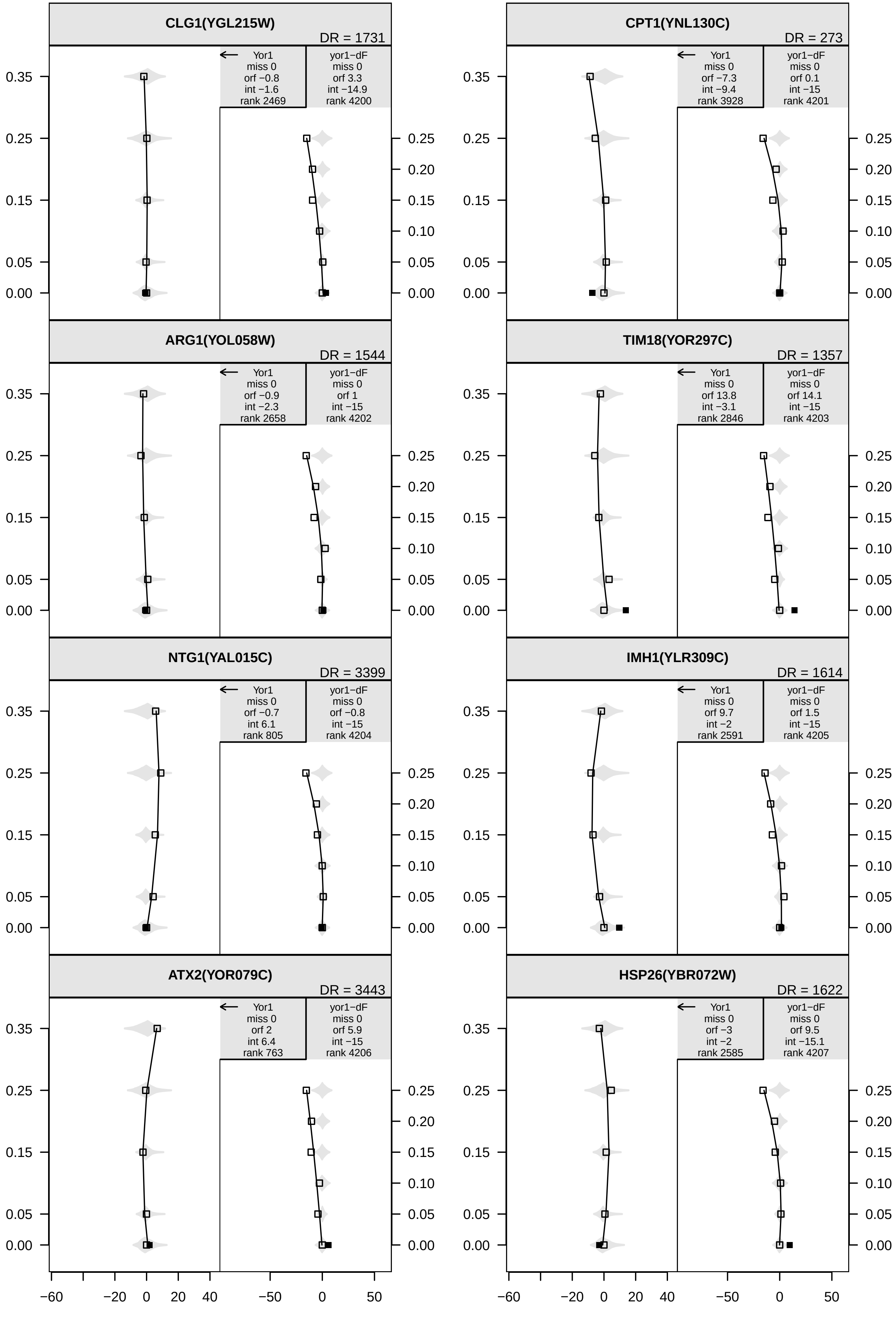
CLG1(YGL215W)
DR = 1731
Yor1 miss 0 orf −0.8 int −1.6 rank 2469
yor1−dF miss 0 orf 3.3 int −14.9 rank 4200
CPT1(YNL130C)
DR = 273
Yor1 miss 0 orf −7.3 int −9.4 rank 3928
yor1−dF miss 0 orf 0.1 int −15 rank 4201
ARG1(YOL058W)
DR = 1544
Yor1 miss 0 orf −0.9 int −2.3 rank 2658
yor1−dF miss 0 orf 1 int −15 rank 4202
TIM18(YOR297C)
DR = 1357
Yor1 miss 0 orf 13.8 int −3.1 rank 2846
yor1−dF miss 0 orf 14.1 int −15 rank 4203
NTG1(YAL015C)
DR = 3399
Yor1 miss 0 orf −0.7 int 6.1 rank 805
yor1−dF miss 0 orf −0.8 int −15 rank 4204
IMH1(YLR309C)
DR = 1614
Yor1 miss 0 orf 9.7 int −2 rank 2591
yor1−dF miss 0 orf 1.5 int −15 rank 4205
ATX2(YOR079C)
DR = 3443
Yor1 miss 0 orf 2 int 6.4 rank 763
yor1−dF miss 0 orf 5.9 int −15 rank 4206
HSP26(YBR072W)
DR = 1622
Yor1 miss 0 orf −3 int −2 rank 2585
yor1−dF miss 0 orf 9.5 int −15.1 rank 4207
0.35 0.25 0.20 0.15 0.10 0.05 0.00
−60 −20 0 20 40 −50 0 50

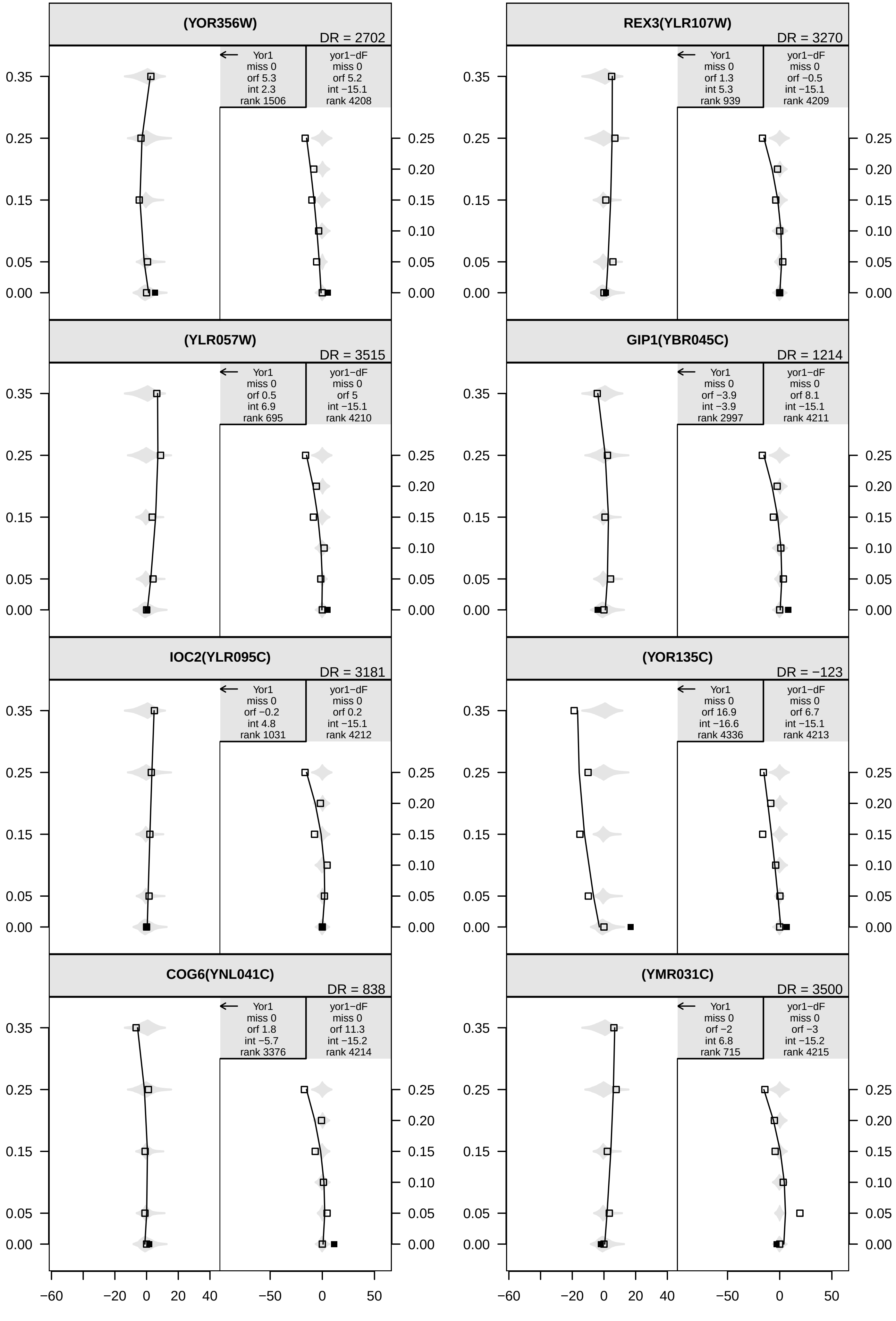

(YOR356W)
DR = 2702
Yor1
miss 0
orf 5.3
int 2.3
rank 1506
yor1−dF
miss 0
orf 5.2
int −15.1
rank 4208
REX3(YLR107W)
DR = 3270
Yor1
miss 0
orf 1.3
int 5.3
rank 939
yor1−dF
miss 0
orf −0.5
int −15.1
rank 4209
(YLR057W)
DR = 3515
Yor1
miss 0
orf 0.5
int 6.9
rank 695
yor1−dF
miss 0
orf 5
int −15.1
rank 4210
GIP1(YBR045C)
DR = 1214
Yor1
miss 0
orf −3.9
int −3.9
rank 2997
yor1−dF
miss 0
orf 8.1
int −15.1
rank 4211
IOC2(YLR095C)
DR = 3181
Yor1
miss 0
orf −0.2
int 4.8
rank 1031
yor1−dF
miss 0
orf 0.2
int −15.1
rank 4212
(YOR135C)
DR = −123
Yor1
miss 0
orf 16.9
int −16.6
rank 4336
yor1−dF
miss 0
orf 6.7
int −15.1
rank 4213
COG6(YNL041C)
DR = 838
Yor1
miss 0
orf 1.8
int −5.7
rank 3376
yor1−dF
miss 0
orf 11.3
int −15.2
rank 4214
(YMR031C)
DR = 3500
Yor1
miss 0
orf −2
int 6.8
rank 715
yor1−dF
miss 0
orf −3
int −15.2
rank 4215
0.00 0.05 0.10 0.15 0.20 0.25 0.35
−60 −20 0 20 40 −50 0 50

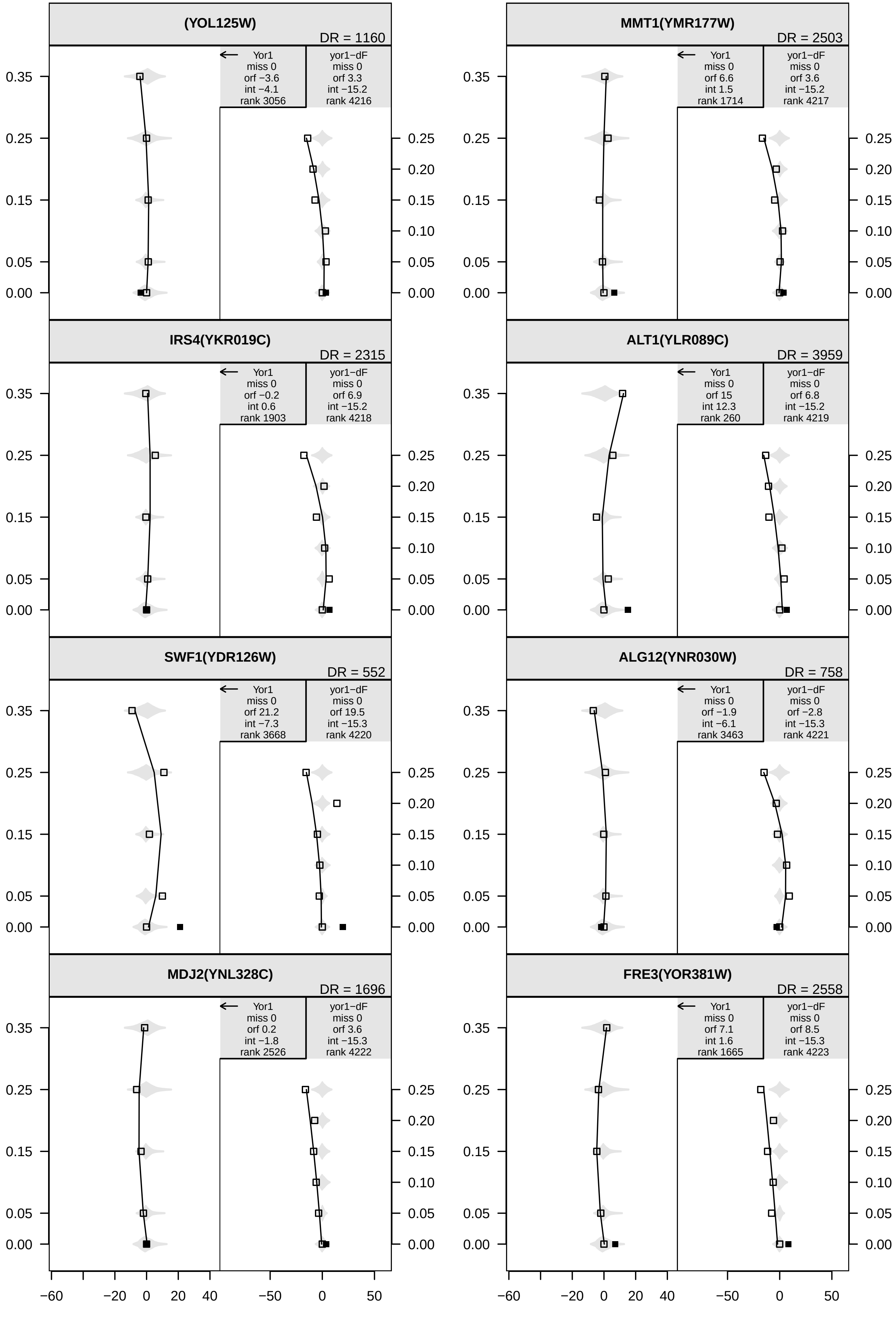

(YOL125W)
DR = 1160
Yor1
miss 0
orf −3.6
int −4.1
rank 3056
yor1−dF
miss 0
orf 3.3
int −15.2
rank 4216
MMT1(YMR177W)
DR = 2503
Yor1
miss 0
orf 6.6
int 1.5
rank 1714
yor1−dF
miss 0
orf 3.6
int −15.2
rank 4217
IRS4(YKR019C)
DR = 2315
Yor1
miss 0
orf −0.2
int 0.6
rank 1903
yor1−dF
miss 0
orf 6.9
int −15.2
rank 4218
ALT1(YLR089C)
DR = 3959
Yor1
miss 0
orf 15
int 12.3
rank 260
yor1−dF
miss 0
orf 6.8
int −15.2
rank 4219
SWF1(YDR126W)
DR = 552
Yor1
miss 0
orf 21.2
int −7.3
rank 3668
yor1−dF
miss 0
orf 19.5
int −15.3
rank 4220
ALG12(YNR030W)
DR = 758
Yor1
miss 0
orf −1.9
int −6.1
rank 3463
yor1−dF
miss 0
orf −2.8
int −15.3
rank 4221
MDJ2(YNL328C)
DR = 1696
Yor1
miss 0
orf 0.2
int −1.8
rank 2526
yor1−dF
miss 0
orf 3.6
int −15.3
rank 4222
FRE3(YOR381W)
DR = 2558
Yor1
miss 0
orf 7.1
int 1.6
rank 1665
yor1−dF
miss 0
orf 8.5
int −15.3
rank 4223
0.35
0.25
0.20
0.15
0.10
0.05
0.00
−60
−20
0
20
40
−50
0
50

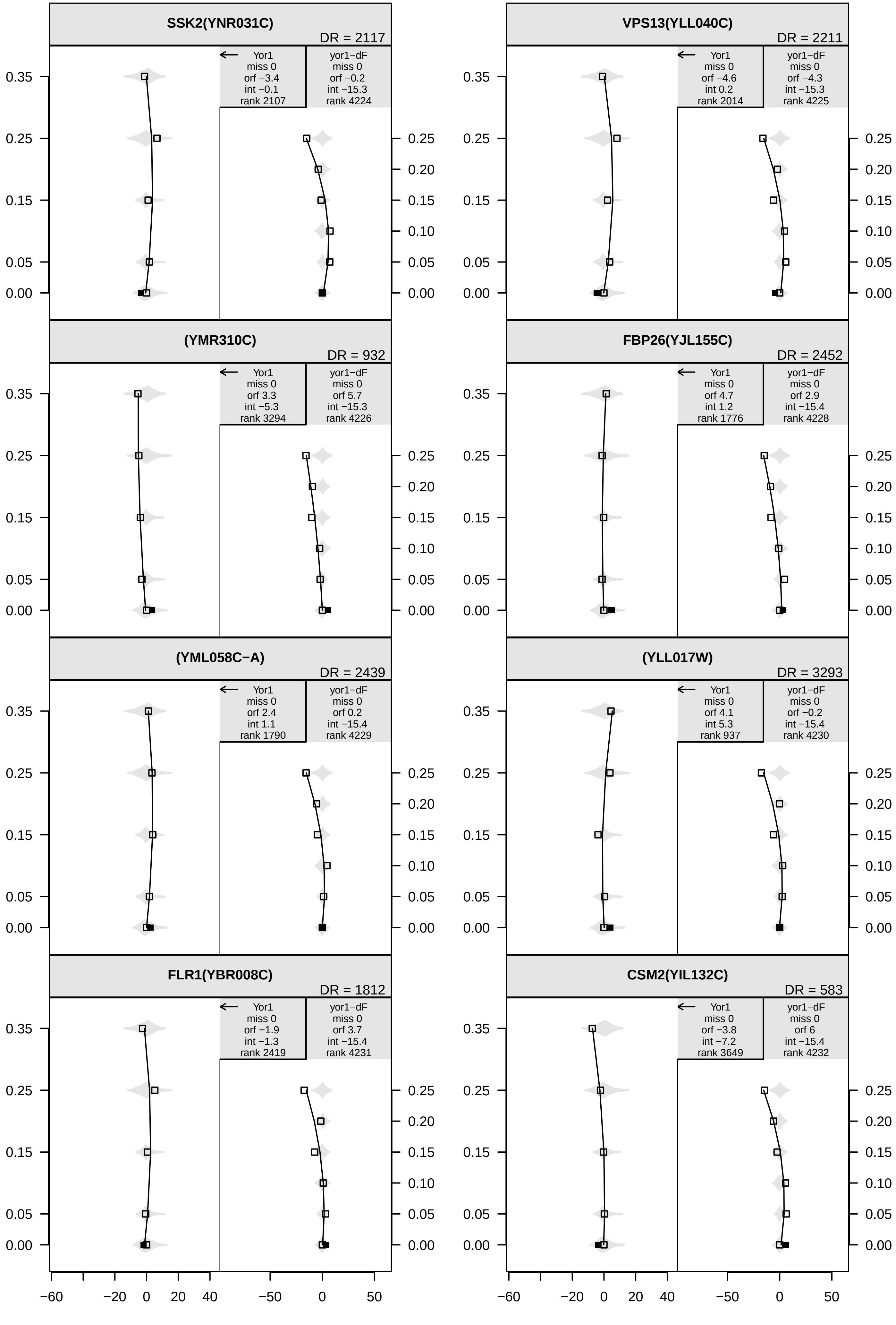

SSK2(YNR031C)
DR = 2117
Yor1
miss 0
orf −3.4
int −0.1
rank 2107
yor1−dF
miss 0
orf −0.2
int −15.3
rank 4224
VPS13(YLL040C)
DR = 2211
Yor1
miss 0
orf −4.6
int 0.2
rank 2014
yor1−dF
miss 0
orf −4.3
int −15.3
rank 4225
(YMR310C)
DR = 932
Yor1
miss 0
orf 3.3
int −5.3
rank 3294
yor1−dF
miss 0
orf 5.7
int −15.3
rank 4226
FBP26(YJL155C)
DR = 2452
Yor1
miss 0
orf 4.7
int 1.2
rank 1776
yor1−dF
miss 0
orf 2.9
int −15.4
rank 4228
(YML058C−A)
DR = 2439
Yor1
miss 0
orf 2.4
int 1.1
rank 1790
yor1−dF
miss 0
orf 0.2
int −15.4
rank 4229
(YLL017W)
DR = 3293
Yor1
miss 0
orf 4.1
int 5.3
rank 937
yor1−dF
miss 0
orf −0.2
int −15.4
rank 4230
FLR1(YBR008C)
DR = 1812
Yor1
miss 0
orf −1.9
int −1.3
rank 2419
yor1−dF
miss 0
orf 3.7
int −15.4
rank 4231
CSM2(YIL132C)
DR = 583
Yor1
miss 0
orf −3.8
int −7.2
rank 3649
yor1−dF
miss 0
orf 6
int −15.4
rank 4232
0.35
0.25
0.20
0.15
0.10
0.05
0.00
−60
−20
0
20
40
−50
0
50

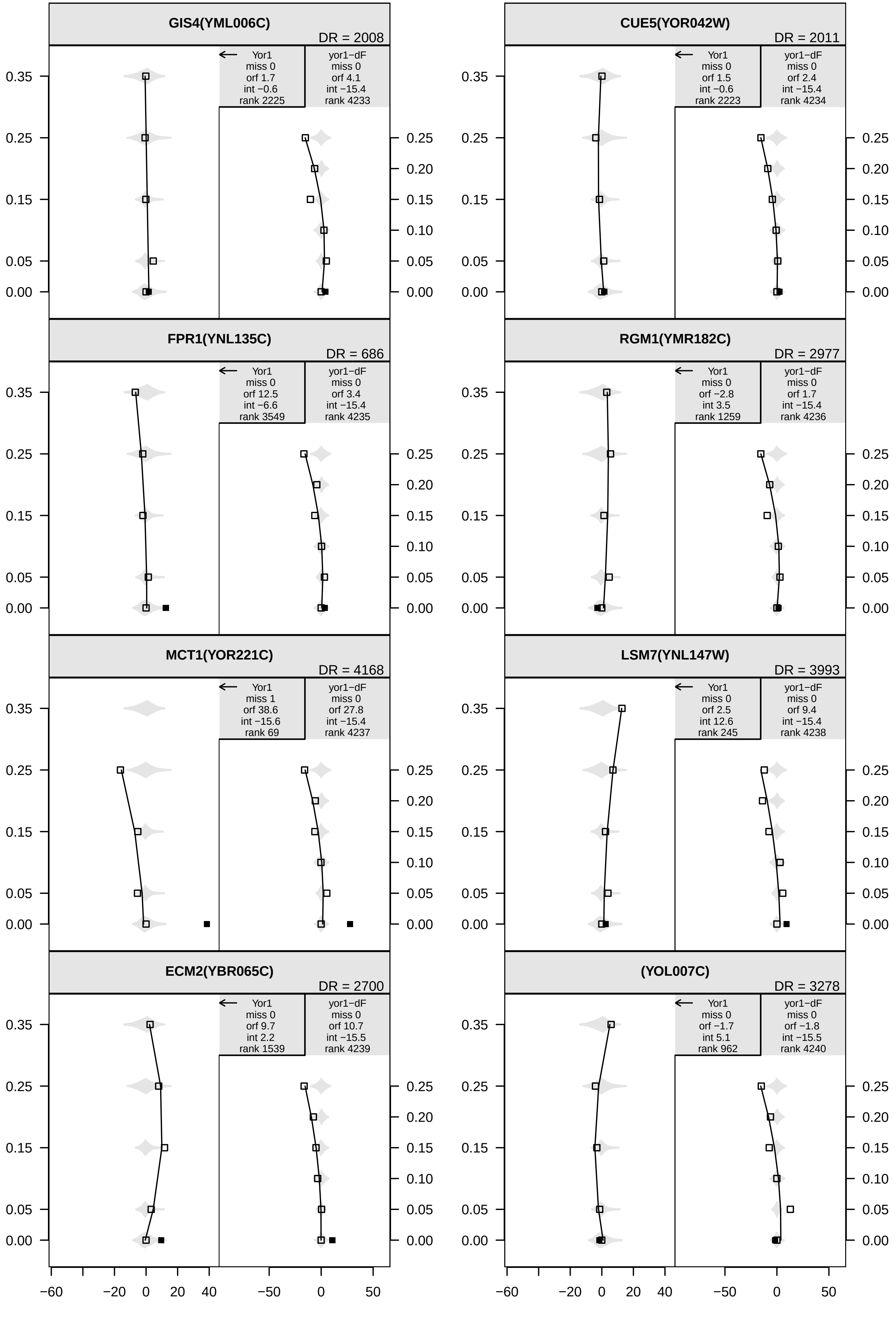

GIS4(YML006C)
DR = 2008
Yor1 miss 0 orf 1.7 int −0.6 rank 2225
yor1−dF miss 0 orf 4.1 int −15.4 rank 4233
CUE5(YOR042W)
DR = 2011
Yor1 miss 0 orf 1.5 int −0.6 rank 2223
yor1−dF miss 0 orf 2.4 int −15.4 rank 4234
FPR1(YNL135C)
DR = 686
Yor1 miss 0 orf 12.5 int −6.6 rank 3549
yor1−dF miss 0 orf 3.4 int −15.4 rank 4235
RGM1(YMR182C)
DR = 2977
Yor1 miss 0 orf −2.8 int 3.5 rank 1259
yor1−dF miss 0 orf 1.7 int −15.4 rank 4236
MCT1(YOR221C)
DR = 4168
Yor1 miss 1 orf 38.6 int −15.6 rank 69
yor1−dF miss 0 orf 27.8 int −15.4 rank 4237
LSM7(YNL147W)
DR = 3993
Yor1 miss 0 orf 2.5 int 12.6 rank 245
yor1−dF miss 0 orf 9.4 int −15.4 rank 4238
ECM2(YBR065C)
DR = 2700
Yor1 miss 0 orf 9.7 int 2.2 rank 1539
yor1−dF miss 0 orf 10.7 int −15.5 rank 4239
(YOL007C)
DR = 3278
Yor1 miss 0 orf −1.7 int 5.1 rank 962
yor1−dF miss 0 orf −1.8 int −15.5 rank 4240
0.35 0.25 0.20 0.15 0.10 0.05 0.00
−60 −20 0 20 40 −50 0 50

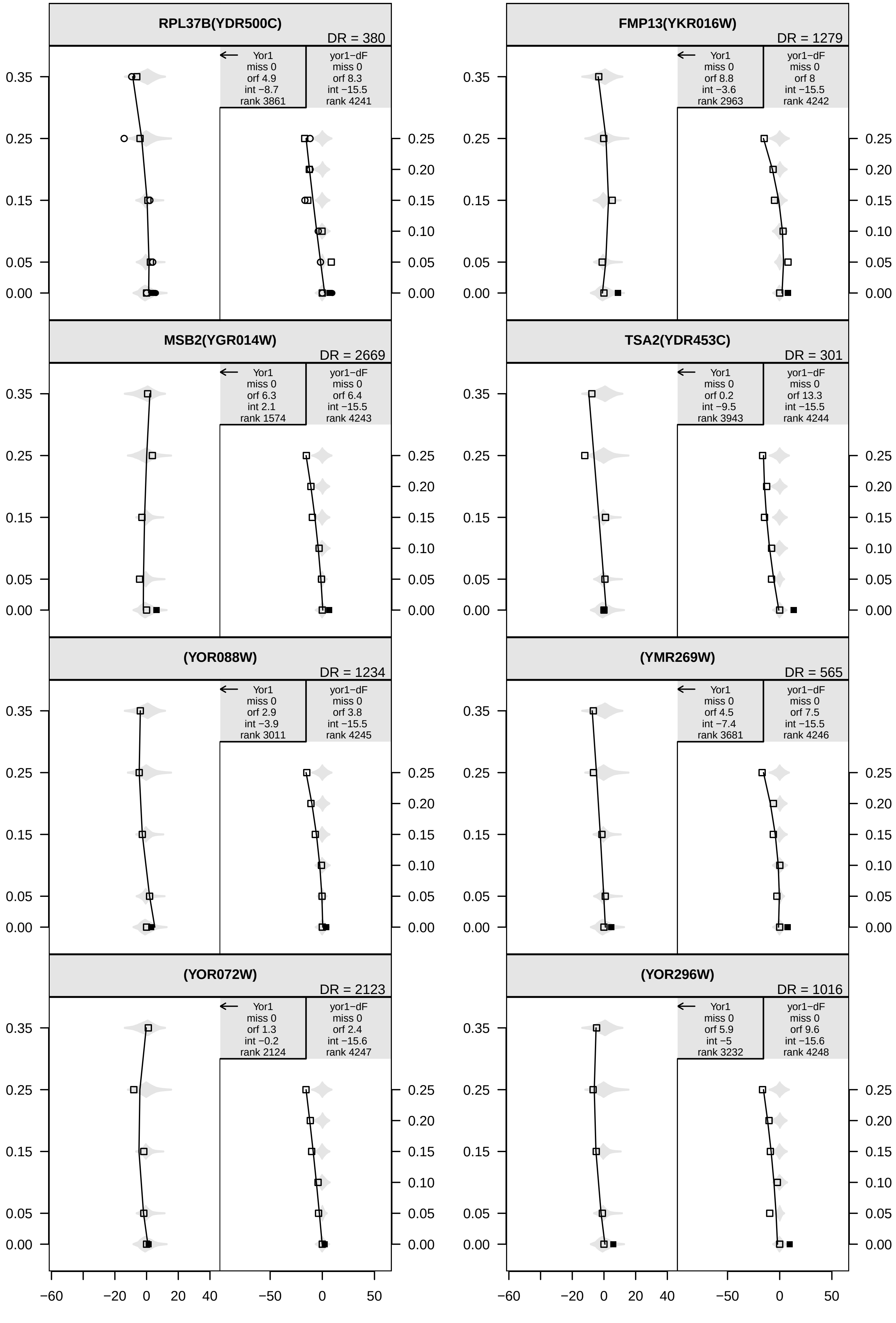
RPL37B(YDR500C)
DR = 380
Yor1
miss 0
orf 4.9
int −8.7
rank 3861
yor1−dF
miss 0
orf 8.3
int −15.5
rank 4241
FMP13(YKR016W)
DR = 1279
Yor1
miss 0
orf 8.8
int −3.6
rank 2963
yor1−dF
miss 0
orf 8
int −15.5
rank 4242
MSB2(YGR014W)
DR = 2669
Yor1
miss 0
orf 6.3
int 2.1
rank 1574
yor1−dF
miss 0
orf 6.4
int −15.5
rank 4243
TSA2(YDR453C)
DR = 301
Yor1
miss 0
orf 0.2
int −9.5
rank 3943
yor1−dF
miss 0
orf 13.3
int −15.5
rank 4244
(YOR088W)
DR = 1234
Yor1
miss 0
orf 2.9
int −3.9
rank 3011
yor1−dF
miss 0
orf 3.8
int −15.5
rank 4245
(YMR269W)
DR = 565
Yor1
miss 0
orf 4.5
int −7.4
rank 3681
yor1−dF
miss 0
orf 7.5
int −15.5
rank 4246
(YOR072W)
DR = 2123
Yor1
miss 0
orf 1.3
int −0.2
rank 2124
yor1−dF
miss 0
orf 2.4
int −15.6
rank 4247
(YOR296W)
DR = 1016
Yor1
miss 0
orf 5.9
int −5
rank 3232
yor1−dF
miss 0
orf 9.6
int −15.6
rank 4248
0.35
0.25
0.20
0.15
0.10
0.05
0.00
−60
−20
0
20
40
−50
0
50

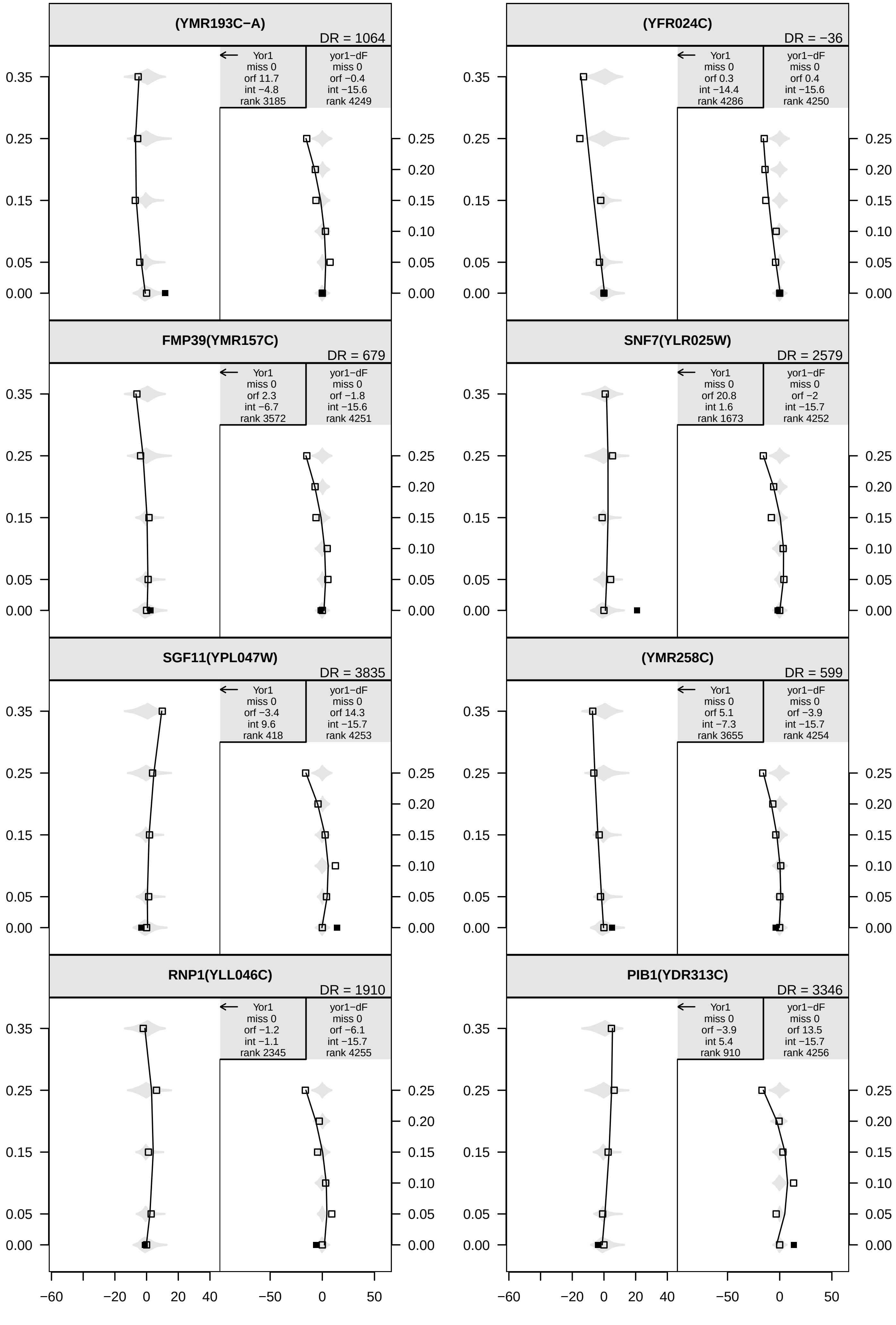

(YMR193C−A)
DR = 1064
Yor1
miss 0
orf 11.7
int −4.8
rank 3185
yor1−dF
miss 0
orf −0.4
int −15.6
rank 4249
(YFR024C)
DR = −36
Yor1
miss 0
orf 0.3
int −14.4
rank 4286
yor1−dF
miss 0
orf 0.4
int −15.6
rank 4250
FMP39(YMR157C)
DR = 679
Yor1
miss 0
orf 2.3
int −6.7
rank 3572
yor1−dF
miss 0
orf −1.8
int −15.6
rank 4251
SNF7(YLR025W)
DR = 2579
Yor1
miss 0
orf 20.8
int 1.6
rank 1673
yor1−dF
miss 0
orf −2
int −15.7
rank 4252
SGF11(YPL047W)
DR = 3835
Yor1
miss 0
orf −3.4
int 9.6
rank 418
yor1−dF
miss 0
orf 14.3
int −15.7
rank 4253
(YMR258C)
DR = 599
Yor1
miss 0
orf 5.1
int −7.3
rank 3655
yor1−dF
miss 0
orf −3.9
int −15.7
rank 4254
RNP1(YLL046C)
DR = 1910
Yor1
miss 0
orf −1.2
int −1.1
rank 2345
yor1−dF
miss 0
orf −6.1
int −15.7
rank 4255
PIB1(YDR313C)
DR = 3346
Yor1
miss 0
orf −3.9
int 5.4
rank 910
yor1−dF
miss 0
orf 13.5
int −15.7
rank 4256
0.35
0.25
0.20
0.15
0.10
0.05
0.00
−60
−20
0
20
40
−50
0
50

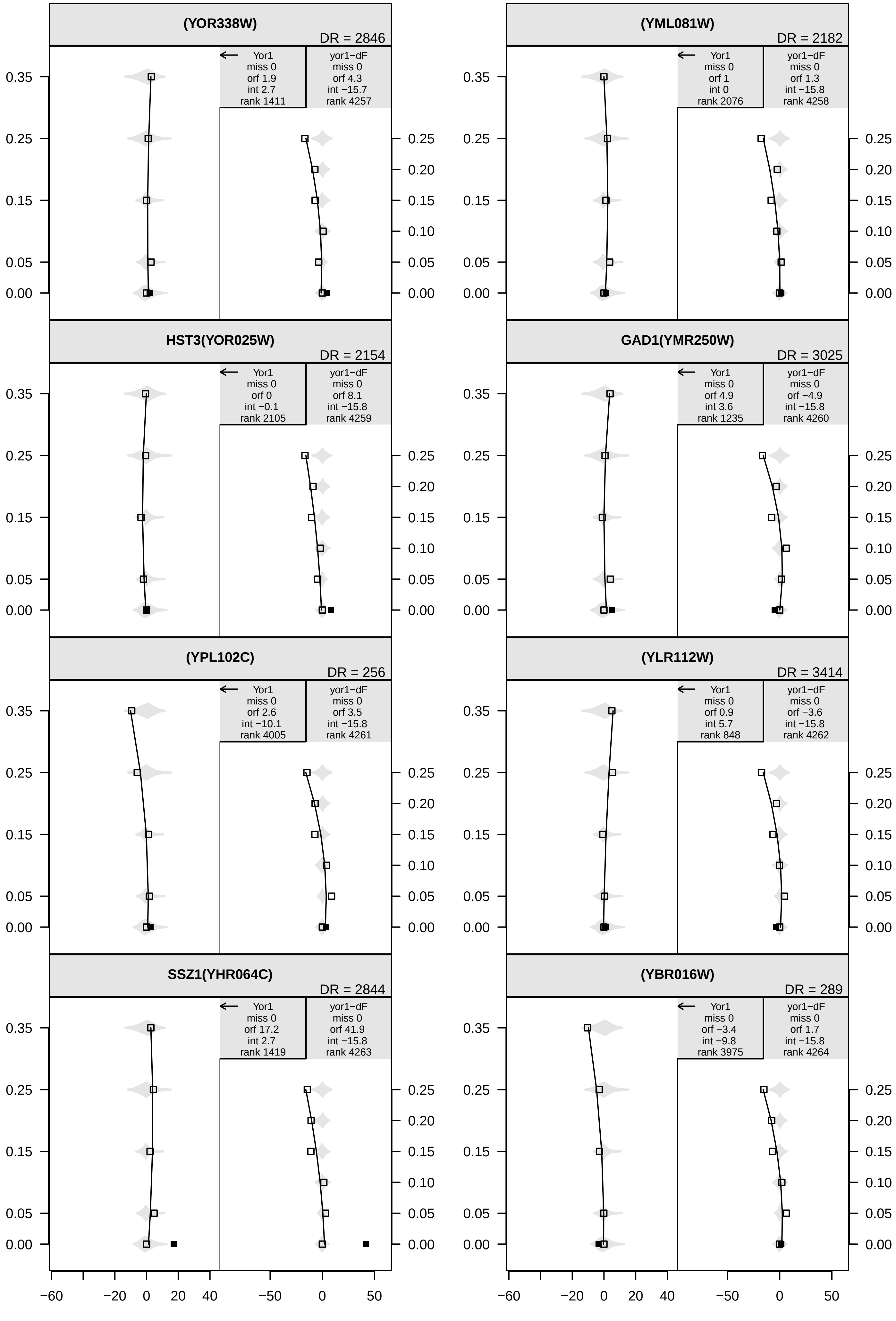

(YOR338W)
DR = 2846
Yor1
miss 0
orf 1.9
int 2.7
rank 1411
yor1−dF
miss 0
orf 4.3
int −15.7
rank 4257
(YML081W)
DR = 2182
Yor1
miss 0
orf 1
int 0
rank 2076
yor1−dF
miss 0
orf 1.3
int −15.8
rank 4258
HST3(YOR025W)
DR = 2154
Yor1
miss 0
orf 0
int −0.1
rank 2105
yor1−dF
miss 0
orf 8.1
int −15.8
rank 4259
GAD1(YMR250W)
DR = 3025
Yor1
miss 0
orf 4.9
int 3.6
rank 1235
yor1−dF
miss 0
orf −4.9
int −15.8
rank 4260
(YPL102C)
DR = 256
Yor1
miss 0
orf 2.6
int −10.1
rank 4005
yor1−dF
miss 0
orf 3.5
int −15.8
rank 4261
(YLR112W)
DR = 3414
Yor1
miss 0
orf 0.9
int 5.7
rank 848
yor1−dF
miss 0
orf −3.6
int −15.8
rank 4262
SSZ1(YHR064C)
DR = 2844
Yor1
miss 0
orf 17.2
int 2.7
rank 1419
yor1−dF
miss 0
orf 41.9
int −15.8
rank 4263
(YBR016W)
DR = 289
Yor1
miss 0
orf −3.4
int −9.8
rank 3975
yor1−dF
miss 0
orf 1.7
int −15.8
rank 4264
0.35
0.25
0.20
0.15
0.10
0.05
0.00
−60
−20
0
20
40
−50
0
50

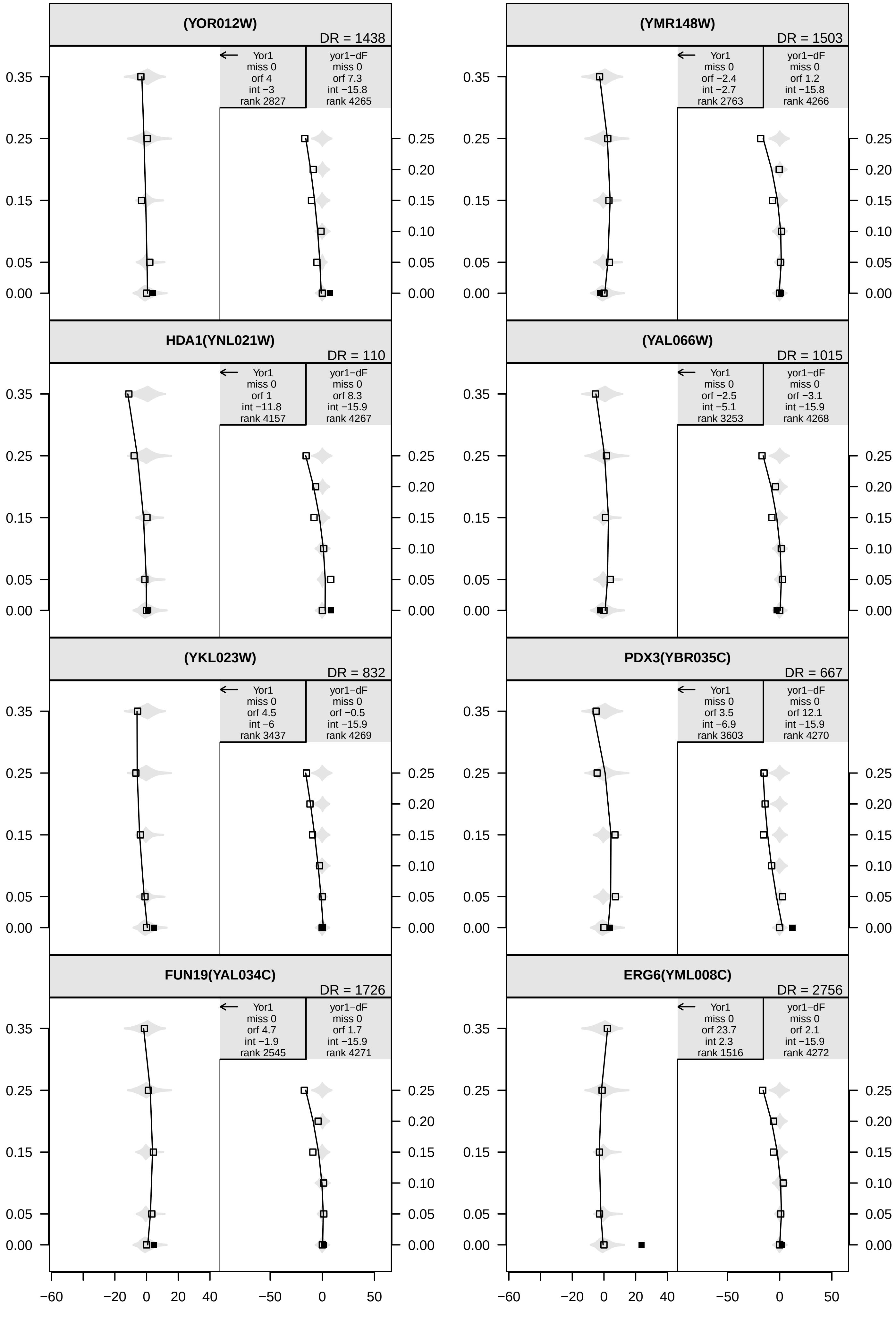
(YOR012W)
DR = 1438
Yor1 miss 0 orf 4 int −3 rank 2827
yor1−dF miss 0 orf 7.3 int −15.8 rank 4265
(YMR148W)
DR = 1503
Yor1 miss 0 orf −2.4 int −2.7 rank 2763
yor1−dF miss 0 orf 1.2 int −15.8 rank 4266
HDA1(YNL021W)
DR = 110
Yor1 miss 0 orf 1 int −11.8 rank 4157
yor1−dF miss 0 orf 8.3 int −15.9 rank 4267
(YAL066W)
DR = 1015
Yor1 miss 0 orf −2.5 int −5.1 rank 3253
yor1−dF miss 0 orf −3.1 int −15.9 rank 4268
(YKL023W)
DR = 832
Yor1 miss 0 orf 4.5 int −6 rank 3437
yor1−dF miss 0 orf −0.5 int −15.9 rank 4269
PDX3(YBR035C)
DR = 667
Yor1 miss 0 orf 3.5 int −6.9 rank 3603
yor1−dF miss 0 orf 12.1 int −15.9 rank 4270
FUN19(YAL034C)
DR = 1726
Yor1 miss 0 orf 4.7 int −1.9 rank 2545
yor1−dF miss 0 orf 1.7 int −15.9 rank 4271
ERG6(YML008C)
DR = 2756
Yor1 miss 0 orf 23.7 int 2.3 rank 1516
yor1−dF miss 0 orf 2.1 int −15.9 rank 4272

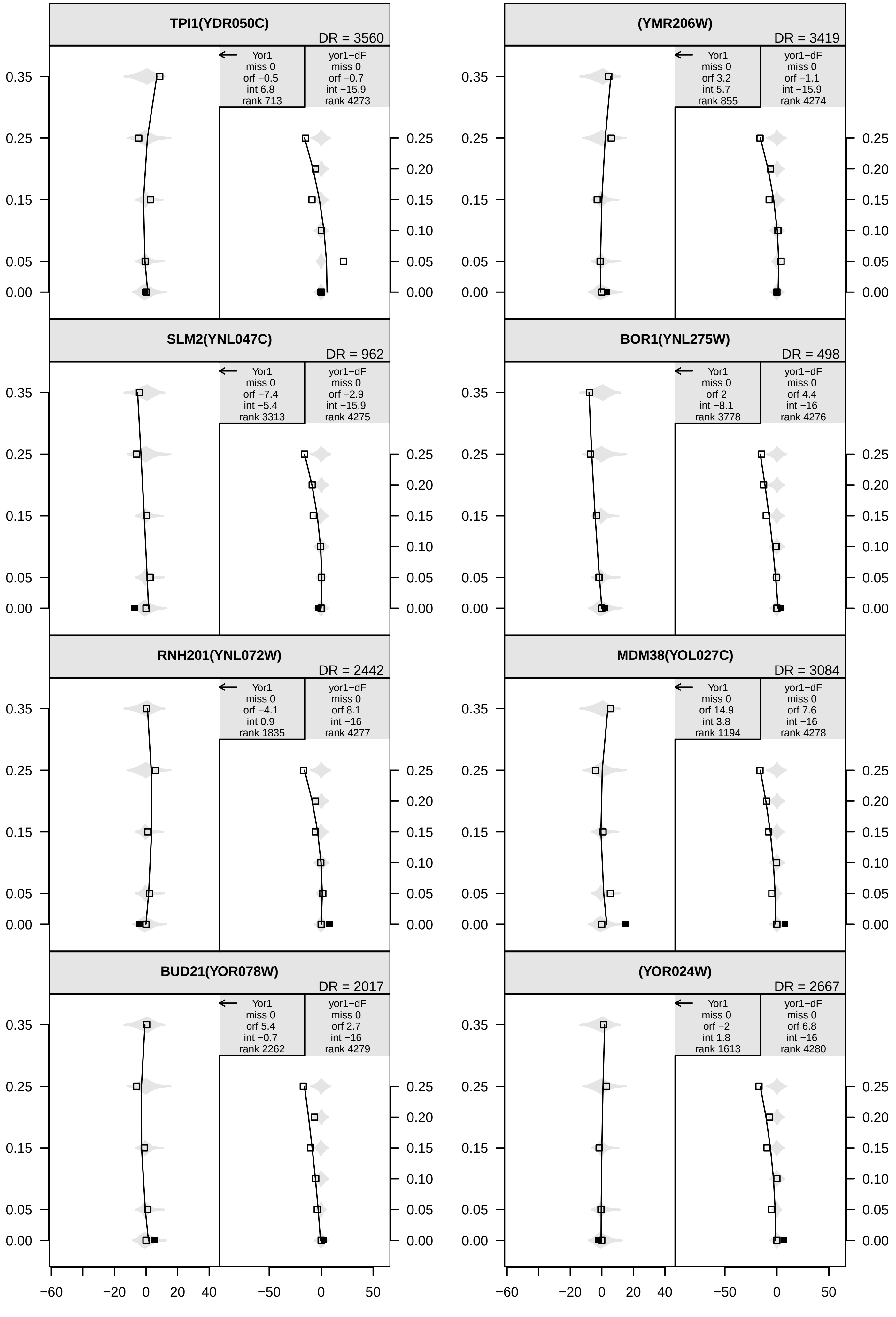

TPI1(YDR050C)
DR = 3560
Yor1 miss 0 orf −0.5 int 6.8 rank 713
yor1−dF miss 0 orf −0.7 int −15.9 rank 4273
(YMR206W)
DR = 3419
Yor1 miss 0 orf 3.2 int 5.7 rank 855
yor1−dF miss 0 orf −1.1 int −15.9 rank 4274
SLM2(YNL047C)
DR = 962
Yor1 miss 0 orf −7.4 int −5.4 rank 3313
yor1−dF miss 0 orf −2.9 int −15.9 rank 4275
BOR1(YNL275W)
DR = 498
Yor1 miss 0 orf 2 int −8.1 rank 3778
yor1−dF miss 0 orf 4.4 int −16 rank 4276
RNH201(YNL072W)
DR = 2442
Yor1 miss 0 orf −4.1 int 0.9 rank 1835
yor1−dF miss 0 orf 8.1 int −16 rank 4277
MDM38(YOL027C)
DR = 3084
Yor1 miss 0 orf 14.9 int 3.8 rank 1194
yor1−dF miss 0 orf 7.6 int −16 rank 4278
BUD21(YOR078W)
DR = 2017
Yor1 miss 0 orf 5.4 int −0.7 rank 2262
yor1−dF miss 0 orf 2.7 int −16 rank 4279
(YOR024W)
DR = 2667
Yor1 miss 0 orf −2 int 1.8 rank 1613
yor1−dF miss 0 orf 6.8 int −16 rank 4280
0.35 0.25 0.15 0.05 0.00
0.25 0.20 0.15 0.10 0.05 0.00
−60 −20 0 20 40
−50 0 50

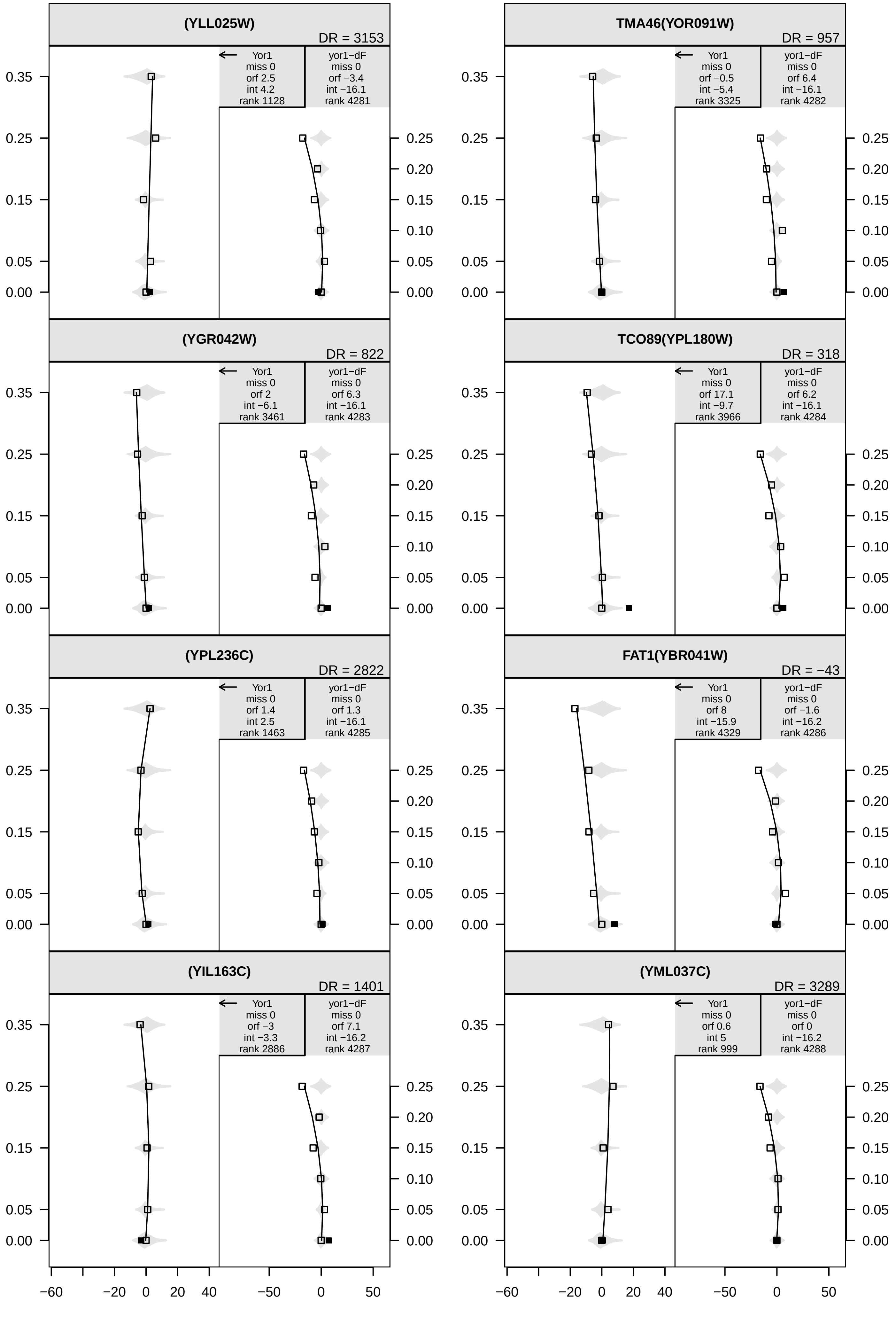

(YLL025W)
DR = 3153
Yor1 miss 0 orf 2.5 int 4.2 rank 1128
yor1−dF miss 0 orf −3.4 int −16.1 rank 4281
TMA46(YOR091W)
DR = 957
Yor1 miss 0 orf −0.5 int −5.4 rank 3325
yor1−dF miss 0 orf 6.4 int −16.1 rank 4282
(YGR042W)
DR = 822
Yor1 miss 0 orf 2 int −6.1 rank 3461
yor1−dF miss 0 orf 6.3 int −16.1 rank 4283
TCO89(YPL180W)
DR = 318
Yor1 miss 0 orf 17.1 int −9.7 rank 3966
yor1−dF miss 0 orf 6.2 int −16.1 rank 4284
(YPL236C)
DR = 2822
Yor1 miss 0 orf 1.4 int 2.5 rank 1463
yor1−dF miss 0 orf 1.3 int −16.1 rank 4285
FAT1(YBR041W)
DR = −43
Yor1 miss 0 orf 8 int −15.9 rank 4329
yor1−dF miss 0 orf −1.6 int −16.2 rank 4286
(YIL163C)
DR = 1401
Yor1 miss 0 orf −3 int −3.3 rank 2886
yor1−dF miss 0 orf 7.1 int −16.2 rank 4287
(YML037C)
DR = 3289
Yor1 miss 0 orf 0.6 int 5 rank 999
yor1−dF miss 0 orf 0 int −16.2 rank 4288

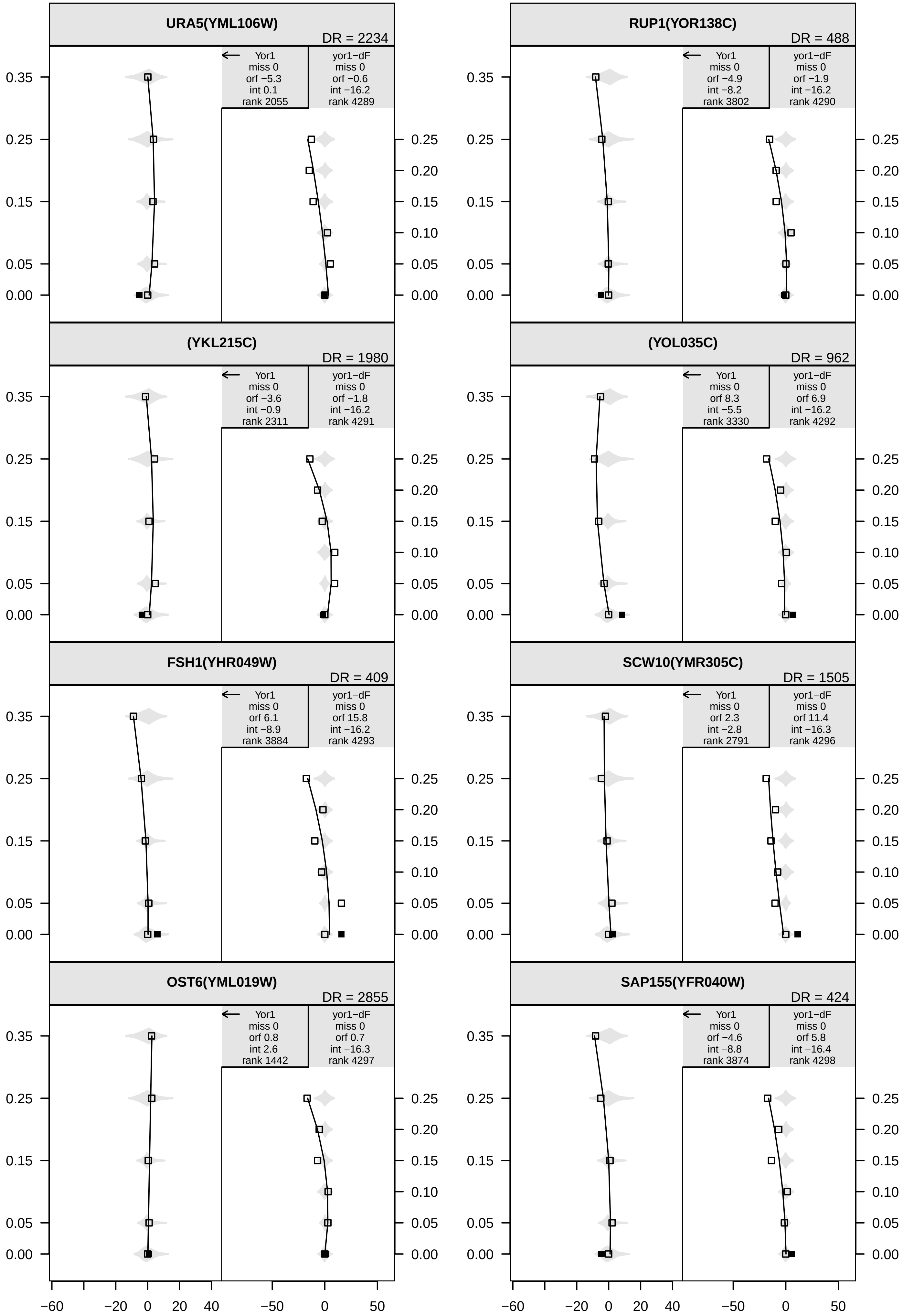
URA5(YML106W)
DR = 2234
Yor1 miss 0 orf −5.3 int 0.1 rank 2055
yor1−dF miss 0 orf −0.6 int −16.2 rank 4289
RUP1(YOR138C)
DR = 488
Yor1 miss 0 orf −4.9 int −8.2 rank 3802
yor1−dF miss 0 orf −1.9 int −16.2 rank 4290
(YKL215C)
DR = 1980
Yor1 miss 0 orf −3.6 int −0.9 rank 2311
yor1−dF miss 0 orf −1.8 int −16.2 rank 4291
(YOL035C)
DR = 962
Yor1 miss 0 orf 8.3 int −5.5 rank 3330
yor1−dF miss 0 orf 6.9 int −16.2 rank 4292
FSH1(YHR049W)
DR = 409
Yor1 miss 0 orf 6.1 int −8.9 rank 3884
yor1−dF miss 0 orf 15.8 int −16.2 rank 4293
SCW10(YMR305C)
DR = 1505
Yor1 miss 0 orf 2.3 int −2.8 rank 2791
yor1−dF miss 0 orf 11.4 int −16.3 rank 4296
OST6(YML019W)
DR = 2855
Yor1 miss 0 orf 0.8 int 2.6 rank 1442
yor1−dF miss 0 orf 0.7 int −16.3 rank 4297
SAP155(YFR040W)
DR = 424
Yor1 miss 0 orf −4.6 int −8.8 rank 3874
yor1−dF miss 0 orf 5.8 int −16.4 rank 4298
0.35 0.25 0.15 0.05 0.00
0.25 0.20 0.15 0.10 0.05 0.00
−60 −20 0 20 40
−50 0 50

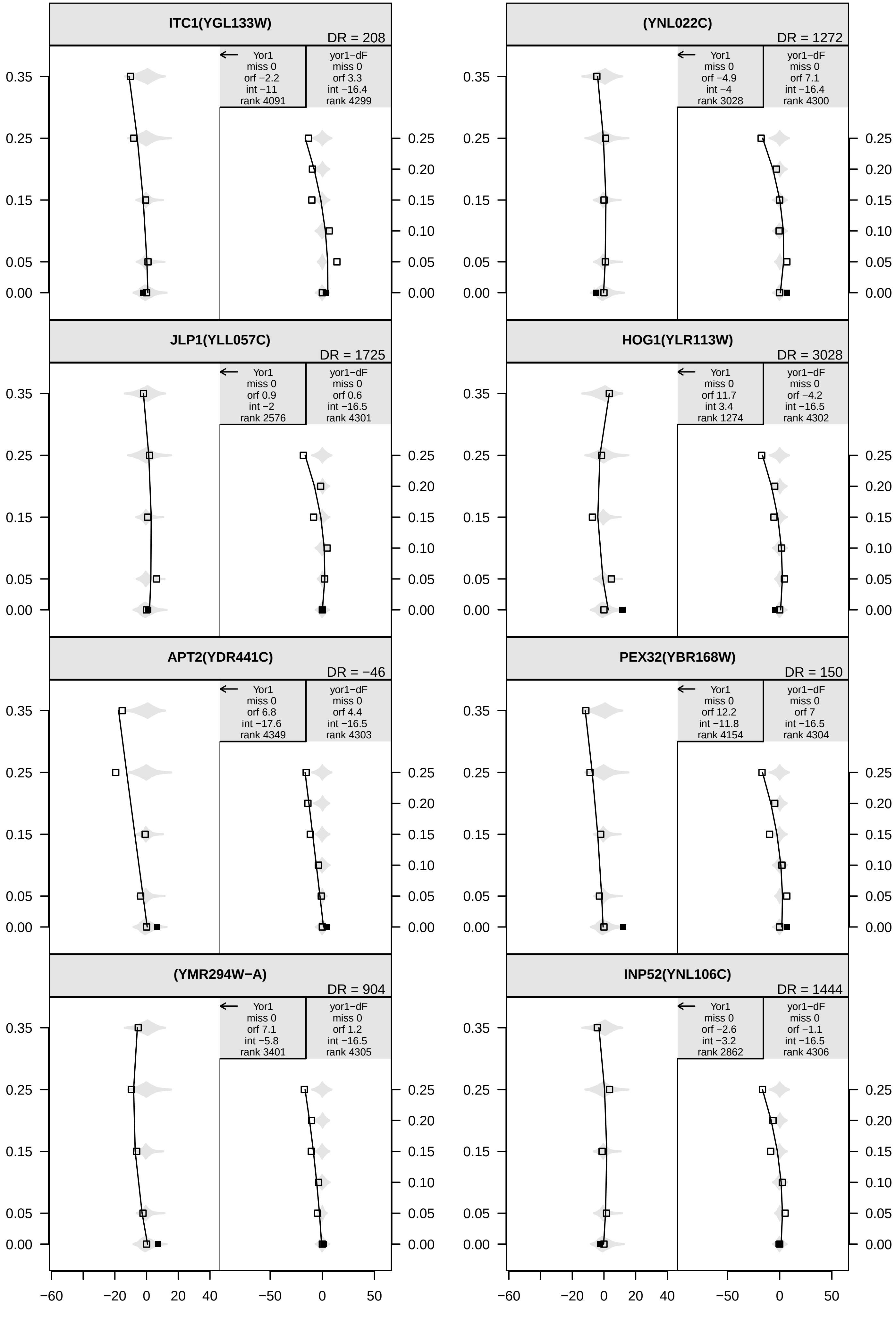
ITC1(YGL133W)
DR = 208
Yor1 miss 0 orf −2.2 int −11 rank 4091
yor1−dF miss 0 orf 3.3 int −16.4 rank 4299
(YNL022C)
DR = 1272
Yor1 miss 0 orf −4.9 int −4 rank 3028
yor1−dF miss 0 orf 7.1 int −16.4 rank 4300
JLP1(YLL057C)
DR = 1725
Yor1 miss 0 orf 0.9 int −2 rank 2576
yor1−dF miss 0 orf 0.6 int −16.5 rank 4301
HOG1(YLR113W)
DR = 3028
Yor1 miss 0 orf 11.7 int 3.4 rank 1274
yor1−dF miss 0 orf −4.2 int −16.5 rank 4302
APT2(YDR441C)
DR = −46
Yor1 miss 0 orf 6.8 int −17.6 rank 4349
yor1−dF miss 0 orf 4.4 int −16.5 rank 4303
PEX32(YBR168W)
DR = 150
Yor1 miss 0 orf 12.2 int −11.8 rank 4154
yor1−dF miss 0 orf 7 int −16.5 rank 4304
(YMR294W−A)
DR = 904
Yor1 miss 0 orf 7.1 int −5.8 rank 3401
yor1−dF miss 0 orf 1.2 int −16.5 rank 4305
INP52(YNL106C)
DR = 1444
Yor1 miss 0 orf −2.6 int −3.2 rank 2862
yor1−dF miss 0 orf −1.1 int −16.5 rank 4306
0.00 0.05 0.10 0.15 0.20 0.25 0.35
−60 −20 0 20 40 −50 0 50

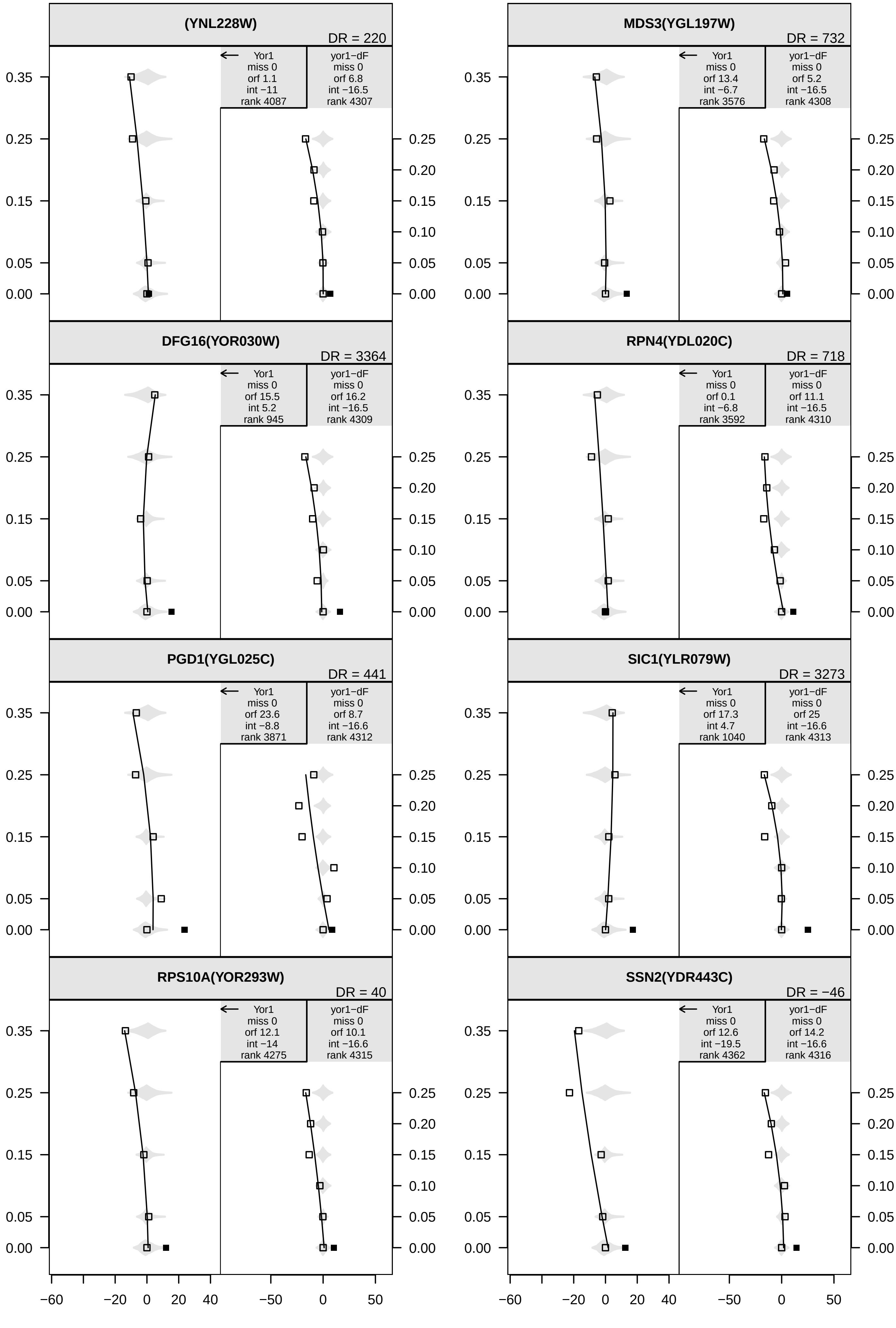
(YNL228W)
DR = 220
Yor1
miss 0
orf 1.1
int −11
rank 4087
yor1−dF
miss 0
orf 6.8
int −16.5
rank 4307
MDS3(YGL197W)
DR = 732
Yor1
miss 0
orf 13.4
int −6.7
rank 3576
yor1−dF
miss 0
orf 5.2
int −16.5
rank 4308
DFG16(YOR030W)
DR = 3364
Yor1
miss 0
orf 15.5
int 5.2
rank 945
yor1−dF
miss 0
orf 16.2
int −16.5
rank 4309
RPN4(YDL020C)
DR = 718
Yor1
miss 0
orf 0.1
int −6.8
rank 3592
yor1−dF
miss 0
orf 11.1
int −16.5
rank 4310
PGD1(YGL025C)
DR = 441
Yor1
miss 0
orf 23.6
int −8.8
rank 3871
yor1−dF
miss 0
orf 8.7
int −16.6
rank 4312
SIC1(YLR079W)
DR = 3273
Yor1
miss 0
orf 17.3
int 4.7
rank 1040
yor1−dF
miss 0
orf 25
int −16.6
rank 4313
RPS10A(YOR293W)
DR = 40
Yor1
miss 0
orf 12.1
int −14
rank 4275
yor1−dF
miss 0
orf 10.1
int −16.6
rank 4315
SSN2(YDR443C)
DR = −46
Yor1
miss 0
orf 12.6
int −19.5
rank 4362
yor1−dF
miss 0
orf 14.2
int −16.6
rank 4316

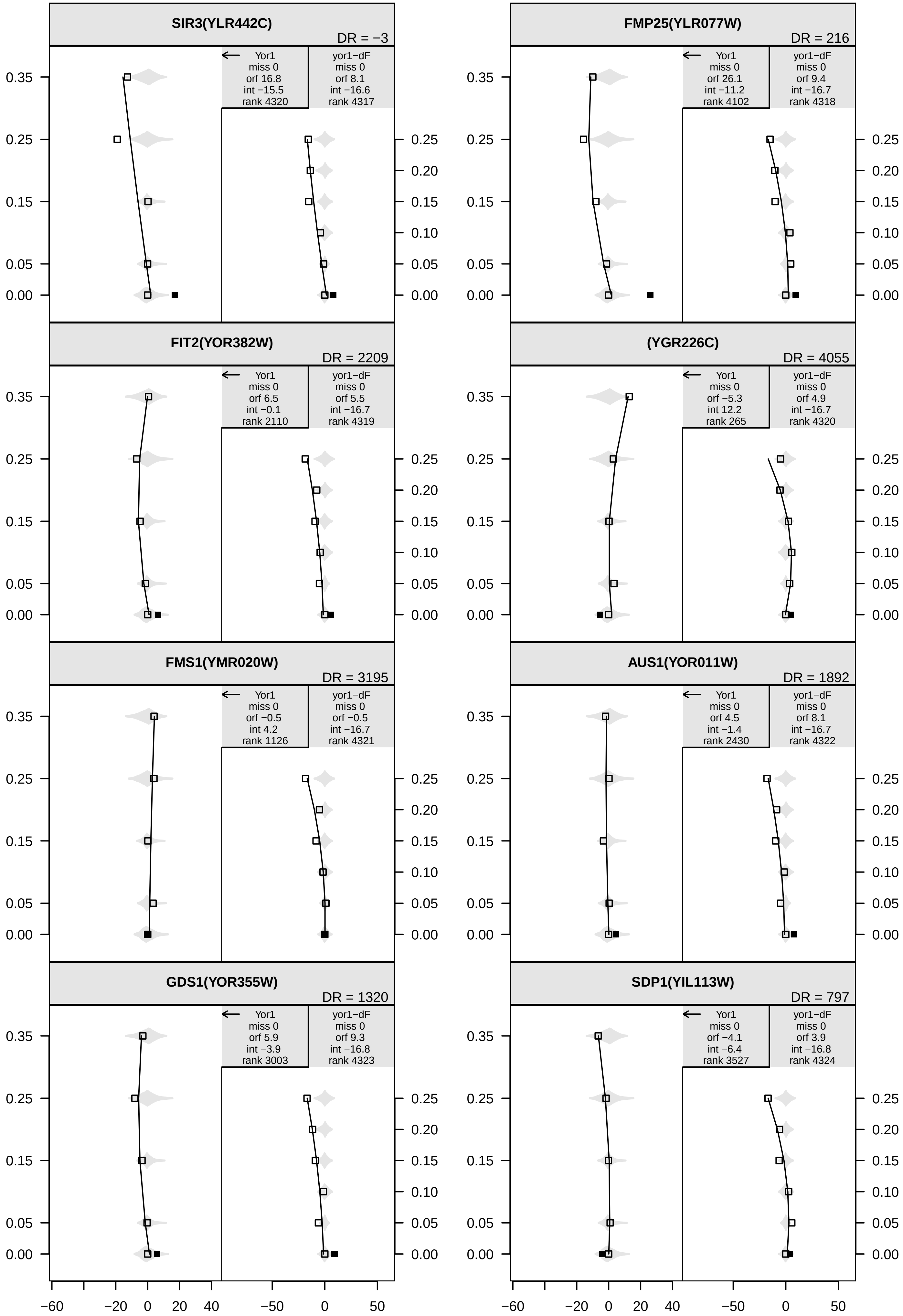

SIR3(YLR442C)
DR = −3
Yor1 miss 0 orf 16.8 int −15.5 rank 4320
yor1−dF miss 0 orf 8.1 int −16.6 rank 4317
FMP25(YLR077W)
DR = 216
Yor1 miss 0 orf 26.1 int −11.2 rank 4102
yor1−dF miss 0 orf 9.4 int −16.7 rank 4318
FIT2(YOR382W)
DR = 2209
Yor1 miss 0 orf 6.5 int −0.1 rank 2110
yor1−dF miss 0 orf 5.5 int −16.7 rank 4319
(YGR226C)
DR = 4055
Yor1 miss 0 orf −5.3 int 12.2 rank 265
yor1−dF miss 0 orf 4.9 int −16.7 rank 4320
FMS1(YMR020W)
DR = 3195
Yor1 miss 0 orf −0.5 int 4.2 rank 1126
yor1−dF miss 0 orf −0.5 int −16.7 rank 4321
AUS1(YOR011W)
DR = 1892
Yor1 miss 0 orf 4.5 int −1.4 rank 2430
yor1−dF miss 0 orf 8.1 int −16.7 rank 4322
GDS1(YOR355W)
DR = 1320
Yor1 miss 0 orf 5.9 int −3.9 rank 3003
yor1−dF miss 0 orf 9.3 int −16.8 rank 4323
SDP1(YIL113W)
DR = 797
Yor1 miss 0 orf −4.1 int −6.4 rank 3527
yor1−dF miss 0 orf 3.9 int −16.8 rank 4324
0.35 0.25 0.20 0.15 0.10 0.05 0.00
−60 −20 0 20 40 −50 0 50

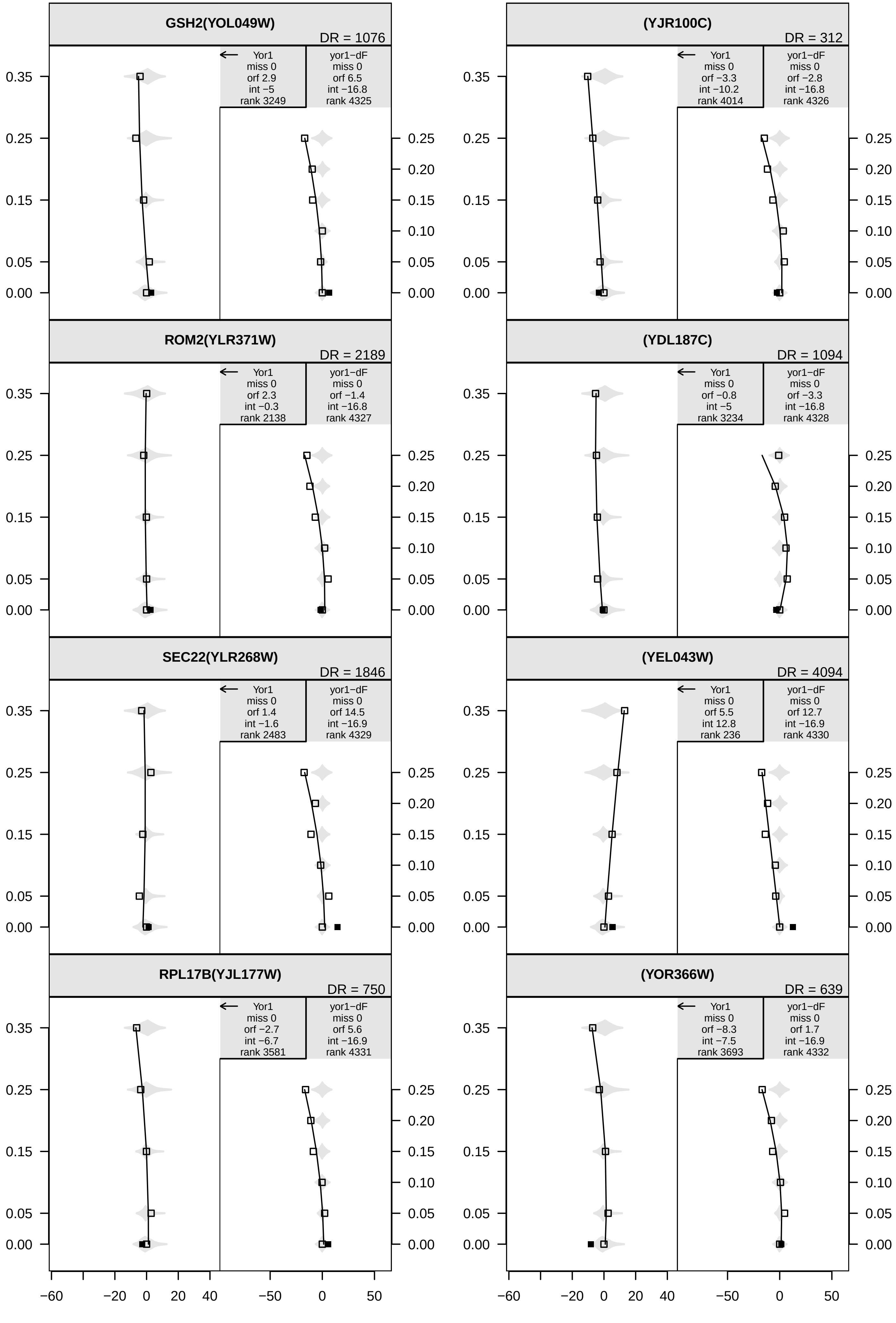
GSH2(YOL049W)
DR = 1076
Yor1
miss 0
orf 2.9
int −5
rank 3249
yor1−dF
miss 0
orf 6.5
int −16.8
rank 4325
(YJR100C)
DR = 312
Yor1
miss 0
orf −3.3
int −10.2
rank 4014
yor1−dF
miss 0
orf −2.8
int −16.8
rank 4326
ROM2(YLR371W)
DR = 2189
Yor1
miss 0
orf 2.3
int −0.3
rank 2138
yor1−dF
miss 0
orf −1.4
int −16.8
rank 4327
(YDL187C)
DR = 1094
Yor1
miss 0
orf −0.8
int −5
rank 3234
yor1−dF
miss 0
orf −3.3
int −16.8
rank 4328
SEC22(YLR268W)
DR = 1846
Yor1
miss 0
orf 1.4
int −1.6
rank 2483
yor1−dF
miss 0
orf 14.5
int −16.9
rank 4329
(YEL043W)
DR = 4094
Yor1
miss 0
orf 5.5
int 12.8
rank 236
yor1−dF
miss 0
orf 12.7
int −16.9
rank 4330
RPL17B(YJL177W)
DR = 750
Yor1
miss 0
orf −2.7
int −6.7
rank 3581
yor1−dF
miss 0
orf 5.6
int −16.9
rank 4331
(YOR366W)
DR = 639
Yor1
miss 0
orf −8.3
int −7.5
rank 3693
yor1−dF
miss 0
orf 1.7
int −16.9
rank 4332
0.35
0.25
0.20
0.15
0.10
0.05
0.00
−60
−20
0
20
40
−50
0
50

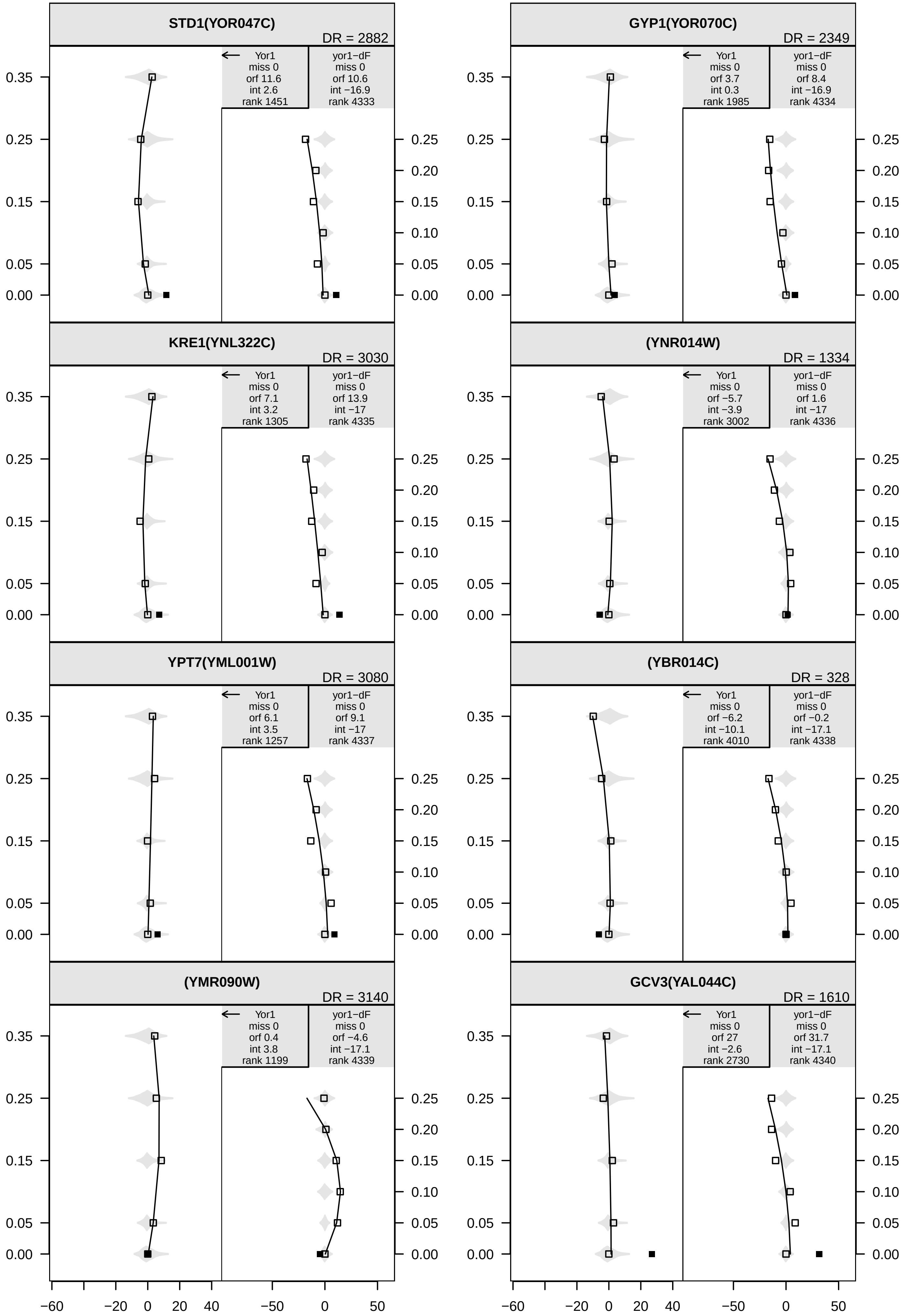

STD1(YOR047C)
DR = 2882
Yor1 miss 0 orf 11.6 int 2.6 rank 1451
yor1−dF miss 0 orf 10.6 int −16.9 rank 4333
GYP1(YOR070C)
DR = 2349
Yor1 miss 0 orf 3.7 int 0.3 rank 1985
yor1−dF miss 0 orf 8.4 int −16.9 rank 4334
KRE1(YNL322C)
DR = 3030
Yor1 miss 0 orf 7.1 int 3.2 rank 1305
yor1−dF miss 0 orf 13.9 int −17 rank 4335
(YNR014W)
DR = 1334
Yor1 miss 0 orf −5.7 int −3.9 rank 3002
yor1−dF miss 0 orf 1.6 int −17 rank 4336
YPT7(YML001W)
DR = 3080
Yor1 miss 0 orf 6.1 int 3.5 rank 1257
yor1−dF miss 0 orf 9.1 int −17 rank 4337
(YBR014C)
DR = 328
Yor1 miss 0 orf −6.2 int −10.1 rank 4010
yor1−dF miss 0 orf −0.2 int −17.1 rank 4338
(YMR090W)
DR = 3140
Yor1 miss 0 orf 0.4 int 3.8 rank 1199
yor1−dF miss 0 orf −4.6 int −17.1 rank 4339
GCV3(YAL044C)
DR = 1610
Yor1 miss 0 orf 27 int −2.6 rank 2730
yor1−dF miss 0 orf 31.7 int −17.1 rank 4340
0.35 0.25 0.15 0.05 0.00
0.25 0.20 0.15 0.10 0.05 0.00
−60 −20 0 20 40 −50 0 50

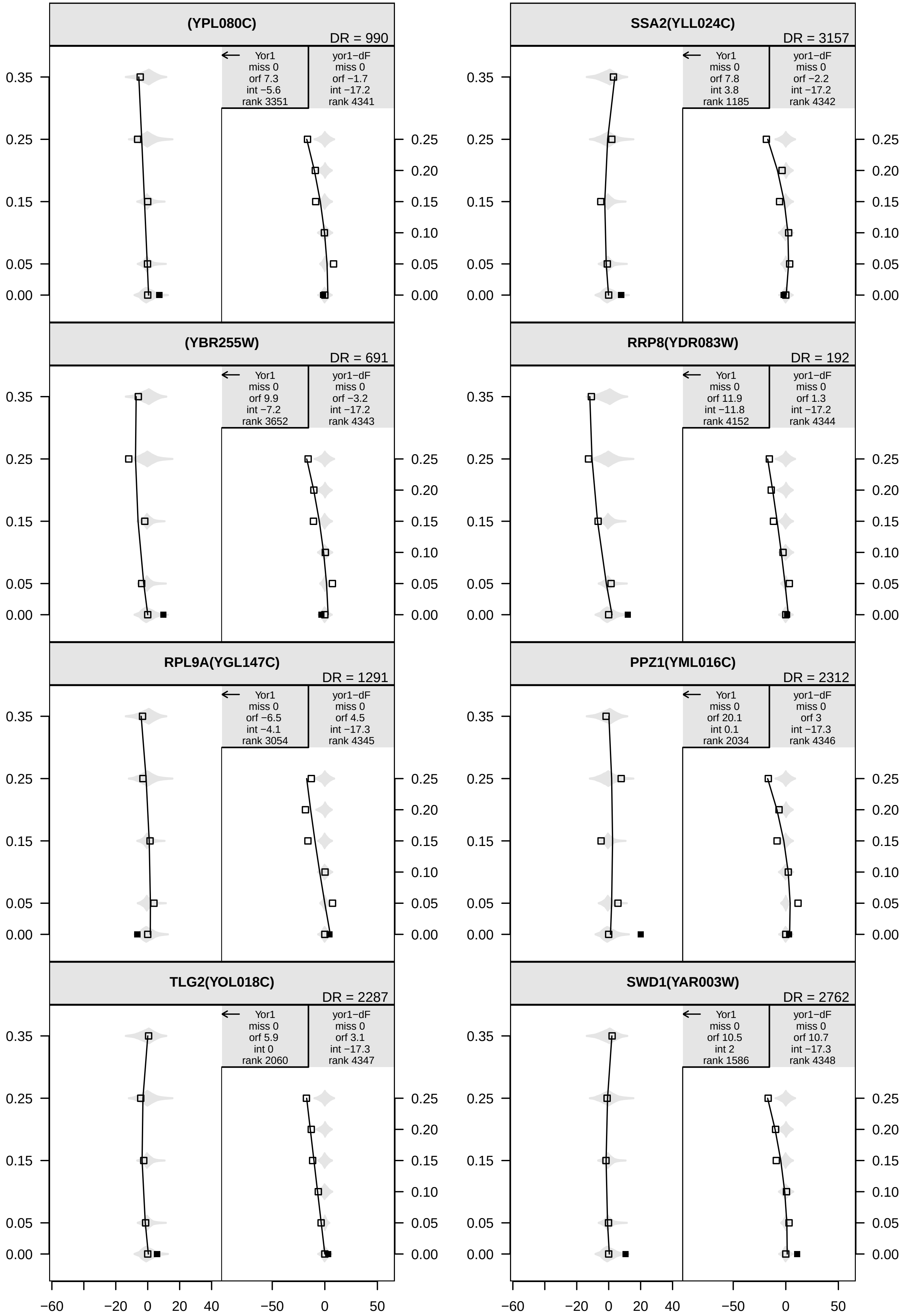

(YPL080C)
DR = 990
Yor1
miss 0
orf 7.3
int −5.6
rank 3351
yor1−dF
miss 0
orf −1.7
int −17.2
rank 4341
SSA2(YLL024C)
DR = 3157
Yor1
miss 0
orf 7.8
int 3.8
rank 1185
yor1−dF
miss 0
orf −2.2
int −17.2
rank 4342
(YBR255W)
DR = 691
Yor1
miss 0
orf 9.9
int −7.2
rank 3652
yor1−dF
miss 0
orf −3.2
int −17.2
rank 4343
RRP8(YDR083W)
DR = 192
Yor1
miss 0
orf 11.9
int −11.8
rank 4152
yor1−dF
miss 0
orf 1.3
int −17.2
rank 4344
RPL9A(YGL147C)
DR = 1291
Yor1
miss 0
orf −6.5
int −4.1
rank 3054
yor1−dF
miss 0
orf 4.5
int −17.3
rank 4345
PPZ1(YML016C)
DR = 2312
Yor1
miss 0
orf 20.1
int 0.1
rank 2034
yor1−dF
miss 0
orf 3
int −17.3
rank 4346
TLG2(YOL018C)
DR = 2287
Yor1
miss 0
orf 5.9
int 0
rank 2060
yor1−dF
miss 0
orf 3.1
int −17.3
rank 4347
SWD1(YAR003W)
DR = 2762
Yor1
miss 0
orf 10.5
int 2
rank 1586
yor1−dF
miss 0
orf 10.7
int −17.3
rank 4348
0.35
0.25
0.20
0.15
0.10
0.05
0.00
−60
−20
0
20
40
−50
0
50

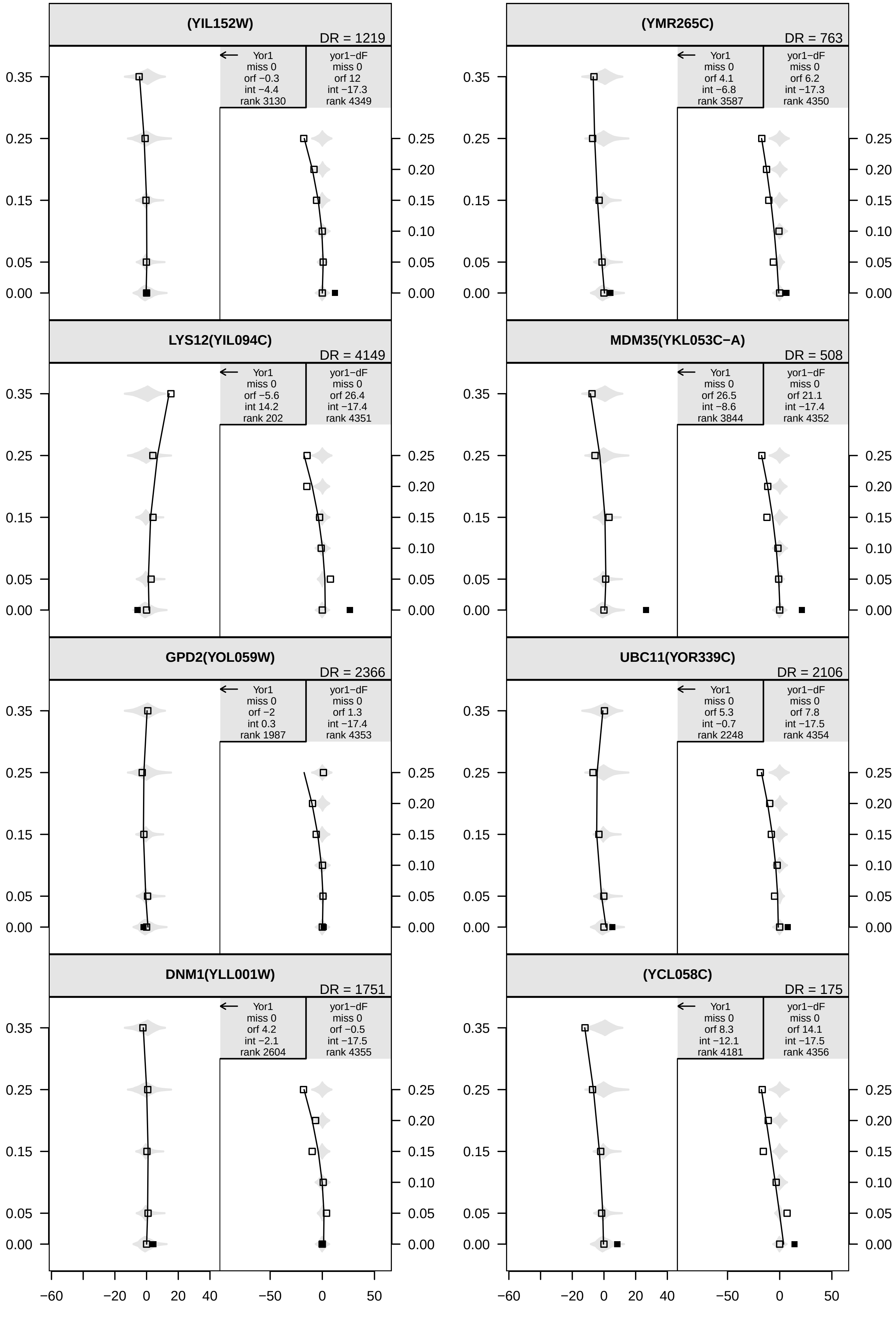
(YIL152W)
DR = 1219
Yor1
miss 0
orf −0.3
int −4.4
rank 3130
yor1−dF
miss 0
orf 12
int −17.3
rank 4349
(YMR265C)
DR = 763
Yor1
miss 0
orf 4.1
int −6.8
rank 3587
yor1−dF
miss 0
orf 6.2
int −17.3
rank 4350
LYS12(YIL094C)
DR = 4149
Yor1
miss 0
orf −5.6
int 14.2
rank 202
yor1−dF
miss 0
orf 26.4
int −17.4
rank 4351
MDM35(YKL053C−A)
DR = 508
Yor1
miss 0
orf 26.5
int −8.6
rank 3844
yor1−dF
miss 0
orf 21.1
int −17.4
rank 4352
GPD2(YOL059W)
DR = 2366
Yor1
miss 0
orf −2
int 0.3
rank 1987
yor1−dF
miss 0
orf 1.3
int −17.4
rank 4353
UBC11(YOR339C)
DR = 2106
Yor1
miss 0
orf 5.3
int −0.7
rank 2248
yor1−dF
miss 0
orf 7.8
int −17.5
rank 4354
DNM1(YLL001W)
DR = 1751
Yor1
miss 0
orf 4.2
int −2.1
rank 2604
yor1−dF
miss 0
orf −0.5
int −17.5
rank 4355
(YCL058C)
DR = 175
Yor1
miss 0
orf 8.3
int −12.1
rank 4181
yor1−dF
miss 0
orf 14.1
int −17.5
rank 4356
0.35
0.25
0.20
0.15
0.10
0.05
0.00
−60
−20
0
20
40
−50
0
50

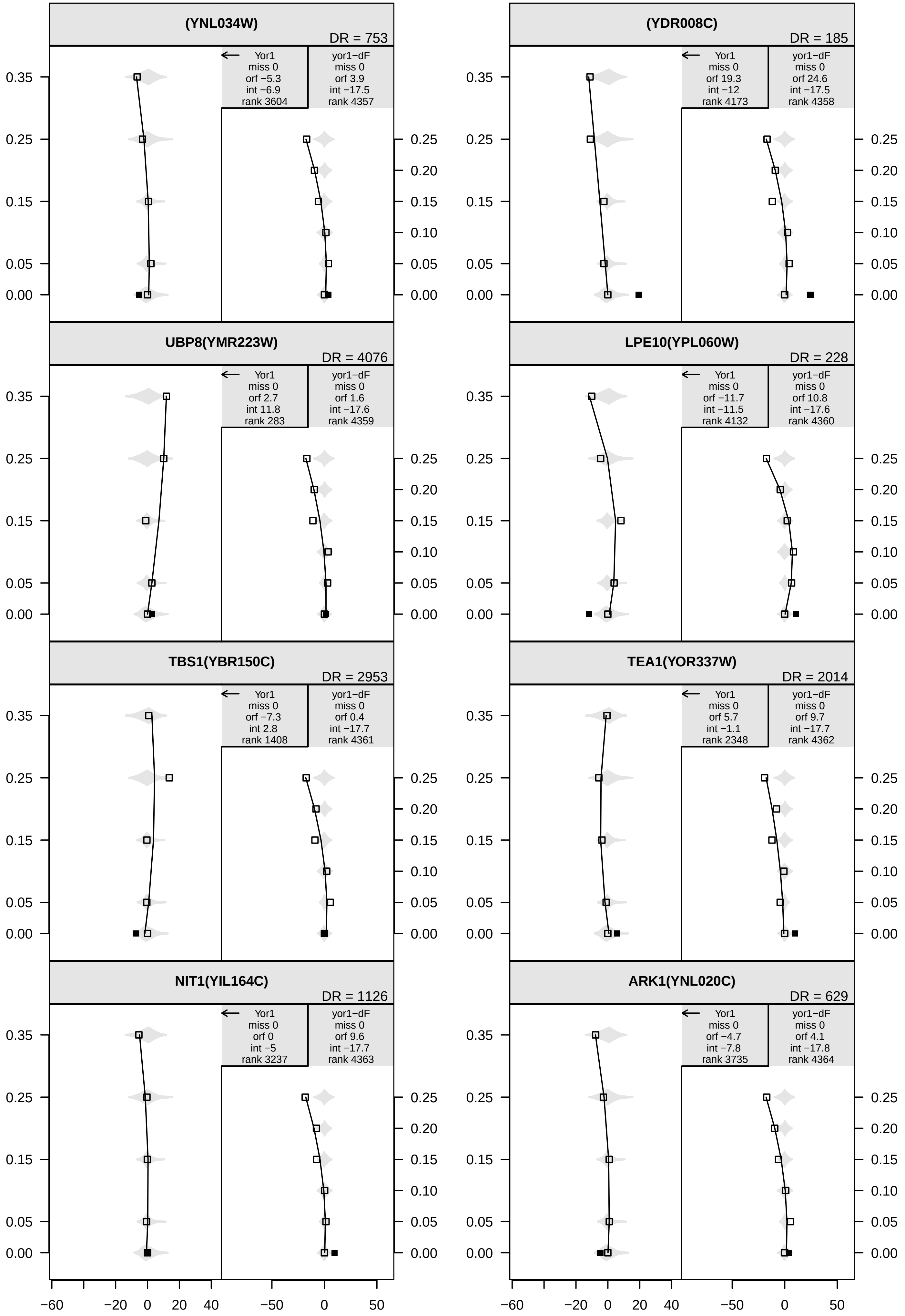
(YNL034W)
DR = 753
Yor1 miss 0 orf −5.3 int −6.9 rank 3604
yor1−dF miss 0 orf 3.9 int −17.5 rank 4357
(YDR008C)
DR = 185
Yor1 miss 0 orf 19.3 int −12 rank 4173
yor1−dF miss 0 orf 24.6 int −17.5 rank 4358
UBP8(YMR223W)
DR = 4076
Yor1 miss 0 orf 2.7 int 11.8 rank 283
yor1−dF miss 0 orf 1.6 int −17.6 rank 4359
LPE10(YPL060W)
DR = 228
Yor1 miss 0 orf −11.7 int −11.5 rank 4132
yor1−dF miss 0 orf 10.8 int −17.6 rank 4360
TBS1(YBR150C)
DR = 2953
Yor1 miss 0 orf −7.3 int 2.8 rank 1408
yor1−dF miss 0 orf 0.4 int −17.7 rank 4361
TEA1(YOR337W)
DR = 2014
Yor1 miss 0 orf 5.7 int −1.1 rank 2348
yor1−dF miss 0 orf 9.7 int −17.7 rank 4362
NIT1(YIL164C)
DR = 1126
Yor1 miss 0 orf 0 int −5 rank 3237
yor1−dF miss 0 orf 9.6 int −17.7 rank 4363
ARK1(YNL020C)
DR = 629
Yor1 miss 0 orf −4.7 int −7.8 rank 3735
yor1−dF miss 0 orf 4.1 int −17.8 rank 4364
0.35 0.25 0.15 0.05 0.00
0.25 0.20 0.15 0.10 0.05 0.00
−60 −20 0 20 40 −50 0 50

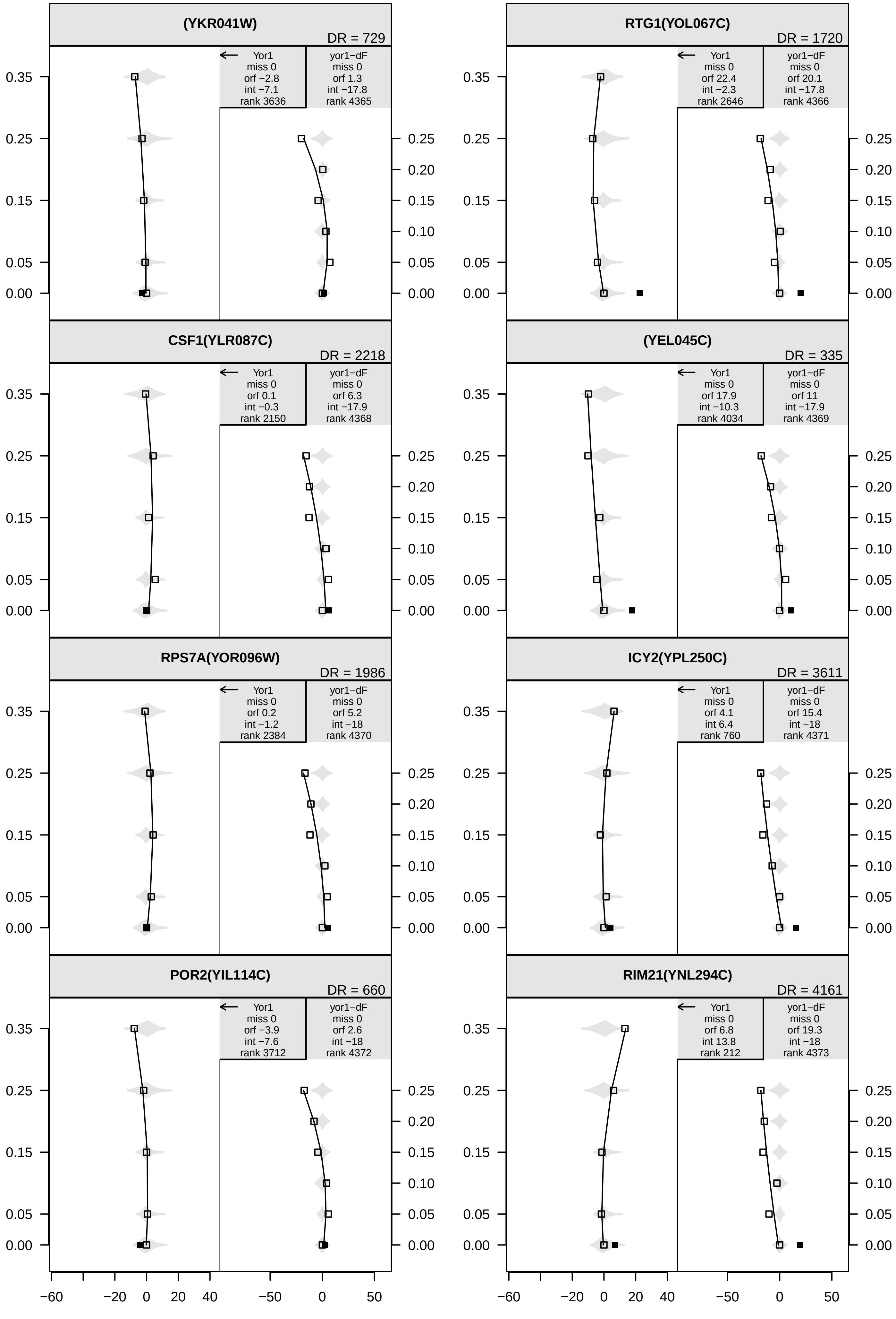
(YKR041W)
DR = 729
Yor1
miss 0
orf −2.8
int −7.1
rank 3636
yor1−dF
miss 0
orf 1.3
int −17.8
rank 4365
RTG1(YOL067C)
DR = 1720
Yor1
miss 0
orf 22.4
int −2.3
rank 2646
yor1−dF
miss 0
orf 20.1
int −17.8
rank 4366
CSF1(YLR087C)
DR = 2218
Yor1
miss 0
orf 0.1
int −0.3
rank 2150
yor1−dF
miss 0
orf 6.3
int −17.9
rank 4368
(YEL045C)
DR = 335
Yor1
miss 0
orf 17.9
int −10.3
rank 4034
yor1−dF
miss 0
orf 11
int −17.9
rank 4369
RPS7A(YOR096W)
DR = 1986
Yor1
miss 0
orf 0.2
int −1.2
rank 2384
yor1−dF
miss 0
orf 5.2
int −18
rank 4370
ICY2(YPL250C)
DR = 3611
Yor1
miss 0
orf 4.1
int 6.4
rank 760
yor1−dF
miss 0
orf 15.4
int −18
rank 4371
POR2(YIL114C)
DR = 660
Yor1
miss 0
orf −3.9
int −7.6
rank 3712
yor1−dF
miss 0
orf 2.6
int −18
rank 4372
RIM21(YNL294C)
DR = 4161
Yor1
miss 0
orf 6.8
int 13.8
rank 212
yor1−dF
miss 0
orf 19.3
int −18
rank 4373
0.35
0.25
0.20
0.15
0.10
0.05
0.00
−60
−20
0
20
40
−50
0
50

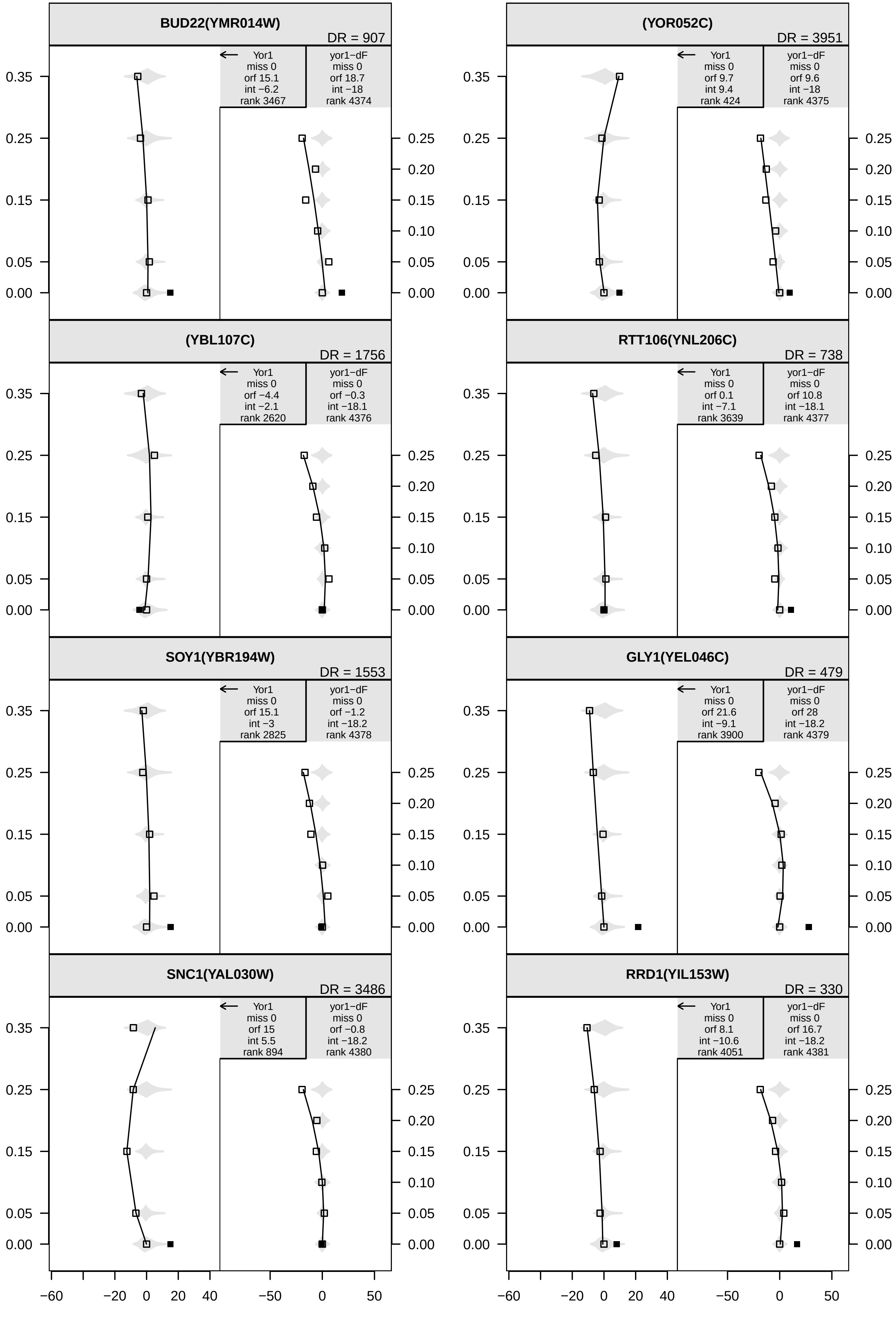
BUD22(YMR014W)
DR = 907
Yor1
miss 0
orf 15.1
int −6.2
rank 3467
yor1−dF
miss 0
orf 18.7
int −18
rank 4374
(YOR052C)
DR = 3951
Yor1
miss 0
orf 9.7
int 9.4
rank 424
yor1−dF
miss 0
orf 9.6
int −18
rank 4375
(YBL107C)
DR = 1756
Yor1
miss 0
orf −4.4
int −2.1
rank 2620
yor1−dF
miss 0
orf −0.3
int −18.1
rank 4376
RTT106(YNL206C)
DR = 738
Yor1
miss 0
orf 0.1
int −7.1
rank 3639
yor1−dF
miss 0
orf 10.8
int −18.1
rank 4377
SOY1(YBR194W)
DR = 1553
Yor1
miss 0
orf 15.1
int −3
rank 2825
yor1−dF
miss 0
orf −1.2
int −18.2
rank 4378
GLY1(YEL046C)
DR = 479
Yor1
miss 0
orf 21.6
int −9.1
rank 3900
yor1−dF
miss 0
orf 28
int −18.2
rank 4379
SNC1(YAL030W)
DR = 3486
Yor1
miss 0
orf 15
int 5.5
rank 894
yor1−dF
miss 0
orf −0.8
int −18.2
rank 4380
RRD1(YIL153W)
DR = 330
Yor1
miss 0
orf 8.1
int −10.6
rank 4051
yor1−dF
miss 0
orf 16.7
int −18.2
rank 4381
0.35
0.25
0.20
0.15
0.10
0.05
0.00
−60
−20
0
20
40
−50
0
50

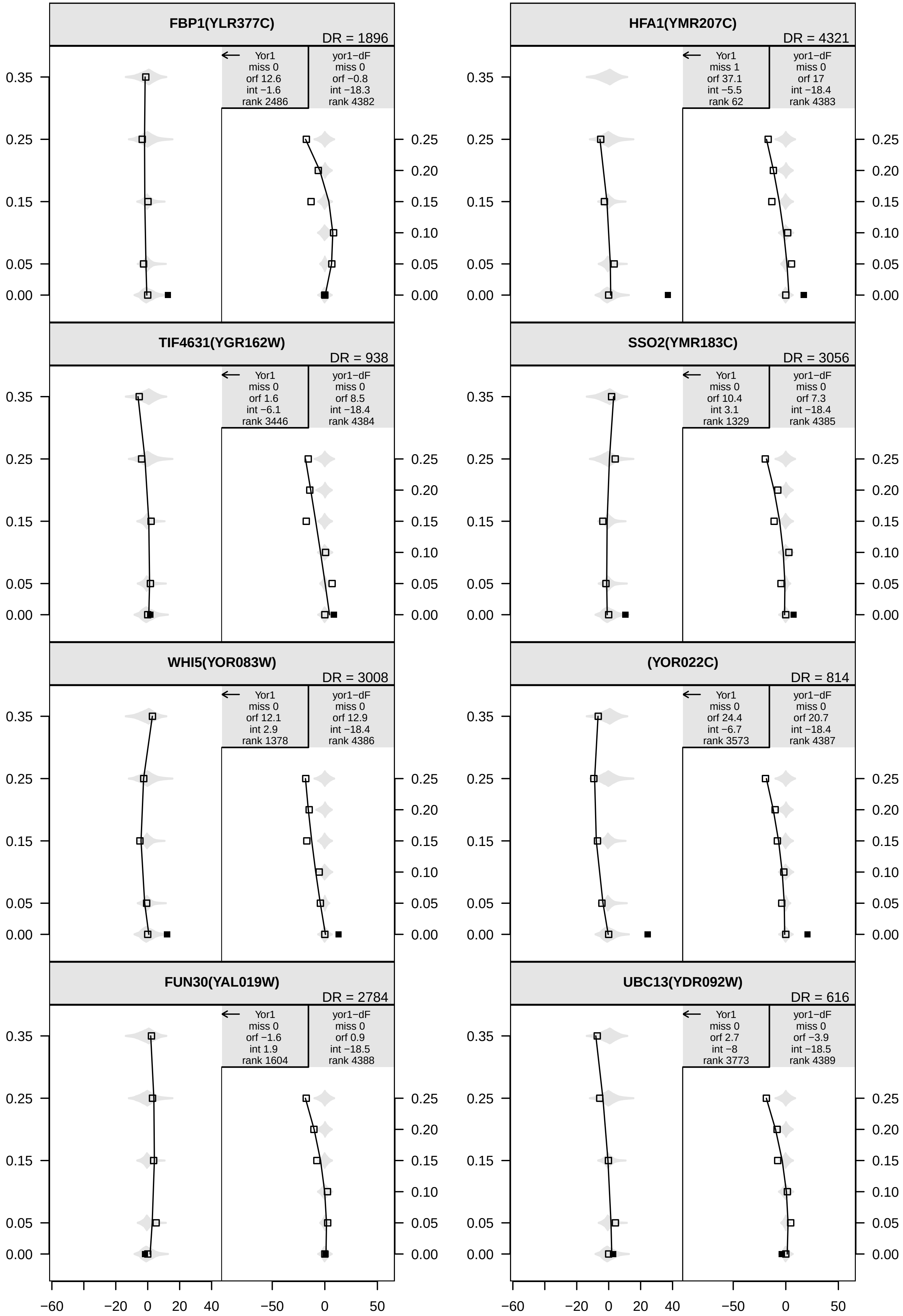
FBP1(YLR377C)
DR = 1896
Yor1
miss 0
orf 12.6
int −1.6
rank 2486
yor1−dF
miss 0
orf −0.8
int −18.3
rank 4382
HFA1(YMR207C)
DR = 4321
Yor1
miss 1
orf 37.1
int −5.5
rank 62
yor1−dF
miss 0
orf 17
int −18.4
rank 4383
TIF4631(YGR162W)
DR = 938
Yor1
miss 0
orf 1.6
int −6.1
rank 3446
yor1−dF
miss 0
orf 8.5
int −18.4
rank 4384
SSO2(YMR183C)
DR = 3056
Yor1
miss 0
orf 10.4
int 3.1
rank 1329
yor1−dF
miss 0
orf 7.3
int −18.4
rank 4385
WHI5(YOR083W)
DR = 3008
Yor1
miss 0
orf 12.1
int 2.9
rank 1378
yor1−dF
miss 0
orf 12.9
int −18.4
rank 4386
(YOR022C)
DR = 814
Yor1
miss 0
orf 24.4
int −6.7
rank 3573
yor1−dF
miss 0
orf 20.7
int −18.4
rank 4387
FUN30(YAL019W)
DR = 2784
Yor1
miss 0
orf −1.6
int 1.9
rank 1604
yor1−dF
miss 0
orf 0.9
int −18.5
rank 4388
UBC13(YDR092W)
DR = 616
Yor1
miss 0
orf 2.7
int −8
rank 3773
yor1−dF
miss 0
orf −3.9
int −18.5
rank 4389
0.35
0.25
0.20
0.15
0.10
0.05
0.00
−60
−20
0
20
40
−50
0
50

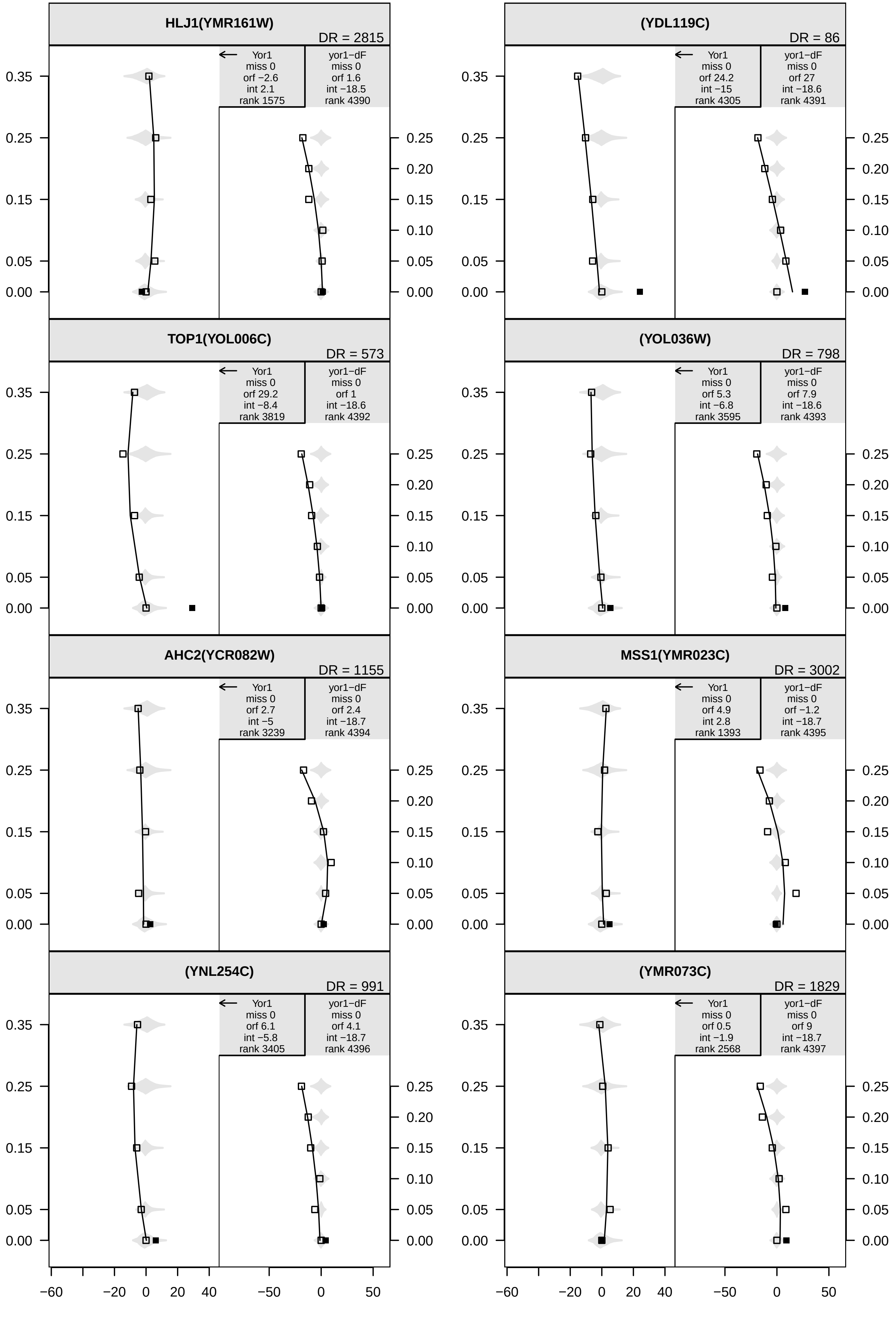

HLJ1(YMR161W)
DR = 2815
Yor1 miss 0 orf −2.6 int 2.1 rank 1575
yor1−dF miss 0 orf 1.6 int −18.5 rank 4390
(YDL119C)
DR = 86
Yor1 miss 0 orf 24.2 int −15 rank 4305
yor1−dF miss 0 orf 27 int −18.6 rank 4391
TOP1(YOL006C)
DR = 573
Yor1 miss 0 orf 29.2 int −8.4 rank 3819
yor1−dF miss 0 orf 1 int −18.6 rank 4392
(YOL036W)
DR = 798
Yor1 miss 0 orf 5.3 int −6.8 rank 3595
yor1−dF miss 0 orf 7.9 int −18.6 rank 4393
AHC2(YCR082W)
DR = 1155
Yor1 miss 0 orf 2.7 int −5 rank 3239
yor1−dF miss 0 orf 2.4 int −18.7 rank 4394
MSS1(YMR023C)
DR = 3002
Yor1 miss 0 orf 4.9 int 2.8 rank 1393
yor1−dF miss 0 orf −1.2 int −18.7 rank 4395
(YNL254C)
DR = 991
Yor1 miss 0 orf 6.1 int −5.8 rank 3405
yor1−dF miss 0 orf 4.1 int −18.7 rank 4396
(YMR073C)
DR = 1829
Yor1 miss 0 orf 0.5 int −1.9 rank 2568
yor1−dF miss 0 orf 9 int −18.7 rank 4397

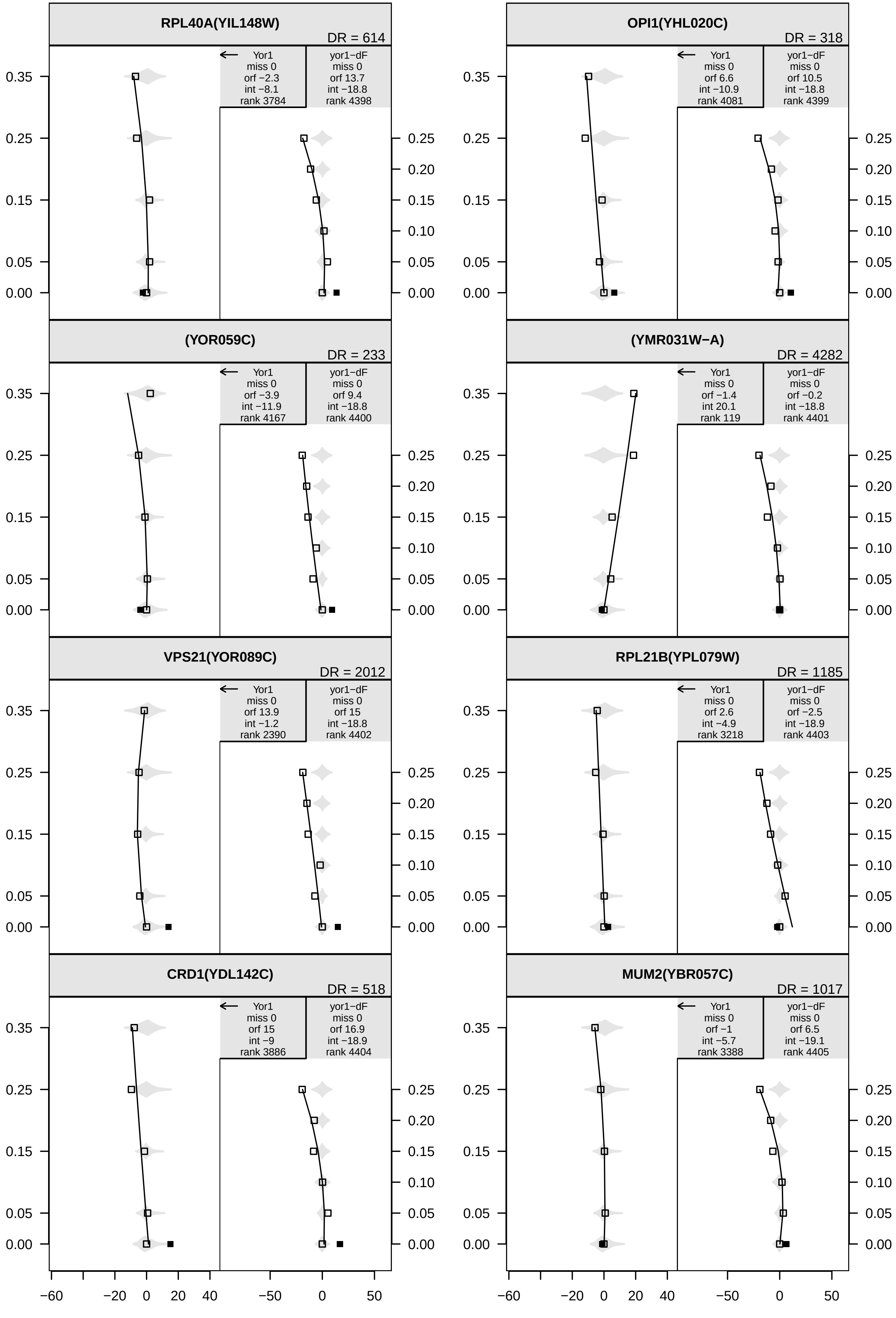

RPL40A(YIL148W)
DR = 614
Yor1 miss 0 orf −2.3 int −8.1 rank 3784
yor1−dF miss 0 orf 13.7 int −18.8 rank 4398
OPI1(YHL020C)
DR = 318
Yor1 miss 0 orf 6.6 int −10.9 rank 4081
yor1−dF miss 0 orf 10.5 int −18.8 rank 4399
(YOR059C)
DR = 233
Yor1 miss 0 orf −3.9 int −11.9 rank 4167
yor1−dF miss 0 orf 9.4 int −18.8 rank 4400
(YMR031W−A)
DR = 4282
Yor1 miss 0 orf −1.4 int 20.1 rank 119
yor1−dF miss 0 orf −0.2 int −18.8 rank 4401
VPS21(YOR089C)
DR = 2012
Yor1 miss 0 orf 13.9 int −1.2 rank 2390
yor1−dF miss 0 orf 15 int −18.8 rank 4402
RPL21B(YPL079W)
DR = 1185
Yor1 miss 0 orf 2.6 int −4.9 rank 3218
yor1−dF miss 0 orf −2.5 int −18.9 rank 4403
CRD1(YDL142C)
DR = 518
Yor1 miss 0 orf 15 int −9 rank 3886
yor1−dF miss 0 orf 16.9 int −18.9 rank 4404
MUM2(YBR057C)
DR = 1017
Yor1 miss 0 orf −1 int −5.7 rank 3388
yor1−dF miss 0 orf 6.5 int −19.1 rank 4405

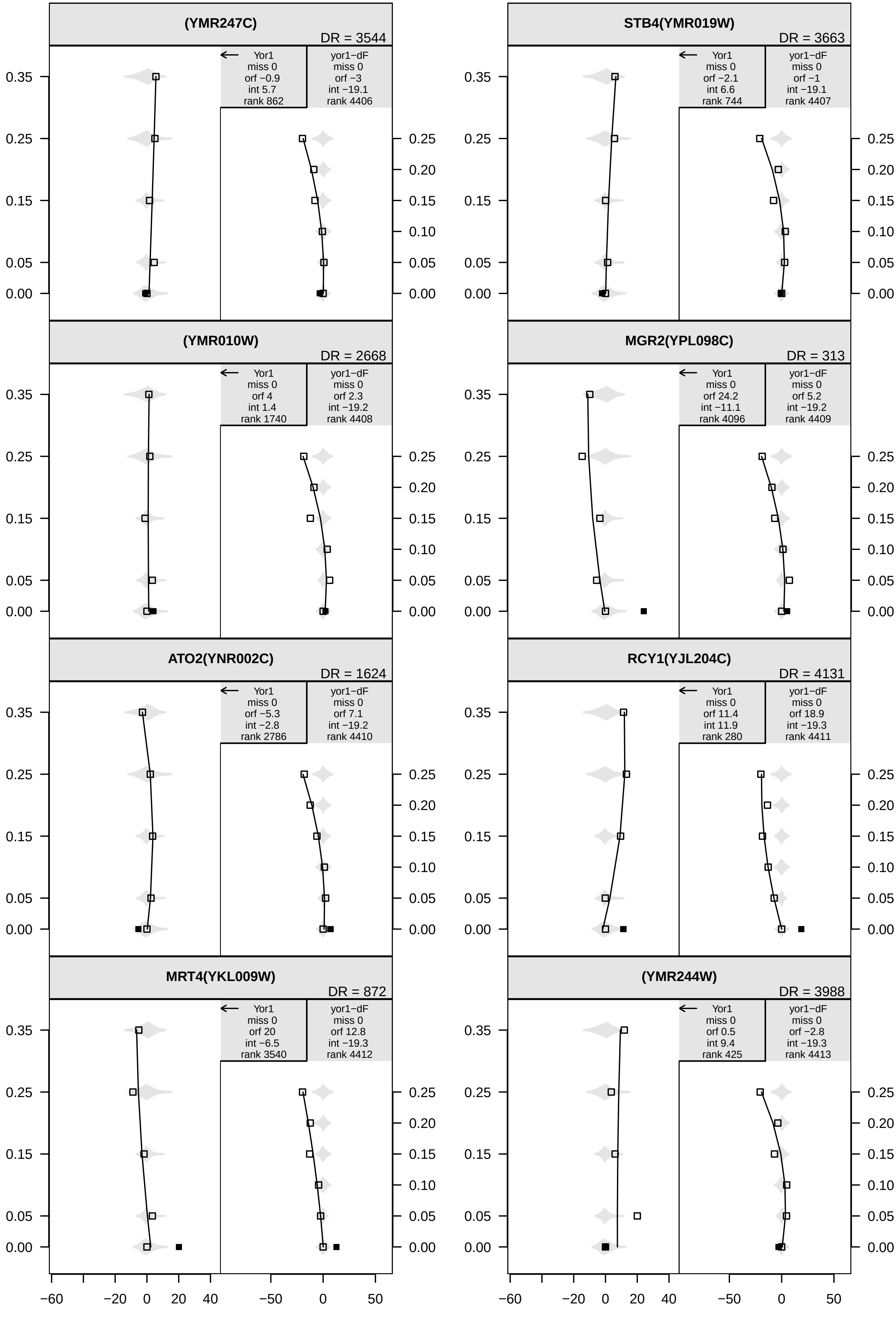

(YMR247C)
DR = 3544
Yor1 miss 0 orf −0.9 int 5.7 rank 862
yor1−dF miss 0 orf −3 int −19.1 rank 4406
STB4(YMR019W)
DR = 3663
Yor1 miss 0 orf −2.1 int 6.6 rank 744
yor1−dF miss 0 orf −1 int −19.1 rank 4407
(YMR010W)
DR = 2668
Yor1 miss 0 orf 4 int 1.4 rank 1740
yor1−dF miss 0 orf 2.3 int −19.2 rank 4408
MGR2(YPL098C)
DR = 313
Yor1 miss 0 orf 24.2 int −11.1 rank 4096
yor1−dF miss 0 orf 5.2 int −19.2 rank 4409
ATO2(YNR002C)
DR = 1624
Yor1 miss 0 orf −5.3 int −2.8 rank 2786
yor1−dF miss 0 orf 7.1 int −19.2 rank 4410
RCY1(YJL204C)
DR = 4131
Yor1 miss 0 orf 11.4 int 11.9 rank 280
yor1−dF miss 0 orf 18.9 int −19.3 rank 4411
MRT4(YKL009W)
DR = 872
Yor1 miss 0 orf 20 int −6.5 rank 3540
yor1−dF miss 0 orf 12.8 int −19.3 rank 4412
(YMR244W)
DR = 3988
Yor1 miss 0 orf 0.5 int 9.4 rank 425
yor1−dF miss 0 orf −2.8 int −19.3 rank 4413
0.35 0.25 0.20 0.15 0.10 0.05 0.00
−60 −20 0 20 40 −50 0 50

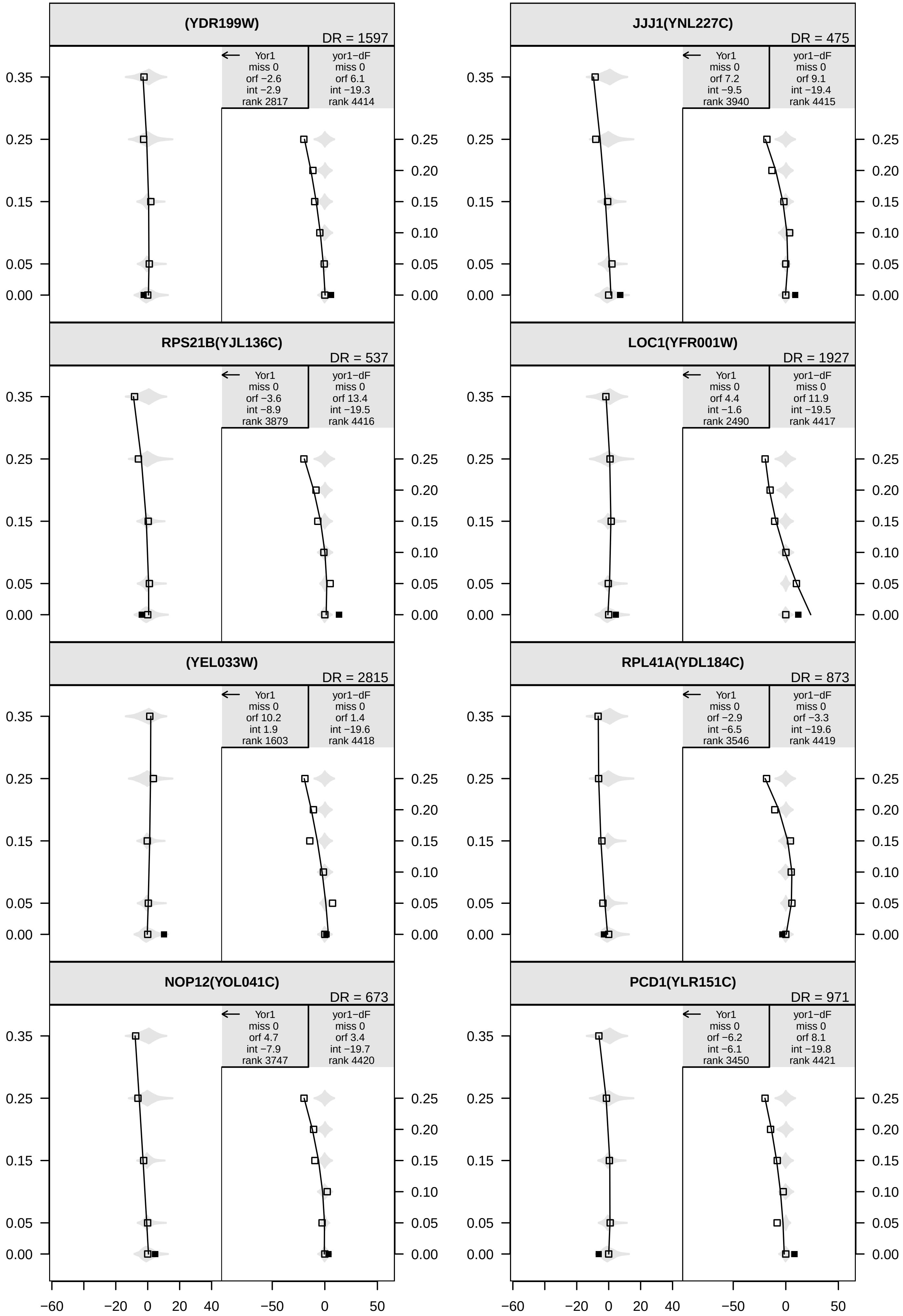

(YDR199W)
DR = 1597
Yor1 miss 0 orf −2.6 int −2.9 rank 2817
yor1−dF miss 0 orf 6.1 int −19.3 rank 4414
JJJ1(YNL227C)
DR = 475
Yor1 miss 0 orf 7.2 int −9.5 rank 3940
yor1−dF miss 0 orf 9.1 int −19.4 rank 4415
RPS21B(YJL136C)
DR = 537
Yor1 miss 0 orf −3.6 int −8.9 rank 3879
yor1−dF miss 0 orf 13.4 int −19.5 rank 4416
LOC1(YFR001W)
DR = 1927
Yor1 miss 0 orf 4.4 int −1.6 rank 2490
yor1−dF miss 0 orf 11.9 int −19.5 rank 4417
(YEL033W)
DR = 2815
Yor1 miss 0 orf 10.2 int 1.9 rank 1603
yor1−dF miss 0 orf 1.4 int −19.6 rank 4418
RPL41A(YDL184C)
DR = 873
Yor1 miss 0 orf −2.9 int −6.5 rank 3546
yor1−dF miss 0 orf −3.3 int −19.6 rank 4419
NOP12(YOL041C)
DR = 673
Yor1 miss 0 orf 4.7 int −7.9 rank 3747
yor1−dF miss 0 orf 3.4 int −19.7 rank 4420
PCD1(YLR151C)
DR = 971
Yor1 miss 0 orf −6.2 int −6.1 rank 3450
yor1−dF miss 0 orf 8.1 int −19.8 rank 4421
0.35 0.25 0.15 0.05 0.00
0.25 0.20 0.15 0.10 0.05 0.00
−60 −20 0 20 40 −50 0 50

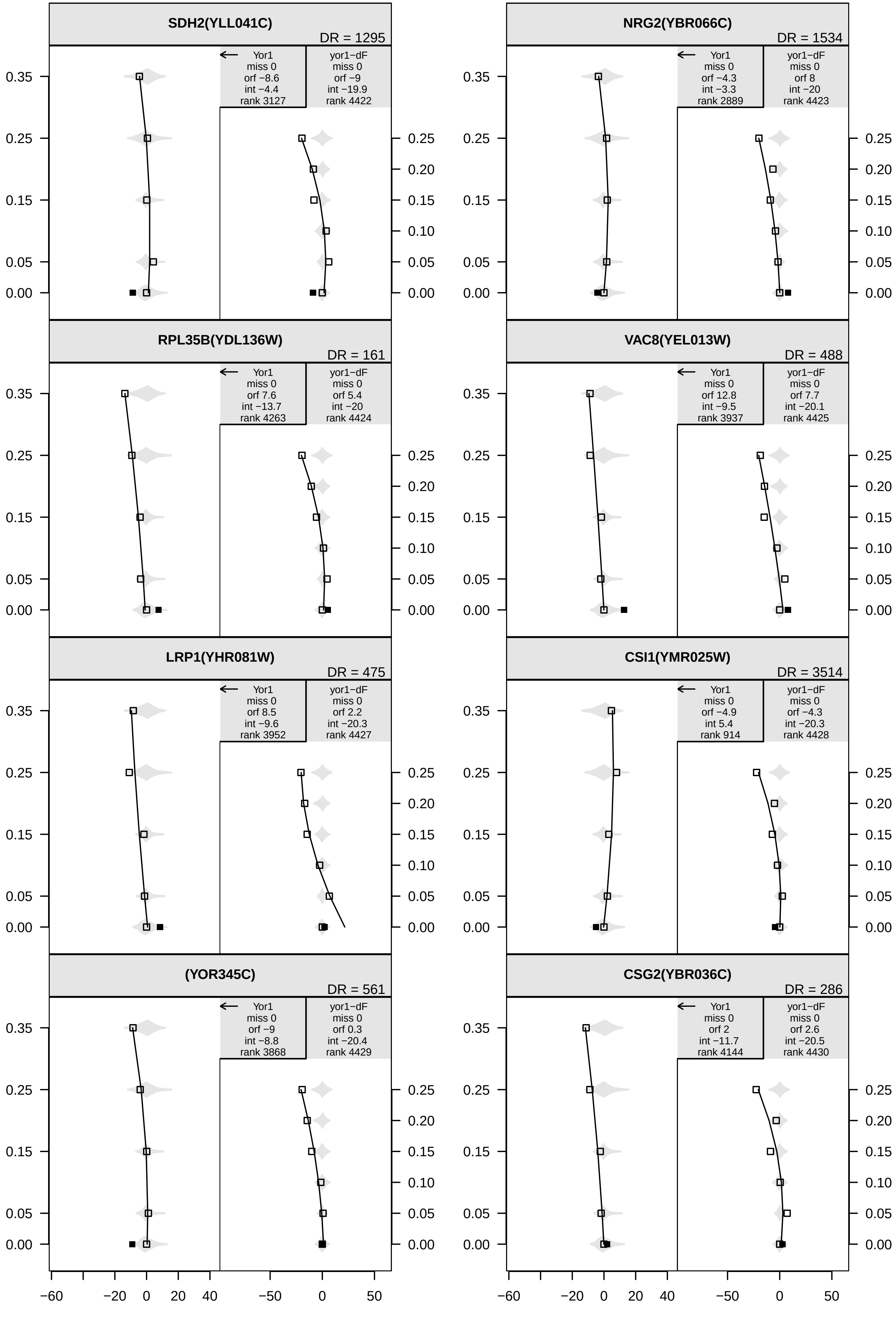

SDH2(YLL041C)
DR = 1295
Yor1 miss 0 orf −8.6 int −4.4 rank 3127
yor1−dF miss 0 orf −9 int −19.9 rank 4422
NRG2(YBR066C)
DR = 1534
Yor1 miss 0 orf −4.3 int −3.3 rank 2889
yor1−dF miss 0 orf 8 int −20 rank 4423
RPL35B(YDL136W)
DR = 161
Yor1 miss 0 orf 7.6 int −13.7 rank 4263
yor1−dF miss 0 orf 5.4 int −20 rank 4424
VAC8(YEL013W)
DR = 488
Yor1 miss 0 orf 12.8 int −9.5 rank 3937
yor1−dF miss 0 orf 7.7 int −20.1 rank 4425
LRP1(YHR081W)
DR = 475
Yor1 miss 0 orf 8.5 int −9.6 rank 3952
yor1−dF miss 0 orf 2.2 int −20.3 rank 4427
CSI1(YMR025W)
DR = 3514
Yor1 miss 0 orf −4.9 int 5.4 rank 914
yor1−dF miss 0 orf −4.3 int −20.3 rank 4428
(YOR345C)
DR = 561
Yor1 miss 0 orf −9 int −8.8 rank 3868
yor1−dF miss 0 orf 0.3 int −20.4 rank 4429
CSG2(YBR036C)
DR = 286
Yor1 miss 0 orf 2 int −11.7 rank 4144
yor1−dF miss 0 orf 2.6 int −20.5 rank 4430
0.35 0.25 0.20 0.15 0.10 0.05 0.00
−60 −20 0 20 40 −50 0 50

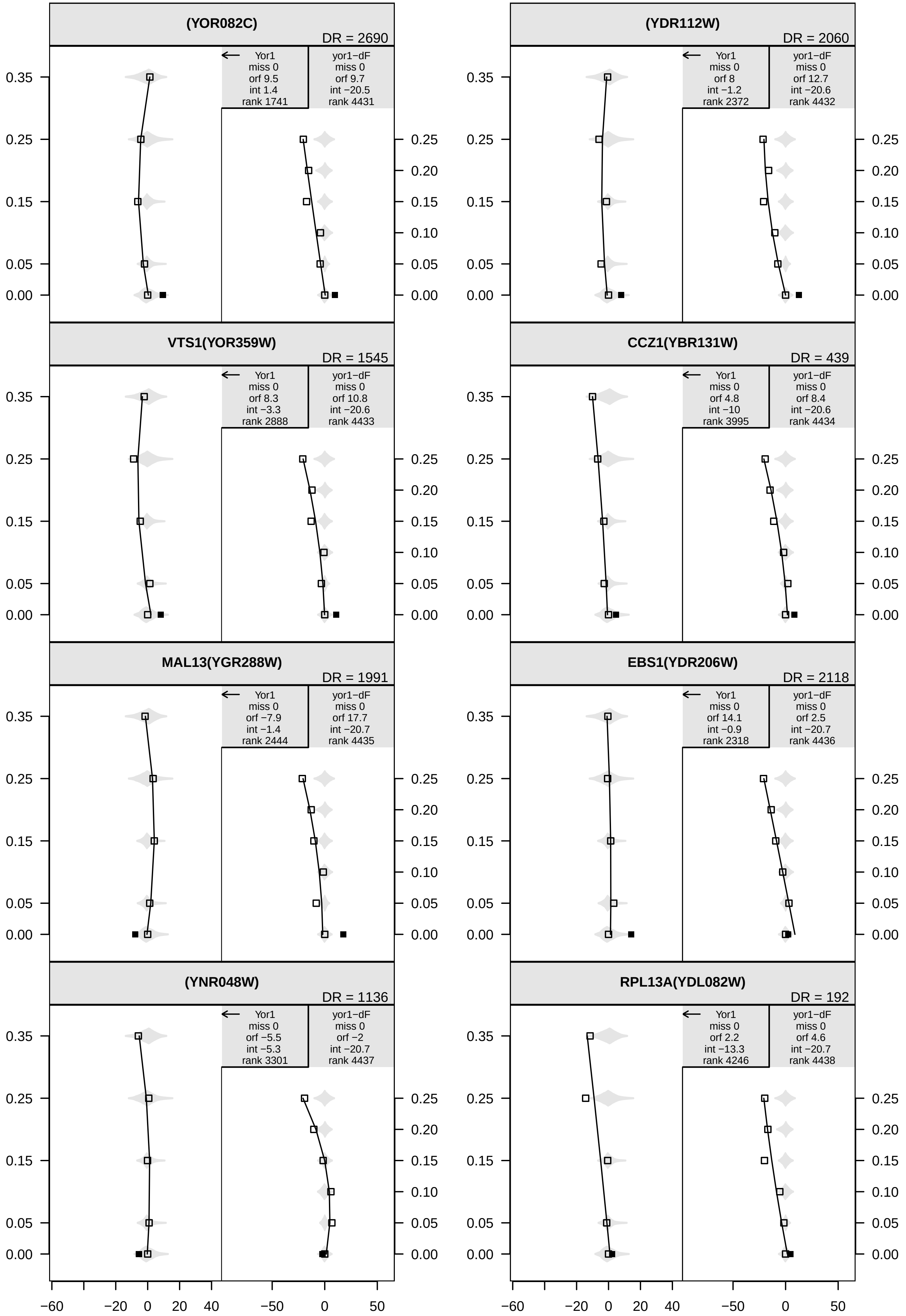
(YOR082C)
DR = 2690
Yor1 miss 0 orf 9.5 int 1.4 rank 1741
yor1−dF miss 0 orf 9.7 int −20.5 rank 4431
(YDR112W)
DR = 2060
Yor1 miss 0 orf 8 int −1.2 rank 2372
yor1−dF miss 0 orf 12.7 int −20.6 rank 4432
VTS1(YOR359W)
DR = 1545
Yor1 miss 0 orf 8.3 int −3.3 rank 2888
yor1−dF miss 0 orf 10.8 int −20.6 rank 4433
CCZ1(YBR131W)
DR = 439
Yor1 miss 0 orf 4.8 int −10 rank 3995
yor1−dF miss 0 orf 8.4 int −20.6 rank 4434
MAL13(YGR288W)
DR = 1991
Yor1 miss 0 orf −7.9 int −1.4 rank 2444
yor1−dF miss 0 orf 17.7 int −20.7 rank 4435
EBS1(YDR206W)
DR = 2118
Yor1 miss 0 orf 14.1 int −0.9 rank 2318
yor1−dF miss 0 orf 2.5 int −20.7 rank 4436
(YNR048W)
DR = 1136
Yor1 miss 0 orf −5.5 int −5.3 rank 3301
yor1−dF miss 0 orf −2 int −20.7 rank 4437
RPL13A(YDL082W)
DR = 192
Yor1 miss 0 orf 2.2 int −13.3 rank 4246
yor1−dF miss 0 orf 4.6 int −20.7 rank 4438
0.35 0.25 0.15 0.05 0.00
0.25 0.20 0.15 0.10 0.05 0.00
−60 −20 0 20 40
−50 0 50

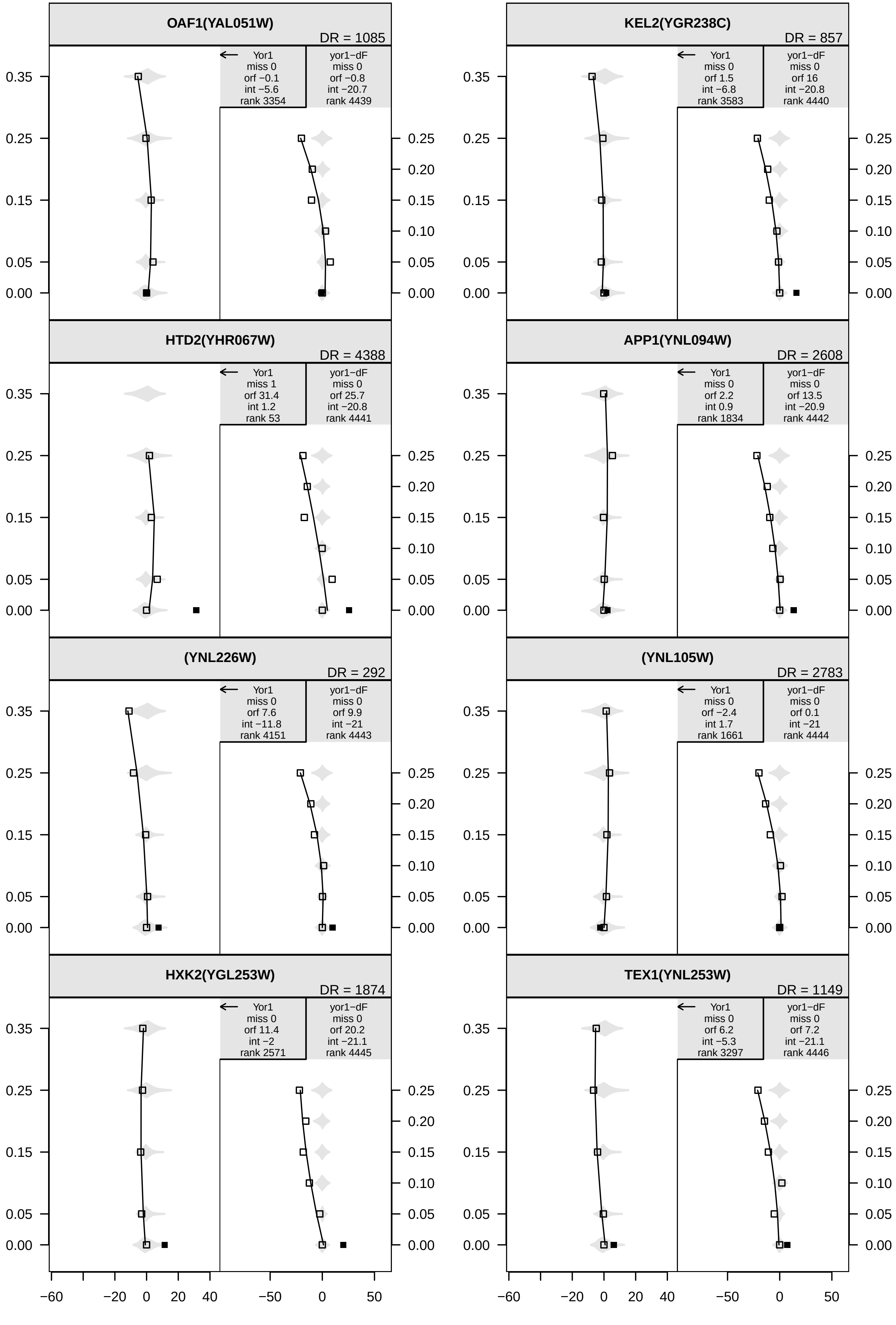
OAF1(YAL051W)
DR = 1085
Yor1 miss 0 orf −0.1 int −5.6 rank 3354
yor1−dF miss 0 orf −0.8 int −20.7 rank 4439
KEL2(YGR238C)
DR = 857
Yor1 miss 0 orf 1.5 int −6.8 rank 3583
yor1−dF miss 0 orf 16 int −20.8 rank 4440
HTD2(YHR067W)
DR = 4388
Yor1 miss 1 orf 31.4 int 1.2 rank 53
yor1−dF miss 0 orf 25.7 int −20.8 rank 4441
APP1(YNL094W)
DR = 2608
Yor1 miss 0 orf 2.2 int 0.9 rank 1834
yor1−dF miss 0 orf 13.5 int −20.9 rank 4442
(YNL226W)
DR = 292
Yor1 miss 0 orf 7.6 int −11.8 rank 4151
yor1−dF miss 0 orf 9.9 int −21 rank 4443
(YNL105W)
DR = 2783
Yor1 miss 0 orf −2.4 int 1.7 rank 1661
yor1−dF miss 0 orf 0.1 int −21 rank 4444
HXK2(YGL253W)
DR = 1874
Yor1 miss 0 orf 11.4 int −2 rank 2571
yor1−dF miss 0 orf 20.2 int −21.1 rank 4445
TEX1(YNL253W)
DR = 1149
Yor1 miss 0 orf 6.2 int −5.3 rank 3297
yor1−dF miss 0 orf 7.2 int −21.1 rank 4446
0.35
0.25
0.20
0.15
0.10
0.05
0.00
−60
−20
0
20
40
−50
0
50

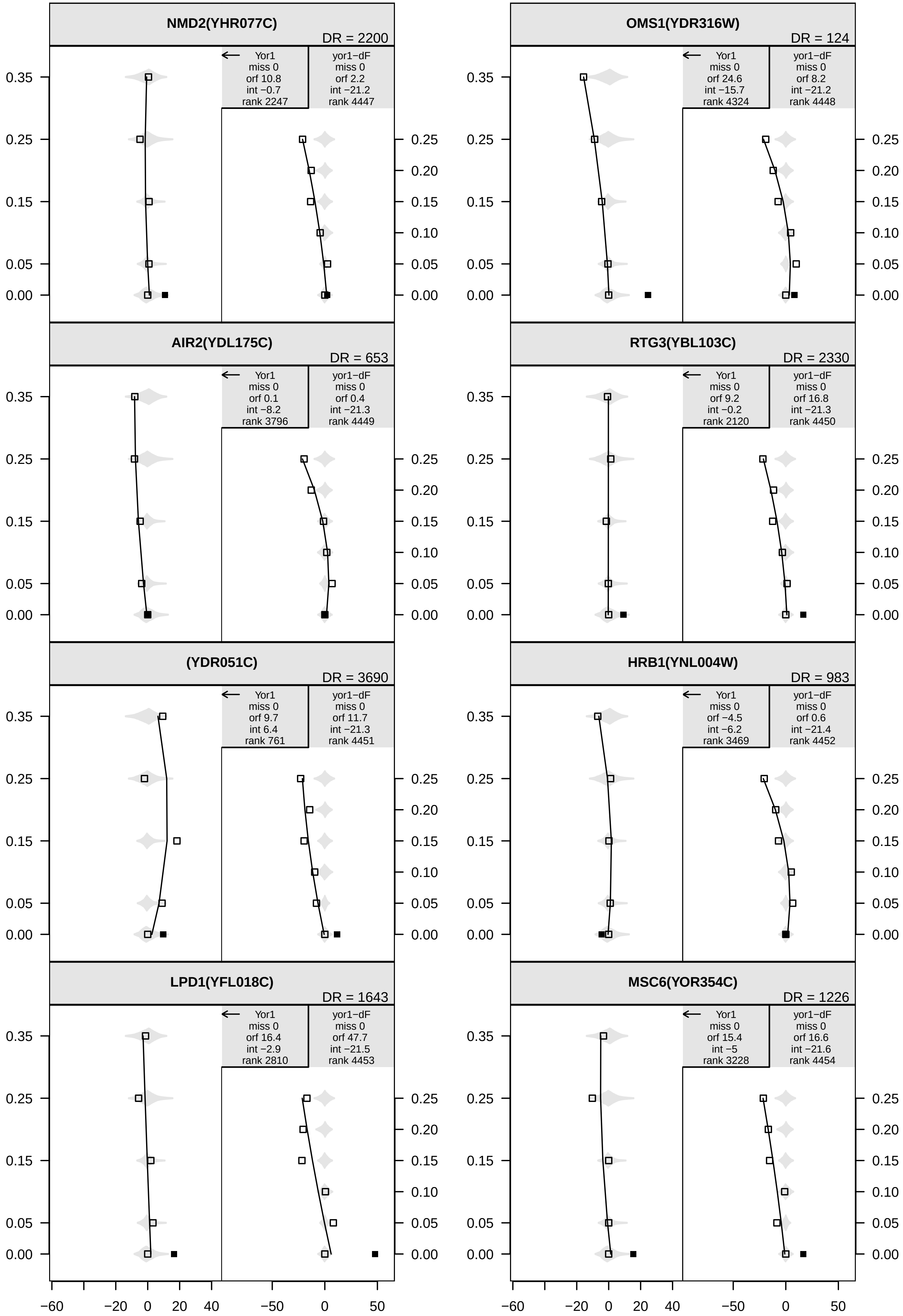
NMD2(YHR077C)
DR = 2200
Yor1 miss 0 orf 10.8 int −0.7 rank 2247
yor1−dF miss 0 orf 2.2 int −21.2 rank 4447
OMS1(YDR316W)
DR = 124
Yor1 miss 0 orf 24.6 int −15.7 rank 4324
yor1−dF miss 0 orf 8.2 int −21.2 rank 4448
AIR2(YDL175C)
DR = 653
Yor1 miss 0 orf 0.1 int −8.2 rank 3796
yor1−dF miss 0 orf 0.4 int −21.3 rank 4449
RTG3(YBL103C)
DR = 2330
Yor1 miss 0 orf 9.2 int −0.2 rank 2120
yor1−dF miss 0 orf 16.8 int −21.3 rank 4450
(YDR051C)
DR = 3690
Yor1 miss 0 orf 9.7 int 6.4 rank 761
yor1−dF miss 0 orf 11.7 int −21.3 rank 4451
HRB1(YNL004W)
DR = 983
Yor1 miss 0 orf −4.5 int −6.2 rank 3469
yor1−dF miss 0 orf 0.6 int −21.4 rank 4452
LPD1(YFL018C)
DR = 1643
Yor1 miss 0 orf 16.4 int −2.9 rank 2810
yor1−dF miss 0 orf 47.7 int −21.5 rank 4453
MSC6(YOR354C)
DR = 1226
Yor1 miss 0 orf 15.4 int −5 rank 3228
yor1−dF miss 0 orf 16.6 int −21.6 rank 4454
0.35 0.25 0.20 0.15 0.10 0.05 0.00
−60 −20 0 20 40 −50 0 50

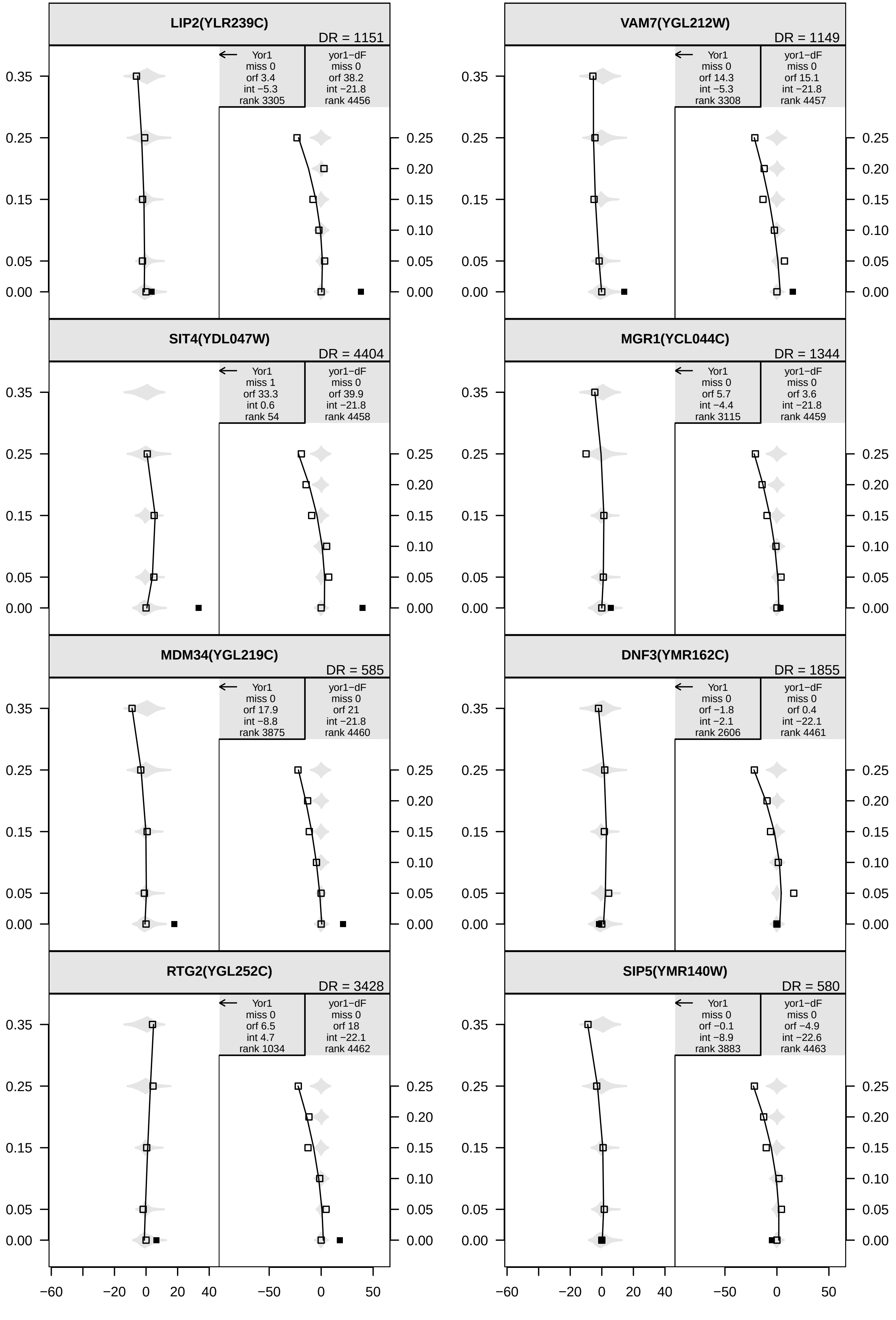

LIP2(YLR239C)
DR = 1151
Yor1 miss 0 orf 3.4 int −5.3 rank 3305
yor1−dF miss 0 orf 38.2 int −21.8 rank 4456
VAM7(YGL212W)
DR = 1149
Yor1 miss 0 orf 14.3 int −5.3 rank 3308
yor1−dF miss 0 orf 15.1 int −21.8 rank 4457
SIT4(YDL047W)
DR = 4404
Yor1 miss 1 orf 33.3 int 0.6 rank 54
yor1−dF miss 0 orf 39.9 int −21.8 rank 4458
MGR1(YCL044C)
DR = 1344
Yor1 miss 0 orf 5.7 int −4.4 rank 3115
yor1−dF miss 0 orf 3.6 int −21.8 rank 4459
MDM34(YGL219C)
DR = 585
Yor1 miss 0 orf 17.9 int −8.8 rank 3875
yor1−dF miss 0 orf 21 int −21.8 rank 4460
DNF3(YMR162C)
DR = 1855
Yor1 miss 0 orf −1.8 int −2.1 rank 2606
yor1−dF miss 0 orf 0.4 int −22.1 rank 4461
RTG2(YGL252C)
DR = 3428
Yor1 miss 0 orf 6.5 int 4.7 rank 1034
yor1−dF miss 0 orf 18 int −22.1 rank 4462
SIP5(YMR140W)
DR = 580
Yor1 miss 0 orf −0.1 int −8.9 rank 3883
yor1−dF miss 0 orf −4.9 int −22.6 rank 4463
0.35 0.25 0.20 0.15 0.10 0.05 0.00
−60 −20 0 20 40 −50 0 50

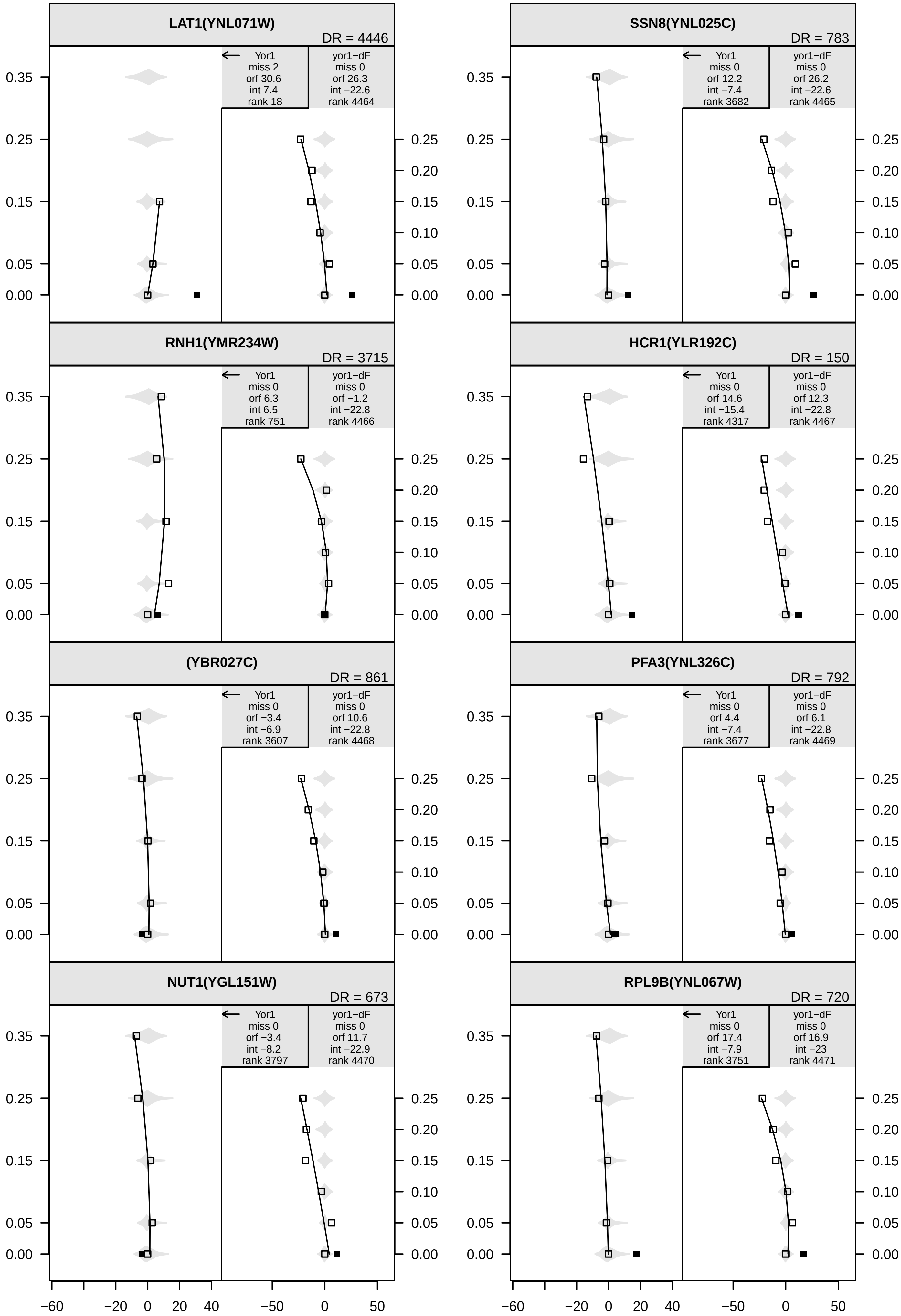
LAT1(YNL071W)
DR = 4446
Yor1 miss 2 orf 30.6 int 7.4 rank 18
yor1−dF miss 0 orf 26.3 int −22.6 rank 4464
SSN8(YNL025C)
DR = 783
Yor1 miss 0 orf 12.2 int −7.4 rank 3682
yor1−dF miss 0 orf 26.2 int −22.6 rank 4465
RNH1(YMR234W)
DR = 3715
Yor1 miss 0 orf 6.3 int 6.5 rank 751
yor1−dF miss 0 orf −1.2 int −22.8 rank 4466
HCR1(YLR192C)
DR = 150
Yor1 miss 0 orf 14.6 int −15.4 rank 4317
yor1−dF miss 0 orf 12.3 int −22.8 rank 4467
(YBR027C)
DR = 861
Yor1 miss 0 orf −3.4 int −6.9 rank 3607
yor1−dF miss 0 orf 10.6 int −22.8 rank 4468
PFA3(YNL326C)
DR = 792
Yor1 miss 0 orf 4.4 int −7.4 rank 3677
yor1−dF miss 0 orf 6.1 int −22.8 rank 4469
NUT1(YGL151W)
DR = 673
Yor1 miss 0 orf −3.4 int −8.2 rank 3797
yor1−dF miss 0 orf 11.7 int −22.9 rank 4470
RPL9B(YNL067W)
DR = 720
Yor1 miss 0 orf 17.4 int −7.9 rank 3751
yor1−dF miss 0 orf 16.9 int −23 rank 4471
0.35 0.25 0.20 0.15 0.10 0.05 0.00
−60 −20 0 20 40 −50 0 50

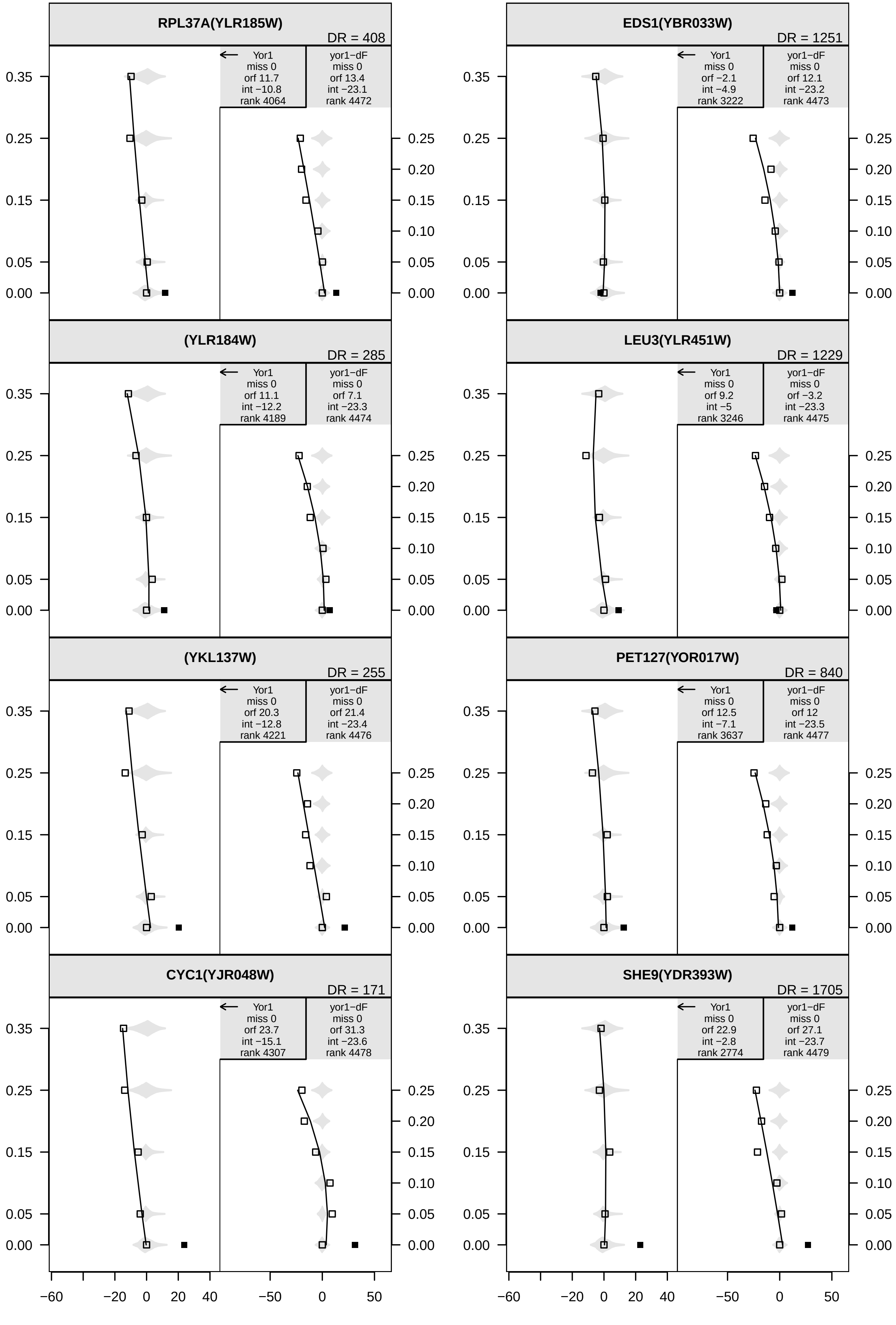

RPL37A(YLR185W)
DR = 408
Yor1
miss 0
orf 11.7
int −10.8
rank 4064
yor1−dF
miss 0
orf 13.4
int −23.1
rank 4472
EDS1(YBR033W)
DR = 1251
Yor1
miss 0
orf −2.1
int −4.9
rank 3222
yor1−dF
miss 0
orf 12.1
int −23.2
rank 4473
(YLR184W)
DR = 285
Yor1
miss 0
orf 11.1
int −12.2
rank 4189
yor1−dF
miss 0
orf 7.1
int −23.3
rank 4474
LEU3(YLR451W)
DR = 1229
Yor1
miss 0
orf 9.2
int −5
rank 3246
yor1−dF
miss 0
orf −3.2
int −23.3
rank 4475
(YKL137W)
DR = 255
Yor1
miss 0
orf 20.3
int −12.8
rank 4221
yor1−dF
miss 0
orf 21.4
int −23.4
rank 4476
PET127(YOR017W)
DR = 840
Yor1
miss 0
orf 12.5
int −7.1
rank 3637
yor1−dF
miss 0
orf 12
int −23.5
rank 4477
CYC1(YJR048W)
DR = 171
Yor1
miss 0
orf 23.7
int −15.1
rank 4307
yor1−dF
miss 0
orf 31.3
int −23.6
rank 4478
SHE9(YDR393W)
DR = 1705
Yor1
miss 0
orf 22.9
int −2.8
rank 2774
yor1−dF
miss 0
orf 27.1
int −23.7
rank 4479
0.35
0.25
0.20
0.15
0.10
0.05
0.00
−60
−20
0
20
40
−50
0
50

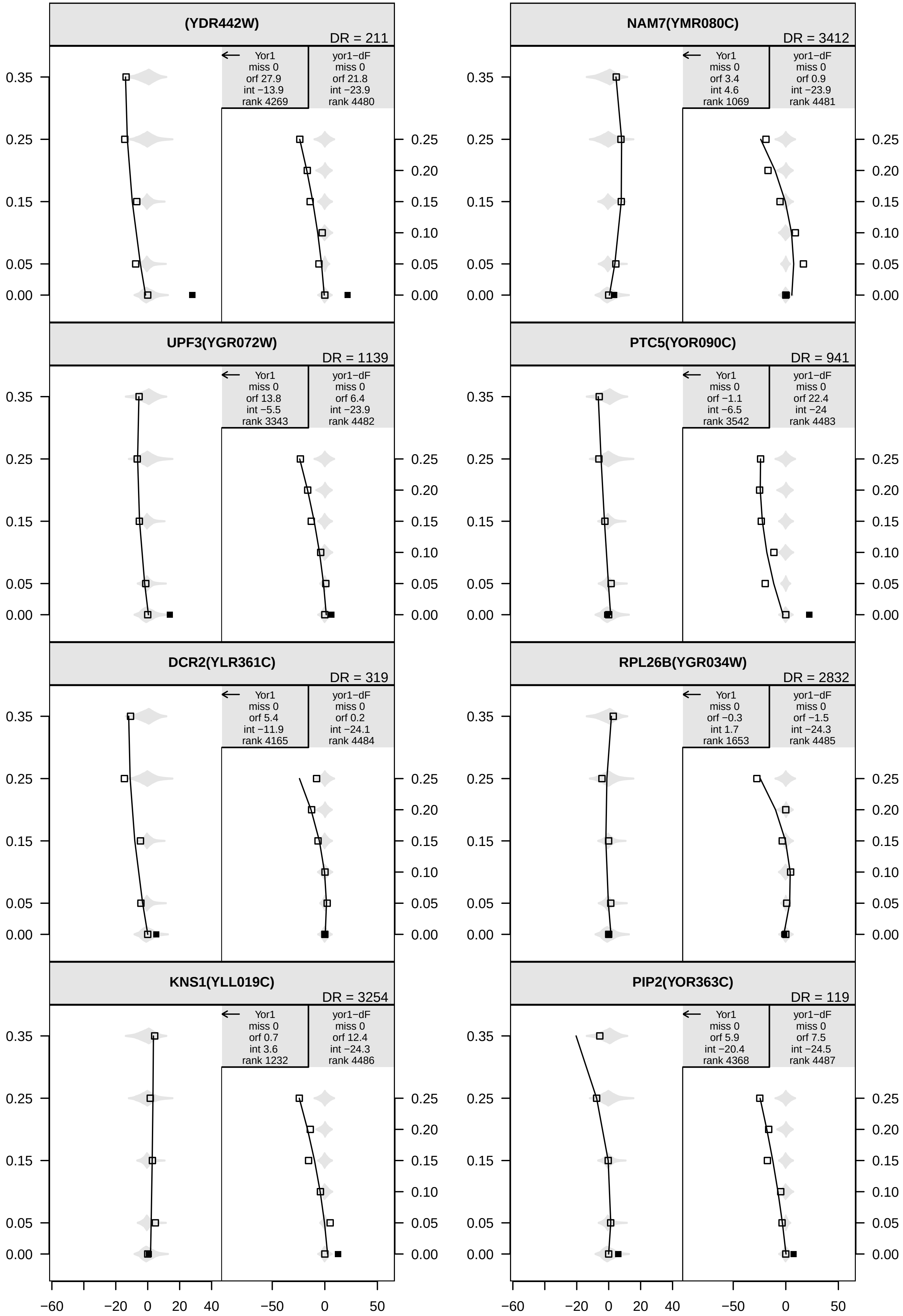
(YDR442W)
DR = 211
Yor1 miss 0 orf 27.9 int −13.9 rank 4269
yor1−dF miss 0 orf 21.8 int −23.9 rank 4480
NAM7(YMR080C)
DR = 3412
Yor1 miss 0 orf 3.4 int 4.6 rank 1069
yor1−dF miss 0 orf 0.9 int −23.9 rank 4481
UPF3(YGR072W)
DR = 1139
Yor1 miss 0 orf 13.8 int −5.5 rank 3343
yor1−dF miss 0 orf 6.4 int −23.9 rank 4482
PTC5(YOR090C)
DR = 941
Yor1 miss 0 orf −1.1 int −6.5 rank 3542
yor1−dF miss 0 orf 22.4 int −24 rank 4483
DCR2(YLR361C)
DR = 319
Yor1 miss 0 orf 5.4 int −11.9 rank 4165
yor1−dF miss 0 orf 0.2 int −24.1 rank 4484
RPL26B(YGR034W)
DR = 2832
Yor1 miss 0 orf −0.3 int 1.7 rank 1653
yor1−dF miss 0 orf −1.5 int −24.3 rank 4485
KNS1(YLL019C)
DR = 3254
Yor1 miss 0 orf 0.7 int 3.6 rank 1232
yor1−dF miss 0 orf 12.4 int −24.3 rank 4486
PIP2(YOR363C)
DR = 119
Yor1 miss 0 orf 5.9 int −20.4 rank 4368
yor1−dF miss 0 orf 7.5 int −24.5 rank 4487
0.35 0.25 0.20 0.15 0.10 0.05 0.00
−60 −20 0 20 40 −50 0 50

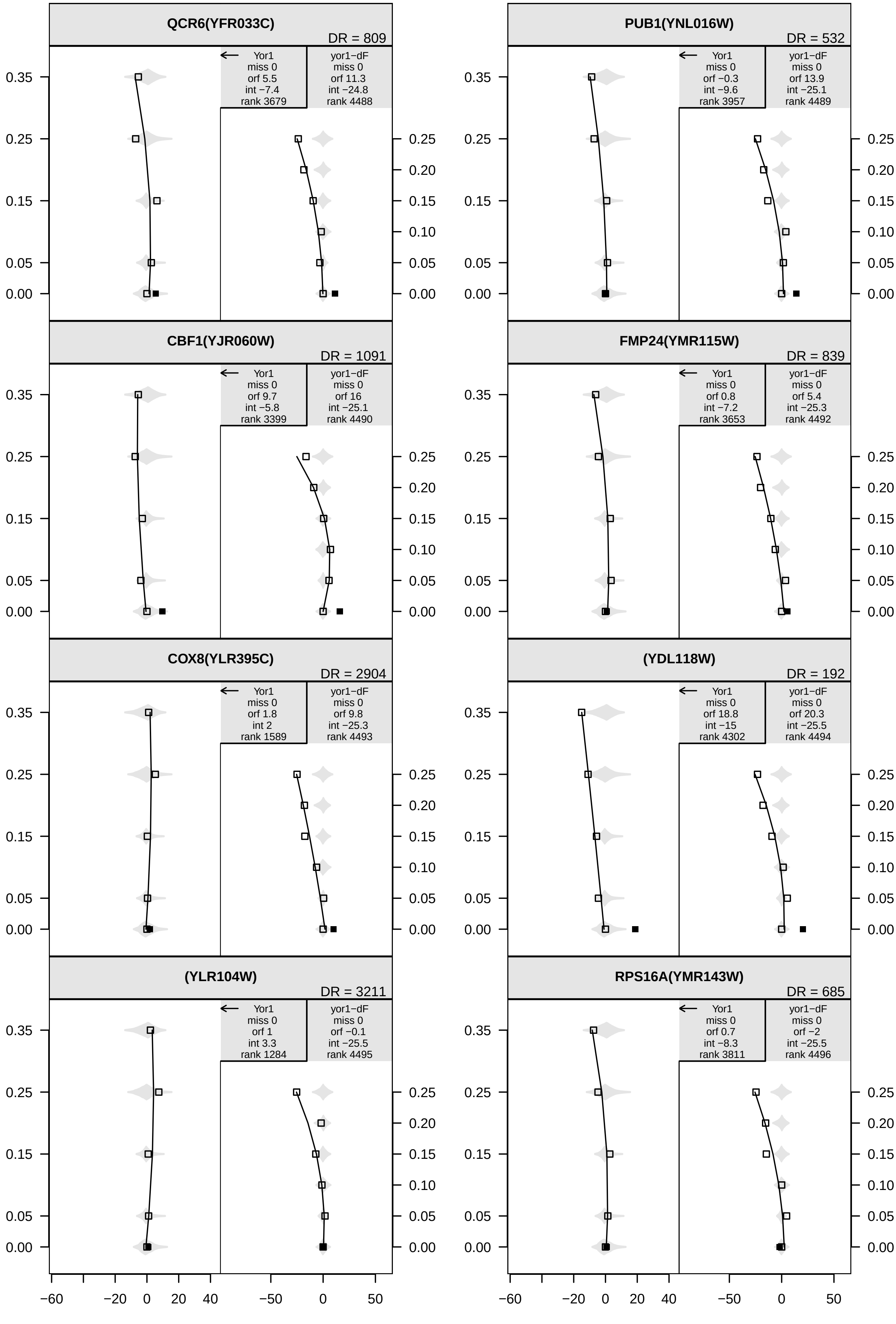
QCR6(YFR033C)
DR = 809
Yor1 miss 0 orf 5.5 int −7.4 rank 3679
yor1−dF miss 0 orf 11.3 int −24.8 rank 4488
PUB1(YNL016W)
DR = 532
Yor1 miss 0 orf −0.3 int −9.6 rank 3957
yor1−dF miss 0 orf 13.9 int −25.1 rank 4489
CBF1(YJR060W)
DR = 1091
Yor1 miss 0 orf 9.7 int −5.8 rank 3399
yor1−dF miss 0 orf 16 int −25.1 rank 4490
FMP24(YMR115W)
DR = 839
Yor1 miss 0 orf 0.8 int −7.2 rank 3653
yor1−dF miss 0 orf 5.4 int −25.3 rank 4492
COX8(YLR395C)
DR = 2904
Yor1 miss 0 orf 1.8 int 2 rank 1589
yor1−dF miss 0 orf 9.8 int −25.3 rank 4493
(YDL118W)
DR = 192
Yor1 miss 0 orf 18.8 int −15 rank 4302
yor1−dF miss 0 orf 20.3 int −25.5 rank 4494
(YLR104W)
DR = 3211
Yor1 miss 0 orf 1 int 3.3 rank 1284
yor1−dF miss 0 orf −0.1 int −25.5 rank 4495
RPS16A(YMR143W)
DR = 685
Yor1 miss 0 orf 0.7 int −8.3 rank 3811
yor1−dF miss 0 orf −2 int −25.5 rank 4496
0.35 0.25 0.20 0.15 0.10 0.05 0.00
−60 −20 0 20 40 −50 0 50

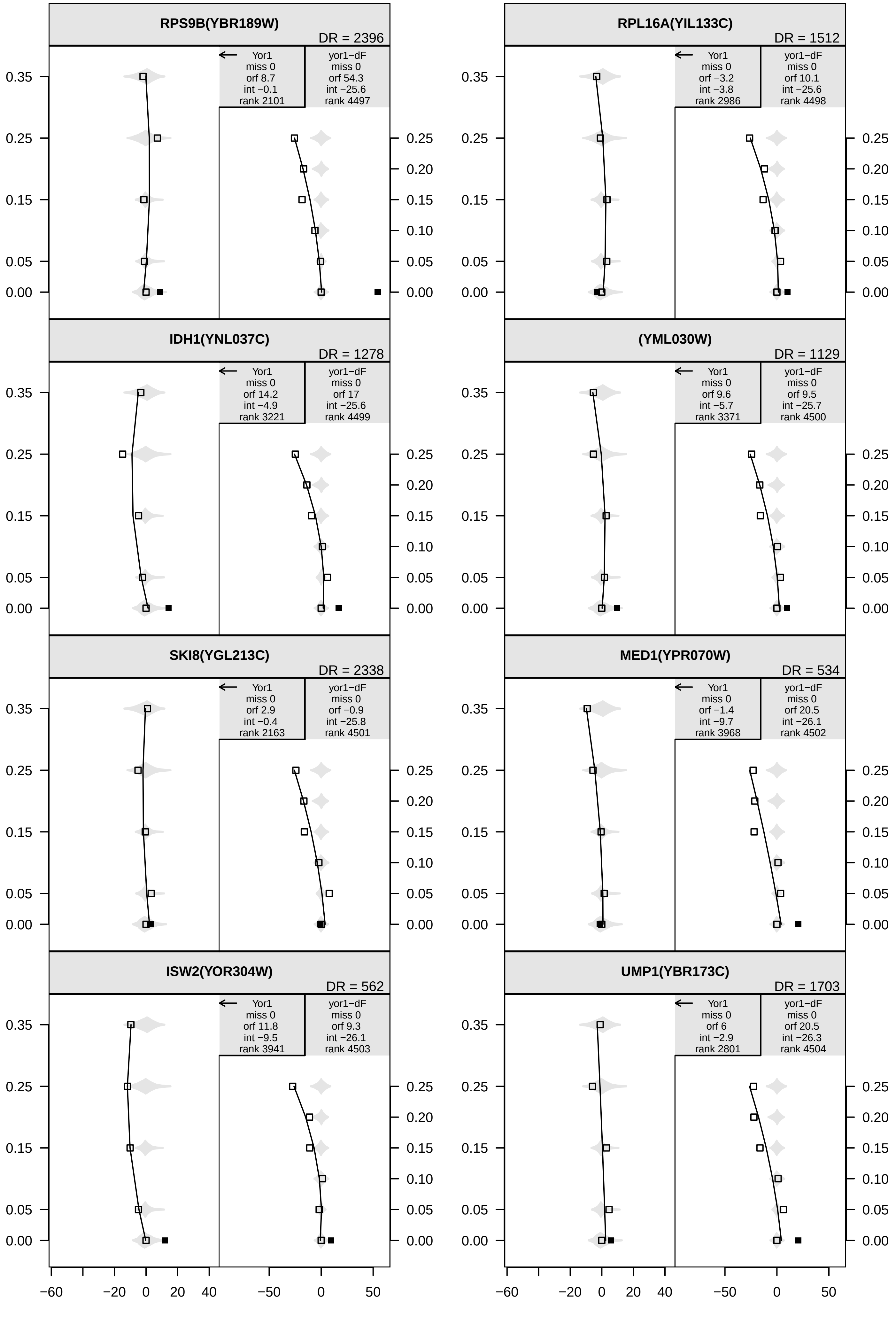
RPS9B(YBR189W)
DR = 2396
Yor1 miss 0 orf 8.7 int −0.1 rank 2101
yor1−dF miss 0 orf 54.3 int −25.6 rank 4497
RPL16A(YIL133C)
DR = 1512
Yor1 miss 0 orf −3.2 int −3.8 rank 2986
yor1−dF miss 0 orf 10.1 int −25.6 rank 4498
IDH1(YNL037C)
DR = 1278
Yor1 miss 0 orf 14.2 int −4.9 rank 3221
yor1−dF miss 0 orf 17 int −25.6 rank 4499
(YML030W)
DR = 1129
Yor1 miss 0 orf 9.6 int −5.7 rank 3371
yor1−dF miss 0 orf 9.5 int −25.7 rank 4500
SKI8(YGL213C)
DR = 2338
Yor1 miss 0 orf 2.9 int −0.4 rank 2163
yor1−dF miss 0 orf −0.9 int −25.8 rank 4501
MED1(YPR070W)
DR = 534
Yor1 miss 0 orf −1.4 int −9.7 rank 3968
yor1−dF miss 0 orf 20.5 int −26.1 rank 4502
ISW2(YOR304W)
DR = 562
Yor1 miss 0 orf 11.8 int −9.5 rank 3941
yor1−dF miss 0 orf 9.3 int −26.1 rank 4503
UMP1(YBR173C)
DR = 1703
Yor1 miss 0 orf 6 int −2.9 rank 2801
yor1−dF miss 0 orf 20.5 int −26.3 rank 4504
0.35
0.25
0.20
0.15
0.10
0.05
0.00
−60
−20
0
20
40
−50
0
50

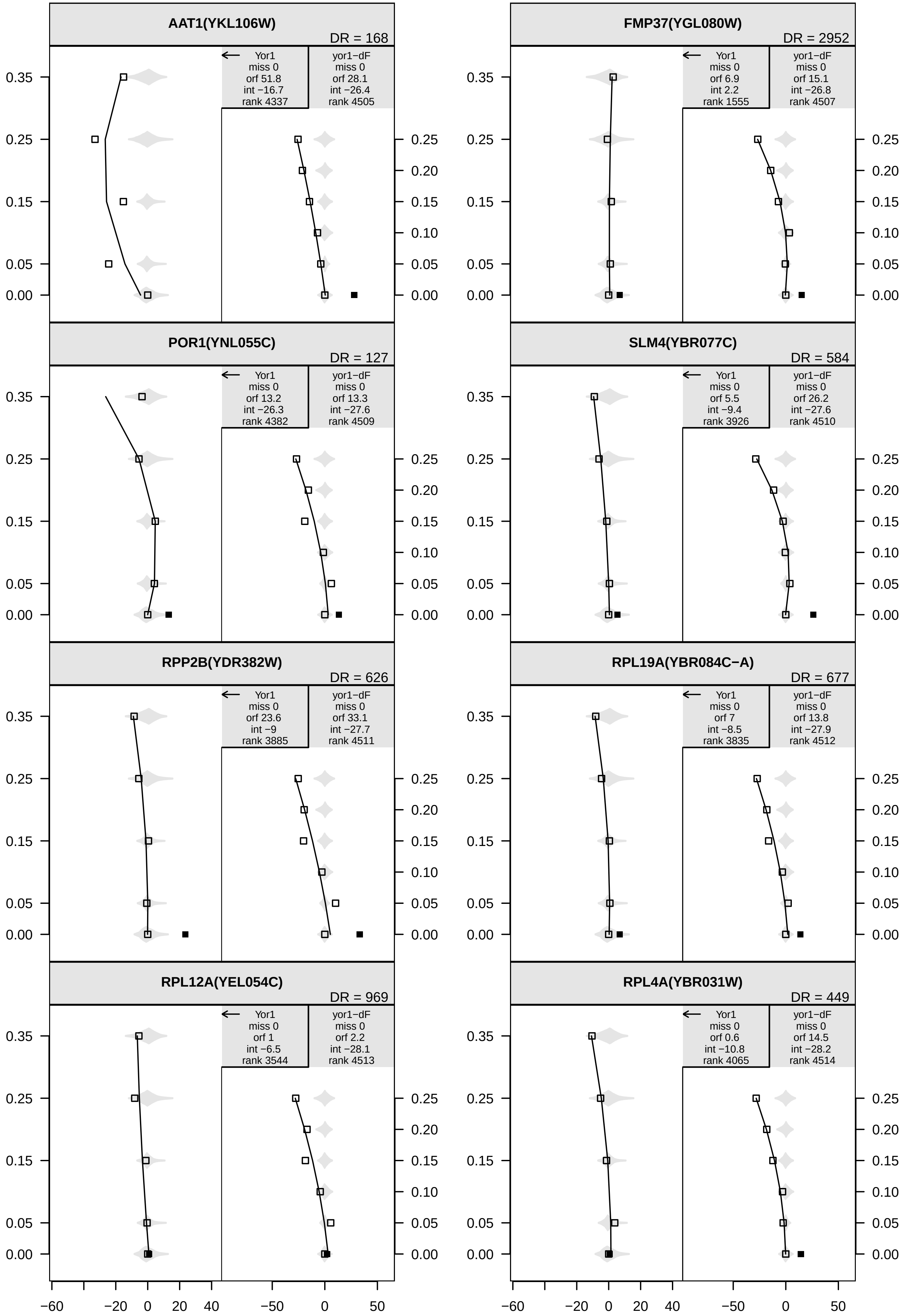

AAT1(YKL106W)
DR = 168
Yor1
miss 0
orf 51.8
int −16.7
rank 4337
yor1−dF
miss 0
orf 28.1
int −26.4
rank 4505
FMP37(YGL080W)
DR = 2952
Yor1
miss 0
orf 6.9
int 2.2
rank 1555
yor1−dF
miss 0
orf 15.1
int −26.8
rank 4507
POR1(YNL055C)
DR = 127
Yor1
miss 0
orf 13.2
int −26.3
rank 4382
yor1−dF
miss 0
orf 13.3
int −27.6
rank 4509
SLM4(YBR077C)
DR = 584
Yor1
miss 0
orf 5.5
int −9.4
rank 3926
yor1−dF
miss 0
orf 26.2
int −27.6
rank 4510
RPP2B(YDR382W)
DR = 626
Yor1
miss 0
orf 23.6
int −9
rank 3885
yor1−dF
miss 0
orf 33.1
int −27.7
rank 4511
RPL19A(YBR084C−A)
DR = 677
Yor1
miss 0
orf 7
int −8.5
rank 3835
yor1−dF
miss 0
orf 13.8
int −27.9
rank 4512
RPL12A(YEL054C)
DR = 969
Yor1
miss 0
orf 1
int −6.5
rank 3544
yor1−dF
miss 0
orf 2.2
int −28.1
rank 4513
RPL4A(YBR031W)
DR = 449
Yor1
miss 0
orf 0.6
int −10.8
rank 4065
yor1−dF
miss 0
orf 14.5
int −28.2
rank 4514
0.00
0.05
0.10
0.15
0.20
0.25
0.35
−60
−20
0
20
40
−50
0
50

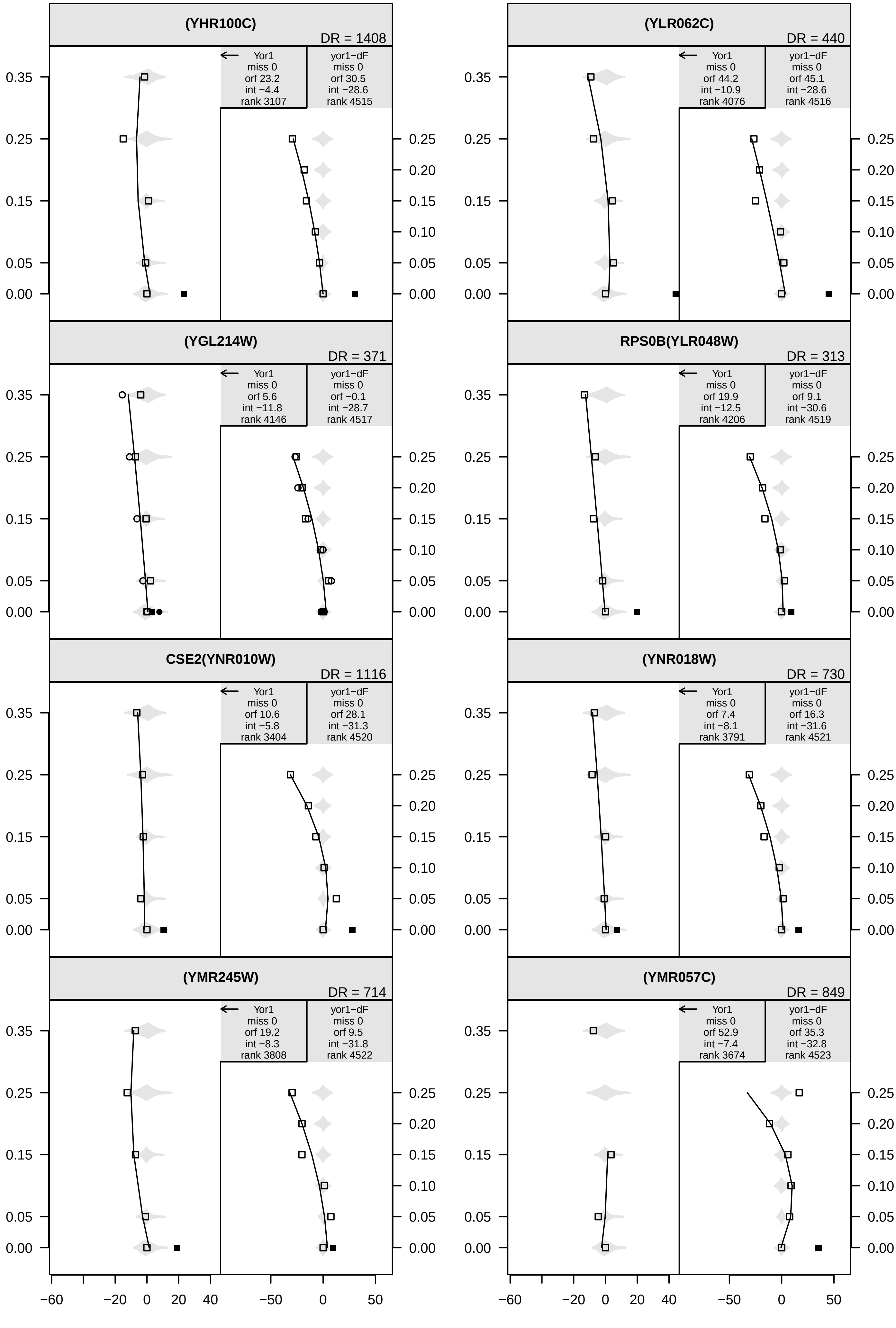

(YHR100C)
DR = 1408
Yor1 miss 0 orf 23.2 int −4.4 rank 3107
yor1−dF miss 0 orf 30.5 int −28.6 rank 4515
(YLR062C)
DR = 440
Yor1 miss 0 orf 44.2 int −10.9 rank 4076
yor1−dF miss 0 orf 45.1 int −28.6 rank 4516
(YGL214W)
DR = 371
Yor1 miss 0 orf 5.6 int −11.8 rank 4146
yor1−dF miss 0 orf −0.1 int −28.7 rank 4517
RPS0B(YLR048W)
DR = 313
Yor1 miss 0 orf 19.9 int −12.5 rank 4206
yor1−dF miss 0 orf 9.1 int −30.6 rank 4519
CSE2(YNR010W)
DR = 1116
Yor1 miss 0 orf 10.6 int −5.8 rank 3404
yor1−dF miss 0 orf 28.1 int −31.3 rank 4520
(YNR018W)
DR = 730
Yor1 miss 0 orf 7.4 int −8.1 rank 3791
yor1−dF miss 0 orf 16.3 int −31.6 rank 4521
(YMR245W)
DR = 714
Yor1 miss 0 orf 19.2 int −8.3 rank 3808
yor1−dF miss 0 orf 9.5 int −31.8 rank 4522
(YMR057C)
DR = 849
Yor1 miss 0 orf 52.9 int −7.4 rank 3674
yor1−dF miss 0 orf 35.3 int −32.8 rank 4523
0.35 0.25 0.15 0.05 0.00
0.25 0.20 0.15 0.10 0.05 0.00
−60 −20 0 20 40 −50 0 50

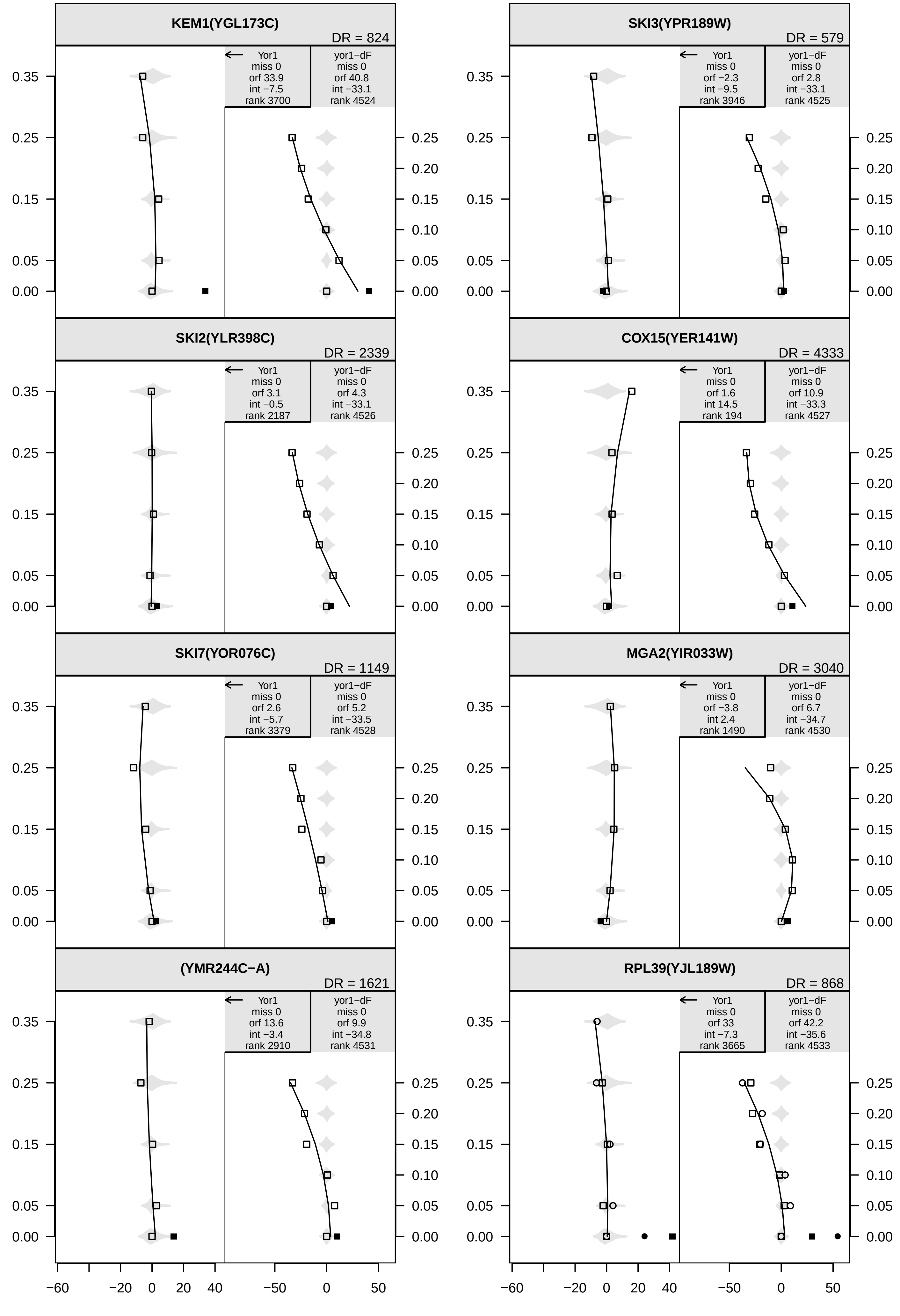
KEM1(YGL173C)
DR = 824
Yor1 miss 0 orf 33.9 int −7.5 rank 3700
yor1−dF miss 0 orf 40.8 int −33.1 rank 4524
SKI3(YPR189W)
DR = 579
Yor1 miss 0 orf −2.3 int −9.5 rank 3946
yor1−dF miss 0 orf 2.8 int −33.1 rank 4525
SKI2(YLR398C)
DR = 2339
Yor1 miss 0 orf 3.1 int −0.5 rank 2187
yor1−dF miss 0 orf 4.3 int −33.1 rank 4526
COX15(YER141W)
DR = 4333
Yor1 miss 0 orf 1.6 int 14.5 rank 194
yor1−dF miss 0 orf 10.9 int −33.3 rank 4527
SKI7(YOR076C)
DR = 1149
Yor1 miss 0 orf 2.6 int −5.7 rank 3379
yor1−dF miss 0 orf 5.2 int −33.5 rank 4528
MGA2(YIR033W)
DR = 3040
Yor1 miss 0 orf −3.8 int 2.4 rank 1490
yor1−dF miss 0 orf 6.7 int −34.7 rank 4530
(YMR244C−A)
DR = 1621
Yor1 miss 0 orf 13.6 int −3.4 rank 2910
yor1−dF miss 0 orf 9.9 int −34.8 rank 4531
RPL39(YJL189W)
DR = 868
Yor1 miss 0 orf 33 int −7.3 rank 3665
yor1−dF miss 0 orf 42.2 int −35.6 rank 4533
0.35 0.25 0.15 0.05 0.00
0.25 0.20 0.15 0.10 0.05 0.00
−60 −20 0 20 40
−50 0 50

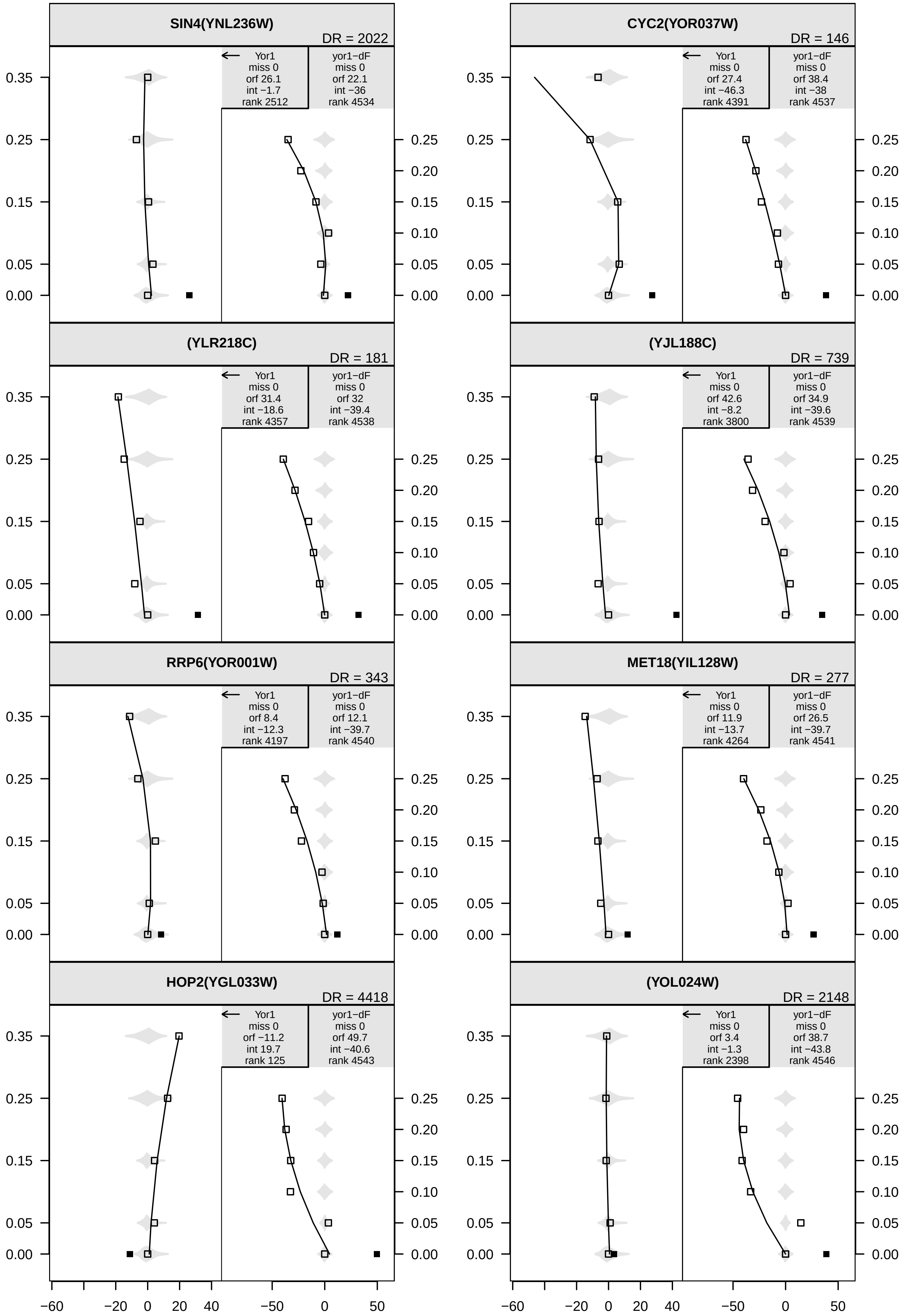
SIN4(YNL236W)
DR = 2022
Yor1
miss 0
orf 26.1
int −1.7
rank 2512
yor1−dF
miss 0
orf 22.1
int −36
rank 4534
CYC2(YOR037W)
DR = 146
Yor1
miss 0
orf 27.4
int −46.3
rank 4391
yor1−dF
miss 0
orf 38.4
int −38
rank 4537
(YLR218C)
DR = 181
Yor1
miss 0
orf 31.4
int −18.6
rank 4357
yor1−dF
miss 0
orf 32
int −39.4
rank 4538
(YJL188C)
DR = 739
Yor1
miss 0
orf 42.6
int −8.2
rank 3800
yor1−dF
miss 0
orf 34.9
int −39.6
rank 4539
RRP6(YOR001W)
DR = 343
Yor1
miss 0
orf 8.4
int −12.3
rank 4197
yor1−dF
miss 0
orf 12.1
int −39.7
rank 4540
MET18(YIL128W)
DR = 277
Yor1
miss 0
orf 11.9
int −13.7
rank 4264
yor1−dF
miss 0
orf 26.5
int −39.7
rank 4541
HOP2(YGL033W)
DR = 4418
Yor1
miss 0
orf −11.2
int 19.7
rank 125
yor1−dF
miss 0
orf 49.7
int −40.6
rank 4543
(YOL024W)
DR = 2148
Yor1
miss 0
orf 3.4
int −1.3
rank 2398
yor1−dF
miss 0
orf 38.7
int −43.8
rank 4546
0.35
0.25
0.20
0.15
0.10
0.05
0.00
−60
−20
0
20
40
−50
0
50

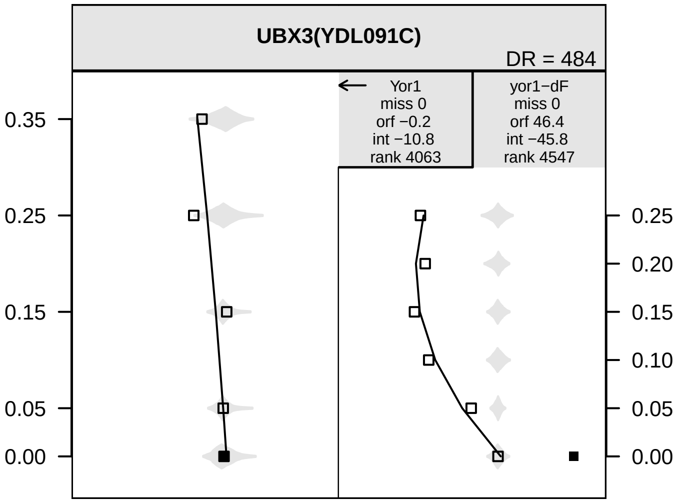

UBX3(YDL091C)
DR = 484
Yor1
miss 0
orf −0.2
int −10.8
rank 4063
yor1−dF
miss 0
orf 46.4
int −45.8
rank 4547
0.35
0.25
0.15
0.05
0.00
0.25
0.20
0.15
0.10
0.05
0.00
